# Genome-wide promoter analysis of histone modifications in human monocyte-derived antigen presenting cells

**DOI:** 10.1186/1471-2164-11-642

**Published:** 2010-11-18

**Authors:** Liina Tserel, Raivo Kolde, Ana Rebane, Kai Kisand, Tõnis Org, Hedi Peterson, Jaak Vilo, Pärt Peterson

**Affiliations:** 1 Molecular Pathology, University of Tartu, Tartu, Estonia; 2 Institute of Computer Science, University of Tartu, Tartu, Estonia

## Abstract

**Background:**

Monocyte-derived macrophages and dendritic cells (DCs) are important in inflammatory processes and are often used for immunotherapeutic approaches. Blood monocytes can be differentiated into macrophages and DCs, which is accompanied with transcriptional changes in many genes, including chemokines and cell surface markers.

**Results:**

To study the chromatin modifications associated with this differentiation, we performed a genome wide analysis of histone H3 trimethylation on lysine 4 (H3K4me3) and 27 (H3K27me3) as well as acetylation of H3 lysines (AcH3) in promoter regions. We report that both H3K4me3 and AcH3 marks significantly correlate with transcriptionally active genes whereas H3K27me3 mark is associated with inactive gene promoters. During differentiation, the H3K4me3 levels decreased on monocyte-specific CD14, CCR2 and CX3CR1 but increased on DC-specific TM7SF4/DC-STAMP, TREM2 and CD209/DC-SIGN genes. Genes associated with phagocytosis and antigen presentation were marked by H3K4me3 modifications. We also report that H3K4me3 levels on clustered chemokine and surface marker genes often correlate with transcriptional activity.

**Conclusion:**

Our results provide a basis for further functional correlations between gene expression and histone modifications in monocyte-derived macrophages and DCs.

## Background

Human peripheral blood monocytes are derived from myeloid progenitors in bone marrow. After their maturation, they migrate to the periphery and form approximately 5-10% of human leukocytes. Monocytes circulate in the blood stream for several days and then enter to peripheral tissues where they undergo a phenotypic change and differentiate further into macrophages or dendritic cells (DCs) [1-4]. In tissues, macrophages have a central role to locally phagocytose and destroy pathogens, to clear senescent cells and to repair tissues after the inflammatory processes [5]. Activated macrophages show strong inflammatory responses through the high production of pro-inflammatory cytokines and increased endocytic and antigen presentation activity. Similarly to macrophages, DCs are located in peripheral tissues where they encounter and phagocytose microbes. The recognition of microbial antigen occurs in the immature state of DCs and leads to a maturation process, after which they have increased antigen presentation but downregulated antigen recognition capacity. The maturation is often accompanied with the migration of DCs to secondary lymphoid organs [6] and several murine DC subsets with variable phenotype, function or tissue locations have been reported [7].

The peripheral blood monocytes exhibit a heterogeneous phenotype with respect to their size and nuclear morphology. They can be relatively easily identified by high expression of the cell surface marker CD14 and can be divided into at least two major subpopulations according to their CD16 expression [3]. DCs derived from monocytes are particularly important during inflammatory processes and are efficient in stimulating CD4 and CD8 positive T cell responses [8-11]. Recent studies showed that monocytes can also differentiate into macrophages and DCs under non-inflammatory conditions and that several subsets of antigen presenting cells located in antigen capture areas, such as skin, mucosa, gastrointestinal and respiratory track, are in fact derived from monocytes [12,13]. To generate a large number of human macrophages or DCs, monocytes are typically cultured for several days in the presence of granulocyte macrophage colony-stimulating factor (GM-CSF) alone or GM-CSF with interleukin 4 (IL-4), respectively [14,15]. Although these culture conditions give rise to only one subset of differentiated cells and do not represent the full heterogeneity of macrophages or DCs *in vivo*, it still is the most efficient way to obtain human antigen presenting cells. Furthermore, monocyte-derived DCs are by far the most common type of cells used in immunotherapeutic clinical approaches [16,17].

Histone modifications within promoter regions have an important function in regulation of gene expression [18]. The majority of modifications occur at the N-terminal ends of the core histones in a nucleosome. These modifications are often dynamic as well as reversible and have a functional impact on many aspects of chromatin accessibility that may determine the transcriptional status of a given gene. One of the most frequent histone modifications is the trimethylation of histone H3 lysine 4 (H3K4me3), which in gene promoters is usually associated with the transcriptional activation, whereas the trimethylation of histone H3 lysine 27 (H3K27me3) generally results in gene silencing. The presence of these two opposing modifications as bivalent marks on promoters is often associated with poised gene transcription. Similar to the H3K4me3 mark, the acetylation of lysines on histone H3 (AcH3) is often associated with transcriptional activation [18]. How exactly these combinations of chromatin modifications translate to gene transcription is currently under extensive investigation in several cell types. Transcriptional control is further mediated by chromatin associated proteins; for example H3K4me3 may serve as a docking site for PHD finger domain and AcH3 for bromodomains [19].

Here we studied the histone H3K4me3, AcH3 and H3K27me3 modifications in human monocytes, monocyte-derived macrophages and DCs. To gain insight into the role of these modifications during the differentiation process, we compared genome-wide gene expression profiles with histone changes in promoter regions over the genome. We show that H3K4me3 and AcH3 marks generally correlate well with gene expression and that H3K27me3 is associated with inactive genes. We also show that H3K4me3 levels are increased on the promoters of several marker genes and gene groups during differentiation. In addition, we provide evidence that the chromatin statuses of gene families within genomic clusters, including chemokine and surface receptor genes, are coordinately modified.

## Results

### Genome-wide mRNA expression profiles

We first identified the expression profiles of monocytes, macrophages and DCs using the Illumina Human-6 v2 BeadChip expression array. Overall, the gene expression profiles in these three cell subsets were similar with a high correlation coefficient (Additional file 1, Figure S1). However, we found a significantly higher correlation between the expression profiles of macrophage and DCs than between monocytes and macrophages or monocytes and DCs. When we used a selection criteria of a 2-fold change and differential *P *value less than 0.05, we found that compared to monocytes, a similar number of genes were upregulated in macrophages and DCs (1663 and 1630 genes, respectively). This represents 8% of the total number (ca 21 000) of human genes (Table 1). Approximately the same proportion of all genes were also downregulated during macrophage (1952; 9%) or DC (1654; 8%) differentiation. The majority of upregulated (1037) or downregulated (1328) genes in macrophage and DC cultures overlapped, indicating again a similarity between the two cell populations.

**Table 1:** Upregulated and downregulated genes in macrophages and DCs

Cell	Upregulated	Downregulated
MF only	626	624
DC only	593	326
MF and DC	1037	1328

According to the expression array, the monocyte population had a very high expression of CD14, CCR2, CSF1R and SELL/CD62L mRNA, which were all downregulated after the differentiation to macrophages or DCs (Table 2). In contrast, marker genes specific for human CD16+ monocyte subpopulation, FCGR3A/CD16A and FCGR3B/CD16B, were expressed at low levels. In both macrophage and DC populations, we observed a highly increased expression of surface marker TM7SF4/DC-STAMP, TREM2 and chemokine CCL3 and CCL22 transcripts. In DCs, several additional surface markers such as CD209/DC-SIGN, SLAM and CD1 family genes, were strongly upregulated, as well as the chemokines CCL2, CCL13, CCL17, CCL23 and CCL26. Altogether, the expression array data showed that macrophages and DCs share similar global mRNA expression pattern that is different from their monocyte precursors.

**Table 2:** Selected marker and cytokine gene expression signals and their differential scores after differentiation to macrophages and DCs

Gene	MO	MF		DC	
	Signal	Signal	Score*	Signal	Score
CD1A	226	160	-4	7934	48
CD1B	34	794	9	11970	103
CD1C	308	105	-16	4012	39
CD4	1191	494	-26	452	-30
CD14	13904	4056	-41	122	-77
CD40	57	624	28	762	85
CCL2	19	1542	6	1022	54
CCL3	72	6355	49	3436	99
CCL13	7	462	6	21325	103
CCL17	1	65	21	19817	75
CCL22	15	7026	124	14991	103
CCL23	1	406	16	2200	18
CCL26	14	13	0	2295	26
CCR2	912	1	-26	2	-26
CSF1R	11566	3911	-26	6423	-11
LY9/SLAMF3	13	113	42	635	52
SLAMF7	63	851	58	1226	76
SLAMF8	52	2745	68	2182	98
SLAMF9	11	469	108	217	55
SELL/CD62L	2807	10	-77	7	-77
DC-SIGN/CD209	46	798	28	8191	102
DC-STAMP/TM7SF4	1	8783	125	3809	103
TREM2	1	1810	123	2834	103

*Illumina BeadStudio differential expression score indicates statistical difference between expression levels. The differential score >13 corresponds to statistically significant change with P-value less than 0.05. Positive values correspond to upregulation and negative values to downregulation of expression.

### Genome-wide chromatin profiling of promoter regions

To investigate the correlation between gene expression profiles and histone modifications in monocytes, macrophages and DCs, we used a high-resolution promoter microarray screen with immunoprecipitated chromatin (ChIP-chip). Cells were cross-linked and chromatin was immunoprecipitated with antibodies to H3K4me3, H3K27me3 and AcH3, as well as to general H3. The immunoprecipitated material was then hybridized to the NimbleGen promoter array covering 19222 gene promoters in the human genome that overlap with 17289 genes on the Illumina Human-6 v2 BeadChip expression array. The ChIP-chip data with each modification-specific antibody was further normalized to random genomic background and to general H3.

**Figure 1:**
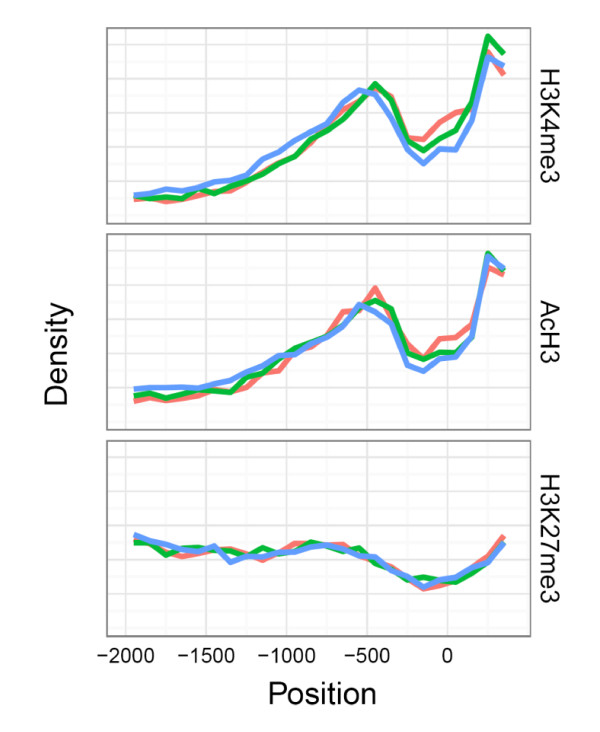
**Location of H3K4me3, AcH3 and H3K27me3 peaks on gene promoters in relation to transcription start sites**. Average density of the peaks across the all studied promoters in monocytes (red), macrophages (green) and DCs (blue) is shown. The analyzed promoter regions extended 2.0 kb upstream and 0.5 kb downstream, 0 corresponds to transcription start site. The data are normalized to H3 levels.

We first studied the occupancy and position of H3K4me3, AcH3 and H3K27me3 marks in relation to transcription start sites within the promoter regions. H3K4me3 and AcH3 signals coincided up to 1000 bp upstream and 500 bp downstream showing almost complete overlap on both sides of the transcriptional start sites (Figure 1). This pattern was different from H3K27me3, which was distributed more equally over the promoter areas. The transcriptional start site itself had remarkably lower H3K4me3 and AcH3 levels, however, H3K27me3 signals that associate with silenced genes (see below) were not significantly decreased at the transcription start sites.

### H3K4me3 and AcH3 have a high correlation on gene promoters

**Figure 2:**
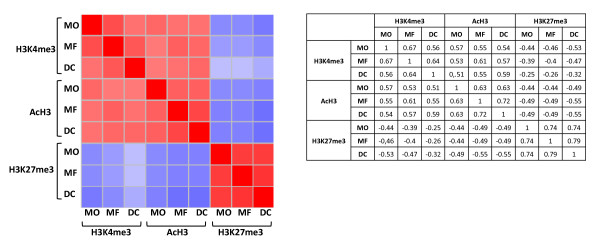
**Correlation between H3K4me3, AcH3 and H3K27me3 modifications on gene promoters within monocyte, macrophage and DC populations**. Pearson correlation analysis of the studied modifications is shown in a color scale from red (high correlation rate) to blue (low correlation). The corresponding correlation coefficients are shown in table. MO, monocytes; macrophages, MF; dendritic cells, DC.

We next analyzed the correlation between three marks in three cell populations. As shown in Figure 2, there was a highly significant correlation between H3K4me3 and AcH3 in three cell populations, while the H3K27me3 mark was present on a distinct pool of gene promoters. The most prevalent mark was the H3K4me3 present on 45% of all genes in monocytes, slightly less in macrophages (43%) and even less in DCs (37%) (Additional file 1, Figure S2). The AcH3 mark was found in three cell subsets with nearly equal frequency, being present on 30-32% of gene promoters. Of these three modifications, the H3K27me3 mark had the lowest frequency, and we found it more often in monocytes (26%) or DCs (27%) than in macrophages (23%). Notably, a substantial proportion, more than one third (36 to 39%) of gene promoters, lacked the histone modifications that were studied here. As expected, the most prevalent combination in all cells was the double H3K4me3/AcH3 mark (25-28%) whereas the combination of H3K4me3 and H3K27me3 was present in only 5-6% of gene promoters (Additional file 1, Figure S3). A notable proportion of genes (16-19%) was positive for the H3K27me3 modification alone.

### Transcriptionally active genes have a double modification of H3K4me3 and AcH3

**Figure 3:**
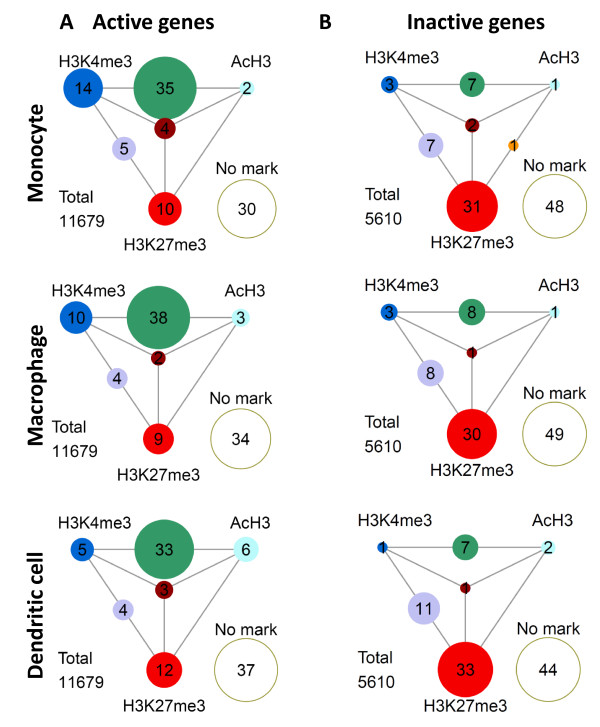
**Frequencies of H3K4me3, AcH3 and H3K27me3 marks and their combinations among (A) transcriptionally active and (B) inactive gene promoters**. Frequencies of modifications are shown as percentages in scaled circles positioned to outer corners of the triangles. Frequencies of co-occuring modifications are shown in between. Studied cell types are indicated left. "No mark" represents the lack of histone modifications analyzed in this study.

The H3K4me3 modification often overlaps with the AcH3 mark on active genes, but in combination with H3K27me3, it often marks genes poised for either activation or repression [20]. We therefore analyzed the presence of histone marks among a subset of transcriptionally active genes (Figure 3A). Similar to all genes in the genome, the most common combination among expressed genes was the H3K4me3/AcH3 modification present in 33-38% of expressed gene promoters. The double H3K4 and H3K27 trimethylation, marking so-called bivalent poised genes was present in 4-5% of genes; however, a mark for silenced genes, H3K27me3 alone, was clearly decreased (9-12%) among expressed genes. Interestingly, we noted that during the differentiation from monocytes to DCs, the proportion of genes with H3K4me3 alone decreased from 14% to 5%, in contrast to the AcH3 mark, which increased from 2% to 6% (Figure 3A). These changes, albeit to a lesser extent, also occurred in macrophage differentiation (H3K4me3 decreased to 9% and AcH3 increased to 3%). Modifications on transcriptionally inactive genes were clearly different; the main modification on these gene promoters was H3K27me3 alone (30-33%) or in combination with H3K4me3 (7-11%). Only 7% to 8% of genes had the double H3K4me3 and AcH3 mark (Figure 3B).

We next analyzed ChIP-chip data in relation to gene expression levels to see the correlation of modifications with gene activity. Analysis of the 2000 most highly expressed genes in monocytes revealed that a vast majority of genes have either the H3K4me3 or the AcH3 modification, 89% and 67% respectively, on their promoter whereas only 10% have the H3K27me3 mark (Table 3). A highly significant correlation of H3K4me3 and AcH3 marks with a high expression level was also seen in macrophage and DC populations. Though when compared to monocytes, the proportion of genes in DCs with the H3K4me3 modification was lower (82%) but higher with the AcH3 mark (72%). The positive correlation between an increased expression level and H3K4me3 and AcH3 marks was even stronger among the top 200 most highly expressed genes (data not shown). In contrast, among the 2000 lowly expressed genes, we saw a correlation with the H3K27me3 modification in all three cell populations (monocytes, 56%; macrophages, 47%; DCs, 54%), while the number of genes having both the H3K4me3 and the AcH3 marks was clearly smaller than in the 2000 highly expressed genes (Table 3). It should be noted that the number of genes without any of the three marks was rather similar in both subsets of expressed genes.

**Table 3:** Frequency of H3K4me3, AcH3 and H3K27me3 modifications on 2000 genes with high or low expression and their correlation P-values

Modification	MO		MF		DC	
	High	Low	High	Low	High	Low
**H3K4me3**	88.5*	49.2	81.1	46.5	81.7	41.5
	2^e-167^		6^e-118^		2^e-65^	
**AcH3**	67.3	31.6	71.6	29.7	72.4	29.7
	2^e-114^		3^e-159^		4^e-165^	
**H3K27me3**	9.9	56.4	7.7	47.1	10.1	53.8
	4^e-230^		2^e-185^		6^e-207^	

*Frequency of marks is shown as % of the genes either with high or low expression.

### Histone marks H3K4me3 and AcH3 alone are unstable

**Figure 4:**
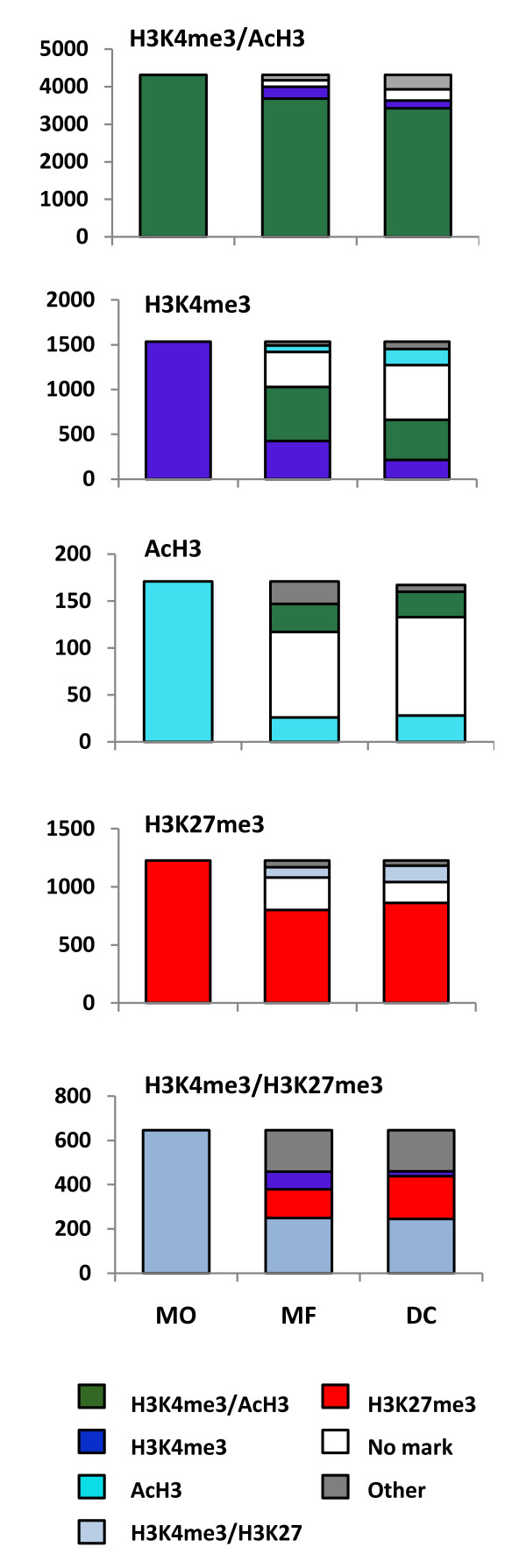
**Dynamics of histone modification and their combinations**. Gene sets with indicated histone modifications or the combination of modifications present in monocytes (MO) were analyzed further in macrophages (MFs) and DCs. The scale indicates the number of genes in each set in monocyte population.

Histone modifications are highly dynamic. We therefore analyzed the dynamics of histone modifications during differentiation from monocytes to macrophages or DCs. When we followed the presence of histone modifications alone or in combination with each other during differentiation, we found that the most prevalent mark characteristic for active genes, the double H3K4me3/AcH3, often persisted on genes throughout the differentiation process (Figure 4). However, we noted a striking decrease of H3K4me3 or AcH3 modifications when occurring alone. For example, when present alone in monocytes, the H3K4me3 mark was usually lost or turned into double mark by an additional acetylation of 72% or 86% of corresponding genes in macrophages or DCs, respectively. Similarly, 84-85% of genes with the AcH3 mark alone in monocytes lost this modification status during differentiation to macrophages or DCs. In contrast, the H3K27me3 modification alone was relatively stable but less so when occurring in combination with H3K4me3 (Figure 4). Together, these results demonstrate a dynamic nature of histone modifications during differentiation, and that the H3K4me3 and AcH3 modifications alone lead to either a loss of one of these marks or a gain of the H3K4me3/AcH3 combinatorial mark.

### Modifications on marker genes often correlate with transcriptional activity

**Figure 5:**
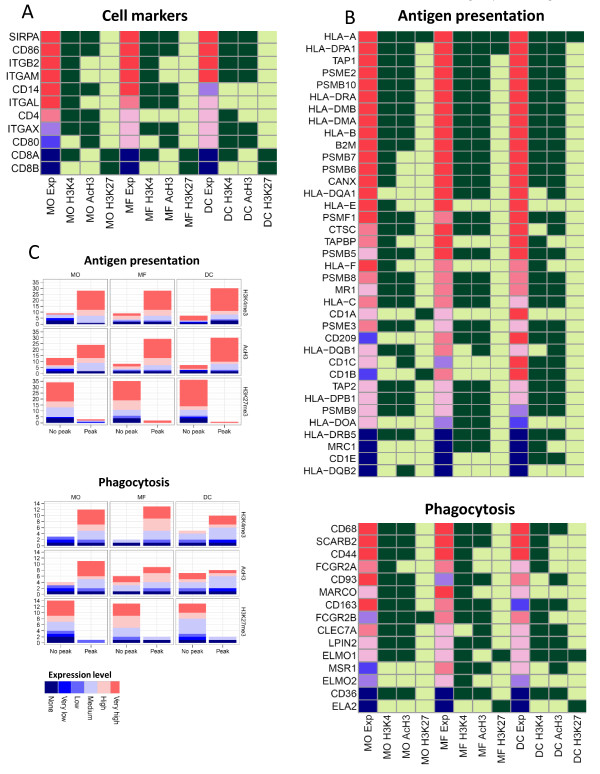
**Comparison of gene expression levels and H3K4me3, AcH3 and H3K27me3 modifications among functional gene groups in monocytes, macrophages and DCs**. Histone modifications and their correlation with expression levels among (A) individual genes of cell markers and (B) genes involved in antigen presentation and phagocytosis. (C) Functional gene groups of antigen presention and phagocytosis according to their expression levels and histone modification status in three cell populations. Color scale indicates expression level from no expression (blue) to high expression (red). Dark green represents the presence and light green the absence of the modification.

To study the chromatin changes of genes associated with macrophage and DC differentiation, we analyzed the histone modifications on the promoters of several well-established marker genes (Figure 5A). For example, CD14 and CD4 genes had double H3K4me3/AcH3 mark on their promoters in monocytes, where they are expressed at high levels, but in agreement with their transcriptional downregulation, they lost these marks after differentiation. CD8A and CD8B genes were not expressed in any of these cells and were marked by repressive H3K27me3 modification. The gene encoding costimulatory ligand CD86 is relatively highly expressed in all three cell types and has double H3K4me3/AcH3 mark but functionally related CD80 gene, which is expressed at lower level, was marked by AcH3 only. Also, the highly expressed integrin marker ITGAM/CD11b had the H3K4me3 modification in monocytes as well as macrophages and acquired the additional AcH3 mark in DCs. The expression of ITGAL/CD11a decreased during differentiation with a corresponding loss of H3K4me3 and AcH3 marks in DCs. Active H3K4me3/AcH3 chromatin marks were also present on integrin ITGAX/CD11c and SIRPA genes. In contrast, the expression of CD40 and CD83 genes did not correlate with histone modifications (data not shown).

### Genes related to phagocytosis and antigen presentation have active chromatin marks

Phagocytosis and antigen presentation are the two most central functions of monocytes and monocyte-derived cells. We therefore studied the histone modifications of genes involved in these two key categories of antigen presenting cells in correlation with their expression levels. Out of 15 genes associated with phagocytic function, the double H3K4me3/AcH3 mark was present on 11 promoters in monocytes, on 8 promoters in macrophage and on 6 promoters in DC populations (Figure 5B, C). Also, a majority of the 37 genes associated with antigen presentation, including HLA (HLA-A, HLA-B, HLA-C, HLA-DRA and HLA-DRB5, HLA-DPA1, HLA-DPB1, HLA-DMA and HLA-DMB), beta-2-microglobulin (B2M), proteasome-associated (PSMB5, PSMB8, PSMB9, PSMB10, PSMF1, PSME2, and PSME3) and peptide transporter (TAP1 and TAP2) had the double H3K4me3/AcH3 mark on their promoters (23, 28 and 27 genes in monocytes, macrophages and DC, respectively) (Figure 5B, C). These data are in good agreement with the general functionality of antigen-presenting cells, where monocytes and macrophages are more active in phagocytic activities while macrophages and DCs are more efficient in antigen presentation.

### A majority of active transcription factor promoters have the H3K4me3 mark

We next studied histone modifications on genes associated with transcriptional processes. A majority of the expressed transcription factor genes had the H3K4me3 modification on their promoter, often together with the AcH3 mark, and a few with the H3K27me3 (Additional file 1, Figure S4). More specifically, the upregulated transcription factors BHLHE41/BHLHB3 (basic helix-loop-helix family, member e41), EGR2 (early growth response factor 2), EPAS1/HIF2A (hypoxia-inducible factor 2 alpha), and MSC (musculin, activated B cell factor 1) had the bivalent H3K4me3/H3K27me3 mark in monocytes; however, the H3K27me3 mark was lost in macrophages and DCs. Other upregulated transcriptional factors such as PIR (pirin, iron-binding nuclear protein), ESR1 (estrogen receptor), HES6 (hairy and enhancer of split 6) and IRF4 (interferon responsive factor 4) and KLF5 (Kruppel like factor 5) maintained the H3K27me3 mark even after their upregulation during differentiation. Interestingly, the ETS family member EHF/ESE-3 did not have any modification. Notably, many transcription factor families involved in differentiation or lineage-specific regulation in other cell types or tissues were marked with the silencing H3K27me3 mark (Additional file 1, Figure S5). For example, 38/39 HOX, 20/30 FOX, 11/195 SOX, 9/9 PAX, 8/8 LHX, 5/6 SIX, 8/11 POU, 6/6 NKX, 5/6 IRX, 6/6 NEURO and 5/6 GATA family transcription factors were positive for the H3K27me3 modification in all three cell populations. This also correlated with the overall transcriptionally inactive status in all three cell types.

Next we used the program Opossum to compare the occurrence of transcription factor binding motifs in gene promoters with and without histone modifications. We found a remarkable enrichment of ETS and C2H2 zinc finger family transcription factor sites in gene promoters enriched with AcH3 modifications. The genes with H3K4me3 marks were enriched for homeobox and high mobility group (HMG) box transcription factor binding sites. It is likely that the presence of these transcription factors binding sites reflects their activity in recruitment of histone modifier proteins to the promoters [21,22].

### The H3K4me3 modification correlates with the expression in gene clusters

**Figure 6:**
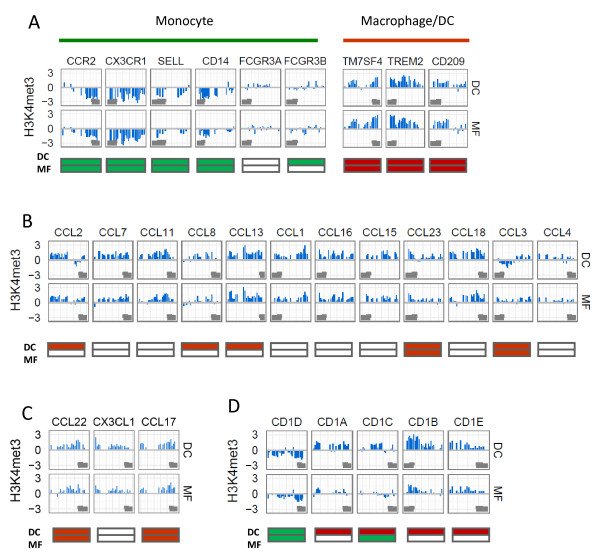
**Changes of H3K4me3 modification on selected gene promoters in DCs and macrophages compared to monocytes**. Changes are shown as log2 differences in peak intensities either in DCs or macrophages (MF) compared to monocytes and are presented either as gain (positive values) or loss (negative values) of the modification. The filled gray shapes represent the positions of transcripts. The red and green colors in boxes indicate up- and downregulation compared to the mRNA levels in monocytes. H3K4me3 modification changes of (A) cellular marker genes of monocytes, DCs and macrophages, (B, C) chemokine gene clusters on chromosome chr17:29,606,409-31,457,127 and chr16:55,950,219-56,007,475, respectively, and (D) CD1 cluster on chromosome chr1:156,490,551-156,593,967.

We noticed that while many genes had lower peak scores, they still gained or lost their histone modifications during differentiation. This was particularly evident with many cell surface marker and chemokine genes. To identify these changes on all promoters, we subtracted the monocyte-specific peak scores from those identified in macrophages or DCs. This additional normalization identified further changes that were not apparent by standard peak identification. The profiling by Gene Ontology indicated that the gain of H3K4me3 mark often occurs on genes associated with signal transduction, inflammation and chemokine activity both in macrophages and DCs after the differentiation whereas the AcH3 was more associated with cell cycle, general mRNA processing and gene expression regulation (Additional file 2, Table S2). We then studied changes among specific gene groups that were up- or downregulated during differentiation. The H3K4me3 and AcH3 modifications were clearly decreased among monocyte marker genes, such as CD14, CCR2, CX3CR1 and SELL. In both macrophage and DC subsets, we found a prominent increase in H3K4 methylation among their marker genes TM7SF4/DC-STAMP, TREM2 and CD209/DC-SIGN (Figure 6A). In addition to these individual genes, we identified several gene families that showed either a gain or loss of the H3K4me3 mark. Many of these gene families were located in genomic clusters. For example, we noticed that a gene clusters encoding upregulated chemokines CCL2, CCL3, CCL13 and CCL23 on chromosome 17q11.2 and CCL17, CX3CL1 and CCL22 on chromosome 16q13 predominantly showed the increased H3K4me3 levels in macrophages and DCs (Figure 6B, C). Furthermore, the H3K4 methylation levels were modified in several other gene clusters associated with inflammatory and cell-cell interaction functions. We found increased H3K4 methylation in the clusters for: 5 family members of the CD1 transmembrane proteins that are structurally related to HLA, 3 complement subunit C1Q genes and 9 genes belonging to the SLAM family receptors that modulate T-cell responses as costimulatory signaling proteins (Figure 6D; Additional file 1 Figure S6). Genes from these clusters were all upregulated in macrophages and DCs. In contrast, the genes from clusters of the CD300 inhibitory receptor family and the IFITM genes encoding interferon-inducible membrane proteins were downregulated and showed reduced H3K4me3 levels in macrophages and DCs (Additional file 1, Figure S6). Interestingly, in many of these clusters we found that the H3K4me3 changes were similar in macrophages and DCs in spite of their differences in expression levels. This suggests that chromatin changes are needed to induce a permissive chromatin state on gene promoters during differentiation, and that the mRNA expression levels of each individual gene is further regulated by other factors.

To confirm the ChIP-chip data, we studied the H3K4me3 modification on several marker gene promoters by conventional ChIP assay. As expected, the CD14 gene showed reduced whereas the TM7SF4/DC-STAMP, TREM2, CD209/DC-SIGN and CD1A gene promoters demonstrated increased levels of H3K4me3 (Additional file 1, Figure S7). Similarly, the chemokine genes CCL13, CCL23, CCL17 and CCL22 showed strongly elevated levels of the H3K4me3 marks. This increase in H3K4me3 on chemokine genes was also present in macrophages, albeit at lower level. Taken together, our results demonstrate that several marker genes and clustered gene families, including cell surface receptors and CC chemokines that are activated during differentiation into macrophages and DCs, show the increase in H3K4 methylation on their promoters.

## Discussion

The recent advent of new technologies analyzing genome-wide chromatin changes has greatly improved our understanding of gene regulation and control. These global approaches provide a large-scale picture to the extent of changes and can be used for further dissection of gene-specific modifications. Our genome-wide mRNA expression profile and map of three major histone modifications associated with gene expression in monocytes, macrophages and DCs are an addition to previous studies in immune cells [23-27].

We found a high concordance rate between two active promoter marks, H3K4me3 and AcH3, in all three populations. Furthermore, the overall density of H3K4me3 and AcH3 marks on promoter regions displayed a similar pattern, with two elevated peaks surrounding transcription start site and a significant decline at the point of transcription, representing reduced levels of H3K4me3 and AcH3 in a region covered by RNA polymerase II and other regulatory proteins [28,29]. As the respective peaks of H3K4me3 and AcH3 marks by and large coincide, it is likely that they also often co-occur in the same nucleosomes. Thus, our data confirm a tight association of H3K4me3 and AcH3 modifications with permissive chromatin for transcription in antigen-presenting cells. In contrast, H3K27me3 had an overall very low concordance with active marks and was equally distributed over the promoter regions with only slight decline at transcription start site.

The H3K4me3 mark was clearly a predominant modification on gene promoters, and most commonly was coupled with AcH3. This is in agreement with the earlier reported genome-wide studies from embryonic stem cells [30,31], CD4+ and CD8+ T cells [24-26], all reporting H3K4me3 as the most characteristic modification in promoter regions. The H3K4me3 mark was also strongly correlated with the transcriptional activity of a gene. Interestingly, we noticed a gradual decrease of the H3K4me3 mark among active genes during the differentiation of monocytes to DCs, and accordingly, the number of genes without any modification increased. It should be noted that the global number of genes with a detectable expression signal between the three cell populations did not change significantly. This suggests that during differentiation, a subset of genes lose their H3K4me3 mark independently of their gene expression and also hints at a highly dynamic nature of histone modifications during differentiation. When studying the dynamics of histone modifications, we found that both the H3K4me3 and AcH3 marks are unstable when they occur alone and are prone to either lose the modification (i.e. become a promoter without mark) or to gain another permissive mark. In contrast, having the combination of both the H3K4me3 and AcH3 marks in a gene promoter was highly stable, and the majority of genes having this combination of histone marks in monocytes also had it in macrophages and DC. The overall decrease of the H3K4me3 mark and the reciprocal increase of genes with or without the H3K27me3 mark in the DC population suggests that the *ex vivo *differentiation in the presence of IL-4 induces a global shift towards less active chromatin.

At the single gene level we found many differences in histone modification profiles, in particular between monocytes and *in vitro *cultivated cells, which correlate with their transcriptional changes. As expected, the expression levels of many marker genes showed a good correlation with the presence of active histone modifications. When we analyzed the gain or loss of marks during differentiation, we found a remarkable decline in the H3K4me3 mark on several monocyte-specific marker genes and a corresponding increase in promoters of DC-specific genes. The prominent exceptions were the CD83 and CD40 genes, which were not associated with any histone mark. Similar to marker genes, the genes associated with antigen presentation activity and phagocytosis were expressed at high levels and had either the H3K4me3 or AcH3 mark. Notably, genes that are associated with phagocytosis had more permissive H3K4me3/AcH3 mark in their promoters in the monocyte population, whereas the genes associated with antigen presentation had more H3K4me3/AcH3 mark in macrophages and DCs. Although there was no dramatic difference between the cell populations in general, this correlation of histone modifications argues in favor of the role of histone modifications in these two key functions of antigen-presenting cells. In case of transcription factors, the situation was more complex, and in many cases the pattern of transcriptional activity of the gene did not match with expected histone modifications. However, the silencing H3K27me3 modification marked a vast majority of transcription factors involved in development or lineage decision in other cell types.

In our analysis, we identified several genomic clusters with changes in their chromatin status suggesting that histone modifications in genomic loci are coordinately regulated. In particular, we found that in macrophages and DCs, H3K4me3 levels increased in a large genomic cluster of proinflammatory and chemotactic CC chemokines on chromosomes 17q11.2 and 16q13 [32]. Typically, CC chemokines attract mononuclear cells and/or are induced in an inflammatory environment, thus our findings suggest the requirement of histone modifications on chemokine gene clusters for their responses to inflammatory situations. These changes in H3K4me3 modifications were not limited to chemokine gene clusters as we found similar changes in other gene clusters involved in inflammatory processes (CD1, C1Q, SLAM, CD300 and IFITM families). Interestingly, we noted that even when the gene expression levels in macrophages and DCs differed, the histone marks in these two cell populations were often similar. This indicates that the histone modification status might be established before the actual expression of the gene and serve as a prerequisite for rapid activation by gene-specific transcription factors upon inflammatory stimuli. However, changes in chromatin modifications may also occur during subsequent activation steps. For example, studies in murine bone marrow derived macrophages showed that genes induced by TLR4 stimulation can be divided into two subclasses based on their gene-specific activation and histone H4 acetylation [33]. Our work here provides a basis for the further studies to address the role of histone modifications in chemokine and other gene clusters in human macrophages and DCs after the activation with inflammatory stimuli.

## Conclusion

The results of genome-wide promoter analysis of H3K4me3, H3K27me3 and AcH3 histone modifications in monocyte-derived macrophage and DCs show specific correlation between the expression of functional gene groups, including marker and chemokine genes, and their chromatin state on promoters. Our data describe several differences of H3K4me3 modification profiles in monocytes, macrophages and DCs but also suggest that the chromatin status of functionally important gene clusters is coordinately modified. Together, the results provide a functional correlation between gene expression and histone modifications in monocyte-derived macrophages and DCs.

## Methods

### Cell culture

Monocytes were purified from freshly collected buffy coats obtained from blood donors of Tartu University Hospital's Blood Center. The study was approved by the Ethics Review Committee on Human Research of the University of Tartu, Estonia (protocol numbers 170/T-7 and 182/M-1). All participants were older than 18-years and gave written informed consent. PBMC were prepared by density gradient centrifugation on Ficoll-Paque™PLUS (GE Healthcare Bio-Sciences AB). Monocytes were purified by positive sorting using anti-CD14-conjugated magnetic microbeads and by two runs through LS columns (both from Miltenyi Biotech) to obtain purities over 99%. The cells were differentiated into macrophages using 50 ng/ml GM-CSF and into DCs using 50 ng/ml GM-CSF and 25 ng/ml IL-4 (both from PeproTech) for 6 d at 1 million cells/ml in RPMI 1640 supplemented with 2 mM L-glutamine, 100 U/ml penicillin, 100 μg/ml streptomycin and 10% FCS (all from PAA). The expression of surface proteins on monocytes, macrophages and DCs were analyzed using fluorescence conjugated antibodies CD14, DC-SIGN, CD80 and CD83 (Miltenyi) and FACSCalibur (BD Biosciences) to confirm the characteristic phenotype (Additional file 1, Figure S8).

### Illumina gene expression array

RNA was extracted using the Trizol reagent (Invitrogen) and RNeasy Mini Kit (Qiagen). The concentration of RNA was assessed with NanoDrop ND-1000 spectrophotometer (NanoDrop Technologies) and the quality with Agilent RNA 6000 Nano Kit and Agilent 2100 Bioanalyzer (Agilent Technologies). RNA integrity numbers (RIN) indicated good quality of RNA, ranging from 6.5 to 9.3. Illumina TotalPrep RNA amplification Kit (Ambion Inc.) was used to amplify 330 ng RNA for hybridization on Illumina Human-6 v2 BeadChips (Illumina). The data were analyzed with BeadStudio Gene Expression Module v3.3.7 (Illumina) using Illumina's custom rank invariant method. The complete lists of upregulated and downregulated genes are available at ArrayExpress databank with accession number E-TABM-976 (see also Additional file 2, Tables S3, S4).

### Chromatin immunoprecipitation (ChIP)

ChIP with some modifications was performed according to Upstate Chromatin Immunoprecipitation Assay protocol (Upstate). Briefly, formaldehyde crosslinking was carried out in PBS containing 1% formaldehyde and 0.5 mM EGTA with the density of 7x10^7 ^cells in 70 ml. To process monocyte samples in parallel with macrophages and DCs, monocyte samples were crosslinked, lysed and subjected to sonication in day one and the lysates were kept at 4°C until macrophage and DC samples were collected. About 1x10^7 ^cells, 30 μl of packed protein G sepharose beads (GE Healthcare; pre-absorbed with 100 μg/ml BSA and 500 μg/ml of sheared salmon sperm DNA) and 4 μg of H3 (Abcam, ab1791), H3K4me3 (Abcam, ab8580), H3K27me3 (Upstate, 07-449) or AcH3 (Upstate, 06-599) antibodies were used in each immunoprecipitation. DNA probes from ChIP were amplified using GenomePlex Complete Whole Genome Amplification (WGA) Kit and reamplified using GenomePlex WGA Reamplification Kit (both from Sigma-Aldrich). NimbleGen Systems using 385K RefSeq Promoters array set performed sample labelling, hybridization and data extraction. The NimbleGen RefSeq Promoter array data is available at ArrayExpress databank with accession number E-TABM-979 (see also Additional file 2, Tables S5, S6).

For conventional ChIP, purified DNA samples were analyzed in triplicate by quantitative PCR (qPCR) as described in [34]. In addition, datasets of each primer pairs were normalized to monocyte H3 values. The primers were designed with MPrimer3 http://bioinfo.ut.ee/mprimer3/[35] and are shown in Additional file 2, Table S1.

### Bioinformatic analyses

The intensities of the histone H3, H3K4me3, H3K27me3 and AcH3 probes were first normalized to the random genomic background. We additionally normalized the modification intensities by subtracting H3 intensities from the corresponding modification intensities to get specific values for each modification. The normalized values correspond to log2 ratio between corresponding modification and H3 signals. We used the same normalization to find the changes induced by differentiation but then we subtracted the values of probe intensities in monocyte from corresponding probe intensities in macrophage and DCs. Both sets of data were further used to find peaks in the promoters. The peaks were mapped to the genes, where they fell into the range 2000 bp upstream to 500 bp downstream from the transcription start site. The peak scan and assembly with normal RefSeq genes for human genome (HG18, NCBI Build 36) was performed using Nimblescan 2.5 software with default settings. The number of transcript probes in Illumina gene expression matrix was 42620 and the Nimblegen promoter database contained 19222 genes. In the analyses combining gene expression and promoter modification data, the overlap between these two sets (17289 genes) was used. The genes for functional classes were selected from Immunome Database http://bioinf.uta.fi/Immunome/[36] and the list of transcription factors was accumulated using Ingenuity Pathway Analysis software http://www.ingenuity.com/[37].

The transcription factor analysis was performed using web resource Opossum [38], which identifies transcription factor binding motifs that are overrepresented in input genes compared to background of all genes. We used only vertebrate motifs that are most conserved from the top 10% of the most conserved regions (minimum conservation 70%). The matrix match threshold was 80% of the maximal match score. The significance threshold was combined from Z-score, calculated assuming binomial distribution of total number of motif occurrences, and Fisher one-tail test p-value, comparing number of promoters having the motif. The corresponding thresholds were 10 for Z-score and 0.01 for Fisher test p-value. The promoters used in this case were from 2000 bp upstream to transcription start site, as this choice was in the best agreement with the tiling chip promoter areas.

The functional profiling for the groups of genes with changes in the studied modifications was carried out using the g:GOSt tool at g:Profiler website http://biit.cs.ut.ee/gprofiler/[39] that retrieves most significant Gene Ontology (GO) terms, KEGG and REACTOME pathways [40].

## Abbreviations

AcH3: acetylation of histone H3 lysines; ChIP: chromatin immunoprecipitation; DC: dendritic cell; FCS: fetal calf serum; GM-CSF: granulocyte macrophage colony-stimulating factor; HLA: human leukocyte antigen; H3K27me3: histone H3 trimethylation of lysine 27; H3K4me3: histone H3 trimethylation of lysine 4; IL-4: interleukin-4; MF: macrophage; MO: monocyte; PBMC: peripheral blood mononuclear cell; RIN: RNA integrity number

## Authors' contributions

LT and RK performed experiments and analyzed data; AR, KK and TO designed research and analyzed data; HP and JV contributed analytical tools for bioinformatics analyses; PP designed the research and wrote the manuscript. All authors have read and approved the final manuscript.

## Supplementary Material

Additional file 1 **Tserel et al BMC Genomics**. Contains Supplementary Table S1 and Supplementary Figures S1-S8. Size 1.2 MB Click here for file

Additional file 2 **Tserel et al BMC Genomics**. Contains Supplementary Tables S2-S6. Size 7.2 MB Click here for file
