# Supplementary material for: Genome-wide promoter analysis of histone modifications in human monocyte-derived antigen presenting cells
Source: BMC Genomics. 2010 Nov 18;11:642. doi: 10.1186/1471-2164-11-642 (PMC3091769; doi:10.1186/1471-2164-11-642)
Supplement: Additional file 1 — Tserel et al BMC Genomics. Contains Supplementary Table S1 and Supplementary Figures S1-S8. Size 1.2 MB [file 1471-2164-11-642-S1.PDF]

Supplementary Table 1. qPCR primers

Gene	Forward sequence	Reverse sequence
CCL13	AGGAGCCCAGGAATGGGCCAAA	TTGATGCAGGCCCCGTGTTTCC
CCL17	AGCTGTGCGTGGAGGCTTTTCA	ACAGGGGTTGGGGGCATGAAGA
CCL22	TGCAGACACCTGGGCTGAGACA	GCCTCAGTTGCTTGAAGCGCCA
CCL23	ACGACATCCCGTAATGTACAGCCCA	TCCCATGTGCTCATCCTTGCCCT
CD1A	TGCCACATCAGACTTGTTCCATAGCAG	CCAGCTTCTCTCCTTGTCACCAACC
CD14	TGCAGGGCATCTAGGGTTCTGTGT	AGTCAACAGGGCATTCACCGCC
CD209	TCCTGGAAGCACTGTCCCCTGT	GCCCCAACTCCCAAAGTCAGCA
TM7SF4	AGAGGCGGCTCCTACGAACCAT	TCCCCACATCCACTCGAGCCAT
TREM2	GCCCTGCACTGCTCAGTTTCCT	TGCAGCTGGTGGAGGGTCTGAA

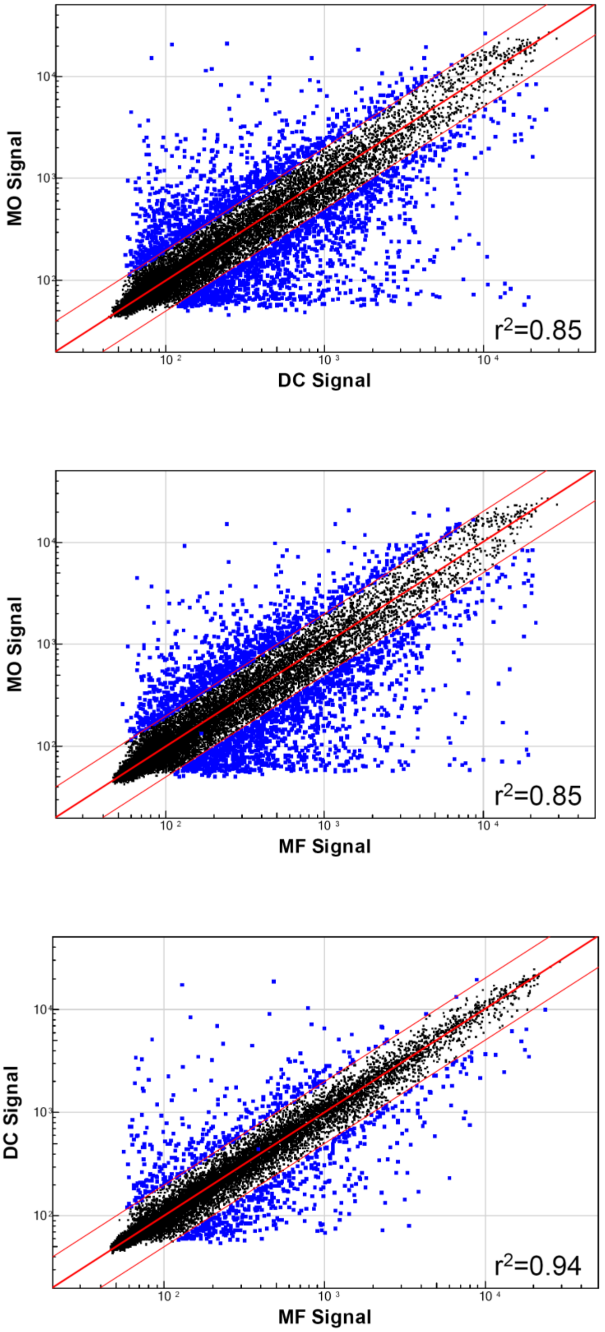

Supplementary Figure 1. Gene expression profiles of monocytes, macrophages and DCs. The expression profiles are shown as scatter blots of (A) monocytes vs macrophages, (B) monocytes vs DCs and (C) macrophages vs DCs. The scatter blots display gene expression values for all probes on Illumina Human-6v2 BeadChips in logarithmic scale. The blue dots indicate genes with fold change value over 2. The r2 indicates correlation coefficent between two samples compared.

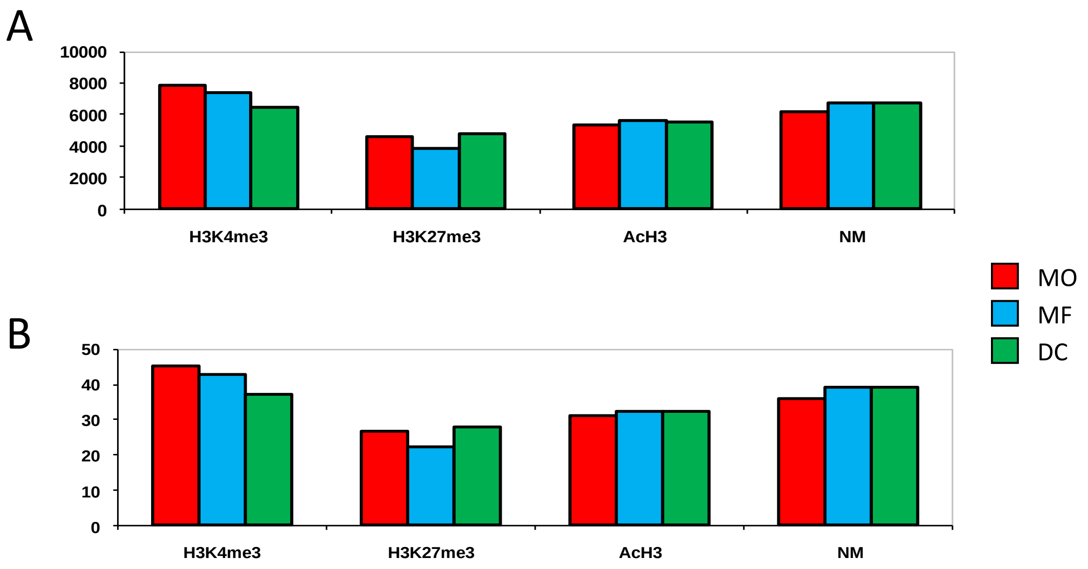

Supplementary Figure 2. The presence of H3K4me3, H3K27me3 and AcH3 in monocyte (MO), macrophage (MF) and DC subpopulations. (A) Total number of the gene promoters which posess the respective modification or no mark (NM) is shown. (B) The presence of modification is presented as percentage of positive genes.

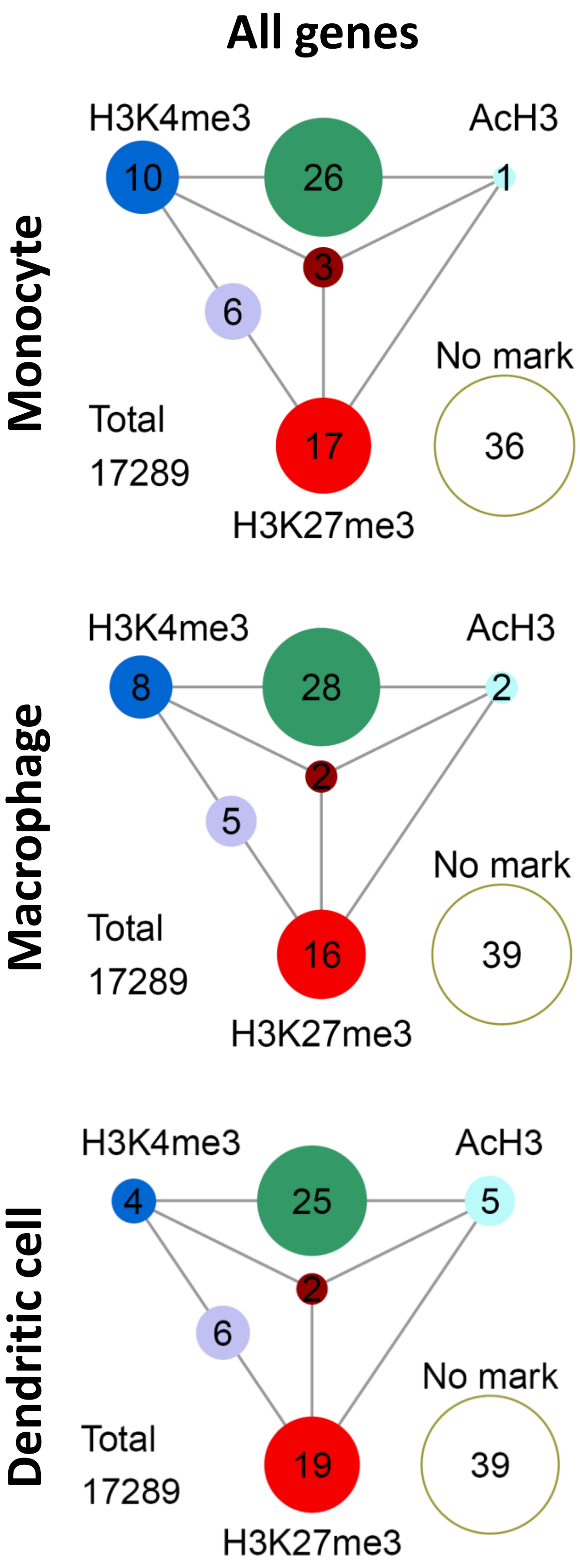

Supplementary Figure 3. Frequencies of H3K4me3, AcH3 and H3K27me3 modifications among all genes. Frequencies of modifications are shown as percentages in scaled circles positioned to outer corners of the triangles. Frequencies of co-occuring modifications are shown in between. Studied cell types are indicated left. "No mark" represents the lack of histone modifications analyzed in this study.

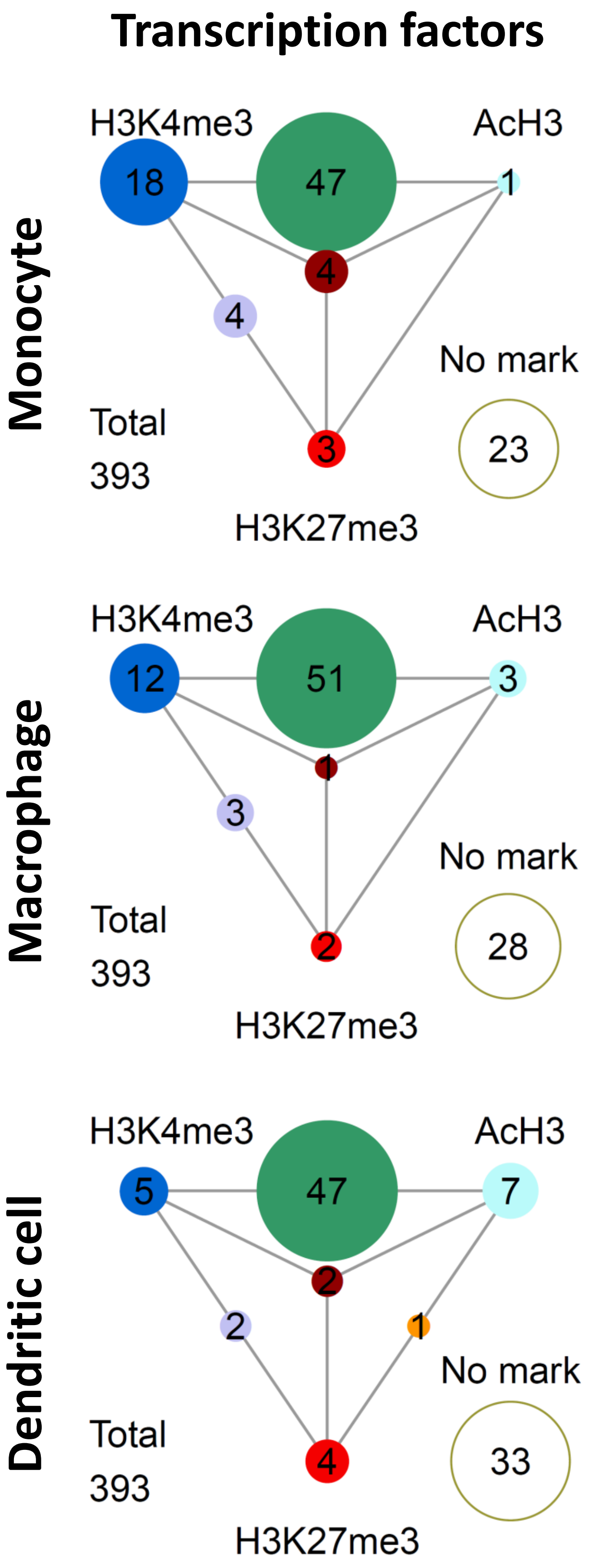

Supplementary Figure 4. Frequencies of H3K4me3, AcH3 and H3K27me3 modifications and their combinations among transcription factors. Frequencies of modifications are shown as percentages in scaled circles positioned to outer corners of the triangles. Frequencies of co-occuring modifications are shown in between. Studied cell types are indicated left.

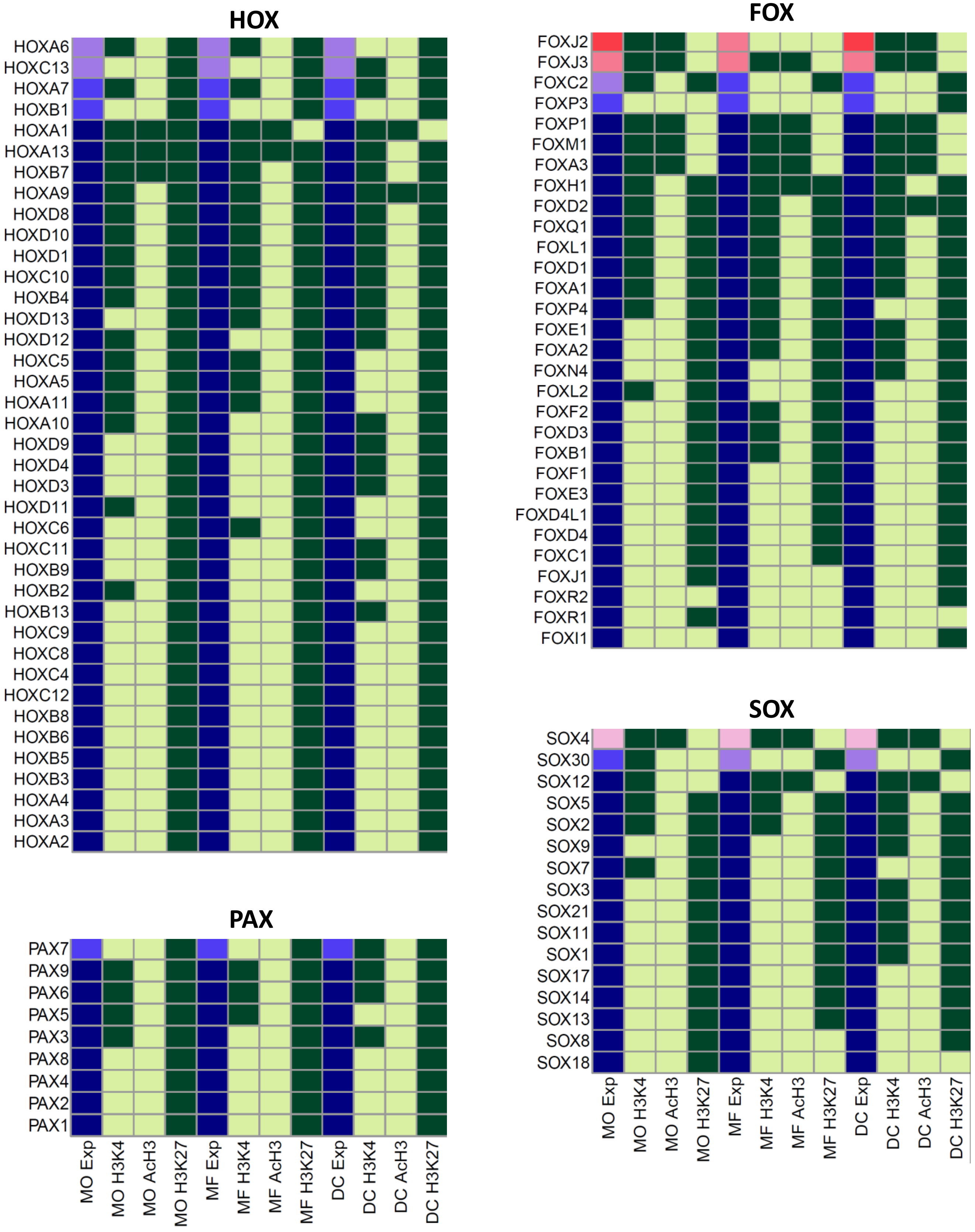

Supplementary Figure 5. Comparison of gene expression level and H3K4me3, AcH3 and H3K27me3 modifications of differentiation and lineage specific transcription factors of other cell types and tissues. Color scale indicates expression level from no expression (blue) to high expression (red). Dark green represents the presence and light green the absence of the modification.

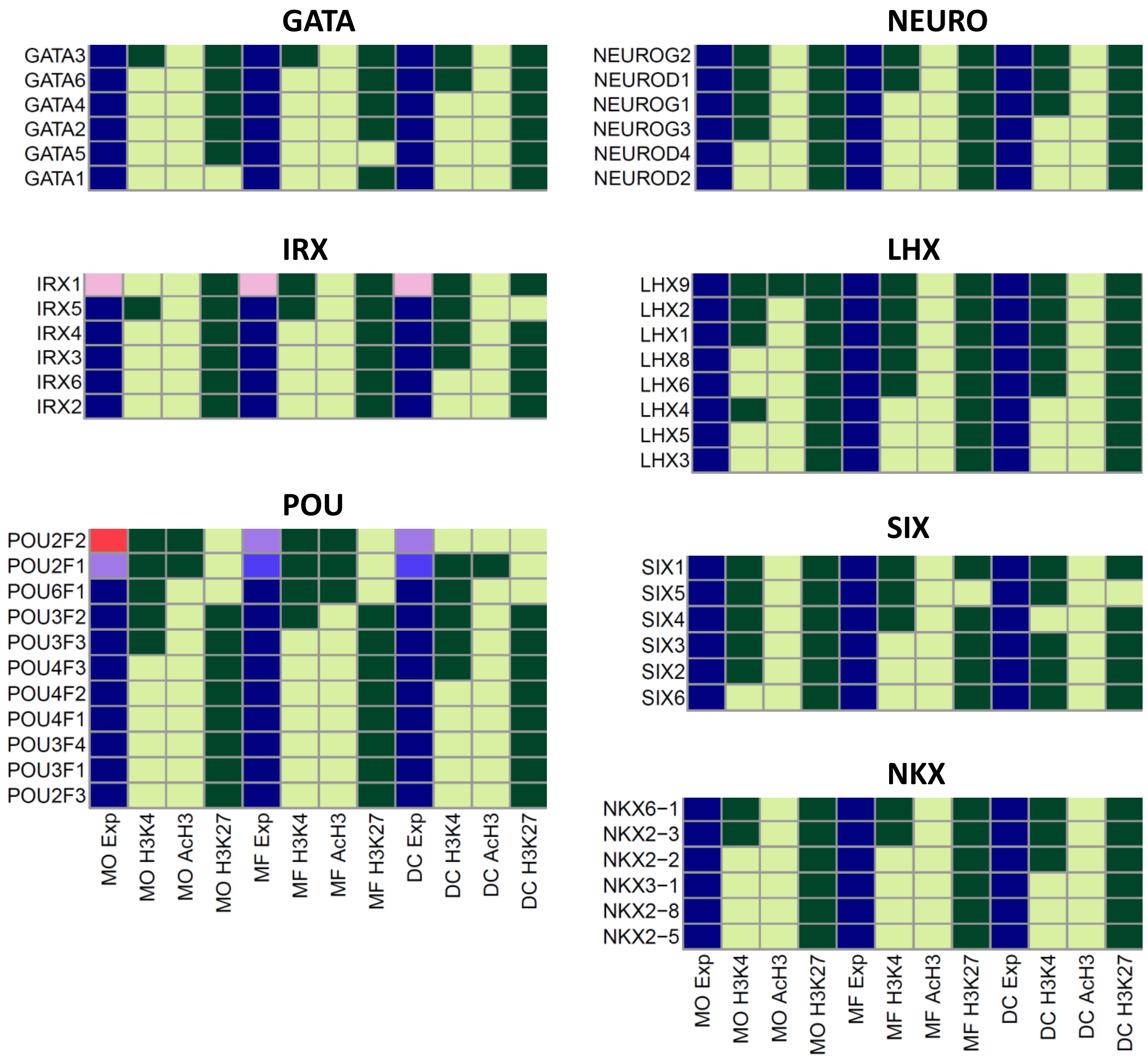

Supplementary Figure 5 (continued). Comparison of gene expression level and H3K4me3, AcH3 and H3K27me3 modifications of differentiation and lineage specific transcription factors of other cell types and tissues. Color scale indicates expression level from no expression (blue) to high expression (red). Dark green represents the presence and light green the absence of the modification.

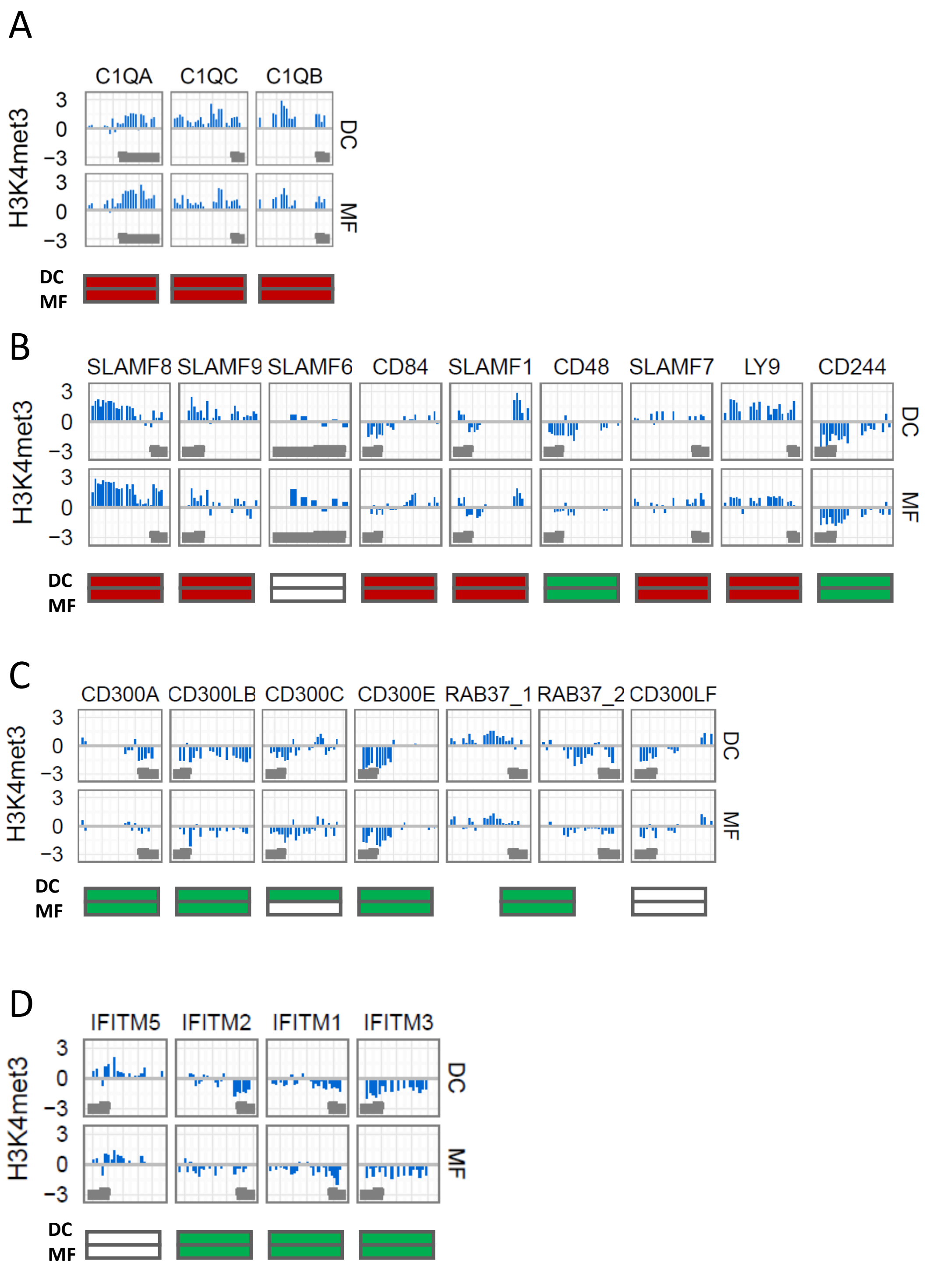

Supplementary Figure 6. Changes of H3K4me3 modifications in gene clusters associated with inflammatory functions. The H3K4me3 levels in (A) C1Q (chr1:22,835,705-22,860,616), (B) SLAMF (chr1:158,063,103-158,073,906), (C) CD300 (chr17:69,974,117-70,220,703) and (D) IFITM (chr11:288,203-310,914) gene clusters in MFs and DCs are shown compared to the levels in monocytes. Changes are shown as log2 differences in peak intensities and are presented either as gain (positive values) or loss (negative values) of corresponding modification. The filled gray shapes represent the position of transcripts. The red and green colors in boxes indicate up- and downregulated genes, respectively.

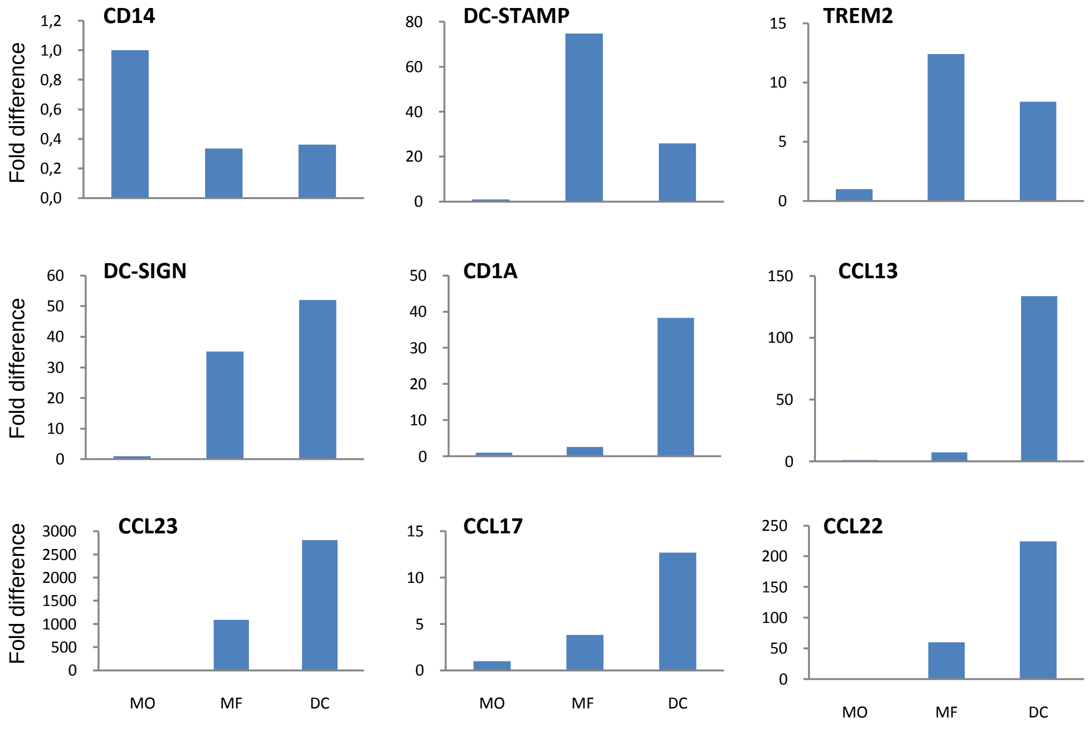

Supplementary Figure 7. Conventional ChIP analysis of H3K4me3 modifications on selected genes. Immunoprecipitated chromatin from MO, MF and DC cell populations was analyzed by qPCR using primers specific to indicated gene promoters. Fold difference shows relative level of H3K4me3, is normalized to input and histone H3 values and is presented compared to the respective level in monocytes (=1). The presented data represents one from three independent experiments carried out on cells from different donors.

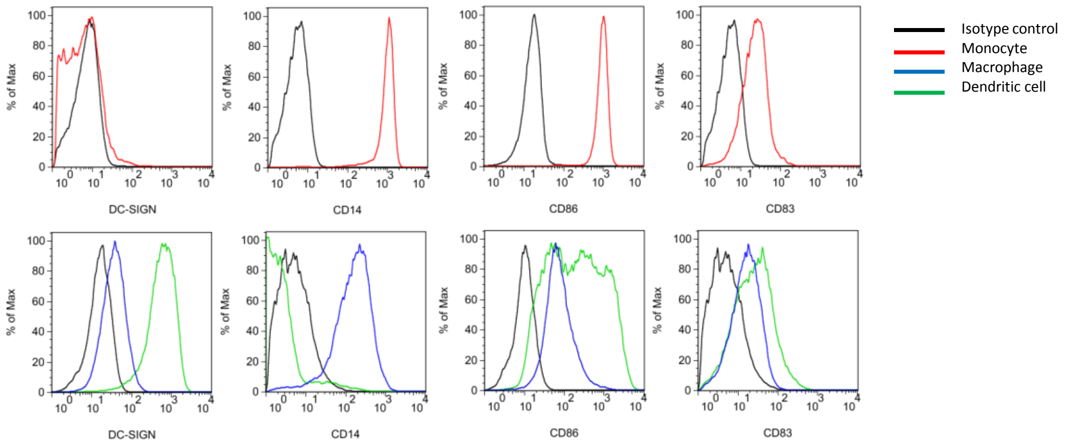

Supplementary Figure 8. Flow cytometry analysis of MO, MF and DC subsets. Mean fluorescence intensities of CD14, DC-SIGN, CD86 and CD83 are shown as % of maximum. The black lines indicate isotype control which is a measurement of unlabeled cells using the same settings as for the labeled cells.
