# Supplementary material for: Genome-wide promoter analysis of histone modifications in human monocyte-derived antigen presenting cells
Source: BMC Genomics. 2010 Nov 18;11:642. doi: 10.1186/1471-2164-11-642 (PMC3091769; doi:10.1186/1471-2164-11-642)
Supplement: Additional file 2 — Tserel et al BMC Genomics. Contains Supplementary Tables S2-S6. Size 7.2 MB [file 1471-2164-11-642-S2.ZIP › Supplementary Table 5. H3 peaks..pdf]

Supplementary Table 5. Histone modifications (normalized to H3) in monocyte, macrophage and dendritic cell subpopulations. (1=peak, 0=no peak).

	DC AcH3	DC H3K27	DC H3K4	MF AcH3	MF H3K27	MF H3K4	mo AcH3	mo H3K27	mo H3K4
15E1.2	1	0	1	1	0	1	1	0	1
76P	1	0	1	1	0	1	1	0	1
A2M	1	0	1	0	0	1	0	0	0
A4GALT	0	1	1	0	1	0	0	1	1
A4GNT	0	0	0	1	0	0	0	0	0
AAAS	1	0	0	1	0	0	0	0	1
AADACL1	1	0	1	1	0	1	1	0	1
AAMP	1	0	1	1	0	1	0	0	1
AARSD1	1	0	1	1	0	1	1	0	1
AASDH	1	0	1	1	0	1	1	0	1
AASDHPPT	1	0	1	1	0	1	1	0	1
AATF	1	1	1	1	1	1	1	1	1
ABCA1	0	0	0	0	0	0	1	1	1
ABCA3	0	1	0	0	1	0	0	1	0
ABCA5	0	0	0	0	0	1	0	0	0
ABCA6	0	0	0	0	0	0	1	0	0
ABCB6	0	0	0	0	0	0	0	1	0
ABCB7	1	1	1	1	1	1	1	0	1
ABCB8	0	0	0	0	1	0	0	0	1
ABCB9	1	0	1	1	0	1	1	0	1
ABCC11	1	0	1	1	0	1	1	0	1
ABCC13	0	1	0	0	0	0	0	1	0
ABCC3	0	0	1	0	0	0	0	1	0
ABCC4	0	1	1	0	1	1	0	1	1
ABCC5	0	0	1	0	0	1	1	0	1
ABCC6	1	1	0	0	1	0	1	1	1
ABCD1	1	1	0	0	0	1	0	1	1
ABCD3	1	0	1	1	0	1	1	0	1
ABCD4	0	0	0	1	0	1	0	0	1
ABCE1	1	0	1	1	0	1	1	0	1
ABCF1	1	0	1	1	0	1	1	0	1
ABCF2	1	0	0	1	0	1	0	1	1
ABCF3	1	0	0	1	0	1	0	0	1
ABCG1	1	1	1	1	1	1	0	1	1
ABCG2	1	0	1	1	0	1	0	1	1
ABCG4	0	1	1	0	1	1	0	1	1
ABHD10	1	1	1	1	0	1	1	0	1
ABHD11	0	0	0	0	1	0	0	0	0
ABHD12	1	0	1	1	0	1	1	0	1
ABHD13	1	0	1	1	0	1	1	0	1
ABHD14A	0	0	1	0	0	1	1	0	1
ABHD14B	0	0	1	0	0	1	1	0	1
ABHD3	1	0	1	1	0	1	0	0	1
ABHD4	1	1	1	1	0	1	1	1	1
ABHD5	0	1	0	0	0	0	0	1	0
ABHD6	0	1	0	0	0	1	0	1	1
ABHD7	0	1	0	0	1	0	0	1	0
ABHD9	0	1	0	0	0	0	0	1	0
ABI1	1	0	0	0	0	0	0	0	0
ABI3	0	0	0	0	0	1	1	0	1
ABL1	1	0	1	1	0	1	1	0	1
ABL2	1	0	1	1	0	1	1	0	1
ABO	0	1	0	0	1	0	0	1	0
ABP1	0	0	0	0	0	0	0	1	0
ABR	0	0	0	0	0	1	1	0	1
ABTB2	0	1	1	0	0	1	0	0	1
ACAA1	1	0	1	1	0	1	0	0	1
ACAA2	1	0	1	1	0	1	1	0	1

ACACA	1	0	1	1	0	1	1	0	1
ACAD10	1	0	1	1	0	1	1	0	1
ACAD11	1	0	1	1	0	1	1	0	1
ACAD8	1	0	1	1	0	1	1	0	1
ACAD9	1	0	1	1	0	1	1	0	1
ACADM	1	0	1	1	0	1	1	0	1
ACADS	0	0	0	0	0	0	1	0	0
ACADSB	1	0	1	1	0	1	1	0	1
ACADVL	0	0	1	0	0	1	0	0	1
ACAT2	1	0	1	1	0	1	1	0	1
ACBD3	1	0	1	1	0	1	0	0	1
ACBD4	0	0	0	0	0	1	0	0	1
ACBD5	1	0	1	1	0	1	1	0	1
ACBD6	1	0	1	1	0	1	1	0	1
ACCN2	0	1	0	0	1	0	0	1	0
ACCN4	0	1	0	0	1	0	0	1	0
ACD	1	0	0	0	0	1	0	0	1
ACE	0	0	0	0	0	0	0	1	0
ACE2	0	1	0	0	1	0	0	1	0
ACIN1	1	0	1	1	0	1	1	0	1
ACLY	1	0	1	0	0	1	0	0	1
ACN9	0	0	1	1	0	1	1	0	1
ACO1	1	1	1	1	0	1	1	1	1
ACO2	1	0	1	1	0	1	1	0	1
ACOT1	0	0	0	1	0	1	0	0	1
ACOT11	0	0	0	0	0	1	1	0	0
ACOT12	0	1	1	0	1	0	0	1	1
ACOT2	1	0	1	1	0	1	0	0	0
ACOT7	1	0	1	0	0	1	1	0	1
ACOT8	1	0	1	1	0	1	1	0	1
ACOT9	1	1	1	1	1	1	1	1	1
ACOX1	1	0	1	1	0	1	1	0	1
ACOX2	0	0	0	1	0	0	1	0	0
ACOX3	1	0	1	1	0	1	0	0	1
ACP1	1	0	1	1	0	1	1	1	1
ACP2	0	0	1	1	0	1	1	0	1
ACP5	0	0	1	0	0	1	0	0	0
ACP6	0	0	1	0	0	1	1	0	1
ACPL2	0	0	0	0	0	0	0	0	1
ACPP	1	0	1	1	0	1	1	0	1
ACRBP	0	0	1	0	0	1	0	0	1
ACRC	0	1	0	0	0	0	0	0	0
ACSL1	1	0	1	1	0	1	1	0	1
ACSL3	1	0	1	1	0	0	1	0	1
ACSL4	1	1	1	1	1	1	1	1	1
ACSL5	1	0	0	0	0	1	0	0	0
ACSM3	1	0	1	1	0	1	1	0	1
ACSS1	0	1	0	0	0	0	1	0	1
ACSS2	1	0	1	1	0	1	1	0	1
ACTB	1	0	1	1	0	1	1	0	1
ACTG1	0	0	0	0	0	0	0	0	1
ACTL6B	0	1	0	0	1	0	0	1	0
ACTN1	0	0	1	0	0	1	0	1	1
ACTR10	1	0	1	1	0	1	1	0	1
ACTR1A	1	0	1	1	0	1	1	0	1
ACTR2	1	0	1	1	0	1	1	0	1
ACTR3	1	0	1	1	0	1	1	0	1
ACTR5	0	0	1	0	0	0	0	1	0
ACTR6	1	0	1	1	0	1	1	0	1
ACTR8	0	0	1	0	0	1	1	0	1
ACVR1	1	0	1	1	0	1	1	0	1
ACVR1B	0	1	0	0	0	0	0	1	0

ACVR2A	1	1	1	1	0	1	0	1	1
ACVR2B	0	1	1	0	1	1	0	1	1
ACVRL1	0	0	1	0	0	0	0	1	0
ACY1	1	0	1	1	0	1	0	0	0
ACY1L2	1	0	0	1	0	1	1	0	1
ACY3	0	1	0	0	0	1	0	0	1
ACYP1	1	0	1	1	0	1	1	0	1
ACYP2	1	0	0	1	0	1	0	0	0
ADA	1	0	1	1	0	1	1	0	1
ADAL	1	0	1	1	0	1	1	1	1
ADAM10	0	0	1	0	0	1	0	0	1
ADAM11	0	1	0	0	1	0	0	1	0
ADAM12	0	1	0	0	1	0	0	1	0
ADAM15	1	0	0	1	0	0	0	0	1
ADAM17	1	0	1	1	0	1	1	0	1
ADAM23	0	1	1	0	1	1	1	1	1
ADAM28	0	0	0	0	0	1	0	0	1
ADAM30	0	1	0	0	0	0	0	0	0
ADAM9	1	0	1	1	0	1	1	0	1
ADAMDEC1	0	0	0	0	0	0	1	0	0
ADAMTS1	0	1	1	0	1	1	0	1	1
ADAMTS17	0	1	0	0	1	0	0	1	0
ADAMTS5	1	1	1	0	1	1	1	1	1
ADAMTS8	0	1	0	0	1	0	0	1	0
ADAR	0	0	1	1	0	1	1	0	1
ADARB1	0	1	0	0	0	0	0	1	0
ADAT1	1	0	1	1	0	1	0	0	1
ADC	0	1	0	0	0	1	0	1	1
ADCK4	1	0	1	1	0	1	1	0	1
ADCY1	0	1	1	0	1	1	0	1	0
ADCY3	0	1	1	0	0	1	0	1	1
ADCY4	0	1	1	0	1	1	0	1	1
ADCY9	0	1	0	0	0	0	0	0	0
ADD1	1	0	0	0	0	0	1	0	1
ADH5	1	0	1	1	0	1	1	0	1
ADHFE1	1	0	1	1	0	1	0	0	1
ADI1	1	0	1	1	0	1	1	0	1
ADIPOR1	1	0	0	1	0	0	0	0	0
ADIPOR2	1	0	1	1	0	1	1	0	1
ADK	1	0	1	1	0	1	1	0	1
ADM	1	0	1	1	0	1	1	0	1
ADM2	0	1	0	0	1	0	0	1	0
ADNP	0	0	0	0	0	0	1	0	0
ADORA2B	0	0	0	0	0	0	0	0	1
ADORA3	0	0	1	0	0	0	0	0	0
ADPGK	1	0	1	1	0	1	1	0	1
ADPRH	1	0	1	1	0	1	1	0	1
ADRA1D	0	1	0	0	1	0	0	1	0
ADRA2A	0	1	1	0	1	1	0	1	1
ADRA2B	0	1	0	0	1	0	0	1	0
ADRA2C	0	1	0	0	0	0	0	1	0
ADRB1	0	1	1	0	1	1	1	1	1
ADRB2	1	0	1	1	0	1	1	0	1
ADRBK2	1	1	1	1	1	1	1	1	1
ADSL	0	0	1	0	0	1	0	0	1
AFF1	1	0	1	1	0	1	1	0	1
AFG3L1	1	0	1	1	0	1	0	0	1
AFG3L2	1	0	1	1	0	1	0	0	1
AFMID	0	0	0	0	0	1	0	0	1
AGA	1	0	1	1	0	1	1	0	1
AGBL3	0	0	0	0	0	1	1	0	1
AGGF1	1	0	1	0	0	1	0	0	1

AGL	1	0	1	1	0	1	1	0	1
AGPAT1	1	0	1	1	0	1	1	0	1
AGPAT3	0	0	0	0	0	0	0	1	0
AGPAT4	1	0	0	0	0	1	1	0	0
AGPAT5	1	0	1	1	0	1	1	0	1
AGPAT6	1	0	0	0	0	0	1	0	1
AGPAT7	1	0	1	1	0	1	1	0	1
AGPS	1	0	1	1	0	1	1	0	1
AGRP	1	0	1	1	0	1	1	0	1
AGTPBP1	1	0	1	1	0	1	1	0	1
AGTR2	0	1	0	0	1	0	0	0	0
AHI1	1	0	1	1	0	1	1	0	1
AHNAK	0	0	0	0	0	1	0	1	0
AHR	1	0	1	0	0	1	0	0	1
AHSA1	1	0	1	1	0	1	1	0	1
AHSA2	1	0	1	1	0	1	1	0	1
AHSG	0	1	0	0	0	0	0	0	0
AIF1	1	0	1	1	0	1	1	0	1
AIG1	1	0	0	1	0	1	1	0	1
AIM1	1	0	1	1	0	1	1	0	1
AIM2	1	0	1	1	0	0	1	0	1
AIP	1	0	1	1	0	1	1	0	1
AIPL1	0	1	0	0	1	0	0	1	0
AK1	0	1	0	0	1	0	0	1	0
AK2	1	0	1	0	0	1	0	0	1
AK3L1	0	1	1	0	1	1	0	1	1
AK7	1	1	1	0	0	1	1	1	1
AKAP1	1	0	1	1	0	1	1	0	1
AKAP10	1	0	1	0	0	0	0	0	0
AKAP11	0	0	0	0	0	0	0	0	1
AKAP12	0	1	1	0	1	1	0	1	1
AKAP13	1	1	1	1	1	1	1	1	1
AKAP14	0	1	0	0	1	0	0	0	0
AKAP5	1	0	1	0	1	1	1	1	1
AKAP7	0	0	0	1	0	0	1	0	0
AKAP8	1	0	1	1	0	0	0	0	0
AKAP8L	1	0	0	1	0	1	1	0	1
AKAP9	0	0	1	1	0	1	1	0	1
AKR1B1	0	0	0	0	0	0	0	1	1
AKR1C3	1	0	1	1	0	1	1	0	1
AKR1CL2	0	0	0	0	0	0	0	1	0
AKR7A2	1	0	1	1	0	1	1	0	1
AKR7A3	0	1	0	0	0	0	0	1	0
AKT1	1	0	1	1	0	1	1	0	1
AKT1S1	1	0	1	1	0	1	0	0	1
AKT2	1	0	0	1	0	0	1	0	1
AKT3	0	0	0	1	0	1	0	0	0
ALCAM	1	0	1	1	0	1	1	0	1
ALDH16A1	1	0	1	1	0	1	1	0	1
ALDH1A1	1	0	1	1	0	1	1	0	1
ALDH1A2	0	1	0	0	1	0	0	1	0
ALDH1A3	0	1	0	0	1	0	0	1	0
ALDH1B1	0	1	0	0	1	0	0	1	1
ALDH1L2	0	0	1	0	0	1	0	0	1
ALDH3B1	0	0	0	0	0	0	0	0	1
ALDH4A1	1	0	1	1	0	0	0	0	0
ALDH5A1	0	1	1	0	1	1	0	1	1
ALDH6A1	1	0	1	1	0	1	1	0	1
ALDH9A1	0	0	0	0	0	0	1	0	1
ALDOA	0	0	0	0	0	0	0	0	1
ALDOB	0	0	0	0	1	0	0	0	0
ALDOC	0	0	0	0	0	1	0	0	0

ALG1	1	0	0	0	0	1	0	0	1
ALG11	1	1	1	1	1	1	1	1	1
ALG14	1	0	1	1	0	1	1	0	1
ALG2	1	0	1	1	0	1	1	0	1
ALG3	1	0	1	1	0	1	1	0	1
ALG5	1	0	1	1	0	1	1	0	1
ALG8	1	0	1	1	0	1	1	0	1
ALG9	1	0	0	0	0	1	0	0	0
ALK	0	1	1	0	1	1	0	1	0
ALKBH1	1	0	1	1	0	1	1	0	1
ALKBH2	1	0	1	1	0	0	0	0	1
ALKBH3	0	1	1	0	1	1	0	1	1
ALKBH4	0	0	0	1	0	1	0	0	0
ALKBH5	1	0	1	1	0	1	1	0	1
ALKBH6	0	0	1	0	0	1	0	0	1
ALKBH7	0	0	1	0	0	1	1	0	1
ALKBH8	1	0	1	1	0	1	1	0	1
ALMS1	1	1	1	1	0	1	0	1	1
ALOX12B	0	1	0	0	1	0	0	1	0
ALOX15	0	1	1	0	1	0	0	1	0
ALOX5	0	1	0	0	0	0	0	1	1
ALOX5AP	1	0	1	1	0	1	1	1	1
ALPI	0	0	0	0	1	0	0	0	0
ALPK2	0	1	0	0	1	0	0	1	0
ALPL	0	1	0	0	1	0	0	1	0
ALS2	1	1	1	1	0	1	1	0	1
ALS2CR13	0	0	1	0	0	0	1	0	1
ALS2CR14	0	0	0	0	0	1	0	1	1
ALS2CR2	1	0	1	1	0	1	1	1	1
ALS2CR4	0	0	0	1	0	0	0	0	0
ALS2CR8	1	1	1	1	1	1	1	1	1
ALX3	0	1	0	0	1	0	0	1	0
AMACR	1	0	1	1	0	1	1	0	1
AMD1	1	0	1	1	0	1	1	0	1
AMDHD1	1	0	1	1	0	1	1	0	1
AMDHD2	0	0	0	0	0	1	0	0	0
AMICA1	1	0	1	1	0	1	1	0	1
AMIGO1	0	1	0	0	0	0	0	0	0
AMIGO2	0	0	0	0	0	1	0	0	1
AMMECR1	0	1	1	0	1	1	0	1	1
AMOTL1	0	1	0	0	1	0	0	1	1
AMPD2	1	0	1	1	0	1	1	0	1
AMPD3	0	0	0	0	0	0	1	0	1
ANAPC1	0	0	1	1	0	1	0	0	1
ANAPC10	1	0	1	1	0	1	1	0	1
ANAPC11	1	0	1	1	0	1	1	0	1
ANAPC13	1	0	1	1	0	1	1	0	1
ANAPC2	1	0	1	1	0	1	1	0	1
ANAPC4	0	0	1	0	0	1	0	0	1
ANAPC5	1	0	1	1	0	1	1	0	1
ANAPC7	1	0	1	1	0	1	1	0	1
ANG	1	0	1	1	0	1	1	0	1
ANGEL2	0	0	1	1	0	1	1	0	1
ANGPT1	1	1	1	1	0	1	1	1	1
ANGPTL4	0	0	0	0	1	0	0	1	0
ANGPTL6	0	0	0	1	0	1	0	0	0
ANK1	0	1	0	1	1	0	0	1	0
ANKH	0	0	0	0	0	0	0	1	0
ANKHD1	1	0	1	1	0	1	1	0	1
ANKMY1	0	0	0	0	0	1	0	0	0
ANKMY2	1	0	1	1	0	1	1	0	1
ANKRA2	1	0	1	1	0	1	1	0	1

ANKRD10	1	0	1	1	0	1	1	0	1
ANKRD12	1	0	1	1	0	1	1	0	1
ANKRD13C	1	0	1	1	0	1	1	0	1
ANKRD13D	0	0	0	0	0	0	0	0	1
ANKRD15	0	1	0	0	1	0	1	1	1
ANKRD16	1	0	1	1	0	1	0	0	1
ANKRD17	1	0	1	1	0	1	1	0	1
ANKRD20A1	0	1	0	0	1	0	0	1	0
ANKRD22	0	0	0	1	0	0	1	0	0
ANKRD25	0	1	0	0	0	0	0	0	0
ANKRD26	1	0	1	1	0	1	0	0	1
ANKRD28	0	1	1	0	0	1	0	1	1
ANKRD29	0	1	0	0	1	1	0	1	0
ANKRD35	0	1	1	0	1	1	0	1	1
ANKRD37	1	0	1	1	0	1	1	1	1
ANKRD38	0	1	0	0	1	0	0	1	0
ANKRD39	0	1	0	0	1	0	0	1	0
ANKRD40	1	0	1	1	0	1	1	0	1
ANKRD44	0	0	0	0	0	1	1	0	0
ANKRD49	1	0	1	1	0	1	1	0	1
ANKRD50	1	1	1	1	0	1	1	0	1
ANKRD53	0	0	0	0	0	0	0	1	1
ANKS1A	1	0	1	1	0	1	1	0	1
ANKS1B	0	1	1	0	1	0	0	1	1
ANKS3	1	0	1	1	0	1	1	0	1
ANKZF1	1	0	1	1	0	1	1	1	1
ANP32A	1	0	1	1	0	1	1	0	1
ANP32B	1	1	1	1	0	1	1	1	1
ANP32E	1	0	1	1	0	1	0	0	1
ANTXR1	0	1	0	0	1	1	0	1	1
ANTXR2	0	0	0	0	0	0	1	0	1
ANUBL1	0	0	0	0	0	1	1	0	1
ANXA1	1	0	1	1	0	1	1	0	1
ANXA11	1	0	0	1	0	1	0	0	1
ANXA13	0	1	0	0	0	0	0	0	0
ANXA2	0	0	0	1	0	1	1	0	1
ANXA3	0	1	0	0	1	0	0	1	1
ANXA4	0	0	0	0	0	0	1	0	0
ANXA5	1	0	1	1	0	1	1	1	1
ANXA7	1	1	1	1	1	1	1	1	1
AOAH	1	0	1	1	0	1	1	0	1
AOC3	0	0	0	0	1	0	0	0	0
AOF1	1	0	1	1	0	1	1	0	1
AOF2	1	0	1	1	0	1	1	0	1
AP1G1	1	0	1	1	0	1	1	0	1
AP1G2	0	0	1	0	0	1	0	0	0
AP1GBP1	1	0	0	0	0	1	1	0	1
AP1M1	1	0	1	1	0	1	0	0	1
AP1M2	0	1	0	0	1	0	0	1	0
AP1S2	1	1	1	1	1	1	1	1	1
AP2A1	1	0	1	1	0	1	1	0	1
AP2B1	0	0	0	0	0	0	0	0	1
AP3B1	1	0	1	1	0	1	1	0	1
AP3B2	0	1	0	0	0	0	0	1	1
AP3M1	1	0	1	1	0	1	1	0	1
AP3M2	0	0	0	0	0	0	0	0	1
AP3S2	1	0	1	1	0	1	1	0	1
AP4B1	1	0	1	1	0	1	1	0	1
AP4E1	1	0	1	1	0	1	1	0	1
AP4M1	1	0	1	1	0	1	0	0	1
APAF1	1	0	1	1	0	1	1	0	1
APBA2BP	0	0	0	1	0	1	0	0	1

APBA3	1	0	1	1	0	1	1	0	1
APBB1IP	1	0	0	1	0	1	1	0	1
APBB2	0	1	0	0	1	1	0	1	1
APBB3	1	0	1	1	0	1	1	0	1
APC2	0	1	0	0	1	1	0	1	0
APCDD1	0	1	0	0	1	0	0	1	0
APEX1	1	0	1	1	0	1	1	0	1
APEX2	1	1	1	1	1	1	0	0	1
APH1A	1	0	1	1	0	1	1	0	1
APH1B	0	0	0	1	0	0	1	0	1
API5	1	0	1	1	0	1	1	0	1
APIP	1	0	1	1	0	1	1	0	1
APLP2	1	0	0	1	0	1	1	0	1
APOA1BP	1	0	0	1	0	1	1	0	1
APOB48R	1	0	1	1	0	1	1	0	1
APOBEC3A	0	0	0	0	0	0	0	0	1
APOBEC3C	0	0	0	0	0	1	0	0	1
APOBEC3F	0	0	0	0	0	1	0	0	1
APOBEC3G	0	0	0	1	0	1	1	0	1
APOC1	1	0	1	0	0	1	0	0	0
APOD	0	1	0	0	1	0	0	1	0
APOE	0	1	0	0	0	0	0	1	0
APOL1	1	0	0	0	0	1	1	0	1
APOL2	0	0	0	1	0	0	0	0	0
APOL3	1	0	1	1	0	1	1	0	1
APOL4	1	0	0	0	0	0	0	0	0
APOL6	0	0	0	1	0	1	1	0	1
APOM	1	0	1	1	0	1	1	0	1
APP	0	0	0	0	0	1	1	0	1
APPBP1	0	1	0	1	1	0	0	0	0
APPBP2	1	0	1	1	0	1	1	0	1
AQP3	0	0	1	0	0	1	0	1	1
AQP9	1	0	1	1	0	1	1	0	1
AQR	0	0	0	1	0	0	0	0	1
ARAF	0	1	1	0	1	0	0	1	0
ARCN1	1	0	0	0	0	0	0	0	0
ARD1A	0	0	0	0	1	0	0	1	0
ARF1	1	0	0	0	0	0	1	0	1
ARF3	1	1	1	1	0	1	1	0	1
ARF4	1	0	1	1	0	1	1	0	1
ARF5	0	0	1	1	0	1	1	0	1
ARF6	1	0	1	0	0	0	1	0	1
ARFGAP1	0	0	0	0	0	0	0	1	0
ARFGAP3	1	0	0	0	1	0	0	0	0
ARFGEF1	1	0	1	1	0	1	1	0	1
ARFGEF2	0	0	0	0	0	1	0	0	0
ARFIP1	1	0	1	1	0	1	1	0	1
ARFIP2	1	0	1	1	0	1	1	0	1
ARFRP1	1	0	1	1	0	1	0	0	1
ARG2	0	0	1	0	0	1	1	0	1
ARHGAP1	1	0	1	1	0	1	1	0	1
ARHGAP10	0	0	1	1	0	1	0	1	1
ARHGAP11A	1	0	1	1	0	1	1	0	1
ARHGAP12	1	0	1	1	0	1	1	0	1
ARHGAP15	1	0	1	1	0	1	1	0	1
ARHGAP18	1	0	1	1	0	1	1	0	1
ARHGAP19	1	0	0	1	0	1	0	0	1
ARHGAP21	0	0	1	0	0	1	0	1	0
ARHGAP22	0	0	1	0	0	1	0	0	1
ARHGAP24	0	0	1	0	0	1	1	1	1
ARHGAP25	1	0	1	1	0	1	1	0	1
ARHGAP26	0	0	1	0	0	1	0	0	1

ARHGAP30	0	0	0	0	0	0	1	1	1
ARHGAP4	0	0	1	0	0	1	0	0	1
ARHGAP5	1	1	1	1	0	1	0	1	1
ARHGAP6	0	1	0	0	1	0	0	1	0
ARHGAP8	0	1	0	0	1	0	0	1	0
ARHGAP9	1	0	1	1	0	1	1	0	1
ARHGDIB	1	0	1	1	0	1	1	0	1
ARHGDIG	0	1	0	0	1	0	0	1	0
ARHGEF1	1	0	1	1	0	1	1	0	1
ARHGEF10L	0	1	0	0	1	0	0	1	0
ARHGEF11	1	0	1	1	0	1	1	0	1
ARHGEF12	1	1	1	0	1	1	0	1	1
ARHGEF2	1	0	1	1	0	1	1	0	1
ARHGEF3	1	0	1	0	0	1	1	0	1
ARHGEF6	1	1	1	1	0	1	1	0	1
ARHGEF7	1	1	1	1	1	1	0	1	1
ARHGEF9	0	1	0	0	1	0	0	0	0
ARID1A	1	0	1	1	0	1	1	0	1
ARID1B	1	0	1	1	0	1	1	0	1
ARID2	1	0	1	1	0	1	1	0	1
ARID3A	1	0	0	0	0	1	0	0	1
ARID3B	1	0	1	1	0	1	1	0	1
ARID4A	1	0	1	1	0	1	1	0	1
ARID4B	1	0	1	1	0	1	1	0	1
ARID5B	1	0	1	1	0	1	1	0	1
ARIH1	1	0	1	1	0	1	1	0	1
ARL1	0	0	0	0	0	1	0	0	1
ARL13B	0	0	0	0	0	0	0	1	1
ARL15	1	0	1	1	0	1	1	0	1
ARL16	1	0	1	1	0	1	1	0	1
ARL17P1	1	0	1	1	0	1	1	0	1
ARL2BP	0	0	1	1	0	1	0	0	1
ARL3	1	0	1	1	0	1	1	0	1
ARL4A	1	0	1	1	0	1	0	0	1
ARL4C	0	1	0	0	1	0	0	1	0
ARL5A	0	0	0	1	0	1	1	0	1
ARL5B	1	0	0	1	0	1	0	0	1
ARL6IP2	0	0	0	0	0	0	0	0	1
ARL6IP4	1	0	1	1	0	1	0	0	1
ARL6IP5	1	0	1	1	0	1	1	0	1
ARL6IP6	1	0	1	1	0	1	1	0	1
ARMC1	1	0	1	1	0	1	1	0	1
ARMC5	1	0	1	1	0	1	0	0	1
ARMC6	1	0	1	1	0	1	1	0	1
ARMC7	0	0	0	0	0	0	1	0	1
ARMC8	1	1	1	1	0	1	1	1	1
ARMCX1	0	1	0	0	0	1	0	0	1
ARMCX2	0	1	0	1	1	1	0	1	0
ARMCX3	0	1	0	1	1	1	1	1	1
ARMCX4	0	1	0	0	1	0	0	1	0
ARMCX5	1	1	0	1	1	1	1	0	1
ARMCX6	0	1	0	1	1	1	1	1	1
ARMET	1	0	1	1	0	1	1	0	1
ARMETL1	1	0	1	1	0	1	1	0	1
ARNTL	1	1	1	0	1	1	0	1	1
ARNTL2	1	0	1	1	0	1	1	1	1
ARPC1A	1	0	0	0	0	0	0	0	1
ARPC2	0	0	1	0	0	1	0	0	1
ARPC3	0	0	0	1	0	0	0	0	0
ARPC4	1	0	1	1	0	1	1	0	1
ARPC5	1	0	1	1	0	1	1	1	1
ARPC5L	1	0	1	1	0	1	1	0	1

ARPM1	1	0	1	1	0	1	1	0	1
ARPP-19	0	0	0	0	0	1	1	0	1
ARPP-21	0	1	0	0	1	0	0	1	0
ARRB1	1	0	1	1	0	1	1	0	1
ARRDC3	1	0	1	1	0	1	1	0	1
ARRDC4	1	0	1	1	0	1	0	0	1
ARSB	1	0	1	1	0	1	1	0	1
ARSG	1	0	0	0	0	0	0	0	0
ARTN	0	1	0	0	1	1	0	1	1
ARTS-1	1	0	1	1	0	1	1	0	1
ARV1	1	0	1	1	0	1	1	0	1
ARVCF	0	1	0	0	1	1	0	1	1
ARX	0	1	1	0	1	1	0	1	1
AS3MT	0	1	0	0	1	0	0	1	0
ASAH1	1	0	1	1	0	1	1	0	1
ASAH3	0	1	0	0	0	0	0	0	0
ASAHL	1	0	0	0	0	0	0	0	0
ASB13	0	0	1	1	0	1	1	0	1
ASB16	0	0	0	0	1	0	0	0	0
ASB3	1	0	1	1	0	1	1	0	1
ASB7	1	0	1	1	0	1	1	0	1
ASB8	0	0	0	0	0	1	1	0	1
ASB9	0	1	0	0	1	0	0	1	0
ASCC1	1	0	1	1	0	1	1	0	1
ASCC2	0	0	0	0	0	0	0	0	1
ASCC3	1	0	1	1	0	1	1	0	1
ASCC3L1	1	0	1	1	0	1	1	0	1
ASCL1	0	1	1	0	1	1	0	1	0
ASCL2	0	1	0	0	1	0	0	1	0
ASF1B	1	0	1	1	0	1	1	0	1
ASH1L	1	0	1	1	0	1	1	0	1
ASH2L	1	0	1	1	0	1	1	0	1
ASL	1	0	1	0	0	0	0	0	0
ASNA1	1	0	1	1	0	1	1	0	1
ASNS	0	1	0	0	1	0	0	1	0
ASNSD1	1	0	1	1	0	1	1	0	1
ASPH	0	0	0	1	0	0	0	0	1
ASPHD1	0	1	0	0	0	0	0	1	0
ASPHD2	0	0	0	0	0	1	0	0	1
ASPM	0	0	1	1	0	1	1	0	1
ASTE1	1	0	1	1	0	1	1	0	1
ASTN2	0	1	1	0	1	1	1	1	1
ASXL1	1	0	1	1	0	1	1	0	1
ATAD1	0	0	0	0	0	1	1	0	1
ATAD2	1	0	1	1	0	1	1	0	1
ATAD3A	0	0	0	0	0	1	0	0	1
ATBF1	1	0	1	1	0	1	1	0	1
ATE1	1	0	1	1	0	1	1	0	1
ATF1	1	0	1	0	0	0	0	0	0
ATF2	1	0	1	1	0	1	1	0	1
ATF3	1	1	1	1	1	1	1	1	1
ATF4	1	0	1	1	0	1	1	0	1
ATF5	1	0	1	0	0	1	0	0	1
ATF6	1	0	1	1	0	1	1	0	1
ATF7	0	0	0	0	0	0	1	0	1
ATF7IP	0	0	0	0	0	1	1	0	1
ATG10	1	0	1	1	0	1	1	0	1
ATG12	1	0	1	1	0	1	1	0	1
ATG16L2	1	0	1	1	0	1	1	0	1
ATG3	1	0	1	1	0	1	1	0	1
ATG4A	1	1	1	1	1	1	1	1	1
ATG4C	1	0	1	1	0	1	1	0	1

ATG5	1	0	1	1	0	1	1	0	1
ATG9A	1	0	1	1	0	1	1	0	1
ATG9B	0	0	0	0	1	0	0	0	1
ATHL1	0	1	0	0	0	0	0	0	0
ATN1	0	0	0	0	0	0	0	1	0
ATOH1	0	1	0	0	1	1	0	1	1
ATOH8	0	1	0	0	1	0	0	1	1
ATOX1	1	0	1	1	0	1	1	0	1
ATP10A	0	1	0	0	1	0	0	1	0
ATP10D	0	0	0	0	0	1	0	0	1
ATP11B	1	0	1	1	0	1	1	0	1
ATP11C	0	0	0	0	0	0	1	0	0
ATP13A1	1	0	1	1	0	1	1	0	1
ATP1A3	0	1	0	0	1	0	0	1	0
ATP1B1	0	0	0	0	1	0	0	1	1
ATP1B2	0	1	1	0	1	1	0	1	1
ATP1B3	1	0	1	1	0	1	1	0	1
ATP1B4	0	1	0	0	1	0	0	0	0
ATP2A2	1	0	1	1	0	1	1	0	1
ATP2A3	0	0	0	0	0	1	0	0	0
ATP2B1	0	0	0	0	0	0	1	0	0
ATP2B3	0	1	0	0	1	0	0	0	0
ATP2B4	1	0	0	0	0	1	1	0	1
ATP2C1	1	0	1	1	0	1	1	0	1
ATP4A	0	1	0	0	0	0	0	0	0
ATP5A1	1	0	1	1	0	1	1	0	1
ATP5B	1	0	1	1	0	1	1	0	1
ATP5D	0	0	0	0	0	0	0	1	0
ATP5F1	1	0	1	1	0	1	1	0	1
ATP5G1	1	0	1	1	0	1	1	0	1
ATP5G2	1	1	1	1	0	1	1	0	1
ATP5G3	1	0	1	1	0	1	1	0	1
ATP5H	1	0	1	1	0	1	0	0	1
ATP5J	1	0	1	1	0	1	1	0	1
ATP5J2	0	0	0	0	0	0	0	0	1
ATP5L	1	0	1	1	0	1	1	0	1
ATP5O	0	0	0	1	0	0	0	0	1
ATP5S	1	0	1	1	0	1	1	0	1
ATP6AP1	0	1	0	0	0	1	0	0	0
ATP6AP2	0	1	1	1	1	1	1	1	1
ATP6V0A1	1	0	0	1	0	1	1	0	1
ATP6V0B	1	0	1	0	0	1	0	0	1
ATP6V0D1	1	0	1	1	0	1	1	0	1
ATP6V0D2	0	0	0	1	0	1	0	0	0
ATP6V1A	1	0	1	1	0	1	1	0	1
ATP6V1B2	1	0	0	0	0	1	0	0	0
ATP6V1C1	1	0	1	1	0	1	1	0	1
ATP6V1D	1	0	1	1	0	1	1	0	1
ATP6V1E1	1	0	1	1	0	1	1	0	1
ATP6V1F	0	0	0	0	0	0	1	0	1
ATP6V1G1	1	0	0	1	0	1	1	0	1
ATP6V1G2	1	0	1	1	0	1	0	0	1
ATP6V1H	1	0	1	1	0	1	1	0	1
ATP7A	1	1	1	1	1	1	0	0	1
ATP7B	1	1	1	1	1	1	1	1	1
ATP8A2	0	1	1	0	1	0	0	1	0
ATP8B3	1	0	0	1	0	1	0	0	1
ATP8B4	1	0	0	1	0	1	1	0	1
ATP9A	0	1	0	0	1	0	0	1	0
ATP9B	0	0	0	0	1	0	0	0	0
ATPAF1	0	0	0	0	0	0	0	0	1
ATPAF2	1	0	1	1	0	1	1	0	1

ATPBD1B	1	0	1	1	0	0	1	0	1
ATPBD1C	1	0	1	1	0	1	1	0	1
ATPBD4	1	0	1	1	0	1	1	0	1
ATPIF1	1	0	1	1	0	1	1	0	1
ATR	1	0	1	1	0	1	1	0	1
ATRN	1	0	0	0	0	0	0	0	0
ATXN1	1	0	1	1	0	1	1	0	1
ATXN10	1	0	0	0	0	0	0	0	0
ATXN2	1	0	1	1	0	1	1	0	1
ATXN2L	1	0	1	1	0	1	0	0	1
ATXN7L1	0	1	0	1	1	1	0	1	1
ATXN7L2	0	1	0	0	1	0	1	1	1
AUH	0	0	0	0	0	0	1	0	1
AUP1	1	0	1	1	0	1	0	0	1
AURKA	1	0	1	1	0	1	1	0	1
AURKB	1	0	1	1	0	1	1	0	1
AVEN	1	0	1	0	0	1	1	0	1
AVP	0	1	0	0	1	0	0	1	0
AVPI1	1	0	0	1	0	1	1	0	1
AVPR2	0	0	0	0	1	0	0	1	0
AXL	0	1	0	0	1	0	0	0	0
AYTL1	0	0	0	0	0	0	0	0	1
AZI1	0	0	0	0	0	0	0	0	1
AZI2	1	0	1	1	0	1	1	0	1
AZIN1	1	0	1	1	0	1	1	0	1
B2M	1	0	1	1	0	1	1	0	1
B3GALNT2	0	0	0	0	0	1	0	0	0
B3GALT4	1	0	1	1	0	1	1	0	1
B3GALT6	1	0	1	1	0	1	1	0	1
B3GAT1	0	1	0	0	1	0	0	1	0
B3GAT3	1	0	0	0	0	1	1	0	1
B3GNT1	0	1	1	0	1	1	0	1	1
B3GNT3	0	1	0	0	1	0	0	1	0
B3GNT5	1	0	1	1	0	1	1	1	1
B3GNTL1	1	0	0	0	0	0	0	0	0
B4GALT1	0	0	0	0	0	1	1	0	1
B4GALT2	1	0	1	1	0	1	0	0	1
B4GALT3	1	0	0	1	0	0	0	0	1
B4GALT5	0	0	0	1	0	1	1	0	1
B4GALT6	0	1	0	0	0	0	0	0	0
B4GALT7	1	0	1	0	0	1	0	0	1
BAALC	0	1	0	0	1	0	0	1	0
BACE1	0	0	0	0	1	0	1	1	1
BACE2	0	1	0	0	1	0	0	1	0
BACH1	1	0	1	1	0	1	1	0	1
BAG1	1	0	1	1	0	1	1	0	1
BAG3	0	0	0	0	0	0	0	1	0
BAG4	1	0	1	1	0	1	1	0	1
BAHD1	0	1	1	0	1	1	0	1	1
BAI1	0	1	0	0	1	0	0	1	0
BAIAP2	1	0	1	1	0	1	1	0	1
BAMBI	0	1	1	0	1	1	0	1	1
BANF1	1	0	1	1	0	1	1	0	1
BANP	1	0	0	1	0	1	0	0	1
BAP1	1	0	1	1	0	1	1	0	1
BARD1	1	1	1	1	0	1	1	1	1
BARHL2	0	1	1	0	1	1	0	1	1
BASP1	0	0	0	0	0	1	0	0	0
BAT1	1	0	1	1	0	1	0	0	1
BAT2	1	0	1	1	0	1	1	0	1
BAT2D1	1	0	1	1	0	1	1	0	1
BAT3	1	0	1	1	0	1	1	0	1

BAT4	1	0	1	1	0	1	1	0	1
BAT5	1	0	1	1	0	1	1	0	1
BAZ1A	0	0	0	0	0	0	0	0	1
BAZ2A	1	0	1	1	0	1	1	0	1
BAZ2B	1	0	1	1	0	1	1	0	1
BBC3	0	0	0	0	0	1	1	1	0
BBS1	1	0	1	1	0	1	1	0	1
BBS2	1	0	0	0	0	1	1	0	1
BBS4	1	0	1	1	0	1	0	0	1
BBS7	1	0	1	0	0	1	0	0	1
BBX	0	0	0	1	0	1	1	0	1
BCAM	0	1	0	0	0	0	0	1	0
BCAN	0	1	0	0	1	0	0	1	0
BCAP29	1	0	1	1	0	1	1	0	1
BCAP31	1	1	0	0	0	0	0	0	1
BCAR1	0	1	0	0	1	1	0	1	0
BCAR3	1	0	1	1	0	1	0	1	1
BCAS2	1	1	1	1	0	1	1	1	1
BCAS3	1	0	1	1	0	1	1	0	1
BCAT1	0	1	0	0	0	0	0	0	1
BCCIP	1	0	1	1	0	1	1	0	1
BCDO2	0	0	0	0	0	1	0	0	0
BCKDHA	1	0	1	1	0	1	1	0	1
BCKDHB	0	1	1	1	1	1	1	1	1
BCKDK	1	0	1	0	0	1	0	0	0
BCL10	0	0	0	0	0	1	1	0	1
BCL11A	0	0	1	0	0	1	1	0	1
BCL11B	0	1	1	0	1	0	0	1	0
BCL2	1	0	1	1	0	1	1	0	1
BCL2A1	0	0	1	1	0	1	1	0	1
BCL2L1	1	0	1	0	0	1	1	0	1
BCL2L11	0	0	0	0	0	0	0	0	1
BCL2L12	1	0	1	1	0	1	1	0	1
BCL2L13	1	0	1	1	0	1	1	0	1
BCL2L14	0	1	0	0	1	0	1	0	0
BCL2L2	1	0	1	1	0	1	1	0	1
BCL3	0	0	0	0	0	0	0	0	1
BCL6	1	0	1	1	0	1	1	0	1
BCL7A	0	1	1	0	1	0	0	1	0
BCL7C	0	0	0	0	0	1	0	0	1
BCL9	0	1	0	0	1	0	0	1	0
BCL9L	0	1	0	0	0	0	0	0	0
BCLAF1	1	0	1	1	0	1	1	0	1
BCOR	0	1	0	0	1	0	0	1	0
BCORL1	0	0	0	0	0	1	0	0	1
BCS1L	1	0	1	1	0	1	0	0	1
BDKRB2	0	1	0	0	1	0	0	1	0
BDNF	0	1	1	0	1	1	0	1	1
BDP1	0	0	0	0	0	1	0	0	1
BECN1	0	0	0	1	0	1	1	0	1
BET1	1	1	1	1	0	1	1	1	1
BET1L	1	0	1	1	0	1	1	0	1
BEX1	0	1	0	0	1	0	0	1	0
BFAR	1	0	1	0	0	0	0	0	0
BHLHB2	0	0	0	1	0	1	1	0	1
BHLHB3	1	0	1	1	0	1	0	1	1
BHLHB5	0	1	1	0	1	1	0	1	1
BHLHB9	1	1	1	1	1	1	1	1	1
BICD1	0	0	0	0	1	0	0	1	1
BICD2	1	0	1	1	0	1	0	0	1
BID	0	0	0	0	0	1	0	0	1
BIN1	0	0	1	0	0	1	0	1	1

BIN2	0	0	0	0	0	0	0	0	1
BIN3	1	0	0	1	0	1	0	0	1
BIRC2	1	0	1	1	0	1	1	0	1
BIRC3	1	0	1	1	0	1	1	0	1
BIRC4	1	1	1	0	0	1	0	1	0
BIRC6	1	0	0	1	0	1	0	0	1
BIVM	0	1	1	0	1	1	0	1	1
BLCAP	1	0	1	1	0	1	0	0	1
BLM	1	0	0	1	0	1	1	0	1
BLMH	0	1	0	0	0	0	0	1	1
BLNK	1	0	0	1	0	1	1	0	1
BLOC1S1	1	0	0	0	0	1	0	1	1
BLOC1S2	1	0	1	1	0	1	1	0	1
BLR1	0	1	0	0	0	0	0	0	0
BLVRA	0	0	1	0	0	1	1	0	1
BLVRB	1	0	1	1	0	1	1	1	1
BLZF1	1	0	1	1	0	1	1	0	1
BMF	0	0	1	0	0	1	1	0	1
BMP1	0	1	0	0	1	0	0	1	0
BMP2K	1	0	1	1	0	1	1	0	1
BMP6	0	1	0	0	1	0	0	1	1
BMP8B	0	0	0	0	0	0	0	0	1
BMPER	0	1	0	0	1	0	0	1	0
BMPR1A	0	1	0	0	1	0	0	1	0
BMPR2	1	0	1	1	0	1	1	1	1
BMX	0	1	0	0	1	0	1	1	0
BNIP1	0	0	0	0	0	0	0	0	1
BNIP2	0	0	0	1	0	1	1	0	1
BNIP3	0	1	0	0	1	0	0	1	0
BNIP3L	0	0	1	1	0	1	0	0	1
BOK	0	1	0	0	1	1	0	1	0
BOLA2	1	0	1	1	0	1	1	0	1
BOLA3	1	0	1	1	0	1	1	0	1
BOP1	1	0	1	1	0	1	1	0	1
BPGM	1	0	1	1	0	1	1	0	1
BPI	0	0	0	0	0	0	1	1	0
BPNT1	1	0	1	1	0	1	1	0	1
BRAF	1	0	1	1	0	1	1	0	1
BRAP	1	0	1	1	0	1	1	0	1
BRCA1	1	0	1	1	0	1	1	0	1
BRCC3	1	0	1	1	0	1	1	0	1
BRD1	1	0	1	1	0	1	1	0	1
BRD2	1	0	1	1	0	1	1	0	1
BRD3	0	1	0	0	1	0	0	1	0
BRD4	0	0	0	0	0	0	1	0	0
BRD8	1	0	1	1	0	1	1	0	1
BRD9	0	0	1	0	0	1	0	0	1
BRDG1	1	0	0	0	0	0	1	0	1
BRE	1	0	1	1	0	1	1	0	1
BRF1	0	0	0	0	0	0	0	1	0
BRF2	1	0	1	1	0	1	1	0	1
BRI3	0	0	0	0	0	1	0	0	1
BRI3BP	0	0	1	1	0	1	1	0	1
BRIP1	1	0	1	1	0	1	1	0	1
BRMS1	1	1	1	0	1	1	0	1	1
BRMS1L	0	0	1	1	0	1	0	0	1
BRP44	1	0	1	1	0	1	1	0	1
BRP44L	0	0	0	1	0	1	0	0	1
BRPF1	1	0	1	1	0	1	0	0	1
BRSK1	0	1	0	0	1	0	0	1	0
BRUNOL4	0	1	1	0	1	1	0	1	1
BRUNOL6	0	0	0	0	0	0	0	1	0

BRWD1	1	0	1	1	0	1	1	0	1
BRWD2	1	0	0	1	0	1	1	0	1
BRWD3	1	0	1	0	0	1	1	0	1
BSCL2	1	0	1	1	0	1	1	0	1
BSDC1	1	0	1	1	0	1	1	0	1
BSG	1	0	1	1	0	1	1	0	1
BST1	1	0	0	0	0	0	1	0	1
BST2	1	0	1	1	0	1	1	0	1
BTAF1	1	0	1	1	0	1	1	0	1
BTBD1	0	0	0	0	0	0	0	0	1
BTBD10	1	0	1	1	0	1	1	0	1
BTBD12	1	0	1	1	0	1	1	0	1
BTBD14B	1	0	1	1	0	1	1	0	1
BTBD15	1	0	1	1	0	1	1	0	1
BTBD3	0	1	0	0	1	0	1	1	1
BTBD6	0	0	0	0	0	0	0	1	0
BTBD7	1	0	1	1	0	1	1	0	1
BTD	1	0	1	1	0	1	1	0	1
BTF3	0	0	1	0	0	1	0	0	1
BTF3L4	1	0	1	1	0	1	1	0	1
BTG1	1	0	0	1	0	1	1	0	1
BTG2	1	0	1	1	0	1	1	0	1
BTK	1	1	1	1	1	1	1	0	1
BTN1A1	0	1	0	0	1	0	0	1	0
BTN2A1	1	0	0	1	0	0	1	0	1
BTN2A2	1	0	1	1	0	1	1	0	1
BTN2A3	1	0	1	1	0	1	1	0	1
BTN3A1	1	0	1	1	0	1	1	0	1
BTN3A2	1	0	1	1	0	1	1	0	1
BTN3A3	1	0	1	1	0	1	1	0	1
BTRC	0	0	0	0	0	1	0	0	0
BUB1	1	0	1	1	0	1	1	0	1
BUB1B	1	0	1	0	0	1	0	0	1
BUB3	0	1	0	0	1	1	0	1	1
BUD13	1	0	1	1	0	1	0	0	1
BUD31	1	0	1	1	0	1	1	0	1
BXDC1	1	0	1	1	0	1	1	0	1
BXDC2	1	0	1	1	0	1	1	0	1
BXDC5	0	1	1	0	0	1	0	1	1
BYSL	1	0	1	1	0	1	1	0	1
BZRAP1	1	0	0	0	0	1	1	0	1
BZW2	1	0	1	1	0	1	1	0	1
C10ORF104	1	0	1	1	0	1	1	0	1
C10ORF118	1	0	1	1	0	1	1	0	1
C10ORF119	1	0	1	1	0	1	1	0	1
C10ORF132	0	1	0	0	1	0	0	1	0
C10ORF137	1	0	1	1	0	1	1	0	1
C10ORF22	1	0	1	1	0	1	0	0	1
C10ORF26	1	0	1	1	0	1	0	0	0
C10ORF28	1	0	1	1	0	1	1	0	1
C10ORF35	0	1	0	0	1	0	0	1	0
C10ORF46	0	0	0	0	0	1	0	1	0
C10ORF55	1	0	1	0	0	1	0	0	0
C10ORF56	0	1	0	0	0	1	0	1	1
C10ORF58	0	1	1	1	1	1	1	1	1
C10ORF59	0	0	0	1	0	1	1	0	1
C10ORF6	1	0	0	1	0	0	0	0	0
C10ORF64	1	0	1	1	0	1	1	0	1
C10ORF71	0	1	0	0	0	0	0	0	0
C10ORF72	0	1	0	0	0	0	0	1	0
C10ORF78	1	0	1	1	0	1	1	0	1
C10ORF83	0	0	1	0	0	1	0	0	1

C10ORF88	0	1	0	0	1	0	0	1	0
C10ORF93	0	1	0	0	1	0	0	1	0
C10ORF97	0	1	1	1	0	1	1	0	1
C11ORF10	1	0	1	1	0	1	1	0	1
C11ORF17	0	0	0	0	0	1	0	1	1
C11ORF2	1	0	0	0	0	0	0	0	1
C11ORF24	0	0	1	1	0	1	1	0	1
C11ORF30	1	0	1	1	0	1	1	0	1
C11ORF31	1	0	1	1	0	1	1	0	1
C11ORF45	1	0	1	1	0	1	1	0	1
C11ORF46	1	1	1	0	0	1	0	0	1
C11ORF48	1	0	1	1	0	1	1	0	1
C11ORF51	1	0	0	1	0	0	1	0	1
C11ORF52	0	1	0	0	1	0	0	1	0
C11ORF54	1	0	1	1	0	1	1	0	1
C11ORF56	1	0	1	1	0	1	1	0	1
C11ORF57	1	0	1	1	1	1	1	0	1
C11ORF58	1	0	1	1	0	1	1	0	1
C11ORF59	1	0	0	1	0	1	0	0	1
C11ORF60	1	0	1	1	0	1	0	0	1
C11ORF61	1	1	1	1	0	1	0	1	1
C11ORF63	0	1	1	0	1	0	1	1	1
C11ORF67	1	0	1	1	0	1	0	0	1
C11ORF68	1	0	1	0	0	0	0	0	0
C11ORF71	1	0	1	1	0	1	1	0	1
C11ORF73	0	0	1	0	0	0	1	0	1
C11ORF74	0	1	0	1	1	1	0	1	1
C11ORF9	0	1	0	0	1	0	0	1	1
C12ORF10	1	0	1	1	0	1	1	0	1
C12ORF11	1	0	1	1	0	1	1	0	1
C12ORF23	0	1	0	0	1	0	0	1	1
C12ORF24	1	0	1	1	0	1	1	0	1
C12ORF26	1	0	1	1	0	1	1	0	1
C12ORF29	0	0	1	0	0	1	0	0	1
C12ORF30	1	0	0	1	0	1	1	0	1
C12ORF31	1	0	1	1	0	1	1	0	1
C12ORF32	1	0	1	1	0	1	1	0	1
C12ORF34	0	1	0	0	1	0	0	1	0
C12ORF35	1	0	1	1	0	1	1	0	1
C12ORF4	1	0	1	1	0	1	1	0	1
C12ORF41	0	0	0	0	0	0	0	0	1
C12ORF43	0	0	0	0	0	1	0	0	0
C12ORF44	0	0	0	0	0	0	1	0	1
C12ORF47	1	0	1	1	0	1	1	0	1
C12ORF48	1	0	1	1	0	1	1	0	1
C12ORF49	1	1	1	1	0	1	0	1	1
C12ORF5	1	0	0	0	0	0	1	0	1
C12ORF52	1	0	1	1	0	1	1	0	1
C12ORF57	1	0	0	1	0	0	1	0	1
C12ORF60	1	0	1	1	0	1	1	0	1
C12ORF61	1	0	1	1	0	1	1	0	1
C12ORF62	0	0	0	0	0	0	1	0	1
C13ORF18	1	1	1	1	0	1	1	1	1
C13ORF23	1	0	1	1	0	1	1	0	1
C13ORF24	1	0	1	1	0	1	1	0	1
C13ORF26	0	1	0	0	1	0	0	1	0
C13ORF3	1	0	1	1	0	1	1	0	1
C13ORF7	1	0	1	1	0	1	1	0	1
C13ORF8	0	0	0	1	0	1	0	0	0
C14ORF1	1	0	1	1	0	1	1	0	1
C14ORF100	1	0	1	1	0	1	1	0	1
C14ORF102	1	0	1	1	0	1	1	0	1

C14ORF106	0	0	0	0	0	0	0	0	1
C14ORF108	1	0	1	1	0	1	1	0	1
C14ORF112	1	1	1	1	1	1	1	1	1
C14ORF118	0	0	0	0	0	1	0	0	1
C14ORF122	1	0	1	1	0	1	1	0	1
C14ORF124	1	0	1	1	0	1	1	0	1
C14ORF126	0	0	0	1	0	0	1	0	1
C14ORF130	1	0	1	1	0	1	0	0	1
C14ORF133	1	0	1	1	0	1	1	0	1
C14ORF138	1	0	1	1	0	0	1	0	1
C14ORF140	1	0	1	1	0	1	1	0	1
C14ORF142	1	0	1	1	0	1	0	0	1
C14ORF145	0	0	0	1	0	0	0	0	0
C14ORF147	0	0	1	0	0	0	0	0	0
C14ORF149	1	0	1	1	0	1	1	0	1
C14ORF152	0	1	0	0	0	0	0	0	0
C14ORF153	1	0	1	1	0	1	1	0	1
C14ORF156	1	0	1	1	0	1	1	0	1
C14ORF159	1	0	1	1	0	1	1	0	1
C14ORF162	0	1	0	0	1	0	0	1	0
C14ORF166	0	0	1	1	0	1	0	0	1
C14ORF174	1	0	1	0	0	1	1	0	1
C14ORF2	1	0	1	1	0	1	1	0	1
C14ORF21	1	0	1	1	0	1	0	0	1
C14ORF24	1	0	1	1	0	1	1	0	1
C14ORF37	0	1	1	0	1	1	1	1	1
C14ORF43	0	0	0	0	0	0	1	0	1
C14ORF48	0	1	0	0	0	0	0	1	0
C14ORF93	1	0	0	0	0	0	0	0	1
C14ORF94	1	0	1	1	0	1	1	0	1
C15ORF15	1	0	1	1	0	1	1	0	1
C15ORF17	1	0	1	1	0	1	1	0	1
C15ORF23	0	0	0	1	0	0	0	0	1
C15ORF24	1	0	1	1	0	1	1	0	1
C15ORF27	0	1	1	0	1	1	1	1	1
C15ORF38	0	0	0	0	0	1	0	0	0
C15ORF39	0	1	0	0	0	0	0	0	1
C15ORF40	1	0	1	0	0	0	0	0	1
C15ORF44	0	0	0	0	0	0	0	0	1
C15ORF5	0	0	0	0	0	0	1	0	0
C16ORF24	0	1	0	0	0	0	0	1	1
C16ORF30	0	1	0	0	1	0	0	1	0
C16ORF33	1	0	0	1	0	1	0	0	1
C16ORF45	0	1	0	0	1	0	0	1	0
C16ORF46	0	0	1	0	0	1	0	0	1
C16ORF48	0	0	0	1	0	0	0	0	0
C16ORF5	1	0	1	1	0	1	1	0	1
C16ORF52	0	0	1	0	0	1	1	1	1
C16ORF53	0	1	0	0	1	1	0	1	1
C16ORF54	0	0	0	0	0	1	0	0	1
C16ORF57	1	0	1	0	0	1	1	0	1
C16ORF58	1	0	1	1	0	1	1	0	1
C16ORF59	0	0	0	0	0	0	0	0	1
C16ORF61	1	0	1	1	0	1	1	0	1
C16ORF63	1	0	0	1	0	1	0	0	1
C16ORF68	1	1	1	1	1	1	1	1	1
C16ORF7	0	0	0	0	0	1	0	0	1
C16ORF70	0	0	0	1	0	1	0	0	0
C17ORF32	1	0	1	1	0	1	1	0	1
C17ORF39	1	0	1	1	0	1	1	0	1
C17ORF45	1	0	1	1	0	1	1	0	1
C17ORF48	1	0	1	1	0	1	1	0	1

C17ORF49	1	0	1	1	0	1	1	0	1
C17ORF56	1	0	1	1	0	1	0	0	1
C17ORF58	1	0	1	1	0	1	1	1	1
C17ORF59	1	0	1	1	0	1	0	0	1
C17ORF61	1	1	1	1	1	1	1	1	1
C17ORF62	1	0	1	1	0	1	1	0	1
C17ORF63	1	0	1	1	0	1	1	0	1
C17ORF64	1	0	1	0	0	0	0	0	0
C17ORF65	1	0	1	1	0	1	1	0	1
C17ORF67	0	0	1	0	0	1	0	0	0
C17ORF68	1	0	1	1	0	1	1	0	1
C17ORF70	0	0	1	1	0	1	1	0	1
C17ORF71	1	0	0	1	0	1	1	0	1
C17ORF75	0	0	0	1	0	0	0	0	0
C17ORF76	0	1	0	0	1	0	0	1	0
C17ORF77	0	1	0	0	0	0	0	0	0
C17ORF79	1	0	0	0	0	1	1	0	0
C17ORF80	1	0	1	1	0	1	1	0	1
C17ORF81	1	0	1	1	0	1	1	0	1
C18ORF1	1	0	1	1	0	1	1	1	1
C18ORF10	1	0	1	1	0	1	1	0	1
C18ORF19	1	0	1	1	0	1	1	0	1
C18ORF21	1	0	1	1	0	1	1	0	1
C18ORF22	0	0	0	0	0	0	0	0	1
C18ORF24	0	0	0	0	0	0	0	0	1
C18ORF25	0	0	0	1	0	0	0	0	1
C18ORF37	1	0	0	1	0	1	0	0	1
C18ORF45	1	0	1	1	0	1	1	0	1
C18ORF54	1	1	1	1	0	1	1	0	1
C18ORF55	1	0	1	1	0	1	1	0	1
C18ORF56	0	1	0	0	0	1	0	0	0
C18ORF8	0	0	0	0	0	0	0	0	1
C19ORF12	0	0	0	1	0	1	0	0	1
C19ORF22	0	1	0	0	0	0	0	1	0
C19ORF23	1	0	1	1	0	1	1	0	1
C19ORF24	1	0	1	0	0	0	0	0	0
C19ORF26	0	0	0	0	0	0	0	1	0
C19ORF28	0	0	0	0	0	1	1	0	1
C19ORF30	0	1	0	0	1	0	0	0	0
C19ORF35	0	0	0	0	0	0	1	0	1
C19ORF39	1	0	1	1	0	1	1	0	1
C19ORF40	1	0	1	1	0	1	1	0	1
C19ORF43	1	0	1	1	0	1	1	0	1
C19ORF6	0	0	0	0	0	0	1	0	0
C1D	1	0	1	1	0	1	1	0	1
C1QA	0	0	1	1	0	1	0	0	0
C1QBP	0	0	0	1	0	0	0	0	0
C1QL1	0	1	1	0	1	0	0	1	1
C1QL2	0	1	1	0	1	0	0	1	0
C1QL3	0	1	1	0	1	0	0	1	0
C1QTNF2	0	1	1	1	1	1	0	1	1
C1ORF102	1	0	1	1	0	1	1	0	1
C1ORF103	0	0	1	0	0	1	1	0	1
C1ORF104	0	0	0	0	0	0	0	0	1
C1ORF105	1	0	0	0	0	0	0	0	0
C1ORF107	1	0	1	0	0	1	1	1	1
C1ORF108	0	0	0	0	0	0	1	0	1
C1ORF112	1	0	1	1	0	1	1	0	1
C1ORF115	0	1	0	0	1	0	0	1	0
C1ORF119	1	0	1	1	0	1	0	0	1
C1ORF120	0	1	0	0	0	0	0	0	0
C1ORF121	0	0	1	1	0	1	1	0	1

C1ORF122	1	0	1	1	0	1	0	0	1
C1ORF123	1	0	0	1	0	1	0	0	1
C1ORF124	1	0	1	1	0	1	1	0	1
C1ORF128	1	0	1	1	0	1	1	0	1
C1ORF130	0	1	0	0	1	0	0	1	0
C1ORF131	1	0	1	1	0	1	1	0	1
C1ORF135	1	0	1	1	0	1	1	0	1
C1ORF142	0	0	0	1	0	1	0	0	1
C1ORF149	1	0	0	1	0	1	1	0	1
C1ORF156	1	0	1	1	0	1	1	0	1
C1ORF159	0	1	0	0	1	0	0	1	0
C1ORF162	1	0	1	1	0	1	1	0	1
C1ORF164	0	1	1	0	1	1	0	1	1
C1ORF166	0	0	0	0	0	1	0	0	1
C1ORF174	0	0	0	0	0	1	0	0	1
C1ORF176	1	0	1	1	0	1	1	0	1
C1ORF181	1	0	1	0	0	1	1	0	1
C1ORF183	1	1	1	1	1	1	1	1	1
C1ORF187	0	1	0	0	1	0	0	1	0
C1ORF19	1	1	1	0	1	1	1	1	1
C1ORF190	0	1	1	1	1	1	0	1	1
C1ORF198	0	1	0	0	1	0	0	1	0
C1ORF2	0	0	0	0	0	0	0	0	1
C1ORF201	1	0	1	1	0	1	1	0	1
C1ORF21	0	1	1	0	1	1	1	1	1
C1ORF25	1	0	1	1	0	1	1	0	1
C1ORF26	1	0	1	1	0	1	1	0	1
C1ORF27	1	0	1	1	0	1	1	0	1
C1ORF31	1	0	1	1	0	1	1	0	1
C1ORF32	0	1	1	0	1	0	0	1	1
C1ORF35	0	0	0	1	0	1	0	0	1
C1ORF38	1	0	1	1	0	1	1	0	1
C1ORF41	1	0	1	1	0	1	1	0	1
C1ORF43	1	0	1	1	0	1	1	0	1
C1ORF50	1	0	1	1	0	1	1	0	1
C1ORF51	1	1	1	1	1	1	1	1	1
C1ORF53	0	0	1	0	0	0	1	0	0
C1ORF54	1	0	1	1	0	1	1	0	1
C1ORF55	1	0	1	1	0	1	1	0	1
C1ORF56	0	1	0	0	0	0	1	1	1
C1ORF57	1	0	1	1	0	1	1	0	1
C1ORF58	1	0	1	1	0	1	1	0	1
C1ORF59	0	0	0	0	1	0	1	0	0
C1ORF63	1	0	1	1	0	1	1	0	1
C1ORF66	1	0	1	1	0	1	1	0	1
C1ORF69	0	0	1	0	0	1	0	1	1
C1ORF71	1	0	1	1	0	1	1	0	1
C1ORF74	1	0	1	1	0	1	1	0	1
C1ORF77	1	0	1	1	0	1	1	0	1
C1ORF78	0	0	1	0	0	1	0	0	1
C1ORF83	1	0	1	1	0	1	1	0	1
C1ORF84	1	0	1	1	0	1	1	0	1
C1ORF85	1	0	1	1	0	1	1	0	1
C1ORF88	0	1	1	0	0	1	1	1	1
C1ORF89	1	0	1	1	0	1	1	0	1
C1ORF9	1	0	1	1	0	1	1	0	1
C1ORF91	1	0	0	1	0	1	0	0	0
C1ORF92	0	1	0	0	1	0	0	1	0
C1ORF93	0	0	0	0	0	1	0	0	0
C1ORF94	0	1	1	0	1	0	0	1	0
C1ORF95	0	1	0	0	1	0	0	1	0
C1ORF96	1	0	1	0	0	1	0	0	1

C1ORF97	1	0	1	1	0	1	1	0	1
C2	1	0	1	1	0	1	1	0	1
C20ORF103	0	1	1	0	1	0	0	1	1
C20ORF11	1	0	1	1	0	1	1	0	1
C20ORF111	0	0	1	1	0	1	0	0	1
C20ORF116	1	0	1	1	0	1	1	0	1
C20ORF117	1	0	1	1	0	1	0	1	1
C20ORF12	1	0	1	1	0	1	1	0	1
C20ORF121	1	0	0	0	0	0	0	0	1
C20ORF133	0	1	1	0	1	1	0	1	1
C20ORF134	0	0	0	1	0	0	0	0	0
C20ORF149	0	1	0	0	1	0	0	1	0
C20ORF177	1	0	1	1	0	1	1	0	1
C20ORF19	1	1	1	1	0	1	1	1	1
C20ORF23	1	0	1	1	0	1	1	0	1
C20ORF24	0	0	0	0	0	0	1	0	1
C20ORF26	1	0	1	1	0	1	1	0	1
C20ORF27	0	0	0	1	0	0	0	0	0
C20ORF29	1	0	1	1	0	0	1	0	1
C20ORF3	1	0	1	1	0	1	1	0	1
C20ORF30	1	0	1	1	0	1	1	0	1
C20ORF32	1	0	1	1	0	1	1	0	1
C20ORF39	0	1	1	0	1	0	0	1	0
C20ORF4	1	0	1	1	0	1	1	0	1
C20ORF43	1	0	1	1	0	1	1	0	1
C20ORF46	0	1	0	0	1	0	0	1	0
C20ORF52	1	0	1	1	0	1	1	0	1
C20ORF67	1	0	1	1	0	1	1	0	1
C20ORF72	1	0	1	1	0	1	1	0	1
C20ORF94	1	0	1	1	0	1	1	0	1
C21ORF119	1	0	1	1	0	1	1	0	1
C21ORF122	0	1	0	0	0	0	0	1	0
C21ORF124	0	0	1	1	0	1	1	0	1
C21ORF129	0	1	0	0	1	0	0	1	0
C21ORF13	1	1	1	1	0	1	1	1	1
C21ORF2	1	0	1	1	0	1	1	0	1
C21ORF33	1	0	1	1	0	0	0	0	0
C21ORF34	1	0	1	1	0	1	1	0	0
C21ORF51	1	0	1	1	0	0	1	0	1
C21ORF55	1	0	1	1	0	1	1	0	1
C21ORF56	1	0	0	0	0	1	0	0	1
C21ORF57	1	0	1	1	0	1	1	0	1
C21ORF59	0	0	0	0	0	1	0	0	1
C21ORF63	0	0	0	0	0	0	0	1	1
C21ORF66	1	0	1	1	0	1	1	0	1
C21ORF67	1	0	1	0	0	1	1	0	1
C21ORF7	1	0	0	1	0	1	1	0	1
C21ORF70	1	0	1	0	0	1	1	0	1
C21ORF81	0	1	0	0	0	0	0	1	0
C21ORF91	1	0	1	1	0	1	1	0	1
C22ORF13	1	0	1	1	0	1	0	0	1
C22ORF25	0	1	0	0	1	1	0	1	1
C22ORF9	1	0	1	1	0	1	1	0	1
C2ORF13	1	0	1	1	0	1	1	0	1
C2ORF15	1	1	1	1	1	1	1	1	1
C2ORF16	0	1	0	0	1	0	0	0	0
C2ORF21	0	1	0	0	0	0	0	1	0
C2ORF24	1	0	1	1	0	1	1	0	1
C2ORF25	1	1	1	1	0	1	1	1	1
C2ORF28	1	0	1	1	0	1	1	0	1
C2ORF29	1	0	1	0	0	1	0	0	1
C2ORF30	1	0	1	1	0	1	1	0	1

C2ORF32	0	1	1	0	0	1	1	1	1
C2ORF33	1	0	1	1	0	1	1	0	1
C2ORF34	1	0	1	1	0	1	1	0	1
C2ORF37	1	0	1	1	0	1	1	0	1
C2ORF7	1	0	1	1	0	1	1	0	1
C3AR1	1	0	1	1	0	1	1	0	1
C3ORF1	1	0	1	1	0	1	1	0	1
C3ORF14	0	1	1	0	1	1	0	1	1
C3ORF17	1	0	0	0	0	1	1	0	1
C3ORF21	1	0	1	1	0	1	1	0	1
C3ORF23	1	0	1	1	0	1	1	0	1
C3ORF26	1	0	1	1	0	1	1	0	1
C3ORF28	1	0	1	1	0	1	1	0	1
C3ORF31	1	1	0	1	1	1	1	1	1
C3ORF37	0	0	0	0	0	0	0	0	1
C3ORF38	1	0	1	1	0	1	1	0	1
C3ORF39	0	1	1	1	0	1	1	1	0
C3ORF54	0	1	0	0	1	0	0	0	0
C3ORF58	1	0	1	1	0	1	0	0	1
C3ORF59	1	1	1	1	0	1	1	1	1
C3ORF60	1	0	1	1	0	1	1	0	1
C3ORF62	1	0	0	1	0	0	1	0	1
C3ORF64	0	0	1	0	0	1	0	0	1
C4BPB	0	0	0	1	0	0	1	0	0
C4ORF14	1	0	1	1	0	1	1	0	1
C4ORF16	1	0	1	1	0	1	1	0	1
C4ORF18	1	0	1	1	0	1	1	0	1
C4ORF20	1	1	1	1	0	1	1	1	1
C5AR1	0	0	0	0	0	1	1	0	1
C5ORF13	0	1	0	0	0	0	0	0	0
C5ORF15	1	0	1	1	0	1	1	0	1
C5ORF22	1	0	1	1	0	1	1	0	1
C5ORF24	1	0	0	1	0	0	0	0	0
C5ORF3	1	0	1	1	0	1	1	0	1
C5ORF4	1	0	1	1	0	1	1	0	1
C5ORF5	1	0	1	1	0	1	1	0	1
C6ORF106	1	0	1	1	0	1	1	0	1
C6ORF108	0	1	0	0	0	0	0	0	0
C6ORF113	1	0	1	1	0	1	1	0	1
C6ORF114	1	0	1	1	0	1	0	1	1
C6ORF120	1	0	1	1	0	1	1	0	1
C6ORF128	1	0	1	1	0	1	1	0	1
C6ORF129	0	0	1	1	0	1	1	0	1
C6ORF130	1	0	1	1	0	1	1	0	1
C6ORF134	1	0	1	1	0	1	1	0	1
C6ORF145	0	0	0	0	0	0	0	1	0
C6ORF151	0	0	0	0	0	0	0	0	1
C6ORF153	0	0	0	0	0	0	0	0	1
C6ORF157	1	0	1	1	0	1	1	0	1
C6ORF166	1	0	1	1	0	1	1	0	1
C6ORF167	1	0	1	1	0	1	1	0	1
C6ORF173	0	0	1	1	0	1	1	0	1
C6ORF174	0	1	0	0	1	1	0	1	0
C6ORF182	1	0	1	1	0	1	1	0	1
C6ORF192	1	0	1	1	0	1	1	0	1
C6ORF199	1	0	1	1	0	1	1	0	1
C6ORF203	0	0	0	0	0	0	0	0	1
C6ORF204	0	0	0	0	0	1	0	0	1
C6ORF211	1	0	1	1	0	1	1	0	1
C6ORF25	0	0	0	0	1	0	0	0	0
C6ORF47	1	0	1	1	0	1	1	0	1
C6ORF48	1	0	1	1	0	1	1	0	1

C6ORF49	0	0	0	0	0	1	0	0	1
C6ORF52	1	0	1	1	0	1	1	0	1
C6ORF62	1	0	1	1	0	1	1	0	1
C6ORF64	0	0	0	0	0	0	0	0	1
C6ORF66	1	0	1	1	0	1	1	0	1
C6ORF70	1	0	1	1	0	1	1	0	1
C6ORF72	0	1	0	0	1	1	0	1	0
C6ORF85	0	1	0	0	1	0	0	0	0
C6ORF89	0	0	0	1	0	0	0	0	1
C6ORF97	1	0	1	0	0	1	1	0	1
C7ORF10	1	1	1	1	1	1	1	1	1
C7ORF11	1	1	1	1	1	1	1	1	1
C7ORF13	1	1	1	1	1	1	1	1	1
C7ORF23	0	1	1	0	1	1	0	1	1
C7ORF24	1	0	0	1	0	1	1	0	1
C7ORF28A	0	0	0	1	0	1	0	0	0
C7ORF28B	0	0	0	1	0	1	0	0	0
C7ORF31	0	1	0	0	0	0	0	1	0
C7ORF36	1	0	1	1	0	1	1	0	1
C7ORF38	1	0	1	1	0	1	0	0	1
C8B	0	1	0	0	0	0	0	0	0
C8ORF13	0	1	1	0	0	1	0	1	1
C8ORF32	1	0	1	1	0	1	1	0	1
C8ORF33	1	0	1	1	0	1	1	1	1
C8ORF38	0	0	0	0	0	0	0	0	1
C8ORF40	1	0	1	1	0	1	1	0	1
C8ORF41	1	0	1	1	0	1	1	1	1
C8ORF44	1	0	1	1	0	1	1	0	1
C8ORF48	0	1	1	0	1	1	0	1	1
C8ORF51	1	0	1	1	0	1	1	1	1
C8ORF53	1	0	1	1	0	1	1	0	1
C8ORF54	0	0	0	0	0	0	1	0	0
C8ORF58	0	0	0	0	0	0	0	1	0
C8ORF70	0	0	1	1	1	1	1	0	1
C8ORF79	0	1	1	0	1	1	1	1	1
C9ORF100	1	0	1	0	0	1	1	0	1
C9ORF106	0	0	0	0	0	1	0	0	1
C9ORF114	0	0	0	0	0	0	0	0	1
C9ORF116	1	0	1	0	0	0	0	0	0
C9ORF119	1	0	1	1	0	1	1	0	1
C9ORF123	0	0	0	1	0	1	1	0	1
C9ORF127	0	1	0	0	1	0	0	1	0
C9ORF128	0	1	0	0	1	0	0	1	0
C9ORF130	1	0	1	1	0	1	1	0	1
C9ORF142	0	0	0	0	0	0	0	1	0
C9ORF156	1	0	1	1	0	1	1	0	1
C9ORF16	0	0	0	0	0	0	0	0	1
C9ORF18	1	0	1	1	0	1	1	0	1
C9ORF21	0	0	1	1	0	1	0	0	1
C9ORF23	1	0	1	1	0	1	0	0	1
C9ORF24	0	1	0	0	1	0	0	1	0
C9ORF25	0	1	1	1	0	1	0	1	1
C9ORF3	0	0	0	0	0	1	0	0	1
C9ORF30	1	0	1	1	0	1	1	1	1
C9ORF37	1	0	1	1	0	1	1	0	1
C9ORF40	0	0	0	0	0	0	1	0	1
C9ORF41	0	0	1	0	0	0	0	0	1
C9ORF46	1	0	1	1	0	0	1	0	1
C9ORF58	0	1	0	0	1	0	0	1	0
C9ORF6	1	0	1	1	0	1	1	0	1
C9ORF64	0	0	0	1	0	1	0	0	1
C9ORF66	1	0	1	1	0	1	1	0	1

C9ORF72	1	0	1	1	0	1	1	0	1
C9ORF78	1	0	1	1	0	1	1	0	1
C9ORF80	0	0	0	1	0	1	1	0	1
C9ORF82	1	0	1	0	0	1	1	0	1
C9ORF85	1	0	1	1	0	1	1	0	1
C9ORF9	0	1	0	0	1	0	0	1	0
C9ORF90	1	0	1	1	0	1	1	0	1
C9ORF91	0	0	0	0	0	1	1	0	1
C9ORF93	0	1	1	1	0	1	1	0	1
C9ORF95	1	0	1	1	0	1	1	0	1
C9ORF97	1	0	1	1	0	1	1	0	1
C9ORF98	0	1	0	0	1	0	0	1	0
CA11	0	0	0	0	0	1	0	1	1
CA12	0	1	0	0	1	0	0	1	0
CA13	1	0	1	1	0	1	1	0	1
CA2	1	0	0	0	0	0	0	0	0
CA4	0	1	0	0	1	0	0	1	0
CA5A	0	1	0	0	0	0	0	0	0
CA5B	1	1	1	1	1	1	0	1	1
CAB39	0	0	1	0	0	0	0	0	0
CAB39L	0	0	0	1	0	0	1	0	0
CABC1	1	0	0	0	0	1	1	0	1
CABIN1	1	0	1	1	0	1	1	0	1
CABYR	1	1	0	0	0	1	0	1	1
CACNA1A	0	1	1	0	1	0	0	1	0
CACNA2D2	0	1	0	0	1	0	0	1	0
CACNA2D3	0	1	0	0	1	0	0	1	0
CACNA2D4	1	0	1	0	0	1	1	0	1
CACNB1	1	0	0	1	0	1	1	0	1
CACNB4	0	1	0	0	1	1	0	1	1
CACNG2	0	1	0	0	1	0	0	1	0
CACNG3	0	1	0	0	1	0	0	1	0
CACNG7	0	1	0	0	1	0	0	1	0
CACYBP	1	0	1	1	0	1	1	0	1
CAD	1	0	1	1	0	1	1	0	1
CAGE1	1	0	1	1	0	1	1	0	1
CALCB	0	1	0	0	1	0	0	1	0
CALCOCO1	1	0	1	1	0	1	1	0	1
CALCOCO2	0	0	0	0	0	1	0	0	0
CALCR	0	1	1	0	1	0	0	1	0
CALCRL	1	0	1	1	0	1	1	0	1
CALD1	0	1	0	0	1	0	0	1	0
CALM1	1	0	1	1	0	1	1	0	1
CALM2	1	0	1	1	0	1	1	0	1
CALM3	0	0	0	0	0	0	1	0	1
CALML4	0	0	0	0	0	1	0	0	1
CALML6	0	0	0	0	1	0	0	0	0
CALN1	0	1	0	0	1	0	0	1	0
CALR	1	0	1	1	0	1	1	0	1
CALU	1	0	1	1	0	1	0	0	1
CAMK1D	0	0	0	0	0	0	0	1	0
CAMK1G	0	1	0	0	1	0	0	1	0
CAMK2B	0	1	0	0	1	0	0	1	0
CAMK2D	1	0	1	1	0	1	1	0	1
CAMK2G	0	0	0	0	0	0	0	0	1
CAMKK1	0	0	0	0	0	1	0	0	1
CAMLG	1	0	1	1	0	1	1	0	1
CAMSAP1L1	0	0	0	1	0	1	1	1	1
CAMTA1	1	0	1	1	0	1	1	0	1
CAMTA2	0	0	0	0	0	1	0	0	1
CAND1	1	0	1	1	0	1	1	0	1
CANT1	0	0	0	1	0	1	0	0	1

CANX	1	0	1	1	0	1	0	0	1
CAP1	1	0	1	1	0	1	1	0	1
CAPG	1	0	1	1	0	1	0	0	0
CAPN1	1	0	0	0	0	1	1	0	1
CAPN10	1	0	1	1	0	1	1	0	1
CAPN5	0	0	0	0	0	1	0	1	0
CAPN6	0	1	0	0	1	0	0	1	0
CAPN7	1	0	0	0	0	0	1	0	1
CAPNS1	0	0	0	0	0	0	0	0	1
CARD14	0	1	0	0	1	0	0	0	0
CARD6	1	0	0	1	0	1	1	0	1
CARD8	1	0	1	1	0	1	1	0	1
CARD9	1	0	1	0	0	1	0	0	1
CASC3	1	0	1	1	0	1	1	0	1
CASC4	1	0	1	1	0	1	1	0	1
CASC5	1	0	1	1	0	1	1	0	1
CASD1	0	0	0	0	0	0	0	0	1
CASK	1	1	1	0	1	1	0	1	1
CASKIN1	0	1	0	0	1	0	0	1	0
CASKIN2	0	0	0	0	0	1	0	1	0
CASP1	1	0	1	1	0	1	1	0	1
CASP10	1	0	1	1	0	1	1	0	1
CASP2	1	0	1	1	0	1	1	0	1
CASP3	1	0	1	1	0	1	1	0	1
CASP4	1	0	1	1	0	1	1	0	1
CASP7	0	0	1	0	0	1	0	0	1
CASP8	1	0	1	1	0	1	1	0	1
CASP8AP2	1	0	1	1	0	1	1	0	1
CASP9	1	0	0	0	0	1	0	0	1
CAT	1	0	1	0	0	0	0	0	0
CATSPER1	0	1	0	0	1	0	0	0	0
CATSPER2	1	0	0	0	0	1	1	0	1
CAV1	0	1	0	0	1	0	0	1	0
CBARA1	1	0	1	1	0	1	1	0	1
CBFA2T2	0	0	0	0	0	0	1	0	0
CBFA2T3	0	1	0	0	0	0	0	0	0
CBFB	1	0	1	1	0	1	1	0	1
CBL	1	0	0	1	0	0	1	0	1
CBLB	1	0	1	1	0	1	0	0	1
CBLL1	1	0	1	1	0	1	1	0	1
CBLN2	0	1	0	0	1	0	0	1	0
CBR3	0	0	0	0	1	1	1	1	1
CBR4	1	0	1	1	0	1	1	0	1
CBS	0	1	0	0	1	0	0	1	0
CBX1	0	0	1	1	0	1	1	0	1
CBX2	0	1	1	0	1	1	0	1	1
CBX3	1	0	1	1	0	1	1	0	1
CBX4	0	0	0	0	0	0	0	1	0
CBX5	1	0	1	1	0	1	1	0	1
CBX6	0	0	1	0	1	0	1	1	1
CBX7	0	1	0	0	1	0	0	1	0
CBX8	0	0	1	0	0	1	0	1	0
CC2D1A	0	0	1	1	0	1	1	0	1
CC2D1B	0	0	0	0	0	0	0	1	1
CCAR1	0	0	0	0	0	0	1	0	1
CCBL1	1	0	1	1	0	1	1	0	1
CCDC12	0	0	0	0	0	1	0	0	1
CCDC14	1	0	0	0	0	0	0	0	0
CCDC15	1	1	1	1	1	1	1	1	1
CCDC16	1	1	1	1	0	1	1	0	1
CCDC18	1	0	1	1	0	1	1	0	1
CCDC19	0	1	0	0	0	0	0	0	0

CCDC22	0	1	1	0	0	0	0	0	0
CCDC23	1	0	1	1	0	1	1	0	1
CCDC24	0	0	0	0	0	1	0	0	1
CCDC25	1	0	1	1	0	1	1	0	1
CCDC26	0	0	0	0	1	0	0	0	0
CCDC28A	1	0	1	1	0	1	1	0	1
CCDC3	0	1	0	0	1	0	0	1	0
CCDC32	1	1	1	1	0	1	1	1	1
CCDC34	1	0	0	1	0	1	1	0	1
CCDC4	0	1	1	0	1	1	0	1	1
CCDC41	1	0	1	1	0	1	1	0	1
CCDC42	0	0	0	0	0	1	0	0	0
CCDC43	1	0	1	1	0	1	1	0	1
CCDC45	1	0	1	1	0	1	1	0	1
CCDC47	1	0	1	1	0	1	1	0	1
CCDC49	1	0	0	1	0	1	1	0	1
CCDC5	1	0	1	1	0	1	1	0	1
CCDC50	0	1	0	0	1	0	1	1	1
CCDC51	1	0	1	1	0	1	0	0	1
CCDC53	0	0	0	0	0	1	0	0	1
CCDC55	1	0	1	1	0	1	1	0	1
CCDC56	1	0	1	1	0	1	1	0	1
CCDC58	1	0	1	1	0	1	1	0	1
CCDC59	1	0	1	1	0	1	1	0	1
CCDC6	1	0	1	1	0	1	1	0	1
CCDC60	0	1	0	0	1	0	0	1	0
CCDC64	0	1	1	0	1	0	0	1	1
CCDC66	1	1	1	1	0	1	1	0	1
CCDC69	0	0	0	0	0	0	0	0	1
CCDC72	1	0	1	1	0	1	0	0	1
CCDC76	1	0	1	1	0	1	1	0	1
CCDC8	0	1	1	0	1	0	0	1	0
CCDC81	0	1	0	0	0	0	0	0	0
CCDC82	1	0	1	1	0	1	1	0	1
CCDC84	1	0	1	1	0	1	0	0	1
CCDC85B	1	0	1	1	0	1	1	0	1
CCDC9	0	0	0	1	0	1	0	0	0
CCDC92	1	0	1	1	0	1	1	1	1
CCDC95	1	0	1	1	0	1	0	0	1
CCDC97	1	0	1	1	0	1	1	0	1
CCDC98	0	0	0	0	0	0	1	0	0
CCDC99	1	0	1	1	0	1	1	0	1
CCHCR1	1	0	1	1	0	1	0	0	1
CCL1	0	1	0	0	0	0	0	0	0
CCL13	0	0	1	0	0	0	0	0	0
CCL17	0	0	0	0	0	0	0	1	0
CCL2	0	1	0	0	0	1	0	0	0
CCL20	0	1	0	0	0	0	0	1	0
CCL22	1	0	0	1	0	0	0	0	0
CCL24	0	0	0	0	0	0	0	1	0
CCL27	0	0	0	0	0	0	0	0	1
CCL3	1	0	1	1	0	1	1	0	1
CCL3L1	1	0	0	1	0	1	1	0	1
CCL7	0	1	0	0	0	0	0	0	0
CCM2	1	0	1	1	0	1	1	0	1
CCNA1	0	1	1	0	0	1	0	1	1
CCNA2	1	0	1	1	0	1	1	0	1
CCNB1	0	0	1	0	0	1	1	0	1
CCNB2	1	0	1	1	0	1	1	0	1
CCNC	0	0	0	1	0	1	0	0	1
CCND1	0	1	1	0	1	1	0	1	1
CCND2	0	0	1	1	0	1	0	1	1

CCND3	1	0	0	0	0	0	0	0	1
CCNDBP1	1	0	1	1	0	1	1	0	1
CCNG1	1	0	1	1	0	1	1	0	1
CCNG2	1	0	1	1	0	1	1	0	1
CCNH	1	0	1	1	0	1	1	0	1
CCNI	1	0	1	1	0	1	1	0	1
CCNJ	0	0	0	1	0	1	0	0	0
CCNK	1	0	1	1	0	1	1	0	1
CCNL1	1	0	0	1	0	0	1	0	1
CCNT1	1	0	1	1	0	1	1	0	1
CCNT2	1	0	1	1	0	1	1	0	1
CCPG1	1	0	0	0	0	0	0	0	1
CCR1	1	0	1	1	0	1	1	0	1
CCR10	0	1	0	0	1	1	0	1	0
CCR2	1	0	0	0	0	0	1	0	1
CCR7	0	1	0	0	1	0	0	0	0
CCR9	0	0	0	0	0	0	0	0	0
CCRN4L	1	0	1	1	0	1	1	0	1
CCS	1	0	1	1	0	1	1	0	1
CCT3	1	0	1	1	0	1	1	0	1
CCT4	0	0	1	1	0	1	1	0	1
CCT5	1	0	1	1	0	1	1	0	1
CCT6A	1	0	0	1	0	1	1	0	1
CCT6B	1	1	1	1	0	1	1	0	1
CCT7	1	0	1	1	0	1	1	0	1
CCT8	1	0	1	1	0	0	1	0	1
CD109	0	0	1	1	0	1	0	0	1
CD14	0	0	0	1	0	1	1	0	1
CD151	0	1	0	0	0	0	0	1	0
CD163	1	0	1	1	0	1	1	0	1
CD163L1	0	0	0	0	0	0	1	0	0
CD164	1	0	1	1	0	1	1	0	1
CD180	1	0	1	1	0	1	0	0	1
CD19	1	0	0	0	0	0	0	0	0
CD1A	0	0	0	0	0	0	0	1	0
CD1B	1	0	1	0	0	0	0	1	0
CD1C	1	0	1	0	0	0	1	0	0
CD1E	1	0	1	0	0	0	0	0	0
CD2	1	0	1	0	0	0	0	0	0
CD200	0	1	1	0	1	1	1	0	1
CD200R1	1	0	1	1	0	1	1	0	1
CD209	1	0	1	1	0	1	0	0	0
CD24	0	0	0	0	1	0	0	1	0
CD244	0	0	0	0	0	0	1	0	1
CD274	1	0	0	1	0	1	0	0	1
CD276	0	0	0	0	0	0	0	1	0
CD2AP	0	1	0	1	1	1	1	1	1
CD2BP2	1	0	1	1	0	1	0	0	1
CD300A	0	0	0	0	0	1	0	0	0
CD300E	0	0	0	0	0	0	1	0	1
CD300LB	0	0	1	1	0	1	1	0	1
CD300LF	1	0	1	1	0	1	1	0	1
CD302	0	0	0	0	0	0	1	0	1
CD33	1	0	1	1	0	1	1	0	1
CD36	1	0	1	1	0	1	1	0	1
CD37	0	0	1	1	0	1	1	0	1
CD38	0	1	1	0	0	0	0	0	1
CD3EAP	1	1	1	1	1	1	0	1	1
CD4	0	0	1	0	0	0	1	0	1
CD40	0	0	0	1	0	0	0	0	1
CD44	0	0	1	0	0	1	1	0	1
CD46	1	0	1	1	0	1	1	0	1

CD47	0	1	0	0	1	0	0	1	1
CD48	1	0	1	1	0	1	1	0	1
CD52	1	0	1	1	0	1	1	0	1
CD53	1	0	1	1	0	1	1	0	1
CD58	0	0	0	0	0	0	0	0	1
CD59	0	1	0	0	0	0	0	1	0
CD6	0	1	0	0	1	0	0	1	0
CD63	1	0	0	0	0	0	0	0	0
CD68	1	0	1	1	0	1	1	0	1
CD69	0	0	0	0	0	1	1	0	1
CD74	1	0	0	1	0	0	1	0	1
CD80	1	0	1	1	0	0	1	0	0
CD81	0	1	0	0	1	0	0	1	0
CD82	0	0	1	1	0	1	0	0	0
CD84	1	0	1	1	0	1	1	0	1
CD86	1	0	1	1	0	1	1	0	1
CD9	0	0	0	1	0	1	0	1	0
CD93	1	0	0	1	0	1	1	0	1
CD97	0	0	0	1	0	1	0	0	1
CD99	0	0	0	0	0	0	0	1	0
CD99L2	0	0	0	0	1	0	0	0	0
CDA	0	1	0	0	1	0	0	0	0
CDADC1	1	0	1	1	0	1	1	0	1
CDAN1	0	0	0	1	0	1	0	0	0
CDC14A	0	0	1	1	0	1	1	0	1
CDC2	1	0	1	1	0	1	1	0	1
CDC20	1	0	1	1	0	1	1	0	1
CDC23	1	0	1	1	0	1	1	0	1
CDC25B	0	0	0	1	0	0	0	0	0
CDC25C	0	0	0	1	0	1	1	0	1
CDC26	1	0	1	1	0	1	1	0	1
CDC27	1	0	1	1	0	1	1	0	1
CDC2L1	1	0	1	1	0	1	1	0	1
CDC2L2	1	0	1	1	0	1	1	0	1
CDC2L5	1	0	1	1	0	1	1	0	1
CDC2L6	1	0	0	0	0	0	1	0	1
CDC37	1	0	1	1	0	1	1	1	1
CDC37L1	1	1	1	0	1	1	1	1	1
CDC40	1	0	1	1	0	1	1	0	1
CDC42BPA	0	1	1	0	1	1	0	1	1
CDC42BPB	1	0	0	1	1	1	0	1	1
CDC42EP1	0	1	0	0	1	0	0	1	0
CDC42EP2	0	0	1	0	0	0	0	1	1
CDC42EP3	1	0	0	1	0	1	1	0	1
CDC42EP5	0	1	0	0	1	0	0	1	0
CDC42SE1	1	0	1	1	0	1	1	0	1
CDC42SE2	1	0	1	1	0	1	1	0	1
CDC45L	1	0	1	1	0	1	1	0	1
CDC5L	1	0	1	1	0	1	1	0	1
CDC7	1	0	1	1	0	1	1	0	1
CDC73	0	1	0	0	0	1	0	1	1
CDCA2	1	0	1	1	0	1	1	0	1
CDCA3	1	0	1	1	0	1	1	0	1
CDCA4	1	0	1	1	0	1	1	0	1
CDCA5	1	0	1	1	0	1	1	0	1
CDCA7L	0	0	0	0	0	1	0	0	1
CDCA8	1	0	1	1	0	1	1	0	1
CDCP1	0	0	0	0	0	0	0	1	1
CDGAP	1	0	1	1	0	1	1	0	1
CDH1	0	1	0	0	1	0	0	1	0
CDH15	0	1	0	0	1	0	0	1	0
CDH2	0	1	1	0	1	0	0	1	1

CDH23	0	1	0	0	1	0	0	1	0
CDH26	0	0	0	1	0	0	1	0	1
CDH7	0	1	0	0	1	0	0	1	0
CDIPT	1	0	0	1	0	0	0	0	1
CDK2	1	0	1	1	0	1	1	0	1
CDK2AP1	1	0	0	0	0	1	1	0	1
CDK2AP2	0	0	1	0	0	0	0	1	0
CDK4	1	0	1	1	0	1	1	1	1
CDK5	1	0	1	1	0	1	1	0	1
CDK5R1	0	0	0	0	1	0	0	0	0
CDK5RAP1	0	0	1	0	0	0	0	0	1
CDK5RAP2	0	0	1	0	0	0	0	0	1
CDK6	0	0	0	0	0	1	1	0	1
CDK7	1	0	1	1	0	1	1	0	1
CDK8	1	0	1	1	0	1	1	0	1
CDKAL1	1	0	1	1	0	1	0	0	1
CDKL1	1	0	1	0	0	1	1	0	1
CDKL3	1	0	1	1	0	1	1	0	1
CDKN1A	1	0	1	1	0	1	0	1	0
CDKN1B	0	0	0	1	0	1	1	0	1
CDKN1C	0	1	0	0	0	0	0	1	0
CDKN2A	1	1	1	1	1	1	1	1	1
CDKN2C	1	0	1	1	0	1	1	0	1
CDKN2D	1	0	1	1	0	1	1	0	1
CDKN3	0	0	0	0	0	1	0	0	0
CDR2	1	0	1	1	0	1	1	1	1
CDR2L	0	1	0	0	1	0	0	1	0
CDS1	1	1	1	1	0	1	1	1	1
CDS2	1	0	1	1	0	1	1	0	1
CDV3	1	0	1	1	0	1	1	0	1
CDY2B	0	0	0	0	0	0	0	1	0
CDYL	0	1	0	0	1	1	0	1	1
CDYL2	0	1	0	0	0	0	0	1	0
CEACAM1	0	1	0	0	0	0	0	0	0
CEACAM16	0	1	0	0	1	0	0	1	0
CEACAM21	1	0	1	1	0	1	1	0	1
CEACAM3	0	0	0	0	0	0	0	1	0
CEACAM4	1	0	1	0	0	1	0	0	1
CEACAM8	0	1	0	0	0	0	0	0	0
CEBPA	1	0	0	0	0	0	0	0	0
CEBPG	1	0	1	1	0	1	1	0	1
CEBPZ	1	0	1	1	0	1	1	0	1
CECR5	0	0	0	1	0	1	0	0	1
CECR6	0	0	1	0	0	0	0	1	1
CEECAM1	0	1	0	0	1	0	0	1	0
CELSR1	0	0	0	0	0	1	0	1	0
CELSR2	0	1	0	0	0	0	0	1	0
CELSR3	0	0	0	0	0	0	0	1	1
CENPA	1	0	1	1	0	1	1	0	1
CENPC1	1	0	0	1	0	1	0	0	1
CENPE	1	0	1	1	0	1	1	0	1
CENPF	1	0	1	1	0	1	1	0	1
CENPH	1	0	1	1	0	1	0	0	1
CENPJ	1	0	1	0	0	1	1	0	1
CENTA2	1	0	1	1	0	1	1	0	1
CENTB2	1	0	1	1	0	1	1	0	1
CENTD2	1	0	1	1	0	1	0	0	1
CENTG1	0	0	1	1	0	1	1	0	1
CENTG3	0	0	0	0	0	1	0	1	1
CEP135	1	0	1	1	0	1	1	0	1
CEP152	1	0	1	1	0	1	1	0	1
CEP192	0	0	0	1	0	0	1	0	0

CEP250	1	0	0	1	0	1	1	0	1
CEP27	1	0	1	1	0	1	1	0	1
CEP290	1	0	1	1	0	1	1	0	1
CEP350	1	0	1	1	0	1	1	0	1
CEP55	1	0	1	1	0	1	1	0	1
CEP57	1	0	1	1	0	1	1	0	1
CEP63	1	0	1	1	0	1	1	0	1
CEP68	1	0	1	1	0	1	0	0	1
CEP70	0	1	1	1	0	1	1	1	1
CEP76	1	0	1	1	0	1	1	0	1
CEPT1	1	0	1	1	0	1	1	0	1
CER1	0	1	0	0	0	0	0	0	0
CERKL	0	1	0	0	1	1	0	1	0
CES1	0	0	0	1	0	0	0	0	0
CES2	1	0	1	1	0	1	1	0	1
CES7	0	1	0	0	1	0	0	0	0
CETN2	1	1	1	1	1	1	0	0	1
CETN3	1	0	1	1	0	1	1	0	1
CFDP1	1	0	1	1	0	1	1	0	1
CFH	1	0	1	1	0	1	1	0	1
CFL1	1	0	1	1	0	1	1	0	1
CFL2	1	1	1	1	0	1	1	1	1
CFLAR	1	0	0	1	0	0	1	0	1
CFP	0	1	0	0	0	0	0	0	1
CGGBP1	1	0	1	1	0	1	1	0	1
CGI-09	1	0	1	1	0	1	1	0	1
CGN	0	1	1	0	1	1	0	1	1
CGNL1	0	1	0	0	1	0	0	1	0
CGRRF1	0	0	0	0	0	0	0	0	1
CH25H	1	0	1	1	1	1	1	1	1
CHAC2	1	0	1	1	0	1	1	0	1
CHAD	0	1	0	0	1	0	0	1	0
CHAF1A	1	0	1	1	0	1	1	0	1
CHCHD1	1	0	1	1	0	1	1	0	1
CHCHD3	1	0	1	1	0	1	0	0	1
CHCHD4	1	0	1	1	0	1	1	0	1
CHCHD5	1	0	1	1	0	1	1	0	1
CHCHD6	0	1	0	0	1	1	0	1	1
CHCHD7	1	1	1	0	1	1	1	1	1
CHCHD8	1	0	1	1	0	1	1	0	1
CHD1	1	0	1	1	0	1	1	0	1
CHD1L	1	0	1	1	0	1	1	0	1
CHD3	0	0	1	1	0	1	1	0	1
CHD6	0	1	0	0	1	1	0	1	1
CHD7	1	1	1	1	1	1	0	1	1
CHD8	1	0	1	1	0	1	1	0	1
CHDH	0	1	1	0	1	1	0	1	1
CHEK1	1	0	1	1	0	1	1	0	1
CHEK2	1	0	1	1	0	1	1	0	1
CHERP	1	0	1	1	0	1	1	0	1
CHFR	1	0	1	1	0	1	1	0	1
CHI3L1	0	0	0	0	0	1	0	0	0
CHIC2	0	0	0	1	0	1	1	0	1
CHKB	0	0	0	0	0	1	0	0	0
CHM	1	1	1	1	1	1	1	1	1
CHML	0	0	0	0	0	1	0	0	0
CHMP1B	1	0	1	1	0	1	1	0	1
CHMP2A	1	0	1	1	0	1	1	0	1
CHMP2B	1	1	1	1	0	1	1	1	1
CHMP4A	1	0	1	1	0	1	1	0	1
CHMP4B	1	0	0	1	0	0	1	0	1
CHMP4C	1	1	1	0	1	1	1	1	1

CHMP5	1	0	1	1	0	1	1	0	1
CHMP6	0	0	0	0	1	0	0	0	0
CHN2	0	1	0	0	1	0	0	1	0
CHORDC1	1	0	1	1	0	1	1	0	1
CHP	1	0	1	1	0	1	1	0	1
CHPT1	1	0	1	1	0	1	1	0	1
CHRAC1	0	0	0	1	0	0	0	0	0
CHRD	0	1	0	0	1	0	0	1	0
CHRM4	0	1	0	0	1	0	0	1	0
CHRNA3	0	1	0	0	1	0	0	1	0
CHRNA5	0	1	0	0	1	0	0	1	0
CHRNB1	0	1	0	0	1	0	0	1	0
CHRNB2	0	1	1	0	1	0	0	1	0
CHRNE	0	1	0	0	0	0	0	1	0
CHST1	0	1	0	0	1	0	0	1	0
CHST10	1	0	0	0	0	0	0	0	1
CHST2	0	1	1	1	1	1	0	1	1
CHST3	0	1	0	0	1	0	0	1	0
CHST7	0	1	1	0	1	1	0	1	0
CHST8	0	1	0	0	1	0	0	1	0
CHSY1	1	0	1	1	0	1	1	0	1
CHUK	1	0	0	0	0	1	0	0	1
CHURC1	1	0	1	1	0	1	1	0	1
CHX10	0	1	0	0	1	0	0	1	0
CIAPIN1	1	0	1	1	0	1	1	0	1
CIB1	1	0	1	1	0	1	1	0	1
CIC	1	0	1	1	0	1	0	0	1
CIDEB	0	0	0	0	0	1	1	0	1
CIDEC	1	0	1	1	0	1	1	0	1
CIITA	1	0	1	1	0	1	1	0	1
CINP	1	0	1	1	0	1	1	0	1
CIP29	1	0	1	1	0	1	1	0	1
CIR	1	0	1	1	0	1	1	0	1
CIRBP	1	0	1	1	0	1	1	0	1
CIRH1A	1	0	1	1	0	1	1	0	1
CISH	1	0	1	1	0	1	1	0	1
CIT	0	0	0	0	0	1	0	0	0
CITED1	0	1	1	1	1	1	0	1	1
CITED2	1	0	1	1	0	1	1	0	1
CITED4	0	0	0	0	0	1	0	0	0
CIZ1	0	1	0	0	1	0	0	1	0
CKAP2	1	0	1	1	0	1	1	0	1
CKAP2L	0	0	0	0	0	0	0	0	1
CKAP4	0	0	0	1	0	1	1	0	1
CKAP5	1	0	0	1	0	1	1	0	1
CKB	0	1	0	0	1	0	0	1	0
CKLF	1	0	1	1	0	1	1	0	1
CKM	0	1	0	0	1	0	0	1	0
CKS1B	1	0	1	1	0	1	1	0	1
CKS2	0	0	1	0	0	0	0	0	0
CLASP1	1	0	1	1	0	1	1	0	1
CLASP2	1	0	1	1	0	1	1	0	1
CLC	0	1	0	0	0	0	1	0	0
CLCN1	1	0	0	0	0	0	0	0	0
CLCN2	1	0	1	1	0	1	1	0	1
CLCN3	1	0	1	1	0	1	1	0	1
CLCN5	0	1	0	0	1	0	0	0	0
CLCN6	1	0	1	1	0	1	1	0	1
CLCN7	0	0	0	1	0	1	0	0	0
CLDN1	1	1	1	0	1	1	0	1	0
CLDN12	0	0	0	0	0	1	1	1	1
CLDN23	1	1	1	1	0	1	1	1	1

CLDN5	0	1	0	0	1	0	0	1	0
CLDN7	0	0	1	0	0	1	1	1	1
CLDN9	0	1	0	0	1	0	0	1	0
CLDND1	1	0	1	1	0	1	1	0	1
CLDND2	0	0	0	0	0	0	0	0	1
CLEC10A	1	0	1	0	0	0	0	0	0
CLEC11A	0	1	0	0	1	1	0	1	0
CLEC12A	1	0	0	1	0	1	1	0	1
CLEC14A	0	1	0	0	1	0	0	1	0
CLEC1B	0	0	0	0	0	0	1	0	0
CLEC2B	1	0	1	1	0	1	1	0	1
CLEC4A	1	0	0	1	0	1	1	0	1
CLEC4D	0	0	0	1	0	0	1	0	1
CLEC4E	1	0	1	1	0	1	1	0	1
CLEC5A	0	0	0	0	0	1	0	0	0
CLEC6A	0	0	0	1	0	0	1	0	1
CLEC7A	1	0	1	1	0	0	1	0	1
CLIC1	1	0	1	1	0	1	1	0	1
CLIC2	1	0	1	0	0	0	0	0	0
CLIC3	0	0	0	0	0	0	0	1	0
CLIC6	0	1	1	0	1	0	0	1	0
CLK1	0	0	0	0	0	0	1	0	1
CLK2	0	0	0	1	0	0	0	0	1
CLK4	1	0	0	1	0	1	1	0	1
CLN3	1	0	1	1	0	1	1	0	1
CLN6	0	0	1	0	0	1	0	0	1
CLN8	1	0	1	1	0	1	1	0	1
CLNS1A	1	0	0	1	0	1	1	0	1
CLPTM1	1	0	1	1	0	1	1	0	1
CLPX	0	0	0	0	0	1	0	0	1
CLSTN1	0	0	0	0	0	0	0	0	1
CLTC	1	0	1	1	0	1	1	0	1
CLTCL1	0	1	1	1	1	1	1	0	1
CLUAP1	0	0	1	0	0	0	0	1	0
CLUL1	0	1	0	0	1	0	0	1	0
CLYBL	1	0	1	1	0	1	1	0	1
CMIP	0	0	0	0	0	0	0	1	0
CMKLR1	0	0	0	0	0	0	0	1	0
CMPK	1	0	1	1	0	1	1	0	1
CMTM2	0	1	0	0	1	0	0	1	0
CMTM3	0	0	0	0	0	0	0	0	1
CMTM4	0	0	0	0	0	1	0	0	0
CMTM6	0	0	1	0	0	0	0	0	0
CMTM7	1	0	1	1	0	1	1	0	1
CMTM8	0	1	0	0	1	1	0	1	1
CNDP2	1	0	1	1	0	1	1	0	1
CNFN	0	1	0	0	1	0	0	1	0
CNIH	1	0	0	1	0	1	0	0	1
CNIH2	0	0	0	0	0	0	0	1	0
CNIH3	1	1	1	0	1	1	0	1	1
CNNM2	0	1	0	0	1	1	0	1	0
CNNM3	1	0	1	0	0	1	1	0	1
CNO	0	0	0	0	0	1	0	0	1
CNOT1	1	0	0	1	0	1	1	0	1
CNOT10	1	0	1	1	0	1	1	0	1
CNOT2	1	0	1	1	0	1	1	0	1
CNOT3	1	1	1	1	0	1	0	0	1
CNOT4	1	0	1	1	0	1	1	0	1
CNOT7	1	0	1	1	0	1	1	0	1
CNOT8	1	0	0	0	0	1	0	0	1
CNP	1	0	1	1	0	1	1	0	1
CNTFR	0	0	0	0	1	1	0	1	1

CNTNAP1	0	1	0	0	1	1	0	1	1
CNTNAP5	0	1	1	0	1	0	0	1	0
CNTROB	1	0	1	1	0	1	1	0	1
COASY	1	0	1	1	0	1	1	0	1
COBL	0	1	0	0	1	0	0	1	0
COBLL1	0	0	0	0	0	0	0	0	1
COCH	0	1	0	0	1	0	0	1	1
COG1	0	0	1	0	0	1	0	0	1
COG2	0	0	0	1	0	1	1	0	1
COG3	1	0	1	1	0	1	1	0	1
COG4	1	0	1	1	0	1	1	0	1
COG5	1	0	1	1	0	1	1	0	1
COG7	1	0	1	1	0	1	0	0	1
COG8	1	0	1	1	0	1	1	0	1
COIL	0	0	0	1	0	0	0	0	0
COL11A1	0	1	0	0	1	0	0	1	0
COL15A1	0	1	0	0	1	0	0	1	0
COL16A1	0	1	0	0	1	0	0	1	0
COL22A1	0	1	0	0	1	0	0	1	0
COL23A1	0	1	0	0	1	1	0	1	1
COL24A1	0	1	0	0	0	1	1	0	1
COL25A1	0	1	1	0	1	1	0	1	1
COL27A1	0	1	0	0	1	0	0	1	0
COL4A1	0	1	1	0	1	1	0	1	0
COL4A2	0	1	0	0	1	0	0	1	0
COL4A3BP	1	0	1	1	0	1	1	0	1
COL5A1	0	1	0	0	1	0	0	1	0
COL6A1	0	1	0	0	1	0	0	1	0
COL6A3	0	1	0	0	0	0	0	0	0
COL7A1	0	0	0	0	0	0	0	1	0
COL9A1	0	1	0	0	1	0	0	1	0
COL9A2	0	1	0	0	1	0	0	1	0
COLEC12	0	1	0	0	1	1	0	1	1
COLQ	0	0	0	0	0	0	1	0	1
COMMD1	0	0	1	1	0	1	1	0	1
COMMD10	1	0	1	1	0	1	1	0	1
COMMD2	1	0	1	1	0	1	1	0	1
COMMD3	1	0	1	1	0	1	1	0	1
COMMD5	1	0	1	0	0	0	1	0	1
COMMD8	0	0	0	0	0	1	0	0	0
COMMD9	1	0	1	1	0	1	1	0	1
COMP	0	1	1	0	1	0	0	1	0
COMT	1	0	1	1	0	1	1	0	1
COMTD1	1	0	0	1	0	0	0	0	0
COP1	1	0	1	1	0	1	1	0	1
COPA	1	0	1	1	0	1	1	0	1
COPB2	1	0	1	1	0	1	1	0	1
COPE	1	0	1	1	0	1	1	0	1
COPG	1	0	1	1	0	1	1	0	1
COPG2	1	0	1	1	0	1	1	0	1
COPS2	1	0	1	1	0	1	1	0	1
COPS3	0	0	1	1	0	1	0	0	1
COPS4	1	0	1	1	0	1	1	0	1
COPS5	1	0	1	1	0	1	1	0	1
COPS6	0	0	0	1	0	1	0	0	1
COPS7A	1	0	1	1	0	1	0	0	1
COPS7B	1	0	1	1	0	1	1	0	1
COPS8	1	0	1	1	0	1	1	0	1
COPZ1	1	0	1	1	0	1	1	0	1
COPZ2	0	1	0	0	1	0	0	1	1
COQ10A	0	0	1	1	0	1	0	0	0
COQ10B	1	0	1	1	0	1	1	0	1

COQ2	1	0	1	1	0	1	1	0	1
COQ3	1	0	1	0	0	1	0	0	1
COQ4	1	0	1	1	0	1	1	0	1
COQ5	1	0	1	1	0	1	1	0	1
COQ6	0	0	0	0	0	1	0	0	1
COQ7	1	0	0	0	0	0	0	0	1
COQ9	1	0	1	1	0	1	1	0	1
CORIN	0	1	1	0	1	0	0	1	1
CORO1C	0	0	0	0	0	0	0	0	1
CORO2A	0	0	0	0	0	0	0	0	1
CORO7	1	0	0	0	0	1	0	0	0
COX10	0	0	0	0	0	1	0	0	0
COX11	1	1	1	1	0	1	1	0	1
COX15	1	0	1	1	0	1	1	0	1
COX17	1	0	1	1	0	1	1	0	1
COX4I1	1	0	1	1	0	1	1	0	1
COX4NB	1	0	1	1	0	1	1	0	1
COX5B	0	0	0	0	0	1	0	0	0
COX6A1	1	0	1	1	0	1	0	0	1
COX6B1	1	0	1	1	0	1	1	0	1
COX6C	1	1	1	1	1	1	1	1	1
COX7A2	1	0	1	1	0	1	1	1	1
COX7A2L	1	0	1	0	0	1	1	1	1
COX7B	1	0	1	1	0	1	1	0	1
COX7C	0	0	0	0	0	1	1	0	1
COX8A	1	0	1	1	0	1	0	0	1
CP110	0	0	0	0	0	0	0	0	1
CPA6	1	0	0	0	0	0	1	0	0
CPAMD8	0	1	0	0	0	0	0	1	0
CPD	0	1	1	0	0	1	1	1	1
CPE	0	1	1	0	1	1	0	1	1
CPEB1	0	1	0	0	0	0	0	0	0
CPEB3	1	0	1	1	0	1	1	0	1
CPEB4	1	0	1	1	0	1	1	0	1
CPLX2	0	1	0	0	1	0	0	1	0
CPLX3	0	1	0	0	1	0	0	1	0
CPM	1	0	1	0	0	1	0	1	1
CPNE1	1	0	0	1	0	0	1	0	1
CPNE2	0	0	0	0	0	1	1	0	1
CPNE6	0	1	0	0	1	0	0	0	0
CPNE8	1	0	1	1	0	1	1	0	1
CPNE9	0	1	0	0	0	0	0	1	0
CPOX	0	0	0	1	0	0	0	0	0
CPSF1	0	0	0	0	0	1	0	0	0
CPSF2	1	0	1	1	0	1	1	0	1
CPSF3	1	0	1	1	0	1	1	0	1
CPSF3L	1	0	0	1	0	0	0	0	0
CPSF4	1	0	1	1	0	1	1	0	1
CPSF6	1	0	1	0	0	1	1	0	1
CPT1A	0	0	1	0	0	1	0	0	1
CPT1B	0	0	0	0	0	1	0	1	0
CPT1C	0	1	0	0	1	0	0	1	0
CPVL	1	0	1	1	0	1	1	0	1
CPXM2	0	1	0	0	1	0	0	1	0
CPZ	0	1	0	0	1	0	0	1	0
CRABP2	0	1	0	0	1	0	0	1	0
CRADD	1	0	1	1	0	1	1	0	1
CRAMP1L	1	1	1	1	1	1	1	1	1
CRAT	1	0	1	1	0	1	1	0	1
CREB1	1	0	1	1	0	1	1	0	1
CREB3	1	0	1	1	0	1	1	0	1
CREB3L3	0	1	0	0	0	0	0	0	0

CREB3L4	1	0	1	1	0	1	1	0	1
CREBBP	0	0	0	0	0	0	0	1	0
CREBL1	1	0	1	1	0	1	1	0	1
CREBL2	0	0	1	1	0	1	1	0	1
CREG1	1	0	0	1	0	1	1	0	1
CRELD1	1	0	0	0	0	1	1	0	1
CREM	1	0	1	1	0	1	1	0	1
CRH	0	1	1	0	1	0	0	1	0
CRHBP	0	1	1	0	1	0	0	1	1
CRHR2	0	1	0	0	1	0	0	1	0
CRIM1	0	1	0	0	1	1	0	1	1
CRIP1	0	0	0	0	0	0	0	1	0
CRIP3	0	1	0	0	0	0	0	0	0
CRIPT	1	0	1	1	0	1	1	0	1
CRISPLD2	0	1	0	0	0	0	0	1	1
CRK	0	1	0	0	0	0	0	1	0
CRKRS	1	0	1	1	0	1	1	0	1
CRLF1	0	1	0	0	1	0	0	1	0
CRLF3	0	0	0	1	0	0	0	0	0
CRLS1	0	0	1	0	0	0	0	0	1
CRNKL1	1	0	1	1	0	1	1	0	1
CROP	0	0	0	0	0	0	1	0	1
CROT	1	0	1	1	0	1	1	0	1
CRSP2	1	1	1	1	0	1	1	1	1
CRSP3	1	0	1	1	0	1	1	0	1
CRSP6	1	0	1	1	0	1	0	0	1
CRSP8	1	0	1	1	0	1	1	0	1
CRSP9	1	0	1	1	0	1	1	0	1
CRTAP	1	0	1	1	0	1	0	0	1
CRTC1	1	0	1	1	0	1	1	0	1
CRTC2	0	0	1	0	0	1	0	0	0
CRX	0	1	0	0	1	0	0	1	0
CRY1	1	0	1	1	0	1	0	0	1
CRY2	0	0	0	0	0	0	0	1	0
CRYBB2	0	1	0	0	0	0	0	0	0
CRYGS	0	0	0	1	0	0	0	0	0
CRYZ	1	0	1	1	0	1	1	0	1
CRYZL1	1	0	1	1	0	1	1	0	1
CS	1	0	1	1	0	1	0	0	1
CSAD	1	0	1	1	0	1	1	0	1
CSDA	1	1	1	1	0	1	1	1	1
CSDE1	0	0	0	1	0	1	1	0	1
CSE1L	1	0	0	1	0	1	1	0	1
CSF1	0	0	0	0	0	0	0	1	0
CSF2RA	1	0	1	1	0	1	1	0	1
CSF3R	1	0	1	1	0	1	1	0	1
CSGLCA-T	1	0	0	1	0	1	0	1	1
CSK	1	0	1	0	0	1	1	0	1
CSMD1	0	1	0	0	1	0	0	1	0
CSNK1A1	1	0	1	1	0	1	1	0	1
CSNK1G1	0	0	1	1	0	1	0	0	0
CSNK2A1	0	0	0	0	0	1	0	1	1
CSNK2A2	0	1	0	0	1	0	0	0	0
CSNK2B	1	0	1	1	0	1	1	0	1
CSPG4	0	1	0	0	1	0	0	1	0
CSPG5	0	1	1	0	1	0	0	1	1
CSPP1	1	0	1	1	0	1	1	0	1
CSRP1	1	0	1	1	0	1	1	0	1
CSRP2	0	1	1	1	1	1	0	1	1
CST6	0	1	0	0	1	0	0	1	0
CSTA	1	0	1	1	0	1	1	0	1
CSTF1	1	0	1	1	0	1	1	0	1

CSTF2	0	1	1	0	1	1	0	1	1
CSTF2T	1	0	1	1	0	1	1	0	1
CSTF3	1	0	1	1	0	1	1	0	1
CTAGE5	1	0	1	1	0	1	1	0	1
CTBP2	0	0	0	0	0	0	0	1	1
CTBS	1	0	1	1	0	1	1	0	1
CTCF	1	0	1	1	0	1	0	0	1
CTDP1	1	0	1	1	0	1	0	0	1
CTDSP1	0	0	0	1	0	1	0	0	1
CTDSP2	0	1	0	0	0	0	0	0	0
CTDSPL2	1	0	1	1	0	1	1	0	1
CTH	1	0	1	1	0	1	1	0	1
CTLA4	0	0	0	0	0	0	0	1	0
CTNNA1	0	0	0	0	0	0	0	0	1
CTNNAL1	1	0	0	0	0	0	0	0	1
CTNNB1	1	1	0	0	0	0	0	1	1
CTNNBIP1	1	0	1	1	0	1	1	0	1
CTNNBL1	1	0	1	1	0	1	1	0	1
CTNS	1	0	1	1	0	1	0	0	1
CTPS2	0	1	0	0	1	0	0	1	0
CTRB2	0	1	0	0	1	0	0	1	0
CTSB	0	0	0	0	0	1	0	0	0
CTSC	1	0	0	1	0	1	1	0	1
CTSD	0	0	0	1	0	1	0	0	0
CTSF	0	1	0	0	1	0	0	1	0
CTSG	0	0	0	0	0	0	1	0	0
CTSK	0	0	0	1	0	1	1	0	0
CTSL2	1	1	1	1	0	1	0	1	0
CTSS	1	0	1	1	0	1	1	0	1
CTSZ	0	0	0	1	0	0	0	0	0
CTTN	0	1	0	0	1	0	0	1	0
CTTNBP2NL	1	0	0	1	0	1	0	0	1
CUEDC1	0	0	0	0	0	0	1	0	0
CUEDC2	0	0	0	1	0	1	1	0	1
CUGBP1	1	0	1	1	0	1	1	0	0
CUGBP2	1	1	1	1	1	1	1	1	1
CUL1	1	0	1	1	0	1	1	0	1
CUL2	1	0	1	1	0	1	1	0	1
CUL4A	1	0	1	1	0	1	1	0	1
CUL4B	0	1	0	0	0	0	0	0	0
CUTA	0	1	1	0	0	1	0	0	0
CUTC	1	0	1	1	0	1	1	0	1
CUTL1	1	0	1	1	0	1	1	0	1
CUTL2	0	1	0	0	1	0	0	1	0
CWF19L1	1	0	1	1	0	1	1	0	1
CX3CL1	0	1	0	0	1	0	0	0	0
CX3CR1	0	0	0	0	0	0	1	0	1
CXCL1	0	1	0	1	0	1	1	0	1
CXCL10	0	0	0	0	0	0	1	0	0
CXCL14	0	1	0	0	1	0	0	1	0
CXCL2	1	0	1	1	0	1	1	0	1
CXCL3	1	1	1	1	0	1	1	1	1
CXCL5	0	1	0	0	0	0	0	1	0
CXCR4	1	0	1	1	0	1	1	0	1
CXXC1	1	0	1	1	0	1	1	0	1
CXXC5	0	0	1	0	1	1	0	1	1
CXORF21	1	0	1	1	0	1	1	0	1
CXORF23	0	1	0	0	1	0	0	1	0
CXORF26	1	1	0	0	1	1	0	1	0
CXORF34	0	1	0	0	1	1	0	0	1
CXORF38	0	1	1	0	1	0	0	1	0
CXORF39	0	1	1	0	1	1	1	0	1

CXORF40A	0	0	0	0	0	0	0	0	1
CXORF42	1	1	1	1	1	1	1	1	1
CXORF45	0	1	0	0	1	0	0	0	0
CXORF48	0	1	0	0	0	0	0	0	0
CXORF56	1	1	1	1	1	1	1	1	1
CXORF6	0	1	0	0	0	0	0	0	0
CXORF9	1	1	1	0	0	1	0	0	1
CYB561	0	0	1	0	0	1	1	0	1
CYB561D1	0	0	0	1	0	0	0	0	0
CYB561D2	1	0	1	1	0	1	1	0	1
CYB5A	1	0	1	1	0	1	1	0	1
CYB5B	1	0	1	1	0	1	1	0	1
CYB5D1	1	0	1	1	0	1	1	0	1
CYB5D2	1	0	1	1	0	1	1	0	1
CYB5R1	1	0	0	1	0	1	1	0	1
CYB5R2	0	1	0	0	1	0	0	1	1
CYB5R3	1	0	1	0	0	1	0	0	0
CYB5R4	1	1	1	1	1	1	1	1	1
CYBASC3	1	0	1	1	0	1	1	0	1
CYBB	1	0	0	1	0	0	1	0	1
CYBRD1	1	0	1	1	0	1	1	0	1
CYCS	1	0	1	1	0	1	1	0	1
CYFIP1	0	0	0	0	0	1	1	1	1
CYFIP2	0	0	0	0	0	1	1	0	1
CYGB	0	1	0	0	1	0	0	1	0
CYP11A1	0	1	0	0	1	0	0	1	0
CYP19A1	0	1	0	0	1	0	0	1	0
CYP1B1	0	0	0	0	0	1	0	0	1
CYP20A1	0	0	0	1	0	0	1	0	1
CYP26A1	0	1	0	0	1	0	0	1	0
CYP26C1	0	1	0	0	1	0	0	1	0
CYP27A1	0	0	0	1	0	1	0	1	0
CYP27B1	0	0	1	1	0	1	1	1	1
CYP2A13	0	1	0	0	1	0	0	1	0
CYP2S1	0	0	1	1	0	1	1	1	1
CYP2U1	0	0	1	0	0	1	1	0	1
CYP39A1	1	1	1	0	1	1	1	1	1
CYP3A5	0	1	0	0	0	0	0	0	0
CYP4V2	1	0	1	1	0	1	0	0	1
CYP4X1	0	1	0	0	1	0	0	1	0
CYP51A1	1	0	1	1	0	1	1	0	1
CYSLTR1	1	1	1	1	0	1	1	0	1
CYYR1	0	1	0	0	1	0	0	1	0
CYORF15A	0	0	0	0	1	0	0	0	0
D15WSU75E	1	0	1	1	0	1	1	0	1
DAAM1	1	1	1	1	1	1	1	1	1
DAB2	1	0	1	1	0	1	1	1	0
DACH1	1	1	1	1	1	1	1	1	1
DACT1	0	1	1	0	1	1	0	1	1
DAD1	1	0	1	1	0	1	1	0	1
DAK	0	0	0	1	0	1	1	0	1
DAP	1	0	0	0	0	0	1	0	1
DAP3	1	0	1	1	0	1	1	0	1
DAPK2	0	1	0	0	1	0	0	1	0
DAPK3	1	0	1	1	0	1	1	0	1
DAPP1	0	0	0	0	0	1	1	0	1
DARS	1	0	0	0	0	1	1	0	1
DARS2	1	0	1	1	0	1	1	0	1
DAXX	1	0	1	1	0	1	1	0	1
DAZAP2	0	0	1	0	0	1	1	0	1
DBF4B	1	0	1	1	0	1	1	0	1
DBI	1	0	1	1	0	1	1	0	1

DBN1	0	1	0	0	0	0	0	1	0
DBNDD2	1	0	0	1	1	1	0	1	1
DBNL	1	0	0	0	0	0	0	0	0
DBP	0	0	0	1	0	1	0	0	1
DBR1	1	0	1	1	0	1	1	0	1
DBT	1	0	1	1	0	1	1	0	1
DC2	1	0	1	1	0	1	1	0	1
DCAKD	1	0	1	1	0	1	1	0	1
DCBLD1	0	1	0	0	0	1	0	1	1
DCBLD2	0	1	0	0	0	1	0	0	1
DCD	0	1	0	0	0	0	0	0	0
DCDC2	0	1	0	0	1	0	0	1	0
DCHS2	0	1	1	0	1	0	0	1	1
DCK	0	0	0	1	0	1	0	0	1
DCLRE1A	1	0	1	1	0	1	1	0	1
DCLRE1B	1	0	1	1	0	1	1	0	1
DCLRE1C	1	0	0	1	0	1	1	0	1
DCP1A	0	0	0	1	0	1	0	0	1
DCP1B	0	0	0	0	0	1	0	0	0
DCP2	1	0	1	1	0	1	1	0	1
DCPS	0	0	0	1	0	0	0	0	1
DCTD	1	0	1	1	0	1	1	0	1
DCTN1	1	0	0	1	0	0	0	0	0
DCTN2	1	1	1	1	1	1	1	1	1
DCTN3	1	1	1	1	1	1	0	1	1
DCTN4	1	0	1	1	0	1	1	0	1
DCTN5	1	0	1	1	0	1	1	0	1
DCTN6	1	0	1	1	0	1	1	0	1
DCUN1D2	1	0	1	1	0	1	1	0	1
DCUN1D3	1	0	1	1	0	1	0	0	1
DCUN1D4	0	1	1	1	1	1	1	0	1
DCUN1D5	0	1	1	0	1	1	1	1	1
DCX	0	1	0	0	1	0	0	1	0
DCXR	0	0	0	0	0	1	0	0	1
DDAH2	0	0	0	0	0	0	0	0	1
DDB1	0	0	0	1	0	1	1	0	1
DDB2	0	0	0	0	0	0	0	0	1
DDC	0	1	0	0	1	0	0	0	0
DDEF2	1	0	1	1	0	1	1	1	1
DDHD1	0	0	0	0	0	0	0	0	1
DDI2	1	0	1	1	0	1	0	0	1
DDIT3	1	0	1	1	0	1	0	0	1
DDIT4	0	0	1	0	0	0	0	0	1
DDIT4L	0	1	1	0	1	1	1	1	1
DDO	1	0	1	1	0	1	0	0	0
DDOST	0	0	1	1	0	1	0	0	1
DDR1	0	0	0	0	1	0	0	0	0
DDT	0	1	0	0	1	0	0	1	1
DDX10	1	0	1	0	0	1	1	0	1
DDX17	0	0	0	0	0	0	1	0	0
DDX18	1	1	0	0	1	1	0	1	1
DDX19-DDX19L	1	0	1	1	0	1	1	0	1
DDX19A	1	0	1	1	0	1	1	0	1
DDX19B	1	0	1	1	0	1	1	0	1
DDX20	1	0	1	1	0	1	1	0	1
DDX21	1	0	1	1	0	1	1	0	1
DDX23	1	0	1	1	0	1	1	0	1
DDX24	1	0	1	1	0	1	1	0	1
DDX25	1	1	1	1	1	1	1	1	1
DDX27	1	0	1	1	0	1	1	0	1
DDX28	1	0	1	1	0	1	1	0	1
DDX31	1	0	1	1	0	1	1	0	1

DDX39	0	0	0	0	0	1	0	0	0
DDX3X	1	0	1	1	0	1	1	1	1
DDX41	1	0	1	1	0	1	0	0	0
DDX42	1	0	1	1	0	1	1	0	1
DDX46	1	0	1	1	0	1	1	0	1
DDX47	1	0	1	1	0	1	1	0	1
DDX49	1	0	1	1	0	1	1	0	1
DDX5	1	0	1	1	0	1	1	0	1
DDX50	0	0	0	1	0	1	0	0	1
DDX51	1	0	0	1	0	1	0	0	1
DDX52	1	1	1	1	1	1	1	1	1
DDX54	1	0	1	1	0	1	1	0	1
DDX55	1	0	1	0	0	1	0	0	0
DDX56	1	0	0	1	0	1	0	0	1
DDX58	1	0	1	1	0	1	1	0	1
DDX59	1	0	1	1	0	1	1	0	1
DDX6	1	0	0	1	0	1	1	0	1
DEADC1	1	0	1	1	0	1	1	0	1
DEAF1	1	0	1	1	0	1	1	0	1
DECR1	0	0	1	1	0	1	0	0	1
DECR2	0	0	0	0	0	0	0	0	1
DEDD	1	0	1	1	0	1	1	0	1
DEDD2	1	0	1	1	0	1	0	0	1
DEFA4	0	1	0	0	1	0	0	1	0
DEFB108B	0	0	0	0	0	0	0	1	0
DEFB125	0	1	0	1	1	0	0	1	0
DEFB126	0	1	0	0	1	0	0	1	0
DEFB128	0	1	0	0	0	0	0	0	0
DEGS1	1	0	0	0	0	0	1	0	1
DEK	0	0	1	0	0	1	0	0	1
DENND1C	0	0	0	1	0	1	0	0	0
DENND2C	0	0	1	1	1	1	1	0	1
DENND2D	1	0	1	1	0	1	0	0	1
DENND3	0	0	0	1	0	1	1	0	1
DENND4A	0	0	0	1	0	1	1	0	1
DENR	0	0	0	0	0	0	1	0	0
DEPDC1	0	0	1	1	0	1	1	0	1
DEPDC5	1	0	1	1	0	1	1	0	1
DEPDC6	0	0	0	1	0	1	0	0	0
DEPDC7	1	0	1	0	0	1	1	1	1
DERA	1	0	0	0	0	0	0	0	0
DERL1	0	0	0	0	0	0	1	0	1
DERL2	1	0	1	1	0	1	1	0	1
DEXI	0	0	0	1	0	1	0	0	0
DFFA	0	0	1	0	0	1	0	0	1
DFFB	0	0	0	0	0	1	0	0	0
DFNA5	0	1	0	1	1	1	0	1	1
DGAT2L3	0	1	0	0	0	0	0	0	0
DGCR14	1	1	1	1	0	1	1	1	1
DGKA	1	0	1	1	0	1	1	0	1
DGKD	0	0	0	0	0	0	0	0	1
DGKE	0	0	1	1	0	1	0	0	0
DGKG	0	0	0	0	0	1	1	0	1
DGKZ	1	0	1	1	1	1	1	1	1
DGUOK	1	0	1	1	0	1	0	0	1
DHCR24	0	1	1	0	0	1	0	1	1
DHCR7	1	0	1	1	0	1	1	0	1
DHDDS	1	1	1	1	0	1	1	1	1
DHDH	1	1	0	0	0	0	0	0	0
DHFR	1	0	1	1	0	1	1	0	1
DHFRL1	1	0	1	1	0	1	1	0	1
DHODH	0	0	1	1	0	1	0	0	1

DHPS	1	0	1	1	0	0	1	0	1
DHRS1	1	0	1	1	0	1	0	0	1
DHRS3	0	0	1	1	0	1	1	1	1
DHRS4	0	0	1	1	0	1	0	0	1
DHRS4L2	1	0	1	1	0	1	1	0	1
DHRS7	0	0	0	0	0	0	1	0	0
DHRS7B	1	0	1	1	0	1	1	0	1
DHRS8	1	0	1	1	0	1	1	0	1
DHX15	0	0	0	0	0	0	0	0	1
DHX16	1	0	1	1	0	1	1	0	1
DHX29	1	0	1	1	0	1	1	0	1
DHX30	0	0	0	0	0	0	1	0	1
DHX33	0	0	0	0	0	1	0	0	0
DHX34	1	0	1	1	0	1	1	0	1
DHX35	1	0	1	1	0	1	1	0	1
DHX36	1	0	1	1	0	1	1	0	1
DHX37	0	0	1	1	0	1	1	0	1
DHX38	1	0	1	1	0	1	1	0	1
DHX57	1	0	1	1	0	1	1	0	1
DHX8	1	0	1	1	0	1	1	0	1
DHX9	0	0	0	0	0	1	0	0	1
DICER1	1	0	0	1	0	1	1	0	1
DIDO1	1	0	1	1	0	1	1	0	1
DIO1	1	0	1	1	0	1	1	0	1
DIO3	0	1	0	0	1	0	0	1	0
DIP2A	1	0	1	1	0	1	1	0	1
DIP2C	0	1	1	0	1	0	0	1	0
DIRAS1	0	0	0	0	1	0	0	1	0
DIRAS2	0	1	0	0	1	0	0	1	0
DIRC2	1	0	1	1	0	1	1	0	1
DISC1	0	0	0	0	0	1	0	0	0
DISP1	0	0	0	0	0	0	1	0	0
DIXDC1	0	1	1	0	1	0	1	1	1
DKC1	1	1	1	1	1	1	1	0	1
DKFZP434A0131	1	0	1	1	0	1	1	0	1
DKFZP434B0335	1	0	1	1	0	1	1	0	1
DKFZP564J0863	1	0	0	0	0	0	0	0	0
DKFZP564O0523	1	0	1	1	0	1	1	0	1
DKFZP564O0823	0	1	1	0	1	0	0	1	1
DKFZP586P0123	1	0	1	1	0	1	1	0	1
DKFZP434K1815	0	0	0	1	0	1	0	0	0
DKFZP451M2119	1	0	1	1	0	1	1	0	1
DKFZP666G057	1	1	1	1	1	1	1	1	1
DKFZP686I15217	1	0	1	0	0	1	0	0	1
DKFZP686O24166	0	0	0	0	0	0	0	1	0
DKFZP761B107	1	0	1	1	0	1	1	0	1
DKFZP761E198	0	0	0	0	0	0	0	1	0
DKFZP762E1312	0	0	1	1	0	1	0	0	1
DKKL1	0	1	0	0	1	1	0	1	0
DLAT	1	0	1	1	0	1	1	0	1
DLD	1	0	1	1	0	1	1	0	1
DLEU7	0	1	0	0	0	0	0	0	0
DLG1	1	1	1	1	0	1	0	1	1
DLG4	1	0	1	0	0	1	0	0	1
DLG7	1	0	1	1	0	1	1	0	1
DLGAP1	0	0	0	0	0	0	1	0	0
DLST	1	0	1	1	0	1	1	0	1
DLX2	1	1	1	0	1	1	1	1	1
DLX4	0	1	1	0	1	0	0	1	1
DMAP1	0	0	0	0	0	1	0	0	1
DMN	0	1	0	0	1	0	0	1	0
DMPK	0	1	0	0	1	0	0	1	0

DMRT1	0	1	0	0	1	0	0	1	0
DMRTC1	0	1	0	0	1	0	0	1	0
DMTF1	1	0	1	1	0	1	1	0	1
DMWD	0	1	0	0	1	1	0	1	1
DMXL1	1	0	1	1	0	1	1	0	1
DMXL2	1	0	1	1	0	1	1	0	1
DNAH3	1	0	0	1	0	1	1	0	1
DNAH7	0	0	0	0	0	1	1	0	1
DNAI1	1	0	1	1	0	1	0	1	1
DNAJA1	1	0	1	1	0	1	1	0	1
DNAJA2	1	0	1	1	0	1	1	0	1
DNAJA3	1	0	1	1	0	1	1	0	1
DNAJA5	1	0	1	1	0	1	1	0	1
DNAJB11	1	0	1	1	0	1	1	0	1
DNAJB12	0	0	0	0	0	1	0	0	1
DNAJB14	1	0	1	1	0	1	0	0	1
DNAJB2	0	0	0	0	0	0	1	0	1
DNAJB4	1	0	1	1	0	1	1	0	1
DNAJB5	0	0	1	0	0	0	0	0	1
DNAJB6	0	0	0	0	0	0	0	0	1
DNAJB9	1	0	1	1	0	1	1	0	1
DNAJC1	0	0	0	0	0	0	0	0	1
DNAJC10	1	0	1	1	0	1	1	0	1
DNAJC12	0	0	0	0	0	0	1	0	0
DNAJC13	1	0	1	1	0	1	1	0	1
DNAJC14	0	0	1	0	0	1	1	0	1
DNAJC15	0	0	0	0	0	0	0	0	1
DNAJC16	1	0	0	0	0	1	0	0	1
DNAJC17	0	0	1	1	0	1	1	0	1
DNAJC19	1	1	1	1	0	1	1	0	1
DNAJC3	1	0	1	1	0	1	1	0	1
DNAJC5	0	0	0	0	0	0	0	0	1
DNAJC5B	1	0	0	0	0	0	0	0	0
DNAJC7	1	0	1	1	0	1	1	0	1
DNAJC8	1	0	1	1	0	1	1	0	1
DNAJC9	1	0	0	1	0	0	0	0	1
DNAL4	0	1	0	0	1	0	0	0	0
DNASE1L1	1	1	1	1	1	1	0	0	1
DNASE1L3	0	0	0	0	0	1	0	0	0
DNASE2B	0	0	0	1	0	1	0	0	0
DNM2	0	0	0	1	0	0	0	0	0
DNM3	0	0	0	0	0	1	0	1	1
DNMT1	0	0	0	1	0	1	0	0	1
DNTTIP1	0	0	1	1	0	1	1	0	1
DNTTIP2	1	0	0	1	0	1	1	0	1
DOC2A	0	1	0	0	0	0	0	1	0
DOCK11	0	1	0	0	0	0	0	0	0
DOCK2	1	0	0	0	0	0	0	0	0
DOCK3	0	0	0	0	0	0	0	1	0
DOCK4	1	0	1	1	0	1	1	0	1
DOCK5	1	1	1	0	0	1	0	1	1
DOCK6	0	1	0	0	1	0	0	1	0
DOCK7	1	0	1	1	0	1	1	0	1
DOCK8	1	0	1	0	0	1	1	0	1
DOCK9	0	1	1	0	1	1	0	1	1
DOK2	1	0	1	1	0	1	1	0	1
DOK3	0	0	0	1	0	1	0	0	1
DOLPP1	1	0	1	1	0	1	0	0	1
DOM3Z	1	0	1	1	0	1	0	0	1
DONSON	0	0	0	0	0	1	1	0	1
DOPEY1	1	0	1	1	0	1	1	0	1
DP58	0	1	0	0	0	0	0	1	0

DPAGT1	1	0	1	1	0	1	1	0	1
DPCR1	0	1	0	0	1	0	0	1	0
DPEP2	0	0	0	0	0	1	0	0	1
DPF1	0	1	0	0	1	0	0	1	0
DPH2	1	0	1	0	0	1	0	0	0
DPH5	1	0	1	1	0	1	1	0	1
DPM1	1	0	1	1	0	1	1	0	1
DPM2	1	0	1	1	0	1	0	0	1
DPM3	1	0	1	1	0	1	0	0	1
DPP3	1	0	1	1	0	1	1	0	1
DPP4	0	1	1	0	1	1	0	1	1
DPP8	1	0	1	1	0	1	1	0	1
DPP9	0	0	0	1	0	1	0	0	1
DPY19L2	0	1	1	0	1	1	0	1	1
DPY19L3	0	1	0	0	0	0	0	1	0
DPY19L4	1	0	1	1	0	1	1	0	1
DPYS	0	1	1	0	1	1	0	1	0
DPYSL2	1	0	1	1	0	1	1	1	1
DPYSL3	0	1	1	0	1	1	0	1	1
DPYSL4	0	1	0	0	1	0	0	1	0
DR1	1	0	0	1	0	1	1	0	1
DRAP1	1	0	1	0	0	0	0	0	0
DRD3	0	1	0	0	0	0	0	0	0
DRD4	0	1	0	0	1	0	0	1	0
DRG1	1	0	1	1	0	1	1	0	1
DRG2	1	0	1	1	0	1	1	0	1
DRP2	0	1	0	0	1	0	0	1	0
DSC2	0	1	0	0	1	1	0	1	0
DSCAM	0	1	0	0	1	0	0	1	0
DSCR1	1	0	1	1	0	1	1	0	1
DSCR10	0	1	0	0	0	0	0	1	0
DSCR2	1	0	1	1	0	1	1	0	1
DSCR3	1	0	1	1	0	1	0	0	0
DSCR4	0	0	0	0	0	0	1	1	0
DSCR6	0	1	0	0	0	0	0	1	0
DSP	0	1	1	0	1	1	0	1	1
DSTN	0	1	1	0	1	1	1	1	1
DTL	1	0	1	1	0	1	1	0	1
DTNA	0	1	0	0	1	0	1	1	0
DTNB	0	0	0	0	0	0	0	0	1
DTNBP1	0	0	0	0	0	1	0	0	1
DTWD1	1	0	1	1	0	1	1	0	1
DTWD2	0	0	1	0	0	0	0	0	0
DTX3L	1	0	1	1	0	1	1	0	1
DULLARD	1	0	1	1	0	1	1	0	1
DUOX1	0	1	1	0	1	1	0	1	0
DUS1L	0	0	1	0	0	0	0	0	1
DUS2L	1	0	1	1	0	1	1	0	1
DUS3L	0	0	0	0	0	1	0	0	0
DUS4L	1	0	1	1	0	1	1	0	1
DUSP1	1	0	0	1	0	1	1	0	1
DUSP10	1	0	1	1	0	1	1	0	1
DUSP11	1	0	1	1	0	1	1	0	1
DUSP12	0	0	0	0	0	0	0	0	1
DUSP13	1	0	0	1	0	1	1	0	1
DUSP14	0	0	1	0	0	1	0	1	1
DUSP18	1	1	1	1	0	1	1	1	1
DUSP23	1	0	0	0	0	0	0	0	0
DUSP26	0	1	1	0	1	0	0	1	0
DUSP3	1	0	1	1	0	1	1	0	1
DUSP5	1	0	1	1	0	1	1	1	1
DUSP6	1	0	1	0	0	1	1	0	1

DUSP8	0	0	0	0	0	0	0	1	0
DUSP9	0	1	0	0	1	0	0	1	0
DUT	1	0	1	1	0	1	1	0	1
DVL2	0	0	1	1	0	1	0	0	1
DYNC1I2	1	0	1	1	0	1	1	0	1
DYNC1LI1	0	0	1	1	0	1	0	0	1
DYNC1LI2	0	1	0	1	1	0	0	1	0
DYNLRB1	1	0	1	1	0	1	1	0	1
DYNLT1	0	0	1	1	0	1	0	0	1
DYNLT3	0	1	0	0	0	0	0	0	0
DYRK1A	0	0	0	1	0	0	1	0	0
DYRK2	0	0	0	0	0	1	0	1	1
DYRK3	0	1	1	0	0	1	1	1	1
DYSF	0	0	1	1	0	1	1	0	1
E2F2	1	0	0	0	0	1	0	0	1
E2F3	1	0	1	1	0	1	1	0	1
E2F8	0	1	0	0	0	1	0	0	1
EAF1	1	0	1	1	0	1	1	0	1
EAF2	1	0	1	1	0	1	1	0	1
EARS2	1	0	1	1	0	1	0	0	1
EBAG9	1	0	1	1	0	1	1	0	1
EBI2	1	0	1	1	0	0	1	0	0
EBI3	0	1	0	0	1	0	0	1	0
EBNA1BP2	1	1	1	1	1	1	1	1	1
EBP	0	1	1	0	1	1	1	0	1
ECD	1	0	1	1	0	1	1	0	1
ECE1	0	0	0	0	1	0	0	1	0
ECE2	1	1	1	1	1	1	1	1	1
ECGF1	1	0	0	1	0	1	1	0	1
ECH1	1	0	1	1	0	1	1	0	1
ECHDC1	1	0	1	1	0	1	1	0	1
ECHDC2	0	1	0	0	0	0	0	1	0
ECHDC3	0	1	1	0	0	0	1	0	1
ECOP	0	0	1	1	0	1	1	0	1
ECT2	1	0	1	1	0	1	1	1	1
EDEM1	1	0	0	1	0	1	1	0	1
EDEM2	1	0	1	0	0	1	1	0	1
EDEM3	1	0	1	1	0	1	1	0	1
EDG1	1	1	1	0	1	1	1	1	1
EDG2	0	0	0	1	0	1	0	1	1
EDG3	0	0	0	0	0	0	1	0	1
EDG6	1	0	0	0	0	0	0	0	0
EDN1	1	1	1	1	0	1	1	1	1
EEA1	1	0	1	1	0	1	1	0	1
EED	1	0	1	1	0	1	1	0	1
EEF1A1	1	0	1	1	0	1	1	0	1
EEF1B2	1	0	1	1	0	1	1	0	1
EEF1D	1	0	1	1	0	1	1	0	1
EEF2	1	1	1	1	0	1	1	0	1
EEF2K	1	0	1	1	0	1	0	0	0
EFCAB2	1	0	1	1	0	1	1	0	1
EFHA1	0	1	1	0	1	1	0	1	0
EFHB	1	1	1	1	0	1	1	1	1
EFHC1	0	0	1	0	0	1	0	0	1
EFHC2	0	1	0	0	1	0	1	1	1
EFHD1	0	1	0	0	0	0	0	1	0
EFHD2	0	1	0	0	0	0	0	1	1
EFNA1	1	1	1	0	1	1	0	1	1
EFNA3	0	1	0	0	1	0	0	1	0
EFNA4	0	0	0	0	0	0	0	0	1
EFNB1	0	1	0	0	1	0	0	0	0
EFTUD1	0	1	1	1	1	1	1	1	1

EFTUD2	1	0	1	1	0	1	1	0	1
EGFL7	0	1	0	0	1	0	0	1	0
EGFR	0	1	0	0	1	0	0	1	0
EGLN1	0	1	0	0	1	0	0	1	0
EGLN3	1	1	1	0	1	1	0	1	1
EGR1	0	0	0	0	0	0	0	0	1
EGR2	1	0	1	1	0	1	1	1	1
EGR4	0	1	1	0	1	0	0	1	0
EHBP1	1	0	1	1	0	1	1	0	1
EHD1	0	0	0	0	0	1	0	0	1
EHD4	1	0	0	1	0	0	0	0	0
EHMT1	1	0	1	1	0	1	1	0	1
EHMT2	1	0	1	1	0	1	1	0	1
EIF1	1	0	1	1	0	1	1	0	1
EIF1AX	0	1	0	0	0	1	1	1	1
EIF1B	0	0	0	0	0	1	0	0	0
EIF2A	1	0	1	1	0	1	1	0	1
EIF2AK1	1	0	0	0	0	1	0	0	0
EIF2AK2	1	0	1	1	0	1	1	0	1
EIF2AK3	1	0	0	1	0	1	1	0	1
EIF2AK4	1	0	1	0	0	1	1	0	1
EIF2B1	1	0	1	1	0	1	1	0	1
EIF2B2	1	0	1	1	0	0	1	0	1
EIF2B3	1	0	1	0	0	1	0	0	0
EIF2B4	1	0	1	1	0	1	1	0	1
EIF2B5	1	0	1	1	0	1	1	0	1
EIF2C1	0	0	0	1	0	1	0	0	1
EIF2C3	1	0	0	1	0	0	0	0	0
EIF2C4	0	0	0	0	0	0	0	0	1
EIF2S1	1	0	1	1	0	1	1	0	1
EIF2S2	1	0	1	1	0	1	1	0	1
EIF2S3	0	0	1	1	0	1	0	0	1
EIF3S7	0	0	0	0	0	1	0	0	0
EIF4A1	1	0	1	1	0	1	1	0	1
EIF4A2	1	0	1	1	0	1	1	0	1
EIF4B	0	0	1	0	0	0	0	0	1
EIF4E2	1	0	1	1	0	1	1	0	1
EIF4E3	0	1	0	0	1	0	1	1	1
EIF4EBP2	0	0	0	1	0	0	1	0	1
EIF4ENIF1	1	0	1	1	0	1	1	0	1
EIF4G1	1	0	1	0	0	1	1	0	1
EIF4G2	1	0	1	1	0	1	1	0	1
EIF5	1	0	0	1	0	0	0	0	1
EIF5A2	0	1	0	0	0	0	0	1	1
EIF5B	1	0	1	1	0	1	1	0	1
ELA2	0	1	0	0	1	0	0	0	0
ELAC1	0	0	0	0	0	0	1	0	1
ELAC2	1	0	1	1	0	1	0	0	1
ELAVL1	1	0	1	1	0	1	1	0	1
ELF1	1	0	1	1	0	1	1	0	1
ELF2	1	0	1	1	0	1	1	0	1
ELF5	0	0	0	1	0	0	0	0	0
ELK1	0	1	1	0	1	1	0	0	0
ELK4	1	0	1	1	0	1	1	0	1
ELL2	0	0	0	0	0	0	0	1	0
ELL3	1	0	1	1	0	1	1	0	1
ELMO1	1	1	1	0	1	1	1	0	1
ELMO2	0	0	0	0	0	1	0	0	0
ELMOD2	0	0	0	0	1	1	0	0	1
ELOVL1	1	0	1	1	0	1	1	0	1
ELOVL3	0	1	0	0	0	0	0	0	1
ELOVL5	1	0	1	1	0	1	1	0	1

ELOVL6	0	1	1	0	0	1	0	1	1
ELOVL7	0	1	0	0	1	0	0	1	0
ELP3	1	0	1	1	0	1	1	0	1
ELP4	1	0	1	1	0	1	1	0	1
EME1	1	0	1	1	0	1	1	0	1
EME2	1	0	0	0	0	0	0	0	1
EMG1	1	0	1	1	0	1	1	0	1
EMILIN2	0	0	0	1	0	0	0	0	1
EML2	0	0	1	0	0	1	1	0	1
EML3	1	0	1	1	0	1	0	0	1
EML4	0	0	0	0	0	0	0	1	0
EMP1	1	0	1	1	0	1	0	0	1
EMR1	0	0	0	0	0	0	1	0	1
EMR2	1	0	0	1	0	0	0	0	0
EMR3	0	0	0	1	0	1	1	0	1
EMX2	0	1	1	0	1	0	0	1	0
EN1	0	1	1	0	1	1	0	1	1
ENC1	0	0	1	1	0	1	1	0	1
ENDOG	0	0	0	0	0	0	1	0	1
ENDOGL1	0	0	1	1	0	1	1	0	1
ENO1	1	0	1	1	0	1	1	0	1
ENO2	0	1	0	0	1	1	0	1	1
ENOSF1	0	1	0	0	0	0	0	1	1
ENPP2	1	0	1	1	0	1	1	0	1
ENPP4	1	1	1	1	0	1	1	0	1
ENSA	1	0	1	1	0	1	1	0	1
ENTPD1	1	0	1	1	0	1	1	0	1
ENTPD5	1	0	0	1	0	1	0	0	1
ENTPD7	0	0	0	0	0	1	0	0	1
ENTPD8	0	1	0	0	1	1	0	1	0
ENY2	1	0	1	1	0	1	1	0	1
EP300	1	0	1	1	0	1	1	0	1
EP400	1	0	0	0	0	0	0	0	0
EPAS1	0	0	1	0	0	1	0	1	1
EPB41L1	0	1	0	0	1	0	0	1	0
EPB41L3	0	0	0	0	0	0	0	1	0
EPB41L4A	0	1	0	0	1	0	0	1	1
EPB42	1	0	0	0	0	0	0	0	0
EPB49	0	1	0	0	1	0	0	1	0
EPC1	0	0	0	0	0	1	1	0	0
EPC2	0	0	0	1	0	1	0	0	1
EPDR1	0	1	0	0	1	0	0	1	1
EPHA2	0	0	0	0	0	0	0	1	0
EPHA3	0	1	1	0	1	1	0	1	1
EPHA5	0	1	1	0	1	0	0	1	0
EPHA8	0	1	0	0	1	0	0	1	0
EPHB1	0	1	1	0	1	1	0	1	1
EPHB3	0	1	0	0	1	1	1	0	1
EPHB4	0	1	0	0	1	1	0	1	1
EPHB6	1	0	1	1	0	1	1	0	1
EPM2A	1	0	1	1	0	1	1	0	1
EPM2AIP1	1	0	1	1	0	1	1	0	1
EPN2	0	1	0	0	1	1	0	1	1
EPN3	0	1	0	0	1	0	0	1	0
EPO	0	1	0	0	1	0	0	1	0
EPPB9	0	0	0	0	0	0	0	1	0
EPRS	0	0	0	0	0	1	0	0	1
EPS8	1	1	1	1	1	1	1	1	1
EPS8L1	0	1	0	0	1	1	0	1	1
EPSTI1	1	0	1	1	0	1	1	0	1
ERAL1	1	0	1	1	0	1	1	0	1
ERBB2	1	1	1	1	1	1	1	1	1

ERBB2IP	1	0	1	1	0	1	1	0	1
ERBB4	0	1	1	0	1	1	0	1	0
ERCC1	1	0	0	1	0	1	1	0	1
ERCC2	1	0	1	1	0	0	1	0	1
ERCC4	0	1	0	1	1	0	1	0	0
ERCC5	0	0	0	1	0	1	0	0	1
ERCC6	1	0	1	0	0	1	1	0	1
ERCC8	1	0	1	1	0	1	1	0	1
ERF	0	0	0	0	0	0	1	1	1
ERGIC2	1	1	1	1	0	1	1	1	1
ERGIC3	0	0	0	0	0	0	0	0	1
ERH	1	0	1	1	0	1	1	0	1
ERICH1	0	1	0	0	1	0	0	1	0
ERMAP	1	0	1	1	0	1	1	0	1
ERN1	1	0	0	0	0	0	0	0	0
ERO1L	0	0	0	1	0	0	0	0	0
ERO1LB	1	0	1	0	0	1	1	0	1
ERRFI1	0	1	0	0	1	0	0	1	0
ESCO1	0	0	0	0	0	0	0	0	1
ESCO2	1	0	1	1	0	1	1	0	1
ESD	1	0	0	1	0	1	0	0	1
ESPL1	1	0	1	1	0	1	1	0	1
ESR1	0	1	0	0	1	1	0	1	1
ESRRA	0	0	1	0	0	1	1	0	1
ESX1	0	1	1	0	1	1	0	1	1
ETF1	1	0	1	1	0	1	1	0	1
ETFA	1	0	0	1	0	1	1	0	1
ETFB	1	0	1	0	0	1	1	0	1
ETFDH	1	0	1	1	0	1	1	0	1
ETHE1	1	0	1	0	0	1	0	0	1
ETNK1	0	0	0	1	0	1	1	0	1
ETNK2	0	1	0	0	1	0	0	1	0
ETS1	1	0	1	1	0	1	1	0	1
ETV2	0	0	0	0	0	0	0	1	0
ETV3	0	0	0	0	0	0	0	0	1
ETV5	1	0	1	1	1	1	0	1	1
ETV6	1	0	1	1	0	1	1	0	1
ETV7	0	1	0	0	0	0	0	1	1
EVA1	0	1	0	1	0	1	1	1	1
EVC	0	1	0	0	1	0	0	1	1
EVI2A	1	0	1	1	0	0	1	0	1
EVI2B	1	0	1	1	0	1	1	0	1
EVI5	0	0	0	0	0	0	0	1	0
EVI5L	0	1	0	0	0	0	0	0	0
EVL	1	0	1	1	0	1	0	0	1
EWSR1	1	0	1	1	0	1	1	0	1
EXO1	0	0	1	0	0	1	1	0	1
EXOC1	0	0	0	0	1	0	0	0	0
EXOC3	0	0	0	0	0	1	0	0	1
EXOC5	1	0	1	1	0	1	1	0	1
EXOC6	1	0	1	1	0	1	1	0	1
EXOC7	1	0	0	0	0	1	0	0	0
EXOC8	1	0	1	1	0	1	1	0	1
EXOD1	1	0	1	0	0	1	1	0	1
EXOSC1	1	0	1	1	0	1	0	0	1
EXOSC10	0	0	0	0	0	0	0	0	1
EXOSC2	1	0	1	1	0	1	1	0	1
EXOSC3	1	0	1	1	0	0	1	0	1
EXOSC4	1	0	1	1	0	1	0	0	1
EXOSC5	1	0	1	1	0	1	1	0	1
EXOSC7	1	0	1	1	0	1	1	0	1
EXOSC8	1	0	1	1	0	1	1	0	1

EXOSC9	1	0	1	1	0	1	1	0	1
EXT1	1	1	1	1	1	1	1	1	1
EXTL1	0	0	0	0	0	0	0	1	0
EXTL2	1	0	1	1	0	1	1	0	1
EXTL3	0	0	1	0	0	0	1	0	1
EYA3	1	0	1	1	0	1	1	0	1
EZH1	0	1	0	0	0	0	0	1	0
EZH2	1	0	1	1	0	0	1	0	1
F11R	0	0	1	0	0	1	1	0	1
F13A1	0	1	0	0	1	0	0	0	0
F2RL1	0	1	1	0	1	1	0	1	0
F2RL3	0	0	0	0	0	0	0	1	0
F3	1	0	1	1	0	1	1	0	1
F8	0	0	0	0	0	1	0	0	0
FA2H	0	0	0	0	0	0	0	1	0
FABP2	0	1	0	0	0	0	0	0	0
FABP3	0	0	0	0	0	1	0	1	0
FABP4	1	0	0	0	0	0	0	0	0
FABP5	0	0	0	0	0	0	1	0	1
FABP7	0	1	0	0	1	0	0	1	0
FADD	0	0	0	1	0	1	0	0	1
FADS1	1	0	1	1	0	1	1	0	0
FADS2	1	0	1	1	0	1	0	0	0
FADS3	0	0	0	0	0	0	0	1	0
FAF1	1	0	1	1	0	1	0	0	1
FAH	1	0	1	1	0	1	1	0	1
FAHD1	1	0	1	1	0	1	0	0	1
FAHD2A	0	0	1	1	0	1	1	1	1
FAIM2	0	1	0	0	1	0	0	1	0
FAIM3	0	0	0	1	0	1	1	0	1
FAM100B	0	0	0	1	0	0	0	0	0
FAM101A	0	1	0	0	0	0	0	0	0
FAM102A	0	0	0	1	0	0	1	0	0
FAM102B	1	0	1	1	0	1	1	0	1
FAM103A1	1	0	1	1	0	1	1	0	1
FAM104A	1	0	1	1	0	1	1	0	1
FAM105A	0	0	0	0	0	0	1	0	1
FAM107A	0	1	0	0	0	0	0	0	0
FAM108A1	0	0	0	0	0	0	0	0	1
FAM109A	0	0	1	1	0	1	1	0	1
FAM109B	1	0	1	1	0	1	0	0	1
FAM111A	1	0	1	1	0	1	1	0	1
FAM111B	0	1	1	0	1	1	0	1	1
FAM112B	0	0	0	1	0	0	0	0	0
FAM113A	1	0	1	1	0	1	1	0	1
FAM113B	0	0	0	0	0	1	1	0	1
FAM13A1	1	0	1	1	0	1	1	0	1
FAM14A	0	1	0	0	0	0	0	1	0
FAM14B	1	0	1	1	0	1	1	0	1
FAM18B	1	0	1	1	0	1	1	0	1
FAM19A2	0	1	1	1	0	1	0	1	1
FAM19A3	1	0	1	0	0	1	0	1	1
FAM20A	1	0	1	1	0	0	1	1	1
FAM21C	1	0	1	1	0	1	1	0	1
FAM24B	0	1	1	0	1	1	0	1	1
FAM26B	0	0	0	0	0	0	0	0	1
FAM32A	0	0	0	1	0	0	1	0	1
FAM33A	1	0	1	1	0	1	1	0	1
FAM35A	1	0	1	1	0	1	1	0	1
FAM36A	1	0	1	1	0	1	1	0	1
FAM3A	0	1	0	0	1	1	0	1	1
FAM3C	1	0	0	0	0	0	0	0	0

FAM40A	0	0	0	0	0	1	1	0	1
FAM40B	0	1	0	0	1	1	0	1	1
FAM44A	1	0	1	1	0	1	0	0	1
FAM44B	1	0	0	1	0	1	0	0	0
FAM46A	0	0	0	0	0	1	1	0	1
FAM46C	0	1	1	0	1	1	0	1	1
FAM48A	0	0	1	1	0	1	0	0	1
FAM49A	0	0	0	0	0	1	1	0	1
FAM49B	0	0	0	0	0	1	0	0	0
FAM50A	0	1	0	0	0	0	0	0	0
FAM53A	1	0	1	1	0	1	0	0	1
FAM53B	1	0	1	0	0	0	1	0	1
FAM53C	0	0	1	1	0	1	1	0	1
FAM54A	1	0	1	1	0	1	1	0	1
FAM54B	1	0	0	1	0	1	0	0	1
FAM57A	0	1	0	0	1	0	0	1	0
FAM58A	0	1	1	0	1	1	0	1	1
FAM62A	1	0	1	1	0	1	1	0	1
FAM62B	1	0	1	1	0	1	1	0	1
FAM63A	1	0	1	1	0	1	1	0	1
FAM63B	1	0	1	1	0	1	0	0	1
FAM64A	0	1	0	0	0	1	1	0	1
FAM65A	0	0	0	1	0	0	0	1	0
FAM69A	1	0	1	1	0	1	0	1	0
FAM69B	0	1	0	0	0	0	0	1	0
FAM70A	0	1	0	0	1	0	0	1	0
FAM70B	1	0	1	1	0	1	0	0	1
FAM71C	1	0	0	0	0	0	0	0	0
FAM72A	0	0	1	1	0	1	0	0	1
FAM73A	0	0	0	1	0	0	0	0	1
FAM73B	1	0	0	1	0	1	0	0	0
FAM76A	0	0	0	0	0	0	0	0	1
FAM76B	1	0	1	1	0	1	1	0	1
FAM78A	1	0	1	1	0	1	1	0	1
FAM79A	1	0	1	0	0	1	0	0	1
FAM80A	0	1	0	0	1	0	0	1	0
FAM81A	0	1	1	0	1	1	0	1	1
FAM82B	1	0	1	0	0	0	0	0	1
FAM82C	1	0	0	1	0	1	0	0	1
FAM83F	0	1	0	0	1	0	0	1	0
FAM84A	0	1	1	0	1	1	0	1	0
FAM86A	1	1	1	1	1	1	1	1	1
FAM86B1	1	1	1	1	1	1	1	1	1
FAM86C	1	1	1	1	1	1	1	1	1
FAM89A	0	1	0	0	1	0	0	1	0
FAM89B	1	0	1	1	0	1	0	0	1
FAM8A1	0	0	0	0	0	1	0	0	1
FAM91A1	0	0	0	0	0	0	1	0	0
FAM96A	0	0	1	1	0	1	1	0	1
FAM96B	1	0	1	1	0	1	1	1	1
FAM98A	1	0	1	1	0	1	1	0	1
FAM9C	0	1	0	0	1	0	0	1	0
FANCB	1	1	1	1	0	1	1	0	1
FANCE	0	0	0	0	0	0	0	1	0
FANCF	1	0	1	1	0	1	1	0	1
FANCG	0	0	0	0	0	0	0	0	1
FANCL	0	0	1	0	0	1	1	0	1
FARS2	1	0	1	1	0	1	1	0	1
FARSLB	1	0	1	1	0	1	1	0	1
FAS	1	0	1	1	0	1	1	0	1
FASTK	0	0	0	0	0	1	0	1	1
FAU	1	0	1	1	0	1	1	0	1

FBL	1	0	1	1	0	1	1	0	1
FBN2	0	1	0	0	0	1	0	1	1
FBP1	0	0	0	1	0	0	0	0	0
FBS1	1	0	0	0	0	0	0	0	0
FBXL10	1	0	1	1	0	1	1	0	1
FBXL11	1	0	1	1	0	1	1	0	1
FBXL13	1	0	1	1	0	1	1	0	1
FBXL14	1	0	0	0	0	0	0	0	1
FBXL15	0	1	1	1	1	1	1	1	1
FBXL16	0	1	0	0	1	0	0	1	0
FBXL17	1	0	1	0	0	1	1	0	1
FBXL19	0	0	0	0	0	0	0	0	1
FBXL3	1	0	1	1	0	1	1	0	1
FBXL6	1	0	1	1	0	1	0	0	1
FBXL8	0	1	0	0	1	0	0	1	1
FBXO11	1	0	1	1	0	1	1	0	1
FBXO15	1	0	1	1	0	1	1	0	1
FBXO16	1	1	1	0	1	1	1	1	1
FBXO21	0	1	0	0	1	0	0	1	0
FBXO27	0	1	0	0	1	0	0	1	0
FBXO28	1	0	1	1	0	1	1	0	1
FBXO3	0	1	0	0	1	0	0	1	0
FBXO30	1	0	1	1	0	1	1	0	1
FBXO31	1	0	0	0	0	0	0	0	0
FBXO32	0	1	0	0	1	1	0	1	1
FBXO33	1	0	1	1	0	1	1	0	1
FBXO38	1	0	1	1	0	1	1	0	1
FBXO4	1	0	1	0	0	0	1	0	1
FBXO42	1	0	0	1	0	1	1	0	1
FBXO43	1	0	1	1	0	0	1	0	1
FBXO44	0	1	0	0	1	0	0	1	0
FBXO5	1	0	1	1	0	1	1	0	1
FBXO7	1	0	0	0	0	0	0	0	1
FBXO8	1	0	1	1	0	1	1	0	1
FBXO9	1	0	1	1	0	1	1	1	1
FBXW11	1	0	1	1	0	1	1	0	1
FBXW2	1	0	1	1	0	1	1	0	1
FBXW4	0	0	0	1	0	0	0	0	1
FBXW7	1	0	1	1	0	1	1	0	1
FBXW8	0	1	0	0	1	0	0	1	0
FBXW9	0	0	0	0	0	0	0	0	1
FCER1G	0	0	1	1	0	0	1	0	1
FCGBP	0	1	0	0	1	0	0	1	0
FCGR2A	0	0	1	0	0	1	0	0	1
FCGR2B	0	0	1	1	0	1	1	1	1
FCGRT	1	0	1	0	0	1	1	0	1
FCHO1	0	0	1	0	0	0	0	0	0
FCHO2	1	0	1	1	0	1	1	0	1
FCHSD2	0	0	0	0	0	0	1	0	1
FCN1	0	1	0	0	0	1	1	0	1
FCRL3	0	1	0	0	1	0	0	1	0
FCRL5	0	1	0	0	0	0	0	1	0
FDFT1	1	0	1	0	0	1	0	0	1
FDPS	1	0	0	1	0	1	1	0	1
FDX1	0	0	0	0	0	0	0	0	1
FDXR	1	0	1	1	0	1	1	0	1
FEM1B	1	0	1	1	0	1	1	0	1
FEN1	1	0	1	1	0	1	1	0	1
FER	1	0	1	1	0	1	1	0	1
FER1L3	1	0	1	1	0	1	1	0	1
FERD3L	0	1	0	0	1	0	0	1	0
FES	0	0	0	1	0	1	0	0	0

FFAR1	0	0	0	0	1	0	0	0	0
FGA	0	1	0	0	0	0	0	0	0
FGD2	0	0	0	0	0	1	0	0	1
FGD3	1	0	0	1	0	0	1	0	1
FGD4	1	0	0	0	0	0	1	0	0
FGD5	0	1	0	0	0	0	0	0	0
FGD6	1	0	1	1	0	1	1	0	1
FGF11	1	1	1	0	1	1	0	1	1
FGF12	0	1	1	0	1	0	0	1	0
FGF13	0	1	0	1	1	0	1	1	0
FGF14	0	1	1	0	1	0	0	1	0
FGF17	0	1	0	0	1	0	0	1	0
FGF18	0	1	0	0	0	0	0	1	0
FGF19	0	1	0	0	1	0	0	1	0
FGF20	0	1	1	0	1	0	0	1	1
FGFR1OP	0	0	0	1	0	0	0	0	1
FGFR1OP2	1	0	1	1	0	1	1	0	1
FGL2	1	0	1	1	0	1	1	0	1
FGR	0	0	1	1	0	1	0	0	0
FH	1	0	1	1	0	1	1	0	1
FHIT	1	0	1	1	0	1	1	0	1
FHL1	0	1	1	0	1	1	0	1	1
FHL2	0	1	0	0	1	0	0	1	0
FHL5	0	1	0	0	0	0	0	1	0
FHOD1	0	0	0	0	0	0	0	1	1
FIBCD1	0	1	0	0	1	0	0	1	0
FIBP	1	0	1	1	0	1	1	0	1
FIGLA	0	0	0	0	0	0	0	1	0
FIGN	0	1	0	0	1	0	0	1	0
FIGNL1	0	0	1	0	0	1	1	0	1
FIP1L1	0	0	0	1	0	1	0	0	1
FJX1	0	1	1	0	0	1	0	1	1
FKBP11	0	0	0	0	0	0	1	0	1
FKBP14	1	0	1	1	0	1	1	0	1
FKBP1A	0	0	1	1	0	1	0	0	1
FKBP2	0	0	0	0	0	0	0	0	1
FKBP3	1	0	1	1	0	1	1	0	1
FKBP4	0	1	0	0	1	1	0	1	0
FKBP6	1	1	0	0	1	0	0	1	0
FKBP8	0	0	0	0	0	1	1	0	1
FKBP9	0	1	1	0	0	0	0	1	0
FKBPL	0	0	1	1	0	1	0	0	1
FKRP	1	0	1	1	0	1	0	0	1
FKSG83	0	1	0	0	0	0	0	1	0
FLAD1	1	0	1	1	0	1	1	0	1
FLI1	0	0	1	0	0	1	1	1	1
FLII	1	0	1	0	0	1	1	0	1
FLJ10154	0	0	0	0	0	1	0	0	1
FLJ10241	1	0	1	1	0	1	0	0	1
FLJ10292	1	0	1	1	0	1	1	0	1
FLJ10324	0	1	0	0	1	0	0	1	0
FLJ10357	0	0	0	0	1	0	0	1	0
FLJ10769	0	0	0	0	0	1	0	0	0
FLJ10781	0	1	1	0	1	0	0	1	0
FLJ10803	1	0	1	1	0	1	1	0	1
FLJ10986	1	0	1	1	0	1	1	0	1
FLJ11184	1	0	1	1	0	1	1	0	1
FLJ11235	0	1	0	0	0	0	0	1	0
FLJ11506	1	1	1	1	1	1	1	1	1
FLJ11783	0	1	1	1	0	1	1	0	1
FLJ12716	1	0	1	1	0	1	1	0	1
FLJ13611	1	0	1	1	0	1	1	0	1

FLJ14154	0	0	0	1	0	1	0	0	0
FLJ14213	0	1	0	0	1	0	0	1	1
FLJ14803	1	0	1	1	0	1	1	0	1
FLJ16124	1	0	0	1	0	1	0	1	1
FLJ20035	1	0	0	0	0	0	0	0	1
FLJ20186	0	1	0	0	1	0	0	1	0
FLJ20294	1	0	1	1	0	1	1	0	1
FLJ20309	1	0	1	1	0	1	1	0	1
FLJ20489	0	0	0	0	0	1	1	0	1
FLJ20628	1	0	1	1	0	1	1	0	1
FLJ20699	1	1	0	0	0	1	0	1	1
FLJ20850	1	0	0	0	0	0	1	0	1
FLJ21839	1	0	1	1	0	1	1	1	1
FLJ21963	0	1	0	0	1	0	0	1	0
FLJ21986	1	0	1	1	0	1	1	0	1
FLJ22222	1	0	1	1	0	1	1	0	1
FLJ22639	1	0	1	1	1	1	1	1	1
FLJ22662	1	0	1	1	0	1	1	0	1
FLJ23356	0	0	1	1	0	1	1	1	1
FLJ25715	0	0	1	1	0	1	0	0	1
FLJ30679	1	0	1	1	0	1	1	1	1
FLJ30934	0	1	0	0	0	0	0	1	0
FLJ31438	1	0	1	1	0	1	1	0	1
FLJ31818	1	0	0	1	0	1	0	0	1
FLJ32549	0	0	0	0	0	0	1	0	1
FLJ33590	0	1	0	0	1	0	0	1	0
FLJ33790	0	1	0	0	1	1	0	1	1
FLJ34047	0	0	0	1	0	1	1	0	1
FLJ34870	0	1	0	0	1	0	0	1	0
FLJ34931	0	0	0	1	0	0	0	0	0
FLJ35767	0	1	0	0	1	0	0	0	0
FLJ35801	1	1	1	1	1	1	1	1	1
FLJ36492	0	0	0	0	0	0	0	1	0
FLJ36874	0	0	0	1	0	0	1	0	0
FLJ37078	0	1	0	0	1	0	0	1	0
FLJ38377	0	1	0	0	1	0	0	1	0
FLJ38482	1	0	1	1	0	1	1	0	1
FLJ38717	1	0	1	1	0	1	1	0	0
FLJ39378	0	0	0	0	0	0	0	1	1
FLJ39653	1	0	1	1	0	1	1	0	1
FLJ39779	0	0	0	0	0	0	0	1	1
FLJ40125	1	1	1	1	0	0	0	1	0
FLJ40852	1	0	1	1	0	1	1	0	1
FLJ41327	0	0	0	0	1	0	0	1	0
FLJ41423	0	1	0	0	1	0	0	1	0
FLJ42133	0	1	0	0	1	0	0	0	0
FLJ42875	0	1	0	0	0	0	0	1	0
FLJ42957	0	0	0	0	0	0	1	0	1
FLJ43093	0	0	0	0	0	0	1	0	0
FLJ43276	0	1	0	0	1	1	0	1	0
FLJ43752	0	1	0	0	1	0	0	1	0
FLJ43870	0	1	0	0	1	0	0	1	0
FLJ44186	0	1	0	0	0	0	0	0	0
FLJ44635	0	1	0	0	1	0	0	1	0
FLJ45055	1	0	0	1	0	1	1	1	1
FLJ45537	0	1	0	0	1	0	0	1	0
FLJ45983	0	1	1	0	1	1	0	1	1
FLJ46082	1	1	0	0	1	0	0	1	1
FLJ46154	1	1	1	1	1	1	1	1	1
FLJ46347	0	1	0	0	1	0	0	1	0
FLJ46380	0	0	0	0	0	0	0	1	0
FLJ46836	0	1	0	0	0	0	0	1	0

FLJ90709	1	0	1	1	0	1	1	0	1
FLJ90757	1	0	1	1	0	1	1	0	1
FLOT1	1	0	1	1	0	1	1	0	1
FLOT2	1	0	1	0	0	0	0	0	1
FLRT2	0	1	1	1	1	1	0	1	1
FLT1	0	1	0	0	0	0	0	1	1
FLT3	0	1	0	0	0	0	0	0	0
FLT3LG	0	0	0	0	0	1	0	0	0
FLYWCH1	0	0	0	0	0	0	0	0	1
FMNL1	1	0	0	1	0	1	0	0	1
FMNL2	0	1	0	1	1	1	0	1	1
FMNL3	1	0	0	0	0	0	0	0	0
FMO1	0	1	0	0	1	0	0	1	0
FMO2	0	0	0	0	0	0	0	1	0
FMO4	1	0	1	1	0	1	1	0	1
FMO5	1	0	0	1	0	1	1	0	1
FN3KRP	0	0	1	1	0	1	1	0	1
FNBP1	1	0	1	1	0	1	1	0	1
FNBP4	1	0	1	1	0	1	1	0	1
FNDC3A	1	0	1	1	0	1	1	0	1
FNDC8	0	1	0	0	1	0	0	0	0
FNTA	0	0	0	1	0	1	0	0	0
FNTB	1	1	1	1	0	1	1	0	1
FOLR3	0	0	0	0	0	0	0	0	1
FOS	1	0	1	1	0	1	1	0	1
FOSB	1	1	1	1	0	1	0	1	1
FOSL2	1	0	1	1	0	1	1	0	1
FOXA1	0	1	1	0	1	1	0	1	1
FOXC2	0	1	0	0	1	0	0	1	1
FOXD2	1	1	1	0	1	1	0	1	1
FOXD4L1	0	1	0	0	1	0	0	1	0
FOXE1	0	1	1	0	1	1	0	1	0
FOXI1	0	1	0	0	0	0	0	0	0
FOXJ2	1	0	1	0	0	0	1	0	1
FOXJ3	1	0	1	1	0	1	1	0	1
FOXM1	1	0	1	1	0	1	1	0	1
FOXP1	1	0	1	1	0	1	1	0	1
FOXP3	0	1	1	0	1	1	0	0	1
FOXP4	0	1	0	0	1	1	0	1	1
FOXQ1	0	1	1	0	1	1	0	1	1
FOXRED1	1	0	1	1	0	1	1	0	1
FPGS	1	0	1	1	0	1	0	0	1
FPGT	1	0	1	1	0	1	1	0	1
FPR1	0	0	0	1	0	1	1	0	1
FPRL1	0	0	0	0	0	0	1	0	1
FPRL2	1	0	1	0	0	0	0	0	0
FRAG1	0	0	1	1	0	1	0	0	0
FRAP1	1	0	1	1	0	1	1	0	1
FRAT2	1	0	0	0	0	0	1	0	0
FREQ	0	1	0	0	1	0	0	1	0
FRG1	0	0	1	0	0	0	0	0	1
FRMD3	0	1	0	0	1	0	0	1	0
FRMD4A	0	1	0	0	1	0	0	1	0
FRMD6	0	0	0	0	0	1	0	0	1
FRRS1	1	0	1	1	0	1	1	0	1
FRS2	0	0	0	0	0	0	0	0	1
FRS3	0	0	0	0	0	1	0	0	1
FRY	1	0	1	1	0	1	1	0	1
FRZB	0	1	0	0	1	0	0	1	0
FSCN1	0	0	0	0	0	0	0	1	0
FSD1L	0	0	0	0	0	0	1	0	0
FST	1	1	1	0	1	1	1	1	1

FSTL1	0	1	1	0	1	0	0	1	0
FSTL3	0	1	0	0	1	0	0	1	1
FTH1	1	0	1	1	0	1	1	0	1
FTL	1	1	1	1	1	1	1	0	1
FTSJ1	1	1	1	1	0	1	1	0	1
FTSJ2	1	0	0	0	0	0	0	0	1
FTSJ3	1	0	1	1	0	1	1	0	1
FUBP1	1	0	1	1	0	1	1	0	1
FUCA1	0	0	0	0	0	0	0	0	1
FUCA2	1	0	1	0	0	1	1	0	1
FUK	1	0	1	1	0	1	1	0	1
FUNDC1	1	1	1	1	0	1	1	1	1
FURIN	1	0	1	0	0	1	0	0	0
FUSIP1	0	0	1	1	0	1	0	0	1
FUT10	1	0	1	1	0	1	1	0	1
FUT11	0	0	0	0	0	0	0	0	1
FUT4	1	0	0	0	0	0	1	0	1
FVT1	1	0	1	1	0	1	1	0	1
FXC1	1	0	1	1	0	1	1	0	1
FXR1	1	0	0	1	0	1	1	0	1
FXR2	0	0	1	0	0	1	1	0	1
FXYD3	0	0	0	1	1	0	0	0	0
FXYD6	0	1	1	0	1	0	0	1	0
FYB	1	0	1	1	0	1	1	0	1
FYCO1	0	0	1	0	0	1	1	0	1
FYN	1	0	1	1	0	1	1	0	1
FYTTD1	1	0	1	1	0	1	1	1	1
FZD1	1	1	1	1	1	1	1	1	1
FZD2	1	0	1	1	0	0	1	0	1
FZD3	1	1	1	0	1	1	1	1	1
FZD5	1	0	1	0	0	1	0	1	1
FZD7	1	1	1	1	0	1	0	1	0
G0S2	0	0	0	0	0	0	0	0	1
G3BP2	1	0	1	1	0	1	1	0	1
G6PC3	1	0	0	1	0	1	1	0	1
G6PD	1	1	1	1	0	1	0	0	1
GAA	1	0	1	1	0	1	1	0	1
GAB2	1	0	1	1	0	1	1	0	1
GAB3	0	1	0	0	1	1	1	1	1
GABARAP	1	0	1	1	0	1	1	0	1
GABARAPL1	0	0	0	0	0	0	0	0	1
GABARAPL2	1	0	1	1	0	1	1	1	1
GABPA	1	0	1	1	0	1	1	0	1
GABPB2	1	0	1	1	0	1	1	0	1
GADD45A	0	0	0	0	0	1	1	0	1
GADD45B	1	0	1	1	0	1	1	0	1
GADD45G	0	1	1	0	0	1	0	1	1
GAL	0	1	0	0	1	0	0	1	0
GAL3ST4	0	1	1	0	1	1	1	1	1
GALE	1	0	1	1	0	1	1	0	1
GALK2	1	0	1	1	0	1	1	0	1
GALM	0	0	0	0	0	1	1	0	1
GALNACT-2	1	0	1	1	0	1	1	0	1
GALNS	0	0	0	1	0	1	0	0	1
GALNT11	0	1	0	0	0	0	0	0	0
GALNT12	0	0	0	0	0	1	0	1	0
GALNT13	0	0	0	0	0	0	0	0	0
GALNT2	1	0	1	1	0	1	1	0	1
GALNT4	1	0	1	1	0	1	1	0	1
GALNT7	1	0	1	1	0	1	1	0	1
GALNTL1	0	1	1	0	1	1	0	1	0
GALNTL4	0	1	0	0	1	0	0	1	0

GALR2	0	1	0	0	0	0	0	1	0
GALT	0	0	0	1	0	1	0	0	0
GAMT	0	0	0	0	0	0	0	1	0
GAN	1	0	1	1	0	1	1	0	1
GANAB	0	0	0	0	0	0	0	0	1
GANC	1	0	1	1	0	1	1	0	1
GAPDH	0	0	1	0	0	1	0	0	0
GARNL1	0	0	0	0	0	0	0	0	1
GARS	1	0	1	1	0	1	1	0	1
GART	1	0	1	1	0	1	1	0	1
GAS2L1	0	0	0	0	0	0	0	1	1
GAS2L3	0	0	0	0	0	0	0	0	1
GAS6	1	1	1	1	1	1	0	1	1
GAS7	0	0	1	1	0	1	0	0	1
GAS8	0	1	1	0	0	0	0	1	1
GATAD1	1	0	0	1	0	0	0	0	0
GATAD2A	0	0	0	1	0	0	0	0	0
GATS	0	0	0	1	1	1	0	0	1
GBA	1	0	1	1	0	1	0	0	1
GBE1	0	0	0	0	0	0	1	0	0
GBF1	1	1	1	1	1	1	1	1	1
GBGT1	0	0	0	0	0	0	0	1	0
GBP1	1	0	1	1	0	1	1	0	1
GBP2	1	0	1	1	0	1	1	0	1
GBP3	1	0	1	1	0	1	1	0	1
GBP4	1	0	1	1	0	1	1	0	1
GBP5	1	0	0	1	0	0	1	0	1
GBX2	0	1	0	0	1	0	0	1	0
GCA	1	0	1	1	0	1	1	0	1
GCAT	1	1	1	0	1	1	1	1	1
GCC1	0	0	1	1	0	1	1	0	1
GCC2	1	0	1	1	0	1	1	0	1
GCDH	0	0	0	0	0	0	0	0	1
GCET2	1	0	0	0	0	0	1	0	0
GCH1	0	0	0	0	0	0	0	0	1
GCHFR	0	0	1	0	0	1	0	0	1
GCLC	0	0	1	0	0	1	0	1	1
GCLM	1	0	0	0	0	0	1	0	1
GCN1L1	1	0	0	0	0	1	0	0	1
GCNT1	0	0	0	0	0	1	0	0	0
GCNT2	1	0	1	1	0	1	1	0	1
GCNT3	0	1	0	0	0	0	0	1	0
GCS1	1	0	1	1	0	1	1	0	1
GDAP1	0	1	0	0	1	0	0	1	0
GDAP2	1	0	1	1	0	1	1	0	1
GDF1	0	1	0	0	1	0	0	1	0
GDF15	0	0	0	0	0	0	0	1	0
GDF5	1	1	0	1	1	1	1	1	1
GDF9	1	0	0	1	0	1	0	0	1
GDI1	1	1	0	0	1	1	0	0	1
GDI2	1	0	1	1	0	1	1	0	1
GDPD1	1	0	0	0	0	1	1	0	1
GDPD2	0	1	0	0	1	0	0	0	0
GDPD5	0	0	1	1	0	1	1	0	1
GEM	0	1	1	0	1	1	1	1	1
GEMIN6	1	1	1	1	0	1	1	1	1
GFI1	0	0	0	0	1	0	0	0	1
GFM1	0	0	1	1	0	1	1	0	1
GFM2	1	0	1	1	0	1	1	0	1
GFOD1	0	0	0	0	0	1	0	0	1
GFOD2	1	0	1	1	0	1	1	0	1
GFPT1	0	0	1	0	0	1	1	0	1

GFRA3	0	1	1	0	1	1	0	1	0
GGA1	1	0	1	1	0	1	1	0	0
GGA3	1	0	1	1	0	1	1	0	1
GGCX	0	0	0	0	0	1	0	0	1
GGH	1	0	1	0	0	1	1	0	1
GGPS1	1	0	1	1	0	1	1	0	1
GGTL3	1	0	1	1	0	1	1	0	1
GHITM	1	1	1	1	0	1	1	1	1
GHSR	0	1	1	0	1	1	0	1	0
GIF	0	0	0	0	0	0	0	0	0
GIMAP1	1	0	1	1	0	1	1	0	1
GIMAP2	1	0	1	1	0	1	1	0	1
GIMAP4	1	0	1	1	0	1	1	0	1
GIMAP5	1	0	1	1	0	1	1	0	1
GIMAP6	0	0	0	0	0	1	1	0	1
GIMAP7	1	0	1	1	0	1	1	0	1
GIMAP8	0	0	0	1	0	0	0	0	1
GIOT-1	0	0	1	0	0	1	0	0	0
GIP	0	1	0	0	1	0	0	1	0
GIPC1	0	0	0	0	0	1	0	1	0
GIPC3	0	1	0	0	1	0	0	1	0
GIPR	0	1	1	0	1	1	0	1	1
GIT1	1	0	1	0	0	1	1	1	1
GIT2	1	0	0	1	0	0	1	0	1
GIYD2	1	0	1	1	0	1	1	0	1
GJA4	0	1	0	0	1	0	0	1	0
GJA5	0	1	0	0	1	0	0	0	0
GJA7	0	1	0	0	1	1	0	1	1
GJB2	0	1	0	0	1	0	0	1	1
GJB6	0	1	1	0	1	1	0	1	1
GK	1	1	1	1	0	1	0	1	1
GKAP1	0	1	1	0	0	1	1	1	1
GLA	1	1	1	1	1	1	1	0	1
GLB1	1	0	1	1	0	1	1	0	1
GLB1L	1	0	1	1	0	1	0	0	1
GLCCI1	1	0	1	1	0	1	1	0	1
GLCE	0	0	1	0	0	1	1	0	1
GLG1	1	1	1	1	0	1	0	1	1
GLI3	0	1	1	0	1	1	0	1	1
GLI4	0	1	0	0	1	0	0	1	0
GLIPR1	1	0	1	1	0	1	1	0	1
GLIS1	0	1	0	0	1	0	0	1	0
GLO1	1	0	0	0	0	0	0	0	1
GLRX	1	0	1	1	0	1	1	0	1
GLRX2	1	0	1	1	0	1	1	0	1
GLRX5	1	0	1	1	0	1	0	0	1
GLS	0	0	0	0	0	1	0	0	1
GLT1D1	0	1	0	0	1	0	0	0	1
GLT25D1	0	0	0	0	0	1	1	0	0
GLT8D1	1	0	1	1	0	1	1	0	1
GLTP	0	0	1	1	0	1	1	0	1
GLUD1	1	0	1	0	0	0	0	0	1
GLUD2	0	1	0	0	1	0	0	1	0
GLUL	1	0	1	1	0	1	1	0	1
GLYCTK	0	0	0	1	0	0	0	0	1
GM2A	1	0	0	0	0	1	1	0	1
GMCL1	0	0	1	1	0	1	1	0	1
GMDS	1	0	1	1	0	1	1	0	1
GMEB1	1	0	1	1	0	1	0	0	0
GMFG	1	0	1	1	0	1	1	0	1
GMIP	0	0	0	1	0	0	0	0	1
GMNN	0	0	0	0	0	1	0	0	1

GMPPA	1	1	0	0	1	0	0	1	1
GMPPB	1	0	0	0	0	1	0	0	1
GMPR	1	0	0	1	0	1	1	0	1
GMPR2	1	0	1	1	0	1	1	0	1
GNA12	0	0	0	0	0	1	0	0	1
GNAI2	1	0	1	1	0	1	1	0	1
GNAI3	1	0	1	1	0	1	1	0	1
GNAQ	1	0	1	0	0	1	1	0	1
GNAS	0	1	1	0	1	1	0	1	1
GNB1	0	0	0	1	0	1	0	0	1
GNB1L	1	0	0	1	0	1	0	0	1
GNB2L1	1	0	1	1	0	1	0	0	1
GNB4	1	0	1	1	0	1	1	0	1
GNB5	0	1	0	0	1	0	0	1	0
GNE	1	0	1	1	0	0	0	0	0
GNG11	0	1	0	0	1	0	1	1	0
GNG13	0	1	0	0	1	0	0	1	0
GNG5	1	0	1	1	0	1	1	0	1
GNGT2	0	0	0	0	0	1	1	0	1
GNL1	1	0	1	1	0	1	1	0	1
GNL2	1	0	1	1	0	1	1	0	1
GNL3	1	0	1	1	0	1	1	0	1
GNL3L	0	1	1	0	1	1	0	0	1
GNPAT	1	0	1	1	0	1	1	0	1
GNPDA1	0	0	0	0	0	1	0	0	1
GNPDA2	1	1	1	1	0	1	1	0	1
GNPTAB	1	0	0	1	0	1	0	0	1
GNPTG	1	0	1	1	0	1	1	0	1
GNS	1	0	1	1	0	1	1	0	1
GOLGA1	1	0	1	1	0	1	1	0	1
GOLGA2	1	0	1	1	0	1	1	0	1
GOLGA3	1	0	1	0	0	1	0	0	1
GOLGA4	1	0	1	1	0	1	1	0	1
GOLGA5	1	0	1	1	0	1	1	0	1
GOLGA7	0	0	0	0	0	1	0	0	1
GOLGB1	1	0	1	1	0	1	1	0	1
GOLPH2	1	1	1	0	1	1	1	1	1
GOLPH3	1	0	1	1	0	1	1	0	1
GOLPH3L	1	0	1	1	0	1	1	0	1
GOLPH4	0	0	0	0	0	1	0	0	1
GOLT1B	1	0	1	1	0	1	1	0	1
GON4L	1	0	0	0	0	0	0	0	0
GOPC	1	0	1	1	0	1	0	0	1
GORASP1	1	0	1	1	0	1	1	0	1
GORASP2	1	0	1	1	0	1	1	0	1
GOSR1	1	0	0	1	0	1	1	0	1
GOSR2	1	0	1	1	0	1	1	0	1
GOT1	0	0	1	0	0	1	0	0	1
GOT2	0	0	0	0	0	1	1	0	0
GPAM	1	0	0	1	0	1	1	1	1
GPBAR1	0	0	0	0	0	0	1	0	0
GPBP1	1	0	1	1	0	1	1	0	1
GPC3	0	1	0	0	1	0	0	1	0
GPC4	0	1	1	0	1	0	0	1	0
GPC5	0	1	0	0	1	0	0	1	0
GPC6	0	1	0	0	1	0	0	1	0
GPD1	1	0	0	0	0	0	0	0	0
GPHN	0	0	0	0	0	1	1	0	1
GPKOW	1	1	1	1	1	1	0	0	1
GPNMB	1	0	1	1	0	1	0	0	0
GPR107	1	0	1	1	0	1	1	0	1
GPR108	0	0	0	0	0	0	0	0	1

GPR109A	1	1	0	1	0	1	1	0	0
GPR109B	1	0	0	1	0	0	1	0	0
GPR120	0	0	0	0	0	0	0	0	1
GPR125	0	1	1	0	1	1	0	1	1
GPR126	0	1	0	0	1	0	0	1	1
GPR132	1	0	1	1	0	1	1	0	1
GPR133	1	0	0	0	0	0	1	0	1
GPR135	0	1	0	0	1	0	0	1	0
GPR137B	0	1	0	0	1	1	0	1	1
GPR142	0	1	0	0	1	0	0	0	0
GPR143	0	1	0	0	1	0	0	1	0
GPR146	0	1	0	0	1	0	0	1	0
GPR149	0	1	1	0	1	0	0	1	1
GPR155	0	0	0	0	0	1	0	0	1
GPR158	0	1	1	0	1	0	0	1	1
GPR160	0	0	1	0	0	1	0	0	1
GPR161	0	1	0	0	0	0	0	1	0
GPR162	0	0	0	0	0	1	0	0	0
GPR172A	1	0	1	1	0	1	0	0	1
GPR176	0	0	1	0	0	1	0	1	1
GPR177	0	1	1	1	1	1	1	1	1
GPR23	0	1	0	1	1	0	1	0	0
GPR26	0	1	0	0	1	0	0	1	0
GPR27	0	1	0	0	1	0	0	1	1
GPR3	0	1	0	0	0	0	0	1	0
GPR34	1	1	0	1	0	1	1	0	0
GPR44	0	1	0	0	0	0	0	1	0
GPR55	0	0	0	0	0	0	1	0	0
GPR64	0	1	0	0	0	0	0	0	0
GPR65	1	0	1	1	0	1	1	0	1
GPR68	0	1	0	0	0	0	0	1	0
GPR82	0	1	0	0	1	0	0	1	0
GPR84	0	0	1	1	0	1	1	0	1
GPRASP1	0	1	0	0	0	0	0	0	0
GPRASP2	0	1	1	0	1	0	0	1	0
GPRC5A	0	1	0	0	1	0	0	1	0
GPRC5B	0	1	1	0	1	1	0	1	1
GPRC5C	0	1	1	0	1	0	0	1	1
GPS1	1	0	0	1	0	1	1	0	1
GPS2	1	0	1	1	0	1	0	0	1
GPSM3	1	0	1	1	0	1	1	0	1
GPSN2	0	0	0	0	0	1	0	0	1
GPT2	0	0	1	0	0	1	0	0	1
GPX1	1	0	0	0	0	0	0	0	0
GPX7	0	1	0	0	1	0	0	1	1
GRAMD1A	0	0	0	0	0	0	1	0	1
GRAMD2	0	0	0	0	0	0	0	1	0
GRAP	0	0	0	0	0	0	1	0	0
GRASP	0	1	0	0	0	0	0	1	1
GRB2	1	0	1	1	0	1	1	0	1
GREM1	0	1	1	0	1	1	1	1	1
GRHPR	1	0	0	1	0	0	1	0	1
GRIA2	0	1	1	0	1	1	0	1	0
GRIA3	0	1	0	0	1	0	0	1	0
GRIK1	0	1	0	0	1	0	0	1	0
GRIK5	0	1	0	0	1	0	0	1	0
GRIN1	0	1	0	0	1	0	0	1	0
GRIN2D	0	1	0	0	1	0	0	1	0
GRIN3A	0	1	0	0	1	0	0	1	0
GRINA	0	0	1	1	0	1	0	0	1
GRIPAP1	1	0	1	1	0	1	0	0	1
GRK4	1	0	1	1	0	1	1	0	1

GRK5	1	0	0	0	0	0	0	0	0
GRM1	0	1	1	0	1	0	0	1	0
GRM2	0	1	0	0	1	0	0	1	0
GRN	1	0	0	1	0	1	1	0	1
GRP	0	1	0	0	1	0	0	1	0
GRPEL1	0	1	0	0	0	0	0	1	1
GRPEL2	1	0	1	1	0	1	1	0	1
GRPR	0	1	0	0	1	0	0	1	0
GRWD1	1	1	1	0	0	1	0	1	1
GSC	0	1	1	0	1	1	0	1	1
GSDMDC1	0	0	1	0	0	1	0	0	0
GSG1	0	0	0	0	0	0	1	0	1
GSG2	1	0	1	1	0	1	1	0	1
GSK3B	1	0	0	1	0	1	1	0	1
GSN	0	0	1	1	0	1	0	0	1
GSPT1	1	0	1	0	0	1	0	0	1
GSPT2	0	1	0	0	1	0	0	1	0
GSS	1	0	1	1	0	1	1	0	1
GSTA4	1	0	1	0	0	1	1	0	1
GSTCD	1	0	1	1	0	1	1	0	1
GSTK1	0	0	0	0	0	1	0	0	1
GSTM2	0	0	1	1	0	1	1	0	1
GSTM3	0	1	0	0	1	1	0	1	0
GSTM4	0	0	1	1	0	1	0	0	1
GSTO1	0	0	0	1	0	0	1	0	1
GSTO2	1	0	1	0	0	0	1	0	1
GSTT1	1	0	1	1	0	0	0	0	1
GSTT2	0	1	0	0	1	0	0	1	1
GTDC1	0	0	0	1	0	1	0	0	0
GTF2A1	1	0	0	1	0	1	1	0	0
GTF2B	1	0	1	1	0	1	1	0	1
GTF2E1	1	0	1	1	0	1	1	0	1
GTF2F1	1	0	1	1	0	1	1	0	1
GTF2F2	1	0	1	1	0	1	1	0	1
GTF2H1	1	0	1	1	0	1	1	0	1
GTF2H2	1	0	1	1	0	1	1	0	1
GTF2H3	1	0	1	1	0	1	1	0	1
GTF2H4	1	0	0	1	0	1	0	0	1
GTF2H5	0	0	0	0	0	1	1	0	1
GTF2I	1	0	1	1	0	1	1	0	1
GTF2IRD1	0	0	0	0	0	0	0	1	0
GTF3A	1	0	0	1	0	1	1	0	1
GTF3C1	1	0	1	1	0	1	1	0	1
GTF3C2	1	0	1	1	0	1	1	0	1
GTF3C3	1	1	1	1	0	1	1	0	1
GTF3C4	1	0	1	1	0	1	1	0	1
GTF3C5	1	0	1	1	0	1	0	0	1
GTPBP1	1	0	1	1	0	1	1	0	1
GTPBP2	1	0	1	1	0	1	1	0	1
GTPBP3	1	0	1	1	0	1	1	0	1
GTPBP4	1	0	1	1	0	1	1	0	1
GTPBP8	1	0	1	1	0	1	1	0	1
GTSE1	1	0	1	0	0	1	0	0	0
GUCA1A	0	1	0	0	0	0	0	0	0
GUCY1A3	0	1	1	0	1	1	1	1	1
GUCY2D	1	0	0	0	0	1	1	0	1
GUF1	1	1	1	1	0	1	1	1	1
GUSBL1	1	0	0	0	0	1	1	0	1
GUSBL2	1	1	1	1	1	1	1	1	1
GYPC	1	0	0	1	0	1	1	0	1
GYS1	1	0	1	1	0	1	1	0	1
GYS2	1	0	0	0	0	0	0	0	0

GCOM1	0	0	0	0	1	0	0	1	0
H1F0	0	1	1	0	1	1	0	1	1
H1FNT	0	1	0	0	1	0	0	0	0
H2AFJ	1	0	1	1	0	1	1	0	1
H2AFV	0	0	0	0	0	1	1	0	1
H2AFY	0	0	0	0	0	0	0	0	1
H2AFY2	0	1	1	0	1	1	0	1	1
H2AFZ	1	0	1	1	0	1	0	0	1
H3F3A	1	0	1	1	0	1	1	0	1
H6PD	0	0	0	0	0	0	0	0	1
HAAO	1	0	1	1	0	1	1	0	1
HABP4	0	1	1	0	1	1	0	1	1
HADHA	1	0	1	1	0	1	1	0	1
HAGH	1	0	1	1	0	1	0	0	1
HAGHL	0	0	0	0	0	0	0	1	0
HAL	0	0	0	1	0	0	1	0	1
HAP1	0	1	0	0	1	0	0	1	0
HAPLN3	0	0	0	0	0	0	0	1	0
HAPLN4	0	1	0	0	1	0	0	1	0
HARS	1	0	1	1	0	1	1	0	1
HARS2	0	0	0	0	0	1	0	0	0
HAS1	0	1	0	0	1	0	0	1	0
HAS2	0	1	1	0	1	1	1	1	1
HAT1	1	0	0	1	0	1	0	0	1
HAVCR2	1	0	1	1	0	1	1	0	1
HAX1	1	0	1	1	0	1	1	0	1
HBA2	0	1	0	0	1	0	0	1	0
HBEGF	0	0	0	1	0	1	1	0	1
HBP1	1	0	1	1	0	1	1	0	1
HBS1L	1	0	1	1	0	1	1	0	1
HCCA2	0	0	0	1	0	1	0	0	0
HCCS	1	1	1	1	0	1	1	0	1
HCFC1	0	1	0	0	1	1	0	1	1
HCFC1R1	0	0	1	0	0	1	0	0	1
HCFC2	0	1	0	0	0	1	0	1	1
HCG18	1	0	1	1	0	1	1	0	1
HCG27	0	0	0	0	0	0	1	0	1
HCK	1	0	1	1	0	1	1	0	1
HCLS1	1	0	0	1	0	1	1	0	1
HCN3	0	0	0	1	0	0	0	0	1
HCP5	1	0	1	1	0	1	1	0	1
HCRTR2	0	1	1	0	1	0	0	1	0
HCST	0	0	1	1	0	1	0	0	1
HDAC1	1	0	1	1	0	1	1	0	1
HDAC2	1	0	1	1	0	1	1	0	1
HDAC3	1	0	1	1	0	1	1	0	1
HDAC4	0	0	1	1	0	1	1	0	1
HDAC6	1	1	0	0	1	1	0	0	0
HDAC7A	0	0	0	0	0	0	1	0	0
HDAC8	1	1	1	1	1	1	1	1	1
HDAC9	1	0	1	0	0	1	1	0	0
HDDC3	1	0	1	0	0	0	1	0	1
HDGF	1	0	1	1	0	1	1	0	1
HDGF2	1	0	1	1	0	1	1	0	1
HDGFRP3	0	1	1	0	1	0	0	1	1
HDHD1A	0	0	1	0	0	1	1	0	1
HDHD2	1	0	1	1	0	1	1	0	1
HDHD3	0	0	0	1	0	0	0	0	0
HDLBP	1	0	1	1	0	1	1	0	1
HEATR1	1	0	1	1	0	1	1	0	1
HEBP1	1	0	1	0	0	1	1	0	1
HECTD1	1	0	1	1	0	1	1	0	1

HECTD2	0	0	0	1	0	1	1	1	1
HECTD3	1	0	1	1	0	1	1	0	1
HECW2	0	1	1	0	1	1	0	1	1
HEL308	1	0	1	1	0	1	1	0	1
HELB	1	0	1	1	0	1	1	0	1
HELLS	1	0	1	1	0	1	1	0	1
HELZ	0	1	0	0	1	0	1	0	1
HEMGN	1	0	0	0	0	0	0	0	0
HERC1	0	0	1	1	0	1	1	0	1
HERC2	1	0	1	1	0	1	1	0	1
HERC3	1	0	1	1	0	1	1	0	1
HERC4	1	0	0	1	0	0	1	0	1
HERC5	1	0	1	1	0	1	1	0	1
HERPUD2	1	0	1	1	0	1	1	0	1
HES1	0	1	0	0	0	0	0	1	0
HES2	0	1	0	0	1	0	0	1	0
HES3	0	1	0	0	1	0	0	1	0
HES6	0	1	0	0	1	0	0	1	0
HEXA	1	0	1	1	0	1	1	0	1
HEXDC	1	0	1	1	0	1	1	0	1
HEXIM1	0	0	0	0	0	0	0	0	1
HEXIM2	1	0	1	1	0	1	1	0	1
HEY1	0	0	0	0	0	0	0	1	1
HFE2	0	1	0	0	0	0	0	0	0
HGF	0	0	0	1	0	1	1	0	1
HGS	1	0	1	1	0	1	1	0	1
HHAT	0	0	0	0	0	1	0	0	1
HHEX	1	0	1	1	0	1	1	0	1
HHLA2	0	0	0	0	0	0	1	0	0
HHLA3	1	0	1	1	0	1	1	0	1
HIAT1	1	0	1	1	0	1	1	0	1
HIBCH	0	0	0	0	0	0	0	0	1
HIC1	0	0	0	0	1	0	0	1	0
HIC2	0	0	0	0	0	0	0	1	0
HIF1A	1	0	1	1	0	1	1	0	1
HIF1AN	0	0	0	1	0	1	0	0	1
HIF3A	0	1	1	0	1	0	0	1	0
HIG2	1	0	1	1	0	1	1	0	1
HIGD1A	0	1	1	1	1	1	1	1	1
HIGD2A	1	0	1	1	0	1	1	0	1
HINT1	1	0	0	0	0	0	1	0	0
HINT2	0	0	1	0	0	1	1	0	1
HINT3	0	0	0	0	0	0	1	0	0
HIP2	0	0	1	0	0	0	0	0	1
HIPK1	1	0	1	1	0	1	1	0	1
HIRA	1	0	1	1	0	1	1	0	1
HIRIP3	1	0	1	1	0	1	0	0	1
HIST1H1C	1	0	1	0	0	1	1	0	1
HIST1H1D	1	0	0	1	0	1	1	0	1
HIST1H2AC	1	0	1	1	0	1	1	0	1
HIST1H2AH	1	0	1	1	0	1	1	0	1
HIST1H2AJ	1	0	1	1	0	1	1	0	1
HIST1H2AK	1	0	1	1	0	1	1	0	1
HIST1H2AL	1	0	1	1	0	1	1	0	1
HIST1H2BC	1	0	1	1	0	1	1	0	1
HIST1H2BD	1	0	1	1	0	1	1	0	1
HIST1H2BJ	1	0	1	1	0	1	1	0	1
HIST1H2BK	1	0	1	1	0	1	1	0	1
HIST1H2BO	1	0	1	1	0	1	1	0	1
HIST1H3D	1	0	1	1	0	1	1	0	1
HIST1H3F	1	0	1	1	0	1	1	0	1
HIST1H3G	1	0	1	1	0	1	1	0	1

HIST1H3I	1	0	1	1	0	1	1	0	1
HIST1H4A	1	0	1	1	0	1	1	1	1
HIST1H4C	1	0	1	1	0	1	1	0	1
HIST1H4E	1	0	1	1	0	1	1	0	1
HIST1H4H	1	0	1	1	0	1	1	0	1
HIST1H4K	1	0	1	1	0	1	1	0	1
HIST2H2AB	1	0	1	1	0	1	1	0	1
HIST2H2AC	1	0	1	1	0	1	1	0	1
HIST2H2BE	1	0	1	1	0	1	1	0	1
HIST3H2BB	1	1	1	1	1	1	1	1	1
HIVEP1	1	0	1	1	0	1	1	0	1
HIVEP3	0	1	1	0	1	1	0	1	1
HK2	0	0	0	0	0	0	1	0	0
HK3	1	0	1	1	0	1	1	0	1
HKR1	0	0	1	0	0	1	0	0	1
HLA-A	1	1	1	1	1	1	1	1	1
HLA-B	1	0	1	1	0	1	1	0	1
HLA-C	1	0	1	1	0	1	1	0	1
HLA-DMA	1	0	1	1	0	1	1	0	1
HLA-DMB	1	0	1	1	0	1	1	0	1
HLA-DOA	0	0	0	1	0	1	0	0	0
HLA-DPA1	1	0	1	1	1	1	1	0	1
HLA-DPB1	1	0	1	1	1	1	1	0	1
HLA-DQA1	1	0	0	1	0	1	0	0	0
HLA-DQB1	1	0	0	1	0	0	1	0	1
HLA-DQB2	0	0	0	0	0	0	1	0	0
HLA-DRA	1	0	1	1	0	1	1	0	1
HLA-DRB1	1	0	1	1	0	1	1	0	1
HLA-DRB5	1	0	1	1	0	1	1	0	1
HLA-E	0	0	0	0	0	0	0	0	1
HLA-F	0	0	1	0	0	0	0	0	1
HLA-G	0	1	1	0	1	0	0	1	0
HLCS	0	0	0	0	0	0	0	1	0
HM13	0	0	1	1	0	1	1	0	1
HMBOX1	1	0	1	1	0	1	1	0	1
HMBS	1	0	1	1	0	1	0	0	1
HMG20A	1	0	1	1	0	1	1	0	1
HMG2L1	0	0	1	0	0	1	1	0	1
HMGB1	0	0	0	1	0	1	1	0	0
HMGB2	0	0	1	1	0	1	1	0	1
HMGCL	1	0	0	1	0	1	1	0	1
HMGCR	1	0	1	1	1	1	1	0	1
HMGCS1	0	0	0	0	0	1	0	0	0
HMGN2	0	0	1	1	0	1	1	0	1
HMGN3	0	0	1	1	0	1	1	0	1
HMGN4	1	0	1	1	0	1	1	0	1
HMMR	1	0	1	1	0	1	1	0	1
HMOX1	0	0	0	0	0	1	0	0	1
HMP19	0	1	0	0	1	0	0	0	0
HNMT	1	0	1	1	0	1	1	0	1
HNRPA0	0	0	0	0	0	0	0	0	1
HNRPA1	1	0	1	1	0	1	1	0	1
HNRPA2B1	1	0	1	1	0	1	1	0	1
HNRPA3	1	0	1	1	0	1	1	0	1
HNRPAB	0	0	0	0	0	0	0	0	1
HNRPC	1	0	1	1	0	1	1	0	1
HNRPD	1	0	1	1	0	1	1	0	1
HNRPDL	1	0	1	1	0	1	1	0	1
HNRPF	1	0	1	1	0	0	1	0	0
HNRPH1	0	0	0	1	0	1	0	0	1
HNRPH2	1	1	1	1	1	1	1	0	1
HNRPH3	1	0	0	1	0	1	1	0	1

HNRPK	1	0	1	1	0	1	1	0	1
HNRPL	1	0	1	1	0	1	1	0	1
HNRPLL	1	0	1	1	0	1	1	0	1
HNRPM	0	0	0	1	0	1	1	0	1
HNRPR	1	0	1	1	0	1	1	0	1
HNRPU	1	0	1	1	0	1	1	0	1
HNRPUL1	1	0	1	1	0	1	1	1	1
HOM-TES-103	0	0	1	1	0	1	0	0	1
HOMER1	0	0	0	1	0	1	0	1	1
HOMER2	0	1	0	0	1	0	0	1	0
HOMER3	0	0	0	0	0	0	0	0	1
HOOK1	0	1	0	0	1	0	0	1	0
HOOK2	1	0	1	0	0	1	0	0	1
HOOK3	1	0	1	1	0	1	1	0	1
HOP	0	1	1	0	1	1	0	1	1
HOXA2	0	1	0	0	1	0	0	1	0
HOXA5	0	1	0	0	1	1	0	1	1
HOXA6	0	1	0	0	1	1	0	1	1
HOXA7	0	1	1	0	1	1	0	1	1
HOXB1	0	1	0	0	1	0	0	1	0
HOXB2	0	1	0	0	1	0	0	1	1
HOXB3	0	1	1	0	1	1	0	1	1
HOXB5	0	1	0	0	1	0	0	1	0
HOXB6	0	1	0	0	1	0	0	1	0
HOXB9	0	1	1	0	1	0	0	1	0
HOXC13	0	1	1	0	1	0	0	1	0
HOXC4	0	1	0	0	1	0	0	1	0
HOXC8	0	1	0	0	1	0	0	1	0
HP	0	0	0	0	0	0	1	0	1
HP1BP3	1	0	0	0	0	0	0	0	0
HPRT1	0	1	0	0	0	0	0	0	1
HPS3	1	0	1	1	0	1	1	0	1
HPS4	1	0	1	1	0	1	1	0	1
HPS5	1	0	1	1	0	1	1	0	1
HPSE	0	0	0	0	0	0	0	0	1
HRASLS3	0	0	0	0	0	1	0	1	1
HRES1	1	0	1	1	0	1	1	0	1
HRG	0	1	0	0	1	0	0	1	0
HRK	0	1	0	0	1	0	0	1	0
HRSP12	1	0	1	1	0	1	1	0	1
HS1BP3	0	0	0	0	0	0	0	0	1
HS2ST1	1	0	1	1	0	1	1	0	1
HS3ST1	1	1	1	0	1	0	0	1	1
HS3ST2	0	1	1	0	1	1	0	1	0
HS3ST3B1	1	1	1	1	1	1	1	1	1
HS3ST6	0	1	0	0	1	0	0	1	0
HS6ST1	0	0	0	0	1	0	0	1	0
HSD11B1	0	1	1	1	1	1	0	1	0
HSD11B1L	1	0	1	1	0	1	0	0	1
HSD17B2	0	1	0	0	1	0	0	0	0
HSD17B7	1	0	1	0	0	1	1	0	1
HSD17B8	1	0	1	1	0	1	1	0	1
HSD3B7	0	0	0	0	0	1	0	0	0
HSDL1	1	1	1	1	1	1	1	1	1
HSDL2	1	0	1	1	0	1	1	0	1
HSF1	1	0	1	1	0	1	1	0	1
HSF2	0	0	0	0	0	0	0	0	1
HSF2BP	1	0	1	1	0	1	0	1	1
HSF4	0	1	0	0	0	1	0	1	1
HSMPP8	0	0	0	1	0	1	0	0	1
HSP90AA1	1	1	1	1	1	1	1	1	1
HSP90AB1	1	0	1	0	0	1	1	0	1

HSP90B1	0	0	1	1	0	1	1	0	1
HSPA14	1	0	1	1	0	1	1	0	1
HSPA1A	1	0	1	1	0	1	1	0	1
HSPA1B	1	0	1	1	0	1	1	0	1
HSPA1L	0	0	1	1	0	1	0	0	1
HSPA4	1	0	1	1	0	1	0	0	1
HSPA4L	1	1	1	1	1	1	1	1	1
HSPA5	1	0	1	1	0	1	1	0	1
HSPA6	0	1	0	0	1	0	0	1	0
HSPA8	1	0	1	1	0	1	1	0	1
HSPB2	0	1	0	0	1	0	0	1	0
HSPB7	0	1	0	0	1	0	0	0	0
HSPBAP1	1	0	1	1	0	1	1	0	1
HSPBP1	0	1	0	0	1	0	0	1	0
HSPC111	1	0	1	1	0	1	1	0	1
HSPC152	1	0	1	1	0	1	1	0	1
HSPC159	0	1	0	0	0	0	0	1	1
HSPC171	1	0	1	1	0	1	0	0	1
HSPD1	1	0	1	1	0	1	1	0	1
HSPE1	1	0	1	1	0	1	1	0	1
HSPH1	1	0	1	1	0	1	1	0	1
HTATIP2	0	0	0	0	0	1	0	0	0
HTATSF1	1	1	1	1	0	1	1	0	1
HTF9C	1	0	1	1	0	1	0	0	1
HTR2B	0	0	0	0	0	0	1	0	0
HTR3B	0	1	0	0	0	0	0	0	0
HTR7	0	0	0	0	0	0	1	0	1
HTRA1	0	1	0	0	1	0	0	1	0
HTRA2	1	0	1	1	0	1	0	0	1
HTRA4	1	0	0	1	0	1	1	0	0
HUWE1	0	1	0	0	1	0	1	1	0
HYAL1	0	0	0	1	0	0	1	0	1
HYAL3	0	0	0	1	0	0	1	0	1
HYLS1	0	1	0	0	0	0	0	0	0
HYOU1	1	0	1	1	0	1	0	0	1
HYPE	0	0	0	0	0	1	0	0	0
HYPK	1	0	1	1	0	1	1	0	1
IARS	1	0	1	1	0	1	1	0	1
IBRDC2	1	0	0	0	0	1	0	0	1
IBRDC3	0	0	1	0	0	0	0	0	1
IBTK	0	0	0	0	0	1	0	0	1
ICA1	0	1	0	0	1	0	1	1	0
ICAM3	0	0	0	0	0	0	1	0	1
ICAM4	0	0	0	1	0	0	0	1	1
ICAM5	0	0	0	1	0	0	0	1	1
ICF45	1	0	1	1	0	1	0	0	1
ICK	1	0	1	1	0	1	1	1	1
ICMT	0	1	0	0	0	0	0	1	0
ICT1	0	0	0	0	0	1	0	0	1
ID1	0	0	1	0	0	1	0	0	1
ID2	1	0	1	1	0	1	0	1	1
ID2B	1	0	1	0	0	0	0	0	1
ID3	1	0	0	1	0	1	0	1	1
ID4	0	1	1	0	1	0	0	1	0
IDE	1	0	1	1	0	1	1	0	1
IDH1	1	0	1	0	0	1	1	0	1
IDH2	0	1	0	0	0	0	0	0	0
IDH3A	1	0	0	1	0	0	0	0	0
IDH3B	0	0	0	0	0	1	0	0	1
IDH3G	1	1	1	1	1	1	0	1	1
IDI1	1	0	1	1	0	1	1	0	1
IDS	0	1	1	0	0	1	0	0	1

IDUA	0	0	0	1	0	1	0	0	0
IER2	1	0	1	1	0	1	1	0	1
IER3	1	0	1	1	0	1	1	0	1
IER3IP1	0	1	0	0	0	0	0	0	0
IER5	1	0	0	0	0	1	0	0	1
IFI16	1	0	1	1	0	1	1	0	1
IFI30	1	0	0	1	0	1	1	0	1
IFI35	0	0	0	1	0	1	1	0	0
IFI44L	1	0	0	1	0	0	1	0	1
IFIH1	1	0	1	1	0	1	1	0	1
IFIT1	0	0	0	0	0	1	1	0	1
IFIT2	1	0	1	1	0	1	1	0	1
IFIT3	1	0	1	1	0	1	1	0	1
IFIT5	1	0	1	1	0	1	1	0	1
IFITM1	0	1	0	0	1	0	1	1	1
IFITM3	0	1	0	1	1	1	1	1	1
IFITM5	0	1	0	0	1	0	0	1	0
IFNA13	0	1	0	0	1	0	0	1	0
IFNAR1	1	0	1	1	0	1	1	0	1
IFNAR2	1	0	1	1	0	1	0	0	1
IFNGR1	1	0	1	1	0	0	1	0	1
IFRD1	1	0	1	1	0	1	1	0	1
IFRD2	1	0	0	1	0	1	0	0	0
IFRG15	0	0	0	1	0	0	0	0	0
IFT122	1	0	1	1	0	1	1	0	1
IFT140	0	0	0	1	0	0	0	0	0
IFT172	0	0	1	1	0	1	1	0	1
IFT52	1	0	1	1	0	1	1	0	1
IFT57	1	0	1	1	0	1	1	0	1
IFT74	1	0	1	1	0	1	1	0	1
IFT80	1	0	1	1	0	1	1	0	1
IFT88	0	0	0	0	0	0	0	0	1
IGBP1	0	1	0	1	1	1	0	0	1
IGF2BP2	0	1	0	0	1	0	0	1	1
IGF2BP3	0	1	1	0	1	1	0	1	1
IGFBP2	0	1	1	0	1	0	0	1	0
IGFBP6	0	0	1	0	0	1	0	1	1
IGFBP7	1	0	1	1	0	1	1	0	1
IGFBPL1	0	1	0	0	1	0	0	1	0
IGFL3	0	0	0	0	1	0	0	1	0
IGHMBP2	1	0	1	1	0	1	1	0	1
IGSF10	0	1	0	0	1	0	0	1	0
IGSF11	0	1	1	0	1	0	0	1	0
IGSF2	0	0	0	1	0	0	0	0	0
IGSF6	1	0	1	1	0	1	1	0	1
IGSF8	1	0	0	0	0	0	0	0	0
IHH	0	1	0	0	1	0	0	1	0
IHPK2	1	0	1	1	1	1	1	0	1
IK	1	0	1	1	0	1	1	0	1
IKBKB	0	0	1	1	0	1	1	0	1
IKBKE	0	0	0	0	0	1	0	0	0
IKBKG	1	1	1	1	0	1	0	0	1
IKIP	1	0	1	1	0	1	1	0	1
IL10RA	0	0	0	0	0	0	0	0	1
IL10RB	1	0	1	1	0	1	1	0	1
IL11RA	0	0	0	1	0	1	0	0	0
IL12RB1	0	0	0	1	0	1	1	0	1
IL15	0	0	1	0	0	0	1	1	1
IL16	1	1	0	0	1	1	1	1	1
IL17C	0	1	0	0	1	0	0	1	0
IL17RB	0	1	1	1	1	1	0	1	1
IL17RC	1	0	1	1	0	1	1	0	1

IL18	1	0	1	0	0	1	1	0	1
IL18RAP	1	0	0	0	0	0	0	0	0
IL1A	1	0	0	0	0	0	0	0	0
IL1B	0	0	0	1	0	1	1	0	1
IL1F10	0	1	0	0	0	0	0	0	0
IL1F8	0	1	0	0	0	0	0	0	0
IL1RAP	0	0	0	0	0	0	0	0	1
IL1RAPL1	0	1	0	0	1	0	0	1	0
IL1RL2	1	0	0	0	0	0	0	1	0
IL1RN	1	0	0	0	0	1	1	0	1
IL2	1	0	0	1	0	0	1	0	0
IL21	0	1	0	0	0	0	0	0	0
IL21R	1	0	1	1	0	1	1	1	1
IL26	0	1	0	0	0	0	0	0	0
IL27	0	0	0	0	0	1	1	0	1
IL27RA	0	0	0	0	0	0	1	0	1
IL28A	0	1	0	0	1	0	0	1	0
IL28RA	0	0	0	0	0	0	0	1	0
IL2RG	0	1	1	1	1	1	0	0	0
IL3RA	1	0	1	1	0	1	1	0	1
IL4I1	0	0	0	0	0	0	0	0	1
IL4R	1	0	0	0	0	1	0	0	1
IL6	0	0	0	0	0	0	0	1	0
IL6R	0	0	0	0	0	0	0	0	1
IL6ST	1	0	0	0	0	1	1	0	1
IL7R	1	0	0	0	0	0	0	0	0
IL8	0	0	0	1	0	1	1	0	0
IL8RA	0	0	1	0	0	0	0	0	0
IL8RB	1	0	0	0	0	1	1	0	1
ILF2	1	0	1	1	0	1	1	0	1
ILF3	1	0	1	0	0	1	0	0	1
ILK	1	0	1	1	0	1	1	0	1
ILVBL	0	0	0	1	0	1	1	0	1
IMMP2L	1	0	1	0	0	0	0	0	1
IMMT	1	0	1	1	0	1	1	0	1
IMP4	1	0	1	1	0	1	0	0	1
IMPA1	1	0	1	1	0	1	0	0	1
IMPA2	1	0	0	1	0	1	1	0	1
IMPACT	1	0	1	0	0	1	0	0	1
IMPAD1	1	0	1	1	0	1	1	0	1
INCA	1	0	1	1	0	1	1	0	1
INCENP	1	0	1	1	0	0	0	0	0
INDO	1	0	1	1	0	1	1	0	1
ING1	1	0	1	1	0	1	1	0	1
ING2	1	0	1	1	0	1	1	0	1
ING3	1	0	1	1	0	1	1	0	1
ING4	0	0	0	0	0	1	0	0	0
INHBB	0	0	0	0	1	0	0	1	0
INOC1	1	0	1	1	0	1	1	0	1
INPP1	0	0	0	0	0	0	0	0	1
INPP4A	0	0	0	1	0	0	0	0	0
INPP5A	0	0	0	0	0	0	0	0	1
INPP5B	0	0	1	0	0	1	0	0	0
INPP5F	1	0	1	1	0	1	0	0	1
INPPL1	0	0	0	0	0	1	1	0	1
INSIG1	1	0	1	0	0	1	0	0	0
INSIG2	0	0	1	1	0	1	0	0	1
INSM1	0	1	0	0	1	1	0	1	1
INSM2	0	1	0	0	1	0	0	1	1
INSR	0	0	0	1	0	1	1	0	1
INSRR	0	1	0	0	1	0	0	1	0
INTS10	0	0	0	1	0	1	0	0	1

INTS12	1	0	1	1	0	1	1	0	1
INTS2	1	0	1	1	0	1	1	0	1
INTS4	1	0	1	1	0	1	1	0	1
INTS5	0	0	0	0	0	1	0	0	0
INTS6	1	0	1	1	0	1	1	0	1
INTS7	1	0	1	1	0	1	1	0	1
INTS8	1	0	1	0	0	1	0	0	1
INVS	1	0	1	1	0	1	1	0	1
IPO11	0	0	0	0	0	1	0	0	1
IPO13	1	0	1	1	0	1	1	0	1
IPO9	1	0	0	1	0	1	0	0	0
IPPK	1	0	1	1	0	1	0	0	1
IQCB1	1	0	1	1	0	1	1	0	1
IQCC	0	0	0	0	0	1	1	0	0
IQCG	1	0	1	1	0	1	1	0	1
IQCH	1	0	1	1	1	1	1	1	1
IQGAP2	1	0	1	1	0	1	1	0	1
IQGAP3	0	0	1	0	0	1	1	0	1
IQSEC2	1	1	0	0	1	1	1	1	0
IQWD1	1	0	1	1	0	1	1	0	1
IRAK1	0	1	0	0	1	0	0	1	0
IRAK4	1	0	1	1	0	1	1	0	1
IREB2	1	0	1	1	0	1	1	0	1
IRF1	0	0	0	0	0	0	0	0	1
IRF2	1	0	1	1	0	1	1	0	1
IRF2BP1	1	0	1	1	0	1	1	0	1
IRF2BP2	0	0	0	0	1	0	0	0	0
IRF3	1	0	1	1	0	1	1	0	1
IRF4	0	1	0	0	1	1	0	1	1
IRF5	0	0	0	0	0	1	0	0	0
IRF8	1	0	1	1	0	1	1	0	1
IRGQ	1	0	1	1	0	1	1	0	1
IRS1	0	1	1	0	1	1	0	1	1
IRX1	0	1	1	0	1	1	0	1	0
IRX2	0	1	0	0	1	0	0	1	0
IRX6	0	1	0	0	1	0	0	1	0
ISG20L2	1	0	1	1	0	1	1	0	1
ISGF3G	0	0	0	0	0	0	0	0	1
ISOC1	0	1	0	0	0	0	0	1	1
ISOC2	0	0	1	0	0	1	0	0	0
ISYNA1	0	0	0	0	1	0	0	1	0
ITFG1	1	0	1	1	0	1	1	0	1
ITFG2	0	0	1	1	0	1	0	0	1
ITGA11	0	1	1	0	1	0	0	1	0
ITGA2	0	1	1	0	1	1	1	1	1
ITGA3	1	1	1	0	1	1	1	1	1
ITGA4	0	0	0	1	0	1	1	0	1
ITGA5	0	0	1	1	0	1	1	0	1
ITGA7	0	1	0	0	0	0	0	1	1
ITGAL	0	0	0	1	0	1	0	0	1
ITGAM	1	0	1	0	0	1	0	0	1
ITGAV	1	0	1	0	0	0	1	0	1
ITGAX	1	0	1	1	0	1	1	0	1
ITGB1	1	0	1	1	0	1	1	0	1
ITGB1BP1	1	0	1	1	0	1	1	0	1
ITGB1BP2	0	1	0	0	0	0	0	0	0
ITGB1BP3	0	1	0	0	1	0	0	1	0
ITGB2	1	0	1	0	0	1	0	0	1
ITGB3	0	1	0	0	1	0	0	1	0
ITGB3BP	1	0	1	1	0	1	1	0	1
ITGB4	0	1	0	0	1	0	0	1	0
ITGB7	0	0	0	0	0	0	1	0	1

ITIH5L	0	1	0	0	1	0	0	0	0
ITM2B	0	1	0	1	0	1	1	1	1
ITM2C	0	1	0	0	1	0	0	1	0
ITPA	1	0	1	1	0	1	1	0	1
ITPK1	0	0	0	0	0	0	1	0	1
ITPKA	0	1	0	0	1	0	0	1	1
ITPKB	1	0	1	1	0	1	1	0	1
ITPKC	0	0	1	1	0	1	0	0	1
ITPR1	1	0	1	1	0	1	1	0	1
ITPR2	0	0	1	1	0	1	1	0	1
ITPR3	0	1	0	0	1	1	1	1	1
ITSN1	1	0	1	1	0	1	1	0	1
IVL	0	1	0	0	1	0	0	0	0
IVNS1ABP	1	0	1	1	0	1	1	0	1
IWS1	1	0	1	1	0	1	1	0	1
IZUMO1	0	1	0	0	0	0	0	1	0
JAG1	1	0	0	1	1	1	1	1	1
JAK2	1	0	1	1	0	1	1	0	1
JAK3	0	0	0	0	0	0	1	0	0
JAKMIP2	0	0	0	1	0	1	1	0	0
JARID1A	1	0	1	1	0	1	1	0	1
JARID1B	1	0	1	0	0	1	1	1	1
JARID2	1	0	1	1	0	1	1	0	1
JAZF1	0	0	0	0	0	0	1	0	1
JDP2	0	0	0	0	0	1	0	0	0
JMJD1A	0	0	0	0	0	0	0	0	1
JMJD2C	1	0	1	1	0	1	1	0	1
JMJD2D	1	0	1	1	0	1	1	0	1
JMJD4	0	0	0	1	0	1	0	0	1
JMJD5	1	0	1	1	0	1	1	0	1
JOSD1	1	0	1	1	0	1	1	0	1
JOSD2	1	1	1	1	0	1	0	1	0
JPH2	0	1	0	0	1	0	0	1	0
JPH3	0	1	0	0	1	0	0	1	0
JPH4	0	1	0	0	1	0	0	1	0
JRKL	1	0	1	1	0	1	1	0	1
JTB	1	0	0	0	0	1	0	0	0
JTV1	1	0	1	1	0	1	0	0	1
JUB	1	1	1	1	1	1	1	1	1
JUN	1	0	1	1	0	1	1	1	1
JUNB	1	0	1	1	0	1	1	0	1
JUND	1	0	0	1	0	1	1	0	1
KAL1	0	1	0	0	1	0	0	1	0
KARS	1	0	1	1	0	1	1	0	1
KATNAL1	1	0	1	1	0	1	1	0	1
KATNAL2	1	1	1	1	0	1	1	1	1
KATNB1	1	0	1	1	0	1	1	0	1
KBTBD11	1	1	1	1	1	1	1	1	1
KBTBD2	1	0	1	1	0	1	1	0	1
KBTBD3	1	0	1	1	0	1	1	0	1
KBTBD6	1	0	1	1	0	1	1	0	1
KBTBD8	1	0	1	1	0	1	1	0	1
KCMF1	1	0	1	1	0	1	1	0	1
KCNA3	0	0	0	0	0	0	0	1	1
KCNAB1	0	1	0	0	1	0	0	1	0
KCNAB2	0	0	0	0	0	1	0	0	0
KCNAB3	1	0	1	1	0	1	1	0	1
KCNC4	0	0	0	0	0	0	0	1	1
KCNE1L	0	1	0	0	1	0	0	0	0
KCNE3	1	0	1	1	0	1	1	0	1
KCNG1	0	1	0	0	1	0	0	1	0
KCNG4	0	1	1	0	0	0	0	1	0

KCNH3	0	1	0	0	0	1	0	0	1
KCNH6	0	1	0	0	1	0	0	1	0
KCNIP1	0	1	0	0	1	0	0	1	0
KCNJ10	0	1	0	0	0	0	0	1	1
KCNJ11	0	0	0	0	1	0	0	1	0
KCNJ2	1	1	1	1	0	1	1	0	1
KCNJ4	0	1	0	0	1	0	0	1	0
KCNJ5	0	1	0	0	1	1	0	1	0
KCNK13	0	0	0	0	0	0	0	1	0
KCNK3	0	1	0	0	1	0	0	1	0
KCNK5	0	0	1	0	0	1	0	0	0
KCNK6	1	0	1	0	0	1	0	0	1
KCNK9	0	1	0	0	0	0	0	1	0
KCNMA1	0	1	0	0	1	1	0	1	0
KCNMB1	1	0	0	0	0	1	1	0	1
KCNMB2	0	1	0	0	1	0	1	1	0
KCNMB4	0	1	0	0	1	1	1	1	1
KCNN4	0	0	0	1	0	1	0	0	0
KCNQ1	0	0	0	0	0	0	0	1	0
KCNS3	0	1	0	0	1	0	0	1	0
KCTD10	1	0	1	1	0	1	1	0	1
KCTD12	0	0	0	0	0	0	0	0	1
KCTD13	1	0	0	1	0	1	1	0	1
KCTD17	0	0	0	0	0	0	1	0	1
KCTD18	1	0	1	1	0	1	1	0	1
KCTD2	1	0	1	1	0	1	0	0	1
KCTD3	1	0	0	1	0	1	1	0	1
KCTD5	0	0	0	0	0	0	0	0	1
KCTD6	1	0	0	0	0	0	0	0	0
KCTD7	1	0	0	1	0	1	1	0	1
KCTD8	0	1	0	0	1	0	0	1	0
KCTD9	1	0	1	1	0	1	1	0	1
KDELR1	0	1	0	0	1	0	0	1	0
KDR	0	1	1	0	1	1	0	1	1
KHDRBS1	0	0	0	0	0	1	0	0	1
KHDRBS3	0	1	0	0	1	1	0	1	0
KHK	0	0	0	0	0	0	0	0	1
KIAA0020	1	0	0	1	0	0	1	0	0
KIAA0040	1	0	0	1	0	1	1	0	1
KIAA0090	1	0	1	1	0	1	1	0	1
KIAA0100	1	0	1	1	0	1	1	0	1
KIAA0101	1	0	0	1	0	1	1	0	1
KIAA0133	1	0	1	1	0	1	1	0	1
KIAA0141	1	0	1	1	0	1	1	0	1
KIAA0152	0	0	0	1	0	0	0	0	1
KIAA0174	1	0	1	1	0	1	0	0	1
KIAA0179	1	0	0	1	0	1	0	1	1
KIAA0182	1	0	0	1	0	1	1	0	1
KIAA0195	1	0	1	1	0	1	1	0	1
KIAA0196	1	0	1	1	0	1	1	0	1
KIAA0232	1	0	0	0	0	0	0	0	1
KIAA0241	1	0	1	1	0	1	1	0	1
KIAA0247	1	0	1	0	0	1	0	0	0
KIAA0251	1	0	0	1	0	1	0	1	1
KIAA0256	0	0	0	0	0	1	1	0	1
KIAA0265	1	0	1	1	0	1	1	0	1
KIAA0284	0	1	0	0	1	0	0	1	0
KIAA0286	1	0	1	1	0	1	1	0	1
KIAA0319L	1	0	1	1	0	1	1	0	1
KIAA0323	0	0	0	1	0	1	0	0	0
KIAA0329	1	0	1	1	0	1	1	0	1
KIAA0372	1	0	1	1	0	1	1	0	1

KIAA0391	1	0	1	1	0	1	1	0	1
KIAA0406	1	0	1	1	0	1	1	0	1
KIAA0408	0	0	0	0	0	0	0	1	0
KIAA0409	1	0	1	1	0	1	1	0	1
KIAA0423	1	0	1	1	0	1	1	0	1
KIAA0427	0	0	0	0	0	0	0	0	1
KIAA0460	0	0	0	0	0	0	0	0	1
KIAA0494	1	0	0	1	0	1	1	0	1
KIAA0513	1	0	1	1	0	1	1	0	1
KIAA0556	1	0	1	1	0	1	1	0	1
KIAA0586	1	0	1	1	0	1	1	0	1
KIAA0644	0	1	0	0	1	0	0	1	1
KIAA0649	0	0	0	0	0	0	0	1	0
KIAA0652	1	0	1	1	0	1	1	0	1
KIAA0664	1	0	1	1	0	1	0	0	1
KIAA0701	1	0	1	0	0	1	0	0	0
KIAA0738	0	0	0	1	0	0	1	0	1
KIAA0746	0	1	0	0	0	0	0	1	0
KIAA0753	1	0	1	1	0	1	1	0	1
KIAA0776	1	0	1	1	0	1	1	0	1
KIAA0802	0	0	0	0	1	0	0	0	0
KIAA0828	1	0	1	1	0	1	0	0	1
KIAA0831	1	0	1	1	0	1	1	0	1
KIAA0859	1	0	1	1	0	1	1	0	1
KIAA0892	1	0	1	1	0	1	1	0	1
KIAA0907	1	0	1	1	0	1	1	0	1
KIAA0922	0	1	1	0	0	0	1	0	1
KIAA0999	0	0	0	1	0	1	0	0	1
KIAA1009	1	1	1	1	1	1	1	1	1
KIAA1012	1	0	1	1	0	1	1	0	1
KIAA1024	0	1	0	0	0	0	0	0	0
KIAA1026	0	1	0	0	1	0	0	1	0
KIAA1033	1	1	1	1	0	1	1	1	1
KIAA1143	1	0	1	1	0	1	0	0	1
KIAA1160	1	0	1	1	0	1	1	0	1
KIAA1161	1	1	1	1	1	1	1	1	1
KIAA1191	0	1	0	1	1	1	0	1	0
KIAA1199	0	1	1	0	1	0	0	1	0
KIAA1219	0	0	1	0	0	1	1	0	1
KIAA1267	0	0	0	1	0	0	0	0	0
KIAA1274	0	1	0	0	1	0	0	1	0
KIAA1279	1	0	1	0	0	1	1	0	1
KIAA1328	1	0	1	1	0	1	1	0	1
KIAA1333	1	0	1	1	0	1	1	0	1
KIAA1344	0	1	0	0	0	1	1	1	1
KIAA1370	1	0	1	1	0	1	1	0	1
KIAA1407	1	0	1	1	0	1	1	0	1
KIAA1429	1	0	1	1	0	1	1	0	1
KIAA1446	0	1	0	0	1	0	0	1	0
KIAA1467	0	1	0	0	0	1	0	0	0
KIAA1505	1	0	1	1	0	1	1	0	1
KIAA1524	1	0	1	1	0	1	1	0	1
KIAA1530	1	0	1	1	0	1	1	0	1
KIAA1539	0	0	0	0	0	1	0	0	1
KIAA1542	0	0	0	0	0	1	0	1	0
KIAA1586	1	0	1	1	0	1	1	0	1
KIAA1598	0	0	0	0	0	0	0	0	1
KIAA1600	0	0	0	0	0	0	1	0	1
KIAA1604	1	0	1	1	0	1	1	0	1
KIAA1627	1	0	1	1	0	1	1	0	1
KIAA1704	1	0	1	1	0	1	1	0	1
KIAA1706	1	0	0	0	0	0	0	0	0

KIAA1715	1	1	1	1	0	1	1	1	1
KIAA1727	0	1	0	0	1	0	0	1	0
KIAA1729	0	1	0	0	1	1	0	1	0
KIAA1737	1	0	1	1	0	1	0	0	1
KIAA1754	1	0	0	0	0	0	0	0	0
KIAA1754L	0	0	1	0	0	1	1	0	1
KIAA1787	1	0	1	0	0	0	1	0	1
KIAA1799	1	0	1	1	0	1	1	0	1
KIAA1804	0	0	0	0	0	0	0	1	0
KIAA1822	0	1	0	0	1	0	0	1	0
KIAA1826	1	0	1	0	0	1	0	0	1
KIAA1958	0	0	0	0	0	1	0	0	1
KIAA1984	0	1	0	0	1	0	0	1	0
KIAA2010	1	0	1	1	0	1	1	0	1
KIF11	0	0	1	0	0	1	1	0	1
KIF13A	1	0	1	1	0	1	1	0	1
KIF13B	0	0	0	1	0	1	0	0	0
KIF14	1	0	1	1	0	1	1	0	0
KIF15	1	0	1	1	0	1	0	0	1
KIF17	0	1	0	0	1	0	0	1	0
KIF18A	1	0	1	1	0	1	1	0	1
KIF1B	0	0	0	0	0	0	0	0	1
KIF1C	0	0	0	0	0	0	0	1	0
KIF20A	1	0	1	1	0	1	1	0	1
KIF21A	0	1	0	0	1	0	0	1	0
KIF24	1	0	1	1	0	1	1	0	1
KIF2C	0	0	1	0	0	1	0	0	1
KIF4A	0	1	1	1	1	1	0	0	1
KIF5B	0	0	1	1	0	1	1	0	1
KIFAP3	1	0	1	1	0	1	1	0	1
KIFC1	0	0	0	0	0	1	0	0	0
KIFC3	0	0	0	0	0	0	0	1	0
KIR2DS3	1	1	0	0	1	0	0	1	0
KIR2DS4	0	1	0	0	1	0	0	1	0
KIRREL2	0	1	0	0	1	0	0	1	0
KIRREL3	0	1	1	0	1	0	0	1	0
KL	0	1	0	0	1	0	0	1	1
KLC2	0	1	1	0	1	1	0	1	1
KLC3	0	1	0	0	1	0	0	1	0
KLC4	1	0	1	1	0	1	1	0	1
KLF10	0	0	1	0	0	1	1	0	1
KLF11	0	0	0	0	0	0	0	0	1
KLF12	0	1	0	0	1	1	0	1	1
KLF13	1	0	1	1	0	1	1	0	1
KLF15	0	1	0	0	1	0	0	1	0
KLF2	0	0	0	0	1	0	0	1	0
KLF3	1	0	1	1	0	1	0	0	1
KLF4	0	0	0	0	0	1	1	1	1
KLF5	0	1	1	0	1	1	0	1	1
KLF6	1	0	0	0	0	0	0	0	0
KLF7	1	0	1	1	0	1	1	0	1
KLF9	1	0	1	1	0	1	1	0	1
KLHDC1	1	0	1	1	0	1	1	0	1
KLHDC2	1	0	1	1	0	1	1	0	1
KLHDC3	1	0	1	1	0	1	0	0	1
KLHDC4	1	0	0	0	0	0	0	0	0
KLHDC5	0	0	1	0	0	1	0	0	1
KLHDC8B	1	0	1	1	0	0	0	0	0
KLHL15	1	1	0	0	0	0	0	1	0
KLHL17	1	0	1	0	0	1	0	0	0
KLHL18	1	0	1	1	0	1	1	0	1
KLHL2	1	0	1	1	0	1	1	0	1

KLHL20	0	0	0	0	0	1	0	1	1
KLHL21	1	0	1	1	0	0	1	0	1
KLHL24	1	0	1	1	0	1	1	0	1
KLHL25	0	1	0	0	1	0	0	0	0
KLHL3	0	0	0	0	0	1	1	0	0
KLHL5	1	0	1	1	0	1	1	0	1
KLHL6	1	0	1	1	0	1	1	0	1
KLHL7	0	0	1	0	0	0	0	0	1
KLHL8	1	0	1	1	0	1	1	0	1
KLK1	0	1	0	0	1	0	0	1	0
KLK10	0	1	0	0	0	0	0	1	0
KLK15	0	1	0	0	1	0	0	1	0
KLK4	0	1	0	0	1	0	0	1	0
KLK7	0	1	0	0	1	0	0	1	0
KLK8	0	1	0	0	1	0	0	1	0
KLRG1	0	0	1	0	0	0	1	0	0
KMO	1	0	1	1	0	1	1	0	1
KNTC1	1	0	1	1	0	1	1	0	1
KPNA1	0	0	1	0	0	1	0	0	1
KPNA3	1	0	1	1	0	1	1	0	1
KPNA4	1	0	1	1	0	1	1	0	1
KPNA5	1	0	1	1	0	1	1	0	1
KPNA6	1	0	1	1	0	1	1	0	1
KPNB1	1	0	1	1	0	1	1	0	1
KPTN	0	0	0	0	0	0	0	0	1
KREMEN1	0	1	1	0	1	1	0	1	1
KREMEN2	0	1	0	0	1	0	0	1	0
KRIT1	1	0	1	1	0	1	1	0	1
KRT10	0	1	0	0	1	0	0	1	0
KRT23	0	1	0	0	0	0	0	0	0
KRT24	0	1	0	0	1	0	1	1	0
KRT3	0	1	0	0	0	0	0	0	0
KRT6B	0	1	0	0	0	0	0	0	0
KRTAP10-2	0	1	0	0	0	0	0	0	0
KRTAP12-1	0	1	0	0	0	0	0	0	0
KRTAP12-4	0	1	1	0	1	0	0	0	0
KRTAP3-2	0	1	0	0	1	0	0	0	0
KRTAP5-1	0	1	0	0	0	0	0	1	0
KRTAP9-3	0	1	0	0	1	0	0	0	0
KRTAP9-4	0	1	0	0	0	0	0	0	0
KRTCAP2	1	0	1	1	0	1	1	0	1
KTI12	0	0	0	0	0	1	0	0	1
KTN1	0	0	0	1	0	1	1	0	1
KYNU	1	0	1	1	0	1	1	0	1
KUA	0	0	0	0	0	0	1	0	1
KUA-UEV	0	0	0	0	0	0	1	0	1
L1CAM	0	1	0	0	1	0	0	1	0
L2HGDH	1	0	1	1	0	1	1	0	1
L3MBTL	0	0	0	0	0	0	0	0	1
L3MBTL3	0	0	0	0	0	1	0	0	0
LACE1	0	0	1	1	0	1	0	0	1
LACTB	0	0	0	0	0	0	0	0	1
LACTB2	0	0	1	1	0	1	1	0	1
LAIR1	0	0	1	0	0	1	1	0	1
LAIR2	0	1	0	0	1	0	0	1	0
LAMA1	0	1	0	0	1	0	0	1	0
LAMA2	0	1	0	0	1	0	0	1	0
LAMA3	0	1	1	0	1	1	0	1	1
LAMB1	0	1	1	0	1	0	0	1	1
LAMB2	0	0	0	0	0	1	0	0	0
LAMC1	0	1	0	0	1	0	1	1	1
LAMC2	0	1	0	0	1	0	0	1	0

LAMP2	0	1	0	0	0	1	0	1	1
LAMP3	1	1	1	0	0	1	0	1	1
LANCL1	1	0	0	0	0	1	0	0	1
LANCL2	1	1	1	1	0	1	1	1	1
LAPTM4A	1	0	1	1	0	1	0	0	1
LAPTM4B	1	0	1	0	0	1	0	1	1
LAPTM5	1	0	0	1	0	1	1	0	1
LARGE	0	1	0	0	0	0	0	1	0
LARP1	1	0	1	1	0	0	0	0	1
LARP2	1	0	1	0	0	0	1	0	1
LARP4	1	0	1	1	0	1	1	0	1
LARS	1	0	1	1	0	1	0	0	1
LARS2	0	0	0	1	0	1	1	0	1
LAS1L	1	1	1	1	1	1	0	1	1
LASP1	1	0	1	1	0	1	1	0	1
LASS2	0	0	0	0	0	0	0	0	1
LASS4	0	1	0	0	1	0	0	1	0
LASS5	0	0	0	0	0	0	0	0	1
LATS1	0	0	0	0	0	1	1	0	1
LAYN	0	1	0	0	1	0	0	1	0
LBH	0	1	1	0	1	0	0	1	1
LBP	0	1	0	0	1	0	0	0	0
LBR	1	0	1	1	0	1	1	0	1
LBXCOR1	0	1	1	0	1	1	0	1	1
LCE1A	0	1	0	0	0	0	0	0	0
LCMT1	1	0	1	1	0	1	1	0	1
LCMT2	1	0	1	1	0	1	1	1	1
LCN1	0	1	0	0	0	0	0	0	0
LCP1	1	0	1	1	0	1	1	0	1
LCP2	1	0	1	1	0	1	1	0	1
LDB1	1	0	1	1	0	1	1	0	1
LDHA	1	0	1	0	0	0	0	0	1
LDHB	0	0	0	0	0	0	1	0	1
LDLR	0	0	0	1	0	0	0	0	1
LDLRAD2	0	1	0	0	0	0	0	0	0
LDLRAD3	0	1	0	0	0	0	0	0	1
LELP1	0	1	0	0	1	0	0	0	0
LEMD3	1	0	1	1	0	1	1	0	1
LENG1	1	0	1	1	0	1	0	0	1
LENG4	1	0	1	0	0	1	1	0	1
LENG8	1	0	1	1	0	1	1	0	1
LENG9	1	0	1	1	0	1	1	0	1
LEO1	0	0	0	0	0	1	0	0	1
LEPR	0	0	0	0	0	0	0	0	1
LEPRE1	1	0	1	1	0	1	1	0	1
LEPREL1	0	1	0	0	1	0	0	1	0
LEPREL2	0	0	0	0	1	0	0	1	0
LEPROT	0	0	0	0	0	0	0	0	1
LETMD1	1	0	1	1	0	1	1	0	1
LGALS12	0	0	0	0	0	1	1	0	1
LGALS2	1	0	0	0	0	0	1	0	1
LGALS3	1	0	1	0	0	0	1	0	0
LGALS4	1	0	0	0	0	0	0	0	0
LGALS9	0	0	1	0	0	1	1	0	1
LGI2	0	1	0	0	1	0	0	1	0
LGICZ1	0	1	1	0	1	0	0	1	0
LGMN	0	1	0	0	1	0	0	1	1
LGR4	0	1	0	0	1	0	0	1	1
LGR6	0	1	0	0	1	0	0	1	0
LHB	0	1	1	0	1	1	1	1	1
LHFP	0	1	0	0	1	1	1	1	1
LHFPL1	0	1	1	1	1	1	0	1	1

LHFPL2	1	0	1	0	0	1	0	0	1
LHX2	0	1	1	0	1	1	0	1	1
LHX6	0	1	1	0	1	1	0	1	0
LIAS	1	0	1	1	0	1	1	0	1
LIG1	1	0	1	1	0	1	0	0	1
LIG3	1	0	1	1	0	1	1	0	1
LIG4	1	0	1	1	0	1	1	0	1
LILRA1	0	0	1	0	1	1	1	0	1
LILRA2	0	0	0	0	0	1	0	0	1
LILRA3	0	0	0	0	0	0	0	0	1
LILRA5	0	0	0	0	0	0	0	0	1
LILRA6	0	1	1	0	0	1	0	1	0
LILRB1	0	0	1	0	0	1	1	0	1
LILRB2	0	0	1	0	0	1	1	0	1
LILRB3	0	0	0	0	0	1	0	0	0
LILRB4	1	0	1	1	0	1	1	0	1
LILRB5	0	1	1	0	0	1	0	1	0
LIMA1	1	0	1	1	0	1	1	0	1
LIMD1	1	0	0	0	0	0	1	0	1
LIMD2	0	0	0	0	0	0	0	0	1
LIME1	0	1	0	0	1	0	0	1	0
LIMK2	1	0	0	1	0	1	1	0	1
LIMS1	1	0	1	1	0	1	1	0	1
LIMS2	0	1	0	0	1	0	0	1	0
LIN7A	1	1	1	1	1	1	1	1	1
LIN7C	1	0	1	1	0	1	1	0	1
LIN9	0	1	1	0	1	1	0	0	1
LINS1	1	0	1	1	0	1	1	0	1
LIPA	1	0	1	1	0	1	1	0	1
LIPG	0	1	1	0	1	1	0	1	0
LIPT1	1	0	1	1	0	1	1	0	1
LITAF	1	0	1	1	0	1	1	0	1
LIX1L	0	0	1	1	1	1	1	1	1
LMAN1	1	1	1	0	1	1	1	1	1
LMAN2	1	0	1	1	0	1	1	0	1
LMAN2L	1	0	1	1	0	1	1	0	1
LMBR1	1	0	1	1	0	1	1	0	1
LMBR1L	1	0	1	0	0	0	0	0	1
LMBRD2	1	0	1	1	0	1	1	0	1
LMLN	1	0	1	1	0	1	1	0	1
LMNA	0	0	0	0	0	0	0	1	0
LMNB1	1	0	1	1	0	1	1	0	1
LMO2	1	0	1	1	0	1	1	0	1
LMO4	0	0	0	0	0	0	1	0	0
LMOD1	0	1	0	0	1	0	0	1	0
LMTK2	0	1	0	0	1	0	0	1	0
LMX1B	0	1	0	0	1	0	0	1	0
LNPEP	1	0	1	1	0	1	1	0	1
LNX2	1	0	1	1	0	1	1	0	1
LOC113386	1	0	1	1	0	1	0	0	1
LOC123688	0	1	0	0	0	0	0	1	0
LOC124216	0	0	0	0	0	1	0	0	1
LOC124446	1	0	1	1	0	1	1	0	1
LOC124512	1	0	1	1	0	1	1	0	1
LOC128977	0	0	1	1	0	1	1	0	1
LOC129607	0	0	0	0	0	1	0	0	1
LOC130074	0	0	0	0	0	0	0	0	1
LOC130355	1	0	1	1	0	1	1	0	1
LOC130951	0	1	0	0	1	0	0	1	0
LOC134145	1	0	1	1	0	1	1	0	1
LOC143188	0	1	0	0	0	0	0	1	0
LOC143941	0	1	0	0	1	0	0	1	1

LOC144097	0	1	1	0	1	1	0	1	1
LOC148137	1	0	1	1	0	1	0	1	1
LOC150051	0	0	0	0	0	0	0	1	0
LOC150383	1	0	1	0	1	1	0	0	1
LOC152485	0	1	1	1	1	1	1	1	1
LOC153222	1	0	1	1	0	1	1	0	1
LOC153328	0	1	0	0	1	0	0	1	0
LOC153364	0	0	1	0	0	1	1	0	1
LOC153684	1	0	1	1	0	1	1	0	1
LOC155060	1	0	0	0	0	0	0	0	1
LOC158572	0	1	1	0	0	0	0	0	0
LOC196752	1	0	1	1	0	1	1	0	1
LOC198437	0	1	0	0	1	0	0	1	0
LOC201725	1	0	1	1	0	1	1	0	1
LOC203547	0	0	0	0	0	0	1	0	1
LOC205251	0	1	0	0	1	0	0	1	0
LOC220686	0	0	0	0	0	0	0	1	0
LOC222699	1	0	1	1	0	1	1	0	1
LOC257358	0	0	0	1	0	0	0	0	0
LOC283392	0	1	1	0	1	1	0	1	1
LOC283874	0	0	0	0	0	1	1	0	1
LOC283932	0	0	0	0	0	0	0	0	1
LOC284009	1	0	0	0	0	0	0	0	0
LOC284296	0	1	0	0	1	0	0	1	0
LOC285016	0	1	0	0	1	0	0	1	0
LOC285074	1	0	1	0	0	0	0	0	1
LOC285636	1	0	1	1	0	1	1	1	1
LOC286016	1	0	1	1	0	1	0	0	1
LOC286187	1	1	1	1	1	1	1	1	1
LOC339229	0	0	1	1	0	1	0	0	1
LOC339344	0	0	0	0	0	0	0	0	1
LOC339745	1	0	1	1	0	1	0	0	1
LOC342897	0	1	0	0	1	0	0	1	0
LOC374395	1	0	1	1	0	1	1	0	1
LOC374920	1	0	1	1	0	1	0	0	1
LOC387790	1	0	1	1	0	1	1	0	1
LOC387882	0	0	0	0	0	0	0	0	1
LOC388284	0	0	0	1	0	0	0	0	0
LOC388438	1	0	0	0	0	0	0	1	0
LOC388564	1	0	1	1	0	1	1	0	1
LOC388610	0	1	0	0	1	0	0	1	0
LOC388969	0	0	1	0	0	0	0	0	0
LOC389118	0	1	0	0	1	0	0	0	0
LOC389791	1	0	1	0	0	1	0	0	1
LOC389833	0	0	0	0	0	1	0	0	0
LOC390637	1	0	1	1	0	1	1	0	1
LOC391356	1	0	1	1	0	1	1	0	1
LOC399818	1	0	1	1	0	1	1	0	1
LOC399900	1	0	1	1	0	1	1	0	1
LOC400027	1	0	1	1	0	1	1	0	1
LOC400451	0	1	1	0	1	0	0	1	0
LOC400464	1	1	1	1	1	1	1	1	1
LOC400506	1	0	0	1	0	1	0	0	0
LOC400566	1	0	0	0	0	1	0	0	0
LOC400657	1	0	1	1	0	1	0	0	1
LOC400707	0	1	0	0	1	0	0	1	1
LOC400708	0	0	0	0	0	0	1	0	1
LOC401019	1	0	1	1	0	1	1	0	1
LOC401152	1	0	1	1	0	1	1	0	1
LOC401233	1	0	1	1	0	1	1	0	1
LOC401398	1	0	1	1	0	1	1	0	1
LOC401431	1	0	1	1	0	1	1	1	1

LOC401720	0	1	0	0	1	0	1	0	1
LOC439985	0	1	0	0	1	0	0	1	0
LOC440093	0	0	0	0	0	0	0	0	1
LOC440258	1	0	0	0	0	0	0	0	0
LOC440354	1	0	1	1	0	1	1	0	1
LOC440731	1	0	1	1	0	1	1	0	1
LOC440836	1	0	0	1	0	1	1	0	1
LOC440944	1	0	1	1	0	1	1	0	1
LOC441087	1	0	1	1	0	1	1	0	1
LOC441135	0	1	0	0	1	0	0	1	0
LOC441136	1	1	1	1	0	1	0	1	1
LOC441150	1	0	1	1	0	1	1	0	1
LOC441193	1	0	1	1	0	1	1	0	1
LOC441208	1	0	1	0	0	1	0	0	0
LOC441268	0	1	0	0	1	0	0	1	0
LOC441461	0	0	0	0	0	1	1	1	1
LOC441763	0	1	1	0	1	1	0	1	0
LOC442535	1	0	1	1	0	1	1	0	1
LOC442582	1	0	1	1	0	1	1	0	1
LOC442597	0	0	0	0	0	1	1	0	1
LOC493869	0	0	0	1	1	0	1	1	0
LOC51035	1	0	0	1	0	1	1	0	1
LOC51057	1	0	1	1	0	1	1	0	1
LOC51136	1	0	1	1	0	1	1	0	1
LOC51252	0	1	0	0	1	0	0	1	0
LOC54103	1	0	0	1	0	0	0	0	0
LOC552891	1	0	1	0	0	1	0	0	1
LOC55565	1	0	1	0	0	1	0	1	1
LOC56964	0	0	1	0	0	1	1	0	1
LOC606495	1	0	1	1	0	1	1	0	1
LOC63920	1	0	1	1	0	1	1	0	1
LOC63928	0	1	0	0	1	0	0	1	0
LOC642420	0	0	0	0	0	1	1	1	1
LOC643011	1	0	1	1	0	1	1	0	1
LOC643152	0	1	1	0	1	1	0	1	0
LOC643339	1	0	1	1	0	1	1	0	1
LOC643396	1	0	0	1	0	1	0	0	1
LOC643493	1	0	1	1	0	1	1	0	1
LOC644001	0	1	0	0	1	0	0	1	1
LOC644011	1	0	1	1	1	1	1	1	1
LOC644733	1	0	1	1	0	1	0	0	1
LOC644760	1	0	1	1	0	1	1	0	1
LOC644869	1	0	1	1	0	1	1	0	1
LOC644923	0	0	0	1	0	1	0	1	1
LOC645052	1	1	1	1	0	1	1	0	1
LOC645676	0	0	0	0	0	0	0	0	1
LOC646407	1	0	0	0	0	1	0	0	1
LOC646496	0	1	1	0	1	1	0	1	0
LOC646574	1	0	1	1	0	1	1	0	1
LOC646632	1	1	1	1	1	1	1	1	1
LOC646881	0	0	0	0	0	1	1	0	1
LOC646897	1	0	0	1	0	0	0	0	0
LOC647115	0	0	0	1	0	0	0	0	0
LOC647243	1	0	0	1	0	0	0	0	0
LOC653240	0	1	0	0	0	0	0	0	0
LOC653352	1	0	1	1	0	1	1	0	1
LOC653604	1	0	1	1	0	1	1	0	1
LOC653610	1	0	1	1	0	1	1	1	1
LOC653696	0	1	0	0	0	0	0	0	0
LOC81691	1	0	1	0	0	1	1	0	1
LOC90624	1	0	1	1	0	1	1	0	1
LOC90826	1	0	1	1	0	1	1	0	1

LOC90835	1	0	1	1	0	1	1	0	1
LOC92017	0	0	0	1	0	0	0	0	0
LOC92345	0	1	0	0	0	0	0	1	0
LOH11CR2A	1	0	1	1	0	1	1	0	1
LOH12CR1	0	0	0	0	0	0	0	0	1
LONRF3	0	1	0	0	1	0	0	1	0
LOR	0	1	0	0	1	0	0	1	0
LOXL1	0	1	0	0	1	0	0	1	0
LOXL3	0	0	0	0	0	0	0	0	1
LPGAT1	1	0	1	1	0	1	0	0	1
LPHN1	0	0	1	0	1	1	0	1	1
LPIN2	1	0	1	1	0	1	1	0	1
LPL	1	1	1	1	1	1	0	1	1
LPPR4	0	1	0	0	1	0	0	1	0
LPXN	1	0	1	1	0	1	1	0	1
LRAP	1	0	0	1	0	1	1	0	1
LRBA	1	0	1	1	0	1	1	0	1
LRCH4	1	0	1	1	0	1	0	0	1
LRFN4	1	0	0	1	0	1	0	0	1
LRG1	0	0	0	1	0	0	0	0	1
LRIG1	0	1	0	0	1	0	0	1	1
LRIG2	1	0	0	1	0	1	0	0	1
LRMP	1	0	0	0	0	1	1	0	1
LRP1	0	0	0	0	0	0	0	1	1
LRP10	0	0	1	0	0	0	1	0	1
LRP12	0	0	1	0	0	1	0	1	1
LRP2	0	1	1	0	1	1	0	1	1
LRPAP1	0	1	0	0	1	0	0	0	0
LRPPRC	1	0	1	1	0	1	1	0	1
LRRC1	0	1	1	1	1	1	1	1	1
LRRC14	1	0	1	1	0	1	0	0	1
LRRC15	0	1	0	0	1	0	0	1	0
LRRC16	0	1	1	1	0	1	0	0	1
LRRC17	0	0	0	1	0	0	1	0	0
LRRC23	1	0	0	1	0	0	0	0	1
LRRC25	1	0	1	0	0	1	0	0	1
LRRC28	1	0	1	1	0	1	1	1	1
LRRC29	1	0	1	1	0	1	0	0	1
LRRC3	0	0	0	0	0	0	0	1	0
LRRC32	0	1	0	1	1	0	0	1	0
LRRC33	1	0	1	1	0	1	1	0	1
LRRC34	0	1	0	0	0	1	1	0	1
LRRC40	1	0	1	1	0	1	1	0	1
LRRC41	1	0	1	1	0	1	1	0	1
LRRC42	1	0	1	1	0	1	1	0	1
LRRC46	1	0	1	1	0	1	1	0	1
LRRC4C	0	1	0	0	1	1	0	1	0
LRRC51	1	0	1	1	0	1	0	0	1
LRRC57	1	0	1	1	0	1	1	0	1
LRRC6	1	0	1	1	0	1	1	0	1
LRRC61	0	0	0	0	0	1	0	0	1
LRRC8A	1	0	1	1	0	1	1	0	1
LRRC8B	1	0	0	1	0	0	0	0	0
LRRC8C	1	0	0	0	0	1	1	0	1
LRRC8D	1	0	1	1	0	1	1	0	1
LRRCC1	1	0	1	1	0	1	1	0	1
LRRFIP1	1	0	1	1	0	1	0	0	1
LRRFIP2	1	0	1	1	0	1	1	0	1
LRRIQ2	0	0	1	0	0	1	0	0	1
LRRK1	0	1	1	1	1	1	0	1	1
LRRK2	1	0	1	1	0	1	1	0	1
LRSAM1	1	0	1	1	0	1	1	0	1

LSAMP	0	1	0	0	1	0	0	1	0
LSG1	1	0	1	1	0	1	1	0	1
LSM1	1	0	1	1	0	1	1	0	1
LSM10	1	0	0	0	0	0	0	0	1
LSM14A	1	0	1	1	0	1	1	0	1
LSM2	0	0	0	0	0	0	0	0	1
LSM3	1	0	1	1	0	1	1	0	1
LSM4	1	0	1	1	0	1	1	0	1
LSM5	1	0	1	1	0	1	1	0	1
LSM6	1	0	0	1	0	0	0	0	0
LSM7	0	0	0	0	0	1	0	0	1
LSM8	1	0	1	1	0	1	1	0	1
LSMD1	1	0	1	1	0	1	1	0	1
LSP1	0	0	1	0	0	1	0	0	1
LSR	0	0	0	0	1	1	0	1	0
LSS	0	0	1	0	0	0	0	0	1
LST1	1	0	1	1	0	1	1	0	1
LTA4H	0	0	1	0	0	1	1	0	1
LTB	1	0	1	1	0	1	1	0	1
LTB4DH	0	0	0	0	0	0	0	1	0
LTB4R	0	0	0	0	0	1	1	0	1
LTB4R2	0	0	0	0	0	0	0	0	1
LTBP1	0	1	0	0	1	1	0	1	1
LTBP3	0	1	0	0	0	0	0	1	0
LTF	0	1	0	0	1	0	0	1	0
LTK	0	1	0	0	1	0	0	1	1
LTV1	1	0	1	1	0	1	1	0	1
LUC7L2	1	0	1	1	0	1	1	0	1
LUZP1	0	0	1	0	0	0	0	0	0
LXN	0	0	0	0	0	1	1	0	1
LY6E	0	0	0	0	1	1	0	1	0
LY6G5C	0	0	1	1	0	1	0	0	1
LY75	0	0	0	0	0	1	0	0	1
LY86	1	0	0	1	0	1	1	0	1
LY96	1	0	1	1	0	1	1	0	1
LYAR	1	0	1	1	0	1	1	0	1
LYCAT	0	0	1	1	0	1	1	0	1
LYK5	1	0	1	1	0	1	1	0	1
LYN	0	0	0	0	0	1	1	0	1
LYPD1	0	1	0	0	1	0	0	1	0
LYPD3	0	1	0	0	0	0	0	1	0
LYPLA2	0	0	1	1	0	1	0	0	1
LYPLA3	0	0	1	0	0	1	0	0	1
LYPLAL1	0	0	0	0	0	1	0	0	1
LYSMD2	0	1	0	0	1	0	0	1	1
LYSMD3	1	0	1	1	0	1	1	0	1
LYST	1	0	1	1	0	0	0	0	1
LYZ	1	0	0	1	0	1	1	0	1
LZIC	1	0	1	1	0	1	1	0	1
LZTFL1	0	0	0	0	0	1	0	1	0
LZTR1	1	0	1	1	0	1	0	0	1
M6PR	1	0	1	1	0	1	1	0	1
MACF1	0	1	0	0	1	0	0	1	0
MAD1L1	0	0	0	1	0	0	0	0	0
MAD2L1	1	0	1	1	0	1	1	0	1
MAD2L1BP	1	0	1	1	0	1	1	0	1
MAD2L2	0	1	0	0	1	1	0	1	1
MADD	1	0	0	1	0	1	0	0	1
MAEA	1	0	0	1	0	0	0	0	0
MAF	1	1	1	1	0	1	1	1	1
MAF1	1	0	1	1	0	1	1	0	1
MAFB	0	1	0	0	1	1	0	1	1

MAFF	1	0	1	0	0	0	0	0	0
MAFG	1	1	0	0	0	1	0	1	0
MAGEA2	0	1	0	0	1	0	0	0	0
MAGEB1	0	1	0	0	1	0	0	0	0
MAGED1	0	1	1	0	1	1	1	1	1
MAGED2	0	1	0	0	1	0	0	1	0
MAGEF1	0	0	0	0	0	1	1	0	0
MAGEH1	0	1	0	0	1	1	0	1	1
MAGI1	0	1	0	0	1	0	0	1	1
MAGOH	1	0	0	1	0	1	0	0	1
MAK	0	0	0	0	0	0	0	0	0
MALT1	0	0	1	0	0	1	0	0	1
MAML3	1	0	1	1	0	1	1	0	1
MAN1A1	1	0	1	1	0	1	1	0	1
MAN1A2	1	0	1	1	0	1	1	0	1
MAN1B1	1	0	0	0	0	0	0	0	1
MAN2A1	0	0	0	1	0	0	1	0	1
MAN2A2	1	0	1	1	0	1	1	0	1
MAN2B1	1	0	1	1	0	1	0	0	1
MAN2B2	0	1	0	0	1	0	0	1	0
MAN2C1	1	0	1	1	0	1	1	0	1
MANBA	0	1	1	1	0	1	1	1	1
MANEA	1	0	1	1	0	1	1	0	1
MANEAL	0	1	0	0	1	0	0	1	0
MAOA	1	1	1	1	0	1	0	1	1
MAOB	0	1	0	0	1	0	0	1	0
MAP1A	0	1	1	0	1	0	0	1	0
MAP1LC3A	1	1	1	1	1	1	1	1	1
MAP1LC3C	1	0	0	1	0	1	1	0	1
MAP2K1	1	0	1	1	0	1	1	0	1
MAP2K1IP1	1	0	1	1	0	1	0	0	1
MAP2K4	0	1	0	0	1	0	0	1	0
MAP2K5	1	0	1	1	0	1	1	0	1
MAP2K6	1	0	1	1	0	1	1	0	1
MAP2K7	1	0	1	1	0	1	0	0	0
MAP3K10	0	0	0	0	0	1	0	0	1
MAP3K11	1	0	1	1	0	1	1	0	1
MAP3K12	1	0	1	1	0	1	1	0	1
MAP3K3	0	0	0	0	0	1	0	0	0
MAP3K4	1	1	1	1	0	1	1	1	1
MAP3K7	0	1	1	0	1	1	0	1	1
MAP3K7IP1	0	0	0	0	0	0	0	0	1
MAP3K8	0	0	1	1	0	1	0	0	1
MAP3K9	0	1	0	0	1	0	0	1	1
MAP4K1	1	0	1	1	0	1	0	0	1
MAP4K2	1	0	1	0	0	1	0	0	1
MAP4K3	1	0	1	1	0	1	1	0	1
MAP4K5	0	0	0	1	0	1	1	0	1
MAP6	0	1	0	0	1	0	0	1	0
MAP7	0	1	1	0	1	0	0	1	0
MAPBPIP	1	0	1	1	0	1	0	0	1
MAPK1	1	0	1	1	0	1	1	0	1
MAPK14	0	0	0	0	0	0	1	0	0
MAPK3	1	0	0	0	0	0	0	0	0
MAPK7	1	0	1	1	0	1	1	0	1
MAPK8IP1	0	1	0	0	1	0	0	1	0
MAPK8IP2	0	1	1	0	1	1	0	1	1
MAPK8IP3	1	0	0	0	0	0	0	0	0
MAPKAP1	0	0	0	0	0	0	0	0	1
MAPKAPK2	0	1	0	0	0	0	0	0	0
MAPKAPK3	1	0	1	1	0	1	1	0	1
MAPKAPK5	1	0	1	1	0	1	1	0	1

MAPKBP1	1	0	1	1	0	1	1	0	1
MAPRE2	0	0	1	1	0	1	1	0	1
MAPRE3	0	1	0	0	0	1	0	1	1
MARCKS	1	0	1	1	0	1	1	0	1
MARCO	0	0	0	0	0	1	1	0	1
MARK2	1	0	1	1	0	1	1	0	1
MARS	1	0	1	1	0	1	1	0	1
MARS2	1	0	1	1	0	0	0	0	1
MARVELD1	0	0	1	0	0	1	0	0	0
MARVELD2	1	1	0	0	1	0	0	1	0
MAS1	0	0	0	0	0	0	0	1	0
MASK-BP3	1	0	1	1	0	1	1	0	1
MAST2	0	0	0	1	0	0	0	0	1
MASTL	1	0	1	1	0	1	1	0	1
MAT2A	1	0	1	1	0	1	1	0	1
MAT2B	1	0	1	1	0	1	1	0	1
MATK	0	1	0	0	0	0	0	1	0
MATN4	0	1	0	0	1	0	0	1	0
MATR3	1	0	0	1	0	1	1	0	1
MAX	0	0	1	1	0	1	1	0	1
MAZ	0	0	1	1	0	1	0	0	1
MBD1	1	0	1	1	0	1	1	0	1
MBD4	1	0	1	1	0	1	1	0	1
MBD6	1	0	1	1	0	1	0	0	1
MBIP	0	1	0	0	1	1	0	1	1
MBNL1	1	0	1	1	0	1	1	0	1
MBNL2	1	0	1	1	0	1	1	0	1
MBNL3	0	1	0	0	1	0	1	1	0
MBP	0	0	0	0	0	1	1	0	1
MBTPS1	1	0	1	1	0	1	1	0	1
MBTPS2	1	1	0	1	1	1	1	1	1
MC1R	0	1	0	0	0	1	0	1	0
MCART1	0	0	0	1	0	0	0	0	1
MCART6	1	1	1	1	1	1	0	0	1
MCCC1	0	0	0	0	0	0	0	0	1
MCEE	1	0	1	1	0	1	1	0	1
MCFD2	0	0	0	0	0	1	0	0	1
MCHR1	0	1	0	0	1	0	0	1	0
MCL1	1	0	1	1	0	1	1	0	1
MCM10	0	0	0	0	0	0	0	0	1
MCM3	1	0	1	1	0	1	1	0	1
MCM3AP	1	0	1	1	0	1	1	0	1
MCM4	1	0	1	1	0	1	0	0	1
MCM5	0	0	0	0	0	1	0	0	1
MCM6	0	0	0	1	0	0	0	0	1
MCM7	1	0	1	1	0	1	0	0	1
MCOLN1	1	0	0	0	0	1	0	0	0
MCOLN2	1	1	1	0	1	1	1	1	1
MCOLN3	0	1	0	0	1	0	0	1	0
MCRS1	1	0	1	1	0	1	1	0	1
MCTP1	1	1	1	1	1	1	1	1	1
MCTP2	1	0	1	1	0	1	1	0	0
MCTS1	0	0	0	1	1	1	0	0	1
MDC1	1	0	1	1	0	1	0	0	1
MDGA1	0	1	0	0	1	0	0	1	0
MDH1	1	0	1	1	0	1	1	0	1
MDH2	1	0	1	1	0	1	1	0	1
MDM1	1	0	1	1	0	1	0	0	1
MDM2	1	0	1	1	0	1	1	0	0
MDM4	1	0	0	1	0	1	1	0	1
MDN1	0	0	0	1	0	1	0	0	1
MDP-1	1	0	1	1	0	1	1	0	1

MDS1	0	1	1	0	1	1	0	1	1
ME1	0	0	0	0	0	0	0	1	0
ME2	1	0	1	1	0	1	1	0	1
ME3	0	1	0	0	0	0	0	1	0
MEA1	1	0	1	1	0	1	0	0	1
MECP2	0	1	0	0	1	1	0	1	1
MECR	1	1	0	1	1	1	0	1	1
MED10	0	0	0	0	0	1	0	0	1
MED11	0	0	0	0	0	0	0	0	1
MED12	0	1	0	1	1	1	0	0	0
MED18	0	0	0	1	0	1	1	0	1
MED19	1	0	1	1	0	1	1	0	1
MED25	1	0	1	1	0	1	1	0	1
MED28	1	0	1	1	0	1	1	0	1
MED31	1	0	1	1	0	1	0	1	1
MED4	1	0	1	1	0	1	1	0	1
MED6	1	0	1	1	0	1	1	1	1
MED8	1	0	1	1	0	1	1	0	1
MED9	0	0	0	1	0	1	0	0	0
MEF2B	1	0	1	1	0	1	1	0	1
MEF2C	1	0	1	1	0	1	1	0	1
MEF2D	1	0	1	0	0	0	1	0	1
MEFV	0	0	0	0	0	1	1	0	1
MEIS3	0	1	0	0	1	0	0	1	0
MEOX1	0	1	0	0	0	0	0	0	0
MEP1A	0	1	0	0	0	0	0	0	0
MERTK	0	1	0	0	0	1	0	0	1
MESDC1	1	0	1	1	0	1	1	0	1
MESDC2	1	0	1	1	0	1	1	0	1
MESP1	0	1	0	0	1	0	0	0	0
MEST	0	1	0	0	1	0	0	1	1
MET	0	1	1	0	1	1	0	1	1
METAP2	1	0	1	1	0	1	1	0	1
METRN	0	1	0	0	1	0	0	1	0
METRNL	0	1	0	0	1	0	0	1	0
METT10D	1	0	1	1	0	1	0	0	1
METT5D1	1	0	1	1	0	1	1	0	1
METTL1	1	0	1	1	0	1	1	0	1
METTL2A	1	0	1	1	0	1	1	0	1
METTL2B	1	0	1	1	0	1	1	0	1
METTL3	0	0	0	0	0	1	0	0	0
METTL4	1	0	1	1	0	1	1	0	1
METTL5	1	0	1	1	0	1	1	0	1
METTL6	1	0	1	1	0	1	1	0	1
METTL7A	1	0	1	1	0	1	1	0	1
MFAP1	1	0	1	1	0	1	1	0	1
MFAP3	1	0	1	1	0	1	1	0	1
MFGE8	0	0	0	0	0	1	0	0	0
MFNG	0	0	0	1	0	0	0	0	0
MFRP	0	1	0	0	1	0	0	1	0
MFSD1	0	0	1	1	0	0	0	0	1
MFSD2	0	0	0	1	1	0	0	0	0
MFSD3	0	0	0	0	0	1	0	0	1
MFSD5	0	0	0	1	0	1	0	0	1
MFSD7	0	0	0	1	0	0	1	0	1
MGAT1	1	0	0	1	0	1	1	0	1
MGAT2	1	0	1	1	0	1	1	0	1
MGAT4A	1	0	0	1	0	0	0	0	0
MGAT4B	1	0	1	0	0	1	0	0	1
MGC11102	1	0	1	1	0	1	1	0	1
MGC12966	0	0	0	0	0	0	1	0	0
MGC13057	0	1	0	0	1	1	0	1	0

MGC13379	0	0	0	0	0	1	0	0	1
MGC14327	1	0	1	1	0	1	1	0	1
MGC14376	1	0	1	1	0	1	0	0	1
MGC15523	0	0	0	0	0	0	0	0	1
MGC16169	1	0	1	1	0	1	1	0	1
MGC16824	1	0	1	1	0	1	1	0	1
MGC19604	0	0	0	1	0	1	0	0	1
MGC20983	0	0	1	1	0	1	0	0	1
MGC24039	0	1	1	0	1	1	1	1	1
MGC2752	0	0	1	0	0	1	0	0	1
MGC3207	1	0	0	0	0	1	0	0	1
MGC33556	1	0	1	1	0	1	0	0	1
MGC35361	1	0	1	1	0	1	0	0	1
MGC35440	0	1	0	0	0	0	0	1	0
MGC39900	0	1	0	0	1	0	1	1	0
MGC40499	1	0	1	1	0	1	1	0	1
MGC40574	0	0	0	1	0	1	1	0	1
MGC4093	1	0	1	1	0	1	1	0	1
MGC4172	0	0	0	1	0	0	0	0	0
MGC42105	0	1	0	0	1	0	0	1	1
MGC45491	0	1	0	0	1	0	0	1	0
MGC4677	1	0	1	0	0	1	1	0	1
MGC52000	0	1	1	1	1	1	1	1	1
MGC52110	1	0	1	1	0	1	1	0	1
MGC61571	0	0	1	1	0	1	0	0	1
MGC70857	0	0	0	0	1	0	0	0	0
MGC71993	1	0	1	1	0	1	1	0	1
MGC72104	0	0	0	0	0	0	0	0	1
MGEA5	0	0	0	1	0	0	0	0	1
MGLL	0	0	0	0	0	1	0	0	0
MGMT	0	1	0	0	0	0	0	0	0
MGRN1	1	0	0	0	0	0	0	0	1
MGST1	1	0	1	1	0	1	1	0	1
MGST2	1	0	1	1	0	1	1	0	1
MGST3	1	0	1	0	0	1	1	0	1
MIB1	1	0	1	1	0	1	1	0	1
MICA	1	0	1	1	0	1	0	0	1
MICAL2	0	0	1	0	0	1	0	0	1
MICB	1	0	1	1	0	1	1	0	1
MID1IP1	1	1	1	1	1	1	1	0	1
MID2	1	1	0	0	0	0	0	1	0
MIDN	1	0	0	0	0	0	0	0	0
MIER1	0	0	1	1	0	1	0	0	1
MIF	0	0	1	0	0	1	0	0	1
MINA	1	0	1	1	0	1	1	0	1
MINK1	0	1	0	0	0	0	0	0	0
MINPP1	1	0	1	1	0	1	0	1	1
MIPEP	1	0	1	0	0	1	0	0	0
MIR16	0	0	0	0	0	0	0	0	1
MIS12	1	0	1	1	0	1	1	0	1
MITF	1	0	1	1	0	1	1	0	1
MIZF	1	0	1	1	0	1	1	0	1
MKI67	0	0	0	0	0	0	0	0	1
MKI67IP	1	0	1	1	0	1	1	0	1
MKKS	1	0	1	1	0	1	1	0	1
MKL1	1	0	1	1	0	1	1	0	1
MKL2	0	1	0	0	1	0	0	1	0
MKLN1	1	0	1	1	0	1	1	0	1
MKNK1	1	0	1	1	0	1	1	0	1
MKRN1	0	0	0	0	0	1	0	0	1
MKRN2	0	0	1	0	0	1	1	1	1
MKS1	0	0	1	0	0	0	0	0	1

MLC1	1	1	1	1	0	1	1	0	1
MLF1IP	1	0	1	1	0	0	0	0	0
MLF2	1	0	0	1	0	1	0	0	0
MLH1	1	0	1	1	0	1	1	0	1
MLKL	1	0	0	1	0	1	1	0	1
MLL	0	1	1	1	0	1	1	0	1
MLL3	1	1	1	1	1	1	1	1	1
MLL5	1	0	1	1	0	1	1	0	1
MLLT10	1	0	1	1	0	1	1	0	1
MLLT11	1	0	1	1	0	1	1	0	1
MLLT3	1	1	1	1	0	1	1	1	1
MLLT4	0	1	1	0	1	1	0	1	1
MLLT6	1	0	0	1	0	1	1	0	1
MLPH	0	1	0	0	1	0	0	1	0
MLSTD1	1	0	1	0	0	0	0	0	0
MLSTD2	0	0	1	1	0	1	1	0	1
MLXIPL	0	1	0	0	1	0	0	1	0
MLYCD	0	0	0	0	0	0	0	1	0
MMAB	1	0	1	1	0	1	1	0	1
MMD	0	1	0	0	1	0	0	1	0
MME	1	1	1	0	1	1	1	1	1
MMP1	0	1	0	0	0	0	0	0	0
MMP10	0	1	0	0	1	0	0	1	0
MMP12	0	1	0	0	0	0	0	0	0
MMP13	0	1	0	0	0	0	0	0	0
MMP14	0	0	0	0	0	1	1	1	1
MMP15	0	1	0	0	1	0	0	1	0
MMP19	0	0	0	1	0	0	0	0	0
MMP25	0	0	0	0	0	0	1	1	1
MMP7	0	0	0	0	0	0	1	0	0
MMP9	0	0	0	0	0	0	0	1	0
MMPL1	0	0	0	0	0	0	1	1	1
MMS19L	1	0	1	1	0	1	0	0	1
MN1	0	1	1	0	1	1	0	1	1
MNAT1	1	1	1	1	1	1	1	1	1
MND1	0	1	0	0	1	0	0	1	0
MNDA	1	0	0	1	0	1	1	0	1
MNS1	1	0	1	1	0	1	1	0	1
MNT	1	0	1	1	0	1	1	0	1
MOAP1	1	0	1	1	0	1	0	0	0
MOBKL1A	1	0	1	1	0	1	1	0	1
MOBKL2A	0	0	1	0	0	0	0	0	0
MOBKL2B	1	0	1	1	0	1	0	0	0
MOBKL2C	0	0	1	1	0	1	1	0	1
MOCOS	0	1	1	0	1	1	0	1	1
MOCS2	1	0	1	0	0	1	1	0	1
MOCS3	1	0	1	1	0	1	1	0	1
MOGAT1	1	0	1	1	0	1	1	0	1
MON1B	1	0	1	1	0	1	1	0	1
MON2	1	0	1	1	0	1	1	0	1
MORC2	1	0	1	1	0	1	1	0	1
MORC4	0	1	0	0	0	0	0	0	0
MORF4L1	0	0	0	0	0	1	0	0	1
MORF4L2	1	1	1	1	0	1	0	1	1
MORG1	1	0	1	1	0	1	0	0	1
MORN2	1	0	1	1	0	1	1	0	1
MOS	0	1	1	0	1	0	0	1	0
MOSC1	0	0	0	0	0	0	1	0	1
MOSC2	0	1	1	1	1	1	0	1	1
MOSPD2	1	1	1	1	0	1	1	0	1
MOSPD3	1	0	1	1	0	1	1	0	1
MOV10	1	0	0	0	0	1	1	0	1

MPDU1	1	0	1	1	0	1	1	0	1
MPHOSPH1	1	0	1	1	0	1	1	0	1
MPHOSPH10	1	0	1	1	0	1	1	0	1
MPHOSPH6	1	1	1	1	1	0	0	1	1
MPI	1	0	1	1	0	1	1	0	1
MPO	0	0	0	0	0	0	1	0	0
MPP1	0	0	0	0	0	0	1	0	1
MPP5	1	1	1	1	0	1	1	1	1
MPP6	0	1	1	1	1	1	1	1	1
MPPE1	1	0	1	1	0	1	1	0	1
MPPED2	0	1	0	0	1	0	0	1	0
MPST	1	0	1	1	0	1	1	0	1
MPV17	1	0	1	1	0	1	1	0	1
MPZL1	1	0	1	1	0	1	1	0	1
MR1	1	0	1	1	0	1	1	0	1
MRAS	0	0	0	1	0	0	0	0	0
MRC1	0	0	1	1	0	1	0	0	0
MRC2	0	0	0	0	0	0	0	1	0
MRCL3	1	0	1	1	0	1	1	0	1
MRE11A	1	0	1	1	0	1	1	0	1
MRFAP1	1	0	1	1	0	1	0	0	1
MRFAP1L1	1	0	1	1	0	1	1	0	1
MRGPRF	0	1	0	0	0	0	0	1	0
MRLC2	1	0	1	1	0	1	1	0	1
MRP63	1	0	1	1	0	1	1	0	1
MRPL1	1	0	1	1	0	1	1	0	1
MRPL11	1	0	1	0	0	1	0	0	1
MRPL12	0	0	0	1	0	0	0	0	0
MRPL13	1	0	1	1	0	1	1	0	1
MRPL14	1	0	1	1	0	1	1	0	1
MRPL15	1	0	1	1	0	1	1	0	1
MRPL16	1	0	1	1	0	1	1	0	1
MRPL17	1	0	1	1	0	1	1	0	1
MRPL18	1	0	1	1	0	1	1	0	1
MRPL19	0	0	0	1	0	1	1	0	1
MRPL2	1	0	1	1	0	1	1	0	1
MRPL20	1	0	1	1	0	1	1	0	1
MRPL21	1	0	1	1	0	1	1	0	1
MRPL22	1	0	1	1	0	1	1	0	1
MRPL24	1	0	1	1	0	1	1	0	1
MRPL27	1	0	1	1	0	1	1	0	1
MRPL3	1	0	1	0	0	1	0	0	1
MRPL30	1	1	1	1	1	1	1	1	1
MRPL32	1	0	1	1	0	1	1	0	1
MRPL33	1	0	1	1	0	1	1	0	1
MRPL35	1	0	1	1	0	1	1	0	1
MRPL36	1	0	1	1	0	1	1	0	1
MRPL37	1	0	1	1	0	1	1	0	1
MRPL39	1	0	1	1	0	1	1	0	1
MRPL40	1	0	1	1	0	1	1	0	1
MRPL41	1	0	1	1	0	1	0	0	0
MRPL42	0	0	0	1	0	1	1	0	1
MRPL43	1	0	1	0	0	1	0	0	1
MRPL44	1	0	1	1	0	1	1	0	1
MRPL46	1	0	1	1	0	1	1	0	1
MRPL47	1	0	1	1	0	1	1	0	1
MRPL48	1	0	1	1	0	1	0	0	1
MRPL49	1	0	1	1	0	1	1	0	1
MRPL50	1	0	1	1	0	1	1	0	1
MRPL51	1	0	1	1	0	1	1	0	1
MRPL52	0	0	0	1	0	1	0	0	1
MRPL53	1	0	1	1	0	1	0	0	1

MRPL54	1	0	1	1	0	1	1	0	1
MRPL55	0	0	0	1	0	1	0	0	0
MRPL9	1	0	1	1	0	1	1	0	1
MRPS10	1	0	1	1	0	1	1	0	1
MRPS11	1	0	1	1	0	1	1	0	1
MRPS12	1	0	1	1	0	1	1	0	1
MRPS14	1	0	1	1	0	1	1	0	1
MRPS15	1	0	1	1	0	1	0	0	1
MRPS16	1	0	1	1	0	1	1	0	1
MRPS18A	1	1	1	1	1	1	1	1	1
MRPS18B	1	0	1	1	0	1	1	0	1
MRPS18C	1	0	1	1	0	1	1	0	1
MRPS2	0	0	1	0	0	1	0	0	1
MRPS21	1	0	1	1	0	1	1	0	1
MRPS22	1	0	1	1	0	1	1	0	1
MRPS23	1	0	1	1	0	1	1	0	1
MRPS24	0	0	0	0	0	0	0	0	1
MRPS25	1	0	0	1	0	1	1	0	1
MRPS26	0	0	0	0	0	0	0	0	1
MRPS27	1	0	0	0	0	1	0	0	1
MRPS28	1	0	1	1	0	1	1	0	1
MRPS30	1	0	1	1	0	1	1	0	1
MRPS31	1	0	1	1	0	1	1	0	1
MRPS33	1	1	1	1	1	1	1	1	1
MRPS34	1	0	0	0	0	0	0	0	1
MRPS35	1	0	1	1	0	1	1	0	1
MRPS36	1	0	0	1	0	1	0	0	1
MRPS5	1	0	1	1	0	1	0	0	1
MRPS7	1	0	1	1	0	1	1	0	1
MRPS9	1	0	1	1	0	1	1	1	1
MRRF	1	0	1	1	0	1	1	0	1
MRS2L	0	1	0	0	0	0	0	1	0
MRVI1	0	1	0	0	1	0	0	0	0
MS4A3	0	0	0	0	0	0	1	0	1
MS4A6A	1	0	1	1	0	1	1	0	1
MS4A7	1	0	0	1	0	0	1	0	1
MSC	1	0	1	1	0	1	1	1	1
MSH2	0	0	0	0	0	1	0	0	1
MSH3	1	0	1	1	0	1	1	0	1
MSH5	1	0	1	1	0	1	1	0	1
MSH6	0	0	0	0	0	0	1	0	1
MSI2	1	1	1	1	1	1	1	1	1
MSL2L1	1	0	1	1	0	1	1	0	1
MSL3L1	0	1	1	0	0	0	0	0	1
MSN	0	1	0	0	1	0	0	1	0
MSR1	1	0	0	1	0	1	0	0	0
MSRA	1	0	1	1	0	1	0	0	1
MST1	1	1	1	1	1	1	1	1	1
MST150	1	0	1	1	0	1	1	0	1
MSTO1	0	0	1	0	0	1	0	0	1
MT1A	0	1	0	0	1	1	0	1	0
MT1B	0	1	0	0	1	0	0	1	0
MT1F	0	1	0	0	0	0	0	0	1
MT1G	0	1	1	0	1	1	0	1	0
MT1X	0	1	0	0	1	0	1	1	1
MT2A	0	0	1	0	0	1	0	0	1
MTA1	0	1	0	0	1	0	0	0	0
MTA2	0	0	0	0	0	0	1	0	1
MTA3	0	1	0	0	1	0	0	1	0
MTAP	1	1	1	1	0	1	0	1	1
MTCH1	1	0	1	1	0	1	1	0	1
MTCP1	1	0	1	1	0	1	1	0	1

MTDH	1	0	1	1	0	1	1	0	1
MTERFD1	1	0	1	1	0	1	1	0	1
MTF1	1	0	1	1	0	1	1	0	1
MTF2	1	0	1	1	0	1	1	0	1
MTFMT	0	1	0	0	0	0	0	1	0
MTFR1	1	1	1	1	1	1	1	1	1
MTHFD1	1	0	1	1	0	1	1	0	1
MTHFR	1	0	1	1	0	1	1	0	1
MTHFS	0	0	0	1	0	1	1	0	1
MTHFSD	1	1	1	1	0	1	1	1	1
MTIF2	1	0	1	1	0	1	1	0	1
MTIF3	1	0	1	1	0	1	1	0	1
MTM1	0	1	0	0	0	0	0	0	0
MTMR1	0	1	0	0	0	1	1	0	0
MTMR10	1	0	0	1	0	1	0	0	1
MTMR2	0	1	1	1	0	1	1	1	1
MTMR4	1	0	1	1	0	0	1	0	1
MTMR9	0	1	1	0	1	1	0	1	1
MTO1	1	0	0	1	0	1	1	0	1
MTP18	1	0	1	1	0	1	1	0	1
MTPN	0	0	0	0	0	0	0	0	1
MTR	1	0	0	1	0	1	1	0	1
MTRF1	0	0	1	1	0	1	1	0	1
MTRF1L	1	0	1	1	0	1	1	0	1
MTRR	1	0	1	1	0	1	1	0	1
MTSS1	0	1	1	1	1	1	0	1	1
MTX1	1	0	1	1	0	1	0	0	1
MTX3	0	1	1	0	0	1	0	0	1
MUC1	0	0	0	0	0	0	0	1	1
MUM1	1	0	0	1	0	1	1	0	1
MUS81	1	0	1	1	0	1	1	0	1
MUT	1	0	1	1	0	1	1	0	1
MUTYH	1	0	1	1	0	1	1	0	1
MVK	1	0	1	1	0	1	1	0	1
MVP	1	1	1	1	1	1	1	1	1
MX1	0	0	1	0	0	1	0	0	1
MX2	0	0	0	0	0	1	1	0	1
MXD1	1	0	0	0	0	0	1	0	1
MXI1	0	1	1	1	1	1	1	1	1
MXRA7	0	0	0	0	0	0	1	1	1
MYADM	0	0	1	0	0	1	0	0	0
MYB	0	0	0	0	0	1	0	0	0
MYBBP1A	0	0	0	1	0	1	0	0	1
MYC	1	0	1	1	0	1	1	0	1
MYCBP	1	0	1	1	0	1	1	0	1
MYCBP2	1	0	1	1	0	1	1	0	1
MYCN	0	1	0	0	1	0	0	1	0
MYD88	1	0	1	1	0	1	0	0	1
MYEF2	0	1	0	0	1	0	0	1	1
MYEOV	0	1	0	0	1	0	0	0	0
MYEOV2	0	1	0	0	1	0	0	1	0
MYF5	0	1	1	0	1	0	0	1	0
MYH11	0	1	1	0	1	1	0	1	1
MYH14	0	1	0	0	1	0	0	1	0
MYH9	1	0	1	1	0	1	1	0	1
MYL6	1	0	1	1	0	1	1	0	1
MYL6B	1	0	1	1	0	1	1	0	1
MYL9	1	0	0	0	0	0	0	0	0
MYLIP	0	0	0	0	0	1	1	0	0
MYLK	0	1	0	0	1	0	0	1	1
MYNN	1	0	1	1	0	1	1	0	1
MYO10	0	1	1	0	1	1	0	1	1

MYO18A	1	0	1	0	0	0	0	0	0
MYO1B	0	1	0	0	1	0	0	1	0
MYO1C	0	0	1	0	0	0	0	1	0
MYO1D	0	0	0	0	0	0	0	1	0
MYO1E	1	0	1	1	0	1	1	1	1
MYO1F	0	0	1	0	0	1	0	0	1
MYO1G	0	0	0	1	0	1	1	0	1
MYO5A	0	1	0	0	1	1	0	1	1
MYO6	1	1	1	1	0	1	0	1	1
MYO7A	1	1	0	0	0	0	0	1	0
MYO9A	1	0	1	1	0	1	1	0	1
MYO9B	1	0	1	1	0	1	1	0	1
MYOD1	0	1	0	0	1	0	0	1	0
MYOHD1	1	0	1	1	0	1	1	0	1
MYOM2	0	1	1	0	1	0	0	1	0
MYOZ1	0	0	0	1	0	0	0	0	0
MYST1	0	0	0	0	0	1	0	0	0
MYST2	1	0	1	1	0	1	1	0	1
MYST3	0	0	0	0	0	0	0	0	1
MYST4	1	0	0	1	0	1	1	0	0
MAGMAS	0	0	1	0	0	0	0	0	1
N-PAC	1	0	1	1	0	1	1	0	1
N4BP1	0	0	0	0	0	0	1	0	0
NAALADL1	0	1	0	0	0	0	0	0	0
NAB1	0	0	1	0	0	1	0	0	1
NAB2	0	0	0	0	0	0	0	0	1
NACA	1	0	1	1	0	1	1	0	1
NADK	0	0	0	0	0	1	1	0	1
NADSYN1	1	0	1	1	0	1	1	0	1
NAG	1	1	1	1	0	1	1	0	1
NAGA	1	0	1	1	0	1	0	0	1
NAGK	1	0	1	1	0	1	1	0	1
NAGLU	1	0	0	0	0	0	0	0	0
NAGPA	1	0	1	1	0	1	1	0	1
NAGS	0	1	0	0	1	0	0	1	0
NANOG	1	0	1	0	0	1	1	0	0
NANP	1	0	1	1	0	1	0	0	0
NANS	1	0	0	0	0	0	0	0	0
NAP1L1	1	0	0	1	0	1	1	0	1
NAP1L2	0	1	0	0	1	0	0	1	1
NAP1L5	0	0	0	0	0	1	0	0	1
NAPA	1	0	0	0	0	0	1	0	1
NAPE-PLD	0	0	0	0	0	0	0	0	1
NAPG	1	0	1	1	0	1	1	0	1
NARF	1	0	1	1	0	1	1	0	1
NARG1	1	0	1	1	0	1	1	0	1
NARG1L	0	0	0	1	0	1	0	0	1
NARG2	1	0	1	1	0	1	1	0	1
NARS2	1	0	1	1	0	1	1	0	1
NASP	1	0	1	1	0	1	0	0	1
NAT1	1	0	1	1	0	1	1	0	1
NAT10	1	0	1	1	0	1	1	0	1
NAT5	1	0	1	1	0	1	1	0	1
NAT6	0	0	0	1	0	0	1	0	1
NAT9	1	0	1	1	0	1	1	0	1
NAV1	0	1	0	0	1	0	0	1	0
NAV2	0	1	0	0	0	0	0	0	1
NBEA	0	1	0	0	1	0	0	1	1
NBL1	0	1	1	0	1	0	0	1	1
NBPF3	0	1	0	0	1	0	0	1	0
NCAM1	0	1	0	0	1	0	0	1	0
NCBP1	1	0	1	1	0	1	1	0	1

NCBP2	1	0	1	1	0	1	1	0	1
NCDN	1	0	1	1	0	1	1	0	1
NCF1	0	0	0	0	0	1	0	0	1
NCF2	1	0	1	1	0	1	1	0	1
NCF4	0	0	0	1	0	0	0	0	0
NCK1	0	0	1	0	0	1	1	0	1
NCK2	0	1	0	0	0	1	0	0	1
NCKAP1	0	1	0	0	1	0	0	1	0
NCKAP1L	1	0	1	1	0	1	0	0	1
NCKIPSD	0	0	0	0	0	0	0	0	1
NCL	0	0	0	1	0	1	0	0	0
NCLN	0	0	0	0	0	1	0	0	1
NCOA2	0	0	0	0	0	0	1	0	1
NCOA3	1	0	1	1	0	1	1	0	1
NCOA4	1	0	1	1	0	0	1	0	0
NCOA5	1	0	1	1	0	1	1	0	1
NCOA6	0	0	0	1	0	1	0	0	1
NCOA7	1	0	1	1	0	1	1	0	1
NCOR1	1	0	1	1	0	1	1	0	1
NCR2	0	1	0	0	0	0	0	0	0
NCSTN	1	0	1	1	0	1	1	0	1
NDE1	1	0	1	1	0	1	1	0	1
NDFIP2	0	1	0	0	1	0	0	1	1
NDN	0	1	0	0	0	0	0	1	0
NDNL2	1	0	1	0	0	1	1	0	1
NDP	0	1	0	1	1	0	0	1	1
NDRG1	0	0	0	0	0	0	0	0	1
NDRG2	0	1	0	0	0	0	0	1	0
NDRG3	1	0	0	0	0	1	0	0	1
NDRG4	0	1	0	0	1	0	0	1	0
NDUFA1	1	1	1	1	0	1	0	1	1
NDUFA10	0	1	0	0	0	1	0	1	0
NDUFA11	1	0	0	1	0	1	1	0	1
NDUFA12	1	0	0	1	0	1	0	0	1
NDUFA13	1	0	1	1	0	1	1	0	1
NDUFA2	1	0	1	1	0	1	1	0	1
NDUFA3	0	0	0	1	0	1	0	0	1
NDUFA4	1	0	1	1	0	1	1	0	1
NDUFA5	1	0	1	1	0	1	1	0	1
NDUFA6	1	0	0	1	0	1	0	0	1
NDUFA7	1	0	1	1	0	1	1	0	1
NDUFA8	1	1	1	1	1	1	1	0	1
NDUFA9	1	0	1	1	0	1	1	1	1
NDUFAF1	1	0	1	1	0	1	1	0	1
NDUFB1	1	0	1	1	0	1	1	0	1
NDUFB11	1	1	1	1	1	1	0	1	1
NDUFB3	1	0	1	1	0	1	1	0	1
NDUFB5	1	0	1	1	0	1	1	0	1
NDUFB6	1	0	1	1	0	1	1	0	1
NDUFB8	1	0	1	1	0	1	1	0	1
NDUFB9	1	0	1	1	0	1	1	0	1
NDUFC1	1	0	1	1	0	1	1	0	1
NDUFC2	1	0	1	1	0	1	1	0	1
NDUFS1	1	0	1	1	0	1	1	0	1
NDUFS3	1	0	1	1	0	1	1	0	1
NDUFS4	1	0	1	0	0	1	1	0	1
NDUFS5	1	0	1	1	0	1	1	0	1
NDUFS6	1	0	1	1	0	1	1	0	1
NDUFS8	1	0	1	1	0	1	1	0	1
NDUFV1	1	0	0	0	0	1	1	0	1
NDUFV2	1	0	1	1	0	1	1	0	1
NDUFV3	1	0	1	1	0	1	1	0	1

NECAP1	0	0	0	1	0	1	0	0	1
NECAP2	1	0	1	1	0	1	0	0	1
NEDD1	0	0	1	1	0	1	1	1	1
NEDD4	0	1	0	1	0	1	1	1	1
NEDD4L	0	0	1	0	0	1	1	1	1
NEDD8	1	0	1	1	0	1	1	0	1
NEDD9	1	0	1	1	0	1	1	0	1
NEFH	0	1	0	0	1	0	0	1	0
NEIL1	1	1	1	0	1	1	1	1	1
NEIL3	0	0	0	1	0	1	1	0	1
NEK1	1	0	1	1	0	1	1	0	1
NEK11	1	0	1	1	0	1	1	0	1
NEK3	1	0	1	0	0	1	1	1	1
NEK4	0	0	1	1	0	1	1	0	1
NEK8	0	0	0	1	0	1	0	0	1
NEK9	0	0	0	1	0	0	0	0	0
NEO1	0	1	0	0	1	0	0	1	0
NES	0	1	0	0	1	0	0	1	0
NET1	1	0	1	1	0	0	1	0	1
NETO2	0	1	0	0	1	0	0	1	1
NEU1	1	0	1	1	0	1	1	0	1
NEU3	1	0	0	0	0	0	1	0	1
NEURL	0	1	0	0	1	0	0	1	0
NEUROG3	0	1	0	0	1	0	0	1	1
NF1	0	0	0	1	0	0	1	0	1
NFAT5	1	0	1	1	0	1	1	0	1
NFATC1	0	1	1	0	1	1	0	1	1
NFATC3	0	0	0	1	0	1	0	0	0
NFE2	0	0	0	0	0	0	1	0	1
NFE2L1	0	0	1	1	0	1	0	0	1
NFE2L2	1	0	0	1	0	1	1	0	1
NFE2L3	0	0	0	0	0	1	0	0	0
NFIA	1	1	0	1	0	1	1	1	1
NFIC	0	0	0	0	0	0	0	1	0
NFIL3	1	0	0	0	0	1	1	1	1
NFIX	0	0	0	0	1	0	0	0	0
NFKB1	1	0	0	1	0	0	1	0	1
NFKB2	0	0	0	0	0	0	0	0	1
NFKBIA	1	0	1	1	0	1	1	0	1
NFKBIB	1	0	1	1	0	1	1	0	1
NFKBIL1	1	0	1	1	0	1	0	0	1
NFKBIZ	1	0	1	1	0	1	1	0	1
NFRKB	1	0	1	1	0	1	1	0	1
NFS1	1	0	1	1	0	1	1	0	1
NFX1	0	0	0	0	0	1	0	0	1
NFXL1	1	0	1	1	0	1	1	0	1
NFYA	1	0	1	1	0	1	1	0	1
NFYC	0	0	0	1	0	1	1	0	1
NGFRAP1	0	1	0	0	1	0	0	0	0
NGFRAP1L1	0	1	0	0	1	0	0	1	0
NGLY1	1	0	1	1	0	1	1	0	1
NGRN	1	0	1	0	0	1	0	0	1
NHEJ1	1	0	1	0	0	1	1	0	1
NHLRC1	1	0	0	0	0	0	0	0	0
NHLRC2	1	0	1	1	0	1	1	0	1
NHN1	1	0	1	1	0	1	1	0	1
NHP2L1	1	0	0	1	0	1	1	0	1
NIBP	0	0	0	0	0	0	0	0	1
NICN1	1	0	0	0	0	1	0	0	1
NIF3L1	1	0	1	1	0	1	1	0	1
NIN	1	0	1	1	0	1	1	0	1
NIP30	1	0	1	1	0	1	1	0	1

NIP7	1	0	1	1	0	1	1	0	1
NIPA2	0	0	0	0	0	0	1	0	1
NIPBL	1	0	1	1	0	1	1	0	1
NIPSNAP3A	1	0	1	1	0	1	1	0	1
NISCH	0	0	0	0	0	0	0	0	1
NIT1	1	0	1	1	0	1	1	0	1
NIT2	1	0	1	0	0	1	1	0	1
NKAP	0	0	0	1	1	1	0	0	1
NKIRAS1	1	0	1	1	0	1	1	0	1
NKIRAS2	1	0	1	1	0	1	1	0	1
NKPD1	0	1	0	0	1	0	0	1	0
NKRF	0	1	1	0	1	1	1	1	1
NKTR	1	0	1	1	0	1	1	0	1
NKX3-1	0	1	0	0	1	0	0	1	0
NLF2	0	1	1	0	1	0	0	1	1
NLGN2	1	1	1	1	1	1	1	1	1
NLGN4Y	0	0	0	0	1	0	0	1	0
NLK	0	0	1	0	0	1	1	0	1
NLN	1	0	1	1	0	1	1	0	1
NMB	0	0	0	1	0	0	1	0	0
NMD3	1	0	1	1	0	0	0	0	0
NME1	1	0	1	1	0	1	1	0	1
NME1-NME2	1	0	1	1	0	1	1	0	1
NME3	1	0	0	0	0	0	0	0	1
NME6	1	0	1	1	0	1	1	0	1
NME7	1	0	1	1	0	1	1	0	1
NMI	1	0	1	1	0	1	1	0	1
NMNAT1	1	0	1	1	0	1	1	0	1
NMNAT2	0	1	1	0	1	1	0	1	0
NMNAT3	0	0	0	1	0	0	0	0	1
NMT1	1	0	1	1	0	1	1	0	1
NMT2	0	1	0	0	1	0	0	1	0
NNT	1	0	1	1	0	1	1	0	1
NOC2L	1	0	1	0	0	1	0	0	0
NOC3L	1	1	1	1	1	1	1	1	1
NOC4L	1	0	0	0	0	1	0	0	0
NOL1	0	0	1	1	0	1	0	0	1
NOL11	1	0	1	1	0	1	1	0	1
NOL3	0	1	1	0	0	1	0	1	1
NOL5A	1	0	1	1	0	1	1	0	1
NOL6	1	0	1	1	0	1	1	0	1
NOL7	1	0	1	1	0	1	1	0	1
NOL8	1	0	1	1	0	1	1	0	1
NOL9	1	0	0	1	0	0	0	0	0
NOLA1	1	0	1	1	0	1	1	0	1
NOLA2	0	0	0	0	0	1	0	0	1
NOLA3	1	0	0	0	0	0	1	0	1
NOLC1	0	0	0	1	0	1	1	0	1
NOMO1	1	0	1	1	0	1	1	0	1
NOMO3	0	0	0	1	0	1	1	0	1
NONO	1	1	1	0	0	1	1	0	1
NOP5/NOP58	1	0	0	1	0	1	0	0	1
NOS3	0	0	0	0	1	0	0	0	0
NOSIP	1	0	1	1	0	1	1	0	1
NOTCH2NL	1	0	1	1	0	1	1	0	1
NOTCH4	0	1	0	0	0	0	0	0	0
NOV	0	0	0	0	0	1	0	0	1
NOX1	0	1	0	0	1	0	0	1	0
NOXA1	1	0	1	1	0	1	1	0	1
NPAL1	0	1	0	0	1	1	1	1	1
NPAL2	1	0	0	0	0	0	1	0	0
NPAL3	1	0	1	1	0	1	1	0	1

NPAS1	0	1	0	0	0	0	0	1	0
NPAS3	0	1	1	0	1	0	0	1	0
NPAT	1	0	1	1	0	1	1	0	1
NPBWR1	0	1	1	0	1	1	1	1	1
NPC1	0	0	0	0	0	1	0	0	0
NPC2	1	0	1	1	0	1	1	0	1
NPDC1	0	1	0	0	1	0	0	1	0
NPFFR1	0	1	0	1	0	0	0	0	0
NPHP3	1	0	1	1	0	1	0	0	1
NPHP4	0	0	0	0	0	1	0	0	1
NPHS2	0	1	0	0	1	0	0	1	0
NPL	1	0	1	1	0	1	0	0	1
NPM2	0	1	0	0	0	0	0	1	0
NPR1	1	1	1	0	0	1	0	1	1
NPTN	0	0	0	1	0	0	0	0	0
NPTXR	0	1	1	0	1	0	0	1	0
NQO1	1	0	1	1	0	1	1	0	1
NQO2	0	0	0	1	0	1	1	0	1
NR1D2	0	1	0	0	0	1	0	1	1
NR1H2	1	0	1	1	0	0	0	0	1
NR1H3	1	0	1	1	0	1	1	0	1
NR1I2	0	0	0	0	0	0	1	0	0
NR2C1	0	0	0	0	0	1	0	0	1
NR2C2	1	0	1	1	0	1	1	0	1
NR2E1	0	1	1	0	1	0	0	1	0
NR2F2	0	1	1	0	1	1	0	1	1
NR3C1	0	0	1	1	0	1	1	0	1
NR3C2	1	1	1	0	1	1	1	1	1
NR4A1	0	0	0	0	0	0	0	1	1
NR4A2	1	0	1	0	0	1	0	0	1
NR4A3	1	1	1	1	1	1	1	1	1
NR5A1	0	0	0	0	0	0	0	1	0
NRAS	1	0	1	1	0	1	1	0	1
NRBP1	1	0	1	1	0	1	1	0	1
NRCAM	1	1	1	0	1	1	0	1	0
NRD1	1	0	1	1	0	1	1	0	1
NRF1	1	0	1	1	0	1	1	0	1
NRG1	0	1	0	0	1	0	0	1	1
NRG2	0	1	1	0	1	1	0	1	1
NRG3	0	1	1	0	1	0	0	1	0
NRG4	1	0	0	1	0	1	1	0	1
NRGN	0	0	0	0	0	0	0	0	1
NRIP3	0	1	0	0	1	0	0	1	0
NRL	1	0	1	1	0	1	1	0	1
NRM	1	0	1	1	0	1	1	0	1
NRP1	1	0	0	1	0	1	0	1	1
NRP2	1	1	1	0	1	0	0	1	1
NRXN1	0	1	0	0	1	0	1	1	0
NRXN2	0	1	0	0	1	0	0	1	0
NRXN3	0	1	0	0	1	0	0	1	0
NSBP1	0	1	1	0	1	1	1	1	1
NSD1	1	0	1	1	0	1	1	0	1
NSDHL	1	1	1	1	1	1	0	0	1
NSF	1	0	1	1	0	1	1	0	1
NSFL1C	0	0	0	0	0	1	1	0	1
NSMAF	1	0	1	1	0	1	1	0	1
NSUN2	1	0	1	1	0	1	1	0	1
NSUN3	1	0	1	1	0	1	1	0	1
NSUN4	1	0	1	1	0	1	1	0	1
NSUN5	0	0	1	0	0	0	0	0	0
NSUN5C	0	0	1	0	0	0	0	0	1
NSUN6	1	0	1	1	0	1	1	0	1

NT5C	0	0	1	0	0	0	0	0	0
NT5C2	0	0	1	1	0	1	1	0	1
NT5C3	1	0	1	1	0	1	1	0	1
NT5C3L	0	1	0	1	1	1	0	1	0
NT5DC1	0	1	1	0	0	1	0	0	1
NTF5	0	1	0	0	1	0	0	1	0
NTHL1	1	0	1	1	0	1	1	0	1
NTNG2	0	1	0	0	1	0	0	1	0
NTSR1	0	1	0	0	1	1	0	1	0
NTSR2	0	1	0	0	1	0	0	1	0
NUAK2	0	0	0	0	0	0	0	0	1
NUBP1	0	0	0	1	0	1	1	0	1
NUBP2	1	0	1	1	0	1	1	0	1
NUBPL	1	0	1	1	0	1	1	0	1
NUCB1	1	1	1	1	1	1	1	1	1
NUCB2	0	0	0	0	0	0	0	0	1
NUCKS1	0	0	0	1	0	1	1	0	1
NUDC	0	0	0	0	0	1	0	0	1
NUDCD1	1	0	1	1	0	1	1	0	1
NUDCD2	1	0	1	1	0	1	1	0	1
NUDCD3	1	0	1	1	0	1	0	0	1
NUDT1	1	0	0	0	0	0	0	0	1
NUDT12	0	0	1	1	0	1	1	0	1
NUDT14	0	1	0	0	0	0	0	1	0
NUDT15	1	0	1	1	0	1	1	0	1
NUDT16	1	0	1	0	0	1	0	0	1
NUDT16P	1	1	1	1	1	1	0	1	1
NUDT17	0	0	1	1	0	1	1	0	1
NUDT2	1	0	1	1	0	1	1	0	1
NUDT21	1	0	1	1	0	1	1	0	1
NUDT22	0	0	0	0	0	1	0	0	1
NUDT3	0	0	0	0	0	1	0	0	0
NUDT4	1	0	1	1	0	1	1	0	1
NUDT5	1	0	1	1	0	1	1	0	1
NUDT6	1	0	1	1	0	1	1	0	1
NUDT9	1	0	0	0	0	1	1	0	1
NUFIP2	1	0	1	1	0	1	1	0	1
NUMA1	1	0	1	1	0	1	0	0	1
NUMB	0	0	1	1	0	0	0	0	0
NUP107	1	0	1	1	0	1	0	0	1
NUP133	1	0	0	1	0	1	1	0	1
NUP155	1	0	1	1	0	1	1	1	1
NUP160	1	0	1	1	0	1	1	0	1
NUP188	1	0	1	1	0	1	1	0	1
NUP205	1	0	1	1	0	1	1	0	1
NUP214	1	0	1	1	0	1	1	0	1
NUP35	1	1	1	1	1	1	1	1	1
NUP37	1	0	1	1	0	1	1	0	1
NUP43	1	0	1	1	0	1	1	0	1
NUP50	1	0	1	1	0	1	1	0	1
NUP54	1	0	1	1	0	1	1	0	1
NUP62	0	0	0	0	0	0	0	0	1
NUP85	0	0	0	1	0	1	0	0	1
NUP88	1	0	1	1	0	1	1	0	1
NUPL2	0	0	1	1	0	1	0	1	1
NUSAP1	1	0	1	1	0	1	1	0	1
NUT	1	0	0	0	0	0	1	0	1
NUTF2	1	0	1	1	0	1	1	0	1
NVL	1	0	1	1	0	1	1	0	1
NXF1	0	0	0	1	0	1	1	0	1
NXN	1	0	0	0	0	0	0	0	0
NXPH2	0	1	1	0	1	1	0	1	0

NXPH3	0	1	0	0	1	0	0	1	0
NXT1	1	0	0	1	0	1	0	0	1
NXT2	0	1	0	0	1	0	0	0	0
NY-REN-7	0	1	0	0	1	0	0	1	0
NY-SAR-48	1	0	1	1	0	1	1	0	1
NYD-SP21	0	0	0	1	0	0	0	0	0
OAF	0	1	1	0	0	1	1	0	1
OAS1	0	0	1	1	1	1	1	0	1
OAS2	1	0	1	1	0	1	1	0	1
OAS3	1	0	0	0	0	1	0	0	1
OASL	0	0	0	0	0	1	1	0	1
OAT	0	1	0	0	1	0	0	1	0
OAZ1	1	0	1	1	1	1	0	0	1
OAZ2	1	0	1	1	0	1	1	0	1
OAZ3	1	0	1	1	0	1	1	0	1
OBFC1	1	0	1	1	0	1	1	0	1
OBFC2A	1	0	0	1	0	1	1	0	1
OBFC2B	1	0	1	1	0	1	0	0	1
OCIAD1	0	0	0	0	0	1	0	0	1
OCRL	0	1	0	0	0	0	0	0	0
ODF2L	1	0	1	1	0	1	1	0	1
ODF4	0	0	0	0	0	0	1	0	1
ODZ1	1	0	0	0	0	0	0	0	0
OFCC1	0	1	0	0	0	0	0	1	0
OFD1	1	1	1	1	1	1	1	1	1
OGDH	1	0	1	1	0	1	1	0	1
OGDHL	0	1	1	0	1	0	0	1	0
OGFOD1	1	0	1	1	0	1	1	0	1
OGFRL1	1	0	0	0	0	1	1	0	1
OGT	1	1	1	1	1	1	1	1	1
OIP5	1	0	1	1	0	1	1	0	1
OKL38	0	0	0	0	1	0	0	0	0
OLFM1	0	1	1	0	1	0	0	1	0
OLFML2B	0	0	0	1	0	1	0	1	1
OLFML3	1	0	0	0	0	0	1	0	0
OLIG1	0	1	0	0	1	0	0	0	0
OLR1	1	0	1	1	0	1	0	0	0
OMA1	1	0	0	1	0	0	1	0	0
OPA1	1	0	1	1	0	1	1	0	1
OPA3	0	0	0	0	0	0	0	0	1
OPLAH	0	0	0	0	0	0	0	1	0
OPN1MW	0	1	0	0	0	0	0	0	0
OPN5	1	1	0	1	0	0	1	0	0
OPRD1	0	1	0	0	1	0	0	1	0
OPRS1	1	0	1	1	0	1	0	0	1
OPTN	0	1	1	0	1	0	0	0	0
OR10G3	0	1	0	0	0	0	0	0	0
OR10G8	0	0	0	0	1	0	0	0	0
OR10K1	0	1	0	0	1	0	0	1	0
OR13H1	0	1	0	0	1	0	0	0	0
OR1A2	0	1	0	0	0	0	0	0	0
OR1F1	0	1	0	0	1	0	0	1	0
OR2AG1	0	1	0	0	0	0	0	0	0
OR2H2	0	1	0	0	0	0	0	0	0
OR4D2	0	1	0	0	1	0	0	1	0
OR4F21	0	1	0	1	1	0	1	1	0
OR4M2	0	1	0	0	1	0	0	1	0
OR52I1	0	0	0	0	1	0	0	0	0
OR52K2	0	0	0	0	0	0	1	0	0
OR56A1	0	0	0	0	0	0	0	1	0
OR56A3	0	0	0	0	1	0	0	0	0
OR5BU1	0	1	0	0	1	0	0	0	0

OR6C4	0	1	0	0	1	0	0	0	0
OR6S1	0	1	0	0	1	0	0	1	0
ORAOV1	0	1	0	0	1	0	0	1	0
ORC1L	1	0	1	1	0	1	1	0	1
ORC2L	1	0	0	0	0	0	1	0	1
ORC3L	1	0	1	1	0	1	1	0	1
ORC4L	1	0	1	1	0	1	1	0	1
ORC5L	1	1	1	1	0	1	1	0	1
ORC6L	1	0	1	1	0	1	1	0	1
ORMDL1	1	0	1	1	0	1	1	0	1
ORMDL2	1	0	1	1	0	1	1	0	1
ORMDL3	1	0	1	1	0	1	1	0	1
OS9	1	0	0	1	0	1	0	0	1
OSBP	1	0	0	1	0	1	1	0	1
OSBP2	0	1	1	0	1	1	0	1	1
OSBPL10	0	1	0	0	1	0	0	1	0
OSBPL11	1	0	1	1	0	1	1	0	1
OSBPL1A	0	0	0	1	0	1	1	0	1
OSBPL2	0	0	0	1	0	0	0	0	1
OSBPL3	1	0	1	1	0	1	1	0	1
OSBPL5	1	1	1	0	1	1	0	0	0
OSBPL6	0	1	1	0	1	1	0	1	1
OSBPL7	0	0	0	0	0	1	0	0	0
OSBPL8	1	0	1	1	0	1	1	0	1
OSBPL9	1	0	1	1	0	1	1	0	1
OSCAR	0	0	0	1	0	1	0	0	1
OSGEP	1	0	1	1	0	1	1	0	1
OSGEPL1	1	0	1	1	0	1	1	0	1
OSM	0	0	0	0	0	1	0	0	1
OSTF1	1	0	1	1	0	1	1	0	1
OSTBETA	0	0	0	1	0	0	0	0	0
OTOF	0	1	0	0	1	0	0	1	0
OTOP3	0	1	0	0	1	0	0	1	0
OTUD4	0	0	0	0	0	1	0	0	0
OTUD5	0	1	0	0	1	0	0	0	0
OTUD6B	1	0	1	1	0	1	1	0	1
OXA1L	1	0	1	1	0	1	1	0	1
OXCT1	0	1	0	0	0	0	0	0	1
OXNAD1	1	0	1	1	0	1	1	0	1
OXR1	1	0	1	1	0	1	1	0	1
OXSM	1	0	1	1	0	1	1	0	1
OXSR1	1	0	0	0	0	0	1	0	0
P117	1	0	1	1	0	1	0	0	1
P18SRP	1	0	1	1	0	1	1	0	1
P2RX1	0	0	0	0	0	0	0	0	1
P2RX2	0	1	0	0	1	0	0	1	0
P2RX7	0	0	0	0	0	0	0	0	1
P2RY1	1	0	1	1	0	1	0	0	1
P2RY10	0	1	0	0	1	0	0	0	0
P2RY13	1	0	1	1	0	1	1	0	1
P2RY2	0	0	0	0	0	1	0	0	1
P2RY4	0	1	0	0	0	0	0	0	0
P2RY6	0	1	0	0	1	1	0	1	0
P2RY8	1	0	1	1	0	1	1	0	1
P4HA1	1	0	1	1	0	0	0	0	1
P4HA2	0	1	0	0	1	0	0	1	0
P4HA3	0	1	0	0	0	0	0	1	0
PA2G4	1	0	0	1	0	1	0	0	1
PABPC1	1	0	1	1	0	1	1	0	1
PABPC3	0	1	0	0	1	0	0	1	0
PABPN1	0	0	0	0	0	0	0	0	1
PACRG	0	0	0	0	1	0	0	1	0

PACS1	0	0	0	0	0	0	1	0	1
PACSIN1	0	1	0	0	1	0	0	1	0
PACSIN2	1	0	1	0	0	1	1	0	1
PADI1	0	1	0	0	0	0	0	0	0
PADI2	0	0	0	0	0	1	1	0	1
PAF1	1	0	1	1	0	1	0	0	1
PAFAH1B1	1	0	0	0	0	0	1	0	1
PAFAH1B2	0	0	1	0	0	1	1	0	1
PAFAH2	1	0	1	1	0	1	1	0	1
PAG1	0	0	0	0	0	0	0	0	1
PAICS	1	0	1	1	0	1	1	0	1
PAIP2	1	0	1	1	0	1	1	0	1
PAK1	0	1	0	0	1	1	1	1	1
PAK1IP1	1	0	1	1	0	1	1	0	1
PAK4	0	0	1	0	0	0	0	0	1
PALM	0	1	0	0	1	0	0	1	0
PAM	0	0	1	0	0	1	1	0	1
PANK1	1	1	1	1	0	1	1	0	1
PANK3	0	0	1	0	0	0	0	0	1
PANK4	0	1	0	0	1	0	0	1	0
PANX1	1	0	1	0	0	1	1	0	1
PANX2	0	1	0	0	1	0	0	1	0
PAPD1	1	0	1	1	0	1	1	0	1
PAPD4	1	0	1	1	0	1	1	0	1
PAPD5	0	0	0	1	0	0	0	0	0
PAPLN	0	1	0	0	1	0	0	1	0
PAPOLA	1	0	1	1	0	1	1	0	1
PAPOLG	1	0	1	1	0	1	1	0	1
PAPSS1	0	0	0	0	0	0	0	0	1
PAQR3	1	0	1	1	0	1	1	0	1
PAQR4	0	1	0	0	1	0	0	1	0
PAQR5	0	1	0	0	1	0	0	1	0
PAQR6	0	0	0	0	1	0	0	0	0
PAQR7	0	0	0	0	0	0	1	0	0
PAQR8	0	0	0	1	0	1	0	0	1
PARC	1	0	1	1	0	1	1	0	1
PARD3	0	1	0	0	1	0	0	1	0
PARD6A	1	0	0	0	0	1	0	0	1
PARK7	1	0	1	1	0	1	1	0	1
PARL	0	0	1	0	0	1	0	0	1
PARN	1	0	1	0	0	0	0	0	0
PARP10	0	0	1	1	0	1	0	0	1
PARP12	0	0	0	0	0	0	1	0	1
PARP16	0	0	0	0	0	1	0	0	1
PARP2	1	0	1	1	0	1	1	0	1
PARP3	1	0	1	1	0	1	1	0	1
PARP4	0	0	0	1	1	1	0	1	1
PARP6	1	0	1	1	0	1	1	0	1
PARP9	1	0	1	1	0	1	1	0	1
PARS2	1	0	1	1	0	1	0	0	1
PARVG	0	0	0	1	0	1	1	0	1
PASK	1	1	1	1	0	1	0	0	1
PAWR	0	1	0	0	1	0	0	1	0
PAX3	0	1	1	0	1	0	0	1	1
PAX5	0	1	0	0	1	1	0	1	1
PAX7	0	1	1	0	1	0	0	1	0
PAX8	0	1	0	0	1	0	0	1	0
PAX9	0	1	1	0	1	1	0	1	1
PAXIP1	1	0	1	1	0	1	0	0	0
PBEF1	0	0	0	1	0	1	1	0	1
PBX1	1	0	1	1	0	1	1	0	1
PBX2	1	0	1	1	0	1	1	0	1

PBX3	1	0	1	0	0	1	1	0	1
PBXIP1	1	0	1	1	0	1	1	0	1
PCAF	1	0	1	1	0	1	1	0	1
PCBP1	1	0	1	1	0	1	1	0	1
PCBP4	0	1	0	0	1	0	0	1	0
PCCA	1	0	1	1	0	1	0	0	1
PCCB	1	0	1	1	0	1	1	0	1
PCDH10	0	1	1	0	1	1	0	1	1
PCDH7	0	1	1	0	1	1	0	1	1
PCDH9	0	1	1	1	1	1	1	1	1
PCDHAC2	0	1	1	0	1	0	0	1	0
PCDHGA1	0	1	0	0	1	0	0	1	0
PCDHGA6	0	1	1	0	1	1	0	1	1
PCDHGB1	0	1	1	0	1	0	0	1	0
PCDHGB5	0	1	1	0	1	1	0	1	1
PCDHGB6	0	1	1	0	1	1	0	1	1
PCDHGC3	0	1	1	1	1	1	1	1	1
PCF11	1	0	1	1	0	1	1	0	1
PCGF1	0	1	0	0	1	1	0	1	1
PCGF2	1	0	1	1	0	1	1	1	1
PCGF5	0	1	0	0	0	1	0	1	1
PCGF6	1	0	1	1	0	1	0	0	0
PCID2	1	0	1	1	0	1	1	0	1
PCM1	1	0	0	1	0	0	1	0	1
PCMT1	1	0	1	1	0	1	1	0	1
PCNA	1	0	1	1	0	1	1	0	1
PCNP	1	0	1	1	0	1	1	0	1
PCNT	1	0	1	1	0	1	1	0	1
PCNX	1	0	1	1	0	1	1	0	1
PCNXL3	1	0	1	1	0	1	1	0	1
PCOLCE2	0	1	0	0	1	0	0	1	1
PCQAP	1	0	1	1	0	1	1	0	1
PCSK4	0	1	0	0	1	0	0	1	0
PCSK5	1	0	1	1	0	1	1	0	1
PCSK6	0	1	0	0	1	0	0	1	0
PCSK7	1	0	1	1	0	1	1	0	1
PCSK9	0	1	1	0	1	1	0	1	0
PCTK1	1	0	1	1	0	1	1	0	1
PCTK2	1	0	1	1	0	1	1	0	1
PCYOX1	1	0	1	1	0	1	0	0	1
PCYT1A	1	0	1	1	0	1	1	0	1
PDCD10	1	0	1	1	0	1	1	0	1
PDCD11	1	0	1	1	0	1	1	0	1
PDCD1LG2	1	0	1	1	0	1	0	0	0
PDCD2	1	0	1	1	0	1	0	0	1
PDCD2L	0	0	0	0	0	0	0	0	1
PDCD4	0	0	0	0	0	0	0	0	1
PDCD5	0	1	0	0	1	0	0	0	0
PDCD6IP	1	0	1	1	0	1	1	0	1
PDCD7	1	0	1	1	0	1	1	0	1
PDCL	0	0	0	1	0	1	0	0	1
PDCL3	0	0	0	1	0	1	1	0	1
PDE1B	0	0	0	0	0	0	0	1	0
PDE3A	0	1	0	0	1	0	0	1	0
PDE3B	1	0	1	1	0	1	0	0	0
PDE4B	1	1	1	1	0	1	1	1	1
PDE4DIP	1	1	1	1	1	1	1	1	1
PDE6D	1	0	1	1	0	1	1	0	1
PDE7A	1	0	1	1	0	1	1	0	1
PDE7B	1	0	1	1	0	1	1	0	1
PDE8A	0	0	0	0	0	1	0	0	1
PDE8B	0	1	0	0	1	0	0	1	0

PDGFB	0	1	1	0	1	0	0	1	0
PDGFC	1	0	0	1	0	1	1	0	1
PDGFRB	0	1	0	0	0	0	0	1	0
PDHA1	0	1	0	0	1	1	0	1	0
PDHB	0	0	0	1	0	0	0	0	0
PDHX	1	0	1	1	0	1	1	0	1
PDIA4	1	0	1	1	0	1	1	0	1
PDIK1L	1	0	1	1	0	1	1	0	1
PDK1	1	0	1	1	0	1	0	0	1
PDK2	0	0	0	0	0	1	1	0	1
PDK3	0	1	0	0	1	1	1	0	1
PDK4	1	0	1	1	0	1	1	0	1
PDLIM1	0	1	0	0	1	0	0	1	1
PDLIM4	0	1	0	0	1	0	0	1	0
PDLIM5	1	1	1	1	0	1	1	1	1
PDLIM7	0	1	0	0	0	0	0	1	0
PDP2	1	0	1	0	0	1	1	1	1
PDPN	0	1	1	0	1	0	0	1	0
PDPR	1	0	1	1	0	1	1	0	1
PDRG1	1	0	0	1	0	0	0	0	0
PDSS1	1	0	0	0	0	1	0	0	1
PDSS2	0	0	0	0	0	1	0	0	1
PDXK	0	0	0	0	0	0	0	1	0
PDXP	1	0	1	0	0	1	0	0	1
PDZD11	0	1	1	1	1	1	0	0	1
PDZD8	1	1	1	0	0	1	1	0	1
PEA15	0	0	0	1	0	0	0	0	0
PECAM1	0	0	0	0	0	0	1	0	1
PECI	1	0	1	1	0	1	1	0	1
PECR	1	0	1	0	0	1	1	1	1
PEF1	0	0	1	0	0	1	0	0	1
PEG3	0	1	1	0	1	1	0	1	0
PELI1	0	0	0	0	0	0	1	0	0
PELI2	0	0	0	0	0	0	1	0	1
PELI3	0	0	0	0	0	0	0	0	1
PELO	0	1	1	0	1	1	1	1	1
PELP1	0	0	0	0	0	1	0	0	1
PEO1	1	0	1	0	0	1	0	0	1
PEPD	0	1	0	0	0	1	0	1	1
PER1	0	0	0	0	0	0	0	0	1
PER2	0	0	0	0	0	0	0	0	1
PER3	1	0	1	1	0	1	1	0	1
PERLD1	1	0	1	1	0	1	1	0	1
PES1	1	1	1	1	0	1	0	0	1
PET112L	1	1	1	1	0	1	1	1	1
PEX1	1	0	1	1	0	1	1	0	1
PEX11A	0	0	1	0	0	1	1	0	1
PEX11B	1	0	1	1	0	1	1	0	1
PEX13	1	0	1	1	0	1	1	0	1
PEX14	0	0	1	0	0	1	0	0	1
PEX16	0	1	1	0	1	1	0	1	1
PEX19	1	0	1	1	0	1	1	0	1
PEX26	1	0	1	1	0	1	1	0	1
PEX3	1	0	1	1	0	1	1	0	1
PEX5	1	0	1	1	0	1	1	0	1
PEX6	1	0	1	1	0	1	1	0	1
PEX7	1	0	1	1	0	1	1	0	1
PFAAP5	1	0	1	1	0	1	1	0	1
PFAS	1	0	1	1	0	1	1	0	1
PFDN1	1	0	1	1	0	1	1	0	1
PFDN2	1	0	1	1	0	1	1	0	1
PFDN5	1	0	1	1	0	1	1	0	1

PFDN6	1	0	1	1	0	1	1	0	1
PFKFB2	1	0	1	1	0	1	1	0	1
PFKFB3	0	0	0	0	0	1	0	0	1
PFKM	1	0	1	1	0	1	1	0	1
PFKP	0	1	0	0	1	0	0	1	0
PFN2	0	1	0	0	1	0	0	1	1
PGA5	0	1	0	0	1	0	1	0	1
PGAM1	1	0	0	1	0	0	0	0	1
PGAM5	1	0	1	1	0	1	1	0	1
PGAP1	1	0	1	1	0	1	1	0	1
PGBD1	0	1	0	0	0	0	0	1	0
PGBD2	1	0	0	0	0	1	0	0	0
PGBD3	1	0	1	0	0	1	1	0	1
PGBD4	1	0	1	1	0	1	1	0	1
PGD	1	0	1	1	0	1	0	0	1
PGDS	1	0	0	1	0	1	0	0	0
PGGT1B	1	0	1	0	0	1	0	0	1
PGK1	1	0	1	1	0	1	1	0	1
PGLS	1	0	0	1	0	1	0	0	0
PGLYRP4	0	1	0	0	1	0	0	0	0
PGM1	0	0	1	1	0	1	1	0	1
PGM2L1	0	0	0	0	0	1	0	0	1
PGM3	1	0	1	1	0	1	1	1	1
PGM5	0	1	0	1	1	1	0	1	1
PGRMC1	0	1	0	0	1	0	0	1	0
PGRMC2	1	0	1	1	0	1	1	0	1
PH-4	1	0	1	0	0	1	1	0	1
PHACS	1	0	0	0	0	0	1	0	1
PHACTR1	0	1	0	0	1	0	0	0	0
PHACTR3	0	1	0	0	1	0	0	1	1
PHACTR4	1	0	1	1	0	1	1	0	0
PHB	1	0	1	1	0	1	1	0	1
PHB2	1	0	1	1	0	1	1	0	1
PHC1	1	0	1	1	0	1	1	1	1
PHF1	1	0	1	1	0	1	1	0	1
PHF10	0	0	0	0	0	0	0	1	1
PHF11	1	0	1	1	0	1	1	0	1
PHF12	1	0	1	1	0	1	1	0	1
PHF13	1	0	1	1	0	1	1	1	1
PHF14	0	0	0	1	0	0	0	0	1
PHF16	0	1	0	0	0	0	0	1	0
PHF17	1	0	0	1	0	1	1	0	1
PHF19	1	0	1	0	0	1	1	0	1
PHF20L1	0	0	0	0	0	0	0	0	1
PHF21A	1	0	1	1	0	1	1	0	1
PHF23	1	0	1	1	0	1	1	0	1
PHF3	1	0	0	0	0	0	0	0	0
PHF5A	1	0	1	1	0	1	1	0	1
PHF7	1	0	1	1	0	1	1	0	1
PHGDH	0	1	0	0	0	1	0	0	0
PHIP	1	0	1	1	0	1	1	0	1
PHKA2	1	1	1	1	1	1	0	1	1
PHKB	1	0	1	1	0	1	1	0	1
PHKG2	0	0	0	1	0	0	0	0	0
PHLDA1	1	0	1	1	0	1	1	1	1
PHLDA2	0	0	0	0	1	0	0	0	0
PHLDA3	0	1	0	0	1	0	0	1	0
PHLDB1	0	1	1	0	1	0	0	1	0
PHLPPL	1	0	0	0	0	0	0	0	0
PHOSPHO1	0	0	0	0	1	1	0	0	1
PHOSPHO2	0	1	0	0	1	0	0	1	0
PHTF1	1	0	0	1	0	0	1	0	1

PHTF2	1	0	1	1	0	1	1	0	1
PHYHIPL	0	1	0	0	1	0	0	1	0
PI4K2B	1	0	0	0	0	0	0	0	0
PIAS2	1	0	1	0	1	1	1	0	1
PIAS3	0	0	0	1	0	1	0	0	1
PIAS4	0	0	0	0	0	1	0	0	0
PICALM	1	0	0	1	0	0	1	0	1
PICK1	0	1	0	0	0	1	0	0	1
PIGA	1	1	1	1	1	1	0	1	1
PIGB	1	0	0	1	0	1	1	0	1
PIGC	1	0	0	0	0	0	0	0	0
PIGF	1	0	1	1	0	1	1	0	1
PIGG	1	0	1	1	0	1	0	0	1
PIGK	1	0	1	1	0	1	1	0	1
PIGL	1	0	1	1	0	1	1	0	1
PIGM	0	1	1	1	1	1	1	1	1
PIGN	1	0	1	1	0	1	1	0	1
PIGP	1	0	1	1	0	1	1	0	1
PIGS	1	0	1	1	0	1	0	0	1
PIGT	0	0	0	0	0	1	0	0	1
PIGV	1	0	1	1	0	1	1	0	1
PIGW	1	0	1	1	0	1	1	0	1
PIGX	1	0	1	1	0	1	1	0	1
PIGZ	0	0	0	0	0	0	0	1	0
PIK3AP1	0	0	1	0	0	1	0	0	1
PIK3C2B	0	1	0	0	1	0	0	1	0
PIK3C3	1	0	1	1	0	1	1	0	1
PIK3CA	1	0	1	1	0	1	1	0	1
PIK3CD	0	0	0	0	0	0	0	0	1
PIK3CG	1	0	1	1	0	1	1	0	1
PIK3R1	0	0	0	1	0	0	1	0	1
PIK3R2	1	0	1	1	0	1	1	0	1
PIK3R3	1	1	1	1	1	1	0	1	1
PIK3R4	1	0	1	1	0	1	1	0	1
PIK4CA	1	0	1	1	0	1	1	0	1
PILRB	1	0	1	1	0	1	0	0	1
PIM1	0	0	0	1	0	1	0	1	1
PIM2	0	1	0	0	1	0	0	0	0
PIN4	0	1	1	0	1	1	0	1	1
PIP3-E	1	0	1	1	0	1	1	0	1
PIP5K1A	1	0	1	1	0	1	1	0	1
PIP5K2A	0	0	0	0	0	0	0	0	1
PIP5K2B	1	0	0	1	0	1	1	0	1
PIP5K2C	0	0	0	0	0	1	0	0	0
PIP5K3	1	0	1	1	0	1	1	0	1
PIR	0	1	1	1	1	1	0	1	0
PISD	0	0	0	0	0	1	0	0	1
PITPNB	1	0	1	1	0	1	1	0	1
PITPNC1	1	0	1	1	0	1	1	0	1
PITPNM1	0	0	1	0	0	1	0	1	0
PITX3	1	1	1	1	1	1	1	1	1
PKD1	1	0	0	1	0	1	0	0	1
PKD1L1	0	0	0	0	0	1	0	0	0
PKD2	0	1	0	0	0	0	0	1	1
PKIA	0	1	0	0	0	1	1	0	1
PKIB	1	1	1	1	1	1	1	1	1
PKM2	1	0	1	1	0	1	0	0	1
PKMYT1	0	0	0	0	0	0	1	0	1
PKN1	1	0	0	0	0	0	0	0	0
PKN2	1	0	1	1	0	1	1	0	1
PKNOX1	0	0	0	0	0	0	0	0	1
PKP2	0	1	0	0	0	0	1	1	1

PKP4	0	1	1	0	1	1	0	1	1
PLA1A	0	0	0	0	0	0	1	0	0
PLA2G10	0	1	0	0	1	0	0	1	0
PLA2G12A	1	0	1	1	0	1	1	0	1
PLA2G4A	1	0	1	1	0	1	1	0	1
PLA2G4C	0	0	1	0	0	1	1	0	1
PLA2G5	0	1	0	0	0	0	0	0	0
PLA2G6	0	0	0	0	0	0	1	0	1
PLA2G7	1	0	1	1	0	1	0	1	1
PLAA	1	0	1	1	0	1	1	0	1
PLAC1	0	1	0	0	1	0	0	0	0
PLAC8	0	0	0	0	0	0	1	0	1
PLAG1	1	0	1	0	0	1	1	1	1
PLAGL2	1	0	1	1	0	1	1	0	1
PLAU	1	0	1	1	0	1	0	1	0
PLAUR	1	0	1	1	0	1	1	0	1
PLCB1	0	1	0	0	1	1	0	0	1
PLCB2	0	0	0	0	0	1	0	0	1
PLCB3	1	0	0	0	0	0	0	0	1
PLCD1	0	0	0	0	0	0	0	1	0
PLCG1	0	1	0	0	1	0	1	1	1
PLCL1	0	1	0	0	0	0	0	0	0
PLCL2	0	0	0	0	0	0	1	0	0
PLCXD1	0	0	0	0	0	0	0	0	1
PLCXD3	0	1	0	0	1	0	0	1	0
PLD2	0	1	1	0	0	0	0	1	1
PLD3	1	0	1	1	0	1	1	0	1
PLDN	1	0	1	1	0	1	1	0	1
PLEC1	0	0	0	0	0	1	0	0	1
PLEK	1	0	1	1	0	1	1	0	1
PLEK2	0	1	0	0	0	0	0	1	0
PLEKHA1	0	1	1	1	1	1	0	1	1
PLEKHA3	1	0	1	1	0	1	1	0	1
PLEKHA6	0	1	0	0	1	0	0	1	0
PLEKHA7	0	1	1	0	1	1	0	1	0
PLEKHA9	1	0	1	1	0	1	1	0	1
PLEKHB1	1	0	0	0	0	0	1	0	0
PLEKHB2	1	0	1	1	0	1	1	0	1
PLEKHC1	0	1	1	0	1	0	0	1	0
PLEKHG2	1	0	1	0	0	1	1	0	1
PLEKHG3	0	1	0	0	0	1	0	1	1
PLEKHG4	1	0	0	0	0	0	0	0	0
PLEKHG5	1	1	0	0	1	0	0	1	0
PLEKHH2	0	1	0	0	1	1	0	1	1
PLEKHJ1	1	0	1	1	0	1	1	0	1
PLEKHM1	0	0	1	1	0	1	0	0	1
PLEKHO1	1	0	0	0	0	0	1	1	1
PLEKHQ1	0	0	0	0	0	1	0	0	0
PLK1	1	0	1	1	0	1	1	0	1
PLK4	1	0	1	1	0	1	1	0	1
PLLP	0	1	0	0	1	0	0	1	0
PLOD1	0	0	0	0	0	0	1	0	1
PLOD2	1	1	1	1	1	1	1	1	1
PLOD3	1	0	1	1	0	1	1	0	1
PLRG1	1	0	1	1	0	1	1	0	1
PLSCR4	0	1	1	0	1	1	0	1	0
PLVAP	0	0	0	0	1	0	0	0	0
PLXDC1	1	1	1	1	0	1	1	1	1
PLXDC2	1	0	1	1	0	1	1	1	1
PLXNA2	0	1	0	0	0	1	0	0	1
PLXNC1	0	1	0	0	1	0	0	1	1
PMAIP1	0	0	1	1	0	1	1	1	1

PMF1	1	0	1	1	0	1	1	0	1
PML	1	0	1	0	0	1	0	0	1
PMM2	1	0	1	1	0	1	1	0	1
PMP22	0	1	1	1	1	1	0	1	1
PMPCA	1	0	1	1	0	1	0	0	1
PMPCB	1	0	0	1	0	0	0	0	0
PMS1	1	0	1	1	0	1	1	0	1
PMS2	1	0	1	1	0	1	0	0	1
PMS2L2	1	0	1	1	0	1	1	0	1
PMS2L3	0	0	0	1	0	1	0	0	1
PMS2L5	1	0	1	1	0	1	1	0	1
PMVK	1	0	1	1	0	1	1	0	1
PNKD	1	0	1	1	0	1	0	0	1
PNKP	1	0	0	0	0	1	0	0	0
PNMA3	0	1	0	0	1	0	0	1	0
PNN	1	0	1	1	0	1	1	0	1
PNOC	0	1	0	0	1	0	0	1	0
PNPLA1	0	0	0	0	0	1	1	0	1
PNPLA4	0	0	1	0	0	1	1	0	1
PNPLA5	0	1	0	0	1	0	0	1	0
PNPO	0	0	1	0	0	1	0	0	1
PNPT1	1	0	1	1	0	1	1	0	1
PNRC1	1	0	1	1	0	1	1	0	1
PNRC2	0	0	0	0	0	0	1	0	1
PODN	0	1	0	0	1	0	0	1	0
PODXL2	0	1	0	0	1	0	0	1	0
POFUT1	1	0	1	1	0	1	1	0	1
POGK	1	0	1	1	0	1	1	0	1
POGZ	0	0	0	0	0	0	0	0	1
POLA	1	0	1	1	1	1	1	1	1
POLA2	1	0	1	1	0	1	1	0	1
POLB	1	0	1	1	0	1	1	0	1
POLD3	0	0	0	0	0	1	0	0	1
POLD4	0	0	0	1	0	0	0	0	1
POLDIP2	1	0	1	1	0	1	1	0	1
POLDIP3	1	0	0	1	0	0	1	0	1
POLE2	1	0	0	1	0	1	1	0	1
POLE3	1	0	1	1	0	1	1	0	1
POLG	0	0	0	0	0	0	0	1	1
POLG2	1	0	1	1	0	1	1	0	1
POLH	1	0	1	1	0	1	1	0	1
POLI	0	0	0	1	0	0	0	0	0
POLL	1	0	0	0	0	1	0	0	1
POLM	0	0	0	0	0	1	0	0	0
POLQ	1	0	1	1	0	1	1	0	1
POLR1A	1	0	1	1	0	1	1	0	1
POLR1B	1	0	1	1	0	1	1	0	1
POLR1C	1	0	1	1	0	1	1	0	1
POLR1D	1	0	1	1	0	1	1	0	1
POLR2A	1	0	1	1	0	1	1	0	1
POLR2B	1	0	1	1	0	1	1	0	1
POLR2C	1	0	1	1	0	1	1	0	1
POLR2D	0	0	1	0	0	1	0	0	1
POLR2F	1	1	1	0	1	1	1	1	1
POLR2G	1	0	0	0	0	0	0	0	0
POLR2H	1	0	1	1	0	1	1	0	1
POLR2I	1	0	1	1	0	1	0	0	1
POLR2J	1	0	1	1	0	1	1	0	1
POLR2J3	1	0	1	1	0	1	1	0	1
POLR2K	1	0	1	1	0	1	1	0	1
POLR3A	1	0	1	1	0	1	1	0	1
POLR3B	1	0	1	1	0	1	1	0	1

POLR3C	1	1	1	1	1	1	1	1	1
POLR3D	0	0	0	0	0	1	0	0	1
POLR3E	1	0	1	1	0	1	1	0	1
POLR3F	1	0	1	1	0	1	1	0	1
POLR3GL	1	0	1	1	1	1	1	1	1
POLR3K	1	0	0	0	0	1	0	0	1
POLRMT	0	1	0	1	0	1	0	0	1
POMC	0	1	1	0	1	1	0	1	0
POMGNT1	0	0	1	1	1	1	0	1	1
POMT2	1	0	1	1	0	1	1	0	1
POMZP3	1	0	1	1	0	1	1	0	1
PON2	0	0	1	1	0	1	1	0	1
POP1	1	0	1	1	0	1	1	0	1
POP4	1	0	1	1	0	1	1	0	1
POP5	1	0	1	1	0	1	0	0	1
POR	0	0	0	1	0	0	0	0	0
PORCN	0	1	1	0	1	1	1	1	1
POU2F1	1	0	1	1	0	1	1	0	1
POU2F2	0	0	0	1	0	1	1	0	1
POU4F1	0	1	0	0	1	0	0	1	0
POU6F1	0	0	1	1	0	1	0	0	1
PPA1	0	0	0	1	0	1	0	0	0
PPAP2A	0	1	0	0	0	1	0	1	1
PPAP2B	1	0	1	1	0	1	1	1	1
PPAP2C	0	1	0	0	1	0	0	1	0
PPAPDC1B	1	0	0	1	0	1	0	0	0
PPAPDC2	1	0	1	0	0	1	1	0	1
PPARBP	1	0	1	1	0	1	1	0	1
PPARG	1	0	0	1	0	1	0	1	1
PPARGC1A	0	1	0	0	1	0	0	1	0
PPAT	1	0	1	1	0	1	1	0	1
PPCS	1	0	1	1	0	1	1	0	1
PPFIA1	0	1	0	0	1	0	0	1	0
PPFIA3	0	0	0	0	0	0	0	1	1
PPFIBP1	1	0	1	1	0	1	1	0	1
PPFIBP2	1	0	1	1	0	1	1	0	1
PPHLN1	1	0	1	1	0	1	1	0	1
PPIA	0	0	0	0	0	1	0	0	0
PPIB	1	0	0	1	0	1	1	0	1
PPIC	0	1	0	0	1	0	0	1	0
PPID	1	0	1	1	0	1	1	0	1
PPIE	1	0	1	1	0	1	0	0	1
PPIG	1	0	1	1	0	1	1	0	1
PPIH	1	0	1	1	0	1	1	0	1
PPIL1	1	0	1	1	0	1	1	0	1
PPIL2	1	0	0	0	0	0	0	0	0
PPIL3	1	0	1	1	0	1	1	0	1
PPIL5	1	0	1	1	0	1	1	0	1
PPM1A	1	0	1	1	0	1	1	0	1
PPM1B	1	0	1	1	0	1	1	0	1
PPM1D	1	0	1	1	0	1	1	0	1
PPM1G	1	0	1	1	0	1	0	0	1
PPM1K	1	0	1	1	0	1	1	0	1
PPM1L	1	0	1	1	0	1	1	0	1
PPM1M	1	0	0	1	0	0	0	0	1
PPM2C	0	0	1	0	0	1	1	0	1
PPME1	1	0	1	1	0	1	1	0	1
PPOX	1	0	1	0	0	1	0	0	1
PPP1CB	1	0	1	1	0	1	1	0	1
PPP1CC	0	0	1	0	0	1	1	0	1
PPP1R10	1	0	1	1	0	1	1	0	1
PPP1R11	1	0	1	1	0	1	1	0	1

PPP1R12A	1	0	1	1	0	1	1	0	1
PPP1R12C	0	0	1	0	0	1	1	0	1
PPP1R13B	0	1	0	0	1	0	0	1	0
PPP1R13L	1	1	1	1	1	1	0	1	1
PPP1R14A	0	1	0	0	1	0	0	1	0
PPP1R14B	1	0	0	0	0	0	0	0	1
PPP1R14C	0	1	1	1	1	1	0	1	1
PPP1R15A	1	0	0	1	0	1	0	0	1
PPP1R15B	1	0	0	1	0	0	0	0	0
PPP1R16B	1	1	0	0	1	0	1	1	0
PPP1R1B	0	1	0	0	1	0	0	1	0
PPP1R2	0	1	0	0	1	0	0	1	1
PPP1R3B	1	1	1	1	1	1	1	1	1
PPP1R3D	1	0	1	1	0	1	1	0	1
PPP1R3F	0	1	1	0	1	1	0	0	1
PPP1R7	1	1	1	1	0	1	0	0	1
PPP1R9B	0	0	0	0	0	1	1	0	1
PPP2CA	1	0	1	1	0	1	1	0	1
PPP2CB	0	1	0	0	1	0	0	1	0
PPP2R1A	1	0	1	1	0	1	1	0	1
PPP2R1B	1	0	1	1	0	1	1	0	1
PPP2R2B	0	1	1	0	1	1	0	1	1
PPP2R3A	0	1	0	0	0	0	0	1	0
PPP2R3B	1	0	1	1	0	1	1	0	1
PPP2R4	1	0	1	1	0	1	1	0	1
PPP2R5A	1	0	1	1	0	1	1	0	1
PPP2R5C	1	0	1	1	0	1	1	0	1
PPP2R5D	1	0	1	1	0	1	1	0	1
PPP2R5E	1	0	1	1	0	1	1	0	1
PPP3CA	0	0	1	0	0	1	0	0	1
PPP3CB	1	0	1	1	0	1	1	0	1
PPP3CC	1	0	1	1	0	1	0	0	1
PPP3R1	1	0	1	1	0	1	1	0	1
PPP4R1	0	0	0	1	0	0	1	0	1
PPP4R1L	1	0	1	1	0	1	1	0	1
PPP5C	1	0	1	1	0	1	0	0	1
PPP6C	1	0	1	1	0	1	1	0	1
PPRC1	0	0	0	1	0	1	0	0	1
PPT1	0	0	0	0	0	1	0	0	1
PPT2	0	1	0	0	1	1	0	1	1
PPTC7	0	0	1	1	0	1	1	0	1
PPWD1	1	0	1	1	0	1	1	0	1
PQBP1	1	0	1	1	0	1	0	0	1
PRAM1	0	0	0	1	0	1	0	0	1
PRC1	0	0	0	0	0	0	0	0	1
PRCC	1	0	1	1	0	1	1	0	1
PRCP	1	0	1	1	0	1	1	0	1
PRDM1	1	0	1	1	0	1	1	0	1
PRDM10	1	0	1	1	0	1	1	0	1
PRDM14	0	1	0	0	1	1	0	1	0
PRDM15	1	0	0	1	0	1	0	0	1
PRDM4	0	0	0	0	0	0	0	1	1
PRDM8	0	1	1	0	1	1	1	1	1
PRDX1	1	0	1	1	0	1	1	0	1
PRDX2	0	1	1	0	1	1	0	1	0
PRDX3	0	0	0	1	0	1	0	0	1
PRDX4	0	1	0	0	1	0	0	1	0
PRDX5	1	0	1	1	0	1	1	0	1
PRDX6	1	0	1	1	0	1	1	0	1
PREI3	1	0	1	1	0	1	1	0	1
PREP	0	1	1	0	0	1	0	1	0
PREPL	1	0	1	1	0	1	1	0	1

PREX1	1	0	0	1	0	1	0	0	1
PRICKLE1	0	1	1	0	1	1	1	1	1
PRIM1	0	0	0	0	0	0	0	0	1
PRIM2A	1	0	1	1	0	1	1	0	1
PRKAA1	0	0	0	0	0	0	0	0	1
PRKAB1	1	0	1	1	0	1	1	0	1
PRKAB2	1	0	1	1	0	1	1	0	1
PRKACA	1	0	1	1	0	1	1	0	1
PRKAG1	1	0	1	1	0	1	1	0	1
PRKAG2	0	0	1	1	0	1	1	0	1
PRKAR1A	1	0	1	1	0	1	1	0	1
PRKAR1B	0	1	0	0	1	0	0	1	0
PRKCA	1	1	1	1	1	1	0	1	1
PRKCB1	0	1	0	0	0	0	0	0	1
PRKCDBP	0	1	0	0	1	0	0	1	1
PRKCE	1	0	1	1	0	1	1	0	1
PRKCH	0	1	0	0	0	0	0	0	1
PRKCI	1	0	1	1	0	1	0	0	1
PRKCQ	0	1	1	0	1	1	1	0	1
PRKCSH	0	0	1	1	0	1	0	0	1
PRKD2	0	0	0	0	0	0	0	0	1
PRKD3	1	0	0	1	0	0	1	0	1
PRKDC	1	0	1	1	0	1	0	0	1
PRKRA	1	0	1	1	0	1	1	0	1
PRKRIP1	1	0	1	1	0	1	1	0	1
PRKRIR	1	0	1	1	0	1	1	0	1
PRKX	0	0	0	0	0	1	1	0	1
PRLR	0	0	1	0	0	1	1	0	1
PRMT1	1	0	1	1	0	1	0	0	1
PRMT2	1	0	1	1	0	1	1	0	1
PRMT3	0	0	0	1	0	1	1	0	1
PRMT5	0	0	1	1	0	1	1	0	1
PRMT6	0	0	0	0	0	1	0	0	1
PRMT7	1	0	1	1	0	1	1	0	1
PRNP	1	0	0	0	0	1	0	0	1
PROC	0	0	0	0	0	0	0	1	1
PROCA1	0	0	0	0	1	0	0	0	0
PROCR	0	0	0	0	0	1	0	0	0
PROK2	0	1	1	0	1	1	1	1	1
PROS1	1	1	1	1	0	1	0	1	1
PROSC	1	0	1	1	0	1	1	0	1
PRPF18	0	1	1	0	1	1	0	1	0
PRPF19	1	0	1	1	0	1	1	0	1
PRPF3	1	0	1	1	0	1	1	0	1
PRPF31	1	0	1	1	0	1	1	0	1
PRPF38A	1	0	1	1	0	1	1	0	1
PRPF38B	1	0	1	0	0	1	0	0	1
PRPF39	1	0	0	0	0	0	0	1	0
PRPF4	1	0	1	1	0	1	1	0	1
PRPF4B	1	0	1	1	0	1	0	0	1
PRPF6	1	0	1	1	0	1	1	0	1
PRPF8	0	0	1	1	0	1	0	0	1
PRPS1	0	1	1	0	1	1	0	1	1
PRPS2	0	1	1	0	1	1	0	1	1
PRPSAP1	0	0	0	0	0	1	0	0	1
PRPSAP2	1	0	1	1	0	1	0	0	1
PRR11	1	0	1	1	0	1	1	0	1
PRR13	1	0	1	1	0	1	1	0	1
PRR14	1	0	0	1	0	0	1	0	1
PRR3	1	0	1	1	0	1	1	0	1
PRR5	0	1	0	0	1	0	0	1	0
PRR6	0	0	0	0	1	0	0	1	0

PRR8	1	0	0	0	0	0	0	0	0
PRRG1	0	1	0	0	0	0	0	0	0
PRRG2	1	0	1	1	0	1	1	0	1
PRRG4	0	0	1	0	0	1	1	0	1
PRRT1	0	1	0	0	1	1	0	1	1
PRRT2	0	1	0	0	1	1	0	1	1
PRRX2	0	1	0	0	0	0	0	1	0
PRSS2	0	1	0	0	0	0	0	0	0
PRSS3	0	1	0	0	0	0	0	0	0
PRSSL1	0	0	0	1	0	0	0	0	0
PRTFDC1	0	1	0	0	1	0	0	1	0
PRUNE	1	0	1	1	0	1	1	0	1
PSAP	0	0	0	0	0	1	0	0	0
PSAT1	0	1	0	0	1	0	0	1	1
PSCD1	1	0	0	0	0	0	0	0	0
PSCD4	1	0	0	0	0	1	0	0	0
PSCDBP	1	0	1	1	0	1	1	0	1
PSD	0	1	1	1	1	1	1	1	1
PSD2	0	1	0	0	1	0	0	1	0
PSD3	0	1	0	0	1	0	1	1	0
PSEN2	1	0	1	0	0	1	0	1	1
PSENEN	1	0	1	1	0	1	0	0	1
PSIP1	1	0	1	0	0	1	1	0	1
PSKH1	1	0	0	0	0	1	0	0	0
PSMA1	1	0	1	1	0	1	1	0	1
PSMA2	1	0	1	1	0	1	1	0	1
PSMA3	1	0	1	1	0	1	1	0	1
PSMA4	1	0	0	1	0	1	1	0	1
PSMA5	1	0	1	1	0	1	0	0	1
PSMA6	1	0	1	1	0	1	1	0	1
PSMA7	1	0	1	1	0	1	1	0	1
PSMB1	1	0	1	1	0	1	1	0	1
PSMB10	1	0	1	1	0	1	1	0	1
PSMB2	1	0	1	1	0	1	1	0	1
PSMB3	1	0	1	1	0	1	1	1	1
PSMB4	1	0	1	1	0	1	1	0	1
PSMB5	1	0	1	1	0	1	1	0	1
PSMB6	1	0	1	1	0	1	0	0	1
PSMB7	1	0	1	1	0	1	0	0	1
PSMB8	1	0	1	1	0	1	1	0	1
PSMB9	1	0	1	1	0	1	1	0	1
PSMC1	0	1	0	0	1	0	0	1	1
PSMC2	1	0	0	1	0	1	1	0	1
PSMC3	1	0	1	1	0	1	1	0	1
PSMC3IP	1	0	1	1	0	1	0	0	1
PSMC4	1	0	1	1	0	1	1	1	1
PSMC5	1	0	1	1	0	1	1	0	1
PSMC6	1	0	0	1	0	1	0	0	1
PSMD10	1	1	1	1	1	1	1	1	1
PSMD11	0	1	0	0	1	0	0	1	0
PSMD13	1	0	1	1	0	1	1	0	1
PSMD14	1	0	1	1	0	1	1	0	1
PSMD2	0	0	0	0	0	0	0	0	1
PSMD3	1	0	1	1	0	1	1	0	1
PSMD5	1	0	1	1	0	0	0	0	1
PSMD6	0	0	0	1	0	1	1	0	1
PSMD7	1	0	1	1	0	1	1	0	1
PSME2	1	0	1	1	0	1	1	0	1
PSME3	1	0	1	1	0	1	1	0	1
PSME4	1	0	1	1	0	1	1	0	1
PSMF1	1	0	1	1	0	1	1	0	1
PSPC1	1	0	1	1	0	1	1	0	1

PSPH	1	0	0	1	0	1	1	0	1
PSRC1	1	0	1	1	0	1	1	0	1
PTAFR	0	0	0	0	0	1	0	0	0
PTBP2	0	0	0	0	0	1	1	1	1
PTCD1	1	0	1	1	0	1	1	0	1
PTCD2	1	0	0	0	0	1	0	0	1
PTDSS1	1	0	1	1	0	1	1	0	1
PTDSS2	0	0	0	0	0	1	0	0	0
PTEN	0	0	1	0	0	1	1	0	1
PTGER2	1	0	1	1	0	1	1	0	1
PTGER3	0	1	0	0	1	0	0	1	0
PTGER4	1	0	1	1	0	1	1	0	1
PTGES	0	1	0	0	1	0	0	1	0
PTGES2	1	0	1	0	0	1	0	0	1
PTGFR	0	1	0	0	1	0	0	1	1
PTGFRN	0	1	0	0	0	0	0	1	0
PTGIR	0	0	1	0	0	0	1	0	1
PTGS1	0	0	0	0	0	1	1	0	1
PTGS2	1	0	1	1	0	1	1	0	1
PTK2	0	0	0	0	0	1	1	0	1
PTK2B	1	0	1	1	0	1	0	0	1
PTMA	1	0	1	1	0	1	1	0	1
PTMS	0	0	0	0	0	1	0	1	1
PTOV1	0	0	0	0	0	0	0	0	1
PTP4A1	0	1	1	0	0	1	1	1	1
PTP4A2	0	0	0	0	0	0	0	0	1
PTPLA	0	1	0	0	0	0	0	0	0
PTPLAD1	1	0	1	0	0	1	1	0	1
PTPLAD2	0	0	1	0	0	1	0	0	1
PTPLB	1	0	1	1	0	1	1	0	1
PTPN12	1	0	1	1	0	1	1	0	1
PTPN13	0	1	0	0	0	1	0	1	1
PTPN18	0	0	1	1	0	1	0	0	1
PTPN2	1	0	1	1	0	1	1	0	1
PTPN22	1	0	1	1	0	1	1	0	1
PTPN23	0	0	0	1	0	1	0	0	0
PTPN3	0	1	0	0	1	0	0	1	0
PTPN4	0	0	0	0	0	0	1	0	1
PTPN5	0	1	0	0	1	0	0	1	0
PTPN6	1	0	0	1	0	0	1	0	1
PTPN9	0	0	0	0	0	0	1	0	1
PTPRA	0	0	0	0	0	1	0	1	1
PTPRC	1	0	1	1	0	1	1	0	1
PTPRE	1	0	1	1	0	1	0	0	1
PTPRJ	0	0	0	0	0	0	0	1	0
PTPRM	0	1	1	0	0	0	0	1	1
PTPRN2	0	1	0	0	1	0	0	1	0
PTPRO	1	1	1	1	1	1	1	1	1
PTPRR	0	0	0	0	0	0	0	1	0
PTRF	0	1	0	0	1	0	0	1	0
PTRH1	0	0	1	0	0	1	0	0	1
PTRH2	1	0	1	1	0	1	1	0	1
PTS	0	0	0	1	0	1	0	0	0
PTTG1	1	0	1	1	0	1	1	0	1
PTTG1IP	1	0	1	1	0	1	1	0	1
PTX3	0	0	0	0	0	0	1	0	1
PUM1	0	0	0	0	0	0	1	0	1
PUM2	1	0	0	1	0	0	0	0	0
PURA	0	1	0	0	1	0	0	1	0
PURB	1	0	1	1	0	1	1	0	1
PUS3	1	1	1	1	1	1	1	1	1
PUS7	0	0	0	0	0	0	1	0	1

PUS7L	1	0	1	1	0	1	1	0	1
PVALB	0	1	0	0	1	0	0	1	0
PVRL1	0	0	0	0	0	1	0	0	1
PWP1	1	0	1	1	0	1	0	0	1
PXMP3	1	0	1	1	0	1	1	0	1
PXMP4	0	0	0	1	0	1	0	0	1
PXN	1	0	0	0	0	0	0	0	0
PXT1	0	1	0	0	0	0	0	0	0
PYCARD	1	0	1	1	0	1	1	0	1
PYCR1	0	1	0	0	0	0	0	1	0
PYCR2	0	0	0	0	0	0	0	0	1
PYGB	1	0	1	1	0	1	1	1	1
PYGL	1	0	1	1	0	1	1	0	1
PYGO2	1	0	1	0	0	0	0	0	0
PYY	0	1	0	0	1	0	0	1	0
QARS	1	0	1	1	0	1	1	0	1
QDPR	1	0	1	1	0	1	1	0	1
QKI	1	0	1	1	0	1	1	0	1
QPCT	1	0	0	0	0	0	0	0	1
QPCTL	1	0	1	1	0	1	1	0	1
QPRT	0	0	0	0	0	0	1	0	0
QRICH1	1	0	0	1	0	1	0	0	1
QRSL1	1	0	1	1	0	1	1	0	1
QTRT1	0	0	0	1	0	1	0	0	1
QTRTD1	1	0	1	1	0	1	1	0	1
R3HDM1	1	0	1	1	0	1	1	0	1
R3HDML	0	1	0	0	0	0	0	1	0
RAB10	1	0	1	0	0	1	0	0	0
RAB11A	0	0	0	0	0	0	0	0	1
RAB11B	0	0	0	0	0	0	0	0	1
RAB11FIP1	1	0	0	1	0	0	1	0	1
RAB11FIP2	1	0	1	1	0	1	1	0	1
RAB11FIP3	0	0	0	0	0	0	0	1	1
RAB11FIP4	0	1	0	0	1	0	0	1	0
RAB11FIP5	0	1	0	0	1	0	0	1	0
RAB12	0	0	0	0	0	0	1	1	1
RAB13	1	0	1	1	0	1	1	0	1
RAB14	0	0	0	1	0	1	0	0	1
RAB15	0	1	0	0	1	0	0	1	1
RAB18	0	0	0	1	0	1	1	0	0
RAB1A	1	0	0	1	0	1	0	0	1
RAB1B	0	0	0	1	0	1	1	0	1
RAB20	1	0	0	1	0	1	0	0	1
RAB21	0	0	0	0	0	1	0	0	1
RAB22A	1	0	1	1	0	1	1	0	1
RAB23	1	0	1	1	0	1	1	1	1
RAB24	1	0	1	1	0	1	1	0	1
RAB28	1	1	1	0	1	0	0	1	1
RAB2B	1	0	1	1	0	1	1	0	1
RAB30	1	0	1	1	0	1	1	0	1
RAB31	1	0	1	1	0	1	1	0	1
RAB33A	1	1	0	1	1	1	0	0	1
RAB33B	1	0	1	1	0	1	1	0	1
RAB34	1	0	1	1	0	1	0	0	1
RAB35	1	0	1	1	0	1	1	0	1
RAB36	0	0	0	0	0	1	0	0	0
RAB37	0	1	0	0	1	0	0	1	0
RAB38	0	0	0	0	0	1	1	0	1
RAB3B	0	1	1	0	1	1	0	1	1
RAB3GAP1	1	0	1	1	0	1	1	0	1
RAB3GAP2	1	0	1	1	0	1	1	0	1
RAB3IL1	0	1	0	0	1	0	0	1	0

RAB3IP	0	1	0	0	0	0	0	1	1
RAB40C	1	0	1	1	0	1	1	0	0
RAB4A	1	0	1	1	0	1	1	0	1
RAB4B	1	0	1	1	0	1	1	0	1
RAB5A	1	0	1	1	0	1	1	0	1
RAB5B	0	0	1	1	0	1	1	0	1
RAB6A	0	1	0	0	1	0	0	0	0
RAB6B	0	1	0	0	1	0	0	1	0
RAB6IP1	0	0	0	0	0	0	0	1	0
RAB7L1	1	0	1	1	0	1	1	0	1
RAB8B	0	0	0	0	0	1	1	0	1
RAB9A	1	1	1	1	1	1	1	1	1
RAB9B	0	1	0	0	1	0	1	1	0
RABEP1	0	0	0	0	0	0	0	0	1
RABEP2	1	0	1	1	0	1	1	0	1
RABEPK	1	0	1	1	0	1	1	0	1
RABGAP1	1	0	1	0	0	1	1	0	1
RABGAP1L	1	0	1	1	0	1	1	0	1
RABGEF1	0	0	0	0	0	1	1	0	1
RABGGTA	1	0	1	1	0	1	0	0	1
RABGGTB	1	0	1	1	0	1	1	0	1
RABIF	1	0	0	0	0	0	0	0	0
RABL2A	1	0	1	1	0	1	0	0	0
RABL2B	1	0	1	1	0	1	0	0	1
RABL3	1	0	1	1	0	1	1	0	1
RABL4	1	0	1	1	0	1	1	0	1
RAC2	0	0	0	0	0	1	1	0	1
RACGAP1	0	0	0	0	0	1	1	0	1
RAD1	1	0	1	1	0	1	1	0	1
RAD17	1	0	1	1	0	1	1	0	1
RAD18	1	0	1	1	0	1	1	0	1
RAD21	0	0	1	0	0	1	0	0	1
RAD23B	1	0	1	1	0	1	1	0	1
RAD50	0	0	0	0	0	1	0	0	1
RAD51	1	0	1	1	0	1	1	0	1
RAD51AP1	1	0	1	1	0	1	1	0	1
RAD51C	1	0	1	1	0	1	1	0	1
RAD51L1	1	0	1	1	0	1	1	0	1
RAD51L3	0	1	0	0	1	0	0	0	0
RAD52	0	0	0	0	0	1	0	0	0
RAD54L	0	0	1	1	0	1	1	0	1
RAF1	1	0	1	1	0	1	1	0	1
RAG1AP1	1	0	1	1	0	1	0	0	1
RAGE	0	0	0	0	0	0	0	0	1
RAI1	0	1	0	0	0	0	0	0	0
RAI14	1	1	1	1	1	1	0	1	1
RALA	1	0	1	1	0	1	1	0	1
RALB	1	0	1	0	0	1	1	0	1
RALBP1	0	0	1	0	0	1	0	0	1
RALGDS	0	0	0	0	0	1	0	0	0
RALGPS1	0	1	0	0	0	0	0	1	0
RALGPS2	0	0	0	0	0	0	1	0	1
RALY	1	0	1	1	0	1	1	0	1
RAMP1	0	1	1	0	1	0	0	1	0
RANBP1	1	0	1	1	0	1	0	0	1
RANBP10	1	0	1	1	0	1	0	0	1
RANBP3	0	0	0	0	0	1	0	0	0
RANBP6	1	0	1	1	0	1	1	0	1
RANBP9	1	0	1	1	0	1	1	0	1
RANGAP1	1	0	1	1	0	1	1	0	1
RAP1A	0	0	0	0	0	1	0	0	0
RAP1B	1	0	1	1	0	1	1	0	1

RAP1GAP	0	0	0	0	1	0	0	1	0
RAP1GDS1	1	0	0	1	0	1	0	0	1
RAP2A	1	0	1	1	0	1	1	0	1
RAP2B	1	0	1	1	0	1	1	0	1
RAP2C	1	1	1	1	1	1	1	1	1
RAPGEF1	1	0	1	1	0	1	1	0	1
RAPGEF3	0	0	0	0	0	0	0	0	1
RAPGEF6	1	0	1	1	0	1	1	0	1
RAPGEFL1	0	0	0	0	0	0	0	1	0
RAPH1	1	0	0	0	0	0	0	1	1
RARA	1	0	1	1	0	1	1	0	1
RARRES2	0	1	0	0	1	0	0	1	0
RARRES3	0	0	1	1	0	1	1	0	1
RARS	1	1	0	1	1	1	0	1	1
RASA2	0	0	1	1	0	1	1	0	1
RASAL1	0	0	0	0	0	0	0	1	0
RASAL2	1	0	1	1	0	1	1	1	1
RASD1	0	1	0	0	0	0	0	0	0
RASD2	0	1	0	0	1	0	0	1	0
RASGEF1A	0	1	0	0	1	0	0	1	0
RASGEF1B	1	0	0	1	0	0	0	0	1
RASGRF1	0	1	0	0	1	0	0	1	0
RASGRP1	0	1	0	0	1	0	0	1	0
RASGRP2	0	0	0	0	0	0	0	0	1
RASIP1	0	1	0	0	1	0	0	1	0
RASL10B	0	1	0	0	1	0	0	1	0
RASL11B	0	1	0	0	1	0	0	1	0
RASSF1	1	0	1	1	0	1	1	0	1
RASSF2	1	0	0	0	0	0	1	0	1
RASSF3	1	0	1	1	0	1	1	0	1
RASSF4	1	0	1	1	0	1	1	0	1
RASSF5	1	1	0	1	0	0	0	1	0
RASSF7	0	1	0	0	0	0	0	1	0
RASSF8	0	1	0	0	0	0	0	1	0
RAVER1	1	0	1	1	0	1	0	0	1
RAVER2	0	1	0	0	1	0	0	1	1
RB1	1	0	1	1	0	1	0	0	1
RB1CC1	1	0	1	1	0	1	1	0	1
RBBP4	1	0	1	1	0	1	1	0	1
RBBP5	1	0	1	1	0	1	1	0	1
RBBP6	0	0	1	0	0	1	0	0	1
RBBP7	0	1	1	0	1	1	1	1	1
RBBP8	1	0	1	1	0	1	1	0	1
RBBP9	1	0	1	1	0	1	1	0	1
RBED1	1	0	1	1	0	1	1	0	1
RBJ	1	0	0	0	0	0	1	0	1
RBKS	1	0	1	1	0	1	1	0	1
RBL2	0	0	0	0	0	1	0	0	0
RBM10	1	1	1	1	1	1	0	1	1
RBM11	0	1	1	0	0	1	0	1	1
RBM12	1	0	0	1	0	0	1	0	1
RBM12B	1	0	1	1	0	1	1	0	1
RBM13	1	0	1	1	0	1	0	0	1
RBM15	1	0	1	1	0	1	1	0	1
RBM16	1	0	1	1	0	1	1	0	1
RBM18	1	0	1	1	0	1	1	0	1
RBM22	0	0	1	1	0	1	1	0	1
RBM23	1	0	1	1	0	1	1	0	1
RBM25	1	0	1	1	0	1	1	0	1
RBM28	1	0	1	1	0	1	1	0	1
RBM3	0	0	0	0	1	1	0	0	1
RBM34	1	0	1	1	0	1	1	0	1

RBM35A	0	1	1	0	1	0	0	1	1
RBM4	1	0	1	1	0	1	1	0	1
RBM4B	1	0	1	1	0	1	1	0	1
RBM5	1	0	1	1	0	1	1	0	1
RBM7	1	0	1	1	0	1	1	0	1
RBM8A	1	0	1	1	0	1	1	0	1
RBMS1	1	1	1	0	1	1	1	1	1
RBMS2	1	0	1	1	0	1	1	0	1
RBMX	1	0	1	1	1	1	1	0	1
RBMX2	0	1	1	0	0	1	1	0	1
RBP1	0	1	1	0	1	1	0	1	0
RBP7	0	1	0	0	0	0	0	1	1
RBPMS2	0	1	0	0	1	0	0	1	0
RBX1	0	0	0	1	0	0	1	0	1
RCBTB1	0	0	1	0	0	1	1	0	1
RCBTB2	1	0	1	1	0	1	1	0	1
RCC2	1	1	0	1	1	1	0	1	1
RCE1	1	0	0	1	0	1	0	0	1
RCHY1	1	0	1	1	0	1	1	0	1
RCL1	0	1	1	0	0	1	0	1	1
RCN1	0	1	0	0	1	0	0	0	1
RCN2	0	0	0	1	0	1	0	0	1
RCOR1	1	0	0	0	0	1	1	0	1
RCOR3	1	0	1	1	0	1	1	0	1
RCP9	0	0	1	1	0	1	1	0	1
RCSD1	1	0	1	0	0	1	1	0	1
RDBP	1	0	1	1	0	1	1	0	1
RDH10	1	0	1	1	0	1	1	0	1
RDH11	1	0	1	1	0	1	0	0	1
RDH13	0	1	0	0	1	1	0	1	0
RDH8	0	1	1	0	1	1	0	1	0
RDHE2	0	1	0	0	1	0	0	1	0
RDM1	0	1	0	0	0	1	1	0	1
RDX	0	0	0	1	0	1	1	1	1
RECK	0	0	1	0	0	0	0	0	1
RECQL	1	0	1	1	0	1	1	0	1
RECQL4	1	0	1	1	0	1	0	0	1
RECQL5	1	0	1	1	0	1	1	0	1
REEP1	0	1	0	0	1	1	0	1	0
REEP3	1	0	1	1	0	1	1	0	1
REEP4	0	0	0	0	0	0	0	0	1
REEP5	0	0	1	1	0	1	1	0	1
REEP6	0	1	0	0	1	0	0	1	0
REL	0	0	0	1	0	0	1	0	1
RELA	1	0	0	1	0	1	1	0	1
REPIN1	1	0	1	1	0	1	1	0	1
REPS2	0	1	0	0	1	0	0	1	0
RER1	1	0	1	1	0	1	0	0	1
RERE	0	0	0	1	0	1	0	0	0
REST	0	0	0	1	0	1	0	0	1
RET	0	1	0	0	1	0	0	1	0
RETN	0	0	0	0	0	1	0	0	0
REV3L	1	0	1	1	0	0	1	0	1
REXO2	0	0	1	0	0	1	0	0	1
REXO4	1	0	0	1	0	1	1	0	1
RFC1	1	0	1	1	0	1	1	0	1
RFC2	0	0	1	0	0	0	0	0	1
RFC3	1	0	1	1	0	1	1	0	1
RFC4	1	0	0	1	0	1	1	0	1
RFC5	1	0	1	1	0	1	0	0	1
RFFL	1	0	0	1	0	1	1	0	1
RFK	1	0	1	1	0	1	1	0	1

RFNG	1	0	0	1	0	1	1	0	1
RFP	1	0	1	0	0	0	0	0	0
RFT1	1	0	1	1	0	1	1	0	1
RFWD2	1	0	1	1	0	1	1	0	1
RFWD3	1	0	1	1	0	1	1	0	1
RFX1	0	0	0	0	0	0	1	0	1
RFX2	0	0	0	1	0	1	0	0	0
RFX3	1	0	1	1	0	1	1	0	1
RFX4	0	1	1	0	1	0	0	1	0
RFX5	1	0	1	1	0	1	1	0	1
RFXANK	1	0	1	1	0	1	1	0	1
RFXAP	0	0	1	1	0	1	1	0	1
RG9MTD1	1	0	1	1	0	1	1	1	1
RG9MTD2	1	0	1	1	0	1	1	0	1
RGAG1	0	1	0	0	1	0	0	1	0
RGAG4	0	1	0	0	1	0	0	1	0
RGL1	1	0	1	1	0	1	1	1	1
RGL2	1	0	1	1	0	1	1	0	1
RGMA	0	1	0	0	1	1	0	1	1
RGMB	0	1	1	0	1	1	0	1	1
RGS1	1	0	1	1	0	1	1	0	0
RGS10	0	0	0	0	0	0	0	1	0
RGS11	0	1	0	0	1	0	0	1	0
RGS12	1	0	1	0	0	1	1	0	1
RGS14	0	0	1	0	0	0	0	0	0
RGS16	0	1	0	0	1	0	0	1	0
RGS19	1	1	1	1	1	1	0	1	1
RGS2	1	0	1	1	0	0	1	0	1
RGS20	0	1	1	0	1	1	0	1	1
RGS6	0	1	1	0	1	1	0	1	0
RHBDD2	0	0	0	0	0	1	0	0	1
RHBDD3	1	0	1	1	0	1	1	0	1
RHBDF1	0	1	0	0	1	0	0	1	0
RHBDF2	0	0	0	0	0	0	1	0	1
RHBDL3	0	1	0	0	1	1	0	1	1
RHBG	0	1	0	0	1	0	0	1	0
RHCG	0	1	0	0	1	0	0	1	0
RHEB	1	0	0	0	0	0	0	0	0
RHOA	1	0	1	1	0	1	1	0	1
RHOB	0	1	0	0	0	0	0	1	0
RHOBTB1	0	1	0	0	1	0	0	1	0
RHOBTB2	0	0	0	0	0	1	0	0	1
RHOBTB3	1	1	1	1	0	1	1	0	1
RHOC	0	1	0	0	1	0	0	1	0
RHOG	0	0	0	1	0	0	0	0	0
RHOQ	1	0	0	0	0	0	0	1	1
RHOT1	1	0	1	1	0	1	1	0	1
RHPN1	1	0	1	1	0	1	1	1	1
RIC8A	1	0	1	1	0	1	1	0	1
RIC8B	1	0	1	1	1	1	1	1	1
RICTOR	0	0	1	1	0	1	1	0	1
RIF1	0	0	0	0	0	1	0	0	1
RILP	0	0	0	0	0	0	0	0	1
RIMS4	0	1	0	0	1	0	0	1	0
RIN1	0	0	0	0	0	0	0	0	1
RIN2	0	1	0	1	0	0	0	0	0
RIN3	1	0	1	0	0	1	1	0	1
RINT1	1	0	1	1	0	1	1	0	1
RIOK1	1	0	1	1	0	1	1	0	1
RIOK2	1	0	1	1	0	1	1	0	1
RIPK1	1	0	0	0	0	0	1	0	0
RIPK2	1	0	1	1	0	1	1	0	1

RIPK3	0	0	0	0	0	0	0	0	1
RIPK4	1	1	1	1	1	1	1	1	1
RIPK5	1	0	1	1	0	1	1	0	1
RIT1	1	0	1	0	0	1	1	0	1
RLF	0	0	0	0	0	0	0	0	1
RLN1	0	1	0	0	1	1	0	1	1
RMND5A	1	0	0	0	0	0	0	0	0
RNASE1	1	0	1	0	0	0	0	0	0
RNASE2	0	0	0	0	0	0	1	0	1
RNASE3	0	0	0	0	0	0	1	0	1
RNASE4	1	0	1	1	0	1	1	0	1
RNASE6	1	0	1	1	0	1	1	0	1
RNASEH1	0	0	0	0	0	1	0	0	0
RNASEL	1	0	1	1	0	1	1	0	1
RNASEN	1	0	1	1	0	1	1	0	1
RND2	0	0	1	0	0	1	1	1	1
RND3	0	1	0	0	1	0	0	1	0
RNF103	1	0	0	0	0	0	1	0	1
RNF11	0	0	1	1	0	1	1	0	1
RNF111	1	0	1	1	0	1	1	0	1
RNF113A	1	1	1	1	0	1	0	1	1
RNF12	1	0	0	0	0	0	1	0	1
RNF121	1	0	1	1	0	1	0	0	1
RNF123	1	1	1	1	1	1	1	1	1
RNF125	0	0	0	1	0	1	1	0	1
RNF13	1	0	0	0	0	0	1	0	1
RNF135	1	0	1	1	0	1	1	0	1
RNF144	0	1	0	0	0	0	0	0	0
RNF146	1	0	1	1	0	1	1	0	1
RNF149	1	0	1	0	0	0	0	0	0
RNF157	0	1	1	0	1	1	0	1	0
RNF166	1	0	1	1	0	1	0	0	1
RNF167	1	0	1	1	0	1	1	0	1
RNF168	1	0	1	1	0	1	1	0	1
RNF170	1	0	1	1	0	1	1	0	1
RNF175	0	1	0	0	1	0	0	1	0
RNF180	0	1	1	0	1	0	0	1	0
RNF182	0	1	0	0	1	0	0	1	0
RNF185	0	0	1	0	0	0	0	1	0
RNF19	1	0	0	0	0	0	1	0	1
RNF20	1	0	1	1	0	1	1	0	1
RNF24	1	0	0	0	0	0	0	0	0
RNF25	1	0	1	1	0	1	1	0	1
RNF26	1	0	1	1	0	1	1	0	1
RNF31	1	0	1	1	0	1	1	0	1
RNF32	1	1	1	1	1	1	1	1	1
RNF38	1	0	1	1	0	1	1	0	1
RNF4	1	0	0	0	0	1	0	0	1
RNF40	1	0	1	1	0	1	1	0	1
RNF41	1	0	1	1	0	1	0	0	1
RNF44	1	0	1	1	0	1	1	0	1
RNF6	1	0	1	1	0	1	1	0	1
RNF7	0	0	0	0	0	0	1	0	0
RNGTT	1	0	0	0	0	1	0	0	1
RNMT	1	0	1	1	0	1	1	0	1
RNMTL1	1	0	1	1	0	1	1	0	1
RNPC2	1	0	1	1	0	1	1	0	1
RNPEPL1	1	0	1	1	0	1	1	0	1
RNPS1	1	1	1	0	0	1	1	1	1
ROBO3	0	1	1	0	1	0	0	1	0
ROBO4	0	0	0	0	1	0	0	0	0
ROCK1	1	0	1	1	0	1	1	0	1

ROCK2	1	0	1	1	0	1	1	0	1
ROD1	1	0	1	1	0	1	1	0	1
ROGDI	0	0	0	0	0	0	1	0	1
ROM1	1	0	1	1	0	1	0	0	1
ROPN1L	0	0	0	1	0	1	0	0	1
RORA	0	1	0	0	1	0	0	1	1
RP11-529I10.4	1	0	0	0	0	1	0	0	1
RP13-360B22.2	0	1	0	0	1	1	0	1	1
RP9	0	1	0	0	1	0	0	1	0
RPA1	1	0	1	1	0	1	1	0	1
RPA2	1	0	1	0	0	1	0	0	1
RPAP1	1	0	1	1	0	1	1	0	1
RPGR	0	1	0	0	1	1	0	0	0
RPH3A	0	1	0	0	1	0	0	1	0
RPH3AL	0	0	0	0	0	0	1	0	0
RPIA	1	0	1	1	0	1	0	0	1
RPL10A	1	0	0	1	0	1	1	0	1
RPL11	0	0	0	0	0	0	0	0	1
RPL12	1	0	1	1	0	1	1	0	1
RPL13	1	0	1	1	0	1	1	0	1
RPL13A	1	0	1	1	0	1	1	0	1
RPL14	1	0	1	1	0	1	1	0	1
RPL15	1	0	1	1	0	1	1	0	1
RPL17	0	0	0	0	0	0	0	0	1
RPL19	1	0	0	1	0	1	1	0	1
RPL22	1	0	0	0	1	0	0	1	1
RPL23	1	0	1	1	0	1	1	0	1
RPL23A	1	0	1	1	0	1	0	0	1
RPL26	1	0	1	1	0	1	0	0	1
RPL26L1	1	0	1	1	0	1	0	0	1
RPL27	1	0	0	1	0	1	1	0	1
RPL27A	1	0	0	1	0	1	1	0	1
RPL28	1	0	1	1	0	1	1	0	1
RPL29	0	0	1	0	0	0	1	0	1
RPL3	1	0	1	1	0	1	1	0	1
RPL30	1	0	1	1	0	1	1	0	1
RPL31	0	0	0	0	0	0	0	0	1
RPL32	1	0	1	1	0	1	1	0	1
RPL34	1	0	1	1	0	1	1	0	1
RPL35A	1	0	1	1	0	1	1	0	1
RPL36A	1	1	1	1	1	1	1	0	1
RPL36AL	1	0	1	1	0	1	1	0	1
RPL37	1	0	0	1	0	1	1	0	1
RPL37A	1	0	1	1	0	1	1	0	1
RPL38	1	1	0	1	1	1	0	1	1
RPL39	1	1	1	0	1	1	0	1	1
RPL4	1	0	1	1	0	1	1	0	1
RPL41	1	0	1	1	0	1	1	0	1
RPL5	1	0	0	0	0	1	1	0	1
RPL6	0	0	1	1	0	1	0	0	1
RPL7	1	0	1	1	0	1	1	0	1
RPL7A	1	0	1	1	0	1	1	0	1
RPL7L1	1	0	1	1	0	1	1	0	1
RPL8	0	0	0	1	0	1	0	1	0
RPL9	1	0	1	1	0	1	1	0	1
RPLP0	1	0	1	0	0	1	0	0	1
RPLP1	1	0	1	1	0	1	1	0	1
RPLP2	1	0	1	1	0	1	1	0	1
RPN1	1	0	1	0	0	1	1	0	1
RPN2	1	0	1	1	0	1	1	0	1
RPP25	0	1	0	0	1	1	0	1	1
RPP30	0	1	1	0	0	1	1	0	1

RPP38	1	1	1	1	1	1	1	1	1
RPP40	1	0	1	1	0	0	1	0	1
RPRM	0	1	0	0	1	0	0	1	0
RPRML	0	1	0	0	1	0	0	1	0
RPS10	0	0	0	1	0	0	0	0	0
RPS11	1	0	1	1	0	1	1	0	1
RPS12	1	0	1	1	0	1	1	0	1
RPS13	1	0	1	1	0	1	0	0	1
RPS14	1	0	1	1	0	1	1	0	1
RPS15A	0	0	0	1	0	1	0	0	1
RPS16	1	0	1	1	0	1	1	0	1
RPS17	1	0	1	1	0	1	0	0	1
RPS18	1	0	1	1	0	1	1	0	1
RPS19	0	0	0	0	0	0	0	0	1
RPS19BP1	1	0	0	0	0	1	0	0	1
RPS20	1	0	0	1	0	0	0	0	0
RPS21	1	0	1	1	0	1	0	0	1
RPS23	1	0	0	1	0	1	0	0	1
RPS24	1	0	1	1	0	1	1	0	1
RPS25	1	0	1	1	0	1	1	0	1
RPS26	1	0	1	1	0	1	1	0	1
RPS27	1	0	1	1	0	1	1	0	1
RPS27A	1	0	1	1	0	1	1	0	1
RPS27L	1	0	1	1	0	1	1	0	1
RPS28	1	0	1	1	0	1	1	0	1
RPS29	1	0	1	1	0	1	1	0	1
RPS3	1	0	0	0	0	0	0	0	0
RPS3A	1	0	1	1	0	1	1	0	1
RPS4X	0	1	0	1	1	0	1	1	1
RPS5	1	0	1	1	0	1	1	0	1
RPS6	1	0	1	1	0	1	1	0	1
RPS6KA1	1	0	1	1	0	1	1	0	1
RPS6KA2	1	1	1	1	1	1	1	1	1
RPS6KA3	0	0	0	0	0	1	0	0	0
RPS6KB1	1	0	1	1	0	1	1	0	1
RPS6KB2	1	0	0	0	0	1	1	0	1
RPS6KC1	0	0	0	0	0	0	0	0	1
RPS6KL1	0	1	0	0	1	0	0	1	0
RPS7	1	0	0	0	0	0	0	0	0
RPS8	1	0	1	1	0	1	1	0	1
RPS9	1	0	1	1	0	1	1	0	1
RPSA	0	0	0	0	0	1	0	0	1
RPUSD2	1	1	1	1	0	1	1	1	1
RPUSD3	1	0	1	0	0	1	0	0	1
RPUSD4	1	0	1	1	0	1	1	0	1
RQCD1	1	0	1	1	0	1	1	0	1
RRAGA	1	0	1	1	0	1	0	0	1
RRAGB	0	0	1	1	0	1	0	0	1
RRAGC	1	0	1	1	0	1	1	0	1
RRAGD	0	0	1	1	0	1	0	0	1
RRAS	1	0	1	1	1	1	1	0	1
RRBP1	0	0	0	1	0	1	0	0	1
RREB1	1	0	0	1	0	0	1	0	1
RRM1	1	0	0	1	0	1	1	0	1
RRM2	1	0	1	1	0	1	1	0	1
RRM2B	1	0	1	1	0	1	1	0	1
RRN3	1	0	1	1	0	1	1	0	1
RRS1	1	0	1	1	0	1	0	0	1
RSAD2	1	0	1	1	0	1	1	0	1
RSBN1	0	0	0	0	0	0	1	0	1
RSBN1L	1	0	1	1	0	1	1	0	1
RSL1D1	1	0	1	1	0	1	1	0	1

RSPO3	0	1	1	0	1	1	0	1	1
RSPRY1	1	0	1	1	0	1	1	0	1
RSRC1	0	1	1	0	1	1	1	1	1
RSU1	1	0	1	1	0	1	1	1	1
RTCD1	1	0	1	1	0	1	1	0	1
RTEL1	0	1	0	1	1	1	0	1	1
RTF1	1	0	1	1	0	1	1	0	1
RTKN	0	0	0	0	0	0	0	1	0
RTN3	0	0	1	0	0	0	0	0	1
RTN4	1	0	1	1	0	1	1	0	1
RTN4IP1	1	0	1	1	0	1	1	0	1
RTN4RL2	0	1	1	0	1	1	0	1	1
RTP4	1	0	1	1	0	1	1	0	1
RTTN	1	0	1	1	0	1	1	0	1
RUFY1	1	0	0	0	0	0	1	0	1
RUFY2	1	0	1	1	0	1	0	0	0
RUNDC1	1	0	1	1	0	1	1	0	1
RUNDC2A	1	0	0	0	0	1	0	0	0
RUNX1	1	0	1	1	0	1	1	0	1
RUNX2	1	1	1	1	1	1	1	1	1
RUTBC1	1	0	1	1	0	1	1	0	1
RUVBL1	0	0	1	0	0	1	0	0	0
RUVBL2	1	0	1	1	0	1	1	0	1
RWDD1	1	0	1	1	0	1	1	0	1
RWDD3	0	1	0	0	1	0	0	1	0
RWDD4A	1	0	1	1	0	1	1	0	1
RXRB	1	0	1	1	0	1	1	0	1
RXRG	0	1	0	0	1	0	0	1	0
RYBP	1	0	1	1	0	1	1	0	1
RYK	1	0	0	0	0	0	0	0	0
RYR1	0	1	0	0	1	0	0	1	0
RETSAT	1	0	1	1	0	1	1	0	1
S100A10	0	1	0	0	1	0	0	1	0
S100A11	1	0	0	1	0	0	1	0	1
S100A12	0	0	0	0	0	0	1	0	1
S100A13	1	0	1	1	0	1	1	1	1
S100A4	0	0	0	0	0	1	0	0	1
S100A5	0	0	1	1	0	1	1	0	1
S100A6	0	0	0	0	0	1	0	0	1
S100A8	0	0	0	1	0	1	1	0	1
S100A9	0	0	0	1	0	1	1	0	1
S100PBP	1	0	1	1	0	1	1	0	1
S100Z	0	0	0	0	0	0	1	0	1
SAAL1	0	1	0	0	0	1	0	1	1
SAC3D1	1	0	1	1	0	1	1	0	1
SACM1L	1	0	1	1	0	1	1	0	1
SAE1	1	0	1	1	0	1	1	0	1
SAFB	1	0	1	1	0	1	1	0	1
SAFB2	1	0	1	1	0	1	1	0	1
SAMD1	0	0	0	0	0	1	0	0	0
SAMD11	0	1	0	0	1	0	0	0	0
SAMD13	0	0	0	0	0	1	0	0	1
SAMD14	0	0	0	0	0	0	0	1	0
SAMD4A	0	0	1	0	0	1	0	1	1
SAMD4B	1	0	1	1	0	1	1	0	1
SAMD8	1	0	0	1	0	1	1	0	1
SAMD9	1	0	1	1	0	1	1	0	1
SAMD9L	1	0	1	1	0	1	0	0	1
SAMHD1	1	0	1	1	0	1	0	0	1
SAMM50	1	0	1	1	0	1	1	0	1
SAMSN1	1	0	0	1	0	1	1	0	1
SAP130	1	0	1	0	0	1	0	0	1

SAP18	1	0	1	1	0	1	1	0	1
SAP30	1	0	1	1	0	1	1	0	1
SAP30BP	1	0	1	1	0	1	1	0	1
SAP30L	1	0	1	1	0	1	1	0	1
SAPS1	1	0	1	0	0	0	0	0	1
SAPS3	1	0	1	0	0	1	0	0	1
SAR1A	0	0	0	0	0	1	0	0	0
SAR1B	1	0	0	1	0	1	1	0	1
SARM1	0	0	0	0	0	0	1	1	0
SARS	1	0	1	0	0	1	1	0	1
SARS2	1	0	1	1	0	1	1	0	1
SART3	1	0	1	1	0	1	1	0	1
SAS10	1	0	1	1	0	1	1	0	1
SASH1	1	1	0	1	1	1	1	1	1
SASS6	1	0	1	1	0	1	1	0	1
SAT2	0	0	0	0	0	1	0	0	1
SATB1	1	0	1	1	0	1	1	0	1
SATB2	1	1	1	0	1	1	1	1	1
SATL1	0	1	0	0	0	0	0	0	0
SAV1	0	0	1	0	0	1	0	0	1
SBDS	1	0	1	1	0	1	1	0	1
SBF2	1	0	1	1	0	1	1	0	1
SC4MOL	0	0	1	0	0	1	0	0	1
SC5DL	1	0	1	1	0	1	1	0	1
SC65	0	1	0	0	1	0	0	1	0
SCAMP1	1	0	1	1	0	1	1	0	1
SCAMP2	0	0	0	0	0	1	1	0	1
SCAMP3	1	0	1	1	0	1	1	0	1
SCAMP4	1	0	0	1	0	1	0	0	0
SCAND1	1	0	1	1	0	1	1	0	1
SCAND2	1	0	1	1	0	1	1	0	1
SCAP	1	0	0	1	0	0	0	0	0
SCARB2	0	0	1	1	0	1	1	0	1
SCARF1	0	0	0	0	0	1	0	0	1
SCD	1	0	1	1	0	1	0	1	1
SCFD2	1	1	1	1	0	1	1	0	1
SCG5	0	0	0	0	0	0	0	1	0
SCGB1C1	0	1	0	0	1	0	1	1	0
SCGN	0	1	0	0	1	0	0	1	0
SCLY	1	0	1	1	0	1	0	0	1
SCMH1	0	0	0	0	0	0	1	0	0
SCML1	0	1	0	0	0	0	0	0	1
SCML2	0	1	0	0	1	0	0	1	0
SCN8A	0	1	0	0	1	0	0	1	0
SCNM1	1	0	1	1	0	1	1	0	1
SCNN1B	0	1	0	0	1	0	0	1	0
SCO1	1	0	1	1	0	1	1	0	1
SCO2	0	0	0	0	0	0	0	0	1
SCP2	1	1	1	1	0	1	1	1	1
SCPEP1	0	0	0	0	0	1	1	0	1
SCRN3	1	0	1	1	0	1	1	0	1
SCRT2	0	0	0	0	0	0	0	1	0
SCTR	0	1	0	0	1	0	0	1	0
SCYE1	1	0	1	1	0	1	1	0	1
SCYL1BP1	1	0	1	1	0	1	1	1	1
SCYL2	1	0	1	1	0	1	1	0	1
SCYL3	1	0	1	1	0	1	1	0	1
SDC2	0	1	0	0	0	0	0	1	0
SDC4	1	0	1	1	0	1	1	0	1
SDCBP	1	0	1	1	0	1	1	1	1
SDCCAG1	1	0	1	1	0	1	1	0	1
SDCCAG10	1	0	1	1	0	1	1	0	1

SDCCAG3	1	0	1	1	0	1	0	0	1
SDCCAG8	1	0	1	1	0	1	1	0	1
SDF2	1	0	1	1	0	1	1	0	1
SDF2L1	1	0	0	1	0	1	1	0	1
SDHA	1	0	1	1	0	1	1	1	1
SDHB	0	0	0	1	0	1	0	0	1
SDHC	1	0	1	1	0	1	1	0	1
SDHD	1	0	1	1	0	1	1	0	1
SDK1	0	1	0	0	1	0	0	1	0
SDPR	0	0	0	1	1	1	1	0	1
SDS	0	0	0	0	0	0	1	0	0
SEC14L1	1	0	0	1	0	1	0	0	1
SEC14L2	0	1	1	0	1	0	0	1	0
SEC23A	1	0	0	1	0	1	1	0	1
SEC23B	0	0	0	1	0	1	1	0	1
SEC23IP	1	0	1	0	0	1	1	0	1
SEC24B	1	0	1	1	0	1	1	0	1
SEC24C	1	0	1	1	0	1	1	0	1
SEC24D	1	0	1	0	0	1	0	0	1
SEC61A1	1	0	1	1	0	1	1	0	1
SEC61B	1	0	1	1	0	1	1	0	1
SEC61G	1	0	1	1	0	1	1	1	1
SEC63	1	1	0	0	0	0	0	1	0
SECISBP2	1	0	1	1	0	1	1	0	1
SECTM1	1	0	1	1	0	1	1	0	1
SEH1L	1	0	1	1	0	1	1	0	1
SEL1L	1	0	1	0	0	1	1	0	1
SELI	1	0	1	1	0	1	1	0	1
SELM	0	0	0	1	1	0	0	0	0
SELS	0	0	0	1	0	1	0	0	1
SELT	1	0	0	1	0	1	0	0	1
SELV	0	1	0	0	1	0	0	1	0
SEMA3A	1	0	1	0	0	0	1	0	1
SEMA4B	0	0	0	0	0	0	0	0	1
SEMA4G	0	0	0	0	0	0	0	1	0
SEMA5B	0	1	0	0	1	1	1	1	1
SEMA6A	0	1	1	0	1	1	0	1	1
SEMA6B	0	0	1	1	1	1	0	0	0
SENP1	1	0	1	1	0	1	1	1	1
SENP2	1	0	0	0	0	1	0	0	1
SENP3	1	0	1	1	0	1	1	0	1
SENP5	0	0	0	1	0	1	0	0	1
SENP6	1	0	0	1	0	1	1	0	1
SENP7	1	0	1	1	0	1	1	0	1
SENP8	1	0	1	1	0	1	1	0	1
SEPHS1	1	0	1	1	0	1	1	0	1
SEPHS2	1	0	1	1	0	1	1	0	1
SEPP1	1	0	0	1	0	1	1	0	0
SEPX1	0	0	0	0	0	1	0	0	1
SERAC1	0	0	0	0	0	1	1	0	1
SERBP1	1	0	1	1	0	1	1	0	1
SERF2	1	0	1	1	0	1	1	0	1
SERGEF	1	0	1	1	1	1	1	1	1
SERINC1	1	0	1	1	0	1	1	0	1
SERINC2	0	1	0	0	0	0	0	1	0
SERINC3	1	0	1	1	0	1	1	0	1
SERINC4	1	0	1	1	0	1	1	0	1
SERINC5	1	0	1	1	0	0	1	0	1
SERP1	1	0	1	1	0	1	1	0	1
SERPINA1	0	0	0	1	0	1	1	0	1
SERPINA12	0	1	0	0	0	0	0	0	0
SERPINA6	0	1	0	0	1	0	0	0	0

SERPINB1	0	0	0	1	0	0	0	0	1
SERPINB2	0	0	0	0	0	0	1	0	0
SERPINB8	1	0	1	1	0	1	1	0	1
SERPINB9	0	0	0	0	0	0	0	1	0
SERPIND1	0	0	0	1	0	0	0	0	0
SERPINE1	1	0	1	0	0	1	0	1	0
SERPINE2	0	1	0	0	1	0	0	1	0
SERPINI1	1	0	1	1	0	1	1	0	1
SERTAD1	1	0	1	1	0	1	1	0	1
SERTAD2	1	0	1	1	0	1	1	0	1
SERTAD3	1	0	1	1	0	1	1	0	1
SERTAD4	0	1	0	0	1	0	0	1	0
SESN1	1	0	1	1	0	1	1	0	1
SESN2	1	0	1	1	0	1	1	0	1
SESN3	1	0	1	1	0	1	1	0	1
SET	0	0	0	0	0	1	0	0	1
SETBP1	0	1	0	0	1	0	0	1	0
SETD1A	0	0	0	1	0	1	0	0	0
SETD3	1	0	1	1	0	1	1	0	1
SETD5	1	0	1	1	0	1	1	0	0
SETD6	0	0	0	1	0	1	0	0	1
SETDB1	1	0	1	1	0	1	1	0	1
SETDB2	1	0	1	1	0	1	0	0	1
SEZ6L	0	0	0	0	0	0	0	1	0
SF1	0	0	0	1	0	0	0	0	1
SF3A1	1	0	1	1	0	1	1	0	1
SF3A2	1	0	1	1	0	1	1	0	1
SF3A3	0	0	0	0	0	1	0	0	1
SF3B1	1	0	1	1	0	1	1	0	1
SF3B14	1	0	1	1	0	1	1	0	1
SF3B2	0	1	0	0	1	0	0	1	0
SF3B3	1	0	1	1	0	1	1	0	1
SF3B4	1	0	1	1	0	1	1	0	1
SF3B5	1	0	1	1	0	1	1	0	1
SF4	1	0	1	1	0	1	1	0	1
SFI1	1	0	1	1	0	1	1	0	1
SFMBT2	0	1	0	0	1	0	0	1	1
SFPQ	1	0	1	1	0	1	1	0	1
SFRP4	0	1	0	0	1	0	0	1	1
SFRS1	1	0	1	0	0	1	1	0	1
SFRS10	1	0	0	1	0	1	1	0	1
SFRS11	1	0	1	1	0	1	1	0	1
SFRS14	1	0	1	1	0	1	1	0	1
SFRS15	1	0	1	1	0	1	1	0	1
SFRS2	1	0	1	1	0	1	1	0	1
SFRS3	1	0	0	1	0	0	1	0	1
SFRS5	1	0	0	1	0	1	1	0	1
SFRS6	1	0	0	1	0	1	1	0	1
SFRS7	0	0	0	1	0	1	0	0	1
SFRS8	0	0	0	1	0	1	0	0	1
SFRS9	1	0	1	1	0	1	1	0	1
SFT2D1	1	0	1	1	0	1	1	0	1
SFT2D2	0	0	0	0	0	1	0	0	1
SFXN1	0	1	0	0	1	0	0	1	1
SFXN2	1	0	1	1	0	1	1	0	1
SFXN3	1	0	1	1	0	1	1	0	1
SFXN4	0	0	0	0	0	1	1	0	1
SFXN5	1	0	1	0	0	1	1	0	1
SGCB	0	1	0	0	1	1	0	1	1
SGK	1	0	1	1	0	1	1	0	1
SGOL1	1	0	1	1	0	1	1	0	1
SGOL2	1	0	1	1	0	1	1	0	1

SGPP1	0	1	1	0	0	0	0	1	1
SGPP2	0	1	1	0	1	1	0	1	1
SGSH	1	0	1	0	0	0	0	1	1
SGTB	1	0	1	1	0	1	1	0	1
SH2D1A	0	1	0	0	0	0	0	0	0
SH2D1B	0	0	0	0	1	0	1	0	0
SH2D3C	1	0	1	1	0	1	1	0	1
SH2D4A	0	1	0	0	1	0	0	1	0
SH3BGR	1	1	1	1	0	1	1	1	1
SH3BGRL	1	1	1	1	0	1	1	0	1
SH3BGRL3	0	0	0	1	0	0	0	0	1
SH3BP1	0	0	1	0	0	1	1	0	1
SH3BP4	0	1	1	0	1	1	0	1	1
SH3BP5	0	1	0	0	0	0	0	1	0
SH3BP5L	1	0	1	1	0	1	1	0	1
SH3GL1	1	0	1	1	0	1	1	0	1
SH3GLB1	1	0	1	1	0	1	1	0	1
SH3PX3	1	0	1	1	0	1	1	0	1
SH3PXD2A	0	0	0	0	0	1	1	1	0
SH3PXD2B	0	1	0	0	1	0	0	1	0
SH3RF1	0	1	0	0	0	1	1	1	1
SH3RF2	0	1	0	0	1	0	0	1	0
SH3YL1	1	0	1	1	0	1	1	1	1
SHANK1	0	1	0	0	1	0	0	1	0
SHARPIN	1	0	1	1	0	1	1	0	1
SHB	0	0	1	0	0	0	1	0	1
SHC1	1	0	1	1	0	1	1	0	1
SHCBP1	0	0	1	0	0	1	1	0	1
SHFM1	1	0	1	1	0	1	1	0	1
SHH	0	1	1	0	1	0	0	1	1
SHMT1	1	0	0	0	0	1	0	0	0
SHMT2	1	1	1	1	1	1	1	1	1
SHOX2	0	1	1	0	1	1	1	1	1
SHPRH	1	0	0	1	0	1	0	0	1
SHQ1	1	1	1	1	1	1	1	0	1
SIAE	1	0	1	1	0	1	1	0	1
SIAH1	1	0	1	1	0	1	1	0	1
SIAH2	1	0	1	1	0	1	1	0	1
SIDT1	1	1	1	1	1	1	1	1	1
SIDT2	1	0	1	1	0	1	1	0	1
SIGLEC10	0	0	0	0	0	0	0	0	1
SIGLEC11	0	0	0	0	0	1	0	0	1
SIGLEC12	0	0	0	0	0	1	0	0	1
SIGLEC7	1	0	1	1	0	1	1	0	1
SIGLEC9	1	0	1	1	0	1	1	0	1
SIKE	1	1	1	1	1	1	1	1	1
SIL1	1	0	1	1	0	1	1	0	1
SILV	1	0	1	1	0	1	1	0	1
SIN3A	1	0	1	1	0	1	1	0	1
SIN3B	0	0	0	0	0	1	0	1	0
SIP1	1	0	1	1	0	1	1	0	1
SIPA1	1	0	1	0	0	1	0	0	0
SIPA1L3	0	0	0	0	0	0	1	1	1
SIRPA	1	0	1	1	0	1	1	0	1
SIRPB1	1	0	0	1	0	1	0	0	1
SIRT1	1	0	1	1	0	1	1	0	1
SIRT2	1	0	1	1	0	1	1	0	1
SIRT5	1	0	1	1	0	1	1	0	1
SIRT6	1	0	1	0	0	1	0	0	0
SIX3	0	1	1	0	1	0	0	1	1
SIX5	0	0	1	0	0	1	0	1	1
SKI	0	0	0	0	0	0	0	1	0

SKIL	1	0	1	1	0	1	1	0	1
SKIP	1	1	0	0	1	0	0	1	0
SKIV2L	1	0	1	1	0	1	1	0	1
SKIV2L2	1	0	1	1	0	1	1	0	1
SKP2	1	0	1	1	0	1	1	0	1
SLA	1	0	1	1	0	1	1	0	1
SLAMF1	0	0	0	0	0	0	1	0	0
SLAMF6	0	0	0	0	0	1	0	0	0
SLAMF7	0	0	1	0	0	0	0	0	0
SLAMF8	1	0	1	1	0	1	0	0	0
SLBP	0	0	0	1	0	1	0	0	1
SLC11A1	0	0	0	1	0	0	0	0	1
SLC11A2	1	0	0	0	0	0	0	0	0
SLC12A2	1	0	1	1	0	1	1	0	1
SLC12A6	1	0	1	1	0	1	1	0	1
SLC12A7	0	1	0	0	1	0	0	1	0
SLC12A8	0	1	0	0	1	0	0	1	0
SLC13A3	0	1	0	1	1	0	1	1	1
SLC15A3	1	0	0	1	0	0	0	0	1
SLC16A10	0	1	1	0	1	1	0	1	1
SLC16A12	0	1	0	0	1	0	0	1	0
SLC16A2	0	1	1	0	1	1	0	1	1
SLC16A5	0	0	1	0	0	0	0	0	1
SLC16A6	1	0	1	1	0	1	1	0	1
SLC16A8	0	1	0	0	1	0	0	1	0
SLC17A5	1	0	1	1	0	1	1	0	1
SLC18A2	0	1	0	0	1	0	0	1	0
SLC19A2	1	0	1	1	0	1	1	0	1
SLC1A2	0	1	1	0	1	1	0	1	1
SLC1A3	1	0	1	1	0	1	1	0	1
SLC1A4	0	0	0	1	0	1	0	0	1
SLC1A5	1	0	1	1	0	1	0	0	1
SLC20A1	1	0	1	1	0	1	1	0	1
SLC20A2	1	0	1	1	0	1	1	0	1
SLC22A15	1	0	1	1	0	1	0	0	1
SLC22A17	0	1	1	0	1	1	0	1	0
SLC22A18	0	0	0	0	0	1	0	0	1
SLC22A4	1	0	0	1	0	1	1	0	1
SLC22A5	0	0	1	1	0	1	1	0	1
SLC22A7	0	1	0	0	1	0	0	0	0
SLC23A1	0	0	0	0	0	0	1	0	1
SLC23A2	1	0	0	0	0	0	0	0	0
SLC24A2	0	1	1	0	1	0	0	1	1
SLC24A3	0	1	0	0	1	0	0	1	0
SLC24A4	0	1	0	0	1	0	0	1	0
SLC24A6	0	0	1	0	0	0	0	0	0
SLC25A10	0	0	1	0	0	0	1	0	1
SLC25A11	1	0	1	1	0	1	1	0	1
SLC25A12	1	0	0	1	0	1	1	0	1
SLC25A13	0	0	0	0	0	0	0	0	1
SLC25A14	0	0	1	0	0	1	0	0	1
SLC25A17	1	0	0	1	0	1	1	0	1
SLC25A19	1	0	0	1	0	1	1	0	1
SLC25A20	0	0	0	0	0	0	0	0	1
SLC25A22	0	0	0	0	0	1	0	0	0
SLC25A23	0	1	1	1	1	1	1	1	1
SLC25A24	1	0	1	1	0	1	1	0	1
SLC25A25	1	0	1	1	0	1	1	0	1
SLC25A28	1	0	1	1	0	1	1	0	1
SLC25A29	0	0	0	1	0	0	0	0	1
SLC25A3	0	0	1	1	0	1	1	0	1
SLC25A30	0	0	1	0	0	1	0	0	1

SLC25A32	1	0	1	1	0	1	1	0	1
SLC25A35	1	0	1	1	0	1	1	0	1
SLC25A36	0	0	1	0	0	1	0	0	1
SLC25A5	0	1	0	0	1	0	0	1	0
SLC26A10	0	1	0	0	1	0	0	1	0
SLC26A2	0	0	0	0	0	0	0	0	1
SLC26A5	0	1	0	0	1	0	0	1	0
SLC26A6	1	0	1	1	0	1	0	0	1
SLC26A8	0	0	0	0	0	0	1	0	0
SLC27A1	0	0	0	0	0	0	0	0	1
SLC27A3	0	0	0	0	0	0	0	0	1
SLC27A4	0	0	0	0	0	0	0	0	1
SLC27A6	0	1	0	0	1	0	0	1	0
SLC29A1	0	0	0	0	0	0	0	0	1
SLC29A2	1	1	0	0	1	0	0	1	0
SLC2A1	0	1	1	0	1	1	0	1	1
SLC2A11	1	0	1	1	0	1	1	1	1
SLC2A14	0	1	0	0	1	0	0	1	0
SLC2A3	1	0	0	1	0	1	1	0	1
SLC2A4RG	0	1	0	0	1	0	0	1	0
SLC2A5	1	0	1	1	0	1	1	0	1
SLC2A6	0	0	0	0	1	1	0	1	1
SLC2A8	0	1	1	1	1	1	0	1	1
SLC2A9	1	0	0	0	0	1	1	0	1
SLC30A1	0	0	0	0	0	1	0	0	0
SLC30A3	0	1	1	0	1	1	0	1	1
SLC30A4	0	1	0	0	1	0	0	1	1
SLC30A6	1	0	1	1	0	1	1	0	1
SLC30A7	1	0	1	1	0	1	1	0	1
SLC30A9	1	0	1	1	0	1	1	0	1
SLC31A1	1	0	1	1	0	1	1	0	1
SLC31A2	0	0	0	1	0	1	0	0	0
SLC33A1	1	0	1	1	0	1	1	0	1
SLC35A1	1	0	1	0	0	1	1	0	1
SLC35A2	0	1	0	0	1	1	0	0	1
SLC35A3	0	0	1	1	0	1	1	0	1
SLC35A4	1	0	1	1	0	1	1	0	1
SLC35A5	1	0	1	1	0	1	1	0	1
SLC35B1	1	0	1	1	0	1	1	0	1
SLC35B2	0	0	0	0	0	1	0	0	1
SLC35B3	1	0	1	1	0	1	1	0	1
SLC35B4	1	0	0	1	0	1	1	0	1
SLC35C2	0	0	0	0	0	1	1	0	1
SLC35D3	0	1	1	0	1	1	1	1	1
SLC35E1	1	0	1	1	0	1	0	0	1
SLC35E2	0	0	0	0	0	1	0	0	0
SLC35E3	1	0	1	1	0	1	1	0	1
SLC35F2	1	1	1	1	1	1	1	1	1
SLC35F3	0	1	0	0	1	0	0	1	0
SLC36A1	1	0	0	0	0	0	0	0	1
SLC36A4	1	0	1	1	0	1	1	0	1
SLC37A3	0	0	0	0	1	0	0	0	0
SLC38A1	0	1	1	0	1	1	0	1	1
SLC38A2	1	1	1	1	0	1	1	0	1
SLC38A5	0	1	0	0	0	0	0	0	0
SLC38A6	1	0	1	1	0	1	1	0	1
SLC39A1	1	0	1	1	0	1	1	0	1
SLC39A10	1	0	0	1	0	1	1	0	1
SLC39A11	0	0	0	1	0	0	0	0	1
SLC39A14	0	1	1	0	1	0	0	1	0
SLC39A3	1	1	1	1	1	1	1	0	1
SLC39A6	1	0	1	1	0	1	1	0	1

SLC39A7	1	0	1	1	0	1	1	0	1
SLC39A8	1	0	1	1	0	1	1	0	1
SLC39A9	1	0	1	1	0	1	1	0	1
SLC3A2	1	0	1	1	0	1	1	0	1
SLC40A1	0	1	0	0	1	1	0	1	1
SLC43A1	0	1	1	0	0	1	0	0	1
SLC43A3	1	0	1	1	0	1	1	0	1
SLC44A1	1	0	1	0	0	0	1	0	1
SLC45A3	0	1	0	0	1	1	0	1	0
SLC4A11	0	1	0	0	1	0	0	1	0
SLC4A1AP	1	0	1	1	0	1	1	0	1
SLC4A2	1	0	1	1	0	1	1	0	1
SLC4A7	0	0	0	0	0	0	1	0	0
SLC4A8	0	1	0	0	1	0	0	1	0
SLC5A2	1	1	0	0	1	0	0	0	0
SLC5A6	1	0	1	1	0	1	1	0	1
SLC5A8	0	1	0	0	1	0	0	1	0
SLC6A12	0	0	0	0	0	1	0	0	1
SLC6A13	0	1	0	0	0	0	0	1	0
SLC6A8	0	1	0	0	0	0	0	1	0
SLC6A9	0	1	0	0	0	0	0	1	0
SLC7A1	0	1	0	0	0	0	0	1	0
SLC7A10	0	1	1	0	1	1	0	1	1
SLC7A11	1	0	1	1	0	1	1	0	0
SLC7A4	0	1	0	0	1	0	0	1	0
SLC7A6	1	0	0	0	0	0	0	0	1
SLC7A6OS	1	0	1	1	0	1	1	0	1
SLC7A7	0	0	0	0	0	0	0	0	1
SLC7A8	1	0	1	1	0	1	1	1	1
SLC9A1	0	0	0	0	0	1	1	0	1
SLC9A5	0	0	0	0	0	0	0	1	1
SLC9A6	1	1	1	1	0	1	1	0	1
SLC9A7	0	1	0	0	1	0	0	1	0
SLC9A9	1	0	1	1	0	1	1	0	1
SLCO2A1	0	1	0	0	1	0	0	1	0
SLCO2B1	1	0	1	1	0	1	0	0	0
SLCO3A1	1	0	1	1	0	1	1	0	1
SLCO4A1	0	1	0	0	1	0	0	1	0
SLCO4C1	0	0	0	0	0	0	0	0	1
SLCO5A1	0	1	1	0	1	1	0	1	1
SLFN11	1	0	0	1	0	1	1	0	1
SLFN12	1	0	1	0	0	1	1	0	1
SLFN13	0	1	0	0	1	0	1	1	1
SLIC1	1	0	1	1	0	1	1	0	1
SLIT2	0	1	1	0	1	0	0	1	1
SLIT3	0	1	1	0	1	0	0	1	0
SLITRK1	0	1	0	0	1	0	0	1	0
SLITRK2	0	1	0	0	1	0	0	1	0
SLITRK4	0	1	0	0	0	1	0	0	1
SLK	1	0	1	1	0	1	1	0	1
SLMAP	1	0	1	1	0	1	1	0	1
SLTM	1	0	1	1	0	1	1	0	1
SLU7	1	0	1	1	0	1	1	0	1
SMA4	0	0	0	0	0	0	1	0	0
SMA5	1	0	1	0	0	1	1	0	1
SMAD1	1	0	1	1	0	1	1	0	1
SMAD2	0	0	1	1	0	1	1	0	1
SMAD3	0	1	0	0	1	0	1	0	1
SMAD4	1	0	1	1	0	1	1	0	1
SMAD6	0	1	0	0	0	1	0	1	1
SMAD7	1	0	1	1	0	1	0	0	1
SMAP1	1	0	0	0	0	1	1	0	1

SMARCA2	1	0	1	1	0	1	1	0	1
SMARCA4	1	0	0	1	0	0	0	0	0
SMARCA5	1	0	1	1	0	1	1	0	1
SMARCAD1	1	0	1	1	0	1	1	0	1
SMARCAL1	0	0	0	1	0	1	0	0	1
SMARCC1	1	0	1	1	0	1	1	0	1
SMARCC2	0	0	0	1	0	0	0	0	0
SMARCD1	1	0	1	1	0	1	1	0	1
SMARCD2	0	0	0	1	0	0	0	0	0
SMARCE1	1	0	1	1	0	1	1	0	1
SMCP	0	1	0	0	0	0	0	0	0
SMEK2	1	0	1	1	0	1	1	0	1
SMG5	1	0	1	1	0	1	1	0	1
SMNDC1	1	0	1	1	0	1	1	0	1
SMOX	0	1	0	0	1	1	0	1	1
SMPD1	0	0	0	0	0	0	1	0	1
SMPD2	0	0	0	1	0	0	0	0	1
SMPDL3A	1	1	1	1	1	1	1	1	1
SMS	0	1	1	0	1	0	0	1	0
SMTN	1	1	1	0	0	1	0	0	1
SMU1	1	0	1	1	0	1	1	0	1
SMUG1	1	1	1	1	1	1	0	1	1
SMURF2	1	0	1	1	0	1	1	0	1
SMYD2	1	0	1	1	0	1	1	0	1
SNAG1	1	0	0	0	0	0	0	1	0
SNAI1	0	0	0	0	0	0	0	1	0
SNAP23	1	0	1	1	0	1	1	0	1
SNAP29	1	0	1	1	0	1	1	0	1
SNAPC1	1	0	1	1	0	1	1	0	1
SNAPC2	0	1	0	0	1	1	0	1	0
SNAPC3	1	0	1	1	0	1	1	0	1
SNAPC4	0	0	1	0	0	0	0	0	0
SNAPC5	1	0	1	1	0	1	1	0	1
SNCA	0	0	1	0	0	1	1	0	1
SNCB	0	1	1	0	1	0	0	1	0
SND1	1	0	1	1	0	1	1	0	1
SNF1LK	0	1	0	0	0	0	1	1	0
SNF1LK2	0	0	0	0	0	0	1	0	0
SNF8	1	0	1	1	0	1	1	0	1
SNFT	0	1	0	0	0	0	0	1	0
SNIP	0	1	0	0	1	0	0	1	0
SNIP1	0	0	0	1	0	0	1	0	1
SNPH	0	1	1	0	1	0	1	1	1
SNRK	0	0	1	0	0	1	0	0	0
SNRP70	0	0	0	1	0	0	0	0	0
SNRPA	1	0	1	1	0	1	1	0	1
SNRPA1	1	0	1	1	0	1	1	0	1
SNRPB	0	0	0	0	0	1	0	0	0
SNRPB2	1	1	1	1	1	1	1	1	1
SNRPC	1	0	1	1	0	1	1	0	1
SNRPD2	1	0	1	1	0	1	1	0	1
SNRPD3	1	0	1	1	0	1	0	0	1
SNRPF	1	1	1	0	1	1	1	1	1
SNRPG	1	0	0	1	0	0	0	0	0
SNRPN	0	1	0	0	1	1	0	1	0
SNTA1	0	1	1	0	0	1	0	0	0
SNTB1	0	1	0	0	1	1	0	1	1
SNTB2	1	0	1	1	0	1	1	0	1
SNURF	0	1	0	0	1	1	0	1	0
SNW1	1	0	1	1	0	1	1	0	1
SNX1	0	0	1	1	0	1	1	0	1
SNX11	1	0	1	1	0	1	1	0	1

SNX12	0	1	0	0	1	1	0	0	1
SNX13	1	0	1	1	0	1	1	0	1
SNX14	1	0	1	1	0	1	1	0	1
SNX15	0	0	0	1	0	1	0	0	0
SNX16	1	0	1	1	0	1	0	0	1
SNX17	1	0	1	1	0	1	1	0	1
SNX19	0	1	0	1	1	1	0	1	1
SNX24	1	0	1	1	0	1	1	0	1
SNX25	0	1	1	0	1	1	0	1	1
SNX27	1	0	1	1	0	0	1	0	1
SNX3	1	0	0	1	0	1	1	0	1
SNX5	1	0	1	1	0	1	1	0	1
SNX6	0	0	0	0	0	0	0	0	1
SOAT1	1	0	0	1	0	0	0	0	1
SOCS1	1	0	0	1	0	1	1	0	1
SOCS2	0	1	1	1	0	1	0	1	0
SOCS4	1	0	1	1	0	1	1	0	1
SOCS7	0	0	0	0	0	0	0	0	1
SOD1	0	0	0	1	0	1	0	0	1
SOD2	1	0	1	1	0	1	1	0	1
SOD3	0	0	1	0	0	1	0	0	1
SOLH	1	0	1	1	0	1	1	0	1
SON	1	0	1	1	0	1	1	0	1
SORBS1	0	1	0	0	0	1	0	0	1
SORCS1	0	1	0	0	1	0	0	1	0
SORCS2	0	1	0	0	1	0	0	1	0
SORL1	0	0	1	0	0	0	1	0	1
SORT1	0	0	0	0	0	1	0	0	1
SOS1	1	0	1	1	0	1	1	0	1
SOS2	1	0	1	0	0	1	1	0	1
SOST	0	1	0	0	0	0	0	1	0
SOX11	0	1	1	0	1	0	0	1	0
SOX13	0	1	0	0	1	0	0	1	0
SOX18	0	0	0	0	0	0	0	1	0
SOX2	0	1	1	0	1	1	0	1	1
SOX30	0	1	0	0	1	0	0	0	1
SOX4	1	0	1	1	0	1	1	0	1
SOX5	0	1	1	0	1	1	0	1	1
SOX8	0	1	0	0	0	0	0	1	0
SOX9	0	1	1	0	1	0	0	1	0
SP1	1	0	1	1	0	1	0	0	1
SP100	1	0	1	1	0	1	1	0	1
SP110	1	0	1	0	0	1	1	0	1
SP140	0	0	0	0	0	1	1	0	1
SP3	1	0	1	1	0	1	1	0	1
SP4	1	0	0	0	0	0	0	0	1
SPA17	1	0	1	1	0	1	1	0	1
SPAG16	1	0	1	1	0	1	1	0	1
SPAG5	0	0	0	1	0	1	1	0	1
SPAG7	1	0	1	1	0	1	0	0	1
SPAG9	1	0	0	1	0	1	1	0	1
SPARC	0	0	0	1	0	1	1	0	1
SPAST	1	0	1	1	0	1	1	0	1
SPATA1	1	0	1	1	0	1	1	0	1
SPATA18	0	1	1	0	1	1	0	1	1
SPATA2	0	0	1	0	0	1	0	0	1
SPATA20	0	0	0	0	0	0	0	1	0
SPATA3	0	1	0	0	1	0	0	0	0
SPATA5	1	0	1	1	0	1	1	0	1
SPATA5L1	1	0	1	1	0	1	1	0	1
SPATA6	1	0	1	1	0	1	1	0	1
SPATS2	1	0	0	0	0	0	1	0	1

SPCS1	1	0	1	1	0	1	1	0	1
SPCS2	1	0	1	1	0	1	1	0	1
SPCS3	1	0	1	1	0	1	1	0	1
SPEN	0	0	0	0	0	1	0	0	1
SPG20	1	0	1	1	0	1	1	0	1
SPG21	1	0	1	1	0	1	1	0	1
SPG3A	0	1	0	0	1	0	0	1	1
SPG7	1	0	1	1	0	1	1	0	1
SPHK2	1	0	1	1	0	1	1	0	1
SPI1	1	0	0	0	0	0	0	0	0
SPIN1	1	0	0	0	0	1	1	0	1
SPIN3	0	1	1	0	0	0	0	1	1
SPINT1	0	0	0	0	0	0	0	1	0
SPINT2	1	0	0	0	0	1	0	0	1
SPIRE2	0	0	0	0	0	0	0	1	0
SPN	1	0	1	1	0	1	1	0	1
SPO11	0	1	0	0	1	0	0	1	0
SPOCK2	0	1	0	0	1	0	0	1	1
SPON2	0	1	0	0	1	0	0	1	0
SPP1	1	1	1	0	0	1	1	1	0
SPPL2A	1	0	0	1	0	1	1	0	1
SPPL2B	0	0	0	0	0	1	0	0	1
SPR	0	1	1	0	1	1	0	1	1
SPRED1	1	0	1	1	0	1	1	1	1
SPRR1A	0	1	0	0	1	0	0	0	0
SPRY2	1	0	1	1	0	1	1	1	1
SPRY3	1	0	1	1	0	1	1	1	1
SPRYD3	0	0	0	0	0	1	0	0	0
SPRYD4	1	0	1	1	0	1	0	0	1
SPSB1	0	0	0	0	0	0	0	1	0
SPSB3	1	0	1	1	0	1	1	0	1
SPTAN1	1	0	1	1	0	1	1	0	1
SPTB	0	1	0	0	0	0	0	0	0
SPTY2D1	1	1	1	1	0	1	1	0	1
SQLE	1	0	1	1	0	1	0	0	1
SQRDL	0	0	0	0	0	1	1	0	1
SQSTM1	1	0	1	1	0	1	0	0	1
SRBD1	1	0	1	1	0	1	1	0	1
SRC	0	0	0	1	0	0	0	1	1
SRD5A1	1	0	1	1	0	1	0	0	1
SRD5A2L	1	0	1	1	0	1	1	0	1
SREBF2	0	0	0	0	0	0	0	0	1
SRF	1	0	0	0	0	1	0	0	1
SRFBP1	1	0	1	1	0	1	1	0	1
SRGAP1	1	0	1	1	0	1	0	1	1
SRI	1	0	1	1	0	1	1	0	1
SRM	1	0	1	1	0	1	1	0	1
SRP19	1	0	1	1	0	1	1	0	1
SRP54	1	0	1	1	0	1	1	0	1
SRP68	0	1	1	0	0	0	0	1	0
SRP72	1	0	1	1	0	1	1	0	1
SRP9	1	0	1	1	0	1	1	0	1
SRPK2	1	0	1	1	0	1	1	0	1
SRPR	1	0	1	1	0	1	1	0	1
SRPRB	0	0	1	0	0	1	0	0	1
SRPX	0	1	1	0	1	1	0	1	1
SRR	1	0	1	1	0	1	1	0	1
SRRM1	1	0	0	1	0	0	0	0	1
SRXN1	1	0	0	0	0	0	0	0	1
SRRP35	0	1	0	0	0	0	0	1	0
SS18	1	0	1	1	0	1	1	0	1
SS18L1	1	0	1	1	0	1	1	0	1

SS18L2	1	0	1	1	0	1	1	0	1
SSB	0	0	0	1	0	1	1	0	1
SSBP1	1	0	1	1	0	1	1	0	1
SSBP2	1	0	1	1	0	0	0	0	1
SSBP3	1	0	1	1	0	1	1	0	1
SSBP4	0	0	0	0	0	1	0	0	1
SSFA2	1	0	1	1	0	1	1	0	1
SSH1	0	0	0	1	0	1	1	0	1
SSH2	1	0	1	1	0	1	1	0	1
SSNA1	1	0	1	1	0	1	1	0	1
SSPN	0	1	0	0	1	0	0	1	0
SSR1	1	0	0	0	0	0	0	0	0
SSR2	0	0	0	0	0	1	0	0	1
SSR3	0	0	0	0	0	1	1	0	1
SSR4	1	1	1	1	1	1	0	1	1
SSRP1	1	0	1	1	0	1	1	0	1
SSSCA1	1	0	1	1	0	1	0	0	1
SST	0	1	0	0	1	0	0	1	0
SSTR2	0	1	0	0	1	1	0	1	0
SSU72	1	0	1	1	0	1	1	0	1
ST13	1	0	1	1	0	1	1	0	1
ST14	0	0	0	0	0	0	0	1	0
ST3GAL1	1	0	1	1	0	1	1	0	1
ST3GAL2	0	0	0	0	0	1	0	0	1
ST3GAL3	1	0	0	0	0	1	0	0	1
ST3GAL4	0	0	0	0	0	0	0	0	1
ST3GAL5	1	0	1	1	0	1	1	0	1
ST3GAL6	1	0	1	1	0	1	1	0	1
ST5	1	0	1	1	0	1	1	1	1
ST6GAL1	0	0	0	0	0	1	0	0	1
ST6GALNAC2	0	1	0	0	1	0	0	1	0
ST6GALNAC4	1	0	1	0	0	1	0	0	0
ST6GALNAC6	0	0	0	0	0	0	0	0	1
ST7	1	0	1	1	0	1	1	0	1
ST7L	1	0	1	1	0	1	1	0	1
ST8SIA4	1	0	1	1	0	1	1	0	1
ST8SIA5	0	1	1	0	1	0	0	1	1
STAB2	0	1	0	0	0	0	0	0	0
STAC	0	0	1	1	0	1	1	1	1
STAC2	0	1	1	0	1	1	0	1	1
STAG1	1	0	1	1	0	1	1	0	1
STAG2	1	1	1	1	0	1	1	0	1
STAG3	0	1	0	0	0	0	0	0	0
STAM	1	0	1	1	0	1	1	0	1
STAM2	1	0	1	1	0	1	1	0	1
STAMBP	1	0	1	0	0	1	1	0	1
STAMBPL1	0	0	0	1	0	1	0	0	1
STAP2	1	0	1	1	1	1	1	0	1
STARD10	0	0	1	1	0	0	1	0	1
STARD13	1	0	1	1	0	1	1	0	1
STARD3	1	0	0	0	0	0	0	0	1
STARD3NL	1	0	1	1	0	1	1	0	1
STARD4	1	0	1	1	0	1	1	0	1
STARD5	0	0	0	0	0	1	0	0	1
STARD8	0	1	0	0	1	0	0	1	0
STAT1	0	0	1	0	0	1	0	0	1
STAT2	1	0	1	1	0	1	0	0	1
STAT4	0	0	1	0	0	1	1	0	1
STAT5A	1	0	1	1	0	1	1	0	1
STAT5B	0	0	0	0	0	0	0	0	1
STAT6	1	0	0	0	0	0	0	0	1
STAU2	1	1	1	1	0	1	1	0	1

STCH	1	0	0	1	0	1	1	0	1
STEAP3	0	1	0	0	1	0	0	0	0
STEAP4	0	0	0	0	0	0	0	0	1
STIL	1	0	1	1	0	1	1	0	1
STIM1	0	0	0	1	0	0	1	0	1
STIM2	1	0	1	1	0	1	1	0	1
STIP1	1	0	1	1	0	1	1	0	1
STK11IP	1	0	1	1	0	1	1	0	1
STK16	1	0	1	1	0	1	0	0	1
STK17B	0	1	0	0	0	0	1	1	1
STK19	1	0	1	1	0	1	0	0	1
STK24	0	0	0	1	0	1	1	0	1
STK25	0	0	0	0	0	0	0	1	0
STK32B	1	1	1	1	0	1	1	0	1
STK32C	0	1	0	0	0	0	0	1	0
STK35	1	0	1	1	0	1	1	0	1
STK36	1	0	1	1	0	1	1	0	1
STK38	1	0	1	1	0	1	1	0	1
STK38L	1	0	0	0	0	1	1	0	1
STK39	0	1	0	1	1	0	1	1	1
STK4	1	0	1	1	0	1	1	0	1
STK40	1	0	0	1	0	1	1	0	1
STMN1	0	0	0	0	0	0	0	1	0
STMN2	0	1	0	0	1	0	0	1	0
STMN3	0	1	0	1	1	1	0	1	1
STOM	1	0	1	1	0	1	0	0	1
STOML1	1	0	1	0	0	1	0	0	1
STOML2	1	0	1	1	0	1	1	0	1
STOX2	0	1	1	0	1	1	0	1	1
STRAP	0	0	0	1	0	0	1	0	1
STRBP	0	1	0	0	1	0	0	1	0
STRN	1	0	1	1	0	1	1	0	1
STRN3	1	0	1	1	0	1	1	0	1
STRN4	0	0	0	1	0	0	0	0	1
STS-1	1	0	1	0	0	1	1	0	1
STT3A	1	0	1	1	0	1	1	0	1
STT3B	1	0	1	1	0	1	1	0	1
STUB1	0	1	0	0	0	0	0	1	0
STX10	1	0	1	1	0	1	1	0	1
STX11	0	0	0	1	0	0	0	0	0
STX16	1	1	1	1	1	1	1	1	1
STX17	0	0	0	0	0	1	1	1	1
STX18	1	0	1	1	0	1	1	0	1
STX1A	0	1	0	0	1	0	0	1	0
STX7	1	0	1	0	0	1	1	0	1
STX8	1	0	1	1	0	1	1	0	1
STXBP5	1	0	1	1	0	1	1	0	1
STYX	0	0	0	0	0	0	0	0	1
STYXL1	1	0	1	1	0	1	1	0	1
SUCLG1	1	0	1	1	0	1	1	0	1
SUCLG2	1	1	1	0	1	1	1	1	1
SUCNR1	1	0	1	1	0	1	1	0	1
SUDS3	1	0	1	1	0	1	0	0	1
SUFU	1	0	1	1	0	1	1	0	1
SUGT1	1	0	1	1	0	1	1	0	1
SULF2	1	0	1	1	1	1	1	1	1
SULT1A3	1	0	1	1	0	1	1	0	1
SULT1A4	1	0	1	1	0	1	1	0	1
SULT1B1	1	0	0	0	0	0	1	0	1
SULT1C2	0	0	0	0	0	0	1	0	0
SULT6B1	0	0	0	0	0	0	1	0	0
SUMF1	1	0	1	0	0	0	1	0	1

SUMF2	1	0	0	1	0	1	1	0	1
SUMO2	1	0	1	0	0	1	0	0	1
SUOX	1	0	1	1	0	1	1	0	1
SUPT16H	1	0	1	1	0	1	1	0	1
SUPT3H	1	0	1	1	0	1	1	0	1
SUPT4H1	1	0	1	1	0	1	1	0	1
SUPT5H	1	0	1	1	0	1	1	0	1
SUPT6H	1	0	1	1	0	1	1	0	1
SUPT7L	1	0	1	1	0	1	1	0	1
SUPV3L1	1	0	1	1	0	1	1	0	1
SURF1	1	0	1	1	0	1	1	0	1
SURF2	1	0	1	1	0	1	1	0	1
SURF4	1	0	1	1	0	1	1	0	1
SURF5	1	0	1	1	0	1	1	0	1
SUSD4	0	1	1	0	1	1	0	1	1
SUV39H1	0	1	0	1	1	1	1	0	1
SUV39H2	1	0	1	1	0	1	1	0	1
SUV420H1	0	0	0	0	0	0	0	1	1
SV2C	0	1	1	0	1	1	0	1	1
SVIL	0	1	0	0	0	0	1	1	1
SWAP70	1	0	1	1	0	1	1	0	1
SYAP1	0	1	1	0	1	1	0	1	1
SYBL1	0	0	1	1	0	1	0	0	1
SYF2	1	0	1	1	0	1	1	0	1
SYK	1	0	1	1	0	1	0	0	1
SYMPK	1	1	1	1	1	1	1	1	1
SYN1	0	1	0	0	1	0	0	0	0
SYN2	0	1	0	0	1	0	0	1	0
SYNCRIP	1	0	0	1	0	1	0	0	1
SYNGR1	0	0	0	0	1	0	1	0	0
SYNJ1	1	0	1	1	0	1	1	0	1
SYNJ2	0	0	0	0	1	0	0	1	1
SYNJ2BP	1	0	1	1	0	1	1	0	1
SYNPO2	0	1	0	0	1	0	0	1	0
SYP	0	1	0	0	0	0	0	1	0
SYPL1	0	0	0	0	0	0	0	0	1
SYT11	1	0	0	0	0	0	0	0	0
SYT15	0	1	0	0	1	0	0	1	0
SYT17	0	0	0	0	0	0	0	1	0
SYT3	0	1	1	0	1	1	0	1	0
SYT6	0	1	0	0	1	0	0	1	1
SYT8	0	0	0	0	0	0	0	1	0
SYTL3	0	0	1	1	0	1	0	0	1
SYVN1	1	0	1	1	0	1	1	0	1
T	0	1	1	0	1	1	0	1	1
TA-NFKBH	0	0	1	1	0	1	0	0	1
TAAR6	0	0	0	0	1	0	0	1	0
TACC1	1	0	1	1	0	1	0	0	0
TACC3	0	0	0	0	0	0	0	0	1
TADA1L	0	0	0	1	0	1	0	0	1
TADA2L	1	0	1	1	0	1	1	0	1
TADA3L	1	0	1	1	0	1	1	0	1
TAF1	0	1	1	1	0	1	0	0	0
TAF10	1	0	1	1	0	1	1	0	1
TAF12	1	0	1	1	0	1	1	0	1
TAF13	1	0	1	1	0	1	1	0	1
TAF15	1	0	1	1	0	1	1	0	1
TAF2	1	0	1	1	0	1	0	0	1
TAF4	1	0	1	1	0	1	1	0	1
TAF5L	1	0	1	1	0	1	1	0	1
TAF6	1	0	1	1	0	1	1	0	1
TAF6L	1	0	1	1	0	1	1	0	1

TAF7	0	0	1	0	0	1	0	0	1
TAF9	1	0	1	1	0	1	1	0	1
TAGAP	1	0	1	1	0	1	1	0	1
TAGLN2	0	0	0	0	0	0	0	0	1
TAGLN3	0	1	1	0	1	1	0	1	0
TAL1	0	1	1	0	1	1	0	1	0
TANC1	0	1	1	0	1	0	0	1	1
TANK	1	0	1	1	0	1	1	0	1
TAOK2	1	0	1	1	0	1	1	0	1
TAP1	1	0	1	1	0	1	1	0	1
TAP2	1	0	1	1	0	1	1	0	1
TAPBP	1	0	1	1	0	1	0	0	1
TAPBPL	0	0	1	1	0	1	0	0	1
TARBP2	1	0	1	1	0	1	1	0	1
TARDBP	1	0	1	1	0	1	1	0	0
TARP	0	0	0	0	0	0	1	0	0
TARS	1	0	1	1	0	1	1	0	1
TASP1	0	0	0	0	0	1	0	0	1
TATDN1	1	0	1	1	0	1	1	0	1
TATDN2	1	0	0	0	0	1	1	0	1
TATDN3	1	0	1	1	0	1	1	0	1
TAX1BP1	1	0	1	1	0	1	1	0	1
TAX1BP3	1	0	1	1	0	1	0	0	0
TAZ	1	1	1	1	1	1	0	0	1
TBC1D10B	1	0	1	0	0	1	1	0	1
TBC1D13	0	0	0	0	0	0	0	0	1
TBC1D14	1	0	1	1	0	1	1	0	1
TBC1D15	1	0	1	1	0	1	1	0	1
TBC1D16	0	1	0	0	1	0	0	1	1
TBC1D17	1	0	1	1	0	1	0	0	1
TBC1D19	1	0	1	1	0	1	1	0	1
TBC1D2	1	0	1	1	0	1	1	0	1
TBC1D20	0	0	0	0	0	1	1	0	1
TBC1D22A	1	0	1	1	0	1	1	0	1
TBC1D22B	1	0	1	1	0	1	1	0	1
TBC1D23	0	0	0	1	0	1	0	0	0
TBC1D2B	0	0	0	0	0	1	0	0	0
TBC1D4	0	0	1	0	0	0	0	0	1
TBC1D5	1	0	1	1	0	1	1	0	1
TBC1D7	1	0	1	1	0	1	1	0	1
TBC1D9	0	0	1	0	0	0	1	0	1
TBCA	1	0	1	1	0	1	1	0	1
TBCC	1	0	1	1	0	1	1	0	1
TBCCD1	1	0	1	1	0	1	1	0	1
TBCE	1	0	1	0	0	1	1	0	1
TBK1	1	0	1	1	0	1	1	0	1
TBKBP1	1	0	1	0	0	1	1	1	1
TBL1X	0	0	0	0	0	0	0	1	1
TBL1XR1	0	0	0	0	0	1	0	0	0
TBL2	0	0	0	0	0	1	0	0	1
TBL3	1	0	1	1	0	1	1	0	1
TBN	1	0	0	1	0	1	1	0	1
TBP	1	0	1	1	0	1	1	0	1
TBPL1	1	0	1	0	0	1	0	1	1
TBRG1	1	0	1	0	0	1	1	0	1
TBRG4	1	0	0	0	0	1	0	0	1
TBX22	0	1	0	0	1	0	0	1	0
TBX3	0	1	1	0	1	1	0	1	1
TBX4	0	1	0	0	1	0	0	1	0
TBX5	0	1	1	0	1	0	0	1	1
TCEA1	1	0	1	1	0	1	1	0	1
TCEA2	1	0	0	0	1	0	0	1	0

TCEA3	0	1	0	0	1	0	0	1	0
TCEAL1	1	1	1	1	1	1	0	1	1
TCEAL3	0	1	1	0	1	0	0	1	0
TCEAL4	0	1	0	0	0	0	0	0	0
TCEAL8	1	1	1	1	1	1	1	1	1
TCEB1	1	0	1	1	0	1	1	0	1
TCEB2	1	0	1	0	0	1	1	0	1
TCERG1	1	0	0	1	0	1	1	0	1
TCF12	1	0	1	1	0	1	1	0	1
TCF19	1	0	1	1	0	1	0	0	1
TCF3	0	0	0	0	0	1	0	1	0
TCF4	1	0	1	1	0	1	1	0	1
TCF7	0	1	1	0	1	1	1	1	1
TCF7L2	0	0	0	1	0	0	0	1	1
TCHP	1	0	1	1	0	1	0	0	1
TCIRG1	1	0	0	0	0	0	0	0	1
TCP1	1	0	1	1	0	1	1	0	1
TCP11L2	0	0	0	1	0	1	1	0	1
TCTA	1	0	1	1	0	1	1	0	1
TCTEX1D1	0	0	0	0	0	1	1	0	1
TDG	1	0	1	1	0	1	1	0	1
TDP1	1	0	1	1	0	1	1	0	1
TDRD7	1	0	0	1	0	0	1	0	0
TDRKH	0	0	0	0	0	1	0	0	1
TEAD2	0	1	0	0	1	1	0	1	0
TEAD3	0	1	0	0	1	0	0	1	0
TEF	0	0	1	0	0	1	1	0	1
TEGT	1	0	0	1	0	1	1	0	1
TEP1	1	0	0	1	0	0	1	0	1
TERF1	1	1	1	1	0	1	0	0	1
TERF2IP	1	0	1	1	0	1	1	0	1
TES	0	0	0	0	0	0	0	0	1
TESC	0	0	0	0	1	0	0	0	0
TESK1	1	0	1	0	0	1	1	0	1
TESK2	1	0	1	1	0	1	1	0	1
TETRAN	1	0	1	1	0	1	0	0	1
TEX10	1	0	1	0	0	0	1	0	1
TEX2	1	0	1	1	0	1	1	0	1
TEX261	0	0	1	1	0	1	1	0	1
TEX264	0	0	0	0	0	1	0	0	0
TF	0	1	0	0	1	0	0	1	0
TFAM	1	0	1	1	0	1	1	0	1
TFB2M	1	0	1	1	0	1	1	0	1
TFCP2	1	0	1	1	0	1	1	0	1
TFCP2L1	0	1	0	0	1	0	0	1	0
TFEC	1	0	1	1	0	1	1	0	1
TFG	0	1	1	0	0	1	0	0	0
TFIP11	1	0	1	1	0	1	1	0	1
TFPI	1	0	0	1	0	0	1	0	0
TFPT	1	0	1	1	0	1	1	0	1
TFRC	1	0	1	1	0	1	1	0	1
TGDS	1	0	1	1	0	1	1	0	1
TGFA	1	0	0	1	0	1	0	1	1
TGFBR1	1	0	1	0	0	1	1	0	1
TGFBR2	1	0	1	1	0	1	1	0	1
TGFBR3	0	1	1	0	1	1	1	1	1
TGFBRAP1	1	0	1	1	0	1	1	0	1
TGIF2	1	0	0	0	0	0	1	1	1
TGM2	0	0	1	0	0	1	0	0	0
TGM6	0	1	0	0	1	0	0	1	0
TGOLN2	1	0	1	0	0	0	0	0	1
THADA	1	0	1	1	0	1	1	0	1

THAP1	0	0	0	0	0	1	0	0	1
THAP10	0	1	0	1	0	1	1	1	1
THAP11	0	0	0	1	0	0	0	0	1
THAP5	1	0	1	1	0	1	1	0	1
THAP6	1	0	1	1	0	1	1	0	1
THAP7	0	0	1	1	0	1	0	0	1
THAP8	1	0	1	1	0	1	1	0	1
THBD	0	0	0	1	0	1	1	0	1
THBS1	1	1	1	1	0	1	0	1	1
THBS3	1	0	1	1	0	1	0	0	1
THEM2	1	0	1	1	0	1	1	0	1
THEM4	1	0	1	1	0	1	1	0	1
THEX1	0	0	0	1	0	1	0	0	1
THNSL1	1	0	1	1	0	1	1	0	1
THOC1	1	0	0	0	0	1	1	0	1
THOC4	1	0	1	1	0	1	1	0	1
THOC5	1	0	1	1	0	1	1	0	1
THOC6	0	0	1	0	0	1	0	0	1
THOC7	1	0	1	1	0	1	1	0	1
THRA	0	1	1	0	1	1	0	1	1
THRAP3	1	0	1	1	0	1	1	0	1
THRAP4	1	0	1	1	0	1	0	0	1
THRB	0	1	0	0	1	0	0	1	0
THUMPD2	0	0	0	0	0	1	1	0	1
THYN1	1	0	1	1	0	1	1	0	1
TIA1	1	0	1	1	0	1	1	0	1
TIAL1	0	0	0	1	0	0	0	0	1
TIAM1	0	1	1	0	1	1	0	1	1
TICAM2	1	0	1	1	0	1	1	0	1
TIGD1	1	0	1	1	0	1	1	0	1
TIGD2	1	0	1	1	0	1	1	0	1
TIGD3	1	0	1	0	0	1	1	0	1
TIGD4	1	0	1	1	0	1	1	0	1
TIGD5	1	0	1	1	0	1	1	0	1
TIGD6	1	0	1	1	0	1	1	0	1
TIGD7	0	0	1	0	0	1	0	0	1
TIMELESS	1	0	1	1	0	1	1	0	1
TIMM10	1	0	1	1	0	1	1	0	1
TIMM17A	0	0	0	0	0	0	0	0	1
TIMM17B	1	0	1	1	0	1	0	0	1
TIMM22	1	0	0	0	0	0	0	0	1
TIMM23	1	0	1	1	0	1	1	0	1
TIMM44	1	0	1	1	0	1	1	0	1
TIMM8A	1	1	1	0	1	1	0	0	1
TIMM8B	1	0	1	1	0	1	1	0	1
TIMM9	1	0	1	1	0	1	1	0	1
TIMP1	0	1	0	0	1	0	0	0	0
TIMP3	0	1	0	0	1	0	0	1	0
TIMP4	0	1	0	0	0	0	0	1	0
TINF2	1	0	0	0	0	0	0	0	1
TINP1	1	0	1	1	0	1	1	0	1
TIPARP	0	0	0	0	0	0	0	0	1
TIPRL	0	0	0	1	0	1	1	0	1
TIRAP	0	0	0	1	0	1	1	0	1
TJAP1	1	0	0	0	0	0	0	0	1
TJP1	0	1	1	0	1	0	0	1	0
TJP2	1	0	1	0	0	1	0	0	1
TK1	0	0	0	0	0	1	0	0	1
TK2	1	0	1	1	0	1	1	0	1
TKT	0	0	0	0	0	0	0	0	0
TLCD1	0	0	0	1	0	1	0	0	1
TLE1	1	1	1	1	0	1	1	1	1

TLE2	0	1	0	0	1	0	0	1	0
TLE3	1	0	1	1	0	1	1	0	1
TLE4	0	0	0	0	0	0	1	0	1
TLE6	0	0	1	0	0	1	0	1	0
TLK1	1	0	1	1	0	1	1	0	1
TLK2	1	0	1	1	0	1	1	0	1
TLN1	1	0	1	1	0	1	1	0	1
TLOC1	1	0	0	0	0	0	1	0	1
TLR1	1	0	1	1	0	1	1	0	1
TLR2	0	0	0	1	0	1	1	0	1
TLR3	1	0	1	1	0	1	1	0	1
TLR4	0	0	0	1	0	0	1	0	1
TLR5	0	0	0	0	0	1	0	0	1
TLR7	0	1	1	0	1	1	1	1	1
TLR8	0	1	1	0	1	1	0	1	1
TM2D1	1	0	1	1	0	1	1	0	1
TM2D2	1	0	1	1	0	1	1	0	1
TM2D3	0	0	0	0	0	0	0	0	1
TM4SF1	0	0	0	0	0	0	1	0	0
TM4SF19	1	0	1	1	0	1	0	0	0
TM6SF1	1	0	1	0	0	1	0	1	1
TM7SF3	1	0	1	1	0	1	1	0	1
TM7SF4	1	0	1	1	0	1	0	0	0
TM9SF1	1	0	1	1	0	1	1	0	1
TM9SF2	1	0	1	1	0	1	0	0	1
TM9SF3	1	0	1	1	0	1	1	0	1
TM9SF4	1	0	0	0	0	0	0	0	0
TMBIM4	1	0	0	0	0	1	0	0	0
TMC6	0	0	0	0	0	0	0	0	1
TMC8	0	0	0	0	0	0	0	0	1
TMCC2	0	1	1	1	1	1	0	1	1
TMCC3	0	1	0	0	0	0	0	1	0
TMCO1	1	0	1	1	0	1	1	0	1
TMCO3	1	0	1	1	0	1	1	0	1
TMCO4	1	0	0	1	0	1	1	0	1
TMED1	1	0	0	0	0	1	0	0	0
TMED10	1	0	1	1	0	1	1	0	1
TMED3	0	1	0	0	0	0	0	1	0
TMED4	1	0	1	1	0	1	1	0	1
TMED5	1	0	1	1	0	1	1	0	1
TMED7	1	0	1	1	0	1	1	0	1
TMED8	1	0	1	0	0	1	1	0	1
TMED9	0	1	0	0	0	0	0	0	0
TMEFF1	0	1	0	0	1	0	0	1	1
TMEFF2	1	1	1	0	1	1	1	1	1
TMEM1	1	0	0	0	0	0	0	0	0
TMEM101	0	0	0	1	0	1	0	0	0
TMEM104	1	0	1	1	0	1	1	0	1
TMEM106B	1	1	1	1	0	1	1	1	1
TMEM106C	0	1	1	1	0	1	1	1	1
TMEM107	1	0	1	1	0	1	0	0	1
TMEM109	1	0	1	1	0	1	1	0	1
TMEM11	1	0	1	1	0	1	1	0	1
TMEM111	1	0	1	1	0	1	1	0	1
TMEM116	1	0	1	1	0	1	1	0	1
TMEM117	0	0	0	0	1	0	0	1	0
TMEM118	1	1	1	1	0	1	0	0	1
TMEM123	1	0	1	0	0	1	1	0	1
TMEM126A	1	0	1	1	0	1	1	0	1
TMEM126B	1	0	1	1	0	1	1	0	1
TMEM127	1	0	1	1	0	1	1	0	1
TMEM128	1	0	1	1	0	1	1	0	1

TMEM129	1	0	0	0	0	0	0	0	1
TMEM135	1	0	1	1	0	1	1	0	1
TMEM138	1	0	1	1	0	1	1	0	1
TMEM140	1	0	1	1	0	1	1	0	1
TMEM141	0	0	0	1	0	0	0	0	0
TMEM142A	0	0	0	1	0	0	0	0	0
TMEM143	0	0	0	0	0	1	0	0	0
TMEM144	1	0	0	1	0	1	1	0	1
TMEM145	0	1	0	0	1	0	0	1	0
TMEM147	1	0	1	0	0	1	0	0	1
TMEM149	1	0	1	1	0	1	0	0	1
TMEM14A	0	1	0	0	1	0	0	1	0
TMEM14B	1	0	1	1	0	1	1	0	1
TMEM14C	1	0	1	1	0	1	1	0	1
TMEM16B	0	1	0	0	1	0	0	1	0
TMEM16F	1	0	1	1	0	1	1	0	1
TMEM16H	1	0	1	1	0	1	1	0	1
TMEM16K	1	0	1	0	0	1	1	0	1
TMEM18	1	0	1	1	0	1	1	0	1
TMEM19	1	0	0	0	0	0	0	0	0
TMEM2	1	1	0	0	1	1	0	1	1
TMEM20	0	0	1	0	0	0	1	0	1
TMEM24	0	0	1	1	0	1	1	0	1
TMEM26	0	1	1	0	1	0	0	1	0
TMEM28	0	1	0	0	1	0	0	1	0
TMEM29	1	1	1	1	1	1	0	1	1
TMEM30A	1	0	1	1	0	1	1	0	1
TMEM33	0	0	1	1	0	1	0	0	1
TMEM34	0	0	1	0	0	1	0	0	1
TMEM37	0	0	0	0	0	0	0	1	0
TMEM38A	1	0	0	1	0	1	0	0	1
TMEM38B	1	1	1	1	0	1	1	1	1
TMEM39A	1	0	1	1	0	1	1	0	1
TMEM4	1	0	1	1	0	1	1	0	1
TMEM41A	0	0	0	0	0	1	0	0	0
TMEM41B	1	0	1	1	0	1	1	0	1
TMEM42	0	0	1	0	0	0	1	0	1
TMEM43	1	0	1	1	0	1	1	0	1
TMEM44	1	0	1	1	0	1	0	0	1
TMEM45A	1	1	1	1	1	1	1	1	1
TMEM45B	0	0	0	0	1	0	0	1	0
TMEM48	0	0	0	0	0	1	0	0	1
TMEM49	1	0	1	1	0	1	1	0	1
TMEM5	0	0	1	0	0	0	0	0	0
TMEM50A	1	0	1	1	0	1	1	0	1
TMEM50B	0	0	0	0	0	1	0	0	0
TMEM51	1	0	1	1	0	1	0	1	1
TMEM53	1	0	1	1	0	1	0	0	1
TMEM54	0	1	1	0	1	1	0	1	0
TMEM55A	1	0	1	0	0	0	1	0	1
TMEM55B	0	0	0	0	0	0	0	0	1
TMEM57	0	0	1	0	0	1	1	0	1
TMEM58	0	1	1	0	1	0	0	1	1
TMEM59	1	0	1	1	0	1	1	0	1
TMEM60	1	0	1	1	0	1	1	0	1
TMEM61	0	1	0	0	1	0	0	1	0
TMEM62	1	0	1	1	0	1	1	0	1
TMEM63B	1	0	1	1	0	1	1	0	1
TMEM63C	0	1	0	0	1	0	0	1	0
TMEM65	0	1	1	0	1	0	0	1	1
TMEM66	0	0	0	0	0	0	1	0	1
TMEM68	1	0	1	1	0	1	1	0	1

TMEM69	1	0	1	1	0	1	1	0	1
TMEM70	1	0	1	1	0	1	1	0	1
TMEM71	0	0	0	1	0	0	1	0	1
TMEM77	1	0	1	1	0	1	1	0	1
TMEM79	1	0	1	1	0	1	1	0	1
TMEM8	1	0	1	1	0	1	1	1	1
TMEM80	1	0	1	1	0	1	1	0	0
TMEM85	1	0	1	1	0	1	1	0	1
TMEM86A	0	0	1	0	0	0	0	0	1
TMEM87A	1	0	1	1	0	1	1	0	1
TMEM87B	1	0	0	1	0	1	0	0	1
TMEM88	1	0	1	0	0	1	0	0	1
TMEM93	1	0	1	1	0	1	0	0	0
TMEM97	1	1	1	1	0	1	1	0	1
TMEM98	0	1	0	0	1	0	0	1	0
TMEM99	1	0	1	1	0	1	1	0	1
TMEM9B	1	0	0	1	0	0	0	0	1
TMEPAI	0	1	1	1	1	1	1	1	1
TMF1	1	0	1	1	0	1	1	0	1
TMLHE	1	0	0	0	0	0	1	0	1
TMOD4	1	0	0	0	0	0	0	0	0
TMPO	1	0	1	1	0	1	1	0	1
TMSB4X	1	1	1	1	1	1	1	1	1
TMTC1	0	1	0	0	1	0	0	1	0
TMTC2	1	0	1	1	0	1	1	0	1
TNC	0	1	0	0	1	0	0	1	0
TNF	1	0	1	1	0	1	1	0	1
TNFAIP1	1	0	1	1	0	1	1	0	1
TNFAIP2	0	0	0	1	0	1	0	0	1
TNFAIP3	0	0	0	1	0	1	1	0	1
TNFAIP6	0	0	0	0	0	0	1	0	0
TNFAIP8L1	0	0	1	0	0	0	0	0	1
TNFAIP8L2	1	0	1	1	0	1	1	0	1
TNFAIP8L3	0	1	0	0	1	0	0	1	0
TNFRSF10A	1	0	0	0	0	0	0	0	0
TNFRSF10C	0	0	0	0	0	1	0	0	0
TNFRSF10D	0	0	0	0	0	0	0	0	1
TNFRSF11A	0	0	0	0	0	1	0	1	1
TNFRSF11B	0	1	0	0	1	0	0	1	0
TNFRSF12A	0	0	0	0	0	1	0	0	0
TNFRSF13C	0	1	0	0	1	0	0	1	0
TNFRSF14	0	0	0	0	0	0	0	0	1
TNFRSF18	0	0	0	0	0	0	0	1	0
TNFRSF1A	0	0	1	1	0	1	1	0	1
TNFRSF21	0	1	0	0	1	0	0	1	0
TNFRSF25	0	1	0	0	1	0	0	1	0
TNFRSF4	0	0	0	0	0	0	0	1	0
TNFRSF9	0	0	0	0	0	1	1	0	1
TNFSF10	0	0	1	0	0	0	1	0	1
TNFSF12	1	0	1	1	0	1	1	0	0
TNFSF12-TNFSF13	1	0	1	1	0	1	1	0	0
TNFSF13	1	0	1	1	0	0	0	0	1
TNFSF13B	1	0	0	0	0	0	1	0	1
TNFSF4	0	0	0	0	0	0	1	0	0
TNFSF5IP1	1	0	1	1	0	1	1	0	1
TNFSF8	0	0	1	1	0	1	1	0	1
TNIK	1	1	1	1	0	1	1	1	1
TNIP1	1	0	1	1	0	1	1	0	1
TNKS	1	0	1	1	0	1	0	0	1
TNKS2	1	0	1	1	0	1	1	0	1
TNNI3K	1	0	1	1	0	1	1	0	1
TNNT1	0	1	0	0	1	0	0	1	0

TNP2	0	1	0	0	1	0	0	1	0
TNPO1	1	0	1	1	0	1	1	0	1
TNPO2	1	0	1	1	0	1	1	0	1
TNPO3	1	0	1	1	0	1	0	0	1
TNRC5	1	0	1	1	0	1	1	0	1
TNRC6A	1	0	1	1	0	1	1	0	1
TNRC6B	1	0	1	1	0	1	1	0	1
TNS1	1	0	0	0	0	1	1	0	0
TNS3	0	0	0	0	0	0	0	1	0
TNS4	0	1	0	0	0	0	0	1	0
TOB1	1	0	1	1	0	1	1	0	1
TOB2	0	0	0	0	0	0	0	0	1
TOE1	1	0	1	1	0	1	1	0	1
TOLLIP	0	0	0	0	0	1	0	0	1
TOM1	1	0	1	1	0	0	1	0	1
TOM1L2	0	0	1	0	0	1	0	0	1
TOMM20	1	0	0	1	0	0	0	0	1
TOMM22	1	0	0	0	0	0	0	0	1
TOMM34	0	0	1	0	0	1	0	0	1
TOMM40	1	0	1	1	0	1	1	0	1
TOMM7	1	0	1	1	0	1	1	1	1
TOMM70A	1	0	1	1	0	1	1	0	1
TOP1	0	0	1	0	0	0	0	0	0
TOP2A	1	0	1	1	0	1	1	0	1
TOP2B	1	0	1	0	0	1	1	0	1
TOP3A	1	0	1	1	0	1	1	0	1
TOP3B	1	0	1	1	0	1	1	0	1
TOPBP1	1	0	1	1	0	1	1	0	1
TOPORS	1	0	1	1	0	1	1	0	1
TOR1AIP1	1	0	1	1	0	1	1	0	1
TOR1AIP2	1	0	1	1	0	1	1	0	1
TOR3A	1	0	1	1	0	1	1	0	1
TOX	1	0	1	1	0	1	1	1	1
TP53	1	0	1	1	0	1	1	0	1
TP53AP1	1	0	1	1	0	1	1	0	1
TP53BP1	0	1	1	1	1	1	0	1	1
TP53BP2	1	0	1	1	0	1	1	0	1
TP53INP1	1	0	1	1	0	1	0	0	1
TP53INP2	0	1	0	1	0	1	0	1	1
TP53RK	0	0	0	0	0	0	1	0	1
TPCN1	1	0	1	1	0	1	1	0	1
TPD52L1	0	0	0	0	0	1	0	1	0
TPD52L2	0	0	1	1	0	1	0	0	1
TPM3	0	0	0	0	0	1	0	0	1
TPM4	1	0	1	0	0	0	0	0	0
TPMT	1	0	1	1	0	1	1	0	1
TPO	0	0	0	0	0	0	0	0	0
TPP1	1	0	1	1	0	1	1	0	1
TPP2	1	0	1	1	0	1	1	0	1
TPRKB	1	0	1	1	0	1	1	0	1
TPST1	0	1	0	0	1	1	0	1	1
TPST2	0	0	0	0	0	0	0	0	1
TPT1	1	0	1	1	0	1	1	0	1
TPX2	1	0	1	1	0	1	1	0	1
TRA16	1	0	1	1	0	1	1	0	1
TRA2A	0	0	0	0	0	0	0	0	1
TRADD	0	1	0	0	1	1	0	1	1
TRAF1	1	0	1	0	0	1	0	0	1
TRAF3IP2	1	0	1	1	0	1	1	0	1
TRAF3IP3	1	0	1	0	0	1	1	0	1
TRAF4	0	0	0	0	0	0	0	0	1
TRAF5	1	0	1	1	0	1	1	0	1

TRAF6	1	0	1	1	0	1	1	0	1
TRAIP	0	0	0	0	0	1	0	0	1
TRAK1	1	0	0	0	0	0	1	0	0
TRAK2	1	0	1	1	0	1	1	1	1
TRAM1	1	0	0	1	0	0	1	0	1
TRAM2	1	0	1	1	0	1	1	0	1
TRAP1	0	0	0	1	0	1	0	0	0
TRAPPC1	1	0	1	1	0	1	1	0	1
TRAPPC2	1	1	1	1	1	1	1	1	1
TRAPPC3	0	0	0	1	0	0	0	0	1
TRAPPC4	1	0	1	1	0	1	1	0	1
TRAPPC6A	1	0	0	1	0	1	1	0	1
TRAPPC6B	1	0	1	1	0	1	1	0	1
TREM1	1	0	0	1	0	1	1	0	1
TREM2	0	0	0	1	0	1	0	0	0
TREX1	1	0	1	1	0	1	0	0	1
TRIAP1	1	0	1	1	0	1	1	0	1
TRIB1	1	0	0	0	0	0	0	0	1
TRIB2	1	0	0	0	1	1	0	1	1
TRIM11	1	0	1	0	0	1	0	0	1
TRIM14	0	0	0	0	0	1	1	0	1
TRIM17	0	1	0	0	0	0	0	1	0
TRIM21	0	0	1	1	0	1	1	0	1
TRIM22	1	0	1	1	0	1	1	0	1
TRIM23	1	0	1	1	0	1	1	0	1
TRIM24	1	0	1	1	0	1	1	0	1
TRIM25	1	0	1	1	0	1	1	0	1
TRIM26	1	0	1	1	0	1	1	0	1
TRIM28	0	0	0	0	0	0	0	1	0
TRIM29	0	1	0	0	0	0	0	0	0
TRIM3	0	0	0	0	0	0	1	0	0
TRIM32	0	1	1	0	1	1	1	1	1
TRIM34	1	0	1	1	0	1	0	0	1
TRIM35	0	0	1	1	0	1	0	0	1
TRIM36	0	1	1	0	1	1	0	1	1
TRIM38	1	0	1	1	0	1	1	0	1
TRIM39	1	0	1	1	0	1	1	0	1
TRIM4	1	0	1	1	0	1	1	1	1
TRIM41	0	0	0	0	0	0	0	0	1
TRIM44	1	1	1	1	1	1	1	1	1
TRIM45	1	1	1	0	1	1	0	1	1
TRIM46	1	0	1	1	0	1	1	0	1
TRIM47	0	1	0	0	0	0	0	1	0
TRIM5	1	0	1	1	0	1	1	0	1
TRIM54	1	1	0	0	1	0	0	0	0
TRIM56	0	0	0	0	0	1	0	0	0
TRIM58	0	1	0	0	1	0	0	1	0
TRIM6	0	0	0	0	0	0	0	0	1
TRIM61	0	0	0	0	0	0	0	0	1
TRIM62	0	1	0	0	1	0	0	1	0
TRIM67	0	1	0	0	1	0	0	1	0
TRIM68	0	0	0	0	0	1	1	0	1
TRIM7	0	1	1	0	0	1	0	1	1
TRIM73	0	1	0	0	1	0	0	1	0
TRIM8	0	0	0	0	0	0	0	0	1
TRIM9	0	1	0	0	1	0	0	1	0
TRIO	1	0	1	1	0	1	1	0	1
TRIOBP	0	0	0	0	0	1	0	0	1
TRIP10	0	0	0	0	0	0	0	0	1
TRIP11	1	0	1	1	0	1	1	0	1
TRIP12	1	0	1	1	0	1	1	0	1
TRIP13	0	0	1	0	0	1	0	0	1

TRIP4	1	0	1	1	0	1	0	0	1
TRIP6	0	0	0	0	0	0	1	0	1
TRIT1	0	0	0	0	0	1	0	0	1
TRMT1	1	0	1	1	0	1	1	0	1
TRMT12	0	0	1	1	0	1	1	0	1
TRMU	1	0	0	0	0	0	0	0	0
TRNT1	1	0	1	0	0	0	1	0	1
TROAP	1	0	1	1	0	1	1	0	1
TROVE2	1	0	1	1	0	1	1	0	1
TRPC4	0	1	0	0	1	0	0	1	0
TRPC4AP	0	0	0	0	0	1	0	0	1
TRPC5	0	1	1	0	1	1	0	1	0
TRPM3	0	1	0	0	1	0	0	0	0
TRPM4	0	1	0	0	1	0	0	1	0
TRPM6	0	1	0	0	1	1	0	1	0
TRPM7	1	0	1	1	0	1	1	0	1
TRPS1	1	0	1	1	0	1	1	0	1
TRPT1	0	0	0	0	0	1	0	0	1
TRPV2	0	0	0	0	0	1	0	0	0
TRPV4	0	0	0	0	0	1	0	1	1
TRRAP	0	0	0	0	0	0	0	0	1
TRSPAP1	1	0	1	1	0	1	1	0	1
TRUB1	1	0	0	0	0	1	1	0	1
TRUB2	1	0	1	1	0	1	1	0	1
TSC1	0	0	1	0	0	1	0	0	1
TSC22D1	0	1	1	1	0	1	1	1	1
TSC22D2	1	0	1	1	0	1	1	0	1
TSC22D3	1	1	1	0	1	1	0	1	1
TSEN2	0	0	1	0	0	1	1	0	1
TSEN34	1	0	1	0	0	1	1	0	1
TSEN54	0	0	0	0	0	1	0	1	0
TSFM	1	0	0	0	0	1	0	0	1
TSG101	1	0	1	1	0	1	1	0	1
TSGA14	1	0	1	1	0	1	1	0	1
TSKS	1	0	1	1	0	1	1	0	1
TSLP	0	1	1	0	1	1	0	1	1
TSN	1	0	1	1	0	1	1	0	1
TSNAX	1	0	1	1	0	1	1	0	1
TSP50	0	1	0	0	1	0	0	0	0
TSPAN1	1	1	0	0	0	0	0	0	0
TSPAN12	0	1	1	0	1	1	0	1	1
TSPAN13	0	1	1	0	0	0	0	1	1
TSPAN14	0	0	0	0	0	1	0	0	0
TSPAN15	0	1	0	0	0	0	0	1	0
TSPAN16	0	0	0	0	0	0	1	0	1
TSPAN18	0	1	0	0	1	0	0	1	0
TSPAN2	0	0	0	0	0	1	0	0	0
TSPAN31	0	0	1	1	0	1	1	0	1
TSPAN32	0	0	1	0	0	1	0	0	1
TSPAN33	0	1	1	0	1	1	1	1	1
TSPAN4	0	0	0	0	0	1	0	0	0
TSPAN5	0	1	1	0	1	1	0	1	1
TSPAN7	0	1	0	0	1	1	0	0	1
TSPAN9	0	1	0	0	1	0	0	1	0
TSPYL1	1	0	1	1	0	1	1	0	1
TSPYL5	0	1	1	0	1	1	0	1	1
TSR1	1	0	1	1	0	1	1	0	1
TSSC1	1	0	1	1	0	1	0	0	1
TSSC4	1	0	1	1	0	1	0	0	1
TSSK6	1	0	1	1	0	1	1	0	1
TST	1	0	1	1	0	1	1	0	1
TSTA3	0	0	0	0	0	1	0	0	0

TTBK2	0	0	1	1	0	1	1	0	1
TTC1	0	0	0	1	0	1	0	1	1
TTC13	1	0	1	1	0	1	1	0	1
TTC14	0	1	1	0	1	1	0	1	1
TTC15	1	0	1	1	0	1	0	0	1
TTC16	0	0	1	0	0	1	0	1	1
TTC17	0	0	1	1	0	1	1	0	1
TTC19	1	0	1	1	0	1	1	0	0
TTC21A	1	0	1	1	0	1	1	0	1
TTC23	1	0	1	1	0	1	1	1	1
TTC25	1	0	1	1	0	1	1	0	1
TTC26	1	0	1	1	0	1	1	0	1
TTC3	1	0	1	1	0	1	1	0	1
TTC4	0	0	0	1	0	1	0	0	1
TTC5	1	0	1	1	0	1	1	0	1
TTC7A	0	0	0	1	0	0	0	0	1
TTC8	1	1	1	1	0	1	0	0	1
TTC9C	1	0	1	1	0	1	1	0	1
TTF1	1	1	0	0	1	0	0	1	1
TTF2	0	0	1	1	0	1	1	0	1
TTK	1	0	1	0	0	1	0	0	1
TTL	1	0	1	1	0	1	1	0	1
TTLL1	1	0	1	1	0	1	1	0	1
TTLL5	1	0	1	1	0	1	1	0	1
TTRAP	1	0	1	1	0	1	1	0	1
TTYH2	1	0	1	1	0	1	1	0	1
TUB	0	1	1	0	1	0	0	1	1
TUBB	1	0	1	1	0	1	0	0	1
TUBB2A	0	1	1	0	1	0	0	1	1
TUBB2B	0	1	1	0	1	0	0	1	0
TUBB3	0	1	0	0	0	0	0	1	0
TUBB4	0	1	0	0	1	0	0	1	0
TUBB6	1	0	1	0	0	1	0	1	1
TUBD1	1	0	1	1	0	1	1	0	1
TUBE1	1	1	1	1	1	1	1	1	1
TUBG1	1	0	1	1	0	1	1	0	1
TUBG2	0	0	0	0	0	0	0	1	0
TUBGCP2	1	0	0	1	0	1	0	0	1
TUBGCP3	0	0	0	0	0	0	0	0	1
TUBGCP5	0	0	0	0	0	1	0	0	1
TUBGCP6	1	0	0	0	0	1	0	0	0
TUFM	1	0	0	1	0	0	0	0	0
TUFT1	0	1	0	0	0	0	0	0	0
TULP2	1	0	1	1	0	1	1	0	1
TULP3	0	0	1	0	0	1	1	0	1
TUSC1	0	1	0	0	0	0	0	1	0
TUSC2	1	0	0	1	0	0	0	0	1
TUSC4	1	0	1	1	0	1	1	0	1
TWISTNB	1	0	1	1	0	1	0	0	1
TWSG1	0	0	1	0	0	1	0	0	1
TXK	0	0	0	0	0	0	1	0	0
TXLNA	0	0	0	0	0	1	0	0	1
TXN	1	0	0	1	0	1	1	0	1
TXN2	1	0	1	1	0	1	1	0	1
TXNDC10	1	0	1	1	0	1	1	0	1
TXNDC11	0	0	0	1	0	0	0	0	0
TXNDC12	1	0	1	1	0	1	1	0	1
TXNDC13	1	0	0	0	0	1	0	0	1
TXNDC14	1	0	1	1	0	1	1	0	1
TXNDC4	1	0	1	1	0	1	1	0	1
TXNDC5	0	1	1	1	1	1	1	1	1
TXNDC9	1	0	1	1	0	1	1	0	1

TXNIP	1	0	1	1	0	1	1	0	1
TXNL1	1	0	1	1	0	1	1	0	1
TXNL4B	1	0	1	1	0	1	1	0	1
TXNL5	1	0	1	1	0	1	1	0	1
TXNL6	0	1	0	0	0	0	0	0	0
TXNRD1	1	0	1	1	0	1	0	0	1
TXNRD2	1	0	1	1	0	1	1	0	1
TYK2	0	0	1	1	0	1	1	0	1
TYMS	0	1	0	0	0	1	0	0	0
TYRO3	0	0	0	0	0	1	0	1	1
TYROBP	0	0	0	1	0	1	0	0	1
TYSND1	1	0	0	1	0	1	1	0	1
U1SNRNPBP	0	0	0	1	0	1	0	0	1
U2AF1	1	0	1	1	0	1	1	0	1
U2AF1L4	1	0	1	1	0	1	0	0	1
U2AF2	0	0	0	0	0	1	1	0	1
UBAP1	1	0	1	1	0	1	1	0	1
UBAP2	1	0	1	0	0	1	0	0	1
UBAP2L	1	0	1	1	0	1	1	0	1
UBB	0	0	0	0	0	0	0	0	1
UBC	1	0	1	1	0	1	1	0	1
UBD	0	1	0	0	0	0	0	0	0
UBE1	1	1	1	1	1	1	1	1	1
UBE1DC1	1	0	1	1	0	1	1	0	1
UBE1L	1	0	1	0	0	1	1	0	1
UBE1L2	1	0	1	1	0	1	1	0	1
UBE2A	1	1	1	1	1	0	0	1	0
UBE2B	1	0	1	1	0	1	1	0	1
UBE2C	0	0	0	0	0	1	0	0	1
UBE2D2	0	0	0	1	0	1	1	0	1
UBE2D3	1	0	1	1	0	1	1	0	1
UBE2D4	1	0	1	1	0	1	1	0	1
UBE2E2	0	0	0	1	0	1	1	1	1
UBE2E3	0	1	0	0	1	1	1	1	1
UBE2G2	0	0	1	1	0	1	0	0	1
UBE2J1	0	0	0	0	0	0	1	0	0
UBE2L6	0	0	0	0	0	1	0	0	0
UBE2M	0	1	1	0	1	0	0	1	0
UBE2N	0	0	1	0	0	1	1	0	1
UBE2Q1	1	0	0	1	0	0	0	0	1
UBE2Q2	0	0	0	0	0	0	1	0	1
UBE2R2	1	0	0	1	0	0	0	0	0
UBE2T	1	0	1	1	0	1	0	0	1
UBE2V1	1	0	0	1	0	1	1	0	1
UBE2W	1	0	1	1	0	1	1	0	1
UBE2Z	1	0	1	1	0	1	0	0	1
UBE3A	1	0	1	1	0	1	1	0	1
UBE3B	1	0	1	1	0	1	1	0	1
UBE3C	0	0	0	0	0	0	0	0	1
UBE4A	1	0	1	1	0	1	1	0	1
UBE4B	1	0	0	1	0	0	1	0	1
UBIAD1	1	0	1	1	0	1	1	0	1
UBL3	1	0	1	1	0	1	1	0	1
UBL4A	0	1	0	0	0	0	0	0	0
UBL5	1	0	0	1	0	1	0	0	1
UBL7	1	0	1	1	0	1	0	0	1
UBLCP1	1	0	1	1	0	1	1	0	1
UBN1	1	0	1	1	0	1	1	0	1
UBP1	1	0	1	1	0	1	0	0	1
UBQLN1	1	0	1	1	0	1	1	0	1
UBQLN2	1	1	1	1	0	1	1	1	1
UBQLN4	1	0	1	1	0	1	0	0	1

UBR1	1	0	1	1	0	1	1	0	1
UBR2	1	0	1	1	0	1	1	0	1
UBTD1	1	0	1	1	0	1	0	0	1
UBTF	1	0	1	1	0	1	0	0	0
UBXD4	0	0	0	1	0	1	1	0	1
UBXD5	1	0	1	1	0	1	1	0	1
UBXD6	1	0	1	1	0	1	1	0	1
UBXD8	1	0	1	1	0	1	1	0	1
UCHL1	0	1	1	0	1	0	0	1	1
UCHL3	1	0	1	1	0	1	1	0	1
UCHL5	1	0	1	1	0	1	1	0	1
UCK2	1	0	1	1	0	1	1	0	1
UCN	0	1	0	0	1	0	0	1	0
UCP1	0	1	0	0	1	0	0	1	0
UCRC	1	0	1	1	0	1	1	0	1
UFC1	1	0	1	1	0	1	1	0	1
UFD1L	1	0	1	1	0	1	1	0	1
UFM1	1	0	1	1	0	1	1	0	1
UGCG	1	0	1	1	0	1	1	0	1
UGCGL1	1	0	0	1	0	1	0	0	1
UGCGL2	0	1	0	0	0	0	0	0	0
UGDH	1	0	0	0	0	1	0	0	1
UGP2	1	0	1	1	0	1	1	0	1
UHMK1	0	0	1	1	0	1	0	0	1
UHRF1	1	0	1	0	0	1	1	0	1
ULBP3	0	0	0	0	1	0	0	0	0
ULK3	0	0	1	1	0	0	0	0	1
UMOD	0	1	0	0	0	0	0	0	0
UNC13B	0	0	1	1	0	1	1	0	1
UNC45A	1	0	1	1	0	1	1	0	1
UNC50	1	0	1	1	0	1	1	0	1
UNC84A	1	0	0	1	0	0	1	0	0
UNC84B	1	0	1	1	0	1	0	0	1
UNC93A	0	1	0	1	1	0	0	1	0
UNQ473	0	1	0	0	1	0	0	0	0
UNQ501	1	0	0	0	0	1	0	0	1
UNQ5830	0	1	0	0	0	0	0	1	0
UNQ846	0	1	0	0	0	0	0	0	0
UNQ9217	1	0	0	0	0	0	0	0	1
UPB1	0	1	1	0	1	0	0	1	0
UPF2	0	0	0	0	0	0	0	0	1
UPF3A	1	0	0	1	0	0	0	0	1
UPF3B	0	1	0	0	1	0	0	1	1
UPK3A	0	0	0	0	1	0	0	0	0
UQCR	1	0	1	1	0	1	1	0	1
UQCRB	1	0	1	1	0	1	1	0	1
UQCRC2	1	0	1	1	0	1	1	0	1
UQCRFS1	0	0	0	1	0	1	1	0	1
UQCRH	1	0	1	1	0	1	1	0	1
UQCRQ	1	0	0	1	0	1	0	0	1
URG4	1	0	1	1	0	1	1	0	1
UROD	1	0	1	1	0	1	1	0	1
UROS	1	0	1	1	0	1	1	0	1
URP2	1	0	1	1	0	1	0	0	1
USF1	1	0	1	1	0	1	1	0	1
USH1G	0	1	0	0	1	0	0	1	0
USHBP1	1	0	1	1	0	1	1	0	1
USMG5	1	0	1	1	0	1	1	0	1
USP1	1	0	1	1	0	1	0	0	1
USP10	1	0	0	1	0	1	0	0	1
USP11	0	1	0	0	1	0	1	1	1
USP12	1	0	1	1	0	1	1	0	1

USP13	0	1	1	1	1	1	1	1	1
USP15	1	0	1	1	0	1	1	0	1
USP16	1	0	0	1	0	1	1	0	1
USP19	0	0	0	0	0	1	1	0	0
USP2	0	1	1	0	1	1	0	1	1
USP20	1	0	1	1	0	1	1	0	1
USP21	0	0	0	0	0	0	0	0	1
USP25	1	0	1	0	0	0	0	0	1
USP3	1	0	0	1	0	0	1	0	1
USP30	1	0	1	1	0	1	1	0	1
USP32	1	0	1	1	0	1	1	0	1
USP33	0	0	1	0	0	1	0	0	1
USP34	1	0	0	0	0	0	0	0	0
USP35	1	0	1	1	0	1	1	0	1
USP36	1	0	0	1	0	0	1	0	0
USP37	1	0	1	1	0	1	1	0	1
USP38	1	0	1	1	0	1	1	0	1
USP4	1	0	0	1	0	0	0	0	1
USP46	0	0	1	0	0	1	0	0	1
USP47	0	0	0	1	1	0	1	1	1
USP48	1	0	0	1	0	0	1	0	1
USP49	1	0	1	1	0	1	1	0	1
USP5	1	0	1	1	0	1	1	0	1
USP51	0	1	1	0	1	1	1	0	1
USP52	1	0	1	1	0	1	1	0	1
USP6	0	1	0	0	0	0	0	1	0
USP8	1	0	1	1	0	1	1	0	1
USP9X	1	1	1	1	1	1	1	1	1
USPL1	1	0	1	1	0	1	1	0	1
UST	0	1	1	1	1	1	0	1	1
UTF1	0	1	0	0	1	0	0	1	0
UTP11L	1	0	1	1	0	1	1	0	1
UTP14A	1	1	1	1	1	1	0	0	1
UTP15	1	0	1	1	0	1	1	0	1
UTP20	0	1	1	0	1	0	0	1	1
UTX	0	1	0	0	0	0	0	1	0
UVRAG	0	0	0	0	0	1	0	0	1
UXS1	1	0	1	1	0	1	1	0	1
UXT	0	1	1	1	1	1	0	0	1
VAMP1	0	0	0	1	0	1	0	0	1
VAMP3	1	0	1	1	0	1	0	0	1
VAMP4	1	0	1	1	0	1	1	0	1
VAMP8	1	0	1	1	0	1	1	0	1
VANGL2	0	1	0	0	1	0	0	1	0
VAPA	0	0	0	1	0	1	0	0	1
VASH1	0	0	1	1	0	1	0	0	0
VASP	1	0	0	1	0	1	0	0	1
VAT1	0	0	1	0	0	1	1	1	1
VAV3	1	0	1	1	0	1	1	0	1
VAX1	0	1	0	0	1	0	0	1	0
VAX2	0	1	0	0	1	1	0	1	0
VBP1	0	0	0	0	0	1	0	0	1
VCPIP1	1	0	1	1	0	1	1	0	1
VDAC1	0	0	0	0	0	0	0	1	1
VDAC2	1	0	0	1	0	1	0	0	1
VDP	1	0	1	1	0	1	1	0	1
VDR	1	1	1	1	1	1	1	1	1
VEGFB	0	0	0	0	0	0	0	1	0
VEPH1	0	1	1	0	1	0	0	1	0
VEZT	0	0	1	1	0	1	1	0	1
VGLL4	0	1	0	0	1	0	0	1	1
VIL2	1	0	0	1	0	1	1	0	1

VILL	0	0	0	0	0	0	1	0	1
VIM	1	0	1	1	0	1	1	0	1
VIPR1	0	1	0	0	1	0	0	1	1
VKORC1	0	0	1	1	0	1	0	0	0
VMAC	1	0	0	1	0	1	1	0	1
VMO1	1	1	1	1	0	1	0	1	0
VNN1	0	0	0	0	0	1	0	0	0
VNN2	1	0	0	0	0	0	1	0	1
VNN3	0	0	0	0	0	0	1	0	1
VPRBP	0	0	0	0	0	1	0	0	1
VPS11	1	0	1	1	0	1	1	0	1
VPS13A	0	1	0	0	1	1	0	1	1
VPS13B	1	0	1	1	0	1	1	0	1
VPS13C	0	1	1	1	1	1	1	1	1
VPS16	1	0	1	1	0	1	1	0	1
VPS24	1	0	0	0	0	0	1	0	1
VPS25	1	0	1	1	0	1	0	0	1
VPS26A	1	0	1	1	0	1	1	0	1
VPS26B	1	0	1	1	0	1	0	0	1
VPS28	0	0	0	0	0	1	0	0	1
VPS29	1	0	1	1	0	1	1	0	1
VPS33A	0	0	0	0	0	1	0	0	1
VPS33B	1	0	0	1	0	1	0	0	1
VPS35	1	0	1	1	0	1	1	0	1
VPS37A	1	0	1	1	0	1	1	0	1
VPS37B	0	0	0	0	0	0	0	1	0
VPS37C	1	0	1	1	0	1	0	0	0
VPS39	1	0	1	1	0	1	1	0	1
VPS41	0	0	0	0	0	1	0	0	0
VPS4B	1	0	1	1	0	1	1	0	1
VPS52	1	0	1	1	0	1	1	0	1
VPS53	0	0	0	0	0	0	0	1	1
VPS54	1	0	0	1	0	1	1	0	1
VPS72	1	0	1	0	0	1	0	0	1
VRK2	1	0	1	1	0	1	0	0	1
VRK3	1	0	1	1	0	1	1	0	1
VSIG4	0	1	1	1	1	1	0	1	0
VSNL1	0	1	1	0	1	0	0	1	1
VSX1	0	1	1	0	1	0	0	1	0
VTI1A	1	0	1	1	0	1	1	0	1
VTI1B	1	0	0	0	0	0	0	0	0
VTN	0	0	0	0	0	0	1	1	0
VWCE	0	1	0	0	1	0	0	1	0
WAC	1	0	1	1	0	1	1	0	1
WAPAL	1	0	1	1	0	1	1	0	1
WARS	1	0	1	1	0	1	1	0	1
WAS	0	1	0	1	1	1	0	0	1
WASF2	1	0	0	1	0	0	1	0	1
WASF3	0	1	1	0	1	0	0	1	0
WASL	1	0	1	1	0	1	0	0	1
WBP2	1	0	1	1	0	1	1	0	1
WBP4	1	0	1	1	0	1	1	0	1
WBP5	0	1	1	1	1	1	1	0	1
WBSCR18	1	0	1	1	0	1	1	0	1
WBSCR22	1	0	1	1	0	1	1	0	1
WDFY2	0	0	0	0	0	0	0	0	1
WDFY3	1	0	1	1	0	1	1	0	1
WDHD1	1	0	1	1	0	1	1	0	1
WDR1	1	0	1	1	0	1	1	0	1
WDR12	1	1	1	1	1	1	1	1	1
WDR19	1	0	1	1	0	1	0	0	1
WDR20	1	1	1	1	1	1	1	1	1

WDR22	0	0	1	1	0	1	1	1	1
WDR23	1	0	1	1	0	1	1	0	1
WDR24	1	1	0	0	1	0	0	1	0
WDR25	1	0	1	1	0	1	1	0	1
WDR26	1	0	1	1	0	1	1	0	1
WDR33	1	0	1	1	0	1	1	0	1
WDR35	0	1	0	0	1	0	0	1	1
WDR36	1	0	1	0	0	1	0	0	1
WDR37	1	0	1	1	0	1	1	0	1
WDR4	1	0	1	1	0	1	0	0	1
WDR40A	1	0	1	1	0	1	1	0	1
WDR41	1	0	0	0	0	0	0	0	0
WDR42A	1	0	1	1	0	1	1	0	1
WDR44	1	0	1	0	1	1	0	0	1
WDR45	1	1	1	1	0	1	0	0	1
WDR45L	0	0	0	0	0	1	0	0	0
WDR46	1	0	1	1	0	1	1	0	1
WDR47	1	0	1	1	0	1	1	0	1
WDR48	0	0	0	0	0	0	0	0	1
WDR49	1	0	0	1	0	0	1	0	1
WDR51A	1	0	1	1	0	0	1	0	1
WDR51B	1	0	1	1	0	1	1	0	1
WDR53	1	0	1	1	0	1	1	0	1
WDR54	0	0	1	0	0	1	1	0	1
WDR55	0	0	0	0	0	0	0	0	1
WDR57	1	0	1	1	0	1	1	0	1
WDR5B	1	0	1	1	0	1	1	0	1
WDR6	0	0	0	0	0	1	0	0	1
WDR61	1	0	1	1	0	1	1	0	1
WDR62	1	0	1	1	0	1	1	0	1
WDR63	0	0	1	1	0	1	1	0	1
WDR65	1	1	1	1	1	1	1	1	1
WDR67	0	0	0	0	0	0	0	0	1
WDR68	1	0	1	1	0	1	1	0	1
WDR7	1	0	1	1	0	1	0	0	1
WDR70	1	0	1	1	0	1	1	0	1
WDR71	1	0	1	1	0	1	1	0	1
WDR73	1	0	1	1	0	1	1	0	1
WDR74	1	0	1	1	0	1	1	0	1
WDR75	1	0	1	1	0	1	1	0	1
WDR77	1	0	1	1	0	1	1	0	1
WDR79	1	0	1	1	0	1	1	0	1
WDR8	0	1	0	0	1	0	0	1	0
WDR81	1	0	1	1	0	1	0	0	1
WDSOF1	1	0	1	1	0	1	1	0	1
WDSUB1	1	0	1	1	0	1	1	0	1
WEE1	0	0	0	0	0	1	0	0	1
WFIKKN2	0	1	0	0	1	0	0	0	0
WFS1	0	1	1	0	1	0	0	1	1
WHSC1L1	1	0	1	1	0	1	1	0	1
WHSC2	1	0	0	0	0	0	1	0	1
WIBG	1	0	1	1	0	1	1	0	1
WIPI1	1	0	0	1	0	1	0	1	1
WIT1	0	1	1	0	1	1	0	1	1
WNK1	0	0	1	0	0	1	1	0	1
WNK3	0	1	1	0	1	1	0	1	1
WNT11	0	1	1	0	1	1	0	1	1
WNT5A	1	1	1	0	1	1	0	1	1
WNT5B	0	0	1	1	1	1	1	1	1
WNT7A	0	1	0	0	1	0	0	1	0
WRB	0	0	1	0	0	1	0	0	1
WRN	1	1	1	1	1	1	0	1	1

WRNIP1	1	0	1	0	0	0	1	0	1
WSB1	1	0	0	1	0	0	1	0	1
WSB2	1	0	1	1	0	1	1	1	1
WT1	0	1	1	0	1	1	0	1	1
WTAP	1	0	1	1	0	1	1	0	1
WWC2	0	0	0	0	0	0	1	0	0
WWC3	0	1	1	0	1	1	0	1	1
WWOX	1	0	1	1	0	1	1	0	1
WWP1	0	0	1	0	0	1	0	0	1
WWP2	0	0	0	1	0	0	0	0	0
WWTR1	0	1	1	0	1	1	0	1	1
XAB1	1	1	1	1	0	1	1	0	1
XAB2	1	0	1	1	0	1	1	0	1
XBP1	1	0	1	1	0	1	1	0	1
XK	0	1	0	0	1	0	0	1	0
XKR4	0	1	1	0	1	0	0	1	0
XKR6	0	1	0	0	1	1	0	1	1
XPA	0	0	0	1	0	0	0	0	0
XPC	1	0	1	1	0	1	1	0	1
XPNPEP1	0	0	0	0	0	0	0	0	1
XPNPEP2	0	1	0	0	1	0	0	0	0
XPO1	1	0	1	1	0	1	1	0	1
XPO4	0	0	0	1	0	0	1	0	0
XPO5	1	0	1	1	0	1	1	0	1
XPO6	1	0	1	1	0	0	0	0	0
XPR1	0	0	0	0	0	0	1	0	0
XRCC1	1	0	1	1	0	1	1	0	1
XRCC2	1	0	1	1	0	1	1	0	1
XRCC3	0	0	1	0	0	1	0	0	0
XRCC4	1	0	1	1	0	1	1	0	1
XRCC6	1	0	1	1	0	1	1	0	1
XRCC6BP1	1	0	1	1	0	1	1	0	1
XRN1	1	0	1	1	0	1	1	0	1
XRN2	1	0	1	1	0	1	1	0	1
XTP3TPA	0	0	0	0	0	1	0	0	1
XYLT1	0	1	0	0	0	0	1	1	1
YAF2	1	0	0	0	0	0	0	0	1
YARS	1	0	1	1	0	1	1	0	1
YARS2	1	0	1	1	0	1	1	0	1
YEATS4	0	0	0	1	0	1	1	0	1
YIF1A	0	1	1	1	1	1	1	1	1
YIF1B	1	0	1	0	0	1	0	0	1
YIPF1	1	0	1	1	0	1	1	0	1
YIPF2	1	0	1	1	0	1	0	0	1
YIPF3	1	0	1	1	0	1	1	0	1
YIPF4	1	0	1	1	0	1	1	0	1
YIPF5	1	1	1	1	0	1	1	1	1
YIPF6	1	1	1	1	1	1	0	0	1
YME1L1	1	0	1	1	0	1	1	0	1
YOD1	1	0	1	1	0	1	1	0	1
YPEL2	0	0	0	0	0	0	1	0	0
YPEL3	0	0	1	0	0	0	0	0	0
YPEL4	0	0	0	0	0	1	0	1	0
YRDC	1	0	1	1	0	1	0	0	1
YTHDC1	0	0	1	0	0	1	0	0	1
YTHDC2	0	1	0	0	1	0	0	1	0
YTHDF1	0	0	0	1	0	1	0	0	0
YTHDF2	1	0	0	1	0	1	1	0	1
YTHDF3	1	0	1	1	0	1	1	0	1
YWHAB	1	0	0	0	0	1	0	0	0
YWHAG	0	0	0	0	0	0	0	0	1
YWHAH	0	0	0	0	0	1	0	0	1

YWHAQ	0	1	0	0	0	0	0	0	0
YWHAZ	1	0	1	1	0	1	1	0	1
YY1	1	0	1	1	0	1	1	0	1
YY1AP1	1	0	1	1	0	1	1	0	1
ZADH1	1	0	1	1	0	1	1	0	1
ZADH2	1	0	1	1	0	1	1	0	1
ZBED3	1	0	1	1	0	1	1	0	1
ZBED4	0	0	0	0	0	0	0	0	1
ZBTB11	1	0	1	1	0	1	1	0	1
ZBTB16	0	1	1	0	1	1	1	1	1
ZBTB2	1	0	1	1	0	1	1	0	1
ZBTB20	1	0	1	1	0	1	1	0	1
ZBTB22	1	0	1	1	0	1	1	0	1
ZBTB24	0	0	0	1	0	1	0	1	0
ZBTB25	1	0	1	1	0	1	1	0	1
ZBTB26	1	0	1	1	0	1	1	0	1
ZBTB3	1	0	1	0	0	1	0	0	1
ZBTB32	0	0	0	0	1	0	0	0	0
ZBTB33	0	1	1	1	1	1	1	1	0
ZBTB39	1	0	0	0	0	0	0	0	0
ZBTB4	1	0	1	1	0	1	1	0	1
ZBTB41	1	0	1	1	0	1	1	0	1
ZBTB43	0	0	0	0	0	0	0	0	1
ZBTB5	0	0	0	1	0	1	1	0	1
ZBTB7A	0	0	1	0	0	1	0	0	1
ZBTB7B	1	0	1	0	0	0	0	0	1
ZBTB9	1	0	1	1	0	1	1	0	1
ZC3H10	1	0	1	1	0	1	1	0	1
ZC3H11A	1	0	0	0	0	1	1	0	0
ZC3H12A	1	0	1	1	0	1	1	1	1
ZC3H3	1	0	0	0	0	1	0	0	0
ZC3H7A	1	0	1	1	0	1	1	0	1
ZC3H8	0	0	0	1	0	0	0	0	0
ZC3HAV1	1	0	1	1	0	1	1	0	1
ZC3HC1	1	1	1	1	0	1	1	1	1
ZCCHC11	0	1	0	0	0	0	0	1	1
ZCCHC12	0	1	0	0	1	0	0	1	0
ZCCHC14	0	1	0	0	1	1	0	1	0
ZCCHC17	1	0	1	1	0	1	1	0	1
ZCCHC3	1	0	1	1	0	1	1	0	1
ZCCHC6	1	0	1	1	0	1	1	0	1
ZCCHC7	1	0	1	1	0	1	1	0	1
ZCCHC8	1	0	0	0	0	1	0	0	1
ZCCHC9	1	0	1	1	0	1	1	0	1
ZCRB1	1	0	1	1	0	1	1	0	1
ZCWPW1	1	0	1	1	0	1	1	0	1
ZDHHC1	0	1	1	0	0	0	0	1	0
ZDHHC12	1	0	1	1	0	1	1	0	1
ZDHHC14	0	0	1	1	0	1	0	0	1
ZDHHC16	1	0	1	1	0	1	0	0	1
ZDHHC17	1	0	0	0	0	1	0	0	1
ZDHHC2	1	0	1	1	1	1	1	0	1
ZDHHC21	0	1	0	0	1	0	0	1	0
ZDHHC22	0	1	0	0	1	0	0	1	0
ZDHHC23	0	1	0	0	0	0	1	1	1
ZDHHC24	0	1	0	1	1	1	0	1	1
ZDHHC3	1	0	1	1	0	1	1	0	1
ZDHHC5	1	0	1	1	0	1	1	0	1
ZDHHC6	1	0	1	1	0	1	1	0	1
ZDHHC7	0	0	1	1	0	1	0	0	1
ZDHHC9	0	1	0	0	0	1	0	0	0
ZFAND1	1	0	1	1	0	1	1	0	1

ZFAND2A	1	0	0	1	0	0	0	0	0
ZFAND2B	0	0	0	1	0	1	1	0	1
ZFAND3	1	0	1	1	0	1	1	0	1
ZFP106	0	0	0	1	0	0	0	0	0
ZFP161	1	0	1	1	0	1	1	0	1
ZFP28	0	0	1	0	0	1	0	0	1
ZFP3	0	0	0	0	0	1	0	0	1
ZFP30	1	0	1	1	0	1	1	1	1
ZFP36	0	0	0	0	0	0	0	0	1
ZFP36L1	1	0	1	1	0	1	1	1	1
ZFP90	0	0	0	0	0	0	0	0	1
ZFP91	1	0	1	1	0	1	1	0	1
ZFPL1	1	0	1	1	0	1	1	0	1
ZFX	1	0	1	1	0	1	1	0	1
ZFYVE1	0	0	0	1	0	1	0	0	1
ZFYVE16	0	0	0	0	0	0	1	0	1
ZFYVE19	0	0	1	1	0	1	1	0	1
ZFYVE20	1	0	1	1	0	1	1	0	1
ZFYVE21	0	0	1	0	0	1	0	0	0
ZFYVE26	1	0	1	1	0	1	1	0	1
ZFYVE28	0	1	0	0	1	0	0	1	0
ZHX1	1	0	1	0	0	0	0	0	0
ZHX2	1	1	1	1	1	1	1	1	1
ZIC2	0	0	0	0	0	0	0	1	0
ZIK1	0	0	0	0	0	1	0	0	1
ZKSCAN1	1	0	1	1	0	1	0	0	1
ZMAT1	0	1	0	0	1	0	0	1	0
ZMAT2	1	0	1	1	0	1	1	0	1
ZMAT4	0	1	0	0	1	0	0	1	0
ZMAT5	1	0	1	1	0	1	1	0	1
ZMPSTE24	1	0	1	1	0	1	1	0	1
ZMYM1	1	0	1	1	0	1	1	0	1
ZMYM3	0	1	1	0	1	0	0	0	0
ZMYM4	0	0	0	0	0	0	0	0	1
ZMYM5	0	0	0	0	1	0	0	0	0
ZMYM6	1	0	1	1	0	1	1	0	1
ZMYND10	1	0	1	1	0	1	1	0	1
ZMYND11	0	1	1	0	1	1	0	1	1
ZMYND12	1	0	1	1	0	1	1	0	1
ZMYND15	1	1	1	1	0	1	1	1	1
ZNF10	1	0	1	1	0	1	1	0	1
ZNF101	0	0	0	0	0	1	1	0	1
ZNF12	0	0	1	0	0	1	0	1	1
ZNF121	1	0	0	1	0	1	0	0	1
ZNF132	0	0	1	0	1	1	0	0	1
ZNF133	0	1	1	0	1	1	0	1	1
ZNF134	0	0	1	1	0	1	0	0	1
ZNF135	0	0	1	0	0	1	0	0	1
ZNF138	0	0	0	0	0	1	1	0	1
ZNF140	1	0	1	1	0	1	1	0	1
ZNF141	1	0	1	1	0	1	1	1	1
ZNF142	1	0	1	1	0	1	0	0	1
ZNF143	1	0	1	1	0	1	1	0	1
ZNF148	1	0	1	1	0	1	1	0	1
ZNF154	0	1	0	0	1	1	0	1	1
ZNF155	0	0	1	1	0	1	0	0	1
ZNF157	0	1	0	0	0	0	0	1	0
ZNF160	1	0	1	1	0	1	1	0	1
ZNF17	1	0	1	1	0	1	1	0	1
ZNF174	1	0	1	1	0	1	1	0	1
ZNF175	1	1	1	1	0	1	1	1	1
ZNF177	0	0	0	0	0	0	0	1	0

ZNF18	1	0	1	1	0	1	1	0	1
ZNF180	1	1	1	0	1	1	0	1	1
ZNF181	1	0	1	1	0	1	1	0	1
ZNF184	0	0	1	1	0	1	1	0	1
ZNF185	0	1	0	0	0	0	0	0	0
ZNF187	1	0	1	1	0	1	1	0	1
ZNF192	1	0	1	1	0	1	1	0	1
ZNF193	1	1	1	1	1	1	1	1	1
ZNF195	0	0	1	0	0	0	0	0	0
ZNF197	0	0	1	0	0	0	0	0	1
ZNF2	1	0	1	1	0	1	1	0	1
ZNF20	0	0	1	1	0	1	1	0	1
ZNF202	1	0	1	1	0	1	1	0	1
ZNF205	0	0	1	1	1	1	1	1	1
ZNF207	1	0	1	1	0	1	1	0	1
ZNF211	0	0	0	0	0	1	0	0	1
ZNF212	0	0	1	1	0	1	0	0	1
ZNF213	1	0	1	1	0	1	0	0	1
ZNF214	1	1	1	0	1	1	1	1	1
ZNF217	1	0	1	1	0	0	1	0	0
ZNF219	1	0	1	0	0	1	0	0	0
ZNF22	1	0	0	1	0	1	1	0	1
ZNF222	1	0	1	1	0	1	1	0	1
ZNF223	1	0	1	1	0	1	1	1	1
ZNF224	1	0	1	1	0	1	1	0	1
ZNF226	1	0	1	1	0	1	1	0	1
ZNF227	1	0	1	1	0	1	1	0	1
ZNF228	0	0	1	1	0	1	0	0	1
ZNF23	1	1	1	1	0	1	0	0	1
ZNF230	1	0	1	1	0	1	1	0	1
ZNF232	0	1	0	0	0	0	0	1	0
ZNF234	1	0	1	1	0	1	1	0	1
ZNF235	0	0	1	0	0	1	0	0	1
ZNF236	0	0	0	1	0	0	1	0	1
ZNF238	1	0	0	1	0	1	1	0	1
ZNF24	1	0	1	1	0	1	1	0	1
ZNF248	1	0	1	1	0	1	1	0	1
ZNF25	0	0	1	0	0	0	0	0	1
ZNF250	1	1	1	1	1	1	1	1	1
ZNF256	0	0	1	0	0	0	0	0	1
ZNF259	1	0	1	1	0	1	1	0	1
ZNF26	0	0	0	0	0	0	0	0	1
ZNF263	0	0	0	1	0	1	0	0	1
ZNF264	0	0	1	1	0	1	0	0	1
ZNF266	1	0	1	1	0	1	1	0	1
ZNF268	1	0	1	1	0	1	0	0	1
ZNF271	1	0	1	1	0	1	1	0	1
ZNF274	0	0	0	0	0	1	0	0	1
ZNF276	0	0	0	0	0	1	0	0	1
ZNF28	1	0	1	1	0	1	1	0	1
ZNF281	1	0	0	1	0	1	1	0	1
ZNF282	0	0	1	1	0	1	0	0	0
ZNF283	0	1	0	0	1	1	0	1	0
ZNF289	1	0	1	1	0	1	1	0	1
ZNF294	1	0	0	1	0	1	1	0	1
ZNF295	0	1	1	1	1	0	0	1	0
ZNF3	1	0	0	1	0	1	1	0	1
ZNF30	0	1	0	0	0	1	0	0	1
ZNF304	1	0	1	1	0	1	1	0	1
ZNF311	0	0	1	0	0	1	0	0	1
ZNF317	1	0	1	1	0	1	1	0	1
ZNF318	0	0	0	0	0	1	0	0	0

ZNF319	1	0	1	0	0	1	1	0	1
ZNF32	0	1	1	0	1	1	1	0	1
ZNF322B	0	1	0	0	0	0	0	1	0
ZNF323	1	0	1	1	0	1	1	0	1
ZNF324	1	1	1	1	0	1	0	1	1
ZNF326	1	0	1	1	0	1	1	0	1
ZNF329	0	0	1	0	0	1	0	0	0
ZNF330	0	0	0	1	0	0	0	0	1
ZNF331	0	0	1	0	0	1	0	0	1
ZNF333	1	0	1	1	0	1	1	0	1
ZNF334	1	1	1	0	1	1	1	1	1
ZNF335	1	0	1	1	0	1	1	0	1
ZNF337	1	0	1	0	0	1	1	0	1
ZNF33A	1	0	1	1	0	1	1	0	1
ZNF341	1	0	1	0	0	1	1	0	1
ZNF342	1	0	1	1	0	1	1	0	1
ZNF343	1	0	1	1	0	1	1	0	1
ZNF345	1	0	1	1	0	1	1	0	1
ZNF346	1	0	1	1	0	1	1	0	1
ZNF347	0	1	1	1	0	1	1	1	1
ZNF35	0	0	0	0	0	1	0	0	1
ZNF350	1	0	1	1	0	1	1	0	1
ZNF358	0	1	0	0	1	0	0	1	0
ZNF364	1	1	1	1	1	1	1	1	1
ZNF365	0	1	1	0	1	1	1	1	1
ZNF366	1	0	1	1	0	1	1	0	1
ZNF367	0	0	0	1	0	0	0	0	0
ZNF37A	1	0	1	1	0	1	1	0	1
ZNF384	1	0	1	1	0	1	1	0	1
ZNF385	0	0	0	0	0	0	0	0	1
ZNF394	1	0	0	0	0	1	0	0	1
ZNF395	1	0	1	1	0	1	1	0	1
ZNF397	0	0	0	1	0	1	1	0	1
ZNF398	1	0	1	1	0	1	1	0	1
ZNF403	1	0	1	1	0	1	1	0	1
ZNF407	0	0	0	1	0	0	1	0	1
ZNF408	1	0	1	1	0	1	1	0	1
ZNF41	1	1	1	1	1	1	1	1	1
ZNF410	1	0	1	1	0	1	1	0	1
ZNF417	1	0	1	1	0	1	1	0	1
ZNF419	0	0	1	0	0	1	0	0	1
ZNF420	1	0	1	1	0	1	1	0	1
ZNF425	1	0	1	1	0	1	1	0	1
ZNF426	1	0	1	1	0	1	1	0	1
ZNF429	0	0	0	0	0	1	0	0	1
ZNF43	1	0	1	1	0	1	1	0	1
ZNF430	1	0	1	1	0	1	1	0	1
ZNF431	1	0	1	1	0	1	1	0	1
ZNF432	1	0	1	1	0	1	1	0	1
ZNF434	1	0	1	1	0	1	1	0	1
ZNF436	1	0	1	1	0	1	1	0	1
ZNF442	0	0	0	0	0	1	0	0	1
ZNF444	1	0	1	1	0	1	0	0	1
ZNF446	1	0	1	1	0	1	0	0	1
ZNF451	1	0	1	1	0	1	1	0	1
ZNF452	0	1	1	0	1	0	0	1	0
ZNF467	0	0	0	0	0	0	0	0	1
ZNF468	1	0	1	0	0	0	0	0	1
ZNF471	0	0	1	0	0	1	0	0	1
ZNF473	1	0	1	1	0	1	1	0	1
ZNF480	1	0	1	1	0	1	1	0	1
ZNF484	1	0	0	1	0	1	1	0	1

ZNF485	0	0	0	0	0	0	0	0	1
ZNF488	0	1	1	0	1	1	0	1	1
ZNF496	1	0	1	0	0	1	0	1	0
ZNF497	0	0	0	0	0	0	0	0	1
ZNF498	0	1	0	1	0	0	0	1	0
ZNF500	0	0	0	0	0	1	0	0	1
ZNF503	0	1	1	0	0	1	0	1	1
ZNF509	1	0	1	1	0	1	1	0	1
ZNF510	1	0	1	1	0	1	1	0	1
ZNF511	1	0	0	1	0	1	0	0	1
ZNF512	1	0	1	1	0	1	1	0	1
ZNF513	0	0	0	0	0	1	0	0	0
ZNF518	0	1	1	0	1	1	0	1	1
ZNF524	1	0	1	0	0	1	0	0	1
ZNF526	1	0	1	1	0	1	0	0	1
ZNF529	1	0	1	1	0	1	1	0	1
ZNF530	0	0	1	0	0	0	0	0	0
ZNF532	0	1	0	0	0	1	0	1	1
ZNF536	0	1	0	0	0	0	0	1	0
ZNF540	0	1	1	1	0	1	1	1	1
ZNF541	0	1	0	0	1	0	0	1	0
ZNF543	1	1	1	1	0	1	1	1	1
ZNF544	0	0	1	0	0	1	0	0	1
ZNF545	0	1	1	0	1	1	0	1	1
ZNF548	1	0	0	1	0	1	1	0	1
ZNF549	0	0	0	0	0	1	0	1	1
ZNF550	1	0	1	1	0	1	0	0	1
ZNF551	0	0	1	1	0	1	0	0	1
ZNF557	1	0	0	1	0	1	1	0	1
ZNF559	0	0	0	0	0	0	0	0	1
ZNF561	0	0	0	1	0	1	1	0	1
ZNF562	0	0	0	1	0	1	0	0	1
ZNF564	1	0	1	1	0	1	1	0	1
ZNF565	1	0	1	1	0	1	1	0	1
ZNF567	1	0	1	1	0	1	0	0	1
ZNF569	0	0	1	0	0	1	0	0	1
ZNF570	0	0	1	0	0	1	0	0	1
ZNF573	1	0	1	1	0	1	1	0	1
ZNF575	0	0	1	0	0	1	1	0	1
ZNF576	1	0	1	1	0	1	1	0	1
ZNF580	0	0	1	1	0	1	1	1	1
ZNF581	1	0	1	1	0	1	1	1	1
ZNF582	1	0	1	1	0	1	0	0	1
ZNF583	1	0	1	1	0	1	0	0	1
ZNF585A	1	0	1	1	0	1	1	0	1
ZNF585B	1	0	1	1	0	1	1	0	1
ZNF587	0	0	0	1	0	1	1	0	1
ZNF592	1	0	1	1	0	1	1	0	1
ZNF593	0	1	0	0	1	0	0	1	0
ZNF595	0	0	0	0	0	0	0	0	1
ZNF597	1	1	1	1	0	1	0	0	1
ZNF606	0	0	1	0	0	1	0	0	1
ZNF609	0	0	0	1	0	0	0	0	0
ZNF613	1	0	1	1	0	1	1	0	1
ZNF614	1	0	1	1	0	1	1	0	1
ZNF615	1	0	1	1	0	1	1	0	1
ZNF616	1	0	1	1	0	1	1	0	1
ZNF618	0	1	0	0	1	0	0	1	0
ZNF619	1	0	1	0	0	1	1	0	1
ZNF620	0	0	1	1	0	1	1	0	1
ZNF621	0	0	0	1	0	1	1	0	1
ZNF622	1	0	1	1	0	1	1	0	1

ZNF624	0	1	0	0	0	1	0	1	1
ZNF626	0	0	1	0	0	1	0	0	0
ZNF628	0	0	0	0	0	0	0	0	1
ZNF630	0	1	1	0	1	1	0	0	1
ZNF642	0	0	1	1	0	1	1	0	1
ZNF643	1	0	1	1	0	1	0	0	1
ZNF644	1	0	1	1	0	1	1	0	1
ZNF646	1	0	1	1	0	1	1	0	1
ZNF649	1	0	1	1	0	1	1	0	1
ZNF653	0	0	0	0	0	1	0	0	1
ZNF655	1	0	1	1	0	1	1	0	1
ZNF658	1	0	1	1	0	1	1	0	1
ZNF663	0	1	0	0	1	1	0	1	0
ZNF664	1	0	1	1	0	1	1	1	1
ZNF668	1	0	1	1	0	1	1	0	1
ZNF669	0	0	0	0	0	1	0	0	0
ZNF671	1	0	1	0	0	1	1	0	1
ZNF672	1	0	1	1	0	1	1	0	1
ZNF673	0	1	1	1	1	1	0	1	1
ZNF675	1	0	1	1	0	1	1	0	1
ZNF680	1	0	1	1	0	1	1	0	1
ZNF683	0	0	0	0	0	0	0	1	0
ZNF684	1	0	1	1	0	1	1	0	1
ZNF687	0	0	0	0	0	0	0	0	1
ZNF688	0	0	1	0	0	1	1	0	1
ZNF689	0	0	0	0	0	1	0	0	1
ZNF69	1	0	1	0	0	1	1	0	1
ZNF691	1	0	1	1	0	1	1	0	1
ZNF692	1	0	1	1	0	1	1	0	1
ZNF696	0	0	1	0	0	1	0	0	1
ZNF7	0	1	0	1	0	0	0	1	0
ZNF700	0	0	1	0	0	1	0	0	1
ZNF701	1	0	1	0	0	1	0	0	1
ZNF702	1	0	1	0	0	1	1	0	1
ZNF706	1	0	1	1	1	1	1	1	1
ZNF707	1	0	1	1	0	1	1	0	1
ZNF708	1	0	1	0	0	1	1	0	1
ZNF71	0	0	0	0	0	0	0	0	1
ZNF710	0	0	0	0	0	1	1	0	1
ZNF714	1	0	0	0	0	0	1	0	1
ZNF720	0	0	0	0	0	0	0	0	1
ZNF721	1	0	1	1	0	1	0	0	1
ZNF740	1	0	1	1	0	1	1	0	1
ZNF746	0	0	0	0	0	1	0	0	0
ZNF747	1	0	1	1	0	1	1	0	1
ZNF75	1	1	1	1	0	1	0	0	1
ZNF75A	0	0	1	0	0	1	0	0	1
ZNF76	1	0	0	0	0	1	0	0	0
ZNF79	1	0	1	1	0	1	1	0	1
ZNF8	1	0	1	1	0	1	0	0	1
ZNF83	1	0	1	1	0	1	1	0	1
ZNF84	0	0	1	0	0	1	0	0	1
ZNF85	1	0	1	0	0	1	1	0	1
ZNF91	1	0	1	0	0	1	1	0	1
ZNF92	1	0	1	0	0	1	1	0	1
ZNF93	0	0	1	1	0	1	1	0	1
ZNFX1	1	0	1	1	0	1	1	0	1
ZNHIT1	1	0	1	1	0	1	1	0	1
ZNHIT2	1	0	1	1	0	1	1	0	1
ZNHIT3	0	0	1	0	0	0	0	0	1
ZNHIT4	1	0	0	1	0	1	0	0	1
ZNRD1	1	0	1	1	0	1	1	0	1

ZRANB3	1	0	1	1	0	1	1	0	1
ZSCAN2	0	0	1	1	0	1	1	0	1
ZSCAN5	0	0	1	0	0	0	0	0	1
ZSWIM1	1	0	1	1	0	1	1	0	1
ZSWIM4	0	0	0	1	0	1	0	0	1
ZW10	1	1	1	1	0	1	1	0	1
ZWILCH	1	0	1	1	0	1	1	0	1
ZWINT	1	0	1	1	0	1	1	0	1
ZXDB	0	1	1	0	1	1	1	0	0
ZXDC	1	0	1	0	0	1	0	0	1
ZYG11B	0	0	0	0	0	0	1	0	1
ZYX	1	0	1	1	0	1	1	0	1
ZZEF1	1	0	1	1	0	1	1	0	1
ZZZ3	1	0	1	1	0	1	1	0	1
BA16L21.2.1	1	0	1	0	0	1	0	0	1
ISG15	0	1	1	0	1	1	0	1	1
MGC40168	1	0	0	1	0	0	1	0	1
IIP45	1	0	0	1	0	1	1	0	1
RCN3	0	1	0	0	1	0	0	1	0
EIF3I	1	1	1	0	1	1	0	1	1
AK3	1	0	0	0	0	0	0	0	0
ZRANB2	0	0	0	0	0	0	1	0	1
LOC149620	0	0	0	0	0	1	0	0	1
SEC22B	1	0	1	1	0	1	1	0	1
VPS45	1	0	0	0	0	0	0	0	1
TRK1	1	0	1	1	0	1	1	0	1
FCRLA	1	0	1	1	0	1	1	0	1
ASTN1	1	0	1	1	0	1	1	0	1
QSOX1	1	0	1	1	0	1	1	0	1
SMC6	0	1	0	0	1	0	0	1	1
HADH	0	1	0	0	0	0	0	1	0
FOXN2	1	0	0	0	0	1	1	0	1
CIAO1	1	0	1	1	0	1	1	0	1
REV1	1	0	0	1	0	1	1	0	1
ZEB2	1	0	1	1	0	1	1	0	1
SPC25	0	1	0	0	1	0	0	1	0
DPH3	0	0	0	1	0	1	1	0	1
SEC22C	1	0	1	1	0	1	1	0	1
SIT1	0	1	1	0	1	0	0	1	0
CAST	1	0	0	0	0	0	0	0	1
APPL1	1	0	1	1	0	1	0	0	1
CAPS	0	1	0	0	1	0	0	1	1
SEC22A	1	0	1	1	0	1	1	0	1
CNBP	1	0	0	0	0	1	1	0	1
HLTF	0	1	0	0	1	0	0	1	0
B3GALNT1	1	0	1	1	0	1	1	0	1
FLJ90036	1	0	1	1	0	1	1	0	1
COX18	0	1	1	0	1	1	0	1	1
SEC31A	0	1	0	0	0	0	0	0	0
PIN1	1	0	1	1	0	1	0	0	0
NAIP	0	1	0	0	1	0	0	1	0
GPR98	1	0	1	1	0	1	1	0	1
HSPA9	0	0	1	1	0	1	1	0	1
CNR2	0	0	0	0	0	1	1	0	1
ZSCAN16	1	0	1	1	0	1	1	0	1
FLJ35429	1	0	1	1	0	1	1	0	1
MED20	1	0	1	1	0	1	1	0	1
VEGFA	1	0	1	1	0	1	0	0	1
LOC441151	1	0	0	1	0	1	0	0	0
MCM9	1	0	1	1	0	1	1	0	1
EIF3B	1	0	1	1	0	1	1	0	1
NOD1	1	0	0	1	0	0	1	0	1

BBS9	1	0	1	1	0	1	1	0	1
EIF4H	1	0	1	0	1	1	1	1	1
CLIP2	1	1	1	1	1	1	0	1	1
ZSCAN21	0	1	0	0	0	0	0	1	0
AGK	1	0	1	0	0	1	0	0	1
DEF6	1	0	1	1	0	1	1	0	1
NEFM	0	0	1	0	0	1	1	0	1
EIF3E	1	1	1	0	1	1	0	0	0
MED30	0	0	1	0	0	1	0	0	1
LOC441459	0	1	0	0	0	0	0	0	0
RMI1	0	1	0	0	0	0	0	0	0
BMI1	1	1	1	0	1	1	0	1	0
ZCD1	1	0	1	1	0	1	1	0	1
DYDC2	1	0	1	1	0	1	0	0	1
LCOR	1	0	0	0	0	0	0	0	0
SMC3	0	1	1	1	0	1	0	0	1
EIF3A	0	0	0	0	0	1	0	0	1
PSTK	0	0	0	1	0	0	0	0	0
CTR9	1	0	1	1	0	1	1	0	1
DPH4	0	0	0	0	0	0	0	0	1
CLP1	0	1	1	0	1	0	0	1	1
RSF1	1	0	1	1	0	1	0	0	1
MGC33948	1	0	1	1	0	1	1	0	1
ERC1	1	0	1	1	0	1	1	0	1
MED21	1	0	0	0	0	0	1	0	0
LOC144983	1	0	1	1	0	1	1	0	1
MGC13168	1	0	1	1	0	1	1	0	1
APPL2	0	1	0	0	1	1	0	1	0
ISCU	1	0	1	1	0	1	1	0	1
ANKRD13A	0	1	1	0	1	1	0	1	1
C12ORF8	0	1	0	0	1	1	0	1	1
MED13L	1	0	1	1	0	1	1	0	1
DNCL1	0	0	0	0	0	1	0	0	1
SPPL3	0	1	0	0	0	0	0	0	0
CLIP1	0	1	0	0	1	0	0	1	0
ZMYM2	1	0	1	1	0	1	1	0	1
PDX1	0	1	0	0	1	0	0	1	0
LOC387921	0	1	0	0	1	0	0	1	0
FOXO1	1	0	1	1	0	1	1	1	1
KIAA0564	1	0	1	1	0	1	1	0	1
KIAA0564	1	0	1	1	0	1	1	0	1
LOC220416	1	0	1	1	0	1	1	0	1
TRIM13	1	0	0	1	0	0	0	0	0
LOC440145	0	1	0	0	0	1	0	0	0
DIS3	0	0	0	0	0	0	0	0	1
RBM26	1	0	1	1	0	1	0	0	1
HOMEZ	1	0	1	1	0	1	1	0	1
EAPP	1	0	1	1	0	1	0	0	1
ODC1	1	0	1	1	0	1	1	0	1
TXNDC1	0	0	0	0	0	0	0	1	0
DNAL1	1	0	0	1	0	1	0	0	0
FOXN3	0	1	0	0	1	0	0	1	0
SERPINA11	1	0	1	1	0	1	1	0	1
KLC1	1	1	1	1	0	1	1	0	1
ZSCAN29	1	0	1	1	0	1	1	0	1
EIF3J	0	1	0	0	1	0	0	0	0
TRIM69	1	0	1	1	0	1	1	0	1
EID1	0	0	0	0	0	0	0	1	0
EDC3	0	1	0	0	1	0	0	1	0
ZFAND6	0	1	0	0	1	0	0	1	0
KIAA0430	1	0	1	1	0	1	1	0	1
MGC3020	1	0	1	1	0	1	1	0	1

NOD2	1	0	1	1	0	1	1	0	1
CTF8	0	0	1	1	0	1	1	0	1
NOB1	0	1	0	0	1	0	0	1	0
CHMP1A	0	1	0	0	1	0	0	1	0
XAF1	1	0	0	0	0	0	0	0	0
GPR158L1	0	0	1	1	0	1	1	0	1
NACA2	1	0	1	1	0	1	1	0	1
BPTF	1	0	1	1	0	1	0	0	1
EIF4A3	1	0	1	1	0	1	1	0	1
KIAA1303	1	0	1	1	0	1	1	0	1
ELP2	0	1	0	0	1	0	0	0	0
EIF3G	1	0	0	0	0	0	0	0	1
ECSIT	0	0	0	1	0	1	0	0	1
MED26	0	1	0	0	1	0	0	1	0
MAP1S	0	1	1	0	1	0	0	1	0
NCAN	1	0	1	1	0	1	1	0	1
SAE2	1	0	1	1	0	1	1	0	1
TBCB	0	0	1	1	0	1	0	0	1
EIF3K	0	0	0	0	0	0	0	1	1
MED29	1	0	1	1	0	1	1	0	1
FIZ1	1	0	1	1	0	1	1	0	1
ZSCAN22	0	0	0	0	0	0	0	0	1
MZF1	0	0	0	0	0	0	0	0	1
RBCK1	1	0	1	1	0	1	1	0	1
FAM110A	1	0	1	1	0	1	1	0	1
KIAA1271	1	0	1	1	0	1	1	0	1
GZF1	1	0	0	0	0	1	0	0	1
KIAA0980	0	0	0	0	0	0	0	0	1
KIAA1755	1	0	0	0	0	1	1	0	1
SNX21	1	0	0	1	0	1	0	0	1
CTSA	0	0	0	0	0	0	1	0	0
ZMYND8	1	0	1	1	0	1	1	0	1
N6AMT1	1	0	1	0	0	1	1	0	1
IL17RA	1	0	1	1	0	1	1	0	1
LOC402055	0	1	1	0	1	1	0	1	1
HSCB	1	0	1	1	0	1	1	0	1
GEMIN8	1	1	1	1	1	1	1	1	1
KSR2	1	0	1	1	0	1	1	0	1
LOC401589	0	0	0	0	0	1	1	0	1
JARID1C	1	0	1	1	0	1	1	0	1
HSD17B10	1	0	1	1	0	1	1	0	1
FAM104B	0	0	1	1	0	1	0	0	1
FOXO4	0	0	1	0	0	1	0	0	1
LOC340527	1	0	1	1	0	1	1	0	1
DKFZP564K142	1	0	1	1	0	1	1	0	1
TAF9L	0	0	1	0	0	1	0	0	1
CENPI	1	0	1	1	0	1	1	0	1
ALG13	1	0	1	1	0	1	1	0	1
