# Supplementary material for: Genome-wide promoter analysis of histone modifications in human monocyte-derived antigen presenting cells
Source: BMC Genomics. 2010 Nov 18;11:642. doi: 10.1186/1471-2164-11-642 (PMC3091769; doi:10.1186/1471-2164-11-642)
Supplement: Additional file 2 — Tserel et al BMC Genomics. Contains Supplementary Tables S2-S6. Size 7.2 MB [file 1471-2164-11-642-S2.ZIP › Supplementary Table 6. Monocyte expression..pdf]

Supplementary Table 6. Histone modifications (normalized to H3 and to monocyte modfication values) in monocyte, macrophage and dendritic cell subpopulations (1=peak, 0=no peak)

	DC AcH3	DC H3K27	DC H3K4	MF AcH3	MF H3K27	MF H3K4
15E1.2	1	0	0	1	0	0
A2M	1	0	1	1	0	1
A4GNT	0	0	1	1	0	1
AADACL1	1	0	0	1	1	1
AAMP	1	0	0	0	0	0
AARSD1	0	1	0	1	1	0
AASDH	1	0	0	1	0	0
AASDHPPT	0	0	0	1	0	0
ABCA12	1	1	1	0	1	1
ABCA3	0	0	0	1	0	1
ABCA5	1	0	1	0	0	1
ABCA9	0	0	1	0	0	1
ABCB4	0	0	1	0	0	1
ABCB6	1	0	0	0	0	0
ABCB7	0	1	0	1	0	0
ABCB9	1	0	0	1	0	1
ABCC10	0	0	0	0	1	1
ABCC11	1	1	1	1	0	1
ABCC13	0	1	1	0	0	1
ABCC2	0	0	1	0	0	1
ABCE1	1	0	1	1	1	1
ABCF1	1	0	0	1	0	0
ABCF2	1	0	0	0	1	0
ABCF3	1	0	0	1	0	0
ABCG1	1	0	1	1	0	1
ABCG2	1	0	0	0	0	0
ABHD10	0	1	0	0	0	0
ABHD11	1	0	0	0	0	0
ABHD12	0	0	0	1	0	0
ABHD13	1	0	0	0	0	0
ABHD14A	1	0	0	0	0	0
ABHD3	1	0	0	1	0	0
ABHD5	1	0	0	0	0	0
ABHD6	1	0	0	1	0	0
ABHD8	1	0	0	0	0	0
ABI1	1	0	0	0	0	0
ABI3	1	0	0	0	0	0
ABL1	1	0	0	1	0	0
ABL2	1	0	1	1	0	1
ABR	0	0	0	0	0	1
ABRA	1	0	1	0	0	1
ACAA1	1	0	0	0	0	0
ACACA	1	0	1	1	0	1
ACACB	0	0	1	0	0	1
ACAD10	1	0	0	1	1	0
ACAD11	1	0	0	1	0	0
ACAD8	1	0	0	1	1	0
ACAD9	1	0	0	0	0	1
ACADM	0	0	1	0	0	0
ACADS	1	1	0	0	1	0
ACADSB	1	0	0	1	0	1
ACADVL	1	0	0	0	1	0
ACAT1	1	0	0	1	1	0
ACAT2	0	0	0	0	1	0
ACBD3	1	0	0	0	0	0

ACBD5	0	0	0	0	1	0
ACBD6	0	0	0	1	1	0
ACCN3	0	0	1	1	0	1
ACCN5	0	1	1	0	0	1
ACE	1	1	1	0	0	1
ACIN1	0	0	1	1	0	1
ACO1	1	0	0	0	0	0
ACO2	1	0	0	1	0	0
ACOT1	0	0	0	1	0	0
ACOT11	0	1	1	1	1	1
ACOT2	1	1	1	1	0	1
ACOT7	0	0	1	0	0	1
ACOT9	0	0	1	1	0	0
ACOX1	1	0	0	1	0	0
ACOX2	0	0	1	0	0	0
ACOX3	1	0	0	1	0	0
ACP1	1	0	0	1	0	0
ACP2	0	0	0	0	1	0
ACP5	0	0	1	1	0	1
ACPL2	0	0	0	0	1	0
ACPP	1	0	0	1	0	0
ACPT	0	0	0	0	0	1
ACR	0	0	1	0	0	1
ACRC	0	1	1	0	0	1
ACRV1	0	0	1	0	0	1
ACSBG2	0	1	1	0	0	1
ACSL3	1	0	0	1	0	0
ACSL4	1	1	0	1	0	0
ACSL5	1	0	1	1	0	1
ACSS2	1	1	0	1	0	1
ACTA2	1	1	1	1	1	1
ACTB	0	0	0	1	1	0
ACTL6A	1	1	0	0	0	0
ACTL6B	0	0	1	0	0	1
ACTL7A	0	1	1	0	1	1
ACTL8	0	0	1	0	0	1
ACTN4	1	0	0	0	0	0
ACTR10	0	0	0	1	0	0
ACTR1A	1	0	0	1	0	0
ACTR2	1	0	0	1	0	0
ACTR3	1	0	0	1	1	1
ACTR5	0	0	0	1	0	0
ACTR8	1	0	0	1	1	0
ACTRT1	0	1	1	0	0	0
ACVR1	1	0	1	0	0	0
ACVR1B	0	0	0	1	0	0
ACVR2A	1	0	0	1	0	0
ACVRL1	0	0	0	0	0	1
ACY1	1	0	0	0	0	0
ACY1L2	0	1	0	0	0	0
ACY3	0	1	0	0	0	0
ACYP1	1	0	1	1	0	1
ACYP2	1	1	1	0	0	1
ADA	0	1	0	0	0	0
ADAL	1	0	0	1	0	0
ADAM12	0	0	1	0	0	0
ADAM15	1	0	0	1	0	0
ADAM17	1	0	0	1	0	0
ADAM18	0	0	0	0	0	1

ADAM21	0	1	1	0	0	1
ADAM30	1	1	1	0	0	1
ADAM7	0	1	1	0	0	1
ADAM8	0	0	0	0	1	0
ADAM9	1	0	0	1	0	0
ADAMDEC1	0	0	0	0	0	1
ADAMTS13	0	0	1	1	0	0
ADAMTS17	1	0	0	1	0	0
ADAMTSL4	1	0	0	0	0	0
ADAR	1	0	0	1	0	0
ADAT1	1	0	0	0	0	0
ADCK4	1	0	0	0	0	0
ADCY7	1	0	0	0	1	0
ADCY9	0	0	0	0	0	1
ADHFE1	1	0	0	1	1	0
ADIPOR1	1	0	0	1	0	0
ADIPOR2	1	0	0	0	0	0
ADK	1	0	0	1	0	0
ADM2	0	0	0	0	0	1
ADMR	1	1	1	0	0	1
ADNP	0	0	0	1	0	0
ADORA3	1	0	0	0	0	0
ADPGK	0	0	0	0	1	1
ADPRH	1	0	1	1	0	1
ADRBK2	1	0	0	1	0	0
ADRM1	1	0	0	0	0	0
ADSS	0	0	1	1	0	0
ADSSL1	0	0	1	0	0	1
AFG3L1	1	0	0	1	0	0
AFMID	1	0	0	1	0	0
AGBL4	0	0	1	0	0	1
AGER	1	0	0	1	0	0
AGGF1	0	0	0	1	0	0
AGL	1	0	1	1	0	0
AGPAT1	1	0	0	1	0	0
AGPAT3	0	0	1	0	0	1
AGPAT5	1	0	0	1	0	0
AGPAT7	1	0	0	1	0	0
AGPS	1	0	0	1	0	1
AGRP	0	1	0	0	0	1
AGT	0	1	1	0	0	1
AGTR2	0	0	1	0	0	1
AHCYL1	0	0	1	1	0	1
AHDC1	1	0	0	1	0	0
AHI1	0	0	0	1	0	0
AHNAK	1	0	0	0	0	0
AHR	1	0	0	0	1	0
AHSA1	1	0	0	1	0	0
AHSG	0	0	1	0	0	1
AICDA	0	0	1	0	0	1
AIM2	0	1	0	0	1	0
AIPL1	0	0	1	0	0	1
AK2	1	0	0	1	0	0
AKAP1	1	0	1	1	0	1
AKAP12	0	0	0	0	0	1
AKAP13	0	0	1	1	0	0
AKAP14	0	1	1	0	0	1
AKAP5	1	0	1	0	0	0
AKAP7	0	1	1	0	0	1

AKAP8	1	0	0	1	0	0
AKAP8L	1	0	0	1	0	0
AKR1C3	0	0	0	1	0	0
AKR7A2	1	0	0	0	0	0
AKT1S1	1	0	0	1	0	0
AKT2	1	0	0	1	0	0
AKT3	0	0	0	0	0	1
ALAS1	0	0	0	0	1	0
ALB	0	1	0	0	0	0
ALCAM	1	0	0	1	0	0
ALDH16A1	1	0	0	0	1	0
ALDH1A1	0	0	0	1	0	0
ALDH1A2	1	0	1	1	0	1
ALDH3B1	1	0	0	1	0	0
ALDH3B2	0	1	1	0	0	0
ALDH4A1	1	0	0	0	0	0
ALDH5A1	1	0	0	1	0	1
ALDH6A1	1	0	0	1	0	0
ALDOB	0	1	1	1	0	1
ALDOC	1	0	0	1	1	0
ALG1	0	0	0	0	0	1
ALG11	1	0	1	1	0	1
ALG12	1	0	0	1	0	0
ALG14	1	0	0	1	0	0
ALG2	1	0	0	1	0	0
ALG3	1	0	0	1	0	0
ALG8	1	0	0	1	0	0
ALG9	1	0	0	1	0	0
ALKBH1	1	0	0	1	1	1
ALKBH2	1	0	0	1	0	1
ALKBH3	0	0	0	1	0	0
ALKBH4	1	0	0	0	0	0
ALKBH8	0	0	0	1	1	0
ALMS1	1	0	0	1	0	0
ALOX12B	0	0	1	0	0	1
ALOX15B	0	1	0	0	0	0
ALOX5AP	1	0	0	1	0	1
ALPI	0	0	1	0	0	1
ALPK2	0	0	1	0	0	1
ALPP	0	1	1	0	0	1
ALS2CL	0	1	1	0	0	1
ALS2CR12	0	0	1	0	0	1
ALS2CR14	1	0	0	1	0	0
ALS2CR2	0	0	0	1	0	0
ALS2CR4	1	0	1	1	0	1
ALS2CR8	1	0	0	1	0	0
AMACR	1	1	0	1	1	0
AMBN	0	0	1	0	0	1
AMDHD2	1	0	0	0	0	0
AMHR2	0	0	1	0	0	0
AMICA1	1	0	0	0	1	0
AMOTL1	0	1	0	0	0	0
AMPD2	1	0	0	1	1	0
AMPD3	0	1	0	0	0	0
AMY1B	0	0	1	0	1	1
AMY1C	1	0	1	1	1	1
AMY2A	0	0	1	0	0	1
AMY2B	0	0	1	0	0	0
AMZ2	1	0	0	1	0	0

ANAPC10	1	0	1	1	1	1
ANAPC11	1	0	0	1	0	0
ANAPC13	0	1	0	0	1	0
ANAPC2	1	0	0	1	0	0
ANAPC5	0	0	0	1	0	0
ANAPC7	1	0	0	1	0	0
ANG	1	1	0	1	1	0
ANGEL1	1	0	0	0	0	0
ANGEL2	0	0	0	1	0	0
ANGPT1	0	1	0	0	0	0
ANGPTL3	0	0	1	0	0	0
ANGPTL6	1	0	1	0	0	1
ANK1	0	1	1	1	1	0
ANK2	1	0	1	0	0	1
ANKDD1A	0	0	1	0	0	1
ANKFY1	0	0	0	0	1	0
ANKHD1	1	0	0	0	0	0
ANKMY1	0	0	0	0	0	1
ANKMY2	1	0	0	1	0	0
ANKRA2	1	0	0	1	0	1
ANKRD10	1	0	0	1	0	1
ANKRD12	1	0	0	0	0	0
ANKRD13C	1	0	0	1	0	1
ANKRD16	1	1	1	1	1	0
ANKRD17	1	0	0	1	0	1
ANKRD22	0	0	1	1	0	1
ANKRD38	0	0	0	1	0	0
ANKRD40	0	0	0	0	1	0
ANKRD41	1	0	0	1	1	0
ANKRD44	0	0	0	0	1	0
ANKRD46	0	0	0	1	0	0
ANKRD49	1	0	0	1	0	0
ANKRD7	0	0	1	0	0	1
ANKS1B	1	0	1	1	0	1
ANKS3	1	0	0	1	0	0
ANKZF1	1	0	0	0	0	0
ANLN	1	0	0	1	0	0
ANP32C	0	0	1	0	0	1
ANPEP	1	0	0	0	0	0
ANTXR1	0	0	0	1	0	0
ANUBL1	0	1	0	0	1	0
ANXA13	0	0	0	0	0	1
ANXA2	1	0	0	1	0	0
ANXA4	1	0	1	1	0	1
ANXA5	0	0	0	1	0	1
ANXA8	0	1	1	0	0	1
AOC3	0	0	1	0	0	1
AOF1	1	0	0	1	0	0
AP1G1	0	1	0	0	0	0
AP1GBP1	1	0	0	0	0	0
AP1M1	1	0	0	1	1	0
AP2A1	0	0	1	1	0	1
AP2M1	1	0	0	1	0	0
AP3M1	1	0	0	1	0	0
AP3M2	1	0	0	0	0	0
AP3S2	1	0	0	1	0	0
AP4E1	1	0	0	0	0	0
AP4M1	1	0	0	1	0	0
APAF1	1	0	0	0	1	0

APBA2	0	0	1	0	0	1
APBB1IP	0	0	0	0	1	0
APBB3	1	0	0	1	0	0
APCDD1	0	0	0	0	0	1
APEX1	1	0	0	1	1	0
APEX2	1	1	1	1	1	1
APH1A	1	0	0	0	0	0
API5	1	0	0	0	0	0
APIP	1	0	0	1	0	0
APITD1	0	0	1	1	0	0
APLP2	0	1	0	0	0	0
APOB48R	0	0	0	0	1	0
APOBEC3A	0	1	0	0	0	0
APOBEC3B	0	1	0	0	0	0
APOBEC3C	1	1	0	0	0	0
APOBEC3G	0	0	0	1	0	0
APOC1	0	0	0	0	0	1
APOC2	1	0	1	1	0	1
APOC4	0	0	1	0	0	1
APOD	0	0	0	0	0	1
APOE	1	0	0	1	0	1
APOL3	0	1	0	1	1	0
APOL4	0	0	1	0	0	1
APOM	1	0	0	1	0	0
APPBP1	0	1	1	1	0	1
AQP1	0	0	1	0	0	1
AQP10	0	0	1	0	0	1
AQP12A	0	1	0	0	0	0
AQP7	0	0	1	0	0	1
AQP9	1	0	0	1	0	1
AQR	1	0	0	1	0	0
ARAF	0	0	0	0	0	1
ARF1	1	0	0	1	0	0
ARF5	1	0	0	1	0	0
ARF6	1	0	0	0	0	0
ARFGAP3	1	0	1	1	0	1
ARFGEF1	0	0	0	1	0	0
ARFIP1	1	0	0	1	0	0
ARFIP2	1	0	1	1	0	1
ARFRP1	1	0	0	1	0	0
ARHGAP1	1	0	0	1	0	0
ARHGAP10	0	0	0	1	0	0
ARHGAP11A	1	1	0	0	0	0
ARHGAP18	1	0	1	1	0	0
ARHGAP19	1	0	0	1	0	0
ARHGAP22	0	0	0	1	0	0
ARHGAP24	0	1	1	1	0	1
ARHGAP25	1	0	0	1	1	0
ARHGAP5	0	0	0	1	0	0
ARHGAP6	0	1	1	0	1	1
ARHGAP9	1	0	0	1	0	0
ARHGDIA	1	0	0	0	0	0
ARHGDIB	1	0	1	0	0	1
ARHGEF1	1	0	0	0	0	0
ARHGEF10L	0	0	1	0	0	1
ARHGEF12	1	0	0	0	0	0
ARHGEF15	0	0	1	0	0	1
ARHGEF16	0	0	1	0	0	1
ARHGEF19	0	0	1	0	0	1

ARHGEF2	0	0	0	0	1	0
ARHGEF5	0	1	0	0	0	0
ARHGEF6	0	1	0	0	0	0
ARHGEF7	1	0	0	1	0	0
ARHGEF9	0	1	1	0	1	1
ARID1A	1	0	0	1	1	0
ARID3B	1	0	0	0	0	0
ARID4A	1	0	0	1	0	0
ARID5A	1	0	0	1	0	0
ARID5B	0	0	0	0	1	0
ARIH1	1	0	0	1	0	0
ARIH2	1	0	0	0	0	0
ARL1	0	0	0	1	0	0
ARL11	0	0	0	0	0	1
ARL13A	0	1	1	0	0	1
ARL14	0	0	1	1	0	0
ARL16	1	0	0	1	0	0
ARL17P1	1	0	1	1	0	1
ARL2BP	0	0	1	0	0	0
ARL3	1	0	0	1	1	0
ARL4A	1	0	0	1	0	0
ARL5A	0	1	1	0	1	0
ARL6IP4	1	0	0	1	0	0
ARL6IP5	0	0	0	1	0	0
ARL6IP6	1	0	0	0	0	0
ARMC1	1	0	0	1	0	0
ARMC5	1	0	0	1	0	0
ARMC6	0	0	0	1	0	1
ARMCX1	0	1	0	0	1	0
ARMCX2	0	0	1	1	0	0
ARMCX3	0	1	1	0	0	1
ARMCX5	0	1	0	0	0	0
ARMCX6	0	1	1	0	0	1
ARMET	1	0	0	1	1	0
ARMETL1	1	0	0	1	0	0
ARNTL	0	0	0	1	0	0
ARNTL2	0	0	0	1	0	0
ARPC1A	0	0	0	1	0	0
ARPC2	1	0	0	0	0	0
ARPC3	1	0	0	1	1	0
ARPC4	1	0	0	1	0	0
ARPC5	1	0	0	1	0	0
ARPC5L	0	0	0	0	1	0
ARPP-19	0	0	0	0	1	0
ARPP-21	0	1	1	0	0	0
ARRDC3	1	0	0	1	0	0
ARRDC4	0	0	0	1	0	0
ARSA	0	0	0	1	0	0
ARSD	0	0	0	1	0	0
ARSF	0	0	1	0	0	0
ARSG	1	1	1	0	0	1
ART3	0	0	1	0	0	1
ARTS-1	1	0	1	1	0	1
ARV1	1	0	0	1	0	1
ASAH1	1	0	0	1	0	0
ASAH3	0	0	1	0	0	1
ASB13	0	1	0	0	0	0
ASB16	0	0	1	0	0	0
ASB17	1	1	0	0	0	0

ASB3	1	0	1	1	0	1
ASB4	1	0	1	1	0	1
ASB7	1	0	0	1	0	1
ASB8	0	0	0	1	0	0
ASB9	0	0	1	0	0	0
ASCC1	1	0	0	1	0	0
ASCC2	0	0	0	1	1	0
ASCC3L1	1	0	0	1	0	0
ASCIZ	0	0	1	0	1	0
ASF1A	1	0	0	1	0	0
ASF1B	1	0	0	0	0	0
ASGR2	0	0	0	0	1	0
ASH1L	0	0	0	1	0	0
ASH2L	1	0	0	0	0	0
ASIP	1	0	1	0	0	1
ASL	1	0	0	0	0	0
ASNA1	1	0	1	1	0	1
ASNSD1	0	0	0	1	0	0
ASPH	1	0	1	1	0	1
ASPHD1	0	0	0	0	0	1
ASTE1	1	0	0	1	1	0
ASTN2	0	1	0	0	0	0
ASXL2	1	1	0	0	1	0
ATAD2	1	0	0	1	0	0
ATAD3B	1	0	0	0	0	0
ATAD4	0	0	1	0	0	1
ATF1	1	0	0	1	1	1
ATF2	1	0	0	1	0	0
ATF3	1	0	1	0	0	1
ATF4	1	0	0	0	0	0
ATF5	1	0	1	1	0	1
ATF6	1	0	0	0	0	0
ATF7IP2	0	0	1	0	0	1
ATG12	1	0	0	1	0	1
ATG16L2	0	0	0	0	1	0
ATG3	1	0	0	0	0	0
ATG4A	1	0	0	1	0	1
ATG5	1	0	0	0	0	0
ATG9A	1	0	0	0	0	0
ATN1	1	0	1	0	0	0
ATOX1	1	0	0	0	0	0
ATP10D	1	0	0	0	0	0
ATP11C	0	1	1	0	0	1
ATP13A1	1	0	0	1	1	0
ATP1B1	1	0	0	0	0	0
ATP1B3	1	0	0	0	0	0
ATP1B4	0	1	1	0	1	1
ATP2B1	0	0	0	1	0	0
ATP2B3	1	0	1	0	0	1
ATP2C1	1	0	0	1	1	0
ATP4A	0	0	1	0	0	1
ATP5A1	1	0	0	1	0	0
ATP5B	1	0	0	1	0	0
ATP5D	1	0	0	1	0	0
ATP5F1	1	1	0	1	1	0
ATP5G1	1	0	0	0	0	0
ATP5G2	1	0	0	0	1	0
ATP5G3	1	1	1	0	0	1
ATP5H	0	0	0	1	0	0

ATP5I	0	0	1	1	0	0
ATP5J	1	0	0	1	0	0
ATP5L	1	0	0	1	0	0
ATP5O	0	0	0	1	0	0
ATP5S	0	0	0	1	0	0
ATP6AP1	1	1	0	1	1	1
ATP6V0B	1	0	0	1	0	0
ATP6V0C	1	0	0	0	0	0
ATP6V0D2	0	0	1	1	0	1
ATP6V1A	1	0	0	0	0	0
ATP6V1B2	1	0	0	1	1	1
ATP6V1C1	0	0	0	1	0	0
ATP6V1D	1	0	0	1	1	1
ATP6V1E1	1	0	0	1	0	0
ATP6V1E2	0	0	1	0	0	1
ATP6V1G1	0	0	0	0	1	0
ATP6V1G2	1	0	1	1	0	1
ATP6V1H	1	0	0	0	0	0
ATP7A	1	1	1	1	0	1
ATP7B	1	0	0	1	0	0
ATP8B3	1	0	0	0	0	0
ATP8B4	0	0	0	1	0	1
ATP9B	1	0	0	0	0	0
ATPAF1	0	0	0	1	0	0
ATPAF2	1	0	0	1	0	0
ATPBD1C	1	0	0	1	1	0
ATPBD3	0	0	0	1	1	0
ATPBD4	1	0	1	0	0	1
ATPIF1	1	0	0	1	0	0
ATR	1	0	0	1	0	0
ATRN	1	0	0	1	0	0
ATXN10	1	0	0	0	0	0
ATXN2	1	0	0	0	0	0
ATXN2L	1	0	0	1	0	1
ATXN3	1	0	0	0	0	0
ATXN7L2	1	0	1	1	0	0
AUP1	1	0	0	1	0	0
AURKA	1	0	0	1	1	0
AURKB	1	0	0	1	0	0
AVIL	0	0	1	0	0	1
AVPI1	1	0	0	1	0	0
AXL	1	0	1	1	0	1
AYTL1	0	0	1	0	0	1
AZI2	0	1	0	1	0	0
AZIN1	1	0	0	0	0	0
B2M	1	0	0	1	1	0
B3GALT4	1	0	0	1	0	1
B3GALT5	0	0	1	0	0	1
B3GALT6	1	0	0	1	1	0
B3GALTL	1	0	1	0	0	1
B3GAT3	1	0	0	1	0	0
B3GNT1	1	0	0	1	0	0
B3GNT2	0	0	0	0	0	1
B3GNT5	1	0	0	1	0	0
B3GNTL1	1	0	0	0	0	0
B4GALT1	1	0	0	0	1	0
B4GALT2	1	0	0	1	0	0
B4GALT6	0	1	0	0	0	0
BACE1	1	0	0	0	0	0

BACH1	1	0	0	1	0	1
BAG1	1	0	0	1	1	0
BAG4	1	0	0	1	0	1
BANF1	1	0	0	1	0	0
BANP	1	0	0	1	0	0
BAP1	1	0	0	1	1	0
BARD1	1	0	0	0	0	0
BAT1	1	0	1	1	0	1
BAT3	1	0	0	1	0	0
BAT4	1	0	0	1	1	0
BATF2	1	0	1	0	0	1
BAZ2A	1	0	0	1	0	0
BAZ2B	1	0	0	1	0	0
BBOX1	0	0	1	0	0	1
BBS1	1	0	0	1	0	0
BBS7	1	0	0	0	0	0
BC37295_3	1	0	0	0	0	0
BCAM	0	0	0	0	0	1
BCAR3	1	0	0	0	0	0
BCAS2	0	1	0	0	1	0
BCAS3	1	0	0	1	0	0
BCAT1	1	1	0	0	0	0
BCCIP	1	0	0	1	0	0
BCDO2	1	0	0	0	0	1
BCHE	0	1	0	0	0	0
BCKDHA	1	0	0	1	1	0
BCKDK	1	0	0	0	0	0
BCL10	0	0	0	1	0	0
BCL2A1	0	1	0	0	1	0
BCL2L11	1	0	0	0	0	0
BCL2L12	1	0	0	1	1	0
BCL2L14	0	1	1	0	1	1
BCL2L2	1	0	0	0	0	0
BCL6	1	0	1	1	1	1
BCL7C	0	1	0	0	1	0
BCR	0	1	0	0	0	0
BCS1L	1	0	0	0	0	0
BDKRB2	0	0	1	0	0	1
BDNF	1	0	1	0	1	0
BECN1	1	0	0	1	0	0
BET1L	1	0	0	0	1	0
BFAR	0	1	0	0	0	0
BHLHB3	1	0	0	1	0	0
BICD2	1	0	0	1	0	0
BIN3	1	0	1	1	0	1
BIRC2	1	0	0	1	0	0
BIRC4	1	0	0	0	0	0
BIRC6	1	0	0	1	0	0
BLCAP	1	0	0	1	0	0
BLMH	0	0	0	1	0	0
BLNK	0	0	0	1	0	1
BLOC1S1	0	0	1	0	0	1
BLOC1S2	1	0	0	0	1	1
BLR1	0	1	1	0	0	1
BLZF1	1	0	0	1	1	0
BMP2K	1	0	0	0	0	0
BMX	1	0	0	0	0	0
BNIP1	0	0	0	1	0	0
BNIP3	0	0	0	1	0	0

BOLA2	1	0	0	1	0	0
BOLA3	0	0	0	1	0	0
BOP1	1	0	0	1	0	0
BPESC1	0	0	1	0	0	1
BPGM	1	0	0	0	0	0
BPNT1	1	0	0	0	0	0
BRAP	1	0	0	1	1	0
BRCA1	1	0	0	1	0	0
BRCC3	1	1	0	0	0	0
BRD1	1	0	0	0	0	0
BRD2	1	0	0	1	0	0
BRD4	0	0	1	0	0	1
BRD8	1	1	1	1	0	1
BRD9	1	0	0	1	0	0
BRDG1	0	0	0	0	0	1
BRE	1	0	0	1	0	0
BRF1	0	0	0	1	0	0
BRF2	1	0	0	1	0	0
BRIP1	1	0	0	0	0	0
BRMS1	1	0	0	1	0	0
BRP44	1	0	1	1	0	1
BRPF1	1	0	0	1	0	0
BRSK1	1	0	0	0	0	0
BRWD3	0	1	0	0	0	0
BSCL2	1	0	0	1	1	1
BSDC1	0	0	0	0	1	0
BSG	1	0	0	0	0	0
BSPRY	0	0	0	1	0	1
BTAF1	0	0	0	1	0	0
BTBD1	0	1	0	1	0	0
BTBD12	1	0	0	0	0	0
BTBD14A	1	0	0	0	0	0
BTBD14B	1	0	0	1	0	0
BTBD15	1	1	0	0	0	0
BTBD3	0	0	1	0	0	0
BTBD6	0	0	0	1	0	0
BTBD7	1	0	0	1	0	0
BTD	1	1	0	1	1	0
BTF3L4	1	0	0	1	0	0
BTG2	1	0	0	1	0	0
BTK	1	1	1	1	1	1
BTN1A1	1	0	0	0	0	0
BTN2A1	0	0	1	0	0	1
BTN2A3	0	1	0	0	0	0
BTN3A3	0	0	1	1	0	1
BTRC	0	1	0	1	0	0
BUB1B	1	0	0	0	0	1
BUB3	0	0	0	1	0	0
BUD13	1	0	0	0	0	0
BUD31	1	0	0	1	1	0
BXDC1	1	0	0	1	1	0
BXDC2	1	0	0	1	0	0
BXDC5	1	0	0	1	0	0
BYSL	1	0	0	1	0	0
BZW2	1	0	0	1	0	0
C10ORF10	1	0	1	0	0	1
C10ORF104	1	0	0	1	0	0
C10ORF11	0	1	1	1	0	0
C10ORF118	0	1	1	0	1	1

C10ORF12	0	0	1	0	0	0
C10ORF129	0	0	1	0	0	1
C10ORF130	0	0	1	0	1	0
C10ORF22	1	0	1	1	0	1
C10ORF26	1	0	0	1	0	1
C10ORF28	1	0	0	0	1	0
C10ORF30	0	0	0	0	0	1
C10ORF35	0	0	1	0	0	1
C10ORF55	1	0	1	1	0	1
C10ORF6	1	0	0	0	0	1
C10ORF61	0	0	0	1	0	0
C10ORF71	0	1	1	0	0	1
C10ORF76	0	0	1	0	0	1
C10ORF78	0	0	0	1	0	0
C10ORF83	1	0	0	0	0	0
C10ORF88	1	0	0	1	0	0
C10ORF92	0	0	1	0	0	1
C10ORF96	0	0	0	1	0	0
C10ORF97	0	0	0	1	0	0
C10ORF99	0	0	1	0	0	0
C11ORF10	1	0	0	1	1	0
C11ORF16	0	0	1	0	1	1
C11ORF17	1	0	0	0	0	0
C11ORF2	1	1	0	1	0	0
C11ORF30	1	0	0	1	0	0
C11ORF31	1	0	0	1	0	0
C11ORF35	0	0	1	0	0	0
C11ORF42	0	1	0	0	0	0
C11ORF45	0	0	0	1	0	0
C11ORF46	1	1	1	0	0	1
C11ORF47	1	0	1	0	0	1
C11ORF51	0	0	0	0	1	0
C11ORF52	0	0	1	0	0	0
C11ORF54	1	0	0	1	0	0
C11ORF57	1	0	0	1	1	0
C11ORF60	1	0	0	1	0	0
C11ORF61	1	0	0	1	0	0
C11ORF63	1	0	0	1	0	0
C11ORF66	1	1	1	0	0	1
C11ORF67	1	0	0	1	0	0
C11ORF68	1	0	0	0	1	0
C11ORF71	1	0	0	1	1	0
C11ORF74	1	1	0	1	0	1
C11ORF75	0	0	1	0	0	0
C11ORF9	1	0	1	1	1	0
C12ORF10	1	0	0	1	1	0
C12ORF11	1	0	0	1	0	0
C12ORF24	1	0	0	0	0	0
C12ORF26	1	0	0	1	0	1
C12ORF28	0	0	1	0	0	1
C12ORF29	0	0	0	1	0	0
C12ORF30	1	0	0	1	0	0
C12ORF31	0	1	0	0	1	0
C12ORF32	1	0	0	0	0	0
C12ORF34	0	0	1	0	0	1
C12ORF4	1	0	0	1	1	1
C12ORF40	0	0	1	0	0	1
C12ORF41	0	0	0	1	0	0
C12ORF47	1	0	0	1	0	1

C12ORF49	1	0	0	1	0	0
C12ORF5	0	0	0	0	0	1
C12ORF52	1	0	0	0	1	0
C12ORF54	0	1	1	0	0	1
C12ORF57	1	0	1	0	0	0
C12ORF60	1	1	1	1	0	1
C12ORF61	1	0	0	1	0	1
C12ORF62	0	0	0	0	0	1
C13ORF18	1	1	0	0	0	0
C13ORF23	1	0	0	1	1	0
C13ORF24	1	0	0	1	0	0
C13ORF26	0	1	1	0	0	1
C13ORF3	1	0	0	1	1	0
C14ORF1	1	0	1	1	0	1
C14ORF100	1	0	0	0	0	0
C14ORF101	0	0	1	1	0	0
C14ORF102	0	0	0	1	0	0
C14ORF106	1	0	0	1	0	0
C14ORF108	1	0	0	1	0	1
C14ORF112	1	0	0	0	0	0
C14ORF121	0	0	1	1	0	1
C14ORF122	1	0	0	1	0	0
C14ORF124	1	0	0	1	0	0
C14ORF126	1	0	0	0	0	0
C14ORF129	1	0	1	1	1	1
C14ORF130	1	0	0	1	0	1
C14ORF131	0	0	1	0	0	0
C14ORF133	1	0	1	1	0	1
C14ORF135	0	0	1	0	0	0
C14ORF139	0	0	1	0	0	1
C14ORF140	1	0	1	1	0	1
C14ORF142	1	0	0	1	0	1
C14ORF145	1	0	0	1	0	0
C14ORF147	0	0	0	1	0	0
C14ORF148	0	0	1	1	0	1
C14ORF149	1	0	0	0	0	0
C14ORF152	0	1	1	0	0	1
C14ORF153	1	0	0	1	1	0
C14ORF155	0	0	1	1	0	0
C14ORF156	1	0	0	1	1	1
C14ORF166	0	1	0	0	1	0
C14ORF166B	0	0	1	0	0	1
C14ORF169	1	0	0	0	0	0
C14ORF172	0	0	0	1	0	0
C14ORF173	0	0	1	0	0	1
C14ORF174	0	1	0	0	0	0
C14ORF2	1	0	0	0	0	0
C14ORF21	1	1	0	1	1	0
C14ORF28	0	0	0	1	0	0
C14ORF4	1	0	0	0	0	0
C14ORF43	1	0	0	1	0	0
C14ORF45	0	0	1	0	0	1
C14ORF48	0	0	1	0	0	1
C14ORF49	0	0	0	1	0	1
C14ORF68	0	0	1	0	0	1
C14ORF79	1	0	1	1	0	0
C15ORF17	0	0	0	1	0	0
C15ORF23	0	0	1	1	0	1
C15ORF24	1	0	0	1	0	0

C15ORF29	1	0	0	1	1	0
C15ORF39	0	1	0	0	0	0
C15ORF41	0	0	1	0	0	1
C15ORF44	1	0	0	0	0	0
C15ORF48	1	0	0	1	0	0
C15ORF5	0	0	1	0	0	1
C16ORF28	0	0	0	1	0	0
C16ORF33	1	0	0	1	1	0
C16ORF48	0	0	0	1	1	0
C16ORF53	1	0	0	1	0	0
C16ORF58	1	0	0	0	0	0
C16ORF61	1	0	0	1	0	0
C16ORF63	1	0	0	1	0	0
C17ORF32	1	0	0	1	1	1
C17ORF37	0	0	0	1	0	0
C17ORF38	0	0	1	0	1	1
C17ORF39	1	0	0	1	0	0
C17ORF45	1	0	0	0	0	0
C17ORF48	1	0	0	1	0	0
C17ORF49	1	0	0	1	0	0
C17ORF58	1	0	0	0	0	0
C17ORF61	0	0	0	1	0	0
C17ORF62	1	1	0	0	1	0
C17ORF64	1	0	0	1	0	1
C17ORF65	1	0	0	1	1	0
C17ORF68	1	0	0	1	1	0
C17ORF74	0	1	0	0	0	1
C17ORF75	0	0	0	0	0	1
C17ORF77	0	0	1	0	0	0
C17ORF79	0	0	0	0	0	1
C17ORF80	1	0	0	1	0	0
C17ORF81	1	0	0	1	0	1
C18ORF1	1	0	1	1	0	1
C18ORF10	0	1	0	0	1	0
C18ORF19	1	0	0	1	0	1
C18ORF21	1	0	0	1	0	0
C18ORF22	1	0	0	0	0	0
C18ORF26	0	1	1	0	0	0
C18ORF37	1	0	1	1	0	1
C18ORF45	0	0	0	1	0	0
C18ORF54	0	1	0	1	0	0
C18ORF55	1	0	0	1	1	1
C19ORF23	1	0	0	1	0	0
C19ORF24	1	0	0	1	0	0
C19ORF30	0	1	1	0	0	1
C19ORF33	0	0	1	0	0	1
C19ORF39	1	0	0	1	0	0
C19ORF40	1	0	0	1	0	0
C19ORF42	0	0	0	1	0	0
C19ORF43	1	0	0	1	0	0
C1D	0	0	0	1	0	0
C1GALT1	1	0	1	0	0	0
C1QA	1	0	1	1	0	1
C1QB	0	0	1	0	0	1
C1QBP	1	0	0	1	0	0
C1QC	0	0	1	0	0	1
C1QTNF6	0	0	1	0	0	1
C1QTNF7	0	0	0	1	1	0
C1QTNF8	0	0	1	0	0	1

C1RL	0	0	1	1	0	0
C1S	0	0	1	0	0	1
C1ORF101	0	0	0	0	1	0
C1ORF102	1	0	0	1	0	1
C1ORF103	0	0	0	1	0	0
C1ORF105	1	0	1	1	0	0
C1ORF107	1	0	1	1	0	0
C1ORF111	0	1	1	0	1	1
C1ORF112	1	0	0	0	0	0
C1ORF120	0	1	1	0	0	1
C1ORF121	0	1	0	0	0	0
C1ORF122	1	0	0	1	1	0
C1ORF123	1	0	0	1	0	0
C1ORF124	1	0	0	1	1	0
C1ORF127	0	0	0	1	0	0
C1ORF128	0	0	0	1	1	0
C1ORF130	0	0	1	0	0	1
C1ORF131	1	0	0	1	0	0
C1ORF135	0	0	0	0	1	0
C1ORF141	0	0	0	0	0	1
C1ORF142	1	0	1	1	0	0
C1ORF144	0	0	0	1	0	0
C1ORF152	0	0	1	0	0	1
C1ORF156	1	0	0	0	0	0
C1ORF160	1	0	0	1	0	0
C1ORF162	0	0	0	1	0	0
C1ORF166	1	0	0	1	0	0
C1ORF174	1	0	0	0	0	0
C1ORF175	0	1	1	0	0	1
C1ORF183	1	0	0	1	0	0
C1ORF2	1	0	0	1	1	0
C1ORF201	0	0	0	0	1	0
C1ORF24	1	0	0	0	0	0
C1ORF25	1	0	0	1	0	1
C1ORF26	1	0	0	1	0	1
C1ORF27	1	0	0	1	0	0
C1ORF31	1	0	0	1	0	0
C1ORF35	0	0	0	1	0	0
C1ORF38	1	0	0	0	0	0
C1ORF41	1	0	0	1	0	0
C1ORF43	1	0	1	1	0	1
C1ORF50	1	0	0	1	0	0
C1ORF51	0	0	0	1	0	0
C1ORF54	1	0	0	1	0	0
C1ORF55	1	0	0	1	0	0
C1ORF57	0	1	0	1	0	0
C1ORF58	1	1	1	1	1	1
C1ORF66	1	0	0	1	1	0
C1ORF71	1	0	0	1	0	0
C1ORF74	1	0	0	1	1	1
C1ORF75	0	1	0	0	0	0
C1ORF77	0	0	1	0	1	1
C1ORF83	1	0	0	1	0	0
C1ORF84	1	0	0	1	0	1
C1ORF91	1	0	0	1	1	0
C1ORF94	0	0	1	1	0	0
C1ORF97	1	0	0	0	0	0
C2	0	0	1	0	0	1
C20ORF11	1	1	0	1	1	0

C20ORF111	1	0	0	1	0	0
C20ORF117	1	0	1	1	1	1
C20ORF12	1	0	0	1	0	1
C20ORF127	0	0	1	0	0	1
C20ORF133	0	0	1	0	0	1
C20ORF165	1	0	0	0	1	0
C20ORF177	1	0	0	0	0	0
C20ORF26	1	0	1	1	0	0
C20ORF29	1	0	0	1	1	0
C20ORF3	1	0	0	1	0	0
C20ORF30	1	0	0	1	0	0
C20ORF32	1	0	0	1	0	0
C20ORF4	0	1	0	0	0	0
C20ORF43	1	0	1	0	0	1
C20ORF52	1	0	0	1	0	0
C20ORF54	0	0	1	0	0	1
C20ORF59	1	0	0	0	0	0
C20ORF72	1	0	0	1	0	0
C20ORF74	0	0	0	1	0	0
C20ORF94	1	0	1	1	0	0
C21ORF119	1	0	0	1	0	0
C21ORF123	0	0	1	0	0	0
C21ORF129	0	0	0	0	0	1
C21ORF2	0	0	1	0	1	0
C21ORF33	1	0	0	1	0	0
C21ORF34	1	0	1	1	0	0
C21ORF45	0	0	0	1	0	0
C21ORF51	1	0	0	0	0	0
C21ORF55	1	0	0	1	0	0
C21ORF57	1	0	0	1	0	0
C21ORF62	0	0	0	0	0	1
C21ORF66	1	1	0	1	1	0
C21ORF67	1	0	0	1	0	0
C21ORF69	1	0	0	1	0	0
C21ORF7	1	0	0	1	0	0
C21ORF70	1	0	0	1	0	0
C21ORF81	0	0	1	0	0	0
C21ORF91	0	0	0	0	1	0
C22ORF13	1	0	0	1	0	0
C22ORF15	0	0	0	1	0	0
C22ORF16	0	0	0	1	0	0
C2ORF13	1	0	0	1	0	0
C2ORF15	1	0	0	1	1	1
C2ORF16	0	0	0	0	0	1
C2ORF21	0	0	1	0	0	1
C2ORF24	1	0	1	1	0	1
C2ORF25	0	0	0	1	0	0
C2ORF27	1	0	1	0	0	1
C2ORF28	1	0	0	1	0	0
C2ORF29	1	0	0	1	0	0
C2ORF30	1	0	0	1	0	0
C2ORF34	1	0	0	1	0	0
C2ORF37	1	0	0	1	0	0
C2ORF7	0	0	0	0	1	0
C3AR1	0	0	0	1	0	0
C3ORF1	0	0	0	1	0	0
C3ORF18	1	0	0	0	0	0
C3ORF20	0	0	1	0	0	1
C3ORF21	1	0	0	1	0	0

C3ORF22	0	0	1	0	0	0
C3ORF23	0	0	0	1	0	1
C3ORF26	1	1	1	1	1	1
C3ORF28	1	0	0	1	0	0
C3ORF31	0	0	0	1	0	0
C3ORF35	1	0	1	0	0	1
C3ORF37	1	0	0	1	0	0
C3ORF38	0	1	0	0	0	0
C3ORF39	0	0	1	0	0	1
C3ORF48	0	1	1	0	1	1
C3ORF54	1	0	1	1	0	0
C3ORF58	0	0	0	0	1	0
C3ORF59	1	0	0	1	0	0
C3ORF60	1	0	0	1	0	0
C3ORF62	1	0	0	1	1	0
C4BPA	1	1	1	1	0	0
C4BPB	1	1	1	1	0	0
C4ORF11	0	0	1	0	0	1
C4ORF14	1	0	0	0	0	0
C4ORF16	1	0	0	1	0	1
C4ORF8	0	0	1	0	0	1
C5	0	0	1	0	0	0
C5ORF13	0	1	1	0	1	1
C5ORF14	0	0	0	1	0	0
C5ORF15	1	0	0	1	0	0
C5ORF20	0	0	1	0	1	1
C5ORF22	1	0	0	1	0	0
C5ORF24	1	0	0	1	0	0
C5ORF3	1	0	0	1	0	0
C5ORF4	1	0	1	1	1	1
C5ORF5	1	1	0	1	0	0
C6ORF105	0	0	0	1	0	1
C6ORF113	1	0	0	0	0	0
C6ORF114	1	0	0	0	0	0
C6ORF120	1	0	0	1	0	0
C6ORF128	1	0	0	1	0	1
C6ORF129	0	0	0	0	1	0
C6ORF130	1	0	0	0	0	0
C6ORF134	0	0	0	1	1	0
C6ORF136	0	0	0	0	1	0
C6ORF145	1	0	0	1	0	0
C6ORF153	0	0	0	1	0	0
C6ORF157	1	0	0	0	0	0
C6ORF162	0	0	1	0	0	1
C6ORF163	1	0	1	1	0	0
C6ORF173	0	0	0	0	0	1
C6ORF182	0	0	1	0	0	1
C6ORF199	1	0	0	1	0	0
C6ORF203	1	0	0	1	0	1
C6ORF211	1	0	0	1	0	0
C6ORF218	0	0	0	0	0	1
C6ORF25	0	1	1	0	0	1
C6ORF27	0	1	1	1	0	1
C6ORF32	0	0	1	0	0	1
C6ORF47	1	0	0	1	0	0
C6ORF49	1	0	0	0	0	0
C6ORF52	1	0	0	1	0	0
C6ORF59	0	1	1	0	1	1
C6ORF61	1	0	0	1	0	0

C6ORF62	1	0	0	0	1	0
C6ORF66	1	0	0	1	0	0
C6ORF70	1	1	0	1	0	0
C6ORF72	1	0	0	0	0	0
C6ORF85	0	1	1	0	0	1
C6ORF89	1	0	1	1	0	0
C7ORF10	1	0	0	1	0	0
C7ORF11	1	0	0	1	0	0
C7ORF13	1	0	0	0	0	0
C7ORF20	1	0	0	0	0	0
C7ORF23	0	0	0	0	1	0
C7ORF25	0	0	1	1	0	0
C7ORF26	1	0	0	1	0	0
C7ORF29	0	1	1	1	1	1
C7ORF38	1	0	0	1	0	0
C8B	0	1	1	0	0	1
C8ORF30A	0	0	1	1	0	0
C8ORF32	1	0	0	1	0	0
C8ORF33	1	0	0	1	0	0
C8ORF34	0	0	1	1	0	1
C8ORF38	0	0	0	1	0	0
C8ORF40	1	0	0	1	0	1
C8ORF41	1	0	0	0	0	0
C8ORF44	1	0	0	1	0	1
C8ORF46	0	0	1	0	0	1
C8ORF51	1	0	0	0	1	0
C8ORF53	1	0	0	0	0	0
C8ORF54	0	0	1	0	0	1
C8ORF58	1	0	0	1	0	0
C8ORF70	1	0	0	1	0	1
C8ORF74	0	0	1	0	0	1
C8ORF76	1	0	0	1	0	0
C9ORF100	0	1	0	0	0	0
C9ORF103	0	1	0	0	0	0
C9ORF116	1	0	0	1	0	0
C9ORF119	1	0	0	0	1	0
C9ORF130	1	0	0	1	0	0
C9ORF140	0	0	0	0	1	0
C9ORF142	0	0	0	1	0	0
C9ORF164	1	0	1	0	0	1
C9ORF18	0	1	0	0	0	0
C9ORF21	1	0	0	1	0	1
C9ORF23	1	0	0	0	0	0
C9ORF24	0	0	1	0	0	1
C9ORF25	0	0	1	1	0	1
C9ORF3	1	0	0	0	0	0
C9ORF30	1	0	0	0	0	0
C9ORF37	1	0	0	0	0	0
C9ORF40	1	0	0	0	0	0
C9ORF45	0	0	1	0	0	1
C9ORF46	1	0	1	1	0	1
C9ORF5	0	1	0	0	0	0
C9ORF6	1	0	0	1	1	0
C9ORF64	1	0	0	0	0	0
C9ORF72	1	0	0	0	0	0
C9ORF78	1	0	0	1	0	0
C9ORF79	0	1	1	0	0	1
C9ORF80	1	0	0	1	0	0
C9ORF85	1	0	0	1	0	1

C9ORF9	1	0	0	0	0	0
C9ORF90	1	0	0	1	0	0
C9ORF93	0	0	0	1	0	0
C9ORF95	1	0	0	0	0	0
C9ORF97	1	0	0	1	0	0
C9ORF98	1	0	0	0	0	0
CA1	0	0	1	0	0	1
CA2	0	0	0	0	0	1
CA5A	0	1	1	0	1	1
CA5B	1	0	0	1	0	0
CAB39L	0	1	1	0	0	1
CABLES1	1	0	1	0	0	1
CABYR	1	0	0	0	0	1
CACNA1I	0	0	1	0	0	1
CACNB1	0	0	0	0	1	0
CACNB4	0	1	1	0	1	1
CACNG3	0	0	1	0	0	1
CACNG7	0	0	1	0	0	0
CACYBP	1	0	0	1	0	0
CAD	1	0	0	1	0	0
CAGE1	1	0	0	1	0	1
CALCOCO1	1	0	0	0	0	0
CALCRL	1	0	0	0	0	0
CALD1	0	1	1	0	0	1
CALM1	1	0	0	0	0	0
CALM2	1	0	1	0	1	1
CALN1	0	0	1	0	0	1
CALR	1	0	1	1	0	1
CAMK1D	1	0	0	0	0	0
CAMK1G	0	0	1	0	0	1
CAMKK1	1	0	0	1	0	0
CAMP	0	0	1	0	1	0
CAMSAP1	1	0	1	1	1	1
CAMTA2	1	0	0	1	0	0
CAND1	1	0	0	0	0	0
CANT1	1	0	0	0	0	0
CAP1	1	0	0	1	0	0
CAPG	1	0	1	1	0	1
CAPN10	1	0	0	1	0	0
CAPN11	0	1	1	0	0	1
CAPN2	0	0	0	1	0	0
CAPN3	0	0	1	0	1	1
CAPN5	0	1	1	0	0	1
CAPN6	0	0	1	0	0	1
CAPNS2	0	0	0	0	0	1
CAPS	0	0	1	0	1	0
CAPSL	0	1	1	0	1	1
CAPZA2	0	0	0	1	0	0
CAPZB	1	0	1	0	0	1
CARD14	0	0	1	0	0	1
CARD6	0	0	0	1	0	0
CARD8	1	0	0	1	0	0
CARD9	1	0	0	0	0	0
CARHSP1	1	0	1	1	0	0
CARM1	1	0	0	1	0	0
CASC3	0	0	0	1	0	0
CASC4	1	0	0	0	0	0
CASC5	0	1	0	1	1	0
CASD1	0	1	0	0	0	0

CASK	0	1	0	1	0	0
CASP10	0	0	0	1	1	0
CASP2	1	0	1	1	0	1
CASP3	1	0	0	1	1	0
CASP5	0	0	0	1	0	0
CASP8	1	0	0	0	0	0
CAST	1	0	1	0	0	0
CASZ1	0	0	0	0	0	1
CATSPER1	0	0	1	1	0	0
CATSPER3	1	0	1	0	0	1
CAV3	0	0	1	0	0	1
CBARA1	0	1	0	0	0	0
CBFA2T2	0	0	0	0	0	1
CBFA2T3	0	1	0	0	0	0
CBFB	0	1	0	0	0	0
CBLB	1	0	0	1	0	0
CBLC	0	0	1	0	0	1
CBLL1	1	0	0	1	0	0
CBR4	1	0	0	0	0	0
CBX1	0	0	0	0	1	0
CBX2	0	0	0	1	0	0
CBX5	1	0	0	1	0	0
CC2D1A	0	0	1	1	1	1
CCBL1	1	0	0	0	0	0
CCDC100	0	0	1	0	0	1
CCDC101	1	0	0	1	0	0
CCDC102B	0	0	1	0	0	1
CCDC12	0	0	0	1	1	0
CCDC14	1	0	0	0	0	0
CCDC15	1	0	0	1	0	0
CCDC16	1	0	0	1	0	1
CCDC17	0	0	0	1	0	0
CCDC18	0	0	0	0	0	1
CCDC19	0	1	0	0	0	0
CCDC21	0	0	0	1	0	0
CCDC22	1	1	1	0	0	0
CCDC23	1	1	0	1	0	0
CCDC26	0	1	1	0	0	1
CCDC3	0	0	1	0	0	0
CCDC33	0	0	1	0	0	1
CCDC34	0	0	0	1	0	0
CCDC43	0	0	0	0	1	0
CCDC44	1	0	0	0	0	1
CCDC45	1	0	0	1	0	0
CCDC46	0	0	1	0	0	1
CCDC47	1	0	0	1	1	0
CCDC48	0	1	1	0	0	1
CCDC51	1	0	0	1	0	0
CCDC53	0	0	1	1	0	1
CCDC55	1	0	0	1	0	0
CCDC56	0	0	0	1	0	0
CCDC58	1	0	0	1	0	0
CCDC59	1	0	0	1	0	1
CCDC6	1	0	0	0	1	0
CCDC60	0	0	1	0	0	1
CCDC66	1	0	0	0	0	0
CCDC71	1	0	0	0	0	0
CCDC72	1	0	0	1	0	0
CCDC76	1	0	0	1	0	0

CCDC77	1	0	0	0	0	0
CCDC81	0	0	0	0	0	1
CCDC82	0	0	0	1	1	0
CCDC84	1	0	0	1	1	0
CCDC85B	0	0	0	0	1	0
CCDC86	1	0	0	1	0	0
CCDC9	0	0	0	1	0	0
CCDC92	1	0	1	1	0	1
CCDC93	0	0	1	0	0	1
CCDC95	1	0	0	1	0	0
CCDC97	1	0	0	0	1	0
CCHCR1	0	0	0	1	0	1
CCL1	0	1	1	0	0	1
CCL13	0	0	1	0	0	1
CCL15	0	1	1	0	1	1
CCL16	0	1	1	0	0	1
CCL17	0	0	1	0	0	1
CCL18	0	0	1	0	0	1
CCL19	0	1	0	0	0	1
CCL2	0	0	1	1	0	1
CCL20	1	0	1	0	0	1
CCL22	0	0	1	0	0	1
CCL23	0	0	1	0	0	0
CCL24	1	0	0	0	0	0
CCL26	0	0	1	0	0	1
CCL27	0	1	0	1	1	0
CCL3	0	0	0	1	1	0
CCL3L1	0	0	0	1	0	1
CCL5	0	1	1	0	0	1
CCL7	0	0	1	0	0	1
CCM2	1	0	0	0	0	0
CCNB2	0	0	1	0	0	1
CCNDBP1	1	0	0	1	1	0
CCNF	0	0	0	1	0	0
CCNG2	0	0	0	0	1	0
CCNH	1	1	1	0	0	0
CCNJ	0	0	0	1	0	0
CCNK	1	0	0	1	0	1
CCNL1	1	0	0	0	1	0
CCR1	1	0	0	1	0	0
CCR2	0	1	0	0	1	0
CCR7	0	1	0	0	0	0
CCR9	0	1	1	0	0	1
CCS	1	0	0	1	0	0
CCT2	1	0	0	0	0	0
CCT3	1	0	0	1	0	0
CCT4	1	0	0	0	0	0
CCT5	1	0	0	0	0	0
CCT6A	1	0	0	1	0	0
CCT6B	1	0	0	1	0	1
CCT7	0	0	0	0	1	0
CCT8	1	0	0	1	0	0
CD14	0	1	0	0	0	0
CD151	1	0	0	1	0	0
CD163	0	0	0	1	0	0
CD163L1	0	1	1	0	0	1
CD1A	1	0	1	0	0	0
CD1B	1	0	1	1	0	1
CD1C	1	0	1	0	0	0

CD1E	0	0	1	0	0	1
CD200	0	1	0	0	0	0
CD200R1	1	0	0	0	0	0
CD207	0	0	1	0	0	1
CD209	1	0	1	1	0	1
CD22	0	0	1	1	0	1
CD247	0	0	1	0	0	1
CD274	1	0	1	1	0	1
CD2BP2	1	0	0	1	0	0
CD300E	0	1	0	0	1	0
CD300LF	0	0	1	1	0	1
CD300LG	0	0	0	0	0	1
CD33	0	0	1	0	0	0
CD36	0	1	1	1	0	0
CD37	0	0	0	1	0	0
CD3EAP	1	0	0	1	0	0
CD44	0	0	0	1	0	0
CD46	1	0	0	1	0	0
CD48	0	0	0	0	1	0
CD52	0	0	0	0	1	0
CD53	1	0	0	0	1	0
CD6	0	0	1	0	0	1
CD63	1	0	0	1	0	1
CD68	1	0	0	1	1	1
CD69	0	0	0	1	0	0
CD72	1	0	1	0	0	1
CD74	0	0	0	1	0	0
CD79A	1	0	0	0	0	0
CD80	1	0	0	1	0	1
CD82	0	0	0	0	1	0
CD83	1	0	0	0	0	0
CD84	0	1	0	0	1	0
CD86	1	0	0	1	0	0
CD9	0	0	0	1	0	0
CD97	0	0	0	1	0	0
CD99	0	0	0	1	0	0
CDA	0	1	1	0	0	1
CDC16	0	0	0	1	0	0
CDC2	1	0	1	1	0	0
CDC25A	1	1	0	1	0	0
CDC25B	1	0	0	0	0	0
CDC25C	1	0	0	1	0	0
CDC26	1	1	0	1	0	0
CDC2L1	1	0	0	1	0	0
CDC2L2	1	0	0	1	0	0
CDC2L6	1	0	0	0	0	0
CDC37L1	0	1	0	0	0	0
CDC42	0	0	0	1	0	0
CDC42BPB	1	0	0	1	0	0
CDC42SE2	1	1	0	0	1	1
CDC45L	1	0	0	1	0	0
CDC5L	0	0	0	1	0	0
CDC7	1	0	0	1	0	0
CDC73	0	0	0	0	0	1
CDCA2	1	0	0	1	0	0
CDCA3	0	0	0	1	0	0
CDCA4	1	0	1	1	0	1
CDCA5	1	0	0	1	1	0
CDCA8	1	0	0	1	1	0

CDH15	0	0	0	0	0	1
CDH17	0	0	1	0	0	1
CDH2	0	0	0	0	0	1
CDH26	0	1	1	0	0	1
CDH7	0	0	1	0	0	0
CDH9	0	0	0	0	1	0
CDK10	0	0	0	0	0	1
CDK2	0	0	0	1	0	0
CDK2AP2	0	0	0	1	0	0
CDK4	0	0	0	1	1	0
CDK5RAP1	1	0	0	0	0	0
CDK5RAP2	0	0	0	1	0	0
CDK5RAP3	1	0	0	1	0	0
CDK7	1	0	0	1	0	0
CDK8	0	1	0	0	0	0
CDKAL1	1	0	0	1	0	0
CDKL3	1	0	0	1	0	1
CDKL4	1	0	0	0	0	0
CDKN1A	1	0	1	1	0	1
CDKN1B	0	1	0	0	1	0
CDKN2D	0	1	0	0	0	0
CDR2	1	0	0	1	0	0
CDRT4	0	1	1	0	0	1
CDS2	1	0	0	1	0	0
CDT1	0	0	1	0	0	0
CDV3	0	0	0	1	1	0
CDYL	0	0	1	0	0	1
CDYL2	0	0	0	1	0	0
CEACAM16	0	0	1	1	0	1
CEACAM19	0	0	1	0	0	1
CEACAM8	0	1	1	1	0	1
CEBPA	1	0	0	0	0	0
CEBPE	0	0	0	1	0	0
CEBPZ	1	0	0	1	1	0
CECR1	0	0	1	1	1	1
CECR5	1	1	1	1	0	0
CELSR1	1	0	0	1	0	0
CELSR3	0	0	1	0	0	0
CENPB	1	0	0	1	1	0
CENPC1	0	1	1	1	0	0
CENPE	1	0	0	1	0	0
CENPF	1	0	0	0	0	0
CENPH	1	0	0	1	1	0
CENTA1	0	1	0	0	0	0
CENTB1	0	0	0	1	0	0
CENTB2	1	0	0	1	0	0
CENTD2	0	0	1	1	0	1
CENTD3	1	0	0	0	0	0
CENTG1	0	0	0	1	0	0
CEP110	0	0	0	1	0	1
CEP152	0	0	0	1	0	0
CEP164	1	0	0	0	0	0
CEP192	0	0	1	0	1	1
CEP250	0	0	0	1	0	0
CEP27	1	0	0	1	0	0
CEP290	1	0	0	1	0	0
CEP350	1	0	0	0	0	0
CEP55	0	1	0	1	0	0
CEP57	1	0	0	0	0	0

CEP63	0	1	0	0	1	0
CEP68	1	0	1	1	0	0
CEP70	0	1	0	0	0	0
CEP76	1	0	0	1	0	0
CEPT1	1	0	0	1	0	1
CER1	0	1	1	1	0	1
CERK	0	0	0	0	0	1
CERKL	1	0	0	0	0	0
CES1	0	0	0	1	0	0
CES2	1	0	0	1	1	0
CES7	0	0	1	0	0	1
CETN2	1	1	1	1	1	1
CETN3	1	0	0	0	0	0
CETP	0	1	1	0	0	0
CFB	1	0	1	0	0	1
CFH	1	0	0	0	0	1
CFI	0	1	1	0	1	0
CFL1	1	0	0	1	0	0
CFL2	0	0	0	1	0	0
CFP	0	1	1	0	0	1
CGGBP1	1	0	0	1	0	0
CGI-09	1	1	0	1	0	0
CH25H	1	0	0	0	0	0
CHAC2	1	0	0	1	0	0
CHAF1A	1	0	0	1	0	0
CHAF1B	0	1	0	0	0	0
CHCHD1	1	0	0	1	0	0
CHCHD3	0	0	0	1	0	0
CHCHD4	1	0	0	1	1	0
CHCHD5	1	1	0	1	0	1
CHCHD7	1	0	0	0	0	0
CHCHD8	1	0	0	1	0	0
CHD3	0	1	0	0	0	0
CHD4	1	1	0	0	1	0
CHD7	1	0	0	0	0	0
CHD8	1	0	0	1	0	0
CHD9	0	0	1	0	1	0
CHDH	1	0	0	1	0	0
CHEK1	1	0	1	1	0	0
CHEK2	1	0	0	1	0	0
CHERP	1	0	0	1	0	0
CHI3L1	0	1	1	0	0	1
CHIC2	0	0	0	1	0	0
CHIT1	0	0	1	0	0	1
CHM	0	1	0	0	0	0
CHML	1	0	0	0	0	1
CHMP2A	1	0	0	1	0	0
CHMP4A	1	0	1	1	0	0
CHMP4B	0	0	0	0	1	0
CHMP5	1	0	0	1	1	0
CHMP7	1	0	0	1	0	0
CHN2	1	0	1	0	0	1
CHORDC1	1	0	0	1	0	0
CHP	1	0	0	1	0	0
CHPT1	0	1	0	0	1	0
CHRAC1	1	0	0	1	0	0
CHRNA10	0	0	0	0	0	1
CHRNA4	0	0	1	0	0	1
CHRNB1	0	0	0	1	0	0

CHRNB3	0	0	1	0	0	1
CHRNE	0	0	0	1	0	0
CHST10	1	0	0	0	0	0
CHST12	1	0	1	0	1	1
CHST7	0	1	1	0	0	0
CHST8	1	0	1	0	0	1
CHSY1	1	0	0	1	0	0
CHTF18	1	0	0	0	0	1
CHURC1	1	0	0	1	0	0
CIAPIN1	1	0	0	0	0	0
CIB1	1	0	0	1	0	0
CIB3	0	1	0	0	0	1
CIC	0	1	0	1	1	0
CIDEB	1	1	0	1	1	0
CIDEC	1	0	1	0	1	1
CINP	1	0	0	1	1	0
CIP29	1	0	0	1	0	0
CIR	1	0	0	1	0	1
CIRBP	1	0	0	1	0	0
CIRH1A	1	0	0	1	1	0
CISH	1	0	1	1	0	1
CIZ1	1	0	0	1	0	0
CKAP2	1	0	0	1	0	0
CKAP4	0	1	0	0	0	0
CKAP5	0	1	0	0	0	0
CKLF	1	0	1	1	0	1
CKS1B	1	0	0	1	1	0
CKS2	1	1	1	1	1	0
CLASP2	0	0	0	0	1	0
CLC	0	0	1	0	0	1
CLCN1	0	0	0	0	1	0
CLCN3	0	0	0	1	0	0
CLCN5	0	1	1	0	0	1
CLCN6	1	0	1	1	1	1
CLDN1	0	0	0	0	0	1
CLDN14	0	0	1	0	0	1
CLDN7	0	0	0	1	0	1
CLDN8	1	0	1	1	0	1
CLDND1	0	1	0	0	1	0
CLEC12B	1	0	0	0	0	0
CLEC1A	1	0	1	0	0	1
CLEC1B	0	0	0	1	0	1
CLEC2D	0	0	1	0	0	0
CLEC3B	0	0	1	0	0	1
CLEC4A	1	0	1	0	0	1
CLEC4D	1	0	0	0	0	0
CLEC5A	0	1	0	0	0	0
CLEC6A	0	0	0	1	0	0
CLEC9A	0	1	1	0	0	1
CLIC1	1	0	0	0	0	0
CLIC2	1	0	1	1	0	0
CLIC3	0	0	0	1	0	0
CLIC4	0	0	0	1	0	0
CLK2	1	0	0	1	1	0
CLK3	0	1	1	0	0	1
CLK4	1	0	0	0	0	0
CLN3	1	0	1	0	1	1
CLN5	0	1	0	1	0	1
CLN6	1	0	0	1	0	0

CLN8	1	0	1	1	0	1
CLOCK	0	0	1	0	0	0
CLPTM1	1	0	0	1	0	0
CLTC	1	0	0	1	0	0
CLTCL1	1	1	0	0	0	0
CLUAP1	0	0	1	1	0	1
CLUL1	0	0	1	1	0	0
CLYBL	0	0	0	1	0	0
CMAS	0	0	0	0	0	1
CMIP	1	0	0	0	0	0
CMPK	1	0	0	1	0	0
CMTM1	1	0	1	1	0	1
CMTM5	0	1	1	0	0	1
CMTM6	1	0	0	0	0	0
CMTM7	1	0	0	0	0	0
CMYA5	0	0	1	0	0	1
CNFN	0	0	0	0	0	1
CNGA1	0	1	1	1	0	1
CNNM2	0	0	0	1	0	0
CNNM3	0	0	0	1	0	0
CNNM4	1	1	0	1	0	0
CNO	0	0	0	1	1	1
CNOT1	1	0	0	1	0	0
CNOT10	1	0	0	1	0	0
CNOT3	1	0	0	1	0	0
CNOT7	1	0	0	1	0	0
CNOT8	0	0	0	1	0	0
CNP	1	0	0	1	0	0
CNTNAP5	0	0	1	0	0	1
CNTROB	1	0	0	1	0	0
COASY	1	0	0	1	0	0
COG3	0	0	0	1	0	0
COG4	1	1	0	1	0	0
COG5	1	0	0	1	0	1
COG7	1	0	0	1	0	0
COG8	1	1	0	1	0	0
COL11A1	0	0	0	1	0	0
COL24A1	0	1	0	0	0	0
COL4A3BP	1	0	0	1	0	0
COL6A3	0	0	1	0	0	1
COL7A1	0	0	1	0	0	0
COL9A1	0	0	1	0	0	1
COLQ	0	0	1	0	0	1
COMMD10	0	0	0	1	0	0
COMMD3	1	0	0	0	0	0
COMMD9	1	0	0	1	0	0
COMT	0	0	1	0	0	1
COMTD1	1	1	0	1	0	0
COPA	1	0	0	1	1	1
COPB2	1	0	0	1	0	0
COPE	1	0	0	1	0	0
COPG	1	0	0	1	0	0
COPS2	1	1	0	1	0	0
COPS5	1	0	0	1	0	0
COPS6	1	0	0	1	0	0
COPS7A	1	0	0	1	1	0
COPS7B	1	0	0	1	0	1
COPS8	1	0	0	1	0	0
COPZ1	1	0	0	0	0	0

COQ10A	0	0	0	1	0	0
COQ10B	1	0	0	0	0	0
COQ2	1	0	0	1	0	0
COQ3	1	0	0	0	0	0
COQ4	1	0	0	1	0	0
COQ5	1	0	0	1	0	0
COQ9	1	0	0	1	0	0
CORO1B	0	0	1	0	1	0
CORO1C	0	0	0	0	1	0
CORO2A	0	0	1	1	0	1
CORO7	1	0	0	0	0	0
CORT	0	0	1	0	0	0
COX10	1	0	0	1	0	0
COX11	1	0	0	1	0	0
COX15	1	0	1	1	0	1
COX17	0	0	0	1	0	0
COX4I1	1	0	0	1	0	0
COX4NB	1	0	0	1	0	0
COX5B	0	0	0	1	0	0
COX6A1	1	0	0	1	0	0
COX6B1	1	0	0	0	1	0
COX6C	1	0	0	0	0	0
COX7A2	1	0	0	1	0	0
COX7B	1	1	0	1	1	0
COX7C	0	0	0	0	1	0
CPB1	0	1	1	0	0	1
CPD	0	0	0	1	0	0
CPEB1	0	1	1	0	0	1
CPEB3	1	0	0	1	0	0
CPEB4	1	0	0	0	0	0
CPLX2	0	0	1	0	0	1
CPLX3	0	0	1	0	0	1
CPNE1	1	0	1	1	0	1
CPNE2	0	0	0	0	1	0
CPNE3	1	0	0	0	0	0
CPNE6	0	0	1	0	0	1
CPOX	0	0	0	1	0	0
CPSF1	1	0	0	1	0	0
CPSF2	1	0	0	0	0	0
CPSF3	1	0	0	1	1	0
CPSF3L	0	0	0	1	0	1
CPSF4	1	0	0	0	0	0
CPT1A	1	1	1	0	0	1
CPT2	0	1	0	1	0	0
CPXM2	0	0	1	0	0	1
CPZ	0	0	1	0	0	0
CRADD	0	0	0	0	1	0
CRAT	1	1	0	0	0	0
CRB2	0	0	1	0	0	1
CREB1	0	0	0	1	0	0
CREB3	1	0	0	1	0	0
CREB3L3	0	1	0	0	0	0
CREB3L4	1	0	0	1	0	0
CREBL1	1	1	1	1	0	1
CREG1	1	0	0	1	1	0
CRELD2	1	0	0	1	0	0
CREM	1	1	1	1	0	1
CRHBP	0	0	0	1	0	0
CRIM1	0	0	0	1	0	0

CRIP3	0	1	0	0	0	0
CRIPT	1	0	0	1	1	0
CRISP3	0	0	0	1	0	0
CRISPLD2	0	1	0	0	0	0
CRKRS	1	0	0	1	0	0
CRLF1	0	0	0	0	0	1
CRLF3	0	0	0	1	0	0
CRLS1	1	0	0	1	0	0
CRNKL1	1	0	1	1	0	0
CROCC	0	0	1	0	0	0
CROP	1	0	0	1	0	0
CROT	1	0	0	1	0	1
CRSP2	1	0	0	1	0	0
CRSP3	0	0	1	1	0	1
CRSP6	1	0	0	1	0	0
CRSP8	0	0	1	0	0	0
CRTAM	0	0	1	0	0	1
CRTAP	1	0	0	0	0	0
CRTC2	1	0	0	1	0	0
CRX	0	0	1	0	0	1
CRY1	1	1	0	1	0	0
CRY2	1	0	0	0	0	1
CRYAA	0	0	0	0	0	1
CRYBB2	0	1	1	0	0	1
CRYBB3	0	1	1	0	1	1
CRYGS	0	0	1	1	0	0
CRYZ	1	0	1	1	0	1
CS	1	0	0	1	0	0
CSAD	1	0	0	1	0	0
CSDA	1	0	0	0	0	0
CSF1	1	0	0	1	0	0
CSF2RA	1	0	1	1	0	0
CSF2RB	1	0	0	0	1	0
CSGLCA-T	1	0	0	0	0	0
CSHL1	0	1	1	0	0	1
CSK	1	0	0	0	1	0
CSMD1	0	0	1	0	0	1
CSNK1A1	1	0	1	1	0	1
CSNK1A1L	0	0	1	0	0	1
CSNK1G1	1	0	1	1	0	0
CSNK2A1	0	0	0	1	0	0
CSNK2A2	0	0	1	0	0	1
CSNK2B	1	0	0	1	1	0
CSPG5	1	0	0	0	0	0
CSPP1	1	0	0	1	0	0
CSRP1	1	0	1	1	0	0
CSRP2	0	0	0	1	0	0
CST2	0	1	1	0	0	0
CST6	0	0	0	1	0	0
CSTA	0	0	0	0	1	0
CSTB	1	0	0	0	0	1
CSTF1	1	0	0	1	1	0
CSTF2	0	1	1	1	1	1
CSTF2T	1	1	0	0	0	0
CSTF3	0	0	0	1	0	0
CT45-1	0	1	1	0	0	1
CTAGE5	1	0	0	1	0	1
CTBP1	0	0	1	0	0	0
CTBP2	1	0	1	0	1	1

CTBS	1	0	0	1	0	0
CTCF	1	1	0	0	1	0
CTDP1	1	0	0	1	0	0
CTDSP2	1	0	1	1	0	1
CTDSPL2	0	0	0	0	1	0
CTLA4	1	0	1	0	0	1
CTNNA1	0	0	1	0	0	1
CTNNAL1	0	1	0	0	0	0
CTNNB1	1	0	0	0	0	0
CTNS	1	0	0	1	0	1
CTPS	0	0	0	1	0	0
CTPS2	1	0	1	1	0	0
CTRB2	0	0	1	0	0	1
CTRC	0	0	1	0	0	0
CTRL	1	0	0	1	1	0
CTSB	1	0	0	1	0	0
CTSC	1	0	0	0	0	0
CTSD	1	0	0	1	0	0
CTSK	0	0	0	0	0	1
CTSO	0	0	0	1	0	0
CTSW	1	0	0	0	1	0
CTSZ	0	0	0	1	0	0
CTTN	1	0	0	0	0	0
CTTNBP2NL	1	0	0	0	0	0
CTXN1	0	0	1	0	0	1
CUEDC1	0	0	0	0	1	0
CUGBP1	1	0	1	1	0	1
CUGBP2	0	0	1	1	1	1
CUL2	0	0	0	1	1	0
CUL4A	1	0	0	1	0	0
CUL4B	0	1	1	0	0	1
CUL7	1	0	0	1	0	0
CUTA	1	0	0	0	0	0
CUTC	1	0	1	1	0	1
CUZD1	1	0	1	0	0	1
CWF19L1	1	0	0	0	0	0
CWF19L2	1	1	0	1	0	0
CX3CL1	0	0	1	0	0	1
CX3CR1	0	1	0	0	0	0
CXCL1	0	1	0	0	0	0
CXCL10	0	1	0	0	1	0
CXCL11	0	1	0	0	0	0
CXCL2	0	0	0	1	0	0
CXCL3	1	0	0	1	0	0
CXCL9	0	0	1	0	0	1
CXCR4	1	1	0	0	0	0
CXXC1	1	0	0	1	0	0
CXXC5	1	0	0	1	0	0
CXORF23	0	1	1	1	0	1
CXORF26	1	0	0	0	0	1
CXORF34	0	1	0	0	1	0
CXORF38	0	0	0	1	0	0
CXORF39	0	1	1	0	0	1
CXORF40A	0	1	1	1	1	1
CXORF45	1	1	1	1	0	1
CXORF48	0	1	1	0	0	1
CXORF6	0	1	1	1	0	1
CXORF9	0	1	0	0	1	0
CYB561	1	0	0	1	0	0

CYB561D1	1	0	0	1	0	0
CYB561D2	1	0	0	1	1	0
CYB5B	1	0	0	1	0	0
CYB5D1	1	0	0	1	0	0
CYB5D2	1	0	0	1	0	0
CYB5R1	1	0	0	1	0	0
CYBASC3	1	0	0	1	0	1
CYFIP1	0	0	1	0	0	1
CYFIP2	0	1	1	1	1	1
CYP11A1	0	0	0	0	0	1
CYP19A1	0	0	1	1	0	0
CYP1B1	1	0	0	0	0	0
CYP20A1	1	0	0	1	0	0
CYP27A1	1	0	0	1	0	1
CYP27B1	1	0	1	0	0	1
CYP2A13	0	0	1	0	0	1
CYP2D6	0	0	1	0	0	1
CYP3A5	0	0	1	0	0	1
CYP4A11	0	1	0	0	0	0
CYP4V2	1	0	0	1	0	0
CYP4X1	0	0	1	0	0	0
CYP51A1	1	0	0	1	0	0
CYSLTR1	0	1	0	0	1	0
CYTL1	0	0	0	1	0	0
CYYR1	1	1	0	1	0	0
D15WSU75E	1	0	0	1	0	0
DAAM1	0	0	0	1	0	0
DAB2	1	0	0	1	0	0
DACH1	0	1	0	0	0	0
DACT1	0	0	1	0	0	0
DAD1	1	0	0	1	1	0
DAK	1	0	0	0	1	0
DAO	0	0	0	0	0	1
DAP	0	0	0	0	1	0
DAP3	1	0	0	0	0	0
DAPK2	0	1	0	0	0	0
DAPK3	1	0	0	0	1	0
DAPP1	0	0	0	0	1	0
DARC	0	0	1	0	0	1
DARS	0	0	0	1	0	0
DARS2	0	0	1	0	0	0
DAXX	1	0	0	1	1	0
DAZAP2	0	1	0	0	1	0
DBI	1	0	0	1	1	1
DBNDD2	1	0	0	1	0	0
DBNL	1	0	0	1	0	0
DBR1	1	0	0	1	0	0
DBT	0	0	0	1	1	0
DC2	1	0	0	1	0	0
DCAKD	1	0	0	1	1	0
DCD	0	0	1	0	0	1
DCHS2	0	1	1	0	0	1
DCI	1	0	0	1	0	0
DCK	1	0	0	1	0	0
DCLRE1A	1	0	0	1	0	0
DCLRE1C	0	0	0	1	0	0
DCP2	1	0	0	1	0	0
DCTN1	1	1	1	0	1	1
DCTN2	0	0	0	1	0	0

DCTN5	1	0	0	1	0	1
DCTN6	1	0	0	0	1	0
DCUN1D2	1	0	1	1	0	1
DCUN1D3	1	0	0	1	0	1
DCX	1	1	1	0	0	1
DDAH2	1	0	0	0	0	0
DDB1	1	0	0	0	1	0
DDB2	1	0	0	0	0	0
DDC	0	0	1	0	0	1
DDEF1	0	0	1	0	0	1
DDEF2	1	0	0	0	0	0
DDHD1	0	0	0	0	0	1
DDI2	1	0	0	1	0	0
DDIT3	1	0	0	0	1	0
DDO	0	0	1	0	1	1
DDOST	0	0	0	1	1	0
DDX1	1	0	0	1	0	0
DDX17	1	0	1	0	0	0
DDX18	1	0	0	1	0	0
DDX19-DDX19L	0	0	0	1	1	0
DDX19A	0	0	0	0	1	0
DDX19B	0	0	0	1	0	0
DDX20	1	0	0	1	0	0
DDX21	1	0	0	1	0	0
DDX23	1	0	0	1	0	0
DDX24	1	0	0	0	0	0
DDX28	1	0	0	1	0	0
DDX31	0	0	0	1	0	0
DDX39	1	0	0	1	0	0
DDX3X	1	0	0	0	0	0
DDX41	1	0	0	0	0	0
DDX42	1	0	0	1	1	0
DDX43	0	0	0	0	0	1
DDX46	1	0	0	1	0	0
DDX49	1	0	0	1	0	0
DDX5	1	0	0	1	0	0
DDX50	1	0	0	1	0	0
DDX51	1	0	0	1	0	0
DDX52	1	0	0	1	0	0
DDX54	1	0	0	0	1	0
DDX55	1	0	0	0	0	0
DDX56	0	0	0	1	0	0
DDX58	1	0	0	0	0	0
DDX59	1	0	0	1	1	0
DEADC1	0	0	1	1	0	1
DEAF1	0	0	1	0	0	1
DEDD	1	0	0	1	0	0
DEDD2	1	0	0	1	1	0
DEFB108B	0	0	1	0	0	1
DEFB121	0	0	1	0	0	0
DEFB123	0	1	1	0	0	1
DEFB125	0	1	1	1	0	1
DEFB126	0	0	1	0	0	1
DEFB128	0	1	1	1	0	1
DENND1C	1	0	0	0	0	0
DENND2D	1	0	0	1	0	0
DENND3	0	0	0	1	0	0
DENND4C	0	0	1	0	0	0
DEPDC1	0	1	0	0	0	1

DEPDC6	1	0	0	1	0	0
DEPDC7	0	1	0	0	0	0
DERL1	1	0	0	1	0	0
DERL2	1	0	0	1	0	0
DEXI	0	0	0	1	0	0
DFFA	0	0	0	1	0	0
DFFB	1	0	0	1	0	0
DFNA5	0	0	0	1	0	0
DGAT2L3	0	1	1	1	0	1
DGCR14	1	0	1	1	0	1
DGKB	0	0	0	0	0	1
DGKG	0	1	0	0	1	0
DGKZ	0	1	0	0	1	0
DGUOK	1	0	0	0	0	0
DHCR7	1	1	0	1	0	1
DHDDS	1	0	0	1	0	0
DHDH	1	0	1	1	0	1
DHFR	1	0	0	1	1	0
DHFRL1	1	0	0	1	0	0
DHODH	1	0	0	0	0	0
DHPS	1	0	0	1	0	0
DHRS1	1	1	0	1	1	0
DHRS2	0	0	1	0	0	0
DHRS3	1	0	0	0	0	0
DHRS4L2	0	0	0	1	0	0
DHRS7	1	0	0	1	0	0
DHRS7B	1	0	0	0	0	0
DHRS9	0	0	1	1	0	1
DHX16	1	0	0	0	0	0
DHX29	1	0	0	1	0	1
DHX32	1	0	1	0	0	1
DHX33	1	0	0	1	0	0
DHX35	1	0	0	1	0	0
DHX36	0	1	0	0	0	0
DHX38	1	0	0	1	0	0
DHX57	0	0	0	0	0	1
DHX8	1	0	0	1	0	0
DHX9	1	0	1	1	0	1
DIAPH1	1	0	1	1	0	0
DIAPH3	0	0	1	0	0	0
DICER1	1	0	0	0	0	0
DIDO1	1	1	0	1	1	0
DIO1	1	0	1	1	0	1
DIP	0	1	1	0	0	1
DIRAS2	0	0	1	0	0	1
DIXDC1	1	1	0	1	0	0
DKC1	0	1	0	1	0	0
DKFZP434B0335	1	0	0	0	0	0
DKFZP564J0863	1	0	0	1	0	0
DKFZP564O0523	1	1	0	1	0	0
DKFZP586P0123	1	1	0	1	1	1
DKFZP434K1815	1	0	0	0	0	0
DKFZP434K191	0	0	1	0	0	1
DKFZP434N035	1	0	0	0	0	0
DKFZP451A211	0	0	1	0	1	1
DKFZP451M2119	1	0	0	1	0	1
DKFZP564N2472	0	0	1	0	0	1
DKFZP666G057	1	0	0	1	0	0
DKFZP686I15217	1	0	0	1	0	0

DKFZP686O24166	0	0	0	0	0	1
DKKL1	0	0	0	1	0	0
DLAT	1	0	0	1	0	0
DLEU7	0	1	1	0	1	0
DLG4	1	0	0	0	1	0
DLG5	0	0	1	0	1	1
DLGAP1	0	1	1	1	0	0
DLGAP4	1	1	1	0	0	1
DMAP1	0	0	0	1	0	0
DMRTC1	0	1	1	0	1	1
DMTF1	1	0	0	1	0	0
DMXL1	1	0	0	1	1	0
DMXL2	0	0	0	1	0	0
DNAH10	0	1	1	0	0	1
DNAH17	0	0	1	0	0	0
DNAH3	1	0	0	0	0	0
DNAH5	0	0	1	0	0	1
DNAI1	0	0	1	1	0	1
DNAI2	0	0	1	0	0	0
DNAJA1	0	0	0	1	0	0
DNAJA3	1	0	0	0	0	0
DNAJA5	1	0	0	0	0	0
DNAJB1	1	0	0	1	1	0
DNAJB11	1	0	0	1	0	0
DNAJB14	1	0	1	1	1	0
DNAJB5	0	0	1	0	0	0
DNAJB7	1	0	1	1	0	1
DNAJB9	1	0	1	1	0	1
DNAJC1	0	1	0	0	0	0
DNAJC10	1	0	0	0	0	0
DNAJC12	0	0	1	0	0	1
DNAJC13	1	0	0	0	0	0
DNAJC17	1	1	0	0	0	0
DNAJC19	1	1	0	0	0	0
DNAJC3	1	0	1	0	0	0
DNAJC5B	1	0	1	1	0	1
DNAJC7	1	0	0	1	0	0
DNAJC8	1	0	0	1	0	0
DNAJC9	1	1	0	1	0	0
DNAL4	0	0	1	0	0	0
DNASE1L1	1	1	0	0	0	0
DNASE1L3	0	0	1	0	0	1
DNASE2B	0	0	1	1	1	1
DNHD1	0	0	1	0	0	1
DNM2	1	0	0	1	0	0
DNMT1	1	0	0	0	0	0
DNTTIP1	1	0	1	1	1	0
DNTTIP2	1	0	0	0	0	0
DOCK1	0	0	1	0	0	1
DOCK10	0	0	1	0	0	1
DOCK11	0	1	0	0	0	0
DOCK3	1	0	0	1	0	0
DOCK4	1	0	0	1	0	0
DOCK5	1	0	0	0	0	0
DOCK7	1	0	1	1	0	0
DOCK8	0	0	0	1	0	0
DOCK9	1	0	0	0	0	0
DOK2	1	0	0	1	0	0
DOM3Z	1	0	0	1	0	0

DONSON	0	0	0	1	0	0
DOPEY1	1	0	0	0	0	0
DOPEY2	0	0	0	1	0	1
DPAGT1	1	0	0	1	0	0
DPCR1	0	0	1	0	0	1
DPH2	1	0	0	1	0	0
DPM1	1	0	0	1	0	1
DPM3	0	0	1	0	0	1
DPP3	1	0	0	1	0	0
DPP8	1	0	0	1	0	0
DPYSL2	1	0	0	1	0	0
DPYSL4	0	0	0	0	0	1
DR1	1	0	0	1	0	0
DRAP1	1	0	0	0	1	0
DRD3	0	1	1	0	0	1
DRG1	1	0	0	1	0	0
DRG2	1	0	1	1	0	1
DRP2	0	1	1	0	0	1
DSCR1	1	1	1	1	1	1
DSCR10	0	0	1	0	0	1
DSCR1L1	0	0	1	0	0	1
DSCR2	1	0	0	0	0	0
DSCR3	1	0	0	1	0	0
DSCR4	1	1	1	0	0	1
DST	0	0	1	0	1	1
DTL	1	0	0	1	1	0
DTNA	0	0	1	0	0	1
DTNBP1	0	0	0	0	1	0
DTWD1	1	0	0	0	1	0
DTWD2	1	0	0	1	0	1
DTX3L	0	1	0	0	0	0
DULLARD	1	0	0	1	1	0
DUOX1	1	0	0	0	0	0
DUS2L	1	0	0	1	1	0
DUS4L	1	0	0	1	0	1
DUSP10	0	0	0	1	0	1
DUSP11	1	0	0	0	1	0
DUSP12	1	0	0	1	0	1
DUSP13	0	0	1	1	0	1
DUSP18	0	0	0	1	0	0
DUSP23	1	0	0	1	1	0
DUSP5	1	0	0	0	0	0
DUT	1	0	0	1	0	0
DVL2	1	0	0	1	1	0
DVL3	1	0	0	1	0	0
DYNC1H1	1	0	0	0	0	0
DYNC1LI1	1	0	0	1	0	0
DYNC1LI2	0	0	0	1	0	0
DYNLL2	1	0	0	1	0	0
DYNLRB1	1	0	0	0	0	0
DYRK1A	1	0	0	0	0	1
DYRK1B	0	1	0	1	0	0
DYRK3	1	1	0	1	0	1
DYRK4	0	0	1	0	0	1
DYSF	0	0	0	0	1	0
DYX1C1	0	0	1	0	0	1
E2F4	1	0	0	0	0	0
E2F8	0	1	0	0	1	0
EAF1	1	0	0	1	1	1

EARS2	1	0	0	1	0	0
EBAG9	0	0	0	1	0	0
EBI2	1	0	0	1	0	0
EBI3	0	0	1	0	0	1
EBNA1BP2	1	0	0	1	0	0
EBP	0	1	0	0	1	0
ECD	0	0	0	1	0	0
ECE2	1	0	0	1	0	1
ECHDC3	0	0	0	1	0	0
ECHS1	0	0	0	1	0	0
ECM1	0	0	1	0	0	1
ECT2	1	0	0	0	0	0
EDEM1	1	0	0	1	0	0
EDEM2	0	1	0	0	0	0
EDEM3	0	0	0	1	1	0
EDG6	1	0	0	1	1	0
EDN1	0	1	0	0	0	0
EED	1	0	0	1	0	0
EEF1B2	1	0	0	1	0	0
EEF1D	1	0	0	1	0	0
EEF2	1	0	0	1	0	0
EEF2K	1	0	0	1	0	0
EFCAB2	1	0	0	1	0	0
EFHA1	1	0	0	1	0	0
EFHB	1	0	0	1	1	0
EFHD2	1	0	0	0	0	0
EFNA3	0	0	0	0	0	1
EFNB1	0	1	1	0	0	0
EFTUD1	1	0	0	1	0	0
EFTUD2	1	0	1	1	0	1
EGFR	0	0	1	0	0	0
EGLN1	0	0	0	1	0	0
EGR1	1	0	0	0	0	0
EGR2	1	0	0	1	0	0
EHBP1	1	1	0	1	1	1
EHD4	1	0	0	1	1	0
EHF	0	0	1	1	1	1
EHMT1	1	0	1	1	1	1
EID3	0	1	0	0	0	1
EIF1B	1	0	0	0	0	0
EIF2A	1	0	0	1	1	0
EIF2AK2	0	0	0	1	0	0
EIF2B1	1	0	0	1	1	0
EIF2B2	1	0	0	0	0	0
EIF2B3	1	0	0	0	0	0
EIF2B4	1	0	0	0	1	0
EIF2B5	1	0	0	1	0	0
EIF2C1	0	1	0	1	0	0
EIF2C3	1	0	0	1	0	0
EIF2S1	1	0	0	1	1	1
EIF4A1	1	0	0	1	1	1
EIF4B	1	0	0	1	0	1
EIF4E2	1	0	0	1	1	0
EIF4E3	0	1	0	0	0	0
EIF4EBP3	1	0	0	1	0	0
EIF4ENIF1	1	0	0	1	0	0
EIF4G1	1	0	0	1	0	1
EIF4G3	1	0	0	1	0	0
EIF5	1	0	0	1	0	0

EIF5B	1	0	0	1	0	0
ELA1	0	0	1	0	0	0
ELA2	0	1	0	0	0	0
ELA2A	0	0	1	0	0	1
ELAC1	1	1	0	0	0	0
ELAC2	1	0	0	1	0	0
ELF2	1	1	1	1	0	0
ELF3	0	0	1	0	0	0
ELF5	0	0	1	0	0	1
ELK1	0	1	1	0	0	0
ELK4	1	0	0	1	0	0
ELL2	0	0	1	0	0	0
ELMO1	1	1	1	0	1	1
ELMOD2	0	0	0	1	1	0
ELOF1	0	0	0	0	0	1
ELOVL3	1	0	0	0	0	0
ELOVL5	1	0	0	0	0	0
ELP3	1	0	0	1	0	0
ELP4	1	0	0	1	0	1
EME1	1	0	0	1	0	1
EME2	1	0	0	1	0	0
EMG1	1	0	0	1	0	0
EMILIN1	1	0	0	1	0	0
EMILIN2	0	0	0	1	0	0
EML3	1	0	0	0	0	0
EML4	1	0	0	0	0	1
EMP1	0	0	1	1	0	1
EMR1	0	0	0	0	1	0
EMR2	1	0	0	1	0	0
EMR3	0	0	0	1	0	0
ENC1	0	0	0	0	1	0
ENO1	1	0	0	0	1	0
ENPP4	0	1	0	0	0	0
ENTPD4	0	0	1	0	0	1
ENTPD5	1	0	0	1	0	0
ENTPD6	1	0	0	0	0	0
ENTPD7	0	0	0	1	0	1
ENTPD8	0	0	1	0	0	1
ENY2	1	0	0	1	0	0
EP400	1	0	0	1	0	0
EPAS1	0	0	0	1	0	0
EPB41L1	0	1	1	1	0	1
EPB41L2	1	0	0	1	1	1
EPB42	0	0	0	0	0	1
EPHX1	0	0	1	0	0	1
EPM2A	1	0	0	0	0	1
EPM2AIP1	1	0	0	1	0	0
EPN1	1	0	0	1	0	0
EPRS	1	0	0	1	0	0
EPS15	1	0	0	0	0	0
EPS8L3	0	1	1	0	0	1
EPSTI1	0	0	0	1	0	0
ERBB2	0	0	0	1	0	1
ERCC2	1	0	0	1	0	0
ERCC4	0	0	1	0	0	1
ERCC5	1	0	0	0	0	0
ERCC8	0	0	0	1	0	0
ERGIC1	0	0	1	0	0	1
ERGIC3	0	0	0	1	0	0

ERH	1	1	0	1	0	0
ERICH1	1	0	0	1	1	0
ERMAP	1	1	0	1	0	1
ERN1	1	0	0	0	0	0
ERO1L	0	1	0	0	0	0
ERO1LB	0	0	0	1	0	0
ERRFI1	1	0	0	1	0	0
ESD	0	1	0	0	0	0
ESPL1	1	1	0	1	1	0
ESR1	0	0	0	1	0	0
ESRRA	1	0	0	1	0	0
ETF1	1	0	0	1	0	0
ETFB	0	0	1	0	0	1
ETFDH	1	0	1	1	0	0
ETHE1	1	1	0	1	1	0
ETS1	1	0	0	0	0	0
ETS2	0	1	0	0	0	0
ETV2	1	0	0	0	1	0
ETV3	1	0	0	1	0	0
ETV5	1	0	0	0	0	0
EVA1	0	1	0	0	0	0
EVI5	0	0	0	0	0	1
EVL	1	0	1	1	0	1
EWSR1	1	0	0	1	0	0
EXDL2	0	0	1	0	0	1
EXO1	1	0	0	1	0	0
EXOC1	1	0	0	0	0	0
EXOC5	1	0	0	1	0	1
EXOC6	0	0	1	0	0	1
EXOC7	1	0	0	0	0	0
EXOC8	1	0	0	1	1	0
EXOSC1	1	0	0	1	0	0
EXOSC2	0	0	0	0	1	0
EXOSC3	1	0	0	1	0	0
EXOSC4	1	0	0	1	0	0
EXOSC5	1	0	0	1	1	0
EXOSC7	1	0	0	1	0	0
EXOSC9	1	0	0	1	0	0
EXTL1	0	0	1	0	0	1
EXTL2	1	0	0	1	0	1
EXTL3	0	0	0	0	0	1
EYA3	1	0	0	1	0	0
EZH1	1	0	0	0	0	0
EZH2	1	0	0	1	0	0
F11R	0	0	0	1	0	0
F12	0	1	0	0	0	0
F13A1	0	0	1	0	1	0
F5	0	1	0	0	0	0
F8	1	0	1	1	0	1
F8A1	1	0	0	1	1	0
F8A3	1	0	0	0	1	0
FA2H	0	0	0	0	0	1
FABP1	0	1	1	0	0	1
FABP2	0	1	1	1	0	1
FABP3	1	0	0	0	0	1
FABP4	1	0	1	0	0	1
FABP6	0	1	1	0	0	1
FABP7	0	0	1	1	0	1
FADD	0	0	0	1	0	0

FADS1	1	0	0	1	0	0
FADS2	1	0	0	1	0	0
FAF1	1	0	0	0	0	0
FAH	0	0	0	1	0	0
FAHD1	1	0	1	1	0	1
FAHD2A	0	1	0	1	0	0
FAIM3	0	0	1	0	0	1
FAM101A	0	1	1	0	0	1
FAM102A	0	1	1	0	1	1
FAM102B	0	0	0	1	0	0
FAM103A1	1	0	0	1	0	0
FAM104A	1	0	0	1	0	0
FAM105A	1	1	0	0	0	0
FAM105B	0	0	1	1	0	0
FAM107A	0	0	1	0	0	1
FAM107B	1	1	1	0	1	1
FAM108A1	1	0	0	0	0	0
FAM109B	1	1	0	0	0	0
FAM111A	1	0	0	1	0	0
FAM112B	0	0	1	0	0	1
FAM113A	0	0	0	1	0	0
FAM113B	0	0	0	0	0	1
FAM116B	0	0	1	0	0	1
FAM14A	1	0	0	0	0	0
FAM14B	1	0	0	0	0	0
FAM18B	0	0	0	1	0	0
FAM19A3	1	0	1	1	0	1
FAM20A	1	0	0	0	0	0
FAM21C	0	1	0	0	1	0
FAM24B	1	0	1	1	0	1
FAM32A	1	0	0	1	0	0
FAM33A	1	0	0	1	0	0
FAM35A	1	0	0	1	0	0
FAM38A	0	0	1	0	0	0
FAM3A	0	1	0	0	0	0
FAM3C	1	0	0	1	0	0
FAM40A	1	0	0	1	0	0
FAM43A	0	0	0	1	0	0
FAM44A	1	0	0	1	0	0
FAM44B	1	0	0	1	0	1
FAM45A	1	0	0	1	0	0
FAM45B	1	1	1	1	0	1
FAM48A	0	0	0	1	0	0
FAM49A	0	1	0	0	0	0
FAM50A	0	1	0	0	0	0
FAM53B	1	0	0	1	0	0
FAM54A	0	0	0	1	0	0
FAM58A	0	0	1	0	0	1
FAM62A	0	0	0	1	0	0
FAM63B	1	0	0	1	0	0
FAM65A	1	0	0	1	0	0
FAM70B	0	0	0	1	0	0
FAM71C	1	0	1	0	0	1
FAM72A	0	0	0	0	1	0
FAM73A	0	0	0	1	0	0
FAM76B	1	0	0	0	0	0
FAM79A	1	0	0	0	0	0
FAM79B	0	0	1	0	0	1
FAM80B	0	0	1	0	0	0

FAM82A	0	0	1	0	0	1
FAM82B	1	0	0	0	0	0
FAM82C	0	0	0	1	0	0
FAM83C	0	1	1	0	0	0
FAM83D	1	0	1	0	0	0
FAM84A	0	0	1	0	0	0
FAM89B	1	0	0	1	0	0
FAM8A1	0	0	1	0	0	0
FAM96A	1	0	0	1	0	0
FAM96B	1	0	0	1	1	0
FAM98C	0	0	0	1	0	0
FANCA	1	0	0	0	0	0
FANCB	1	1	0	0	1	0
FANCE	1	0	0	1	0	0
FANCG	0	0	0	1	0	0
FANK1	1	0	0	0	0	1
FARP2	1	0	0	0	0	0
FARS2	1	0	0	1	0	0
FARSLB	1	0	0	1	0	0
FASN	0	0	0	0	1	0
FASTK	1	0	0	0	0	0
FAT2	0	1	1	0	0	1
FAT4	0	1	0	0	0	0
FAU	1	0	0	1	1	0
FBL	0	1	0	1	0	0
FBP1	1	0	0	1	0	0
FBS1	1	0	0	1	1	0
FBXL14	1	0	0	0	0	0
FBXL16	0	0	0	1	0	0
FBXL17	0	1	0	0	0	0
FBXL19	0	0	0	0	0	1
FBXL22	0	0	1	0	1	1
FBXL3	1	0	0	1	0	0
FBXL6	1	0	0	1	0	0
FBXL8	1	0	0	1	0	0
FBXO15	1	0	0	1	1	1
FBXO22	1	0	0	1	0	0
FBXO27	0	0	0	1	0	0
FBXO28	1	0	0	1	0	0
FBXO30	0	1	0	0	0	1
FBXO31	1	0	0	0	0	0
FBXO32	0	1	1	0	0	1
FBXO34	0	1	0	0	0	0
FBXO38	1	0	1	1	0	1
FBXO4	1	0	0	1	0	0
FBXO43	0	0	0	1	0	0
FBXO5	1	0	0	1	1	0
FBXO7	1	0	0	0	0	0
FBXO8	1	0	0	1	0	0
FBXO9	1	0	0	1	0	0
FBXW11	0	1	0	0	0	0
FBXW2	0	0	0	1	0	0
FBXW4	0	0	0	1	0	0
FBXW7	1	0	1	1	1	1
FCER1G	1	0	0	1	0	0
FCER2	0	0	1	0	0	0
FCGR2A	0	0	0	0	0	1
FCHO2	1	0	1	1	0	1
FCN1	0	1	0	0	1	0

FCN3	1	1	0	0	0	1
FCRL1	1	0	1	0	0	0
FCRL3	0	0	1	0	0	1
FCRL5	0	0	0	0	0	1
FCRL6	0	0	1	0	0	1
FDFT1	1	0	1	0	0	1
FDXR	1	0	1	0	0	0
FEM1B	1	0	0	1	0	0
FEM1C	0	0	1	0	0	0
FEN1	1	0	0	1	1	0
FER	0	0	0	1	0	0
FER1L3	1	0	0	1	0	0
FES	1	0	0	1	0	0
FFAR1	0	0	1	0	0	1
FFAR2	0	0	0	0	1	0
FGA	0	0	1	0	0	1
FGD4	1	0	0	0	0	1
FGD5	0	1	1	0	0	1
FGD6	1	0	0	0	0	0
FGF11	1	0	0	1	0	0
FGF12	0	0	1	0	0	1
FGF13	0	1	1	1	0	1
FGF6	0	0	1	0	0	1
FGFR1OP2	1	0	0	1	0	0
FGG	0	1	1	0	0	1
FHIT	0	1	0	0	1	0
FHL1	0	0	1	0	0	0
FHL5	0	1	1	0	0	1
FIBP	0	0	0	0	1	0
FIGN	0	0	1	1	0	0
FIGNL1	0	0	0	1	0	0
FIP1L1	0	0	0	0	1	0
FIS	0	0	1	0	0	0
FIS1	1	0	0	1	0	0
FJX1	0	0	0	1	0	0
FKBP14	1	0	0	0	0	0
FKBP1A	0	0	0	1	0	0
FKBP3	1	0	0	1	0	0
FKBP6	1	1	1	1	0	1
FKBP9	1	0	0	0	0	0
FKBPL	1	0	0	1	0	0
FKRP	0	0	0	0	1	0
FKSG44	0	0	1	1	0	0
FKSG83	0	0	1	1	0	1
FLAD1	1	0	0	1	0	0
FLJ10081	0	1	0	1	0	0
FLJ10154	0	0	0	0	0	1
FLJ10213	1	0	1	0	0	1
FLJ10241	1	0	0	1	0	0
FLJ10324	0	0	1	0	0	0
FLJ10803	1	0	0	1	0	0
FLJ10986	1	0	1	1	1	0
FLJ11151	0	0	0	0	0	1
FLJ11184	1	0	0	0	0	0
FLJ11506	0	1	1	0	0	0
FLJ11783	0	1	0	0	0	0
FLJ12716	1	0	0	0	0	0
FLJ13611	1	0	0	1	0	0
FLJ14107	0	0	1	0	0	1

FLJ14803	0	0	0	1	0	1
FLJ16478	1	0	0	0	0	0
FLJ20035	1	0	0	0	0	0
FLJ20054	0	0	1	0	0	0
FLJ20160	0	0	1	1	0	0
FLJ20273	1	0	1	0	1	1
FLJ20294	1	1	0	1	0	1
FLJ20309	1	0	0	0	0	1
FLJ20323	0	0	0	1	0	0
FLJ20489	0	0	0	1	0	0
FLJ20581	0	0	1	0	0	1
FLJ20674	1	0	1	0	0	1
FLJ20699	1	0	0	0	1	0
FLJ20850	1	0	0	0	0	1
FLJ21687	1	1	1	1	0	1
FLJ21865	0	0	0	1	0	0
FLJ21963	1	0	0	0	0	0
FLJ21986	1	0	0	0	0	0
FLJ22222	1	0	0	1	0	0
FLJ22639	1	0	0	0	0	0
FLJ25006	0	0	1	0	0	1
FLJ25715	1	0	0	1	0	0
FLJ25758	0	0	1	0	1	1
FLJ25791	0	0	0	0	0	1
FLJ26443	0	0	1	1	0	1
FLJ27255	0	1	1	0	0	1
FLJ30679	1	0	0	1	0	0
FLJ31438	1	0	0	1	0	0
FLJ32679	0	0	1	0	0	1
FLJ33590	1	0	1	0	0	1
FLJ34870	1	1	1	1	0	1
FLJ34931	0	0	1	1	0	1
FLJ35740	0	1	1	0	0	1
FLJ35767	0	1	1	0	0	1
FLJ35773	0	0	1	0	0	1
FLJ35801	1	0	0	1	0	0
FLJ36031	1	0	0	1	0	0
FLJ36144	1	0	1	0	0	0
FLJ36208	0	0	1	0	0	0
FLJ36492	1	0	0	0	0	0
FLJ36701	0	0	1	0	1	0
FLJ36874	1	0	0	1	0	1
FLJ37396	1	0	0	0	0	0
FLJ37464	0	0	1	0	0	0
FLJ37543	0	1	1	0	1	1
FLJ38377	0	0	1	0	0	0
FLJ38482	1	1	0	0	1	0
FLJ38973	0	0	0	1	0	0
FLJ39653	1	0	0	0	0	1
FLJ39779	1	0	0	1	0	0
FLJ39822	0	0	1	0	0	0
FLJ40142	0	0	1	1	0	0
FLJ40288	0	1	1	0	0	1
FLJ40852	1	0	0	1	0	0
FLJ41327	0	0	1	1	0	1
FLJ41423	0	0	1	0	0	1
FLJ42133	0	1	1	0	0	1
FLJ42957	0	1	0	0	1	0
FLJ43879	0	0	1	0	0	1

FLJ43980	1	0	1	0	0	1
FLJ44186	1	0	0	0	0	1
FLJ44385	0	1	1	1	0	1
FLJ44635	0	0	1	0	0	1
FLJ45055	1	0	0	1	0	0
FLJ45202	0	0	1	0	0	1
FLJ45337	0	1	1	0	0	1
FLJ45909	0	0	0	0	0	1
FLJ46082	1	0	0	1	0	0
FLJ46154	1	0	0	1	0	0
FLJ46347	0	0	0	1	0	0
FLJ46481	0	1	1	0	0	1
FLJ90709	0	0	0	0	1	0
FLNB	1	0	0	0	0	0
FLOT1	1	0	0	0	0	0
FLOT2	1	1	0	1	0	0
FLRT2	0	0	0	1	0	0
FLT3	0	1	0	0	0	0
FLYWCH1	0	1	0	0	0	0
FMNL2	0	0	0	1	0	0
FMNL3	1	0	1	1	0	1
FMO1	1	0	1	1	0	1
FMO2	0	0	1	0	0	1
FMO4	1	0	0	1	0	0
FNBP1L	0	0	1	0	0	1
FNDC3B	0	0	1	0	0	1
FNDC8	1	0	1	0	0	0
FNTA	1	0	0	0	0	0
FNTB	1	0	0	1	0	0
FOLR1	0	1	1	0	1	1
FOS	1	0	0	1	0	0
FOSB	1	0	0	1	0	0
FOXD2	1	0	0	0	0	0
FOXI1	0	1	1	0	0	1
FOXJ2	1	0	0	1	0	0
FOXJ3	1	0	0	1	1	0
FOXM1	1	0	0	0	0	0
FOXP3	0	1	1	0	1	0
FOXQ1	1	0	0	0	0	0
FOXRED1	1	0	0	1	0	1
FPGS	1	0	0	0	0	0
FPGT	1	0	0	1	0	0
FPR1	0	0	0	1	0	0
FPRL1	0	1	0	0	1	0
FPRL2	1	0	1	0	0	1
FRAP1	1	0	1	1	0	1
FRAT2	1	0	0	0	0	0
FRG1	1	0	0	1	0	0
FRMD3	1	0	0	0	0	0
FRMD4A	1	0	1	0	0	0
FRS2	1	0	0	1	0	0
FRS3	1	0	0	0	0	0
FSD1L	1	0	0	0	0	1
FSHR	0	1	1	0	0	0
FTH1	1	0	0	1	0	0
FTSJ1	0	1	1	0	0	1
FTSJ2	1	0	0	1	0	0
FTSJ3	1	0	0	1	0	0
FUBP1	1	0	0	1	0	0

FUK	1	0	0	0	0	0
FUNDC2	0	0	1	0	0	1
FURIN	1	1	1	1	0	1
FUT11	1	0	0	1	1	0
FUT3	0	1	1	0	0	1
FUT4	1	0	0	0	0	0
FUT6	0	0	1	0	0	1
FUT7	0	0	0	0	1	0
FVT1	1	0	0	0	0	0
FXC1	1	0	1	1	0	1
FXR1	0	0	0	1	0	0
FXR2	1	1	0	0	0	0
FXYD1	1	0	0	0	0	1
FXYD3	0	0	1	0	0	1
FYN	0	1	1	1	1	1
FYTTD1	0	0	0	1	0	1
G0S2	0	1	0	0	0	0
G3BP2	0	0	0	0	1	0
G6PC3	1	0	0	1	0	1
G6PD	1	1	0	1	0	0
GAB2	0	1	1	0	0	1
GAB3	0	1	1	0	0	0
GABARAP	1	0	0	1	1	0
GABPA	1	0	0	1	0	0
GABPB2	1	0	0	1	1	1
GABRA3	0	1	1	1	0	1
GABRP	0	0	1	0	0	1
GALC	1	0	1	1	0	0
GALE	0	0	0	1	1	0
GALK2	1	1	0	1	1	0
GALM	1	0	0	1	0	0
GALNAC4S-6ST	0	0	1	0	0	1
GALNACT-2	1	0	0	1	0	0
GALNS	1	0	0	1	0	0
GALNT1	0	0	1	0	0	1
GALNT13	0	0	1	0	0	0
GALNT3	0	0	1	0	0	0
GALNT6	0	1	1	0	1	1
GALT	0	1	1	0	0	1
GAN	0	1	0	0	1	0
GANAB	1	0	1	1	0	0
GANC	1	0	1	1	0	1
GARNL3	0	1	1	0	0	1
GARS	0	0	0	1	0	0
GART	1	0	0	1	0	0
GAS6	1	0	0	0	0	0
GATAD1	1	0	0	1	0	0
GATAD2A	1	0	0	1	1	0
GBA	1	0	0	1	0	0
GBA2	1	0	0	1	0	0
GBE1	0	0	0	0	1	0
GBF1	0	0	0	1	0	0
GBP1	1	0	0	0	0	0
GCA	1	0	0	1	0	0
GCAT	1	0	0	0	0	0
GCC1	1	0	0	0	0	0
GCDH	1	0	0	0	1	0
GCET2	0	0	1	0	0	1
GCLC	1	0	0	0	0	0

GCLM	0	1	0	0	0	0
GCN5L2	0	0	0	0	0	1
GCNT2	1	0	1	1	0	1
GCNT3	0	0	1	0	0	0
GCNT4	0	1	1	0	0	1
GCS1	1	0	0	1	0	0
GDAP2	1	0	0	1	0	0
GDF15	0	0	1	0	0	0
GDF2	0	0	1	0	0	1
GDF3	0	1	0	0	0	0
GDF5	0	0	0	1	0	1
GDF9	0	0	0	1	0	0
GDI1	1	1	0	1	1	0
GDPD2	0	1	1	0	0	1
GDPD4	0	0	1	0	0	1
GEMIN4	1	0	0	0	0	0
GEMIN5	1	0	0	1	0	0
GEMIN6	0	1	0	0	0	0
GFM1	0	0	0	1	0	0
GFM2	1	1	0	1	0	0
GFOD1	1	0	0	0	0	0
GFOD2	1	0	0	1	0	0
GFPT1	1	0	0	0	0	0
GFRA2	0	1	1	0	0	1
GGA2	0	0	0	0	1	0
GGA3	1	0	0	1	1	0
GGCX	1	0	0	0	0	0
GGT1	0	1	1	0	0	1
GGTL3	1	1	0	1	0	1
GGTLA1	0	0	1	0	0	1
GGTLA4	0	0	1	0	0	1
GHRH	0	0	0	0	0	1
GHRHR	0	0	1	0	0	1
GHRL	1	0	1	1	0	0
GIF	0	0	1	0	1	1
GIMAP2	1	0	0	1	1	0
GIMAP8	0	1	0	0	1	0
GIOT-1	1	0	0	1	0	0
GIP	0	1	1	0	0	1
GIPC1	0	0	0	0	0	1
GIT2	1	0	0	1	0	0
GIYD2	1	0	0	1	0	0
GJA4	0	0	1	0	0	0
GJA5	0	1	1	0	0	1
GJB7	0	0	1	0	0	0
GK	1	0	0	1	0	0
GLA	1	1	0	1	0	0
GLB1	1	0	0	0	0	0
GLB1L	1	1	0	1	0	0
GLDN	0	1	1	0	0	1
GLE1L	0	0	0	1	0	0
GLG1	0	0	0	1	0	0
GLI4	0	0	0	1	0	1
GLIPR1	1	0	0	1	0	0
GLIS1	0	0	1	1	0	0
GLRX2	0	0	1	1	0	0
GLRX5	1	1	0	1	0	0
GLT1D1	0	1	0	0	0	0
GLT25D1	0	0	0	0	1	0

GLT8D1	1	0	0	1	1	0
GLTP	0	1	0	1	0	0
GLTSCR1	1	0	1	0	0	0
GLUD1	1	0	0	1	0	0
GLUL	1	0	0	1	0	0
GLYAT	0	1	1	0	1	0
GLYCTK	1	0	0	1	0	0
GM2A	1	0	0	0	0	0
GMCL1	1	0	0	1	0	0
GMEB1	1	0	0	1	0	0
GMEB2	1	0	0	0	0	0
GMFB	0	0	0	1	1	1
GMFG	0	0	0	0	1	0
GMIP	0	0	0	1	0	0
GMPPA	1	0	0	1	0	0
GMPPB	1	0	0	1	0	0
GMPR2	1	0	0	1	0	0
GNA13	1	0	0	1	0	0
GNAI2	0	0	0	0	1	0
GNAI3	1	0	0	1	0	0
GNAQ	1	0	0	0	0	0
GNAS	0	0	1	0	0	0
GNB1	0	0	0	1	0	0
GNB1L	1	0	0	1	0	0
GNB4	1	0	0	1	0	0
GNB5	0	1	1	0	0	0
GNE	0	0	0	1	0	0
GNG10	1	0	0	0	0	0
GNG2	0	0	1	0	0	0
GNG5	1	0	0	1	0	0
GNG7	0	0	1	0	0	0
GNGT2	1	0	0	0	0	0
GNL1	1	0	0	1	0	0
GNL2	1	0	0	1	0	0
GNL3	1	0	1	1	0	1
GNL3L	0	1	1	1	1	0
GNPAT	1	0	0	1	0	0
GNPDA1	1	0	0	1	0	1
GNPDA2	0	0	0	1	0	0
GNPTG	1	0	0	1	1	0
GNRH1	0	0	1	0	0	0
GNS	1	0	0	0	0	0
GOLGA1	0	0	0	1	0	0
GOLGA2	1	0	0	0	1	0
GOLGA3	1	0	0	0	1	0
GOLGA4	1	0	0	1	0	0
GOLGA5	1	0	0	0	1	1
GOLGA8A	0	0	1	0	0	1
GOLGA8B	0	0	1	0	0	1
GOLGA8E	0	0	1	0	0	1
GOLGB1	1	0	0	1	0	0
GOLPH2	1	0	0	0	0	0
GOLPH4	1	0	0	1	0	0
GOLT1B	1	0	1	1	0	1
GON4L	1	0	0	1	1	0
GOPC	1	0	0	1	0	0
GORASP1	1	0	0	0	0	0
GORASP2	1	0	0	1	0	0
GOSR1	1	0	0	1	0	0

GOT1	0	0	0	1	0	0
GOT2	0	1	0	1	0	0
GP1BA	1	0	0	1	0	0
GPA33	0	0	1	0	0	1
GPAA1	1	0	0	1	0	0
GPAM	0	0	0	1	1	0
GPBP1L1	0	0	1	1	0	0
GPC3	0	0	1	0	0	0
GPC5	0	0	0	0	0	1
GPD1	0	0	1	0	0	1
GPHA2	0	0	1	0	0	1
GPHB5	1	0	1	0	0	1
GPHN	0	1	0	0	0	0
GPI	1	0	0	1	0	0
GPKOW	1	1	0	1	0	0
GPNMB	1	0	1	1	0	1
GPR107	0	0	0	1	0	0
GPR108	1	0	0	1	0	0
GPR109A	0	1	0	0	0	0
GPR109B	0	1	1	0	0	1
GPR110	0	1	1	0	0	1
GPR113	0	0	0	0	0	1
GPR133	0	1	0	0	0	0
GPR137	0	0	0	1	0	0
GPR141	0	0	1	0	0	0
GPR142	0	1	1	1	0	0
GPR143	0	0	1	0	0	1
GPR151	0	0	1	0	1	1
GPR155	1	0	0	1	0	0
GPR156	0	0	1	0	0	1
GPR161	0	0	1	0	0	1
GPR162	0	0	0	1	0	0
GPR171	0	0	1	0	0	0
GPR172A	1	0	0	1	0	0
GPR176	1	0	0	0	0	0
GPR180	1	1	0	1	0	0
GPR19	1	1	1	1	0	1
GPR20	0	0	0	0	0	1
GPR23	0	1	0	0	0	0
GPR26	0	0	1	0	0	0
GPR27	0	1	0	0	0	0
GPR34	0	1	0	1	0	0
GPR35	0	0	1	0	0	0
GPR37L1	0	1	1	0	1	1
GPR44	0	0	0	0	1	0
GPR45	0	0	1	0	0	1
GPR64	1	1	1	1	1	1
GPR65	0	0	0	1	0	0
GPR82	0	1	0	0	0	0
GPR84	0	0	0	1	0	0
GPRASP1	0	1	1	1	0	1
GPRC5A	0	0	0	0	0	1
GPRC5B	1	0	0	1	0	1
GPS1	1	0	0	1	0	0
GPS2	1	0	0	1	0	0
GPT	1	0	1	0	0	1
GPT2	0	1	0	0	0	0
GPX1	1	0	0	1	0	0
GPX3	1	0	1	1	0	0

GPX4	1	0	0	1	0	0
GRAMD1A	0	0	0	0	0	1
GRAMD2	0	0	0	0	0	1
GRAMD3	0	0	0	0	1	0
GRB2	1	0	0	1	1	0
GRHPR	0	0	0	1	0	0
GRIA3	0	0	1	0	0	0
GRK4	1	0	0	1	0	0
GRK6	1	0	0	1	0	0
GRLF1	0	0	1	0	0	1
GRM2	0	0	1	0	0	1
GRN	1	0	0	1	0	0
GRP	0	0	1	0	0	0
GRPEL1	1	0	0	0	0	0
GRPEL2	1	0	0	1	0	0
GRPR	0	0	1	0	0	1
GRSF1	0	0	0	1	0	0
GRTP1	0	0	1	0	0	1
GRWD1	1	0	0	0	0	0
GSDM1	1	0	1	0	0	1
GSDMDC1	0	0	0	0	1	0
GSDML	0	0	1	0	0	1
GSG1	0	0	0	0	0	1
GSN	0	0	1	1	0	1
GSPT1	1	0	0	1	0	0
GSPT2	0	1	0	0	0	0
GSR	0	0	1	0	0	0
GSS	0	0	0	1	0	0
GSTA4	1	0	0	0	0	0
GSTCD	1	0	0	1	0	0
GSTK1	1	0	0	0	1	0
GSTM2	0	0	0	1	0	1
GSTM4	0	0	0	1	0	1
GSTO2	0	1	0	1	1	0
GSTT1	1	0	1	0	0	0
GTDC1	1	0	1	1	1	1
GTF2B	0	0	0	0	1	0
GTF2E1	0	0	0	1	0	1
GTF2E2	1	0	0	1	0	0
GTF2F2	1	0	1	0	0	1
GTF2H1	1	0	1	1	1	1
GTF2H2	0	0	0	1	0	0
GTF2H3	1	0	0	1	1	0
GTF2H4	1	1	0	1	1	0
GTF2IRD1	0	0	1	0	0	1
GTF3C1	1	0	0	1	0	0
GTF3C2	1	0	0	1	0	1
GTF3C3	0	1	1	1	0	1
GTF3C4	0	0	0	1	0	0
GTPBP1	1	0	1	1	1	0
GTPBP2	0	0	0	1	0	0
GTPBP5	1	0	1	0	0	1
GTPBP6	0	0	1	0	0	0
GTSE1	1	0	0	0	1	0
GUCA1A	0	1	1	0	0	1
GUCA2A	0	1	1	0	0	1
GUK1	0	1	0	1	1	0
GUSBL2	1	0	0	1	0	0
GYPC	0	1	0	0	0	0

GYS1	1	0	0	1	1	0
GYS2	1	0	1	1	1	0
GZMB	0	0	1	0	1	1
H1F0	1	0	0	0	0	0
H1FNT	0	1	1	0	0	1
H2AFV	1	0	0	1	0	0
H2AFY	1	0	0	0	0	0
H2AFZ	1	0	0	1	1	0
H2BFWT	0	1	1	1	0	1
H3F3A	1	0	0	1	0	0
H3F3B	0	0	0	1	0	0
HADHA	1	0	0	1	0	0
HAGH	1	0	1	1	0	1
HAL	0	0	0	0	1	0
HAMP	1	0	1	1	0	1
HAO2	0	0	1	0	0	1
HARS	1	0	1	1	1	1
HAVCR2	1	0	0	1	0	0
HAX1	1	0	0	1	0	0
HBA2	1	0	0	0	0	0
HBB	0	1	0	0	1	1
HBD	0	1	1	0	1	1
HBEGF	0	0	0	1	0	1
HBP1	1	0	0	1	0	0
HBS1L	0	1	0	0	0	0
HCCA2	1	1	1	1	0	1
HCCS	0	1	1	0	0	1
HCFC1R1	0	0	0	0	0	1
HCG18	1	0	0	1	0	1
HCK	1	0	0	0	0	0
HCLS1	0	0	0	1	0	0
HCN3	1	0	0	1	1	0
HCST	0	0	0	0	1	0
HDAC1	1	0	0	0	0	0
HDAC11	1	0	0	0	0	0
HDAC2	0	1	0	0	0	0
HDAC3	1	0	1	1	1	0
HDAC6	1	1	0	0	0	1
HDAC7A	0	0	0	0	1	0
HDAC8	0	1	1	1	0	0
HDAC9	0	1	1	0	0	1
HDDC2	0	0	0	0	1	0
HDDC3	0	0	0	1	0	0
HDGF	1	0	0	1	1	0
HDGF2	1	0	0	0	0	0
HDHD1A	0	0	0	1	0	0
HDLBP	1	0	0	1	0	0
HEATR1	1	0	0	1	0	0
HECA	0	0	0	1	0	1
HECTD3	1	0	0	1	0	0
HECW2	1	0	0	0	0	0
HEL308	1	0	0	1	0	1
HELZ	0	1	0	0	1	0
HEMGN	1	1	1	0	0	1
HEMK1	1	0	0	0	0	0
HERC6	1	0	0	0	0	0
HERPUD1	1	0	1	0	0	1
HERPUD2	1	0	1	1	0	1
HES3	0	0	0	0	0	1

HES6	1	0	0	0	0	0
HEXA	1	0	1	1	0	1
HEXB	1	0	1	0	0	0
HEXDC	1	0	0	1	0	0
HFE	1	0	0	0	0	0
HFE2	0	0	1	0	0	0
HGD	0	1	1	1	1	1
HGF	0	1	0	0	0	0
HGS	1	0	0	1	0	0
HHEX	1	0	0	0	0	0
HHLA2	0	0	1	0	0	1
HHLA3	1	0	0	1	0	1
HIATL1	1	0	0	0	0	0
HIF1A	1	0	0	0	1	0
HIF1AN	1	0	0	1	0	0
HIF3A	0	1	1	0	0	0
HIG2	0	0	0	1	0	0
HIGD1A	1	0	0	1	0	0
HIGD1B	0	0	1	0	0	1
HIGD2A	1	0	0	1	1	0
HINT1	1	0	0	1	0	0
HINT2	1	0	0	1	1	0
HINT3	0	0	0	0	0	1
HIP1	0	0	0	0	0	1
HIP2	1	0	0	1	0	0
HIPK1	1	0	0	1	0	0
HIPK2	0	0	1	0	0	1
HIRA	1	1	0	1	0	0
HIRIP3	1	0	0	1	0	0
HIST1H1C	0	0	0	1	1	0
HIST1H2AH	1	0	0	0	0	0
HIST1H2AL	1	1	0	0	1	0
HIST1H2BK	1	0	0	0	0	0
HIST1H2BO	0	1	0	0	1	0
HIST1H3F	0	1	0	0	0	0
HIST1H3G	0	1	0	0	0	0
HIST1H3I	0	1	0	0	1	0
HIST1H4C	1	1	0	1	0	0
HIST1H4E	1	0	0	1	0	0
HIST2H2AB	0	1	0	0	0	0
HIST2H2AC	0	1	0	0	0	0
HIST3H2BB	1	0	0	1	0	1
HIVEP1	1	0	0	0	0	0
HIVEP2	0	0	1	0	0	1
HK2	0	0	1	0	1	1
HK3	0	0	0	0	1	0
HKDC1	0	1	1	1	0	1
HKR1	0	0	0	1	0	0
HLA-A	0	0	0	0	1	0
HLA-DMA	1	0	0	1	0	1
HLA-DMB	0	0	0	1	1	0
HLA-DPA1	1	0	0	1	1	0
HLA-DPB1	1	0	0	1	1	0
HLA-DQA1	1	0	1	1	0	0
HLA-DQA2	0	0	1	1	0	1
HLA-DQB1	1	0	0	1	0	0
HLA-DQB2	0	1	0	0	0	0
HLA-DRA	1	0	0	1	0	0
HLA-DRB1	1	0	0	1	0	0

HLA-G	0	0	0	1	0	0
HLCS	1	0	0	0	0	0
HMBOX1	1	0	0	1	0	0
HMBS	1	0	0	1	0	0
HMG2L1	0	0	0	0	0	1
HMGB1	0	1	0	0	1	0
HMGB3	0	1	0	1	1	0
HMGCR	1	0	0	0	1	0
HMGCS1	0	0	1	0	0	0
HMGCS2	0	1	1	0	0	1
HMMR	1	0	0	0	1	0
HMOX1	1	0	0	1	0	0
HMP19	0	1	1	0	0	1
HNF4G	1	0	0	0	0	0
HNMT	1	0	0	1	0	0
HNRPA1	1	0	0	1	0	0
HNRPA3	1	0	0	1	0	1
HNRPC	0	0	0	1	0	0
HNRPD	0	0	0	1	0	0
HNRPDL	1	0	0	1	0	0
HNRPH1	1	0	0	0	1	0
HNRPH2	1	1	0	1	0	0
HNRPH3	1	0	0	0	0	0
HNRPK	1	0	0	0	0	0
HNRPLL	1	0	0	0	0	0
HNRPU	1	0	0	1	0	0
HNRPUL1	1	0	0	1	1	1
HOM-TES-103	0	0	0	1	0	0
HOMER1	0	0	0	1	0	0
HOMER3	1	0	0	0	0	0
HOOK2	1	0	0	0	0	0
HOOK3	1	0	0	0	0	0
HOP	0	1	0	0	0	1
HOXA2	1	0	0	0	0	0
HOXA5	1	1	0	0	0	0
HOXA6	1	1	0	0	0	0
HOXB1	0	0	1	0	0	1
HOXB3	1	0	0	0	0	0
HOXC13	0	0	0	1	0	0
HOXC8	0	0	0	0	0	1
HP	0	0	1	0	0	1
HP1BP3	1	0	0	0	0	0
HPR	0	0	1	0	0	1
HPRT1	0	1	0	0	0	0
HPS3	0	0	0	0	0	1
HPS4	1	1	1	1	1	1
HPS5	1	0	1	1	1	1
HPSE	0	0	0	0	1	0
HRES1	1	0	0	1	0	0
HRG	0	0	1	0	0	1
HRH1	1	0	1	1	0	1
HRH2	0	1	0	0	1	0
HRSP12	1	0	0	1	1	0
HS2ST1	1	0	0	1	0	0
HS322B1A	0	0	1	0	0	0
HS3ST1	1	0	0	0	0	0
HS3ST2	0	0	1	0	0	1
HSBP1	0	0	0	1	0	0
HSD11B1	1	0	1	1	0	1

HSD11B1L	1	0	0	1	0	0
HSD17B13	0	0	1	0	0	1
HSD17B2	0	1	1	0	0	1
HSD17B8	1	0	0	1	0	0
HSD3B2	0	0	1	0	0	1
HSD3B7	1	0	1	1	0	1
HSDL1	1	0	0	1	0	0
HSDL2	1	0	0	0	0	0
HSF1	1	0	0	1	0	0
HSF2	1	0	0	0	0	0
HSF2BP	1	0	0	1	0	0
HSH2D	0	0	1	0	0	1
HSN2	0	0	1	1	0	0
HSP90AA1	1	0	0	1	0	0
HSP90AB6P	0	0	1	0	0	1
HSPA14	1	0	0	1	0	0
HSPA1A	1	0	0	1	0	0
HSPA1B	1	0	0	1	0	0
HSPA1L	1	0	0	0	0	0
HSPA4	1	0	0	1	0	0
HSPA5	1	0	0	0	0	0
HSPA8	1	0	0	1	0	0
HSPB2	0	0	1	0	0	0
HSPB7	0	1	1	0	0	1
HSPBP1	1	0	0	0	0	0
HSPC047	0	0	1	1	0	1
HSPC111	1	0	0	1	1	0
HSPC152	1	0	0	1	0	0
HSPC171	1	0	0	1	1	0
HSPD1	1	0	0	1	0	1
HSPE1	1	0	0	1	0	1
HSPH1	1	0	0	1	0	0
HTATIP2	1	0	0	0	0	0
HTATSF1	0	1	0	0	0	0
HTF9C	1	0	0	1	0	0
HTN3	0	1	1	0	0	0
HTR1F	0	0	1	0	0	0
HTR3A	0	1	1	0	0	1
HTR3B	0	1	1	1	0	1
HTRA2	1	0	0	1	0	0
HTRA4	0	0	0	0	0	1
HUS1B	0	0	1	0	0	1
HUWE1	0	0	1	0	0	1
HVCN1	0	1	0	0	0	0
HYAL1	1	0	1	1	0	0
HYAL3	1	0	1	1	0	0
HYI	0	1	1	0	0	1
HYLS1	1	0	1	0	0	1
HYOU1	1	0	0	1	0	0
HYPK	1	0	0	1	0	0
IARS	1	0	0	1	0	0
IBRDC3	1	0	0	0	0	0
IBTK	1	0	0	1	0	0
ICA1	0	1	1	0	0	1
ICAM2	0	0	1	0	0	1
ICAM3	1	0	0	1	0	0
ICAM4	1	0	0	1	0	1
ICF45	1	0	0	1	0	0
ICK	1	0	0	1	0	0

ICMT	0	0	0	1	0	0
ICOSLG	0	0	0	0	0	1
ICT1	1	0	0	0	0	0
ID2	1	0	0	1	1	0
ID2B	0	1	1	0	1	1
ID3	1	0	0	1	0	0
IDH3A	1	0	0	1	0	0
IDH3B	1	0	0	1	0	0
IDH3G	1	0	0	1	0	0
IDI1	1	0	0	1	0	0
IDS	0	1	1	0	0	0
IDUA	0	0	0	1	0	0
IER2	1	0	0	0	0	0
IER3	1	0	0	0	0	0
IER5	1	0	0	0	0	0
IFI16	0	0	0	0	0	1
IFI30	1	0	0	1	0	0
IFI44L	1	0	0	0	0	0
IFIH1	1	0	1	1	0	1
IFIT1	1	0	0	1	1	0
IFIT2	0	0	0	0	1	0
IFIT3	0	0	0	1	0	0
IFITM1	0	1	0	0	0	0
IFITM5	0	0	0	0	0	1
IFNA13	0	1	1	1	0	0
IFNAR2	1	0	0	1	0	1
IFNGR1	1	0	0	1	0	0
IFNGR2	0	0	0	1	0	0
IFP38	1	0	1	1	0	1
IFRD1	1	0	0	1	0	1
IFRD2	1	0	0	0	0	0
IFRG15	1	0	1	1	0	0
IFT122	1	0	0	1	1	0
IFT140	1	0	0	1	0	0
IFT52	0	0	0	1	0	0
IFT74	1	0	0	1	0	0
IFT80	1	0	0	1	0	0
IFT88	0	1	0	0	0	0
IGBP1	0	1	0	1	0	0
IGFALS	0	0	1	0	1	1
IGFL3	0	0	1	0	0	1
IGHMBP2	1	0	0	1	1	0
IGSF11	1	1	1	0	0	1
IGSF2	0	0	0	0	0	1
IGSF6	1	0	0	1	0	0
IGSF8	1	0	0	0	0	0
IHPK3	0	0	1	0	0	1
IK	1	0	0	1	0	0
IKBKG	1	1	0	1	0	0
IKIP	1	0	0	0	1	0
IL10RB	0	0	0	1	0	0
IL11RA	0	1	1	1	1	1
IL12A	0	0	0	0	1	0
IL12B	0	0	1	0	0	1
IL13RA1	0	1	0	0	0	0
IL15	1	1	1	0	0	0
IL16	1	0	1	0	0	1
IL17F	0	1	1	0	0	1
IL17RB	1	0	0	1	0	0

IL17RE	0	1	1	0	0	1
IL18	0	0	0	1	0	1
IL18R1	0	0	1	1	0	1
IL18RAP	0	0	1	0	0	0
IL19	0	0	1	0	0	1
IL1A	1	1	0	1	0	1
IL1F10	0	1	1	0	0	1
IL1F5	0	1	1	0	0	1
IL1F8	0	0	1	0	0	1
IL1R1	1	0	1	0	0	1
IL1R2	0	0	1	0	0	1
IL1RAPL1	0	0	1	0	0	1
IL1RL2	1	0	0	0	0	0
IL1RN	1	0	1	0	1	1
IL21	0	0	1	0	0	1
IL21R	1	0	1	0	0	1
IL22RA2	0	0	1	1	0	1
IL26	0	1	0	0	0	1
IL28A	0	0	1	0	0	0
IL2RA	0	1	1	0	1	1
IL2RG	0	1	1	0	1	1
IL3RA	1	0	0	1	0	0
IL4I1	1	0	1	1	0	1
IL6R	0	1	0	0	1	0
IL7R	1	1	1	1	0	1
IL8	1	0	0	0	0	1
IL8RB	0	0	0	0	0	1
ILF2	1	0	0	1	0	0
ILK	1	1	0	1	1	0
ILVBL	0	0	0	1	0	0
IMMP2L	0	1	0	1	1	1
IMMT	1	0	0	0	0	0
IMP3	1	0	0	1	0	0
IMP4	1	0	0	1	0	0
IMPA2	0	1	0	0	1	0
IMPACT	1	0	0	1	0	0
IMPAD1	1	0	0	1	0	0
IMPDH2	1	0	0	1	0	0
INCA	0	0	0	0	0	1
INCENP	0	0	1	0	0	0
INDO	0	0	0	0	1	0
INDOL1	1	0	1	1	0	1
ING1	1	0	0	1	0	0
ING2	1	0	0	1	0	1
ING4	0	1	0	0	0	0
INHBE	0	0	1	0	0	0
INPP4A	0	0	0	1	0	1
INPP4B	0	0	1	0	0	1
INPP5D	0	0	1	0	1	1
INPP5E	1	0	0	1	0	0
INPP5F	1	0	0	1	0	1
INSIG1	0	0	0	1	0	1
INTS12	1	0	0	1	0	0
INTS4	1	0	0	1	0	0
INTS5	0	0	1	0	0	0
INTS6	1	0	0	0	0	0
INTS7	1	0	0	1	1	0
INTS8	0	0	1	0	0	0
INVS	1	0	0	1	0	0

IPO11	0	0	0	1	0	0
IPO13	1	0	0	0	0	1
IPO4	1	0	0	1	1	0
IPO8	0	0	0	1	0	0
IPO9	1	0	1	1	0	1
IPP	0	0	0	1	0	0
IPPK	1	0	0	1	0	0
IQCG	1	0	1	1	0	1
IQCH	0	1	1	0	0	0
IQSEC1	1	0	0	0	0	0
IQWD1	1	0	0	1	0	1
IRAK4	1	0	0	0	0	0
IREB2	0	0	0	0	1	0
IRF1	0	1	0	0	1	0
IRF2	1	0	0	0	0	0
IRF2BP1	1	0	0	1	0	0
IRF3	1	0	0	1	1	0
IRF8	0	1	0	0	0	0
IRGQ	1	0	0	0	0	0
IRS2	0	0	0	0	0	1
ISG20L1	1	0	0	1	0	1
ISG20L2	1	0	0	1	1	0
ISLR	0	0	1	0	0	1
ISOC1	1	0	0	0	0	0
ISYNA1	1	0	0	0	0	0
ITFG1	1	0	0	1	0	0
ITGA10	1	0	0	1	0	1
ITGA2B	0	0	0	1	0	0
ITGA4	0	1	0	0	0	0
ITGAD	0	0	1	0	0	1
ITGAE	1	0	1	1	1	1
ITGAM	1	0	0	0	0	0
ITGAV	1	0	0	0	0	0
ITGAX	1	0	0	0	1	0
ITGB1	1	0	1	1	0	1
ITGB1BP1	1	0	0	1	1	0
ITGB1BP2	0	1	1	0	0	1
ITGB3	0	0	0	0	0	1
ITGB3BP	1	0	0	1	0	0
ITGB4	0	0	0	0	0	1
ITGB7	0	0	0	0	1	0
ITGBL1	1	1	1	0	0	1
ITIH4	0	1	1	0	1	1
ITIH5L	0	1	1	0	0	1
ITPA	0	0	0	0	1	0
ITPKA	0	0	0	1	0	0
ITPKC	1	0	0	0	0	0
ITPR2	0	1	0	0	0	0
IVL	0	1	1	0	0	1
IVNS1ABP	0	0	0	0	1	0
IWS1	1	0	0	1	0	0
JAG1	1	0	0	1	0	0
JAGN1	1	1	0	1	0	0
JAK1	1	0	1	0	0	1
JAK2	0	0	0	0	1	0
JAKMIP2	0	0	1	1	0	1
JARID1A	1	0	0	1	1	0
JAZF1	1	0	0	0	0	0
JMJD1A	1	0	0	0	0	0

JMJD1B	0	0	1	0	0	0
JMJD2C	1	0	0	1	0	0
JMJD2D	1	0	0	1	0	0
JMJD4	1	0	1	1	0	0
JMJD5	1	1	0	1	1	0
JMY	0	0	1	0	0	0
JOSD1	1	0	1	1	1	0
JOSD2	1	0	0	0	0	0
JPH4	0	0	1	0	0	0
JRK	1	0	0	0	0	1
JRKL	0	0	0	1	1	0
JTB	1	0	0	1	0	0
JTV1	1	1	0	1	0	0
JUB	0	0	1	1	0	1
JUN	1	0	0	1	0	0
JUNB	1	0	0	0	0	0
JUND	0	0	0	1	0	0
JUP	1	0	0	0	0	1
KAL1	0	0	0	1	0	0
KARS	1	1	0	1	0	0
KATNB1	0	0	0	1	0	0
KBTBD11	0	0	0	0	0	1
KBTBD2	1	0	0	0	0	0
KBTBD3	0	0	0	1	0	0
KBTBD6	1	0	0	1	0	0
KBTBD8	1	0	0	1	1	0
KCMF1	1	1	0	1	1	0
KCNA10	0	0	1	1	0	1
KCNA3	0	1	0	1	0	0
KCNAB1	0	1	1	0	0	1
KCNAB2	0	0	0	0	0	1
KCNE1L	0	1	1	0	1	1
KCNG1	0	0	1	0	0	1
KCNG4	0	0	1	0	0	1
KCNH6	0	1	1	0	0	1
KCNIP1	0	1	1	0	0	1
KCNJ1	1	0	1	0	0	1
KCNJ14	1	0	0	0	0	1
KCNJ16	0	0	1	0	0	1
KCNJ4	0	0	1	0	0	1
KCNJ5	0	0	0	1	0	0
KCNK18	0	1	1	0	0	1
KCNK6	1	0	0	1	1	0
KCNK9	0	0	0	0	0	1
KCNMA1	1	0	0	1	0	0
KCNMB1	0	1	0	0	0	0
KCNMB2	0	0	1	0	0	1
KCNQ1	0	0	1	0	0	1
KCNRG	0	0	1	1	0	1
KCNV2	0	0	1	0	0	1
KCTD10	1	0	0	1	1	0
KCTD12	0	0	0	1	0	0
KCTD2	0	0	0	1	0	0
KCTD5	1	0	0	1	0	0
KCTD6	1	1	1	1	1	0
KCTD9	1	0	1	1	0	0
KDELR2	1	0	0	0	0	0
KEL	0	1	1	0	0	1
KERA	0	1	1	0	0	1

KHK	0	0	0	1	0	0
KIAA0020	1	0	0	1	0	1
KIAA0090	1	0	0	1	1	1
KIAA0100	1	0	1	1	0	1
KIAA0101	1	0	0	0	0	0
KIAA0133	1	0	0	1	0	1
KIAA0143	0	0	1	0	0	1
KIAA0152	0	0	0	1	0	0
KIAA0157	1	0	0	0	0	0
KIAA0179	1	0	0	1	0	0
KIAA0195	1	0	0	1	0	0
KIAA0196	1	0	1	1	1	1
KIAA0232	1	0	0	1	0	0
KIAA0240	0	0	1	0	0	1
KIAA0241	1	1	0	1	0	0
KIAA0247	1	0	1	1	0	1
KIAA0251	0	0	0	1	0	0
KIAA0258	1	0	0	1	0	0
KIAA0286	1	0	0	0	0	1
KIAA0319L	1	0	0	0	0	0
KIAA0323	1	0	0	1	0	0
KIAA0329	1	0	0	1	1	0
KIAA0355	0	0	1	0	0	1
KIAA0367	0	1	0	0	0	0
KIAA0372	1	0	0	1	0	0
KIAA0391	1	0	0	1	1	0
KIAA0406	1	1	0	1	1	0
KIAA0408	0	0	1	0	0	1
KIAA0409	1	0	0	1	1	0
KIAA0423	1	0	0	1	1	0
KIAA0427	0	0	0	1	0	0
KIAA0460	1	0	0	1	0	0
KIAA0513	0	0	0	0	1	0
KIAA0528	1	0	0	0	0	0
KIAA0556	1	0	0	1	0	0
KIAA0586	1	0	0	1	0	0
KIAA0652	1	0	0	1	0	0
KIAA0664	1	0	0	1	0	0
KIAA0701	1	0	0	0	0	0
KIAA0738	0	0	0	1	1	0
KIAA0746	0	0	0	1	0	0
KIAA0753	1	0	0	1	0	0
KIAA0802	1	0	1	0	0	1
KIAA0828	1	0	0	1	0	0
KIAA0831	1	0	0	1	0	0
KIAA0889	0	0	1	1	1	1
KIAA0892	1	1	0	0	1	0
KIAA0907	1	0	0	1	0	0
KIAA0922	0	1	0	0	1	0
KIAA1009	1	0	0	0	1	0
KIAA1012	1	0	0	1	0	0
KIAA1024	0	0	1	0	0	1
KIAA1026	0	0	1	0	0	1
KIAA1128	0	0	1	0	0	0
KIAA1143	0	0	0	1	0	0
KIAA1160	1	0	0	1	0	0
KIAA1199	0	1	0	0	0	0
KIAA1219	0	0	1	0	0	1
KIAA1244	0	1	1	0	0	1

KIAA1267	1	0	0	1	1	0
KIAA1279	1	0	0	1	0	0
KIAA1324L	0	1	1	0	0	1
KIAA1328	0	1	0	0	1	0
KIAA1344	0	0	0	0	0	1
KIAA1407	1	0	0	1	0	0
KIAA1429	1	0	0	0	1	0
KIAA1432	0	0	1	0	0	1
KIAA1467	0	1	0	0	0	0
KIAA1468	0	0	1	0	0	0
KIAA1505	1	0	0	0	1	0
KIAA1522	0	0	1	0	0	1
KIAA1524	1	0	0	1	1	0
KIAA1530	0	0	1	1	0	0
KIAA1604	0	0	0	1	0	0
KIAA1627	1	0	0	0	0	0
KIAA1632	0	0	1	1	0	0
KIAA1704	1	0	0	1	0	1
KIAA1706	1	0	0	0	1	0
KIAA1715	0	0	0	1	0	0
KIAA1727	0	0	1	0	0	1
KIAA1729	0	0	0	1	0	0
KIAA1737	1	0	0	1	0	0
KIAA1754	1	0	0	0	0	0
KIAA1754L	1	0	0	0	0	0
KIAA1799	1	0	0	1	0	0
KIAA1826	1	0	0	0	0	0
KIAA1875	1	0	0	1	1	0
KIAA1913	0	0	1	0	0	1
KIAA1967	1	0	0	1	0	0
KIAA2018	0	0	0	0	0	1
KIAA2026	1	0	0	1	0	0
KIF13A	0	0	0	1	0	0
KIF14	1	0	0	0	0	0
KIF15	0	0	0	1	0	0
KIF18A	1	1	0	1	1	1
KIF1C	1	0	0	0	0	0
KIF20A	1	0	0	1	0	0
KIF24	0	0	0	1	0	0
KIF25	0	1	1	1	0	1
KIF4A	0	1	1	1	0	1
KIF5B	0	0	0	0	1	0
KIFAP3	1	0	0	1	0	0
KIFC1	0	1	0	0	1	0
KIR2DL1	1	0	1	0	0	1
KIR2DS3	1	0	1	1	0	1
KIR2DS4	1	0	1	1	0	1
KIRREL3	1	0	0	0	0	0
KLC3	1	0	1	0	0	1
KLC4	1	0	0	1	0	0
KLF1	1	0	0	0	1	0
KLF10	0	0	1	0	0	1
KLF13	0	0	0	0	1	0
KLF15	0	0	0	0	0	1
KLF3	1	0	0	1	0	0
KLF6	1	0	0	0	0	0
KLF7	1	0	0	0	0	0
KLF9	1	0	1	1	0	1
KLHDC3	1	0	0	1	1	0

KLHDC4	1	0	0	0	0	0
KLHDC5	0	1	0	0	0	0
KLHL12	1	0	0	1	0	0
KLHL15	1	0	0	0	0	0
KLHL18	0	0	0	1	0	0
KLHL2	1	1	0	0	1	0
KLHL22	0	0	0	1	0	0
KLHL24	1	0	0	0	0	0
KLHL25	1	0	0	0	0	0
KLHL5	0	0	1	0	1	1
KLHL7	1	0	0	1	0	0
KLK1	0	0	1	0	0	1
KLK15	0	0	1	0	0	1
KLK8	0	0	0	0	0	1
KMO	1	0	0	1	0	0
KNG1	0	1	1	1	0	0
KNTC1	1	0	0	0	1	0
KPNA1	1	0	0	0	0	0
KPNA2	0	0	0	1	0	0
KPNA3	1	0	0	0	0	0
KPNA4	1	0	0	1	0	1
KPNA5	1	0	0	0	0	0
KPNB1	1	0	0	0	0	0
KPTN	0	0	1	1	0	0
KREMEN1	0	0	0	0	0	1
KRIT1	1	0	0	1	0	1
KRT10	1	1	1	0	0	0
KRT17	0	1	1	0	0	1
KRT23	0	1	1	0	0	1
KRT24	0	0	1	0	0	1
KRT3	0	1	1	0	0	1
KRT6B	1	0	1	0	0	1
KRTAP10-10	0	0	1	0	0	1
KRTAP10-2	0	0	1	0	0	1
KRTAP10-8	0	0	1	0	0	1
KRTAP12-1	0	0	1	0	0	1
KRTAP12-4	0	1	1	0	0	1
KRTAP13-1	1	0	1	0	0	1
KRTAP13-4	0	1	1	1	0	1
KRTAP21-1	0	0	1	1	0	1
KRTAP3-2	0	0	1	0	0	1
KRTAP5-1	0	0	0	0	0	1
KRTAP6-1	0	1	1	0	0	1
KRTAP8-1	0	0	1	0	0	0
KRTAP9-3	0	1	1	0	0	1
KRTAP9-4	0	0	1	0	0	1
KSR1	0	0	1	1	0	1
KSR2	1	1	1	0	0	1
KTI12	0	0	0	1	0	0
KYNU	1	0	1	1	0	0
KUA-UEV	1	1	1	1	1	1
L1CAM	0	0	1	0	0	0
L2HGDH	0	0	0	1	0	0
L3MBTL3	0	1	0	1	1	0
LACE1	0	0	0	1	0	0
LACRT	0	1	1	1	1	1
LACTB2	1	0	0	1	1	1
LAIR2	0	0	1	0	0	0
LAMA2	0	0	1	1	0	0

LAMA3	0	1	1	0	0	1
LAMB2	1	0	0	1	0	0
LAMC2	0	0	1	0	0	1
LAMP1	1	0	0	1	0	0
LANCL1	1	0	0	0	0	0
LANCL2	1	1	0	1	0	0
LAP3	1	0	0	1	1	0
LAPTM4B	0	0	0	0	0	1
LARP1	1	1	1	1	0	1
LARP2	1	0	0	0	0	0
LARP4	1	0	0	1	0	0
LARP5	0	1	0	0	1	1
LARS2	1	0	0	0	0	0
LAS1L	1	0	0	1	0	0
LATS2	1	0	0	1	0	0
LBP	0	0	1	0	0	1
LBR	1	0	0	0	0	0
LCAT	0	0	1	0	0	1
LCE1A	0	1	1	0	0	1
LCE1D	0	1	1	0	0	1
LCE3B	0	1	1	0	0	1
LCE3C	0	0	1	0	0	1
LCMT1	0	0	0	0	1	0
LCMT2	1	0	0	1	0	0
LCN1	0	0	1	0	0	1
LCN2	0	1	1	0	0	1
LCN8	0	0	1	0	0	1
LCP1	1	0	0	0	1	0
LCP2	0	0	0	0	1	0
LCT	0	0	1	0	0	1
LDB1	1	0	0	0	1	0
LDHB	0	1	0	0	0	0
LDHD	1	0	0	0	0	0
LDLR	0	0	0	1	0	0
LDLRAP1	1	0	0	1	0	0
LEFTY2	0	1	1	0	0	1
LELP1	0	1	1	0	0	1
LEMD2	1	1	0	1	1	0
LENG1	0	0	0	1	0	0
LENG8	1	0	0	1	1	0
LEPR	0	0	0	1	0	0
LEPRE1	1	0	1	1	0	0
LEPROT	0	0	0	1	0	0
LETMD1	1	1	0	1	1	0
LGALS12	0	0	0	0	1	0
LGALS3	1	0	1	1	0	1
LGALS4	1	0	0	0	0	0
LGALS8	1	0	0	0	0	0
LGALS9	1	0	0	1	0	0
LGMN	1	0	0	1	0	0
LGR6	0	0	1	0	0	1
LGTN	1	0	0	1	0	0
LHFPL1	0	1	0	1	0	1
LHFPL2	0	0	0	0	1	0
LIAS	1	0	0	0	0	0
LIG1	1	0	0	1	0	0
LIG3	1	0	0	1	0	1
LIG4	1	0	0	0	0	0
LILRA1	0	1	0	0	0	0

LILRA2	0	1	1	0	0	0
LILRA4	1	0	1	0	0	1
LILRA6	0	0	0	0	0	1
LILRB4	1	0	0	0	0	0
LILRB5	0	0	0	0	0	1
LIMA1	0	0	0	1	0	0
LIMK2	1	0	0	1	1	0
LIMS1	1	0	0	0	0	0
LIMS3	0	0	1	0	1	1
LIN7C	1	0	1	1	0	1
LIN9	1	1	0	0	0	0
LINS1	1	0	1	1	0	1
LIPA	1	1	1	1	1	1
LIPT1	1	0	0	1	1	1
LITAF	1	0	0	1	0	0
LMAN2	1	0	0	1	0	0
LMAN2L	1	0	0	0	0	0
LMBR1	1	0	0	0	1	0
LMBR1L	0	0	1	1	0	0
LMBRD2	1	0	0	0	0	0
LMLN	0	0	1	1	0	1
LMO4	0	0	0	1	0	0
LMO6	0	1	0	0	0	0
LNPEP	0	0	0	1	0	1
LOC113386	1	0	0	1	1	0
LOC124216	1	0	0	1	0	0
LOC124220	0	0	0	0	0	1
LOC124446	1	0	0	1	1	0
LOC124512	1	0	0	1	0	0
LOC128977	1	0	0	0	0	0
LOC130074	1	0	0	0	0	0
LOC130355	1	0	0	1	1	1
LOC134145	1	0	0	0	0	0
LOC148137	1	1	0	1	0	0
LOC153222	1	0	0	1	0	0
LOC153328	1	0	0	0	0	0
LOC153364	0	0	0	1	0	0
LOC153561	1	0	1	1	1	1
LOC158572	0	1	0	0	1	0
LOC161247	0	0	1	0	0	1
LOC165186	0	1	1	0	1	1
LOC196549	0	0	1	0	0	1
LOC196752	1	0	0	1	0	0
LOC197135	1	0	0	0	0	0
LOC201164	1	0	0	1	0	1
LOC201175	1	0	0	1	0	0
LOC201181	0	0	1	0	0	0
LOC201725	1	0	1	1	0	0
LOC202459	0	0	0	1	0	0
LOC203547	0	1	0	0	0	0
LOC205251	0	0	1	0	0	1
LOC220686	0	0	1	0	1	1
LOC221442	0	0	0	0	0	1
LOC283152	0	0	1	0	1	1
LOC283849	1	0	0	0	0	0
LOC283932	0	0	0	0	0	1
LOC284009	1	0	1	1	0	1
LOC284757	0	0	1	0	0	1
LOC284861	0	0	1	0	0	1

LOC284912	0	0	1	0	0	0
LOC285033	1	0	1	0	0	1
LOC285074	1	1	1	1	1	1
LOC285636	1	0	0	1	1	0
LOC285908	1	0	1	0	0	1
LOC286016	1	0	0	1	0	0
LOC286187	1	0	0	0	0	0
LOC338328	0	0	1	0	0	1
LOC339229	1	0	0	1	1	0
LOC339457	0	0	1	0	0	1
LOC348262	0	0	0	1	0	1
LOC349196	0	0	0	1	0	0
LOC374395	1	0	0	1	0	0
LOC374920	1	0	0	1	0	0
LOC387790	1	1	0	1	0	0
LOC387882	0	1	0	0	0	0
LOC388284	0	0	0	1	1	0
LOC388438	1	0	1	0	0	1
LOC388969	0	0	0	1	0	1
LOC389118	1	0	1	1	0	0
LOC389517	0	1	1	0	0	1
LOC389607	1	0	0	1	1	0
LOC389641	1	0	0	0	0	0
LOC389833	1	0	1	1	1	1
LOC390637	1	0	0	1	0	0
LOC391356	1	0	0	1	0	0
LOC399706	0	0	1	0	0	0
LOC399744	0	0	1	0	0	1
LOC399898	0	1	1	0	0	0
LOC399900	1	0	0	1	0	0
LOC400197	0	0	0	1	0	0
LOC400506	1	0	0	1	0	0
LOC400657	1	0	0	0	0	0
LOC400986	0	0	1	0	0	0
LOC401052	1	0	0	0	0	0
LOC401072	1	0	1	0	0	1
LOC401398	1	0	0	1	0	0
LOC401431	1	0	0	1	0	0
LOC401620	0	1	0	1	0	1
LOC401622	0	0	1	0	0	1
LOC401623	0	0	1	0	0	1
LOC401720	0	1	0	0	1	0
LOC402176	0	0	1	0	0	1
LOC440093	1	0	0	0	0	0
LOC440258	1	0	0	1	0	0
LOC440354	1	0	0	1	1	1
LOC440731	0	0	0	1	0	0
LOC440742	0	1	1	0	0	1
LOC440944	1	0	0	0	0	0
LOC440993	0	0	1	0	0	1
LOC441087	1	0	0	1	0	0
LOC441150	0	0	0	0	1	0
LOC441193	1	0	0	0	0	0
LOC441208	0	1	0	0	0	0
LOC441268	0	0	1	0	0	1
LOC441294	0	0	1	1	0	1
LOC441763	1	0	0	1	0	0
LOC441956	0	1	1	0	0	1
LOC442132	0	0	1	0	0	1

LOC442535	1	0	0	1	0	1
LOC442582	1	1	1	1	0	1
LOC492311	0	0	0	0	0	1
LOC493869	0	0	1	0	0	1
LOC51035	1	0	0	0	0	0
LOC51057	1	0	1	1	0	1
LOC51136	0	0	1	0	0	0
LOC51252	0	0	1	0	0	1
LOC54103	1	0	1	1	0	1
LOC552891	1	1	0	0	0	0
LOC55908	0	0	1	0	0	1
LOC606495	1	0	0	1	0	0
LOC63920	1	0	0	1	0	0
LOC642370	1	0	0	0	0	0
LOC642852	1	0	0	0	0	0
LOC642934	0	0	1	0	0	0
LOC643011	1	0	1	0	0	0
LOC643045	0	0	0	0	0	1
LOC643206	0	0	0	1	0	0
LOC643339	1	0	0	1	0	0
LOC643396	1	0	0	0	0	0
LOC643493	1	1	0	0	0	0
LOC644099	1	0	0	1	1	0
LOC644377	1	0	0	1	0	1
LOC644380	0	0	0	0	0	1
LOC644733	1	0	0	1	0	0
LOC644961	1	0	0	1	0	0
LOC644978	1	0	0	0	0	0
LOC645052	0	1	0	0	0	0
LOC645261	0	0	1	0	0	1
LOC645427	0	0	1	0	0	1
LOC645460	1	1	0	0	0	0
LOC645676	0	0	0	1	0	0
LOC646100	0	1	1	1	0	1
LOC646146	0	0	1	0	0	1
LOC646345	0	1	1	0	0	1
LOC646407	1	0	0	0	0	0
LOC646496	0	0	1	0	0	0
LOC646667	1	0	0	0	0	0
LOC646778	0	1	0	0	0	0
LOC646897	1	0	0	1	0	0
LOC647243	1	0	0	1	0	0
LOC653240	0	0	1	0	0	0
LOC653352	0	0	0	1	0	0
LOC653566	1	0	0	1	0	0
LOC653604	1	1	0	0	0	0
LOC90379	1	0	0	0	0	0
LOC90624	1	0	0	1	0	0
LOC90826	0	0	0	0	1	0
LOC90835	1	0	0	1	0	0
LOC90925	0	1	1	0	1	1
LOC91431	1	0	1	1	0	0
LOC92017	1	0	1	0	1	1
LOC92270	0	0	1	0	0	1
LOC92497	0	0	1	0	0	0
LOC96610	1	0	1	1	0	1
LOH11CR2A	0	0	1	1	0	1
LONRF1	1	0	0	0	0	1
LOXL3	1	0	0	1	1	0

LPGAT1	0	1	0	0	1	0
LPIN1	1	0	1	0	0	1
LPP	1	0	1	1	0	1
LPXN	1	0	0	1	0	0
LRBA	1	0	1	1	0	1
LRCH4	1	0	0	0	0	0
LRDD	1	0	0	0	1	0
LRFN4	0	0	0	0	0	1
LRIG2	1	0	0	1	0	0
LRP10	0	0	1	0	0	1
LRP11	0	0	0	1	0	0
LRP2BP	0	0	1	0	0	1
LRPAP1	0	0	0	1	0	0
LRPPRC	1	0	0	0	0	0
LRRC14	1	0	0	1	1	0
LRRC17	0	0	1	0	1	1
LRRC28	1	0	0	1	0	0
LRRC29	1	0	0	1	1	0
LRRC31	0	0	1	0	1	1
LRRC33	0	0	1	0	0	0
LRRC34	0	1	0	0	0	0
LRRC37A	0	0	1	0	0	1
LRRC40	1	0	0	1	0	1
LRRC41	1	0	0	0	0	0
LRRC42	1	0	0	1	0	0
LRRC46	1	0	0	1	0	0
LRRC51	1	0	0	1	0	0
LRRC57	1	0	0	1	0	0
LRRC59	0	0	0	1	0	0
LRRC6	0	0	0	1	0	0
LRRC61	0	1	1	1	1	1
LRRC8A	1	0	1	0	0	1
LRRC8B	1	0	0	1	0	0
LRRC8C	1	1	0	0	0	0
LRRC8D	0	1	0	0	1	0
LRRFIP1	1	0	0	0	0	0
LRRFIP2	1	1	0	0	0	0
LRRIQ2	0	0	0	1	0	0
LRRK1	1	0	0	1	0	1
LRRK2	0	0	0	0	1	0
LRSAM1	1	0	0	0	1	0
LRTM1	0	0	1	0	0	1
LSAMP	0	0	1	0	0	1
LSG1	1	0	0	1	0	0
LSM1	1	0	0	1	0	1
LSM10	1	0	0	1	0	1
LSM14A	1	0	0	0	0	0
LSM2	1	0	0	1	0	0
LSM3	1	0	0	1	1	1
LSM4	0	0	0	0	1	0
LSM5	1	0	0	1	0	0
LSM7	1	0	0	1	0	0
LSM8	1	0	0	1	0	0
LSMD1	1	0	0	1	0	0
LSP1	0	0	0	1	0	1
LTB4R2	1	0	0	0	0	0
LTBP1	0	1	0	0	0	0
LTBP4	1	0	0	0	0	0
LTC4S	0	0	0	0	0	1

LTV1	0	1	0	0	0	0
LY75	0	0	0	1	1	0
LY86	0	0	1	0	0	1
LY9	1	0	1	0	1	1
LYAR	1	0	0	1	0	0
LYPD2	0	0	1	0	0	0
LYPD3	0	1	0	0	0	0
LYPLA2	0	0	0	1	1	0
LYPLAL1	0	0	0	1	0	1
LYSMD2	1	1	0	1	1	0
LYSMD3	0	0	0	1	0	0
LYSMD4	0	1	0	0	0	1
LYST	1	0	1	1	0	1
LZIC	1	0	0	1	0	0
LZTR1	1	0	0	1	0	0
LZTR2	0	0	0	0	0	1
M6PR	1	0	1	1	0	1
MAD2L1BP	0	0	0	1	1	0
MAEA	1	0	0	1	0	0
MAF	1	0	1	0	0	1
MAF1	1	0	0	1	1	0
MAFF	1	0	1	1	0	1
MAFG	1	0	0	1	0	0
MAG	0	0	1	0	0	1
MAGEA11	0	1	1	0	0	1
MAGEA2	0	1	1	0	0	1
MAGEB1	1	1	1	1	0	1
MAGED1	0	1	1	0	0	0
MAGED2	0	1	1	0	1	1
MAGEF1	0	1	0	0	0	0
MAGEH1	1	1	1	0	0	1
MAGEL2	0	1	1	0	0	1
MAGOH	1	0	0	1	0	0
MAK	1	0	0	0	0	0
MALT1	0	0	0	0	0	1
MAML2	0	0	0	1	0	0
MAML3	1	0	0	0	0	0
MAN1A1	1	0	0	0	0	0
MAN1B1	1	0	0	1	0	0
MAN2A1	0	0	0	1	1	0
MAN2B1	1	0	0	1	1	0
MAN2C1	1	0	0	1	0	0
MANBA	0	0	0	1	0	0
MANEAL	0	0	0	1	0	0
MAOA	1	1	0	0	0	0
MAOB	0	0	1	0	0	1
MAP1A	0	0	1	0	0	0
MAP1LC3A	1	0	1	0	0	1
MAP1LC3C	0	0	1	0	0	1
MAP2K1IP1	1	0	0	1	1	0
MAP2K3	0	0	0	1	0	0
MAP2K4	1	0	0	1	0	0
MAP2K5	0	0	1	0	0	1
MAP2K6	0	0	0	0	1	0
MAP2K7	1	0	0	1	0	0
MAP3K11	1	0	0	1	0	0
MAP3K14	1	0	0	0	0	0
MAP3K2	1	0	0	0	0	0
MAP3K6	0	1	0	0	0	0

MAP3K8	0	0	0	1	0	0
MAP4	0	0	1	0	1	1
MAP4K1	1	0	0	1	0	0
MAP4K2	1	0	0	0	0	0
MAP4K3	1	0	0	1	0	1
MAPBPIP	1	0	0	1	0	0
MAPK1	1	0	0	0	0	0
MAPK13	1	0	1	1	0	1
MAPK14	0	1	0	1	1	0
MAPK15	0	0	1	0	0	0
MAPK3	1	0	0	0	0	1
MAPK4	0	1	1	1	0	1
MAPK6	0	0	1	0	0	0
MAPK7	1	0	0	1	0	0
MAPK8	0	0	1	1	0	1
MAPK9	0	0	1	0	0	1
MAPKAP1	0	0	0	1	0	0
MAPKAPK2	1	1	0	1	0	0
MAPKAPK3	1	0	1	1	0	1
MAPKAPK5	1	0	0	1	0	1
MAPKBP1	1	0	0	1	0	0
MAPRE1	0	0	0	1	0	0
MAPRE3	1	0	0	0	0	1
MARCO	0	0	0	0	0	1
MARK2	0	0	1	0	0	0
MARS	1	0	0	1	1	0
MARVELD2	1	0	0	0	0	0
MAS1	0	0	1	0	0	1
MASK-BP3	1	0	0	1	0	0
MASTL	1	0	0	0	0	0
MAT2A	1	0	0	1	0	0
MATK	0	1	1	0	0	1
MATN1	0	1	1	1	1	1
MATR3	0	0	0	0	0	1
MAZ	1	0	0	1	0	0
MBD1	1	0	0	1	0	0
MBD3L1	0	1	1	0	0	1
MBD4	1	0	0	1	1	0
MBD5	0	0	1	0	0	1
MBD6	1	0	0	0	1	0
MBNL1	1	1	0	1	1	0
MBNL2	1	0	0	0	0	0
MBP	0	0	1	0	0	1
MBTPS1	0	0	0	1	0	0
MBTPS2	1	1	0	0	1	1
MC1R	0	0	0	0	0	1
MC2R	0	1	1	1	1	1
MCART1	1	0	0	1	0	0
MCART6	1	1	1	1	1	1
MCEE	1	0	0	1	0	0
MCFD2	1	0	0	1	0	0
MCHR1	0	0	1	0	0	0
MCM10	1	0	0	0	0	0
MCM2	0	0	0	1	0	0
MCM3AP	1	0	0	1	0	0
MCM4	1	0	0	1	1	0
MCM5	1	0	0	0	0	0
MCM7	1	0	0	1	0	0
MCOLN1	1	0	1	1	0	1

MCOLN3	0	0	1	0	0	1
MCRS1	1	0	0	1	0	0
MCTP2	0	0	1	0	0	0
MCTS1	1	0	0	1	0	0
MDC1	1	0	0	1	0	0
MDFIC	0	1	0	0	0	0
MDH1	1	0	1	1	0	1
MDH2	1	0	0	1	0	0
MDM1	1	1	0	1	1	0
MDM2	1	0	0	0	0	0
MDM4	0	0	0	1	0	0
MDN1	1	0	0	1	0	0
MDP-1	1	0	0	1	0	0
MDS032	1	0	0	1	0	0
ME1	1	0	0	1	0	0
ME2	1	0	0	0	1	0
ME3	0	0	1	0	0	0
MEA1	1	0	0	1	1	0
MECR	1	0	0	1	0	0
MED11	0	0	0	1	0	0
MED12	0	1	1	0	0	1
MED18	0	0	0	1	0	0
MED19	1	0	0	1	0	0
MED28	1	0	0	1	1	0
MED31	1	0	0	1	0	0
MED4	1	0	0	0	0	0
MED6	1	0	0	0	0	0
MED8	1	0	0	1	0	1
MED9	0	0	0	1	0	0
MEF2A	1	0	0	1	0	0
MEF2B	1	0	0	0	0	0
MEGF8	0	0	1	0	0	1
MEN1	1	0	0	0	0	0
MEOX1	0	0	1	0	0	1
MERTK	0	1	0	0	0	0
MESDC1	1	0	0	0	0	0
MESDC2	1	0	0	1	0	0
MESP1	0	0	0	1	0	0
MEST	1	0	0	0	0	0
MET	1	0	0	0	0	0
METAP1	0	0	0	0	0	1
METAP2	1	0	0	1	0	0
METT10D	1	1	1	1	0	1
METT5D1	1	1	0	1	1	1
METTL1	1	0	1	1	0	1
METTL2A	1	0	0	1	0	0
METTL2B	1	0	0	1	0	0
METTL4	1	0	0	0	0	0
METTL6	1	0	0	1	1	1
METTL7A	1	0	0	0	0	0
MFAP3	1	0	0	1	0	0
MFGE8	0	1	0	0	0	0
MFHAS1	1	0	0	0	0	0
MFN2	0	1	0	1	1	0
MFNG	0	0	0	1	0	0
MFRP	0	0	1	0	0	1
MFSD2	0	1	0	0	1	0
MGAT2	1	0	0	1	0	0
MGAT4A	1	0	1	1	1	1

MGAT4B	1	0	0	0	0	1
MGC11102	1	0	0	1	0	0
MGC14327	1	0	0	1	0	0
MGC14376	1	0	0	1	0	1
MGC15885	0	0	1	0	0	1
MGC16169	1	0	0	1	1	1
MGC16824	0	0	0	1	0	0
MGC20983	1	0	0	1	0	0
MGC21675	0	0	1	0	0	0
MGC2752	1	0	0	1	0	0
MGC3207	1	0	0	0	0	0
MGC33556	1	0	0	1	0	0
MGC34761	0	1	1	0	0	1
MGC35361	1	0	0	1	0	0
MGC3731	1	1	1	1	1	1
MGC39900	0	1	0	0	0	0
MGC40499	1	1	0	1	1	0
MGC4093	1	0	0	1	0	0
MGC4172	0	0	0	1	0	0
MGC42630	1	0	0	1	0	0
MGC45491	0	0	0	0	0	1
MGC4677	0	0	0	0	1	0
MGC52000	0	0	1	0	0	1
MGC52110	1	0	0	1	1	0
MGC59937	1	0	0	0	0	0
MGC70924	0	1	0	0	1	0
MGC71993	1	0	0	1	0	0
MGC72104	1	0	0	1	0	0
MGMT	0	0	0	1	0	0
MGRN1	1	0	0	0	0	0
MGST1	0	0	1	0	0	1
MGST2	1	0	0	1	0	0
MIA	1	0	1	1	1	0
MICA	1	0	0	0	0	0
MICAL2	0	0	0	0	1	0
MICALCL	0	0	1	0	0	1
MID1IP1	1	0	0	1	0	0
MID2	1	0	0	0	0	0
MIDN	1	0	0	1	0	0
MIER1	1	0	0	1	0	0
MIF	0	1	0	0	0	0
MIF4GD	1	0	0	1	0	0
MINA	1	0	1	0	1	1
MIPEP	1	0	0	1	0	1
MIS12	1	0	0	1	0	0
MITF	1	0	1	1	1	1
MIZF	0	0	0	0	1	0
MKKS	1	0	1	1	0	0
MKL1	0	1	0	0	1	0
MKNK1	0	0	0	1	0	0
MKS1	1	0	0	1	0	0
MLC1	0	1	0	1	0	0
MLH1	1	0	0	1	0	0
MLL	0	1	0	0	0	0
MLL3	1	0	1	1	0	1
MLLT10	1	0	0	0	0	0
MLLT11	1	0	0	1	0	0
MLLT3	0	1	0	0	0	0
MLSTD1	0	0	1	0	0	1

MLX	1	0	0	1	0	0
MLXIP	1	0	1	1	0	1
MMAA	0	0	1	0	0	0
MMAB	1	0	0	1	0	1
MMACHC	1	0	0	0	1	0
MMP1	0	1	1	0	0	1
MMP10	0	1	1	0	0	1
MMP12	0	1	1	0	0	1
MMP13	0	1	1	0	0	1
MMP14	1	0	0	0	0	0
MMP19	1	0	1	1	0	1
MMP21	0	0	1	0	0	1
MMP7	0	0	1	0	1	0
MMS19L	1	0	0	0	0	0
MND1	0	0	1	0	0	0
MNS1	1	0	0	1	0	0
MNT	1	0	0	1	0	0
MOAP1	1	0	0	1	0	1
MOBKL2B	1	0	1	1	0	1
MOBKL2C	0	0	0	0	1	0
MOCS2	1	0	0	0	0	0
MOCS3	1	0	0	1	0	1
MON1B	1	0	0	1	0	0
MORC4	0	1	1	1	0	0
MORF4L1	1	0	0	0	0	0
MORF4L2	0	1	0	0	0	0
MORG1	1	0	0	1	1	0
MOSPD2	1	1	0	0	1	0
MOV10	1	0	0	0	0	0
MPDU1	1	0	0	1	0	0
MPDZ	0	0	1	0	0	0
MPFL	1	0	0	0	0	1
MPHOSPH10	1	0	0	1	0	0
MPHOSPH6	1	0	0	0	0	0
MPHOSPH9	0	0	1	0	0	0
MPI	1	0	0	0	0	0
MPL	0	1	1	0	1	1
MPP1	0	1	0	0	0	0
MPP7	0	1	1	0	0	0
MPPED2	1	0	1	0	0	1
MPST	1	0	0	0	0	0
MPV17	1	0	1	1	0	1
MPZ	0	0	0	1	0	0
MR1	0	0	0	1	1	1
MRAP	0	0	1	0	0	0
MRAS	0	0	1	0	0	1
MRC1	1	0	0	0	0	1
MRC1L1	1	0	1	1	0	1
MRCL3	1	0	0	1	0	1
MRE11A	1	0	0	1	0	0
MRLC2	1	0	0	0	0	0
MRP63	1	0	0	1	1	0
MRPL11	1	0	0	1	0	0
MRPL12	1	1	0	0	1	0
MRPL13	1	0	0	1	1	1
MRPL14	1	0	0	1	0	0
MRPL17	1	0	0	1	0	0
MRPL18	1	0	1	1	0	1
MRPL19	0	0	0	1	0	0

MRPL2	1	0	0	1	0	0
MRPL20	1	0	0	1	0	0
MRPL21	1	0	0	1	1	0
MRPL22	1	0	0	1	0	0
MRPL24	1	0	0	1	0	0
MRPL27	1	0	0	1	0	1
MRPL30	1	1	1	1	1	1
MRPL32	1	0	0	1	0	0
MRPL33	0	0	0	1	0	0
MRPL34	1	0	0	0	0	0
MRPL35	1	0	0	0	0	0
MRPL37	1	0	0	1	0	0
MRPL38	1	0	0	1	0	0
MRPL39	1	0	0	0	0	0
MRPL40	1	1	0	1	0	0
MRPL41	1	0	0	1	0	0
MRPL42	0	0	1	0	1	1
MRPL43	1	0	0	1	0	0
MRPL44	1	0	0	1	0	0
MRPL46	1	0	0	1	1	0
MRPL47	1	0	0	1	0	0
MRPL48	1	0	0	1	1	0
MRPL49	1	0	0	1	1	0
MRPL50	1	0	0	1	0	1
MRPL51	1	0	0	1	0	0
MRPL52	1	0	0	1	0	0
MRPL53	1	0	0	1	0	0
MRPL55	0	0	0	1	0	0
MRPS11	1	0	0	1	1	0
MRPS12	1	0	0	1	0	0
MRPS14	0	0	0	0	1	0
MRPS15	1	0	0	0	0	0
MRPS16	1	1	0	1	1	0
MRPS17	0	0	0	1	0	0
MRPS18A	1	0	0	1	0	0
MRPS18B	1	0	0	1	0	0
MRPS18C	1	0	0	1	0	1
MRPS2	1	0	0	1	0	0
MRPS21	1	0	0	1	1	0
MRPS22	0	0	1	1	0	0
MRPS23	1	0	0	1	0	0
MRPS24	0	0	1	1	0	1
MRPS26	1	0	0	0	1	1
MRPS27	1	0	0	1	1	0
MRPS30	1	1	1	1	0	1
MRPS34	1	0	0	1	0	0
MRPS36	1	0	0	1	0	0
MRPS5	0	0	0	1	0	0
MRPS6	1	0	1	1	1	1
MRPS7	1	0	0	1	1	0
MRRF	1	0	0	1	0	1
MRVI1	1	1	1	1	1	1
MS4A2	0	1	1	0	1	1
MS4A3	0	1	0	0	0	0
MS4A6A	1	0	0	0	0	0
MS4A7	1	0	0	1	0	0
MSC	1	0	0	1	0	0
MSH3	1	0	0	1	1	0
MSI2	0	1	0	0	0	0

MSL2L1	1	0	0	0	0	0
MSL3L1	0	1	0	0	1	0
MSR1	1	0	0	1	0	0
MSRA	1	0	1	1	0	0
MST1	1	0	0	1	0	0
MSTO1	0	0	0	1	0	0
MT1B	1	0	1	0	0	0
MT1F	1	0	0	0	0	0
MT1G	1	0	0	0	0	0
MTA2	1	0	0	0	0	0
MTA3	0	0	0	1	0	0
MTCH1	1	0	0	0	0	0
MTCP1	1	1	0	0	0	0
MTERFD1	1	0	0	1	0	0
MTF1	1	0	0	1	1	0
MTF2	0	0	0	0	1	0
MTHFD2	1	0	0	1	0	0
MTHFD2L	0	0	1	1	0	1
MTHFR	1	0	0	1	1	0
MTHFS	0	1	0	0	1	0
MTHFSD	1	0	0	1	0	0
MTIF2	1	0	0	1	0	0
MTL5	1	1	0	0	0	0
MTM1	0	1	1	1	0	1
MTMR1	0	1	1	0	0	0
MTMR10	0	1	0	1	0	0
MTMR11	0	0	0	0	1	0
MTMR3	0	0	1	0	0	1
MTMR4	1	0	0	1	0	0
MTMR9	1	0	0	1	0	0
MTR	1	0	0	1	0	0
MTRF1	0	0	0	1	0	0
MTRF1L	1	0	0	1	1	0
MTRR	1	0	0	1	1	0
MTX1	1	0	0	1	0	0
MTX3	0	1	0	0	0	0
MUC13	0	0	1	1	0	1
MUC20	0	1	1	0	0	1
MUS81	1	0	0	1	0	0
MUSTN1	0	1	1	0	1	1
MUT	1	0	0	1	1	0
MUTYH	1	1	0	0	1	0
MVK	1	0	0	1	0	1
MVP	1	0	0	1	0	0
MX1	0	1	0	0	0	0
MXD1	1	0	0	0	0	0
MXD4	1	0	0	0	0	0
MXI1	1	0	0	1	0	0
MYADML	0	0	0	0	1	1
MYB	0	1	0	0	0	0
MYBBP1A	1	0	0	1	0	0
MYBPC1	0	0	0	0	0	1
MYBPC3	1	0	0	0	0	0
MYBPH	0	1	1	0	0	1
MYCBP	1	0	0	1	0	0
MYCBP2	1	0	0	0	0	0
MYD88	1	0	0	0	0	0
MYEOV	0	0	1	0	0	0
MYH6	0	1	1	0	0	1

MYH7	0	1	1	0	0	1
MYH9	1	0	0	0	0	0
MYL2	0	1	1	0	0	1
MYL5	0	0	1	1	0	0
MYL6B	0	0	0	1	1	0
MYL7	0	0	0	0	0	1
MYLIP	0	0	0	1	0	0
MYLK	0	1	1	0	0	1
MYLK2	0	0	1	0	0	0
MYO10	0	1	0	0	0	0
MYO18A	0	1	1	1	1	1
MYO1A	0	0	0	0	0	1
MYO1D	0	0	0	0	0	1
MYO6	0	0	0	1	0	0
MYO9A	1	1	0	0	0	0
MYO9B	1	0	1	1	0	1
MYOHD1	1	0	0	1	0	0
MYOM1	0	1	1	0	0	0
MYOZ1	0	0	1	1	0	1
MYST2	1	0	0	1	0	0
MYST4	1	0	1	1	0	1
N4BP1	0	1	0	0	1	0
N4BP2	0	0	1	0	0	1
NAALADL1	1	0	1	1	0	1
NAALADL2	0	0	1	0	0	0
NAB1	1	0	0	0	0	0
NACA	1	0	0	0	0	0
NADSYN1	1	1	0	1	0	1
NAGA	1	1	0	0	0	0
NAGK	1	0	0	1	0	0
NAGPA	1	0	1	1	0	1
NANOG	0	0	1	0	0	1
NANP	1	0	0	1	0	0
NAP1L1	0	0	0	1	0	0
NAP1L2	0	0	1	0	0	0
NAPG	0	0	0	1	1	0
NAPSA	1	0	0	1	0	0
NARF	1	0	0	0	0	0
NARG1	1	0	0	1	1	1
NARG2	1	0	0	0	0	1
NARS	0	0	0	1	0	0
NAT1	1	0	0	0	0	0
NAT10	0	1	0	0	0	0
NAT5	1	0	1	1	1	0
NAT6	1	0	1	1	0	0
NAT8	0	0	1	0	0	1
NAT9	1	0	0	1	0	0
NAV2	0	1	0	0	0	0
NBPF1	0	0	1	0	0	1
NBPF10	0	0	1	0	0	1
NBPF14	0	0	1	0	0	1
NBPF9	1	0	1	0	0	1
NCALD	0	1	1	0	0	1
NCBP1	1	0	0	1	0	0
NCBP2	1	0	0	1	0	0
NCDN	1	0	0	0	0	0
NCF1	1	0	0	0	1	0
NCF2	0	0	0	1	0	0
NCK1	1	0	0	0	0	0

NCK2	0	1	1	1	1	1
NCKIPSD	0	0	1	1	0	0
NCL	0	0	0	1	0	0
NCLN	1	0	0	1	0	0
NCOA1	1	0	0	0	0	0
NCOA3	1	0	0	0	1	0
NCOA4	1	0	1	1	0	1
NCOA5	1	0	0	1	0	0
NCR2	0	0	1	0	0	1
NCSTN	1	0	0	1	1	1
NDE1	1	0	0	1	0	0
NDN	0	0	1	0	0	0
NDNL2	0	1	0	0	0	0
NDP	0	0	0	1	0	0
NDRG3	0	1	1	0	1	0
NDRG4	1	0	1	1	0	1
NDST1	1	0	1	1	0	1
NDST2	0	0	0	1	0	0
NDUFA1	1	1	0	1	0	1
NDUFA10	1	0	1	0	0	1
NDUFA13	1	0	0	1	0	0
NDUFA2	1	0	0	1	0	0
NDUFA5	0	0	0	1	0	0
NDUFA6	0	0	0	1	0	0
NDUFA7	1	0	0	1	0	0
NDUFA8	0	1	0	0	0	0
NDUFA9	0	0	0	1	0	0
NDUFB1	1	0	0	0	0	0
NDUFB11	1	0	0	1	0	0
NDUFB2	0	1	1	0	0	0
NDUFB3	1	0	0	1	0	0
NDUFB5	1	0	0	1	0	0
NDUFB6	1	0	0	1	1	0
NDUFB7	0	0	0	1	0	0
NDUFB8	1	0	0	1	0	1
NDUFB9	1	0	1	1	0	1
NDUFC1	1	0	0	1	1	1
NDUFS1	1	0	0	1	0	0
NDUFS2	1	0	1	1	0	0
NDUFS3	1	0	0	1	1	1
NDUFS4	1	1	0	0	0	0
NDUFS8	1	0	0	1	0	0
NDUFV1	1	0	0	1	1	0
NEB	0	0	1	0	0	1
NECAP1	0	0	0	1	0	0
NEDD4	0	1	0	0	0	0
NEDD8	1	0	0	1	0	0
NEDD9	0	1	0	0	0	0
NEK1	1	0	0	1	0	0
NEK11	1	0	0	1	1	0
NEK3	1	0	1	0	0	0
NEK4	1	0	0	1	1	0
NEK6	1	0	0	1	0	1
NEK7	0	0	1	0	0	1
NEK9	1	0	0	1	0	0
NENF	0	0	0	1	0	0
NEO1	0	0	0	0	0	1
NET1	1	1	1	1	0	1
NETO2	1	0	0	1	0	0

NEU3	0	0	0	1	0	0
NEUROD6	0	0	1	1	0	1
NEXN	0	0	0	0	0	1
NF1	0	0	1	0	0	1
NFAM1	0	0	0	0	1	0
NFAT5	1	0	0	1	1	0
NFATC3	0	0	0	0	1	0
NFE2L1	0	0	0	1	0	0
NFE2L3	1	0	0	0	0	0
NFIC	1	0	0	0	0	0
NFKBIB	1	1	0	1	0	1
NFKBIE	0	0	0	1	0	0
NFKBIL1	1	0	1	1	0	1
NFKBIZ	1	0	0	1	0	0
NFRKB	1	0	0	0	1	0
NFS1	1	0	0	1	0	0
NFXL1	1	0	0	0	0	0
NFYA	1	0	0	0	0	0
NFYB	0	0	0	1	0	0
NGFRAP1	0	1	0	0	0	0
NGRN	1	0	0	1	0	0
NHEJ1	1	0	0	0	0	0
NHLH1	0	0	1	0	0	1
NHLRC1	1	0	0	0	0	0
NHLRC2	1	0	0	1	0	0
NHN1	1	0	0	1	0	0
NHP2L1	1	0	0	1	1	0
NIBP	0	1	1	0	0	1
NIF3L1	1	0	0	1	0	1
NIN	0	0	0	1	0	0
NIP30	1	0	0	1	0	0
NIP7	1	1	0	1	0	0
NIPBL	1	0	0	0	1	0
NIPSNAP3A	1	0	0	1	1	0
NIT1	1	0	0	1	0	0
NKAP	0	1	0	0	0	0
NKIRAS1	1	0	0	0	0	0
NKIRAS2	1	0	1	1	0	0
NLN	1	0	0	0	0	0
NMB	1	0	0	1	0	0
NMD3	0	0	1	1	0	0
NME1-NME2	0	0	0	0	1	0
NME3	1	0	0	1	0	0
NME4	0	0	0	1	0	0
NME6	1	0	0	0	0	0
NME7	1	0	0	1	1	0
NMI	0	0	0	0	1	0
NMNAT1	1	0	0	1	0	0
NMNAT2	0	0	0	1	0	1
NMT1	1	0	0	1	1	0
NNT	1	0	0	0	0	1
NOC3L	1	0	0	0	0	0
NOC4L	1	0	0	1	0	0
NOL1	0	0	0	1	1	0
NOL10	1	0	0	1	0	0
NOL11	1	0	0	1	0	0
NOL5A	1	0	0	1	0	0
NOL6	1	0	0	1	0	0
NOL7	1	0	0	1	0	0

NOL8	1	0	0	1	1	1
NOL9	1	0	1	1	1	1
NOLA2	1	0	0	1	0	0
NOLA3	1	0	0	1	0	0
NOLC1	0	0	0	1	0	0
NOMO1	1	0	0	1	0	1
NOMO3	1	0	0	1	0	1
NONO	0	1	1	0	0	1
NOP5/NOP58	0	0	0	1	0	0
NOSIP	1	0	0	1	0	0
NOTCH4	0	1	1	0	0	1
NOX1	0	1	1	0	0	0
NOXA1	1	0	0	1	0	0
NPAL2	0	0	0	0	0	1
NPAL3	0	0	0	0	1	0
NPAS1	0	0	0	0	0	1
NPAT	1	0	0	1	0	0
NPC2	1	0	0	1	0	0
NPFF	1	0	0	1	0	0
NPFFR1	0	1	1	0	0	1
NPHP3	1	0	0	1	0	0
NPHS2	0	0	0	0	0	1
NPIP	1	0	1	0	0	1
NPL	1	0	1	1	0	1
NPTN	1	0	0	1	0	0
NQO1	1	0	0	1	0	0
NQO2	1	0	0	1	0	0
NR1H2	1	0	0	1	0	0
NR1H3	1	0	1	1	1	1
NR1H4	0	1	1	0	1	1
NR1I2	0	0	1	0	1	1
NR2C2	1	0	0	0	1	1
NR2E3	0	0	1	0	0	1
NR3C1	1	1	1	1	0	1
NR4A1	1	0	1	0	0	1
NR4A3	1	0	0	0	0	0
NRAS	1	0	0	1	1	0
NRCAM	0	1	0	0	0	1
NRD1	0	0	0	1	0	0
NRF1	0	0	0	1	0	0
NRG2	0	1	0	0	0	0
NRIP1	0	0	1	0	0	0
NRL	1	0	0	1	1	0
NRM	1	0	0	1	0	0
NRP2	1	0	0	1	0	0
NRXN1	0	1	0	0	0	0
NRXN2	0	1	0	0	1	0
NRXN3	0	0	1	0	0	1
NSD1	0	0	0	0	0	1
NSDHL	1	1	1	1	1	1
NSFL1C	0	1	0	0	1	0
NSMAF	0	1	0	1	0	0
NSMCE1	0	0	1	1	0	1
NSUN2	1	0	0	1	0	0
NSUN3	1	0	0	1	0	0
NSUN5	1	0	0	1	0	0
NSUN5B	1	0	0	1	0	0
NSUN5C	1	0	1	1	1	1
NSUN6	1	0	0	0	0	0

NT5C2	0	0	0	0	1	0
NT5C3	0	0	0	1	0	0
NTAN1	0	0	0	1	0	0
NTHL1	1	0	0	1	0	0
NTNG2	1	0	0	0	0	0
NTSR1	0	0	0	1	0	0
NTSR2	0	0	1	0	0	0
NUAK2	0	0	0	1	0	0
NUBP1	1	0	0	1	0	0
NUBP2	1	0	0	0	0	0
NUBPL	1	1	1	1	0	0
NUCB2	0	1	0	0	0	0
NUCKS1	0	0	0	1	0	0
NUDCD1	1	0	0	1	0	0
NUDCD2	1	0	0	0	1	0
NUDCD3	1	0	0	0	0	0
NUDT1	1	0	0	1	0	0
NUDT15	0	0	0	1	0	0
NUDT16	1	0	1	1	0	0
NUDT16P	1	0	1	1	0	1
NUDT18	1	0	0	1	0	0
NUDT2	0	0	0	1	0	0
NUDT21	1	0	0	1	1	0
NUDT4	1	0	0	1	0	0
NUDT5	1	0	0	1	1	0
NUDT6	1	0	0	1	0	1
NUDT9	1	0	0	1	0	0
NUFIP2	1	0	0	1	0	0
NUMA1	1	0	0	1	0	0
NUMB	1	0	0	1	0	0
NUMBL	1	0	0	0	0	0
NUP107	1	0	0	1	0	0
NUP133	0	0	0	1	0	0
NUP155	1	0	0	1	0	0
NUP160	1	0	0	0	1	1
NUP188	1	0	0	1	0	0
NUP210	0	1	0	0	0	0
NUP43	1	0	0	1	0	0
NUP50	1	0	0	0	1	0
NUP54	0	0	0	1	0	0
NUP62	1	0	1	1	0	1
NUP85	0	0	0	1	0	0
NUP88	1	1	0	1	1	0
NUP93	1	0	1	1	0	0
NUPL2	1	0	0	1	0	0
NUSAP1	1	0	0	1	0	0
NUT	1	0	0	1	0	0
NUTF2	1	0	0	1	1	0
NVL	1	1	0	1	0	0
NXF1	0	0	0	0	1	0
NXF3	0	1	1	0	0	1
NXT2	0	1	1	0	1	1
NYD-SP21	0	0	0	1	0	0
OAF	0	1	0	0	1	0
OAS1	0	0	0	0	1	0
OAS3	0	0	0	1	0	0
OASL	0	0	0	1	0	0
OAZ1	1	0	0	1	0	0
OAZ2	0	0	0	0	1	0

OAZ3	0	0	1	0	0	1
OBFC2A	1	0	0	0	0	0
OBFC2B	1	0	1	1	0	1
OCM	1	0	1	0	0	1
OCRL	0	1	1	0	0	1
ODF2	1	0	0	1	0	0
ODZ1	1	1	1	1	0	1
OFCC1	0	0	1	0	0	1
OFD1	1	0	0	0	0	0
OGDHL	0	0	1	0	0	1
OGFOD1	1	0	1	1	1	1
OGFRL1	0	0	0	1	0	0
OGT	0	1	1	0	0	1
OIP5	1	0	0	1	0	0
OIT3	0	0	0	0	0	1
OKL38	0	0	1	0	0	1
OLFML2B	0	0	0	1	0	0
OLIG1	0	1	0	0	1	0
OLR1	1	0	1	1	0	1
OMA1	1	0	0	0	0	0
OMG	0	0	1	0	0	0
OMP	0	1	1	0	0	1
OPA1	0	0	0	1	0	0
OPA3	0	0	0	1	0	0
OPN1MW	0	0	1	0	0	1
OPN1SW	0	0	1	0	0	1
OPN3	1	0	0	1	0	1
OPN5	1	1	1	0	0	1
OPRS1	1	0	0	1	0	0
OPTC	1	0	1	0	0	1
OPTN	0	1	0	0	0	0
OR10G2	0	0	1	0	0	1
OR10G3	1	0	1	0	0	1
OR10G7	0	0	1	0	0	1
OR10G8	0	1	1	0	1	1
OR10K1	0	0	1	0	0	1
OR11A1	0	1	1	0	0	1
OR12D3	0	0	1	0	0	1
OR13H1	1	1	1	0	1	1
OR1A2	0	1	1	0	0	1
OR1F1	0	1	1	1	0	1
OR1L3	0	1	1	0	0	1
OR1L8	0	0	1	0	0	1
OR1N1	0	1	1	0	1	1
OR1Q1	0	1	1	0	0	1
OR2A14	0	1	0	1	0	1
OR2A2	1	1	1	1	1	1
OR2A20P	1	1	1	0	0	1
OR2A42	1	1	1	0	0	1
OR2AG1	0	1	1	0	0	1
OR2AG2	0	1	1	0	0	1
OR2D2	0	0	1	0	0	1
OR2F1	0	0	1	1	0	1
OR2G2	0	1	0	0	0	0
OR2H2	0	0	1	0	0	1
OR2J2	0	0	1	0	0	1
OR2J3	0	0	1	0	0	1
OR2L8	0	0	1	1	0	1
OR2M2	1	0	1	0	0	1

OR2T12	0	0	1	1	0	1
OR2T35	0	1	1	1	0	1
OR4A47	0	0	0	1	0	1
OR4C13	0	0	1	1	0	1
OR4C16	0	0	1	0	0	1
OR4D11	0	1	1	0	1	1
OR4D2	0	1	1	0	0	1
OR4F21	0	0	1	0	0	1
OR4S1	1	0	1	0	0	1
OR4X2	1	1	1	0	0	1
OR51B5	0	1	0	0	0	1
OR51G1	0	1	1	0	0	1
OR51G2	0	0	1	0	0	1
OR51I2	0	1	1	0	0	1
OR51M1	0	0	1	0	0	1
OR51V1	0	1	1	0	0	1
OR52A1	0	0	1	0	0	1
OR52A4	0	0	1	0	0	1
OR52B6	0	1	1	0	0	1
OR52D1	0	1	1	1	0	1
OR52E6	0	1	1	0	0	1
OR52I1	0	0	1	0	0	1
OR52K2	0	0	0	0	0	1
OR52W1	0	0	1	0	1	0
OR56A1	0	0	1	0	0	1
OR56A3	0	0	1	0	0	1
OR56B1	0	0	1	0	0	0
OR5AP2	1	0	1	1	0	1
OR5BU1	0	0	1	1	1	1
OR5M11	0	0	1	0	0	1
OR5M8	0	0	1	0	0	1
OR5U1	0	0	1	0	0	1
OR6C3	0	0	1	0	0	1
OR6C4	0	1	1	0	0	1
OR6C75	0	0	1	0	0	1
OR6K3	0	1	1	0	0	1
OR6S1	0	1	1	0	0	1
OR6T1	0	0	1	1	0	1
OR6V1	0	1	1	0	1	1
OR8B8	0	1	1	0	0	1
OR8G1	0	0	1	0	0	1
OR8G5	0	0	1	0	0	1
OR9A4	0	1	1	1	0	1
OR9Q2	1	1	1	0	0	1
ORC1L	1	0	0	1	0	0
ORC3L	1	0	0	1	0	0
ORC4L	1	0	0	1	0	1
ORC5L	0	0	0	1	0	0
ORC6L	1	0	0	0	1	0
ORM1	0	1	1	0	0	1
ORM2	0	1	1	0	0	1
ORMDL1	1	0	0	0	0	0
ORMDL2	1	0	0	1	0	0
ORMDL3	0	0	1	1	0	1
OS9	0	0	0	1	0	0
OSBP	0	0	0	1	0	0
OSBPL11	1	0	0	1	0	0
OSBPL1A	1	0	1	1	0	1
OSBPL5	0	1	0	0	0	0

OSBPL6	0	1	1	0	1	1
OSBPL7	1	0	0	0	1	0
OSBPL8	1	0	0	0	1	0
OSGEP	1	0	0	1	1	0
OSGEPL1	1	1	0	1	0	0
OSTF1	1	0	0	0	0	0
OSTBETA	1	0	0	0	0	0
OTOA	0	1	1	0	0	1
OTOF	0	0	0	0	0	1
OTOP3	0	0	0	1	0	0
OTUD4	0	0	1	0	0	1
OTUD5	1	1	0	0	1	0
OTUD6B	1	0	1	0	0	0
OVCA2	1	0	0	1	0	0
OVCH1	0	1	1	0	0	1
OVGP1	0	1	1	0	0	1
OXA1L	1	0	0	1	0	0
OXCT1	1	1	0	0	0	0
OXNAD1	1	0	0	1	0	1
OXR1	0	1	0	0	0	0
OXSM	0	0	0	1	0	0
OXSR1	0	0	1	0	0	0
P11	1	1	1	1	0	1
P117	1	0	0	1	0	0
P18SRP	1	0	0	1	0	1
P2RX4	1	0	0	1	1	0
P2RX7	0	0	0	1	0	0
P2RY10	0	1	1	0	0	1
P2RY2	0	0	0	1	0	0
P2RY4	0	1	1	0	1	1
P2RY6	0	0	1	0	0	0
P4HA1	0	0	0	1	0	0
P4HA3	0	1	0	0	0	0
PA2G4	1	0	0	1	0	0
PABPN1	1	1	0	1	0	0
PACS2	1	0	0	0	0	0
PADI1	0	0	1	0	0	1
PADI4	0	1	0	0	1	0
PAEP	0	0	1	0	0	1
PAF1	1	0	0	1	1	0
PAFAH1B3	0	1	0	0	1	0
PAFAH2	0	0	0	1	0	0
PAG1	0	0	0	0	1	0
PAICS	0	1	0	1	0	0
PAK1IP1	1	0	0	1	0	0
PAK2	0	0	0	1	0	0
PAK4	1	0	0	1	0	0
PAN3	0	0	1	1	0	1
PANK1	0	0	0	1	0	0
PANX3	1	0	1	0	0	1
PAPD1	0	1	0	0	0	0
PAPD5	1	0	0	1	0	1
PAPOLA	1	0	0	0	0	0
PAPPA2	0	1	1	1	0	1
PAPSS2	1	0	0	1	0	0
PAQR8	1	0	0	0	0	0
PARC	1	0	0	0	0	0
PARD6A	0	0	0	1	0	0
PARK7	1	0	0	1	0	0

PARN	0	1	0	0	0	0
PARP1	1	0	0	0	0	0
PARP14	0	0	1	0	0	1
PARP15	0	1	0	0	0	1
PARP16	0	0	0	1	0	0
PARP2	0	0	0	1	0	0
PARP3	1	1	0	1	1	0
PARP6	1	0	1	1	1	1
PARP9	0	1	0	0	0	0
PARS2	1	0	0	1	0	0
PARVB	1	1	0	1	0	0
PARVG	1	0	0	1	1	1
PASK	1	1	0	1	0	0
PAXIP1	1	0	0	0	0	0
PBX1	0	0	0	1	0	0
PBX3	1	0	0	0	0	0
PBXIP1	1	0	0	1	1	0
PC	1	0	1	1	0	1
PCAF	0	1	0	0	0	0
PCBD1	0	1	1	1	0	1
PCBP4	1	1	0	0	0	0
PCCB	0	1	0	0	0	0
PCDH12	1	0	0	0	0	0
PCDHAC2	0	0	1	0	0	0
PCDHB7	1	0	0	1	0	0
PCDHGA1	1	1	1	1	0	1
PCDHGB1	1	0	1	1	0	1
PCDHGC3	0	0	1	1	0	1
PCGF1	1	0	0	0	0	0
PCGF2	1	0	0	1	0	0
PCID2	1	0	0	1	0	0
PCM1	1	0	0	1	1	0
PCMT1	1	0	0	1	0	0
PCMTD1	0	0	1	0	0	0
PCMTD2	0	0	1	1	0	0
PCNA	1	0	0	1	0	0
PCNP	0	0	0	1	0	0
PCNT	1	0	0	1	0	0
PCNX	0	1	0	1	0	0
PCNXL3	1	0	0	1	0	0
PCSK7	1	0	0	1	1	0
PCTK1	1	1	0	0	0	0
PCTK2	1	0	0	0	0	0
PCTP	0	0	1	1	0	0
PCYOX1	1	0	0	0	0	0
PCYT1A	1	0	0	0	1	0
PDCD10	1	0	0	1	0	0
PDCD11	1	0	0	1	1	0
PDCD1LG2	1	0	1	0	0	1
PDCD2	1	0	0	1	0	0
PDCD2L	0	0	0	1	0	0
PDCD4	1	0	0	1	0	0
PDCD6IP	1	0	1	1	0	1
PDCD7	0	0	0	0	0	1
PDCL	1	0	0	1	0	0
PDCL2	0	0	1	0	0	0
PDE3B	1	0	0	1	0	1
PDE4A	0	0	1	0	0	1
PDE4B	0	0	1	0	0	0

PDE4D	0	0	1	0	0	1
PDE4DIP	1	0	1	1	0	1
PDE6B	0	0	1	0	0	1
PDE6D	1	0	0	1	0	1
PDE6G	0	0	1	0	0	0
PDE6H	0	0	1	0	0	0
PDE7A	0	0	0	0	0	1
PDE7B	0	0	1	0	0	1
PDGFB	0	0	0	0	0	1
PDGFRB	0	0	1	0	0	0
PDHA1	0	0	1	1	0	1
PDHX	1	0	0	1	0	0
PDIA4	1	1	0	1	0	1
PDIA6	1	0	0	1	0	0
PDIK1L	1	0	0	1	0	0
PDK1	1	0	0	1	0	0
PDK3	0	1	0	0	0	0
PDK4	0	1	0	0	0	0
PDP2	1	0	0	0	0	0
PDPR	1	0	0	1	0	0
PDRG1	1	0	0	1	0	0
PDSS1	1	0	0	0	0	0
PDXP	1	0	0	1	0	0
PDZD11	0	1	1	1	0	1
PEA15	0	0	0	1	0	0
PEBP1	1	0	0	1	0	0
PEBP4	0	1	1	0	0	1
PECI	1	1	0	0	0	0
PEF1	1	0	0	1	0	0
PEO1	1	0	0	1	0	0
PEPD	1	0	0	0	0	0
PER3	0	0	1	0	0	1
PERLD1	0	0	0	1	0	0
PERQ1	1	0	0	1	0	0
PES1	0	0	1	1	0	1
PET112L	0	1	1	0	1	1
PEX1	1	1	0	1	0	0
PEX11B	1	0	0	1	0	1
PEX11G	0	0	0	1	0	0
PEX13	1	0	0	1	0	0
PEX14	0	0	0	1	0	0
PEX19	1	0	0	1	0	0
PEX26	1	0	0	1	0	0
PEX3	0	0	0	1	0	0
PEX5	1	0	0	0	0	0
PEX6	0	0	0	0	1	0
PEX7	1	0	0	0	0	0
PFAAP5	1	1	0	1	0	0
PFAS	1	0	0	1	0	0
PFDN1	0	0	0	0	0	1
PFDN2	1	0	0	1	0	0
PFDN4	1	0	0	1	1	0
PFDN5	1	0	0	1	1	0
PFDN6	1	0	0	1	1	0
PFKFB2	0	0	0	1	0	0
PFKM	1	0	0	1	0	1
PFKP	0	0	0	1	0	0
PFTK1	0	0	1	0	0	1
PGAM1	1	0	0	0	0	0

PGAM4	0	1	1	1	0	1
PGBD2	1	0	0	1	0	0
PGBD3	1	0	1	0	0	0
PGBD4	1	0	0	1	0	0
PGBD5	0	1	1	0	0	1
PGC	0	0	1	0	1	1
PGD	1	0	0	1	0	0
PGDS	0	0	1	1	1	1
PGGT1B	1	0	0	1	0	0
PGK1	1	0	0	0	0	0
PGK2	0	0	1	1	0	1
PGLS	1	0	0	0	1	0
PGLYRP4	0	1	1	0	0	0
PGM1	0	1	0	0	1	0
PGM2L1	0	1	0	0	0	0
PGM3	0	0	0	1	0	0
PGPEP1	0	0	0	0	1	0
PHACS	1	0	0	1	0	0
PHACTR1	0	1	1	0	0	1
PHACTR2	0	0	0	0	0	1
PHACTR4	0	0	1	1	1	1
PHB	1	0	0	0	0	0
PHB2	1	0	0	1	0	0
PHC1	1	0	0	0	0	0
PHC2	0	0	1	0	0	1
PHF1	1	0	0	0	0	0
PHF11	0	0	0	0	1	0
PHF12	1	0	0	1	1	0
PHF13	1	0	0	1	0	0
PHF14	1	0	0	0	0	0
PHF17	1	0	0	1	0	0
PHF19	0	1	0	0	1	0
PHF2	0	0	0	0	0	1
PHF20	1	0	1	1	0	0
PHF20L1	1	0	0	0	0	0
PHF21A	1	0	0	0	0	0
PHF23	1	0	0	1	1	0
PHF3	1	0	0	1	0	0
PHF5A	1	0	0	1	0	0
PHF7	1	0	0	1	1	0
PHGDH	1	1	0	0	0	0
PHIP	1	0	0	0	0	0
PHKA2	1	1	0	1	0	0
PHKB	1	0	0	1	0	0
PHLDA1	1	0	0	1	0	0
PHLDA3	0	0	0	1	0	0
PHLDB1	0	0	0	1	0	0
PHLDB3	0	1	0	0	1	0
PHLPP	1	0	0	1	0	1
PHLPPL	1	0	1	0	0	0
PHOSPHO2	0	0	0	1	0	0
PHTF2	1	0	0	0	0	1
PHYH	1	0	0	0	0	0
PHYHIPL	0	0	1	0	0	0
PI4K2B	1	0	0	0	0	0
PIAS1	1	0	0	1	0	0
PIAS4	0	0	0	1	0	0
PIB5PA	0	0	1	0	0	1
PICALM	1	0	0	1	0	0

PIGB	1	0	0	0	0	0
PIGC	1	0	1	1	0	0
PIGF	1	0	0	1	1	0
PIGG	1	0	0	1	0	0
PIGM	0	1	0	1	0	0
PIGN	1	0	0	0	0	0
PIGO	1	0	0	1	0	0
PIGP	1	0	0	0	0	0
PIGS	1	0	0	1	1	0
PIGT	1	0	0	1	0	0
PIGV	0	0	0	0	1	0
PIGW	1	0	0	1	0	0
PIGX	1	0	0	1	0	0
PIGY	0	1	0	1	0	0
PIGZ	1	0	0	1	0	0
PIK3C2A	1	0	1	0	0	0
PIK3C3	0	0	0	1	0	0
PIK3CB	0	0	1	0	0	1
PIK3CD	0	1	0	0	0	0
PIK3CG	0	0	0	0	1	0
PIK3R1	1	1	1	0	1	1
PIK3R4	1	0	0	0	0	0
PIK3R5	0	0	1	0	0	1
PIK4CA	1	0	1	1	1	1
PILRB	0	0	0	1	0	0
PIM1	0	0	0	1	0	0
PIM2	1	1	0	0	0	0
PIN4	1	0	0	1	0	0
PIP3-E	1	1	0	1	0	0
PIP5K1A	1	0	0	0	0	0
PIP5K1C	1	0	0	0	0	0
PIP5K2B	1	0	0	1	0	0
PIP5K3	1	0	0	0	0	0
PIR	0	0	1	1	0	1
PITPNA	1	0	1	1	0	1
PITPNB	1	0	0	1	0	0
PITX3	0	0	0	1	0	0
PIWIL4	0	0	1	0	0	0
PKD1	1	0	0	0	0	0
PKD1L1	1	0	1	1	0	1
PKD2	0	0	0	1	0	0
PKD2L1	1	0	1	0	1	1
PKIB	1	0	0	1	0	1
PKLR	0	0	1	0	0	1
PKM2	1	0	0	1	0	1
PKN1	1	0	0	1	0	0
PKP2	0	1	0	0	0	0
PKP4	0	1	0	0	0	0
PLA1A	0	0	1	0	0	1
PLA2G10	0	0	1	0	0	0
PLA2G12A	0	0	0	1	0	0
PLA2G12B	0	1	1	0	0	1
PLA2G2E	0	1	1	0	0	1
PLA2G4A	0	0	0	1	0	0
PLA2G4B	1	0	0	0	0	0
PLA2G5	0	0	1	0	0	1
PLA2G6	0	1	1	0	0	1
PLA2G7	1	0	0	1	0	0
PLAA	0	0	1	1	1	1

PLAC1	0	1	1	0	0	1
PLAC4	0	1	1	0	1	1
PLAG1	1	0	0	0	0	0
PLAGL2	1	0	0	1	0	0
PLAU	1	0	1	1	0	1
PLAUR	1	0	0	1	0	1
PLB1	0	1	1	0	1	1
PLCB1	1	1	0	0	0	0
PLCB3	1	0	0	1	0	0
PLCG2	1	0	1	0	0	1
PLCL1	0	1	1	0	0	1
PLCL2	0	0	1	0	0	1
PLCXD3	0	0	1	0	0	1
PLD1	0	0	1	0	0	1
PLD2	1	0	0	1	0	0
PLD3	1	0	0	0	0	1
PLDN	1	0	0	1	0	0
PLEC1	1	0	0	0	1	0
PLEKHA1	1	1	0	1	0	0
PLEKHA9	1	0	0	0	0	0
PLEKHB1	0	0	0	0	0	1
PLEKHB2	1	0	0	1	0	0
PLEKHG2	0	0	0	0	1	0
PLEKHG4	1	0	0	0	0	0
PLEKHG5	1	0	1	0	1	1
PLEKHJ1	0	0	0	1	0	0
PLEKHM1	1	0	0	1	0	0
PLEKHM2	0	0	1	0	0	1
PLK3	0	0	0	1	0	0
PLOD3	1	0	0	1	0	0
PLP2	0	0	0	1	0	0
PLXDC2	1	0	0	0	0	0
PLXNA1	0	0	0	0	0	1
PLXNC1	0	1	0	0	0	0
PLXND1	1	0	0	0	0	0
PMCHL1	0	1	1	0	0	1
PMF1	0	0	0	1	0	0
PMFBP1	0	0	1	0	0	1
PMM2	1	0	0	1	1	0
PMP22	0	0	1	1	0	1
PMP22CD	0	0	1	1	0	1
PMPCA	1	0	0	1	1	0
PMPCB	1	0	0	1	0	0
PMS1	1	0	0	0	0	0
PMS2	1	1	1	1	0	1
PMS2L2	1	1	1	1	0	1
PMS2L5	1	1	0	0	0	0
PMVK	1	1	0	0	1	0
PNKD	1	0	0	0	0	0
PNLDC1	0	0	1	0	0	1
PNLIPRP1	0	1	1	1	0	1
PNLIPRP2	0	0	1	0	0	1
PNMA1	1	0	0	1	0	0
PNMA5	0	1	0	0	0	1
PNOC	0	1	1	0	0	1
PNPLA1	0	1	1	0	0	1
PNPLA4	0	1	0	1	0	0
PNPLA5	0	0	1	0	0	1
PNPO	0	0	0	0	1	0

PNPT1	1	0	0	1	0	0
PODN	0	0	0	1	0	0
PODXL2	1	0	0	0	0	0
POFUT1	1	0	0	1	0	0
POFUT2	1	0	0	0	0	0
POGK	1	0	1	1	0	0
POGZ	0	0	0	1	0	0
POLA2	1	0	0	0	0	0
POLD3	0	0	0	1	0	0
POLD4	0	0	0	1	0	0
POLDIP2	1	0	0	1	1	1
POLE	1	0	0	1	1	0
POLE3	1	1	0	1	0	0
POLE4	0	1	0	0	0	0
POLG2	1	0	0	1	0	0
POLH	1	0	0	1	0	0
POLI	0	0	0	1	0	0
POLL	1	0	0	1	0	0
POLR1A	1	1	0	1	0	1
POLR1C	1	1	0	1	0	0
POLR2A	1	0	0	0	0	0
POLR2B	1	0	0	0	0	0
POLR2C	1	0	0	1	0	0
POLR2G	1	0	0	1	0	0
POLR2I	1	0	0	1	1	0
POLR2J	0	0	1	1	0	1
POLR2K	1	0	0	1	0	0
POLR2L	1	0	0	1	0	0
POLR3A	1	0	0	1	0	0
POLR3B	1	0	1	1	0	1
POLR3C	1	0	0	1	0	0
POLR3D	1	0	0	1	0	0
POLR3E	1	0	0	0	0	0
POLR3F	1	0	0	1	0	0
POLR3K	1	0	0	1	1	0
POMGNT1	1	0	0	1	0	0
POMT1	0	0	0	1	0	0
POMT2	1	0	1	1	0	0
POMZP3	1	0	0	1	0	0
POP1	1	0	0	1	1	0
POPDC2	0	0	1	0	0	1
PORCN	0	1	0	0	1	0
POU2F1	1	0	0	1	0	0
POU5F1	1	0	1	0	1	1
PPA1	1	0	0	0	1	0
PPA2	0	0	0	0	1	0
PPAN	1	0	1	0	0	1
PPAP2B	1	0	0	1	0	0
PPAPDC1B	1	0	0	0	0	0
PPAPDC2	0	1	0	0	0	0
PPAPDC3	0	0	1	0	0	0
PPARBP	1	0	0	1	0	0
PPARD	0	0	0	1	0	1
PPARG	1	0	1	1	0	1
PPARGC1A	0	0	1	0	0	1
PPAT	1	1	0	1	0	0
PPBP	0	0	1	0	0	1
PPCS	1	0	0	1	0	0
PPEF1	0	1	1	0	1	1

PPEF2	0	1	0	0	0	0
PPFIA4	0	0	1	0	0	1
PPFIBP2	0	0	0	1	0	0
PPHLN1	1	0	0	1	0	0
PPID	1	0	0	0	0	0
PPIF	0	0	0	0	0	1
PPIH	1	0	0	1	0	0
PPIL1	1	0	0	1	0	0
PPIL2	0	0	0	1	0	0
PPIL3	1	0	0	1	0	1
PPM1A	1	0	0	0	0	0
PPM1B	0	0	0	1	1	0
PPM1D	0	0	0	0	1	0
PPM1F	0	0	1	0	0	1
PPM1G	1	1	0	1	1	0
PPM1K	0	0	0	0	1	0
PPM1M	1	0	0	0	0	0
PPM2C	0	1	0	0	0	0
PPME1	1	1	0	1	1	1
PPOX	1	0	0	1	0	0
PPP1CB	0	0	0	0	1	0
PPP1R10	1	0	0	1	0	0
PPP1R11	1	0	0	1	0	0
PPP1R12A	1	0	1	1	0	1
PPP1R12C	0	0	0	1	0	0
PPP1R13L	1	0	0	1	0	0
PPP1R14B	1	0	0	1	0	0
PPP1R15B	1	0	0	1	0	0
PPP1R16A	1	0	1	0	0	1
PPP1R1B	1	0	0	0	0	0
PPP1R3D	1	1	1	1	0	0
PPP1R3F	0	1	1	0	1	0
PPP1R7	1	1	1	1	0	0
PPP1R8	1	0	0	1	0	0
PPP2CB	0	0	1	0	0	0
PPP2R1A	1	0	0	0	0	0
PPP2R1B	0	1	0	0	0	0
PPP2R2A	0	0	0	1	0	0
PPP2R2B	1	0	1	1	0	1
PPP2R2D	0	0	1	0	0	1
PPP2R3A	0	0	1	0	0	0
PPP2R3B	0	0	0	1	1	0
PPP2R4	1	1	0	0	0	0
PPP2R5C	1	0	0	1	0	0
PPP3CA	0	1	0	0	0	0
PPP3CB	1	0	1	1	0	1
PPP3CC	0	0	0	1	0	0
PPP3R1	1	0	0	0	0	0
PPP4R1L	1	0	0	1	0	0
PPP4R2	1	0	1	1	0	1
PPP5C	0	1	0	0	0	0
PPP6C	1	1	0	1	0	0
PPRC1	1	0	0	1	0	0
PPT2	1	0	0	0	0	0
PPWD1	1	0	0	1	0	0
PQBP1	0	1	1	1	1	0
PQLC1	0	0	0	1	0	0
PRAF2	0	1	0	0	1	0
PRAME	0	0	1	0	0	1

PRB4	0	1	1	1	1	1
PRCC	1	0	0	1	1	0
PRCP	1	0	0	0	0	0
PRDM1	1	0	0	0	0	0
PRDM10	1	1	1	1	0	1
PRDM15	1	0	0	1	0	0
PRDM4	1	0	0	0	0	0
PRDM7	0	0	1	1	0	1
PRDX1	1	0	0	0	0	0
PRDX5	1	0	0	1	0	0
PRDX6	1	0	0	1	1	0
PREI3	0	0	0	0	1	0
PREP	0	0	0	1	0	0
PREPL	1	0	0	1	0	0
PREX1	1	0	0	1	0	0
PRF1	0	1	1	0	1	1
PRH1	0	0	1	0	0	1
PRIM1	1	0	0	1	0	0
PRIM2A	0	0	0	1	0	0
PRKAB2	1	0	0	1	0	0
PRKCA	0	0	0	0	0	1
PRKCB1	0	1	0	0	0	0
PRKCE	0	1	0	0	0	0
PRKCH	0	1	0	0	0	0
PRKCI	0	0	0	1	0	0
PRKCQ	0	1	0	0	0	0
PRKCSH	1	0	0	1	0	0
PRKD3	1	0	0	0	1	0
PRKDC	1	0	0	1	1	0
PRKRIR	1	1	0	1	0	0
PRMT2	1	0	0	0	0	0
PRMT3	1	0	0	0	0	0
PRMT5	1	0	0	1	1	0
PRMT7	1	0	0	1	1	0
PRNPIP	0	0	0	0	1	0
PROS1	0	0	0	1	0	0
PROSC	0	0	0	1	0	0
PRPF18	0	0	0	1	0	0
PRPF19	0	0	0	1	0	1
PRPF31	1	0	0	1	0	0
PRPF38A	1	0	0	1	0	0
PRPF38B	1	0	0	1	0	0
PRPF39	1	0	0	0	0	0
PRPF4	1	1	0	1	0	0
PRPF4B	1	0	0	1	0	0
PRPF6	1	0	0	0	0	0
PRPF8	1	0	0	0	0	0
PRPS1	0	1	1	0	0	1
PRPS1L1	0	0	1	0	0	1
PRPS2	0	0	0	0	0	1
PRPSAP1	0	0	0	1	0	0
PRPSAP2	1	0	0	1	0	0
PRR11	1	0	0	1	0	0
PRR14	0	0	0	0	1	0
PRR3	1	0	0	1	0	0
PRR4	0	0	1	0	0	1
PRR5	0	0	1	0	0	0
PRR8	1	0	0	0	0	1
PRRG1	0	1	1	0	1	1

PRRG2	1	0	0	1	0	0
PRRG4	0	1	0	0	0	0
PRRT2	1	0	0	1	0	0
PRRT3	0	0	1	0	0	0
PRSS2	0	1	1	0	1	1
PRSS21	0	0	1	0	0	1
PRSS3	0	1	1	0	0	1
PRUNE	1	0	1	1	1	1
PRX	1	0	0	0	0	1
PSCD1	1	0	0	1	0	0
PSCD2	1	0	0	0	0	0
PSCD3	0	0	0	1	0	0
PSCDBP	0	0	0	1	0	0
PSD3	0	0	1	0	0	1
PSEN1	0	0	1	1	0	0
PSEN2	1	0	0	1	0	0
PSENEN	1	0	0	1	1	0
PSG9	0	0	1	0	0	1
PSMA1	1	0	0	1	0	1
PSMA2	1	0	1	1	0	0
PSMA3	0	0	0	1	0	0
PSMA4	1	0	0	0	0	0
PSMA5	1	0	0	1	0	0
PSMA6	1	0	0	1	0	0
PSMA7	1	0	0	1	0	0
PSMA8	1	1	0	1	0	0
PSMB1	1	0	0	0	0	0
PSMB10	1	0	0	1	1	0
PSMB3	1	0	0	1	0	0
PSMB6	0	0	0	1	0	0
PSMB7	1	0	0	1	0	0
PSMB8	1	0	0	1	0	0
PSMB9	0	0	0	0	1	0
PSMC1	0	0	1	0	0	0
PSMC3	1	0	0	1	0	0
PSMC3IP	1	0	0	1	0	0
PSMC4	1	0	0	0	0	0
PSMC5	1	0	0	1	0	0
PSMC6	1	0	0	0	0	0
PSMD1	1	0	0	1	0	0
PSMD10	1	0	0	1	0	1
PSMD13	1	0	0	1	0	0
PSMD14	1	0	0	0	0	0
PSMD2	1	0	0	0	0	0
PSMD5	1	0	0	0	0	0
PSMD7	1	0	0	1	0	0
PSMD9	1	0	0	1	0	0
PSME2	1	0	0	1	0	0
PSME3	1	0	0	1	1	0
PSMF1	1	0	1	1	0	1
PSORS1C1	0	1	1	0	0	1
PSORS1C2	0	0	0	0	0	1
PSPC1	1	0	0	1	0	0
PSPH	1	0	0	1	0	0
PSTPIP2	1	0	0	0	0	0
PTAFR	0	0	0	1	0	0
PTCD1	1	0	0	0	0	0
PTCD2	1	0	0	1	1	0
PTDSS1	1	0	0	1	0	0

PTDSS2	1	0	0	0	0	0
PTEN	0	1	0	0	0	0
PTGER2	1	0	0	0	0	0
PTGER4	1	0	0	1	0	0
PTK2B	1	0	0	1	1	0
PTP4A3	0	0	1	0	0	1
PTPLAD1	1	0	0	0	0	0
PTPLAD2	1	1	0	0	0	1
PTPLB	1	0	0	1	0	0
PTPN1	1	0	0	0	1	0
PTPN12	1	0	0	0	0	0
PTPN18	0	0	0	1	0	0
PTPN2	1	0	0	0	0	0
PTPN22	0	0	0	1	0	0
PTPN3	1	0	1	1	0	1
PTPN5	0	1	1	0	0	1
PTPN6	1	0	1	0	0	0
PTPN7	1	0	0	0	0	0
PTPRA	1	0	1	0	1	1
PTPRCAP	0	0	1	0	1	0
PTPRE	1	1	0	0	1	0
PTPRO	1	0	0	1	0	0
PTPRR	0	0	1	0	0	1
PTRH1	0	0	1	1	0	0
PTS	0	0	0	1	0	0
PTTG1	1	0	0	1	0	0
PTX3	0	1	0	0	0	0
PUM2	1	0	1	1	0	0
PUS7L	1	0	0	0	0	0
PVR	1	0	0	0	0	0
PVRL4	0	0	1	0	0	1
PWP1	1	0	0	1	0	0
PXMP2	1	0	0	1	1	0
PXN	0	0	0	1	0	1
PXT1	0	1	1	0	0	0
PYCARD	1	0	0	0	0	0
PYCR2	0	0	0	1	0	0
PYGM	1	1	0	0	1	0
PYGO2	1	0	0	1	1	0
PYHIN1	0	1	0	0	0	1
QARS	1	0	0	1	1	0
QDPR	0	0	0	1	0	0
QPCT	1	0	0	0	0	0
QPCTL	1	0	0	0	0	0
QRICH1	1	0	0	1	0	0
QRSL1	1	0	0	1	1	0
QTRT1	1	0	0	0	0	0
QTRTD1	1	0	0	1	0	0
R3HDM1	1	0	0	1	1	0
R3HDM2	0	0	1	1	0	1
R3HDML	0	0	1	0	0	1
RAB10	1	0	0	1	0	0
RAB11FIP1	1	0	0	0	1	0
RAB11FIP2	1	0	0	1	1	1
RAB11FIP3	1	0	0	0	0	1
RAB12	1	0	0	0	0	0
RAB13	1	0	0	1	0	0
RAB20	1	0	0	1	0	1
RAB21	0	0	0	1	0	0

RAB22A	1	0	0	1	0	0
RAB23	1	0	0	0	0	0
RAB24	0	0	0	0	1	0
RAB27A	0	0	0	0	0	1
RAB28	1	0	0	1	0	0
RAB2B	1	0	0	1	0	0
RAB30	1	0	0	1	0	0
RAB31	0	1	0	0	0	0
RAB32	0	0	1	0	0	0
RAB33B	1	0	0	1	0	0
RAB35	1	0	0	1	0	0
RAB36	1	0	0	1	0	0
RAB37	0	1	1	1	0	1
RAB40C	0	0	1	1	0	1
RAB42	1	0	0	0	0	0
RAB4A	1	0	0	1	0	0
RAB4B	1	0	1	1	1	0
RAB5A	1	0	0	1	1	0
RAB5B	1	0	0	1	0	0
RAB5C	1	1	0	1	0	1
RAB6A	1	0	0	0	0	0
RAB7L1	1	0	0	0	0	0
RABGAP1	0	1	0	0	0	1
RABGAP1L	1	0	0	0	0	1
RABGGTA	1	0	0	1	0	0
RABGGTB	1	0	1	1	1	1
RABIF	1	0	0	1	0	0
RABL2A	1	0	0	1	0	0
RABL2B	1	0	0	1	0	1
RABL3	0	1	1	1	1	1
RABL4	1	0	0	0	0	1
RACGAP1	1	0	1	1	0	0
RAD1	1	0	0	1	0	0
RAD17	1	0	0	1	0	0
RAD18	0	0	0	1	0	0
RAD23A	1	0	1	1	0	1
RAD23B	1	0	0	0	0	0
RAD50	0	0	1	0	0	0
RAD51	1	0	0	1	0	0
RAD51AP1	1	0	0	1	1	1
RAD51C	1	0	0	1	0	1
RAD51L3	1	0	1	0	0	0
RAD54L	1	0	0	0	0	0
RAD54L2	1	0	1	0	1	1
RAI1	0	0	0	0	0	1
RALA	1	0	0	1	0	0
RALB	1	0	0	1	0	0
RALGDS	0	0	0	0	0	1
RALGPS1	1	0	0	0	0	0
RALY	0	0	0	1	1	0
RAMP1	1	0	0	0	0	0
RANBP1	1	0	0	1	0	0
RANBP10	1	0	0	1	0	0
RANBP3	0	0	1	0	1	1
RANBP5	0	0	1	0	0	1
RANBP6	0	0	0	0	1	0
RANBP9	1	0	0	0	0	0
RAP1A	1	0	0	0	0	0
RAP1B	1	0	0	0	0	0

RAP2C	1	1	0	1	0	0
RAPGEF1	1	0	1	1	0	1
RAPGEF3	1	0	0	1	0	0
RAPGEF6	0	0	0	0	1	0
RAPH1	1	0	0	0	0	0
RAPSN	0	0	1	0	0	1
RARA	1	0	0	0	1	1
RARRES1	0	0	0	0	0	1
RARS	1	0	0	1	0	0
RASAL2	1	0	1	1	0	0
RASD2	0	0	0	0	0	1
RASGEF1B	0	0	0	1	0	0
RASGRF1	0	0	1	1	0	1
RASGRP1	1	0	0	0	0	0
RASGRP2	0	1	0	0	1	0
RASGRP4	0	0	0	1	0	0
RASL10B	0	0	1	1	0	0
RASL11B	0	0	0	0	0	1
RASSF1	1	0	0	1	0	0
RASSF2	0	1	1	0	1	1
RASSF4	1	0	1	0	1	1
RASSF5	1	0	0	1	0	1
RASSF7	0	0	1	0	0	0
RAVER1	1	0	0	1	0	0
RAXL1	0	1	1	0	0	1
RB1	0	0	0	1	0	0
RB1CC1	1	0	0	1	0	0
RBAK	1	0	0	1	0	0
RBBP4	1	0	0	0	1	0
RBBP5	1	0	1	1	0	0
RBBP6	1	0	0	0	0	0
RBBP8	1	0	0	0	0	0
RBED1	1	0	0	1	0	1
RBJ	1	0	0	1	0	0
RBKS	1	0	0	1	0	0
RBL2	1	0	0	0	0	0
RBM10	1	0	0	1	0	0
RBM12	1	0	1	1	0	1
RBM13	1	0	0	1	0	0
RBM14	1	0	0	1	1	0
RBM15B	1	0	0	1	1	0
RBM16	1	0	0	0	1	0
RBM17	0	0	0	1	0	0
RBM18	1	0	0	1	0	1
RBM22	1	0	0	1	0	0
RBM23	1	0	0	1	1	0
RBM25	1	0	0	0	0	0
RBM28	1	0	0	1	0	0
RBM3	0	0	0	0	1	0
RBM33	0	0	0	0	0	1
RBM34	1	0	0	0	0	0
RBM4	1	0	0	1	1	0
RBM7	1	0	0	1	1	0
RBM8A	1	0	0	1	0	0
RBMX	1	0	0	1	0	0
RBMX2	0	1	1	0	1	0
RCBTB1	0	0	0	1	0	0
RCBTB2	1	0	0	1	0	1
RCE1	1	0	0	0	0	0

RCHY1	1	1	0	1	0	0
RCL1	0	0	0	0	1	0
RCN2	0	0	0	1	0	0
RCN3	0	0	0	1	0	0
RCOR3	1	0	0	0	0	0
RCP9	1	0	0	1	0	0
RDBP	1	0	0	1	0	0
RDH10	1	0	0	1	0	0
RDH11	1	0	0	1	0	1
RDH14	0	1	0	0	0	0
RDH5	0	0	1	0	0	1
RDH8	1	0	0	0	0	0
RDHE2	0	0	1	0	0	0
RDM1	0	1	0	0	0	0
RDX	0	0	0	1	0	0
RECQL	1	0	1	1	0	1
RECQL4	1	0	0	1	1	0
RECQL5	1	0	0	1	0	1
REEP3	1	0	0	0	0	1
REEP4	0	0	0	1	0	0
REEP5	0	0	0	1	0	0
REG1A	0	0	1	0	0	1
REP15	0	0	1	0	0	0
REPIN1	1	0	0	1	0	0
REPS1	0	0	0	1	0	0
REPS2	1	0	0	0	0	0
RER1	1	0	0	1	0	0
RETNLB	0	0	1	0	0	1
REV3L	1	0	0	1	0	0
REXO2	1	0	0	1	0	0
RFC1	0	0	0	1	0	0
RFC3	0	0	0	1	0	0
RFC5	0	0	0	1	0	1
RFFL	0	0	0	0	1	0
RFK	1	0	0	0	0	0
RFNG	1	0	0	1	0	0
RFP	0	0	0	1	0	0
RFT1	0	0	0	1	0	0
RFWD2	1	0	0	0	0	0
RFWD3	1	0	0	1	0	0
RFX2	1	0	0	0	0	0
RFX4	0	1	1	0	0	1
RFXANK	1	0	0	1	0	0
RFXAP	0	1	0	0	0	0
RFXDC2	0	0	1	0	0	1
RG9MTD1	1	0	0	0	0	0
RGAG1	0	1	1	0	1	1
RGAG4	0	0	1	0	0	1
RGL1	1	0	1	1	0	1
RGL2	1	0	0	1	1	0
RGMA	0	0	0	1	0	0
RGR	0	1	1	0	0	1
RGS1	1	0	1	1	0	1
RGS10	1	0	1	1	0	1
RGS12	1	1	1	1	1	1
RGS14	0	1	0	0	1	0
RGS20	1	0	0	0	0	0
RHBDD2	0	0	0	1	0	1
RHBDD3	1	0	0	1	0	0

RHBDF1	0	0	0	1	0	0
RHOA	1	0	0	1	0	0
RHOBTB3	1	0	0	0	0	0
RHOQ	1	0	0	0	0	0
RHOT1	1	0	0	1	1	0
RHOT2	1	0	0	1	0	1
RHOU	1	0	0	0	0	0
RHPN1	1	0	0	0	1	0
RIC8A	1	0	0	0	1	0
RIN2	0	1	1	1	0	1
RINT1	0	1	0	0	0	0
RIOK1	1	0	0	1	0	1
RIOK2	1	0	0	0	0	0
RIPK1	1	0	0	0	0	1
RIPK5	1	0	0	1	0	0
RIT1	1	0	0	1	0	0
RLBP1	0	0	0	0	0	1
RLN1	0	1	0	0	0	0
RMND5A	0	0	0	1	0	0
RNASE1	1	0	1	0	1	0
RNASE10	1	1	0	0	0	0
RNASE2	0	1	0	0	1	0
RNASE3	0	1	0	0	1	0
RNASE4	1	1	0	1	1	0
RNASEH1	1	0	0	0	0	0
RNASEN	1	0	0	1	0	0
RNF10	1	0	0	1	0	0
RNF103	0	0	0	1	0	0
RNF113A	1	1	0	1	0	1
RNF113B	0	0	1	0	0	1
RNF12	0	1	0	0	0	0
RNF121	1	0	0	1	1	0
RNF123	1	0	0	1	0	0
RNF125	0	0	0	0	1	0
RNF13	1	0	0	0	0	0
RNF138	1	0	0	0	0	0
RNF144	0	1	0	0	0	0
RNF146	0	0	0	1	1	0
RNF166	1	0	0	1	0	0
RNF167	1	0	0	1	0	0
RNF168	0	0	0	1	0	0
RNF17	0	0	1	0	0	1
RNF170	1	0	0	1	0	0
RNF180	1	0	0	0	0	0
RNF185	1	0	0	1	0	0
RNF186	0	1	1	0	0	1
RNF19	1	0	1	0	0	0
RNF20	0	0	0	1	0	0
RNF25	1	0	0	1	0	0
RNF26	1	1	0	1	0	0
RNF31	1	0	0	1	0	0
RNF32	1	0	0	0	0	0
RNF38	1	0	0	0	0	0
RNF40	1	0	0	1	0	0
RNF41	1	0	0	1	0	0
RNF44	0	0	0	1	0	0
RNF6	1	0	0	0	0	0
RNF7	0	0	0	0	1	0
RNGTT	1	0	0	1	0	0

RNH1	1	0	0	0	0	0
RNMT	1	0	0	1	0	1
RNMTL1	1	0	0	1	1	0
RNPC2	1	0	0	1	0	0
RNPC3	0	0	1	0	0	0
RNPEP	1	0	1	0	0	0
RNUXA	0	0	1	0	1	0
ROBO4	0	0	1	0	0	1
ROCK1	0	0	0	1	0	0
ROCK2	1	0	0	0	0	0
ROD1	1	0	0	0	0	0
ROPN1L	1	0	0	0	0	0
RORA	0	1	1	0	0	0
RORC	0	0	1	0	0	1
RP11-49G10.8	0	0	1	0	0	1
RP11-529I10.4	1	0	0	1	0	0
RP13-15M17.2	1	0	0	1	0	0
RP9	1	0	0	1	0	0
RPA1	1	0	0	1	0	0
RPA3	0	0	1	0	0	0
RPA4	0	1	0	0	0	0
RPGR	1	1	1	1	0	1
RPH3A	0	0	1	0	0	1
RPH3AL	0	0	0	1	0	0
RPIA	1	0	0	1	0	0
RPL12	1	0	0	0	1	0
RPL13	1	0	0	0	0	0
RPL13A	1	0	0	1	0	0
RPL14	1	0	0	1	0	0
RPL15	1	0	0	0	0	0
RPL17	1	0	0	0	0	0
RPL19	0	0	0	0	1	0
RPL26	0	1	0	0	0	0
RPL26L1	1	0	0	0	0	0
RPL27	0	0	0	1	0	0
RPL27A	0	0	0	1	0	0
RPL29	1	0	0	1	0	0
RPL30	1	0	0	0	1	0
RPL32	0	0	0	0	1	0
RPL34	1	0	0	1	0	1
RPL35A	1	0	1	1	0	1
RPL36A	1	1	1	1	1	1
RPL36AL	1	0	0	1	0	0
RPL37	0	0	0	1	0	0
RPL37A	1	0	0	0	0	1
RPL38	0	0	0	1	0	0
RPL39	0	0	1	0	0	0
RPL4	1	0	0	1	0	0
RPL41	1	0	0	0	0	0
RPL6	0	0	0	1	0	0
RPL7	1	0	0	1	0	0
RPL7A	1	0	0	0	0	0
RPL7L1	1	0	0	0	1	0
RPL8	0	0	0	1	0	0
RPL9	1	0	0	0	0	0
RPLP0	1	0	0	0	0	0
RPLP1	1	1	0	0	0	0
RPLP2	1	0	0	0	0	0
RPN2	1	0	0	1	0	0

RPP14	1	0	0	1	0	1
RPP21	1	0	0	0	0	0
RPP38	1	0	0	1	0	0
RPP40	0	1	0	0	0	0
RPS11	0	0	0	1	0	0
RPS12	0	0	0	1	0	0
RPS14	1	0	0	0	0	0
RPS16	1	0	0	1	0	0
RPS17	1	0	0	0	0	0
RPS18	1	0	0	1	0	1
RPS19	1	0	0	1	0	0
RPS21	1	0	0	1	0	0
RPS23	1	0	0	1	1	0
RPS24	1	0	0	1	0	0
RPS25	1	0	0	1	0	0
RPS26	0	0	0	0	0	1
RPS27A	1	0	0	1	0	0
RPS27L	0	0	0	0	1	0
RPS28	1	0	0	1	0	0
RPS29	1	0	0	1	0	1
RPS3	1	1	0	0	0	0
RPS4X	0	0	1	1	0	0
RPS5	1	0	0	0	0	0
RPS6	0	0	0	1	0	0
RPS6KA1	0	0	0	1	1	0
RPS6KB1	1	1	0	1	0	0
RPS6KL1	0	0	1	0	0	0
RPS7	1	0	0	1	0	0
RPSA	1	0	0	1	0	0
RPUSD1	1	0	0	0	0	1
RPUSD3	1	0	0	0	0	0
RPUSD4	1	0	0	1	0	1
RQCD1	1	0	0	1	1	1
RRAGA	0	0	0	1	0	0
RRAGB	1	1	0	0	0	0
RRAGC	1	0	0	0	0	0
RRAS	0	0	0	1	0	0
RREB1	1	0	1	1	1	1
RRM1	1	0	0	1	0	0
RRS1	1	0	0	1	1	0
RS1	0	1	0	0	1	0
RSAD2	0	1	0	0	1	0
RSC1A1	1	0	0	0	0	0
RSPO3	0	0	0	1	0	0
RSPRY1	1	0	1	1	0	0
RSRC1	1	0	0	1	0	0
RTCD1	1	0	0	1	0	0
RTEL1	1	0	0	1	0	0
RTF1	1	0	0	1	0	0
RTN3	1	0	0	0	0	0
RTN4	1	0	0	1	0	1
RTN4IP1	1	0	0	1	1	0
RTN4R	0	0	0	0	0	1
RTN4RL2	0	0	1	0	0	1
RTTN	1	0	0	0	0	0
RUFY1	0	1	0	0	1	0
RUFY3	0	1	0	0	0	0
RUNDC1	0	1	0	1	1	0
RUNDC2A	1	0	0	1	0	0

RUNX1	1	0	0	1	1	0
RUNX3	0	0	0	0	0	1
RUTBC1	1	0	0	1	0	0
RUVBL2	1	0	0	1	1	0
RWDD1	1	0	0	1	0	0
RWDD4A	1	0	0	0	0	0
RXRB	1	0	0	1	0	0
RYK	1	0	0	1	0	0
RYR1	0	0	0	1	0	0
RETSAT	1	0	0	1	0	1
S100A10	0	0	1	0	0	1
S100A11	0	0	0	0	1	0
S100A12	0	1	0	0	1	0
S100A13	0	0	1	0	1	1
S100A16	0	0	1	0	0	1
S100A3	0	0	0	1	1	0
S100A4	0	0	0	1	1	0
S100A5	0	0	0	1	0	0
S100P	0	0	1	0	0	1
S100PBP	1	0	0	1	0	1
SAC	0	0	1	1	0	1
SAC3D1	1	0	0	1	0	0
SACM1L	0	0	0	0	1	0
SACS	0	0	1	0	0	1
SAFB	1	0	0	1	0	0
SAFB2	1	0	0	1	0	0
SAG	0	0	1	0	0	1
SAMD3	0	0	1	0	0	0
SAMD4B	1	0	0	1	1	0
SAMD7	0	1	1	1	0	1
SAMD8	0	0	1	1	0	0
SAMHD1	1	0	0	1	0	0
SAP130	1	0	0	1	0	0
SAP18	1	0	0	0	0	0
SAP30BP	1	0	0	1	0	1
SAP30L	0	1	0	0	0	0
SAR1B	1	0	0	0	0	0
SARS	0	0	0	1	0	0
SARS2	1	0	0	1	0	0
SAS10	1	0	0	1	0	0
SASS6	1	0	0	1	0	0
SAT2	1	0	0	0	0	0
SATB1	0	1	0	0	1	0
SATL1	0	1	1	1	0	1
SAV1	0	0	0	1	0	0
SBDS	1	0	0	0	0	0
SBF2	1	0	0	0	0	0
SCAMP1	1	0	0	0	1	1
SCAMP2	1	0	0	1	0	0
SCAMP3	1	0	0	1	1	0
SCAMP4	1	0	0	1	0	0
SCAND1	1	1	0	1	0	0
SCAND2	1	0	0	0	0	0
SCARB2	0	1	0	0	1	0
SCCPDH	0	1	0	1	0	0
SCD	0	0	0	1	0	0
SCEL	0	0	1	0	0	0
SCFD2	0	1	0	0	0	0
SCG5	0	1	1	0	1	1

SCGB1C1	0	0	0	0	0	1
SCGB3A1	0	0	1	0	0	0
SCGB3A2	0	0	1	0	0	1
SCGN	0	0	0	0	0	1
SCIN	0	0	1	0	0	1
SCLY	1	0	0	1	0	0
SCMH1	0	1	1	0	0	1
SCML1	0	1	0	0	0	0
SCN11A	0	0	1	0	0	1
SCN1A	1	1	1	0	0	0
SCN7A	0	1	1	0	0	1
SCN9A	0	0	1	0	0	0
SCNM1	1	0	0	1	1	0
SCNN1B	0	0	1	0	0	1
SCO1	1	0	0	1	0	0
SCOC	0	0	1	0	0	1
SCOTIN	0	1	0	1	0	0
SCP2	0	0	0	1	0	0
SCRG1	0	1	0	1	0	0
SCRN3	1	0	0	1	0	1
SCTR	0	0	1	0	0	1
SCYE1	1	0	0	1	1	1
SCYL2	1	0	0	0	0	0
SCYL3	1	0	0	1	0	0
SDAD1	0	0	1	0	0	0
SDC2	1	0	0	0	0	0
SDC3	0	0	0	1	0	0
SDC4	0	0	0	1	0	0
SDCBP	0	0	0	1	0	0
SDCBP2	0	0	1	1	1	1
SDCCAG10	1	0	0	1	0	1
SDCCAG3	1	0	0	1	1	0
SDCCAG8	0	0	0	1	0	0
SDF2	1	0	0	1	0	0
SDF2L1	1	0	0	1	0	0
SDHA	1	0	0	1	0	0
SDHB	1	0	0	1	0	0
SDHD	1	0	0	1	0	0
SDK2	0	1	1	0	0	1
SDPR	0	0	0	0	1	0
SDSL	0	0	0	1	0	1
SEC14L1	1	0	0	0	0	0
SEC23A	1	0	0	1	0	0
SEC23IP	1	0	0	1	0	0
SEC24B	1	0	0	1	0	0
SEC24C	1	0	0	1	1	0
SEC24D	1	0	0	0	0	0
SEC61A1	1	0	0	0	0	0
SEC61B	1	0	0	1	0	0
SEC63	1	0	0	1	0	0
SECISBP2	1	1	1	1	1	0
SEL1L	0	0	0	0	0	1
SELI	1	0	0	1	0	0
SELM	0	0	0	1	0	0
SEMA3C	0	0	0	1	0	0
SEMA3D	0	0	1	0	0	0
SEMA4A	0	0	0	0	1	0
SEMA4B	1	1	0	1	0	0
SEMA4D	0	0	1	0	0	1

SEMA5B	0	0	0	1	0	0
SEMA6B	0	0	0	1	0	1
SENP1	1	0	0	1	0	0
SENP2	1	0	0	1	0	0
SENP3	1	0	0	1	1	1
SENP5	1	0	0	1	0	0
SENP7	1	1	0	1	0	0
SENP8	1	1	0	0	0	0
SEPHS2	0	0	0	1	0	0
SEPN1	0	0	0	0	0	1
SEPP1	0	0	1	0	0	1
SERF2	1	0	0	0	0	0
SERINC1	1	0	0	1	0	1
SERINC4	1	0	0	1	0	0
SERINC5	0	0	0	1	0	0
SERP1	1	0	0	1	1	0
SERPINA10	0	1	1	0	0	1
SERPINA12	0	1	1	0	0	1
SERPINA6	0	0	1	0	0	1
SERPINB1	1	0	1	0	0	0
SERPINB2	0	0	0	0	0	1
SERPINB5	0	0	1	0	0	1
SERPINB8	1	0	0	1	0	0
SERPIND1	0	0	1	0	0	1
SERPINE1	0	0	0	0	0	1
SERPINF1	1	0	0	0	0	0
SERPINF2	0	0	1	0	0	1
SERPING1	0	0	0	0	1	0
SERPINI1	1	0	0	1	0	0
SERTAD1	1	0	0	1	0	0
SERTAD3	1	0	0	1	1	0
SESN2	1	0	0	0	0	0
SESTD1	0	0	1	0	0	0
SET	1	0	0	0	0	0
SETBP1	0	0	1	0	0	1
SETD1A	1	0	1	1	1	1
SETD2	1	0	0	0	0	0
SETD3	1	0	0	1	0	1
SETD5	1	0	0	0	0	0
SETD6	0	0	0	1	0	0
SETDB2	1	0	0	1	1	1
SETMAR	1	0	0	1	0	0
SF1	1	0	0	1	0	1
SF3A1	1	0	0	1	1	0
SF3A2	0	0	0	1	0	0
SF3A3	1	0	0	1	0	0
SF3B14	1	0	0	0	0	0
SF3B2	1	0	0	0	0	0
SF3B3	1	1	0	1	0	0
SF3B4	0	0	0	0	1	0
SF4	1	1	0	0	1	0
SFI1	1	0	0	1	0	0
SFMBT2	1	0	0	0	0	0
SFRS1	1	0	0	1	0	0
SFRS11	1	0	0	1	0	1
SFRS12	1	0	1	0	0	0
SFRS14	0	0	0	1	0	1
SFRS15	0	0	0	0	1	0
SFRS2	1	0	0	1	0	1

SFRS3	1	0	0	0	0	0
SFRS6	1	0	0	0	0	0
SFRS8	0	0	0	1	0	0
SFRS9	1	0	0	1	0	0
SFT2D1	1	0	0	1	1	0
SFT2D3	0	0	1	0	0	0
SFTPD	0	1	0	0	1	0
SFXN1	1	0	0	1	0	0
SFXN2	1	0	0	1	1	0
SFXN4	0	0	0	1	1	0
SFXN5	0	1	0	1	0	0
SGCA	1	0	1	1	0	1
SGCB	0	0	0	1	0	0
SGK	0	0	0	1	0	0
SGOL2	1	0	0	1	0	0
SGPL1	1	0	1	1	0	0
SGPP1	0	0	0	1	0	0
SGPP2	1	0	0	0	0	0
SGSH	1	0	0	1	0	0
SGTA	0	0	0	1	0	0
SGTB	1	0	0	0	0	0
SH2D1A	0	1	1	0	1	1
SH2D3C	1	0	0	1	0	1
SH2D4A	1	0	0	0	0	0
SH3BGR	0	1	1	0	0	1
SH3BGRL	1	0	0	0	0	0
SH3BGRL3	0	0	0	1	0	0
SH3BP5	1	0	0	0	0	1
SH3BP5L	0	0	0	1	1	0
SH3GL1	1	0	0	1	0	0
SH3PX3	1	0	0	0	0	0
SH3PXD2B	1	0	0	0	0	0
SH3RF2	0	0	1	0	0	0
SH3TC1	1	0	0	0	0	0
SH3YL1	1	0	0	1	0	0
SHARPIN	1	0	0	1	1	0
SHB	0	0	0	1	0	0
SHC1	1	0	0	1	1	0
SHFM1	0	0	0	1	0	0
SHMT1	1	0	0	0	0	0
SHMT2	1	0	0	1	0	0
SHOX2	1	0	0	1	0	0
SHQ1	1	1	0	1	1	0
SIAE	1	0	0	1	0	0
SIAH1	1	0	0	1	0	0
SIAH2	1	0	0	1	0	0
SIGLEC1	0	0	1	1	1	1
SIGLEC12	1	0	0	0	0	0
SIGLEC5	0	0	1	0	0	0
SIGLEC7	1	0	0	0	0	0
SIGLEC9	0	0	0	1	0	0
SIL1	1	0	1	0	1	1
SIN3A	1	0	0	1	0	0
SIN3B	1	0	0	0	0	0
SIP1	1	0	0	0	0	0
SIPA1L1	0	0	0	1	0	0
SIPA1L2	0	0	1	0	0	1
SIRPA	1	0	0	1	0	0
SIRPB1	0	0	0	1	1	1

SIRPD	0	0	0	0	0	1
SIRT1	0	0	1	0	0	0
SIRT2	1	1	0	1	0	1
SIRT5	1	0	0	1	0	1
SIRT6	1	0	0	0	0	0
SIRT7	0	0	0	1	0	0
SIX5	0	0	1	0	0	0
SKIL	1	0	0	1	0	0
SKIP	1	0	1	1	0	0
SKIV2L	1	0	0	1	0	0
SKIV2L2	1	0	0	1	0	1
SKP2	1	0	0	0	0	0
SLA	0	0	0	0	1	0
SLAMF1	0	0	1	0	0	1
SLAMF6	0	0	0	0	0	1
SLAMF7	1	0	0	1	0	0
SLAMF8	1	0	1	1	0	1
SLAMF9	0	0	1	0	0	0
SLC10A2	0	0	1	0	0	1
SLC10A3	0	1	0	0	0	0
SLC11A1	0	0	0	1	0	0
SLC11A2	1	0	0	1	0	0
SLC12A6	0	0	0	0	1	0
SLC12A7	0	0	0	0	0	1
SLC13A1	0	0	1	0	0	1
SLC13A3	0	1	1	0	1	1
SLC13A4	1	1	1	0	0	1
SLC14A1	0	1	0	1	0	0
SLC15A4	1	0	0	1	1	0
SLC16A5	1	0	0	0	0	0
SLC16A7	0	0	1	1	1	1
SLC16A9	0	0	0	0	0	1
SLC17A4	0	0	1	0	0	1
SLC18A1	1	0	0	1	0	0
SLC1A3	0	0	0	1	0	0
SLC1A4	0	0	0	1	0	0
SLC1A5	0	0	0	1	0	0
SLC1A7	0	0	1	0	0	1
SLC20A1	1	0	0	1	0	0
SLC20A2	1	0	0	1	0	1
SLC22A1	0	0	1	0	0	1
SLC22A15	0	0	0	1	0	0
SLC22A16	0	1	0	0	0	0
SLC22A18	1	0	0	1	0	0
SLC22A18AS	1	0	0	1	0	0
SLC22A5	0	0	0	1	0	0
SLC22A7	0	1	1	0	0	1
SLC23A1	0	1	1	0	0	1
SLC23A2	0	0	1	0	0	1
SLC23A3	1	0	1	1	0	1
SLC24A1	0	0	1	0	0	1
SLC24A2	0	0	0	1	0	0
SLC24A6	1	0	0	1	0	0
SLC25A1	1	0	0	0	0	0
SLC25A10	0	1	0	0	1	0
SLC25A11	1	0	0	1	0	0
SLC25A13	0	1	1	0	0	1
SLC25A14	0	1	0	0	0	0
SLC25A15	0	1	0	0	0	0

SLC25A17	0	0	0	0	1	0
SLC25A22	0	0	0	0	1	0
SLC25A24	1	1	1	1	0	0
SLC25A25	1	0	1	1	1	1
SLC25A26	0	0	1	0	0	0
SLC25A28	1	0	0	0	0	0
SLC25A29	0	0	0	1	0	0
SLC25A31	0	0	1	0	0	1
SLC25A32	1	0	0	1	1	1
SLC25A34	0	0	1	0	0	1
SLC25A35	1	0	0	0	0	0
SLC25A37	0	1	0	1	0	0
SLC25A5	1	1	1	0	0	1
SLC26A1	0	0	0	1	0	0
SLC26A8	0	1	0	1	1	0
SLC27A4	1	0	0	1	0	0
SLC27A5	1	0	1	1	0	0
SLC28A3	0	0	0	0	0	1
SLC29A1	0	0	1	0	0	0
SLC29A2	1	0	0	0	0	0
SLC29A3	0	1	1	0	0	0
SLC2A3	1	0	0	0	0	0
SLC2A5	0	1	0	0	1	0
SLC2A9	1	1	1	1	1	1
SLC30A5	0	0	0	1	0	0
SLC30A6	1	0	0	1	0	0
SLC30A7	1	0	0	1	0	1
SLC31A1	1	0	0	1	0	0
SLC31A2	1	0	0	1	0	0
SLC33A1	1	0	0	0	1	0
SLC35A1	1	0	0	1	1	0
SLC35A2	1	1	0	0	0	0
SLC35A4	1	0	0	1	0	0
SLC35A5	1	0	0	0	0	0
SLC35B1	0	0	0	1	1	0
SLC35B2	0	0	0	1	0	0
SLC35B3	0	0	0	1	0	0
SLC35B4	1	0	0	1	0	0
SLC35C2	0	0	0	0	1	0
SLC35D2	1	0	0	1	0	0
SLC35E1	1	0	0	1	0	0
SLC36A1	0	0	0	1	0	0
SLC36A4	1	0	0	0	0	0
SLC37A1	1	1	1	1	0	0
SLC37A4	1	0	0	1	0	0
SLC38A2	0	1	0	0	0	0
SLC38A5	0	1	1	0	0	1
SLC38A6	1	0	1	1	0	1
SLC39A1	1	0	0	1	0	0
SLC39A12	0	0	1	1	0	0
SLC39A3	1	0	0	1	0	0
SLC39A4	1	0	0	1	0	1
SLC39A5	0	0	1	0	0	1
SLC39A6	1	0	0	0	1	0
SLC39A7	1	0	0	1	0	0
SLC39A9	1	1	0	1	0	0
SLC3A2	1	0	0	1	1	0
SLC40A1	0	1	0	0	0	0
SLC41A2	1	0	1	0	0	1

SLC43A1	0	1	0	0	0	0
SLC43A3	1	0	1	0	1	0
SLC44A1	1	0	1	1	0	1
SLC45A2	1	0	0	1	1	0
SLC4A1AP	1	0	0	1	1	0
SLC4A8	0	0	0	1	0	0
SLC5A11	0	0	1	0	0	1
SLC5A2	0	0	1	0	0	0
SLC5A3	1	0	1	1	1	1
SLC5A6	1	0	0	1	0	0
SLC5A8	0	0	1	0	0	1
SLC6A13	0	1	1	0	0	1
SLC6A16	0	0	1	0	0	0
SLC6A19	0	0	1	0	0	1
SLC6A9	0	0	1	0	0	1
SLC7A11	1	0	1	1	0	1
SLC7A13	0	0	1	0	0	1
SLC7A5	0	0	0	0	1	0
SLC7A6OS	1	0	0	1	1	0
SLC7A7	0	0	0	1	0	0
SLC7A8	1	0	1	1	1	1
SLC8A1	0	0	1	0	0	1
SLC9A6	0	1	1	0	0	1
SLC9A7	1	0	0	1	0	0
SLCO1B1	0	0	1	0	0	1
SLCO2B1	1	0	1	1	0	1
SLCO3A1	1	0	0	0	0	0
SLITRK2	0	0	1	1	0	0
SLTM	0	0	0	1	0	0
SLU7	1	0	0	1	0	0
SLURP1	0	1	1	0	0	1
SMA4	0	0	1	0	0	1
SMA5	1	0	1	1	1	1
SMAD1	0	0	1	0	0	0
SMAD2	0	0	0	1	0	0
SMAD3	0	1	0	0	1	0
SMAD4	0	0	0	0	1	1
SMAD7	1	0	0	0	0	0
SMAP1L	1	0	0	1	0	0
SMARCA4	1	0	0	0	0	0
SMARCA5	1	0	0	1	0	0
SMARCAD1	1	0	0	0	0	0
SMARCAL1	1	0	0	1	0	0
SMARCC1	1	0	0	1	0	0
SMARCC2	0	0	0	1	0	0
SMARCD1	1	0	1	0	0	1
SMARCD2	1	0	0	1	0	0
SMCP	0	1	1	0	0	1
SMEK2	1	0	0	1	0	0
SMG5	1	0	0	1	1	0
SMOX	1	0	0	1	0	1
SMPD1	0	1	0	0	1	0
SMS	0	1	0	1	1	0
SMTN	0	1	0	1	0	0
SMUG1	1	0	0	1	0	0
SMYD2	0	1	0	0	0	0
SMYD3	0	0	1	0	0	1
SNAG1	0	0	0	1	0	0
SNAP29	1	0	0	1	0	0

SNAPC1	0	0	0	0	1	0
SNAPC4	1	0	0	0	0	0
SNAPC5	1	0	0	1	0	0
SNCA	0	0	0	1	0	1
SND1	0	0	1	1	1	1
SNIP	0	0	0	0	0	1
SNIP1	0	0	0	1	0	0
SNN	0	0	0	0	0	1
SNRK	0	1	0	0	0	0
SNRPA	1	0	0	1	0	0
SNRPA1	1	0	0	1	0	0
SNRPB	1	0	1	1	0	0
SNRPC	1	0	0	1	0	0
SNRPD2	1	0	0	0	0	0
SNRPD3	1	0	0	1	0	0
SNRPE	0	0	0	1	0	0
SNRPG	1	0	0	1	0	0
SNRPN	1	0	1	0	0	1
SNTB1	0	1	0	0	0	0
SNW1	1	0	0	1	0	0
SNX1	1	0	0	1	0	0
SNX12	0	1	0	0	0	0
SNX13	1	0	0	1	0	0
SNX14	1	0	0	1	0	0
SNX16	0	0	0	1	0	0
SNX17	1	1	0	1	1	0
SNX19	0	1	0	0	0	0
SNX2	1	0	0	0	0	1
SNX24	0	1	0	1	0	0
SNX25	0	0	0	1	0	0
SNX26	0	1	0	0	0	0
SNX5	1	0	0	1	0	0
SOAT1	1	0	0	0	0	0
SOCS2	0	0	0	1	0	0
SOCS4	1	0	0	1	1	1
SOCS6	1	0	0	0	0	0
SOCS7	1	0	0	1	0	0
SOD2	1	0	0	1	0	0
SOLH	1	0	1	0	0	0
SON	1	0	0	1	0	0
SORBS1	1	1	1	1	1	1
SORL1	0	1	0	0	0	0
SORT1	1	0	1	1	0	1
SOS1	0	0	0	1	0	0
SOS2	0	0	0	1	0	0
SOST	0	0	0	0	0	1
SOX15	1	0	0	0	0	0
SOX18	0	0	0	0	0	1
SOX2	1	0	0	0	0	1
SOX30	0	1	1	0	0	1
SOX5	0	0	1	0	0	0
SP1	1	0	0	1	0	0
SP100	1	1	0	1	1	0
SP110	0	1	0	1	1	0
SP140	0	1	0	1	1	0
SPA17	1	0	0	1	0	0
SPACA4	0	1	0	0	1	0
SPAG16	0	1	0	0	0	0
SPAG7	1	0	0	1	0	0

SPAG9	1	0	1	0	0	1
SPARC	0	1	1	0	0	1
SPATA1	1	0	0	1	0	0
SPATA13	1	0	0	0	0	0
SPATA2	0	0	0	1	0	0
SPATA3	0	0	1	0	0	1
SPATA5	1	0	0	1	0	1
SPATA5L1	1	0	0	1	0	0
SPATA6	1	0	0	1	0	0
SPATA9	0	0	1	0	0	1
SPCS1	1	0	0	1	1	0
SPCS2	1	1	0	1	1	0
SPCS3	1	0	1	1	0	0
SPDEF	0	1	1	0	0	1
SPECC1	0	0	1	0	0	0
SPG21	1	0	0	1	0	0
SPG7	1	0	0	0	1	0
SPHAR	0	0	0	1	0	0
SPHK2	1	0	1	1	1	0
SPI1	1	0	0	0	1	0
SPIC	0	0	1	0	0	1
SPIN3	0	0	1	0	0	0
SPINK1	0	1	1	0	1	1
SPINT2	1	0	0	0	0	0
SPIRE1	0	0	1	0	0	1
SPIRE2	1	0	0	1	0	0
SPN	1	0	0	0	0	0
SPO11	0	0	1	0	0	1
SPOCD1	0	0	1	0	0	1
SPOCK1	0	0	1	0	0	1
SPOCK2	0	0	0	1	0	0
SPP1	1	0	1	1	0	1
SPPL2A	0	0	0	1	0	0
SPPL2B	1	0	0	1	0	0
SPRED1	0	0	0	1	0	0
SPRR1A	0	1	1	1	0	1
SPRY2	1	0	0	1	0	0
SPRYD3	1	0	0	1	0	0
SPRYD4	1	0	0	1	0	0
SPRYD5	0	0	1	0	0	1
SPSB1	0	0	0	0	0	1
SPSB2	1	0	0	1	0	0
SPSB3	1	0	0	1	0	0
SPTAN1	1	0	0	1	0	1
SPTB	0	0	1	1	0	1
SPTBN5	0	0	0	0	1	0
SPTLC1	1	0	0	0	0	0
SPTLC2	0	0	0	1	1	0
SPTY2D1	1	0	0	1	0	0
SQLE	1	0	0	0	0	0
SQRDL	0	0	0	1	0	0
SQSTM1	1	0	0	1	0	0
SR-A1	0	0	0	1	0	0
SRBD1	0	0	0	1	0	0
SRC	0	0	0	1	0	0
SRCRB4D	1	1	1	0	1	1
SRD5A1	1	0	0	1	0	0
SRD5A2L	0	0	0	1	0	0
SRF	1	0	0	0	0	0

SRI	0	0	0	0	1	0
SRP19	0	1	0	0	0	0
SRP9	1	0	0	0	0	0
SRPK2	1	0	0	1	0	0
SRPR	1	0	0	1	0	1
SRR	1	0	0	0	0	1
SS18	0	1	0	0	0	0
SS18L1	1	0	0	1	0	0
SS18L2	1	0	0	1	0	1
SSBP1	1	0	0	1	0	0
SSBP2	1	0	0	0	0	0
SSFA2	1	0	0	1	0	0
SSH2	0	0	0	1	0	0
SSNA1	1	0	0	1	0	0
SSR1	1	0	0	0	0	0
SSR2	1	0	0	1	0	0
SSR4	1	0	0	1	0	0
SSSCA1	1	0	0	1	0	0
SST	0	0	1	0	0	1
SSTR2	1	0	0	0	0	0
SSU72	1	0	0	0	0	0
SSX8	0	0	1	0	0	1
ST13	1	0	0	1	1	0
ST14	1	0	0	1	0	0
ST18	0	1	1	0	0	1
ST3GAL1	1	0	0	1	0	0
ST3GAL3	1	0	0	0	0	0
ST3GAL4	0	1	0	0	1	0
ST3GAL6	1	0	0	1	0	1
ST5	1	0	0	1	0	1
ST6GAL1	0	0	1	0	0	1
ST6GALNAC4	1	0	0	0	1	0
ST7	0	1	0	0	0	0
ST7L	1	0	0	1	0	0
STAB2	0	1	1	0	0	1
STAC3	0	0	1	0	0	1
STAG1	1	0	0	1	0	0
STAG3	1	0	0	1	0	0
STAM	1	0	0	0	0	0
STAMBP	1	0	0	0	0	0
STAMBPL1	1	1	1	1	1	1
STAP2	1	1	0	0	0	0
STARD13	0	0	1	0	0	1
STARD3	1	0	1	0	0	1
STARD4	1	0	0	1	0	1
STARD5	0	0	0	1	0	0
STARD8	0	1	1	0	0	1
STAT1	0	0	0	1	0	0
STAT2	1	0	0	1	0	1
STAT3	0	0	0	1	0	0
STAT4	0	0	0	1	0	0
STAT5A	0	0	0	0	1	0
STAT5B	0	0	0	1	0	0
STAT6	0	0	0	1	0	0
STCH	1	0	0	1	1	0
STH	0	0	1	0	0	1
STIM1	1	1	0	1	0	0
STIM2	1	0	0	1	0	0
STIP1	1	0	0	0	0	0

STK11IP	1	0	0	1	0	0
STK16	1	1	0	1	0	0
STK19	1	0	1	1	0	1
STK24	1	0	1	1	0	1
STK32B	0	1	0	0	0	0
STK35	1	0	0	1	0	0
STK36	0	0	0	1	0	0
STK38	0	0	0	1	0	0
STK40	0	0	0	0	1	0
STMN1	0	0	0	1	0	0
STOML2	1	0	0	1	1	0
STRAP	0	0	0	1	0	0
STRN	0	1	0	0	0	0
STRN3	1	0	0	1	0	0
STRN4	0	0	0	0	1	0
STS	0	1	1	1	0	1
STS-1	1	0	0	1	0	0
STT3A	0	0	0	0	1	0
STT3B	1	0	0	1	0	0
STX10	1	0	0	0	0	0
STX12	1	0	0	1	0	0
STX16	1	0	0	0	0	0
STX6	0	0	0	1	0	0
STX7	0	1	0	0	0	0
STX8	1	0	0	1	0	0
STXBP2	1	0	0	1	0	0
STXBP3	0	0	1	1	0	1
STYX	1	0	0	0	0	0
STYXL1	1	0	0	1	0	0
SUCLA2	1	0	0	0	0	0
SUCLG1	0	1	0	0	0	0
SUCNR1	1	0	1	1	0	1
SUDS3	1	0	0	1	0	0
SUFU	1	0	0	1	0	0
SUGT1	1	0	0	1	0	1
SUHW4	1	0	0	0	0	0
SULF2	1	0	0	0	0	0
SULT1A3	1	0	0	1	0	0
SULT1A4	1	0	0	1	0	0
SUMF2	1	0	0	0	0	0
SUMO2	1	0	0	1	0	0
SUOX	1	0	0	1	0	0
SUPT16H	0	0	0	1	0	0
SUPT3H	1	0	0	0	0	0
SUPT4H1	1	0	0	0	0	0
SUPT6H	1	0	0	1	0	0
SUPT7L	1	0	0	1	1	0
SUPV3L1	1	0	0	1	0	0
SURF1	1	0	0	1	0	0
SURF2	1	0	0	1	0	0
SURF4	1	0	0	1	1	0
SURF5	1	0	0	1	0	0
SURF6	1	0	0	1	0	0
SUSD2	0	0	1	0	0	0
SUV39H1	0	1	0	0	0	0
SUV39H2	1	0	0	0	0	0
SVIL	0	0	0	0	0	1
SYCP2	1	1	1	1	0	0
SYCP3	0	0	0	0	0	1

SYF2	0	0	0	1	0	1
SYK	1	0	0	0	0	0
SYMPK	1	0	0	0	0	0
SYN1	0	1	1	0	0	1
SYNC1	0	0	0	0	0	1
SYNGR1	0	1	1	0	1	1
SYNJ1	1	1	0	0	1	0
SYNPO2	0	0	1	0	0	0
SYP	0	1	0	1	0	0
SYPL1	0	1	0	0	0	0
SYT11	1	0	0	1	1	0
SYT17	1	0	0	1	0	0
SYT8	0	0	0	1	0	0
SYTL3	1	0	0	1	0	0
TA-NFKBH	0	0	0	0	1	0
TAAR2	0	1	1	0	0	1
TAAR6	0	0	1	0	0	1
TAC4	0	0	0	0	0	1
TACC3	0	0	0	1	0	0
TADA1L	1	0	0	1	0	0
TADA2L	1	0	0	1	0	0
TADA3L	1	0	0	1	0	0
TAF1	0	1	1	0	0	0
TAF10	1	1	0	1	0	0
TAF12	1	0	0	1	0	0
TAF13	1	0	0	1	0	0
TAF15	0	1	0	1	1	0
TAF1C	1	0	1	1	0	0
TAF1L	0	0	1	0	0	0
TAF2	1	0	0	1	0	0
TAF5L	1	0	0	1	0	1
TAF6	1	0	0	1	1	0
TAF6L	1	0	0	1	0	0
TAF9	1	0	0	1	0	0
TAGAP	0	0	0	0	1	0
TAGLN2	0	0	0	1	0	0
TAL2	0	1	1	0	0	1
TANK	0	0	0	0	0	1
TAOK2	1	0	0	1	1	0
TAP1	1	0	0	1	1	0
TAP2	1	0	0	1	0	0
TARP	0	1	0	0	1	1
TARS	1	0	0	1	0	0
TAS1R2	0	0	1	0	0	1
TAS2R1	0	0	1	0	0	0
TAS2R10	1	0	1	0	0	0
TAS2R39	0	1	1	0	0	1
TAS2R4	0	0	1	0	0	1
TAS2R5	0	0	0	1	0	1
TAS2R60	0	0	1	0	0	1
TASP1	0	1	0	0	0	0
TATDN1	1	0	1	1	0	1
TATDN3	0	0	0	1	0	0
TAX1BP1	0	0	0	1	0	0
TAX1BP3	1	0	0	1	0	0
TAZ	1	1	0	0	0	0
TBC1D10A	1	0	0	1	1	0
TBC1D14	0	0	0	1	0	0
TBC1D15	1	0	0	1	0	0

TBC1D17	1	0	0	1	0	0
TBC1D19	0	1	0	0	0	0
TBC1D22A	1	0	1	1	0	0
TBC1D22B	1	0	0	1	0	1
TBC1D3	0	1	1	0	0	1
TBC1D3C	0	1	1	0	0	1
TBC1D4	0	0	0	0	0	1
TBC1D5	1	0	0	1	0	1
TBC1D7	0	0	0	1	0	0
TBC1D8	0	0	1	0	0	1
TBCCD1	1	0	1	1	0	0
TBCE	0	0	0	1	0	0
TBK1	0	0	0	1	0	0
TBKBP1	1	0	0	0	0	0
TBL2	0	0	0	0	1	0
TBL3	1	0	0	1	0	0
TBN	1	0	0	0	0	0
TBP	1	0	0	0	0	0
TBPL1	0	0	0	1	0	0
TBRG1	1	0	0	0	0	0
TBRG4	1	0	0	0	0	0
TBX10	0	1	0	0	0	0
TBX19	1	0	1	0	1	1
TBX22	0	0	1	1	0	1
TBX5	0	0	0	1	0	0
TBXAS1	1	1	0	0	1	0
TCEA2	1	0	0	1	0	0
TCEAL1	0	0	1	0	0	1
TCEAL3	0	0	1	0	0	1
TCEAL4	0	1	0	0	0	0
TCEAL8	0	1	1	0	0	1
TCF12	1	0	0	0	0	1
TCF19	1	0	0	1	1	1
TCF20	0	0	1	0	0	1
TCF23	0	1	1	0	0	1
TCF3	1	0	0	0	0	0
TCF7	0	0	0	0	0	1
TCF7L2	0	0	0	1	0	0
TCIRG1	0	0	0	1	0	0
TCL1B	0	1	1	0	0	1
TCL6	0	1	1	0	0	1
TCP1	1	0	0	1	0	0
TCP10L	0	0	1	0	0	1
TCTA	1	0	0	1	0	0
TDG	1	1	0	1	0	0
TDO2	1	0	1	0	0	1
TDP1	1	0	0	1	0	0
TDRD1	0	1	1	0	1	1
TDRD6	0	1	1	0	0	1
TDRD7	1	1	0	0	0	0
TDRD9	0	0	1	1	0	1
TEAD1	1	0	1	0	0	1
TEAD2	0	0	0	1	0	0
TEAD3	0	0	0	0	0	1
TEC	0	1	1	1	1	1
TEDDM1	1	1	1	1	0	0
TEP1	0	0	0	1	1	0
TERF1	0	0	0	1	0	0
TERF2IP	1	1	0	1	0	0

TES	0	1	1	0	0	0
TESC	1	0	0	0	1	0
TESSP2	0	1	0	0	1	0
TESSP5	0	0	1	0	0	1
TEX10	1	0	0	0	1	0
TEX13A	0	1	1	0	1	1
TEX2	1	0	0	0	0	0
TEX264	0	0	1	0	1	1
TFB1M	0	1	1	0	1	1
TFB2M	1	0	0	1	0	0
TFDP1	1	0	0	0	0	0
TFE3	0	1	1	1	1	1
TFF3	0	1	1	0	1	1
TFG	0	0	0	1	0	0
TFIP11	1	0	0	1	0	1
TFPI	0	1	1	0	0	0
TFPT	1	0	0	1	0	0
TFR2	0	0	1	0	0	1
TGDS	1	0	0	1	0	0
TGFA	1	0	0	0	0	0
TGFBI	0	0	1	0	0	0
TGFBR1	0	0	0	1	0	0
TGFBR2	1	0	0	1	0	0
TGM2	1	0	0	1	0	1
TGM3	0	0	0	0	0	1
TGM6	0	0	1	0	0	1
TGM7	0	0	1	0	0	1
TH	0	0	1	0	0	1
THADA	1	0	0	1	0	0
THAP1	1	0	0	1	0	0
THAP10	0	0	0	1	0	0
THAP11	1	0	0	1	1	0
THAP5	1	0	1	1	0	1
THAP6	1	1	0	1	0	0
THAP7	1	0	0	1	0	0
THAP8	1	0	1	1	0	1
THBS3	1	0	0	1	0	0
THEM2	1	0	1	1	0	1
THEM4	0	0	0	1	0	0
THEX1	0	0	0	1	0	0
THNSL1	1	0	0	1	0	0
THOC2	0	1	1	1	1	1
THOC4	1	0	0	1	0	0
THOC5	1	0	0	1	0	0
THOC6	0	0	0	0	0	1
THOC7	0	0	0	0	1	0
THOP1	0	0	0	1	0	1
THTPA	1	0	0	1	0	0
THUMPD1	1	0	0	1	0	0
THYN1	1	0	0	1	1	0
TIAL1	1	0	0	1	0	1
TIAM2	0	1	1	1	1	1
TICAM2	1	0	0	0	1	0
TIE1	0	0	1	0	0	1
TIGA1	0	0	1	0	1	1
TIGD1	1	0	0	1	1	0
TIGD3	1	0	0	0	0	0
TIGD4	1	0	0	1	0	0
TIGD5	1	0	0	1	0	0

TIGD6	1	0	1	1	0	1
TIGD7	1	0	0	0	0	0
TIMM10	1	0	0	1	0	0
TIMM17A	0	0	0	1	0	0
TIMM17B	0	1	1	1	1	0
TIMM22	0	0	0	1	0	0
TIMM23	1	0	0	1	1	0
TIMM8A	1	1	0	1	1	0
TIMM8B	1	0	0	1	0	0
TIMM9	1	0	0	1	0	0
TIMP1	0	1	0	0	0	0
TIMP2	0	0	0	1	0	0
TIMP4	0	0	1	0	0	0
TINAG	1	0	1	0	0	1
TINF2	1	0	0	0	0	0
TINP1	1	1	0	1	0	0
TJAP1	1	0	0	1	0	0
TJP1	0	0	0	1	0	0
TJP2	0	0	0	1	0	0
TJP3	0	0	1	0	0	1
TK1	1	0	0	1	0	0
TK2	0	0	0	1	0	0
TKT	0	1	0	0	0	0
TLE6	1	0	0	1	0	0
TLK1	1	0	0	0	0	0
TLN1	1	0	0	1	0	0
TLN2	0	0	1	0	0	1
TLR10	1	0	0	0	0	0
TLR2	0	1	0	1	0	0
TLR4	0	0	0	0	1	0
TLR6	0	0	1	0	0	0
TLR7	0	1	0	0	1	0
TLR9	0	0	0	0	1	0
TM2D2	1	0	0	1	0	0
TM4SF1	0	0	0	1	0	0
TM4SF19	1	0	0	1	0	1
TM7SF2	1	0	0	1	1	0
TM7SF4	1	0	1	1	0	1
TM9SF1	1	0	0	1	1	0
TM9SF2	1	0	0	1	1	0
TM9SF4	1	0	0	0	0	0
TMBIM4	1	0	0	1	1	0
TMC4	0	0	0	1	0	0
TMCC1	1	0	0	1	0	1
TMCC2	0	0	0	1	0	0
TMCO1	1	0	0	1	0	0
TMCO3	1	0	0	1	0	0
TMCO5	0	1	1	0	0	1
TMED1	0	0	0	1	0	0
TMED10	1	0	0	1	0	0
TMED4	0	0	0	1	0	0
TMED5	0	0	0	0	0	1
TMED7	1	0	0	0	1	0
TMED8	0	1	0	0	0	0
TMEFF2	1	0	0	0	0	0
TMEM1	1	0	0	1	0	0
TMEM101	1	0	0	1	0	0
TMEM102	1	0	0	0	0	0
TMEM103	1	0	0	1	1	0

TMEM104	1	0	0	1	0	0
TMEM11	1	0	0	0	0	0
TMEM110	0	0	0	1	0	1
TMEM111	1	1	0	1	0	1
TMEM115	1	0	0	1	1	0
TMEM116	1	0	0	1	0	0
TMEM118	1	0	0	1	0	0
TMEM123	1	0	0	0	0	0
TMEM126A	1	0	0	0	0	0
TMEM126B	1	0	0	1	0	0
TMEM127	1	0	0	1	1	0
TMEM128	1	0	0	1	0	0
TMEM129	0	0	0	1	0	0
TMEM134	0	0	0	1	0	0
TMEM137	1	0	0	1	1	0
TMEM138	1	0	0	1	0	1
TMEM140	1	0	1	0	0	1
TMEM141	0	0	0	1	0	0
TMEM142A	0	0	0	1	0	0
TMEM143	0	0	0	0	0	1
TMEM144	0	0	1	0	0	1
TMEM147	1	0	0	0	0	0
TMEM149	1	0	0	1	1	0
TMEM14A	0	0	0	1	0	0
TMEM14B	0	1	0	0	1	0
TMEM16B	0	0	1	0	0	1
TMEM16F	1	0	0	0	0	0
TMEM18	1	0	0	0	0	0
TMEM19	1	0	0	1	0	0
TMEM24	1	0	0	0	1	0
TMEM26	1	0	0	0	0	1
TMEM29	1	1	0	1	0	1
TMEM30A	1	0	1	1	0	0
TMEM33	0	0	0	1	1	0
TMEM38A	1	0	0	1	0	0
TMEM39A	1	0	0	1	0	0
TMEM4	1	0	0	1	1	0
TMEM41A	0	0	0	1	0	0
TMEM41B	1	0	0	1	0	0
TMEM43	1	0	0	1	1	0
TMEM44	0	0	0	1	0	0
TMEM45A	1	0	0	0	0	0
TMEM48	0	0	0	1	0	0
TMEM50B	1	0	0	1	0	0
TMEM51	1	0	0	1	0	0
TMEM53	1	0	0	1	0	0
TMEM55A	0	0	0	0	1	0
TMEM55B	1	0	0	1	1	0
TMEM59	1	0	0	1	0	0
TMEM60	1	0	0	0	0	1
TMEM62	1	0	0	1	1	0
TMEM63B	1	1	1	1	0	1
TMEM65	1	0	0	0	0	0
TMEM68	1	0	0	1	0	0
TMEM69	1	0	0	0	0	0
TMEM70	1	0	0	1	0	0
TMEM71	0	0	1	0	1	0
TMEM77	1	0	0	1	0	1
TMEM79	1	0	0	1	1	0

TMEM80	0	0	1	0	0	1
TMEM81	0	0	1	0	0	0
TMEM85	1	0	0	0	0	0
TMEM86A	0	0	0	1	0	0
TMEM86B	0	0	0	0	0	1
TMEM87A	1	0	0	1	1	0
TMEM87B	1	0	0	1	0	0
TMEM88	1	0	0	1	0	0
TMEM9	1	1	0	0	0	0
TMEM93	1	0	0	1	0	0
TMEM99	1	1	1	0	0	0
TMEM9B	1	0	1	1	0	0
TMF1	1	0	0	1	0	0
TMLHE	0	1	1	0	0	1
TMOD3	1	0	0	0	0	0
TMOD4	1	1	1	1	1	0
TMPO	1	0	0	0	0	0
TMPRSS4	0	1	1	0	0	0
TMPRSS9	1	1	0	0	0	1
TMSB10	0	0	0	0	1	0
TMTC1	1	0	0	0	0	1
TMTC4	0	0	1	0	0	1
TNC	0	0	0	1	0	0
TNFAIP1	1	0	0	1	0	0
TNFAIP6	0	0	1	0	1	1
TNFAIP8L3	0	0	1	0	0	1
TNFRSF10A	1	0	0	0	0	0
TNFRSF11B	0	0	0	1	0	0
TNFRSF12A	0	0	0	0	0	1
TNFRSF13B	0	1	1	0	0	1
TNFRSF1A	0	0	0	1	0	0
TNFRSF1B	0	1	0	0	0	0
TNFRSF25	0	0	0	0	0	1
TNFRSF9	0	1	0	0	1	0
TNFSF12	1	0	0	1	1	0
TNFSF12-TNFSF13	1	0	0	1	1	0
TNFSF13	1	0	0	1	1	0
TNFSF13B	0	0	0	1	1	0
TNFSF15	0	1	0	0	1	1
TNFSF4	0	1	0	1	0	0
TNFSF5IP1	1	0	0	1	0	0
TNFSF8	0	1	0	0	0	0
TNIP1	1	1	0	1	0	0
TNIP2	0	0	0	1	0	0
TNIP3	0	1	1	1	1	1
TNKS	1	0	0	1	0	0
TNKS1BP1	1	0	0	0	0	1
TNN	0	0	1	0	0	1
TNNI3K	1	1	0	1	0	0
TNNT1	0	0	0	0	0	1
TNP2	0	0	1	0	0	1
TNPO1	0	0	0	1	0	1
TNPO2	1	0	0	0	0	0
TNPO3	1	0	0	1	0	0
TNR	0	0	1	0	0	0
TNRC15	1	0	1	1	0	0
TNRC5	1	0	0	1	0	0
TNRC6A	1	0	1	1	1	1
TNRC6B	1	0	0	0	0	0

TNS1	1	0	1	0	0	1
TNS3	1	0	1	0	0	1
TNS4	0	0	1	0	0	1
TOB1	1	0	0	1	0	1
TOE1	1	1	0	0	1	0
TOM1L2	1	0	0	1	0	0
TOMM34	1	0	0	0	0	0
TOMM40	1	0	0	1	1	1
TOMM7	0	0	0	0	0	1
TOMM70A	1	0	0	1	1	1
TOP2A	0	0	0	0	1	0
TOP3A	1	0	0	0	1	0
TOPORS	1	0	0	1	0	0
TOR1A	1	0	0	1	0	0
TOR1AIP1	1	0	0	1	0	0
TOR1AIP2	1	0	1	1	0	0
TOR1B	0	0	0	1	0	0
TOR2A	1	0	0	1	0	1
TOR3A	1	0	0	1	0	0
TP53	1	0	0	1	0	0
TP53AP1	1	0	0	1	0	1
TP53BP1	1	0	0	1	0	0
TP53I11	0	0	0	1	0	0
TP53I3	1	0	0	0	0	0
TP53INP1	1	0	0	1	0	1
TP53RK	0	1	1	0	1	1
TPCN1	1	0	0	0	0	0
TPD52L2	0	0	0	0	1	0
TPD52L3	0	0	1	0	0	1
TPM3	0	1	0	0	1	0
TPM4	1	0	0	1	0	0
TPMT	1	0	0	1	0	0
TPO	0	1	1	0	1	1
TPP1	1	1	0	1	0	0
TPP2	1	0	0	1	0	0
TPRKB	1	0	0	1	0	0
TPRX1	0	0	1	0	0	1
TPST2	0	0	0	0	0	1
TPT1	1	0	0	0	0	0
TRA16	0	0	0	1	0	0
TRA2A	0	0	0	1	0	0
TRADD	1	0	0	1	0	0
TRAF3IP2	0	1	0	0	0	0
TRAF5	0	0	1	1	0	1
TRAK1	0	0	1	0	0	1
TRAK2	0	0	0	1	0	0
TRAPPC1	1	0	0	1	0	0
TRAPPC2	1	0	0	0	0	0
TRAPPC4	1	0	0	1	0	0
TRAPPC6B	0	0	0	1	0	0
TRDN	0	0	1	0	0	1
TREM2	1	0	1	1	0	1
TREML1	0	0	0	1	0	1
TREML4	0	0	1	1	0	1
TREX1	1	0	1	1	0	0
TRIAD3	1	0	0	0	1	0
TRIAP1	1	0	0	1	0	0
TRIB1	1	0	0	0	0	0
TRIB2	1	0	0	0	0	0

TRIB3	0	0	0	0	0	1
TRIM11	1	0	0	1	0	0
TRIM15	0	0	1	0	0	1
TRIM17	1	0	0	1	0	0
TRIM2	0	0	1	0	0	1
TRIM23	1	0	0	1	0	0
TRIM25	1	0	0	0	1	0
TRIM29	1	1	1	0	0	1
TRIM3	1	0	0	0	0	0
TRIM32	0	1	0	0	0	0
TRIM34	0	0	0	1	0	0
TRIM35	1	0	0	1	1	0
TRIM38	0	0	0	0	1	0
TRIM39	1	0	0	1	0	1
TRIM4	1	0	0	1	0	1
TRIM41	1	0	0	0	0	0
TRIM43	0	0	1	0	0	1
TRIM45	0	0	0	1	0	0
TRIM5	0	0	0	1	0	0
TRIM54	1	0	1	1	0	1
TRIM6	0	1	0	0	0	0
TRIM61	1	0	1	0	0	0
TRIM65	0	0	0	1	0	0
TRIM67	0	0	0	0	0	1
TRIM73	1	0	1	1	0	1
TRIOBP	0	1	0	0	1	0
TRIP10	1	0	0	1	0	0
TRIP11	0	1	0	0	1	0
TRIP12	1	0	0	1	0	0
TRIP13	1	0	0	1	0	0
TRIP4	1	0	0	0	0	0
TRIP6	1	0	0	1	1	0
TRIT1	1	0	0	1	0	0
TRMT1	1	0	0	1	0	0
TRMT12	1	0	0	0	0	0
TRMU	1	0	0	0	0	0
TROVE2	1	0	0	1	0	0
TRPC4AP	0	0	0	0	1	0
TRPC5	0	0	1	0	0	1
TRPM3	1	0	1	1	0	1
TRPM7	1	0	0	1	0	1
TRPS1	0	0	0	1	0	0
TRPV2	1	0	0	1	0	0
TRPV3	0	0	1	0	0	1
TRPV6	0	1	1	0	0	1
TRUB1	0	1	0	0	0	0
TRUB2	1	0	0	1	0	0
TSC22D1	0	1	0	0	0	0
TSC22D2	1	0	0	1	0	0
TSC22D3	0	1	1	0	0	0
TSC22D4	1	0	0	1	0	0
TSFM	1	0	0	1	0	1
TSG101	1	0	0	1	0	0
TSGA14	1	0	0	1	0	0
TSHZ1	0	0	1	1	0	1
TSHZ3	0	1	1	0	0	1
TSKS	0	0	1	1	0	1
TSN	1	0	0	1	0	0
TSNAX	0	0	0	1	0	0

TSP50	0	1	1	0	0	0
TSPAN1	0	0	0	0	0	1
TSPAN10	0	0	1	0	0	1
TSPAN14	1	0	0	1	0	1
TSPAN18	0	1	1	0	0	1
TSPAN3	1	0	0	1	0	0
TSPAN31	0	0	0	1	1	0
TSPAN4	1	0	0	1	0	0
TSPAN7	0	1	1	0	0	0
TSPYL1	0	0	0	1	0	0
TSPYL6	1	0	1	0	0	1
TSR1	1	0	0	1	0	0
TSSC1	1	0	0	1	0	0
TSSC4	1	0	0	1	1	0
TSSK6	1	0	0	1	0	0
TST	1	0	1	0	0	1
TSTA3	1	0	0	1	0	0
TTBK2	0	1	0	0	0	0
TTC1	0	0	0	0	0	1
TTC13	1	0	0	1	0	1
TTC14	1	0	0	0	0	0
TTC15	1	0	0	1	0	0
TTC16	0	0	0	1	0	0
TTC17	0	0	0	1	0	0
TTC21A	1	0	0	0	0	0
TTC23	1	0	0	1	0	0
TTC25	1	0	0	1	0	0
TTC3	1	0	1	0	0	1
TTC7A	0	0	0	1	0	0
TTC8	1	0	0	1	0	0
TTF1	1	0	0	1	0	0
TTK	0	1	0	1	0	0
TTLL13	1	0	0	1	0	0
TTLL3	1	0	1	0	0	1
TTLL5	1	0	1	1	0	1
TTN	0	1	1	1	1	0
TTRAP	1	0	1	1	0	1
TTYH2	0	1	0	1	1	0
TUB	0	1	1	0	1	1
TUBB	1	0	0	1	0	0
TUBB1	1	0	1	0	0	1
TUBB2C	0	0	0	1	0	0
TUBB3	0	0	0	0	0	1
TUBB4Q	1	0	1	0	0	1
TUBB6	1	0	0	0	0	0
TUBB8	0	0	1	0	0	1
TUBD1	1	1	0	1	0	0
TUBE1	1	0	0	1	0	0
TUBG1	1	0	0	0	0	0
TUBGCP2	1	0	0	1	1	0
TUBGCP6	1	0	0	1	0	0
TUFM	0	0	0	1	0	0
TUFT1	0	0	0	1	0	0
TULP4	0	0	0	0	1	0
TUSC2	1	0	0	1	0	0
TUSC4	1	0	0	1	1	0
TWISTNB	1	0	1	1	0	0
TWSG1	1	0	0	0	0	0
TXK	0	0	1	0	1	1

TXLNA	0	0	0	1	0	0
TXLNB	1	0	0	0	0	0
TXN2	0	0	0	1	0	0
TXNDC10	1	0	0	0	0	0
TXNDC11	1	0	0	1	0	0
TXNDC12	1	0	0	1	0	0
TXNDC13	1	1	0	0	0	0
TXNDC14	1	0	0	1	0	0
TXNDC3	0	0	1	0	0	1
TXNDC4	1	0	0	1	0	0
TXNDC5	0	0	1	0	0	0
TXNDC9	1	0	0	1	0	0
TXNIP	1	0	0	1	0	0
TXNL1	1	0	0	0	0	0
TXNL4A	1	0	0	1	0	0
TXNL4B	1	0	0	1	0	0
TXNL5	1	0	0	1	0	0
TXNL6	0	0	1	0	0	0
TXNRD1	1	1	0	1	0	1
TXNRD2	0	0	0	0	1	0
TYROBP	0	0	0	0	1	0
TYSND1	1	0	0	1	0	0
U2AF1	0	0	0	1	0	0
U2AF1L4	1	0	0	1	1	0
UAP1L1	1	0	0	1	0	0
UBA52	0	0	0	1	0	0
UBAP2	1	0	0	0	0	0
UBAP2L	1	0	0	1	0	0
UBB	1	0	0	1	0	0
UBC	1	0	0	1	0	0
UBD	0	1	1	0	0	1
UBE1	1	1	1	1	0	0
UBE1C	1	0	0	1	0	0
UBE1DC1	1	0	0	1	0	0
UBE1L2	0	0	1	1	0	0
UBE2A	1	0	0	1	0	1
UBE2B	1	0	0	1	0	1
UBE2C	1	0	0	0	1	0
UBE2D2	0	0	0	1	0	0
UBE2D3	1	0	1	1	0	1
UBE2D4	0	0	0	1	1	0
UBE2G2	1	0	0	0	0	0
UBE2H	0	0	0	1	0	0
UBE2J2	0	0	0	1	0	0
UBE2L6	0	0	0	0	0	1
UBE2M	1	0	0	1	0	0
UBE2O	1	0	0	1	0	0
UBE2Q1	1	1	0	1	0	0
UBE2R2	1	0	0	1	0	0
UBE2T	0	0	0	1	0	0
UBE2V1	1	1	0	0	1	0
UBE2W	1	0	0	0	0	0
UBE2Z	1	0	0	1	0	0
UBE3A	1	1	1	1	0	0
UBE3B	1	0	0	1	1	0
UBE3C	0	0	0	1	0	0
UBE4A	1	0	0	1	0	0
UBE4B	1	0	0	1	0	0
UBIAD1	1	0	0	1	0	0

UBL3	1	0	0	0	0	0
UBL4A	1	1	1	0	0	0
UBL5	1	0	0	1	0	0
UBL7	1	1	0	1	0	0
UBN1	0	0	0	1	0	0
UBOX5	0	0	0	1	0	0
UBQLN1	1	0	0	0	1	0
UBQLN2	1	0	0	1	0	0
UBQLN3	0	0	1	0	0	1
UBQLN4	1	0	0	1	0	0
UBR1	0	0	0	1	0	0
UBTD1	1	0	0	0	0	0
UBTF	1	0	0	1	0	0
UBXD2	0	0	0	1	0	0
UBXD5	0	0	0	1	1	0
UCHL3	0	0	0	1	0	0
UCHL5	1	0	0	1	0	0
UCK2	0	1	0	0	0	0
UCN	0	0	1	0	0	1
UCN3	0	1	1	0	0	1
UCP3	1	0	1	0	0	1
UCRC	1	0	0	1	0	0
UFC1	1	0	0	1	0	0
UFD1L	1	0	0	1	0	0
UFM1	0	0	0	1	0	0
UGCGL1	1	0	0	0	0	0
UGDH	1	0	0	0	0	0
UGT2B11	0	0	1	0	0	1
UGT2B17	0	0	1	0	0	0
ULK3	1	0	0	1	0	0
UMOD	1	1	1	0	0	1
UNC119	0	0	0	0	1	0
UNC13D	0	0	1	0	0	1
UNC45A	0	0	0	1	0	0
UNC50	1	0	0	1	1	0
UNC5CL	0	0	1	0	0	1
UNC84A	1	0	1	1	0	1
UNC93A	0	0	1	0	0	1
UNQ1940	1	1	1	0	1	1
UNQ473	0	1	1	0	0	1
UNQ501	1	1	0	0	1	0
UNQ5830	0	0	0	0	0	1
UNQ846	0	1	1	0	0	1
UPF3A	1	0	0	1	0	1
UPK3B	0	0	1	0	0	0
UQCRB	0	0	0	1	0	0
UQCRC2	1	0	0	1	0	1
UQCRFS1	0	0	0	1	0	0
UQCRH	1	0	0	0	0	0
UQCRQ	1	0	0	1	0	1
UROD	1	0	0	1	0	0
UROS	1	0	0	1	0	0
USF1	0	0	0	1	0	0
USH2A	0	1	1	0	0	1
USH3A	1	1	1	0	0	1
USHBP1	1	0	0	1	0	0
USMG5	1	0	0	1	1	0
USP1	1	0	0	1	1	0
USP10	0	1	0	1	0	0

USP15	1	0	0	0	0	0
USP16	1	0	0	1	0	0
USP19	1	0	0	1	1	0
USP2	0	0	0	1	0	0
USP20	1	0	0	1	0	0
USP21	1	0	0	1	0	0
USP25	0	0	0	1	0	0
USP3	1	0	1	0	1	1
USP30	0	0	0	1	0	0
USP32	1	0	0	1	0	0
USP33	0	1	0	0	0	1
USP34	1	0	1	1	0	0
USP35	1	0	0	0	0	0
USP36	1	0	0	1	0	0
USP37	1	0	0	1	1	1
USP38	1	0	0	1	0	0
USP39	0	0	0	0	0	1
USP4	1	0	0	1	1	0
USP42	0	0	0	1	0	0
USP49	1	0	0	0	0	0
USP5	0	0	0	1	0	0
USP51	0	1	0	1	1	0
USP52	1	0	0	1	1	0
USP53	0	0	1	0	0	0
USP6	0	0	1	0	0	1
USP9X	1	0	1	0	0	1
USPL1	1	0	0	0	0	0
UTP11L	0	0	0	1	1	0
UTP14A	0	1	0	1	0	0
UTP14C	0	0	1	0	0	1
UTP15	1	0	0	1	0	1
UTP20	0	0	0	1	0	0
UTS2	1	1	1	0	0	0
UTX	0	0	1	0	0	1
UVRAG	0	0	0	1	0	0
UXS1	0	1	0	1	0	0
UXT	0	1	1	1	0	0
VAC14	1	0	1	0	0	1
VAMP3	1	0	1	1	0	1
VAMP5	1	0	1	0	0	1
VAMP8	0	0	1	0	0	1
VARS	0	0	0	1	0	1
VASH1	0	0	0	1	0	0
VASN	1	0	0	0	0	0
VAV1	1	0	0	1	0	0
VAV2	0	0	0	0	1	0
VBP1	0	1	0	1	1	0
VCAM1	0	1	1	0	0	1
VCP	0	0	0	1	0	0
VCPIP1	1	0	0	1	0	0
VDAC1	1	0	0	1	0	0
VDAC2	1	0	0	0	0	0
VDAC3	1	0	0	1	0	0
VDP	0	0	0	0	0	1
VDR	1	0	0	0	0	0
VEPH1	0	1	1	1	0	1
VEZT	1	1	0	0	0	0
VHL	1	0	0	0	0	0
VIM	1	0	0	0	0	0

VKORC1	0	0	1	1	1	1
VMO1	1	0	0	1	0	0
VPRBP	1	0	0	0	1	0
VPREB3	0	0	0	1	0	0
VPS11	1	0	0	1	1	0
VPS13B	1	0	0	0	0	0
VPS13D	0	0	0	1	0	0
VPS16	0	0	0	1	0	0
VPS24	1	0	0	0	0	0
VPS25	1	0	0	1	0	1
VPS26B	1	0	0	1	1	0
VPS29	1	0	0	1	0	1
VPS35	1	0	0	0	1	0
VPS36	1	0	0	1	0	0
VPS37A	1	0	0	1	0	0
VPS37C	1	0	0	1	0	0
VPS39	1	0	0	1	1	0
VPS41	0	0	1	0	0	1
VPS52	1	0	0	1	0	1
VPS53	1	0	0	0	0	0
VRK1	1	0	0	0	0	0
VRK3	1	0	0	1	1	0
VSIG4	0	1	1	1	0	1
VSIG9	0	1	1	1	0	1
VTI1A	1	0	0	1	0	1
VTI1B	1	0	0	0	0	0
VWF	0	0	1	0	0	1
WAPAL	1	0	0	1	0	0
WARS	1	0	0	1	0	0
WAS	0	1	0	0	0	0
WASF2	1	0	0	1	0	0
WASF3	1	0	0	0	0	0
WASL	1	0	1	0	0	0
WBP2	0	0	1	0	0	1
WBSCR18	1	0	0	1	0	0
WBSCR19	0	1	1	0	0	1
WBSCR22	1	0	0	1	0	0
WDFY1	0	0	0	0	1	0
WDFY3	1	0	1	1	1	1
WDHD1	1	0	0	1	1	1
WDR1	1	0	0	0	0	0
WDR12	1	0	0	1	0	0
WDR13	0	1	0	0	0	0
WDR19	1	0	0	0	0	0
WDR20	1	0	0	1	0	0
WDR23	1	0	0	1	1	0
WDR24	0	0	0	1	0	0
WDR25	1	0	0	1	0	0
WDR26	1	0	0	0	0	0
WDR35	0	0	0	1	0	0
WDR37	1	0	0	1	0	0
WDR4	1	0	0	1	1	0
WDR40A	0	1	0	0	0	0
WDR41	0	0	0	1	0	0
WDR44	1	1	0	0	1	0
WDR45	1	1	0	1	1	0
WDR46	1	0	0	1	1	0
WDR48	0	0	0	1	0	0
WDR51A	0	1	0	0	0	0

WDR53	1	0	0	1	0	0
WDR55	1	0	1	1	1	1
WDR57	1	0	0	1	1	0
WDR5B	1	0	0	1	0	0
WDR62	1	0	0	1	0	0
WDR65	1	0	0	1	0	0
WDR66	0	0	0	1	0	0
WDR67	0	1	0	1	0	0
WDR7	0	0	1	0	0	0
WDR71	1	0	0	1	0	0
WDR73	1	0	0	1	0	0
WDR74	1	0	0	0	0	0
WDR75	0	0	0	1	0	0
WDR77	1	1	0	1	1	0
WDR79	1	0	0	1	0	0
WDR81	1	0	0	1	0	1
WDSOF1	1	0	0	1	1	1
WDSUB1	1	0	0	0	0	0
WEE1	0	0	0	1	0	0
WFIKKN2	0	0	1	0	0	1
WHSC1	1	0	1	1	0	1
WHSC1L1	1	0	0	1	0	0
WIBG	1	0	0	0	0	0
WIPI1	1	0	0	1	0	0
WNK1	0	0	1	1	1	0
WNT5A	1	0	0	0	0	0
WNT5B	1	1	1	0	1	1
WRN	1	0	0	1	0	0
WSB1	1	0	0	1	0	0
WSB2	1	0	0	0	0	0
WTAP	1	0	0	1	1	0
WWC2	1	1	1	1	1	1
WWOX	1	1	1	1	0	1
WWP2	0	0	0	1	0	1
XAB1	1	1	0	1	0	0
XAB2	1	0	0	1	1	1
XCR1	0	1	0	0	1	1
XKR3	1	0	1	0	0	0
XKR4	0	0	1	0	0	0
XKR6	0	0	1	0	0	1
XPA	0	1	0	0	1	0
XPC	1	0	0	1	1	1
XPNPEP1	1	0	0	1	0	0
XPNPEP2	0	1	1	0	1	0
XPO5	1	0	0	1	0	0
XPO6	1	0	1	1	1	0
XPR1	1	0	0	1	0	1
XRCC3	0	1	0	0	0	0
XRCC4	0	0	0	1	1	0
XRCC5	1	0	0	0	0	0
XRCC6	1	0	0	1	0	0
XRN1	1	0	0	1	0	0
XRN2	1	0	0	1	0	0
XYLB	0	1	1	0	0	0
XYLT1	1	0	0	1	0	0
YARS	1	0	0	1	0	1
YARS2	1	0	0	1	0	0
YIF1B	1	0	1	1	1	1
YIPF1	1	0	1	1	0	1

YIPF2	1	0	0	1	0	0
YIPF3	1	1	0	1	0	0
YIPF4	1	0	0	1	0	0
YIPF5	1	0	0	1	0	1
YIPF6	0	1	0	0	0	0
YIPF7	0	1	0	0	0	1
YME1L1	1	0	0	0	0	0
YOD1	0	0	0	1	0	0
YPEL5	0	0	0	1	0	0
YRDC	1	0	0	1	1	0
YTHDC2	1	0	0	1	0	1
YTHDF1	1	0	0	0	0	0
YTHDF2	0	0	0	1	0	0
YTHDF3	1	0	0	1	0	0
YWHAH	0	1	0	0	0	0
YWHAZ	0	0	0	1	0	0
YY1	1	0	0	0	0	0
YY1AP1	1	0	0	0	0	0
ZADH2	1	0	0	0	0	0
ZAK	1	0	0	0	0	0
ZAP70	0	0	1	0	0	1
ZBED3	1	0	1	1	0	1
ZBP1	0	0	0	0	0	1
ZBTB11	1	0	0	1	0	0
ZBTB17	0	1	0	1	0	0
ZBTB2	1	1	0	1	0	1
ZBTB22	1	0	0	1	1	0
ZBTB24	0	0	0	1	0	0
ZBTB26	1	0	0	1	0	0
ZBTB3	0	0	0	1	0	0
ZBTB32	0	0	1	0	0	1
ZBTB39	1	1	1	1	0	1
ZBTB4	1	0	0	0	0	0
ZBTB40	1	1	0	0	1	0
ZBTB9	0	0	0	0	0	1
ZC3H10	1	0	0	1	0	0
ZC3H11A	1	1	0	1	1	0
ZC3H12A	1	0	0	1	0	0
ZC3H3	0	0	0	1	0	0
ZC3H7A	1	0	1	0	0	1
ZC3H8	0	1	0	0	1	0
ZC3HAV1	1	0	0	0	0	0
ZCCHC14	0	0	0	0	0	1
ZCCHC17	1	0	0	1	1	0
ZCCHC2	1	0	1	1	0	1
ZCCHC3	1	1	0	1	0	0
ZCCHC9	1	0	0	1	0	0
ZCRB1	1	0	0	1	0	0
ZDHHC12	1	0	0	0	0	0
ZDHHC14	0	1	0	1	0	0
ZDHHC16	1	0	0	1	0	0
ZDHHC17	0	0	0	0	1	0
ZDHHC19	1	0	0	0	0	0
ZDHHC20	1	0	0	1	0	0
ZDHHC23	0	1	0	0	0	0
ZDHHC3	1	0	0	1	0	0
ZDHHC4	1	1	0	1	0	0
ZDHHC5	1	0	0	1	0	0
ZDHHC6	1	0	0	1	0	1

ZDHHC9	0	1	0	0	0	0
ZFAND1	1	0	0	1	0	0
ZFAND2A	1	0	0	1	0	0
ZFAND3	1	0	0	1	0	0
ZFP106	1	0	1	0	0	1
ZFP161	1	0	0	1	1	0
ZFP36	0	1	0	0	1	0
ZFP91	1	0	1	1	1	0
ZFPL1	1	0	0	1	1	0
ZFR	0	1	0	0	0	0
ZFYVE1	0	0	0	0	0	1
ZFYVE19	1	1	0	0	0	0
ZFYVE21	0	1	0	0	0	0
ZHX3	0	0	1	0	0	1
ZKSCAN1	0	0	0	1	0	0
ZMAT1	1	1	1	0	0	1
ZMAT2	1	0	0	1	0	0
ZMAT4	0	0	1	0	0	1
ZMAT5	1	0	0	1	0	0
ZMPSTE24	1	0	0	1	0	0
ZMYM3	0	1	0	0	0	0
ZMYM4	0	1	0	1	0	0
ZMYM6	1	0	0	1	0	0
ZMYND10	1	0	0	1	0	0
ZMYND11	0	0	0	1	0	0
ZMYND12	1	0	0	1	0	0
ZMYND15	0	0	0	0	0	1
ZMYND17	0	0	1	0	0	1
ZMYND19	0	1	0	1	0	0
ZNF10	0	0	0	1	0	0
ZNF12	0	1	0	0	0	0
ZNF121	1	0	0	1	0	0
ZNF132	1	0	0	1	0	0
ZNF133	0	0	0	1	0	0
ZNF134	0	0	0	1	0	0
ZNF135	0	1	1	0	0	1
ZNF136	1	0	0	0	0	0
ZNF138	0	1	0	0	0	0
ZNF140	1	0	0	0	0	0
ZNF142	1	0	0	0	0	0
ZNF146	0	0	0	0	1	0
ZNF148	0	0	0	1	0	0
ZNF157	0	0	1	0	0	1
ZNF169	1	0	0	0	0	0
ZNF17	1	1	0	0	0	0
ZNF174	1	0	0	1	0	0
ZNF175	1	0	0	0	0	0
ZNF177	0	0	1	0	0	0
ZNF179	0	0	1	0	0	1
ZNF180	1	0	0	0	0	0
ZNF181	1	0	0	1	0	0
ZNF184	1	0	0	0	0	0
ZNF185	0	0	1	0	0	0
ZNF2	1	0	0	0	0	0
ZNF20	0	1	0	0	1	0
ZNF200	1	0	0	0	0	0
ZNF207	1	0	0	0	0	0
ZNF211	1	0	0	1	0	0
ZNF213	1	0	0	1	0	0

ZNF214	0	1	0	0	0	0
ZNF217	1	0	0	1	0	0
ZNF219	1	0	0	1	0	0
ZNF222	1	0	0	1	0	0
ZNF226	1	0	0	1	0	0
ZNF227	1	0	0	0	0	0
ZNF228	1	0	0	1	0	0
ZNF23	0	1	1	0	0	0
ZNF230	1	0	0	1	0	0
ZNF232	0	0	1	0	0	1
ZNF234	1	0	0	1	0	0
ZNF235	0	1	0	1	0	0
ZNF239	0	0	1	0	0	1
ZNF24	1	0	0	1	1	0
ZNF248	1	0	0	0	0	0
ZNF259	1	0	0	1	0	0
ZNF263	0	0	0	1	0	0
ZNF268	1	0	0	1	0	0
ZNF271	0	0	0	0	0	1
ZNF274	0	0	0	1	0	0
ZNF282	0	0	0	1	0	0
ZNF283	0	0	0	1	0	0
ZNF289	1	0	0	0	0	0
ZNF294	0	0	0	0	1	0
ZNF3	1	0	0	1	0	0
ZNF30	0	1	0	0	0	0
ZNF304	1	0	0	1	0	0
ZNF317	1	0	0	1	0	0
ZNF318	0	1	0	1	0	0
ZNF322B	0	0	0	0	0	1
ZNF323	1	0	0	1	1	0
ZNF324	1	0	0	1	0	0
ZNF326	0	1	0	0	0	0
ZNF333	1	0	0	0	0	0
ZNF335	1	0	0	0	0	0
ZNF337	1	0	0	0	0	0
ZNF33A	1	0	0	0	0	0
ZNF342	1	0	0	0	0	0
ZNF343	1	0	0	1	0	0
ZNF345	0	0	0	1	0	0
ZNF350	1	0	0	1	0	0
ZNF358	1	0	1	1	0	1
ZNF364	1	0	0	1	0	0
ZNF365	0	0	1	0	0	1
ZNF366	1	0	1	1	0	1
ZNF367	1	0	0	1	0	0
ZNF384	1	1	0	1	0	0
ZNF385	0	1	0	1	1	0
ZNF395	0	0	0	1	0	0
ZNF397	1	0	0	1	1	1
ZNF398	1	0	0	0	0	1
ZNF403	1	0	0	1	0	0
ZNF404	0	0	0	1	1	0
ZNF407	0	0	0	0	1	0
ZNF408	1	0	0	1	0	0
ZNF41	1	1	0	0	0	0
ZNF410	1	0	0	1	0	0
ZNF420	1	0	0	0	0	0
ZNF425	1	0	0	0	0	0

ZNF431	0	0	0	1	0	0
ZNF434	1	0	0	1	0	0
ZNF436	1	0	0	1	0	0
ZNF444	1	0	0	1	0	0
ZNF446	1	0	0	1	0	0
ZNF45	1	0	1	0	1	1
ZNF451	1	0	0	0	0	0
ZNF452	0	0	0	1	0	0
ZNF473	1	0	0	1	1	0
ZNF484	0	0	0	0	0	1
ZNF496	1	0	0	1	0	0
ZNF498	0	1	1	1	1	1
ZNF509	1	0	0	1	0	0
ZNF511	1	0	0	1	0	0
ZNF512	0	1	0	0	0	1
ZNF513	1	1	0	1	1	0
ZNF524	1	0	0	1	0	0
ZNF526	1	0	0	1	1	0
ZNF529	1	0	0	1	0	0
ZNF530	0	1	0	1	1	0
ZNF536	0	0	1	1	0	1
ZNF541	0	0	0	0	0	1
ZNF544	0	0	0	1	0	0
ZNF550	1	1	0	0	0	0
ZNF554	0	0	0	1	0	1
ZNF558	0	0	1	0	1	0
ZNF561	0	0	0	1	0	1
ZNF564	1	0	0	0	0	0
ZNF567	1	0	0	1	0	0
ZNF569	1	0	0	1	0	0
ZNF570	1	0	0	1	0	0
ZNF576	1	0	0	0	0	0
ZNF582	1	0	0	1	0	0
ZNF583	1	0	0	0	0	0
ZNF585A	1	1	0	0	1	0
ZNF586	0	0	0	1	0	0
ZNF589	1	0	0	1	0	0
ZNF592	1	0	0	0	0	0
ZNF597	1	0	0	1	0	0
ZNF606	1	0	0	0	0	0
ZNF609	0	0	1	1	0	1
ZNF610	0	1	0	0	0	0
ZNF611	1	0	0	1	0	0
ZNF613	0	0	0	0	1	0
ZNF614	1	0	0	0	0	0
ZNF621	0	0	0	0	1	0
ZNF622	1	0	0	0	0	0
ZNF624	0	0	1	0	0	0
ZNF628	1	0	0	0	0	1
ZNF630	0	1	1	0	1	0
ZNF644	1	0	0	0	0	0
ZNF646	1	0	0	1	0	0
ZNF650	0	0	1	0	0	1
ZNF653	1	0	0	0	1	0
ZNF654	0	0	0	1	0	0
ZNF655	1	0	0	1	0	0
ZNF658	1	0	0	0	0	0
ZNF658B	0	0	0	1	0	1
ZNF663	0	0	1	0	0	0

ZNF664	1	0	0	1	0	0
ZNF668	1	0	0	1	0	0
ZNF672	1	0	0	1	1	0
ZNF673	0	1	1	0	0	1
ZNF675	0	1	0	0	0	0
ZNF679	1	0	0	1	0	0
ZNF683	0	0	1	0	0	1
ZNF684	0	1	0	0	0	0
ZNF69	1	0	0	0	0	0
ZNF691	1	0	0	1	0	1
ZNF692	1	0	0	1	1	0
ZNF696	1	0	0	1	0	0
ZNF700	0	0	0	1	0	0
ZNF706	1	0	0	0	0	0
ZNF713	0	0	1	0	0	1
ZNF714	0	0	0	0	1	0
ZNF720	0	0	1	0	0	0
ZNF721	1	0	0	1	0	0
ZNF740	1	0	0	1	0	0
ZNF747	1	0	0	1	0	0
ZNF75	1	1	0	1	1	0
ZNF75A	1	0	0	0	0	0
ZNF76	1	0	0	0	1	0
ZNF77	1	0	0	0	1	0
ZNF79	1	0	0	0	1	0
ZNF84	1	0	0	0	0	0
ZNFX1	1	0	0	0	0	0
ZNHIT1	1	0	0	1	0	0
ZNHIT2	1	0	0	1	1	0
ZNHIT3	0	0	0	0	1	0
ZNHIT4	1	0	0	1	0	0
ZNRD1	1	0	0	1	1	0
ZNRF2	1	0	0	0	0	0
ZP4	0	0	1	0	0	1
ZRANB1	0	0	0	0	0	1
ZRANB3	1	0	0	1	1	0
ZSCAN2	1	0	0	1	1	0
ZSCAN5	0	0	1	0	0	1
ZSWIM1	1	0	0	1	1	0
ZSWIM4	1	0	0	0	0	0
ZW10	1	0	0	1	1	0
ZWILCH	1	0	0	0	0	0
ZWINT	1	0	0	0	0	0
ZXDB	0	1	0	0	1	1
ZXDC	0	1	0	0	0	0
ZYX	1	0	0	0	0	0
ZZEF1	1	0	0	1	0	0
ZZZ3	1	0	0	0	0	0
BA16L21.2.1	1	1	0	0	0	0
DJ341D10.1	0	1	1	0	1	1
AIP	0	0	1	0	0	1
MGC40168	1	0	0	1	0	0
IIP45	0	1	1	1	0	1
EIF3I	1	0	0	1	0	0
ZRANB2	0	1	1	0	1	1
LOC149620	0	0	0	1	0	0
SEC22B	1	0	0	0	0	0
ATP1A1	0	0	0	0	0	1
FCRLA	0	0	0	1	0	0

QSOX1	1	0	0	1	0	0
SMC6	0	0	0	1	0	0
HADH	0	0	0	1	0	1
FOXN2	1	0	0	0	0	0
CA11	0	0	0	1	0	0
REGL	0	0	1	0	0	1
CIAO1	1	0	0	0	0	0
REV1	1	0	0	0	0	1
ZEB2	1	0	0	1	1	0
SPC25	1	0	0	0	0	0
GALNT7	0	0	0	0	0	1
DPH3	0	0	0	1	0	0
SEC22C	1	0	0	1	0	0
CNBP	0	0	1	0	0	1
B3GALNT1	1	0	0	0	0	0
APM-1	0	0	1	0	0	1
SEC31A	1	1	1	1	1	1
DSP	0	0	1	0	0	1
NAIP	0	0	1	0	0	1
POLQ	0	0	1	0	0	1
CNR2	1	0	0	1	1	0
LOC340156	0	0	0	1	0	0
MED20	0	1	0	0	1	0
VEGFA	1	0	0	1	0	0
MCM9	1	0	0	0	0	0
MAGI1	1	0	0	1	0	0
EIF3B	1	0	0	1	1	0
NPSR1	1	0	0	0	0	0
EIF4H	0	0	0	1	0	0
ZSCAN21	1	0	0	1	0	0
MGA	0	0	1	0	0	1
DEF6	1	0	0	1	0	0
FAM110B	0	0	0	0	0	1
EIF3E	1	0	0	1	0	0
MED30	1	0	0	1	0	0
RIF1	1	0	0	0	0	0
LOC441459	0	1	1	0	1	1
RMI1	0	0	1	0	0	1
RC3H2	0	0	1	0	0	1
BMI1	1	0	1	0	0	1
ZCD1	0	1	1	0	0	1
PSAP	1	0	0	1	0	0
LCOR	1	0	0	1	0	0
SMC3	1	0	0	1	0	0
CTR9	1	0	0	1	0	0
DPH4	0	0	0	0	0	1
CLP1	1	0	1	1	0	0
RELT	1	0	0	1	0	0
RSF1	0	1	1	0	0	1
ERC1	0	1	1	0	0	1
CD27	0	1	1	0	1	1
LOC144983	1	1	0	1	1	0
TIFA	1	0	0	1	0	0
MGC13168	0	0	1	0	0	1
APPL2	1	0	0	0	0	0
ANKRD13A	0	0	1	0	0	1
TECT1	1	1	1	0	0	1
C12ORF8	1	0	0	1	0	0
DNCL1	1	0	1	0	1	1

LOC387921	0	0	1	0	0	1
KIAA0564	1	0	0	0	0	1
LOC220416	0	1	1	0	1	1
LOC440145	0	0	1	0	0	1
DIS3	1	0	0	1	0	0
RBM26	0	0	1	0	0	1
C13ORF16	0	0	1	1	0	1
IL25	0	1	1	0	0	1
REC8	1	1	1	1	0	1
EAPP	1	0	0	0	0	0
TXNDC1	1	0	0	1	1	0
SERPINA11	1	0	0	1	0	0
KLC1	1	0	0	0	0	0
EIF3J	1	0	0	1	0	0
EID1	1	0	1	1	0	1
LIPH	1	0	0	1	0	0
EDC3	1	0	0	1	1	0
ZFAND6	0	0	0	0	1	1
HN1L	0	0	0	1	0	0
KIAA0430	0	0	1	0	0	1
CTF8	0	0	0	1	0	0
NOB1	0	1	1	0	0	1
CHMP1A	1	0	0	1	0	0
FRAG1	0	0	0	1	0	0
GPR158L1	1	0	1	1	0	1
EPO	0	1	1	1	0	1
NACA2	0	1	1	0	0	1
KIAA1303	0	0	1	0	0	1
ELP2	0	1	0	0	1	0
APM-1	0	0	1	0	0	1
CDH1	0	0	1	0	0	1
EIF3G	1	0	0	1	1	0
MED26	1	0	0	1	0	0
NCAN	1	0	0	1	0	0
TBCB	1	0	0	1	0	0
EIF3K	0	1	0	0	0	0
MED29	0	0	1	0	0	1
EID2	0	0	1	1	0	1
PLAC8	0	1	0	0	0	0
CPA6	0	0	0	1	1	0
KLK7	1	0	0	1	0	1
FIZ1	1	0	0	0	0	0
ZSCAN22	0	0	1	0	0	1
MZF1	0	1	1	0	0	1
FAM110A	1	0	0	1	0	1
SIRPB2	0	0	1	0	0	1
KIAA1434	1	1	0	1	0	1
GZF1	1	1	1	0	0	1
PIGU	0	0	1	0	0	0
MYH14	0	0	1	0	0	1
SNX21	1	0	0	0	1	0
CTSA	1	0	0	1	0	0
ZMYND8	0	0	0	1	0	0
C20ORF107	0	0	0	1	0	0
SRM	0	0	0	1	0	0
N6AMT1	1	0	0	1	0	0
PWP2	0	0	0	1	1	0
LOC91353	1	0	0	0	0	1
LOC402055	0	0	1	1	0	1

HSCB	0	0	0	1	0	0
PVALB	1	0	0	1	1	0
FLJ46257	0	0	0	1	0	0
LOC401589	1	1	1	0	0	1
HSD17B10	1	0	0	1	0	1
FAM104B	1	0	0	1	0	0
LOC340527	0	0	0	0	1	0
DKFZP564K142	1	0	0	1	0	0
TAF9L	1	0	1	1	0	0
SRPX	0	0	0	1	0	0
CENPI	1	0	0	1	0	0
LOC255313	1	0	0	0	0	0
NOV	1	0	0	1	0	0
