# Supplementary material for: Genome-wide promoter analysis of histone modifications in human monocyte-derived antigen presenting cells
Source: BMC Genomics. 2010 Nov 18;11:642. doi: 10.1186/1471-2164-11-642 (PMC3091769; doi:10.1186/1471-2164-11-642)
Supplement: Additional file 2 — Tserel et al BMC Genomics. Contains Supplementary Tables S2-S6. Size 7.2 MB [file 1471-2164-11-642-S2.ZIP › Supplementary Table 2. GO analysis.pdf]

Supplementary Table 2. GO pathways based on monocyte peaks.

DC H3K4me3

Significance	I	II	III	ID	Functional group
6.69e-09	4068	2042	455	GO:0023052	signaling
1.17e-08	3405	2042	389	GO:0023046	signaling process
1.13e-08	3404	2042	389	GO:0023060	signal transmission
1.17e-08	2999	2042	349	GO:0007165	signal transduction
2.82e-10	4630	2042	518	GO:0032501	multicellular organismal process
4.02e-07	1565	2042	195	GO:0003008	system process
2.17e-06	1257	2042	159	GO:0050877	neurological system process
5.36e-06	862	2042	115	GO:0006629	lipid metabolic process
2.97e-11	4000	2042	463	GO:0050896	response to stimulus
1.49e-09	1445	2042	194	GO:0042221	response to chemical stimulus
4.66e-11	1022	2042	153	GO:0009605	response to external stimulus
2.12e-07	693	2042	102	GO:0006952	defense response
8.84e-08	595	2042	92	GO:0009611	response to wounding
1.26e-07	360	2042	63	GO:0006954	inflammatory response
6.57e-09	2151	2042	265	GO:0005576	extracellular region
2.12e-08	1032	2042	144	GO:0044421	extracellular region part
1.37e-09	747	2042	116	GO:0005615	extracellular space
2.90e-06	7719	2042	773	GO:0016020	membrane
1.66e-10	3953	2042	454	GO:0005886	plasma membrane
4.53e-06	2158	2042	249	GO:0044459	plasma membrane part
8.69e-07	2282	2042	266	GO:0060089	molecular transducer activity
8.69e-07	2282	2042	266	GO:0004871	signal transducer activity
4.21e-07	982	2042	133	GO:0005102	receptor binding
2.39e-07	52	2042	18	GO:0042379	chemokine receptor binding
1.20e-07	50	2042	18	GO:0008009	chemokine activity

Significance between the target list and indicated functional category is presented as p-value calculated by Fisher exact test

I - nr of genes in functional group

II - nr of genes with indicated modification change

III - nr of the overlapping genes

DC H3AcH3

Significance	I	II	III	ID	Functional group
6.02e-14	15757	4145	3081	GO:0008150	biological_process
1.48e-74	6921	4145	1767	GO:0008152	metabolic process
1.17e-13	1523	4145	395	GO:0044281	small molecule metabolic process
7.72e-62	4700	4145	1271	GO:0043170	macromolecule metabolic process
1.37e-15	1678	4145	438	GO:0043412	macromolecule modification
9.39e-37	1512	4145	477	GO:0010467	gene expression
4.08e-69	6042	4145	1573	GO:0044238	primary metabolic process
2.15e-26	3189	4145	813	GO:0019538	protein metabolic process
3.73e-07	1193	4145	289	GO:0006508	proteolysis
3.59e-17	1384	4145	380	GO:0009056	catabolic process
6.67e-17	858	4145	258	GO:0009057	macromolecule catabolic process
3.93e-14	679	4145	206	GO:0030163	protein catabolic process
2.56e-49	2404	4145	725	GO:0006807	nitrogen compound metabolic process
3.25e-30	2052	4145	581	GO:0009058	biosynthetic process
6.61e-25	1260	4145	380	GO:0009059	macromolecule biosynthetic process
8.92e-08	714	4145	189	GO:0055114	oxidation reduction
3.90e-11	2725	4145	633	GO:0016043	cellular component organization
1.02e-06	830	4145	209	GO:0043933	macromolecular complex subunit organization
6.45e-56	10377	4145	2349	GO:0009987	cellular process
7.17e-17	808	4145	246	GO:0007049	cell cycle
5.06e-12	383	4145	127	GO:0000278	mitotic cell cycle
3.42e-11	1143	4145	300	GO:0051641	cellular localization
2.09e-15	596	4145	190	GO:0022402	cell cycle process
3.45e-10	430	4145	133	GO:0022403	cell cycle phase
6.33e-09	344	4145	108	GO:0000279	M phase
2.70e-06	231	4145	72	GO:0000087	M phase of mitotic cell cycle
4.92e-16	1455	4145	391	GO:0006996	organelle organization
2.65e-06	235	4145	73	GO:0048285	organelle fission
3.97e-06	225	4145	70	GO:0000280	nuclear division
3.97e-06	225	4145	70	GO:0007067	mitosis
1.85e-07	564	4145	154	GO:0051276	chromosome organization
7.67e-17	931	4145	275	GO:0051716	cellular response to stimulus
5.07e-89	5724	4145	1571	GO:0044237	cellular metabolic process
2.03e-73	4126	4145	1185	GO:0044260	cellular macromolecule metabolic process
8.96e-34	2670	4145	732	GO:0044267	cellular protein metabolic process
4.86e-14	1588	4145	411	GO:0006464	protein modification process
3.61e-08	213	4145	73	GO:0070647	protein modification by small protein conjugation or removal
2.46e-06	182	4145	60	GO:0032446	protein modification by small protein conjugation
1.26e-11	1307	4145	338	GO:0043687	post-translational protein modification
1.94e-06	169	4145	57	GO:0016567	protein ubiquitination
1.28e-51	2284	4145	704	GO:0034641	cellular nitrogen compound metabolic process
4.13e-56	1987	4145	646	GO:0006139	nucleobase, nucleoside, nucleotide and nucleic acid metabolic process

6.65e-51	1606	4145	539	GO:0090304	nucleic acid metabolic process
5.84e-16	557	4145	182	GO:0006259	DNA metabolic process
3.04e-07	109	4145	43	GO:0006310	DNA recombination
1.12e-34	1056	4145	359	GO:0016070	RNA metabolic process
9.17e-13	294	4145	106	GO:0034660	ncRNA metabolic process
1.29e-06	171	4145	58	GO:0006399	tRNA metabolic process
3.82e-08	106	4145	44	GO:0016072	rRNA metabolic process
8.03e-15	400	4145	139	GO:0016071	mRNA metabolic process
2.18e-27	609	4145	225	GO:0006396	RNA processing
1.25e-11	204	4145	79	GO:0034470	ncRNA processing
3.17e-17	304	4145	119	GO:0008380	RNA splicing
8.76e-09	112	4145	47	GO:0000375	RNA splicing, via transesterification reactions
4.45e-08	103	4145	43	GO:0000377	RNA splicing, via transesterification reactions with bulged adenosine as nucleophile
3.51e-15	332	4145	122	GO:0006397	mRNA processing
4.45e-08	103	4145	43	GO:0000398	nuclear mRNA splicing, via spliceosome
9.94e-07	359	4145	104	GO:0055086	nucleobase, nucleoside and nucleotide metabolic process
1.57e-22	1141	4145	344	GO:0044248	cellular catabolic process
2.16e-17	805	4145	247	GO:0044265	cellular macromolecule catabolic process
2.75e-13	624	4145	190	GO:0043632	modification-dependent macromolecule catabolic process
2.37e-13	665	4145	200	GO:0044257	cellular protein catabolic process
2.87e-13	662	4145	199	GO:0051603	proteolysis involved in cellular protein catabolic process
5.91e-11	112	4145	51	GO:0010498	proteasomal protein catabolic process
2.75e-13	624	4145	190	GO:0019941	modification-dependent protein catabolic process
2.55e-11	266	4145	95	GO:0006511	ubiquitin-dependent protein catabolic process
5.91e-11	112	4145	51	GO:0043161	proteasomal ubiquitin-dependent protein catabolic process
5.93e-10	70	4145	36	GO:0031145	anaphase-promoting complex-dependent proteasomal ubiquitin-dependent protein catabolic process
1.61e-08	221	4145	76	GO:0051186	cofactor metabolic process
1.31e-08	167	4145	62	GO:0006732	coenzyme metabolic process
4.03e-06	33	4145	18	GO:0006084	acetyl-CoA metabolic process
3.14e-10	340	4145	111	GO:0006091	generation of precursor metabolites and energy
4.94e-07	159	4145	56	GO:0015980	energy derivation by oxidation of organic compounds
4.09e-07	110	4145	43	GO:0045333	cellular respiration
4.19e-06	39	4145	20	GO:0009060	aerobic respiration
1.53e-07	647	4145	173	GO:0042180	cellular ketone metabolic process
3.03e-29	1935	4145	551	GO:0044249	cellular biosynthetic process
1.18e-07	291	4145	91	GO:0032774	RNA biosynthetic process
1.16e-06	110	4145	42	GO:0051188	cofactor biosynthetic process
1.46e-25	1234	4145	376	GO:0034645	cellular macromolecule biosynthetic process
7.72e-10	464	4145	140	GO:0006412	translation
4.09e-08	179	4145	64	GO:0006260	DNA replication
3.37e-09	365	4145	114	GO:0006350	transcription
1.20e-07	287	4145	90	GO:0006351	transcription, DNA-dependent
4.17e-07	201	4145	67	GO:0006366	transcription from RNA polymerase II promoter
4.32e-07	643	4145	170	GO:0006082	organic acid metabolic process

7.79e-07	635	4145	167	GO:0043436	oxoacid metabolic process
7.79e-07	635	4145	167	GO:0019752	carboxylic acid metabolic process
1.94e-09	1151	4145	293	GO:0044085	cellular component biogenesis
1.93e-18	203	4145	92	GO:0022613	ribonucleoprotein complex biogenesis
3.30e-10	81	4145	40	GO:0022618	ribonucleoprotein complex assembly
7.70e-11	136	4145	58	GO:0042254	ribosome biogenesis
1.01e-07	102	4145	42	GO:0006364	rRNA processing
4.76e-09	87	4145	40	GO:0051340	regulation of ligase activity
1.88e-12	1275	4145	335	GO:0033036	macromolecule localization
6.88e-10	595	4145	171	GO:0070727	cellular macromolecule localization
2.63e-13	1060	4145	291	GO:0008104	protein localization
4.51e-10	592	4145	171	GO:0034613	cellular protein localization
2.44e-09	3939	4145	863	GO:0019222	regulation of metabolic process
2.20e-10	3554	4145	795	GO:0060255	regulation of macromolecule metabolic process
9.71e-07	3103	4145	674	GO:0010468	regulation of gene expression
2.43e-08	3589	4145	785	GO:0080090	regulation of primary metabolic process
5.08e-09	623	4145	174	GO:0051246	regulation of protein metabolic process
7.85e-10	212	4145	77	GO:0051248	negative regulation of protein metabolic process
2.56e-08	3772	4145	821	GO:0031323	regulation of cellular metabolic process
3.10e-08	554	4145	155	GO:0032268	regulation of cellular protein metabolic process
9.72e-16	918	4145	268	GO:0045184	establishment of protein localization
3.39e-06	295	4145	87	GO:0043086	negative regulation of catalytic activity
3.53e-10	72	4145	37	GO:0051352	negative regulation of ligase activity
5.74e-21	648	4145	219	GO:0033554	cellular response to stress
1.92e-16	430	4145	151	GO:0006974	response to DNA damage stimulus
3.01e-14	333	4145	120	GO:0006281	DNA repair
3.21e-06	64	4145	28	GO:0006302	double-strand break repair
1.26e-11	1048	4145	281	GO:0051649	establishment of localization in cell
8.40e-17	851	4145	256	GO:0046907	intracellular transport
8.62e-09	297	4145	96	GO:0016568	chromatin modification
8.12e-09	147	4145	57	GO:0016569	covalent chromatin modification
7.21e-09	143	4145	56	GO:0016570	histone modification
6.19e-09	78	4145	37	GO:0051351	positive regulation of ligase activity
6.50e-16	911	4145	267	GO:0015031	protein transport
4.54e-10	545	4145	160	GO:0006886	intracellular protein transport
1.25e-06	125	4145	46	GO:0048193	Golgi vesicle transport
1.23e-08	113	4145	47	GO:0031396	regulation of protein ubiquitination
5.23e-09	84	4145	39	GO:0051438	regulation of ubiquitin-protein ligase activity
1.57e-09	75	4145	37	GO:0051439	regulation of ubiquitin-protein ligase activity during mitotic cell cycle
1.27e-10	205	4145	77	GO:0032269	negative regulation of cellular protein metabolic process
4.76e-09	131	4145	53	GO:0031400	negative regulation of protein modification process
8.89e-10	80	4145	39	GO:0031397	negative regulation of protein ubiquitination
3.53e-10	72	4145	37	GO:0051444	negative regulation of ubiquitin-protein ligase activity
3.51e-10	69	4145	36	GO:0051436	negative regulation of ubiquitin-protein ligase activity during mitotic cell cycle

3.86e-09	93	4145	42	GO:0031398	positive regulation of protein ubiquitination
6.65e-09	75	4145	36	GO:0051443	positive regulation of ubiquitin-protein ligase activity
1.62e-09	72	4145	36	GO:0051437	positive regulation of ubiquitin-protein ligase activity during mitotic cell cycle
3.66e-08	346	4145	106	GO:0051726	regulation of cell cycle
1.69e-22	15960	4145	3157	GO:0005623	cell
1.55e-22	15959	4145	3157	GO:0044464	cell part
6.15e-15	672	4145	207	GO:0031975	envelope
3.37e-115	11961	4145	2800	GO:0005622	intracellular
1.39e-08	805	4145	213	GO:0012505	endomembrane system
4.29e-125	11446	4145	2739	GO:0044424	intracellular part
5.83e-76	8015	4145	1984	GO:0005737	cytoplasm
1.26e-62	5407	4145	1421	GO:0044444	cytoplasmic part
3.60e-22	1378	4145	398	GO:0005829	cytosol
3.43e-24	3684	4145	906	GO:0032991	macromolecular complex
2.28e-13	2956	4145	695	GO:0043234	protein complex
1.28e-06	75	4145	32	GO:0000502	proteasome complex
3.01e-20	640	4145	215	GO:0030529	ribonucleoprotein complex
3.39e-59	1811	4145	611	GO:0031974	membrane-enclosed lumen
1.15e-111	9721	4145	2407	GO:0043226	organelle
6.89e-14	2635	4145	632	GO:0043228	non-membrane-bounded organelle
9.07e-127	8624	4145	2246	GO:0043227	membrane-bounded organelle
3.03e-111	9705	4145	2403	GO:0043229	intracellular organelle
6.89e-14	2635	4145	632	GO:0043232	intracellular non-membrane-bounded organelle
6.43e-07	548	4145	148	GO:0005694	chromosome
3.04e-07	309	4145	94	GO:0005840	ribosome
6.34e-08	55	4145	28	GO:0000313	organellar ribosome
2.99e-126	8615	4145	2243	GO:0043231	intracellular membrane-bounded organelle
5.96e-54	5365	4145	1381	GO:0005634	nucleus
5.47e-07	1028	4145	253	GO:0005783	endoplasmic reticulum
1.29e-09	923	4145	244	GO:0005794	Golgi apparatus
1.60e-41	1371	4145	456	GO:0005739	mitochondrion
1.55e-74	4424	4145	1253	GO:0044422	organelle part
4.09e-56	1774	4145	594	GO:0043233	organelle lumen
7.06e-75	4396	4145	1248	GO:0044446	intracellular organelle part
3.81e-58	1733	4145	589	GO:0070013	intracellular organelle lumen
3.81e-16	652	4145	206	GO:0031967	organelle envelope
4.94e-07	159	4145	56	GO:0033279	ribosomal subunit
9.59e-53	1810	4145	594	GO:0044428	nuclear part
9.21e-12	147	4145	63	GO:0005681	spliceosomal complex
2.08e-43	1379	4145	463	GO:0031981	nuclear lumen
1.07e-28	989	4145	326	GO:0005654	nucleoplasm
3.27e-18	627	4145	206	GO:0044451	nucleoplasm part
6.62e-08	208	4145	71	GO:0016604	nuclear body
1.58e-16	460	4145	159	GO:0005730	nucleolus

2.66e-27	621	4145	228	GO:0044429	mitochondrial part
5.27e-18	239	4145	102	GO:0031980	mitochondrial lumen
3.16e-16	436	4145	152	GO:0005740	mitochondrial envelope
5.27e-18	239	4145	102	GO:0005759	mitochondrial matrix
6.34e-08	55	4145	28	GO:0005761	mitochondrial ribosome
8.15e-10	301	4145	100	GO:0044431	Golgi apparatus part
5.98e-24	1134	4145	347	GO:0031090	organelle membrane
1.17e-17	344	4145	131	GO:0019866	organelle inner membrane
1.20e-15	411	4145	144	GO:0031966	mitochondrial membrane
5.60e-09	128	4145	52	GO:0044455	mitochondrial membrane part
1.39e-16	320	4145	122	GO:0005743	mitochondrial inner membrane
2.14e-06	150	4145	52	GO:0044454	nuclear chromosome part
2.64e-18	16728	4145	3261	GO:0003674	molecular_function
1.00e-30	13609	4145	2806	GO:0005488	binding
2.06e-31	8891	4145	1967	GO:0005515	protein binding
9.07e-11	526	4145	158	GO:0008134	transcription factor binding
1.03e-07	365	4145	109	GO:0003712	transcription cofactor activity
1.22e-14	1824	4145	465	GO:0001882	nucleoside binding
1.80e-14	1814	4145	462	GO:0001883	purine nucleoside binding
3.02e-15	3677	4145	854	GO:0003676	nucleic acid binding
9.64e-20	799	4145	253	GO:0003723	RNA binding
2.85e-06	95	4145	37	GO:0008135	translation factor activity, nucleic acid binding
1.88e-19	2525	4145	639	GO:0000166	nucleotide binding
2.04e-13	2070	4145	511	GO:0032553	ribonucleotide binding
1.31e-15	2156	4145	541	GO:0017076	purine nucleotide binding
1.87e-13	2069	4145	511	GO:0032555	purine ribonucleotide binding
2.99e-14	1782	4145	454	GO:0030554	adenyl nucleotide binding
3.82e-12	1698	4145	425	GO:0032559	adenyl ribonucleotide binding
3.30e-12	1673	4145	420	GO:0005524	ATP binding
1.67e-07	293	4145	91	GO:0048037	cofactor binding
1.91e-08	214	4145	74	GO:0050662	coenzyme binding
2.87e-39	5860	4145	1421	GO:0003824	catalytic activity
3.92e-09	483	4145	142	GO:0016874	ligase activity
1.86e-07	265	4145	84	GO:0016879	ligase activity, forming carbon-nitrogen bonds
3.81e-06	233	4145	72	GO:0016881	acid-amino acid ligase activity
1.31e-13	1830	4145	461	GO:0016740	transferase activity
5.45e-08	211	4145	72	GO:0016741	transferase activity, transferring one-carbon groups
1.51e-07	204	4145	69	GO:0008168	methyltransferase activity
3.89e-06	794	4145	198	GO:0016491	oxidoreductase activity
1.13e-12	2705	4145	639	GO:0016787	hydrolase activity
3.36e-09	973	4145	253	GO:0016817	hydrolase activity, acting on acid anhydrides
1.20e-09	954	4145	251	GO:0016818	hydrolase activity, acting on acid anhydrides, in phosphorus-containing anhydrides
2.27e-09	951	4145	249	GO:0016462	pyrophosphatase activity
1.78e-08	921	4145	238	GO:0017111	nucleoside-triphosphatase activity

1.11e-09	169	4145	65	GO:0004386	helicase activity
1.18e-07	131	4145	50	GO:0070035	purine NTP-dependent helicase activity
2.00e-08	426	4145	126	GO:0016887	ATPase activity
1.93e-06	338	4145	98	GO:0042623	ATPase activity, coupled
1.18e-07	131	4145	50	GO:0008026	ATP-dependent helicase activity
1.39e-07	474	4145	134	GO:0000287	magnesium ion binding
1.37e-06	435	4145	121	GO:0016563	transcription activator activity
3.57e-06	3055	4145	659	GO:0046914	transition metal ion binding
2.37e-07	175	4145	61	GO:0004518	nuclease activity
7.81e-07	105	4145	41	GO:0004519	endonuclease activity
4.07e-07	162	4145	57	GO:0043566	structure-specific DNA binding
2.25e-08	56	4145	29	GO:0003697	single-stranded DNA binding
2.08e-07	26	4145	17	GO:0051539	4 iron, 4 sulfur cluster binding
5.09e-07	308	4145	108	REAC:69278	Cell Cycle, Mitotic
6.02e-07	111	4145	49	REAC:69206	G1/S Transition
1.91e-06	87	4145	40	REAC:69002	DNA Replication Pre-Initiation
7.84e-07	118	4145	51	REAC:72203	Processing of Capped Intron-Containing Pre-mRNA
1.48e-07	113	4145	51	REAC:72172	mRNA Splicing
1.48e-07	113	4145	51	REAC:72163	mRNA Splicing - Major Pathway
8.60e-07	109	4145	48	REAC:72143	Lariat Formation and 5'-Splice Site Cleavage
2.13e-07	111	4145	50	REAC:72160	Cleavage at the 3'-Splice Site and Exon Ligation
4.30e-07	110	4145	49	REAC:72130	Formation of an intermediate Spliceosomal C complex
2.13e-07	111	4145	50	REAC:156661	Formation of Exon Junction Complex
6.16e-07	108	4145	48	REAC:72139	Formation of the active Spliceosomal C complex
1.75e-10	420	4145	151	REAC:74160	Gene Expression
1.66e-06	169	4145	66	REAC:75983	Formation and Maturation of mRNA Transcript
2.55e-08	212	4145	84	REAC:392499	Metabolism of proteins
3.91e-06	133	4145	54	REAC:72766	Translation
9.80e-08	124	4145	55	REAC:69620	Cell Cycle Checkpoints
2.85e-07	143	4145	60	REAC:74159	Transcription
1.08e-08	1132	4145	311	KEGG:01100	Metabolic pathways
2.97e-06	132	4145	51	KEGG:04142	Lysosome
5.25e-09	177	4145	71	KEGG:05016	Huntington's disease
3.74e-06	126	4145	49	KEGG:05012	Parkinson's disease

Significance between the target list and indicated functional category is presented as p-value calculated by Fisher exact test

I - nr of genes in functional group

II - nr of genes with indicated modification

III - nr of the overlapping genes

DC H3K27me3

Significance	I	II	III	ID	Functional group
1.04e-06	693	1284	72	GO:0006952	defense response
2.40e-06	42	1284	13	REAC:380108	Chemokine receptors bind chemokines

Significance between the target list and indicated functional category is presented as p-value calculated by Fisher exact test

I - nr of genes in functional group

II - nr of genes with indicated modification change

III - nr of the overlapping genes

MF AcH3

Significance	I	II	III	ID	Functional group
2.57e-06	623	3907	154	GO:0051246	regulation of protein metabolic process
5.89e-14	15757	3907	2904	GO:0008150	biological_process
1.34e-06	3294	3907	672	GO:0051179	localization
1.42e-13	1275	3907	324	GO:0033036	macromolecule localization
3.30e-13	1060	3907	277	GO:0008104	protein localization
1.49e-68	6921	3907	1660	GO:0008152	metabolic process
7.48e-12	1523	3907	367	GO:0044281	small molecule metabolic process
1.23e-54	4700	3907	1186	GO:0043170	macromolecule metabolic process
1.60e-13	1678	3907	407	GO:0043412	macromolecule modification
1.49e-35	1512	3907	453	GO:0010467	gene expression
3.08e-60	6042	3907	1466	GO:0044238	primary metabolic process
6.25e-23	3189	3907	757	GO:0019538	protein metabolic process
4.40e-07	1193	3907	274	GO:0006508	proteolysis
1.95e-15	1384	3907	355	GO:0009056	catabolic process
7.64e-16	858	3907	243	GO:0009057	macromolecule catabolic process
2.14e-12	679	3907	191	GO:0030163	protein catabolic process
7.55e-44	2404	3907	676	GO:0006807	nitrogen compound metabolic process
1.43e-29	2052	3907	552	GO:0009058	biosynthetic process
9.24e-26	1260	3907	366	GO:0009059	macromolecule biosynthetic process
1.45e-07	714	3907	179	GO:0055114	oxidation reduction
1.25e-13	2725	3907	616	GO:0016043	cellular component organization
1.38e-06	830	3907	198	GO:0043933	macromolecular complex subunit organization
5.01e-52	10377	3907	2210	GO:0009987	cellular process
3.58e-14	808	3907	226	GO:0007049	cell cycle
3.28e-13	383	3907	125	GO:0000278	mitotic cell cycle
6.23e-12	1143	3907	289	GO:0051641	cellular localization
4.77e-09	595	3907	160	GO:0070727	cellular macromolecule localization
5.84e-09	592	3907	159	GO:0034613	cellular protein localization
1.67e-13	596	3907	176	GO:0022402	cell cycle process
4.59e-11	430	3907	130	GO:0022403	cell cycle phase
3.56e-11	344	3907	110	GO:0000279	M phase
1.16e-07	231	3907	73	GO:0000087	M phase of mitotic cell cycle
4.21e-17	1455	3907	377	GO:0006996	organelle organization
4.95e-08	235	3907	75	GO:0048285	organelle fission
1.83e-07	225	3907	71	GO:0000280	nuclear division
1.83e-07	225	3907	71	GO:0007067	mitosis
1.59e-06	137	3907	47	GO:0007005	mitochondrion organization
2.12e-09	931	3907	233	GO:0051716	cellular response to stimulus
2.26e-78	5724	3907	1465	GO:0044237	cellular metabolic process
1.22e-64	4126	3907	1104	GO:0044260	cellular macromolecule metabolic process
5.67e-29	2670	3907	679	GO:0044267	cellular protein metabolic process
8.59e-13	1588	3907	385	GO:0006464	protein modification process

6.34e-09	213	3907	72	GO:0070647	protein modification by small protein conjugation or removal
1.25e-07	182	3907	61	GO:0032446	protein modification by small protein conjugation
2.33e-09	1307	3907	310	GO:0043687	post-translational protein modification
1.00e-07	169	3907	58	GO:0016567	protein ubiquitination
3.88e-46	2284	3907	657	GO:0034641	cellular nitrogen compound metabolic process
7.06e-49	1987	3907	599	GO:0006139	nucleobase, nucleoside, nucleotide and nucleic acid metabolic process
2.28e-42	1606	3907	494	GO:0090304	nucleic acid metabolic process
3.55e-13	557	3907	166	GO:0006259	DNA metabolic process
4.73e-07	109	3907	41	GO:0006310	DNA recombination
1.55e-31	1056	3907	336	GO:0016070	RNA metabolic process
7.85e-13	294	3907	102	GO:0034660	ncRNA metabolic process
1.77e-12	106	3907	50	GO:0016072	rRNA metabolic process
7.67e-16	400	3907	136	GO:0016071	mRNA metabolic process
1.01e-24	609	3907	210	GO:0006396	RNA processing
4.47e-12	204	3907	77	GO:0034470	ncRNA processing
1.99e-17	304	3907	115	GO:0008380	RNA splicing
3.92e-09	112	3907	46	GO:0000375	RNA splicing, via transesterification reactions
2.36e-08	103	3907	42	GO:0000377	RNA splicing, via transesterification reactions with bulged adenosine as nucleophile
1.20e-14	332	3907	116	GO:0006397	mRNA processing
2.36e-08	103	3907	42	GO:0000398	nuclear mRNA splicing, via spliceosome
2.33e-08	359	3907	105	GO:0055086	nucleobase, nucleoside and nucleotide metabolic process
1.66e-07	332	3907	96	GO:0006753	nucleoside phosphate metabolic process
1.66e-07	332	3907	96	GO:0009117	nucleotide metabolic process
3.72e-17	1141	3907	310	GO:0044248	cellular catabolic process
5.20e-17	805	3907	235	GO:0044265	cellular macromolecule catabolic process
8.78e-13	624	3907	180	GO:0043632	modification-dependent macromolecule catabolic process
1.98e-12	665	3907	188	GO:0044257	cellular protein catabolic process
2.45e-12	662	3907	187	GO:0051603	proteolysis involved in cellular protein catabolic process
3.20e-10	112	3907	48	GO:0010498	proteasomal protein catabolic process
8.78e-13	624	3907	180	GO:0019941	modification-dependent protein catabolic process
1.85e-12	266	3907	94	GO:0006511	ubiquitin-dependent protein catabolic process
3.20e-10	112	3907	48	GO:0043161	proteasomal ubiquitin-dependent protein catabolic process
1.04e-08	70	3907	33	GO:0031145	anaphase-promoting complex-dependent proteasomal ubiquitin-dependent protein catabolic process
2.53e-09	221	3907	75	GO:0051186	cofactor metabolic process
6.24e-08	167	3907	58	GO:0006732	coenzyme metabolic process
1.59e-11	340	3907	110	GO:0006091	generation of precursor metabolites and energy
5.39e-08	124	3907	47	GO:0022900	electron transport chain
1.17e-06	120	3907	43	GO:0006119	oxidative phosphorylation
2.29e-07	647	3907	164	GO:0042180	cellular ketone metabolic process
5.05e-29	1935	3907	525	GO:0044249	cellular biosynthetic process
1.83e-08	375	3907	109	GO:0044271	cellular nitrogen compound biosynthetic process
1.04e-06	291	3907	84	GO:0032774	RNA biosynthetic process
6.28e-07	110	3907	41	GO:0051188	cofactor biosynthetic process
4.99e-26	1234	3907	361	GO:0034645	cellular macromolecule biosynthetic process

1.74e-10	464	3907	136	GO:0006412	translation
1.57e-07	179	3907	60	GO:0006260	DNA replication
7.94e-07	365	3907	101	GO:0006350	transcription
1.10e-06	287	3907	83	GO:0006351	transcription, DNA-dependent
1.08e-06	643	3907	160	GO:0006082	organic acid metabolic process
7.79e-07	635	3907	159	GO:0043436	oxoacid metabolic process
7.79e-07	635	3907	159	GO:0019752	carboxylic acid metabolic process
6.34e-11	1151	3907	286	GO:0044085	cellular component biogenesis
2.86e-20	203	3907	92	GO:0022613	ribonucleoprotein complex biogenesis
1.60e-08	81	3907	36	GO:0022618	ribonucleoprotein complex assembly
1.14e-13	136	3907	61	GO:0042254	ribosome biogenesis
5.45e-12	102	3907	48	GO:0006364	rRNA processing
2.29e-07	2936	3907	612	GO:0051234	establishment of localization
2.36e-07	2910	3907	607	GO:0006810	transport
7.40e-14	918	3907	249	GO:0045184	establishment of protein localization
2.92e-14	911	3907	249	GO:0015031	protein transport
3.00e-11	1048	3907	266	GO:0051649	establishment of localization in cell
1.98e-15	851	3907	240	GO:0046907	intracellular transport
7.17e-08	78	3907	34	GO:0006839	mitochondrial transport
2.69e-09	545	3907	150	GO:0006886	intracellular protein transport
4.26e-08	87	3907	37	GO:0051340	regulation of ligase activity
2.49e-08	72	3907	33	GO:0051352	negative regulation of ligase activity
5.91e-13	648	3907	186	GO:0033554	cellular response to stress
2.17e-11	430	3907	131	GO:0006974	response to DNA damage stimulus
8.76e-12	333	3907	109	GO:0006281	DNA repair
1.87e-08	78	3907	35	GO:0051351	positive regulation of ligase activity
4.02e-06	125	3907	43	GO:0048193	Golgi vesicle transport
3.86e-06	113	3907	40	GO:0031396	regulation of protein ubiquitination
5.09e-08	84	3907	36	GO:0051438	regulation of ubiquitin-protein ligase activity
5.26e-09	75	3907	35	GO:0051439	regulation of ubiquitin-protein ligase activity during mitotic cell cycle
1.01e-07	93	3907	38	GO:0031398	positive regulation of protein ubiquitination
2.16e-08	75	3907	34	GO:0051443	positive regulation of ubiquitin-protein ligase activity
5.93e-09	72	3907	34	GO:0051437	positive regulation of ubiquitin-protein ligase activity during mitotic cell cycle
5.31e-07	80	3907	33	GO:0031397	negative regulation of protein ubiquitination
2.49e-08	72	3907	33	GO:0051444	negative regulation of ubiquitin-protein ligase activity
6.62e-09	69	3907	33	GO:0051436	negative regulation of ubiquitin-protein ligase activity during mitotic cell cycle
6.28e-07	110	3907	41	GO:0045333	cellular respiration
6.39e-07	341	3907	96	GO:0007264	small GTPase mediated signal transduction
7.33e-17	15960	3907	2951	GO:0005623	cell
6.83e-17	15959	3907	2951	GO:0044464	cell part
1.57e-16	672	3907	203	GO:0031975	envelope
1.28e-86	11961	3907	2581	GO:0005622	intracellular
1.41e-07	805	3907	198	GO:0012505	endomembrane system
5.92e-94	11446	3907	2520	GO:0044424	intracellular part

7.57e-62	8015	3907	1836	GO:0005737	cytoplasm
5.68e-56	5407	3907	1329	GO:0044444	cytoplasmic part
2.90e-15	1378	3907	353	GO:0005829	cytosol
2.53e-21	3684	3907	846	GO:0032991	macromolecular complex
1.79e-11	2956	3907	647	GO:0043234	protein complex
2.61e-21	640	3907	209	GO:0030529	ribonucleoprotein complex
1.52e-54	1811	3907	574	GO:0031974	membrane-enclosed lumen
4.34e-82	9721	3907	2202	GO:0043226	organelle
6.76e-13	2635	3907	594	GO:0043228	non-membrane-bounded organelle
5.73e-94	8624	3907	2048	GO:0043227	membrane-bounded organelle
2.54e-82	9705	3907	2200	GO:0043229	intracellular organelle
6.76e-13	2635	3907	594	GO:0043232	intracellular non-membrane-bounded organelle
2.99e-06	548	3907	138	GO:0005694	chromosome
3.95e-06	309	3907	86	GO:0005840	ribosome
7.98e-94	8615	3907	2046	GO:0043231	intracellular membrane-bounded organelle
2.26e-40	5365	3907	1260	GO:0005634	nucleus
6.34e-07	1028	3907	240	GO:0005783	endoplasmic reticulum
2.27e-40	1371	3907	434	GO:0005739	mitochondrion
2.86e-68	4424	3907	1176	GO:0044422	organelle part
6.06e-51	1774	3907	556	GO:0043233	organelle lumen
3.64e-68	4396	3907	1170	GO:0044446	intracellular organelle part
1.14e-52	1733	3907	551	GO:0070013	intracellular organelle lumen
9.30e-18	652	3907	202	GO:0031967	organelle envelope
7.93e-51	1810	3907	564	GO:0044428	nuclear part
2.06e-12	147	3907	62	GO:0005681	spliceosomal complex
1.52e-41	1379	3907	439	GO:0031981	nuclear lumen
1.00e-25	989	3907	304	GO:0005654	nucleoplasm
3.54e-13	627	3907	182	GO:0044451	nucleoplasm part
1.90e-06	208	3907	64	GO:0016604	nuclear body
1.80e-17	460	3907	155	GO:0005730	nucleolus
3.90e-25	621	3907	214	GO:0044429	mitochondrial part
1.93e-12	239	3907	87	GO:0031980	mitochondrial lumen
1.86e-17	436	3907	149	GO:0005740	mitochondrial envelope
1.93e-12	239	3907	87	GO:0005759	mitochondrial matrix
1.24e-06	301	3907	86	GO:0044431	Golgi apparatus part
7.58e-22	1134	3907	325	GO:0031090	organelle membrane
1.21e-17	344	3907	126	GO:0019866	organelle inner membrane
2.40e-16	411	3907	140	GO:0031966	mitochondrial membrane
2.66e-13	128	3907	58	GO:0044455	mitochondrial membrane part
3.00e-18	320	3907	121	GO:0005743	mitochondrial inner membrane
1.55e-06	76	3907	31	GO:0070469	respiratory chain
1.34e-06	65	3907	28	GO:0005746	mitochondrial respiratory chain
1.62e-06	36	3907	19	GO:0005758	mitochondrial intermembrane space
3.13e-06	198	3907	61	GO:0019787	small conjugating protein ligase activity

2.11e-06	474	3907	123	GO:0000287	magnesium ion binding
9.91e-07	135	3907	47	GO:0016779	nucleotidyltransferase activity
7.59e-15	16728	3907	3058	GO:0003674	molecular_function
2.55e-21	13609	3907	2603	GO:0005488	binding
8.72e-29	8891	3907	1848	GO:0005515	protein binding
7.68e-07	526	3907	136	GO:0008134	transcription factor binding
6.75e-12	1824	3907	428	GO:0001882	nucleoside binding
4.42e-12	1814	3907	427	GO:0001883	purine nucleoside binding
2.58e-07	3677	3907	750	GO:0003676	nucleic acid binding
9.71e-15	799	3907	226	GO:0003723	RNA binding
6.22e-07	95	3907	37	GO:0008135	translation factor activity, nucleic acid binding
2.97e-15	2525	3907	586	GO:0000166	nucleotide binding
4.53e-12	2070	3907	478	GO:0032553	ribonucleotide binding
6.14e-13	2156	3907	500	GO:0017076	purine nucleotide binding
4.18e-12	2069	3907	478	GO:0032555	purine ribonucleotide binding
1.98e-11	1782	3907	417	GO:0030554	adenyl nucleotide binding
1.11e-10	1698	3907	396	GO:0032559	adenyl ribonucleotide binding
7.34e-11	1673	3907	392	GO:0005524	ATP binding
1.11e-30	5860	3907	1309	GO:0003824	catalytic activity
1.12e-08	483	3907	134	GO:0016874	ligase activity
5.57e-08	265	3907	82	GO:0016879	ligase activity, forming carbon-nitrogen bonds
7.75e-09	1830	3907	411	GO:0016740	transferase activity
1.82e-06	794	3907	190	GO:0016491	oxidoreductase activity
1.10e-11	2705	3907	600	GO:0016787	hydrolase activity
2.96e-06	973	3907	225	GO:0016817	hydrolase activity, acting on acid anhydrides
2.16e-06	954	3907	222	GO:0016818	hydrolase activity, acting on acid anhydrides, in phosphorus-containing anhydrides
2.52e-06	951	3907	221	GO:0016462	pyrophosphatase activity
4.51e-08	151	3907	60	KEGG:03040	Spliceosome
6.42e-08	1132	3907	300	KEGG:01100	Metabolic pathways
2.82e-06	135	3907	51	KEGG:00190	Oxidative phosphorylation
6.37e-07	143	3907	57	REAC:74159	Transcription
1.08e-08	308	3907	109	REAC:69278	Cell Cycle, Mitotic
3.05e-06	112	3907	46	REAC:69242	Phase
1.85e-06	147	3907	57	REAC:265764	Glucose Regulation of Insulin Secretion
1.10e-07	124	3907	53	REAC:69620	Cell Cycle Checkpoints
9.81e-07	420	3907	132	REAC:74160	Gene Expression

Significance between the target list and indicated functional category is presented as p-value calculated by Fisher exact test

I - nr of genes in functional group

II - nr of genes with indicated modification change

III - nr of the overlapping genes

MF H3K4me3

Significance	I	II	III	ID	Functional group
2.05e-06	4068	2247	478	GO:0023052	signaling
2.20e-08	4000	2247	486	GO:0050896	response to stimulus
2.80e-07	1445	2247	199	GO:0042221	response to chemical stimulus
5.36e-09	1022	2247	157	GO:0009605	response to external stimulus
1.77e-07	595	2247	98	GO:0009611	response to wounding
4.50e-08	4630	2247	550	GO:0032501	multicellular organismal process
3.08e-06	3294	2247	395	GO:0051179	localization
3.14e-06	2910	2247	354	GO:0006810	transport
9.68e-07	862	2247	128	GO:0006629	lipid metabolic process
4.82e-07	7719	2247	856	GO:0016020	membrane
1.15e-08	3953	2247	483	GO:0005886	plasma membrane
5.78e-07	747	2247	115	GO:0005615	extracellular space
3.46e-08	52	2247	20	GO:0042379	chemokine receptor binding
9.55e-08	50	2247	19	GO:0008009	chemokine activity
3.55e-06	56	2247	18	GO:0033293	monocarboxylic acid binding

Significance between the target list and indicated functional category is presented as p-value calculated by Fisher exact test

I - nr of genes in functional group

II - nr of genes with indicated modification change

III - nr of the overlapping genes

MF H3K27me3

Significance	I	II	III	ID	Functional group
5.28e-12	6921	1233	491	GO:0008152	metabolic process
3.39e-10	4700	1233	349	GO:0043170	macromolecule metabolic process
1.93e-07	1512	1233	131	GO:0010467	gene expression
2.44e-06	1260	1233	109	GO:0009059	macromolecule biosynthetic process
2.98e-10	6042	1233	430	GO:0044238	primary metabolic process
3.48e-06	3189	1233	233	GO:0019538	protein metabolic process
2.99e-06	2404	1233	184	GO:0006807	nitrogen compound metabolic process
1.14e-13	10377	1233	693	GO:0009987	cellular process
3.66e-13	5724	1233	426	GO:0044237	cellular metabolic process
1.04e-11	4126	1233	321	GO:0044260	cellular macromolecule metabolic process
8.43e-07	2670	1233	204	GO:0044267	cellular protein metabolic process
2.23e-06	2284	1233	177	GO:0034641	cellular nitrogen compound metabolic process
9.16e-07	1234	1233	109	GO:0034645	cellular macromolecule biosynthetic process
1.30e-07	15960	1233	947	GO:0005623	cell
1.27e-07	15959	1233	947	GO:0044464	cell part
9.97e-28	11961	1233	827	GO:0005622	intracellular
6.21e-30	11446	1233	808	GO:0044424	intracellular part
3.72e-23	8015	1233	602	GO:0005737	cytoplasm
8.73e-18	5407	1233	427	GO:0044444	cytoplasmic part
7.15e-10	1378	1233	131	GO:0005829	cytosol
2.14e-21	9721	1233	690	GO:0043226	organelle
5.41e-22	8624	1233	632	GO:0043227	membrane-bounded organelle
2.33e-21	9705	1233	689	GO:0043229	intracellular organelle
1.35e-21	8615	1233	630	GO:0043231	intracellular membrane-bounded organelle
6.75e-09	5365	1233	382	GO:0005634	nucleus
4.04e-08	1371	1233	124	GO:0005739	mitochondrion
3.61e-07	1811	1233	150	GO:0031974	membrane-enclosed lumen
1.38e-11	4424	1233	339	GO:0044422	organelle part
7.68e-07	1774	1233	146	GO:0043233	organelle lumen
1.56e-11	4396	1233	337	GO:0044446	intracellular organelle part
5.34e-07	1733	1233	144	GO:0070013	intracellular organelle lumen
2.29e-07	13609	1233	829	GO:0005488	binding
2.69e-09	8891	1233	588	GO:0005515	protein binding
9.06e-07	799	1233	78	GO:0003723	RNA binding

Significance between the target list and indicated functional category is presented as p-value calculated by Fisher exact test

I - nr of genes in functional group

II - nr of genes with indicated modification change

III - nr of the overlapping genes
