# Supplementary material for: Genome-wide promoter analysis of histone modifications in human monocyte-derived antigen presenting cells
Source: BMC Genomics. 2010 Nov 18;11:642. doi: 10.1186/1471-2164-11-642 (PMC3091769; doi:10.1186/1471-2164-11-642)
Supplement: Additional file 2 — Tserel et al BMC Genomics. Contains Supplementary Tables S2-S6. Size 7.2 MB [file 1471-2164-11-642-S2.ZIP › Supplementary Table 3. Average expression..pdf]

Supplementary Table 3. Expression signal log2 values in monocyte, macrophage and dendritic cell subpopulations.

	DC	MF	MO
15E1.2	6,81	7,04	5,87
76P	6,26	6,62	6,36
A2M	13,78	13,02	2,78
A4GALT	4,21	1,97	2,37
A4GNT	4,42	4,06	4,94
AAAS	6,56	6,04	6,20
AADACL1	10,90	11,81	8,79
AAMP	7,80	8,05	8,68
AARSD1	7,26	7,14	7,46
AASDH	7,73	7,96	8,09
AASDHPPT	7,17	7,13	6,76
AATF	9,07	8,95	9,67
ABCA1	10,00	12,03	9,48
ABCA3	6,23	5,14	4,95
ABCA5	5,07	4,32	1,78
ABCA6	6,37	5,74	4,27
ABCB6	6,04	5,11	4,32
ABCB7	8,98	8,81	9,27
ABCB8	4,99	5,30	3,48
ABCB9	3,33	3,92	2,44
ABCC11	4,20	4,41	4,27
ABCC13	3,06	0,48	1,28
ABCC3	10,89	10,88	8,35
ABCC4	7,70	7,02	6,20
ABCC5	8,21	7,51	7,69
ABCC6	3,10	2,62	3,93
ABCD1	8,12	8,47	7,54
ABCD3	7,13	7,40	7,32
ABCD4	4,00	3,39	4,36
ABCE1	9,39	9,68	9,09
ABCF1	10,57	10,55	10,24
ABCF2	6,05	5,73	6,71
ABCF3	7,04	6,41	6,40
ABCG1	8,94	10,43	5,45
ABCG2	4,13	-0,75	-3,32
ABCG4	2,78	3,54	-0,64
ABHD10	8,92	9,26	8,04
ABHD11	3,86	4,01	4,79
ABHD12	8,71	7,42	5,72
ABHD13	4,85	4,80	5,34
ABHD14A	7,48	7,45	7,54
ABHD14B	7,54	7,48	8,17
ABHD3	8,48	8,79	9,14
ABHD4	7,67	7,02	5,67
ABHD5	8,62	9,35	9,28
ABHD6	8,98	8,40	6,53
ABHD7	4,52	3,23	2,91
ABHD9	4,68	4,68	4,95
ABI1	9,28	8,92	9,14
ABI3	8,95	6,62	9,67
ABL1	7,63	6,93	7,44
ABL2	5,77	6,38	2,81
ABO	4,10	4,46	4,00
ABP1	5,85	6,69	4,36
ABR	10,82	10,85	9,68

ABTB2	4,27	3,44	-1,05
ACAA1	9,27	10,34	10,35
ACAA2	10,83	10,61	9,06
ACACA	7,73	7,88	6,33
ACAD10	7,05	6,62	7,49
ACAD11	8,04	8,19	8,50
ACAD8	7,01	7,42	7,49
ACAD9	8,64	9,06	8,43
ACADM	9,46	10,20	8,23
ACADS	6,70	7,12	6,52
ACADSB	3,91	3,99	4,56
ACADVL	11,45	11,52	11,96
ACAT2	11,76	12,42	8,38
ACBD3	10,37	10,77	10,41
ACBD4	3,03	2,56	4,39
ACBD5	6,06	5,78	6,11
ACBD6	7,00	7,05	7,35
ACCN2	-0,69	1,78	-3,32
ACCN4	7,23	7,33	7,53
ACD	6,78	6,05	7,05
ACE	6,31	5,32	3,70
ACE2	3,63	2,80	3,96
ACIN1	8,97	8,34	8,93
ACLY	10,72	10,67	10,46
ACN9	6,74	7,01	7,41
ACO1	11,41	12,90	9,52
ACO2	9,85	9,77	8,69
ACOT1	9,12	10,47	5,29
ACOT11	6,25	7,63	5,23
ACOT12	5,07	4,83	5,51
ACOT2	7,65	8,82	3,18
ACOT7	10,56	9,73	4,21
ACOT8	7,04	7,02	7,11
ACOT9	9,96	9,87	9,25
ACOX1	7,75	8,33	6,65
ACOX2	8,14	9,76	6,56
ACOX3	8,53	7,83	7,73
ACP1	9,29	9,69	9,30
ACP2	9,02	9,83	6,93
ACP5	11,83	11,95	6,07
ACP6	4,50	5,20	4,33
ACPL2	7,25	4,98	7,48
ACPP	6,06	6,54	8,84
ACRBP	4,36	4,47	6,59
ACRC	6,05	5,79	6,34
ACSL1	11,18	11,68	11,33
ACSL3	8,82	8,43	5,73
ACSL4	10,03	10,46	10,17
ACSL5	8,45	7,79	7,80
ACSM3	2,18	4,15	4,68
ACSS1	6,34	7,35	8,29
ACSS2	10,53	10,64	9,57
ACTB	14,47	14,41	14,40
ACTG1	13,12	12,95	12,68
ACTL6B	3,09	3,28	3,33
ACTN1	13,00	13,09	11,30
ACTR10	9,55	9,99	9,25
ACTR1A	10,01	10,03	9,49
ACTR2	12,77	12,75	13,20

ACTR3	12,26	12,30	12,06
ACTR5	6,76	6,62	7,63
ACTR6	9,19	9,64	8,89
ACTR8	6,95	6,60	5,31
ACVR1	10,43	10,17	9,89
ACVR1B	5,65	6,85	6,84
ACVR2A	5,08	4,57	5,80
ACVR2B	4,02	3,19	3,22
ACVRL1	5,36	6,80	4,92
ACY1	8,15	8,61	7,22
ACY1L2	4,81	5,05	6,18
ACY3	4,85	5,07	4,69
ACYP1	8,22	7,91	6,45
ACYP2	4,49	4,96	4,87
ADA	6,12	6,92	8,19
ADAL	4,60	5,27	5,26
ADAM10	8,70	9,31	9,07
ADAM11	4,01	2,95	2,45
ADAM12	5,42	3,57	1,83
ADAM15	11,39	10,83	10,55
ADAM17	6,23	6,15	6,35
ADAM23	5,98	5,87	6,02
ADAM28	-3,32	5,71	5,42
ADAM30	4,58	3,67	4,51
ADAM9	8,31	8,57	7,49
ADAMDEC1	2,84	9,04	4,60
ADAMTS1	3,44	4,67	4,28
ADAMTS17	5,07	4,42	4,76
ADAMTS5	-0,54	-3,32	5,79
ADAMTS8	3,39	3,88	4,10
ADAR	10,55	10,13	11,04
ADARB1	7,28	7,93	6,18
ADAT1	7,30	7,35	7,07
ADC	4,22	4,41	3,05
ADCK4	6,38	6,32	6,84
ADCY1	4,77	-0,19	0,02
ADCY3	11,86	12,09	7,68
ADCY4	5,83	6,58	6,97
ADCY9	6,94	6,35	6,80
ADD1	11,54	11,18	11,86
ADH5	5,21	6,46	5,51
ADHFE1	8,56	7,14	8,90
ADI1	10,93	10,45	11,10
ADIPOR1	10,43	10,69	10,74
ADIPOR2	10,85	10,98	11,14
ADK	7,68	7,67	7,15
ADM	9,56	9,98	10,63
ADM2	5,88	4,92	5,24
ADNP	8,05	7,53	8,03
ADORA2B	7,65	8,73	6,68
ADORA3	8,26	5,73	5,99
ADPGK	10,61	10,31	10,54
ADPRH	7,56	7,79	7,60
ADRA1D	6,05	5,75	6,24
ADRA2A	5,39	5,41	4,17
ADRA2B	4,76	6,21	4,30
ADRA2C	3,18	3,46	3,36
ADRB1	3,62	3,89	3,33
ADRB2	5,41	6,81	6,62

ADRBK2	3,78	3,15	2,31
ADSL	10,34	10,33	10,52
AFF1	5,36	5,04	5,12
AFG3L1	7,59	7,26	8,16
AFG3L2	8,36	8,26	7,41
AFMID	6,05	6,30	7,36
AGA	8,65	8,91	8,43
AGBL3	4,09	2,88	3,61
AGGF1	6,94	6,75	6,19
AGL	5,04	5,13	4,88
AGPAT1	5,45	4,98	5,09
AGPAT3	7,98	7,96	8,40
AGPAT4	8,25	7,85	7,86
AGPAT5	9,11	9,48	9,35
AGPAT6	5,54	5,51	5,61
AGPAT7	7,35	5,85	5,75
AGPS	9,40	10,57	8,45
AGRP	3,83	8,46	4,71
AGTPBP1	9,55	9,51	11,47
AGTR2	3,61	3,26	2,92
AHI1	6,22	7,29	4,33
AHNAK	11,87	11,50	12,32
AHR	12,38	11,95	13,03
AHSA1	9,59	9,52	9,61
AHSA2	9,77	9,02	10,06
AHSG	3,37	4,22	4,36
AIF1	9,78	11,15	12,92
AIG1	6,55	4,43	4,26
AIM1	8,43	8,50	7,96
AIM2	5,88	6,61	8,19
AIP	10,09	9,66	10,27
AIPL1	3,77	3,46	4,30
AK1	5,82	8,82	3,54
AK2	9,59	8,87	9,10
AK3L1	8,98	9,07	5,55
AK7	4,79	4,58	4,39
AKAP1	5,23	4,58	4,99
AKAP10	6,76	6,40	7,20
AKAP11	7,69	6,85	7,17
AKAP12	6,26	2,31	1,10
AKAP13	7,39	7,49	8,93
AKAP14	-0,46	1,46	2,75
AKAP5	4,33	3,31	1,94
AKAP7	6,34	6,53	7,73
AKAP8	7,77	7,44	9,15
AKAP8L	6,97	6,01	8,29
AKAP9	5,32	5,53	6,00
AKR1B1	11,43	12,12	9,77
AKR1C3	6,84	10,54	4,96
AKR1CL2	4,57	4,68	3,89
AKR7A2	11,31	12,10	10,75
AKR7A3	2,69	4,55	1,96
AKT1	7,50	6,94	7,80
AKT1S1	7,31	7,27	7,05
AKT2	4,48	4,25	4,77
AKT3	3,05	3,33	3,02
ALCAM	11,84	11,65	9,51
ALDH16A1	9,35	9,38	9,52
ALDH1A1	10,85	11,90	11,16

ALDH1A2	11,11	11,62	3,47
ALDH1A3	4,21	-0,57	3,14
ALDH1B1	4,61	3,88	4,68
ALDH1L2	7,14	5,03	-0,45
ALDH3B1	8,17	8,75	9,08
ALDH4A1	7,14	7,84	5,95
ALDH5A1	7,79	7,91	-3,32
ALDH6A1	8,64	8,61	8,05
ALDH9A1	12,44	12,45	11,66
ALDOA	11,59	11,90	11,16
ALDOB	5,20	5,50	4,91
ALDOC	8,25	8,18	7,30
ALG1	6,62	6,74	5,62
ALG11	6,56	6,78	6,30
ALG14	7,43	8,19	6,15
ALG2	5,37	4,68	4,83
ALG3	7,94	8,74	8,07
ALG5	9,95	9,91	9,64
ALG8	9,68	10,35	8,75
ALG9	7,88	8,96	7,69
ALK	4,64	8,07	1,10
ALKBH1	7,02	7,08	6,60
ALKBH2	5,35	5,51	6,25
ALKBH3	10,17	10,31	9,40
ALKBH4	5,69	5,30	6,02
ALKBH5	12,31	12,09	12,38
ALKBH6	7,30	8,11	7,15
ALKBH7	10,79	10,71	10,81
ALKBH8	5,78	5,92	5,72
ALMS1	5,82	5,45	6,31
ALOX12B	4,65	4,19	4,52
ALOX15	10,87	-3,32	1,50
ALOX5	6,65	11,24	11,49
ALOX5AP	13,33	14,36	12,48
ALPI	2,87	4,26	3,69
ALPK2	4,31	3,20	3,29
ALPL	5,95	5,39	4,66
ALS2	8,77	8,81	7,86
ALS2CR13	9,03	7,92	11,51
ALS2CR14	6,15	6,15	8,01
ALS2CR2	9,12	8,86	8,32
ALS2CR4	5,42	7,08	5,02
ALS2CR8	5,03	4,44	3,87
ALX3	4,60	4,49	4,41
AMACR	6,34	5,78	4,33
AMD1	10,75	10,91	10,30
AMDHD1	6,27	8,76	6,29
AMDHD2	7,12	6,25	4,26
AMICA1	9,84	12,09	12,93
AMIGO1	4,39	5,07	4,58
AMIGO2	4,85	4,92	5,12
AMMECR1	6,29	6,09	6,52
AMOTL1	3,56	4,13	2,94
AMPD2	-0,11	2,47	2,43
AMPD3	4,81	5,91	4,51
ANAPC1	6,97	7,21	7,85
ANAPC10	8,61	8,99	8,10
ANAPC11	7,94	8,10	7,59
ANAPC13	10,82	10,42	11,22

ANAPC2	5,97	5,66	6,71
ANAPC4	8,88	9,13	9,58
ANAPC5	10,22	10,31	10,99
ANAPC7	6,70	7,46	5,89
ANG	5,69	7,79	9,09
ANGEL2	7,73	7,62	8,88
ANGPT1	-1,16	-0,41	5,80
ANGPTL4	3,79	2,97	3,05
ANGPTL6	6,55	6,44	5,37
ANK1	3,95	3,94	4,42
ANKH	4,06	3,51	2,89
ANKHD1	7,62	7,31	7,98
ANKMY1	5,53	5,13	6,48
ANKMY2	9,24	9,18	8,10
ANKRA2	7,71	7,92	7,53
ANKRD10	8,92	8,29	8,59
ANKRD12	9,22	8,34	8,97
ANKRD13C	6,64	6,78	6,11
ANKRD13D	4,49	3,90	6,75
ANKRD15	3,10	5,01	3,28
ANKRD16	4,96	5,19	5,17
ANKRD17	7,99	8,03	7,97
ANKRD20A1	6,82	6,09	2,67
ANKRD22	3,96	5,47	4,65
ANKRD25	0,32	4,44	4,77
ANKRD26	5,13	5,24	5,42
ANKRD28	1,93	4,37	0,44
ANKRD29	5,59	8,05	-3,32
ANKRD35	3,19	4,16	3,46
ANKRD37	8,23	6,57	5,84
ANKRD38	1,71	4,74	-1,40
ANKRD39	6,17	6,28	5,71
ANKRD40	7,28	7,87	7,12
ANKRD44	-1,17	-2,62	5,48
ANKRD49	8,34	8,29	8,74
ANKRD50	6,56	7,43	8,44
ANKRD53	4,24	4,77	4,66
ANKS1A	8,83	9,04	9,12
ANKS1B	3,63	3,04	2,92
ANKS3	3,14	-1,21	4,37
ANKZF1	6,43	5,90	7,67
ANP32A	7,44	7,42	8,34
ANP32B	12,66	12,50	13,13
ANP32E	2,97	3,52	2,38
ANTXR1	3,97	3,50	3,70
ANTXR2	10,73	9,39	10,26
ANUBL1	3,24	4,30	3,67
ANXA1	12,95	13,12	13,36
ANXA11	9,75	9,56	8,75
ANXA13	1,78	-0,46	3,07
ANXA2	11,00	11,79	9,30
ANXA3	4,02	2,51	-0,51
ANXA4	10,56	8,51	8,29
ANXA5	12,87	12,98	12,26
ANXA7	10,18	9,90	10,20
AOAH	8,15	8,06	11,26
AOC3	-0,02	-0,46	3,52
AOF1	4,93	5,37	6,72
AOF2	9,45	9,19	9,72

AP1G1	9,23	9,26	9,15
AP1G2	7,63	6,98	7,97
AP1GBP1	6,29	6,54	5,69
AP1M1	10,04	10,19	11,00
AP1M2	3,85	4,63	3,63
AP1S2	11,93	12,26	13,57
AP2A1	6,72	6,45	7,20
AP2B1	6,70	7,11	7,37
AP3B1	11,58	11,81	10,88
AP3B2	0,46	-1,35	2,61
AP3M1	8,43	8,68	7,95
AP3M2	8,19	7,63	6,01
AP3S2	6,76	7,02	7,32
AP4B1	8,72	8,85	9,22
AP4E1	8,01	8,63	7,65
AP4M1	4,27	4,23	5,02
APAF1	9,31	9,24	11,69
APBA2BP	5,21	5,00	4,98
APBA3	8,38	7,91	8,18
APBB1IP	10,50	9,59	10,83
APBB2	4,05	5,49	2,06
APBB3	7,59	7,43	9,73
APC2	4,61	4,88	4,77
APCDD1	-3,32	-0,89	4,77
APEX1	10,86	11,11	11,10
APEX2	9,82	9,29	8,02
APH1A	10,86	10,42	10,40
APH1B	8,24	8,48	8,38
API5	10,17	10,16	9,98
APIP	9,82	9,65	9,09
APLP2	9,74	10,91	10,94
APOA1BP	8,91	8,89	8,28
APOB48R	8,42	8,64	9,08
APOBEC3A	4,35	5,30	10,48
APOBEC3C	6,17	6,82	6,66
APOBEC3F	5,58	6,29	6,10
APOBEC3G	5,31	5,76	7,08
APOC1	11,41	13,87	3,51
APOD	6,96	5,25	3,20
APOE	12,39	14,21	0,47
APOL1	3,78	3,72	3,09
APOL2	4,24	3,66	3,39
APOL3	5,33	5,85	8,75
APOL4	6,94	5,86	3,22
APOL6	5,11	5,64	5,93
APOM	4,36	4,50	5,79
APP	8,72	8,30	8,15
APPBP1	8,94	9,10	7,96
APPBP2	4,21	4,41	5,11
AQP3	7,94	7,49	0,07
AQP9	12,52	13,08	11,46
AQR	8,15	8,14	8,67
ARAF	7,71	7,39	8,34
ARCN1	11,03	11,12	10,87
ARD1A	8,57	8,74	9,02
ARF1	11,53	11,19	11,47
ARF3	9,68	9,61	9,89
ARF4	10,42	10,62	10,37
ARF5	9,94	9,91	11,00

ARF6	3,35	-0,29	3,96
ARFGAP1	8,02	8,04	8,10
ARFGAP3	9,53	10,20	9,74
ARFGEF1	9,51	9,42	10,04
ARFGEF2	7,19	7,25	6,37
ARFIP1	8,12	8,55	8,89
ARFIP2	3,75	3,14	3,18
ARFRP1	5,03	4,18	4,87
ARG2	4,42	5,07	3,27
ARHGAP1	10,25	10,03	11,18
ARHGAP10	10,18	8,96	7,18
ARHGAP11A	2,30	3,18	3,06
ARHGAP12	8,25	7,76	7,60
ARHGAP15	9,32	9,98	9,84
ARHGAP18	10,60	10,05	6,86
ARHGAP19	7,29	7,69	9,22
ARHGAP21	10,94	10,19	10,24
ARHGAP22	6,57	6,09	3,29
ARHGAP24	3,00	5,04	8,11
ARHGAP25	8,59	8,62	8,46
ARHGAP26	4,92	2,66	6,42
ARHGAP30	9,30	9,43	10,70
ARHGAP4	9,36	8,93	11,03
ARHGAP5	3,40	4,83	3,90
ARHGAP6	3,93	4,15	5,20
ARHGAP8	4,37	3,57	3,70
ARHGAP9	10,97	10,76	12,30
ARHGDIB	14,29	14,63	14,53
ARHGDIG	4,60	5,24	5,40
ARHGEF1	7,46	7,73	9,66
ARHGEF10L	4,39	5,54	5,63
ARHGEF11	6,33	6,37	6,84
ARHGEF12	4,98	3,99	3,45
ARHGEF2	11,90	12,17	12,06
ARHGEF3	11,13	11,40	11,42
ARHGEF6	10,06	10,26	11,89
ARHGEF7	8,30	8,16	8,54
ARHGEF9	4,89	5,08	4,47
ARID1A	9,22	8,73	10,07
ARID1B	4,11	4,23	4,69
ARID2	5,34	5,32	6,41
ARID3A	10,15	9,15	10,42
ARID3B	5,93	7,11	7,94
ARID4A	7,88	7,10	8,09
ARID4B	6,23	5,92	6,76
ARID5B	7,99	8,07	6,67
ARIH1	8,01	7,81	8,65
ARL1	9,01	9,41	8,42
ARL13B	5,17	5,15	5,12
ARL15	6,76	6,85	4,11
ARL16	7,45	7,50	7,50
ARL17P1	4,41	4,36	5,31
ARL2BP	10,30	10,56	10,69
ARL3	4,56	4,96	4,83
ARL4A	5,19	7,72	7,54
ARL4C	7,34	4,51	6,86
ARL5A	11,38	11,04	11,51
ARL5B	5,36	5,52	3,88
ARL6IP2	6,79	6,77	6,89

ARL6IP4	8,19	7,71	8,48
ARL6IP5	13,19	12,80	12,76
ARL6IP6	9,64	8,94	9,28
ARMC1	9,49	9,40	9,08
ARMC5	5,73	5,31	6,42
ARMC6	6,84	6,38	6,87
ARMC7	7,77	7,38	7,44
ARMC8	5,97	6,15	6,19
ARMCX1	6,62	8,00	6,21
ARMCX2	4,09	6,21	5,51
ARMCX3	6,19	6,28	5,88
ARMCX4	4,83	5,44	4,92
ARMCX5	7,80	7,85	7,63
ARMCX6	7,74	8,37	7,16
ARMET	11,62	11,69	10,82
ARMETL1	4,09	4,29	4,25
ARNTL	7,37	6,86	7,19
ARNTL2	4,36	4,54	4,70
ARPC1A	9,87	9,92	10,08
ARPC2	12,38	12,34	12,15
ARPC3	12,47	12,49	13,19
ARPC4	10,80	10,77	10,67
ARPC5	13,57	13,31	13,25
ARPC5L	10,71	10,47	9,98
ARPM1	4,20	3,95	1,62
ARPP-19	8,33	8,32	8,10
ARPP-21	3,56	2,92	2,75
ARRB1	7,85	8,14	8,55
ARRDC3	6,15	6,79	7,18
ARRDC4	10,36	9,37	8,82
ARSB	7,19	7,67	6,44
ARSG	6,14	5,59	7,31
ARTN	2,13	1,98	1,98
ARTS-1	3,84	3,99	4,03
ARV1	10,63	9,76	8,24
ARVCF	4,37	4,12	4,12
ARX	4,37	4,16	4,62
AS3MT	1,86	2,72	5,25
ASAH1	8,21	8,94	7,75
ASAH3	3,87	3,98	4,56
ASAHL	10,55	8,95	10,71
ASB13	8,19	8,36	8,16
ASB16	4,48	4,45	4,62
ASB3	7,92	8,17	7,88
ASB7	4,70	4,76	5,76
ASB8	8,26	8,19	8,48
ASB9	3,70	4,12	5,48
ASCC1	8,78	9,11	9,14
ASCC2	10,40	10,41	10,92
ASCC3	7,40	7,58	7,56
ASCC3L1	6,27	5,91	7,33
ASCL1	2,98	4,09	-0,47
ASCL2	7,49	6,55	9,59
ASF1B	3,34	6,97	6,41
ASH1L	4,93	4,19	4,86
ASH2L	9,91	9,66	9,68
ASL	3,02	2,92	3,55
ASNA1	6,50	6,47	6,11
ASNS	8,33	6,22	6,59

ASNSD1	9,28	9,66	9,42
ASPH	5,84	6,04	4,26
ASPHD1	3,83	6,62	3,05
ASPHD2	8,07	6,37	8,85
ASPM	-3,32	7,35	-3,32
ASTE1	7,43	6,50	6,40
ASTN2	5,95	-0,96	-3,32
ASXL1	6,83	7,04	7,65
ATAD1	8,34	8,32	7,66
ATAD2	7,38	7,58	5,69
ATAD3A	7,00	6,46	6,79
ATBF1	8,43	8,52	9,88
ATE1	4,65	4,69	5,61
ATF1	8,07	7,42	8,12
ATF2	7,13	6,84	6,72
ATF3	9,50	10,29	6,63
ATF4	12,35	11,66	12,46
ATF5	11,23	9,67	9,29
ATF6	10,47	10,58	9,88
ATF7	3,85	3,49	4,00
ATF7IP	3,20	4,30	4,73
ATG10	8,04	7,43	8,72
ATG12	6,90	6,55	6,86
ATG16L2	6,29	6,06	9,23
ATG3	11,06	10,84	11,70
ATG4A	7,17	8,13	6,55
ATG4C	8,89	8,92	8,79
ATG5	6,16	6,36	7,31
ATG9A	8,83	8,58	7,55
ATG9B	3,12	4,04	4,28
ATHL1	4,09	4,18	7,25
ATN1	5,48	6,05	4,58
ATOH1	4,63	4,15	4,99
ATOH8	2,24	2,87	5,91
ATOX1	12,03	12,29	10,05
ATP10A	4,34	4,84	4,38
ATP10D	4,08	3,97	4,31
ATP11B	6,42	6,83	7,35
ATP11C	7,11	6,68	7,16
ATP13A1	9,32	9,02	9,04
ATP1A3	3,76	3,82	2,73
ATP1B1	11,44	11,52	6,36
ATP1B2	5,45	3,90	3,33
ATP1B3	11,28	11,45	10,58
ATP1B4	-2,05	2,08	-1,53
ATP2A2	9,87	10,15	9,04
ATP2A3	6,77	6,11	4,75
ATP2B1	5,44	4,57	6,37
ATP2B3	0,33	4,16	2,21
ATP2B4	8,20	8,57	8,66
ATP2C1	8,33	9,23	7,58
ATP4A	3,99	4,16	3,78
ATP5A1	13,60	13,77	13,09
ATP5B	14,27	14,20	13,56
ATP5D	10,26	9,82	10,18
ATP5F1	12,00	12,15	11,79
ATP5G1	9,18	9,71	8,46
ATP5G2	11,64	11,88	11,88
ATP5G3	7,23	7,37	5,06

ATP5H	12,98	13,17	12,65
ATP5J	11,76	12,06	11,10
ATP5J2	12,28	12,15	11,70
ATP5L	12,50	12,87	12,39
ATP5O	13,19	13,41	13,03
ATP5S	7,25	7,79	8,27
ATP6AP1	13,85	13,92	12,38
ATP6AP2	13,31	13,67	13,16
ATP6V0A1	12,11	11,84	10,89
ATP6V0B	12,85	13,10	12,43
ATP6V0D1	11,20	12,21	11,34
ATP6V0D2	5,52	9,42	4,81
ATP6V1A	12,93	13,11	12,45
ATP6V1B2	13,81	14,11	12,87
ATP6V1C1	8,48	8,58	7,48
ATP6V1D	12,06	11,96	10,15
ATP6V1E1	12,00	12,27	10,98
ATP6V1F	11,41	11,71	10,20
ATP6V1G1	12,18	11,78	12,04
ATP6V1G2	4,47	4,51	4,61
ATP6V1H	9,77	10,37	7,65
ATP7A	6,65	6,98	5,86
ATP7B	5,34	4,82	4,82
ATP8A2	4,66	4,49	4,63
ATP8B3	4,29	1,98	-3,32
ATP8B4	8,76	9,31	9,50
ATP9A	2,38	3,59	3,23
ATP9B	6,76	7,75	5,58
ATPAF1	8,99	9,38	8,96
ATPAF2	7,19	7,87	6,96
ATPBD1B	7,98	7,59	8,55
ATPBD1C	5,16	4,61	4,42
ATPBD4	5,31	5,34	5,52
ATPIF1	9,29	9,28	8,96
ATR	3,70	3,70	4,29
ATRN	6,56	6,55	6,97
ATXN1	10,02	8,76	9,02
ATXN10	4,96	5,44	6,57
ATXN2	10,29	9,79	10,29
ATXN2L	-1,05	-3,32	4,08
ATXN7L1	3,03	3,42	4,38
ATXN7L2	5,12	5,34	6,33
AUH	9,89	7,91	7,66
AUP1	8,61	8,32	8,82
AURKA	8,05	8,90	5,78
AURKB	3,73	6,83	4,90
AVEN	7,23	6,46	6,81
AVP	2,51	4,44	4,47
AVPI1	10,74	11,04	8,24
AVPR2	4,96	4,82	5,04
AXL	-1,30	4,29	1,74
AYTL1	8,41	8,33	7,15
AZI1	5,32	5,33	5,71
AZI2	7,48	7,85	8,83
AZIN1	9,84	10,22	10,22
B2M	14,36	14,32	14,14
B3GALNT2	6,10	6,06	4,32
B3GALT4	7,64	7,15	7,68
B3GALT6	7,66	7,09	7,73

B3GAT1	2,89	2,28	3,59
B3GAT3	6,24	6,43	6,71
B3GNT1	8,03	8,80	6,08
B3GNT3	3,89	3,72	4,14
B3GNT5	8,47	7,94	6,43
B3GNTL1	5,36	4,57	5,32
B4GALT1	8,78	8,34	8,80
B4GALT2	3,75	3,73	1,26
B4GALT3	9,20	9,37	8,83
B4GALT5	8,91	9,44	8,51
B4GALT6	-1,21	4,22	3,92
B4GALT7	7,99	8,36	8,36
BAALC	3,63	4,81	3,95
BACE1	6,40	6,13	5,62
BACE2	5,05	4,54	4,13
BACH1	4,67	4,89	5,85
BAG1	4,85	5,10	5,20
BAG3	8,53	9,65	7,18
BAG4	8,23	7,99	8,77
BAHD1	6,53	5,91	6,11
BAI1	3,37	2,86	2,68
BAIAP2	7,02	6,64	6,21
BAMBI	4,33	6,60	5,07
BANF1	7,85	7,86	7,44
BANP	10,14	9,59	10,50
BAP1	7,74	7,00	7,18
BARD1	6,04	6,30	5,88
BARHL2	3,90	4,56	4,79
BASP1	11,91	11,83	12,08
BAT1	9,71	9,58	10,51
BAT2	8,24	7,94	8,93
BAT2D1	9,95	9,90	10,45
BAT3	8,81	8,82	9,11
BAT4	5,30	4,96	5,13
BAT5	9,75	9,45	9,21
BAZ1A	9,57	8,66	9,86
BAZ2A	4,12	3,83	4,80
BAZ2B	9,33	8,85	9,30
BBC3	7,20	7,33	7,68
BBS1	6,27	6,02	6,65
BBS2	8,67	8,90	8,90
BBS4	7,86	8,14	6,45
BBS7	4,45	5,15	5,28
BBX	8,57	9,03	10,10
BCAM	3,62	4,03	3,40
BCAN	3,34	3,69	3,15
BCAP29	6,68	7,00	6,62
BCAP31	12,55	13,24	11,24
BCAR1	3,84	4,22	-3,32
BCAR3	11,16	9,15	4,49
BCAS2	6,38	6,57	6,70
BCAS3	3,71	3,57	4,42
BCAT1	8,67	11,40	8,49
BCCIP	8,28	8,54	8,87
BCDO2	7,93	8,11	7,76
BCKDHA	9,64	9,82	10,95
BCKDHB	6,18	6,06	4,97
BCKDK	12,06	11,43	11,38
BCL10	7,03	6,90	6,35

BCL11A	6,67	7,31	7,46
BCL11B	3,53	3,89	4,20
BCL2	6,05	6,13	5,15
BCL2A1	7,38	10,08	8,37
BCL2L1	10,34	10,44	7,80
BCL2L11	4,90	3,33	6,11
BCL2L12	6,97	7,04	6,68
BCL2L13	10,24	10,60	10,44
BCL2L14	1,35	2,31	1,93
BCL2L2	8,88	8,82	9,09
BCL3	6,74	6,25	8,39
BCL6	11,09	10,39	10,85
BCL7A	5,58	4,30	4,12
BCL7C	8,02	7,74	7,35
BCL9	7,16	5,51	6,05
BCL9L	5,04	5,35	5,98
BCLAF1	9,41	9,23	9,96
BCOR	7,19	7,14	7,95
BCORL1	5,18	5,26	5,10
BCS1L	6,88	6,15	6,82
BDKRB2	3,80	4,34	3,88
BDNF	2,96	2,95	3,10
BDP1	5,20	4,70	6,24
BECN1	9,22	9,43	9,66
BET1	8,39	8,39	6,06
BET1L	10,37	10,19	10,59
BEX1	-3,32	5,58	-3,32
BFAR	8,91	9,04	8,45
BHLHB2	11,91	12,81	11,59
BHLHB3	7,52	9,27	-0,29
BHLHB5	0,56	-3,32	-3,32
BHLHB9	4,42	5,20	3,59
BICD1	3,94	5,59	3,40
BICD2	11,14	10,59	11,08
BID	9,52	8,39	9,20
BIN1	8,96	9,00	7,80
BIN2	8,63	8,92	11,47
BIN3	3,82	4,43	6,35
BIRC2	7,53	7,68	7,78
BIRC3	10,02	7,24	7,56
BIRC4	7,32	7,23	6,38
BIRC6	8,19	7,27	8,14
BIVM	5,83	5,94	4,82
BLCAP	9,65	9,71	9,11
BLM	4,93	6,31	5,92
BLMH	9,48	9,95	9,25
BLNK	8,61	8,18	4,87
BLOC1S1	10,99	11,35	10,93
BLOC1S2	5,14	5,46	4,58
BLR1	3,23	3,49	3,75
BLVRA	11,76	11,71	11,96
BLVRB	9,73	8,94	9,73
BLZF1	7,24	7,51	6,92
BMF	5,59	5,47	7,13
BMP1	4,10	4,80	4,56
BMP2K	8,06	8,04	7,87
BMP6	4,35	3,36	-3,32
BMP8B	4,06	4,30	4,18
BMPER	3,04	3,79	0,03

BMPR1A	4,97	5,70	-0,48
BMPR2	8,68	8,60	8,25
BMX	2,94	3,23	3,47
BNIP1	6,02	6,37	6,48
BNIP2	10,84	10,81	11,23
BNIP3	8,66	6,54	7,18
BNIP3L	9,93	10,10	11,26
BOK	4,50	4,04	3,84
BOLA2	9,47	9,56	9,35
BOLA3	10,60	11,22	7,67
BOP1	8,97	8,53	9,90
BPGM	6,06	5,94	5,31
BPI	3,82	7,25	7,14
BPNT1	8,37	7,55	6,46
BRAF	5,09	4,75	4,73
BRAP	3,62	2,97	3,95
BRCA1	4,16	5,20	4,21
BRCC3	7,36	6,94	6,22
BRD1	6,31	5,32	6,94
BRD2	10,51	10,59	11,64
BRD3	8,23	6,96	9,62
BRD4	6,60	6,21	7,37
BRD8	5,85	5,62	6,53
BRD9	9,14	8,85	9,41
BRDG1	3,23	6,91	1,40
BRE	7,88	8,60	8,34
BRF1	4,17	3,06	4,34
BRF2	7,07	7,12	6,63
BRI3	12,70	12,72	12,13
BRI3BP	7,61	7,53	7,58
BRIP1	4,51	4,28	2,52
BRMS1	7,90	7,94	8,07
BRMS1L	6,19	6,83	6,04
BRP44	10,24	11,53	9,45
BRP44L	10,40	10,51	9,48
BRPF1	4,32	4,85	4,09
BRSK1	5,48	5,67	5,52
BRUNOL4	2,87	3,08	2,95
BRUNOL6	6,62	6,03	4,89
BRWD1	6,77	6,23	7,29
BRWD2	7,79	8,24	8,68
BRWD3	6,80	6,06	7,21
BSCL2	10,39	11,03	9,29
BSDC1	9,21	9,29	9,53
BSG	10,29	10,52	9,52
BST1	5,11	7,19	9,29
BST2	10,28	10,62	11,10
BTAF1	9,66	9,23	10,32
BTBD1	9,63	9,62	8,63
BTBD10	8,98	8,85	8,55
BTBD12	9,79	7,85	7,25
BTBD14B	4,56	5,23	4,96
BTBD15	6,71	6,25	8,68
BTBD3	6,66	6,40	6,63
BTBD6	7,56	7,45	7,05
BTBD7	6,76	6,83	7,30
BTD	6,95	7,19	6,65
BTF3	9,23	9,07	10,27
BTF3L4	9,48	9,90	10,18

BTG1	11,85	11,41	13,27
BTG2	8,69	9,75	10,02
BTK	10,57	10,74	11,55
BTN1A1	3,42	4,48	4,25
BTN2A1	7,08	7,52	8,30
BTN2A2	5,61	5,78	6,17
BTN2A3	3,35	3,84	4,15
BTN3A1	6,04	6,80	7,99
BTN3A2	6,94	7,68	8,49
BTN3A3	6,23	7,32	8,73
BTRC	4,61	4,98	2,60
BUB1	5,16	7,84	3,37
BUB1B	-1,38	6,95	-3,32
BUB3	10,33	10,38	10,36
BUD13	7,30	7,34	7,49
BUD31	11,12	11,12	11,05
BXDC1	10,22	10,46	9,74
BXDC2	9,45	9,44	9,03
BXDC5	8,97	9,17	9,22
BYSL	6,78	7,00	6,67
BZRAP1	4,24	4,00	3,81
BZW2	10,09	9,49	9,84
C10ORF104	7,90	7,34	9,08
C10ORF118	5,28	5,44	5,92
C10ORF119	5,94	5,62	7,33
C10ORF132	-3,32	5,59	-3,32
C10ORF137	7,60	7,33	7,75
C10ORF22	10,92	10,66	8,14
C10ORF26	7,61	8,12	8,89
C10ORF28	7,91	8,06	8,38
C10ORF35	-3,32	3,18	-3,32
C10ORF46	5,95	6,47	5,91
C10ORF55	4,73	4,66	4,43
C10ORF56	6,46	6,81	7,42
C10ORF58	8,24	11,35	4,94
C10ORF59	6,23	7,29	6,22
C10ORF6	8,39	8,21	7,85
C10ORF64	6,51	5,06	3,90
C10ORF71	3,75	3,96	3,07
C10ORF72	3,48	3,76	2,91
C10ORF78	6,95	7,30	7,79
C10ORF83	4,56	4,23	2,07
C10ORF88	6,87	6,37	5,65
C10ORF93	2,90	2,66	3,21
C10ORF97	8,91	9,34	9,10
C11ORF10	12,59	12,61	12,44
C11ORF17	9,07	9,47	7,52
C11ORF2	10,22	9,86	10,90
C11ORF24	6,42	6,49	5,76
C11ORF30	4,85	5,10	5,23
C11ORF31	4,84	4,59	5,79
C11ORF45	6,48	8,75	1,86
C11ORF46	8,55	7,91	8,65
C11ORF48	9,15	8,99	8,78
C11ORF51	7,07	7,42	8,18
C11ORF52	5,84	6,12	6,22
C11ORF54	6,51	7,46	5,64
C11ORF56	7,06	7,49	9,00
C11ORF57	7,82	7,77	8,53

C11ORF58	7,26	6,81	7,46
C11ORF59	11,12	11,03	11,19
C11ORF60	6,29	6,38	4,58
C11ORF61	5,36	5,07	5,39
C11ORF63	4,99	5,57	2,90
C11ORF67	8,36	8,01	7,62
C11ORF68	7,74	7,31	7,55
C11ORF71	7,02	7,07	7,32
C11ORF73	8,01	8,07	8,32
C11ORF74	7,63	8,76	8,03
C11ORF9	4,31	4,68	4,55
C12ORF10	8,99	9,29	10,16
C12ORF11	7,67	8,03	7,17
C12ORF23	6,20	6,65	4,87
C12ORF24	7,10	7,75	8,20
C12ORF26	7,77	7,88	8,36
C12ORF29	6,84	6,77	6,83
C12ORF30	8,58	7,40	7,56
C12ORF31	8,40	8,58	9,07
C12ORF32	8,33	7,40	7,28
C12ORF34	5,43	4,81	4,84
C12ORF35	7,74	7,07	9,21
C12ORF4	8,63	8,61	7,51
C12ORF41	9,26	9,51	10,25
C12ORF43	6,16	6,11	6,76
C12ORF44	8,74	8,68	8,79
C12ORF47	10,40	10,72	10,98
C12ORF48	5,13	6,49	4,61
C12ORF49	8,10	8,76	7,42
C12ORF5	9,96	10,05	7,81
C12ORF52	8,27	7,79	7,79
C12ORF57	8,19	7,92	7,93
C12ORF60	4,37	5,28	-0,87
C12ORF61	5,09	5,29	5,11
C12ORF62	9,68	9,36	9,30
C13ORF18	3,25	6,47	6,67
C13ORF23	8,66	8,38	8,89
C13ORF24	6,50	6,17	6,69
C13ORF26	3,70	4,03	3,00
C13ORF3	4,10	5,64	3,86
C13ORF7	7,24	7,20	6,32
C13ORF8	9,30	8,84	8,34
C14ORF1	5,20	5,32	5,23
C14ORF100	10,40	10,26	9,68
C14ORF102	8,36	8,58	9,29
C14ORF106	10,08	9,93	11,20
C14ORF108	9,50	9,51	8,43
C14ORF112	10,46	10,72	10,31
C14ORF118	5,73	5,84	5,77
C14ORF122	6,07	6,19	6,00
C14ORF124	5,22	4,82	5,69
C14ORF126	5,25	5,24	5,81
C14ORF130	7,57	8,21	7,58
C14ORF133	7,28	7,33	7,47
C14ORF138	6,77	6,88	7,95
C14ORF140	5,19	4,72	3,06
C14ORF142	9,86	10,52	8,78
C14ORF145	5,63	5,02	0,08
C14ORF147	8,20	7,62	9,43

C14ORF149	6,36	5,20	5,31
C14ORF152	-0,26	2,63	3,44
C14ORF153	-3,32	-0,33	4,99
C14ORF156	12,21	12,66	12,03
C14ORF159	10,28	10,36	10,52
C14ORF162	3,55	3,90	3,65
C14ORF166	11,31	11,10	11,71
C14ORF174	5,80	6,06	5,94
C14ORF2	9,32	9,31	10,18
C14ORF21	4,54	3,93	4,67
C14ORF24	5,18	4,96	3,37
C14ORF37	4,15	2,56	3,39
C14ORF43	8,50	8,08	9,69
C14ORF48	5,60	5,95	5,89
C14ORF93	7,33	7,47	8,51
C14ORF94	9,24	9,24	9,57
C15ORF15	10,88	10,83	11,92
C15ORF17	7,88	7,32	7,06
C15ORF23	5,20	7,18	5,46
C15ORF24	10,10	10,33	9,58
C15ORF27	7,62	6,29	1,28
C15ORF38	6,70	6,59	6,12
C15ORF39	7,18	7,39	10,91
C15ORF40	6,25	6,28	6,06
C15ORF44	6,04	6,48	5,03
C15ORF5	3,30	3,10	5,65
C16ORF24	8,96	8,78	8,91
C16ORF30	0,05	1,98	2,70
C16ORF33	10,58	10,08	8,87
C16ORF45	4,14	4,27	4,46
C16ORF46	4,96	4,11	5,04
C16ORF48	7,38	7,32	7,99
C16ORF5	4,63	5,07	3,28
C16ORF52	6,06	5,98	5,99
C16ORF53	7,23	7,16	7,62
C16ORF54	4,38	4,89	5,12
C16ORF57	9,22	9,10	9,55
C16ORF58	9,20	9,77	9,41
C16ORF59	-3,32	0,55	-3,32
C16ORF61	11,58	11,80	11,15
C16ORF63	9,79	10,09	9,87
C16ORF68	7,95	8,80	8,54
C16ORF7	8,56	8,52	8,20
C16ORF70	6,27	6,42	6,46
C17ORF32	8,76	8,89	7,92
C17ORF39	6,05	6,32	6,66
C17ORF45	12,13	11,98	12,09
C17ORF48	6,41	6,47	7,47
C17ORF49	9,27	9,24	10,09
C17ORF56	7,69	7,17	9,25
C17ORF58	9,05	7,69	5,83
C17ORF59	10,37	9,48	9,92
C17ORF61	10,49	10,56	10,89
C17ORF62	9,83	9,39	9,78
C17ORF63	7,86	8,21	7,00
C17ORF64	5,71	4,33	3,60
C17ORF65	4,97	4,89	5,85
C17ORF67	5,41	5,97	5,95
C17ORF68	6,70	6,27	6,05

C17ORF70	9,40	9,23	9,93
C17ORF71	6,13	6,01	5,80
C17ORF75	3,51	0,68	3,90
C17ORF76	6,10	6,21	6,10
C17ORF77	3,85	3,75	3,24
C17ORF79	10,56	10,85	8,61
C17ORF80	6,32	6,11	5,16
C17ORF81	5,86	5,72	6,05
C18ORF1	6,52	6,14	6,03
C18ORF10	6,45	7,44	6,40
C18ORF19	7,35	7,22	6,40
C18ORF21	8,58	8,36	9,21
C18ORF22	6,33	6,28	5,84
C18ORF24	2,34	4,99	2,26
C18ORF25	8,43	8,38	9,08
C18ORF37	8,86	9,57	8,39
C18ORF45	7,32	6,97	6,01
C18ORF54	3,40	4,76	4,16
C18ORF55	8,93	8,98	7,71
C18ORF56	-3,32	5,12	-3,32
C18ORF8	9,51	9,33	9,79
C19ORF12	8,37	8,29	6,86
C19ORF22	9,82	9,47	11,01
C19ORF23	6,11	5,72	5,35
C19ORF24	7,99	7,98	7,85
C19ORF26	4,29	4,41	4,62
C19ORF28	11,97	11,73	8,08
C19ORF30	4,70	3,84	4,88
C19ORF35	3,30	3,47	4,69
C19ORF39	4,59	3,94	5,75
C19ORF40	5,20	5,54	4,38
C19ORF43	10,45	10,11	10,93
C19ORF6	6,82	6,74	7,15
C1D	5,76	4,99	5,65
C1QA	10,79	11,68	7,59
C1QBP	10,30	10,08	9,98
C1QL1	2,64	4,09	2,44
C1QL2	5,11	5,16	4,72
C1QL3	4,25	3,72	4,45
C1QTNF2	4,76	4,98	4,68
C1ORF102	4,73	6,06	2,43
C1ORF103	5,20	3,38	-1,20
C1ORF104	2,11	2,65	4,47
C1ORF105	4,35	3,89	3,75
C1ORF107	4,05	3,76	3,96
C1ORF108	9,87	8,48	8,24
C1ORF112	6,21	6,94	6,29
C1ORF115	8,83	6,08	6,02
C1ORF119	9,12	9,14	10,39
C1ORF120	3,85	4,65	4,18
C1ORF121	9,09	9,06	9,13
C1ORF122	10,33	9,36	7,76
C1ORF123	8,76	8,82	10,00
C1ORF124	6,03	5,96	5,34
C1ORF128	9,74	9,87	9,94
C1ORF130	6,93	6,94	7,03
C1ORF131	8,29	8,11	8,27
C1ORF135	3,00	4,17	3,50
C1ORF142	5,78	5,97	5,83

C1ORF149	5,75	5,58	5,71
C1ORF156	5,30	5,78	5,06
C1ORF159	5,24	5,26	5,97
C1ORF162	13,37	12,84	13,64
C1ORF164	5,93	5,65	6,62
C1ORF166	8,29	8,32	7,49
C1ORF174	10,13	9,96	9,84
C1ORF176	7,22	7,30	6,03
C1ORF181	7,90	8,11	7,35
C1ORF183	4,61	4,59	4,97
C1ORF187	-0,50	4,93	1,65
C1ORF19	8,50	8,30	8,30
C1ORF190	3,92	3,74	2,88
C1ORF198	4,60	6,10	5,31
C1ORF2	5,78	5,49	6,28
C1ORF201	4,92	5,30	4,11
C1ORF21	2,75	4,25	0,51
C1ORF25	7,96	7,78	6,91
C1ORF26	6,91	6,35	6,76
C1ORF27	5,12	4,23	3,81
C1ORF31	7,19	7,64	6,21
C1ORF32	3,95	3,80	4,30
C1ORF35	7,15	6,47	7,27
C1ORF38	5,17	5,90	8,35
C1ORF41	9,18	9,05	8,62
C1ORF43	11,01	10,96	10,48
C1ORF50	9,45	9,06	9,01
C1ORF51	4,04	2,71	-0,33
C1ORF53	6,40	6,33	7,11
C1ORF54	11,98	11,94	9,19
C1ORF55	8,28	8,39	8,66
C1ORF56	3,12	4,54	5,03
C1ORF57	8,76	9,05	8,25
C1ORF58	5,67	5,86	5,58
C1ORF59	8,00	8,91	9,38
C1ORF63	10,09	9,60	11,49
C1ORF66	8,32	8,32	8,44
C1ORF69	4,99	4,43	4,74
C1ORF71	9,51	10,11	9,46
C1ORF74	6,46	6,57	4,86
C1ORF77	9,08	9,22	8,89
C1ORF78	6,61	6,43	6,89
C1ORF83	5,96	5,59	6,55
C1ORF84	4,32	4,05	5,21
C1ORF85	11,46	11,82	10,46
C1ORF88	4,64	4,49	4,59
C1ORF89	4,33	3,84	4,02
C1ORF9	5,22	4,17	5,68
C1ORF91	5,17	4,79	5,38
C1ORF92	4,82	5,30	4,97
C1ORF93	8,27	9,14	6,29
C1ORF94	4,19	4,45	3,92
C1ORF95	4,01	4,29	4,57
C1ORF96	7,10	6,32	5,79
C1ORF97	7,07	7,67	5,98
C2	8,51	6,19	5,05
C20ORF103	-0,23	6,31	5,67
C20ORF11	9,60	9,28	9,70
C20ORF111	6,35	5,71	6,20

C20ORF116	9,95	10,17	9,26
C20ORF117	7,02	6,55	6,19
C20ORF12	5,20	5,26	5,39
C20ORF121	5,48	6,33	6,59
C20ORF133	4,01	3,93	3,88
C20ORF134	3,50	3,09	4,52
C20ORF149	7,02	7,39	6,63
C20ORF177	7,98	7,77	9,20
C20ORF19	4,81	4,79	6,75
C20ORF23	6,21	6,71	4,97
C20ORF24	13,25	13,52	12,92
C20ORF26	4,25	4,92	4,93
C20ORF27	9,79	9,69	10,12
C20ORF29	6,86	7,03	6,15
C20ORF3	9,75	10,48	9,05
C20ORF30	12,06	12,32	12,40
C20ORF32	4,42	4,49	3,56
C20ORF39	5,51	5,43	4,99
C20ORF4	8,60	8,64	8,83
C20ORF43	11,41	11,51	11,84
C20ORF46	3,52	4,24	1,28
C20ORF52	11,03	11,03	10,77
C20ORF67	6,20	6,20	7,57
C20ORF72	9,77	9,63	8,98
C20ORF94	6,32	5,71	6,55
C21ORF119	6,47	6,54	6,45
C21ORF122	2,56	1,96	4,30
C21ORF124	6,30	5,71	5,68
C21ORF129	5,97	6,29	6,12
C21ORF13	3,31	3,31	3,48
C21ORF2	6,55	7,38	7,57
C21ORF33	10,99	10,64	10,23
C21ORF34	5,06	4,26	4,09
C21ORF51	4,63	4,51	6,19
C21ORF55	13,66	13,31	14,07
C21ORF56	2,71	4,03	4,75
C21ORF57	6,84	7,15	6,64
C21ORF59	6,52	6,82	6,89
C21ORF63	2,93	3,22	5,35
C21ORF66	6,49	6,61	7,56
C21ORF67	4,85	5,09	4,67
C21ORF7	6,09	6,62	9,05
C21ORF70	4,79	3,88	4,33
C21ORF81	4,73	2,65	1,36
C21ORF91	3,37	3,19	5,59
C22ORF13	11,17	11,07	10,57
C22ORF25	8,67	8,77	8,37
C22ORF9	7,50	7,94	6,92
C2ORF13	4,78	4,73	4,16
C2ORF15	3,03	3,22	3,70
C2ORF16	4,70	4,65	5,28
C2ORF21	5,23	4,98	3,79
C2ORF24	9,06	8,43	8,97
C2ORF25	11,44	11,60	10,99
C2ORF28	10,86	11,16	10,69
C2ORF29	10,67	10,33	10,80
C2ORF30	10,08	10,25	8,89
C2ORF32	6,77	8,39	1,16
C2ORF33	8,27	8,36	8,31

C2ORF34	6,91	6,52	6,72
C2ORF37	4,91	4,52	3,91
C2ORF7	9,07	9,30	7,76
C3AR1	5,93	7,67	7,38
C3ORF1	10,97	11,04	10,57
C3ORF14	5,37	5,53	4,56
C3ORF17	4,01	4,28	3,94
C3ORF21	10,41	8,26	9,70
C3ORF23	6,81	6,84	5,98
C3ORF26	5,01	6,03	5,41
C3ORF28	10,25	10,15	8,64
C3ORF31	8,54	7,93	7,86
C3ORF37	7,16	7,81	6,49
C3ORF38	8,29	8,13	7,94
C3ORF39	6,65	7,32	6,53
C3ORF54	5,19	5,30	5,65
C3ORF58	7,56	7,12	8,66
C3ORF59	7,52	7,66	6,73
C3ORF60	3,11	3,53	3,00
C3ORF62	5,68	5,63	7,18
C3ORF64	7,39	8,30	5,79
C4BPB	4,22	4,01	4,87
C4ORF14	8,76	8,95	10,29
C4ORF16	8,56	8,75	8,23
C4ORF18	9,75	10,72	11,22
C4ORF20	8,77	9,05	8,30
C5AR1	7,64	10,57	11,05
C5ORF13	7,63	8,70	8,83
C5ORF15	10,96	10,64	11,04
C5ORF22	6,93	6,96	6,76
C5ORF24	6,59	6,19	7,22
C5ORF3	6,10	6,08	6,36
C5ORF4	2,57	3,99	2,98
C5ORF5	10,36	9,94	9,46
C6ORF106	7,83	7,79	7,08
C6ORF108	9,60	9,23	7,40
C6ORF113	7,99	7,93	7,90
C6ORF114	5,91	5,22	4,72
C6ORF120	6,48	6,21	7,11
C6ORF128	6,17	6,90	0,28
C6ORF129	8,06	8,78	6,67
C6ORF130	8,55	8,91	8,68
C6ORF134	4,58	4,62	5,04
C6ORF145	6,83	5,65	4,72
C6ORF151	5,27	5,99	6,04
C6ORF153	10,14	10,10	10,19
C6ORF157	4,65	4,81	4,52
C6ORF166	11,26	10,86	11,36
C6ORF167	4,81	5,29	4,71
C6ORF173	7,01	8,11	6,99
C6ORF174	3,88	3,43	4,56
C6ORF182	5,07	4,89	4,07
C6ORF192	7,14	8,89	9,70
C6ORF199	5,02	5,07	4,78
C6ORF203	7,70	7,19	6,58
C6ORF204	5,33	4,96	8,29
C6ORF211	7,59	8,07	6,87
C6ORF25	3,50	3,69	3,71
C6ORF47	4,79	4,72	5,37

C6ORF48	7,01	6,86	8,31
C6ORF49	11,88	11,80	11,69
C6ORF52	4,10	3,73	3,68
C6ORF62	12,18	11,65	11,55
C6ORF64	6,11	6,63	6,70
C6ORF66	8,56	8,67	6,70
C6ORF70	7,14	7,33	7,29
C6ORF72	10,45	10,37	10,52
C6ORF85	4,10	-0,65	-0,08
C6ORF89	6,94	7,03	5,71
C6ORF97	3,02	3,92	5,37
C7ORF10	4,23	3,89	1,39
C7ORF11	7,84	7,80	8,07
C7ORF13	5,14	5,19	5,85
C7ORF23	10,04	10,38	9,40
C7ORF24	9,09	9,30	7,17
C7ORF28A	7,89	8,13	8,59
C7ORF28B	8,26	8,54	8,43
C7ORF31	4,23	4,32	4,40
C7ORF36	7,22	7,48	5,37
C7ORF38	11,14	10,68	12,63
C8B	2,51	3,86	3,72
C8ORF13	0,14	-0,05	4,73
C8ORF32	7,80	7,88	7,33
C8ORF33	8,25	8,43	7,45
C8ORF38	8,41	8,93	6,50
C8ORF40	9,56	9,85	9,17
C8ORF41	8,13	8,18	7,71
C8ORF44	4,96	4,26	5,06
C8ORF48	5,72	5,86	5,96
C8ORF51	5,38	4,71	4,61
C8ORF53	6,86	6,91	7,40
C8ORF54	3,83	3,91	2,67
C8ORF58	6,02	5,20	5,56
C8ORF70	6,95	7,73	7,11
C8ORF79	3,22	3,53	4,17
C9ORF100	3,08	3,27	2,58
C9ORF106	2,67	1,39	4,31
C9ORF114	8,17	7,93	7,95
C9ORF116	4,70	5,19	4,65
C9ORF119	7,97	8,45	7,60
C9ORF123	6,65	6,72	7,04
C9ORF127	4,34	4,66	8,56
C9ORF128	-0,62	2,86	4,14
C9ORF130	7,92	7,48	8,96
C9ORF142	8,88	8,57	10,03
C9ORF156	8,67	8,77	8,22
C9ORF16	7,23	7,12	6,70
C9ORF18	3,60	4,13	4,15
C9ORF21	8,08	7,99	8,32
C9ORF23	7,81	7,64	7,79
C9ORF24	4,53	3,57	3,45
C9ORF25	5,05	4,82	4,98
C9ORF3	4,59	4,29	4,46
C9ORF30	10,43	10,45	6,61
C9ORF37	5,94	5,60	6,01
C9ORF40	5,82	6,17	4,21
C9ORF41	4,82	5,03	4,40
C9ORF46	10,57	10,93	8,48

C9ORF58	6,19	6,04	6,60
C9ORF6	6,35	7,42	7,35
C9ORF64	7,24	7,24	6,94
C9ORF66	6,39	5,78	7,21
C9ORF72	7,65	7,51	10,16
C9ORF78	10,78	10,64	11,35
C9ORF80	8,49	8,47	8,48
C9ORF82	6,92	6,69	7,05
C9ORF85	6,72	6,50	7,11
C9ORF9	6,26	4,72	4,58
C9ORF90	5,76	5,48	5,81
C9ORF91	6,46	6,33	7,89
C9ORF93	3,94	4,33	2,51
C9ORF95	7,43	7,85	6,91
C9ORF97	4,48	4,67	4,34
C9ORF98	6,57	6,76	-3,32
CA11	7,44	9,06	4,25
CA12	4,05	5,25	3,86
CA13	3,24	4,59	5,78
CA2	11,62	12,99	8,26
CA4	3,07	3,85	3,94
CA5A	6,91	3,99	1,24
CA5B	5,78	5,74	8,80
CAB39	11,98	12,11	12,43
CAB39L	6,32	7,25	4,73
CABC1	9,35	8,76	9,85
CABIN1	8,09	6,90	7,62
CABYR	5,29	5,41	5,17
CACNA1A	3,79	3,57	4,29
CACNA2D2	4,10	3,84	3,94
CACNA2D3	-1,84	4,43	9,71
CACNA2D4	5,96	7,01	8,65
CACNB1	2,80	2,50	3,84
CACNB4	3,68	1,66	2,83
CACNG2	5,12	4,91	5,63
CACNG3	-0,18	-0,27	2,73
CACNG7	3,71	2,97	4,02
CACYBP	7,17	7,17	6,40
CAD	6,78	6,61	5,28
CAGE1	3,81	3,81	3,65
CALCB	4,05	3,53	4,36
CALCOCO1	7,19	7,56	7,63
CALCOCO2	6,39	6,12	7,72
CALCR	5,37	3,74	4,30
CALCRL	8,59	4,13	4,57
CALD1	4,74	4,25	4,13
CALM1	12,22	12,06	11,36
CALM2	14,12	13,62	13,37
CALM3	11,89	12,96	12,32
CALML4	7,43	7,69	9,09
CALML6	3,50	3,86	3,89
CALN1	5,30	5,63	5,53
CALR	11,17	11,73	10,08
CALU	10,22	10,04	7,82
CAMK1D	8,26	6,05	7,00
CAMK1G	6,96	6,55	4,86
CAMK2B	2,78	4,15	0,50
CAMK2D	6,47	5,86	5,38
CAMK2G	7,40	7,32	9,04

CAMKK1	2,37	1,49	3,64
CAMLG	10,89	10,91	11,74
CAMSAP1L1	8,75	8,56	6,96
CAMTA1	5,12	5,44	4,41
CAMTA2	5,06	4,72	4,57
CAND1	8,42	8,31	8,05
CANT1	8,37	8,67	8,92
CANX	10,79	10,85	11,07
CAP1	13,66	13,40	13,40
CAPG	11,19	11,77	9,72
CAPN1	10,63	10,75	11,00
CAPN10	3,19	3,25	4,04
CAPN5	4,45	6,76	4,42
CAPN6	7,64	7,65	7,62
CAPN7	7,34	7,11	7,54
CAPNS1	11,08	10,93	11,23
CARD14	4,51	3,56	2,58
CARD6	7,16	7,41	6,51
CARD8	7,59	7,69	8,67
CARD9	11,75	9,47	11,36
CASC3	7,83	7,47	8,61
CASC4	8,90	8,84	9,33
CASC5	2,28	4,64	3,11
CASD1	6,47	6,85	6,95
CASK	5,71	6,21	6,23
CASKIN1	4,77	5,15	4,99
CASKIN2	4,35	4,55	3,42
CASP1	8,64	8,86	11,29
CASP10	4,83	4,99	5,34
CASP2	8,21	7,56	8,39
CASP3	10,06	8,71	8,14
CASP4	8,34	8,77	10,26
CASP7	7,89	6,64	6,64
CASP8	4,38	4,79	6,88
CASP8AP2	6,81	6,47	6,97
CASP9	7,19	7,14	6,89
CAT	11,21	10,84	11,29
CATSPER1	7,47	7,62	8,68
CATSPER2	5,06	5,22	3,78
CAV1	3,26	3,75	3,74
CBARA1	7,81	8,78	9,13
CBFA2T2	5,87	5,50	6,12
CBFA2T3	5,60	4,49	7,55
CBFB	11,07	10,90	11,06
CBL	8,64	8,54	8,98
CBLB	8,68	8,80	7,26
CBLL1	6,22	6,05	7,74
CBLN2	3,41	3,33	3,54
CBR3	9,81	8,57	2,74
CBR4	7,86	8,07	8,05
CBS	10,30	9,80	3,81
CBX1	7,43	7,45	8,29
CBX2	3,45	3,08	3,03
CBX3	7,29	7,72	8,08
CBX4	8,14	7,62	10,07
CBX5	6,99	7,25	6,90
CBX6	11,38	10,99	10,37
CBX7	7,20	7,14	7,90
CBX8	5,42	5,46	5,09

CC2D1A	6,00	5,79	6,07
CC2D1B	5,18	4,52	5,13
CCAR1	10,90	10,31	10,27
CCBL1	5,58	5,90	4,98
CCDC12	9,69	9,80	10,00
CCDC14	8,58	9,23	9,45
CCDC15	4,27	4,84	3,69
CCDC16	7,02	7,04	6,65
CCDC18	5,42	5,82	5,76
CCDC19	5,80	6,18	7,26
CCDC22	7,03	7,35	7,46
CCDC23	10,10	10,15	11,46
CCDC24	5,29	5,60	6,10
CCDC25	7,31	8,01	7,89
CCDC26	5,73	-0,65	3,95
CCDC28A	10,03	9,82	9,93
CCDC3	4,46	4,01	4,23
CCDC32	9,97	9,55	8,85
CCDC34	5,40	6,95	4,10
CCDC4	3,70	3,36	3,69
CCDC41	6,74	6,74	6,68
CCDC42	3,95	4,52	4,38
CCDC43	8,66	8,54	8,37
CCDC45	7,80	7,12	8,96
CCDC47	8,48	8,07	7,59
CCDC49	7,29	6,92	8,45
CCDC5	6,61	6,77	6,85
CCDC50	4,08	4,24	2,79
CCDC51	6,93	7,03	5,64
CCDC53	10,13	10,16	10,11
CCDC55	7,58	7,60	7,54
CCDC56	4,28	4,43	4,34
CCDC58	6,86	7,65	6,84
CCDC59	9,37	9,29	10,88
CCDC6	11,76	10,65	10,75
CCDC60	2,65	3,86	3,49
CCDC64	6,72	5,54	-0,43
CCDC66	6,82	6,06	6,44
CCDC69	8,25	7,33	9,65
CCDC72	11,80	11,86	12,21
CCDC76	6,88	6,33	7,11
CCDC8	4,06	4,40	4,27
CCDC81	5,04	4,42	4,17
CCDC82	5,04	4,83	4,97
CCDC84	7,21	6,74	8,13
CCDC85B	7,02	7,10	7,21
CCDC9	3,18	3,10	4,73
CCDC92	10,53	10,06	8,64
CCDC95	6,89	7,17	7,92
CCDC97	8,07	8,11	8,57
CCDC98	5,73	6,06	7,35
CCDC99	6,33	6,83	5,04
CCHCR1	3,68	4,33	4,60
CCL1	0,01	4,06	3,57
CCL13	14,43	8,02	1,85
CCL17	14,29	5,87	-3,32
CCL2	9,92	9,72	3,29
CCL20	4,83	7,81	3,85
CCL22	13,82	12,80	4,02

CCL24	6,49	7,27	0,25
CCL27	4,65	4,39	4,71
CCL3	11,73	12,67	6,08
CCL3L1	10,22	11,31	5,34
CCL7	6,96	7,70	6,88
CCM2	9,26	9,18	9,48
CCNA1	8,94	8,41	-3,32
CCNA2	4,51	7,82	4,12
CCNB1	3,75	6,36	0,24
CCNB2	6,33	8,70	2,70
CCNC	8,48	8,81	8,33
CCND1	9,96	10,51	-3,32
CCND2	10,48	10,54	7,99
CCND3	11,69	12,72	12,80
CCNDBP1	10,28	10,04	10,52
CCNG1	10,72	10,48	10,52
CCNG2	7,51	5,47	6,90
CCNH	9,96	8,21	7,90
CCNI	13,59	13,03	13,71
CCNJ	5,71	5,01	6,50
CCNK	10,27	9,99	10,55
CCNL1	7,19	7,46	9,08
CCNT1	5,66	5,24	6,13
CCNT2	6,92	6,72	7,67
CCPG1	9,21	9,62	9,71
CCR1	10,79	9,96	8,23
CCR10	2,34	3,79	3,78
CCR2	1,45	-1,01	9,70
CCR7	8,50	-3,32	-3,32
CCR9	4,83	5,25	5,30
CCRN4L	7,39	5,38	7,46
CCS	6,32	6,30	7,28
CCT3	9,58	9,69	9,62
CCT4	6,87	6,71	6,55
CCT5	7,23	7,17	7,29
CCT6A	10,07	9,73	9,52
CCT6B	6,95	7,21	6,79
CCT7	9,55	9,55	9,17
CCT8	11,95	11,87	11,96
CD109	6,69	7,32	2,23
CD14	6,71	12,05	13,50
CD151	7,98	8,59	6,61
CD163	4,98	10,27	11,22
CD163L1	4,70	4,71	4,28
CD164	10,53	10,92	10,74
CD180	4,75	4,82	3,38
CD19	4,02	3,86	4,67
CD1A	12,90	7,14	7,71
CD1B	13,51	9,29	5,13
CD1C	11,94	6,28	8,14
CD1E	7,43	4,18	3,05
CD2	6,94	5,14	6,78
CD200	4,29	4,52	4,07
CD200R1	9,15	3,49	3,23
CD209	12,95	9,60	5,52
CD24	3,43	4,24	4,10
CD244	-0,49	3,67	7,70
CD274	7,07	5,98	0,50
CD276	7,00	6,49	3,34

CD2AP	4,99	6,91	6,34
CD2BP2	9,53	9,42	8,67
CD300A	8,00	8,74	10,63
CD300E	2,98	3,62	5,68
CD300LB	5,40	5,66	8,71
CD300LF	9,82	10,86	10,99
CD302	8,88	9,07	11,73
CD33	9,80	10,15	11,50
CD36	9,28	10,33	12,03
CD37	8,79	10,72	12,29
CD38	7,39	4,00	6,60
CD3EAP	4,46	-0,01	3,78
CD4	8,72	8,94	10,16
CD40	9,52	9,25	5,81
CD44	13,02	12,95	12,80
CD46	11,03	11,16	11,33
CD47	9,51	9,50	10,24
CD48	10,66	11,66	12,86
CD52	7,98	8,35	7,68
CD53	11,37	11,25	11,14
CD58	10,19	10,62	9,92
CD59	8,35	8,47	3,00
CD6	4,17	4,97	-3,32
CD63	12,27	12,96	10,14
CD68	14,36	14,17	13,74
CD69	3,79	4,73	4,85
CD74	13,48	13,29	13,08
CD80	8,32	7,49	5,28
CD81	14,53	14,54	11,85
CD82	7,13	8,75	6,18
CD84	10,19	10,82	7,58
CD86	11,56	10,53	10,85
CD9	12,82	13,70	8,63
CD93	9,62	5,97	13,24
CD97	10,04	10,97	12,10
CD99	11,44	11,28	11,55
CD99L2	8,87	8,93	8,30
CDA	8,67	9,80	10,39
CDADC1	2,87	3,50	4,58
CDAN1	5,96	5,62	5,61
CDC14A	4,04	3,98	6,40
CDC2	-3,32	7,83	-3,32
CDC20	5,29	8,39	3,83
CDC23	9,69	9,45	9,10
CDC25B	8,05	7,93	7,54
CDC25C	-3,32	4,32	-0,41
CDC26	8,85	9,11	9,02
CDC27	5,35	5,37	5,53
CDC2L1	6,68	6,36	7,45
CDC2L2	6,26	6,02	7,03
CDC2L5	8,42	8,10	8,53
CDC2L6	10,19	9,99	10,71
CDC37	12,86	13,10	12,57
CDC37L1	9,54	8,97	7,98
CDC40	9,61	9,86	10,32
CDC42BPA	4,49	4,73	3,57
CDC42BPB	7,96	7,45	6,29
CDC42EP1	6,61	6,42	6,45
CDC42EP2	3,36	1,92	6,04

CDC42EP3	6,33	7,27	8,44
CDC42EP5	2,62	4,18	-3,32
CDC42SE1	7,73	8,18	8,77
CDC42SE2	8,32	7,82	7,86
CDC45L	-3,32	4,72	-1,49
CDC5L	9,19	8,96	8,58
CDC7	5,10	6,15	6,66
CDC73	4,93	5,24	4,59
CDCA2	2,08	5,41	-0,36
CDCA3	1,57	6,16	-1,69
CDCA4	6,90	7,14	7,06
CDCA5	5,19	7,95	4,18
CDCA7L	8,86	9,89	6,48
CDCA8	3,09	5,71	-2,04
CDCP1	6,09	4,43	4,21
CDGAP	7,65	8,02	7,13
CDH1	5,14	4,58	3,90
CDH15	3,88	3,90	3,62
CDH2	7,78	3,15	3,21
CDH23	7,79	8,01	6,58
CDH26	3,04	3,75	4,48
CDH7	2,98	3,07	3,30
CDIPT	6,64	7,06	6,54
CDK2	6,47	6,70	6,60
CDK2AP1	12,51	12,28	11,94
CDK2AP2	7,54	7,32	7,44
CDK4	10,39	10,32	8,65
CDK5	9,78	10,29	8,93
CDK5R1	3,81	4,54	7,06
CDK5RAP1	8,11	7,95	8,17
CDK5RAP2	7,29	8,98	7,49
CDK6	8,35	7,98	7,88
CDK7	9,15	9,27	8,74
CDK8	5,83	5,92	6,11
CDKAL1	6,29	5,88	5,90
CDKL1	2,46	2,75	2,05
CDKL3	4,52	5,36	3,16
CDKN1A	13,24	12,26	10,82
CDKN1B	8,23	7,37	10,02
CDKN1C	-1,24	4,07	8,39
CDKN2A	2,34	3,63	2,66
CDKN2C	3,39	4,59	5,14
CDKN2D	4,20	5,38	9,75
CDKN3	2,66	7,43	3,80
CDR2	10,50	8,52	7,38
CDR2L	5,98	3,86	3,34
CDS1	7,63	7,72	5,01
CDS2	10,05	9,26	8,32
CDV3	9,92	9,92	10,73
CDY2B	3,76	3,85	3,30
CDYL	5,99	5,88	5,35
CDYL2	3,72	0,47	2,94
CEACAM1	2,68	0,50	3,57
CEACAM16	4,09	2,92	4,19
CEACAM21	2,75	4,95	5,03
CEACAM3	-3,32	-3,32	4,45
CEACAM4	5,01	5,03	6,74
CEACAM8	2,06	5,61	-3,32
CEBPA	12,80	12,18	11,54

CEBPG	9,00	8,51	9,44
CEBPZ	10,45	10,04	10,51
CECR5	10,15	10,79	10,72
CECR6	6,81	7,66	7,44
CEECAM1	5,50	4,94	4,31
CELSR1	1,91	3,99	-0,28
CELSR2	7,39	4,02	5,47
CELSR3	5,38	4,11	5,64
CENPA	-3,32	6,40	-3,32
CENPC1	6,51	6,74	6,83
CENPE	4,89	6,77	4,34
CENPF	-2,62	5,60	-0,44
CENPH	5,36	5,75	3,37
CENPJ	6,34	6,86	6,54
CENTA2	10,89	10,72	10,83
CENTB2	7,87	8,45	9,38
CENTD2	7,52	7,76	8,23
CENTG1	3,63	4,17	3,11
CENTG3	9,74	9,72	9,08
CEP135	7,79	7,90	8,51
CEP152	5,09	6,04	6,81
CEP192	6,86	6,82	7,92
CEP250	4,49	3,70	3,56
CEP27	8,99	9,07	10,32
CEP290	5,54	6,24	6,54
CEP350	10,16	9,35	10,83
CEP55	2,88	8,00	-3,32
CEP57	7,21	6,78	7,69
CEP63	9,05	9,02	9,53
CEP68	6,06	5,00	4,79
CEP70	4,24	7,14	2,89
CEP76	6,03	6,13	5,44
CEPT1	9,32	9,17	9,05
CER1	3,68	0,39	4,22
CERKL	-0,33	2,10	3,97
CES1	5,52	10,32	7,63
CES2	8,78	8,80	9,28
CES7	3,29	4,06	2,94
CETN2	10,17	11,03	10,26
CETN3	5,68	5,31	3,51
CFDP1	8,83	8,80	8,40
CFH	5,33	5,95	4,99
CFL1	13,33	13,28	13,10
CFL2	5,10	4,79	4,96
CFLAR	10,48	10,28	10,97
CFP	5,80	3,84	10,87
CGGBP1	10,05	9,80	10,59
CGI-09	7,00	6,57	7,25
CGN	5,07	4,75	4,72
CGNL1	8,54	-0,14	-1,42
CGRRF1	8,22	8,39	8,52
CH25H	10,86	7,36	1,69
CHAC2	4,67	4,93	3,31
CHAD	3,14	3,83	3,11
CHAF1A	5,29	6,25	6,19
CHCHD1	8,47	8,25	7,58
CHCHD3	9,07	8,73	8,65
CHCHD4	9,19	8,71	9,33
CHCHD5	8,40	8,64	8,67

CHCHD6	6,22	8,19	4,26
CHCHD7	10,67	8,73	9,39
CHCHD8	6,75	6,76	6,85
CHD1	9,19	8,45	10,68
CHD1L	8,98	9,27	7,80
CHD3	4,35	4,32	5,13
CHD6	5,64	5,74	6,71
CHD7	6,40	4,51	4,51
CHD8	9,81	9,45	10,27
CHDH	5,02	5,27	-1,46
CHEK1	5,24	6,49	-1,90
CHEK2	3,64	3,72	3,07
CHERP	6,09	5,27	6,77
CHFR	10,99	10,50	10,88
CHI3L1	3,36	5,41	3,98
CHIC2	10,29	9,91	9,91
CHKB	7,46	6,64	8,75
CHM	8,21	8,52	7,69
CHML	1,98	0,48	5,72
CHMP1B	10,39	10,60	11,10
CHMP2A	9,78	10,12	9,99
CHMP2B	10,74	10,59	10,39
CHMP4A	9,00	8,47	8,73
CHMP4B	9,93	10,09	10,49
CHMP4C	3,68	3,38	-0,13
CHMP5	10,35	10,23	9,40
CHMP6	7,14	7,30	7,50
CHN2	7,19	7,09	8,48
CHORDC1	7,91	7,51	7,69
CHP	11,69	11,28	11,83
CHPT1	8,35	9,70	10,25
CHRAC1	6,23	6,16	7,71
CHRD	2,35	4,67	2,47
CHRM4	3,81	3,88	3,36
CHRNA3	3,35	4,42	1,46
CHRNA5	3,46	3,89	3,83
CHRNB1	5,73	5,59	6,79
CHRNB2	4,98	5,09	4,02
CHRNE	4,52	4,81	3,58
CHST1	4,73	4,55	4,88
CHST10	4,20	4,19	2,97
CHST2	4,60	4,79	6,06
CHST3	5,34	3,71	3,62
CHST7	10,20	8,82	9,22
CHST8	4,29	4,04	3,67
CHSY1	9,80	9,94	10,75
CHUK	9,35	9,09	8,95
CHURC1	10,26	10,37	10,64
CHX10	4,61	5,03	4,58
CIAPIN1	7,90	7,61	7,08
CIB1	10,43	10,35	10,40
CIC	7,42	6,71	7,93
CIDEB	5,81	5,04	9,44
CIDEC	3,85	4,50	3,44
CIITA	8,16	7,14	7,80
CINP	6,36	6,53	6,25
CIP29	10,78	10,69	10,68
CIR	10,08	12,04	9,28
CIRBP	11,34	10,58	11,40

CIRH1A	9,26	9,12	8,82
CISH	10,21	8,76	6,57
CIT	3,69	5,87	3,62
CITED1	3,94	4,24	4,43
CITED2	11,06	11,27	10,16
CITED4	4,49	5,53	8,02
CIZ1	4,76	4,70	5,46
CKAP2	6,38	6,65	6,20
CKAP2L	-3,32	6,72	-3,32
CKAP4	7,27	10,49	11,24
CKAP5	9,29	9,45	8,31
CKB	9,30	3,31	4,93
CKLF	12,14	12,53	12,28
CKM	4,66	5,11	5,92
CKS1B	8,88	9,74	7,22
CKS2	8,28	8,63	6,41
CLASP1	8,28	7,98	8,70
CLASP2	8,38	8,23	7,97
CLC	6,81	1,98	-0,74
CLCN1	3,79	3,43	3,71
CLCN2	4,16	4,60	4,27
CLCN3	7,73	8,22	6,37
CLCN5	5,70	5,49	4,98
CLCN6	7,51	7,14	7,75
CLCN7	12,79	12,50	12,01
CLDN1	5,89	-3,32	-3,32
CLDN12	8,32	9,11	4,53
CLDN23	10,72	11,37	7,28
CLDN5	5,07	5,13	4,47
CLDN7	4,14	6,89	5,12
CLDN9	3,38	3,84	4,10
CLDND1	9,42	9,54	9,98
CLDND2	4,55	3,54	5,38
CLEC10A	8,84	3,96	6,49
CLEC11A	7,66	8,04	6,72
CLEC12A	7,93	10,65	11,41
CLEC14A	3,84	2,95	4,44
CLEC1B	-3,32	4,11	1,04
CLEC2B	4,71	5,86	8,28
CLEC4A	12,50	11,33	11,76
CLEC4D	4,61	7,26	9,60
CLEC4E	-3,32	4,81	7,10
CLEC5A	4,24	9,76	7,91
CLEC6A	6,24	9,05	6,65
CLEC7A	8,12	8,13	9,70
CLIC1	7,72	8,07	8,25
CLIC2	8,75	6,89	5,52
CLIC3	5,62	5,73	-1,11
CLIC6	4,67	4,46	-0,48
CLK1	8,72	8,81	9,98
CLK2	6,60	6,05	7,23
CLK4	5,82	5,37	6,82
CLN3	8,75	9,16	8,92
CLN6	6,58	6,97	6,59
CLN8	6,74	7,14	5,70
CLNS1A	11,08	10,47	11,34
CLPTM1	9,41	9,87	9,02
CLPX	8,60	8,50	8,65
CLSTN1	6,13	5,90	5,87

CLTC	9,29	9,29	8,78
CLTCL1	4,29	3,57	5,82
CLUAP1	5,81	5,39	4,63
CLUL1	2,96	3,11	2,97
CLYBL	8,43	8,16	5,54
CMIP	9,57	9,10	10,54
CMKLR1	4,94	5,84	5,07
CMPK	11,21	11,13	11,41
CMTM2	3,69	4,82	7,92
CMTM3	9,67	10,12	10,34
CMTM4	6,16	5,26	6,37
CMTM6	11,85	11,71	11,80
CMTM7	12,09	11,47	11,79
CMTM8	8,60	7,06	6,62
CNDP2	12,70	12,45	12,03
CNFN	-0,69	0,27	5,40
CNIH	10,04	10,44	10,06
CNIH2	3,44	3,07	2,26
CNIH3	8,61	8,05	1,89
CNNM2	3,76	2,74	5,10
CNNM3	7,32	7,00	8,06
CNO	9,64	9,16	9,30
CNOT1	9,38	8,95	9,83
CNOT10	8,99	8,58	9,26
CNOT2	9,59	9,68	10,39
CNOT3	7,11	6,51	7,46
CNOT4	7,85	7,45	7,15
CNOT7	9,22	9,22	9,51
CNOT8	8,63	8,51	10,02
CNP	-0,85	1,21	3,88
CNTFR	3,06	3,55	3,21
CNTNAP1	3,47	1,57	3,28
CNTNAP5	4,30	3,52	3,94
CNTROB	5,41	5,71	5,60
COASY	10,47	10,55	10,54
COBL	7,09	6,38	-3,32
COBLL1	4,34	0,60	3,03
COCH	7,05	1,94	5,92
COG1	5,51	5,81	6,33
COG2	8,06	8,20	8,71
COG3	7,40	7,42	7,94
COG4	7,02	7,12	7,56
COG5	6,23	6,90	6,31
COG7	5,82	5,28	5,58
COG8	5,72	5,45	4,82
COIL	8,30	8,26	8,81
COL11A1	3,15	3,84	3,87
COL15A1	4,80	6,07	-1,01
COL16A1	4,51	4,13	3,76
COL22A1	5,87	11,74	-3,32
COL23A1	4,04	3,92	2,98
COL24A1	0,98	3,92	0,84
COL25A1	3,13	3,14	3,84
COL27A1	3,01	-0,32	3,82
COL4A1	1,70	3,72	3,44
COL4A2	4,84	4,68	4,02
COL4A3BP	9,74	10,66	9,58
COL5A1	4,01	3,88	4,55
COL6A1	5,37	6,13	-0,50

COL6A3	4,39	4,83	-1,72
COL7A1	4,26	3,59	4,16
COL9A1	3,12	3,69	2,95
COL9A2	7,36	7,60	7,33
COLEC12	8,24	10,44	6,09
COLQ	3,24	4,96	4,30
COMMD1	10,90	10,84	10,19
COMMD10	9,92	10,04	10,01
COMMD2	9,83	9,17	8,91
COMMD3	11,92	11,82	11,74
COMMD5	4,83	4,52	6,15
COMMD8	9,30	9,73	10,04
COMMD9	9,71	10,41	10,48
COMP	3,98	3,60	3,25
COMT	10,79	11,32	10,56
COMTD1	7,02	6,33	6,77
COP1	4,85	6,26	8,95
COPA	11,07	11,09	11,16
COPB2	10,11	10,29	9,62
COPE	10,48	10,75	10,87
COPG	9,67	10,20	9,38
COPG2	6,26	6,77	7,24
COPS2	8,71	8,33	9,10
COPS3	10,72	10,76	11,09
COPS4	8,79	9,05	9,15
COPS5	10,86	11,05	11,04
COPS6	7,70	8,36	8,39
COPS7A	10,69	10,89	10,56
COPS7B	9,09	8,44	8,74
COPS8	6,79	7,03	6,42
COPZ1	9,47	10,17	9,74
COPZ2	-2,43	4,53	4,37
COQ10A	6,69	6,54	6,47
COQ10B	10,68	10,86	10,21
COQ2	9,61	11,08	11,19
COQ3	7,00	8,12	6,22
COQ4	7,81	7,67	7,84
COQ5	10,15	10,18	9,65
COQ6	6,52	6,69	6,05
COQ7	6,06	6,08	4,86
COQ9	9,55	9,58	8,92
CORIN	3,94	2,80	2,47
CORO1C	11,87	12,48	11,55
CORO2A	7,93	8,12	6,44
CORO7	9,08	8,88	9,44
COX10	7,19	7,23	7,10
COX11	4,11	4,38	3,27
COX15	7,88	8,02	7,29
COX17	11,19	11,49	10,43
COX4I1	14,01	14,11	13,68
COX4NB	7,38	7,23	7,89
COX5B	13,12	13,71	12,20
COX6A1	11,49	11,60	10,85
COX6B1	13,03	13,24	12,95
COX6C	12,27	12,67	11,98
COX7A2	13,25	13,52	12,46
COX7A2L	11,19	11,51	11,77
COX7B	12,26	12,19	11,63
COX7C	13,73	13,74	13,35

COX8A	13,28	13,39	12,56
CP110	8,44	8,43	9,56
CPA6	4,36	2,48	4,22
CPAMD8	4,20	4,93	7,47
CPD	8,44	10,12	9,82
CPE	-0,16	7,09	-0,84
CPEB1	7,02	5,04	-1,48
CPEB3	7,17	6,32	8,57
CPEB4	8,23	8,57	7,81
CPLX2	3,14	2,98	3,43
CPLX3	2,71	3,80	4,02
CPM	5,14	6,09	4,84
CPNE1	9,12	8,13	9,44
CPNE2	4,53	4,64	5,58
CPNE6	1,84	5,37	1,82
CPNE8	8,14	8,33	9,44
CPNE9	6,81	7,46	5,42
CPOX	9,87	10,31	9,66
CPSF1	8,75	8,58	8,64
CPSF2	7,36	7,14	7,71
CPSF3	10,14	10,35	9,66
CPSF3L	7,19	6,72	7,98
CPSF4	10,48	10,37	10,01
CPSF6	5,29	5,00	5,94
CPT1A	4,99	4,04	5,91
CPT1B	4,35	3,98	5,44
CPT1C	4,23	4,11	3,83
CPVL	10,78	10,91	12,97
CPXM2	2,68	3,95	3,48
CPZ	3,14	3,40	2,93
CRABP2	9,84	12,94	1,95
CRADD	7,42	8,27	7,27
CRAMP1L	6,53	5,89	6,69
CRAT	7,56	7,61	7,67
CREB1	7,45	7,67	7,85
CREB3	6,90	6,61	6,07
CREB3L3	5,03	4,30	4,52
CREB3L4	4,40	3,24	4,55
CREBBP	9,15	8,31	10,44
CREBL1	6,25	6,05	6,91
CREBL2	8,96	7,27	7,39
CREG1	12,93	12,94	11,57
CRELD1	6,52	6,87	6,40
CREM	5,68	5,41	5,51
CRH	8,57	-3,32	-3,32
CRHBP	3,71	4,21	2,69
CRHR2	4,07	3,38	3,21
CRIM1	5,08	6,40	4,21
CRIP1	10,80	10,17	12,39
CRIP3	3,78	3,92	3,96
CRIPT	8,25	9,14	8,11
CRISPLD2	5,06	5,24	11,52
CRK	7,85	7,05	6,86
CRKRS	7,12	6,81	7,70
CRLF1	3,49	3,74	4,17
CRLF3	6,96	7,04	8,58
CRLS1	10,34	11,02	10,67
CRNKL1	5,81	5,90	5,04
CROP	6,44	6,52	8,82

CROT	5,72	6,26	5,22
CRSP2	4,04	3,55	3,89
CRSP3	7,21	7,09	7,80
CRSP6	4,45	4,33	-5,44
CRSP8	7,02	7,27	6,65
CRSP9	8,22	8,24	7,25
CRTAP	7,73	8,07	9,79
CRTC1	5,40	5,31	6,01
CRTC2	6,48	6,02	7,95
CRX	3,43	3,88	4,18
CRY1	7,54	7,65	7,29
CRY2	7,63	6,80	7,72
CRYBB2	4,00	4,43	4,47
CRYGS	5,76	5,02	6,49
CRYZ	11,02	9,42	8,87
CRYZL1	7,32	7,45	7,78
CS	11,23	11,02	11,10
CSAD	3,60	4,22	4,82
CSDA	12,06	12,23	12,85
CSDE1	7,25	7,17	8,00
CSE1L	9,45	9,13	9,07
CSF1	7,31	9,17	-1,94
CSF2RA	9,75	9,15	10,49
CSF3R	5,78	7,24	9,83
CSGLCA-T	7,02	6,77	7,17
CSK	11,19	10,48	12,00
CSMD1	4,76	4,91	5,01
CSNK1A1	7,61	7,79	8,27
CSNK1G1	7,42	7,13	7,19
CSNK2A1	8,35	8,18	7,62
CSNK2A2	8,74	8,91	8,03
CSNK2B	11,72	11,49	11,20
CSPG4	3,41	3,99	3,52
CSPG5	4,42	5,09	4,73
CSPP1	3,86	3,50	4,83
CSRP1	10,78	11,01	9,94
CSRP2	4,46	8,84	-0,38
CST6	5,20	6,59	3,96
CSTA	6,23	7,50	9,30
CSTF1	3,93	3,84	3,31
CSTF2	8,19	8,20	7,68
CSTF2T	6,49	6,07	6,15
CSTF3	8,80	8,65	8,02
CTAGE5	3,24	3,45	4,39
CTBP2	4,46	4,53	5,81
CTBS	9,02	9,78	10,13
CTCF	9,27	9,04	9,43
CTDP1	6,44	5,58	7,18
CTDSP1	8,69	8,22	10,28
CTDSP2	10,17	10,81	11,95
CTDSPL2	7,06	6,72	7,25
CTH	0,60	4,84	-0,42
CTLA4	9,15	5,59	5,76
CTNNA1	12,13	11,65	11,61
CTNNAL1	11,79	9,32	1,65
CTNNB1	7,87	7,25	7,41
CTNNBIP1	6,27	6,47	8,33
CTNNBL1	9,88	9,95	10,31
CTNS	10,57	9,92	7,60

CTPS2	5,74	5,91	6,19
CTRB2	3,95	3,81	3,17
CTSB	12,90	13,02	11,26
CTSC	12,85	11,25	9,84
CTSD	13,57	14,00	12,03
CTSF	3,91	5,33	4,75
CTSG	3,16	8,25	5,51
CTSK	7,69	11,74	8,39
CTSL2	8,16	8,04	0,04
CTSS	10,76	11,88	12,60
CTSZ	14,08	13,39	11,58
CTTN	7,49	7,93	2,74
CTTNBP2NL	6,81	6,16	4,81
CUEDC1	10,44	9,82	9,81
CUEDC2	8,66	8,88	9,39
CUGBP1	6,70	7,11	7,00
CUGBP2	7,48	6,76	9,03
CUL1	9,57	9,17	9,63
CUL2	9,77	10,34	8,99
CUL4A	8,23	8,27	8,52
CUL4B	5,44	5,90	6,79
CUTA	10,36	10,71	10,11
CUTC	8,06	8,05	8,86
CUTL1	4,98	4,97	5,81
CUTL2	3,63	-2,33	-0,51
CWF19L1	7,35	7,49	8,11
CX3CL1	3,69	0,26	-3,32
CX3CR1	4,16	3,54	8,97
CXCL1	5,97	8,95	6,93
CXCL10	3,73	4,73	7,88
CXCL14	5,89	5,39	6,15
CXCL2	4,54	9,52	3,95
CXCL3	4,63	4,96	4,98
CXCL5	-1,25	6,34	3,47
CXCR4	11,57	10,62	11,67
CXXC1	9,33	9,00	9,20
CXXC5	11,19	11,12	9,86
CXORF21	6,15	5,92	6,86
CXORF23	6,43	5,99	6,57
CXORF26	10,96	11,24	9,43
CXORF34	5,06	4,98	6,05
CXORF38	8,46	8,61	9,41
CXORF39	9,18	8,34	7,56
CXORF40A	5,37	5,29	4,14
CXORF42	3,99	4,09	4,43
CXORF45	5,41	5,65	7,01
CXORF48	3,63	3,48	2,74
CXORF56	6,73	6,85	6,61
CXORF6	3,55	6,02	-1,44
CXORF9	10,90	9,93	10,67
CYB561	4,95	4,98	5,35
CYB561D1	7,26	7,14	7,10
CYB561D2	8,41	8,46	8,81
CYB5A	10,24	10,88	8,04
CYB5B	11,52	11,59	10,72
CYB5D1	6,58	6,81	6,13
CYB5D2	7,31	7,62	6,91
CYB5R1	10,50	10,74	9,37
CYB5R2	2,67	6,28	4,67

CYB5R3	8,70	8,24	8,24
CYB5R4	10,01	10,01	10,58
CYBASC3	9,76	10,82	9,25
CYBB	9,98	13,02	12,77
CYBRD1	10,38	9,76	10,03
CYCS	7,35	6,99	6,10
CYFIP1	9,27	9,26	8,31
CYFIP2	8,79	7,78	10,25
CYGB	7,36	7,10	4,98
CYP11A1	4,64	4,27	5,03
CYP19A1	3,73	4,45	2,73
CYP1B1	12,63	12,90	11,14
CYP20A1	8,50	8,05	8,07
CYP26A1	2,22	2,79	2,40
CYP26C1	3,91	2,91	0,03
CYP27A1	11,92	13,58	10,91
CYP27B1	8,96	10,28	1,99
CYP2A13	3,66	3,76	4,47
CYP2S1	7,72	8,17	8,32
CYP2U1	2,58	2,85	5,84
CYP39A1	4,72	4,11	4,33
CYP3A5	1,55	4,43	2,51
CYP4V2	8,50	8,99	6,88
CYP4X1	3,02	4,28	3,61
CYP51A1	11,24	10,44	8,24
CYSLTR1	7,31	8,48	9,03
CYYR1	6,97	4,37	-1,64
CYORF15A	3,87	3,65	5,15
D15WSU75E	9,87	10,00	9,22
DAAM1	7,40	5,42	7,37
DAB2	12,38	12,98	6,97
DACH1	2,41	2,15	5,89
DACT1	8,42	4,74	-3,32
DAD1	12,44	12,73	11,75
DAK	6,40	6,75	6,72
DAP	12,36	11,94	11,79
DAP3	9,95	9,92	10,46
DAPK2	4,29	4,65	4,23
DAPK3	6,08	5,36	6,74
DAPP1	6,79	6,32	8,49
DARS	12,29	11,91	11,45
DARS2	8,27	8,56	6,53
DAXX	7,57	7,17	7,14
DAZAP2	12,96	12,65	13,73
DBF4B	2,98	3,53	3,18
DBI	14,06	14,46	12,24
DBN1	4,13	5,88	3,69
DBNDD2	11,64	10,71	10,25
DBNL	11,66	11,37	12,00
DBP	7,24	7,20	7,45
DBR1	6,89	7,01	6,00
DBT	7,79	7,67	6,77
DC2	11,05	11,42	11,04
DCAKD	8,95	9,02	8,87
DCBLD1	6,54	5,40	5,05
DCBLD2	4,82	6,01	3,78
DCD	4,44	4,20	4,30
DCDC2	3,44	3,71	-0,94
DCHS2	2,48	3,17	-1,16

DCK	9,65	9,57	9,27
DCLRE1A	6,34	6,76	4,71
DCLRE1B	6,91	6,58	5,89
DCLRE1C	7,29	6,98	8,10
DCP1A	7,85	7,60	7,80
DCP1B	5,30	5,18	4,90
DCP2	11,10	9,95	10,71
DCPS	5,53	5,54	7,01
DCTD	9,87	9,95	9,71
DCTN1	5,99	5,78	6,20
DCTN2	11,42	11,47	11,36
DCTN3	8,29	8,58	9,27
DCTN4	9,18	9,02	9,15
DCTN5	9,96	9,94	9,72
DCTN6	9,34	9,77	8,73
DCUN1D2	4,82	3,84	4,03
DCUN1D3	10,11	8,80	6,46
DCUN1D4	5,84	5,74	6,68
DCUN1D5	9,25	9,57	8,56
DCX	2,96	3,32	3,09
DCXR	9,94	9,61	8,76
DDAH2	8,45	7,52	7,52
DDB1	10,33	10,50	10,23
DDB2	7,16	6,92	6,04
DDC	4,01	4,12	3,56
DDEF2	7,52	8,22	6,05
DDHD1	4,76	4,63	2,49
DDI2	0,38	3,62	3,47
DDIT3	8,70	9,24	8,30
DDIT4	7,03	6,15	11,30
DDIT4L	6,07	5,95	2,20
DDO	5,48	6,62	3,61
DDOST	11,35	11,64	11,36
DDR1	3,54	4,35	4,67
DDT	12,32	11,64	11,85
DDX10	8,14	8,18	8,73
DDX17	10,66	10,32	11,46
DDX18	9,91	9,58	10,51
DDX19-DDX19L	5,22	5,24	5,32
DDX19A	7,10	7,30	7,35
DDX19B	5,92	6,10	6,07
DDX20	5,83	4,99	4,88
DDX21	10,02	10,08	10,98
DDX23	8,10	7,71	8,70
DDX24	9,47	9,01	8,88
DDX25	2,35	2,82	2,76
DDX27	9,50	9,10	10,26
DDX28	8,63	8,65	9,30
DDX31	5,09	5,00	4,92
DDX39	9,85	9,70	10,40
DDX3X	10,32	10,25	11,21
DDX41	8,88	8,84	9,26
DDX42	8,78	8,35	9,03
DDX46	8,02	8,09	9,37
DDX47	9,99	10,08	9,78
DDX49	5,65	5,57	6,20
DDX5	12,93	12,63	13,16
DDX50	7,67	7,71	8,94
DDX51	5,58	5,26	7,05

DDX52	4,63	4,46	4,54
DDX54	5,65	5,41	6,12
DDX55	8,87	9,02	8,88
DDX56	8,35	8,11	8,87
DDX58	5,52	5,27	6,53
DDX59	6,62	6,99	7,80
DDX6	5,03	4,97	3,86
DEADC1	4,13	4,18	5,01
DEAF1	5,52	4,79	5,74
DECR1	12,13	12,52	12,04
DECR2	6,11	6,83	6,26
DEDD	6,39	6,21	7,00
DEDD2	10,13	10,36	11,71
DEFA4	2,78	3,57	4,01
DEFB108B	4,29	4,44	4,34
DEFB125	5,59	5,12	5,63
DEFB126	4,24	3,96	3,85
DEFB128	4,84	4,91	4,47
DEGS1	11,15	10,94	10,62
DEK	11,91	11,54	12,04
DENND1C	7,55	7,44	7,97
DENND2C	3,83	4,83	3,72
DENND2D	10,12	10,34	8,02
DENND3	5,02	6,12	8,93
DENND4A	2,80	3,00	4,91
DENR	10,57	10,64	10,42
DEPDC1	-3,32	4,87	-0,66
DEPDC5	5,27	4,97	4,73
DEPDC6	5,83	3,94	4,45
DEPDC7	4,22	5,05	3,41
DERA	11,53	11,52	10,90
DERL1	9,81	10,07	9,26
DERL2	8,45	9,02	8,50
DEXI	9,71	10,14	7,28
DFFA	6,67	6,85	6,62
DFFB	6,46	6,32	7,58
DFNA5	11,08	12,06	6,62
DGAT2L3	4,43	4,37	4,79
DGCR14	6,03	5,81	6,71
DGKA	4,44	4,21	5,76
DGKD	4,50	3,71	7,87
DGKE	2,82	3,91	4,40
DGKG	4,11	5,14	6,80
DGKZ	3,00	3,52	4,44
DGUOK	9,74	9,76	9,69
DHCR24	10,54	10,74	4,10
DHCR7	9,97	9,36	4,33
DHDDS	7,69	7,62	6,92
DHDH	7,56	7,00	3,92
DHFR	4,47	5,09	4,71
DHFRL1	7,94	8,01	6,20
DHODH	-0,48	3,19	3,67
DHPS	8,31	8,24	8,93
DHRS1	8,90	9,84	9,59
DHRS3	9,52	9,45	1,93
DHRS4	7,18	9,21	8,29
DHRS4L2	7,36	9,46	8,56
DHRS7	11,61	12,08	12,00
DHRS7B	7,49	8,27	7,87

DHRS8	2,89	3,81	3,89
DHX15	10,49	10,63	10,66
DHX16	9,86	9,69	9,78
DHX29	8,24	8,52	8,11
DHX30	7,68	7,62	7,71
DHX33	7,34	7,30	8,18
DHX34	5,29	5,31	5,32
DHX35	6,78	6,64	6,91
DHX36	8,34	8,65	8,21
DHX37	4,56	3,58	4,27
DHX38	7,05	7,09	7,65
DHX57	1,64	2,19	6,02
DHX8	7,10	6,93	7,07
DHX9	6,35	6,42	6,67
DICER1	7,67	7,60	8,73
DIDO1	5,85	5,49	6,96
DIO1	-0,78	4,98	-1,73
DIO3	3,28	4,02	0,19
DIP2A	5,32	5,38	5,29
DIP2C	6,98	5,39	3,52
DIRAS1	3,03	2,08	0,24
DIRAS2	4,53	4,96	4,72
DIRC2	11,21	10,55	9,59
DISC1	3,92	4,13	5,59
DISP1	5,55	6,10	4,81
DIXDC1	-0,62	6,34	-0,31
DKC1	10,04	9,73	9,89
DKFZP434A0131	6,05	5,93	7,26
DKFZP434B0335	7,23	7,24	8,54
DKFZP564J0863	9,80	9,56	9,18
DKFZP564O0523	5,98	5,23	5,56
DKFZP564O0823	8,56	-0,13	3,39
DKFZP586P0123	4,44	4,42	4,93
DKFZP434K1815	6,35	6,29	6,28
DKFZP451M2119	3,90	5,20	5,02
DKFZP666G057	1,86	-0,15	2,95
DKFZP686I15217	5,99	5,92	5,64
DKFZP686O24166	7,77	7,97	6,19
DKFZP761B107	3,45	4,06	6,40
DKFZP761E198	6,89	6,82	8,07
DKFZP762E1312	3,93	6,31	3,33
DKKL1	4,91	4,86	4,18
DLAT	8,73	8,49	7,53
DLD	10,21	10,43	9,28
DLEU7	0,74	5,96	6,71
DLG1	5,23	5,08	5,65
DLG4	6,51	6,98	9,21
DLG7	-3,32	7,27	-3,32
DLGAP1	2,70	3,12	1,08
DLST	9,08	9,21	8,35
DLX2	2,72	0,47	-2,08
DLX4	2,19	2,36	3,06
DMAP1	8,56	8,47	8,99
DMN	3,73	3,86	4,59
DMPK	4,25	0,74	4,18
DMRT1	6,64	6,61	6,88
DMRTC1	6,02	6,05	6,47
DMTF1	8,18	7,92	9,18
DMWD	6,31	1,56	4,75

DMXL1	7,35	8,16	7,80
DMXL2	7,80	9,05	9,47
DNAH3	7,05	4,46	4,04
DNAH7	4,78	4,08	4,76
DNAI1	4,37	3,56	4,49
DNAJA1	11,88	12,13	11,81
DNAJA2	11,24	11,07	11,65
DNAJA3	9,92	10,02	9,74
DNAJA5	5,52	5,47	5,93
DNAJB11	11,17	11,42	11,10
DNAJB12	6,26	6,30	6,95
DNAJB14	8,42	8,42	8,81
DNAJB2	9,66	9,89	8,44
DNAJB4	6,32	6,54	5,01
DNAJB5	4,71	5,85	3,14
DNAJB6	10,65	10,36	9,66
DNAJB9	9,61	9,43	8,65
DNAJC1	5,80	5,57	6,16
DNAJC10	7,17	7,28	7,87
DNAJC12	6,78	4,80	2,52
DNAJC13	8,30	8,72	8,98
DNAJC14	7,50	7,63	7,95
DNAJC15	8,73	8,72	8,64
DNAJC16	5,03	4,59	4,88
DNAJC17	7,45	7,57	7,36
DNAJC19	6,47	6,44	6,00
DNAJC3	6,86	7,12	7,14
DNAJC5	7,57	7,43	6,82
DNAJC5B	7,87	8,55	2,36
DNAJC7	10,64	10,23	10,83
DNAJC8	11,79	11,92	11,47
DNAJC9	8,85	9,49	8,65
DNAL4	7,85	8,62	8,45
DNASE1L1	5,90	5,78	6,33
DNASE1L3	12,45	4,03	2,87
DNASE2B	6,98	9,89	1,37
DNM2	8,77	8,85	9,20
DNM3	5,33	6,12	4,37
DNMT1	12,09	11,76	11,13
DNTTIP1	9,52	9,64	10,22
DNTTIP2	7,29	6,66	7,53
DOC2A	7,56	6,99	5,91
DOCK11	10,71	10,50	11,80
DOCK2	11,69	11,30	12,61
DOCK3	8,72	8,60	1,32
DOCK4	3,93	3,19	1,99
DOCK5	7,07	7,23	6,79
DOCK6	5,34	5,65	-3,32
DOCK7	10,07	9,36	7,68
DOCK8	10,54	10,14	11,32
DOCK9	3,42	0,21	3,18
DOK2	9,05	8,01	9,44
DOK3	6,47	7,06	7,11
DOLPP1	7,63	7,57	6,83
DOM3Z	6,46	6,21	6,98
DONSON	5,88	6,25	4,97
DOPEY1	5,44	4,93	6,13
DP58	-3,32	0,58	1,31
DPAGT1	8,67	9,13	8,40

DPCR1	5,06	5,12	5,33
DPEP2	8,65	9,28	10,82
DPF1	4,24	3,51	4,05
DPH2	8,08	7,73	7,95
DPH5	7,24	7,79	7,82
DPM1	10,28	10,10	9,64
DPM2	7,03	7,09	6,45
DPM3	9,84	9,79	9,40
DPP3	7,23	7,44	6,90
DPP4	5,16	4,38	1,34
DPP8	7,93	7,95	8,22
DPP9	10,47	9,52	8,55
DPY19L2	-0,57	0,77	4,19
DPY19L3	3,32	4,22	5,19
DPY19L4	7,01	6,79	5,60
DPYS	5,70	5,44	5,32
DPYSL2	13,22	12,75	12,95
DPYSL3	5,28	7,23	-1,53
DPYSL4	3,96	3,21	2,84
DR1	8,56	8,94	9,64
DRAP1	10,86	10,97	11,02
DRD3	4,75	4,63	6,65
DRD4	4,12	4,38	3,94
DRG1	9,14	9,35	9,38
DRG2	7,27	7,35	7,78
DRP2	4,56	4,50	4,84
DSC2	8,54	8,81	8,52
DSCAM	3,52	3,44	3,90
DSCR1	9,89	9,31	9,11
DSCR10	5,17	5,43	4,51
DSCR2	8,96	9,12	8,30
DSCR3	10,25	10,09	9,85
DSCR4	3,80	3,73	2,10
DSCR6	4,32	4,75	3,54
DSP	-1,21	4,23	1,89
DSTN	9,05	9,20	8,18
DTL	1,96	7,46	3,10
DTNA	9,13	7,15	4,69
DTNB	5,81	5,59	4,47
DTNBP1	6,90	6,54	7,38
DTWD1	7,42	7,46	7,58
DTWD2	5,51	6,56	4,27
DTX3L	6,24	6,29	6,78
DULLARD	9,67	9,54	10,02
DUOX1	7,11	-3,32	-3,32
DUS1L	7,43	6,91	7,56
DUS2L	8,90	9,33	9,43
DUS3L	7,23	7,25	8,28
DUS4L	7,12	7,32	6,90
DUSP1	10,68	12,00	14,19
DUSP10	5,67	6,51	6,08
DUSP11	9,34	9,37	8,83
DUSP12	9,81	9,75	10,58
DUSP13	3,93	4,03	4,04
DUSP14	7,12	8,78	3,62
DUSP18	8,27	8,58	8,44
DUSP23	10,98	11,13	10,54
DUSP26	5,47	5,58	5,08
DUSP3	11,90	11,71	11,72

DUSP5	10,64	10,66	8,49
DUSP6	3,67	6,44	8,26
DUSP8	5,51	4,89	6,08
DUSP9	4,25	4,49	4,75
DUT	6,57	6,72	7,29
DVL2	7,59	7,00	6,51
DYNC1I2	10,22	10,33	10,13
DYNC1LI1	7,67	6,69	7,45
DYNC1LI2	10,84	10,55	10,78
DYNLRB1	10,37	10,44	9,77
DYNLT1	10,45	10,32	10,84
DYNLT3	9,47	9,82	8,59
DYRK1A	8,24	7,84	9,19
DYRK2	7,18	7,87	7,17
DYRK3	3,91	4,89	3,04
DYSF	9,43	6,79	10,08
E2F2	5,13	8,88	8,78
E2F3	10,83	10,79	11,30
E2F8	2,06	4,12	-1,21
EAF1	9,26	10,08	9,79
EAF2	5,92	7,64	8,81
EARS2	7,24	7,57	5,53
EBAG9	7,78	7,85	8,08
EBI2	11,19	8,95	10,41
EBI3	5,84	1,91	4,04
EBNA1BP2	9,66	10,43	9,81
EBP	10,27	10,71	8,11
ECD	7,20	7,61	6,99
ECE1	4,76	3,71	5,63
ECE2	6,70	6,93	6,16
ECGF1	7,45	6,99	10,00
ECH1	10,64	10,70	10,89
ECHDC1	6,20	6,74	6,85
ECHDC2	8,71	9,12	7,90
ECHDC3	6,85	6,53	6,42
ECOP	10,26	9,82	8,59
ECT2	4,53	5,05	3,82
EDEM1	10,31	9,99	9,41
EDEM2	8,98	10,19	9,86
EDEM3	4,78	4,68	5,56
EDG1	6,01	4,88	4,61
EDG2	7,62	8,42	7,81
EDG3	4,37	2,52	8,27
EDG6	8,21	8,20	9,51
EDN1	5,36	6,28	3,50
EEA1	5,81	4,42	3,57
EED	6,37	5,97	6,11
EEF1A1	14,71	14,73	14,74
EEF1B2	10,85	10,69	11,24
EEF1D	8,42	8,25	9,56
EEF2	12,44	12,15	12,98
EEF2K	9,78	9,55	7,53
EFCAB2	3,93	4,39	3,10
EFHA1	10,58	10,57	10,53
EFHB	4,46	4,54	4,87
EFHC1	3,42	3,70	6,27
EFHC2	5,52	2,61	2,00
EFHD1	1,54	3,98	3,56
EFHD2	12,59	12,18	13,14

EFNA1	4,76	3,99	3,34
EFNA3	4,36	4,84	4,79
EFNA4	5,71	5,87	6,35
EFNB1	4,56	5,27	5,50
EFTUD1	7,61	8,05	7,66
EFTUD2	9,98	10,01	10,55
EGFL7	4,43	5,53	3,48
EGFR	3,35	3,36	3,31
EGLN1	6,92	6,58	7,12
EGLN3	7,17	2,36	3,34
EGR1	9,14	10,45	10,71
EGR2	12,75	12,82	7,36
EGR4	3,91	2,93	1,82
EHBP1	7,04	7,21	7,82
EHD1	9,25	9,66	11,01
EHD4	11,73	10,59	9,86
EHMT1	4,34	3,65	4,94
EHMT2	6,21	5,22	5,98
EIF1	11,94	12,20	12,88
EIF1AX	9,54	9,45	9,65
EIF1B	11,56	11,00	10,75
EIF2A	10,40	10,59	10,80
EIF2AK1	9,30	9,56	9,42
EIF2AK2	9,52	9,96	10,69
EIF2AK3	7,02	7,13	7,29
EIF2AK4	8,74	8,32	9,98
EIF2B1	8,74	8,89	9,28
EIF2B2	8,76	9,09	8,22
EIF2B3	8,85	9,26	8,46
EIF2B4	9,64	9,23	9,44
EIF2B5	8,99	9,01	8,76
EIF2C1	7,82	8,68	8,31
EIF2C3	6,30	6,51	5,84
EIF2C4	6,48	6,14	7,91
EIF2S1	8,22	7,90	8,09
EIF2S2	4,97	5,48	6,79
EIF2S3	10,43	10,73	11,93
EIF3S7	11,24	10,97	11,77
EIF4A1	14,26	14,38	14,09
EIF4A2	12,71	12,05	12,86
EIF4B	11,37	11,29	12,36
EIF4E2	9,73	9,63	10,56
EIF4E3	7,10	8,00	9,21
EIF4EBP2	10,53	10,47	12,06
EIF4ENIF1	8,46	8,01	8,11
EIF4G1	8,65	8,48	8,33
EIF4G2	12,88	12,86	12,60
EIF5	8,71	9,01	8,39
EIF5A2	4,63	3,28	3,87
EIF5B	8,65	8,59	7,76
ELA2	4,14	3,71	5,51
ELAC1	4,89	3,85	4,06
ELAC2	6,93	6,74	7,05
ELAVL1	4,98	4,29	4,60
ELF1	11,09	11,15	11,66
ELF2	7,56	7,20	8,88
ELF5	4,16	3,86	3,83
ELK1	10,02	9,64	9,40
ELK4	4,00	3,89	5,67

ELL2	7,45	8,67	5,79
ELL3	6,03	4,09	1,92
ELMO1	7,86	7,38	8,06
ELMO2	5,85	6,88	6,33
ELMOD2	7,98	8,07	5,48
ELOVL1	8,54	8,42	8,32
ELOVL3	-3,37	3,87	4,16
ELOVL5	11,30	11,40	10,91
ELOVL6	1,98	4,41	2,29
ELOVL7	4,34	3,01	1,34
ELP3	8,80	9,23	9,23
ELP4	7,06	7,11	6,85
EME1	2,65	4,20	1,65
EME2	5,16	5,63	5,32
EMG1	9,03	8,97	8,23
EMILIN2	12,17	12,18	11,84
EML2	8,99	9,47	9,42
EML3	9,91	9,64	10,25
EML4	11,36	12,27	9,95
EMP1	11,24	12,99	7,02
EMR1	-3,32	0,15	8,67
EMR2	10,32	10,62	11,39
EMR3	1,86	7,15	7,61
EMX2	4,15	3,66	3,70
EN1	4,28	4,24	4,26
ENC1	6,53	7,05	8,24
ENDOG	7,81	7,10	7,37
ENDOGL1	5,12	5,02	4,12
ENO1	13,76	13,60	12,93
ENO2	6,99	8,09	-1,24
ENOSF1	9,28	9,72	6,72
ENPP2	10,68	8,81	4,24
ENPP4	7,98	9,70	6,06
ENSA	8,42	8,32	8,12
ENTPD1	8,89	9,43	9,74
ENTPD5	5,00	4,79	3,93
ENTPD7	6,22	7,28	4,73
ENTPD8	4,47	4,71	4,73
ENY2	11,37	11,47	11,62
EP300	7,54	7,19	8,78
EP400	8,36	8,20	8,98
EPAS1	11,37	11,70	5,52
EPB41L1	6,81	7,46	3,43
EPB41L3	10,97	11,06	12,09
EPB41L4A	3,40	3,86	3,68
EPB42	3,80	4,69	3,42
EPB49	4,75	4,64	4,64
EPC1	6,95	6,86	8,66
EPC2	5,10	4,71	5,34
EPDR1	9,78	9,47	5,11
EPHA2	4,31	3,73	3,40
EPHA3	3,02	3,03	3,77
EPHA5	3,02	3,41	3,64
EPHA8	4,48	4,29	4,08
EPHB1	3,16	4,46	-1,03
EPHB3	3,73	-0,70	3,55
EPHB4	3,64	3,53	4,77
EPHB6	5,63	6,52	7,14
EPM2A	4,91	4,92	4,48

EPM2AIP1	7,30	7,36	7,82
EPN2	6,06	4,08	2,95
EPN3	3,92	3,86	3,77
EPO	4,22	4,48	4,53
EPPB9	-0,81	3,89	-2,25
EPRS	11,21	11,24	10,65
EPS8	7,37	6,13	4,92
EPS8L1	3,49	3,60	2,42
EPSTI1	8,95	9,12	10,33
ERAL1	9,03	8,87	8,62
ERBB2	4,09	3,64	3,70
ERBB2IP	6,03	6,08	6,90
ERBB4	3,85	4,38	-1,14
ERCC1	6,84	7,73	7,40
ERCC2	6,39	6,46	7,15
ERCC4	3,75	4,40	2,88
ERCC5	10,15	9,79	10,59
ERCC6	5,32	4,42	4,71
ERCC8	4,81	5,40	5,18
ERF	6,45	6,25	7,46
ERGIC2	9,04	9,16	7,94
ERGIC3	7,55	7,89	7,24
ERH	11,48	11,50	11,57
ERICH1	8,18	8,11	10,33
ERMAP	5,56	5,57	7,72
ERN1	5,93	6,40	7,20
ERO1L	9,57	9,36	8,06
ERO1LB	3,34	2,34	4,87
ERRFI1	7,60	7,20	4,43
ESCO1	7,09	6,93	6,29
ESCO2	1,49	4,72	-3,32
ESD	11,55	11,74	11,70
ESPL1	3,93	4,84	4,27
ESR1	6,57	6,44	-0,43
ESRRA	8,46	7,88	8,44
ESX1	3,64	3,90	3,99
ETF1	9,33	9,59	9,05
ETFA	11,66	11,84	10,79
ETFB	9,27	9,29	8,79
ETFDH	8,49	9,25	8,26
ETHE1	7,19	6,81	6,38
ETNK1	7,81	8,31	6,35
ETNK2	5,37	5,66	-3,32
ETS1	8,88	5,74	6,39
ETV2	3,33	2,89	3,36
ETV3	8,77	5,68	7,75
ETV5	9,17	7,76	3,87
ETV6	11,08	9,98	11,13
ETV7	4,92	5,13	5,11
EVA1	1,58	5,57	8,65
EVC	4,31	4,47	3,82
EVI2A	9,13	9,02	10,74
EVI2B	10,96	11,36	12,70
EVI5	8,98	8,79	10,18
EVI5L	8,78	8,16	8,30
EVL	13,64	13,27	11,55
EWSR1	10,40	10,24	11,07
EXO1	3,48	5,72	3,88
EXOC1	9,69	9,77	9,72

EXOC3	7,90	7,08	8,38
EXOC5	7,52	6,98	7,34
EXOC6	7,25	7,67	7,05
EXOC7	8,68	8,70	8,90
EXOC8	8,63	8,55	8,69
EXOD1	2,57	3,89	0,03
EXOSC1	7,35	7,50	7,67
EXOSC10	9,29	9,26	9,65
EXOSC2	5,77	5,71	6,12
EXOSC3	9,53	9,50	8,52
EXOSC4	7,99	7,57	7,39
EXOSC5	7,35	7,78	8,22
EXOSC7	8,21	8,77	8,13
EXOSC8	9,64	9,52	9,20
EXOSC9	8,44	8,76	8,63
EXT1	7,01	5,65	8,74
EXTL1	4,24	3,74	4,51
EXTL2	7,38	7,74	5,13
EXTL3	6,31	6,39	7,89
EYA3	3,44	4,41	6,46
EZH1	5,34	5,29	6,06
EZH2	6,33	5,87	5,52
F11R	9,19	9,49	9,47
F13A1	11,86	2,45	10,01
F2RL1	-3,32	-3,32	7,11
F2RL3	3,53	4,04	4,94
F3	6,33	8,12	-0,54
F8	4,36	4,60	3,44
FA2H	6,35	5,53	-0,96
FABP2	3,31	4,21	3,67
FABP3	6,03	5,56	-3,32
FABP4	13,13	5,59	3,56
FABP5	12,37	11,28	6,66
FABP7	4,77	4,72	4,61
FADD	9,41	9,25	8,28
FADS1	11,65	11,46	8,64
FADS2	7,89	7,52	5,02
FADS3	7,83	8,40	5,91
FAF1	8,08	8,39	8,17
FAH	8,11	9,51	7,22
FAHD1	7,53	7,43	6,32
FAHD2A	3,59	4,17	3,23
FAIM2	1,81	2,11	-3,32
FAIM3	6,34	10,59	4,90
FAM100B	10,10	9,68	10,79
FAM101A	4,20	4,37	4,57
FAM102A	5,37	4,20	2,37
FAM102B	9,34	8,70	9,41
FAM103A1	7,74	7,56	7,27
FAM104A	10,07	10,01	10,33
FAM105A	8,68	8,62	10,02
FAM107A	2,03	3,73	3,60
FAM108A1	3,68	4,52	4,87
FAM109A	7,51	6,69	6,48
FAM109B	5,70	5,70	5,70
FAM111A	8,20	9,02	10,02
FAM111B	2,48	4,41	4,15
FAM112B	5,86	6,29	6,83
FAM113A	7,06	6,83	7,42

FAM113B	7,72	7,71	5,26
FAM13A1	5,08	6,00	7,02
FAM14A	8,25	9,20	8,70
FAM14B	4,83	5,76	-0,59
FAM18B	8,48	8,82	7,03
FAM19A2	3,73	3,10	4,49
FAM19A3	6,94	6,45	1,48
FAM20A	6,02	3,77	-3,32
FAM21C	5,53	5,26	5,60
FAM24B	4,35	4,81	5,68
FAM26B	7,06	7,90	8,46
FAM32A	10,26	9,67	10,52
FAM33A	6,65	6,29	5,99
FAM35A	7,21	7,13	7,56
FAM36A	7,63	7,70	8,23
FAM3A	10,11	10,15	9,69
FAM3C	7,14	7,55	5,81
FAM40A	8,39	8,19	8,49
FAM40B	4,37	4,44	4,00
FAM44A	2,94	4,12	3,96
FAM44B	8,29	8,14	8,15
FAM46A	9,74	10,29	11,30
FAM46C	5,68	2,13	3,96
FAM48A	5,21	5,19	5,63
FAM49A	6,31	7,44	9,04
FAM49B	10,27	9,83	10,97
FAM50A	11,76	11,58	11,47
FAM53A	6,55	4,85	5,93
FAM53B	5,42	4,76	7,38
FAM53C	9,26	9,62	10,09
FAM54A	5,80	7,23	3,57
FAM54B	9,28	9,24	9,41
FAM57A	6,18	6,27	2,84
FAM58A	9,45	8,83	8,27
FAM62A	11,53	11,30	10,34
FAM62B	4,30	4,54	4,94
FAM63A	9,12	9,38	10,18
FAM63B	7,13	7,27	7,26
FAM64A	-2,81	6,01	-3,32
FAM65A	10,36	10,22	10,51
FAM69A	5,33	4,04	3,64
FAM69B	3,02	2,75	3,53
FAM70A	7,31	6,70	3,15
FAM70B	6,09	7,05	5,21
FAM71C	3,02	4,10	3,17
FAM72A	5,56	5,37	6,01
FAM73A	5,56	5,36	6,59
FAM73B	6,94	6,58	7,32
FAM76A	7,62	7,53	7,20
FAM76B	6,66	6,53	9,00
FAM78A	6,04	5,81	6,66
FAM79A	10,67	10,53	10,08
FAM80A	3,16	3,72	3,03
FAM81A	4,20	5,92	-0,59
FAM82B	5,14	5,27	6,02
FAM82C	9,69	11,60	8,74
FAM83F	5,53	5,35	5,47
FAM84A	3,04	3,07	2,87
FAM86A	6,52	6,15	6,30

FAM86B1	4,25	4,68	4,51
FAM86C	4,47	5,11	3,88
FAM89A	7,07	8,42	6,35
FAM89B	9,75	10,46	9,35
FAM8A1	8,97	9,50	10,62
FAM91A1	7,86	7,85	6,76
FAM96A	13,11	12,82	12,05
FAM96B	10,09	10,02	9,71
FAM98A	10,28	9,93	8,08
FAM9C	-0,93	-0,19	0,49
FANCB	2,93	4,81	5,12
FANCE	7,09	9,97	6,81
FANCF	3,64	3,61	4,75
FANCG	6,11	5,84	4,64
FANCL	6,31	6,78	5,92
FARS2	7,77	8,05	8,32
FARSLB	6,01	5,73	7,75
FAS	5,89	5,93	6,05
FASTK	6,72	6,79	6,58
FAU	12,81	12,69	13,50
FBL	8,71	8,66	10,38
FBN2	5,57	5,86	10,10
FBP1	12,83	14,35	10,40
FBS1	5,66	5,08	6,26
FBXL10	10,17	9,57	9,90
FBXL11	8,55	8,57	9,61
FBXL13	4,10	5,23	3,07
FBXL14	3,23	3,53	4,45
FBXL15	8,79	8,76	9,09
FBXL16	3,89	3,49	4,37
FBXL17	5,50	5,90	5,64
FBXL19	6,04	6,43	6,66
FBXL3	8,46	8,21	8,18
FBXL6	6,83	6,38	6,43
FBXL8	4,82	4,23	5,43
FBXO11	8,02	7,81	8,76
FBXO15	6,59	8,38	3,51
FBXO16	5,01	3,07	3,62
FBXO21	9,52	9,25	9,90
FBXO27	4,75	2,68	-1,58
FBXO28	8,29	8,68	8,09
FBXO3	5,93	6,12	6,10
FBXO30	7,84	7,93	7,21
FBXO31	8,38	7,94	8,02
FBXO32	5,81	5,61	4,55
FBXO33	9,71	9,58	10,28
FBXO38	7,85	7,76	7,58
FBXO4	6,41	6,03	5,77
FBXO42	6,67	6,17	6,80
FBXO43	4,51	4,45	4,48
FBXO44	3,88	4,00	4,31
FBXO5	6,15	7,31	6,59
FBXO7	8,73	8,54	8,48
FBXO8	9,13	8,97	7,41
FBXO9	4,39	4,43	4,98
FBXW11	8,39	8,67	8,26
FBXW2	3,94	3,99	4,21
FBXW4	7,32	7,48	7,33
FBXW7	6,14	5,94	7,92

FBXW8	3,86	3,58	3,95
FBXW9	5,72	6,06	6,63
FCER1G	12,89	13,82	13,24
FCGBP	-0,81	1,78	4,43
FCGR2A	8,88	8,98	10,15
FCGR2B	9,85	9,54	6,24
FCGRT	11,27	11,85	12,23
FCHO1	7,28	6,00	7,46
FCHO2	8,50	9,26	7,85
FCHSD2	9,30	9,05	9,95
FCN1	5,44	10,45	14,52
FCRL3	4,86	4,76	4,95
FCRL5	2,69	3,39	4,27
FDFT1	10,79	10,43	9,67
FDPS	8,38	8,20	7,28
FDX1	5,75	8,31	5,38
FDXR	7,74	8,33	7,25
FEM1B	7,89	8,59	7,18
FEN1	5,48	7,06	5,12
FER	5,90	5,96	6,30
FER1L3	11,09	11,76	9,65
FERD3L	4,75	4,92	5,17
FES	9,20	8,42	10,46
FFAR1	3,82	3,82	3,89
FGA	2,17	3,01	2,83
FGD2	8,17	5,31	8,95
FGD3	9,09	9,11	11,92
FGD4	6,34	6,62	7,61
FGD5	8,44	9,10	-1,27
FGD6	6,89	7,49	7,53
FGF11	4,57	3,90	4,12
FGF12	3,25	3,68	3,82
FGF13	3,64	3,90	3,03
FGF14	-0,91	0,21	1,40
FGF17	4,23	4,39	3,98
FGF18	5,63	5,57	5,74
FGF19	4,71	4,59	4,14
FGF20	3,62	3,84	4,51
FGFR1OP	5,45	5,72	4,81
FGFR1OP2	7,63	8,01	8,10
FGL2	12,64	10,47	12,70
FGR	12,15	12,70	13,11
FH	11,45	11,70	10,05
FHIT	5,81	5,69	4,32
FHL1	5,84	6,80	2,95
FHL2	4,27	3,88	2,60
FHL5	4,45	4,63	4,72
FHOD1	10,54	9,21	9,48
FIBCD1	4,36	4,06	4,90
FIBP	9,78	9,79	9,87
FIGLA	3,59	2,86	3,86
FIGN	4,24	4,29	2,44
FIGNL1	4,84	5,91	2,99
FIP1L1	8,25	8,25	8,34
FJX1	5,11	5,03	2,73
FKBP11	6,36	6,27	7,91
FKBP14	6,97	6,39	4,96
FKBP1A	11,00	11,05	10,97
FKBP2	7,06	7,53	6,60

FKBP3	6,77	6,78	6,61
FKBP4	8,58	8,53	7,88
FKBP6	4,55	3,45	4,77
FKBP8	6,68	6,59	6,82
FKBP9	0,01	3,72	2,38
FKBPL	4,64	4,75	4,88
FKRP	4,96	5,23	5,65
FKSG83	2,18	2,27	4,16
FLAD1	7,68	7,46	7,90
FLI1	9,93	9,63	11,34
FLII	9,10	8,90	9,21
FLJ10154	11,09	10,60	12,16
FLJ10241	9,64	9,77	9,26
FLJ10292	4,37	4,07	4,71
FLJ10324	-0,17	2,96	4,48
FLJ10357	5,73	5,69	7,06
FLJ10769	7,97	8,18	8,26
FLJ10781	4,58	4,30	4,29
FLJ10803	5,18	4,39	4,36
FLJ10986	8,20	9,03	6,06
FLJ11184	5,72	5,33	5,80
FLJ11235	3,91	3,24	4,15
FLJ11506	8,66	8,10	8,79
FLJ11783	4,01	3,68	5,43
FLJ12716	8,97	9,10	9,63
FLJ13611	6,91	7,10	6,83
FLJ14154	8,59	8,18	8,67
FLJ14213	5,10	2,62	6,77
FLJ14803	8,56	8,66	8,85
FLJ16124	5,03	4,86	5,15
FLJ20035	5,49	6,96	7,88
FLJ20186	9,22	9,45	9,29
FLJ20294	5,25	5,17	5,04
FLJ20309	7,31	7,43	8,00
FLJ20489	6,65	7,82	5,66
FLJ20628	7,30	7,40	7,56
FLJ20699	7,97	7,84	7,80
FLJ20850	4,49	2,38	4,77
FLJ21839	3,57	4,25	0,10
FLJ21963	3,49	3,74	3,79
FLJ21986	5,02	8,92	9,73
FLJ22222	5,99	6,07	5,87
FLJ22639	3,40	4,61	4,97
FLJ22662	11,25	12,64	13,43
FLJ23356	4,47	4,31	3,37
FLJ25715	4,11	4,25	3,96
FLJ30679	3,99	3,72	3,62
FLJ30934	5,15	5,44	5,15
FLJ31438	5,56	5,82	5,93
FLJ31818	6,24	5,82	6,54
FLJ32549	5,67	6,22	4,82
FLJ33590	2,90	3,69	3,71
FLJ33790	3,61	3,36	0,52
FLJ34047	5,55	5,23	7,14
FLJ34870	3,30	-0,67	-1,19
FLJ34931	5,10	5,76	3,83
FLJ35767	6,35	6,65	6,66
FLJ35801	4,96	5,02	5,05
FLJ36492	0,47	2,71	4,40

FLJ36874	10,24	10,36	10,61
FLJ37078	4,20	0,03	3,29
FLJ38377	3,09	2,64	3,96
FLJ38482	7,56	7,44	6,61
FLJ38717	6,89	6,66	8,03
FLJ39378	4,46	4,50	2,15
FLJ39653	4,73	4,07	4,45
FLJ39779	4,90	5,08	6,11
FLJ40125	2,00	4,41	0,26
FLJ40852	4,14	3,88	3,56
FLJ41327	5,12	5,03	5,06
FLJ41423	4,30	3,59	3,75
FLJ42133	4,34	3,81	4,80
FLJ42875	4,41	3,53	4,78
FLJ42957	-0,27	-1,45	4,68
FLJ43093	5,64	5,75	6,92
FLJ43276	2,40	1,75	2,66
FLJ43752	3,97	3,02	2,52
FLJ43870	6,12	6,14	6,08
FLJ44186	3,22	3,79	4,26
FLJ44635	3,14	2,74	3,86
FLJ45055	7,65	7,80	7,26
FLJ45537	3,94	4,21	4,61
FLJ45983	3,50	4,75	3,15
FLJ46082	2,56	1,78	3,37
FLJ46154	4,06	4,52	4,54
FLJ46347	4,17	4,43	3,48
FLJ46380	4,69	4,06	4,21
FLJ46836	4,22	4,17	4,48
FLJ90709	7,72	7,91	7,32
FLJ90757	7,20	7,00	7,70
FLOT1	8,97	9,90	9,78
FLOT2	11,95	12,02	12,54
FLRT2	5,37	7,97	4,12
FLT1	5,65	4,69	3,26
FLT3	4,65	2,23	7,55
FLT3LG	4,32	3,38	4,20
FLYWCH1	5,30	5,36	5,08
FMNL1	5,62	5,23	7,32
FMNL2	7,52	7,88	5,42
FMNL3	4,84	5,14	2,71
FMO1	3,34	4,06	0,20
FMO2	4,80	4,30	4,91
FMO4	5,24	6,17	5,36
FMO5	4,95	6,11	7,76
FN3KRP	9,64	9,86	9,67
FNBP1	11,53	11,05	12,56
FNBP4	8,05	8,16	9,22
FNDC3A	9,57	9,30	8,74
FNDC8	4,17	3,99	3,49
FNTA	9,67	9,51	9,75
FNTB	6,80	7,32	7,62
FOLR3	6,81	10,11	12,03
FOS	9,68	10,22	13,89
FOSB	1,25	6,39	8,86
FOSL2	7,28	7,08	8,74
FOXA1	2,12	2,84	3,87
FOXC2	4,56	4,60	6,71
FOXD2	6,97	5,13	4,21

FOXD4L1	6,26	2,66	3,36
FOXE1	3,96	3,88	3,67
FOXI1	4,00	4,09	3,58
FOXJ2	10,72	9,72	10,51
FOXJ3	9,06	9,07	9,67
FOXM1	3,77	4,98	3,60
FOXP1	4,93	5,14	6,47
FOXP3	5,28	5,46	5,10
FOXP4	2,69	1,92	4,90
FOXQ1	11,37	-0,13	3,18
FOXRED1	8,31	8,44	7,70
FPGS	5,02	5,05	3,43
FPGT	4,50	3,70	3,95
FPR1	7,29	10,08	13,08
FPRL1	4,97	6,31	7,73
FPRL2	12,20	10,98	6,29
FRAG1	8,96	9,30	8,47
FRAP1	8,69	8,81	8,90
FRAT2	10,10	9,72	10,90
FREQ	6,89	8,33	4,66
FRG1	11,55	11,58	11,16
FRMD3	4,85	5,91	7,47
FRMD4A	9,26	7,42	-3,32
FRMD6	4,75	4,08	1,80
FRRS1	5,43	5,88	4,07
FRS2	4,72	4,84	5,40
FRS3	6,31	6,27	7,47
FRY	4,41	5,19	7,44
FRZB	3,42	3,14	4,46
FSCN1	12,76	11,23	9,56
FSD1L	4,62	4,44	4,50
FST	5,91	2,43	-3,32
FSTL1	4,66	-1,08	-3,32
FSTL3	4,33	4,48	6,48
FTH1	11,82	13,13	11,95
FTL	15,01	15,01	14,69
FTSJ1	7,07	7,32	7,93
FTSJ2	6,87	6,57	6,48
FTSJ3	6,92	6,64	6,88
FUBP1	5,61	6,09	6,50
FUCA1	12,03	12,02	10,66
FUCA2	10,03	10,36	9,21
FUK	8,40	8,55	8,68
FUNDC1	8,84	8,63	7,54
FURIN	9,47	8,56	7,83
FUSIP1	7,73	7,15	7,83
FUT10	3,79	3,30	4,05
FUT11	6,28	6,06	5,98
FUT4	9,07	9,03	10,00
FVT1	6,67	6,91	6,32
FXC1	7,74	7,81	7,34
FXR1	7,86	7,46	8,10
FXR2	6,36	6,21	6,16
FXYD3	3,71	2,99	3,36
FXYD6	5,93	8,26	7,68
FYB	9,44	10,32	11,69
FYCO1	7,02	8,43	7,90
FYN	5,34	5,69	9,14
FYTTD1	9,49	9,27	9,28

FZD1	7,54	6,21	7,98
FZD2	9,66	5,23	9,15
FZD3	5,57	2,11	-0,78
FZD5	5,10	4,92	4,77
FZD7	4,97	3,87	-3,32
G0S2	5,72	5,11	8,93
G3BP2	8,21	8,11	7,73
G6PC3	10,12	10,67	9,88
G6PD	10,04	10,16	8,82
GAA	9,33	9,13	9,32
GAB2	8,68	7,47	9,36
GAB3	6,39	6,46	8,22
GABARAP	8,34	8,49	9,28
GABARAPL1	11,46	12,07	11,36
GABARAPL2	11,69	11,60	11,05
GABPA	7,39	7,69	8,17
GABPB2	8,23	7,70	6,81
GADD45A	8,18	6,81	7,21
GADD45B	7,25	7,50	10,26
GADD45G	8,01	7,90	5,07
GAL	7,16	10,29	-3,32
GAL3ST4	4,40	4,64	4,38
GALE	6,65	6,90	5,24
GALK2	6,94	6,44	6,79
GALM	10,86	10,23	5,43
GALNACT-2	8,06	7,91	8,86
GALNS	8,19	8,67	7,55
GALNT11	10,07	10,13	9,56
GALNT12	10,73	10,03	1,93
GALNT13	5,19	5,07	5,00
GALNT2	4,84	5,11	5,46
GALNT4	6,24	6,46	7,46
GALNT7	5,03	4,93	4,98
GALNTL1	3,77	3,88	2,12
GALNTL4	9,51	-3,32	-1,37
GALR2	-3,32	-3,32	4,29
GALT	6,99	6,97	7,29
GAMT	3,76	4,11	4,05
GAN	3,74	3,55	4,25
GANAB	8,10	8,42	8,55
GANC	5,15	4,95	5,33
GAPDH	12,44	12,68	12,85
GARNL1	6,88	6,91	7,52
GARS	12,18	12,15	10,73
GART	8,55	8,23	7,77
GAS2L1	4,33	3,94	4,56
GAS2L3	8,32	7,69	3,36
GAS6	8,92	6,58	6,22
GAS7	7,58	7,35	9,29
GAS8	3,80	0,51	2,31
GATAD1	7,72	7,48	7,58
GATAD2A	10,54	10,59	10,63
GATS	1,76	4,46	6,34
GBA	10,47	10,99	8,45
GBE1	8,24	9,01	8,40
GBF1	6,91	6,87	7,03
GBGT1	7,48	7,99	8,71
GBP1	7,26	8,30	8,39
GBP2	10,02	10,62	11,50

GBP3	6,69	6,72	6,62
GBP4	5,91	6,96	9,07
GBP5	6,90	6,22	8,07
GBX2	3,45	3,94	4,11
GCA	11,26	12,17	12,78
GCAT	4,26	2,65	3,04
GCC1	9,33	9,11	8,81
GCC2	4,62	4,86	4,34
GCDH	8,23	8,04	8,58
GCET2	4,25	4,02	3,93
GCH1	4,96	5,65	9,14
GCHFR	9,68	12,11	8,37
GCLC	9,44	10,62	8,74
GCLM	8,20	8,38	5,75
GCN1L1	10,20	9,44	9,02
GCNT1	6,37	7,07	7,43
GCNT2	4,96	5,75	5,45
GCNT3	8,03	4,68	0,14
GCS1	6,98	6,93	7,93
GDAP1	4,59	5,00	4,82
GDAP2	6,96	6,68	7,10
GDF1	2,12	2,65	0,46
GDF15	8,67	9,39	4,44
GDF5	3,83	4,12	3,87
GDF9	5,73	-0,51	2,54
GDI1	8,98	8,67	8,80
GDI2	12,57	12,60	12,77
GDPD1	3,78	4,33	3,74
GDPD2	4,03	4,57	3,69
GDPD5	6,92	6,21	7,42
GEM	4,80	6,47	2,92
GEMIN6	8,89	8,93	7,93
GFI1	4,53	4,71	2,19
GFM1	10,01	9,68	9,66
GFM2	6,31	6,82	5,37
GFOD1	11,30	10,09	8,39
GFOD2	5,71	5,40	5,19
GFPT1	9,49	8,73	6,95
GFRA3	4,56	4,16	3,08
GGA1	5,21	5,74	5,39
GGA3	6,90	6,71	7,46
GGCX	6,83	7,15	5,44
GGH	7,36	9,51	6,42
GGPS1	9,68	9,19	9,86
GGTL3	3,48	1,93	3,06
GHITM	12,85	12,81	12,33
GHSR	3,93	4,34	3,37
GIF	2,91	2,77	3,71
GIMAP1	7,00	8,41	10,33
GIMAP2	8,07	8,47	9,78
GIMAP4	10,82	11,64	12,60
GIMAP5	7,42	8,07	7,79
GIMAP6	5,50	6,82	8,27
GIMAP7	7,09	7,69	10,58
GIMAP8	7,66	9,12	11,17
GIOT-1	4,41	4,10	4,78
GIP	3,16	3,81	4,08
GIPC1	6,87	6,80	4,68
GIPC3	3,38	3,83	3,12

GIPR	3,34	3,24	3,98
GIT1	8,02	7,12	7,60
GIT2	8,02	8,05	9,47
GIYD2	6,31	6,18	7,34
GJA4	3,81	4,67	4,36
GJA5	4,86	4,90	4,49
GJA7	4,61	4,02	3,58
GJB2	9,30	11,78	3,07
GJB6	3,26	6,22	3,87
GK	9,26	10,14	7,92
GKAP1	3,82	3,31	5,22
GLA	12,31	12,94	10,80
GLB1	9,52	9,51	8,83
GLB1L	6,79	7,19	6,98
GLCCI1	3,79	3,42	4,71
GLCE	8,68	8,45	8,58
GLG1	11,53	11,46	11,89
GLI3	3,86	3,95	-3,32
GLI4	0,02	-1,65	5,22
GLIPR1	11,01	11,99	11,84
GLIS1	4,09	4,48	4,28
GLO1	10,85	11,18	9,89
GLRX	11,58	12,98	12,71
GLRX2	8,84	8,89	6,62
GLRX5	11,75	11,69	11,64
GLS	10,68	9,98	7,18
GLT1D1	6,33	6,83	8,02
GLT25D1	11,47	12,08	11,32
GLT8D1	6,65	6,51	6,96
GLTP	11,57	11,76	12,31
GLUD1	11,71	11,47	11,95
GLUD2	3,76	4,03	4,32
GLUL	4,74	5,73	5,56
GLYCTK	5,81	6,06	6,63
GM2A	14,27	14,37	11,46
GMCL1	8,28	8,36	8,37
GMDS	8,17	8,43	9,30
GMEB1	6,74	6,80	7,36
GMFG	11,84	12,19	13,03
GMIP	10,49	9,94	10,97
GMNN	7,81	8,05	6,67
GMPPA	7,17	7,68	7,47
GMPPB	5,39	5,61	5,04
GMPR	5,57	6,42	5,06
GMPR2	8,25	8,43	8,71
GNA12	6,61	6,91	5,82
GNAI2	12,23	11,98	12,73
GNAI3	8,74	8,75	9,10
GNAQ	9,32	9,01	9,05
GNAS	11,61	11,95	11,85
GNB1	12,73	12,54	12,87
GNB1L	6,60	6,24	6,53
GNB2L1	14,24	14,21	14,47
GNB4	8,76	7,66	7,87
GNB5	2,26	3,31	0,54
GNE	9,68	9,52	9,16
GNG11	3,61	5,36	4,16
GNG13	5,39	5,55	5,19
GNG5	9,71	9,68	9,27

GNGT2	7,45	4,89	6,83
GNL1	7,66	7,83	7,37
GNL2	10,48	10,61	10,95
GNL3	8,47	8,26	8,04
GNL3L	9,28	8,93	10,02
GNPAT	9,52	9,53	9,54
GNPDA1	11,80	11,57	10,12
GNPDA2	7,57	7,93	7,05
GNPTAB	9,04	10,20	8,93
GNPTG	9,82	9,90	9,59
GNS	14,06	13,79	13,36
GOLGA1	6,77	6,43	7,08
GOLGA2	6,74	6,89	6,69
GOLGA3	10,31	10,04	9,91
GOLGA4	6,79	6,96	6,45
GOLGA5	9,55	9,58	9,31
GOLGA7	9,75	9,73	10,19
GOLGB1	9,68	9,11	8,80
GOLPH2	6,94	5,27	7,20
GOLPH3	10,50	10,53	10,93
GOLPH3L	8,24	7,53	7,88
GOLPH4	7,60	7,58	7,07
GOLT1B	9,31	9,47	8,90
GON4L	7,53	7,11	8,06
GOPC	7,37	7,17	7,48
GORASP1	8,36	7,99	8,86
GORASP2	9,23	9,16	8,61
GOSR1	7,78	7,62	7,33
GOSR2	6,58	6,75	6,39
GOT1	10,01	9,78	7,79
GOT2	10,55	10,56	9,47
GPAM	5,98	6,17	4,64
GPBAR1	3,94	5,96	10,62
GPBP1	9,55	9,46	10,30
GPC3	-1,57	4,97	-3,32
GPC4	7,47	10,90	0,24
GPC5	4,58	3,38	2,07
GPC6	7,51	7,45	7,71
GPD1	5,15	6,78	0,34
GPHN	5,72	5,75	5,01
GPKOW	8,17	8,61	9,21
GPNMB	13,88	14,42	5,22
GPR107	4,33	4,82	4,43
GPR108	6,87	6,94	6,99
GPR109A	2,08	4,62	2,77
GPR109B	3,91	3,97	3,40
GPR120	6,61	5,64	4,54
GPR125	-1,48	2,53	1,91
GPR126	3,63	4,35	3,44
GPR132	6,43	5,30	7,07
GPR133	5,20	4,86	5,03
GPR135	2,33	3,10	3,68
GPR137B	11,91	10,96	8,57
GPR142	5,12	5,23	4,04
GPR143	4,85	2,52	3,78
GPR146	5,70	3,39	-3,32
GPR149	5,50	5,77	5,15
GPR155	3,28	0,94	4,43
GPR158	3,86	4,39	3,69

GPR160	8,49	6,03	7,68
GPR161	3,95	3,79	3,70
GPR162	5,52	6,89	9,81
GPR172A	8,64	8,67	7,99
GPR176	4,32	2,58	2,82
GPR177	4,60	8,08	8,08
GPR23	2,84	4,45	3,31
GPR26	4,20	4,15	4,42
GPR27	5,33	5,10	5,19
GPR3	4,35	4,63	4,28
GPR34	3,05	7,60	5,37
GPR44	2,47	-0,23	5,47
GPR55	4,58	4,39	4,15
GPR64	3,53	1,38	-0,04
GPR65	7,49	8,75	9,06
GPR68	8,39	9,53	-3,32
GPR82	3,38	4,48	2,27
GPR84	4,64	8,09	5,92
GPRASP1	4,67	4,31	4,21
GPRASP2	5,55	5,69	4,41
GPRC5A	3,53	4,27	4,21
GPRC5B	4,20	5,14	4,14
GPRC5C	3,21	3,59	4,13
GPS1	9,09	9,06	9,34
GPS2	8,02	7,98	8,64
GPSM3	9,35	10,51	11,99
GPSN2	11,22	12,05	10,06
GPT2	7,13	7,44	6,66
GPX1	12,71	12,54	12,40
GPX7	2,59	4,32	6,21
GRAMD1A	9,64	8,87	9,11
GRAMD2	3,81	3,12	0,22
GRAP	3,07	2,62	2,93
GRASP	2,91	4,44	6,49
GRB2	9,48	9,91	10,44
GREM1	-3,32	6,35	-3,32
GRHPR	8,44	8,58	8,59
GRIA2	5,29	5,20	5,22
GRIA3	2,89	2,66	3,09
GRIK1	2,99	2,51	2,51
GRIK5	4,69	4,51	4,25
GRIN1	2,75	1,94	1,52
GRIN2D	3,04	3,26	3,51
GRIN3A	4,01	5,12	2,72
GRINA	9,64	10,08	10,30
GRIPAP1	7,35	6,64	7,30
GRK4	2,56	2,70	3,31
GRK5	3,32	4,08	6,11
GRM1	2,22	3,90	0,32
GRM2	3,90	4,33	5,62
GRN	12,99	13,27	12,50
GRP	4,90	4,14	5,02
GRPEL1	9,53	8,94	9,86
GRPEL2	8,12	7,77	7,51
GRPR	1,89	0,60	2,07
GRWD1	7,79	7,59	7,14
GSC	2,82	4,37	0,26
GSDMDC1	8,71	8,68	9,79
GSG1	3,92	4,07	3,45

GSG2	-0,35	4,43	0,12
GSK3B	9,35	9,47	9,21
GSN	9,61	10,10	6,60
GSPT1	12,10	12,02	11,43
GSPT2	8,70	8,36	7,00
GSS	7,76	8,38	7,74
GSTA4	3,75	3,74	4,05
GSTCD	4,00	3,81	3,90
GSTK1	12,41	11,90	12,16
GSTM2	3,48	4,37	4,28
GSTM3	3,13	4,83	-3,32
GSTM4	8,31	8,71	6,57
GSTO1	12,68	13,41	11,86
GSTO2	3,75	4,23	4,33
GSTT1	11,33	9,01	6,62
GSTT2	2,98	4,12	-3,32
GTDC1	6,71	6,06	5,07
GTF2A1	2,48	2,22	2,71
GTF2B	8,21	7,79	8,49
GTF2E1	8,63	8,24	6,96
GTF2F1	8,68	8,66	9,02
GTF2F2	10,34	10,23	10,12
GTF2H1	8,63	7,88	7,54
GTF2H2	6,06	5,93	5,75
GTF2H3	7,42	8,35	6,55
GTF2H4	6,68	6,80	7,04
GTF2H5	10,35	10,50	9,97
GTF2I	5,59	6,36	4,90
GTF2IRD1	5,67	5,37	3,20
GTF3A	11,54	12,11	11,89
GTF3C1	7,60	7,42	7,57
GTF3C2	10,51	10,39	10,17
GTF3C3	8,35	8,46	8,47
GTF3C4	3,52	3,81	3,58
GTF3C5	8,11	8,04	8,40
GTPBP1	2,86	-0,35	4,96
GTPBP2	5,65	5,68	5,71
GTPBP3	6,42	6,18	7,02
GTPBP4	10,33	10,44	10,19
GTPBP8	7,36	7,09	7,13
GTSE1	6,16	6,48	6,22
GUCA1A	8,74	3,21	3,27
GUCY1A3	3,58	4,08	5,86
GUCY2D	3,27	4,47	4,73
GUF1	8,15	8,46	6,69
GUSBL1	9,18	9,65	8,55
GUSBL2	6,52	6,62	7,20
GYPC	7,06	8,53	8,06
GYS1	6,73	6,67	7,10
GYS2	4,27	4,41	3,89
GCOM1	5,27	-0,76	1,93
H1F0	3,10	4,37	4,26
H1FNT	5,38	4,84	5,85
H2AFJ	8,61	8,60	8,92
H2AFV	5,59	5,46	5,69
H2AFY	10,99	11,21	11,38
H2AFY2	7,68	7,97	7,19
H2AFZ	12,69	12,90	12,45
H3F3A	13,61	13,54	13,96

H6PD	8,19	6,77	6,80
HAAO	5,09	5,50	4,77
HABP4	5,53	5,64	3,91
HADHA	8,55	8,53	8,77
HAGH	11,07	10,90	9,97
HAGHL	6,49	6,20	4,11
HAL	3,84	4,83	6,98
HAP1	3,77	4,08	3,17
HAPLN3	2,33	-0,25	1,61
HAPLN4	4,93	2,90	3,31
HARS	9,43	9,57	9,18
HARS2	7,94	7,52	7,41
HAS1	4,54	4,33	4,43
HAS2	2,83	2,55	2,83
HAT1	9,58	9,76	9,36
HAVCR2	13,50	13,25	11,18
HAX1	9,04	9,38	8,75
HBA2	4,97	4,91	4,38
HBEGF	6,42	9,11	8,53
HBP1	9,09	8,86	9,24
HBS1L	3,34	2,68	4,77
HCCA2	6,95	6,78	7,37
HCCS	7,87	7,60	6,83
HCFC1	10,48	10,11	11,14
HCFC1R1	10,50	10,45	9,71
HCFC2	7,59	7,03	7,80
HCG18	6,34	6,13	6,76
HCG27	1,80	3,85	5,44
HCK	11,01	11,04	12,04
HCLS1	11,98	12,13	12,86
HCN3	4,35	4,14	2,99
HCP5	10,85	11,16	9,82
HCRTR2	4,74	4,59	4,58
HCST	13,36	13,87	13,19
HDAC1	11,05	10,78	11,60
HDAC2	11,87	11,07	11,24
HDAC3	9,92	9,49	9,90
HDAC4	8,07	8,12	8,93
HDAC6	7,60	7,22	7,63
HDAC7A	3,99	4,46	6,28
HDAC8	5,22	6,06	4,66
HDAC9	4,59	4,09	6,09
HDDC3	8,52	8,44	7,86
HDGF	9,15	9,08	9,66
HDGF2	6,17	5,72	5,54
HDGFRP3	4,55	5,49	3,55
HDHD1A	8,82	9,29	9,52
HDHD2	9,62	9,56	9,65
HDHD3	7,23	7,13	6,40
HDLBP	4,62	4,79	4,45
HEATR1	8,10	8,25	7,84
HEBP1	9,74	10,14	10,15
HECTD1	9,61	9,98	9,91
HECTD2	5,14	4,42	5,50
HECTD3	7,75	7,47	6,64
HECW2	0,83	3,24	5,18
HEL308	6,49	6,52	7,01
HELB	4,80	4,99	5,07
HELLS	1,79	4,87	2,95

HELZ	8,15	8,01	9,78
HEMGN	3,51	3,12	3,27
HERC1	9,30	9,09	9,15
HERC2	8,16	7,98	7,00
HERC3	7,24	7,26	7,36
HERC4	8,80	8,89	8,25
HERC5	8,21	9,64	10,04
HERPUD2	8,73	7,88	8,49
HES1	4,37	4,21	3,56
HES2	-0,73	4,84	-3,32
HES3	4,15	3,90	3,24
HES6	10,26	8,48	7,29
HEXA	7,15	7,29	5,69
HEXDC	7,13	6,28	8,18
HEXIM1	5,72	5,62	6,53
HEXIM2	4,93	4,39	4,65
HEY1	3,95	5,30	4,69
HFE2	2,82	3,02	-0,28
HGF	-0,61	3,56	5,13
HGS	10,28	10,20	10,73
HHAT	4,38	6,03	4,77
HHEX	8,79	8,65	10,51
HHLA2	4,38	3,20	3,99
HHLA3	4,00	5,60	0,68
HIAT1	8,96	8,85	8,49
HIBCH	9,06	9,82	7,93
HIC1	3,82	3,68	4,03
HIC2	7,40	7,73	6,84
HIF1A	8,73	8,33	9,89
HIF1AN	7,34	7,53	6,84
HIF3A	2,76	2,86	3,07
HIG2	6,86	7,00	5,15
HIGD1A	11,52	11,64	10,32
HIGD2A	12,28	11,98	12,50
HINT1	12,68	12,79	12,26
HINT2	9,08	9,11	8,85
HINT3	7,58	7,48	5,86
HIP2	9,75	9,87	9,05
HIPK1	2,37	-0,99	3,59
HIRA	6,23	5,81	6,16
HIRIP3	7,16	7,45	6,85
HIST1H1C	7,26	8,48	7,68
HIST1H1D	2,13	3,78	4,81
HIST1H2AC	6,72	6,77	6,22
HIST1H2AH	3,87	3,77	3,71
HIST1H2AJ	4,34	4,91	4,55
HIST1H2AK	3,90	4,66	4,48
HIST1H2AL	6,36	6,20	6,60
HIST1H2BC	3,49	4,62	4,28
HIST1H2BD	7,23	6,79	5,58
HIST1H2BJ	4,12	4,07	-0,99
HIST1H2BK	10,90	10,91	10,52
HIST1H2BO	4,22	4,53	4,53
HIST1H3D	4,31	4,52	3,37
HIST1H3F	4,08	4,26	4,13
HIST1H3G	4,16	4,23	4,30
HIST1H3I	3,80	3,08	3,85
HIST1H4A	-2,74	2,68	3,71
HIST1H4C	10,73	12,31	11,21

HIST1H4E	0,03	3,15	3,81
HIST1H4H	5,08	5,47	3,59
HIST1H4K	4,62	5,94	6,08
HIST2H2AB	3,93	3,34	5,45
HIST2H2AC	8,67	8,76	10,00
HIST2H2BE	7,42	7,80	8,09
HIST3H2BB	3,68	3,52	3,89
HIVEP1	5,27	6,32	7,07
HIVEP3	4,29	4,43	4,52
HK2	9,93	11,04	10,13
HK3	9,69	11,61	11,26
HKR1	6,02	5,94	6,40
HLA-A	13,22	13,74	13,76
HLA-B	13,56	14,01	13,99
HLA-C	8,64	9,57	10,04
HLA-DMA	13,46	13,62	12,90
HLA-DMB	13,66	13,68	13,43
HLA-DOA	5,64	6,73	7,00
HLA-DPA1	14,14	13,71	13,13
HLA-DPB1	8,96	8,65	8,57
HLA-DQA1	13,55	13,57	12,11
HLA-DQB1	8,88	9,29	8,58
HLA-DQB2	3,26	1,81	3,62
HLA-DRA	14,42	14,46	14,07
HLA-DRB1	10,86	10,50	9,96
HLA-DRB5	8,92	8,03	8,73
HLA-E	12,00	12,30	13,04
HLA-F	9,04	9,79	11,17
HLA-G	7,57	8,28	8,73
HLCS	4,27	4,67	0,32
HM13	8,14	9,21	7,71
HMBOX1	7,57	7,02	8,52
HMBS	7,94	8,05	7,56
HMG20A	7,39	7,47	7,65
HMG2L1	4,00	4,60	4,26
HMGB1	8,17	7,44	8,58
HMGB2	5,97	7,69	9,94
HMGCL	10,03	10,39	8,61
HMGCR	10,91	11,23	9,61
HMGCS1	11,41	10,41	8,55
HMGN2	6,34	6,49	7,54
HMGN3	8,83	6,95	7,25
HMGN4	10,24	10,15	11,04
HMMR	4,39	7,83	-3,32
HMOX1	9,68	9,54	10,66
HMP19	4,28	4,61	4,40
HNMT	9,50	9,78	9,64
HNRPA0	9,84	9,55	9,65
HNRPA1	5,74	5,90	5,85
HNRPA2B1	12,78	12,64	12,86
HNRPA3	8,03	8,28	8,27
HNRPAB	11,30	11,26	10,95
HNRPC	12,42	12,15	12,31
HNRPD	11,75	11,43	12,17
HNRPDL	8,03	7,95	9,60
HNRPF	7,01	6,34	6,73
HNRPH1	11,18	11,34	12,41
HNRPH2	7,06	6,85	7,59
HNRPH3	9,40	9,14	10,22

HNRPK	8,17	8,26	8,26
HNRPL	9,22	9,20	9,70
HNRPLL	8,76	8,72	8,05
HNRPM	10,91	10,78	11,44
HNRPR	11,41	10,95	11,62
HNRPU	3,88	3,78	5,58
HNRPUL1	8,35	8,73	8,82
HOM-TES-103	6,63	6,39	7,40
HOMER1	5,72	6,77	2,85
HOMER2	11,58	6,00	4,79
HOMER3	6,03	6,63	7,38
HOOK1	2,89	0,47	2,19
HOOK2	6,08	5,27	6,24
HOOK3	7,08	6,58	7,35
HOP	5,14	3,70	2,44
HOXA2	4,20	3,58	3,76
HOXA5	3,82	0,03	7,05
HOXA6	5,92	5,73	6,04
HOXA7	5,40	4,88	5,58
HOXB1	5,06	4,95	5,44
HOXB2	-0,45	2,47	4,22
HOXB3	2,02	2,96	3,89
HOXB5	3,64	4,30	4,03
HOXB6	4,62	4,32	2,64
HOXB9	3,29	4,38	3,76
HOXC13	6,16	6,12	6,12
HOXC4	2,88	3,06	2,95
HOXC8	4,45	2,97	4,63
HP	4,09	3,72	7,07
HP1BP3	6,88	6,49	7,26
HPRT1	9,36	9,14	8,96
HPS3	8,50	8,05	7,93
HPS4	4,05	4,49	4,18
HPS5	8,72	8,17	6,54
HPSE	-0,52	4,40	9,97
HRASLS3	7,63	6,62	8,74
HRES1	3,29	-0,73	0,51
HRG	3,84	3,37	2,60
HRK	3,90	6,68	-3,32
HRSP12	6,90	7,51	6,66
HS1BP3	8,97	9,08	9,01
HS2ST1	7,96	7,76	7,65
HS3ST1	8,28	2,88	1,28
HS3ST2	11,13	9,79	-2,22
HS3ST3B1	4,51	4,01	2,96
HS3ST6	4,24	4,08	4,61
HS6ST1	5,34	5,67	4,91
HSD11B1	10,79	11,43	-3,32
HSD11B1L	4,62	4,69	4,45
HSD17B2	3,09	2,85	2,54
HSD17B7	10,97	10,54	12,47
HSD17B8	7,05	6,94	6,98
HSD3B7	8,52	9,46	4,34
HSDL1	7,34	7,16	7,14
HSDL2	7,89	7,74	8,61
HSF1	6,38	5,83	6,51
HSF2	7,87	7,67	8,22
HSF2BP	5,44	6,29	3,82
HSF4	3,68	2,14	3,50

HSMPP8	7,92	7,69	8,01
HSP90AA1	11,27	11,33	10,81
HSP90AB1	11,92	11,83	11,10
HSP90B1	12,42	12,48	11,90
HSPA14	8,32	8,35	8,36
HSPA1A	11,67	11,66	12,18
HSPA1B	10,27	9,63	8,66
HSPA1L	5,68	5,67	8,02
HSPA4	9,13	8,90	8,28
HSPA4L	3,87	4,34	3,67
HSPA5	7,76	8,67	9,36
HSPA6	8,07	8,00	10,61
HSPA8	13,18	13,16	12,19
HSPB2	4,71	4,99	4,59
HSPB7	3,58	2,98	4,04
HSPBAP1	8,19	8,13	9,32
HSPBP1	4,16	4,52	4,42
HSPC111	7,77	7,61	7,51
HSPC152	11,58	11,41	11,56
HSPC159	6,80	6,93	-3,32
HSPC171	9,05	9,51	8,83
HSPD1	9,87	9,96	8,97
HSPE1	11,90	11,69	10,90
HSPH1	12,37	11,52	9,71
HTATIP2	9,06	9,24	7,99
HTATSF1	8,17	8,47	8,43
HTF9C	4,75	4,55	4,81
HTR2B	3,79	2,40	-0,12
HTR3B	5,56	5,17	6,65
HTR7	3,19	0,31	2,97
HTRA1	-3,32	7,03	6,41
HTRA2	6,83	7,05	6,93
HTRA4	4,69	6,04	3,80
HUWE1	6,74	6,80	6,94
HYAL1	4,34	4,05	4,15
HYAL3	8,39	7,42	6,59
HYLS1	7,07	5,77	5,89
HYOU1	8,00	8,16	7,68
HYPE	8,13	8,07	6,10
HYPK	3,92	2,37	5,35
IARS	8,99	9,50	7,00
IBRDC2	9,12	8,60	10,23
IBRDC3	8,36	7,21	8,53
IBTK	10,47	10,18	9,97
ICA1	7,73	7,86	9,22
ICAM3	9,44	9,32	12,89
ICAM4	4,98	4,97	5,50
ICAM5	3,02	3,44	0,96
ICF45	4,25	4,23	5,30
ICK	6,33	5,83	7,19
ICMT	8,15	8,06	6,61
ICT1	8,63	8,94	8,66
ID1	-3,32	1,27	7,14
ID2	12,01	11,18	12,28
ID2B	4,34	4,44	5,27
ID3	6,89	8,47	6,27
ID4	5,04	5,22	4,73
IDE	7,85	8,32	8,01
IDH1	10,77	10,91	8,09

IDH2	9,21	9,74	9,27
IDH3A	8,26	8,21	8,64
IDH3B	9,53	9,72	10,08
IDH3G	8,79	8,69	9,06
IDI1	10,27	9,93	8,20
IDS	8,88	9,36	9,16
IDUA	7,01	6,97	7,71
IER2	5,91	5,93	7,96
IER3	10,91	11,24	11,71
IER3IP1	9,18	9,32	8,94
IER5	9,38	8,99	10,28
IFI16	10,46	10,51	10,92
IFI30	14,35	14,22	14,15
IFI35	8,75	9,36	9,92
IFI44L	3,99	4,85	9,36
IFIH1	9,84	9,48	9,40
IFIT1	4,17	7,54	7,68
IFIT2	7,18	8,92	9,55
IFIT3	4,99	7,98	6,96
IFIT5	5,49	5,74	5,87
IFITM1	4,57	3,64	8,80
IFITM3	5,86	7,29	11,41
IFITM5	3,58	3,06	4,21
IFNA13	3,12	2,96	3,24
IFNAR1	9,51	9,72	8,71
IFNAR2	9,10	8,98	9,05
IFNGR1	12,57	12,23	12,66
IFRD1	7,43	7,26	8,28
IFRD2	7,15	7,03	6,34
IFRG15	4,83	5,11	3,90
IFT122	4,30	5,82	4,96
IFT140	3,98	4,26	1,71
IFT172	4,19	4,78	5,29
IFT52	6,28	6,84	7,03
IFT57	3,82	4,53	6,72
IFT74	7,88	8,06	7,91
IFT80	3,72	3,34	4,43
IFT88	5,41	5,89	6,08
IGBP1	9,58	9,31	10,14
IGF2BP2	9,59	8,87	8,99
IGF2BP3	5,25	8,01	5,92
IGFBP2	5,46	6,21	1,72
IGFBP6	4,38	7,30	-2,41
IGFBP7	6,81	8,54	10,21
IGFBPL1	3,05	3,64	4,07
IGFL3	5,00	5,69	5,50
IGHMBP2	5,67	5,74	5,75
IGSF10	2,16	4,55	2,96
IGSF11	3,07	3,50	2,59
IGSF2	3,46	4,21	5,87
IGSF6	14,62	14,32	13,11
IGSF8	2,49	4,28	3,40
IHH	3,44	3,69	4,07
IHPK2	6,60	6,52	6,57
IK	9,56	9,82	10,67
IKBKB	8,54	8,03	9,12
IKBKE	7,42	8,02	8,21
IKBKG	10,11	9,89	10,38
IKIP	6,79	6,89	6,77

IL10RA	10,91	9,84	11,88
IL10RB	9,18	9,84	9,67
IL11RA	5,60	6,07	7,59
IL12RB1	4,72	4,36	6,22
IL15	4,97	5,36	8,43
IL16	6,09	5,23	7,61
IL17C	4,06	4,28	4,72
IL17RB	8,30	8,08	-3,32
IL17RC	4,62	4,09	4,26
IL18	12,90	12,81	13,66
IL18RAP	4,06	4,00	4,55
IL1A	7,30	9,79	-0,38
IL1B	6,24	10,71	7,56
IL1F10	5,08	5,28	5,25
IL1F8	-0,76	4,94	-1,83
IL1RAP	7,40	5,14	6,40
IL1RAPL1	4,70	5,28	5,16
IL1RL2	3,78	-0,71	3,21
IL1RN	11,80	12,62	8,56
IL2	3,73	3,75	4,60
IL21	4,22	2,49	2,94
IL21R	7,62	6,98	4,54
IL26	5,02	5,01	4,74
IL27	4,05	4,48	5,59
IL27RA	11,14	10,47	10,50
IL28A	2,28	3,07	3,89
IL28RA	3,92	4,82	2,44
IL2RG	5,46	6,45	4,99
IL3RA	8,14	7,83	5,40
IL4I1	8,79	7,05	3,98
IL4R	10,73	10,31	10,16
IL6	2,76	5,61	-1,55
IL6R	6,33	5,78	7,56
IL6ST	5,89	3,81	4,78
IL7R	8,87	9,84	3,91
IL8	9,05	12,29	10,49
IL8RA	7,54	5,03	3,95
IL8RB	5,69	6,86	7,55
ILF2	11,45	11,48	11,08
ILF3	8,00	8,09	8,66
ILK	9,17	9,25	9,47
ILVBL	8,40	8,71	7,27
IMMP2L	7,10	5,86	5,71
IMMT	11,53	11,32	11,09
IMP4	9,08	9,15	9,09
IMPA1	9,05	9,03	8,25
IMPA2	7,09	8,65	11,10
IMPACT	4,04	4,06	0,88
IMPAD1	8,45	8,55	7,37
INCA	-2,05	1,08	4,84
INCENP	4,25	3,87	3,37
INDO	5,34	4,33	5,06
ING1	6,17	5,87	7,59
ING2	7,96	7,98	8,20
ING3	5,05	4,95	6,57
ING4	5,57	5,30	6,76
INHBB	5,01	4,68	5,38
INOC1	7,09	7,13	7,96
INPP1	8,64	8,69	8,68

INPP4A	3,69	3,82	4,77
INPP5A	7,21	6,63	7,60
INPP5B	6,36	6,11	6,36
INPP5F	4,75	4,92	4,93
INPPL1	10,05	9,86	10,37
INSIG1	11,45	11,67	8,63
INSIG2	10,01	8,93	8,42
INSM1	5,55	5,65	5,40
INSM2	5,20	-3,32	-1,35
INSR	5,16	5,73	5,50
INSRR	3,32	4,10	3,16
INTS10	9,25	9,59	9,86
INTS12	8,90	9,08	9,04
INTS2	7,66	7,60	6,87
INTS4	7,15	7,80	7,85
INTS5	7,60	7,33	6,64
INTS6	9,55	9,46	9,11
INTS7	5,96	6,08	5,98
INTS8	9,19	8,71	10,15
INVS	4,21	4,25	3,72
IPO11	7,82	8,22	7,15
IPO13	8,93	8,12	7,12
IPO9	7,41	7,39	7,54
IPPK	5,88	5,71	3,85
IQCB1	7,73	7,69	8,13
IQCC	4,56	4,30	3,42
IQCG	8,19	8,17	6,77
IQCH	3,23	3,24	3,73
IQGAP2	8,33	9,31	9,86
IQGAP3	6,67	10,67	4,66
IQSEC2	6,57	6,55	7,02
IQWD1	9,64	9,96	9,32
IRAK1	10,62	10,39	10,46
IRAK4	6,20	5,86	7,67
IREB2	8,14	7,71	8,28
IRF1	9,21	9,71	11,63
IRF2	6,04	5,94	6,57
IRF2BP1	5,36	5,43	6,01
IRF2BP2	9,02	8,65	10,03
IRF3	7,90	7,64	8,32
IRF4	10,17	4,58	6,82
IRF5	6,57	6,31	7,81
IRF8	11,00	11,35	11,60
IRGQ	3,23	3,49	2,51
IRS1	-3,32	-3,32	2,72
IRX1	7,89	7,91	7,69
IRX2	3,36	3,34	3,80
IRX6	3,81	3,52	2,21
ISG20L2	8,06	7,78	8,68
ISGF3G	10,66	10,31	11,07
ISOC1	9,62	9,56	8,01
ISOC2	8,76	9,37	7,11
ISYNA1	7,53	5,23	2,82
ITFG1	10,16	10,24	9,29
ITFG2	8,27	7,43	8,88
ITGA11	6,33	3,70	-3,32
ITGA2	4,28	4,33	4,40
ITGA3	6,48	7,66	-3,32
ITGA4	6,09	5,95	9,09

ITGA5	11,11	10,99	10,24
ITGA7	3,91	2,81	3,86
ITGAL	7,57	9,09	10,41
ITGAM	12,74	12,49	11,59
ITGAV	9,19	9,42	8,03
ITGAX	7,29	7,38	6,76
ITGB1	11,02	10,93	10,06
ITGB1BP1	10,31	11,43	9,24
ITGB1BP2	4,17	4,35	5,24
ITGB1BP3	4,38	5,14	0,20
ITGB2	14,22	14,55	14,34
ITGB3	6,17	6,25	6,04
ITGB3BP	6,41	6,58	6,50
ITGB4	3,81	4,30	3,15
ITGB7	0,20	4,23	6,69
ITIH5L	4,14	3,12	3,67
ITM2B	13,07	13,21	13,52
ITM2C	4,13	3,20	4,86
ITPA	8,96	9,09	8,46
ITPK1	10,35	9,46	10,10
ITPKA	4,85	4,59	4,13
ITPKB	8,90	8,61	7,67
ITPKC	3,55	4,15	3,54
ITPR1	8,54	10,01	8,70
ITPR2	8,04	8,43	7,54
ITPR3	4,25	4,98	6,55
ITSN1	6,32	6,05	7,11
IVL	2,71	3,52	2,85
IVNS1ABP	9,37	9,47	9,83
IWS1	9,14	9,17	9,18
IZUMO1	4,07	4,21	3,59
JAG1	6,82	5,86	5,66
JAK2	8,41	7,49	9,52
JAK3	-0,66	-2,60	4,23
JAKMIP2	7,36	10,74	4,52
JARID1A	9,38	9,11	9,66
JARID1B	8,90	8,51	8,88
JARID2	9,64	9,66	10,49
JAZF1	8,88	9,06	11,40
JDP2	6,58	6,43	8,43
JMJD1A	8,20	7,97	8,23
JMJD2C	5,09	4,76	6,30
JMJD2D	4,20	4,84	2,95
JMJD4	5,59	5,41	4,79
JMJD5	4,51	4,02	3,71
JOSD1	9,88	10,06	9,85
JOSD2	6,82	6,93	6,93
JPH2	4,58	5,11	5,20
JPH3	4,06	3,33	3,23
JPH4	3,67	4,25	4,23
JRKL	4,72	4,60	4,68
JTB	6,64	6,70	6,67
JTV1	10,22	10,19	9,88
JUB	2,29	2,98	2,33
JUN	7,76	7,35	5,54
JUNB	-0,95	-1,73	2,51
JUND	13,56	13,24	13,66
KAL1	4,80	7,95	-3,32
KARS	8,84	8,90	9,20

KATNAL1	5,36	5,63	3,13
KATNAL2	4,71	4,71	-0,45
KATNB1	6,75	7,23	7,15
KBTBD11	7,09	7,24	10,39
KBTBD2	9,57	9,69	10,24
KBTBD3	4,95	5,25	5,00
KBTBD6	4,57	4,04	4,00
KBTBD8	9,87	9,72	6,82
KCMF1	4,01	4,27	4,24
KCNA3	3,07	3,73	4,52
KCNAB1	5,38	6,80	2,71
KCNAB2	5,57	5,84	4,88
KCNAB3	2,20	3,24	4,31
KCNC4	3,59	3,57	3,77
KCNE1L	5,81	5,70	5,44
KCNE3	0,98	-0,07	5,19
KCNG1	3,29	3,16	3,29
KCNG4	2,66	2,16	1,71
KCNH3	3,88	-1,27	7,26
KCNH6	2,14	3,39	3,06
KCNIP1	4,78	4,89	4,40
KCNJ10	7,74	7,88	7,72
KCNJ11	5,14	5,27	4,84
KCNJ2	6,17	7,18	5,10
KCNJ4	4,37	4,32	4,40
KCNJ5	4,36	6,87	-3,32
KCNK13	10,10	8,06	6,72
KCNK3	6,04	5,99	6,24
KCNK5	4,48	4,11	4,19
KCNK6	10,92	9,47	9,10
KCNK9	4,84	4,88	5,00
KCNMA1	4,67	6,29	4,36
KCNMB1	6,54	8,19	8,00
KCNMB2	1,04	2,20	-1,36
KCNMB4	2,96	2,30	5,23
KCNN4	10,02	10,36	7,86
KCNQ1	3,93	3,53	3,97
KCNS3	3,62	4,28	2,98
KCTD10	8,62	8,42	7,65
KCTD12	10,43	11,10	11,98
KCTD13	7,64	7,55	7,10
KCTD17	10,43	9,41	9,61
KCTD18	7,90	8,18	8,33
KCTD2	6,14	6,15	6,07
KCTD3	7,78	7,41	7,66
KCTD5	9,97	10,10	9,02
KCTD6	8,46	6,82	7,66
KCTD7	4,13	3,27	3,58
KCTD8	4,06	4,17	4,74
KCTD9	8,11	8,35	6,61
KDELR1	6,38	6,33	6,53
KDR	4,73	4,00	4,55
KHDRBS1	8,68	8,67	9,59
KHDRBS3	5,31	5,10	3,91
KHK	3,32	3,79	3,04
KIAA0020	8,48	8,26	8,44
KIAA0040	5,81	4,79	6,65
KIAA0090	8,29	8,42	7,63
KIAA0100	12,08	10,54	10,37

KIAA0101	3,50	7,79	2,97
KIAA0133	5,59	5,68	6,40
KIAA0141	7,59	7,59	8,94
KIAA0152	10,69	10,93	9,14
KIAA0174	11,76	11,52	11,50
KIAA0179	9,31	6,96	6,49
KIAA0182	9,38	8,73	9,72
KIAA0195	9,31	9,40	9,46
KIAA0196	10,43	10,79	10,08
KIAA0232	6,87	6,33	6,84
KIAA0241	7,41	7,61	6,52
KIAA0247	9,99	10,03	10,12
KIAA0251	4,70	5,31	4,20
KIAA0256	8,65	8,94	7,97
KIAA0265	5,20	5,27	4,59
KIAA0284	2,52	3,66	2,62
KIAA0286	7,96	7,52	6,60
KIAA0319L	6,50	6,42	7,69
KIAA0323	8,41	8,45	8,12
KIAA0329	7,60	7,62	7,62
KIAA0372	9,75	9,87	9,56
KIAA0391	9,11	9,33	8,14
KIAA0406	5,48	5,65	6,00
KIAA0408	5,18	4,35	6,39
KIAA0409	8,61	8,55	8,52
KIAA0423	8,05	7,50	7,23
KIAA0427	5,84	5,39	6,69
KIAA0460	6,55	5,98	6,93
KIAA0494	10,01	9,93	10,68
KIAA0513	8,49	8,84	10,13
KIAA0556	8,31	8,13	8,62
KIAA0586	6,46	7,25	5,81
KIAA0644	5,03	4,40	4,84
KIAA0649	6,46	5,48	5,47
KIAA0652	6,61	6,67	7,08
KIAA0664	7,61	7,08	6,44
KIAA0701	6,66	6,36	7,50
KIAA0738	2,11	3,18	0,62
KIAA0746	9,63	10,84	5,80
KIAA0753	6,22	6,29	6,69
KIAA0776	7,45	7,89	7,43
KIAA0802	4,05	-0,24	2,27
KIAA0828	7,56	7,16	7,76
KIAA0831	9,35	8,78	9,49
KIAA0859	8,42	8,66	7,15
KIAA0892	7,60	7,29	8,65
KIAA0907	8,89	8,45	9,01
KIAA0922	5,60	5,75	8,79
KIAA0999	8,75	8,80	11,22
KIAA1009	4,45	5,30	5,07
KIAA1012	9,84	9,81	10,04
KIAA1024	4,71	3,69	3,25
KIAA1026	4,56	4,49	4,35
KIAA1033	8,69	8,96	9,66
KIAA1143	7,32	7,31	7,92
KIAA1160	8,06	8,13	8,67
KIAA1161	6,41	2,46	-3,32
KIAA1191	9,26	9,18	9,09
KIAA1199	-2,48	5,05	1,32

KIAA1219	7,84	7,65	8,19
KIAA1267	8,09	7,90	10,07
KIAA1274	7,10	5,38	5,26
KIAA1279	9,06	8,79	6,20
KIAA1328	4,46	4,73	5,12
KIAA1333	6,01	5,78	6,52
KIAA1344	5,25	4,98	6,19
KIAA1370	7,43	8,16	8,10
KIAA1407	5,07	4,81	4,85
KIAA1429	6,86	7,24	7,48
KIAA1446	4,64	4,88	4,34
KIAA1467	5,81	6,10	4,46
KIAA1505	6,90	5,59	5,47
KIAA1524	4,37	5,65	4,03
KIAA1530	6,27	5,60	6,69
KIAA1539	9,98	9,56	9,80
KIAA1542	9,86	9,65	9,62
KIAA1586	3,98	3,93	5,07
KIAA1598	11,48	11,42	11,25
KIAA1600	8,91	9,05	9,41
KIAA1604	6,31	6,01	6,95
KIAA1627	4,94	4,68	4,97
KIAA1704	6,34	6,75	6,41
KIAA1706	6,97	7,09	5,95
KIAA1715	5,53	5,55	5,09
KIAA1727	2,88	2,91	4,16
KIAA1729	8,44	7,99	8,17
KIAA1737	9,74	9,06	7,89
KIAA1754	9,25	10,03	9,95
KIAA1754L	5,70	2,93	-3,32
KIAA1787	6,23	5,50	5,71
KIAA1799	3,23	4,13	4,21
KIAA1804	5,18	4,09	3,85
KIAA1822	4,26	4,55	3,74
KIAA1826	8,95	8,54	7,34
KIAA1958	4,17	4,54	5,56
KIAA1984	4,54	4,29	5,04
KIAA2010	4,82	4,66	6,84
KIF11	3,17	6,78	3,73
KIF13A	4,55	4,85	6,26
KIF13B	8,51	8,31	8,88
KIF14	3,50	6,04	4,12
KIF15	-0,83	5,42	-3,32
KIF17	5,39	5,44	3,22
KIF18A	3,30	3,71	3,96
KIF1B	9,23	9,13	8,27
KIF1C	6,66	6,34	5,64
KIF20A	-0,75	6,88	-3,32
KIF21A	5,97	7,26	5,09
KIF24	3,60	4,56	2,18
KIF2C	2,37	5,95	-0,62
KIF4A	1,72	5,18	3,00
KIF5B	9,47	8,87	8,54
KIFAP3	7,83	7,44	6,81
KIFC1	4,18	3,87	4,54
KIFC3	5,13	4,61	-3,32
KIR2DS3	4,89	4,46	5,02
KIR2DS4	4,49	4,38	3,80
KIRREL2	4,08	4,03	3,79

KIRREL3	4,58	4,13	4,57
KL	2,97	3,18	5,34
KLC2	3,69	3,52	3,02
KLC3	2,90	3,12	3,48
KLC4	2,74	2,87	2,85
KLF10	4,93	5,68	9,19
KLF11	9,01	8,05	10,57
KLF12	2,54	1,44	5,92
KLF13	9,85	9,46	11,48
KLF15	4,89	4,86	5,05
KLF2	8,46	9,00	12,92
KLF3	5,08	4,76	4,99
KLF4	8,43	7,46	9,73
KLF5	6,03	1,78	3,38
KLF6	11,22	11,02	11,45
KLF7	4,25	4,11	5,02
KLF9	10,25	11,01	10,93
KLHDC1	6,48	6,24	6,81
KLHDC2	9,29	9,38	9,86
KLHDC3	10,76	10,67	10,84
KLHDC4	6,93	6,92	7,68
KLHDC5	8,60	8,80	8,19
KLHDC8B	4,71	5,25	4,50
KLHL15	4,76	4,69	6,84
KLHL17	4,58	4,40	5,10
KLHL18	7,58	7,44	8,44
KLHL2	9,46	9,96	9,19
KLHL20	7,40	7,36	6,79
KLHL21	8,08	7,74	7,94
KLHL24	7,31	6,80	7,94
KLHL25	5,28	5,05	5,37
KLHL3	7,53	6,41	4,33
KLHL5	10,12	10,08	10,01
KLHL6	8,36	9,20	6,91
KLHL7	6,85	6,68	6,59
KLHL8	7,57	7,94	9,18
KLK1	3,13	2,37	4,47
KLK10	3,78	3,74	4,32
KLK15	3,73	3,63	4,35
KLK4	5,85	7,43	-3,69
KLK7	3,71	4,62	2,90
KLK8	3,23	2,64	3,20
KLRG1	7,38	7,15	5,85
KMO	8,13	8,43	7,56
KNTC1	6,89	7,65	6,17
KPNA1	8,96	9,14	8,80
KPNA3	9,84	9,63	9,41
KPNA4	10,84	10,21	9,91
KPNA5	5,23	4,67	4,18
KPNA6	8,59	8,51	8,84
KPNB1	11,86	11,74	11,44
KPTN	4,66	4,58	5,62
KREMEN1	3,76	4,42	4,14
KREMEN2	5,39	5,50	5,69
KRIT1	7,05	6,69	7,41
KRT10	8,36	8,94	9,33
KRT23	-1,85	1,03	3,62
KRT24	4,44	4,50	4,46
KRT3	4,72	4,95	5,13

KRT6B	3,88	3,81	3,82
KRTAP10-2	4,05	4,75	4,31
KRTAP12-1	2,41	3,60	3,64
KRTAP12-4	3,92	3,75	3,45
KRTAP3-2	3,74	3,51	4,34
KRTAP5-1	5,53	5,86	6,06
KRTAP9-3	3,89	3,66	3,39
KRTAP9-4	4,32	4,12	4,24
KRTCAP2	11,57	11,67	11,04
KTI12	8,30	8,30	8,49
KTN1	7,58	6,15	8,00
KYNU	11,30	12,29	11,37
KUA	6,30	6,12	6,61
KUA-UEV	6,71	6,56	6,02
L1CAM	3,92	3,37	3,64
L2HGDH	5,82	6,66	4,17
L3MBTL	2,90	2,91	1,21
L3MBTL3	5,22	5,22	7,68
LACE1	6,87	7,00	5,72
LACTB	11,01	10,93	10,27
LACTB2	9,14	9,61	8,24
LAIR1	6,49	8,25	7,12
LAIR2	5,60	7,78	5,60
LAMA1	3,15	3,07	1,96
LAMA2	4,18	4,46	4,15
LAMA3	2,42	3,95	2,81
LAMB1	4,66	-0,60	-0,01
LAMB2	7,13	7,17	6,24
LAMC1	8,65	8,77	6,64
LAMC2	3,16	3,45	3,30
LAMP2	11,52	11,85	10,83
LAMP3	11,06	5,17	4,33
LANCL1	7,79	8,00	8,37
LANCL2	6,30	5,99	6,62
LAPTM4A	10,15	10,24	10,21
LAPTM4B	6,47	7,52	5,16
LAPTM5	12,04	13,13	13,08
LARGE	4,56	5,00	2,74
LARP1	10,84	10,42	10,67
LARP2	6,79	6,44	6,84
LARP4	5,75	5,34	4,86
LARS	9,93	9,78	9,79
LARS2	6,98	7,33	5,70
LAS1L	6,88	7,28	7,83
LASP1	13,34	13,74	13,00
LASS2	9,29	9,71	10,00
LASS4	3,88	4,81	5,78
LASS5	9,26	9,57	8,87
LATS1	4,11	3,88	5,45
LAYN	4,42	6,22	-1,11
LBH	1,75	2,71	1,26
LBP	3,03	3,82	3,93
LBR	9,90	10,19	10,78
LBXCOR1	3,26	3,78	3,25
LCE1A	2,50	3,94	3,00
LCMT1	8,38	8,47	7,79
LCMT2	6,78	6,67	5,13
LCN1	3,71	4,35	3,12
LCP1	13,90	14,03	13,93

LCP2	10,17	11,48	11,87
LDB1	4,69	4,61	4,62
LDHA	13,33	13,18	12,69
LDHB	11,31	11,55	10,99
LDLR	11,18	11,39	9,23
LDLRAD2	3,85	2,65	2,25
LDLRAD3	5,02	6,23	8,43
LELP1	5,19	4,99	4,50
LEMD3	8,63	8,68	9,10
LENG1	5,94	5,80	6,01
LENG4	8,62	9,23	10,33
LENG8	5,56	5,66	6,83
LENG9	4,27	-1,15	3,72
LEO1	8,21	8,23	6,95
LEPR	0,25	3,00	1,84
LEPRE1	8,26	7,56	7,97
LEPREL1	3,80	3,50	4,14
LEPREL2	6,05	4,57	4,89
LEPROT	10,84	11,40	10,83
LETMD1	7,58	7,60	8,68
LGALS12	3,30	4,98	7,33
LGALS2	7,96	9,74	11,24
LGALS3	12,57	12,22	10,79
LGALS4	3,24	1,40	2,85
LGALS9	8,15	8,62	8,14
LGI2	3,92	2,95	3,30
LGICZ1	4,59	4,35	3,63
LGMN	9,72	11,02	5,99
LGR4	8,13	7,42	5,72
LGR6	2,96	3,55	2,93
LHB	3,73	4,40	4,35
LHFP	6,53	4,90	4,84
LHFPL1	4,45	4,47	4,14
LHFPL2	9,76	11,10	7,30
LHX2	4,54	4,91	-1,84
LHX6	4,44	3,84	4,64
LIAS	7,72	7,23	7,39
LIG1	5,60	6,43	4,22
LIG3	4,86	4,95	5,39
LIG4	4,68	4,67	4,37
LILRA1	4,43	4,17	6,66
LILRA2	11,22	11,58	12,39
LILRA3	9,87	10,68	11,35
LILRA5	3,81	6,77	9,26
LILRA6	5,34	5,91	6,34
LILRB1	7,42	7,03	8,89
LILRB2	4,19	4,39	6,02
LILRB3	10,34	11,17	12,35
LILRB4	5,13	5,87	4,55
LILRB5	3,97	5,00	2,38
LIMA1	9,42	8,69	4,66
LIMD1	4,39	4,21	4,97
LIMD2	4,12	3,35	6,03
LIME1	3,35	3,37	4,45
LIMK2	4,70	5,29	5,60
LIMS1	11,62	10,95	9,33
LIMS2	7,34	6,19	4,55
LIN7A	3,33	5,07	5,81
LIN7C	6,83	7,06	6,93

LIN9	6,00	6,42	5,71
LINS1	6,02	6,15	5,83
LIPA	14,85	14,54	12,32
LIPG	3,93	4,56	3,26
LIPT1	5,42	5,05	6,47
LITAF	13,84	13,80	12,43
LIX1L	7,22	6,99	7,19
LMAN1	7,60	7,75	7,19
LMAN2	7,45	7,41	7,45
LMAN2L	8,87	9,31	7,85
LMBR1	8,73	8,61	7,97
LMBR1L	9,35	8,40	8,65
LMBRD2	5,30	5,81	4,65
LMLN	5,42	5,27	2,68
LMNA	9,80	9,83	7,01
LMNB1	6,08	7,48	9,32
LMO2	11,19	10,86	12,10
LMO4	9,68	10,14	10,10
LMOD1	5,20	5,09	4,54
LMTK2	3,57	4,38	6,14
LMX1B	3,55	4,26	4,26
LNPEP	6,27	7,03	5,77
LNX2	6,48	5,64	5,90
LOC113386	7,84	7,75	7,42
LOC123688	5,14	5,58	5,08
LOC124216	4,60	4,11	5,88
LOC124446	10,43	10,55	10,48
LOC124512	9,34	9,37	9,25
LOC128977	6,75	6,60	6,47
LOC129607	-1,57	0,27	4,69
LOC130074	8,52	7,95	8,08
LOC130355	7,55	7,75	7,61
LOC130951	5,65	6,72	4,69
LOC134145	7,54	7,46	7,18
LOC143188	4,95	4,53	4,45
LOC143941	3,85	4,29	2,01
LOC144097	5,75	5,93	5,76
LOC148137	5,28	5,35	5,82
LOC150051	3,97	1,25	4,23
LOC150383	3,60	2,26	4,29
LOC152485	6,42	4,40	-0,08
LOC153222	8,52	7,47	9,56
LOC153328	5,42	2,45	-0,91
LOC153364	4,68	4,98	5,16
LOC153684	4,08	3,01	5,20
LOC155060	0,48	3,41	4,65
LOC158572	4,42	4,91	4,65
LOC196752	7,11	6,25	8,28
LOC198437	-0,67	2,10	4,49
LOC201725	4,98	5,00	6,42
LOC203547	10,14	10,17	10,58
LOC205251	8,49	8,71	7,73
LOC220686	7,39	7,38	7,18
LOC222699	5,62	5,81	5,71
LOC257358	3,20	4,29	3,30
LOC283392	-0,84	3,66	4,29
LOC283874	6,76	6,87	7,49
LOC283932	6,21	5,84	5,71
LOC284009	5,24	5,52	5,44

LOC284296	4,19	4,77	4,04
LOC285016	3,71	4,33	4,40
LOC285074	5,43	4,98	6,68
LOC285636	10,60	9,83	9,42
LOC286016	10,45	10,41	10,15
LOC286187	5,57	5,16	5,38
LOC339229	5,97	5,33	6,00
LOC339344	9,49	8,95	10,07
LOC339745	7,95	8,18	9,79
LOC342897	6,47	2,99	3,90
LOC374395	11,51	11,75	11,89
LOC374920	4,31	4,44	4,17
LOC387790	4,15	3,34	3,54
LOC387882	3,21	4,41	4,13
LOC388284	5,95	5,14	6,54
LOC388438	4,04	3,49	3,69
LOC388564	6,99	6,87	6,79
LOC388610	3,69	3,02	5,55
LOC388969	6,06	5,19	6,87
LOC389118	3,75	3,30	3,86
LOC389791	5,27	3,52	4,39
LOC389833	4,51	4,74	4,40
LOC390637	4,01	4,25	3,21
LOC391356	7,05	7,18	8,11
LOC399818	5,19	4,81	5,19
LOC399900	0,58	3,01	7,33
LOC400027	8,01	8,18	9,85
LOC400451	3,50	3,47	5,00
LOC400464	5,42	5,05	5,18
LOC400506	5,49	5,61	5,07
LOC400566	7,49	7,30	7,08
LOC400657	8,01	7,83	6,37
LOC400707	3,72	4,86	4,45
LOC400708	3,71	4,69	3,64
LOC401019	13,73	13,64	13,99
LOC401152	7,94	7,38	8,51
LOC401233	7,85	7,56	7,72
LOC401398	4,62	4,09	2,48
LOC401431	4,28	4,69	3,15
LOC401720	5,32	5,14	5,06
LOC439985	4,02	3,93	3,25
LOC440093	10,43	10,41	11,28
LOC440258	5,20	5,34	6,74
LOC440354	9,13	8,63	9,90
LOC440731	6,56	7,08	8,22
LOC440836	3,33	3,20	5,57
LOC440944	5,01	4,94	5,35
LOC441087	13,06	12,66	13,73
LOC441135	3,89	3,59	4,42
LOC441136	3,89	4,38	4,04
LOC441150	5,64	5,42	6,70
LOC441193	4,01	3,35	5,38
LOC441208	4,19	4,37	4,24
LOC441268	5,76	6,92	9,45
LOC441461	3,94	3,44	3,33
LOC441763	11,02	11,22	10,76
LOC442535	4,45	5,14	4,03
LOC442582	6,74	6,39	8,01
LOC442597	3,41	3,01	-1,49

LOC493869	3,52	0,47	-3,32
LOC51035	10,58	10,15	11,24
LOC51057	4,79	4,21	4,91
LOC51136	9,08	9,44	7,87
LOC51252	4,17	0,96	2,39
LOC54103	8,45	8,04	8,65
LOC552891	8,39	8,31	6,97
LOC55565	5,84	5,58	5,13
LOC56964	4,12	4,61	2,95
LOC606495	3,74	4,60	4,26
LOC63920	5,92	5,31	1,58
LOC63928	3,89	4,44	3,22
LOC642420	3,96	4,08	3,47
LOC643011	5,06	5,00	4,45
LOC643152	3,60	4,75	2,60
LOC643339	0,78	-0,21	-3,32
LOC643396	5,30	0,77	-0,07
LOC643493	3,50	3,27	5,32
LOC644001	3,84	3,18	3,81
LOC644011	5,51	5,57	5,20
LOC644733	3,84	3,57	3,74
LOC644760	4,59	4,35	4,99
LOC644869	6,35	6,58	5,59
LOC644923	4,04	3,25	2,55
LOC645052	-3,32	-3,32	4,96
LOC645676	4,70	4,22	4,94
LOC646407	-1,21	-2,63	1,81
LOC646496	1,10	2,97	0,50
LOC646574	3,16	2,41	3,40
LOC646632	4,26	4,08	2,25
LOC646881	4,16	3,88	4,04
LOC646897	5,85	5,32	6,68
LOC647115	5,27	5,68	4,64
LOC647243	3,80	4,29	1,34
LOC653240	2,89	3,05	2,28
LOC653352	3,23	3,42	4,68
LOC653604	4,49	3,69	5,36
LOC653610	4,78	5,18	8,18
LOC653696	3,77	3,15	4,00
LOC81691	5,11	6,12	-0,07
LOC90624	6,25	6,79	6,03
LOC90826	7,34	7,87	5,99
LOC90835	4,72	5,06	5,09
LOC92017	9,35	8,43	8,94
LOC92345	6,19	6,86	5,75
LOH11CR2A	8,85	8,98	5,90
LOH12CR1	5,45	4,90	3,72
LONRF3	5,84	7,22	4,82
LOR	4,83	-3,32	-0,62
LOXL1	3,03	2,31	3,06
LOXL3	6,78	6,98	6,82
LPGAT1	8,13	7,98	9,51
LPHN1	3,61	4,22	3,94
LPIN2	7,74	7,77	7,69
LPL	10,90	12,71	5,74
LPPR4	2,37	0,27	2,11
LPXN	12,93	13,32	12,00
LRAP	7,48	7,84	8,52
LRBA	4,36	4,97	4,68

LRCH4	7,02	7,20	10,08
LRFN4	7,93	7,18	6,65
LRG1	5,66	5,32	7,25
LRIG1	4,14	0,07	3,60
LRIG2	6,42	6,35	8,26
LRMP	9,02	8,59	10,28
LRP1	7,57	8,01	8,36
LRP10	11,21	10,90	10,65
LRP12	5,14	5,02	3,91
LRP2	4,40	3,51	4,54
LRPAP1	10,10	10,72	10,05
LRPPRC	8,18	7,87	8,12
LRRC1	6,79	3,24	4,37
LRRC14	6,99	6,64	6,89
LRRC15	7,90	7,75	7,57
LRRC16	4,20	5,92	3,99
LRRC17	2,13	3,45	1,09
LRRC23	6,56	7,26	6,67
LRRC25	6,48	6,87	9,70
LRRC28	7,35	6,73	7,65
LRRC29	5,89	5,86	5,88
LRRC3	5,66	4,90	4,10
LRRC32	5,25	4,55	4,30
LRRC33	10,79	10,50	9,65
LRRC34	4,04	4,86	1,13
LRRC40	6,51	6,50	6,32
LRRC41	9,40	9,33	8,92
LRRC42	8,62	8,82	7,94
LRRC46	4,08	3,55	4,20
LRRC4C	3,19	4,37	2,72
LRRC51	4,85	4,82	4,74
LRRC57	7,55	7,46	7,37
LRRC6	4,92	5,40	7,49
LRRC61	4,30	3,01	1,86
LRRC8A	7,41	8,48	6,12
LRRC8B	5,55	4,85	3,72
LRRC8C	9,76	9,09	9,07
LRRC8D	9,84	10,44	10,97
LRRCC1	2,31	3,14	3,95
LRRFIP1	10,13	9,34	10,59
LRRFIP2	8,10	7,98	8,90
LRRIQ2	3,84	4,23	4,65
LRRK1	7,63	5,94	6,05
LRRK2	6,71	6,73	10,19
LRSAM1	7,54	7,12	7,29
LSAMP	4,10	4,15	3,86
LSG1	6,63	6,69	6,68
LSM1	9,43	9,14	9,00
LSM10	9,72	9,69	10,14
LSM14A	10,14	9,80	11,51
LSM2	10,65	10,72	10,23
LSM3	10,51	11,04	10,76
LSM4	10,26	10,63	9,89
LSM5	10,96	10,80	10,58
LSM6	8,43	8,31	9,01
LSM7	10,16	9,94	10,86
LSM8	7,18	6,64	7,21
LSMD1	11,12	11,18	11,36
LSP1	7,53	7,75	8,10

LSR	4,63	4,35	3,56
LSS	7,40	7,85	5,82
LST1	8,67	9,42	11,49
LTA4H	12,18	14,30	13,93
LTB	4,55	5,04	7,33
LTB4DH	8,85	9,86	1,99
LTB4R	7,00	7,38	10,08
LTB4R2	2,83	2,91	5,51
LTBP1	3,19	3,39	3,29
LTBP3	5,01	6,12	4,61
LTF	4,08	3,13	3,72
LTK	2,20	2,30	3,54
LTV1	10,75	10,24	9,83
LUC7L2	6,49	6,50	6,97
LUZP1	7,93	7,79	6,62
LXN	8,20	9,35	6,53
LY6E	10,03	11,09	10,89
LY6G5C	4,32	4,43	4,76
LY75	9,45	6,19	7,18
LY86	10,10	11,18	12,41
LY96	12,22	12,28	11,84
LYAR	8,18	8,12	8,83
LYCAT	5,72	6,44	6,36
LYK5	9,40	9,05	8,66
LYN	11,73	11,58	13,26
LYPD1	4,27	1,86	-0,90
LYPD3	7,24	3,50	4,26
LYPLA2	7,80	7,33	7,57
LYPLA3	9,28	9,85	7,30
LYPLAL1	8,45	9,17	7,50
LYSMD2	7,97	9,78	11,04
LYSMD3	7,48	7,41	7,30
LYST	8,30	7,54	10,04
LYZ	13,45	14,75	15,01
LZIC	7,87	8,17	7,46
LZTFL1	5,54	5,46	6,84
LZTR1	9,77	9,42	9,82
M6PR	12,71	12,60	11,21
MACF1	8,97	8,39	8,76
MAD1L1	8,44	8,47	7,82
MAD2L1	4,65	7,38	2,25
MAD2L1BP	7,78	7,55	7,05
MAD2L2	9,93	9,91	10,09
MADD	6,70	7,07	7,37
MAEA	6,16	5,85	6,06
MAF	9,06	7,52	5,42
MAF1	7,54	7,33	8,14
MAFB	11,06	9,65	11,13
MAFF	7,28	6,78	0,28
MAFG	7,02	6,27	6,49
MAGEA2	3,64	2,35	0,08
MAGEB1	4,65	4,19	4,08
MAGED1	8,75	10,04	7,87
MAGED2	6,31	5,44	5,55
MAGEF1	5,01	4,61	5,33
MAGEH1	4,23	5,86	6,12
MAGI1	1,02	2,50	3,17
MAGOH	10,08	10,01	10,91
MAK	3,68	3,77	5,67

MALT1	8,67	8,20	9,16
MAML3	4,81	5,23	5,59
MAN1A1	9,53	9,36	7,83
MAN1A2	7,11	6,77	6,73
MAN1B1	9,77	9,76	9,26
MAN2A1	11,79	10,92	11,20
MAN2A2	6,34	5,85	8,80
MAN2B1	10,47	10,16	9,87
MAN2B2	11,34	11,32	11,29
MAN2C1	9,54	9,27	10,18
MANBA	10,15	10,46	11,10
MANEA	6,06	6,19	4,96
MANEAL	4,77	4,05	-0,64
MAOA	12,36	6,52	3,43
MAOB	4,00	3,27	1,75
MAP1A	7,89	6,75	4,55
MAP1LC3A	3,86	3,67	2,90
MAP1LC3C	3,32	3,80	-0,24
MAP2K1	11,23	11,33	11,55
MAP2K1IP1	11,30	11,15	10,27
MAP2K4	8,70	8,55	8,44
MAP2K5	6,40	6,29	6,87
MAP2K6	5,58	4,77	5,06
MAP2K7	6,12	5,79	6,48
MAP3K10	4,40	4,66	5,55
MAP3K11	9,02	9,19	9,46
MAP3K12	6,25	6,04	6,56
MAP3K3	6,70	7,09	8,27
MAP3K4	8,92	7,28	7,84
MAP3K7	9,87	10,13	10,37
MAP3K7IP1	6,19	5,89	6,36
MAP3K8	8,33	8,98	9,03
MAP3K9	3,39	3,70	0,64
MAP4K1	8,88	5,38	9,93
MAP4K2	10,64	10,88	11,15
MAP4K3	7,40	7,51	6,43
MAP4K5	8,39	7,89	8,57
MAP6	3,31	1,35	3,22
MAP7	7,02	9,26	6,83
MAPBPIP	11,05	11,36	10,43
MAPK1	9,41	8,30	9,74
MAPK14	5,37	5,85	6,19
MAPK3	10,28	10,81	11,46
MAPK7	5,84	4,74	7,16
MAPK8IP1	2,08	2,21	0,87
MAPK8IP2	4,17	3,89	4,15
MAPK8IP3	7,58	6,87	9,06
MAPKAP1	7,65	8,33	6,90
MAPKAPK2	7,46	7,63	7,87
MAPKAPK3	12,26	12,17	12,07
MAPKAPK5	8,50	8,40	8,11
MAPKBP1	5,58	4,86	4,72
MAPRE2	9,69	11,01	10,78
MAPRE3	5,85	8,03	5,40
MARCKS	12,73	11,58	12,57
MARCO	7,74	11,45	8,14
MARK2	5,40	4,87	6,21
MARS	9,48	8,94	9,35
MARS2	6,95	6,79	6,20

MARVELD1	8,39	8,39	8,77
MARVELD2	4,85	3,66	3,44
MAS1	4,61	4,69	3,20
MASK-BP3	6,08	5,40	6,45
MAST2	6,72	7,06	6,10
MASTL	5,83	6,88	5,96
MAT2A	12,72	11,91	11,71
MAT2B	10,98	10,33	10,55
MATK	7,49	8,42	2,83
MATN4	3,43	3,83	3,97
MATR3	11,06	10,75	11,35
MAX	5,52	5,88	6,53
MAZ	4,11	3,86	4,07
MBD1	5,52	5,07	5,22
MBD4	11,48	11,98	11,40
MBD6	8,48	8,04	9,35
MBIP	6,81	7,21	7,45
MBNL1	10,18	9,81	10,81
MBNL2	7,68	7,29	8,04
MBNL3	3,68	4,44	3,80
MBP	10,66	10,82	10,50
MBTPS1	8,73	8,63	9,54
MBTPS2	5,44	5,67	4,07
MC1R	5,98	6,18	3,09
MCART1	12,21	12,05	13,55
MCART6	4,05	4,40	4,53
MCCC1	9,43	9,60	9,45
MCEE	8,42	8,85	8,78
MCFD2	8,40	8,47	8,52
MCHR1	4,33	4,33	3,88
MCL1	8,49	8,55	9,24
MCM10	-1,04	5,96	-0,19
MCM3	6,88	7,76	7,07
MCM3AP	9,89	9,71	9,79
MCM4	6,67	8,40	6,31
MCM5	8,06	8,23	8,18
MCM6	4,58	6,27	-0,51
MCM7	6,93	8,53	8,18
MCOLN1	10,70	10,60	9,13
MCOLN2	10,21	7,15	6,24
MCOLN3	8,45	9,21	-3,32
MCRS1	7,04	6,92	6,98
MCTP1	5,43	7,28	7,43
MCTP2	4,43	4,45	4,97
MCTS1	10,06	10,37	10,11
MDC1	8,80	8,47	7,49
MDGA1	4,31	3,67	4,46
MDH1	14,23	13,60	11,61
MDH2	11,80	11,99	11,27
MDM1	5,97	6,28	6,98
MDM2	5,50	5,46	4,28
MDM4	5,90	5,39	7,98
MDN1	4,62	3,82	4,70
MDP-1	7,90	8,35	7,57
MDS1	4,69	5,49	5,17
ME1	7,51	9,64	6,78
ME2	10,33	10,75	10,15
ME3	5,35	5,79	2,81
MEA1	9,54	9,40	8,74

MECP2	5,76	5,24	5,89
MECR	2,77	3,99	2,87
MED10	9,26	9,50	9,82
MED11	5,81	5,89	6,19
MED12	7,33	6,97	7,17
MED18	5,83	5,33	4,73
MED19	8,01	7,58	7,24
MED25	8,40	8,15	8,14
MED28	10,10	9,52	9,71
MED31	4,34	4,73	4,32
MED4	8,65	8,58	9,44
MED6	10,01	10,28	9,33
MED8	7,68	7,72	7,14
MED9	7,02	7,02	6,15
MEF2B	5,92	5,73	6,52
MEF2C	4,61	5,37	9,59
MEF2D	8,33	8,63	10,33
MEFV	4,86	4,63	9,66
MEIS3	2,72	4,06	4,12
MEOX1	2,76	3,46	0,97
MEP1A	6,75	5,85	1,65
MERTK	5,04	7,30	7,39
MESDC1	7,43	6,53	7,15
MESDC2	4,29	4,50	2,87
MESP1	3,49	5,03	3,25
MEST	2,39	3,26	3,38
MET	4,58	5,15	4,80
METAP2	11,13	11,01	11,02
METRN	3,79	4,09	4,06
METRNL	9,67	8,79	8,95
METT10D	6,42	6,47	6,60
METT5D1	5,55	5,80	5,18
METTL1	7,69	7,76	4,53
METTL2A	5,07	5,24	4,81
METTL2B	3,90	2,64	0,90
METTL3	8,46	8,34	8,77
METTL4	4,79	5,65	5,26
METTL5	9,68	9,84	9,05
METTL6	6,51	7,27	6,47
METTL7A	10,99	8,86	10,65
MFAP1	8,72	8,63	6,42
MFAP3	6,98	7,07	5,94
MFGE8	6,97	9,00	5,86
MFNG	10,89	9,76	11,53
MFRP	2,48	3,19	3,01
MFSD1	12,57	12,48	12,15
MFSD2	7,98	7,95	7,45
MFSD3	9,35	9,05	8,18
MFSD5	9,66	9,54	9,33
MFSD7	8,08	8,79	6,64
MGAT1	12,57	12,09	11,99
MGAT2	8,52	8,24	8,14
MGAT4A	9,54	8,92	8,35
MGAT4B	7,45	7,53	6,92
MGC11102	6,36	5,66	5,95
MGC12966	9,01	8,85	8,29
MGC13057	4,21	4,64	3,48
MGC13379	7,68	7,60	8,63
MGC14327	6,22	6,02	6,36

MGC14376	9,44	9,17	11,48
MGC15523	8,04	8,22	6,75
MGC16169	8,20	8,25	7,05
MGC16824	7,35	7,14	7,55
MGC19604	6,70	6,61	5,13
MGC20983	6,14	0,45	7,22
MGC24039	5,37	6,20	0,25
MGC2752	6,64	6,47	6,59
MGC3207	7,98	7,73	9,16
MGC33556	4,67	6,43	9,26
MGC35361	5,36	5,56	5,44
MGC35440	2,98	1,94	4,07
MGC39900	6,04	4,61	4,28
MGC40499	4,87	5,03	5,77
MGC40574	2,94	3,41	5,08
MGC4093	7,31	7,07	7,71
MGC4172	11,53	8,97	6,00
MGC42105	3,78	1,98	2,90
MGC45491	4,33	3,74	4,16
MGC4677	6,27	5,13	4,98
MGC52000	8,14	7,97	9,20
MGC52110	8,68	8,46	9,14
MGC61571	8,44	8,78	6,65
MGC70857	7,19	6,43	6,90
MGC71993	12,60	12,47	12,03
MGC72104	9,27	9,39	9,50
MGEA5	10,87	10,97	11,71
MGLL	7,51	8,01	4,16
MGMT	9,10	9,44	9,12
MGRN1	8,11	7,57	7,85
MGST1	9,23	9,72	8,93
MGST2	10,74	11,84	11,34
MGST3	12,42	12,84	11,05
MIB1	5,14	4,80	6,41
MICA	8,96	8,66	8,30
MICAL2	5,48	5,50	7,00
MICB	8,62	8,55	9,14
MID1IP1	10,19	9,69	9,98
MID2	7,68	3,99	-0,07
MIDN	9,19	8,63	10,71
MIER1	8,47	8,06	8,61
MIF	10,59	10,44	9,84
MINA	6,05	5,99	6,45
MINK1	4,55	3,53	4,96
MINPP1	8,75	8,66	7,26
MIPEP	7,92	7,86	4,95
MIR16	8,55	8,70	8,58
MIS12	9,31	9,28	8,56
MITF	7,22	7,92	5,13
MIZF	6,76	6,42	7,15
MKI67	4,34	5,58	3,35
MKI67IP	10,00	9,86	9,99
MKKS	8,65	8,39	8,07
MKL1	8,94	7,69	9,22
MKL2	6,22	6,29	5,11
MKLN1	9,22	9,21	10,49
MKNK1	9,35	9,27	8,80
MKRN1	11,42	11,17	12,51
MKRN2	9,06	9,22	9,55

MKS1	4,57	4,79	4,62
MLC1	-0,50	1,62	2,29
MLF1IP	5,57	6,74	5,56
MLF2	9,75	10,26	9,55
MLH1	9,07	9,41	8,81
MLKL	8,29	9,43	10,16
MLL	6,24	5,87	6,88
MLL3	5,97	5,65	7,24
MLL5	5,54	5,47	7,03
MLLT10	7,68	7,19	8,48
MLLT11	6,39	8,05	4,84
MLLT3	8,16	8,25	8,21
MLLT4	3,59	2,67	2,66
MLLT6	9,47	8,25	8,77
MLPH	5,00	7,20	2,11
MLSTD1	10,32	8,49	7,06
MLSTD2	5,88	6,24	8,24
MLXIPL	3,25	4,40	3,72
MLYCD	6,78	7,17	5,61
MMAB	4,00	4,18	3,47
MMD	10,40	9,96	8,23
MME	4,32	4,77	4,27
MMP1	5,79	3,50	-3,32
MMP10	6,64	4,38	-3,32
MMP12	11,84	6,34	4,51
MMP13	4,51	4,64	3,69
MMP14	4,80	5,79	-0,72
MMP15	4,44	4,39	4,49
MMP19	5,67	6,63	3,98
MMP25	7,27	5,15	8,29
MMP7	4,24	10,95	0,35
MMP9	11,95	14,35	6,29
MMPL1	4,58	4,49	4,04
MMS19L	10,20	9,87	9,82
MN1	3,59	3,16	5,12
MNAT1	7,46	7,45	6,32
MND1	-3,32	4,95	-3,32
MNDA	11,64	12,94	13,37
MNS1	0,58	4,42	2,52
MNT	8,85	8,77	9,92
MOAP1	9,20	9,67	9,49
MOBKL1A	9,56	8,66	9,12
MOBKL2A	8,33	7,94	9,62
MOBKL2B	8,24	8,88	5,77
MOBKL2C	7,67	7,32	6,64
MOCOS	8,93	5,45	5,23
MOCS2	6,10	6,14	4,47
MOCS3	4,44	2,95	2,05
MOGAT1	5,73	5,74	5,66
MON1B	7,46	7,63	7,38
MON2	8,22	8,26	8,85
MORC2	8,54	8,29	7,68
MORC4	5,80	5,52	3,63
MORF4L1	10,88	10,70	10,81
MORF4L2	9,68	10,27	9,11
MORG1	7,38	7,61	6,75
MORN2	6,13	7,04	5,55
MOS	7,33	7,44	7,34
MOSC1	6,42	7,91	10,48

MOSC2	7,08	4,85	2,72
MOSPD2	9,52	9,54	9,09
MOSPD3	6,41	6,87	6,77
MOV10	6,96	7,33	7,19
MPDU1	9,61	9,70	8,23
MPHOSPH1	6,42	7,32	6,33
MPHOSPH10	8,97	8,78	8,74
MPHOSPH6	9,46	9,22	8,66
MPI	5,68	5,67	5,62
MPO	6,88	7,09	7,77
MPP1	10,72	11,11	10,68
MPP5	6,19	6,06	6,37
MPP6	5,69	5,94	5,10
MPPE1	7,80	7,85	8,93
MPPED2	4,93	4,09	4,23
MPST	5,10	4,93	5,84
MPV17	10,68	10,90	9,52
MPZL1	7,80	8,33	7,60
MR1	9,71	10,33	8,69
MRAS	8,15	8,56	4,91
MRC1	4,39	1,55	-1,02
MRC2	6,11	4,27	-0,43
MRCL3	13,83	13,25	13,28
MRE11A	5,58	5,99	5,55
MRFAP1	10,99	10,88	11,19
MRFAP1L1	8,73	8,50	7,99
MRGPRF	2,62	5,97	-3,32
MRLC2	12,95	12,82	12,36
MRP63	3,25	2,12	4,57
MRPL1	9,97	10,20	9,56
MRPL11	7,75	7,49	8,44
MRPL12	8,88	8,63	7,89
MRPL13	10,51	10,77	9,51
MRPL14	8,36	8,39	8,06
MRPL15	11,96	11,71	10,77
MRPL16	8,97	9,25	9,43
MRPL17	10,08	10,65	8,90
MRPL18	9,79	9,90	9,56
MRPL19	9,39	9,50	9,03
MRPL2	7,40	7,29	7,50
MRPL20	9,55	9,40	9,41
MRPL21	9,48	9,75	9,42
MRPL22	9,70	10,04	9,42
MRPL24	9,89	10,24	9,78
MRPL27	7,46	7,58	7,03
MRPL3	11,41	10,90	10,50
MRPL30	7,15	7,17	7,31
MRPL32	10,03	9,92	9,62
MRPL33	11,29	11,40	11,53
MRPL35	7,68	7,72	6,28
MRPL36	10,51	10,43	9,74
MRPL37	10,53	11,28	9,64
MRPL39	8,47	9,07	7,70
MRPL40	9,69	10,06	8,71
MRPL41	9,37	8,93	9,21
MRPL42	6,84	7,09	6,87
MRPL43	8,58	8,87	8,53
MRPL44	9,22	9,36	8,97
MRPL46	8,60	8,79	7,83

MRPL47	7,83	8,22	7,57
MRPL48	8,82	9,24	9,57
MRPL49	9,80	9,83	9,10
MRPL50	9,76	9,60	8,26
MRPL51	11,70	11,71	10,97
MRPL52	5,42	5,66	5,82
MRPL53	8,90	9,05	9,32
MRPL54	9,88	10,05	10,04
MRPL55	6,58	6,61	7,11
MRPL9	10,06	9,92	9,59
MRPS10	10,60	10,65	10,13
MRPS11	9,19	9,68	9,28
MRPS12	6,56	6,78	6,42
MRPS14	5,83	5,49	5,72
MRPS15	10,18	10,76	9,70
MRPS16	8,68	9,02	8,70
MRPS18A	7,47	7,33	7,53
MRPS18B	6,96	6,96	6,91
MRPS18C	9,97	10,45	9,57
MRPS2	7,45	7,12	7,91
MRPS21	10,73	10,45	10,81
MRPS22	10,32	10,40	10,35
MRPS23	8,07	8,31	7,67
MRPS24	9,53	9,89	10,14
MRPS25	7,60	7,44	7,53
MRPS26	8,18	7,86	8,19
MRPS27	9,15	9,18	8,93
MRPS28	9,35	9,52	8,74
MRPS30	10,16	10,22	9,86
MRPS31	8,59	8,55	8,80
MRPS33	9,11	9,07	8,46
MRPS34	8,13	7,93	7,99
MRPS35	9,75	9,64	9,31
MRPS36	6,76	6,44	6,13
MRPS5	9,49	9,63	9,71
MRPS7	10,04	10,25	9,32
MRPS9	8,14	7,98	7,91
MRRF	7,96	8,22	7,78
MRS2L	6,91	7,01	6,03
MRVI1	1,53	0,70	3,91
MS4A3	2,59	2,27	2,81
MS4A6A	12,14	10,42	13,33
MS4A7	10,51	12,19	12,24
MSC	8,81	8,55	3,87
MSH2	6,19	6,06	4,89
MSH3	7,48	7,81	8,48
MSH5	4,25	4,04	4,49
MSH6	9,73	9,87	9,49
MSI2	5,06	5,27	5,40
MSL2L1	4,19	4,56	5,05
MSL3L1	7,67	7,78	9,06
MSN	12,11	12,29	13,13
MSR1	7,66	9,47	5,33
MSRA	8,87	9,14	8,59
MST1	6,70	6,53	6,78
MST150	5,72	7,56	7,29
MSTO1	7,11	7,14	7,11
MT1A	6,63	8,71	10,10
MT1B	4,66	4,13	3,82

MT1F	5,58	7,58	9,42
MT1G	2,89	6,94	4,38
MT1X	6,53	7,89	9,32
MT2A	7,14	9,41	11,05
MTA1	7,30	6,81	8,41
MTA2	9,39	8,91	9,75
MTA3	6,31	6,91	5,29
MTAP	7,47	7,73	7,57
MTCH1	12,26	12,19	12,11
MTCP1	10,09	10,39	9,34
MTDH	10,75	10,85	10,97
MTERFD1	8,85	8,70	8,23
MTF1	9,00	9,46	9,27
MTF2	9,27	9,08	9,71
MTFMT	8,38	8,32	7,47
MTFR1	8,32	8,29	7,07
MTHFD1	7,60	8,09	6,55
MTHFR	7,43	7,19	7,91
MTHFS	7,95	10,36	9,59
MTHFSD	4,87	5,32	5,71
MTIF2	8,67	8,84	8,37
MTIF3	9,98	10,08	9,94
MTM1	7,91	7,99	9,02
MTMR1	4,59	5,20	4,43
MTMR10	7,57	7,69	7,67
MTMR2	6,53	7,44	6,56
MTMR4	9,35	9,33	9,00
MTMR9	8,52	8,06	8,95
MTO1	5,23	4,61	5,07
MTP18	9,37	9,01	7,48
MTPN	11,00	11,23	11,81
MTR	9,07	8,56	8,30
MTRF1	8,06	8,18	8,14
MTRF1L	4,44	4,54	3,04
MTRR	8,71	8,30	8,35
MTSS1	5,97	5,02	8,02
MTX1	9,00	9,34	8,59
MTX3	7,21	7,16	6,89
MUC1	5,45	3,82	3,95
MUM1	8,16	8,47	7,90
MUS81	8,06	7,69	8,09
MUT	9,06	9,37	7,93
MUTYH	6,76	6,91	7,89
MVK	7,15	6,65	4,28
MVP	10,18	10,32	10,72
MX1	8,20	10,32	11,84
MX2	0,93	3,68	6,60
MXD1	9,01	8,39	10,89
MXI1	5,02	4,75	5,30
MXRA7	1,82	3,60	1,59
MYADM	8,36	10,11	10,39
MYB	5,93	7,07	7,84
MYBBP1A	4,87	4,42	5,31
MYC	9,34	8,79	7,39
MYCBP	4,95	4,52	5,36
MYCBP2	8,93	9,31	10,59
MYCN	4,00	3,27	3,61
MYD88	8,87	10,90	10,87
MYEF2	5,40	5,30	5,24

MYEOV	-1,46	4,35	2,43
MYEOV2	4,65	4,59	4,58
MYF5	3,51	4,28	3,87
MYH11	4,71	3,49	-0,36
MYH14	5,15	4,72	4,93
MYH9	12,62	12,47	12,90
MYL6	14,50	14,26	13,74
MYL6B	8,38	9,18	7,18
MYL9	5,73	6,80	3,67
MYLIP	10,65	8,87	11,10
MYLK	-0,53	0,10	-0,71
MYNN	7,93	7,32	7,85
MYO10	5,80	6,95	5,67
MYO18A	10,05	8,10	8,17
MYO1B	5,77	6,20	5,18
MYO1C	7,35	6,83	4,14
MYO1D	6,02	7,53	0,45
MYO1E	7,24	6,77	4,15
MYO1F	8,94	8,93	10,53
MYO1G	7,54	6,69	11,83
MYO5A	10,27	10,03	9,22
MYO6	-2,10	4,22	-3,32
MYO7A	3,73	4,73	5,74
MYO9A	7,45	7,10	7,55
MYO9B	9,86	9,89	10,11
MYOD1	3,73	4,25	4,36
MYOHD1	4,05	3,06	3,67
MYOM2	4,23	1,62	3,41
MYOZ1	6,99	8,58	5,33
MYST1	8,46	8,07	9,29
MYST2	5,54	5,24	5,95
MYST3	9,39	8,53	10,34
MYST4	4,01	4,30	5,00
MAGMAS	8,33	8,27	8,37
N-PAC	8,92	8,52	9,12
N4BP1	2,46	0,72	3,21
NAALADL1	4,31	4,77	7,00
NAB1	7,44	7,28	7,59
NAB2	6,09	5,40	5,41
NACA	8,31	8,84	8,30
NADK	8,29	8,65	9,94
NADSYN1	9,63	9,31	9,66
NAG	6,39	6,60	6,89
NAGA	10,05	8,97	10,27
NAGK	12,04	12,10	12,29
NAGLU	9,59	9,70	8,34
NAGPA	11,44	9,97	9,48
NAGS	3,74	4,01	4,59
NANOG	3,69	3,98	2,75
NANP	5,48	5,39	4,10
NANS	10,24	10,42	9,49
NAP1L1	9,43	9,97	10,47
NAP1L2	3,46	4,48	4,13
NAP1L5	5,20	6,05	4,73
NAPA	6,89	6,97	6,78
NAPE-PLD	5,10	5,44	4,29
NAPG	7,73	8,05	7,71
NARF	8,16	7,66	8,70
NARG1	6,43	6,69	6,39

NARG1L	6,64	7,47	7,81
NARG2	4,53	4,26	4,51
NARS2	7,69	7,48	7,07
NASP	4,66	5,21	6,75
NAT1	6,26	6,67	4,20
NAT10	8,52	8,60	8,61
NAT5	11,14	11,32	10,52
NAT6	6,31	4,50	6,53
NAT9	7,70	7,65	8,06
NAV1	6,76	6,84	6,52
NAV2	4,22	4,51	4,42
NBEA	5,78	5,19	4,70
NBL1	4,75	4,99	5,49
NBPF3	1,30	2,49	0,91
NCAM1	4,27	4,37	4,57
NCBP1	9,71	9,57	8,94
NCBP2	10,11	10,24	9,47
NCDN	5,00	5,45	5,79
NCF1	5,78	6,97	11,17
NCF2	11,91	12,31	12,28
NCF4	7,82	8,53	9,60
NCK1	10,39	10,51	8,42
NCK2	9,35	8,51	9,25
NCKAP1	7,92	8,30	3,27
NCKAP1L	11,20	10,98	10,76
NCKIPSD	8,92	8,37	7,30
NCL	7,59	7,60	7,92
NCLN	9,68	10,46	9,34
NCOA2	3,51	3,53	4,98
NCOA3	10,69	9,82	9,92
NCOA4	13,12	13,75	13,92
NCOA5	8,43	7,38	7,37
NCOA6	10,05	9,40	10,57
NCOA7	9,41	9,52	8,90
NCOR1	7,57	7,69	7,91
NCR2	4,38	3,79	3,94
NCSTN	12,14	12,11	11,71
NDE1	9,09	8,77	10,26
NDFIP2	9,81	7,09	0,66
NDN	6,66	6,24	4,73
NDNL2	5,81	5,71	5,95
NDP	7,68	7,66	-3,32
NDRG1	10,57	10,17	10,23
NDRG2	6,87	7,68	5,49
NDRG3	9,31	9,66	9,42
NDRG4	3,05	3,39	3,37
NDUFA1	13,74	13,71	12,92
NDUFA10	8,89	9,39	9,25
NDUFA11	10,61	11,12	10,64
NDUFA12	11,14	11,12	11,53
NDUFA13	10,62	10,74	10,00
NDUFA2	11,82	11,76	11,78
NDUFA3	12,21	12,41	11,66
NDUFA4	13,58	13,23	12,85
NDUFA5	6,38	5,94	5,97
NDUFA6	9,68	9,85	9,41
NDUFA7	9,10	9,44	8,73
NDUFA8	10,95	11,08	9,91
NDUFA9	10,22	10,45	9,44

NDUFAF1	8,92	9,45	7,55
NDUFB1	5,49	5,65	4,42
NDUFB11	11,37	11,37	11,37
NDUFB3	10,96	11,49	10,45
NDUFB5	12,16	12,20	11,89
NDUFB6	10,31	11,17	9,63
NDUFB8	12,74	12,82	12,23
NDUFB9	11,53	12,61	11,38
NDUFC1	9,11	9,10	8,87
NDUFC2	6,11	5,47	4,54
NDUFS1	7,47	7,34	6,84
NDUFS3	11,35	12,08	10,77
NDUFS4	11,69	11,60	11,16
NDUFS5	12,85	12,73	11,64
NDUFS6	12,85	13,01	12,28
NDUFS8	12,22	12,13	11,25
NDUFV1	10,42	10,18	9,86
NDUFV2	12,21	11,79	10,88
NDUFV3	7,49	7,49	7,19
NECAP1	9,39	9,55	9,93
NECAP2	11,22	10,83	10,57
NEDD1	6,16	6,10	6,30
NEDD4	3,54	4,02	4,69
NEDD4L	5,45	4,80	5,27
NEDD8	10,39	10,38	10,20
NEDD9	3,53	5,08	5,93
NEFH	7,38	7,88	6,26
NEIL1	4,28	4,18	4,66
NEIL3	-3,32	4,08	-3,32
NEK1	5,61	5,95	5,66
NEK11	3,82	4,02	2,92
NEK3	6,47	5,12	6,65
NEK4	-0,82	0,27	3,63
NEK8	-1,08	3,54	5,72
NEK9	4,07	4,57	4,25
NEO1	7,40	7,57	7,46
NES	2,55	7,81	-3,32
NET1	10,01	6,94	6,62
NETO2	9,24	8,16	8,51
NEU1	11,01	10,89	9,10
NEU3	3,94	2,49	3,28
NEURL	3,87	4,00	3,84
NEUROG3	3,96	3,99	0,37
NF1	4,16	4,54	5,41
NFAT5	6,00	5,38	6,30
NFATC1	6,11	5,87	7,14
NFATC3	6,52	6,52	6,68
NFE2	9,43	7,17	11,20
NFE2L1	9,19	8,93	7,56
NFE2L2	11,54	11,41	10,22
NFE2L3	8,86	8,06	8,96
NFIA	5,39	5,50	7,24
NFIC	7,42	7,51	7,78
NFIL3	10,58	8,79	10,54
NFIX	4,50	3,58	7,15
NFKB1	11,66	11,01	12,06
NFKB2	7,38	7,23	7,63
NFKBIA	12,90	13,14	13,99
NFKBIB	6,65	6,53	6,30

NFKBIL1	5,56	5,41	6,02
NFKBIZ	6,80	10,04	11,14
NFRKB	5,81	5,80	6,08
NFS1	4,44	4,29	4,03
NFX1	8,10	7,47	7,49
NFXL1	9,48	7,10	8,79
NFYA	5,40	5,50	6,07
NFYC	9,26	9,27	9,99
NGFRAP1	3,11	7,18	3,35
NGFRAP1L1	3,23	3,88	2,60
NGLY1	9,22	8,93	9,46
NGRN	9,54	9,23	8,82
NHEJ1	4,78	4,81	4,55
NHLRC1	1,86	3,65	3,20
NHLRC2	7,28	7,38	7,28
NHN1	6,79	6,85	7,47
NHP2L1	8,98	9,26	9,49
NIBP	6,89	6,84	7,05
NICN1	9,36	9,23	8,55
NIF3L1	10,12	10,23	9,59
NIN	8,62	8,55	10,08
NIP30	7,58	7,77	8,21
NIP7	9,32	8,77	7,79
NIPA2	9,97	10,13	9,37
NIPBL	6,46	6,11	7,63
NIPSNAP3A	7,33	7,39	7,93
NISCH	9,18	8,03	10,00
NIT1	6,53	6,59	6,51
NIT2	9,23	9,63	8,40
NKAP	6,62	6,90	6,61
NKIRAS1	8,59	8,44	5,79
NKIRAS2	7,39	7,09	7,97
NKPD1	3,77	4,36	3,53
NKRF	7,71	7,46	7,22
NKTR	8,89	8,62	10,62
NKX3-1	4,00	4,08	4,80
NLF2	3,67	2,72	3,58
NLGN2	2,58	2,87	3,10
NLGN4Y	4,64	4,50	4,80
NLK	8,95	7,79	7,33
NLN	5,60	6,44	5,57
NMB	7,04	7,96	3,88
NMD3	10,03	9,10	8,92
NME1	10,05	10,35	9,15
NME1-NME2	10,78	10,96	10,29
NME3	8,97	8,35	9,47
NME6	5,39	5,42	5,88
NME7	6,34	7,22	6,47
NMI	10,61	10,23	11,22
NMNAT1	5,40	6,23	5,40
NMNAT2	4,31	4,27	4,43
NMNAT3	6,27	-1,25	2,69
NMT1	7,55	7,71	7,41
NMT2	6,58	8,15	5,39
NNT	9,34	9,14	8,61
NOC2L	6,96	6,63	7,75
NOC3L	6,92	6,91	6,95
NOC4L	5,46	5,50	5,80
NOL1	8,82	8,45	8,49

NOL11	9,36	9,21	9,15
NOL3	3,78	4,52	3,83
NOL5A	9,71	9,37	9,63
NOL6	8,32	8,13	8,44
NOL7	11,30	11,19	11,29
NOL8	9,44	9,36	9,43
NOL9	4,41	4,73	5,42
NOLA1	6,24	6,40	6,31
NOLA2	8,30	8,68	8,63
NOLA3	12,79	13,35	12,28
NOLC1	5,89	5,85	5,98
NOMO1	7,98	8,43	7,60
NOMO3	6,22	6,58	5,12
NONO	7,13	7,05	7,25
NOP5/NOP58	10,29	9,84	10,08
NOS3	4,69	4,72	3,98
NOSIP	9,50	9,51	10,23
NOTCH2NL	7,19	7,70	8,54
NOTCH4	4,60	5,29	6,26
NOV	0,68	5,11	4,32
NOX1	3,83	2,26	3,53
NOXA1	6,24	5,77	6,43
NPAL1	6,16	4,06	0,28
NPAL2	5,09	5,57	5,52
NPAL3	7,96	7,67	6,40
NPAS1	-3,32	4,16	-0,46
NPAS3	3,80	3,95	3,66
NPAT	8,10	7,67	7,59
NPBWR1	3,58	4,02	3,90
NPC1	11,26	11,53	8,00
NPC2	14,57	14,55	13,81
NPDC1	5,12	4,02	4,88
NPFFR1	5,01	4,67	4,23
NPHP3	6,66	6,42	7,50
NPHP4	4,53	4,82	5,65
NPHS2	3,44	3,70	3,95
NPL	11,06	11,73	10,46
NPM2	4,00	4,28	3,32
NPR1	5,11	5,13	4,23
NPTN	10,74	10,64	10,34
NPTXR	3,87	3,24	3,98
NQO1	9,58	10,28	4,91
NQO2	9,41	9,39	9,60
NR1D2	5,67	5,49	6,78
NR1H2	9,14	9,13	9,28
NR1H3	10,32	12,02	7,45
NR1I2	3,13	3,19	3,50
NR2C1	6,79	6,36	7,51
NR2C2	6,04	5,85	6,58
NR2E1	3,80	3,82	4,39
NR2F2	5,38	5,24	5,53
NR3C1	5,77	6,65	6,40
NR3C2	3,01	4,12	4,59
NR4A1	2,62	2,55	5,44
NR4A2	4,83	6,32	10,35
NR4A3	6,12	4,59	4,24
NR5A1	3,74	4,01	4,01
NRAS	8,72	9,12	7,94
NRBP1	8,18	8,26	7,52

NRCAM	3,07	3,58	-1,47
NRD1	11,00	10,99	11,10
NRF1	4,50	4,69	4,47
NRG1	-0,69	0,95	7,06
NRG2	4,43	3,97	3,97
NRG3	4,65	4,66	4,89
NRG4	1,47	3,84	2,91
NRGN	8,30	8,63	10,66
NRIP3	10,14	11,36	7,39
NRL	2,81	3,53	3,72
NRM	6,01	5,99	7,68
NRP1	10,27	10,25	5,22
NRP2	5,99	5,94	3,39
NRXN1	3,45	3,72	3,63
NRXN2	4,07	3,15	2,75
NRXN3	3,24	4,38	3,68
NSBP1	5,23	5,95	4,66
NSD1	5,25	5,29	6,71
NSDHL	9,45	9,33	8,13
NSF	10,41	10,36	9,41
NSFL1C	7,76	8,00	9,67
NSMAF	11,36	11,33	10,31
NSUN2	10,89	10,62	11,23
NSUN3	6,88	7,11	6,66
NSUN4	7,86	7,70	7,61
NSUN5	7,91	7,17	8,17
NSUN5C	5,44	4,62	5,73
NSUN6	6,48	6,79	6,87
NT5C	9,21	8,63	9,97
NT5C2	11,68	11,62	11,60
NT5C3	7,76	8,08	9,26
NT5C3L	7,48	8,17	8,03
NT5DC1	8,00	7,85	8,63
NTF5	4,45	4,45	4,71
NTHL1	6,51	6,36	6,53
NTNG2	2,47	-0,48	7,69
NTSR1	-3,32	-3,32	5,66
NTSR2	-0,35	2,24	2,08
NUAK2	4,47	4,45	8,36
NUBP1	7,71	7,65	8,38
NUBP2	5,41	5,58	5,67
NUBPL	6,82	7,03	6,40
NUCB1	13,42	13,67	12,86
NUCB2	6,86	9,01	8,87
NUCKS1	8,68	8,37	7,91
NUDC	9,25	9,23	9,51
NUDCD1	4,70	4,50	4,26
NUDCD2	8,08	7,98	8,02
NUDCD3	7,58	7,61	7,27
NUDT1	9,48	9,83	9,75
NUDT12	4,41	4,42	3,40
NUDT14	9,89	9,41	9,05
NUDT15	7,47	7,67	7,87
NUDT16	8,04	6,58	7,99
NUDT16P	8,89	6,38	7,65
NUDT17	2,03	4,04	3,83
NUDT2	8,42	8,78	8,35
NUDT21	7,73	8,08	8,97
NUDT22	8,44	8,37	8,44

NUDT3	10,05	9,73	10,30
NUDT4	3,77	3,99	4,08
NUDT5	10,61	10,62	9,91
NUDT6	5,27	5,43	3,90
NUDT9	8,21	8,67	7,12
NUFIP2	9,37	8,54	10,24
NUMA1	5,30	4,69	5,65
NUMB	9,89	11,50	11,26
NUP107	9,26	9,32	8,84
NUP133	8,52	8,50	8,51
NUP155	7,12	7,35	7,21
NUP160	8,52	8,85	8,39
NUP188	7,88	7,97	7,09
NUP205	9,37	9,30	9,20
NUP214	7,94	8,91	11,85
NUP35	6,01	6,22	5,37
NUP37	9,09	9,46	8,97
NUP43	5,79	5,50	6,21
NUP50	7,36	6,96	7,44
NUP54	8,21	7,96	8,56
NUP62	12,01	11,54	11,87
NUP85	9,46	9,09	9,33
NUP88	9,19	9,52	9,68
NUPL2	8,75	8,55	9,03
NUSAP1	6,87	9,15	6,50
NUT	4,09	4,24	3,03
NUTF2	8,35	8,32	8,31
NVL	5,99	6,30	6,02
NXF1	9,46	9,35	11,03
NXN	3,79	4,27	1,34
NXPH2	3,85	3,99	3,97
NXPH3	4,91	2,31	3,40
NXT1	8,63	8,11	9,77
NXT2	8,76	8,99	9,19
NY-REN-7	4,68	4,50	4,88
NY-SAR-48	6,15	6,33	5,89
NYD-SP21	6,02	7,15	9,46
OAF	6,84	6,69	10,44
OAS1	8,10	7,63	8,51
OAS2	6,44	7,86	9,50
OAS3	7,58	7,57	9,16
OASL	2,85	4,33	6,80
OAT	8,67	8,89	8,88
OAZ1	14,63	14,66	14,49
OAZ2	9,11	9,70	10,25
OAZ3	5,41	5,18	4,18
OBFC1	6,90	7,36	8,51
OBFC2A	11,82	12,00	9,68
OBFC2B	6,83	7,11	6,69
OCIAD1	7,80	7,55	7,61
OCRL	8,48	8,63	7,94
ODF2L	4,07	4,95	4,05
ODF4	4,87	4,09	4,48
ODZ1	6,27	6,23	6,56
OFCC1	3,05	-1,70	3,36
OFD1	7,05	6,41	7,34
OGDH	6,58	6,46	7,39
OGDHL	5,62	5,21	5,46
OGFOD1	7,78	7,68	7,09

OGFRL1	9,12	7,88	8,97
OGT	9,65	9,02	10,74
OIP5	-1,18	5,39	-1,09
OKL38	9,27	8,81	6,99
OLFM1	1,68	2,52	7,98
OLFML2B	4,00	5,16	5,09
OLFML3	6,32	4,26	2,32
OLIG1	3,72	4,53	10,14
OLR1	10,28	12,71	2,33
OMA1	7,24	7,89	8,40
OPA1	7,84	8,16	7,97
OPA3	5,06	5,25	4,57
OPLAH	7,53	7,16	6,74
OPN1MW	4,17	4,24	4,50
OPN5	2,23	2,58	2,92
OPRD1	5,07	4,81	4,64
OPRS1	4,38	4,81	3,96
OPTN	9,55	9,29	5,69
OR10G3	4,35	3,63	5,22
OR10G8	5,20	4,73	6,21
OR10K1	3,96	4,15	3,67
OR13H1	3,68	3,84	3,76
OR1A2	4,36	4,23	4,69
OR1F1	4,60	4,75	4,87
OR2AG1	1,26	-2,32	3,61
OR2H2	4,62	4,72	4,32
OR4D2	4,48	4,25	4,33
OR4F21	3,02	3,08	2,56
OR4M2	3,85	4,15	3,45
OR52I1	2,75	3,74	3,88
OR52K2	4,14	4,36	6,27
OR56A1	3,89	4,43	4,32
OR56A3	3,46	3,01	3,08
OR5BU1	4,98	5,13	4,61
OR6C4	4,66	3,96	4,16
OR6S1	3,86	3,83	3,24
ORAOV1	7,16	7,26	7,87
ORC1L	5,09	6,22	3,27
ORC2L	7,12	7,48	7,42
ORC3L	7,39	7,37	7,66
ORC4L	5,47	5,14	5,54
ORC5L	7,20	7,60	6,71
ORC6L	5,75	5,65	4,89
ORMDL1	10,40	10,42	11,08
ORMDL2	6,65	6,32	5,49
ORMDL3	5,73	5,54	5,65
OS9	9,38	9,23	9,93
OSBP	10,56	10,49	10,63
OSBP2	4,00	7,10	3,88
OSBPL10	6,31	4,42	-0,48
OSBPL11	9,68	10,08	9,54
OSBPL1A	8,88	8,18	6,66
OSBPL2	9,54	9,41	9,66
OSBPL3	5,83	5,83	5,40
OSBPL5	6,66	6,15	7,94
OSBPL6	2,66	3,21	2,80
OSBPL7	5,71	5,04	7,30
OSBPL8	10,81	10,97	11,31
OSBPL9	9,37	9,41	8,73

OSCAR	9,88	10,72	10,50
OSGEP	8,17	8,38	8,58
OSGEPL1	6,39	6,21	3,81
OSM	5,58	7,61	8,95
OSTF1	11,84	11,92	11,87
OSTBETA	4,06	4,72	-0,30
OTOF	3,26	2,87	3,98
OTOP3	4,31	4,11	4,63
OTUD4	10,10	10,45	10,44
OTUD5	8,34	8,11	8,41
OTUD6B	7,57	6,21	7,20
OXA1L	10,12	10,03	10,33
OXCT1	6,46	5,37	5,51
OXNAD1	7,33	7,31	6,10
OXR1	9,15	9,68	10,24
OXSM	6,86	6,43	5,53
OXSR1	9,67	10,21	10,15
P117	11,18	10,57	10,46
P18SRP	4,09	4,74	3,96
P2RX1	7,32	8,17	9,48
P2RX2	3,47	3,69	3,14
P2RX7	8,15	8,10	9,12
P2RY1	4,48	4,07	2,15
P2RY10	4,89	4,48	4,13
P2RY13	6,19	8,67	9,46
P2RY2	3,42	4,06	4,82
P2RY4	4,83	4,37	4,59
P2RY6	6,01	5,33	4,44
P2RY8	8,01	7,20	10,26
P4HA1	9,44	9,09	9,06
P4HA2	9,13	8,32	0,25
P4HA3	3,61	3,35	4,19
PA2G4	9,41	9,46	9,26
PABPC1	13,09	12,62	13,60
PABPC3	7,84	7,12	8,81
PABPN1	9,31	8,95	10,11
PACRG	5,26	5,42	5,23
PACS1	8,45	7,23	7,84
PACSIN1	4,87	4,56	1,84
PACSIN2	11,23	11,24	10,95
PADI1	3,99	4,15	4,08
PADI2	0,23	0,25	4,47
PAF1	8,78	8,51	9,19
PAFAH1B1	9,69	9,72	10,06
PAFAH1B2	6,22	6,32	5,61
PAFAH2	8,06	8,46	6,46
PAG1	5,28	6,39	6,30
PAICS	9,25	9,83	8,71
PAIP2	9,16	9,32	10,07
PAK1	8,61	8,55	10,48
PAK1IP1	9,17	9,62	9,04
PAK4	5,43	5,24	5,23
PALM	5,25	4,50	4,02
PAM	10,20	9,72	7,70
PANK1	3,88	3,84	2,89
PANK3	8,42	7,87	6,61
PANK4	6,17	6,16	6,63
PANX1	5,69	6,46	6,10
PANX2	8,68	6,15	7,20

PAPD1	8,83	8,91	8,58
PAPD4	11,04	10,73	10,81
PAPD5	9,78	8,90	9,59
PAPLN	7,24	8,22	5,07
PAPOLA	11,06	10,78	11,59
PAPOLG	4,00	4,05	4,07
PAPSS1	11,02	12,05	10,80
PAQR3	6,52	6,86	6,85
PAQR4	8,25	8,55	8,06
PAQR5	1,95	5,44	-0,10
PAQR6	3,29	2,68	3,79
PAQR7	5,41	5,05	8,42
PAQR8	7,92	8,78	7,71
PARC	8,18	7,09	6,86
PARD3	3,87	3,04	0,06
PARD6A	6,03	6,08	6,39
PARK7	11,95	12,15	11,93
PARL	10,79	10,68	11,33
PARN	8,74	8,54	8,41
PARP10	7,08	6,77	7,82
PARP12	10,90	10,83	9,43
PARP16	5,47	5,59	6,02
PARP2	5,62	6,36	4,39
PARP3	5,82	5,78	5,46
PARP4	11,68	11,21	11,42
PARP6	9,47	9,74	9,44
PARP9	8,92	9,22	9,63
PARS2	5,24	5,45	2,64
PARVG	9,15	10,41	11,16
PASK	6,08	5,50	5,51
PAWR	3,05	2,90	3,76
PAX3	1,15	2,95	2,35
PAX5	3,40	3,71	3,12
PAX7	5,38	4,90	5,23
PAX8	4,27	4,78	3,85
PAX9	4,36	4,22	4,40
PAXIP1	7,01	7,00	7,69
PBEF1	6,14	6,91	9,25
PBX1	4,89	4,60	3,62
PBX2	7,72	6,79	9,43
PBX3	11,01	10,94	9,77
PBXIP1	6,15	5,57	6,27
PCAF	9,55	9,46	10,20
PCBP1	12,76	12,51	13,09
PCBP4	1,43	3,16	1,95
PCCA	7,74	8,51	8,44
PCCB	10,62	10,92	9,60
PCDH10	4,26	4,49	4,22
PCDH7	2,42	2,16	2,89
PCDH9	3,08	3,96	4,24
PCDHAC2	4,06	3,95	3,18
PCDHGA1	3,88	3,71	3,52
PCDHGA6	3,87	3,75	0,93
PCDHGB1	4,32	4,28	3,97
PCDHGB5	3,84	3,88	4,56
PCDHGB6	6,20	6,17	6,38
PCDHGC3	-0,49	2,97	2,03
PCF11	8,28	8,07	9,00
PCGF1	7,10	7,29	7,58

PCGF2	6,31	7,38	4,70
PCGF5	6,04	5,73	5,97
PCGF6	7,42	7,40	7,13
PCID2	9,93	9,97	10,30
PCM1	11,37	10,24	10,23
PCMT1	11,46	11,66	11,76
PCNA	8,60	9,38	7,82
PCNP	10,65	10,64	11,43
PCNT	9,05	8,38	9,15
PCNX	7,70	9,04	9,61
PCNXL3	7,59	7,22	7,61
PCOLCE2	6,01	8,44	0,75
PCQAP	5,70	5,52	6,49
PCSK4	3,57	0,10	3,12
PCSK5	10,15	9,13	9,72
PCSK6	-3,32	4,84	-3,32
PCSK7	9,15	8,67	8,94
PCSK9	4,03	4,72	3,19
PCTK1	4,06	3,82	3,82
PCTK2	4,67	4,57	3,88
PCYOX1	8,29	8,27	7,65
PCYT1A	5,10	5,11	4,90
PDCD10	9,39	9,43	8,64
PDCD11	8,38	8,05	7,75
PDCD1LG2	7,67	7,05	3,84
PDCD2	8,61	8,47	9,04
PDCD2L	5,92	6,04	6,21
PDCD4	6,80	6,52	8,36
PDCD5	8,31	8,50	8,26
PDCD6IP	8,34	8,71	9,71
PDCD7	8,30	8,03	8,45
PDCL	6,75	7,26	6,19
PDCL3	8,94	8,79	8,85
PDE1B	4,63	5,88	5,52
PDE3A	5,00	4,66	4,38
PDE3B	9,07	9,29	7,20
PDE4B	-3,32	5,33	7,85
PDE4DIP	4,50	4,86	4,02
PDE6D	8,97	8,47	8,21
PDE7A	5,42	5,21	4,83
PDE7B	4,07	4,30	5,90
PDE8A	7,25	7,00	6,50
PDE8B	5,77	6,29	5,21
PDGFB	6,83	3,87	2,09
PDGFC	9,74	8,75	8,10
PDGFRB	3,94	5,20	3,54
PDHA1	11,24	11,22	10,29
PDHB	11,87	11,22	11,37
PDHX	9,46	9,45	8,62
PDIA4	10,58	10,50	9,17
PDIK1L	8,20	8,03	8,06
PDK1	4,27	3,97	6,13
PDK2	5,04	5,65	5,63
PDK3	7,68	8,40	7,97
PDK4	-2,00	3,90	8,65
PDLIM1	-3,32	-0,16	4,47
PDLIM4	6,27	5,54	1,54
PDLIM5	5,88	6,30	6,25
PDLIM7	8,10	8,52	7,63

PDP2	4,77	3,97	0,21
PDPN	-1,84	5,70	-3,32
PDPR	7,99	7,34	7,95
PDRG1	7,67	7,89	6,93
PDSS1	8,76	8,93	7,95
PDSS2	9,47	9,21	8,42
PDXK	12,27	11,69	9,96
PDXP	9,92	9,04	8,66
PDZD11	4,27	3,84	4,77
PDZD8	6,63	6,33	7,75
PEA15	13,14	12,75	11,80
PECAM1	10,59	10,83	11,65
PECI	8,67	9,01	8,45
PECR	7,13	7,57	7,22
PEF1	8,96	8,75	8,50
PEG3	4,43	4,76	-1,19
PELI1	7,11	7,38	10,83
PELI2	7,59	7,27	10,63
PELI3	4,98	5,19	4,98
PELO	9,32	9,84	8,06
PELP1	9,13	7,40	7,54
PEO1	5,68	5,22	4,66
PEPD	11,86	11,93	10,91
PER1	1,37	2,62	6,94
PER2	8,44	7,32	7,83
PER3	6,39	5,99	3,30
PERLD1	8,19	8,51	7,62
PES1	7,23	7,48	7,74
PET112L	8,71	9,22	8,02
PEX1	6,44	6,25	6,69
PEX11A	5,61	6,02	5,03
PEX11B	9,56	9,33	9,34
PEX13	7,95	7,81	7,01
PEX14	7,12	8,21	6,54
PEX16	8,00	7,94	8,58
PEX19	9,94	10,27	8,69
PEX26	6,88	7,00	6,70
PEX3	6,97	7,38	6,55
PEX5	8,83	9,10	9,62
PEX6	6,11	6,84	7,08
PEX7	7,47	7,74	6,97
PFAAP5	6,89	7,02	9,10
PFAS	6,94	6,88	6,82
PFDN1	9,44	10,64	9,29
PFDN2	7,72	7,97	7,94
PFDN5	13,32	13,25	13,69
PFDN6	8,27	8,34	8,54
PFKFB2	6,52	6,42	5,64
PFKFB3	6,10	6,92	8,22
PFKM	5,91	6,17	5,21
PFKP	11,46	9,57	7,68
PFN2	4,38	3,18	2,82
PGA5	2,40	4,43	6,60
PGAM1	9,09	10,43	8,58
PGAM5	6,06	6,03	6,21
PGAP1	1,96	0,00	4,75
PGBD1	4,60	3,55	4,33
PGBD2	5,79	5,62	5,65
PGBD3	6,28	5,35	5,87

PGBD4	0,47	3,19	2,49
PGD	12,25	12,93	11,94
PGDS	5,98	7,59	0,68
PGGT1B	5,87	6,09	6,97
PGK1	12,10	12,37	11,88
PGLS	9,90	9,92	11,07
PGLYRP4	4,31	4,11	4,21
PGM1	9,11	10,26	10,35
PGM2L1	4,30	5,50	0,33
PGM3	7,02	7,88	5,49
PGM5	4,67	6,36	5,25
PGRMC1	10,47	10,87	10,48
PGRMC2	9,69	9,81	9,87
PH-4	5,36	5,32	4,95
PHACS	7,26	7,35	8,52
PHACTR1	7,39	7,91	3,36
PHACTR3	1,87	0,53	3,50
PHACTR4	9,55	9,86	9,74
PHB	8,95	9,20	8,24
PHB2	9,88	9,81	10,22
PHC1	6,87	6,21	5,87
PHF1	7,05	6,87	6,76
PHF10	9,87	9,80	10,49
PHF11	10,35	10,28	11,66
PHF12	5,33	4,78	5,93
PHF13	9,70	9,43	8,91
PHF14	6,64	6,66	7,25
PHF16	5,69	3,63	6,37
PHF17	6,34	6,30	7,33
PHF19	6,78	6,67	7,03
PHF20L1	8,19	7,94	8,86
PHF21A	10,08	10,11	11,68
PHF23	9,12	8,68	8,31
PHF3	8,51	8,14	9,45
PHF5A	7,52	7,42	7,93
PHF7	3,66	3,39	2,95
PHGDH	8,84	7,34	4,84
PHIP	9,93	8,90	10,89
PHKA2	8,61	8,49	9,72
PHKB	8,00	7,72	8,31
PHKG2	6,88	6,20	7,94
PHLDA1	10,45	7,75	3,99
PHLDA2	5,35	5,03	3,08
PHLDA3	8,45	9,77	-3,32
PHLDB1	5,67	6,13	-0,14
PHLPPL	7,21	5,85	6,11
PHOSPHO1	4,73	4,04	3,23
PHOSPHO2	4,21	4,16	-2,08
PHTF1	8,39	8,51	7,25
PHTF2	10,28	9,51	8,56
PHYHIPL	4,73	5,02	4,98
PI4K2B	8,14	8,77	8,21
PIAS2	6,93	6,46	6,72
PIAS3	7,50	7,68	8,77
PIAS4	9,26	9,19	9,58
PICALM	10,14	8,86	9,22
PICK1	3,78	3,51	3,66
PIGA	7,37	7,46	8,49
PIGB	8,19	8,62	8,81

PIGC	8,56	8,61	9,13
PIGF	7,81	8,28	7,85
PIGG	7,16	7,24	6,38
PIGK	9,10	9,35	7,65
PIGL	3,86	4,79	5,46
PIGM	6,91	7,85	8,60
PIGN	9,04	8,99	8,24
PIGP	8,50	8,82	8,02
PIGS	8,65	8,46	8,84
PIGT	9,48	10,14	9,27
PIGV	8,34	8,63	7,76
PIGW	6,46	5,98	5,93
PIGX	8,12	8,04	9,08
PIGZ	5,43	5,33	4,85
PIK3AP1	11,61	11,91	11,71
PIK3C2B	3,79	4,07	2,53
PIK3C3	8,46	8,83	8,77
PIK3CA	6,90	6,31	7,07
PIK3CD	9,10	8,62	9,80
PIK3CG	10,19	10,34	9,58
PIK3R1	8,78	7,38	7,60
PIK3R2	10,15	9,97	9,75
PIK3R3	2,21	0,43	2,57
PIK3R4	8,45	8,55	8,16
PIK4CA	10,24	10,12	10,02
PILRB	4,51	4,07	6,27
PIM1	9,53	12,12	9,66
PIM2	7,97	6,39	7,58
PIN4	4,42	4,91	5,52
PIP3-E	9,38	7,93	9,65
PIP5K1A	5,26	5,16	4,76
PIP5K2A	8,25	7,65	8,45
PIP5K2B	9,15	8,94	10,52
PIP5K2C	8,25	8,16	6,86
PIP5K3	3,02	2,98	2,95
PIR	9,47	10,31	1,56
PISD	9,07	9,40	10,61
PITPNB	10,90	10,93	10,47
PITPNC1	6,63	7,25	8,22
PITPNM1	9,92	9,11	10,73
PITX3	4,30	4,33	3,70
PKD1	5,86	5,33	6,10
PKD1L1	4,72	5,00	3,84
PKD2	9,47	8,20	6,99
PKIA	3,82	6,79	0,74
PKIB	6,79	3,40	2,73
PKM2	8,62	9,26	7,84
PKMYT1	-3,32	5,29	-3,32
PKN1	7,42	6,73	8,29
PKN2	7,35	6,30	8,85
PKNOX1	6,40	6,20	5,96
PKP2	-0,76	2,79	4,78
PKP4	5,40	5,88	6,56
PLA1A	2,77	4,18	-3,32
PLA2G10	4,16	3,73	2,77
PLA2G12A	4,42	4,95	3,62
PLA2G4A	9,13	8,60	7,27
PLA2G4C	9,25	10,04	7,09
PLA2G5	7,14	-2,20	2,04

PLA2G6	3,74	3,44	3,28
PLA2G7	13,05	12,90	10,28
PLAA	7,24	7,16	7,26
PLAC1	4,63	4,64	4,80
PLAC8	-0,31	5,44	10,08
PLAG1	3,66	2,91	3,43
PLAGL2	7,82	7,88	8,85
PLAU	11,51	11,39	5,39
PLAUR	10,12	11,70	10,12
PLCB1	5,75	4,83	7,35
PLCB2	10,63	10,12	10,44
PLCB3	6,27	5,77	6,36
PLCD1	4,88	6,47	5,22
PLCG1	5,88	4,77	2,88
PLCL1	6,78	6,75	4,86
PLCL2	9,24	9,36	11,25
PLCXD1	7,65	8,48	7,47
PLCXD3	4,12	3,98	3,96
PLD2	6,60	6,11	7,23
PLD3	10,23	11,06	9,05
PLDN	10,87	9,06	9,00
PLEC1	5,64	4,96	6,00
PLEK	13,03	13,37	12,69
PLEK2	3,27	6,59	2,71
PLEKHA1	9,39	8,66	8,60
PLEKHA3	7,09	6,91	6,34
PLEKHA6	5,43	-3,32	-3,32
PLEKHA7	4,27	6,07	4,25
PLEKHA9	6,26	6,82	7,47
PLEKHB1	3,24	3,45	-0,05
PLEKHB2	9,64	9,68	8,18
PLEKHC1	2,80	4,53	-3,32
PLEKHG2	6,10	5,98	6,31
PLEKHG3	5,12	7,36	9,65
PLEKHG4	6,32	6,27	4,83
PLEKHG5	3,88	4,21	3,27
PLEKHH2	4,80	4,31	4,84
PLEKHJ1	5,89	5,33	5,71
PLEKHM1	7,81	8,06	8,30
PLEKHO1	10,26	10,27	10,84
PLEKHQ1	10,40	10,13	9,44
PLK1	0,13	4,46	-0,51
PLK4	5,28	6,90	4,94
PLLP	4,07	4,13	3,67
PLOD1	11,02	11,02	10,72
PLOD2	2,48	-0,44	-3,32
PLOD3	7,69	7,21	6,91
PLRG1	9,94	10,00	9,49
PLSCR4	2,16	2,82	3,47
PLVAP	3,57	1,79	5,56
PLXDC1	4,46	5,08	5,06
PLXDC2	10,62	11,16	10,22
PLXNA2	3,96	4,08	4,24
PLXNC1	6,04	7,32	8,05
PMAIP1	5,24	5,12	6,37
PMF1	6,44	6,38	7,82
PML	4,54	4,22	4,91
PMM2	8,16	8,55	8,04
PMP22	8,33	10,11	5,75

PMPCA	8,37	8,05	8,50
PMPCB	10,24	10,23	10,52
PMS1	6,60	6,04	6,95
PMS2	2,39	4,09	4,76
PMS2L2	2,36	4,02	4,61
PMS2L3	6,21	6,26	6,62
PMS2L5	5,55	6,67	7,33
PMVK	7,42	7,52	7,22
PNKD	6,11	7,64	7,23
PNKP	9,11	8,78	9,07
PNMA3	-0,31	4,38	4,78
PNN	10,03	9,66	11,15
PNOC	4,51	3,94	5,93
PNPLA1	2,79	4,11	5,00
PNPLA4	2,91	4,44	3,61
PNPLA5	4,83	5,08	4,54
PNPO	11,26	11,54	10,30
PNPT1	5,32	5,30	6,31
PNRC1	5,00	4,85	6,57
PNRC2	6,66	6,74	6,79
PODN	3,47	4,42	4,13
PODXL2	2,55	2,09	4,07
POFUT1	6,01	5,98	6,59
POGK	10,93	10,70	10,49
POGZ	4,43	3,84	5,41
POLA	5,57	5,76	6,25
POLA2	7,72	8,04	7,30
POLB	8,75	9,04	9,58
POLD3	6,29	6,60	7,77
POLD4	7,62	7,65	7,96
POLDIP2	6,80	6,90	6,64
POLDIP3	8,57	8,12	8,66
POLE2	4,66	6,38	0,62
POLE3	11,54	11,41	11,62
POLG	10,21	10,05	10,74
POLG2	6,91	6,28	7,25
POLH	4,98	5,00	5,10
POLI	-0,50	-3,32	4,98
POLL	4,63	4,89	5,41
POLM	6,46	6,14	6,77
POLQ	4,20	6,63	2,72
POLR1A	4,50	4,72	2,50
POLR1B	4,24	2,89	4,35
POLR1C	8,34	8,26	7,91
POLR1D	10,61	10,30	11,38
POLR2A	12,55	12,04	12,26
POLR2B	10,84	10,58	10,86
POLR2C	8,31	8,52	8,87
POLR2D	8,24	8,25	7,88
POLR2F	10,48	10,83	10,81
POLR2G	10,84	10,82	10,89
POLR2H	10,19	10,05	9,86
POLR2I	9,39	9,69	9,36
POLR2J	6,97	6,96	7,19
POLR2J3	9,73	9,87	9,92
POLR2K	6,31	6,27	6,78
POLR3A	6,34	6,40	6,33
POLR3B	7,63	8,20	7,75
POLR3C	9,88	9,63	10,00

POLR3D	4,64	4,46	4,51
POLR3E	7,47	7,44	7,79
POLR3F	7,65	7,46	7,56
POLR3GL	10,18	10,40	10,50
POLR3K	8,17	8,10	7,17
POLRMT	8,23	7,57	7,91
POMC	4,56	5,63	6,05
POMGNT1	8,91	9,58	8,02
POMT2	7,22	6,76	7,07
POMZP3	6,39	6,37	6,35
PON2	9,37	8,40	7,42
POP1	7,01	7,01	5,05
POP4	10,44	10,86	10,03
POP5	6,71	6,86	7,25
POR	8,76	8,50	8,39
PORCN	3,99	4,46	4,61
POU2F1	5,70	5,47	5,87
POU2F2	6,83	6,63	11,74
POU4F1	3,31	2,70	4,11
POU6F1	4,14	3,63	4,52
PPA1	10,49	10,79	11,85
PPAP2A	4,98	4,50	2,64
PPAP2B	8,80	8,05	-3,32
PPAP2C	3,63	3,67	3,94
PPAPDC1B	7,25	7,78	6,55
PPAPDC2	6,69	6,55	5,07
PPARBP	10,07	9,63	10,13
PPARG	10,25	10,06	4,64
PPARGC1A	-0,31	-3,32	5,02
PPAT	7,62	7,78	6,34
PPCS	11,17	10,92	10,71
PPFIA1	9,39	9,10	8,57
PPFIA3	4,64	5,32	4,75
PPFIBP1	7,43	5,93	3,73
PPFIBP2	11,45	9,75	9,57
PPHLN1	8,63	8,39	8,45
PPIA	7,49	7,77	7,51
PPIB	11,06	11,48	11,55
PPIC	9,53	9,34	-3,32
PPID	8,65	8,72	8,32
PPIE	7,19	7,34	7,68
PPIG	9,31	8,96	9,54
PPIH	9,06	8,73	8,97
PPIL1	7,00	6,74	5,41
PPIL2	4,51	4,24	4,24
PPIL3	10,87	10,52	9,77
PPIL5	7,78	7,61	6,89
PPM1A	5,83	5,88	5,86
PPM1B	6,08	5,67	7,59
PPM1D	8,43	7,84	7,84
PPM1G	7,82	7,41	7,88
PPM1K	7,07	7,39	6,73
PPM1L	5,89	3,30	-0,29
PPM1M	11,88	11,32	11,20
PPM2C	7,87	7,42	10,16
PPME1	7,92	8,19	6,50
PPOX	6,85	6,99	7,69
PPP1CB	9,59	9,48	11,47
PPP1CC	12,11	12,27	12,60

PPP1R10	8,18	7,22	6,83
PPP1R11	11,23	11,46	11,74
PPP1R12A	9,20	8,59	9,62
PPP1R12C	8,08	7,80	8,75
PPP1R13B	6,90	6,89	4,82
PPP1R13L	4,67	4,15	4,85
PPP1R14A	10,31	5,51	5,72
PPP1R14B	11,87	11,51	10,69
PPP1R14C	3,02	5,63	1,69
PPP1R15A	10,78	11,55	11,97
PPP1R15B	6,77	6,05	7,46
PPP1R16B	9,20	-3,32	3,47
PPP1R1B	3,26	2,89	2,78
PPP1R2	9,75	9,38	10,45
PPP1R3B	5,83	4,18	0,38
PPP1R3D	7,06	6,86	7,35
PPP1R3F	6,14	6,36	5,03
PPP1R7	11,00	9,83	9,36
PPP1R9B	4,99	3,79	6,00
PPP2CA	12,14	12,04	12,16
PPP2CB	8,42	8,61	7,84
PPP2R1A	11,56	11,72	11,22
PPP2R1B	5,48	5,79	5,11
PPP2R2B	5,03	2,93	0,57
PPP2R3A	5,87	6,07	6,44
PPP2R3B	3,76	3,88	4,01
PPP2R4	8,50	8,31	7,51
PPP2R5A	8,74	9,36	10,26
PPP2R5C	7,28	7,48	7,93
PPP2R5D	6,94	6,84	7,00
PPP2R5E	10,37	10,09	10,59
PPP3CA	6,62	7,00	6,80
PPP3CB	9,90	9,96	10,35
PPP3CC	8,35	8,69	8,29
PPP3R1	10,35	10,41	11,11
PPP4R1	9,99	9,90	10,62
PPP4R1L	2,32	2,69	5,13
PPP5C	4,28	4,43	4,45
PPP6C	11,52	11,33	11,90
PPRC1	8,59	8,34	8,36
PPT1	14,20	13,87	13,35
PPT2	3,19	3,86	3,90
PPTC7	9,33	9,01	10,25
PPWD1	6,18	5,89	7,15
PQBP1	6,21	5,64	6,85
PRAM1	9,46	10,35	11,24
PRC1	4,77	8,31	6,22
PRCC	7,02	6,78	7,19
PRCP	13,36	12,68	12,59
PRDM1	7,63	8,14	6,85
PRDM10	6,27	6,25	6,77
PRDM14	4,91	4,65	4,24
PRDM15	4,54	4,50	4,16
PRDM4	9,44	9,40	8,84
PRDM8	5,81	2,88	4,60
PRDX1	14,22	14,48	12,80
PRDX2	8,06	7,85	7,43
PRDX3	11,70	12,10	11,11
PRDX4	10,35	11,68	8,94

PRDX5	11,72	11,68	11,60
PRDX6	11,35	11,99	11,06
PREI3	7,30	7,49	8,07
PREP	9,08	9,20	9,18
PREPL	8,37	7,98	8,04
PREX1	7,40	6,64	9,59
PRICKLE1	3,63	4,80	7,83
PRIM1	6,40	7,48	6,09
PRIM2A	5,37	5,76	5,56
PRKAA1	7,44	7,77	8,03
PRKAB1	7,52	7,19	7,67
PRKAB2	6,49	6,48	7,47
PRKACA	3,26	3,92	5,16
PRKAG1	5,12	5,48	5,38
PRKAG2	8,56	8,73	8,74
PRKAR1A	12,67	12,55	12,19
PRKAR1B	3,77	4,11	3,97
PRKCA	9,31	9,63	8,09
PRKCB1	8,90	10,14	11,69
PRKCDBP	7,77	7,46	6,01
PRKCE	5,32	6,00	7,07
PRKCH	5,91	8,72	7,62
PRKCI	7,26	6,56	7,47
PRKCQ	5,15	4,98	4,89
PRKCSH	9,14	9,26	10,02
PRKD2	7,81	7,59	9,02
PRKD3	7,22	6,47	7,23
PRKDC	7,74	7,82	8,10
PRKRA	10,06	9,65	10,27
PRKRIP1	7,41	7,16	8,16
PRKRIR	11,08	10,79	10,86
PRKX	8,22	7,47	8,31
PRLR	3,76	4,13	4,71
PRMT1	9,52	9,06	9,14
PRMT2	8,30	7,99	8,59
PRMT3	6,42	6,76	6,68
PRMT5	8,03	7,98	8,17
PRMT6	8,29	7,68	7,22
PRMT7	8,30	8,40	8,75
PRNP	12,38	12,49	12,52
PROC	4,08	6,02	3,16
PROCA1	5,09	5,04	5,58
PROCR	4,44	7,60	4,30
PROK2	3,77	1,85	9,99
PROS1	7,90	9,74	5,04
PROSC	10,57	10,53	10,96
PRPF18	8,72	8,85	9,09
PRPF19	8,78	8,93	9,12
PRPF3	9,19	9,17	9,84
PRPF31	9,55	9,52	10,24
PRPF38A	7,13	7,24	8,20
PRPF38B	6,48	6,15	7,20
PRPF39	3,88	3,29	5,47
PRPF4	9,50	9,56	9,47
PRPF4B	6,37	6,59	8,06
PRPF6	6,60	6,79	7,22
PRPF8	11,50	11,07	11,03
PRPS1	10,89	9,29	8,58
PRPS2	7,94	7,94	7,63

PRPSAP1	9,65	9,96	10,33
PRPSAP2	8,75	8,73	9,45
PRR11	5,80	6,20	5,81
PRR13	6,06	6,24	6,67
PRR14	10,83	10,19	11,32
PRR3	5,68	5,49	6,16
PRR5	5,27	6,63	4,41
PRR6	8,52	8,40	8,26
PRR8	4,03	3,46	6,37
PRRG1	5,49	6,35	4,89
PRRG2	0,86	2,79	2,18
PRRG4	5,67	4,96	6,92
PRRT1	3,78	2,63	4,28
PRRT2	4,96	4,35	4,77
PRRX2	4,59	4,04	2,50
PRSS2	1,09	3,65	0,95
PRSS3	3,67	4,60	2,98
PRSSL1	1,28	3,84	3,18
PRTFDC1	4,27	4,46	4,56
PRUNE	6,74	6,49	7,45
PSAP	13,94	13,88	13,87
PSAT1	6,82	4,80	-3,84
PSCD1	9,72	9,52	9,80
PSCD4	10,91	10,79	10,88
PSCDBP	10,03	9,43	10,76
PSD	3,26	4,25	3,79
PSD2	3,56	4,02	4,77
PSD3	6,37	7,79	-0,24
PSEN2	9,61	9,27	6,80
PSENEN	7,11	7,48	7,67
PSIP1	6,62	6,39	7,93
PSKH1	5,81	5,29	4,83
PSMA1	10,23	10,56	10,41
PSMA2	8,59	8,32	8,75
PSMA3	10,77	11,13	10,38
PSMA4	10,46	10,59	10,91
PSMA5	11,72	11,83	11,30
PSMA6	10,69	11,01	10,66
PSMA7	9,72	10,29	9,12
PSMB1	12,96	13,03	12,93
PSMB10	12,26	12,13	12,90
PSMB2	10,70	10,99	10,54
PSMB3	12,23	12,39	12,21
PSMB4	11,26	11,40	11,57
PSMB5	10,75	10,76	8,56
PSMB6	10,54	11,30	10,66
PSMB7	10,84	10,92	11,15
PSMB8	9,30	9,96	10,07
PSMB9	6,61	7,24	8,40
PSMC1	12,19	12,46	11,83
PSMC2	12,11	11,96	10,82
PSMC3	10,22	10,78	9,93
PSMC3IP	4,11	4,01	4,13
PSMC4	9,42	9,70	8,78
PSMC5	11,35	11,21	11,33
PSMC6	10,52	10,76	9,97
PSMD10	10,57	11,08	9,97
PSMD11	6,97	7,18	6,49
PSMD13	7,45	7,90	8,03

PSMD14	10,95	11,13	9,26
PSMD2	10,13	10,30	9,74
PSMD3	6,79	7,11	6,61
PSMD5	7,03	7,34	6,74
PSMD6	9,59	9,83	9,37
PSMD7	10,79	10,96	10,33
PSME2	11,99	12,34	12,38
PSME3	8,92	8,91	9,42
PSME4	10,15	10,09	10,29
PSMF1	10,33	10,21	10,60
PSPC1	7,74	7,54	8,54
PSPH	7,39	7,61	5,73
PSRC1	4,46	5,05	6,94
PTAFR	7,67	8,71	8,67
PTBP2	6,52	6,61	8,40
PTCD1	6,86	6,62	6,00
PTCD2	5,39	5,58	5,76
PTDSS1	10,61	10,98	10,95
PTDSS2	7,01	7,08	7,34
PTEN	8,80	8,35	10,63
PTGER2	8,31	7,69	8,53
PTGER3	3,56	2,96	3,24
PTGER4	11,49	11,46	10,77
PTGES	5,28	5,44	5,04
PTGES2	7,57	6,78	6,63
PTGFR	0,72	3,61	2,03
PTGFRN	6,91	5,57	4,31
PTGIR	3,37	3,43	5,68
PTGS1	11,46	10,52	9,27
PTGS2	3,23	6,54	8,65
PTK2	8,31	8,81	8,17
PTK2B	4,35	5,95	7,37
PTMA	12,77	12,13	13,03
PTMS	8,33	8,33	5,56
PTOV1	9,20	8,99	9,40
PTP4A1	7,70	7,75	8,46
PTP4A2	7,22	6,74	9,03
PTPLA	8,50	8,34	5,13
PTPLAD1	9,77	10,23	7,24
PTPLAD2	9,98	9,89	11,42
PTPLB	10,73	10,20	10,29
PTPN12	10,13	10,35	10,47
PTPN13	2,53	4,61	-3,32
PTPN18	6,32	5,85	6,54
PTPN2	7,24	6,23	7,53
PTPN22	6,94	7,93	7,41
PTPN23	9,71	9,12	9,41
PTPN3	3,79	4,27	3,61
PTPN4	7,24	7,06	7,34
PTPN5	3,36	4,19	4,36
PTPN6	9,29	9,69	10,42
PTPN9	6,40	6,02	6,24
PTPRA	7,57	7,61	7,12
PTPRC	6,44	6,76	7,96
PTPRE	11,01	9,74	10,45
PTPRJ	4,66	4,56	4,51
PTPRM	5,53	7,78	0,18
PTPRN2	0,83	2,89	3,31
PTPRO	11,36	10,36	5,99

PTPRR	2,22	3,36	2,76
PTRF	9,57	8,58	4,03
PTRH1	7,60	7,29	6,61
PTRH2	9,07	8,77	8,28
PTS	7,32	7,90	7,28
PTTG1	5,89	8,32	5,07
PTTG1IP	10,74	10,47	10,79
PTX3	4,40	6,49	6,37
PUM1	10,77	10,71	11,19
PUM2	9,80	9,18	10,75
PURA	7,76	7,03	7,44
PURB	10,70	10,41	9,44
PUS3	7,23	7,14	7,29
PUS7	7,78	7,34	7,17
PUS7L	6,17	5,89	5,32
PVALB	6,01	6,69	4,33
PVRL1	4,31	4,31	4,07
PWP1	9,48	9,58	9,21
PXMP3	7,38	7,87	8,15
PXMP4	5,40	5,35	4,72
PXN	7,45	6,56	8,72
PXT1	3,25	3,32	3,32
PYCARD	11,11	11,53	12,37
PYCR1	3,22	1,87	3,12
PYCR2	9,16	9,26	8,95
PYGB	9,06	8,59	8,51
PYGL	11,54	11,40	12,12
PYGO2	6,88	6,05	6,98
PYY	4,40	3,52	3,82
QARS	12,36	12,03	12,42
QDPR	9,35	10,21	8,95
QKI	6,90	6,61	7,38
QPCT	13,05	12,79	11,62
QPCTL	4,80	5,68	4,58
QPRT	9,82	5,96	4,28
QRICH1	5,88	5,39	6,17
QRSL1	3,81	0,83	-0,07
QTRT1	5,50	5,84	5,67
QTRTD1	5,27	5,17	5,57
R3HDM1	7,24	7,41	7,95
R3HDML	3,89	3,62	2,81
RAB10	12,42	12,12	11,97
RAB11A	11,16	11,70	11,58
RAB11B	5,47	5,01	5,72
RAB11FIP1	7,40	7,37	8,31
RAB11FIP2	7,28	7,36	7,65
RAB11FIP3	7,98	7,50	7,73
RAB11FIP4	5,31	5,01	4,81
RAB11FIP5	0,22	4,76	4,56
RAB12	6,02	5,51	8,93
RAB13	8,71	9,83	6,40
RAB14	2,92	2,28	4,17
RAB15	5,29	3,39	4,40
RAB18	7,39	7,47	6,84
RAB1A	10,42	10,68	9,80
RAB1B	7,35	7,79	7,53
RAB20	11,52	12,22	11,13
RAB21	9,88	9,83	9,67
RAB22A	9,40	9,50	9,03

RAB23	4,32	5,69	3,30
RAB24	8,07	7,81	10,69
RAB28	8,00	8,56	8,85
RAB2B	10,56	10,49	10,04
RAB30	5,98	4,27	4,65
RAB31	13,16	12,74	12,75
RAB33A	9,93	0,34	3,15
RAB33B	7,24	6,65	9,16
RAB34	8,75	8,65	8,89
RAB35	9,84	9,20	9,40
RAB36	-0,51	4,36	0,39
RAB37	3,49	3,67	5,92
RAB38	9,69	8,61	5,96
RAB3B	4,43	4,59	4,31
RAB3GAP1	10,40	10,17	10,40
RAB3GAP2	10,22	10,10	10,40
RAB3IL1	7,76	8,94	-0,07
RAB3IP	6,96	7,11	8,68
RAB40C	4,53	4,27	6,01
RAB4A	7,56	7,75	7,85
RAB4B	7,84	7,88	8,00
RAB5A	11,58	11,02	11,11
RAB5B	11,42	11,20	11,45
RAB6A	8,77	8,58	8,71
RAB6B	4,07	4,20	7,15
RAB6IP1	10,73	10,45	11,49
RAB7L1	9,08	9,07	8,96
RAB8B	7,77	7,92	7,91
RAB9A	11,02	10,95	10,60
RAB9B	4,54	4,77	4,65
RABEP1	9,87	9,70	8,99
RABEP2	3,90	3,38	3,59
RABEPK	11,84	11,14	9,77
RABGAP1	10,57	9,91	10,44
RABGAP1L	7,28	7,17	8,61
RABGEF1	8,60	9,00	8,48
RABGGTA	7,86	8,35	7,75
RABGGTB	10,82	10,82	10,63
RABIF	5,57	5,75	6,50
RABL2A	4,70	4,81	4,99
RABL2B	5,47	5,17	5,75
RABL3	8,02	8,37	6,23
RABL4	7,50	8,13	7,45
RAC2	13,39	14,12	13,29
RACGAP1	4,81	5,41	3,33
RAD1	4,90	4,61	4,43
RAD17	6,05	6,27	5,98
RAD18	3,97	4,29	-1,16
RAD21	8,15	7,81	8,46
RAD23B	8,47	8,26	9,54
RAD50	6,33	6,20	6,42
RAD51	-2,93	4,96	-3,32
RAD51AP1	7,36	8,51	5,44
RAD51C	6,80	7,49	5,89
RAD51L1	4,66	4,42	4,51
RAD51L3	5,67	5,84	5,32
RAD52	4,04	4,26	4,35
RAD54L	1,67	5,05	-1,46
RAF1	8,95	9,11	9,90

RAG1AP1	10,20	9,99	8,62
RAGE	4,25	5,28	5,48
RAI1	6,56	6,00	6,71
RAI14	6,70	9,55	4,57
RALA	10,36	11,27	9,86
RALB	11,50	11,79	11,92
RALBP1	8,69	8,85	9,21
RALGDS	11,93	12,06	10,07
RALGPS1	5,39	5,32	5,91
RALGPS2	1,91	1,59	2,96
RALY	10,33	9,70	10,43
RAMP1	10,77	2,55	1,65
RANBP1	8,92	9,43	8,86
RANBP10	7,02	7,29	7,91
RANBP3	6,20	5,60	5,74
RANBP6	7,80	7,98	8,55
RANBP9	8,88	8,52	8,62
RANGAP1	9,92	10,00	9,56
RAP1A	7,56	6,88	7,82
RAP1B	9,01	9,16	9,51
RAP1GAP	11,54	9,89	-1,57
RAP1GDS1	8,87	9,24	8,54
RAP2A	10,97	11,40	9,77
RAP2B	5,03	4,97	5,69
RAP2C	9,74	9,54	9,94
RAPGEF1	10,02	9,80	8,93
RAPGEF3	7,44	8,30	2,79
RAPGEF6	7,95	7,84	8,43
RAPGEFL1	4,14	4,55	5,72
RAPH1	7,20	6,53	4,54
RARA	7,92	8,12	10,09
RARRES2	5,84	5,62	6,39
RARRES3	6,47	7,69	10,37
RARS	11,31	11,52	10,60
RASA2	5,50	5,75	6,93
RASAL1	7,16	3,03	-3,32
RASAL2	7,19	7,10	2,87
RASD1	3,04	3,30	5,16
RASD2	3,27	4,24	3,50
RASGEF1A	3,86	4,44	4,35
RASGEF1B	4,13	6,80	4,22
RASGRF1	3,05	4,40	3,00
RASGRP1	7,07	5,70	-0,15
RASGRP2	4,27	4,56	8,77
RASIP1	5,56	5,63	5,57
RASL10B	5,13	4,60	4,98
RASL11B	3,14	6,36	-3,32
RASSF1	7,41	6,56	8,00
RASSF2	9,14	8,32	10,71
RASSF3	5,60	5,24	5,80
RASSF4	9,25	10,42	9,24
RASSF5	5,24	5,95	6,93
RASSF7	9,21	8,18	9,02
RASSF8	5,08	4,81	4,86
RAVER1	8,98	8,68	9,61
RAVER2	3,37	5,18	5,66
RB1	7,17	7,01	7,71
RB1CC1	9,16	9,17	9,89
RBBP4	6,09	5,84	6,41

RBBP5	7,81	7,64	7,63
RBBP6	4,11	4,50	5,93
RBBP7	10,25	10,23	9,74
RBBP8	2,90	3,27	3,76
RBBP9	6,67	6,56	6,55
RBED1	6,29	5,79	6,71
RBJ	5,77	6,25	6,51
RBKS	5,68	5,76	6,26
RBL2	9,41	9,09	10,33
RBM10	6,71	6,34	7,14
RBM11	7,78	4,81	4,78
RBM12	6,72	6,66	7,06
RBM12B	7,54	7,05	7,39
RBM13	8,17	8,25	7,40
RBM15	9,16	8,92	9,05
RBM16	7,39	6,92	8,03
RBM18	8,81	8,89	8,35
RBM22	11,43	11,25	12,16
RBM23	10,25	10,21	11,01
RBM25	10,16	9,86	10,57
RBM28	6,45	6,17	6,38
RBM3	7,10	7,31	7,98
RBM34	9,54	9,44	9,54
RBM35A	3,71	4,11	3,75
RBM4	6,86	6,15	7,71
RBM4B	8,31	7,91	8,06
RBM5	10,51	10,08	11,17
RBM7	8,37	7,78	8,21
RBM8A	3,72	4,62	4,40
RBMS1	9,10	8,84	9,07
RBMS2	9,08	6,72	6,91
RBMX	10,81	10,61	11,41
RBMX2	5,36	5,08	5,74
RBP1	4,46	5,11	-1,35
RBP7	5,73	7,40	11,18
RBPMS2	-0,97	0,89	-0,98
RBX1	12,13	12,04	11,89
RCBTB1	6,85	6,11	5,98
RCBTB2	5,77	8,04	7,71
RCC2	11,15	11,38	11,54
RCE1	8,19	7,82	8,34
RCHY1	5,69	5,33	6,95
RCL1	6,22	6,99	4,94
RCN1	9,24	9,50	9,39
RCN2	9,52	9,84	8,13
RCOR1	2,56	-1,22	4,94
RCOR3	8,96	8,43	9,64
RCP9	5,29	5,16	5,12
RCSD1	8,22	7,74	10,85
RDBP	6,40	6,37	6,57
RDH10	7,52	8,16	6,61
RDH11	11,49	11,69	10,20
RDH13	6,65	6,00	6,04
RDH8	4,26	4,23	3,95
RDHE2	4,08	3,73	4,21
RDM1	3,77	3,94	3,79
RDX	9,14	9,77	7,84
RECK	4,08	4,73	5,53
RECQL	8,42	8,27	8,24

RECQL4	0,00	4,37	2,62
RECQL5	3,99	3,77	4,42
REEP1	5,69	6,97	4,15
REEP3	6,43	7,21	4,93
REEP4	6,56	6,37	7,47
REEP5	12,37	11,41	12,12
REEP6	3,51	3,61	4,43
REL	7,67	7,01	8,21
RELA	6,99	6,58	6,87
REPIN1	8,57	7,64	8,26
REPS2	4,95	4,24	6,46
RER1	8,89	8,92	7,96
RERE	9,18	8,57	10,36
REST	5,31	5,10	4,83
RET	2,51	3,57	3,20
RETN	-2,45	6,69	8,71
REV3L	7,07	6,68	8,13
REXO2	9,71	10,26	9,18
REXO4	8,90	8,66	8,49
RFC1	6,25	6,51	6,69
RFC2	5,95	6,13	5,86
RFC3	4,30	5,90	5,08
RFC4	7,99	8,52	6,89
RFC5	6,71	7,48	7,18
RFFL	6,89	6,78	7,63
RFK	5,22	5,28	4,65
RFNG	8,12	7,77	7,61
RFP	6,66	6,46	8,23
RFT1	4,08	4,27	3,35
RFWD2	11,11	11,08	11,48
RFWD3	8,49	8,52	7,71
RFX1	7,63	7,04	8,96
RFX2	4,99	4,95	5,58
RFX3	6,48	6,64	7,00
RFX4	3,30	3,73	3,81
RFX5	10,42	9,92	10,77
RFXANK	8,03	7,85	8,38
RFXAP	7,53	6,13	6,81
RG9MTD1	7,61	7,39	6,36
RG9MTD2	5,46	5,20	5,54
RGAG1	4,30	4,20	2,78
RGAG4	5,99	5,66	4,37
RGL1	11,60	10,00	7,11
RGL2	9,67	8,76	10,30
RGMA	4,63	4,61	5,96
RGMB	2,43	3,34	3,11
RGS1	9,77	10,49	0,64
RGS10	10,44	9,85	10,22
RGS11	4,84	4,18	4,16
RGS12	8,34	8,51	5,95
RGS14	6,03	4,62	7,20
RGS16	5,97	8,50	3,27
RGS19	10,31	9,96	11,73
RGS2	7,86	7,55	12,76
RGS20	3,29	7,77	-3,32
RGS6	3,24	4,09	4,31
RHBDD2	9,73	10,75	9,42
RHBDD3	6,42	6,27	5,57
RHBDF1	8,78	9,55	1,67

RHBDF2	8,14	10,20	10,14
RHBDL3	4,39	1,47	0,78
RHBG	3,22	4,04	3,73
RHCG	3,85	1,57	4,10
RHEB	12,10	12,01	10,57
RHOA	13,19	13,10	13,57
RHOB	8,18	7,93	11,52
RHOBTB1	2,72	2,83	2,09
RHOBTB2	4,75	5,51	4,53
RHOBTB3	5,29	6,56	-2,71
RHOC	10,00	10,79	9,75
RHOG	11,64	11,29	12,56
RHOQ	12,30	12,39	12,13
RHOT1	8,94	9,19	9,95
RHPN1	4,89	2,97	1,08
RIC8A	9,87	9,87	9,92
RIC8B	7,52	6,95	7,32
RICTOR	9,26	8,92	10,28
RIF1	5,01	5,06	5,35
RILP	6,50	5,87	4,94
RIMS4	5,24	5,34	6,08
RIN1	5,50	5,35	6,99
RIN2	10,29	10,11	9,83
RIN3	6,98	6,82	8,97
RINT1	6,22	6,55	6,23
RIOK1	5,65	6,09	6,61
RIOK2	8,81	8,79	8,17
RIPK1	8,32	8,21	8,41
RIPK2	10,32	10,65	11,07
RIPK3	7,32	7,57	7,83
RIPK4	4,11	4,35	3,26
RIPK5	6,96	7,05	7,24
RIT1	9,15	9,46	8,40
RLF	7,39	6,94	8,13
RLN1	3,87	4,10	3,69
RMND5A	2,87	3,23	6,30
RNASE1	12,06	8,41	6,77
RNASE2	3,47	5,71	11,53
RNASE3	1,96	3,20	6,60
RNASE4	5,13	6,57	8,54
RNASE6	11,00	10,49	11,34
RNASEH1	8,45	8,80	8,94
RNASEL	8,66	9,01	7,65
RNASEN	8,11	8,04	8,37
RND2	4,37	3,30	3,63
RND3	0,46	2,42	-3,32
RNF103	7,81	8,29	7,57
RNF11	7,79	8,03	7,76
RNF111	4,54	4,17	4,42
RNF113A	9,35	9,32	9,49
RNF12	1,92	2,32	3,32
RNF121	6,48	6,85	5,95
RNF123	7,49	7,52	7,68
RNF125	5,68	6,04	6,92
RNF13	10,05	9,97	9,59
RNF135	8,47	8,77	9,07
RNF144	2,42	5,36	5,70
RNF146	5,46	5,18	5,43
RNF149	11,08	11,84	11,90

RNF157	3,78	4,08	3,91
RNF166	7,16	6,74	8,98
RNF167	8,73	8,61	9,02
RNF168	4,45	4,11	4,68
RNF170	8,26	7,89	7,19
RNF175	0,97	3,62	4,50
RNF180	4,89	4,91	5,08
RNF182	3,23	3,30	3,91
RNF185	6,10	6,27	5,20
RNF19	9,96	9,21	9,16
RNF20	10,16	9,95	10,35
RNF24	8,23	7,08	8,32
RNF25	5,22	5,53	5,34
RNF26	6,88	7,15	7,22
RNF31	8,09	7,31	8,79
RNF32	3,86	4,05	4,09
RNF38	9,63	9,26	10,72
RNF4	8,44	8,08	8,11
RNF40	7,38	7,07	7,70
RNF41	5,87	6,19	6,97
RNF44	8,36	7,54	10,00
RNF6	6,13	6,31	5,51
RNF7	10,82	11,06	10,63
RNGTT	8,81	9,07	8,56
RNMT	9,62	9,18	8,75
RNMTL1	8,58	8,59	8,55
RNPC2	4,56	4,03	6,53
RNPEPL1	6,67	6,35	6,98
RNPS1	9,31	9,08	10,02
ROBO3	6,26	6,18	4,74
ROBO4	3,26	3,14	4,17
ROCK1	8,22	8,47	8,48
ROCK2	7,44	7,32	8,06
ROD1	11,83	11,45	12,44
ROGDI	8,80	7,64	9,35
ROM1	6,34	6,53	6,59
ROPN1L	-0,53	1,14	4,85
RORA	3,30	3,87	3,61
RP11-529I10.4	7,56	7,91	7,74
RP13-360B22.2	4,94	5,14	5,27
RP9	8,78	8,61	8,85
RPA1	8,72	9,12	8,49
RPA2	8,68	8,56	8,88
RPAP1	6,33	6,32	6,45
RPGR	5,82	6,43	8,04
RPH3A	3,79	3,88	4,69
RPH3AL	3,72	4,94	3,12
RPIA	9,12	8,29	9,80
RPL10A	11,25	11,20	12,11
RPL11	14,15	14,14	14,67
RPL12	13,26	13,01	13,37
RPL13	8,29	7,97	9,81
RPL13A	11,78	11,19	12,33
RPL14	11,97	11,87	12,80
RPL15	11,87	11,93	12,86
RPL17	12,54	12,40	13,04
RPL19	13,55	13,58	14,14
RPL22	12,74	12,68	13,09
RPL23	12,86	12,73	13,44

RPL23A	8,81	8,62	9,51
RPL26	12,38	12,23	13,17
RPL26L1	8,00	8,54	8,30
RPL27	13,45	13,38	13,78
RPL27A	10,23	9,94	11,15
RPL28	6,76	6,20	8,05
RPL29	6,33	6,68	5,41
RPL3	13,63	13,39	13,86
RPL30	13,29	13,32	13,97
RPL31	13,49	13,38	14,15
RPL32	12,88	12,77	13,22
RPL34	7,19	7,22	8,03
RPL35A	12,72	12,66	13,65
RPL36A	4,71	4,33	5,54
RPL36AL	13,00	12,94	13,13
RPL37	5,28	5,47	6,77
RPL37A	7,67	7,66	8,35
RPL38	14,23	14,15	14,69
RPL39	13,72	13,73	14,24
RPL4	12,00	11,51	12,68
RPL41	4,81	5,81	5,72
RPL5	13,77	13,50	13,98
RPL6	13,22	13,13	13,52
RPL7	11,22	11,04	12,43
RPL7A	11,65	11,33	12,59
RPL7L1	8,63	8,53	8,21
RPL8	12,61	12,43	12,84
RPL9	13,03	12,99	13,25
RPLP0	13,56	13,34	13,10
RPLP1	12,23	11,98	12,97
RPLP2	13,33	13,15	13,79
RPN1	11,84	12,08	11,85
RPN2	11,15	11,40	11,11
RPP25	7,53	7,43	6,83
RPP30	4,73	5,18	5,34
RPP38	5,49	5,11	4,57
RPP40	6,61	7,05	8,37
RPRM	3,63	3,96	2,91
RPRML	3,80	3,99	4,16
RPS10	13,78	13,82	13,96
RPS11	14,03	13,86	14,24
RPS12	13,78	13,91	13,98
RPS13	13,38	13,32	14,24
RPS14	13,55	13,39	14,13
RPS15A	13,65	13,71	14,37
RPS16	14,42	14,33	14,46
RPS17	12,67	12,15	12,90
RPS18	12,98	12,62	13,54
RPS19	14,27	14,19	14,32
RPS19BP1	9,35	9,36	8,25
RPS20	13,87	13,91	14,12
RPS21	9,61	10,08	10,10
RPS23	9,94	9,72	11,22
RPS24	14,23	14,11	14,26
RPS25	14,33	14,28	14,62
RPS26	5,05	4,82	3,97
RPS27	14,10	13,78	14,17
RPS27A	13,46	13,27	13,93
RPS27L	13,02	13,00	11,85

RPS28	14,04	13,86	13,98
RPS29	14,66	14,59	14,53
RPS3	12,93	12,89	13,60
RPS3A	13,22	12,98	13,67
RPS4X	12,47	12,38	13,02
RPS5	12,29	12,30	12,45
RPS6	13,77	13,72	14,56
RPS6KA1	10,23	10,38	9,35
RPS6KA2	6,69	7,76	4,58
RPS6KA3	7,27	7,47	7,36
RPS6KB1	8,72	8,66	10,16
RPS6KB2	6,67	6,57	6,85
RPS6KC1	6,27	6,33	7,11
RPS6KL1	5,17	4,53	5,11
RPS7	7,27	7,99	7,08
RPS8	14,02	13,53	13,78
RPS9	12,54	12,71	13,38
RPSA	4,70	4,86	5,55
RPUSD2	8,29	8,32	7,52
RPUSD3	8,88	8,89	8,88
RPUSD4	10,08	10,02	10,58
RQCD1	6,24	6,40	4,91
RRAGA	10,22	10,35	10,25
RRAGB	7,72	8,03	7,57
RRAGC	10,03	9,67	8,72
RRAGD	11,77	11,08	9,07
RRAS	9,52	10,51	9,99
RRBP1	6,59	6,77	6,00
RREB1	3,23	3,13	3,26
RRM1	10,25	10,84	9,18
RRM2	3,35	6,61	3,81
RRM2B	8,86	9,08	8,53
RRN3	9,19	8,74	8,68
RRS1	10,33	8,01	8,28
RSAD2	-0,40	4,48	6,48
RSBN1	8,80	8,03	9,04
RSBN1L	7,79	7,58	7,51
RSL1D1	9,62	9,48	10,45
RSPO3	2,48	4,45	0,14
RSPRY1	10,14	9,80	9,95
RSRC1	8,09	8,43	7,30
RSU1	9,42	9,12	8,98
RTCD1	9,61	9,75	8,32
RTEL1	4,70	4,20	4,11
RTF1	5,94	6,29	6,75
RTKN	6,44	6,14	3,90
RTN3	9,12	9,42	10,29
RTN4	11,75	11,61	10,96
RTN4IP1	8,22	8,74	7,52
RTN4RL2	3,84	4,59	4,37
RTP4	5,74	5,50	5,52
RTTN	2,67	4,02	3,90
RUFY1	7,55	7,33	7,51
RUFY2	5,58	5,16	5,61
RUNDC1	8,58	8,02	8,58
RUNDC2A	5,05	4,89	5,57
RUNX1	7,52	7,13	7,41
RUNX2	5,63	4,25	6,92
RUTBC1	10,51	10,14	10,44

RUVBL1	6,12	6,38	6,32
RUVBL2	7,99	8,35	8,59
RWDD1	3,90	3,82	5,87
RWDD3	6,81	6,81	6,45
RWDD4A	8,93	9,03	9,04
RXRB	8,29	8,42	8,90
RXRG	3,96	3,88	3,96
RYBP	10,93	9,66	10,59
RYK	10,03	9,20	9,21
RYR1	6,33	6,65	6,28
RETSAT	6,39	6,61	5,39
S100A10	13,23	13,30	13,84
S100A11	12,44	12,85	12,29
S100A12	-3,32	-1,18	12,31
S100A13	5,37	6,72	4,67
S100A4	13,94	14,43	14,70
S100A5	5,52	4,42	4,23
S100A6	13,86	13,99	13,77
S100A8	7,39	12,67	14,58
S100A9	7,03	10,91	13,54
S100PBP	5,69	5,51	6,00
S100Z	2,28	6,17	8,87
SAAL1	8,69	8,67	8,46
SAC3D1	8,80	8,68	8,32
SACM1L	10,05	9,84	9,97
SAE1	11,50	11,31	11,23
SAFB	8,77	8,35	9,41
SAFB2	9,06	8,32	9,95
SAMD1	6,14	5,78	5,98
SAMD11	4,11	4,36	4,19
SAMD13	3,90	4,49	-3,32
SAMD14	4,00	4,24	3,55
SAMD4A	7,52	6,73	7,92
SAMD4B	6,36	6,02	6,53
SAMD8	3,95	3,70	4,28
SAMD9	4,55	4,30	5,03
SAMD9L	8,76	8,48	9,23
SAMHD1	6,59	7,22	7,70
SAMM50	9,56	10,00	9,24
SAMSN1	10,82	8,84	9,98
SAP130	8,32	9,01	8,42
SAP18	7,61	7,80	8,39
SAP30	9,04	8,99	10,75
SAP30BP	7,27	7,27	7,61
SAP30L	9,84	9,50	10,34
SAPS1	7,40	7,62	8,05
SAPS3	8,35	8,42	9,61
SAR1A	9,48	9,92	10,06
SAR1B	10,15	10,17	8,80
SARM1	4,10	3,03	4,82
SARS	11,48	11,20	11,41
SARS2	8,78	8,56	9,01
SART3	4,66	4,36	4,52
SAS10	10,42	10,24	10,16
SASH1	7,36	7,56	9,04
SASS6	5,90	6,25	6,38
SAT2	9,90	9,78	10,91
SATB1	3,68	3,54	6,29
SATB2	6,65	5,55	5,19

SATL1	4,58	4,94	4,37
SAV1	8,21	9,06	6,60
SBDS	9,03	8,72	9,03
SBF2	4,86	3,68	5,47
SC4MOL	11,52	11,47	6,41
SC5DL	9,44	9,85	6,67
SC65	6,20	6,17	6,61
SCAMP1	9,84	10,34	10,06
SCAMP2	7,64	8,01	7,44
SCAMP3	10,06	10,55	9,21
SCAMP4	5,94	5,94	5,78
SCAND1	8,88	8,80	9,51
SCAND2	3,66	3,07	4,00
SCAP	9,53	9,45	10,08
SCARB2	11,75	12,44	11,42
SCARF1	4,64	3,79	5,01
SCD	13,47	13,77	6,96
SCFD2	7,07	7,49	7,55
SCG5	3,36	10,90	2,87
SCGB1C1	3,99	3,77	4,55
SCGN	1,91	3,13	4,13
SCLY	6,30	5,37	5,91
SCMH1	4,95	6,53	5,93
SCML1	8,57	8,20	7,37
SCML2	3,67	3,85	4,26
SCN8A	4,01	4,25	4,04
SCNM1	8,51	8,44	9,22
SCNN1B	4,09	1,64	4,03
SCO1	9,80	9,85	8,30
SCO2	7,11	6,74	9,19
SCP2	6,76	6,97	5,45
SCPEP1	11,28	12,41	12,27
SCRN3	4,46	4,67	3,29
SCRT2	3,96	3,67	4,76
SCTR	3,44	-1,06	3,96
SCYE1	2,87	2,05	5,19
SCYL1BP1	7,84	8,50	7,70
SCYL2	10,61	10,39	10,17
SCYL3	5,87	5,60	6,03
SDC2	8,78	9,31	5,02
SDC4	10,84	11,13	6,09
SDCBP	12,88	12,80	11,88
SDCCAG1	9,15	8,98	8,43
SDCCAG10	8,77	9,39	9,59
SDCCAG3	8,15	7,76	8,92
SDCCAG8	7,63	7,60	6,75
SDF2	10,24	9,95	9,94
SDF2L1	10,29	10,94	10,29
SDHA	9,14	9,49	8,69
SDHB	12,02	12,40	11,71
SDHC	9,96	10,50	9,06
SDHD	11,44	11,18	10,62
SDK1	5,10	4,89	4,43
SDPR	5,34	3,38	3,02
SDS	6,44	6,83	4,04
SEC14L1	7,43	7,33	7,56
SEC14L2	2,01	4,58	-3,32
SEC23A	6,85	6,98	6,69
SEC23B	10,43	10,32	10,04

SEC23IP	8,51	8,75	8,52
SEC24B	5,29	5,20	6,05
SEC24C	9,68	9,55	9,68
SEC24D	9,21	9,54	9,17
SEC61A1	9,38	9,46	9,28
SEC61B	10,02	9,80	9,67
SEC61G	12,41	12,62	12,64
SEC63	7,95	8,26	8,72
SECISBP2	7,97	7,74	8,97
SECTM1	2,71	3,30	5,34
SEH1L	7,45	7,52	6,84
SEL1L	6,78	7,09	6,53
SELI	6,16	6,74	6,47
SELM	4,52	5,06	3,50
SELS	10,24	10,30	9,63
SELT	9,69	9,17	9,00
SELV	5,37	5,40	3,92
SEMA3A	3,54	5,05	-0,81
SEMA4B	4,85	5,52	8,04
SEMA4G	3,80	4,18	4,62
SEMA5B	4,23	3,91	3,72
SEMA6A	5,25	4,35	4,23
SEMA6B	5,38	5,84	4,92
SENP1	4,04	2,61	3,25
SENP2	6,32	6,43	7,10
SENP3	4,46	4,51	4,92
SENP5	6,97	6,58	5,72
SENP6	8,51	7,66	9,14
SENP7	3,04	2,44	5,28
SENP8	5,43	4,76	3,99
SEPHS1	7,64	7,10	6,91
SEPHS2	10,37	10,68	8,60
SEPP1	3,45	5,65	-3,32
SEPX1	11,47	11,51	12,45
SERAC1	6,35	6,61	6,57
SERBP1	9,72	9,60	10,04
SERF2	13,47	13,43	12,51
SERGEF	8,18	8,07	8,06
SERINC1	10,42	10,27	10,17
SERINC2	7,87	8,18	6,46
SERINC3	9,37	9,26	9,15
SERINC4	3,96	3,75	4,24
SERINC5	4,95	4,19	4,89
SERP1	9,77	9,35	10,62
SERPINA1	8,91	11,56	12,35
SERPINA12	5,38	5,19	4,98
SERPINA6	3,54	3,66	4,08
SERPINB1	11,20	11,14	12,19
SERPINB2	4,40	3,89	6,95
SERPINB8	8,16	8,50	8,12
SERPINB9	3,93	3,67	6,64
SERPIND1	4,39	4,02	3,73
SERPINE1	7,34	7,02	-0,60
SERPINE2	5,83	4,26	-0,38
SERPINI1	4,76	6,70	0,92
SERTAD1	10,16	9,89	8,33
SERTAD2	9,82	10,32	10,81
SERTAD3	7,49	6,45	8,32
SERTAD4	4,35	3,95	3,34

SESN1	7,42	8,30	7,27
SESN2	5,84	5,28	5,05
SESN3	5,51	4,57	5,79
SET	11,49	11,25	11,61
SETBP1	6,46	-0,14	6,55
SETD1A	8,43	7,82	8,68
SETD3	10,32	10,48	9,63
SETD5	5,44	5,93	6,04
SETD6	5,60	5,41	7,07
SETDB1	6,87	6,55	7,73
SETDB2	10,68	10,35	8,94
SEZ6L	4,28	1,31	4,08
SF1	5,69	5,22	7,61
SF3A1	6,97	6,45	7,63
SF3A2	8,97	8,60	9,97
SF3A3	9,45	9,31	10,16
SF3B1	9,83	9,74	10,55
SF3B14	10,67	10,49	10,77
SF3B2	12,00	11,95	12,18
SF3B3	8,56	8,30	8,49
SF3B4	10,48	10,11	11,17
SF3B5	10,51	10,53	10,96
SF4	6,90	6,62	7,21
SFI1	3,40	3,99	4,25
SFMBT2	5,56	5,76	5,93
SFPQ	9,25	8,75	9,64
SFRP4	4,47	3,61	2,97
SFRS1	11,60	11,47	11,66
SFRS10	10,52	10,58	10,91
SFRS11	9,28	8,98	10,09
SFRS14	9,16	8,91	9,68
SFRS15	7,88	7,18	7,56
SFRS2	11,56	11,75	12,10
SFRS3	8,92	8,88	9,26
SFRS5	11,34	11,42	12,40
SFRS6	11,40	11,46	12,79
SFRS7	9,70	9,81	8,90
SFRS8	7,80	7,57	8,76
SFRS9	12,09	12,11	12,28
SFT2D1	12,34	12,20	11,41
SFT2D2	5,86	5,38	4,98
SFXN1	7,61	7,41	6,67
SFXN2	2,39	5,19	0,41
SFXN3	10,48	10,45	9,81
SFXN4	8,19	8,51	7,54
SFXN5	6,11	5,81	6,92
SGCB	4,56	4,39	4,60
SGK	13,22	13,73	12,36
SGOL1	-3,32	4,26	-3,32
SGOL2	5,11	6,09	4,24
SGPP1	11,44	11,20	9,87
SGPP2	4,16	0,29	-3,32
SGSH	7,35	6,77	7,78
SGTB	3,42	3,84	4,34
SH2D1A	4,79	4,65	4,86
SH2D1B	-2,42	-3,32	5,19
SH2D3C	7,37	7,79	8,79
SH2D4A	3,19	0,93	3,30
SH3BGR	5,06	5,35	4,43

SH3BGRL	12,76	12,89	12,72
SH3BGRL3	12,99	12,81	12,66
SH3BP1	8,75	8,50	9,52
SH3BP4	6,80	5,79	7,00
SH3BP5	0,97	5,09	2,60
SH3BP5L	7,54	7,75	6,98
SH3GL1	8,09	7,80	8,17
SH3GLB1	9,07	9,48	8,70
SH3PX3	7,37	6,72	6,57
SH3PXD2A	9,87	9,51	6,45
SH3PXD2B	5,05	6,75	4,05
SH3RF1	3,14	5,92	6,34
SH3RF2	3,82	4,06	4,43
SH3YL1	8,14	7,97	7,43
SHANK1	3,57	3,78	4,05
SHARPIN	8,13	8,20	8,23
SHB	8,33	7,40	3,89
SHC1	9,01	8,87	8,94
SHCBP1	3,96	4,58	2,50
SHFM1	6,92	7,30	6,42
SHH	3,35	3,56	2,67
SHMT1	5,51	6,52	6,85
SHMT2	11,12	10,45	10,39
SHOX2	3,50	4,22	3,59
SHPRH	5,58	4,98	6,38
SHQ1	6,72	7,07	7,15
SIAE	7,39	7,37	6,79
SIAH1	9,30	8,90	8,68
SIAH2	7,18	6,84	8,59
SIDT1	-3,32	4,64	4,03
SIDT2	12,93	12,50	12,00
SIGLEC10	9,96	7,41	9,40
SIGLEC11	2,18	5,65	5,14
SIGLEC12	7,11	4,12	3,73
SIGLEC7	6,70	6,96	6,44
SIGLEC9	8,02	7,95	7,31
SIKE	4,26	-0,35	4,43
SIL1	10,28	11,15	9,49
SILV	4,71	4,82	4,22
SIN3A	8,55	8,30	8,97
SIN3B	6,89	6,05	8,24
SIP1	7,12	7,29	7,32
SIPA1	9,00	8,56	10,34
SIPA1L3	6,08	5,78	5,76
SIRPA	13,95	13,74	13,01
SIRPB1	6,93	7,92	9,74
SIRT1	9,30	8,12	9,62
SIRT2	7,33	7,03	7,85
SIRT5	6,77	6,88	6,41
SIRT6	4,97	4,63	3,83
SIX3	3,48	4,48	3,84
SIX5	5,45	4,33	3,81
SKI	5,75	5,49	5,62
SKIL	5,24	5,88	5,39
SKIP	4,05	3,56	4,56
SKIV2L	9,10	8,76	8,66
SKIV2L2	9,58	9,64	9,22
SKP2	6,12	6,15	7,61
SLA	12,50	10,29	10,56

SLAMF1	8,26	5,59	3,07
SLAMF6	3,84	5,66	3,04
SLAMF7	10,19	9,74	5,76
SLAMF8	11,03	11,45	5,67
SLBP	8,36	8,52	8,50
SLC11A1	5,82	9,58	10,53
SLC11A2	12,24	11,20	9,52
SLC12A2	5,72	5,55	5,21
SLC12A6	6,93	7,59	7,09
SLC12A7	6,59	6,28	6,73
SLC12A8	5,92	8,10	4,05
SLC13A3	3,23	2,68	3,52
SLC15A3	12,57	12,71	11,11
SLC16A10	7,03	9,79	5,68
SLC16A12	3,81	2,23	1,58
SLC16A2	5,11	5,01	4,67
SLC16A5	7,19	7,21	8,89
SLC16A6	7,75	7,39	7,74
SLC16A8	4,67	4,50	4,34
SLC17A5	11,81	12,13	9,20
SLC18A2	6,05	-3,32	-3,32
SLC19A2	6,81	7,18	7,12
SLC1A2	5,88	5,03	4,74
SLC1A3	8,93	10,79	5,05
SLC1A4	6,39	8,41	5,96
SLC1A5	8,85	9,11	8,48
SLC20A1	11,79	11,97	10,57
SLC20A2	6,33	6,38	6,14
SLC22A15	5,92	7,30	8,56
SLC22A17	3,46	3,73	6,22
SLC22A18	7,36	7,55	7,33
SLC22A4	5,73	5,96	7,73
SLC22A5	6,81	7,20	6,49
SLC22A7	3,78	3,49	2,94
SLC23A1	5,61	5,72	5,21
SLC23A2	5,87	6,39	6,07
SLC24A2	3,32	3,91	4,12
SLC24A3	3,07	5,85	1,87
SLC24A4	5,64	5,23	8,23
SLC24A6	10,19	10,54	9,33
SLC25A10	4,54	5,11	4,23
SLC25A11	8,60	8,49	8,07
SLC25A12	7,54	7,39	6,92
SLC25A13	8,81	8,83	8,24
SLC25A14	6,44	7,02	7,50
SLC25A17	6,09	6,02	5,71
SLC25A19	9,12	10,06	7,89
SLC25A20	8,19	8,07	8,73
SLC25A22	6,50	6,36	7,08
SLC25A23	8,58	8,30	5,80
SLC25A24	6,61	6,78	6,24
SLC25A25	9,01	7,59	7,46
SLC25A28	9,56	9,82	11,09
SLC25A29	7,97	7,16	7,03
SLC25A3	11,31	11,27	11,04
SLC25A30	5,17	4,67	4,23
SLC25A32	4,23	4,23	4,82
SLC25A35	6,84	6,79	5,41
SLC25A36	7,82	7,59	8,66

SLC25A5	13,91	14,03	13,56
SLC26A10	3,15	3,53	3,42
SLC26A2	7,66	7,37	7,70
SLC26A5	3,28	3,06	2,74
SLC26A6	10,32	9,24	9,33
SLC26A8	3,06	3,28	4,03
SLC27A1	8,39	9,48	9,05
SLC27A3	12,60	12,15	11,33
SLC27A4	1,17	4,29	4,38
SLC27A6	5,90	5,98	5,97
SLC29A1	8,81	9,64	6,73
SLC29A2	5,04	5,29	3,32
SLC2A1	4,50	4,06	-1,16
SLC2A11	4,40	3,02	3,79
SLC2A14	8,25	8,25	8,48
SLC2A3	12,76	12,16	12,98
SLC2A4RG	5,35	5,60	6,00
SLC2A5	5,57	8,44	4,32
SLC2A6	11,06	9,91	10,71
SLC2A8	8,33	8,48	7,16
SLC2A9	6,59	7,80	7,05
SLC30A1	7,52	8,13	9,05
SLC30A3	7,05	10,59	3,81
SLC30A4	4,74	-3,32	-2,43
SLC30A6	5,14	4,74	3,69
SLC30A7	10,26	10,16	10,53
SLC30A9	10,27	10,21	10,02
SLC31A1	9,93	10,90	9,27
SLC31A2	10,21	11,01	11,11
SLC33A1	8,91	8,88	8,04
SLC35A1	9,91	10,21	11,37
SLC35A2	7,85	7,96	6,90
SLC35A3	8,39	8,71	8,74
SLC35A4	7,01	7,18	7,23
SLC35A5	11,07	10,56	10,86
SLC35B1	10,05	10,65	10,31
SLC35B2	9,03	7,87	8,87
SLC35B3	7,82	7,82	7,76
SLC35B4	4,54	5,19	4,80
SLC35C2	6,51	6,79	7,43
SLC35D3	3,03	4,12	2,70
SLC35E1	9,14	8,79	9,19
SLC35E2	3,36	2,96	4,32
SLC35E3	10,22	9,82	9,12
SLC35F2	-3,32	1,18	-0,23
SLC35F3	4,92	3,97	4,05
SLC36A1	10,00	10,70	9,88
SLC36A4	8,39	8,76	9,10
SLC37A3	5,93	3,51	5,44
SLC38A1	4,27	6,63	5,87
SLC38A2	10,68	11,13	11,17
SLC38A5	4,58	4,48	4,69
SLC38A6	13,12	12,21	7,95
SLC39A1	10,01	10,16	9,13
SLC39A10	9,04	9,25	7,14
SLC39A11	10,17	11,31	9,01
SLC39A14	4,00	5,08	2,69
SLC39A3	7,64	7,68	6,86
SLC39A6	8,92	7,80	7,37

SLC39A7	7,68	7,95	7,42
SLC39A8	9,58	8,32	5,92
SLC39A9	6,76	6,39	5,77
SLC3A2	10,04	10,03	8,73
SLC40A1	3,65	3,27	6,91
SLC43A1	3,43	5,82	5,87
SLC43A3	7,68	8,76	8,04
SLC44A1	10,75	10,47	10,08
SLC45A3	6,20	9,07	3,09
SLC4A11	6,24	5,42	3,58
SLC4A1AP	7,63	7,75	7,54
SLC4A2	8,55	8,38	8,22
SLC4A7	7,00	6,99	6,24
SLC4A8	5,74	6,50	6,07
SLC5A2	3,73	3,21	4,30
SLC5A6	8,47	8,70	7,77
SLC5A8	7,01	6,75	8,80
SLC6A12	8,76	10,15	7,77
SLC6A13	3,78	3,95	4,55
SLC6A8	4,91	5,16	3,14
SLC6A9	5,45	5,27	4,40
SLC7A1	9,81	9,60	7,67
SLC7A10	4,34	4,55	5,02
SLC7A11	7,49	7,70	3,13
SLC7A4	3,84	3,73	4,02
SLC7A6	7,09	6,50	8,29
SLC7A6OS	7,19	7,06	7,04
SLC7A7	12,13	13,06	13,23
SLC7A8	8,77	7,85	2,96
SLC9A1	10,08	10,98	9,55
SLC9A5	5,74	4,97	4,69
SLC9A6	9,01	9,27	8,63
SLC9A7	6,51	6,82	3,53
SLC9A9	5,59	6,69	8,35
SLCO2A1	3,95	2,35	2,89
SLCO2B1	10,67	11,18	0,10
SLCO3A1	7,28	6,57	8,47
SLCO4A1	7,35	7,85	-0,16
SLCO4C1	7,88	7,86	6,20
SLCO5A1	5,54	1,59	-0,06
SLFN11	10,23	11,33	10,92
SLFN12	7,59	7,32	8,54
SLFN13	3,09	3,33	0,48
SLIC1	5,35	5,32	6,01
SLIT2	4,61	4,40	5,02
SLIT3	1,74	6,08	-1,26
SLITRK1	3,53	3,92	0,09
SLITRK2	4,31	3,65	3,32
SLITRK4	5,45	8,67	9,13
SLK	8,76	8,58	8,97
SLMAP	9,45	9,58	9,93
SLTM	8,52	8,51	9,58
SLU7	8,27	8,33	9,11
SMA4	7,31	7,83	7,42
SMA5	2,99	3,12	4,32
SMAD1	4,92	4,22	4,21
SMAD2	5,09	5,20	5,72
SMAD3	4,77	3,61	8,81
SMAD4	9,54	9,35	9,73

SMAD6	7,33	5,90	5,28
SMAD7	8,34	8,09	8,34
SMAP1	8,66	8,17	9,41
SMARCA2	9,92	9,87	10,11
SMARCA4	10,32	10,13	10,55
SMARCA5	8,69	8,47	8,31
SMARCAD1	7,49	7,83	7,52
SMARCAL1	7,78	7,97	8,09
SMARCC1	9,72	9,71	10,48
SMARCC2	7,63	7,11	7,84
SMARCD1	4,54	4,14	4,98
SMARCD2	8,00	7,52	8,08
SMARCE1	8,83	8,59	9,64
SMCP	4,46	4,61	4,54
SMEK2	10,03	9,85	9,78
SMG5	8,62	8,33	8,62
SMNDC1	9,73	9,52	10,12
SMOX	7,15	6,29	5,31
SMPD1	7,38	7,46	6,09
SMPD2	6,76	6,48	6,09
SMPDL3A	7,54	9,36	9,86
SMS	11,34	11,54	11,14
SMTN	3,89	3,21	4,37
SMU1	7,91	8,04	8,56
SMUG1	9,30	9,40	9,00
SMURF2	3,72	4,00	4,27
SMYD2	6,53	7,11	7,02
SNAG1	3,87	3,95	4,30
SNAI1	4,71	5,02	7,59
SNAP23	9,84	9,73	10,02
SNAP29	8,58	8,72	8,64
SNAPC1	3,45	1,64	3,48
SNAPC2	8,96	8,37	8,19
SNAPC3	8,35	8,33	9,00
SNAPC4	8,95	7,69	9,27
SNAPC5	7,30	7,48	6,50
SNCA	8,02	9,97	8,73
SNCB	3,57	3,87	3,57
SND1	11,64	11,64	11,40
SNF1LK	3,92	5,02	9,03
SNF1LK2	3,19	3,77	5,15
SNF8	9,86	10,16	10,03
SNFT	10,56	5,39	7,45
SNIP	4,24	3,32	3,43
SNIP1	8,30	8,21	8,86
SNPH	4,50	4,28	2,65
SNRK	8,72	8,72	10,24
SNRP70	9,82	9,82	10,51
SNRPA	8,38	8,39	9,08
SNRPA1	10,34	10,75	10,91
SNRPB	10,66	10,79	10,69
SNRPB2	10,72	10,84	10,97
SNRPC	8,95	9,15	8,64
SNRPD2	9,46	9,87	10,37
SNRPD3	7,66	7,39	7,90
SNRPF	10,24	10,54	10,88
SNRPG	12,58	12,41	12,12
SNRPN	8,43	8,29	7,22
SNTA1	5,91	4,91	5,76

SNTB1	7,52	10,75	9,58
SNTB2	6,33	5,81	5,75
SNURF	9,19	9,00	7,82
SNW1	9,34	9,32	9,71
SNX1	6,56	6,50	7,57
SNX11	8,73	8,63	8,63
SNX12	7,60	7,31	6,51
SNX13	8,74	8,61	8,28
SNX14	9,94	9,79	9,47
SNX15	8,10	7,96	8,87
SNX16	7,48	7,04	7,98
SNX17	10,58	10,61	11,09
SNX19	10,04	10,20	9,71
SNX24	6,19	6,74	3,73
SNX25	5,40	4,57	3,57
SNX27	10,86	11,17	11,70
SNX3	12,17	12,26	11,90
SNX5	9,48	9,34	8,69
SNX6	8,52	8,42	8,10
SOAT1	10,79	10,39	8,44
SOCS1	9,93	4,95	-3,32
SOCS2	6,39	5,82	1,80
SOCS4	7,25	7,22	7,07
SOCS7	3,24	2,59	2,96
SOD1	12,43	12,89	12,13
SOD2	9,58	10,63	11,00
SOD3	4,57	4,85	3,33
SOLH	5,37	4,57	6,85
SON	6,60	6,69	7,61
SORBS1	-0,32	5,12	-1,69
SORCS1	3,75	3,82	3,76
SORCS2	-3,32	-0,40	4,05
SORL1	5,11	7,11	10,39
SORT1	10,79	11,67	10,28
SOS1	5,34	5,58	5,60
SOS2	6,18	5,84	7,01
SOST	4,37	4,39	4,32
SOX11	3,21	3,31	3,13
SOX13	6,60	8,10	4,21
SOX18	4,42	4,47	4,26
SOX2	3,85	4,30	3,94
SOX30	5,96	5,96	5,54
SOX4	8,78	7,75	8,90
SOX5	3,26	3,57	4,03
SOX8	7,05	5,03	3,16
SOX9	-0,31	3,58	0,51
SP1	8,69	8,52	9,87
SP100	4,98	4,93	6,87
SP110	7,63	8,22	9,99
SP140	4,38	6,09	6,00
SP3	7,81	7,36	8,32
SP4	7,02	6,80	6,42
SPA17	7,05	8,15	7,17
SPAG16	5,40	5,36	2,59
SPAG5	8,77	7,31	5,20
SPAG7	8,47	8,59	8,67
SPAG9	8,10	8,09	7,27
SPARC	2,59	10,01	-3,32
SPAST	7,83	7,71	8,46

SPATA1	4,24	2,59	3,92
SPATA18	6,57	7,43	-2,26
SPATA2	4,55	4,90	5,76
SPATA20	9,11	9,17	8,38
SPATA3	3,25	2,87	3,23
SPATA5	2,82	4,11	2,53
SPATA5L1	7,94	7,60	8,08
SPATA6	-0,87	3,83	6,31
SPATS2	6,79	6,84	5,28
SPCS1	11,97	11,97	11,79
SPCS2	11,12	11,49	10,79
SPCS3	7,96	8,28	7,81
SPEN	10,03	9,59	10,82
SPG20	6,72	6,02	6,01
SPG21	8,16	8,58	7,94
SPG3A	6,56	7,73	-0,69
SPG7	8,05	7,99	8,90
SPHK2	9,25	9,17	9,36
SPI1	12,38	11,92	12,36
SPIN1	7,07	7,30	7,04
SPIN3	5,60	5,09	6,22
SPINT1	7,09	6,71	5,38
SPINT2	12,84	9,96	10,10
SPIRE2	3,70	4,26	2,15
SPN	9,10	8,57	8,03
SPO11	2,16	3,18	2,38
SPOCK2	3,81	4,37	4,42
SPON2	5,09	5,25	4,10
SPP1	13,97	14,42	4,00
SPPL2A	12,08	12,38	11,16
SPPL2B	3,51	2,95	5,21
SPR	8,79	9,00	6,43
SPRED1	11,68	11,60	8,69
SPRR1A	6,54	6,81	6,76
SPRY2	9,52	9,36	3,16
SPRY3	2,14	3,25	4,83
SPRYD3	9,51	9,45	9,08
SPRYD4	6,64	6,91	5,91
SPSB1	7,83	7,80	2,96
SPSB3	9,09	8,42	10,84
SPTAN1	11,76	11,32	9,17
SPTB	3,90	3,80	3,28
SPTY2D1	7,07	7,44	6,91
SQLE	7,87	8,34	4,36
SQRDL	11,48	11,96	10,66
SQSTM1	10,83	11,43	8,43
SRBD1	8,72	9,10	9,40
SRC	7,82	8,47	7,45
SRD5A1	6,62	6,48	7,79
SRD5A2L	6,70	6,82	5,38
SREBF2	10,12	9,80	8,62
SRF	10,35	9,63	10,17
SRFBP1	6,39	6,57	5,48
SRGAP1	8,05	5,73	4,34
SRI	6,50	6,46	6,42
SRM	7,98	8,04	7,43
SRP19	10,02	9,77	9,72
SRP54	9,78	10,15	9,39
SRP68	9,31	9,31	9,48

SRP72	9,27	9,41	9,49
SRP9	7,58	7,65	7,12
SRPK2	8,46	7,46	7,15
SRPR	8,52	8,81	8,79
SRPRB	10,27	10,48	9,75
SRPX	4,99	7,05	0,05
SRR	6,73	6,42	5,20
SRRM1	10,55	10,28	11,41
SRXN1	11,07	11,41	9,43
SRRP35	4,11	4,28	2,79
SS18	7,05	7,01	7,77
SS18L1	7,85	7,35	7,72
SS18L2	11,61	11,43	11,48
SSB	10,49	10,45	10,69
SSBP1	11,38	11,45	11,36
SSBP2	6,65	6,30	8,87
SSBP3	6,93	7,81	6,93
SSBP4	5,23	5,29	5,73
SSFA2	7,01	7,17	7,73
SSH1	7,16	6,93	7,28
SSH2	8,50	8,63	11,02
SSNA1	8,46	8,36	9,03
SSPN	3,37	5,98	-1,28
SSR1	11,11	11,36	11,76
SSR2	9,45	9,97	9,76
SSR3	7,32	7,07	6,87
SSR4	11,44	11,82	11,81
SSRP1	9,95	9,72	9,99
SSSCA1	6,59	6,71	6,81
SST	3,76	4,03	4,13
SSTR2	6,74	6,93	6,93
SSU72	11,36	11,55	11,22
ST13	10,89	10,74	9,56
ST14	8,70	8,21	6,39
ST3GAL1	8,58	8,59	8,21
ST3GAL2	6,87	6,29	6,81
ST3GAL3	5,11	5,30	5,11
ST3GAL4	8,41	8,30	7,51
ST3GAL5	9,75	10,02	9,34
ST3GAL6	8,69	9,66	8,27
ST5	5,35	5,75	3,61
ST6GAL1	10,16	8,66	9,24
ST6GALNAC2	7,35	7,75	7,47
ST6GALNAC4	8,51	9,38	7,56
ST6GALNAC6	8,60	9,18	7,24
ST7	9,19	8,54	5,91
ST7L	5,58	5,64	4,59
ST8SIA4	5,73	5,52	8,04
ST8SIA5	4,15	4,52	4,75
STAB2	4,76	4,79	4,29
STAC	6,97	8,26	4,31
STAC2	3,28	3,85	4,29
STAG1	7,30	7,37	7,26
STAG2	8,81	8,79	10,18
STAG3	8,64	6,77	3,24
STAM	8,39	8,70	7,71
STAM2	7,30	7,44	6,59
STAMBP	9,18	9,14	8,73
STAMBPL1	8,89	6,30	5,02

STAP2	3,72	3,44	3,39
STARD10	7,30	7,03	7,42
STARD13	4,38	4,45	3,94
STARD3	8,49	7,52	7,83
STARD3NL	9,46	10,00	10,10
STARD4	6,08	6,69	-0,07
STARD5	5,88	6,36	4,70
STARD8	8,63	9,09	7,59
STAT1	10,54	11,04	11,55
STAT2	10,99	11,30	12,15
STAT4	6,13	7,83	2,68
STAT5A	10,76	10,17	10,78
STAT5B	8,79	8,38	10,19
STAT6	9,94	9,40	10,46
STAU2	6,53	6,66	6,71
STCH	3,95	3,67	4,22
STEAP3	7,02	8,68	6,49
STEAP4	-3,32	-1,41	5,15
STIL	7,32	7,91	6,04
STIM1	8,39	8,70	9,35
STIM2	7,02	6,37	6,67
STIP1	11,18	11,08	9,25
STK11IP	7,06	6,96	7,76
STK16	6,49	6,19	6,45
STK17B	7,95	7,35	11,87
STK19	8,13	7,53	8,21
STK24	10,82	10,50	10,92
STK25	8,91	8,46	9,18
STK32B	6,78	6,52	6,52
STK32C	7,16	5,52	6,43
STK35	8,47	8,65	7,98
STK36	7,50	8,25	8,66
STK38	11,52	11,57	12,19
STK38L	5,14	4,54	6,54
STK39	8,96	8,71	8,54
STK4	12,14	11,66	12,32
STK40	4,27	4,72	5,02
STMN1	5,06	6,55	3,50
STMN2	4,44	4,22	3,43
STMN3	6,46	5,20	8,64
STOM	10,02	11,26	9,58
STOML1	5,41	5,31	5,04
STOML2	10,00	10,16	9,90
STOX2	3,80	6,58	-3,32
STRAP	11,05	11,11	11,19
STRBP	8,07	7,37	5,06
STRN	6,80	6,93	6,68
STRN3	8,47	9,03	8,92
STRN4	6,43	6,17	7,64
STS-1	9,93	10,47	8,71
STT3A	10,98	10,65	9,84
STT3B	10,57	10,91	10,44
STUB1	9,49	9,33	9,00
STX10	9,66	9,79	10,88
STX11	9,50	10,41	11,93
STX16	9,01	9,21	10,08
STX17	5,34	5,25	4,79
STX18	4,41	4,17	5,27
STX1A	4,04	4,41	4,00

STX7	9,39	9,36	9,00
STX8	9,44	9,68	9,85
STXBP5	7,72	8,02	8,31
STYX	4,11	4,54	4,58
STYXL1	7,99	8,21	7,88
SUCLG1	10,56	10,79	10,46
SUCLG2	10,05	9,76	10,04
SUCNR1	10,74	10,22	-3,32
SUDS3	6,64	6,40	6,69
SUFU	8,34	7,59	7,36
SUGT1	10,93	10,89	10,99
SULF2	11,09	7,05	11,04
SULT1A3	7,02	7,04	9,20
SULT1A4	4,22	4,20	5,21
SULT1B1	-2,55	2,00	7,08
SULT1C2	6,43	7,47	1,28
SULT6B1	3,65	4,04	3,68
SUMF1	10,07	10,59	9,83
SUMF2	8,87	8,77	8,90
SUMO2	12,28	12,01	12,59
SUOX	9,35	8,04	7,20
SUPT16H	9,78	9,60	10,07
SUPT3H	4,84	4,91	4,99
SUPT4H1	9,32	9,39	9,91
SUPT5H	9,12	8,72	9,04
SUPT6H	8,14	7,98	8,17
SUPT7L	3,46	1,48	4,06
SUPV3L1	7,16	7,38	7,21
SURF1	9,34	9,33	9,33
SURF2	5,81	5,51	5,97
SURF4	10,23	10,69	9,92
SURF5	9,12	8,61	8,93
SUSD4	3,09	2,44	2,84
SUV39H1	7,18	7,30	7,36
SUV39H2	4,35	3,80	3,94
SUV420H1	6,37	6,21	7,45
SV2C	2,76	4,03	3,53
SVIL	7,71	8,74	8,77
SWAP70	10,94	10,86	9,84
SYAP1	8,07	8,15	8,32
SYBL1	11,06	11,06	10,77
SYF2	10,07	9,89	10,93
SYK	10,03	9,77	10,68
SYMPK	6,86	6,20	6,67
SYN1	3,25	3,43	3,29
SYN2	3,97	-0,15	4,05
SYNCRIP	9,94	10,11	10,28
SYNGR1	4,74	7,46	6,23
SYNJ1	8,88	8,06	8,34
SYNJ2	8,42	7,11	6,48
SYNJ2BP	8,65	9,20	9,02
SYNPO2	3,68	3,53	3,29
SYP	3,85	6,43	3,05
SYPL1	8,56	9,27	9,14
SYT11	6,30	7,38	8,20
SYT15	5,00	5,34	6,03
SYT17	8,74	4,18	3,69
SYT3	3,95	4,52	4,71
SYT6	5,17	3,37	3,72

SYT8	6,07	6,21	5,90
SYTL3	5,82	8,32	8,38
SYVN1	10,20	10,03	10,02
T	3,70	4,11	3,44
TA-NFKBH	6,30	6,12	6,86
TAAR6	3,56	4,08	4,13
TACC1	12,03	12,01	12,32
TACC3	7,80	7,82	9,82
TADA1L	5,00	4,30	5,37
TADA2L	5,58	5,27	5,83
TADA3L	8,10	7,93	8,09
TAF1	3,43	1,71	5,15
TAF10	10,41	10,44	10,87
TAF12	7,79	7,76	7,80
TAF13	6,84	7,12	6,07
TAF15	10,91	10,92	11,90
TAF2	8,09	8,01	8,31
TAF4	8,49	7,96	9,36
TAF5L	7,70	7,66	7,69
TAF6	5,18	4,81	5,22
TAF6L	7,93	7,65	8,41
TAF7	9,00	8,88	8,61
TAF9	8,57	8,57	8,08
TAGAP	5,81	6,50	7,95
TAGLN2	9,11	10,00	10,70
TAGLN3	-1,54	4,36	-2,98
TAL1	5,87	3,65	3,58
TANC1	4,74	4,66	4,80
TANK	9,69	9,66	9,45
TAOK2	6,41	6,06	6,13
TAP1	11,13	11,44	11,52
TAP2	6,92	7,57	8,21
TAPBP	10,79	10,55	10,26
TAPBPL	7,55	8,00	7,91
TARBP2	6,83	7,05	6,73
TARDBP	6,64	6,15	8,32
TARP	1,91	4,17	3,94
TARS	10,34	10,25	9,37
TASP1	5,51	6,02	5,26
TATDN1	8,33	8,82	8,54
TATDN2	8,05	8,23	8,70
TATDN3	7,65	7,63	7,74
TAX1BP1	12,00	11,84	11,76
TAX1BP3	10,78	10,21	9,19
TAZ	6,59	6,36	7,03
TBC1D10B	8,00	7,21	8,17
TBC1D13	8,39	7,11	7,29
TBC1D14	9,63	9,57	10,38
TBC1D15	9,51	9,65	9,57
TBC1D16	6,39	6,80	4,67
TBC1D17	5,29	4,99	5,06
TBC1D19	4,89	5,09	4,00
TBC1D2	10,65	11,60	10,02
TBC1D20	6,46	6,77	6,82
TBC1D22A	9,95	9,22	9,13
TBC1D22B	9,00	9,11	8,52
TBC1D23	7,53	7,65	6,80
TBC1D2B	7,48	7,63	6,66
TBC1D4	7,62	7,20	6,58

TBC1D5	5,40	5,47	5,48
TBC1D7	9,12	9,56	7,88
TBC1D9	9,05	10,22	10,30
TBCA	12,40	12,69	12,20
TBCC	8,05	7,36	7,79
TBCCD1	6,01	5,59	5,50
TBCE	9,12	9,46	9,20
TBK1	9,92	9,90	10,20
TBKBP1	5,47	5,10	5,37
TBL1X	8,58	8,02	10,22
TBL1XR1	8,01	7,94	7,75
TBL2	7,51	8,13	7,54
TBL3	5,57	5,47	6,17
TBN	4,69	4,33	4,76
TBP	7,88	7,43	8,08
TBPL1	8,40	8,41	9,28
TBRG1	4,84	5,18	5,45
TBRG4	8,01	7,76	7,68
TBX22	4,29	4,83	4,10
TBX3	2,26	2,36	3,79
TBX4	4,08	3,80	3,92
TBX5	3,63	0,02	2,95
TCEA1	6,73	6,20	6,59
TCEA2	5,15	4,84	5,75
TCEA3	3,18	4,22	6,04
TCEAL1	6,84	7,03	5,82
TCEAL3	8,56	8,86	7,29
TCEAL4	9,96	9,94	8,78
TCEAL8	9,45	9,60	8,35
TCEB1	9,62	10,01	8,88
TCEB2	12,49	13,00	12,19
TCERG1	7,96	7,97	8,82
TCF12	7,67	7,41	7,18
TCF19	2,03	5,01	3,13
TCF3	6,32	5,10	6,97
TCF4	8,69	6,93	7,60
TCF7	3,28	3,00	3,38
TCF7L2	2,81	7,19	8,07
TCHP	8,10	8,18	9,54
TCIRG1	9,72	10,26	9,78
TCP1	9,99	9,86	10,02
TCP11L2	3,91	3,99	6,25
TCTA	7,63	7,26	7,37
TCTEX1D1	5,72	5,50	5,92
TDG	10,49	10,14	9,92
TDP1	5,82	5,73	6,30
TDRD7	7,98	7,66	8,06
TDRKH	4,14	3,73	3,37
TEAD2	2,21	4,44	-0,56
TEAD3	4,05	5,33	3,70
TEF	6,74	6,50	5,59
TEGT	13,38	13,33	13,02
TEP1	3,81	3,56	4,58
TERF1	6,76	6,68	6,30
TERF2IP	10,27	10,28	10,42
TES	7,27	6,64	9,14
TESC	4,69	6,77	10,35
TESK1	8,36	8,43	7,23
TESK2	6,09	7,14	7,59

TETRAN	11,36	11,38	11,56
TEX10	7,96	7,70	7,22
TEX2	9,70	10,75	8,64
TEX261	10,13	9,85	9,50
TEX264	8,99	9,16	8,42
TF	3,41	3,82	3,11
TFAM	8,43	8,80	8,65
TFB2M	7,69	8,04	7,46
TFCP2	8,95	8,84	8,90
TFCP2L1	4,23	4,93	2,17
TFEC	6,03	5,61	4,98
TFG	9,10	9,10	8,45
TFIP11	6,88	6,94	7,19
TFPI	6,92	8,01	-0,76
TFPT	9,19	9,29	7,53
TFRC	14,55	14,66	10,88
TGDS	8,63	8,21	7,77
TGFA	8,90	6,58	5,50
TGFBR1	5,83	6,94	5,87
TGFBR2	10,27	10,31	10,22
TGFBR3	3,79	4,35	0,27
TGFBRAP1	7,19	7,29	7,70
TGIF2	7,73	8,23	8,22
TGM2	12,10	10,73	4,04
TGM6	4,90	5,27	5,66
TGOLN2	11,37	11,39	11,36
THADA	6,10	6,98	6,77
THAP1	7,18	6,98	6,93
THAP10	2,30	4,54	0,19
THAP11	10,11	9,80	11,08
THAP5	2,52	3,16	4,09
THAP6	5,30	4,69	2,94
THAP7	4,61	4,19	4,30
THAP8	3,25	4,16	0,19
THBD	5,50	1,83	5,29
THBS1	3,36	0,66	7,36
THBS3	8,09	6,49	6,84
THEM2	10,71	11,16	8,22
THEM4	2,30	4,71	-3,32
THEX1	9,04	6,30	5,50
THNSL1	5,35	5,15	3,16
THOC1	9,15	9,23	8,81
THOC4	8,74	8,61	8,89
THOC5	6,29	7,16	6,85
THOC6	7,68	7,01	8,19
THOC7	11,40	11,47	11,43
THRA	4,45	5,00	5,19
THRAP3	7,08	6,99	7,38
THRAP4	7,40	7,59	7,52
THRB	3,49	4,19	3,71
THUMPD2	7,27	7,61	7,92
THYN1	9,55	9,66	10,07
TIA1	8,07	8,09	8,70
TIAL1	8,17	8,19	8,75
TIAM1	8,32	7,69	7,69
TICAM2	8,34	8,20	9,51
TIGD1	5,25	4,82	5,91
TIGD2	5,50	5,82	5,26
TIGD3	4,10	4,57	4,53

TIGD4	4,42	3,57	4,37
TIGD5	7,42	6,84	7,46
TIGD6	4,04	4,06	0,17
TIGD7	5,88	5,73	5,71
TIMELESS	7,96	8,28	7,06
TIMM10	9,49	10,07	7,96
TIMM17A	5,39	5,35	2,98
TIMM17B	7,72	7,63	8,38
TIMM22	7,71	7,52	8,10
TIMM23	10,29	10,48	9,84
TIMM44	6,73	6,39	6,82
TIMM8A	6,61	6,78	5,61
TIMM8B	8,43	8,35	7,78
TIMM9	8,38	8,40	9,01
TIMP1	11,66	12,76	12,38
TIMP3	5,30	4,73	4,73
TIMP4	3,29	5,23	2,62
TINF2	9,69	10,09	10,14
TINP1	10,68	10,84	11,53
TIPARP	9,12	9,41	8,91
TIPRL	7,73	8,47	8,04
TIRAP	5,78	5,62	5,59
TJAP1	9,52	9,25	10,00
TJP1	5,47	4,92	4,43
TJP2	6,40	5,33	5,74
TK1	4,51	8,03	4,30
TK2	7,33	7,00	7,43
TKT	12,70	12,53	13,46
TLCD1	5,20	5,01	3,44
TLE1	8,96	7,32	4,86
TLE2	2,03	4,23	0,12
TLE3	6,29	7,41	7,85
TLE4	6,30	5,97	7,77
TLE6	4,37	4,94	-0,69
TLK1	9,17	8,73	8,38
TLK2	7,30	7,29	7,84
TLN1	9,61	9,52	9,98
TLOC1	6,86	7,15	7,69
TLR1	8,32	9,37	9,54
TLR2	2,16	3,31	7,09
TLR3	5,95	4,34	4,05
TLR4	8,40	8,82	8,67
TLR5	7,95	8,30	10,85
TLR7	4,28	7,76	8,88
TLR8	7,54	8,90	10,49
TM2D1	9,47	9,94	8,97
TM2D2	7,89	7,06	4,92
TM2D3	9,67	9,74	9,11
TM4SF1	5,64	9,27	-3,32
TM4SF19	9,25	11,69	2,43
TM6SF1	10,44	10,36	9,27
TM7SF3	8,67	8,91	8,51
TM7SF4	11,89	13,13	-3,32
TM9SF1	9,28	9,40	8,64
TM9SF2	11,95	12,02	11,84
TM9SF3	10,31	10,10	9,57
TM9SF4	9,58	9,66	10,96
TMBIM4	12,59	12,69	12,63
TMC6	10,42	10,66	9,66

TMC8	5,17	5,75	5,85
TMCC2	4,75	0,30	-0,71
TMCC3	5,23	6,26	6,37
TMCO1	11,09	11,30	11,08
TMCO3	11,68	11,83	10,13
TMCO4	6,32	6,01	6,13
TMED1	9,08	9,30	9,26
TMED10	8,72	8,97	8,69
TMED3	11,75	12,61	10,72
TMED4	8,57	8,84	8,26
TMED5	11,80	11,60	11,30
TMED7	9,95	10,25	10,29
TMED8	3,84	4,26	4,07
TMED9	11,31	11,54	10,66
TMEFF1	4,69	5,01	-2,00
TMEFF2	3,46	3,72	3,84
TMEM1	7,24	6,68	6,90
TMEM101	9,15	9,35	8,94
TMEM104	6,50	7,59	5,36
TMEM106B	5,02	5,29	4,68
TMEM106C	8,04	9,37	7,11
TMEM107	4,15	5,39	4,56
TMEM109	10,47	10,68	10,65
TMEM11	7,05	7,22	7,72
TMEM111	12,40	11,92	10,96
TMEM116	6,68	6,90	6,58
TMEM117	8,67	8,13	-3,32
TMEM118	5,59	6,78	-3,32
TMEM123	12,10	11,16	11,91
TMEM126A	10,86	11,04	9,90
TMEM126B	10,15	10,56	9,64
TMEM127	8,68	8,26	8,08
TMEM128	5,06	5,84	4,10
TMEM129	5,72	5,73	5,75
TMEM135	8,65	8,54	6,72
TMEM138	9,76	10,68	8,35
TMEM140	8,97	9,59	5,44
TMEM141	8,95	9,35	8,74
TMEM142A	8,72	8,35	8,50
TMEM143	5,69	5,82	5,02
TMEM144	10,08	9,37	8,57
TMEM145	4,15	3,75	4,16
TMEM147	11,02	11,46	10,63
TMEM149	10,83	9,39	9,94
TMEM14A	6,05	5,91	-3,32
TMEM14B	11,08	11,56	11,24
TMEM14C	11,52	11,60	10,62
TMEM16B	3,50	4,41	4,51
TMEM16F	9,91	9,95	10,28
TMEM16H	5,33	4,82	6,14
TMEM16K	7,09	8,21	7,06
TMEM18	5,44	5,10	6,87
TMEM19	7,33	7,15	6,28
TMEM2	7,51	8,71	8,76
TMEM20	5,11	5,93	3,27
TMEM24	6,25	6,16	5,96
TMEM26	7,70	5,79	5,04
TMEM28	4,20	2,89	4,39
TMEM29	7,37	7,81	8,60

TMEM30A	9,32	9,36	9,89
TMEM33	4,51	4,42	3,65
TMEM34	3,51	3,77	-0,04
TMEM37	4,31	5,43	-0,44
TMEM38A	3,72	4,26	5,69
TMEM38B	9,37	10,75	7,01
TMEM39A	7,54	7,42	7,16
TMEM4	9,48	10,57	10,08
TMEM41A	6,94	6,76	6,37
TMEM41B	10,49	10,34	9,13
TMEM42	7,99	8,24	8,09
TMEM43	10,73	10,68	11,00
TMEM44	6,51	6,53	-0,74
TMEM45A	2,85	4,92	3,36
TMEM45B	8,12	6,65	2,18
TMEM48	6,68	7,60	6,22
TMEM49	9,02	8,79	9,84
TMEM5	8,90	9,61	8,73
TMEM50A	7,88	8,37	8,69
TMEM50B	4,26	4,14	6,16
TMEM51	11,26	10,68	8,04
TMEM53	5,98	6,44	3,30
TMEM54	4,10	4,76	4,07
TMEM55A	7,87	7,10	8,67
TMEM55B	8,96	8,47	8,36
TMEM57	6,89	6,64	5,82
TMEM58	6,39	6,82	6,70
TMEM59	12,41	12,48	12,08
TMEM60	11,00	10,81	9,43
TMEM61	4,56	3,43	4,40
TMEM62	9,14	9,44	9,06
TMEM63B	7,05	6,79	6,42
TMEM63C	3,53	3,94	4,54
TMEM65	5,93	6,20	6,59
TMEM66	13,50	13,32	13,49
TMEM68	5,05	4,74	4,13
TMEM69	8,03	8,32	8,09
TMEM70	7,21	7,33	7,51
TMEM71	8,85	7,87	11,02
TMEM77	9,20	9,26	9,32
TMEM79	4,82	4,65	5,02
TMEM8	9,65	9,49	9,19
TMEM80	5,22	5,29	6,92
TMEM85	10,56	10,89	10,53
TMEM86A	7,01	6,47	5,23
TMEM87A	11,14	11,19	11,21
TMEM87B	6,03	6,52	5,86
TMEM88	4,70	5,09	6,32
TMEM93	9,29	9,31	9,27
TMEM97	9,57	9,38	8,74
TMEM98	2,77	3,50	3,00
TMEM99	7,03	6,93	5,85
TMEM9B	11,50	11,38	11,34
TMEPAI	2,83	4,08	3,16
TMF1	6,59	6,63	6,69
TMLHE	7,31	7,10	7,27
TMOD4	3,34	2,68	4,64
TMPO	5,11	5,98	6,49
TMSB4X	14,85	14,92	14,92

TMTC1	5,67	4,79	5,35
TMTC2	4,19	2,94	6,93
TNC	5,02	0,77	-3,32
TNF	7,23	9,10	7,90
TNFAIP1	7,90	7,64	6,68
TNFAIP2	8,56	9,19	10,88
TNFAIP3	9,12	10,35	10,66
TNFAIP6	5,89	8,44	6,20
TNFAIP8L1	7,93	8,72	7,85
TNFAIP8L2	7,09	7,12	7,61
TNFAIP8L3	7,21	7,49	-3,32
TNFRSF10A	7,51	7,30	7,04
TNFRSF10C	4,72	4,17	4,57
TNFRSF10D	5,20	5,77	6,19
TNFRSF11A	7,16	-3,32	-1,05
TNFRSF11B	5,81	4,60	4,39
TNFRSF12A	8,02	8,39	5,57
TNFRSF13C	4,64	4,49	4,43
TNFRSF14	12,80	12,44	12,27
TNFRSF18	-1,23	3,02	-3,32
TNFRSF1A	9,91	9,85	10,84
TNFRSF21	8,98	10,85	6,05
TNFRSF25	5,51	3,70	5,28
TNFRSF4	4,43	3,81	3,78
TNFRSF9	4,32	3,75	5,75
TNFSF10	3,52	6,90	10,94
TNFSF12	8,99	9,21	9,22
TNFSF12-TNFSF13	6,03	6,41	6,27
TNFSF13	8,25	9,37	9,24
TNFSF13B	11,23	10,58	11,60
TNFSF4	4,00	3,98	2,69
TNFSF5IP1	11,43	11,47	11,95
TNFSF8	6,59	7,46	8,43
TNIK	6,03	7,95	4,14
TNIP1	10,33	9,09	10,30
TNKS	4,70	3,73	4,09
TNKS2	3,88	4,84	4,12
TNNI3K	3,82	3,70	3,17
TNNT1	2,69	7,10	8,73
TNP2	4,37	4,15	3,78
TNPO1	8,96	9,00	7,89
TNPO2	9,28	8,80	8,70
TNPO3	9,16	9,07	9,25
TNRC5	8,32	8,76	10,17
TNRC6A	6,28	5,70	6,48
TNRC6B	7,62	7,46	8,45
TNS1	6,49	7,29	4,66
TNS3	11,81	12,81	10,83
TNS4	5,15	5,71	5,34
TOB1	9,51	9,99	9,97
TOB2	5,76	4,83	6,73
TOE1	5,66	5,80	6,36
TOLLIP	8,03	8,04	7,03
TOM1	5,75	5,58	6,30
TOM1L2	5,10	4,91	4,70
TOMM20	11,81	11,75	12,02
TOMM22	9,54	9,63	9,99
TOMM34	9,43	8,80	8,32
TOMM40	11,06	11,89	9,88

TOMM7	13,15	13,25	13,52
TOMM70A	10,22	10,17	9,36
TOP1	7,19	6,76	6,65
TOP2A	-3,32	8,03	-3,32
TOP2B	10,30	10,26	11,08
TOP3A	4,92	4,25	6,00
TOP3B	7,71	7,59	8,03
TOPBP1	9,75	9,76	9,82
TOPORS	7,50	7,40	7,71
TOR1AIP1	9,85	9,94	10,33
TOR1AIP2	6,24	6,25	4,44
TOR3A	9,89	9,73	9,12
TOX	4,84	3,89	4,00
TP53	5,97	5,41	6,32
TP53AP1	6,01	6,58	5,89
TP53BP1	6,83	6,53	5,45
TP53BP2	9,95	8,87	8,92
TP53INP1	9,36	9,11	10,36
TP53INP2	8,39	8,40	5,81
TP53RK	7,90	7,75	7,17
TPCN1	6,95	6,76	6,83
TPD52L1	2,17	4,43	2,78
TPD52L2	11,80	11,57	12,02
TPM3	9,02	8,95	8,85
TPM4	8,91	8,42	7,37
TPMT	7,15	6,91	6,38
TPO	3,60	3,46	-0,04
TPP1	12,48	12,78	12,25
TPP2	7,70	6,94	8,01
TPRKB	8,00	8,08	7,94
TPST1	2,39	4,66	6,23
TPST2	9,96	10,11	9,23
TPT1	14,44	14,57	14,66
TPX2	3,73	6,45	2,22
TRA16	6,84	6,91	6,46
TRA2A	7,90	7,74	9,04
TRADD	6,45	6,26	6,54
TRAF1	7,56	5,74	5,22
TRAF3IP2	4,63	7,40	5,74
TRAF3IP3	6,09	5,98	8,77
TRAF4	4,21	3,78	2,10
TRAF5	6,81	4,50	4,41
TRAF6	5,61	4,63	5,86
TRAIP	4,44	4,87	4,37
TRAK1	8,88	8,66	8,39
TRAK2	9,13	9,34	8,71
TRAM1	12,95	12,97	12,84
TRAM2	7,63	7,77	8,52
TRAP1	8,64	8,40	7,23
TRAPPC1	9,70	9,87	10,15
TRAPPC2	7,00	7,00	7,49
TRAPPC3	10,02	10,13	10,02
TRAPPC4	10,10	9,99	9,03
TRAPPC6A	8,43	8,24	8,02
TRAPPC6B	8,30	8,52	8,38
TREM1	3,91	7,64	8,65
TREM2	11,43	10,89	-0,48
TREX1	3,34	4,25	3,56
TRIAP1	9,36	9,16	8,72

TRIB1	10,40	10,87	10,30
TRIB2	8,32	5,52	5,41
TRIM11	7,25	6,87	7,60
TRIM14	3,53	3,20	3,84
TRIM17	3,28	1,97	0,48
TRIM21	7,65	8,39	8,19
TRIM22	8,42	9,26	10,71
TRIM23	5,79	6,01	5,99
TRIM24	8,35	8,32	7,96
TRIM25	5,47	5,61	5,83
TRIM26	8,36	8,04	8,65
TRIM28	8,33	7,78	8,13
TRIM29	3,95	3,73	3,15
TRIM3	5,12	4,81	5,62
TRIM32	6,73	6,77	4,58
TRIM34	2,45	2,83	3,61
TRIM35	4,63	4,93	2,09
TRIM36	5,72	4,15	3,78
TRIM38	8,53	8,49	9,79
TRIM39	7,10	6,91	6,65
TRIM4	6,12	5,83	6,28
TRIM41	7,71	7,58	8,13
TRIM44	8,71	9,01	10,19
TRIM45	2,71	2,27	3,99
TRIM46	4,17	2,20	2,84
TRIM47	7,12	7,06	2,33
TRIM5	7,11	6,83	7,78
TRIM54	3,30	5,47	3,92
TRIM56	7,23	6,86	7,68
TRIM58	4,30	4,66	4,88
TRIM6	2,91	4,93	1,45
TRIM61	4,16	4,80	5,56
TRIM62	2,46	2,59	4,86
TRIM67	2,81	4,23	3,18
TRIM68	7,90	8,26	7,05
TRIM7	3,62	3,54	5,49
TRIM73	4,23	4,43	4,52
TRIM8	10,38	10,20	11,50
TRIM9	4,81	5,21	4,54
TRIO	6,76	6,38	5,95
TRIOBP	7,68	7,86	8,61
TRIP10	7,05	5,91	3,72
TRIP11	8,91	8,83	8,22
TRIP12	10,62	10,46	10,71
TRIP13	3,07	6,44	-1,85
TRIP4	8,82	8,82	8,72
TRIP6	9,10	9,23	6,69
TRIT1	8,62	8,13	9,79
TRMT1	8,56	9,57	9,78
TRMT12	8,60	8,69	8,29
TRMU	6,06	5,72	5,20
TRNT1	6,28	6,18	5,40
TROAP	-0,22	5,16	-0,29
TROVE2	7,57	6,81	7,24
TRPC4	4,40	4,58	4,55
TRPC4AP	8,88	8,86	9,19
TRPC5	-0,44	3,99	3,95
TRPM3	2,45	3,22	3,47
TRPM4	7,44	7,34	7,72

TRPM6	4,51	3,82	4,85
TRPM7	5,95	6,03	5,30
TRPS1	6,51	6,51	8,17
TRPT1	6,19	5,91	6,65
TRPV2	11,13	10,98	8,23
TRPV4	7,76	9,80	7,13
TRRAP	10,20	9,72	10,03
TRSPAP1	5,43	5,30	5,36
TRUB1	4,56	5,02	-0,10
TRUB2	9,86	9,88	9,50
TSC1	6,00	6,20	6,37
TSC22D1	6,24	10,23	6,10
TSC22D2	7,78	7,70	8,42
TSC22D3	7,23	7,69	11,48
TSEN2	7,44	7,47	6,60
TSEN34	8,80	8,54	10,43
TSEN54	4,25	4,62	5,19
TSFM	8,99	9,59	8,94
TSG101	10,61	10,70	10,53
TSGA14	5,37	5,48	4,86
TSKS	1,86	3,76	4,28
TSLP	4,09	4,50	3,88
TSN	6,66	6,45	7,69
TSNAX	8,98	9,51	9,30
TSP50	4,54	4,18	4,76
TSPAN1	4,40	4,56	4,58
TSPAN12	3,48	3,14	0,57
TSPAN13	2,78	5,04	3,09
TSPAN14	9,32	10,12	9,41
TSPAN15	5,36	3,72	0,11
TSPAN16	4,77	2,05	1,54
TSPAN18	2,19	2,12	1,67
TSPAN2	-0,74	2,69	4,35
TSPAN31	8,63	9,15	8,74
TSPAN32	8,68	9,10	10,19
TSPAN33	10,46	10,17	9,09
TSPAN4	9,43	9,12	8,45
TSPAN5	5,12	5,37	4,83
TSPAN7	5,75	-3,32	-3,32
TSPAN9	4,54	4,36	5,09
TSPYL1	10,11	9,84	9,89
TSPYL5	6,10	5,91	0,30
TSR1	6,82	6,46	6,55
TSSC1	8,34	8,78	8,18
TSSC4	8,06	7,75	8,77
TSSK6	4,05	2,45	3,98
TST	10,47	10,94	10,76
TSTA3	6,46	6,32	6,47
TTBK2	3,41	3,85	4,86
TTC1	7,97	8,31	7,69
TTC13	7,98	7,14	8,33
TTC14	8,22	7,99	9,45
TTC15	9,41	9,20	9,68
TTC16	4,69	4,81	4,44
TTC17	8,63	8,23	9,44
TTC19	8,39	8,15	9,07
TTC21A	4,94	4,26	6,62
TTC23	7,96	8,33	3,25
TTC25	1,81	3,14	4,14

TTC26	4,93	4,71	3,85
TTC3	10,77	10,88	10,33
TTC4	9,77	10,03	9,53
TTC5	8,51	8,59	8,63
TTC7A	6,87	6,29	6,51
TTC8	7,08	7,21	7,30
TTC9C	9,47	7,05	6,45
TTF1	6,91	7,28	7,67
TTF2	7,89	8,09	7,74
TTK	3,06	6,85	-3,32
TTL	10,79	10,42	9,51
TTLL1	5,95	5,58	5,73
TTLL5	6,73	6,91	6,96
TTRAP	7,56	8,21	6,43
TTYH2	6,34	4,51	6,44
TUB	3,14	2,73	2,54
TUBB	10,01	10,49	8,55
TUBB2A	7,55	9,16	5,14
TUBB2B	7,13	0,28	-3,32
TUBB3	5,91	6,30	-3,32
TUBB4	-3,32	6,07	-0,49
TUBB6	10,60	10,24	6,47
TUBD1	6,92	6,09	6,54
TUBE1	6,39	5,89	4,63
TUBG1	7,45	7,91	5,24
TUBG2	6,52	6,48	5,90
TUBGCP2	8,86	8,72	9,41
TUBGCP3	7,76	7,68	7,91
TUBGCP5	6,69	7,00	5,62
TUBGCP6	6,21	5,88	7,36
TUFM	10,43	10,25	10,43
TUFT1	6,30	6,59	6,36
TULP2	6,81	6,91	6,94
TULP3	5,28	4,98	5,38
TUSC1	5,79	5,43	3,75
TUSC2	7,02	7,09	7,30
TUSC4	9,70	9,33	9,93
TWISTNB	6,54	6,93	6,58
TWSG1	6,98	7,60	5,46
TXK	4,36	4,56	4,04
TXLNA	11,18	10,60	10,40
TXN	14,44	14,35	12,14
TXN2	8,15	8,02	7,62
TXNDC10	7,24	7,46	8,03
TXNDC11	8,17	8,70	8,66
TXNDC12	12,01	12,07	11,65
TXNDC13	8,05	8,35	8,20
TXNDC14	10,69	10,81	9,88
TXNDC4	5,62	5,58	6,67
TXNDC5	8,69	9,40	8,46
TXNDC9	9,76	9,65	8,86
TXNIP	12,22	11,98	14,40
TXNL1	9,74	9,62	9,08
TXNL4B	3,54	4,29	4,16
TXNL5	12,55	12,50	11,60
TXNL6	3,44	-0,44	5,08
TXNRD1	10,98	11,49	8,60
TXNRD2	6,56	6,59	7,21
TYK2	10,88	10,20	11,61

TYMS	-2,19	8,48	-3,32
TYRO3	3,63	4,57	-1,22
TYROBP	13,80	14,18	14,04
TYSND1	8,89	8,92	8,61
U1SNRNPBP	5,53	5,40	5,14
U2AF1	8,64	8,57	9,64
U2AF1L4	8,13	6,68	7,63
U2AF2	10,33	10,20	10,93
UBAP1	8,82	9,48	9,55
UBAP2	9,06	9,68	9,14
UBAP2L	10,32	8,90	9,17
UBB	13,87	13,76	12,61
UBC	14,26	14,29	14,25
UBD	7,32	1,83	3,30
UBE1	9,35	9,16	9,67
UBE1DC1	5,52	5,66	5,65
UBE1L	9,15	9,52	10,14
UBE1L2	9,10	9,45	9,31
UBE2A	3,67	3,50	7,16
UBE2B	5,21	5,08	7,07
UBE2C	2,74	6,60	2,58
UBE2D2	6,05	6,33	6,91
UBE2D3	9,71	9,87	10,74
UBE2D4	9,39	9,42	7,66
UBE2E2	11,20	10,96	10,49
UBE2E3	5,36	5,49	6,22
UBE2G2	8,84	8,39	8,85
UBE2J1	9,99	10,13	9,87
UBE2L6	10,64	10,88	11,05
UBE2M	11,33	11,33	11,02
UBE2N	9,75	9,72	10,10
UBE2Q1	7,16	7,05	7,07
UBE2Q2	10,10	9,67	9,64
UBE2R2	6,03	5,96	7,52
UBE2T	5,71	6,69	4,03
UBE2V1	5,55	5,68	5,22
UBE2W	8,15	8,17	7,58
UBE2Z	10,91	9,76	9,32
UBE3A	9,23	9,02	9,47
UBE3B	8,24	8,25	8,31
UBE3C	8,56	8,50	8,47
UBE4A	9,10	9,47	9,72
UBE4B	9,38	9,59	9,86
UBIAD1	8,31	8,02	8,34
UBL3	11,17	10,20	10,40
UBL4A	9,06	9,09	8,18
UBL5	11,69	11,84	11,27
UBL7	7,35	7,22	7,52
UBLCP1	9,67	10,08	9,63
UBN1	9,99	9,42	9,61
UBP1	10,16	9,87	10,57
UBQLN1	10,04	10,05	10,13
UBQLN2	10,96	10,79	11,27
UBQLN4	9,66	9,81	9,30
UBR1	5,61	6,10	5,98
UBR2	9,21	9,23	9,42
UBTD1	9,22	9,86	8,24
UBTF	6,53	5,85	5,93
UBXD4	7,01	7,40	7,61

UBXD5	4,26	4,64	5,76
UBXD6	5,75	6,53	6,09
UBXD8	10,78	10,81	10,31
UCHL1	9,40	10,77	-0,37
UCHL3	9,00	10,32	9,51
UCHL5	8,83	8,63	7,60
UCK2	7,74	8,22	7,69
UCN	5,38	5,52	5,70
UCP1	4,69	4,76	5,44
UCRC	8,44	8,43	6,86
UFC1	9,86	10,03	9,81
UFD1L	5,43	5,81	4,80
UFM1	9,67	10,00	9,84
UGCG	10,40	10,62	7,49
UGCGL1	6,21	6,36	6,92
UGCGL2	6,93	7,42	6,49
UGDH	9,71	10,03	8,47
UGP2	10,88	10,24	8,62
UHMK1	6,97	6,86	6,90
UHRF1	3,42	7,91	4,63
ULBP3	3,64	3,75	3,98
ULK3	4,88	5,17	5,22
UMOD	3,73	3,77	4,42
UNC13B	6,76	6,62	3,87
UNC45A	7,48	7,52	7,39
UNC50	11,07	11,16	10,78
UNC84A	7,89	7,61	9,25
UNC84B	8,96	9,76	9,86
UNC93A	3,05	4,07	2,89
UNQ473	4,47	4,34	4,43
UNQ501	10,13	11,14	10,96
UNQ5830	3,54	3,63	3,87
UNQ846	6,72	6,88	6,76
UNQ9217	4,46	5,36	5,18
UPB1	4,35	3,22	4,22
UPF2	7,88	7,75	8,71
UPF3A	8,34	8,44	8,56
UPF3B	8,65	8,66	8,51
UPK3A	-0,23	1,70	6,04
UQCR	6,25	6,03	5,42
UQCRB	10,25	10,78	9,63
UQCRC2	11,32	11,28	10,87
UQCRFS1	12,01	12,02	11,87
UQCRH	11,35	11,13	11,06
UQCRQ	12,65	12,74	11,90
URG4	7,85	7,38	6,62
UROD	11,21	11,60	10,59
UROS	10,59	10,64	9,52
URP2	10,61	10,62	10,68
USF1	9,05	8,83	9,85
USH1G	5,07	4,18	5,26
USHBP1	5,13	5,29	5,36
USMG5	5,46	5,40	5,30
USP1	7,43	7,24	7,74
USP10	7,99	8,03	8,66
USP11	5,82	6,36	5,64
USP12	7,00	7,38	6,19
USP13	6,03	7,06	6,29
USP15	7,29	7,25	9,04

USP16	7,94	7,83	8,06
USP19	4,09	3,22	4,88
USP2	4,40	5,42	4,46
USP20	3,65	3,36	3,56
USP21	5,23	4,86	6,07
USP25	4,97	5,27	6,58
USP3	10,26	10,20	12,26
USP30	6,29	6,99	5,59
USP32	4,93	5,04	6,69
USP33	8,13	8,34	9,01
USP34	7,81	7,70	8,68
USP35	4,81	4,32	5,27
USP36	7,25	6,37	8,02
USP37	6,96	6,41	5,96
USP38	9,92	9,87	9,06
USP4	8,01	7,66	8,34
USP46	5,80	5,74	4,94
USP47	5,76	6,15	6,84
USP48	6,86	6,77	7,74
USP49	11,47	11,33	12,79
USP5	10,04	10,02	9,83
USP51	5,52	5,97	6,23
USP52	7,26	7,17	8,69
USP6	6,74	6,64	7,87
USP8	9,66	8,98	9,18
USP9X	9,35	9,40	9,51
USPL1	8,55	8,55	9,39
UST	3,14	3,85	4,03
UTF1	-3,32	-0,94	4,44
UTP11L	7,85	7,99	7,48
UTP14A	7,25	7,58	7,36
UTP15	5,63	5,55	5,01
UTP20	2,21	-0,81	2,95
UTX	7,65	7,32	9,24
UVRAG	8,94	7,95	8,68
UXS1	6,38	6,80	6,25
UXT	10,88	10,68	11,10
VAMP1	5,67	6,02	7,70
VAMP3	11,71	11,48	11,23
VAMP4	8,67	9,26	8,49
VAMP8	13,19	13,26	12,74
VANGL2	-3,32	3,87	-3,32
VAPA	6,71	6,42	7,90
VASH1	10,98	10,31	8,51
VASP	9,50	9,87	10,52
VAT1	11,63	11,59	7,24
VAV3	6,43	7,03	8,34
VAX1	4,66	4,42	4,98
VAX2	5,30	4,49	5,15
VBP1	9,45	9,48	9,30
VCPIP1	8,90	8,10	8,54
VDAC1	12,18	12,07	11,14
VDAC2	12,72	12,81	12,08
VDP	11,01	11,14	10,42
VDR	6,09	6,42	4,70
VEGFB	10,16	10,33	6,61
VEPH1	3,91	3,90	3,68
VEZT	8,41	8,82	8,09
VGLL4	9,32	9,62	8,75

VIL2	11,05	10,71	11,48
VILL	4,61	3,89	5,83
VIM	14,62	14,53	14,27
VIPR1	-1,87	-1,99	9,14
VKORC1	11,10	11,32	10,06
VMAC	4,62	4,97	4,98
VMO1	3,75	4,95	4,18
VNN1	3,62	8,67	10,94
VNN2	5,23	8,18	11,10
VNN3	1,98	3,56	7,59
VPRBP	4,99	5,23	4,89
VPS11	7,36	7,24	7,46
VPS13A	4,37	4,91	4,23
VPS13B	3,70	3,87	4,95
VPS13C	5,56	5,31	6,28
VPS16	8,20	8,10	8,49
VPS24	7,61	7,75	7,34
VPS25	8,11	8,12	8,06
VPS26A	10,07	10,09	9,06
VPS26B	8,13	8,13	8,83
VPS28	9,74	9,69	10,30
VPS29	11,97	12,47	12,46
VPS33A	7,27	7,54	7,39
VPS33B	8,13	8,20	8,32
VPS35	10,73	10,81	10,25
VPS37A	8,66	8,64	7,65
VPS37B	8,16	8,33	8,65
VPS37C	11,84	11,94	10,86
VPS39	8,47	8,74	8,78
VPS41	9,98	10,19	9,05
VPS4B	10,09	10,17	10,17
VPS52	7,75	7,67	7,86
VPS53	5,51	5,67	5,27
VPS54	7,01	7,11	7,92
VPS72	7,61	7,46	7,47
VRK2	7,39	7,14	7,76
VRK3	7,68	7,47	8,02
VSIG4	3,55	9,66	5,60
VSNL1	-1,78	0,55	2,10
VSX1	2,77	3,84	3,15
VTI1A	6,39	6,48	6,60
VTI1B	9,33	9,41	8,53
VTN	4,63	-0,08	-3,32
VWCE	4,84	1,65	-0,10
WAC	9,55	9,00	10,21
WAPAL	5,69	6,18	6,17
WARS	11,71	12,07	11,16
WAS	11,58	11,40	13,17
WASF2	9,34	9,21	10,00
WASF3	4,92	-1,85	-3,32
WASL	8,49	8,46	8,39
WBP2	13,30	13,23	12,52
WBP4	8,71	8,66	8,14
WBP5	8,11	10,11	5,16
WBSCR18	5,29	4,16	5,86
WBSCR22	8,32	8,34	8,54
WDFY2	6,40	6,74	8,44
WDFY3	4,88	5,04	6,32
WDHD1	4,18	5,12	1,83

WDR1	11,97	11,82	11,49
WDR12	9,11	9,18	7,70
WDR19	6,98	7,50	7,38
WDR20	8,02	7,85	7,45
WDR22	6,99	7,16	7,64
WDR23	5,87	6,49	7,38
WDR24	5,20	4,50	6,09
WDR25	6,05	6,94	7,15
WDR26	8,52	9,11	9,96
WDR33	7,90	7,38	7,71
WDR35	3,25	3,98	4,08
WDR36	8,39	7,41	7,58
WDR37	8,78	8,79	8,09
WDR4	6,52	6,22	6,23
WDR40A	9,05	9,92	10,16
WDR41	9,31	9,10	8,86
WDR42A	9,43	9,50	9,98
WDR44	7,11	7,12	7,24
WDR45	5,62	5,30	6,69
WDR45L	8,72	8,68	9,33
WDR46	7,02	6,79	7,84
WDR47	7,29	7,22	7,34
WDR48	8,41	7,90	8,72
WDR49	-0,82	-3,32	5,61
WDR51A	3,24	4,44	-0,48
WDR51B	8,40	8,73	9,01
WDR53	6,54	6,37	6,11
WDR54	7,66	5,49	8,00
WDR55	7,94	8,31	8,32
WDR57	8,89	8,79	9,09
WDR5B	4,40	4,47	4,31
WDR6	9,91	9,23	10,16
WDR61	10,26	10,61	10,40
WDR62	4,80	5,30	4,51
WDR63	5,75	5,77	4,53
WDR65	5,38	5,28	5,08
WDR67	7,97	8,24	7,02
WDR68	10,08	9,94	9,56
WDR7	8,65	8,62	7,31
WDR70	7,79	7,80	8,32
WDR71	8,35	8,24	8,18
WDR73	6,96	6,90	7,12
WDR74	6,59	6,63	7,26
WDR75	9,40	9,02	9,65
WDR77	4,52	3,39	3,90
WDR79	5,25	5,44	6,33
WDR8	7,17	7,11	8,75
WDR81	7,87	7,54	7,49
WDSOF1	8,35	9,02	7,98
WDSUB1	9,14	9,20	8,95
WEE1	-3,32	5,42	-2,61
WFIKKN2	4,16	3,23	1,06
WFS1	9,95	8,64	4,33
WHSC1L1	5,46	5,38	6,42
WHSC2	5,95	4,69	6,66
WIBG	8,67	8,31	8,03
WIPI1	8,51	9,09	8,09
WIT1	3,36	4,13	4,09
WNK1	9,16	9,05	9,81

WNK3	3,03	3,23	2,57
WNT11	3,14	3,52	3,28
WNT5A	8,09	-3,32	-3,32
WNT5B	7,98	4,78	5,35
WNT7A	5,38	5,05	5,24
WRB	8,28	8,64	7,86
WRN	7,33	7,49	7,29
WRNIP1	9,23	8,96	9,29
WSB1	8,97	8,68	9,50
WSB2	11,64	11,33	9,99
WT1	3,11	4,06	3,96
WTAP	7,85	7,72	8,37
WWC2	4,47	2,03	0,17
WWC3	5,84	5,72	7,49
WWOX	3,62	3,99	3,81
WWP1	10,27	10,52	10,85
WWP2	6,18	6,00	7,88
WWTR1	3,70	6,27	-0,81
XAB1	9,39	9,51	9,76
XAB2	8,70	8,40	9,25
XBP1	10,09	10,11	10,27
XK	3,16	3,62	2,09
XKR4	0,46	-3,32	-3,32
XKR6	5,30	5,07	4,79
XPA	6,97	7,00	7,56
XPC	10,24	10,13	9,69
XPNPEP1	11,61	11,34	11,10
XPNPEP2	7,55	6,41	5,15
XPO1	10,16	10,07	10,10
XPO4	8,69	8,65	8,91
XPO5	8,46	7,96	7,87
XPO6	10,14	9,14	9,59
XPR1	9,00	9,73	9,08
XRCC1	7,46	7,53	7,73
XRCC2	4,16	3,70	4,46
XRCC3	4,54	5,37	4,36
XRCC4	2,99	4,01	4,32
XRCC6	9,83	9,51	9,60
XRCC6BP1	7,38	7,73	7,46
XRN1	5,04	5,34	5,52
XRN2	8,19	8,37	9,20
XTP3TPA	9,58	9,23	8,08
XYLT1	6,31	4,50	5,66
YAF2	4,80	4,36	6,41
YARS	11,42	11,05	10,69
YARS2	9,25	9,39	8,21
YEATS4	7,45	7,69	6,77
YIF1A	9,49	10,07	9,09
YIF1B	8,62	8,65	7,94
YIPF1	9,36	9,90	9,55
YIPF2	6,13	6,23	5,62
YIPF3	9,68	9,70	10,19
YIPF4	9,95	9,53	8,99
YIPF5	6,61	6,80	5,59
YIPF6	9,61	9,71	8,80
YME1L1	9,48	9,41	9,15
YOD1	8,03	7,74	9,37
YPEL2	8,87	7,82	10,45
YPEL3	7,46	8,10	10,41

YPEL4	7,21	7,87	1,77
YRDC	9,53	9,12	10,87
YTHDC1	8,90	8,61	9,63
YTHDC2	5,97	5,86	7,12
YTHDF1	9,47	9,12	9,91
YTHDF2	10,28	10,05	10,08
YTHDF3	9,13	9,30	9,74
YWHAB	11,90	11,89	12,27
YWHAG	10,18	10,09	9,26
YWHAH	13,39	12,73	12,21
YWHAQ	12,90	12,78	12,37
YWHAZ	11,95	12,22	12,15
YY1	11,75	11,38	12,14
YY1AP1	8,85	8,51	9,45
ZADH1	2,94	3,52	1,52
ZADH2	8,20	7,83	8,48
ZBED3	6,77	6,21	6,26
ZBED4	8,41	8,02	8,50
ZBTB11	7,17	6,36	8,23
ZBTB16	3,67	3,60	7,81
ZBTB2	7,08	6,48	8,24
ZBTB20	5,90	5,50	7,52
ZBTB22	7,27	7,22	7,82
ZBTB24	7,46	6,44	7,06
ZBTB25	5,75	5,80	6,25
ZBTB26	5,27	4,55	4,77
ZBTB3	7,02	6,73	5,73
ZBTB32	3,74	2,79	2,80
ZBTB33	10,60	10,26	10,15
ZBTB39	5,66	5,39	6,05
ZBTB4	8,47	8,15	8,37
ZBTB41	6,70	6,04	6,55
ZBTB43	9,38	9,21	9,47
ZBTB5	5,11	4,22	5,34
ZBTB7A	7,04	7,11	7,26
ZBTB7B	5,50	5,21	7,37
ZBTB9	7,40	8,17	5,59
ZC3H10	7,12	6,65	5,87
ZC3H11A	4,29	4,04	5,49
ZC3H12A	7,89	8,74	8,67
ZC3H3	6,08	5,82	6,77
ZC3H7A	9,47	9,29	10,66
ZC3H8	9,14	8,48	8,33
ZC3HAV1	9,17	8,25	9,49
ZC3HC1	8,93	9,08	8,22
ZCCHC11	6,39	6,99	7,33
ZCCHC12	4,41	4,91	4,13
ZCCHC14	7,34	7,20	7,39
ZCCHC17	8,78	9,24	8,82
ZCCHC3	6,27	6,00	6,59
ZCCHC6	10,30	10,00	10,68
ZCCHC7	8,71	8,24	9,70
ZCCHC8	6,28	5,84	6,59
ZCCHC9	9,49	9,50	9,17
ZCRB1	6,77	6,51	5,78
ZCWPW1	7,11	6,53	6,60
ZDHHC1	5,39	4,34	8,39
ZDHHC12	8,25	8,61	7,91
ZDHHC14	7,88	8,54	7,62

ZDHHC16	8,94	9,43	8,61
ZDHHC17	7,95	7,98	8,76
ZDHHC2	5,53	5,87	7,05
ZDHHC21	3,35	5,61	-0,71
ZDHHC22	3,24	3,13	3,67
ZDHHC23	4,68	4,66	5,39
ZDHHC24	7,67	7,90	6,14
ZDHHC3	9,81	10,51	9,82
ZDHHC5	8,99	9,06	8,54
ZDHHC6	8,89	9,07	9,22
ZDHHC7	11,31	11,30	12,29
ZDHHC9	4,61	5,29	3,63
ZFAND1	8,11	8,14	9,39
ZFAND2A	8,64	8,67	10,59
ZFAND2B	8,19	7,90	8,91
ZFAND3	7,26	6,82	8,29
ZFP106	9,84	10,17	11,24
ZFP161	8,64	8,52	8,91
ZFP28	4,55	4,61	4,07
ZFP3	7,11	6,40	4,70
ZFP30	6,12	5,41	2,72
ZFP36	9,79	10,46	13,15
ZFP36L1	11,37	9,85	12,14
ZFP90	7,84	7,62	8,28
ZFP91	10,11	9,97	10,54
ZFPL1	6,75	6,82	6,38
ZFX	6,23	6,10	7,54
ZFYVE1	7,90	7,66	7,51
ZFYVE16	8,83	9,15	7,74
ZFYVE19	8,03	8,11	8,15
ZFYVE20	10,00	9,73	9,33
ZFYVE21	10,20	9,66	8,80
ZFYVE26	10,68	11,04	9,08
ZFYVE28	5,44	5,28	4,64
ZHX1	9,84	8,95	8,79
ZHX2	5,26	5,50	6,16
ZIC2	4,27	4,15	2,89
ZIK1	6,05	5,58	8,90
ZKSCAN1	7,33	6,86	7,14
ZMAT1	4,53	3,99	5,55
ZMAT2	9,43	9,90	10,03
ZMAT4	6,02	6,06	6,49
ZMAT5	7,05	7,39	7,42
ZMPSTE24	11,56	12,11	11,13
ZMYM1	7,65	7,29	7,41
ZMYM3	6,64	6,33	6,61
ZMYM4	8,12	8,25	9,22
ZMYM5	5,94	5,84	7,28
ZMYM6	9,32	9,76	9,71
ZMYND10	4,70	4,87	5,26
ZMYND11	7,29	7,58	7,29
ZMYND12	5,19	6,12	4,89
ZMYND15	7,74	9,26	7,79
ZNF10	5,51	5,46	4,43
ZNF101	6,24	5,42	5,76
ZNF12	7,32	6,62	7,53
ZNF121	6,45	5,82	5,96
ZNF132	4,43	3,66	-0,59
ZNF133	5,94	5,98	6,41

ZNF134	6,87	6,60	6,06
ZNF135	4,82	4,96	4,79
ZNF138	3,97	2,83	3,11
ZNF140	7,61	7,00	6,19
ZNF141	4,45	3,11	4,16
ZNF142	7,52	6,66	7,62
ZNF143	8,14	8,10	8,79
ZNF148	9,45	9,11	10,02
ZNF154	5,20	5,18	5,13
ZNF155	4,56	4,42	4,05
ZNF157	4,33	4,03	3,45
ZNF160	5,68	5,61	6,46
ZNF17	7,41	7,26	6,42
ZNF174	5,67	5,34	5,06
ZNF175	7,22	6,45	5,58
ZNF177	5,48	5,33	4,74
ZNF18	7,24	7,36	7,38
ZNF180	8,36	8,55	8,22
ZNF181	6,25	5,72	4,43
ZNF184	5,65	5,63	5,26
ZNF185	8,54	8,51	10,14
ZNF187	6,01	5,59	5,75
ZNF192	3,97	3,74	3,74
ZNF193	6,95	5,84	6,35
ZNF195	7,54	7,22	7,44
ZNF197	4,57	3,78	3,11
ZNF2	5,57	5,28	5,11
ZNF20	6,71	6,14	6,15
ZNF202	5,13	4,98	3,61
ZNF205	4,34	4,05	3,30
ZNF207	11,04	10,96	10,96
ZNF211	7,45	6,97	8,31
ZNF212	7,65	7,39	7,68
ZNF213	6,83	6,87	6,56
ZNF214	4,19	4,43	4,11
ZNF217	10,41	9,44	9,65
ZNF219	8,43	8,48	5,74
ZNF22	7,54	6,68	8,65
ZNF222	4,78	4,53	2,42
ZNF223	9,56	9,44	11,18
ZNF224	6,63	6,29	6,68
ZNF226	6,76	7,04	7,12
ZNF227	6,05	5,73	6,13
ZNF228	5,90	6,02	4,74
ZNF23	6,42	6,20	6,21
ZNF230	5,59	5,13	5,35
ZNF232	5,14	5,04	5,02
ZNF234	7,12	6,91	6,36
ZNF235	5,71	5,48	5,49
ZNF236	5,76	5,14	5,47
ZNF238	3,72	3,25	6,02
ZNF24	6,50	6,93	7,78
ZNF248	5,68	5,04	5,28
ZNF25	8,84	8,65	9,23
ZNF250	6,37	5,21	5,00
ZNF256	7,12	7,10	5,78
ZNF259	8,22	7,93	7,92
ZNF26	6,63	6,35	7,42
ZNF263	9,76	9,26	9,47

ZNF264	6,71	5,91	6,68
ZNF266	8,89	8,67	9,83
ZNF268	4,88	4,62	3,74
ZNF271	7,78	7,89	8,15
ZNF274	9,01	8,51	7,02
ZNF276	4,48	4,45	5,36
ZNF28	6,53	6,14	5,28
ZNF281	8,92	9,18	10,80
ZNF282	6,81	6,29	6,27
ZNF283	5,22	4,59	5,15
ZNF289	8,52	8,21	8,71
ZNF294	7,55	7,39	8,08
ZNF295	8,05	7,73	7,57
ZNF3	4,88	4,68	5,37
ZNF30	5,47	4,99	2,57
ZNF304	4,99	4,76	3,92
ZNF311	3,50	4,02	2,18
ZNF317	9,62	8,65	8,51
ZNF318	9,34	9,21	9,20
ZNF319	8,17	7,50	9,15
ZNF32	6,60	6,67	6,31
ZNF322B	4,53	4,19	3,20
ZNF323	6,44	5,77	3,30
ZNF324	6,73	6,00	6,86
ZNF326	4,74	4,36	5,04
ZNF329	7,37	7,40	5,43
ZNF330	9,82	10,17	9,90
ZNF331	7,44	6,66	6,46
ZNF333	5,23	5,00	7,05
ZNF334	3,51	4,12	3,81
ZNF335	6,22	6,14	7,70
ZNF337	7,22	6,99	7,75
ZNF33A	2,51	1,21	4,85
ZNF341	6,40	6,31	6,66
ZNF342	7,48	6,33	9,14
ZNF343	5,66	5,41	5,66
ZNF345	4,30	4,34	4,76
ZNF346	4,23	1,98	5,50
ZNF347	3,90	2,57	-0,89
ZNF35	7,81	7,69	5,96
ZNF350	7,90	7,94	6,76
ZNF358	7,81	7,66	8,12
ZNF364	9,64	9,34	9,38
ZNF365	1,03	3,65	-3,32
ZNF366	9,82	8,44	5,29
ZNF367	4,67	5,14	4,65
ZNF37A	4,66	3,62	4,34
ZNF384	7,10	6,90	8,02
ZNF385	8,71	8,89	9,20
ZNF394	7,00	6,26	7,82
ZNF395	6,32	7,71	9,66
ZNF397	4,07	2,67	0,56
ZNF398	5,51	5,18	6,00
ZNF403	9,70	9,68	9,86
ZNF407	6,73	6,54	6,94
ZNF408	5,23	4,52	5,89
ZNF41	3,20	3,78	4,13
ZNF410	8,25	8,93	8,93
ZNF417	4,89	4,85	4,92

ZNF419	7,33	7,06	6,00
ZNF420	5,25	4,48	3,17
ZNF425	4,84	2,50	2,41
ZNF426	6,02	6,08	5,78
ZNF429	3,85	2,98	2,64
ZNF43	4,13	3,94	4,22
ZNF430	6,97	6,29	6,39
ZNF431	6,10	5,40	6,76
ZNF432	5,68	5,78	5,56
ZNF434	7,74	7,85	7,44
ZNF436	6,50	6,41	6,21
ZNF442	4,56	4,59	2,47
ZNF444	5,81	6,42	5,74
ZNF446	5,33	5,07	5,97
ZNF451	6,79	6,48	7,55
ZNF452	3,36	2,41	3,38
ZNF467	5,75	5,92	11,61
ZNF468	6,08	6,02	5,59
ZNF471	5,15	4,64	4,85
ZNF473	3,17	3,03	3,62
ZNF480	6,63	6,56	5,91
ZNF484	5,39	5,96	6,01
ZNF485	4,96	4,75	4,61
ZNF488	4,29	0,53	4,08
ZNF496	4,60	4,42	4,54
ZNF497	2,56	3,76	3,92
ZNF498	5,43	5,46	5,92
ZNF500	6,22	5,57	6,89
ZNF503	5,75	5,53	7,57
ZNF509	4,76	5,28	4,92
ZNF510	5,03	4,92	5,42
ZNF511	9,19	9,19	10,11
ZNF512	9,15	8,85	9,88
ZNF513	7,39	6,94	7,64
ZNF518	7,73	7,08	8,19
ZNF524	8,59	8,20	9,23
ZNF526	7,16	6,66	7,34
ZNF529	7,57	7,16	6,52
ZNF530	3,62	2,97	2,44
ZNF532	3,16	4,87	6,69
ZNF536	4,92	4,94	5,13
ZNF540	3,73	3,98	3,89
ZNF541	2,98	3,96	0,33
ZNF543	5,34	4,65	2,36
ZNF544	7,87	8,01	6,30
ZNF545	6,28	5,09	1,40
ZNF548	6,85	6,52	7,17
ZNF549	2,43	0,43	2,17
ZNF550	4,36	4,68	4,45
ZNF551	5,08	4,81	5,58
ZNF557	7,44	7,41	7,22
ZNF559	8,00	7,59	7,97
ZNF561	6,22	5,73	5,17
ZNF562	8,85	8,20	8,52
ZNF564	6,41	5,68	5,76
ZNF565	5,72	5,36	6,02
ZNF567	5,22	4,20	3,81
ZNF569	7,13	6,92	6,79
ZNF570	5,45	5,12	4,94

ZNF573	7,59	6,92	6,68
ZNF575	3,77	4,01	4,26
ZNF576	6,59	6,31	5,23
ZNF580	5,93	5,43	6,39
ZNF581	9,10	8,24	10,31
ZNF582	4,23	3,14	3,19
ZNF583	6,66	7,00	7,08
ZNF585A	7,46	7,56	6,41
ZNF585B	6,63	6,54	5,02
ZNF587	4,23	2,50	4,28
ZNF592	4,60	4,32	5,44
ZNF593	8,68	8,85	8,62
ZNF595	5,56	5,16	5,12
ZNF597	5,65	5,08	5,39
ZNF606	5,33	4,22	3,73
ZNF609	7,03	6,63	6,72
ZNF613	7,37	6,52	4,99
ZNF614	6,65	6,71	5,24
ZNF615	7,99	7,41	6,83
ZNF616	7,31	6,67	0,06
ZNF618	5,31	5,61	4,84
ZNF619	2,65	3,52	3,75
ZNF620	4,35	3,58	3,72
ZNF621	8,26	8,41	7,93
ZNF622	11,05	10,90	10,41
ZNF624	5,11	4,79	4,66
ZNF626	6,28	5,52	2,98
ZNF628	3,42	3,51	4,82
ZNF630	5,12	4,24	5,70
ZNF642	7,02	4,16	5,61
ZNF643	4,12	3,71	3,19
ZNF644	6,80	6,48	6,83
ZNF646	6,05	5,81	6,05
ZNF649	6,79	6,35	4,12
ZNF653	6,96	6,48	7,38
ZNF655	6,06	6,06	6,49
ZNF658	4,78	4,75	4,11
ZNF663	4,46	4,81	4,79
ZNF664	9,60	9,76	8,44
ZNF668	8,07	8,03	8,27
ZNF669	5,17	4,46	5,00
ZNF671	7,36	7,01	6,89
ZNF672	9,50	8,77	8,39
ZNF673	4,49	4,11	5,02
ZNF675	4,76	3,98	4,18
ZNF680	7,26	6,59	5,85
ZNF683	5,45	5,40	5,10
ZNF684	6,55	6,67	6,36
ZNF687	4,43	4,31	5,09
ZNF688	5,08	5,08	6,06
ZNF689	8,85	8,12	9,43
ZNF69	6,06	5,68	5,31
ZNF691	6,08	5,58	4,67
ZNF692	5,16	5,03	5,52
ZNF696	6,23	5,34	5,00
ZNF7	7,12	6,87	7,06
ZNF700	7,26	6,83	7,77
ZNF701	7,07	6,73	5,88
ZNF702	6,09	6,59	4,97

ZNF706	7,21	7,12	7,10
ZNF707	5,84	5,20	5,71
ZNF708	4,54	3,86	3,63
ZNF71	3,74	3,85	4,39
ZNF710	4,93	5,17	5,33
ZNF714	4,90	5,12	4,01
ZNF720	3,95	3,73	3,47
ZNF721	8,52	7,91	8,03
ZNF740	5,63	5,06	5,49
ZNF746	9,30	8,59	9,69
ZNF747	3,69	4,45	4,17
ZNF75	5,74	4,96	6,27
ZNF75A	6,71	5,56	6,49
ZNF76	6,12	6,21	6,22
ZNF79	5,41	4,55	3,31
ZNF8	4,02	4,06	3,84
ZNF83	9,12	9,27	9,28
ZNF84	8,15	6,96	6,40
ZNF85	4,84	4,40	4,96
ZNF91	7,86	7,30	8,51
ZNF92	2,70	2,71	3,49
ZNF93	6,10	5,93	6,80
ZNFX1	9,93	9,62	8,80
ZNHIT1	10,45	10,41	10,17
ZNHIT2	6,17	5,77	6,54
ZNHIT3	9,46	9,50	9,72
ZNHIT4	5,55	5,76	7,10
ZNRD1	7,32	7,66	7,92
ZRANB3	2,68	4,24	3,00
ZSCAN2	4,75	4,53	5,42
ZSCAN5	7,57	7,63	6,46
ZSWIM1	8,54	8,49	8,35
ZSWIM4	5,60	6,09	5,19
ZW10	4,53	4,74	3,53
ZWILCH	5,15	4,93	2,99
ZWINT	4,44	5,97	3,21
ZXDB	6,98	6,47	7,04
ZXDC	6,16	6,31	7,78
ZYG11B	10,72	10,54	10,62
ZYX	11,90	12,43	12,53
ZZEF1	9,15	8,92	9,71
ZZZ3	8,51	9,02	8,33
BA16L21.2.1	8,63	8,41	7,48
ISG15	8,64	9,28	10,53
MGC40168	4,57	3,99	4,08
IIP45	6,47	6,23	7,24
RCN3	5,45	5,74	7,77
EIF3I	10,67	10,90	11,06
AK3	10,25	10,14	8,64
ZRANB2	9,50	9,58	10,29
LOC149620	4,53	4,36	3,30
SEC22B	9,45	9,87	8,94
VPS45	8,82	9,11	9,17
TRK1	5,83	5,46	4,84
FCRLA	5,91	8,78	4,23
ASTN1	4,04	3,90	2,79
QSOX1	8,97	7,96	6,71
SMC6	7,40	6,99	7,15
HADH	9,94	10,28	8,90

FOXN2	10,70	10,31	11,18
CIAO1	8,71	8,83	8,53
REV1	8,71	8,22	9,26
ZEB2	10,41	10,60	11,47
SPC25	-3,32	5,40	-3,32
DPH3	9,12	9,23	7,50
SEC22C	8,88	9,20	7,52
SIT1	4,44	4,42	4,10
CAST	8,16	7,90	9,16
APPL1	9,31	9,13	9,13
CAPS	4,35	4,73	5,64
SEC22A	8,12	8,39	7,66
CNBP	11,86	12,09	12,56
HLTF	6,37	7,27	5,94
B3GALNT1	5,20	2,08	-3,32
FLJ90036	4,59	4,58	4,95
COX18	4,55	4,14	4,16
SEC31A	8,11	8,17	8,35
PIN1	10,08	10,42	10,01
NAIP	5,45	4,95	6,53
GPR98	4,26	3,66	3,37
HSPA9	12,10	12,09	11,31
CNR2	4,63	4,68	3,95
ZSCAN16	7,34	6,80	7,80
FLJ35429	5,51	5,56	5,24
MED20	8,24	8,39	9,39
VEGFA	4,67	4,24	5,22
LOC441151	1,84	1,89	-0,59
MCM9	3,48	2,98	6,31
EIF3B	10,01	9,85	10,27
NOD1	7,27	6,46	6,60
BBS9	5,51	5,96	6,30
EIF4H	9,67	10,07	10,44
CLIP2	9,33	8,72	8,82
ZSCAN21	7,50	7,12	7,29
AGK	7,78	8,24	7,79
DEF6	8,03	7,83	10,09
NEFM	4,22	4,54	4,47
EIF3E	13,01	12,70	13,56
MED30	7,87	7,83	9,16
LOC441459	3,86	4,45	4,31
RMI1	6,81	6,45	5,70
BMI1	9,99	9,24	9,41
ZCD1	8,76	8,34	6,98
DYDC2	5,33	5,38	6,01
LCOR	7,98	7,49	9,05
SMC3	8,33	8,51	8,86
EIF3A	11,78	11,33	11,79
PSTK	6,00	5,79	6,27
CTR9	7,40	7,26	7,46
DPH4	6,70	7,01	6,62
CLP1	8,24	7,95	8,29
RSF1	7,11	6,49	6,40
MGC33948	2,82	2,97	0,26
ERC1	4,24	4,49	4,52
MED21	6,57	6,13	5,61
LOC144983	1,59	1,92	-0,92
MGC13168	5,30	5,20	5,34
APPL2	8,87	8,27	7,75

ISCU	9,67	9,47	8,78
ANKRD13A	10,72	10,17	11,36
C12ORF8	4,43	4,65	5,32
MED13L	6,67	6,06	8,12
DNCL1	13,05	12,89	12,21
SPPL3	5,66	5,02	6,30
CLIP1	5,56	5,75	6,03
ZMYM2	8,20	7,84	8,81
PDX1	4,15	3,67	4,07
LOC387921	10,15	9,81	8,42
FOXO1	8,32	6,19	8,25
KIAA0564	6,83	8,04	6,52
KIAA0564	6,83	8,04	6,52
LOC220416	4,40	4,45	3,09
TRIM13	7,63	5,87	5,26
LOC440145	6,81	7,02	6,54
DIS3	3,69	4,21	4,13
RBM26	7,74	7,45	8,24
HOMEZ	8,82	8,25	6,85
EAPP	8,95	9,00	10,03
ODC1	8,78	9,62	8,28
TXNDC1	11,41	11,12	11,34
DNAL1	5,55	6,01	4,44
FOXN3	4,68	4,50	4,60
SERPINA11	4,83	4,03	4,44
KLC1	7,77	7,57	7,52
ZSCAN29	4,92	4,79	4,97
EIF3J	8,67	8,62	8,43
TRIM69	3,74	3,20	3,83
EID1	5,58	4,46	6,24
EDC3	6,34	6,37	6,28
ZFAND6	9,61	10,16	8,54
KIAA0430	10,59	10,67	11,52
MGC3020	5,10	3,47	-0,95
NOD2	6,40	7,80	10,85
CTF8	5,19	5,21	5,09
NOB1	5,68	5,33	6,87
CHMP1A	9,19	9,09	9,15
XAF1	8,54	8,35	10,74
GPR158L1	3,35	2,72	4,41
NACA2	4,29	4,13	5,07
BPTF	5,92	5,60	6,31
EIF4A3	11,55	11,24	11,08
KIAA1303	4,58	4,54	4,21
ELP2	9,00	8,43	9,12
EIF3G	11,09	10,46	11,95
ECSIT	6,67	6,59	7,16
MED26	7,90	7,37	8,51
MAP1S	11,77	9,19	8,80
NCAN	4,04	4,13	4,32
SAE2	5,89	5,49	5,97
TBCB	10,31	10,26	10,61
EIF3K	12,04	12,16	12,44
MED29	8,57	8,51	9,10
FIZ1	6,55	6,23	6,49
ZSCAN22	4,60	3,58	3,94
MZF1	7,60	7,30	8,51
RBCK1	8,26	8,02	9,43
FAM110A	8,11	8,38	10,05

KIAA1271	3,66	4,55	4,59
GZF1	5,55	3,59	6,76
KIAA0980	6,28	6,73	2,51
KIAA1755	4,10	3,47	0,08
SNX21	4,05	4,44	5,21
CTSA	10,78	11,04	10,08
ZMYND8	6,59	7,15	6,74
N6AMT1	4,06	4,33	0,97
IL17RA	8,62	9,16	9,92
LOC402055	7,51	7,48	6,45
HSCB	6,71	6,70	7,36
GEMIN8	4,58	4,86	4,42
KSR2	3,22	3,53	3,65
LOC401589	2,21	3,55	1,58
JARID1C	5,58	4,79	5,63
HSD17B10	9,52	9,69	10,19
FAM104B	6,63	7,64	6,69
FOXO4	7,96	8,67	9,40
LOC340527	-1,09	2,83	4,21
DKFZP564K142	10,59	10,10	11,46
TAF9L	5,90	6,29	6,33
CENPI	3,26	5,14	-0,70
ALG13	8,89	9,20	8,80
