# Supplementary material for: Genome-wide promoter analysis of histone modifications in human monocyte-derived antigen presenting cells
Source: BMC Genomics. 2010 Nov 18;11:642. doi: 10.1186/1471-2164-11-642 (PMC3091769; doi:10.1186/1471-2164-11-642)
Supplement: Additional file 2 — Tserel et al BMC Genomics. Contains Supplementary Tables S2-S6. Size 7.2 MB [file 1471-2164-11-642-S2.ZIP › Supplementary Table 4. Expression fold change..pdf]

Supplementary Table 4. Expression signal log2 fold changes between monocyte, macrophage and dendritic cell subpopulations.

	DC - MF	DC - MO	MF - MO
15E1.2	-0,23	0,93	1,16
A2M	0,76	11,00	10,24
A4GNT	0,36	-0,53	-0,89
AADACL1	-0,91	2,12	3,02
AAMP	-0,25	-0,88	-0,63
AARSD1	0,12	-0,20	-0,32
AASDH	-0,24	-0,36	-0,13
AASDHPPT	0,04	0,41	0,38
ABCA12	-0,37	-0,23	0,14
ABCA3	1,09	1,28	0,19
ABCA5	0,75	3,29	2,54
ABCA9	-0,20	0,16	0,35
ABCB4	2,00	6,49	4,48
ABCB6	0,93	1,72	0,80
ABCB7	0,18	-0,29	-0,46
ABCB9	-0,59	0,89	1,48
ABCC10	0,06	0,19	0,14
ABCC11	-0,21	-0,07	0,14
ABCC13	2,58	1,78	-0,80
ABCC2	-0,61	-0,19	0,43
ABCE1	-0,29	0,31	0,59
ABCF1	0,02	0,33	0,31
ABCF2	0,32	-0,66	-0,97
ABCF3	0,64	0,65	0,01
ABCG1	-1,49	3,48	4,98
ABCG2	4,88	7,45	2,57
ABHD10	-0,34	0,88	1,22
ABHD11	-0,15	-0,93	-0,78
ABHD12	1,29	3,00	1,71
ABHD13	0,05	-0,49	-0,54
ABHD14A	0,02	-0,06	-0,08
ABHD3	-0,30	-0,66	-0,36
ABHD5	-0,73	-0,66	0,07
ABHD6	0,58	2,45	1,87
ABHD8	0,24	0,81	0,57
ABI1	0,36	0,14	-0,22
ABI3	2,33	-0,72	-3,05
ABL1	0,71	0,19	-0,52
ABL2	-0,61	2,96	3,57
ABR	-0,03	1,14	1,17
ABRA	-1,05	1,70	2,75
ACAA1	-1,07	-1,08	-0,01
ACACA	-0,15	1,40	1,55
ACACB	-0,17	0,05	0,22
ACAD10	0,43	-0,44	-0,87
ACAD11	-0,15	-0,46	-0,31
ACAD8	-0,41	-0,48	-0,07
ACAD9	-0,42	0,21	0,62
ACADM	-0,75	1,23	1,98
ACADS	-0,42	0,18	0,60
ACADSB	-0,08	-0,65	-0,57
ACADVL	-0,06	-0,51	-0,45
ACAT1	-0,30	1,14	1,44
ACAT2	-0,66	3,38	4,04
ACBD3	-0,39	-0,04	0,35

ACBD5	0,28	-0,05	-0,33
ACBD6	-0,04	-0,35	-0,30
ACCN3	0,10	0,32	0,22
ACCN5	0,03	0,10	0,06
ACE	0,99	2,61	1,62
ACIN1	0,63	0,04	-0,59
ACO1	-1,49	1,89	3,38
ACO2	0,07	1,15	1,08
ACOT1	-1,35	3,82	5,17
ACOT11	-1,37	1,03	2,40
ACOT2	-1,17	4,47	5,64
ACOT7	0,83	6,35	5,52
ACOT9	0,09	0,70	0,62
ACOX1	-0,58	1,10	1,68
ACOX2	-1,62	1,58	3,20
ACOX3	0,70	0,80	0,10
ACP1	-0,40	-0,01	0,39
ACP2	-0,81	2,08	2,90
ACP5	-0,12	5,76	5,88
ACPL2	2,27	-0,23	-2,50
ACPP	-0,47	-2,78	-2,31
ACPT	0,83	0,20	-0,63
ACR	0,34	-0,60	-0,93
ACRC	0,26	-0,29	-0,56
ACRV1	0,69	0,61	-0,09
ACSBG2	-0,37	0,72	1,09
ACSL3	0,39	3,09	2,70
ACSL4	-0,43	-0,14	0,29
ACSL5	0,66	0,66	-0,01
ACSS2	-0,11	0,96	1,06
ACTA2	-0,46	2,46	2,91
ACTB	0,06	0,07	0,01
ACTL6A	0,00	0,28	0,28
ACTL6B	-0,19	-0,23	-0,04
ACTL7A	0,21	0,29	0,08
ACTL8	-1,43	-0,96	0,47
ACTN4	0,16	-0,04	-0,20
ACTR10	-0,44	0,30	0,74
ACTR1A	-0,02	0,51	0,54
ACTR2	0,01	-0,44	-0,45
ACTR3	-0,04	0,21	0,25
ACTR5	0,15	-0,86	-1,01
ACTR8	0,35	1,64	1,29
ACTRT1	0,29	0,41	0,12
ACVR1	0,26	0,54	0,28
ACVR1B	-1,20	-1,19	0,01
ACVR2A	0,51	-0,72	-1,24
ACVRL1	-1,44	0,44	1,87
ACY1	-0,45	0,93	1,38
ACY1L2	-0,24	-1,37	-1,13
ACY3	-0,23	0,15	0,38
ACYP1	0,31	1,77	1,46
ACYP2	-0,47	-0,38	0,09
ADA	-0,80	-2,08	-1,27
ADAL	-0,67	-0,67	0,00
ADAM12	1,84	3,59	1,74
ADAM15	0,56	0,84	0,29
ADAM17	0,08	-0,12	-0,20
ADAM18	-0,21	-0,22	0,00

ADAM21	-0,17	0,61	0,78
ADAM30	0,91	0,07	-0,84
ADAM7	-0,05	-0,82	-0,77
ADAM8	1,32	-1,75	-3,07
ADAM9	-0,26	0,81	1,08
ADAMDEC1	-6,20	-1,76	4,44
ADAMTS13	-0,16	-0,32	-0,16
ADAMTS17	0,65	0,31	-0,33
ADAMTSL4	0,96	1,51	0,55
ADAR	0,42	-0,49	-0,91
ADAT1	-0,04	0,24	0,28
ADCK4	0,06	-0,47	-0,53
ADCY7	0,62	-0,85	-1,47
ADCY9	0,59	0,14	-0,45
ADHFE1	1,42	-0,33	-1,75
ADIPOR1	-0,26	-0,32	-0,05
ADIPOR2	-0,12	-0,29	-0,16
ADK	0,01	0,53	0,52
ADM2	0,96	0,64	-0,33
ADMR	-0,08	-0,52	-0,43
ADNP	0,52	0,02	-0,50
ADORA3	2,53	2,27	-0,26
ADPGK	0,30	0,07	-0,23
ADPRH	-0,24	-0,04	0,20
ADRBK2	0,63	1,47	0,84
ADRM1	-0,01	0,36	0,37
ADSS	-0,53	-0,84	-0,31
ADSSL1	-0,39	-1,70	-1,32
AFG3L1	0,33	-0,57	-0,90
AFMID	-0,25	-1,31	-1,06
AGBL4	0,17	-0,02	-0,19
AGER	2,93	-1,39	-4,32
AGGF1	0,19	0,75	0,56
AGL	-0,08	0,16	0,25
AGPAT1	0,46	0,36	-0,11
AGPAT3	0,02	-0,42	-0,44
AGPAT5	-0,38	-0,24	0,13
AGPAT7	1,50	1,61	0,11
AGPS	-1,17	0,95	2,12
AGRP	-4,64	-0,88	3,75
AGT	0,02	-0,64	-0,65
AGTR2	0,34	0,68	0,34
AHCYL1	-0,70	0,43	1,12
AHDC1	-0,29	-0,36	-0,07
AHI1	-1,07	1,89	2,95
AHNAK	0,38	-0,45	-0,82
AHR	0,43	-0,65	-1,08
AHSA1	0,07	-0,02	-0,10
AHSG	-0,84	-0,99	-0,15
AICDA	-0,72	0,94	1,66
AIM2	-0,73	-2,31	-1,58
AIPL1	0,31	-0,53	-0,84
AK2	0,72	0,50	-0,22
AKAP1	0,65	0,24	-0,41
AKAP12	3,95	5,16	1,21
AKAP13	-0,10	-1,54	-1,44
AKAP14	-1,92	-3,21	-1,29
AKAP5	1,03	2,40	1,37
AKAP7	-0,19	-1,39	-1,20

AKAP8	0,33	-1,38	-1,70
AKAP8L	0,96	-1,32	-2,28
AKR1C3	-3,70	1,88	5,58
AKR7A2	-0,79	0,56	1,35
AKT1S1	0,04	0,26	0,22
AKT2	0,23	-0,28	-0,52
AKT3	-0,28	0,04	0,32
ALAS1	-0,72	3,11	3,84
ALB	0,27	0,04	-0,23
ALCAM	0,19	2,32	2,14
ALDH16A1	-0,03	-0,17	-0,14
ALDH1A1	-1,05	-0,31	0,74
ALDH1A2	-0,52	7,64	8,16
ALDH3B1	-0,58	-0,91	-0,33
ALDH3B2	-0,38	0,29	0,68
ALDH4A1	-0,70	1,19	1,89
ALDH5A1	-0,12	11,11	11,23
ALDH6A1	0,03	0,59	0,56
ALDOB	-0,30	0,29	0,59
ALDOC	0,07	0,95	0,88
ALG1	-0,12	1,00	1,11
ALG11	-0,21	0,26	0,48
ALG12	-0,02	0,05	0,06
ALG14	-0,76	1,28	2,03
ALG2	0,69	0,54	-0,15
ALG3	-0,80	-0,13	0,67
ALG8	-0,67	0,93	1,60
ALG9	-1,08	0,19	1,27
ALKBH1	-0,06	0,42	0,48
ALKBH2	-0,16	-0,89	-0,73
ALKBH3	-0,14	0,76	0,90
ALKBH4	0,39	-0,33	-0,72
ALKBH8	-0,13	0,06	0,20
ALMS1	0,37	-0,49	-0,86
ALOX12B	0,46	0,12	-0,33
ALOX15B	-1,14	0,52	1,65
ALOX5AP	-1,03	0,85	1,88
ALPI	-1,39	-0,82	0,57
ALPK2	1,12	1,03	-0,09
ALPP	0,34	-1,48	-1,82
ALS2CL	0,15	0,29	0,13
ALS2CR12	-0,13	-0,71	-0,57
ALS2CR14	0,00	-1,86	-1,86
ALS2CR2	0,25	0,80	0,54
ALS2CR4	-1,65	0,41	2,06
ALS2CR8	0,59	1,16	0,57
AMACR	0,56	2,01	1,45
AMBN	1,24	1,42	0,18
AMDHD2	0,87	2,86	1,99
AMHR2	0,11	-1,78	-1,89
AMICA1	-2,24	-3,08	-0,84
AMOTL1	-0,57	0,62	1,19
AMPD2	-2,58	-2,54	0,04
AMPD3	-1,10	0,30	1,40
AMY1B	0,49	0,55	0,06
AMY1C	0,12	0,20	0,08
AMY2A	-0,92	-1,29	-0,37
AMY2B	-1,88	-1,06	0,83
AMZ2	0,19	1,16	0,97

ANAPC10	-0,39	0,51	0,90
ANAPC11	-0,15	0,35	0,51
ANAPC13	0,41	-0,40	-0,81
ANAPC2	0,31	-0,74	-1,05
ANAPC5	-0,08	-0,76	-0,68
ANAPC7	-0,76	0,81	1,58
ANG	-2,10	-3,40	-1,30
ANGEL1	-0,54	1,52	2,06
ANGEL2	0,11	-1,15	-1,26
ANGPT1	-0,75	-6,96	-6,21
ANGPTL3	0,33	0,45	0,12
ANGPTL6	0,11	1,18	1,07
ANK1	0,00	-0,48	-0,48
ANK2	-0,20	0,81	1,02
ANKDD1A	-0,22	-0,86	-0,64
ANKFY1	0,37	-0,29	-0,66
ANKHD1	0,30	-0,36	-0,67
ANKMY1	0,40	-0,95	-1,35
ANKMY2	0,07	1,14	1,07
ANKRA2	-0,20	0,19	0,39
ANKRD10	0,64	0,33	-0,30
ANKRD12	0,88	0,26	-0,63
ANKRD13C	-0,14	0,53	0,67
ANKRD16	-0,23	-0,21	0,02
ANKRD17	-0,04	0,02	0,06
ANKRD22	-1,50	-0,69	0,81
ANKRD38	-3,03	3,11	6,15
ANKRD40	-0,59	0,16	0,75
ANKRD41	-1,00	-1,99	-0,99
ANKRD44	1,45	-6,65	-8,10
ANKRD46	0,80	1,14	0,34
ANKRD49	0,04	-0,40	-0,45
ANKRD7	-0,31	-0,51	-0,20
ANKS1B	0,59	0,72	0,13
ANKS3	4,35	-1,24	-5,58
ANKZF1	0,53	-1,23	-1,77
ANLN	-4,02	-1,04	2,98
ANP32C	-0,04	-0,68	-0,64
ANPEP	1,49	0,92	-0,57
ANTXR1	0,47	0,27	-0,20
ANUBL1	-1,06	-0,43	0,63
ANXA13	2,25	-1,28	-3,53
ANXA2	-0,78	1,70	2,49
ANXA4	2,04	2,27	0,22
ANXA5	-0,12	0,60	0,72
ANXA8	0,00	-9,10	-9,10
AOC3	0,44	-3,54	-3,98
AOF1	-0,44	-1,79	-1,35
AP1G1	-0,04	0,08	0,12
AP1GBP1	-0,26	0,59	0,85
AP1M1	-0,15	-0,96	-0,81
AP2A1	0,27	-0,48	-0,75
AP2M1	1,11	1,54	0,43
AP3M1	-0,25	0,48	0,73
AP3M2	0,56	2,17	1,62
AP3S2	-0,26	-0,56	-0,30
AP4E1	-0,62	0,36	0,98
AP4M1	0,04	-0,75	-0,79
APAF1	0,08	-2,38	-2,46

APBA2	-0,27	-0,72	-0,45
APBB1IP	0,91	-0,33	-1,24
APBB3	0,16	-2,14	-2,30
APCDD1	-2,43	-8,09	-5,66
APEX1	-0,26	-0,24	0,01
APEX2	0,53	1,80	1,28
APH1A	0,44	0,46	0,02
API5	0,01	0,19	0,18
APIP	0,17	0,73	0,56
APITD1	-0,75	1,13	1,88
APLP2	-1,16	-1,20	-0,04
APOB48R	-0,22	-0,66	-0,44
APOBEC3A	-0,96	-6,13	-5,17
APOBEC3B	-9,49	-2,35	7,14
APOBEC3C	-0,65	-0,49	0,16
APOBEC3G	-0,44	-1,77	-1,32
APOC1	-2,46	7,90	10,36
APOC2	-2,10	2,81	4,91
APOC4	-2,42	6,00	8,42
APOD	1,70	3,76	2,05
APOE	-1,82	11,92	13,74
APOL3	-0,52	-3,42	-2,90
APOL4	1,08	3,72	2,64
APOM	-0,14	-1,43	-1,29
APPBP1	-0,16	0,98	1,14
AQP1	-3,61	-3,01	0,60
AQP10	-0,04	0,53	0,57
AQP12A	-1,57	-1,90	-0,33
AQP7	0,23	0,35	0,12
AQP9	-0,55	1,06	1,62
AQR	0,01	-0,52	-0,53
ARAF	0,32	-0,63	-0,95
ARF1	0,34	0,07	-0,28
ARF5	0,03	-1,07	-1,10
ARF6	3,63	-0,61	-4,25
ARFGAP3	-0,68	-0,21	0,47
ARFGEF1	0,09	-0,53	-0,62
ARFIP1	-0,43	-0,77	-0,33
ARFIP2	0,61	0,57	-0,04
ARFRP1	0,85	0,16	-0,69
ARHGAP1	0,22	-0,93	-1,15
ARHGAP10	1,21	2,99	1,78
ARHGAP11A	-0,88	-0,76	0,12
ARHGAP18	0,55	3,75	3,19
ARHGAP19	-0,40	-1,93	-1,53
ARHGAP22	0,49	3,28	2,79
ARHGAP24	-2,03	-5,11	-3,08
ARHGAP25	-0,04	0,12	0,16
ARHGAP5	-1,43	-0,50	0,93
ARHGAP6	-0,22	-1,27	-1,05
ARHGAP9	0,21	-1,34	-1,54
ARHGDIA	0,24	-0,25	-0,49
ARHGDIB	-0,34	-0,24	0,10
ARHGEF1	-0,27	-2,20	-1,93
ARHGEF10L	-1,16	-1,25	-0,09
ARHGEF12	0,99	1,53	0,54
ARHGEF15	0,30	0,37	0,07
ARHGEF16	-5,73	1,42	7,14
ARHGEF19	-0,07	-1,67	-1,60

ARHGEF2	-0,26	-0,15	0,11
ARHGEF5	-0,46	1,09	1,55
ARHGEF6	-0,20	-1,83	-1,63
ARHGEF7	0,14	-0,24	-0,38
ARHGEF9	-0,19	0,42	0,61
ARID1A	0,48	-0,86	-1,34
ARID3B	-1,17	-2,00	-0,83
ARID4A	0,78	-0,21	-0,99
ARID5A	0,10	0,03	-0,08
ARID5B	-0,08	1,32	1,40
ARIH1	0,20	-0,64	-0,84
ARIH2	-0,05	-0,59	-0,54
ARL1	-0,40	0,58	0,98
ARL11	0,22	-0,90	-1,12
ARL13A	0,19	0,36	0,18
ARL14	0,16	-0,31	-0,47
ARL16	-0,05	-0,04	0,01
ARL17P1	0,05	-0,90	-0,96
ARL2BP	-0,26	-0,38	-0,12
ARL3	-0,40	-0,27	0,13
ARL4A	-2,54	-2,35	0,18
ARL5A	0,34	-0,14	-0,47
ARL6IP4	0,48	-0,28	-0,76
ARL6IP5	0,39	0,42	0,04
ARL6IP6	0,69	0,35	-0,34
ARMC1	0,09	0,41	0,32
ARMC5	0,43	-0,69	-1,11
ARMC6	0,46	-0,03	-0,49
ARMCX1	-1,38	0,41	1,79
ARMCX2	-2,13	-1,42	0,71
ARMCX3	-0,09	0,31	0,40
ARMCX5	-0,05	0,17	0,22
ARMCX6	-0,63	0,58	1,21
ARMET	-0,06	0,80	0,87
ARMETL1	-0,20	-0,15	0,04
ARNTL	0,51	0,18	-0,33
ARNTL2	-0,18	-0,33	-0,16
ARPC1A	-0,05	-0,20	-0,16
ARPC2	0,04	0,23	0,19
ARPC3	-0,02	-0,72	-0,70
ARPC4	0,03	0,13	0,10
ARPC5	0,26	0,32	0,06
ARPC5L	0,24	0,73	0,49
ARPP-19	0,01	0,23	0,22
ARPP-21	0,64	0,81	0,16
ARRDC3	-0,65	-1,03	-0,39
ARRDC4	0,99	1,54	0,55
ARSA	-0,24	0,01	0,25
ARSD	0,02	-1,12	-1,14
ARSF	-0,39	0,06	0,45
ARSG	0,55	-1,17	-1,72
ART3	-0,05	0,18	0,23
ARTS-1	-0,15	-0,19	-0,04
ARV1	0,86	2,39	1,53
ASAH1	-0,73	0,46	1,19
ASAH3	-0,11	-0,68	-0,58
ASB13	-0,16	0,03	0,19
ASB16	0,03	-0,13	-0,16
ASB17	-0,41	-0,34	0,08

ASB3	-0,26	0,04	0,30
ASB4	-0,20	-0,07	0,12
ASB7	-0,06	-1,06	-1,00
ASB8	0,07	-0,22	-0,29
ASB9	-0,42	-1,77	-1,35
ASCC1	-0,33	-0,35	-0,02
ASCC2	-0,01	-0,52	-0,51
ASCC3L1	0,36	-1,06	-1,42
ASCIZ	0,03	0,22	0,19
ASF1A	0,13	1,10	0,97
ASF1B	-3,63	-3,08	0,56
ASGR2	2,13	-4,23	-6,36
ASH1L	0,74	0,07	-0,67
ASH2L	0,25	0,23	-0,02
ASIP	1,61	5,54	3,93
ASL	0,10	-0,53	-0,62
ASNA1	0,02	0,39	0,37
ASNSD1	-0,38	-0,13	0,25
ASPH	-0,20	1,58	1,78
ASPHD1	-2,79	0,79	3,57
ASTE1	0,94	1,03	0,10
ASTN2	6,91	9,28	2,36
ASXL2	0,18	-0,72	-0,90
ATAD2	-0,21	1,68	1,89
ATAD3B	0,42	0,06	-0,36
ATAD4	0,21	0,38	0,17
ATF1	0,65	-0,05	-0,70
ATF2	0,29	0,41	0,12
ATF3	-0,79	2,87	3,65
ATF4	0,69	-0,11	-0,80
ATF5	1,56	1,94	0,38
ATF6	-0,11	0,59	0,70
ATF7IP2	0,51	0,14	-0,37
ATG12	0,35	0,04	-0,31
ATG16L2	0,23	-2,94	-3,17
ATG3	0,22	-0,64	-0,86
ATG4A	-0,96	0,63	1,59
ATG5	-0,20	-1,15	-0,96
ATG9A	0,26	1,29	1,03
ATN1	-0,56	0,90	1,46
ATOX1	-0,26	1,97	2,24
ATP10D	0,11	-0,23	-0,34
ATP11C	0,43	-0,05	-0,48
ATP13A1	0,30	0,28	-0,02
ATP1B1	-0,08	5,08	5,16
ATP1B3	-0,16	0,70	0,87
ATP1B4	-4,13	-0,53	3,61
ATP2B1	0,88	-0,92	-1,80
ATP2B3	-3,84	-1,88	1,96
ATP2C1	-0,90	0,75	1,65
ATP4A	-0,18	0,21	0,39
ATP5A1	-0,17	0,51	0,68
ATP5B	0,07	0,71	0,64
ATP5D	0,45	0,08	-0,37
ATP5F1	-0,15	0,21	0,36
ATP5G1	-0,53	0,72	1,25
ATP5G2	-0,24	-0,24	0,00
ATP5G3	-0,14	2,16	2,31
ATP5H	-0,18	0,33	0,52

ATP5I	0,07	0,16	0,09
ATP5J	-0,30	0,66	0,96
ATP5L	-0,37	0,11	0,48
ATP5O	-0,22	0,16	0,38
ATP5S	-0,54	-1,02	-0,48
ATP6AP1	-0,08	1,46	1,54
ATP6V0B	-0,25	0,43	0,67
ATP6V0C	0,14	0,25	0,12
ATP6V0D2	-3,91	0,71	4,61
ATP6V1A	-0,19	0,47	0,66
ATP6V1B2	-0,31	0,94	1,24
ATP6V1C1	-0,10	1,00	1,11
ATP6V1D	0,10	1,91	1,81
ATP6V1E1	-0,27	1,02	1,29
ATP6V1E2	-0,42	-1,42	-1,00
ATP6V1G1	0,40	0,13	-0,27
ATP6V1G2	-0,04	-0,14	-0,10
ATP6V1H	-0,60	2,12	2,72
ATP7A	-0,33	0,78	1,12
ATP7B	0,52	0,52	0,00
ATP8B3	2,31	7,61	5,30
ATP8B4	-0,55	-0,74	-0,19
ATP9B	-0,99	1,18	2,17
ATPAF1	-0,39	0,03	0,42
ATPAF2	-0,68	0,23	0,91
ATPBD1C	0,56	0,75	0,19
ATPBD3	-0,01	-0,61	-0,60
ATPBD4	-0,03	-0,21	-0,18
ATPIF1	0,00	0,33	0,32
ATR	0,00	-0,58	-0,58
ATRN	0,01	-0,41	-0,42
ATXN10	-0,49	-1,61	-1,13
ATXN2	0,49	0,00	-0,50
ATXN2L	2,27	-5,13	-7,40
ATXN3	0,43	-0,34	-0,77
ATXN7L2	-0,22	-1,21	-0,99
AUP1	0,29	-0,21	-0,50
AURKA	-0,84	2,27	3,11
AURKB	-3,10	-1,17	1,94
AVIL	-1,58	-2,52	-0,93
AVPI1	-0,30	2,50	2,80
AXL	-5,59	-3,04	2,55
AYTL1	0,09	1,26	1,18
AZI2	-0,37	-1,35	-0,98
AZIN1	-0,38	-0,38	0,00
B2M	0,05	0,22	0,17
B3GALT4	0,50	-0,04	-0,54
B3GALT5	0,05	3,30	3,25
B3GALT6	0,57	-0,07	-0,64
B3GALTL	0,66	1,78	1,11
B3GAT3	-0,19	-0,47	-0,28
B3GNT1	-0,77	1,95	2,72
B3GNT2	-1,55	0,94	2,49
B3GNT5	0,53	2,04	1,51
B3GNTL1	0,78	0,03	-0,75
B4GALT1	0,45	-0,02	-0,46
B4GALT2	0,02	2,48	2,47
B4GALT6	-5,42	-5,13	0,29
BACE1	0,27	0,79	0,52

BACH1	-0,22	-1,18	-0,96
BAG1	-0,25	-0,35	-0,10
BAG4	0,24	-0,54	-0,78
BANF1	-0,01	0,41	0,43
BANP	0,55	-0,36	-0,91
BAP1	0,74	0,56	-0,18
BARD1	-0,26	0,16	0,42
BAT1	0,13	-0,80	-0,93
BAT3	-0,01	-0,30	-0,29
BAT4	0,34	0,17	-0,16
BATF2	1,11	1,44	0,33
BAZ2A	0,28	-0,68	-0,96
BAZ2B	0,48	0,03	-0,44
BBOX1	0,79	0,32	-0,47
BBS1	0,25	-0,38	-0,63
BBS7	-0,70	-0,83	-0,13
BC37295_3	-0,10	0,11	0,21
BCAM	-0,42	0,22	0,63
BCAR3	2,01	6,67	4,66
BCAS2	-0,19	-0,32	-0,12
BCAS3	0,14	-0,71	-0,84
BCAT1	-2,74	0,18	2,91
BCCIP	-0,26	-0,60	-0,33
BCDO2	-0,19	0,17	0,35
BCHE	-1,23	1,43	2,66
BCKDHA	-0,18	-1,30	-1,13
BCKDK	0,63	0,68	0,05
BCL10	0,13	0,68	0,55
BCL2A1	-2,70	-0,99	1,72
BCL2L11	1,57	-1,22	-2,78
BCL2L12	-0,07	0,29	0,36
BCL2L14	-0,95	-0,58	0,37
BCL2L2	0,05	-0,21	-0,26
BCL6	0,70	0,24	-0,46
BCL7C	0,28	0,67	0,39
BCR	0,14	0,01	-0,13
BCS1L	0,72	0,05	-0,67
BDKRB2	-0,55	-0,08	0,46
BDNF	0,00	-0,14	-0,14
BECN1	-0,22	-0,44	-0,22
BET1L	0,17	-0,23	-0,40
BFAR	-0,13	0,46	0,59
BHLHB3	-1,75	7,81	9,56
BICD2	0,55	0,06	-0,49
BIN3	-0,61	-2,53	-1,92
BIRC2	-0,15	-0,25	-0,10
BIRC4	0,09	0,94	0,85
BIRC6	0,92	0,05	-0,88
BLCAP	-0,06	0,54	0,60
BLMH	-0,47	0,23	0,70
BLNK	0,43	3,74	3,31
BLOC1S1	-0,36	0,07	0,42
BLOC1S2	-0,31	0,56	0,87
BLR1	-0,26	-0,53	-0,27
BLZF1	-0,27	0,33	0,59
BMP2K	0,02	0,19	0,17
BMX	-0,29	-0,53	-0,24
BNIP1	-0,35	-0,45	-0,11
BNIP3	2,12	1,48	-0,64

BOLA2	-0,09	0,12	0,21
BOLA3	-0,62	2,93	3,56
BOP1	0,44	-0,94	-1,38
BPESC1	0,02	0,02	0,00
BPGM	0,12	0,75	0,63
BPNT1	0,82	1,91	1,09
BRAP	0,65	-0,33	-0,98
BRCA1	-1,04	-0,05	0,99
BRCC3	0,43	1,15	0,72
BRD1	0,99	-0,64	-1,63
BRD2	-0,08	-1,12	-1,05
BRD4	0,39	-0,78	-1,17
BRD8	0,23	-0,68	-0,91
BRD9	0,30	-0,27	-0,56
BRDG1	-3,68	1,83	5,52
BRE	-0,73	-0,47	0,26
BRF1	1,11	-0,17	-1,28
BRF2	-0,05	0,44	0,49
BRIP1	0,22	1,99	1,76
BRMS1	-0,03	-0,16	-0,13
BRP44	-1,29	0,78	2,07
BRPF1	-0,53	0,23	0,76
BRSK1	-0,19	-0,04	0,15
BRWD3	0,74	-0,41	-1,15
BSCL2	-0,65	1,09	1,74
BSDC1	-0,08	-0,31	-0,23
BSG	-0,23	0,77	1,00
BSPRY	-0,14	-0,39	-0,24
BTAF1	0,43	-0,66	-1,09
BTBD1	0,00	0,99	0,99
BTBD12	1,94	2,54	0,60
BTBD14A	-0,22	-2,85	-2,63
BTBD14B	-0,68	-0,40	0,28
BTBD15	0,46	-1,96	-2,43
BTBD3	0,25	0,03	-0,22
BTBD6	0,11	0,51	0,40
BTBD7	-0,07	-0,53	-0,47
BTD	-0,24	0,30	0,54
BTF3L4	-0,42	-0,70	-0,28
BTG2	-1,05	-1,33	-0,27
BTK	-0,17	-0,98	-0,81
BTN1A1	-1,06	-0,83	0,23
BTN2A1	-0,44	-1,23	-0,79
BTN2A3	-0,49	-0,80	-0,31
BTN3A3	-1,09	-2,50	-1,41
BTRC	-0,37	2,01	2,38
BUB1B	-8,33	1,95	10,27
BUB3	-0,06	-0,03	0,02
BUD13	-0,04	-0,18	-0,15
BUD31	0,00	0,06	0,07
BXDC1	-0,24	0,48	0,72
BXDC2	0,00	0,41	0,41
BXDC5	-0,20	-0,25	-0,05
BYSL	-0,22	0,11	0,33
BZW2	0,60	0,25	-0,35
C10ORF10	0,17	3,05	2,88
C10ORF104	0,57	-1,18	-1,75
C10ORF11	0,29	-0,22	-0,52
C10ORF118	-0,15	-0,63	-0,48

C10ORF12	-0,21	-0,23	-0,02
C10ORF129	0,71	0,03	-0,69
C10ORF130	-0,13	0,57	0,70
C10ORF22	0,26	2,78	2,52
C10ORF26	-0,51	-1,28	-0,77
C10ORF28	-0,15	-0,48	-0,33
C10ORF30	0,26	-0,01	-0,27
C10ORF35	-6,50	0,00	6,50
C10ORF55	0,06	0,30	0,23
C10ORF6	0,18	0,54	0,36
C10ORF61	-0,42	1,26	1,67
C10ORF71	-0,21	0,67	0,88
C10ORF76	0,15	0,40	0,25
C10ORF78	-0,36	-0,84	-0,49
C10ORF83	0,33	2,49	2,15
C10ORF88	0,49	1,22	0,72
C10ORF92	-0,95	3,05	4,00
C10ORF96	0,17	0,13	-0,04
C10ORF97	-0,43	-0,19	0,24
C10ORF99	0,86	-0,63	-1,49
C11ORF10	-0,02	0,15	0,17
C11ORF16	0,19	1,22	1,03
C11ORF17	-0,40	1,55	1,95
C11ORF2	0,36	-0,68	-1,03
C11ORF30	-0,25	-0,38	-0,13
C11ORF31	0,26	-0,94	-1,20
C11ORF35	3,46	-1,81	-5,28
C11ORF42	0,23	0,33	0,10
C11ORF45	-2,26	4,62	6,89
C11ORF46	0,65	-0,09	-0,74
C11ORF47	0,36	-0,63	-0,99
C11ORF51	-0,36	-1,11	-0,76
C11ORF52	-0,28	-0,39	-0,10
C11ORF54	-0,96	0,87	1,83
C11ORF57	0,05	-0,71	-0,76
C11ORF60	-0,09	1,71	1,80
C11ORF61	0,28	-0,03	-0,32
C11ORF63	-0,58	2,09	2,67
C11ORF66	0,54	-0,49	-1,03
C11ORF67	0,35	0,74	0,39
C11ORF68	0,43	0,19	-0,24
C11ORF71	-0,05	-0,30	-0,25
C11ORF74	-1,14	-0,40	0,73
C11ORF75	-0,10	0,07	0,18
C11ORF9	-0,37	-0,24	0,13
C12ORF10	-0,30	-1,17	-0,87
C12ORF11	-0,35	0,51	0,86
C12ORF24	-0,65	-1,10	-0,45
C12ORF26	-0,11	-0,59	-0,48
C12ORF28	0,04	-0,55	-0,59
C12ORF29	0,07	0,01	-0,06
C12ORF30	1,18	1,02	-0,16
C12ORF31	-0,18	-0,67	-0,50
C12ORF32	0,93	1,05	0,12
C12ORF34	0,62	0,59	-0,03
C12ORF4	0,02	1,12	1,10
C12ORF40	0,37	0,68	0,31
C12ORF41	-0,25	-0,99	-0,74
C12ORF47	-0,32	-0,58	-0,26

C12ORF49	-0,65	0,68	1,34
C12ORF5	-0,10	2,15	2,25
C12ORF52	0,49	0,49	0,00
C12ORF54	0,99	-3,45	-4,45
C12ORF57	0,27	0,26	-0,01
C12ORF60	-0,92	5,24	6,16
C12ORF61	-0,20	-0,02	0,18
C12ORF62	0,31	0,38	0,07
C13ORF18	-3,22	-3,41	-0,20
C13ORF23	0,28	-0,23	-0,51
C13ORF24	0,33	-0,20	-0,52
C13ORF26	-0,33	0,70	1,03
C13ORF3	-1,53	0,25	1,78
C14ORF1	-0,12	-0,03	0,09
C14ORF100	0,15	0,72	0,57
C14ORF101	0,45	0,00	-0,45
C14ORF102	-0,22	-0,92	-0,70
C14ORF106	0,15	-1,12	-1,27
C14ORF108	-0,01	1,06	1,07
C14ORF112	-0,26	0,15	0,41
C14ORF121	-1,87	0,13	2,00
C14ORF122	-0,12	0,07	0,19
C14ORF124	0,40	-0,47	-0,87
C14ORF126	0,01	-0,56	-0,57
C14ORF129	-0,49	1,29	1,77
C14ORF130	-0,64	-0,01	0,63
C14ORF131	-0,40	-0,68	-0,28
C14ORF133	-0,05	-0,19	-0,14
C14ORF135	-0,15	-0,25	-0,10
C14ORF139	1,34	-1,06	-2,40
C14ORF140	0,47	2,13	1,66
C14ORF142	-0,66	1,08	1,74
C14ORF145	0,60	5,55	4,94
C14ORF147	0,58	-1,22	-1,80
C14ORF148	-2,41	-4,96	-2,55
C14ORF149	1,17	1,05	-0,12
C14ORF152	-2,88	-3,70	-0,81
C14ORF153	-2,99	-8,32	-5,33
C14ORF155	-0,16	0,45	0,61
C14ORF156	-0,45	0,19	0,64
C14ORF166	0,20	-0,41	-0,61
C14ORF166B	-0,09	-0,43	-0,34
C14ORF169	-0,26	1,50	1,76
C14ORF172	0,65	0,55	-0,10
C14ORF173	0,18	2,01	1,83
C14ORF174	-0,26	-0,14	0,12
C14ORF2	0,02	-0,86	-0,88
C14ORF21	0,61	-0,13	-0,74
C14ORF28	0,60	0,17	-0,42
C14ORF4	-0,30	-0,09	0,20
C14ORF43	0,42	-1,19	-1,61
C14ORF45	-1,60	1,58	3,18
C14ORF48	-0,35	-0,29	0,06
C14ORF49	0,28	-0,46	-0,74
C14ORF68	0,64	0,43	-0,21
C14ORF79	0,15	0,14	-0,01
C15ORF17	0,56	0,82	0,26
C15ORF23	-1,97	-0,26	1,72
C15ORF24	-0,23	0,52	0,75

C15ORF29	1,06	0,08	-0,98
C15ORF39	-0,21	-3,73	-3,52
C15ORF41	-0,50	0,45	0,95
C15ORF44	-0,44	1,01	1,45
C15ORF48	-0,91	6,15	7,06
C15ORF5	0,20	-2,35	-2,55
C16ORF28	0,73	0,33	-0,40
C16ORF33	0,50	1,70	1,21
C16ORF48	0,07	-0,60	-0,67
C16ORF53	0,07	-0,39	-0,46
C16ORF58	-0,57	-0,21	0,36
C16ORF61	-0,22	0,43	0,65
C16ORF63	-0,30	-0,08	0,21
C17ORF32	-0,13	0,84	0,97
C17ORF37	-0,26	-0,17	0,08
C17ORF38	0,07	0,30	0,23
C17ORF39	-0,28	-0,61	-0,33
C17ORF45	0,16	0,04	-0,11
C17ORF48	-0,06	-1,06	-1,00
C17ORF49	0,03	-0,82	-0,84
C17ORF58	1,36	3,22	1,86
C17ORF61	-0,07	-0,40	-0,33
C17ORF62	0,44	0,05	-0,40
C17ORF64	1,38	2,11	0,72
C17ORF65	0,09	-0,88	-0,96
C17ORF68	0,42	0,64	0,22
C17ORF74	-0,32	0,12	0,43
C17ORF75	2,83	-0,39	-3,22
C17ORF77	0,10	0,61	0,51
C17ORF79	-0,28	1,96	2,24
C17ORF80	0,21	1,16	0,95
C17ORF81	0,14	-0,19	-0,33
C18ORF1	0,39	0,49	0,10
C18ORF10	-0,99	0,06	1,04
C18ORF19	0,13	0,95	0,82
C18ORF21	0,23	-0,63	-0,86
C18ORF22	0,05	0,49	0,44
C18ORF26	1,48	-2,47	-3,95
C18ORF37	-0,71	0,48	1,19
C18ORF45	0,35	1,30	0,95
C18ORF54	-1,36	-0,76	0,60
C18ORF55	-0,04	1,22	1,27
C19ORF23	0,39	0,76	0,37
C19ORF24	0,01	0,14	0,13
C19ORF30	0,86	-0,18	-1,04
C19ORF33	5,63	5,25	-0,37
C19ORF39	0,65	-1,17	-1,82
C19ORF40	-0,35	0,81	1,16
C19ORF42	-0,12	0,67	0,79
C19ORF43	0,34	-0,48	-0,82
C1D	0,77	0,11	-0,65
C1GALT1	0,28	-0,70	-0,98
C1QA	-0,89	3,21	4,09
C1QB	1,53	4,51	2,98
C1QBP	0,22	0,32	0,10
C1QC	2,51	6,22	3,71
C1QTNF6	0,21	-0,14	-0,35
C1QTNF7	-1,14	-0,45	0,69
C1QTNF8	-1,39	-0,89	0,50

C1RL	-0,48	-0,01	0,47
C1S	-0,08	2,71	2,79
C1ORF101	0,03	0,17	0,14
C1ORF102	-1,33	2,31	3,63
C1ORF103	1,82	6,39	4,57
C1ORF105	0,47	0,60	0,13
C1ORF107	0,30	0,10	-0,20
C1ORF111	0,73	0,09	-0,64
C1ORF112	-0,73	-0,09	0,64
C1ORF120	-0,81	-0,33	0,47
C1ORF121	0,03	-0,04	-0,08
C1ORF122	0,97	2,56	1,59
C1ORF123	-0,06	-1,24	-1,18
C1ORF124	0,08	0,69	0,62
C1ORF127	1,68	-2,03	-3,70
C1ORF128	-0,12	-0,20	-0,07
C1ORF130	-0,01	-0,09	-0,09
C1ORF131	0,19	0,02	-0,16
C1ORF135	-1,17	-0,50	0,67
C1ORF141	-0,17	0,28	0,45
C1ORF142	-0,19	-0,05	0,15
C1ORF144	0,01	0,79	0,77
C1ORF152	0,22	-1,27	-1,49
C1ORF156	-0,47	0,24	0,72
C1ORF160	-0,28	-0,24	0,03
C1ORF162	0,53	-0,28	-0,81
C1ORF166	-0,03	0,80	0,83
C1ORF174	0,17	0,29	0,12
C1ORF175	-1,75	0,09	1,84
C1ORF183	0,02	-0,36	-0,38
C1ORF2	0,29	-0,50	-0,79
C1ORF201	-0,38	0,81	1,19
C1ORF24	0,07	0,52	0,45
C1ORF25	0,18	1,05	0,87
C1ORF26	0,56	0,15	-0,42
C1ORF27	0,89	1,30	0,41
C1ORF31	-0,45	0,98	1,43
C1ORF35	0,68	-0,12	-0,80
C1ORF38	-0,73	-3,17	-2,45
C1ORF41	0,13	0,56	0,43
C1ORF43	0,05	0,53	0,49
C1ORF50	0,39	0,43	0,05
C1ORF51	1,33	4,37	3,04
C1ORF54	0,04	2,79	2,75
C1ORF55	-0,11	-0,38	-0,27
C1ORF57	-0,29	0,50	0,79
C1ORF58	-0,19	0,08	0,27
C1ORF66	0,00	-0,12	-0,11
C1ORF71	-0,60	0,05	0,65
C1ORF74	-0,11	1,60	1,71
C1ORF75	0,31	1,15	0,84
C1ORF77	-0,14	0,19	0,33
C1ORF83	0,37	-0,59	-0,96
C1ORF84	0,27	-0,89	-1,16
C1ORF91	0,39	-0,21	-0,60
C1ORF94	-0,26	0,27	0,53
C1ORF97	-0,60	1,09	1,69
C2	2,32	3,46	1,14
C20ORF11	0,32	-0,10	-0,42

C20ORF111	0,64	0,16	-0,48
C20ORF117	0,48	0,83	0,35
C20ORF12	-0,06	-0,19	-0,13
C20ORF127	-0,69	-0,84	-0,15
C20ORF133	0,08	0,13	0,05
C20ORF165	0,34	-0,76	-1,10
C20ORF177	0,21	-1,22	-1,43
C20ORF26	-0,66	-0,67	-0,01
C20ORF29	-0,17	0,72	0,89
C20ORF3	-0,74	0,70	1,43
C20ORF30	-0,26	-0,34	-0,08
C20ORF32	-0,08	0,86	0,93
C20ORF4	-0,04	-0,22	-0,18
C20ORF43	-0,10	-0,43	-0,33
C20ORF52	-0,01	0,26	0,27
C20ORF54	0,04	1,17	1,13
C20ORF59	-0,03	2,05	2,07
C20ORF72	0,14	0,79	0,64
C20ORF74	-0,64	-2,06	-1,42
C20ORF94	0,61	-0,24	-0,84
C21ORF119	-0,07	0,02	0,09
C21ORF123	-0,51	-0,44	0,07
C21ORF129	-0,32	-0,15	0,18
C21ORF2	-0,83	-1,02	-0,19
C21ORF33	0,34	0,76	0,42
C21ORF34	0,80	0,97	0,17
C21ORF45	-0,34	-0,11	0,23
C21ORF51	0,12	-1,56	-1,68
C21ORF55	0,35	-0,41	-0,76
C21ORF57	-0,31	0,20	0,51
C21ORF62	-0,04	0,57	0,62
C21ORF66	-0,12	-1,06	-0,95
C21ORF67	-0,25	0,18	0,43
C21ORF69	-0,52	0,36	0,88
C21ORF7	-0,54	-2,96	-2,43
C21ORF70	0,92	0,46	-0,46
C21ORF81	2,08	3,37	1,29
C21ORF91	0,18	-2,22	-2,39
C22ORF13	0,10	0,61	0,50
C22ORF15	0,06	0,53	0,47
C22ORF16	0,12	1,24	1,12
C2ORF13	0,05	0,61	0,56
C2ORF15	-0,19	-0,67	-0,48
C2ORF16	0,04	-0,58	-0,62
C2ORF21	0,24	1,44	1,19
C2ORF24	0,63	0,08	-0,55
C2ORF25	-0,16	0,45	0,61
C2ORF27	-0,41	-0,41	0,00
C2ORF28	-0,30	0,17	0,47
C2ORF29	0,34	-0,13	-0,47
C2ORF30	-0,16	1,19	1,36
C2ORF34	0,39	0,19	-0,20
C2ORF37	0,39	1,00	0,61
C2ORF7	-0,22	1,31	1,54
C3AR1	-1,74	-1,45	0,28
C3ORF1	-0,07	0,41	0,47
C3ORF18	1,64	5,65	4,01
C3ORF20	-0,33	0,22	0,55
C3ORF21	2,15	0,71	-1,44

C3ORF22	-0,36	-0,03	0,33
C3ORF23	-0,03	0,84	0,87
C3ORF26	-1,03	-0,40	0,62
C3ORF28	0,10	1,61	1,51
C3ORF31	0,61	0,68	0,07
C3ORF35	0,06	-0,37	-0,43
C3ORF37	-0,65	0,67	1,32
C3ORF38	0,16	0,34	0,18
C3ORF39	-0,67	0,11	0,78
C3ORF48	-0,57	-1,02	-0,45
C3ORF54	-0,12	-0,46	-0,35
C3ORF58	0,44	-1,10	-1,54
C3ORF59	-0,14	0,79	0,93
C3ORF60	-0,42	0,11	0,54
C3ORF62	0,05	-1,50	-1,55
C4BPA	-0,22	-0,60	-0,37
C4BPB	0,21	-0,65	-0,86
C4ORF11	2,93	4,74	1,82
C4ORF14	-0,19	-1,53	-1,35
C4ORF16	-0,19	0,33	0,52
C4ORF8	0,32	-0,68	-1,00
C5	-1,94	-1,65	0,28
C5ORF13	-1,07	-1,19	-0,12
C5ORF14	-0,25	-0,28	-0,03
C5ORF15	0,32	-0,08	-0,40
C5ORF20	1,06	1,06	0,01
C5ORF22	-0,03	0,17	0,20
C5ORF24	0,41	-0,63	-1,04
C5ORF3	0,03	-0,26	-0,29
C5ORF4	-1,42	-0,41	1,01
C5ORF5	0,41	0,89	0,48
C6ORF105	-1,73	5,58	7,31
C6ORF113	0,07	0,10	0,03
C6ORF114	0,68	1,19	0,51
C6ORF120	0,27	-0,62	-0,90
C6ORF128	-0,73	5,89	6,62
C6ORF129	-0,72	1,39	2,12
C6ORF130	-0,36	-0,13	0,23
C6ORF134	-0,04	-0,45	-0,41
C6ORF136	0,37	0,18	-0,19
C6ORF145	1,19	2,11	0,92
C6ORF153	0,04	-0,05	-0,08
C6ORF157	-0,16	0,13	0,29
C6ORF162	0,25	0,84	0,59
C6ORF163	-0,15	-0,27	-0,12
C6ORF173	-1,10	0,02	1,12
C6ORF182	0,18	1,00	0,82
C6ORF199	-0,05	0,25	0,29
C6ORF203	0,51	1,12	0,61
C6ORF211	-0,47	0,72	1,19
C6ORF218	0,26	-0,32	-0,58
C6ORF25	-0,19	-0,22	-0,02
C6ORF27	-0,89	-0,45	0,44
C6ORF32	-3,97	-10,76	-6,78
C6ORF47	0,07	-0,58	-0,66
C6ORF49	0,08	0,19	0,11
C6ORF52	0,37	0,42	0,05
C6ORF59	1,53	-0,28	-1,81
C6ORF61	0,14	-0,26	-0,40

C6ORF62	0,53	0,63	0,10
C6ORF66	-0,11	1,85	1,96
C6ORF70	-0,18	-0,15	0,04
C6ORF72	0,08	-0,07	-0,15
C6ORF85	4,75	4,17	-0,58
C6ORF89	-0,09	1,23	1,32
C7ORF10	0,34	2,84	2,50
C7ORF11	0,04	-0,23	-0,27
C7ORF13	-0,06	-0,71	-0,66
C7ORF20	0,36	0,32	-0,04
C7ORF23	-0,34	0,64	0,98
C7ORF25	0,08	1,65	1,57
C7ORF26	-0,03	-1,07	-1,04
C7ORF29	0,75	1,18	0,43
C7ORF38	0,46	-1,49	-1,95
C8B	-1,35	-1,21	0,14
C8ORF30A	0,13	0,59	0,46
C8ORF32	-0,07	0,47	0,54
C8ORF33	-0,18	0,80	0,98
C8ORF34	0,35	0,67	0,33
C8ORF38	-0,53	1,91	2,44
C8ORF40	-0,29	0,39	0,68
C8ORF41	-0,06	0,42	0,48
C8ORF44	0,70	-0,10	-0,81
C8ORF46	1,71	1,77	0,06
C8ORF51	0,67	0,77	0,10
C8ORF53	-0,05	-0,55	-0,50
C8ORF54	-0,08	1,16	1,24
C8ORF58	0,82	0,45	-0,37
C8ORF70	-0,78	-0,16	0,63
C8ORF74	-0,33	-0,27	0,06
C8ORF76	0,25	0,68	0,43
C9ORF100	-0,19	0,50	0,69
C9ORF103	-0,86	-0,01	0,86
C9ORF116	-0,49	0,05	0,54
C9ORF119	-0,48	0,37	0,85
C9ORF130	0,44	-1,05	-1,49
C9ORF140	0,03	-1,26	-1,29
C9ORF142	0,31	-1,14	-1,46
C9ORF164	1,20	-2,65	-3,85
C9ORF18	-0,53	-0,56	-0,03
C9ORF21	0,09	-0,23	-0,32
C9ORF23	0,17	0,02	-0,15
C9ORF24	0,96	1,08	0,12
C9ORF25	0,23	0,08	-0,16
C9ORF3	0,30	0,13	-0,17
C9ORF30	-0,02	3,83	3,84
C9ORF37	0,34	-0,07	-0,41
C9ORF40	-0,35	1,62	1,97
C9ORF45	0,63	1,33	0,70
C9ORF46	-0,36	2,10	2,45
C9ORF5	0,32	0,63	0,31
C9ORF6	-1,07	-1,00	0,07
C9ORF64	0,00	0,30	0,30
C9ORF72	0,15	-2,50	-2,65
C9ORF78	0,13	-0,57	-0,71
C9ORF79	0,50	-0,14	-0,64
C9ORF80	0,02	0,01	-0,01
C9ORF85	0,22	-0,39	-0,61

C9ORF9	1,55	1,68	0,14
C9ORF90	0,28	-0,05	-0,33
C9ORF93	-0,39	1,43	1,82
C9ORF95	-0,42	0,52	0,94
C9ORF97	-0,19	0,14	0,33
C9ORF98	-0,19	9,89	10,08
CA1	-0,04	0,66	0,70
CA2	-1,37	3,37	4,73
CA5A	2,92	5,67	2,76
CA5B	0,04	-3,02	-3,06
CAB39L	-0,93	1,60	2,53
CABLES1	0,94	5,93	4,99
CABYR	-0,12	0,12	0,24
CACNA1I	-0,27	-0,05	0,22
CACNB1	0,30	-1,03	-1,33
CACNB4	2,02	0,85	-1,17
CACNG3	0,08	-2,91	-3,00
CACNG7	0,73	-0,31	-1,04
CACYBP	0,00	0,77	0,77
CAD	0,17	1,50	1,33
CAGE1	0,01	0,16	0,15
CALCOCO1	-0,37	-0,44	-0,07
CALCRL	4,46	4,02	-0,44
CALD1	0,49	0,61	0,12
CALM1	0,16	0,86	0,70
CALM2	0,50	0,74	0,25
CALN1	-0,33	-0,23	0,10
CALR	-0,56	1,09	1,65
CAMK1D	2,21	1,26	-0,95
CAMK1G	0,40	2,10	1,69
CAMKK1	0,87	-1,27	-2,14
CAMP	-3,76	-0,35	3,41
CAMSAP1	-0,55	0,24	0,79
CAMTA2	0,34	0,49	0,15
CAND1	0,11	0,37	0,27
CANT1	-0,29	-0,54	-0,25
CAP1	0,25	0,26	0,00
CAPG	-0,58	1,47	2,06
CAPN10	-0,06	-0,85	-0,79
CAPN11	-1,01	2,08	3,08
CAPN2	-0,20	-2,25	-2,05
CAPN3	0,96	-0,05	-1,01
CAPN5	-2,31	0,03	2,34
CAPN6	-0,01	0,02	0,03
CAPNS2	2,33	1,86	-0,47
CAPS	-0,38	-1,29	-0,91
CAPSL	0,11	0,91	0,80
CAPZA2	0,01	-0,40	-0,41
CAPZB	0,05	-0,21	-0,25
CARD14	0,94	1,93	0,98
CARD6	-0,25	0,65	0,90
CARD8	-0,10	-1,08	-0,98
CARD9	2,28	0,40	-1,88
CARHSP1	-0,65	-0,51	0,15
CARM1	0,69	0,79	0,10
CASC3	0,36	-0,78	-1,15
CASC4	0,06	-0,43	-0,49
CASC5	-2,36	-0,83	1,54
CASD1	-0,38	-0,48	-0,10

CASK	-0,50	-0,52	-0,02
CASP10	-0,16	-0,51	-0,35
CASP2	0,65	-0,18	-0,83
CASP3	1,35	1,92	0,57
CASP5	-0,57	-3,00	-2,43
CASP8	-0,41	-2,50	-2,09
CAST	0,26	-1,01	-1,26
CASZ1	2,02	0,58	-1,43
CATSPER1	-0,15	-1,21	-1,06
CATSPER3	0,53	0,85	0,32
CAV3	0,36	-0,15	-0,52
CBARA1	-0,97	-1,31	-0,34
CBFA2T2	0,37	-0,24	-0,61
CBFA2T3	1,11	-1,95	-3,06
CBFB	0,17	0,01	-0,16
CBLB	-0,11	1,43	1,54
CBLC	-0,08	0,28	0,36
CBLL1	0,17	-1,52	-1,69
CBR4	-0,21	-0,19	0,03
CBX1	-0,02	-0,86	-0,85
CBX2	0,37	0,42	0,05
CBX5	-0,26	0,09	0,35
CC2D1A	0,22	-0,06	-0,28
CCBL1	-0,32	0,60	0,92
CCDC100	0,52	0,17	-0,35
CCDC101	0,27	-0,92	-1,19
CCDC102B	-0,57	3,95	4,52
CCDC12	-0,11	-0,31	-0,20
CCDC14	-0,64	-0,87	-0,22
CCDC15	-0,56	0,59	1,15
CCDC16	-0,03	0,37	0,40
CCDC17	-0,28	-0,16	0,11
CCDC18	-0,40	-0,34	0,06
CCDC19	-0,39	-1,46	-1,08
CCDC21	0,32	-0,04	-0,36
CCDC22	-0,32	-0,43	-0,11
CCDC23	-0,05	-1,36	-1,31
CCDC26	6,38	1,78	-4,59
CCDC3	0,45	0,23	-0,22
CCDC33	-0,15	0,42	0,57
CCDC34	-1,56	1,30	2,86
CCDC43	0,12	0,29	0,17
CCDC44	-0,20	1,11	1,32
CCDC45	0,68	-1,16	-1,84
CCDC46	3,50	6,40	2,90
CCDC47	0,41	0,89	0,48
CCDC48	0,22	0,28	0,06
CCDC51	-0,10	1,30	1,40
CCDC53	-0,02	0,02	0,04
CCDC55	-0,02	0,05	0,07
CCDC56	-0,15	-0,05	0,09
CCDC58	-0,79	0,02	0,81
CCDC59	0,08	-1,51	-1,59
CCDC6	1,11	1,01	-0,10
CCDC60	-1,20	-0,84	0,37
CCDC66	0,76	0,38	-0,38
CCDC71	0,17	0,00	-0,16
CCDC72	-0,07	-0,41	-0,34
CCDC76	0,54	-0,24	-0,78

CCDC77	-0,80	-0,81	-0,01
CCDC81	0,62	0,87	0,25
CCDC82	0,21	0,07	-0,14
CCDC84	0,47	-0,92	-1,39
CCDC85B	-0,08	-0,18	-0,11
CCDC86	-0,26	-0,37	-0,11
CCDC9	0,08	-1,55	-1,63
CCDC92	0,47	1,89	1,41
CCDC93	0,49	1,78	1,29
CCDC95	-0,28	-1,04	-0,75
CCDC97	-0,03	-0,50	-0,47
CCHCR1	-0,65	-0,92	-0,27
CCL1	-4,05	-3,55	0,49
CCL13	6,42	12,58	6,17
CCL15	2,11	3,09	0,98
CCL16	0,04	-0,52	-0,56
CCL17	8,42	17,61	9,20
CCL18	3,12	11,07	7,95
CCL19	2,15	0,69	-1,46
CCL2	0,20	6,63	6,43
CCL20	-2,97	0,98	3,96
CCL22	1,02	9,80	8,78
CCL23	2,48	10,09	7,62
CCL24	-0,78	6,25	7,02
CCL26	7,42	7,07	-0,35
CCL27	0,27	-0,05	-0,32
CCL3	-0,94	5,65	6,59
CCL3L1	-1,09	4,88	5,97
CCL5	-0,72	1,35	2,07
CCL7	-0,74	0,07	0,81
CCM2	0,07	-0,23	-0,30
CCNB2	-2,38	3,62	6,00
CCNDBP1	0,24	-0,24	-0,48
CCNF	-2,17	3,11	5,27
CCNG2	2,05	0,61	-1,44
CCNH	1,75	2,07	0,31
CCNJ	0,69	-0,79	-1,48
CCNK	0,28	-0,28	-0,56
CCNL1	-0,27	-1,89	-1,62
CCR1	0,83	2,56	1,73
CCR2	2,46	-8,24	-10,71
CCR7	11,83	11,83	0,00
CCR9	-0,42	-0,47	-0,05
CCS	0,02	-0,96	-0,98
CCT2	-0,22	0,11	0,33
CCT3	-0,11	-0,04	0,07
CCT4	0,15	0,31	0,16
CCT5	0,06	-0,06	-0,12
CCT6A	0,34	0,56	0,21
CCT6B	-0,26	0,16	0,42
CCT7	0,01	0,38	0,37
CCT8	0,09	0,00	-0,09
CD14	-5,34	-6,79	-1,44
CD151	-0,61	1,37	1,98
CD163	-5,29	-6,24	-0,95
CD163L1	-0,01	0,42	0,43
CD1A	5,76	5,20	-0,56
CD1B	4,22	8,38	4,16
CD1C	5,66	3,80	-1,86

CD1E	3,25	4,38	1,13
CD200	-0,24	0,21	0,45
CD200R1	5,66	5,92	0,26
CD207	0,46	0,49	0,03
CD209	3,35	7,43	4,08
CD22	-0,03	1,77	1,80
CD247	0,56	0,23	-0,33
CD274	1,09	6,57	5,48
CD2BP2	0,12	0,86	0,74
CD300E	-0,64	-2,70	-2,06
CD300LF	-1,05	-1,18	-0,13
CD300LG	0,27	1,63	1,37
CD33	-0,35	-1,70	-1,35
CD36	-1,05	-2,75	-1,70
CD37	-1,93	-3,50	-1,57
CD3EAP	4,47	0,68	-3,78
CD44	0,08	0,23	0,15
CD46	-0,13	-0,30	-0,17
CD48	-1,00	-2,20	-1,20
CD52	-0,36	0,31	0,67
CD53	0,12	0,23	0,11
CD6	-0,80	7,49	8,29
CD63	-0,69	2,12	2,81
CD68	0,19	0,62	0,43
CD69	-0,94	-1,06	-0,12
CD72	-2,75	-2,41	0,34
CD74	0,19	0,40	0,21
CD79A	-0,73	-1,41	-0,68
CD80	0,83	3,03	2,21
CD82	-1,61	0,95	2,56
CD83	0,52	3,31	2,78
CD84	-0,63	2,61	3,23
CD86	1,03	0,71	-0,32
CD9	-0,88	4,18	5,07
CD97	-0,93	-2,06	-1,13
CD99	0,16	-0,11	-0,27
CDA	-1,13	-1,72	-0,58
CDC16	0,09	0,06	-0,03
CDC2	-11,15	0,00	11,15
CDC25A	-1,83	-0,76	1,07
CDC25B	0,12	0,51	0,39
CDC25C	-7,64	-2,91	4,73
CDC26	-0,25	-0,17	0,09
CDC2L1	0,31	-0,77	-1,08
CDC2L2	0,25	-0,77	-1,01
CDC2L6	0,20	-0,53	-0,73
CDC37L1	0,57	1,56	0,99
CDC42	-0,05	-0,52	-0,47
CDC42BPB	0,52	1,68	1,16
CDC42SE2	0,50	0,46	-0,05
CDC45L	-8,04	-1,83	6,21
CDC5L	0,23	0,61	0,38
CDC7	-1,05	-1,56	-0,51
CDC73	-0,30	0,34	0,65
CDCA2	-3,33	2,44	5,77
CDCA3	-4,59	3,26	7,85
CDCA4	-0,23	-0,15	0,08
CDCA5	-2,77	1,00	3,77
CDCA8	-2,62	5,13	7,75

CDH15	-0,02	0,26	0,28
CDH17	-0,23	0,21	0,44
CDH2	4,63	4,57	-0,06
CDH26	-0,71	-1,44	-0,73
CDH7	-0,08	-0,32	-0,24
CDH9	-0,16	-0,46	-0,29
CDK10	0,35	-1,36	-1,71
CDK2	-0,23	-0,12	0,11
CDK2AP2	0,22	0,10	-0,12
CDK4	0,07	1,74	1,67
CDK5RAP1	0,16	-0,06	-0,22
CDK5RAP2	-1,70	-0,20	1,50
CDK5RAP3	0,73	-0,29	-1,02
CDK7	-0,12	0,41	0,53
CDK8	-0,09	-0,28	-0,19
CDKAL1	0,41	0,39	-0,01
CDKL3	-0,84	1,36	2,20
CDKL4	-0,84	-0,92	-0,09
CDKN1A	0,98	2,42	1,44
CDKN1B	0,86	-1,79	-2,64
CDKN2D	-1,17	-5,54	-4,37
CDR2	1,98	3,12	1,14
CDRT4	-1,14	0,22	1,36
CDS2	0,79	1,73	0,94
CDT1	-3,09	0,12	3,22
CDV3	0,00	-0,81	-0,81
CDYL	0,11	0,64	0,53
CDYL2	3,25	0,79	-2,47
CEACAM16	1,17	-0,09	-1,27
CEACAM19	0,22	-0,55	-0,77
CEACAM8	-3,55	5,38	8,93
CEBPA	0,61	1,25	0,64
CEBPE	-0,98	-1,11	-0,14
CEBPZ	0,41	-0,06	-0,47
CECR1	0,13	-0,35	-0,48
CECR5	-0,64	-0,57	0,08
CELSR1	-2,08	2,19	4,26
CELSR3	1,27	-0,26	-1,53
CENPB	0,08	-0,43	-0,51
CENPC1	-0,23	-0,32	-0,09
CENPE	-1,88	0,55	2,43
CENPF	-8,22	-2,18	6,04
CENPH	-0,39	1,99	2,38
CENTA1	0,99	-1,65	-2,64
CENTB1	0,21	-1,68	-1,88
CENTB2	-0,58	-1,52	-0,93
CENTD2	-0,24	-0,72	-0,47
CENTD3	-2,21	-2,25	-0,04
CENTG1	-0,53	0,53	1,06
CEP110	-0,85	-2,36	-1,51
CEP152	-0,95	-1,73	-0,78
CEP164	0,58	-0,02	-0,60
CEP192	0,03	-1,07	-1,10
CEP250	0,79	0,93	0,14
CEP27	-0,08	-1,33	-1,25
CEP290	-0,70	-1,01	-0,30
CEP350	0,81	-0,67	-1,48
CEP55	-5,12	6,20	11,32
CEP57	0,44	-0,48	-0,91

CEP63	0,03	-0,48	-0,51
CEP68	1,06	1,27	0,21
CEP70	-2,90	1,35	4,25
CEP76	-0,11	0,59	0,69
CEPT1	0,14	0,27	0,12
CER1	3,29	-0,54	-3,82
CERK	1,65	0,12	-1,53
CERKL	-2,43	-4,30	-1,87
CES1	-4,80	-2,12	2,69
CES2	-0,02	-0,50	-0,48
CES7	-0,77	0,36	1,13
CETN2	-0,86	-0,09	0,77
CETN3	0,37	2,18	1,80
CETP	-1,01	-3,97	-2,96
CFB	1,03	7,59	6,56
CFH	-0,62	0,34	0,96
CFI	1,65	1,44	-0,21
CFL1	0,06	0,24	0,18
CFL2	0,30	0,13	-0,17
CFP	1,96	-5,07	-7,03
CGGBP1	0,24	-0,54	-0,78
CGI-09	0,43	-0,25	-0,68
CH25H	3,50	9,18	5,67
CHAC2	-0,26	1,36	1,62
CHAF1A	-0,97	-0,90	0,06
CHAF1B	-2,66	3,12	5,78
CHCHD1	0,22	0,90	0,67
CHCHD3	0,35	0,42	0,07
CHCHD4	0,48	-0,14	-0,63
CHCHD5	-0,25	-0,27	-0,03
CHCHD7	1,95	1,29	-0,66
CHCHD8	-0,02	-0,10	-0,09
CHD3	0,03	-0,78	-0,81
CHD4	-0,15	-0,35	-0,19
CHD7	1,89	1,89	0,00
CHD8	0,37	-0,46	-0,83
CHD9	-0,26	0,54	0,80
CHDH	-0,25	6,48	6,73
CHEK1	-1,26	7,14	8,39
CHEK2	-0,08	0,56	0,65
CHERP	0,82	-0,68	-1,50
CHI3L1	-2,05	-0,62	1,43
CHIC2	0,38	0,38	0,00
CHIT1	-4,91	1,12	6,03
CHM	-0,31	0,52	0,83
CHML	1,50	-3,74	-5,24
CHMP2A	-0,34	-0,21	0,13
CHMP4A	0,52	0,26	-0,26
CHMP4B	-0,15	-0,55	-0,40
CHMP5	0,13	0,95	0,83
CHMP7	0,00	0,01	0,02
CHN2	0,10	-1,29	-1,39
CHORDC1	0,41	0,23	-0,18
CHP	0,41	-0,15	-0,55
CHPT1	-1,35	-1,89	-0,55
CHRAC1	0,07	-1,47	-1,54
CHRNA10	0,73	0,79	0,05
CHRNA4	-0,47	0,48	0,95
CHRNB1	0,14	-1,06	-1,20

CHRNB3	0,24	0,34	0,10
CHRNE	-0,29	0,94	1,23
CHST10	0,02	1,24	1,22
CHST12	0,14	0,41	0,28
CHST7	1,38	0,98	-0,40
CHST8	0,25	0,62	0,36
CHSY1	-0,13	-0,94	-0,81
CHTF18	-0,93	-1,87	-0,94
CHURC1	-0,11	-0,38	-0,27
CIAPIN1	0,28	0,82	0,54
CIB1	0,08	0,03	-0,05
CIB3	-0,37	-1,03	-0,66
CIC	0,71	-0,51	-1,22
CIDEB	0,76	-3,64	-4,40
CIDEC	-0,65	0,41	1,05
CINP	-0,17	0,11	0,28
CIP29	0,09	0,10	0,02
CIR	-1,97	0,80	2,77
CIRBP	0,76	-0,07	-0,82
CIRH1A	0,14	0,44	0,30
CISH	1,45	3,63	2,19
CIZ1	0,06	-0,70	-0,76
CKAP2	-0,27	0,18	0,45
CKAP4	-3,22	-3,97	-0,75
CKAP5	-0,16	0,97	1,13
CKLF	-0,39	-0,14	0,25
CKS1B	-0,86	1,65	2,51
CKS2	-0,35	1,87	2,22
CLASP2	0,15	0,41	0,26
CLC	4,83	7,55	2,72
CLCN1	0,36	0,08	-0,28
CLCN3	-0,49	1,36	1,85
CLCN5	0,21	0,72	0,51
CLCN6	0,37	-0,25	-0,62
CLDN1	9,21	9,21	0,00
CLDN14	-0,06	-0,41	-0,35
CLDN7	-2,75	-0,98	1,77
CLDN8	0,09	0,39	0,30
CLDND1	-0,12	-0,56	-0,45
CLEC12B	-2,60	-4,15	-1,55
CLEC1A	-0,05	0,21	0,26
CLEC1B	-7,43	-4,36	3,07
CLEC2D	0,18	0,21	0,03
CLEC3B	-0,40	-1,65	-1,25
CLEC4A	1,17	0,74	-0,43
CLEC4D	-2,66	-4,99	-2,33
CLEC5A	-5,52	-3,67	1,85
CLEC6A	-2,81	-0,42	2,39
CLEC9A	0,52	-0,32	-0,84
CLIC1	-0,34	-0,53	-0,18
CLIC2	1,86	3,23	1,37
CLIC3	-0,11	6,73	6,84
CLIC4	-0,39	1,49	1,89
CLK2	0,55	-0,63	-1,18
CLK3	0,41	-1,07	-1,48
CLK4	0,45	-1,00	-1,45
CLN3	-0,41	-0,16	0,24
CLN5	-0,40	1,34	1,74
CLN6	-0,40	-0,02	0,38

CLN8	-0,40	1,03	1,43
CLOCK	-0,07	-0,47	-0,40
CLPTM1	-0,46	0,39	0,84
CLTC	0,00	0,52	0,52
CLTCL1	0,72	-1,52	-2,25
CLUAP1	0,42	1,18	0,76
CLUL1	-0,15	-0,01	0,15
CLYBL	0,26	2,88	2,62
CMAS	-1,27	0,34	1,61
CMIP	0,47	-0,97	-1,45
CMPK	0,08	-0,20	-0,28
CMTM1	0,75	-0,35	-1,10
CMTM5	-0,20	-0,85	-0,65
CMTM6	0,14	0,05	-0,09
CMTM7	0,62	0,30	-0,32
CMYA5	-0,22	1,97	2,19
CNFN	-0,96	-6,10	-5,14
CNGA1	1,16	7,69	6,53
CNNM2	1,02	-1,34	-2,36
CNNM3	0,31	-0,74	-1,06
CNNM4	-0,57	0,53	1,10
CNO	0,48	0,34	-0,14
CNOT1	0,44	-0,45	-0,89
CNOT10	0,41	-0,27	-0,68
CNOT3	0,59	-0,35	-0,94
CNOT7	0,01	-0,29	-0,29
CNOT8	0,12	-1,39	-1,51
CNP	-2,06	-4,73	-2,66
CNTNAP5	0,79	0,37	-0,42
CNTROB	-0,30	-0,19	0,11
COASY	-0,08	-0,06	0,02
COG3	-0,02	-0,53	-0,51
COG4	-0,11	-0,54	-0,43
COG5	-0,67	-0,08	0,58
COG7	0,54	0,25	-0,30
COG8	0,27	0,90	0,63
COL11A1	-0,69	-0,72	-0,03
COL24A1	-2,94	0,13	3,07
COL4A3BP	-0,92	0,16	1,08
COL6A3	-0,45	6,10	6,55
COL7A1	0,67	0,10	-0,57
COL9A1	-0,57	0,16	0,74
COLQ	-1,72	-1,06	0,65
COMMD10	-0,12	-0,10	0,03
COMMD3	0,10	0,18	0,08
COMMD9	-0,70	-0,77	-0,07
COMT	-0,53	0,23	0,76
COMTD1	0,69	0,25	-0,44
COPA	-0,02	-0,08	-0,07
COPB2	-0,18	0,49	0,68
COPE	-0,27	-0,39	-0,13
COPG	-0,54	0,29	0,83
COPS2	0,38	-0,38	-0,77
COPS5	-0,20	-0,18	0,01
COPS6	-0,66	-0,69	-0,03
COPS7A	-0,21	0,12	0,33
COPS7B	0,65	0,35	-0,30
COPS8	-0,24	0,37	0,61
COPZ1	-0,70	-0,27	0,43

COQ10A	0,15	0,22	0,07
COQ10B	-0,18	0,46	0,65
COQ2	-1,47	-1,58	-0,10
COQ3	-1,12	0,78	1,90
COQ4	0,14	-0,03	-0,17
COQ5	-0,02	0,50	0,53
COQ9	-0,03	0,63	0,66
CORO1B	0,48	0,98	0,50
CORO1C	-0,61	0,32	0,93
CORO2A	-0,19	1,49	1,68
CORO7	0,19	-0,37	-0,56
CORT	-0,11	-0,41	-0,30
COX10	-0,03	0,09	0,12
COX11	-0,27	0,84	1,11
COX15	-0,14	0,58	0,73
COX17	-0,30	0,76	1,06
COX4I1	-0,10	0,32	0,42
COX4NB	0,15	-0,51	-0,67
COX5B	-0,59	0,92	1,51
COX6A1	-0,11	0,64	0,75
COX6B1	-0,21	0,08	0,29
COX6C	-0,39	0,30	0,69
COX7A2	-0,27	0,79	1,06
COX7B	0,07	0,63	0,56
COX7C	-0,01	0,38	0,39
CPB1	-0,06	0,68	0,74
CPD	-1,68	-1,38	0,30
CPEB1	1,98	8,50	6,52
CPEB3	0,85	-1,40	-2,26
CPEB4	-0,35	0,42	0,77
CPLX2	0,16	-0,29	-0,45
CPLX3	-1,09	-1,32	-0,23
CPNE1	0,98	-0,32	-1,31
CPNE2	-0,11	-1,05	-0,94
CPNE3	0,16	-0,63	-0,78
CPNE6	-3,53	0,02	3,55
CPOX	-0,44	0,20	0,65
CPSF1	0,17	0,11	-0,06
CPSF2	0,21	-0,35	-0,57
CPSF3	-0,21	0,48	0,69
CPSF3L	0,47	-0,79	-1,26
CPSF4	0,11	0,47	0,36
CPT1A	0,95	-0,92	-1,87
CPT2	-0,15	1,81	1,95
CPXM2	-1,27	-0,80	0,47
CPZ	-0,26	0,21	0,47
CRADD	-0,85	0,15	0,99
CRAT	-0,06	-0,12	-0,06
CRB2	0,55	0,02	-0,53
CREB1	-0,22	-0,39	-0,17
CREB3	0,29	0,83	0,54
CREB3L3	0,73	0,51	-0,22
CREB3L4	1,16	-0,14	-1,30
CREBL1	0,19	-0,67	-0,86
CREG1	-0,01	1,36	1,37
CRELD2	-0,39	-0,19	0,20
CREM	0,27	0,17	-0,10
CRHBP	-0,49	1,03	1,52
CRIM1	-1,31	0,88	2,19

CRIP3	-0,14	-0,18	-0,04
CRIPT	-0,90	0,13	1,03
CRISP3	-0,37	-1,10	-0,72
CRISPLD2	-0,18	-6,46	-6,28
CRKRS	0,31	-0,58	-0,89
CRLF1	-0,25	-0,68	-0,43
CRLF3	-0,08	-1,62	-1,54
CRLS1	-0,68	-0,32	0,36
CRNKL1	-0,09	0,76	0,85
CROCC	0,11	-1,40	-1,50
CROP	-0,07	-2,37	-2,30
CROT	-0,54	0,50	1,04
CRSP2	0,48	0,15	-0,34
CRSP3	0,12	-0,59	-0,70
CRSP6	0,12	9,89	9,77
CRSP8	-0,25	0,38	0,62
CRTAM	-1,55	0,18	1,74
CRTAP	-0,35	-2,06	-1,71
CRTC2	0,46	-1,48	-1,93
CRX	-0,45	-0,75	-0,30
CRY1	-0,11	0,25	0,36
CRY2	0,82	-0,10	-0,92
CRYAA	0,44	0,15	-0,29
CRYBB2	-0,43	-0,47	-0,04
CRYBB3	-0,43	-1,10	-0,67
CRYGS	0,74	-0,74	-1,47
CRYZ	1,59	2,15	0,56
CS	0,20	0,13	-0,07
CSAD	-0,62	-1,22	-0,60
CSDA	-0,17	-0,80	-0,63
CSF1	-1,86	9,25	11,12
CSF2RA	0,61	-0,74	-1,35
CSF2RB	0,46	0,92	0,46
CSGLCA-T	0,25	-0,15	-0,40
CSHL1	-0,96	-1,12	-0,16
CSK	0,71	-0,81	-1,52
CSMD1	-0,15	-0,25	-0,10
CSNK1A1	-0,19	-0,66	-0,47
CSNK1A1L	0,08	-0,05	-0,13
CSNK1G1	0,29	0,23	-0,06
CSNK2A1	0,18	0,73	0,56
CSNK2A2	-0,17	0,71	0,88
CSNK2B	0,23	0,52	0,29
CSPG5	-0,67	-0,31	0,36
CSPP1	0,36	-0,97	-1,33
CSRP1	-0,22	0,84	1,06
CSRP2	-4,38	4,84	9,22
CST2	1,49	1,03	-0,46
CST6	-1,39	1,24	2,63
CSTA	-1,28	-3,07	-1,79
CSTB	0,07	2,53	2,46
CSTF1	0,09	0,62	0,53
CSTF2	0,00	0,52	0,52
CSTF2T	0,42	0,34	-0,08
CSTF3	0,15	0,78	0,63
CT45-1	-0,74	-0,32	0,42
CTAGE5	-0,21	-1,15	-0,94
CTBP1	0,55	-0,40	-0,96
CTBP2	-0,07	-1,35	-1,28

CTBS	-0,76	-1,11	-0,35
CTCF	0,23	-0,16	-0,39
CTDP1	0,86	-0,74	-1,60
CTDSP2	-0,64	-1,78	-1,14
CTDSPL2	0,33	-0,20	-0,53
CTLA4	3,56	3,39	-0,17
CTNNA1	0,48	0,52	0,04
CTNNAL1	2,47	10,14	7,67
CTNNB1	0,62	0,46	-0,16
CTNS	0,65	2,97	2,32
CTPS	1,28	1,76	0,48
CTPS2	-0,17	-0,45	-0,28
CTRB2	0,13	0,78	0,64
CTRC	0,00	-6,85	-6,85
CTRL	-0,57	-3,31	-2,74
CTSB	-0,12	1,64	1,75
CTSC	1,60	3,01	1,41
CTSD	-0,42	1,55	1,97
CTSK	-4,05	-0,70	3,35
CTSO	0,20	-0,87	-1,07
CTSW	-0,69	1,93	2,63
CTSZ	0,69	2,50	1,81
CTTN	-0,43	4,75	5,19
CTTNBP2NL	0,65	2,00	1,34
CTXN1	1,01	1,21	0,21
CUEDC1	0,62	0,63	0,02
CUGBP1	-0,41	-0,30	0,11
CUGBP2	0,71	-1,55	-2,26
CUL2	-0,58	0,78	1,36
CUL4A	-0,04	-0,29	-0,25
CUL4B	-0,46	-1,36	-0,89
CUL7	-0,30	0,03	0,33
CUTA	-0,35	0,25	0,59
CUTC	0,01	-0,80	-0,81
CUZD1	2,03	-0,42	-2,46
CWF19L1	-0,14	-0,76	-0,62
CWF19L2	0,16	-0,43	-0,59
CX3CL1	3,43	7,01	3,58
CX3CR1	0,63	-4,81	-5,44
CXCL1	-2,97	-0,96	2,01
CXCL10	-1,00	-4,14	-3,15
CXCL11	-0,68	-1,75	-1,07
CXCL2	-4,98	0,59	5,57
CXCL3	-0,32	-0,35	-0,03
CXCL9	0,00	-8,02	-8,02
CXCR4	0,94	-0,11	-1,05
CXXC1	0,33	0,13	-0,20
CXXC5	0,07	1,33	1,25
CXORF23	0,44	-0,14	-0,58
CXORF26	-0,28	1,53	1,81
CXORF34	0,08	-0,99	-1,07
CXORF38	-0,16	-0,96	-0,80
CXORF39	0,84	1,63	0,78
CXORF40A	0,08	1,24	1,15
CXORF45	-0,24	-1,60	-1,36
CXORF48	0,15	0,89	0,74
CXORF6	-2,47	4,99	7,46
CXORF9	0,97	0,23	-0,74
CYB561	-0,03	-0,40	-0,37

CYB561D1	0,11	0,16	0,04
CYB561D2	-0,05	-0,40	-0,35
CYB5B	-0,08	0,80	0,87
CYB5D1	-0,23	0,45	0,68
CYB5D2	-0,31	0,40	0,71
CYB5R1	-0,24	1,13	1,37
CYBASC3	-1,06	0,51	1,57
CYFIP1	0,01	0,96	0,94
CYFIP2	1,01	-1,46	-2,47
CYP11A1	0,37	-0,39	-0,76
CYP19A1	-0,72	1,00	1,72
CYP1B1	-0,27	1,50	1,76
CYP20A1	0,45	0,43	-0,02
CYP27A1	-1,67	1,01	2,68
CYP27B1	-1,32	6,97	8,29
CYP2A13	-0,10	-0,80	-0,71
CYP2D6	-0,63	-0,37	0,25
CYP3A5	-2,88	-0,97	1,92
CYP4A11	-0,82	-0,66	0,17
CYP4V2	-0,49	1,62	2,11
CYP4X1	-1,26	-0,59	0,67
CYP51A1	0,80	3,00	2,20
CYSLTR1	-1,17	-1,72	-0,55
CYTL1	-3,06	3,29	6,35
CYYR1	2,60	8,62	6,02
D15WSU75E	-0,13	0,65	0,77
DAAM1	1,99	0,03	-1,96
DAB2	-0,60	5,41	6,01
DACH1	0,26	-3,48	-3,74
DACT1	3,68	11,74	8,07
DAD1	-0,29	0,69	0,98
DAK	-0,35	-0,32	0,03
DAO	0,01	-0,01	-0,02
DAP	0,42	0,57	0,14
DAP3	0,02	-0,51	-0,54
DAPK2	-0,35	0,07	0,42
DAPK3	0,71	-0,66	-1,37
DAPP1	0,47	-1,70	-2,17
DARC	2,67	5,76	3,09
DARS	0,38	0,84	0,46
DARS2	-0,28	1,74	2,03
DAXX	0,39	0,43	0,04
DAZAP2	0,31	-0,77	-1,08
DBI	-0,40	1,82	2,22
DBNDD2	0,92	1,38	0,46
DBNL	0,29	-0,34	-0,63
DBR1	-0,12	0,89	1,01
DBT	0,12	1,02	0,90
DC2	-0,36	0,01	0,38
DCAKD	-0,07	0,08	0,15
DCD	0,25	0,14	-0,10
DCHS2	-0,70	3,64	4,34
DCI	0,13	0,24	0,11
DCK	0,08	0,37	0,30
DCLRE1A	-0,42	1,63	2,05
DCLRE1C	0,31	-0,81	-1,12
DCP2	1,14	0,39	-0,75
DCTN1	0,21	-0,21	-0,41
DCTN2	-0,05	0,06	0,11

DCTN5	0,02	0,24	0,22
DCTN6	-0,44	0,60	1,04
DCUN1D2	0,98	0,78	-0,19
DCUN1D3	1,31	3,65	2,34
DCX	-0,36	-0,14	0,23
DDAH2	0,93	0,93	0,00
DDB1	-0,17	0,10	0,27
DDB2	0,24	1,12	0,88
DDC	-0,11	0,45	0,56
DDEF1	0,84	1,21	0,37
DDEF2	-0,70	1,47	2,17
DDHD1	0,13	2,27	2,14
DDI2	-3,24	-3,09	0,15
DDIT3	-0,54	0,40	0,94
DDO	-1,14	1,87	3,01
DDOST	-0,30	-0,01	0,29
DDX1	-0,39	-0,01	0,38
DDX17	0,34	-0,80	-1,14
DDX18	0,32	-0,61	-0,93
DDX19-DDX19L	-0,02	-0,10	-0,08
DDX19A	-0,20	-0,25	-0,05
DDX19B	-0,18	-0,16	0,02
DDX20	0,84	0,95	0,11
DDX21	-0,06	-0,96	-0,90
DDX23	0,39	-0,61	-1,00
DDX24	0,45	0,59	0,14
DDX28	-0,02	-0,67	-0,65
DDX31	0,09	0,17	0,08
DDX39	0,16	-0,54	-0,70
DDX3X	0,07	-0,90	-0,97
DDX41	0,04	-0,38	-0,42
DDX42	0,43	-0,26	-0,69
DDX43	0,16	-0,31	-0,47
DDX46	-0,07	-1,35	-1,28
DDX49	0,08	-0,55	-0,63
DDX5	0,30	-0,23	-0,53
DDX50	-0,04	-1,27	-1,23
DDX51	0,33	-1,47	-1,79
DDX52	0,17	0,09	-0,08
DDX54	0,24	-0,47	-0,71
DDX55	-0,16	-0,02	0,14
DDX56	0,25	-0,52	-0,76
DDX58	0,25	-1,01	-1,27
DDX59	-0,37	-1,18	-0,81
DEADC1	-0,04	-0,88	-0,84
DEAF1	0,73	-0,22	-0,95
DEDD	0,17	-0,61	-0,78
DEDD2	-0,23	-1,58	-1,35
DEFB108B	-0,15	-0,05	0,10
DEFB121	0,49	0,90	0,41
DEFB123	-0,35	0,34	0,70
DEFB125	0,46	-0,05	-0,51
DEFB126	0,28	0,39	0,11
DEFB128	-0,06	0,38	0,44
DENND1C	0,11	-0,42	-0,53
DENND2D	-0,22	2,10	2,32
DENND3	-1,09	-3,91	-2,81
DENND4C	0,43	2,06	1,63
DEPDC1	-8,19	-2,66	5,54

DEPDC6	1,90	1,39	-0,51
DEPDC7	-0,83	0,81	1,64
DERL1	-0,27	0,55	0,81
DERL2	-0,57	-0,05	0,51
DEXI	-0,43	2,43	2,86
DFFA	-0,18	0,05	0,23
DFFB	0,14	-1,12	-1,26
DFNA5	-0,98	4,46	5,44
DGAT2L3	0,06	-0,35	-0,42
DGCR14	0,22	-0,68	-0,91
DGKB	-0,08	0,06	0,14
DGKG	-1,03	-2,69	-1,66
DGKZ	-0,52	-1,44	-0,92
DGUOK	-0,02	0,05	0,07
DHCR7	0,61	5,64	5,03
DHDDS	0,07	0,77	0,70
DHDH	0,56	3,64	3,08
DHFR	-0,62	-0,24	0,38
DHFRL1	-0,07	1,74	1,81
DHODH	-3,68	-4,16	-0,48
DHPS	0,07	-0,63	-0,70
DHRS1	-0,94	-0,69	0,25
DHRS2	6,24	4,89	-1,35
DHRS3	0,07	7,59	7,52
DHRS4L2	-2,10	-1,20	0,90
DHRS7	-0,48	-0,39	0,08
DHRS7B	-0,78	-0,38	0,40
DHRS9	-0,73	2,53	3,25
DHX16	0,16	0,07	-0,09
DHX29	-0,28	0,13	0,41
DHX32	-0,31	0,35	0,66
DHX33	0,03	-0,84	-0,88
DHX35	0,14	-0,12	-0,26
DHX36	-0,31	0,12	0,44
DHX38	-0,03	-0,60	-0,57
DHX57	-0,55	-4,38	-3,83
DHX8	0,16	0,02	-0,14
DHX9	-0,08	-0,32	-0,25
DIAPH1	0,09	-0,42	-0,51
DIAPH3	-6,01	-3,99	2,01
DICER1	0,08	-1,06	-1,13
DIDO1	0,36	-1,11	-1,47
DIO1	-5,76	0,95	6,70
DIP	-0,02	-0,60	-0,58
DIRAS2	-0,43	-0,19	0,23
DIXDC1	-6,96	-0,31	6,65
DKC1	0,31	0,15	-0,15
DKFZP434B033	-0,01	-1,31	-1,30
DKFZP564J086	0,24	0,62	0,38
DKFZP564O052	0,75	0,42	-0,33
DKFZP586P012	0,02	-0,49	-0,51
DKFZP434K181	0,06	0,06	0,00
DKFZP434K191	1,01	-0,82	-1,83
DKFZP434N035	-0,21	-0,35	-0,14
DKFZP451A211	-0,15	0,43	0,57
DKFZP451M211	-1,30	-1,13	0,18
DKFZP564N247	1,39	0,78	-0,61
DKFZP666G057	2,01	-1,09	-3,10
DKFZP686I152	0,08	0,36	0,28

DKFZP686O241	-0,20	1,58	1,78
DKKL1	0,06	0,73	0,67
DLAT	0,24	1,20	0,96
DLEU7	-5,22	-5,96	-0,74
DLG4	-0,47	-2,70	-2,23
DLG5	-0,33	-0,02	0,32
DLGAP1	-0,42	1,62	2,04
DLGAP4	0,18	0,32	0,14
DMAP1	0,10	-0,43	-0,53
DMRTC1	-0,03	-0,45	-0,42
DMTF1	0,26	-1,01	-1,27
DMXL1	-0,81	-0,45	0,35
DMXL2	-1,24	-1,66	-0,42
DNAH10	-0,03	-1,29	-1,25
DNAH17	0,08	0,05	-0,03
DNAH3	2,59	3,01	0,42
DNAH5	0,20	0,23	0,03
DNAI1	0,81	-0,12	-0,93
DNAI2	-0,05	-0,10	-0,05
DNAJA1	-0,24	0,07	0,31
DNAJA3	-0,09	0,18	0,28
DNAJA5	0,06	-0,40	-0,46
DNAJB1	0,31	0,02	-0,29
DNAJB11	-0,24	0,07	0,31
DNAJB14	0,00	-0,38	-0,39
DNAJB5	-1,14	1,57	2,71
DNAJB7	-0,11	0,19	0,30
DNAJB9	0,18	0,96	0,78
DNAJC1	0,23	-0,35	-0,58
DNAJC10	-0,10	-0,70	-0,60
DNAJC12	1,98	4,26	2,28
DNAJC13	-0,42	-0,69	-0,27
DNAJC17	-0,12	0,10	0,21
DNAJC19	0,03	0,47	0,44
DNAJC3	-0,25	-0,28	-0,03
DNAJC5B	-0,68	5,51	6,20
DNAJC7	0,42	-0,19	-0,61
DNAJC8	-0,14	0,31	0,45
DNAJC9	-0,65	0,20	0,85
DNAL4	-0,77	-0,60	0,16
DNASE1L1	0,12	-0,44	-0,55
DNASE1L3	8,42	9,58	1,16
DNASE2B	-2,91	5,60	8,51
DNHD1	-0,30	0,20	0,50
DNM2	-0,08	-0,43	-0,35
DNMT1	0,33	0,96	0,63
DNTTIP1	-0,12	-0,70	-0,58
DNTTIP2	0,63	-0,25	-0,88
DOCK1	1,35	9,96	8,60
DOCK10	1,21	1,15	-0,05
DOCK11	0,21	-1,09	-1,30
DOCK3	0,12	7,40	7,28
DOCK4	0,74	1,95	1,20
DOCK5	-0,16	0,28	0,44
DOCK7	0,71	2,39	1,68
DOCK8	0,40	-0,79	-1,18
DOCK9	3,21	0,25	-2,97
DOK2	1,04	-0,38	-1,43
DOM3Z	0,24	-0,52	-0,76

DONSON	-0,36	0,91	1,27
DOPEY1	0,51	-0,69	-1,20
DOPEY2	-0,04	1,01	1,05
DPAGT1	-0,46	0,27	0,73
DPCR1	-0,06	-0,27	-0,22
DPH2	0,35	0,13	-0,22
DPM1	0,18	0,64	0,46
DPM3	0,06	0,44	0,39
DPP3	-0,21	0,33	0,54
DPP8	-0,02	-0,29	-0,27
DPYSL2	0,47	0,27	-0,20
DPYSL4	0,76	1,12	0,37
DR1	-0,39	-1,08	-0,70
DRAP1	-0,11	-0,16	-0,05
DRD3	0,12	-1,90	-2,02
DRG1	-0,21	-0,24	-0,03
DRG2	-0,08	-0,51	-0,43
DRP2	0,06	-0,28	-0,33
DSCR1	0,58	0,78	0,20
DSCR10	-0,26	0,66	0,92
DSCR1L1	-0,18	0,02	0,20
DSCR2	-0,17	0,65	0,82
DSCR3	0,17	0,40	0,23
DSCR4	0,08	1,70	1,62
DST	-0,08	-0,69	-0,61
DTL	-5,49	-1,14	4,36
DTNA	1,98	4,44	2,46
DTNBP1	0,37	-0,48	-0,85
DTWD1	-0,04	-0,16	-0,12
DTWD2	-1,05	1,24	2,29
DTX3L	-0,05	-0,54	-0,48
DULLARD	0,13	-0,35	-0,48
DUOX1	10,43	10,43	0,00
DUS2L	-0,43	-0,52	-0,09
DUS4L	-0,20	0,22	0,42
DUSP10	-0,84	-0,40	0,44
DUSP11	-0,03	0,51	0,54
DUSP12	0,07	-0,76	-0,83
DUSP13	-0,10	-0,11	0,00
DUSP18	-0,31	-0,17	0,14
DUSP23	-0,14	0,44	0,58
DUSP5	-0,01	2,15	2,16
DUT	-0,15	-0,72	-0,57
DVL2	0,59	1,08	0,49
DVL3	0,03	-0,12	-0,15
DYNC1H1	0,73	0,42	-0,31
DYNC1LI1	0,98	0,22	-0,76
DYNC1LI2	0,30	0,06	-0,24
DYNLL2	0,05	-1,35	-1,39
DYNLRB1	-0,06	0,61	0,67
DYRK1A	0,41	-0,95	-1,35
DYRK1B	0,43	-1,02	-1,45
DYRK3	-0,98	0,87	1,85
DYRK4	-0,15	0,60	0,74
DYSF	2,64	-0,65	-3,29
DYX1C1	-1,09	0,79	1,88
E2F4	0,23	-0,64	-0,87
E2F8	-2,05	3,27	5,33
EAF1	-0,83	-0,54	0,29

EARS2	-0,33	1,71	2,04
EBAG9	-0,07	-0,30	-0,23
EBI2	2,25	0,78	-1,46
EBI3	3,93	1,80	-2,13
EBNA1BP2	-0,77	-0,15	0,61
EBP	-0,44	2,16	2,59
ECD	-0,41	0,21	0,62
ECE2	-0,22	0,54	0,77
ECHDC3	0,31	0,43	0,11
ECHS1	0,00	1,21	1,22
ECM1	-0,72	5,96	6,68
ECT2	-0,52	0,71	1,23
EDEM1	0,33	0,91	0,58
EDEM2	-1,21	-0,88	0,34
EDEM3	0,10	-0,78	-0,88
EDG6	0,01	-1,30	-1,31
EDN1	-0,92	1,86	2,78
EED	0,40	0,26	-0,14
EEF1B2	0,17	-0,39	-0,56
EEF1D	0,17	-1,14	-1,31
EEF2	0,29	-0,55	-0,84
EEF2K	0,23	2,25	2,01
EFCAB2	-0,46	0,83	1,28
EFHA1	0,01	0,06	0,05
EFHB	-0,08	-0,41	-0,33
EFHD2	0,42	-0,55	-0,96
EFNA3	-0,49	-0,44	0,05
EFNB1	-0,71	-0,94	-0,23
EFTUD1	-0,45	-0,05	0,39
EFTUD2	-0,03	-0,58	-0,54
EGFR	-0,01	0,04	0,05
EGLN1	0,34	-0,19	-0,54
EGR1	-1,31	-1,57	-0,26
EGR2	-0,07	5,38	5,45
EHBP1	-0,17	-0,78	-0,61
EHD4	1,14	1,86	0,72
EHF	3,43	4,46	1,03
EHMT1	0,69	-0,59	-1,29
EID3	0,38	0,08	-0,30
EIF1B	0,55	0,81	0,26
EIF2A	-0,19	-0,40	-0,21
EIF2AK2	-0,45	-1,17	-0,73
EIF2B1	-0,15	-0,54	-0,39
EIF2B2	-0,33	0,54	0,87
EIF2B3	-0,40	0,40	0,80
EIF2B4	0,41	0,21	-0,21
EIF2B5	-0,02	0,23	0,25
EIF2C1	-0,86	-0,48	0,37
EIF2C3	-0,21	0,46	0,67
EIF2S1	0,32	0,14	-0,19
EIF4A1	-0,12	0,18	0,29
EIF4B	0,08	-0,98	-1,07
EIF4E2	0,09	-0,83	-0,93
EIF4E3	-0,90	-2,12	-1,21
EIF4EBP3	-0,80	-0,99	-0,19
EIF4ENIF1	0,46	0,36	-0,10
EIF4G1	0,17	0,31	0,15
EIF4G3	0,19	0,88	0,70
EIF5	-0,30	0,32	0,62

EIF5B	0,06	0,89	0,83
ELA1	-3,60	-6,16	-2,55
ELA2	0,43	-1,36	-1,80
ELA2A	-0,55	0,44	1,00
ELAC1	1,04	0,84	-0,21
ELAC2	0,20	-0,12	-0,32
ELF2	0,36	-1,31	-1,67
ELF3	-0,26	1,79	2,05
ELF5	0,30	0,33	0,03
ELK1	0,38	0,62	0,24
ELK4	0,11	-1,67	-1,78
ELL2	-1,22	1,66	2,88
ELMO1	0,48	-0,20	-0,68
ELMOD2	-0,08	2,50	2,58
ELOF1	-0,15	-0,17	-0,01
ELOVL3	-7,24	-7,53	-0,29
ELOVL5	-0,10	0,39	0,49
ELP3	-0,43	-0,43	0,00
ELP4	-0,05	0,21	0,26
EME1	-1,55	1,00	2,55
EME2	-0,47	-0,15	0,32
EMG1	0,06	0,80	0,74
EMILIN1	-6,08	1,28	7,36
EMILIN2	-0,01	0,33	0,34
EML3	0,27	-0,34	-0,61
EML4	-0,91	1,41	2,33
EMP1	-1,75	4,22	5,97
EMR1	-3,47	-11,99	-8,52
EMR2	-0,30	-1,07	-0,77
EMR3	-5,29	-5,75	-0,46
ENC1	-0,51	-1,71	-1,20
ENO1	0,16	0,83	0,67
ENPP4	-1,73	1,92	3,64
ENTPD4	0,35	-0,17	-0,52
ENTPD5	0,21	1,07	0,86
ENTPD6	0,68	0,24	-0,44
ENTPD7	-1,06	1,49	2,55
ENTPD8	-0,24	-0,26	-0,01
ENY2	-0,10	-0,25	-0,15
EP400	0,16	-0,62	-0,78
EPAS1	-0,33	5,85	6,18
EPB41L1	-0,64	3,39	4,03
EPB41L2	1,71	2,35	0,64
EPB42	-0,89	0,39	1,27
EPHX1	-0,88	1,73	2,61
EPM2A	0,00	0,44	0,44
EPM2AIP1	-0,06	-0,52	-0,46
EPN1	0,43	-0,19	-0,61
EPRS	-0,02	0,56	0,59
EPS15	0,94	-0,25	-1,20
EPS8L3	0,90	-0,89	-1,79
EPSTI1	-0,16	-1,37	-1,21
ERBB2	0,45	0,39	-0,06
ERCC2	-0,08	-0,76	-0,68
ERCC4	-0,65	0,87	1,52
ERCC5	0,36	-0,44	-0,80
ERCC8	-0,59	-0,37	0,23
ERGIC1	-0,51	-0,54	-0,03
ERGIC3	-0,33	0,31	0,65

ERH	-0,03	-0,09	-0,06
ERICH1	0,07	-2,15	-2,22
ERMAP	-0,01	-2,16	-2,15
ERN1	-0,47	-1,26	-0,80
ERO1L	0,21	1,51	1,30
ERO1LB	1,00	-1,53	-2,53
ERRFI1	0,40	3,17	2,77
ESD	-0,19	-0,15	0,04
ESPL1	-0,91	-0,34	0,57
ESR1	0,13	7,01	6,88
ESRRA	0,58	0,02	-0,56
ETF1	-0,27	0,28	0,54
ETFB	-0,02	0,48	0,50
ETFDH	-0,75	0,23	0,99
ETHE1	0,38	0,81	0,43
ETS1	3,13	2,48	-0,65
ETS2	-0,98	-3,52	-2,54
ETV2	0,44	-0,03	-0,47
ETV3	3,09	1,01	-2,07
ETV5	1,40	5,30	3,90
EVA1	-3,99	-7,07	-3,08
EVI5	0,19	-1,21	-1,39
EVL	0,37	2,09	1,72
EWSR1	0,16	-0,67	-0,83
EXDL2	-0,44	0,97	1,40
EXO1	-2,24	-0,40	1,84
EXOC1	-0,08	-0,04	0,05
EXOC5	0,55	0,18	-0,37
EXOC6	-0,42	0,20	0,62
EXOC7	-0,02	-0,21	-0,20
EXOC8	0,08	-0,06	-0,14
EXOSC1	-0,15	-0,32	-0,17
EXOSC2	0,05	-0,35	-0,40
EXOSC3	0,04	1,02	0,98
EXOSC4	0,43	0,61	0,18
EXOSC5	-0,44	-0,87	-0,43
EXOSC7	-0,56	0,08	0,64
EXOSC9	-0,32	-0,19	0,13
EXTL1	0,50	-0,26	-0,76
EXTL2	-0,36	2,25	2,61
EXTL3	-0,08	-1,59	-1,50
EYA3	-0,97	-3,02	-2,05
EZH1	0,05	-0,72	-0,77
EZH2	0,45	0,81	0,35
F11R	-0,30	-0,28	0,02
F12	-1,25	-1,98	-0,74
F13A1	9,41	1,85	-7,56
F5	-3,54	-9,46	-5,93
F8	-0,24	0,92	1,16
F8A1	0,13	-0,02	-0,16
F8A3	0,21	-0,32	-0,53
FA2H	0,82	7,31	6,49
FABP1	-0,75	-0,23	0,52
FABP2	-0,91	-0,36	0,55
FABP3	0,47	9,35	8,88
FABP4	7,54	9,57	2,03
FABP6	-0,03	0,09	0,11
FABP7	0,05	0,16	0,11
FADD	0,16	1,13	0,96

FADS1	0,19	3,01	2,82
FADS2	0,37	2,87	2,50
FAF1	-0,31	-0,09	0,22
FAH	-1,40	0,89	2,28
FAHD1	0,10	1,21	1,11
FAHD2A	-0,58	0,36	0,94
FAIM3	-4,25	1,44	5,69
FAM101A	-0,17	-0,37	-0,20
FAM102A	1,17	3,00	1,83
FAM102B	0,64	-0,07	-0,71
FAM103A1	0,17	0,47	0,29
FAM104A	0,06	-0,26	-0,32
FAM105A	0,06	-1,34	-1,40
FAM105B	0,12	1,16	1,05
FAM107A	-1,70	-1,57	0,13
FAM107B	-1,33	-1,41	-0,08
FAM108A1	-0,85	-1,20	-0,35
FAM109B	0,00	0,00	0,00
FAM111A	-0,82	-1,81	-0,99
FAM112B	-0,43	-0,97	-0,54
FAM113A	0,23	-0,36	-0,59
FAM113B	0,01	2,46	2,45
FAM116B	-0,48	-2,36	-1,88
FAM14A	-0,95	-0,45	0,50
FAM14B	-0,93	5,42	6,35
FAM18B	-0,34	1,46	1,79
FAM19A3	0,49	5,45	4,96
FAM20A	2,25	9,34	7,09
FAM21C	0,28	-0,07	-0,34
FAM24B	-0,46	-1,33	-0,87
FAM32A	0,59	-0,27	-0,85
FAM33A	0,36	0,66	0,30
FAM35A	0,07	-0,35	-0,42
FAM38A	0,27	-0,18	-0,45
FAM3A	-0,04	0,42	0,46
FAM3C	-0,40	1,33	1,74
FAM40A	0,20	-0,10	-0,30
FAM43A	1,04	0,52	-0,51
FAM44A	-1,18	-1,02	0,17
FAM44B	0,15	0,14	-0,01
FAM45A	0,10	-1,60	-1,71
FAM45B	-0,31	-2,00	-1,69
FAM48A	0,02	-0,42	-0,44
FAM49A	-1,13	-2,73	-1,60
FAM50A	0,18	0,29	0,11
FAM53B	0,66	-1,96	-2,62
FAM54A	-1,43	2,23	3,65
FAM58A	0,62	1,18	0,56
FAM62A	0,23	1,19	0,96
FAM63B	-0,14	-0,13	0,01
FAM65A	0,14	-0,15	-0,29
FAM70B	-0,96	0,88	1,84
FAM71C	-1,08	-0,15	0,93
FAM72A	0,19	-0,45	-0,64
FAM73A	0,21	-1,03	-1,23
FAM76B	0,12	-2,34	-2,46
FAM79A	0,14	0,58	0,44
FAM79B	3,56	5,19	1,63
FAM80B	-0,24	-1,57	-1,33

FAM82A	-0,12	0,38	0,50
FAM82B	-0,13	-0,88	-0,75
FAM82C	-1,91	0,95	2,86
FAM83C	-0,47	-0,24	0,23
FAM83D	-4,54	0,83	5,37
FAM84A	-0,03	0,17	0,20
FAM89B	-0,71	0,40	1,11
FAM8A1	-0,54	-1,66	-1,12
FAM96A	0,29	1,07	0,77
FAM96B	0,08	0,39	0,31
FAM98C	-0,05	-1,39	-1,34
FANCA	-0,79	3,33	4,13
FANCB	-1,88	-2,19	-0,31
FANCE	-2,88	0,28	3,16
FANCG	0,27	1,48	1,20
FANK1	1,52	0,62	-0,91
FARP2	-0,15	0,77	0,92
FARS2	-0,28	-0,55	-0,27
FARSLB	0,29	-1,73	-2,02
FASN	0,21	4,23	4,02
FASTK	-0,07	0,14	0,21
FAT2	0,11	0,56	0,44
FAT4	1,11	0,78	-0,33
FAU	0,12	-0,70	-0,82
FBL	0,05	-1,67	-1,72
FBP1	-1,53	2,43	3,95
FBS1	0,58	-0,60	-1,18
FBXL14	-0,29	-1,22	-0,93
FBXL16	0,39	-0,48	-0,87
FBXL17	-0,40	-0,14	0,26
FBXL19	-0,39	-0,61	-0,22
FBXL22	-1,59	-1,35	0,23
FBXL3	0,25	0,28	0,02
FBXL6	0,44	0,40	-0,04
FBXL8	0,59	-0,61	-1,21
FBXO15	-1,79	3,08	4,88
FBXO22	-0,23	0,42	0,65
FBXO27	2,07	6,33	4,27
FBXO28	-0,39	0,20	0,59
FBXO30	-0,09	0,63	0,72
FBXO31	0,44	0,36	-0,09
FBXO32	0,21	1,26	1,05
FBXO34	-0,09	-0,90	-0,81
FBXO38	0,08	0,26	0,18
FBXO4	0,39	0,64	0,26
FBXO43	0,06	0,03	-0,03
FBXO5	-1,16	-0,44	0,73
FBXO7	0,20	0,25	0,05
FBXO8	0,16	1,72	1,56
FBXO9	-0,04	-0,59	-0,55
FBXW11	-0,28	0,13	0,41
FBXW2	-0,05	-0,27	-0,22
FBXW4	-0,16	-0,01	0,15
FBXW7	0,20	-1,78	-1,97
FCER1G	-0,93	-0,35	0,58
FCER2	4,29	5,81	1,52
FCGR2A	-0,11	-1,28	-1,17
FCHO2	-0,75	0,65	1,41
FCN1	-5,01	-9,08	-4,07

FCN3	-0,13	-0,24	-0,12
FCRL1	0,21	-0,33	-0,54
FCRL3	0,11	-0,08	-0,19
FCRL5	-0,69	-1,58	-0,88
FCRL6	0,03	0,27	0,23
FDFT1	0,37	1,13	0,76
FDXR	-0,59	0,50	1,08
FEM1B	-0,70	0,71	1,41
FEM1C	1,21	0,44	-0,77
FEN1	-1,58	0,36	1,95
FER	-0,06	-0,40	-0,34
FER1L3	-0,67	1,44	2,11
FES	0,78	-1,26	-2,05
FFAR1	0,00	-0,07	-0,07
FFAR2	-0,97	-1,89	-0,92
FGA	-0,84	-0,67	0,18
FGD4	-0,27	-1,27	-1,00
FGD5	-0,66	9,71	10,37
FGD6	-0,60	-0,65	-0,05
FGF11	0,67	0,44	-0,22
FGF12	-0,43	-0,57	-0,14
FGF13	-0,26	0,61	0,87
FGF6	0,06	-0,29	-0,36
FGFR1OP2	-0,38	-0,47	-0,09
FGG	-0,23	-0,10	0,12
FHIT	0,12	1,50	1,37
FHL1	-0,96	2,89	3,85
FHL5	-0,19	-0,28	-0,09
FIBP	-0,01	-0,09	-0,08
FIGN	-0,05	1,80	1,85
FIGNL1	-1,07	1,84	2,92
FIP1L1	0,00	-0,09	-0,09
FIS	-0,44	-0,14	0,30
FIS1	-0,10	0,50	0,59
FJX1	0,08	2,38	2,30
FKBP14	0,58	2,01	1,43
FKBP1A	-0,05	0,02	0,08
FKBP3	-0,01	0,17	0,17
FKBP6	1,11	-0,21	-1,32
FKBP9	-3,72	-2,38	1,34
FKBPL	-0,10	-0,24	-0,14
FKRP	-0,27	-0,70	-0,42
FKSG44	0,51	0,00	-0,51
FKSG83	-0,09	-1,98	-1,89
FLAD1	0,21	-0,23	-0,44
FLJ10081	-0,17	0,02	0,20
FLJ10154	0,48	-1,07	-1,56
FLJ10213	-0,73	-1,55	-0,82
FLJ10241	-0,13	0,38	0,51
FLJ10324	-3,13	-4,66	-1,52
FLJ10803	0,79	0,82	0,03
FLJ10986	-0,83	2,14	2,97
FLJ11151	0,48	-2,11	-2,59
FLJ11184	0,38	-0,08	-0,47
FLJ11506	0,57	-0,13	-0,70
FLJ11783	0,33	-1,42	-1,75
FLJ12716	-0,13	-0,67	-0,54
FLJ13611	-0,19	0,08	0,26
FLJ14107	0,97	-0,99	-1,95

FLJ14803	-0,10	-0,29	-0,19
FLJ16478	-0,25	0,84	1,09
FLJ20035	-1,47	-2,40	-0,92
FLJ20054	1,76	2,94	1,18
FLJ20160	-0,36	1,87	2,23
FLJ20273	0,71	0,83	0,12
FLJ20294	0,07	0,20	0,13
FLJ20309	-0,11	-0,69	-0,57
FLJ20323	0,09	-0,18	-0,27
FLJ20489	-1,16	0,99	2,16
FLJ20581	0,98	2,80	1,82
FLJ20674	-0,59	0,36	0,95
FLJ20699	0,13	0,17	0,04
FLJ20850	2,11	-0,29	-2,39
FLJ21687	1,58	-0,59	-2,17
FLJ21865	-0,27	-2,05	-1,78
FLJ21963	-0,25	-0,30	-0,05
FLJ21986	-3,90	-4,71	-0,82
FLJ22222	-0,08	0,12	0,20
FLJ22639	-1,20	-1,56	-0,36
FLJ25006	0,72	0,58	-0,14
FLJ25715	-0,13	0,15	0,28
FLJ25758	-0,18	-0,66	-0,48
FLJ25791	-3,97	-0,24	3,73
FLJ26443	-0,52	-0,53	-0,01
FLJ27255	-0,28	0,02	0,30
FLJ30679	0,27	0,37	0,10
FLJ31438	-0,26	-0,37	-0,11
FLJ32679	0,14	-0,16	-0,30
FLJ33590	-0,79	-0,81	-0,03
FLJ34870	3,97	4,49	0,53
FLJ34931	-0,66	1,27	1,93
FLJ35740	-0,38	-0,78	-0,40
FLJ35767	-0,30	-0,31	-0,01
FLJ35773	0,28	0,03	-0,24
FLJ35801	-0,06	-0,09	-0,03
FLJ36031	4,63	6,28	1,65
FLJ36144	-0,24	1,56	1,80
FLJ36208	-4,75	-7,35	-2,60
FLJ36492	-2,24	-3,93	-1,69
FLJ36701	-0,93	3,04	3,97
FLJ36874	-0,12	-0,37	-0,25
FLJ37396	0,50	-0,67	-1,18
FLJ37464	-0,30	-1,09	-0,79
FLJ37543	0,45	0,22	-0,23
FLJ38377	0,46	-0,86	-1,32
FLJ38482	0,12	0,95	0,83
FLJ38973	0,63	0,80	0,17
FLJ39653	0,67	0,28	-0,39
FLJ39779	-0,17	-1,21	-1,03
FLJ39822	-0,42	-0,61	-0,20
FLJ40142	0,98	0,13	-0,85
FLJ40288	-1,26	-0,60	0,65
FLJ40852	0,26	0,58	0,32
FLJ41327	0,08	0,06	-0,03
FLJ41423	0,71	0,55	-0,16
FLJ42133	0,52	-0,46	-0,99
FLJ42957	1,18	-4,95	-6,13
FLJ43879	-0,35	-0,74	-0,38

FLJ43980	0,19	0,52	0,32
FLJ44186	-0,57	-1,04	-0,47
FLJ44385	-0,27	-0,21	0,07
FLJ44635	0,40	-0,72	-1,12
FLJ45055	-0,15	0,39	0,54
FLJ45202	0,50	-0,61	-1,11
FLJ45337	0,05	-0,65	-0,70
FLJ45909	-1,60	-2,17	-0,57
FLJ46082	0,79	-0,80	-1,59
FLJ46154	-0,46	-0,47	-0,01
FLJ46347	-0,25	0,69	0,95
FLJ46481	-0,29	0,41	0,70
FLJ90709	-0,20	0,40	0,60
FLNB	1,21	2,55	1,34
FLOT1	-0,94	-0,82	0,12
FLOT2	-0,07	-0,59	-0,52
FLRT2	-2,60	1,25	3,84
FLT3	2,42	-2,89	-5,32
FLYWCH1	-0,06	0,22	0,28
FMNL2	-0,36	2,09	2,46
FMNL3	-0,31	2,13	2,44
FMO1	-0,72	3,14	3,86
FMO2	0,49	-0,11	-0,60
FMO4	-0,92	-0,12	0,80
FNBP1L	0,01	2,90	2,89
FNDC3B	0,18	0,62	0,43
FNDC8	0,18	0,67	0,50
FNTA	0,16	-0,08	-0,24
FNTB	-0,52	-0,83	-0,31
FOLR1	-0,64	-0,13	0,51
FOS	-0,54	-4,22	-3,67
FOSB	-5,14	-7,61	-2,47
FOXD2	1,84	2,76	0,92
FOXI1	-0,09	0,42	0,51
FOXJ2	1,00	0,21	-0,79
FOXJ3	-0,01	-0,60	-0,60
FOXM1	-1,21	0,17	1,37
FOXP3	-0,18	0,18	0,36
FOXQ1	11,50	8,20	-3,30
FOXRED1	-0,13	0,62	0,74
FPGS	-0,03	1,58	1,62
FPGT	0,80	0,56	-0,24
FPR1	-2,79	-5,79	-3,00
FPRL1	-1,34	-2,76	-1,42
FPRL2	1,23	5,91	4,68
FRAP1	-0,11	-0,20	-0,09
FRAT2	0,38	-0,80	-1,19
FRG1	-0,03	0,39	0,41
FRMD3	-1,06	-2,61	-1,56
FRMD4A	1,85	12,59	10,74
FRS2	-0,12	-0,68	-0,55
FRS3	0,04	-1,16	-1,20
FSD1L	0,17	0,11	-0,06
FSHR	0,10	0,34	0,24
FTH1	-1,32	-0,13	1,19
FTSJ1	-0,26	-0,87	-0,61
FTSJ2	0,29	0,39	0,10
FTSJ3	0,28	0,04	-0,24
FUBP1	-0,48	-0,88	-0,40

FUK	-0,15	-0,28	-0,13
FUNDC2	-0,12	-0,13	-0,02
FURIN	0,90	1,64	0,73
FUT11	0,23	0,30	0,08
FUT3	-0,66	-0,66	0,00
FUT4	0,04	-0,93	-0,97
FUT6	-0,19	-0,07	0,12
FUT7	1,77	-0,50	-2,27
FVT1	-0,24	0,35	0,59
FXC1	-0,07	0,40	0,47
FXR1	0,40	-0,24	-0,64
FXR2	0,15	0,21	0,06
FXYD1	-0,73	-0,08	0,66
FXYD3	0,72	0,35	-0,37
FYN	-0,35	-3,81	-3,46
FYTTD1	0,22	0,20	-0,02
G0S2	0,61	-3,21	-3,82
G3BP2	0,11	0,48	0,38
G6PC3	-0,55	0,24	0,79
G6PD	-0,11	1,23	1,34
GAB2	1,21	-0,68	-1,89
GAB3	-0,08	-1,83	-1,75
GABARAP	-0,15	-0,93	-0,79
GABPA	-0,30	-0,78	-0,48
GABPB2	0,53	1,42	0,89
GABRA3	0,44	-0,24	-0,68
GABRP	-2,26	-1,56	0,70
GALC	-0,30	0,08	0,38
GALE	-0,25	1,41	1,66
GALK2	0,50	0,16	-0,34
GALM	0,63	5,43	4,80
GALNAC4S-6ST	-0,52	-1,33	-0,81
GALNACT-2	0,15	-0,80	-0,95
GALNS	-0,48	0,64	1,12
GALNT1	0,06	0,61	0,55
GALNT13	0,12	0,19	0,07
GALNT3	0,43	-1,67	-2,10
GALNT6	-0,66	-0,06	0,60
GALT	0,01	-0,30	-0,31
GAN	0,19	-0,51	-0,70
GANAB	-0,32	-0,45	-0,13
GANC	0,20	-0,18	-0,38
GARNL3	0,73	-0,58	-1,31
GARS	0,03	1,45	1,42
GART	0,31	0,78	0,47
GAS6	2,34	2,70	0,36
GATAD1	0,24	0,14	-0,10
GATAD2A	-0,05	-0,08	-0,04
GBA	-0,52	2,02	2,55
GBA2	0,52	-0,13	-0,65
GBE1	-0,77	-0,16	0,61
GBF1	0,04	-0,11	-0,15
GBP1	-1,05	-1,13	-0,08
GCA	-0,91	-1,52	-0,61
GCAT	1,61	1,22	-0,39
GCC1	0,21	0,52	0,30
GCDH	0,19	-0,35	-0,54
GCET2	0,23	0,32	0,10
GCLC	-1,18	0,70	1,88

GCLM	-0,18	2,45	2,63
GCN5L2	0,34	-0,65	-0,99
GCNT2	-0,79	-0,49	0,30
GCNT3	3,35	7,89	4,54
GCNT4	-0,17	-0,21	-0,04
GCS1	0,05	-0,95	-1,00
GDAP2	0,27	-0,14	-0,42
GDF15	-0,71	4,24	4,95
GDF2	0,30	0,32	0,02
GDF3	-0,72	-0,52	0,21
GDF5	-0,29	-0,04	0,25
GDF9	6,24	3,19	-3,05
GDI1	0,32	0,18	-0,14
GDPD2	-0,54	0,34	0,88
GDPD4	0,21	0,49	0,28
GEMIN4	-0,51	-0,20	0,31
GEMIN5	-0,10	-0,14	-0,05
GEMIN6	-0,04	0,96	1,00
GFM1	0,33	0,34	0,02
GFM2	-0,50	0,94	1,44
GFOD1	1,21	2,91	1,70
GFOD2	0,31	0,52	0,21
GFPT1	0,75	2,53	1,78
GFRA2	2,47	1,72	-0,76
GGA2	0,66	0,63	-0,03
GGA3	0,19	-0,55	-0,74
GGCX	-0,32	1,39	1,71
GGT1	-0,73	-0,80	-0,07
GGTL3	1,55	0,42	-1,14
GGTLA1	1,93	2,72	0,79
GGTLA4	-0,30	-1,28	-0,98
GHRH	0,19	-0,12	-0,31
GHRHR	-0,70	0,47	1,17
GHRL	-6,43	-10,30	-3,87
GIF	0,14	-0,79	-0,93
GIMAP2	-0,40	-1,71	-1,31
GIMAP8	-1,46	-3,51	-2,05
GIOT-1	0,31	-0,37	-0,68
GIP	-0,64	-0,91	-0,27
GIPC1	0,07	2,19	2,11
GIT2	-0,03	-1,45	-1,43
GIYD2	0,13	-1,03	-1,16
GJA4	-0,86	-0,55	0,31
GJA5	-0,04	0,36	0,40
GJB7	-2,52	-3,48	-0,96
GK	-0,88	1,34	2,22
GLA	-0,63	1,50	2,14
GLB1	0,01	0,69	0,68
GLB1L	-0,40	-0,19	0,22
GLDN	0,91	2,38	1,47
GLE1L	0,09	0,37	0,28
GLG1	0,07	-0,36	-0,43
GLI4	1,67	-5,20	-6,87
GLIPR1	-0,98	-0,83	0,15
GLIS1	-0,39	-0,19	0,20
GLRX2	-0,04	2,22	2,26
GLRX5	0,06	0,11	0,05
GLT1D1	-0,50	-1,69	-1,19
GLT25D1	-0,61	0,15	0,76

GLT8D1	0,14	-0,30	-0,45
GLTP	-0,19	-0,74	-0,55
GLTSCR1	0,55	-0,58	-1,13
GLUD1	0,24	-0,24	-0,47
GLUL	-0,99	-0,82	0,16
GLYAT	-1,08	0,68	1,76
GLYCTK	-0,25	-0,82	-0,57
GM2A	-0,10	2,81	2,91
GMCL1	-0,08	-0,09	-0,01
GMEB1	-0,06	-0,62	-0,56
GMEB2	0,42	-0,39	-0,81
GMFB	0,16	1,30	1,14
GMFG	-0,35	-1,20	-0,84
GMIP	0,55	-0,48	-1,03
GMPPA	-0,51	-0,30	0,21
GMPPB	-0,21	0,35	0,57
GMPR2	-0,18	-0,46	-0,28
GNA13	0,28	-0,31	-0,59
GNAI2	0,25	-0,50	-0,75
GNAI3	-0,01	-0,36	-0,35
GNAQ	0,30	0,27	-0,04
GNAS	-0,33	-0,23	0,10
GNB1	0,19	-0,14	-0,33
GNB1L	0,35	0,07	-0,28
GNB4	1,10	0,89	-0,21
GNB5	-1,05	1,72	2,77
GNE	0,16	0,53	0,37
GNG10	-0,01	1,35	1,36
GNG2	-0,13	-3,22	-3,09
GNG5	0,03	0,44	0,41
GNG7	0,75	-0,50	-1,24
GNGT2	2,56	0,62	-1,94
GNL1	-0,17	0,29	0,46
GNL2	-0,12	-0,47	-0,34
GNL3	0,21	0,43	0,22
GNL3L	0,35	-0,74	-1,09
GNPAT	-0,02	-0,02	0,00
GNPDA1	0,24	1,68	1,45
GNPDA2	-0,36	0,52	0,88
GNPTG	-0,07	0,23	0,30
GNRH1	0,16	-0,93	-1,09
GNS	0,27	0,70	0,43
GOLGA1	0,34	-0,31	-0,65
GOLGA2	-0,15	0,05	0,20
GOLGA3	0,27	0,40	0,12
GOLGA4	-0,17	0,34	0,51
GOLGA5	-0,03	0,24	0,27
GOLGA8A	3,72	2,47	-1,25
GOLGA8B	5,57	6,49	0,93
GOLGA8E	0,51	0,92	0,41
GOLGB1	0,58	0,88	0,30
GOLPH2	1,66	-0,26	-1,93
GOLPH4	0,02	0,53	0,51
GOLT1B	-0,16	0,41	0,57
GON4L	0,41	-0,54	-0,95
GOPC	0,20	-0,11	-0,30
GORASP1	0,37	-0,51	-0,88
GORASP2	0,07	0,62	0,55
GOSR1	0,16	0,45	0,28

GOT1	0,23	2,22	1,98
GOT2	-0,01	1,08	1,09
GP1BA	0,34	0,67	0,33
GPA33	-0,81	-0,48	0,32
GPAA1	-0,05	0,51	0,56
GPAM	-0,19	1,34	1,52
GPBP1L1	-0,03	-0,32	-0,30
GPC3	-6,54	1,75	8,30
GPC5	1,20	2,51	1,31
GPD1	-1,63	4,81	6,44
GPHA2	0,23	-0,27	-0,50
GPHB5	3,22	-1,70	-4,92
GPHN	-0,02	0,71	0,73
GPI	0,18	1,03	0,85
GPKOW	-0,44	-1,04	-0,60
GPNMB	-0,54	8,66	9,20
GPR107	-0,49	-0,10	0,38
GPR108	-0,07	-0,12	-0,05
GPR109A	-2,54	-0,68	1,85
GPR109B	-0,05	0,52	0,57
GPR110	0,06	-0,70	-0,76
GPR113	0,11	-0,39	-0,50
GPR133	0,34	0,16	-0,17
GPR137	-0,03	0,68	0,70
GPR141	0,07	1,50	1,43
GPR142	-0,11	1,08	1,19
GPR143	2,33	1,07	-1,26
GPR151	-0,13	4,20	4,33
GPR155	2,34	-1,16	-3,50
GPR156	0,42	0,61	0,19
GPR161	0,16	0,25	0,10
GPR162	-1,37	-4,29	-2,92
GPR171	2,80	3,41	0,61
GPR172A	-0,03	0,65	0,68
GPR176	1,74	1,50	-0,24
GPR180	-0,42	1,04	1,46
GPR19	0,63	0,21	-0,42
GPR20	-0,14	0,25	0,39
GPR23	-1,62	-0,47	1,14
GPR26	0,05	-0,22	-0,27
GPR27	0,23	0,14	-0,09
GPR34	-4,55	-2,32	2,23
GPR35	3,59	1,42	-2,18
GPR37L1	0,47	0,08	-0,40
GPR44	2,70	-3,00	-5,70
GPR45	-0,26	0,36	0,62
GPR64	2,15	3,58	1,42
GPR65	-1,26	-1,57	-0,31
GPR82	-1,10	1,11	2,21
GPR84	-3,45	-1,28	2,17
GPRASP1	0,35	0,46	0,11
GPRC5A	-0,74	-0,68	0,06
GPRC5B	-0,95	0,06	1,00
GPS1	0,03	-0,25	-0,28
GPS2	0,03	-0,63	-0,66
GPT	2,28	6,37	4,08
GPT2	-0,31	0,47	0,78
GPX1	0,17	0,30	0,13
GPX3	-1,19	4,30	5,48

GPX4	0,58	1,63	1,05
GRAMD1A	0,77	0,53	-0,23
GRAMD2	0,69	3,59	2,90
GRAMD3	-1,15	1,15	2,30
GRB2	-0,43	-0,96	-0,53
GRHPR	-0,14	-0,15	-0,01
GRIA3	0,24	-0,19	-0,43
GRK4	-0,13	-0,74	-0,61
GRK6	-0,15	-0,68	-0,53
GRLF1	0,58	0,44	-0,14
GRM2	-0,43	-1,73	-1,29
GRN	-0,27	0,49	0,77
GRP	0,75	-0,12	-0,88
GRPEL1	0,59	-0,33	-0,92
GRPEL2	0,36	0,61	0,25
GRPR	1,29	-0,17	-1,46
GRSF1	0,44	0,54	0,10
GRTP1	-1,26	-0,87	0,39
GRWD1	0,20	0,65	0,44
GSDM1	0,22	0,75	0,53
GSDMDC1	0,03	-1,08	-1,11
GSDML	-0,31	-1,81	-1,50
GSG1	-0,15	0,47	0,62
GSN	-0,49	3,01	3,50
GSPT1	0,08	0,67	0,59
GSPT2	0,34	1,70	1,36
GSR	-0,55	0,22	0,77
GSS	-0,62	0,02	0,64
GSTA4	0,02	-0,30	-0,31
GSTCD	0,19	0,10	-0,09
GSTK1	0,51	0,26	-0,26
GSTM2	-0,90	-0,81	0,09
GSTM4	-0,40	1,74	2,14
GSTO2	-0,48	-0,59	-0,11
GSTT1	2,32	4,71	2,39
GTDC1	0,65	1,65	1,00
GTF2B	0,42	-0,28	-0,70
GTF2E1	0,40	1,67	1,28
GTF2E2	0,35	0,72	0,37
GTF2F2	0,11	0,21	0,11
GTF2H1	0,75	1,09	0,35
GTF2H2	0,13	0,32	0,19
GTF2H3	-0,93	0,87	1,80
GTF2H4	-0,12	-0,36	-0,24
GTF2IRD1	0,30	2,47	2,17
GTF3C1	0,18	0,03	-0,14
GTF3C2	0,11	0,33	0,22
GTF3C3	-0,11	-0,12	-0,01
GTF3C4	-0,29	-0,06	0,24
GTPBP1	3,21	-2,11	-5,32
GTPBP2	-0,03	-0,06	-0,03
GTPBP5	0,75	0,90	0,15
GTPBP6	0,08	-0,21	-0,29
GTSE1	-0,32	-0,06	0,26
GUCA1A	5,54	5,48	-0,06
GUCA2A	0,10	1,30	1,19
GUK1	0,02	0,41	0,39
GUSBL2	-0,10	-0,69	-0,58
GYPC	-1,47	-1,00	0,47

GYS1	0,06	-0,37	-0,43
GYS2	-0,14	0,38	0,52
GZMB	-1,47	-0,07	1,40
H1F0	-1,27	-1,16	0,11
H1FNT	0,54	-0,47	-1,01
H2AFV	0,13	-0,10	-0,23
H2AFY	-0,22	-0,39	-0,17
H2AFZ	-0,21	0,24	0,45
H2BFWT	0,30	2,32	2,02
H3F3A	0,06	-0,36	-0,42
H3F3B	0,85	-2,73	-3,58
HADHA	0,02	-0,21	-0,23
HAGH	0,16	1,09	0,93
HAL	-0,99	-3,14	-2,15
HAMP	-0,99	7,17	8,15
HAO2	1,24	3,74	2,50
HARS	-0,14	0,25	0,39
HAVCR2	0,25	2,32	2,07
HAX1	-0,34	0,29	0,63
HBA2	0,06	0,59	0,53
HBB	3,14	-1,80	-4,94
HBD	-0,50	-0,18	0,32
HBEGF	-2,69	-2,11	0,58
HBP1	0,23	-0,15	-0,38
HBS1L	0,66	-1,43	-2,09
HCCA2	0,17	-0,41	-0,58
HCCS	0,27	1,04	0,77
HCFC1R1	0,04	0,79	0,74
HCG18	0,21	-0,42	-0,63
HCK	-0,03	-1,03	-0,99
HCLS1	-0,15	-0,87	-0,72
HCN3	0,21	1,36	1,15
HCST	-0,51	0,17	0,68
HDAC1	0,27	-0,55	-0,81
HDAC11	0,22	0,36	0,14
HDAC2	0,80	0,63	-0,17
HDAC3	0,43	0,01	-0,42
HDAC6	0,39	-0,02	-0,41
HDAC7A	-0,47	-2,29	-1,82
HDAC8	-0,84	0,57	1,41
HDAC9	0,50	-1,50	-2,00
HDDC2	-1,79	-1,42	0,37
HDDC3	0,08	0,66	0,58
HDGF	0,07	-0,51	-0,58
HDGF2	0,45	0,63	0,19
HDHD1A	-0,47	-0,70	-0,23
HDLBP	-0,17	0,17	0,35
HEATR1	-0,15	0,26	0,41
HECA	0,33	-1,12	-1,45
HECTD3	0,29	1,12	0,83
HECW2	-2,41	-4,35	-1,94
HEL308	-0,03	-0,53	-0,50
HELZ	0,14	-1,64	-1,77
HEMGN	0,39	0,24	-0,15
HEMK1	0,58	0,70	0,12
HERC6	-0,73	-1,28	-0,55
HERPUD1	0,21	-0,61	-0,82
HERPUD2	0,85	0,24	-0,61
HES3	0,26	0,92	0,66

HES6	1,78	2,97	1,19
HEXA	-0,13	1,46	1,59
HEXB	-0,29	1,08	1,37
HEXDC	0,84	-1,05	-1,89
HFE	-2,81	-1,75	1,06
HFE2	-0,20	3,10	3,30
HGD	0,07	-0,13	-0,20
HGF	-4,17	-5,74	-1,57
HGS	0,09	-0,44	-0,53
HHEX	0,15	-1,72	-1,87
HHLA2	1,17	0,38	-0,79
HHLA3	-1,60	3,32	4,92
HIATL1	0,34	0,07	-0,27
HIF1A	0,40	-1,16	-1,57
HIF1AN	-0,19	0,50	0,70
HIF3A	-0,10	-0,31	-0,21
HIG2	-0,14	1,71	1,85
HIGD1A	-0,12	1,20	1,32
HIGD1B	0,42	3,95	3,53
HIGD2A	0,30	-0,23	-0,52
HINT1	-0,10	0,42	0,52
HINT2	-0,03	0,23	0,26
HINT3	0,10	1,72	1,62
HIP1	0,72	-0,29	-1,01
HIP2	-0,12	0,70	0,82
HIPK1	3,36	-1,22	-4,58
HIPK2	-0,02	0,20	0,21
HIRA	0,42	0,07	-0,35
HIRIP3	-0,29	0,32	0,60
HIST1H1C	-1,22	-0,42	0,80
HIST1H2AH	0,10	0,16	0,06
HIST1H2AL	0,15	-0,25	-0,40
HIST1H2BK	-0,01	0,38	0,39
HIST1H2BO	-0,30	-0,31	-0,01
HIST1H3F	-0,18	-0,05	0,14
HIST1H3G	-0,07	-0,14	-0,07
HIST1H3I	0,71	-0,05	-0,76
HIST1H4C	-1,58	-0,48	1,10
HIST1H4E	-3,12	-3,79	-0,67
HIST2H2AB	0,59	-1,51	-2,11
HIST2H2AC	-0,08	-1,33	-1,25
HIST3H2BB	0,16	-0,21	-0,37
HIVEP1	-1,05	-1,80	-0,76
HIVEP2	0,21	-0,86	-1,07
HK2	-1,10	-0,19	0,91
HK3	-1,92	-1,57	0,35
HKDC1	0,46	0,65	0,20
HKR1	0,08	-0,38	-0,46
HLA-A	-0,53	-0,55	-0,02
HLA-DMA	-0,16	0,55	0,71
HLA-DMB	-0,01	0,23	0,24
HLA-DPA1	0,43	1,01	0,58
HLA-DPB1	0,32	0,39	0,08
HLA-DQA1	-0,02	1,44	1,46
HLA-DQA2	-0,29	0,29	0,58
HLA-DQB1	-0,41	0,30	0,71
HLA-DQB2	1,46	-0,36	-1,82
HLA-DRA	-0,04	0,35	0,39
HLA-DRB1	0,36	0,89	0,53

HLA-G	-0,71	-1,16	-0,45
HLCS	-0,40	3,95	4,35
HMBOX1	0,55	-0,96	-1,50
HMBS	-0,11	0,39	0,50
HMG2L1	-0,60	-0,26	0,34
HMGB1	0,73	-0,41	-1,14
HMGB3	-0,13	-0,05	0,08
HMGCR	-0,32	1,31	1,62
HMGCS1	1,00	2,86	1,86
HMGCS2	0,31	1,77	1,47
HMMR	-3,44	7,71	11,16
HMOX1	0,14	-0,98	-1,12
HMP19	-0,33	-0,12	0,21
HNF4G	0,06	-0,16	-0,22
HNMT	-0,28	-0,14	0,14
HNRPA1	-0,16	-0,11	0,05
HNRPA3	-0,25	-0,25	0,00
HNRPC	0,27	0,11	-0,16
HNRPD	0,32	-0,42	-0,74
HNRPDL	0,08	-1,56	-1,64
HNRPH1	-0,16	-1,23	-1,07
HNRPH2	0,21	-0,53	-0,74
HNRPH3	0,25	-0,82	-1,07
HNRPK	-0,09	-0,08	0,00
HNRPLL	0,04	0,72	0,68
HNRPU	0,10	-1,70	-1,80
HNRPUL1	-0,38	-0,48	-0,10
HOM-TES-103	0,24	-0,77	-1,01
HOMER1	-1,05	2,88	3,93
HOMER3	-0,60	-1,35	-0,75
HOOK2	0,81	-0,17	-0,98
HOOK3	0,51	-0,26	-0,77
HOP	1,44	2,71	1,26
HOXA2	0,62	0,44	-0,18
HOXA5	3,80	-3,23	-7,02
HOXA6	0,18	-0,12	-0,31
HOXB1	0,10	-0,38	-0,48
HOXB3	-0,94	-1,86	-0,92
HOXC13	0,04	0,04	0,00
HOXC8	1,48	-0,18	-1,66
HP	0,37	-2,99	-3,35
HP1BP3	0,40	-0,37	-0,77
HPR	0,69	0,67	-0,02
HPRT1	0,21	0,40	0,18
HPS3	0,46	0,57	0,11
HPS4	-0,44	-0,13	0,31
HPS5	0,55	2,18	1,64
HPSE	-4,92	-10,48	-5,56
HRES1	4,02	2,78	-1,24
HRG	0,47	1,24	0,77
HRH1	2,38	5,87	3,49
HRH2	-0,01	-0,37	-0,36
HRSP12	-0,62	0,24	0,86
HS2ST1	0,20	0,31	0,11
HS322B1A	2,00	1,98	-0,02
HS3ST1	5,40	7,00	1,60
HS3ST2	1,34	13,34	12,00
HSBP1	0,22	-1,47	-1,69
HSD11B1	-0,64	14,11	14,75

HSD11B1L	-0,07	0,18	0,24
HSD17B13	0,00	-7,25	-7,25
HSD17B2	0,24	0,55	0,31
HSD17B8	0,11	0,07	-0,05
HSD3B2	0,55	0,13	-0,41
HSD3B7	-0,95	4,18	5,12
HSDL1	0,17	0,19	0,02
HSDL2	0,15	-0,71	-0,86
HSF1	0,55	-0,13	-0,68
HSF2	0,19	-0,36	-0,55
HSF2BP	-0,86	1,61	2,47
HSH2D	8,14	-3,02	-11,16
HSN2	0,16	-2,43	-2,59
HSP90AA1	-0,06	0,47	0,52
HSP90AB6P	-0,47	2,79	3,26
HSPA14	-0,03	-0,05	-0,02
HSPA1A	0,01	-0,51	-0,52
HSPA1B	0,65	1,61	0,96
HSPA1L	0,01	-2,34	-2,36
HSPA4	0,23	0,85	0,62
HSPA5	-0,91	-1,60	-0,69
HSPA8	0,02	0,99	0,98
HSPB2	-0,28	0,12	0,40
HSPB7	0,59	-0,47	-1,06
HSPBP1	-0,36	-0,26	0,10
HSPC047	0,74	-0,90	-1,64
HSPC111	0,17	0,27	0,10
HSPC152	0,17	0,02	-0,15
HSPC171	-0,46	0,22	0,68
HSPD1	-0,09	0,89	0,99
HSPE1	0,21	1,01	0,79
HSPH1	0,86	2,66	1,81
HTATIP2	-0,18	1,08	1,25
HTATSF1	-0,30	-0,26	0,04
HTF9C	0,19	-0,06	-0,26
HTN3	-0,58	-1,63	-1,06
HTR1F	-1,49	-1,60	-0,11
HTR3A	0,93	0,96	0,02
HTR3B	0,39	-1,08	-1,48
HTRA2	-0,22	-0,10	0,12
HTRA4	-1,35	0,89	2,24
HUS1B	0,28	-0,29	-0,57
HUWE1	-0,06	-0,20	-0,14
HVCN1	-1,52	-1,19	0,33
HYAL1	0,29	0,19	-0,10
HYAL3	0,97	1,80	0,83
HYI	-0,38	0,14	0,51
HYLS1	1,29	1,18	-0,12
HYOU1	-0,16	0,31	0,48
HYPK	1,55	-1,42	-2,97
IARS	-0,51	1,99	2,49
IBRDC3	1,16	-0,17	-1,33
IBTK	0,30	0,51	0,21
ICA1	-0,13	-1,49	-1,36
ICAM2	-1,33	-4,13	-2,81
ICAM3	0,11	-3,46	-3,57
ICAM4	0,01	-0,51	-0,52
ICF45	0,03	-1,05	-1,07
ICK	0,50	-0,86	-1,36

ICMT	0,09	1,54	1,45
ICOSLG	0,71	1,97	1,26
ICT1	-0,31	-0,03	0,28
ID2	0,83	-0,27	-1,10
ID2B	-0,10	-0,93	-0,83
ID3	-1,59	0,62	2,21
IDH3A	0,06	-0,37	-0,43
IDH3B	-0,19	-0,55	-0,36
IDH3G	0,10	-0,27	-0,38
IDI1	0,35	2,07	1,72
IDS	-0,48	-0,28	0,20
IDUA	0,04	-0,69	-0,74
IER2	-0,02	-2,05	-2,03
IER3	-0,33	-0,80	-0,47
IER5	0,39	-0,89	-1,28
IFI16	-0,05	-0,46	-0,40
IFI30	0,13	0,20	0,07
IFI44L	-0,86	-5,37	-4,51
IFIH1	0,36	0,44	0,08
IFIT1	-3,37	-3,52	-0,14
IFIT2	-1,74	-2,37	-0,63
IFIT3	-3,00	-1,97	1,02
IFITM1	0,93	-4,24	-5,16
IFITM5	0,52	-0,63	-1,15
IFNA13	0,16	-0,12	-0,28
IFNAR2	0,12	0,05	-0,07
IFNGR1	0,34	-0,09	-0,43
IFNGR2	1,08	0,89	-0,18
IFP38	0,47	-1,12	-1,59
IFRD1	0,17	-0,85	-1,02
IFRD2	0,11	0,81	0,70
IFRG15	-0,28	0,93	1,21
IFT122	-1,53	-0,67	0,86
IFT140	-0,28	2,27	2,55
IFT52	-0,56	-0,75	-0,19
IFT74	-0,18	-0,04	0,15
IFT80	0,38	-0,71	-1,09
IFT88	-0,48	-0,67	-0,19
IGBP1	0,27	-0,56	-0,82
IGFALS	0,48	0,83	0,35
IGFL3	-0,70	-0,50	0,19
IGHMBP2	-0,07	-0,08	-0,01
IGSF11	-0,43	0,48	0,91
IGSF2	-0,75	-2,40	-1,66
IGSF6	0,30	1,51	1,21
IGSF8	-1,79	-0,91	0,88
IHPK3	0,47	0,92	0,45
IK	-0,26	-1,11	-0,86
IKBKG	0,22	-0,27	-0,49
IKIP	-0,10	0,02	0,12
IL10RB	-0,66	-0,48	0,17
IL11RA	-0,47	-1,99	-1,52
IL12A	1,57	-1,23	-2,80
IL12B	1,11	0,56	-0,55
IL13RA1	0,51	-0,36	-0,88
IL15	-0,39	-3,46	-3,07
IL16	0,87	-1,51	-2,38
IL17F	0,73	-0,27	-1,00
IL17RB	0,22	11,62	11,40

IL17RE	-0,21	-0,93	-0,72
IL18	0,09	-0,76	-0,85
IL18R1	3,22	5,42	2,20
IL18RAP	0,05	-0,49	-0,55
IL19	2,24	0,68	-1,56
IL1A	-2,48	7,69	10,17
IL1F10	-0,20	-0,17	0,03
IL1F5	-0,25	0,09	0,34
IL1F8	-5,70	1,07	6,77
IL1R1	2,43	4,26	1,83
IL1R2	3,86	1,53	-2,33
IL1RAPL1	-0,59	-0,46	0,13
IL1RL2	4,49	0,57	-3,92
IL1RN	-0,82	3,25	4,07
IL21	1,72	1,28	-0,44
IL21R	0,64	3,09	2,44
IL22RA2	3,47	5,14	1,67
IL26	0,01	0,28	0,27
IL28A	-0,79	-1,61	-0,82
IL2RA	-6,82	-2,98	3,84
IL2RG	-0,99	0,47	1,46
IL3RA	0,31	2,73	2,42
IL4I1	1,74	4,82	3,08
IL6R	0,56	-1,23	-1,79
IL7R	-0,96	4,96	5,93
IL8	-3,24	-1,44	1,81
IL8RB	-1,17	-1,86	-0,69
ILF2	-0,03	0,37	0,40
ILK	-0,08	-0,30	-0,22
ILVBL	-0,31	1,13	1,44
IMMP2L	1,23	1,39	0,16
IMMT	0,21	0,45	0,24
IMP3	0,28	0,32	0,04
IMP4	-0,07	-0,01	0,06
IMPA2	-1,56	-4,01	-2,45
IMPACT	-0,02	3,16	3,18
IMPAD1	-0,10	1,07	1,17
IMPDH2	0,10	0,00	-0,09
INCA	-3,13	-6,89	-3,76
INCENP	0,38	0,88	0,49
INDO	1,00	0,28	-0,72
INDOL1	0,10	0,25	0,14
ING1	0,29	-1,43	-1,72
ING2	-0,03	-0,25	-0,22
ING4	0,27	-1,19	-1,46
INHBE	1,79	2,32	0,53
INPP4A	-0,14	-1,09	-0,95
INPP4B	-0,33	-0,33	0,00
INPP5D	1,27	-0,55	-1,82
INPP5E	0,76	-0,36	-1,12
INPP5F	-0,17	-0,18	-0,02
INSIG1	-0,22	2,82	3,04
INTS12	-0,18	-0,14	0,04
INTS4	-0,65	-0,71	-0,05
INTS5	0,27	0,96	0,69
INTS6	0,09	0,44	0,35
INTS7	-0,12	-0,02	0,10
INTS8	0,48	-0,96	-1,45
INVS	-0,04	0,49	0,53

IPO11	-0,40	0,67	1,07
IPO13	0,80	1,80	1,00
IPO4	0,53	0,34	-0,18
IPO8	-0,64	-0,73	-0,09
IPO9	0,02	-0,12	-0,15
IPP	0,10	-0,30	-0,40
IPPK	0,16	2,03	1,87
IQCG	0,02	1,41	1,40
IQCH	-0,02	-0,51	-0,49
IQSEC1	-0,56	-1,20	-0,64
IQWD1	-0,33	0,31	0,64
IRAK4	0,35	-1,46	-1,81
IREB2	0,43	-0,14	-0,56
IRF1	-0,50	-2,41	-1,91
IRF2	0,10	-0,53	-0,64
IRF2BP1	-0,07	-0,65	-0,58
IRF3	0,26	-0,42	-0,68
IRF8	-0,35	-0,60	-0,25
IRGQ	-0,26	0,72	0,98
IRS2	-0,81	-3,60	-2,78
ISG20L1	0,45	0,82	0,38
ISG20L2	0,28	-0,62	-0,90
ISLR	1,46	1,75	0,29
ISOC1	0,06	1,61	1,55
ISYNA1	2,29	4,71	2,41
ITFG1	-0,07	0,87	0,94
ITGA10	-0,27	-1,06	-0,79
ITGA2B	1,60	1,21	-0,39
ITGA4	0,13	-3,00	-3,14
ITGAD	1,50	1,75	0,26
ITGAE	0,70	0,17	-0,53
ITGAM	0,25	1,15	0,90
ITGAV	-0,24	1,16	1,40
ITGAX	-0,09	0,54	0,62
ITGB1	0,09	0,96	0,87
ITGB1BP1	-1,12	1,06	2,19
ITGB1BP2	-0,19	-1,07	-0,89
ITGB3	-0,09	0,13	0,21
ITGB3BP	-0,17	-0,09	0,08
ITGB4	-0,48	0,67	1,15
ITGB7	-4,03	-6,49	-2,46
ITGBL1	-2,38	-0,82	1,56
ITIH4	-0,32	-2,70	-2,39
ITIH5L	1,02	0,47	-0,55
ITPA	-0,14	0,50	0,63
ITPKA	0,26	0,72	0,47
ITPKC	-0,60	0,02	0,62
ITPR2	-0,39	0,50	0,89
IVL	-0,81	-0,14	0,67
IVNS1ABP	-0,11	-0,46	-0,36
IWS1	-0,04	-0,05	-0,01
JAG1	0,96	1,16	0,20
JAGN1	-0,36	-0,33	0,03
JAK1	0,40	-0,34	-0,73
JAK2	0,92	-1,12	-2,03
JAKMIP2	-3,38	2,83	6,21
JARID1A	0,27	-0,28	-0,55
JAZF1	-0,18	-2,52	-2,34
JMJD1A	0,23	-0,03	-0,26

JMJD1B	0,36	-0,73	-1,09
JMJD2C	0,33	-1,21	-1,55
JMJD2D	-0,65	1,25	1,89
JMJD4	0,18	0,80	0,62
JMJD5	0,50	0,80	0,30
JMY	-0,44	-2,77	-2,32
JOSD1	-0,19	0,02	0,21
JOSD2	-0,11	-0,11	0,01
JPH4	-0,58	-0,56	0,02
JRK	0,22	0,81	0,59
JRKL	0,12	0,04	-0,08
JTB	-0,06	-0,04	0,03
JTV1	0,02	0,33	0,31
JUB	-0,68	-0,04	0,64
JUN	0,41	2,22	1,81
JUNB	0,78	-3,47	-4,25
JUND	0,32	-0,10	-0,42
JUP	0,57	0,18	-0,39
KAL1	-3,14	8,12	11,27
KARS	-0,06	-0,36	-0,30
KATNB1	-0,48	-0,39	0,09
KBTBD11	-0,16	-3,30	-3,15
KBTBD2	-0,12	-0,68	-0,55
KBTBD3	-0,30	-0,05	0,25
KBTBD6	0,53	0,57	0,04
KBTBD8	0,15	3,05	2,90
KCMF1	-0,26	-0,23	0,03
KCNA10	-0,36	0,99	1,35
KCNA3	-0,67	-1,45	-0,78
KCNAB1	-1,42	2,67	4,09
KCNAB2	-0,26	0,69	0,95
KCNE1L	0,11	0,37	0,26
KCNG1	0,13	0,00	-0,14
KCNG4	0,49	0,94	0,45
KCNH6	-1,24	-0,92	0,32
KCNIP1	-0,10	0,39	0,49
KCNJ1	-3,73	5,24	8,97
KCNJ14	-0,79	-0,45	0,34
KCNJ16	-0,19	-0,44	-0,25
KCNJ4	0,05	-0,03	-0,08
KCNJ5	-2,51	7,68	10,19
KCNK18	0,07	1,00	0,93
KCNK6	1,45	1,82	0,37
KCNK9	-0,04	-0,17	-0,12
KCNMA1	-1,62	0,31	1,93
KCNMB1	-1,65	-1,46	0,19
KCNMB2	-1,16	2,40	3,57
KCNQ1	0,40	-0,04	-0,44
KCNRG	0,73	-0,36	-1,09
KCNV2	-0,33	-0,30	0,03
KCTD10	0,19	0,96	0,77
KCTD12	-0,66	-1,54	-0,88
KCTD2	-0,02	0,07	0,09
KCTD5	-0,14	0,95	1,09
KCTD6	1,64	0,80	-0,84
KCTD9	-0,24	1,50	1,75
KDELR2	-0,45	0,15	0,60
KEL	-8,19	0,00	8,19
KERA	-1,46	-2,19	-0,73

KHK	-0,47	0,28	0,75
KIAA0020	0,21	0,04	-0,17
KIAA0090	-0,13	0,66	0,79
KIAA0100	1,54	1,71	0,17
KIAA0101	-4,29	0,53	4,82
KIAA0133	-0,09	-0,81	-0,72
KIAA0143	0,30	0,46	0,16
KIAA0152	-0,24	1,56	1,80
KIAA0157	0,29	0,72	0,44
KIAA0179	2,35	2,82	0,47
KIAA0195	-0,09	-0,15	-0,06
KIAA0196	-0,35	0,36	0,71
KIAA0232	0,54	0,03	-0,52
KIAA0240	0,14	-1,50	-1,65
KIAA0241	-0,20	0,89	1,10
KIAA0247	-0,04	-0,14	-0,10
KIAA0251	-0,60	0,50	1,10
KIAA0258	0,21	0,69	0,47
KIAA0286	0,45	1,36	0,91
KIAA0319L	0,08	-1,19	-1,27
KIAA0323	-0,04	0,30	0,33
KIAA0329	-0,02	-0,02	0,00
KIAA0355	0,69	-0,18	-0,87
KIAA0367	-3,00	-4,64	-1,63
KIAA0372	-0,12	0,19	0,31
KIAA0391	-0,22	0,97	1,19
KIAA0406	-0,18	-0,52	-0,35
KIAA0408	0,84	-1,21	-2,04
KIAA0409	0,06	0,09	0,03
KIAA0423	0,55	0,82	0,27
KIAA0427	0,45	-0,85	-1,30
KIAA0460	0,57	-0,37	-0,95
KIAA0513	-0,35	-1,64	-1,29
KIAA0528	-0,67	-1,09	-0,42
KIAA0556	0,19	-0,31	-0,49
KIAA0586	-0,78	0,65	1,43
KIAA0652	-0,07	-0,48	-0,41
KIAA0664	0,53	1,17	0,64
KIAA0701	0,31	-0,84	-1,14
KIAA0738	-1,07	1,49	2,55
KIAA0746	-1,21	3,83	5,04
KIAA0753	-0,07	-0,47	-0,39
KIAA0802	4,30	1,78	-2,52
KIAA0828	0,40	-0,21	-0,61
KIAA0831	0,57	-0,14	-0,72
KIAA0889	0,41	0,02	-0,39
KIAA0892	0,31	-1,05	-1,36
KIAA0907	0,43	-0,12	-0,55
KIAA0922	-0,14	-3,19	-3,04
KIAA1009	-0,85	-0,62	0,23
KIAA1012	0,03	-0,20	-0,23
KIAA1024	1,02	1,46	0,44
KIAA1026	0,08	0,22	0,14
KIAA1128	0,08	0,04	-0,05
KIAA1143	0,01	-0,60	-0,61
KIAA1160	-0,07	-0,61	-0,54
KIAA1199	-7,54	-3,81	3,73
KIAA1219	0,19	-0,35	-0,54
KIAA1244	-1,72	-0,27	1,45

KIAA1267	0,19	-1,98	-2,17
KIAA1279	0,26	2,85	2,59
KIAA1324L	-0,50	-0,59	-0,09
KIAA1328	-0,27	-0,66	-0,39
KIAA1344	0,28	-0,94	-1,22
KIAA1407	0,26	0,22	-0,04
KIAA1429	-0,38	-0,63	-0,25
KIAA1432	0,23	-1,04	-1,27
KIAA1467	-0,29	1,35	1,63
KIAA1468	0,27	-0,85	-1,12
KIAA1505	1,31	1,42	0,12
KIAA1522	0,14	1,76	1,62
KIAA1524	-1,28	0,34	1,62
KIAA1530	0,67	-0,42	-1,09
KIAA1604	0,29	-0,65	-0,94
KIAA1627	0,26	-0,03	-0,29
KIAA1632	0,28	0,66	0,38
KIAA1704	-0,41	-0,07	0,34
KIAA1706	-0,12	1,02	1,15
KIAA1715	-0,02	0,44	0,46
KIAA1727	-0,03	-1,28	-1,25
KIAA1729	0,45	0,27	-0,18
KIAA1737	0,68	1,85	1,17
KIAA1754	-0,78	-0,70	0,08
KIAA1754L	2,77	9,02	6,25
KIAA1799	-0,90	-0,98	-0,08
KIAA1826	0,41	1,61	1,20
KIAA1875	0,43	-1,00	-1,42
KIAA1913	9,12	9,12	0,00
KIAA1967	0,20	-0,41	-0,61
KIAA2018	0,05	-1,55	-1,60
KIAA2026	-0,03	-1,19	-1,16
KIF13A	-0,30	-1,71	-1,41
KIF14	-2,54	-0,62	1,92
KIF15	-6,25	2,49	8,74
KIF18A	-0,41	-0,65	-0,24
KIF1C	0,32	1,03	0,71
KIF20A	-7,63	2,58	10,21
KIF24	-0,95	1,43	2,38
KIF25	0,56	0,26	-0,31
KIF4A	-3,46	-1,28	2,18
KIF5B	0,60	0,94	0,33
KIFAP3	0,39	1,01	0,63
KIFC1	0,31	-0,36	-0,67
KIR2DL1	-0,05	-0,37	-0,32
KIR2DS3	0,43	-0,13	-0,56
KIR2DS4	0,11	0,69	0,58
KIRREL3	0,44	0,01	-0,44
KLC3	-0,22	-0,59	-0,37
KLC4	-0,13	-0,11	0,02
KLF1	-0,25	0,01	0,26
KLF10	-0,76	-4,27	-3,51
KLF13	0,39	-1,63	-2,02
KLF15	0,03	-0,16	-0,19
KLF3	0,32	0,09	-0,23
KLF6	0,20	-0,23	-0,43
KLF7	0,14	-0,77	-0,91
KLF9	-0,77	-0,68	0,08
KLHDC3	0,09	-0,08	-0,16

KLHDC4	0,01	-0,75	-0,76
KLHDC5	-0,20	0,41	0,61
KLHL12	-0,24	0,43	0,68
KLHL15	0,07	-2,08	-2,14
KLHL18	0,14	-0,87	-1,00
KLHL2	-0,49	0,27	0,76
KLHL22	-0,08	0,63	0,71
KLHL24	0,51	-0,63	-1,14
KLHL25	0,23	-0,09	-0,32
KLHL5	0,03	0,10	0,07
KLHL7	0,17	0,26	0,09
KLK1	0,76	-1,34	-2,10
KLK15	0,10	-0,62	-0,72
KLK8	0,58	0,03	-0,55
KMO	-0,30	0,57	0,87
KNG1	0,54	0,19	-0,35
KNTC1	-0,77	0,71	1,48
KPNA1	-0,19	0,16	0,35
KPNA2	-0,27	0,51	0,79
KPNA3	0,21	0,43	0,22
KPNA4	0,62	0,92	0,30
KPNA5	0,56	1,05	0,50
KPNB1	0,11	0,42	0,31
KPTN	0,08	-0,96	-1,04
KREMEN1	-0,66	-0,38	0,28
KRIT1	0,36	-0,36	-0,72
KRT10	-0,58	-0,97	-0,39
KRT17	2,05	4,35	2,31
KRT23	-2,89	-5,47	-2,59
KRT24	-0,05	-0,02	0,03
KRT3	-0,23	-0,41	-0,19
KRT6B	0,08	0,06	-0,02
KRTAP10-10	0,51	0,37	-0,15
KRTAP10-2	-0,69	-0,26	0,44
KRTAP10-8	-1,25	-1,55	-0,30
KRTAP12-1	-1,19	-1,23	-0,04
KRTAP12-4	0,16	0,47	0,30
KRTAP13-1	0,62	0,52	-0,10
KRTAP13-4	-0,93	0,54	1,47
KRTAP21-1	-0,02	0,29	0,32
KRTAP3-2	0,23	-0,60	-0,83
KRTAP5-1	-0,33	-0,53	-0,20
KRTAP6-1	-0,06	-0,76	-0,70
KRTAP8-1	-0,40	0,17	0,57
KRTAP9-3	0,22	0,50	0,28
KRTAP9-4	0,20	0,08	-0,12
KSR1	-0,48	-0,54	-0,06
KSR2	-0,31	-0,43	-0,12
KTI12	0,01	-0,19	-0,20
KYNU	-1,00	-0,07	0,93
KUA-UEV	0,14	0,69	0,54
L1CAM	0,55	0,29	-0,27
L2HGDH	-0,85	1,65	2,49
L3MBTL3	0,00	-2,45	-2,45
LACE1	-0,13	1,15	1,28
LACRT	-0,02	0,50	0,51
LACTB2	-0,47	0,91	1,38
LAIR2	-2,19	-0,01	2,18
LAMA2	-0,28	0,03	0,31

LAMA3	-1,53	-0,38	1,14
LAMB2	-0,05	0,88	0,93
LAMC2	-0,29	-0,14	0,15
LAMP1	-0,07	1,32	1,39
LANCL1	-0,21	-0,58	-0,37
LANCL2	0,32	-0,31	-0,63
LAP3	0,49	0,94	0,45
LAPTM4B	-1,06	1,30	2,36
LARP1	0,42	0,18	-0,25
LARP2	0,35	-0,05	-0,40
LARP4	0,41	0,89	0,48
LARP5	0,26	-0,58	-0,84
LARS2	-0,36	1,28	1,64
LAS1L	-0,40	-0,95	-0,54
LATS2	0,30	-0,84	-1,13
LBP	-0,79	-0,91	-0,11
LBR	-0,29	-0,88	-0,59
LCAT	-2,93	-5,76	-2,83
LCE1A	-1,45	-0,50	0,95
LCE1D	-1,17	-0,43	0,74
LCE3B	-0,29	0,03	0,32
LCE3C	0,57	0,32	-0,24
LCMT1	-0,09	0,59	0,68
LCMT2	0,11	1,65	1,54
LCN1	-0,64	0,58	1,22
LCN2	-0,10	0,38	0,48
LCN8	-0,36	-0,49	-0,12
LCP1	-0,13	-0,03	0,10
LCP2	-1,32	-1,71	-0,39
LCT	-0,03	-0,19	-0,16
LDB1	0,08	0,06	-0,02
LDHB	-0,24	0,32	0,56
LDHD	0,16	3,42	3,26
LDLR	-0,21	1,94	2,15
LDLRAP1	-1,70	1,08	2,78
LEFTY2	0,07	0,51	0,44
LELP1	0,20	0,69	0,49
LEMD2	0,21	-0,38	-0,59
LENG1	0,14	-0,06	-0,20
LENG8	-0,09	-1,26	-1,17
LEPR	-2,76	-1,59	1,17
LEPRE1	0,70	0,29	-0,40
LEPROT	-0,56	0,01	0,57
LETMD1	-0,03	-1,11	-1,08
LGALS12	-1,68	-4,03	-2,35
LGALS3	0,35	1,78	1,43
LGALS4	1,84	0,39	-1,45
LGALS8	-0,49	-1,10	-0,61
LGALS9	-0,47	0,01	0,48
LGMN	-1,30	3,73	5,03
LGR6	-0,59	0,03	0,62
LGTN	-0,31	-0,86	-0,54
LHFPL1	-0,02	0,31	0,33
LHFPL2	-1,34	2,46	3,80
LIAS	0,49	0,33	-0,16
LIG1	-0,83	1,37	2,20
LIG3	-0,09	-0,53	-0,44
LIG4	0,01	0,31	0,30
LILRA1	0,26	-2,23	-2,49

LILRA2	-0,36	-1,17	-0,81
LILRA4	-0,45	0,18	0,64
LILRA6	-0,57	-1,01	-0,43
LILRB4	-0,74	0,58	1,32
LILRB5	-1,03	1,59	2,62
LIMA1	0,72	4,76	4,03
LIMK2	-0,59	-0,90	-0,30
LIMS1	0,66	2,28	1,62
LIMS3	0,25	-0,31	-0,57
LIN7C	-0,23	-0,10	0,13
LIN9	-0,42	0,29	0,72
LINS1	-0,12	0,20	0,32
LIPA	0,32	2,53	2,22
LIPT1	0,37	-1,05	-1,42
LITAF	0,04	1,42	1,37
LMAN2	0,03	0,00	-0,04
LMAN2L	-0,44	1,02	1,45
LMBR1	0,12	0,77	0,65
LMBR1L	0,95	0,70	-0,25
LMBRD2	-0,52	0,64	1,16
LMLN	0,15	2,74	2,59
LMO4	-0,45	-0,41	0,04
LMO6	1,26	-0,43	-1,69
LNPEP	-0,75	0,50	1,25
LOC113386	0,09	0,41	0,32
LOC124216	0,49	-1,28	-1,77
LOC124220	0,66	0,36	-0,30
LOC124446	-0,12	-0,05	0,06
LOC124512	-0,03	0,09	0,12
LOC128977	0,16	0,29	0,13
LOC130074	0,58	0,44	-0,13
LOC130355	-0,21	-0,07	0,14
LOC134145	0,08	0,36	0,29
LOC148137	-0,07	-0,55	-0,48
LOC153222	1,05	-1,04	-2,09
LOC153328	2,96	6,33	3,37
LOC153364	-0,30	-0,47	-0,17
LOC153561	0,96	-1,99	-2,95
LOC158572	-0,48	-0,22	0,26
LOC161247	0,03	-1,07	-1,10
LOC165186	0,29	0,15	-0,14
LOC196549	0,39	-6,53	-6,92
LOC196752	0,86	-1,17	-2,03
LOC197135	0,42	0,40	-0,02
LOC201164	0,73	1,26	0,54
LOC201175	0,62	-1,58	-2,20
LOC201181	-0,94	3,15	4,09
LOC201725	-0,02	-1,44	-1,42
LOC202459	-2,68	-3,04	-0,36
LOC203547	-0,03	-0,44	-0,41
LOC205251	-0,22	0,76	0,98
LOC220686	0,01	0,21	0,20
LOC221442	-0,09	-1,83	-1,74
LOC283152	-3,29	-4,56	-1,27
LOC283849	-0,66	1,12	1,78
LOC283932	0,37	0,50	0,13
LOC284009	-0,27	-0,20	0,07
LOC284757	-0,83	-1,82	-0,99
LOC284861	0,14	-0,95	-1,09

LOC284912	-0,25	0,52	0,77
LOC285033	-2,43	-2,93	-0,50
LOC285074	0,45	-1,26	-1,70
LOC285636	0,77	1,18	0,41
LOC285908	-0,05	-0,30	-0,25
LOC286016	0,03	0,29	0,26
LOC286187	0,41	0,19	-0,22
LOC338328	-0,14	-0,42	-0,28
LOC339229	0,64	-0,03	-0,67
LOC339457	0,59	2,16	1,57
LOC348262	-0,15	-0,31	-0,16
LOC349196	-0,16	-0,07	0,08
LOC374395	-0,24	-0,38	-0,14
LOC374920	-0,13	0,14	0,27
LOC387790	0,80	0,61	-0,20
LOC387882	-1,20	-0,92	0,28
LOC388284	0,81	-0,59	-1,40
LOC388438	0,55	0,35	-0,20
LOC388969	0,86	-0,81	-1,68
LOC389118	0,45	-0,11	-0,56
LOC389517	-0,17	-0,78	-0,62
LOC389607	0,42	-0,51	-0,93
LOC389641	0,12	0,05	-0,08
LOC389833	-0,23	0,11	0,34
LOC390637	-0,25	0,79	1,04
LOC391356	-0,12	-1,06	-0,94
LOC399706	-1,58	4,76	6,34
LOC399744	-0,89	-3,21	-2,32
LOC399898	0,34	-0,48	-0,82
LOC399900	-2,43	-6,75	-4,32
LOC400197	-0,25	-1,33	-1,08
LOC400506	-0,12	0,42	0,54
LOC400657	0,19	1,64	1,46
LOC400986	0,06	0,50	0,44
LOC401052	-2,14	-1,36	0,79
LOC401072	-1,39	-1,34	0,05
LOC401398	0,53	2,15	1,61
LOC401431	-0,41	1,13	1,54
LOC401620	0,84	-1,29	-2,13
LOC401622	0,38	-2,30	-2,68
LOC401623	1,35	-0,64	-1,99
LOC401720	0,18	0,26	0,08
LOC402176	-0,44	-1,33	-0,89
LOC440093	0,02	-0,85	-0,87
LOC440258	-0,14	-1,54	-1,40
LOC440354	0,50	-0,76	-1,26
LOC440731	-0,52	-1,66	-1,14
LOC440742	-0,32	-0,81	-0,49
LOC440944	0,07	-0,35	-0,41
LOC440993	-0,06	0,33	0,39
LOC441087	0,40	-0,67	-1,07
LOC441150	0,22	-1,07	-1,29
LOC441193	0,66	-1,37	-2,03
LOC441208	-0,19	-0,06	0,13
LOC441268	-1,16	-3,68	-2,53
LOC441294	0,41	1,13	0,73
LOC441763	-0,20	0,26	0,45
LOC441956	0,73	0,47	-0,25
LOC442132	0,29	0,45	0,16

LOC442535	-0,69	0,42	1,12
LOC442582	0,36	-1,27	-1,62
LOC492311	0,39	0,79	0,40
LOC493869	3,04	6,84	3,80
LOC51035	0,42	-0,66	-1,08
LOC51057	0,58	-0,12	-0,70
LOC51136	-0,36	1,20	1,57
LOC51252	3,21	1,77	-1,44
LOC54103	0,41	-0,20	-0,62
LOC552891	0,08	1,42	1,34
LOC55908	0,28	0,25	-0,03
LOC606495	-0,87	-0,52	0,35
LOC63920	0,61	4,34	3,73
LOC642370	0,25	-0,24	-0,50
LOC642852	0,56	0,36	-0,20
LOC642934	0,35	-1,58	-1,93
LOC643011	0,06	0,61	0,54
LOC643045	2,32	3,25	0,93
LOC643206	0,24	0,68	0,44
LOC643339	0,99	4,10	3,11
LOC643396	4,54	5,38	0,84
LOC643493	0,23	-1,82	-2,04
LOC644099	0,51	-0,05	-0,56
LOC644377	-0,81	-1,36	-0,54
LOC644380	0,24	-0,50	-0,75
LOC644733	0,27	0,11	-0,17
LOC644961	0,17	-0,62	-0,79
LOC644978	0,87	1,25	0,38
LOC645052	0,00	-8,28	-8,28
LOC645261	-1,08	3,43	4,51
LOC645427	-0,15	0,81	0,96
LOC645460	1,36	-0,47	-1,83
LOC645676	0,48	-0,24	-0,72
LOC646100	0,38	-1,23	-1,61
LOC646146	-0,22	7,13	7,35
LOC646345	0,00	-0,62	-0,63
LOC646407	1,41	-3,02	-4,43
LOC646496	-1,88	0,60	2,48
LOC646667	0,32	0,28	-0,04
LOC646778	-0,30	1,62	1,92
LOC646897	0,53	-0,83	-1,36
LOC647243	-0,48	2,46	2,95
LOC653240	-0,17	0,60	0,77
LOC653352	-0,19	-1,45	-1,26
LOC653566	-0,28	0,49	0,77
LOC653604	0,80	-0,88	-1,68
LOC90379	0,35	0,31	-0,05
LOC90624	-0,54	0,22	0,76
LOC90826	-0,53	1,35	1,88
LOC90835	-0,34	-0,37	-0,03
LOC90925	-0,04	-0,13	-0,09
LOC91431	-0,28	-0,91	-0,63
LOC92017	0,92	0,41	-0,51
LOC92270	-0,09	0,21	0,30
LOC92497	3,96	-1,91	-5,88
LOC96610	0,08	-0,48	-0,55
LOH11CR2A	-0,12	2,96	3,08
LONRF1	0,07	1,58	1,51
LOXL3	-0,21	-0,05	0,16

LPGAT1	0,16	-1,38	-1,53
LPIN1	0,43	2,19	1,77
LPP	1,19	1,04	-0,14
LPXN	-0,38	0,93	1,31
LRBA	-0,62	-0,33	0,29
LRCH4	-0,18	-3,05	-2,87
LRDD	-0,06	-1,05	-1,00
LRFN4	0,75	1,28	0,53
LRIG2	0,07	-1,84	-1,91
LRP10	0,31	0,56	0,25
LRP11	-0,36	1,21	1,57
LRP2BP	-0,15	-0,56	-0,41
LRPAP1	-0,62	0,05	0,67
LRPPRC	0,31	0,06	-0,26
LRRC14	0,35	0,11	-0,24
LRRC17	-1,31	1,05	2,36
LRRC28	0,62	-0,30	-0,92
LRRC29	0,02	0,01	-0,02
LRRC31	0,13	-0,06	-0,19
LRRC33	0,29	1,14	0,85
LRRC34	-0,83	2,91	3,74
LRRC37A	0,54	-0,49	-1,03
LRRC40	0,01	0,19	0,18
LRRC41	0,07	0,48	0,41
LRRC42	-0,20	0,68	0,88
LRRC46	0,53	-0,12	-0,65
LRRC51	0,03	0,11	0,08
LRRC57	0,09	0,18	0,09
LRRC59	-0,22	-0,12	0,10
LRRC6	-0,47	-2,56	-2,09
LRRC61	1,29	2,44	1,15
LRRC8A	-1,07	1,29	2,36
LRRC8B	0,71	1,83	1,13
LRRC8C	0,67	0,70	0,03
LRRC8D	-0,61	-1,13	-0,52
LRRFIP1	0,79	-0,46	-1,25
LRRFIP2	0,12	-0,80	-0,92
LRRIQ2	-0,39	-0,80	-0,41
LRRK1	1,69	1,58	-0,11
LRRK2	-0,01	-3,48	-3,47
LRSAM1	0,43	0,26	-0,17
LRTM1	-0,20	-0,19	0,01
LSAMP	-0,05	0,24	0,29
LSG1	-0,06	-0,05	0,01
LSM1	0,29	0,43	0,14
LSM10	0,02	-0,42	-0,44
LSM14A	0,34	-1,38	-1,72
LSM2	-0,07	0,42	0,49
LSM3	-0,53	-0,25	0,28
LSM4	-0,37	0,37	0,74
LSM5	0,17	0,38	0,22
LSM7	0,22	-0,70	-0,92
LSM8	0,53	-0,03	-0,56
LSMD1	-0,06	-0,25	-0,18
LSP1	-0,22	-0,58	-0,35
LTB4R2	-0,07	-2,68	-2,61
LTBP1	-0,20	-0,09	0,11
LTBP4	-0,73	-1,42	-0,69
LTC4S	2,01	1,49	-0,52

LTV1	0,51	0,92	0,41
LY75	3,26	2,26	-0,99
LY86	-1,08	-2,31	-1,22
LY9	2,44	5,53	3,09
LYAR	0,06	-0,65	-0,71
LYPD2	-0,38	-0,98	-0,60
LYPD3	3,73	2,98	-0,75
LYPLA2	0,47	0,23	-0,24
LYPLAL1	-0,72	0,96	1,68
LYSMD2	-1,81	-3,08	-1,27
LYSMD3	0,07	0,18	0,10
LYSMD4	-0,71	-0,22	0,49
LYST	0,75	-1,75	-2,50
LZIC	-0,30	0,41	0,71
LZTR1	0,35	-0,05	-0,40
LZTR2	-0,59	-0,71	-0,12
M6PR	0,11	1,49	1,39
MAD2L1BP	0,23	0,74	0,51
MAEA	0,30	0,10	-0,21
MAF	1,55	3,65	2,10
MAF1	0,20	-0,61	-0,81
MAFF	0,50	7,00	6,50
MAFG	0,76	0,54	-0,22
MAG	-0,48	-0,26	0,22
MAGEA11	0,78	0,96	0,18
MAGEA2	1,29	3,56	2,27
MAGEB1	0,45	0,56	0,11
MAGED1	-1,28	0,88	2,17
MAGED2	0,87	0,76	-0,11
MAGEF1	0,40	-0,32	-0,72
MAGEH1	-1,63	-1,89	-0,26
MAGEL2	-0,56	0,18	0,74
MAGOH	0,06	-0,83	-0,90
MAK	-0,09	-2,00	-1,90
MALT1	0,47	-0,49	-0,96
MAML2	-0,73	0,12	0,86
MAML3	-0,41	-0,78	-0,36
MAN1A1	0,17	1,69	1,53
MAN1B1	0,01	0,52	0,51
MAN2A1	0,87	0,59	-0,28
MAN2B1	0,32	0,60	0,28
MAN2C1	0,27	-0,64	-0,91
MANBA	-0,31	-0,95	-0,64
MANEAL	0,72	5,41	4,69
MAOA	5,84	8,92	3,09
MAOB	0,73	2,26	1,52
MAP1A	1,15	3,34	2,19
MAP1LC3A	0,19	0,96	0,77
MAP1LC3C	-0,48	3,56	4,04
MAP2K1IP1	0,15	1,03	0,88
MAP2K3	-1,24	-1,89	-0,65
MAP2K4	0,16	0,26	0,10
MAP2K5	0,11	-0,47	-0,59
MAP2K6	0,81	0,52	-0,29
MAP2K7	0,33	-0,36	-0,69
MAP3K11	-0,17	-0,44	-0,27
MAP3K14	1,09	0,19	-0,91
MAP3K2	-0,05	-0,71	-0,66
MAP3K6	0,02	0,10	0,08

MAP3K8	-0,65	-0,69	-0,05
MAP4	0,11	0,47	0,36
MAP4K1	3,49	-1,06	-4,55
MAP4K2	-0,24	-0,51	-0,27
MAP4K3	-0,11	0,98	1,09
MAPBPIP	-0,30	0,62	0,92
MAPK1	1,11	-0,34	-1,44
MAPK13	-0,68	5,11	5,79
MAPK14	-0,48	-0,81	-0,33
MAPK15	-0,53	0,34	0,87
MAPK3	-0,53	-1,18	-0,65
MAPK4	-0,90	-0,07	0,83
MAPK6	-0,13	1,02	1,15
MAPK7	1,10	-1,32	-2,42
MAPK8	0,15	-0,47	-0,62
MAPK9	0,20	0,57	0,37
MAPKAP1	-0,68	0,75	1,43
MAPKAPK2	-0,18	-0,42	-0,24
MAPKAPK3	0,09	0,19	0,10
MAPKAPK5	0,10	0,39	0,30
MAPKBP1	0,72	0,86	0,14
MAPRE1	-0,07	0,03	0,10
MAPRE3	-2,18	0,45	2,63
MARCO	-3,71	-0,40	3,31
MARK2	0,52	-0,81	-1,34
MARS	0,53	0,13	-0,40
MARVELD2	1,19	1,41	0,22
MAS1	-0,08	1,41	1,49
MASK-BP3	0,68	-0,37	-1,05
MASTL	-1,05	-0,13	0,92
MAT2A	0,81	1,01	0,21
MATK	-0,93	4,66	5,59
MATN1	-0,16	1,22	1,38
MATR3	0,31	-0,29	-0,60
MAZ	0,24	0,04	-0,20
MBD1	0,45	0,30	-0,15
MBD3L1	0,44	-0,06	-0,50
MBD4	-0,50	0,08	0,58
MBD5	-0,05	0,50	0,55
MBD6	0,44	-0,88	-1,32
MBNL1	0,37	-0,64	-1,01
MBNL2	0,40	-0,35	-0,75
MBP	-0,16	0,15	0,31
MBTPS1	0,10	-0,81	-0,91
MBTPS2	-0,23	1,38	1,61
MC1R	-0,20	2,89	3,09
MC2R	-0,03	-0,30	-0,27
MCART1	0,16	-1,35	-1,50
MCART6	-0,35	-0,48	-0,13
MCEE	-0,44	-0,36	0,08
MCFD2	-0,07	-0,12	-0,05
MCHR1	-0,01	0,45	0,45
MCM10	-7,00	-0,85	6,15
MCM2	-1,77	2,33	4,11
MCM3AP	0,18	0,10	-0,08
MCM4	-1,73	0,37	2,10
MCM5	-0,17	-0,12	0,05
MCM7	-1,61	-1,26	0,35
MCOLN1	0,09	1,57	1,47

MCOLN3	-0,76	11,77	12,53
MCRS1	0,12	0,06	-0,06
MCTP2	-0,01	-0,53	-0,52
MCTS1	-0,32	-0,05	0,27
MDC1	0,34	1,32	0,98
MDFIC	-0,34	0,56	0,90
MDH1	0,63	2,62	1,98
MDH2	-0,19	0,53	0,72
MDM1	-0,31	-1,01	-0,70
MDM2	0,03	1,21	1,18
MDM4	0,52	-2,08	-2,60
MDN1	0,80	-0,08	-0,88
MDP-1	-0,45	0,33	0,78
MDS032	0,36	-0,99	-1,35
ME1	-2,13	0,72	2,85
ME2	-0,42	0,17	0,59
ME3	-0,44	2,54	2,98
MEA1	0,14	0,80	0,66
MECR	-1,23	-0,10	1,12
MED11	-0,08	-0,38	-0,30
MED12	0,36	0,16	-0,20
MED18	0,50	1,11	0,60
MED19	0,42	0,76	0,34
MED28	0,59	0,40	-0,19
MED31	-0,40	0,02	0,42
MED4	0,07	-0,79	-0,86
MED6	-0,27	0,67	0,95
MED8	-0,04	0,54	0,58
MED9	0,00	0,87	0,87
MEF2A	-0,15	-0,34	-0,19
MEF2B	0,19	-0,60	-0,79
MEGF8	0,33	0,62	0,28
MEN1	0,19	-0,53	-0,72
MEOX1	-0,70	1,79	2,49
MERTK	-2,26	-2,35	-0,09
MESDC1	0,90	0,28	-0,61
MESDC2	-0,20	1,42	1,62
MESP1	-1,53	0,24	1,77
MEST	-0,87	-1,00	-0,12
MET	-0,56	-0,22	0,34
METAP1	-0,05	-0,10	-0,05
METAP2	0,12	0,10	-0,01
METT10D	-0,05	-0,18	-0,13
METT5D1	-0,25	0,37	0,62
METTL1	-0,07	3,16	3,23
METTL2A	-0,17	0,26	0,43
METTL2B	1,26	3,00	1,75
METTL4	-0,86	-0,47	0,39
METTL6	-0,77	0,04	0,81
METTL7A	2,13	0,34	-1,79
MFAP3	-0,09	1,04	1,13
MFGE8	-2,04	1,10	3,14
MFHAS1	0,55	2,45	1,90
MFN2	-0,06	0,53	0,59
MFNG	1,13	-0,64	-1,77
MFRP	-0,70	-0,52	0,18
MFSD2	0,03	0,53	0,50
MGAT2	0,28	0,38	0,10
MGAT4A	0,62	1,19	0,57

MGAT4B	-0,08	0,53	0,61
MGC11102	0,70	0,41	-0,29
MGC14327	0,21	-0,13	-0,34
MGC14376	0,27	-2,04	-2,31
MGC15885	0,22	1,01	0,79
MGC16169	-0,04	1,15	1,20
MGC16824	0,21	-0,19	-0,40
MGC20983	5,69	-1,08	-6,77
MGC21675	-0,44	-0,87	-0,43
MGC2752	0,17	0,05	-0,12
MGC3207	0,24	-1,19	-1,43
MGC33556	-1,75	-4,59	-2,84
MGC34761	0,82	-0,42	-1,24
MGC35361	-0,20	-0,08	0,12
MGC3731	-0,40	-0,98	-0,58
MGC39900	1,43	1,76	0,33
MGC40499	-0,16	-0,89	-0,73
MGC4093	0,24	-0,40	-0,64
MGC4172	2,56	5,53	2,97
MGC42630	0,53	5,23	4,71
MGC45491	0,59	0,16	-0,43
MGC4677	1,14	1,29	0,15
MGC52000	0,17	-1,06	-1,23
MGC52110	0,23	-0,45	-0,68
MGC59937	-0,95	-0,11	0,84
MGC70924	0,18	0,24	0,06
MGC71993	0,12	0,56	0,44
MGC72104	-0,12	-0,23	-0,11
MGMT	-0,35	-0,03	0,32
MGRN1	0,54	0,26	-0,28
MGST1	-0,50	0,30	0,80
MGST2	-1,10	-0,60	0,50
MIA	-0,14	-0,12	0,02
MICA	0,29	0,66	0,36
MICAL2	-0,02	-1,53	-1,50
MICALCL	1,06	-1,94	-3,00
MID1IP1	0,50	0,21	-0,29
MID2	3,69	7,75	4,06
MIDN	0,56	-1,51	-2,07
MIER1	0,40	-0,15	-0,55
MIF	0,15	0,74	0,59
MIF4GD	0,04	0,02	-0,02
MINA	0,06	-0,41	-0,47
MIPEP	0,07	2,97	2,91
MIS12	0,03	0,75	0,72
MITF	-0,70	2,09	2,80
MIZF	0,34	-0,39	-0,73
MKKS	0,26	0,58	0,32
MKL1	1,25	-0,29	-1,53
MKNK1	0,08	0,55	0,47
MKS1	-0,22	-0,05	0,17
MLC1	-2,12	-2,80	-0,67
MLH1	-0,34	0,26	0,60
MLL	0,37	-0,64	-1,01
MLL3	0,32	-1,27	-1,59
MLLT10	0,49	-0,80	-1,29
MLLT11	-1,65	1,55	3,20
MLLT3	-0,09	-0,04	0,05
MLSTD1	1,83	3,26	1,44

MLX	0,51	-0,31	-0,82
MLXIP	0,73	-0,51	-1,23
MMAA	0,01	0,92	0,91
MMAB	-0,18	0,53	0,71
MMACHC	0,45	1,19	0,74
MMP1	2,29	9,12	6,82
MMP10	2,25	9,96	7,71
MMP12	5,50	7,32	1,82
MMP13	-0,13	0,82	0,95
MMP14	-0,99	5,52	6,51
MMP19	-0,96	1,69	2,65
MMP21	-0,28	-0,52	-0,24
MMP7	-6,71	3,89	10,60
MMS19L	0,33	0,37	0,04
MND1	-8,28	0,00	8,28
MNS1	-3,84	-1,93	1,91
MNT	0,08	-1,08	-1,15
MOAP1	-0,48	-0,29	0,19
MOBKL2B	-0,64	2,46	3,10
MOBKL2C	0,35	1,03	0,68
MOCS2	-0,04	1,63	1,67
MOCS3	1,49	2,39	0,90
MON1B	-0,17	0,08	0,25
MORC4	0,28	2,16	1,88
MORF4L1	0,18	0,07	-0,11
MORF4L2	-0,59	0,57	1,16
MORG1	-0,23	0,63	0,86
MOSPD2	-0,03	0,42	0,45
MOV10	-0,38	-0,23	0,15
MPDU1	-0,09	1,39	1,48
MPDZ	0,83	1,08	0,26
MPFL	0,04	0,22	0,18
MPHOSPH10	0,18	0,22	0,04
MPHOSPH6	0,24	0,81	0,56
MPHOSPH9	0,00	0,19	0,19
MPI	0,00	0,06	0,05
MPL	-0,70	3,80	4,51
MPP1	-0,39	0,04	0,43
MPP7	-0,47	-1,84	-1,37
MPPED2	0,84	0,70	-0,14
MPST	0,17	-0,75	-0,92
MPV17	-0,23	1,16	1,38
MPZ	-0,27	-0,58	-0,30
MR1	-0,62	1,02	1,64
MRAP	-0,78	-1,75	-0,96
MRAS	-0,41	3,24	3,64
MRC1	2,84	5,41	2,57
MRC1L1	0,28	8,12	7,84
MRCL3	0,58	0,55	-0,03
MRE11A	-0,41	0,04	0,44
MRLC2	0,13	0,59	0,46
MRP63	1,13	-1,32	-2,45
MRPL11	0,26	-0,69	-0,95
MRPL12	0,25	0,99	0,74
MRPL13	-0,26	1,00	1,26
MRPL14	-0,02	0,30	0,32
MRPL17	-0,57	1,18	1,75
MRPL18	-0,11	0,23	0,34
MRPL19	-0,11	0,35	0,47

MRPL2	0,10	-0,10	-0,20
MRPL20	0,15	0,14	-0,01
MRPL21	-0,27	0,06	0,33
MRPL22	-0,34	0,28	0,63
MRPL24	-0,35	0,10	0,46
MRPL27	-0,12	0,43	0,54
MRPL30	-0,01	-0,16	-0,15
MRPL32	0,11	0,42	0,31
MRPL33	-0,11	-0,24	-0,13
MRPL34	0,49	0,89	0,40
MRPL35	-0,04	1,40	1,44
MRPL37	-0,75	0,89	1,64
MRPL38	-0,11	-0,26	-0,16
MRPL39	-0,60	0,77	1,37
MRPL40	-0,37	0,98	1,35
MRPL41	0,44	0,16	-0,27
MRPL42	-0,25	-0,03	0,22
MRPL43	-0,30	0,05	0,35
MRPL44	-0,14	0,25	0,39
MRPL46	-0,19	0,78	0,97
MRPL47	-0,39	0,26	0,65
MRPL48	-0,42	-0,74	-0,33
MRPL49	-0,03	0,69	0,72
MRPL50	0,16	1,50	1,34
MRPL51	-0,01	0,72	0,74
MRPL52	-0,23	-0,39	-0,16
MRPL53	-0,15	-0,42	-0,27
MRPL55	-0,03	-0,53	-0,50
MRPS11	-0,49	-0,09	0,40
MRPS12	-0,22	0,14	0,36
MRPS14	0,34	0,11	-0,23
MRPS15	-0,57	0,48	1,06
MRPS16	-0,34	-0,01	0,32
MRPS17	-0,29	0,88	1,17
MRPS18A	0,13	-0,07	-0,20
MRPS18B	-0,01	0,05	0,05
MRPS18C	-0,48	0,40	0,88
MRPS2	0,33	-0,46	-0,79
MRPS21	0,28	-0,08	-0,36
MRPS22	-0,09	-0,03	0,06
MRPS23	-0,24	0,41	0,65
MRPS24	-0,36	-0,61	-0,25
MRPS26	0,32	-0,01	-0,33
MRPS27	-0,03	0,22	0,25
MRPS30	-0,07	0,29	0,36
MRPS34	0,20	0,14	-0,06
MRPS36	0,33	0,64	0,31
MRPS5	-0,14	-0,22	-0,08
MRPS6	0,67	1,79	1,11
MRPS7	-0,21	0,71	0,93
MRRF	-0,25	0,18	0,43
MRVI1	0,83	-2,38	-3,21
MS4A2	0,04	0,50	0,46
MS4A3	0,32	-0,22	-0,54
MS4A6A	1,71	-1,19	-2,90
MS4A7	-1,69	-1,73	-0,04
MSC	0,27	4,95	4,68
MSH3	-0,33	-1,00	-0,67
MSI2	-0,20	-0,34	-0,13

MSL2L1	-0,37	-0,87	-0,50
MSL3L1	-0,10	-1,39	-1,29
MSR1	-1,82	2,33	4,15
MSRA	-0,27	0,28	0,55
MST1	0,17	-0,08	-0,25
MSTO1	-0,03	0,00	0,03
MT1B	0,54	0,84	0,30
MT1F	-2,00	-3,84	-1,84
MT1G	-4,05	-1,49	2,56
MTA2	0,48	-0,36	-0,84
MTA3	-0,60	1,02	1,62
MTCH1	0,07	0,15	0,08
MTCP1	-0,30	0,75	1,05
MTERFD1	0,15	0,62	0,47
MTF1	-0,46	-0,27	0,19
MTF2	0,19	-0,44	-0,63
MTHFD2	0,53	1,93	1,40
MTHFD2L	-0,79	-0,65	0,15
MTHFR	0,24	-0,48	-0,72
MTHFS	-2,42	-1,65	0,77
MTHFSD	-0,45	-0,83	-0,39
MTIF2	-0,17	0,30	0,47
MTL5	0,11	-0,92	-1,03
MTM1	-0,08	-1,11	-1,03
MTMR1	-0,61	0,16	0,77
MTMR10	-0,13	-0,10	0,03
MTMR11	-1,22	-5,03	-3,80
MTMR3	-0,32	-1,85	-1,53
MTMR4	0,02	0,36	0,34
MTMR9	0,46	-0,44	-0,90
MTR	0,51	0,76	0,26
MTRF1	-0,12	-0,08	0,03
MTRF1L	-0,11	1,40	1,51
MTRR	0,41	0,37	-0,05
MTX1	-0,35	0,40	0,75
MTX3	0,05	0,32	0,27
MUC13	0,25	0,31	0,07
MUC20	-0,10	1,82	1,92
MUS81	0,37	-0,02	-0,39
MUSTN1	-1,37	-2,05	-0,68
MUT	-0,32	1,13	1,45
MUTYH	-0,14	-1,13	-0,98
MVK	0,50	2,87	2,37
MVP	-0,14	-0,54	-0,40
MX1	-2,13	-3,65	-1,52
MXD1	0,61	-1,88	-2,50
MXD4	0,22	-1,93	-2,14
MXI1	0,28	-0,28	-0,55
MYADML	-0,78	-1,10	-0,32
MYB	-1,14	-1,91	-0,77
MYBBP1A	0,46	-0,44	-0,89
MYBPC1	-0,58	-0,65	-0,08
MYBPC3	0,66	-0,65	-1,31
MYBPH	-2,84	-0,12	2,72
MYCBP	0,44	-0,40	-0,84
MYCBP2	-0,39	-1,67	-1,28
MYD88	-2,03	-2,00	0,03
MYEOV	-5,82	-3,89	1,93
MYH6	0,05	0,12	0,07

MYH7	0,04	0,62	0,58
MYH9	0,15	-0,28	-0,43
MYL2	-0,57	0,00	0,57
MYL5	1,15	0,27	-0,88
MYL6B	-0,80	1,20	2,00
MYL7	0,57	0,26	-0,31
MYLIP	1,78	-0,45	-2,23
MYLK	-0,63	0,18	0,81
MYLK2	-0,34	-0,30	0,04
MYO10	-1,15	0,13	1,27
MYO18A	1,95	1,88	-0,07
MYO1A	-0,82	-1,73	-0,91
MYO1D	-1,51	5,57	7,08
MYO6	-6,32	1,22	7,55
MYO9A	0,35	-0,10	-0,45
MYO9B	-0,03	-0,25	-0,22
MYOHD1	0,99	0,38	-0,61
MYOM1	0,40	0,03	-0,37
MYOZ1	-1,59	1,66	3,25
MYST2	0,29	-0,41	-0,70
MYST4	-0,30	-1,00	-0,70
N4BP1	1,74	-0,74	-2,48
N4BP2	-0,07	-0,39	-0,33
NAALADL1	-0,46	-2,70	-2,23
NAALADL2	0,21	0,16	-0,05
NAB1	0,16	-0,15	-0,31
NACA	-0,53	0,01	0,54
NADSYN1	0,32	-0,03	-0,35
NAGA	1,08	-0,21	-1,29
NAGK	-0,06	-0,24	-0,18
NAGPA	1,47	1,96	0,49
NANOG	-0,29	0,94	1,23
NANP	0,09	1,39	1,29
NAP1L1	-0,54	-1,04	-0,50
NAP1L2	-1,02	-0,68	0,34
NAPG	-0,32	0,02	0,33
NAPSA	1,86	-2,81	-4,67
NARF	0,50	-0,54	-1,04
NARG1	-0,26	0,04	0,30
NARG2	0,27	0,02	-0,25
NARS	0,01	0,33	0,33
NAT1	-0,41	2,07	2,47
NAT10	-0,08	-0,08	0,00
NAT5	-0,18	0,62	0,80
NAT6	1,80	-0,22	-2,02
NAT8	0,39	1,09	0,70
NAT9	0,04	-0,37	-0,41
NAV2	-0,29	-0,20	0,09
NBPF1	-0,32	-0,78	-0,46
NBPF10	-0,53	-0,93	-0,40
NBPF14	-0,42	-1,63	-1,21
NBPF9	-1,61	-1,86	-0,24
NCALD	-0,11	1,13	1,24
NCBP1	0,14	0,77	0,64
NCBP2	-0,13	0,65	0,77
NCDN	-0,45	-0,79	-0,34
NCF1	-1,19	-5,39	-4,20
NCF2	-0,40	-0,37	0,03
NCK1	-0,12	1,96	2,08

NCK2	0,84	0,11	-0,73
NCKIPSD	0,55	1,62	1,07
NCL	-0,01	-0,33	-0,32
NCLN	-0,78	0,34	1,12
NCOA1	-0,38	-1,67	-1,29
NCOA3	0,87	0,77	-0,10
NCOA4	-0,63	-0,80	-0,17
NCOA5	1,06	1,07	0,01
NCR2	0,59	0,45	-0,14
NCSTN	0,03	0,43	0,40
NDE1	0,32	-1,17	-1,48
NDN	0,42	1,93	1,51
NDNL2	0,10	-0,15	-0,24
NDP	0,02	11,00	10,98
NDRG3	-0,35	-0,10	0,25
NDRG4	-0,34	-0,32	0,01
NDST1	-0,41	-0,03	0,38
NDST2	0,47	-0,64	-1,11
NDUFA1	0,04	0,82	0,78
NDUFA10	-0,50	-0,36	0,14
NDUFA13	-0,12	0,62	0,74
NDUFA2	0,06	0,04	-0,03
NDUFA5	0,43	0,41	-0,03
NDUFA6	-0,17	0,27	0,43
NDUFA7	-0,34	0,37	0,71
NDUFA8	-0,12	1,04	1,16
NDUFA9	-0,23	0,78	1,01
NDUFB1	-0,15	1,07	1,23
NDUFB11	-0,01	0,00	0,01
NDUFB2	-0,30	0,49	0,79
NDUFB3	-0,53	0,51	1,04
NDUFB5	-0,04	0,27	0,31
NDUFB6	-0,87	0,68	1,55
NDUFB7	-0,26	0,08	0,34
NDUFB8	-0,08	0,51	0,59
NDUFB9	-1,08	0,15	1,23
NDUFC1	0,01	0,24	0,23
NDUFS1	0,13	0,63	0,50
NDUFS2	-0,29	0,05	0,34
NDUFS3	-0,74	0,58	1,32
NDUFS4	0,10	0,53	0,43
NDUFS8	0,09	0,98	0,89
NDUFV1	0,24	0,55	0,32
NEB	0,78	0,30	-0,48
NECAP1	-0,15	-0,54	-0,38
NEDD4	-0,48	-1,15	-0,67
NEDD8	0,01	0,18	0,17
NEDD9	-1,54	-2,40	-0,86
NEK1	-0,33	-0,05	0,28
NEK11	-0,20	0,90	1,10
NEK3	1,35	-0,18	-1,53
NEK4	-1,09	-4,45	-3,36
NEK6	-0,16	3,63	3,79
NEK7	0,10	-0,31	-0,41
NEK9	-0,50	-0,18	0,32
NENF	-0,18	0,90	1,08
NEO1	-0,17	-0,07	0,10
NET1	3,07	3,39	0,32
NETO2	1,07	0,73	-0,35

NEU3	1,45	0,65	-0,79
NEUROD6	-1,13	1,86	2,99
NEXN	-3,25	-4,93	-1,68
NF1	-0,38	-1,25	-0,87
NFAM1	-0,30	-2,32	-2,02
NFAT5	0,62	-0,31	-0,93
NFATC3	0,01	-0,16	-0,17
NFE2L1	0,26	1,62	1,37
NFE2L3	0,80	-0,10	-0,89
NFIC	-0,09	-0,36	-0,27
NFKBIB	0,11	0,34	0,23
NFKBIE	0,45	1,63	1,18
NFKBIL1	0,15	-0,47	-0,61
NFKBIZ	-3,24	-4,34	-1,10
NFRKB	0,00	-0,27	-0,28
NFS1	0,15	0,41	0,26
NFXL1	2,39	0,69	-1,69
NFYA	-0,10	-0,67	-0,57
NFYB	0,34	-0,17	-0,51
NGFRAP1	-4,07	-0,24	3,83
NGRN	0,30	0,72	0,42
NHEJ1	-0,03	0,23	0,26
NHLH1	0,40	0,01	-0,39
NHLRC1	-1,79	-1,34	0,46
NHLRC2	-0,10	0,00	0,10
NHN1	-0,06	-0,68	-0,62
NHP2L1	-0,28	-0,51	-0,23
NIBP	0,04	-0,16	-0,21
NIF3L1	-0,11	0,53	0,64
NIN	0,07	-1,46	-1,52
NIP30	-0,19	-0,63	-0,44
NIP7	0,55	1,53	0,98
NIPBL	0,34	-1,18	-1,52
NIPSNAP3A	-0,06	-0,60	-0,54
NIT1	-0,06	0,02	0,08
NKAP	-0,28	0,01	0,29
NKIRAS1	0,15	2,80	2,65
NKIRAS2	0,30	-0,57	-0,88
NLN	-0,84	0,03	0,87
NMB	-0,92	3,17	4,08
NMD3	0,93	1,11	0,18
NME1-NME2	-0,18	0,49	0,67
NME3	0,62	-0,50	-1,12
NME4	-0,52	0,30	0,82
NME6	-0,03	-0,49	-0,46
NME7	-0,88	-0,12	0,76
NMI	0,38	-0,61	-0,99
NMNAT1	-0,83	0,00	0,83
NMNAT2	0,04	-0,11	-0,15
NMT1	-0,17	0,13	0,30
NNT	0,20	0,73	0,53
NOC3L	0,01	-0,03	-0,04
NOC4L	-0,04	-0,34	-0,30
NOL1	0,38	0,33	-0,05
NOL10	-0,03	-1,21	-1,18
NOL11	0,15	0,21	0,06
NOL5A	0,33	0,08	-0,26
NOL6	0,19	-0,12	-0,31
NOL7	0,10	0,01	-0,09

NOL8	0,08	0,01	-0,07
NOL9	-0,32	-1,01	-0,69
NOLA2	-0,37	-0,32	0,05
NOLA3	-0,55	0,51	1,07
NOLC1	0,04	-0,09	-0,13
NOMO1	-0,45	0,38	0,83
NOMO3	-0,36	1,10	1,46
NONO	0,08	-0,12	-0,20
NOP5/NOP58	0,45	0,21	-0,24
NOSIP	-0,01	-0,73	-0,72
NOTCH4	-0,69	-1,66	-0,97
NOX1	1,57	0,30	-1,27
NOXA1	0,47	-0,20	-0,67
NPAL2	-0,48	-0,44	0,04
NPAL3	0,29	1,56	1,27
NPAS1	-7,48	-2,86	4,62
NPAT	0,42	0,51	0,09
NPC2	0,03	0,76	0,73
NPFF	0,53	-1,33	-1,86
NPFFR1	0,33	0,77	0,44
NPHP3	0,24	-0,83	-1,08
NPHS2	-0,26	-0,50	-0,25
NPIP	0,28	-1,20	-1,48
NPL	-0,67	0,60	1,27
NPTN	0,10	0,41	0,31
NQO1	-0,70	4,67	5,37
NQO2	0,02	-0,19	-0,21
NR1H2	0,01	-0,15	-0,15
NR1H3	-1,71	2,87	4,57
NR1H4	0,30	0,59	0,30
NR1I2	-0,07	-0,37	-0,30
NR2C2	0,19	-0,54	-0,72
NR2E3	-0,32	-0,41	-0,09
NR3C1	-0,88	-0,63	0,25
NR4A1	0,07	-2,82	-2,89
NR4A3	1,53	1,88	0,35
NRAS	-0,40	0,79	1,18
NRCAM	-0,50	4,54	5,04
NRD1	0,01	-0,10	-0,11
NRF1	-0,19	0,04	0,23
NRG2	0,46	0,46	0,00
NRIP1	-1,24	-1,44	-0,20
NRL	-0,72	-0,90	-0,19
NRM	0,02	-1,67	-1,69
NRP2	0,05	2,59	2,55
NRXN1	-0,27	-0,18	0,09
NRXN2	0,93	1,32	0,40
NRXN3	-1,14	-0,44	0,70
NSD1	-0,04	-1,46	-1,42
NSDHL	0,12	1,32	1,20
NSFL1C	-0,24	-1,91	-1,67
NSMAF	0,03	1,05	1,02
NSMCE1	0,01	0,45	0,44
NSUN2	0,27	-0,34	-0,61
NSUN3	-0,23	0,22	0,46
NSUN5	0,74	-0,26	-1,00
NSUN5B	0,45	0,18	-0,28
NSUN5C	0,82	-0,29	-1,11
NSUN6	-0,31	-0,39	-0,08

NT5C2	0,06	0,08	0,02
NT5C3	-0,32	-1,50	-1,17
NTAN1	0,27	1,86	1,59
NTHL1	0,15	-0,02	-0,17
NTNG2	2,96	-5,22	-8,18
NTSR1	0,00	-8,98	-8,98
NTSR2	-2,59	-2,43	0,15
NUAK2	0,02	-3,89	-3,91
NUBP1	0,06	-0,67	-0,73
NUBP2	-0,17	-0,26	-0,09
NUBPL	-0,20	0,42	0,63
NUCB2	-2,15	-2,01	0,14
NUCKS1	0,31	0,78	0,47
NUDCD1	0,20	0,44	0,24
NUDCD2	0,10	0,06	-0,04
NUDCD3	-0,03	0,31	0,34
NUDT1	-0,35	-0,27	0,08
NUDT15	-0,19	-0,40	-0,20
NUDT16	1,46	0,05	-1,40
NUDT16P	2,51	1,25	-1,27
NUDT18	0,00	0,42	0,42
NUDT2	-0,36	0,07	0,43
NUDT21	-0,35	-1,24	-0,89
NUDT4	-0,22	-0,32	-0,09
NUDT5	-0,01	0,70	0,71
NUDT6	-0,16	1,37	1,53
NUDT9	-0,45	1,09	1,54
NUFIP2	0,83	-0,87	-1,70
NUMA1	0,61	-0,35	-0,97
NUMB	-1,61	-1,37	0,24
NUMBL	-0,12	3,72	3,85
NUP107	-0,06	0,42	0,48
NUP133	0,02	0,01	-0,01
NUP155	-0,23	-0,09	0,14
NUP160	-0,33	0,13	0,46
NUP188	-0,09	0,79	0,88
NUP210	-0,19	-0,08	0,11
NUP43	0,28	-0,42	-0,70
NUP50	0,40	-0,08	-0,48
NUP54	0,25	-0,35	-0,59
NUP62	0,47	0,14	-0,33
NUP85	0,37	0,13	-0,24
NUP88	-0,33	-0,49	-0,16
NUP93	-0,02	-0,70	-0,68
NUPL2	0,20	-0,28	-0,47
NUSAP1	-2,27	0,37	2,64
NUT	-0,15	1,06	1,21
NUTF2	0,04	0,05	0,01
NVL	-0,31	-0,03	0,28
NXF1	0,11	-1,57	-1,68
NXF3	-1,50	3,02	4,52
NXT2	-0,22	-0,43	-0,21
NYD-SP21	-1,12	-3,44	-2,32
OAF	0,15	-3,61	-3,76
OAS1	0,47	-0,42	-0,89
OAS3	0,02	-1,57	-1,59
OASL	-1,48	-3,95	-2,47
OAZ1	-0,03	0,15	0,18
OAZ2	-0,59	-1,13	-0,55

OAZ3	0,23	1,22	0,99
OBFC2A	-0,17	2,15	2,32
OBFC2B	-0,28	0,14	0,42
OCM	-0,08	-0,14	-0,06
OCRL	-0,16	0,54	0,70
ODF2	1,50	0,09	-1,41
ODZ1	0,04	-0,29	-0,34
OFCC1	4,75	-0,32	-5,06
OFD1	0,64	-0,29	-0,93
OGDHL	0,41	0,16	-0,25
OGFOD1	0,10	0,69	0,59
OGFRL1	1,24	0,15	-1,09
OGT	0,62	-1,09	-1,72
OIP5	-6,57	-0,09	6,48
OIT3	-0,59	0,64	1,23
OKL38	0,46	2,28	1,82
OLFML2B	-1,15	-1,09	0,06
OLIG1	-0,82	-6,42	-5,61
OLR1	-2,44	7,94	10,38
OMA1	-0,65	-1,16	-0,52
OMG	-0,37	-3,76	-3,38
OMP	0,10	-0,38	-0,48
OPA1	-0,32	-0,14	0,19
OPA3	-0,18	0,49	0,67
OPN1MW	-0,08	-0,34	-0,26
OPN1SW	0,47	0,20	-0,27
OPN3	-0,03	0,95	0,98
OPN5	-0,35	-0,69	-0,34
OPRS1	-0,43	0,42	0,85
OPTC	-0,38	-0,66	-0,28
OPTN	0,26	3,86	3,61
OR10G2	-0,84	-0,38	0,47
OR10G3	0,72	-0,87	-1,59
OR10G7	0,27	0,33	0,06
OR10G8	0,46	-1,01	-1,48
OR10K1	-0,19	0,29	0,48
OR11A1	0,44	0,40	-0,04
OR12D3	-0,23	0,74	0,97
OR13H1	-0,16	-0,08	0,08
OR1A2	0,13	-0,33	-0,46
OR1F1	-0,15	-0,27	-0,12
OR1L3	0,62	0,55	-0,07
OR1L8	-0,13	0,32	0,45
OR1N1	-0,55	2,00	2,56
OR1Q1	-0,94	-3,13	-2,19
OR2A14	0,35	0,58	0,23
OR2A2	-0,06	-0,53	-0,47
OR2A20P	-0,28	-0,29	-0,01
OR2A42	1,25	0,83	-0,42
OR2AG1	3,59	-2,35	-5,94
OR2AG2	-1,45	-1,94	-0,48
OR2D2	0,05	0,42	0,36
OR2F1	-0,84	-0,08	0,75
OR2G2	-0,03	0,49	0,52
OR2H2	-0,10	0,30	0,40
OR2J2	0,46	-0,19	-0,65
OR2J3	-3,51	-1,71	1,81
OR2L8	-0,31	-0,02	0,29
OR2M2	0,35	-0,08	-0,43

OR2T12	-1,09	0,20	1,30
OR2T35	-0,04	-0,43	-0,39
OR4A47	-0,15	-0,61	-0,46
OR4C13	0,01	0,57	0,55
OR4C16	-0,17	-0,29	-0,12
OR4D11	-0,44	-0,55	-0,10
OR4D2	0,23	0,15	-0,08
OR4F21	-0,06	0,46	0,52
OR4S1	0,10	-0,07	-0,17
OR4X2	0,01	0,65	0,64
OR51B5	1,45	0,31	-1,15
OR51G1	-0,05	-0,13	-0,08
OR51G2	0,53	0,83	0,30
OR51I2	0,07	1,17	1,10
OR51M1	-1,10	-2,05	-0,96
OR51V1	0,29	0,39	0,10
OR52A1	-0,24	-0,27	-0,03
OR52A4	0,28	-0,20	-0,48
OR52B6	0,28	0,07	-0,21
OR52D1	-0,74	-0,12	0,62
OR52E6	-0,25	0,29	0,54
OR52I1	-0,99	-1,13	-0,14
OR52K2	-0,22	-2,13	-1,91
OR52W1	0,28	0,32	0,04
OR56A1	-0,55	-0,43	0,12
OR56A3	0,46	0,39	-0,07
OR56B1	0,32	-0,04	-0,36
OR5AP2	0,39	-0,58	-0,96
OR5BU1	-0,15	0,38	0,52
OR5M11	-0,07	-0,27	-0,20
OR5M8	0,07	0,56	0,49
OR5U1	-0,42	-0,07	0,36
OR6C3	0,51	0,29	-0,23
OR6C4	0,70	0,50	-0,20
OR6C75	0,49	3,60	3,11
OR6K3	-3,28	-1,08	2,20
OR6S1	0,04	0,62	0,58
OR6T1	-0,06	-0,13	-0,08
OR6V1	0,17	-0,22	-0,39
OR8B8	0,49	0,60	0,11
OR8G1	1,31	3,58	2,27
OR8G5	1,59	4,41	2,82
OR9A4	-0,31	-0,23	0,09
OR9Q2	-0,25	-0,29	-0,04
ORC1L	-1,12	1,83	2,95
ORC3L	0,03	-0,27	-0,29
ORC4L	0,33	-0,07	-0,41
ORC5L	-0,41	0,49	0,89
ORC6L	0,10	0,86	0,75
ORM1	-5,16	-9,00	-3,84
ORM2	-6,28	-3,64	2,64
ORMDL1	-0,02	-0,68	-0,66
ORMDL2	0,34	1,16	0,83
ORMDL3	0,19	0,08	-0,11
OS9	0,15	-0,55	-0,70
OSBP	0,06	-0,07	-0,14
OSBPL11	-0,39	0,15	0,54
OSBPL1A	0,70	2,22	1,52
OSBPL5	0,51	-1,27	-1,78

OSBPL6	-0,55	-0,15	0,40
OSBPL7	0,67	-1,59	-2,26
OSBPL8	-0,16	-0,50	-0,34
OSGEP	-0,21	-0,41	-0,20
OSGEPL1	0,18	2,58	2,39
OSTF1	-0,08	-0,04	0,05
OSTBETA	-0,67	4,35	5,02
OTOA	0,05	0,17	0,11
OTOF	0,39	-0,72	-1,12
OTOP3	0,20	-0,31	-0,51
OTUD4	-0,35	-0,34	0,01
OTUD5	0,23	-0,07	-0,30
OTUD6B	1,36	0,38	-0,98
OVCA2	0,30	0,54	0,24
OVCH1	0,08	0,23	0,14
OVGP1	0,64	-0,85	-1,49
OXA1L	0,09	-0,21	-0,30
OXCT1	1,09	0,95	-0,14
OXNAD1	0,02	1,23	1,21
OXR1	-0,53	-1,09	-0,56
OXSM	0,43	1,33	0,90
OXSR1	-0,54	-0,48	0,06
P11	0,90	0,95	0,05
P117	0,60	0,72	0,11
P18SRP	-0,66	0,13	0,78
P2RX4	-0,70	0,35	1,06
P2RX7	0,05	-0,96	-1,01
P2RY10	0,41	0,75	0,34
P2RY2	-0,63	-1,39	-0,76
P2RY4	0,47	0,25	-0,22
P2RY6	0,68	1,56	0,89
P4HA1	0,35	0,37	0,03
P4HA3	0,26	-0,59	-0,84
PA2G4	-0,05	0,15	0,19
PABPN1	0,35	-0,80	-1,15
PACS2	0,52	0,65	0,13
PADI1	-0,16	-0,09	0,08
PADI4	-0,14	-8,29	-8,15
PAEP	-0,02	-0,23	-0,21
PAF1	0,27	-0,41	-0,69
PAFAH1B3	0,26	-0,29	-0,54
PAFAH2	-0,40	1,60	2,00
PAG1	-1,12	-1,02	0,10
PAICS	-0,58	0,54	1,12
PAK1IP1	-0,45	0,13	0,58
PAK2	0,61	-0,48	-1,09
PAK4	0,18	0,19	0,01
PAN3	0,29	-1,57	-1,86
PANK1	0,04	0,99	0,95
PANX3	0,06	-0,57	-0,64
PAPD1	-0,08	0,25	0,32
PAPD5	0,88	0,20	-0,68
PAPOLA	0,28	-0,53	-0,81
PAPPA2	-0,54	0,54	1,08
PAPSS2	0,55	2,01	1,46
PAQR8	-0,86	0,21	1,07
PARC	1,09	1,31	0,23
PARD6A	-0,06	-0,36	-0,31
PARK7	-0,20	0,02	0,22

PARN	0,20	0,34	0,13
PARP1	0,22	0,32	0,10
PARP14	0,07	-0,42	-0,50
PARP15	-1,13	0,57	1,71
PARP16	-0,12	-0,55	-0,43
PARP2	-0,74	1,23	1,97
PARP3	0,05	0,36	0,32
PARP6	-0,27	0,03	0,30
PARP9	-0,30	-0,70	-0,40
PARS2	-0,21	2,60	2,82
PARVB	-0,62	0,63	1,25
PARVG	-1,26	-2,01	-0,75
PASK	0,58	0,57	-0,01
PAXIP1	0,01	-0,68	-0,69
PBX1	0,29	1,26	0,98
PBX3	0,07	1,24	1,17
PBXIP1	0,58	-0,11	-0,70
PC	0,19	0,51	0,32
PCAF	0,09	-0,65	-0,75
PCBD1	0,20	2,20	2,00
PCBP4	-1,73	-0,52	1,21
PCCB	-0,29	1,03	1,32
PCDH12	-6,24	-6,97	-0,73
PCDHAC2	0,11	0,88	0,78
PCDHB7	-0,11	-0,34	-0,23
PCDHGA1	0,17	0,36	0,18
PCDHGB1	0,04	0,34	0,30
PCDHGC3	-3,46	-2,52	0,94
PCGF1	-0,19	-0,48	-0,29
PCGF2	-1,07	1,61	2,67
PCID2	-0,04	-0,37	-0,33
PCM1	1,13	1,14	0,01
PCMT1	-0,19	-0,30	-0,11
PCMTD1	0,09	-0,61	-0,70
PCMTD2	-0,09	-1,93	-1,84
PCNA	-0,78	0,78	1,56
PCNP	0,01	-0,77	-0,78
PCNT	0,67	-0,10	-0,77
PCNX	-1,35	-1,91	-0,56
PCNXL3	0,36	-0,02	-0,39
PCSK7	0,47	0,21	-0,26
PCTK1	0,23	0,23	0,00
PCTK2	0,11	0,80	0,69
PCTP	-0,72	-1,16	-0,44
PCYOX1	0,02	0,64	0,62
PCYT1A	-0,01	0,20	0,21
PDCD10	-0,05	0,74	0,79
PDCD11	0,32	0,63	0,31
PDCD1LG2	0,62	3,83	3,21
PDCD2	0,15	-0,43	-0,57
PDCD2L	-0,12	-0,29	-0,18
PDCD4	0,27	-1,56	-1,83
PDCD6IP	-0,37	-1,37	-1,00
PDCD7	0,27	-0,15	-0,42
PDCL	-0,51	0,56	1,07
PDCL2	0,52	0,37	-0,14
PDE3B	-0,22	1,87	2,09
PDE4A	-0,19	0,58	0,77
PDE4B	-8,65	-11,17	-2,52

PDE4D	0,36	-5,00	-5,36
PDE4DIP	-0,36	0,48	0,84
PDE6B	-0,79	-0,68	0,11
PDE6D	0,50	0,75	0,25
PDE6G	0,74	0,39	-0,35
PDE6H	-2,81	-2,11	0,70
PDE7A	0,21	0,59	0,38
PDE7B	-0,23	-1,83	-1,60
PDGFB	2,96	4,74	1,79
PDGFRB	-1,26	0,41	1,66
PDHA1	0,02	0,95	0,93
PDHX	0,02	0,84	0,83
PDIA4	0,09	1,42	1,33
PDIA6	-0,61	0,49	1,10
PDIK1L	0,17	0,14	-0,03
PDK1	0,30	-1,86	-2,15
PDK3	-0,72	-0,29	0,43
PDK4	-5,91	-10,66	-4,75
PDP2	0,80	4,57	3,76
PDPR	0,65	0,04	-0,61
PDRG1	-0,22	0,74	0,95
PDSS1	-0,17	0,81	0,98
PDXP	0,88	1,26	0,38
PDZD11	0,43	-0,50	-0,93
PEA15	0,39	1,33	0,94
PEBP1	0,58	2,36	1,78
PEBP4	-1,44	-0,10	1,34
PECI	-0,34	0,22	0,56
PEF1	0,21	0,46	0,24
PEO1	0,46	1,02	0,56
PEPD	-0,07	0,95	1,02
PER3	0,39	3,09	2,69
PERLD1	-0,32	0,57	0,89
PERQ1	-0,02	-0,28	-0,26
PES1	-0,25	-0,51	-0,26
PET112L	-0,51	0,69	1,20
PEX1	0,19	-0,24	-0,43
PEX11B	0,24	0,22	-0,01
PEX11G	-0,15	1,10	1,25
PEX13	0,14	0,94	0,80
PEX14	-1,09	0,58	1,67
PEX19	-0,33	1,25	1,58
PEX26	-0,13	0,18	0,31
PEX3	-0,41	0,42	0,82
PEX5	-0,27	-0,79	-0,52
PEX6	-0,73	-0,97	-0,24
PEX7	-0,27	0,50	0,77
PFAAP5	-0,13	-2,21	-2,08
PFAS	0,06	0,12	0,06
PFDN1	-1,21	0,15	1,35
PFDN2	-0,25	-0,23	0,03
PFDN4	0,60	0,81	0,21
PFDN5	0,06	-0,37	-0,43
PFDN6	-0,07	-0,27	-0,20
PFKFB2	0,10	0,88	0,78
PFKM	-0,26	0,70	0,96
PFKP	1,89	3,77	1,89
PFTK1	-2,55	0,26	2,81
PGAM1	-1,34	0,51	1,85

PGAM4	-0,93	-0,08	0,85
PGBD2	0,17	0,15	-0,03
PGBD3	0,92	0,40	-0,52
PGBD4	-2,72	-2,02	0,69
PGBD5	-1,89	-1,00	0,89
PGC	0,41	0,37	-0,04
PGD	-0,68	0,31	0,99
PGDS	-1,60	5,30	6,90
PGGT1B	-0,23	-1,10	-0,87
PGK1	-0,27	0,22	0,49
PGK2	0,07	-0,24	-0,31
PGLS	-0,02	-1,18	-1,16
PGLYRP4	0,20	0,09	-0,11
PGM1	-1,15	-1,24	-0,09
PGM2L1	-1,20	3,97	5,17
PGM3	-0,86	1,53	2,38
PGPEP1	0,19	1,43	1,24
PHACS	-0,10	-1,26	-1,16
PHACTR1	-0,52	4,03	4,55
PHACTR2	-0,21	0,23	0,43
PHACTR4	-0,32	-0,19	0,13
PHB	-0,24	0,72	0,96
PHB2	0,07	-0,34	-0,40
PHC1	0,66	1,00	0,34
PHC2	-0,52	-1,37	-0,84
PHF1	0,18	0,29	0,11
PHF11	0,07	-1,32	-1,38
PHF12	0,55	-0,60	-1,15
PHF13	0,27	0,79	0,52
PHF14	-0,02	-0,61	-0,59
PHF17	0,04	-1,00	-1,04
PHF19	0,11	-0,25	-0,35
PHF2	0,58	-1,17	-1,75
PHF20	0,36	-0,98	-1,33
PHF20L1	0,25	-0,68	-0,92
PHF21A	-0,04	-1,60	-1,56
PHF23	0,44	0,81	0,37
PHF3	0,37	-0,94	-1,31
PHF5A	0,10	-0,42	-0,51
PHF7	0,27	0,71	0,45
PHGDH	1,50	3,99	2,49
PHIP	1,04	-0,95	-1,99
PHKA2	0,11	-1,12	-1,23
PHKB	0,29	-0,31	-0,60
PHLDA1	2,70	6,46	3,76
PHLDA3	-1,33	11,77	13,10
PHLDB1	-0,46	5,81	6,27
PHLDB3	1,08	1,05	-0,03
PHLPP	-0,05	1,80	1,84
PHLPPL	1,36	1,10	-0,26
PHOSPHO2	0,05	6,29	6,24
PHTF2	0,77	1,71	0,94
PHYH	-0,04	1,28	1,32
PHYHIPL	-0,29	-0,25	0,04
PI4K2B	-0,64	-0,07	0,56
PIAS1	0,23	-0,91	-1,14
PIAS4	0,07	-0,32	-0,39
PIB5PA	0,16	0,52	0,36
PICALM	1,28	0,92	-0,35

PIGB	-0,43	-0,62	-0,19
PIGC	-0,05	-0,57	-0,52
PIGF	-0,47	-0,04	0,43
PIGG	-0,08	0,77	0,86
PIGM	-0,94	-1,68	-0,75
PIGN	0,05	0,80	0,75
PIGO	0,00	-0,04	-0,04
PIGP	-0,32	0,48	0,80
PIGS	0,19	-0,19	-0,38
PIGT	-0,67	0,21	0,88
PIGV	-0,29	0,58	0,88
PIGW	0,47	0,52	0,05
PIGX	0,07	-0,96	-1,04
PIGY	0,02	-0,20	-0,22
PIGZ	0,11	0,58	0,47
PIK3C2A	-0,26	0,31	0,56
PIK3C3	-0,37	-0,31	0,07
PIK3CB	-0,69	0,22	0,92
PIK3CD	0,47	-0,71	-1,18
PIK3CG	-0,15	0,61	0,76
PIK3R1	1,40	1,17	-0,22
PIK3R4	-0,09	0,29	0,38
PIK3R5	1,06	-0,33	-1,38
PIK4CA	0,12	0,22	0,10
PILRB	0,44	-1,77	-2,20
PIM1	-2,60	-0,13	2,46
PIM2	1,57	0,38	-1,19
PIN4	-0,49	-1,10	-0,61
PIP3-E	1,45	-0,28	-1,73
PIP5K1A	0,10	0,51	0,41
PIP5K1C	0,46	1,04	0,57
PIP5K2B	0,21	-1,37	-1,58
PIP5K3	0,04	0,07	0,03
PIR	-0,84	7,91	8,75
PITPNA	1,73	1,68	-0,05
PITPNB	-0,03	0,43	0,46
PITX3	-0,04	0,60	0,64
PIWIL4	0,02	-0,56	-0,58
PKD1	0,53	-0,23	-0,77
PKD1L1	-0,28	0,88	1,16
PKD2	1,28	2,48	1,20
PKD2L1	-2,66	0,77	3,43
PKIB	3,39	4,06	0,67
PKLR	-0,48	0,38	0,86
PKM2	-0,64	0,78	1,42
PKN1	0,69	-0,87	-1,56
PKP2	-3,55	-5,54	-1,99
PKP4	-0,48	-1,16	-0,68
PLA1A	-1,41	6,10	7,50
PLA2G10	0,42	1,39	0,97
PLA2G12A	-0,53	0,81	1,34
PLA2G12B	-0,17	0,18	0,35
PLA2G2E	-0,43	0,08	0,52
PLA2G4A	0,53	1,85	1,33
PLA2G4B	0,40	-2,31	-2,71
PLA2G5	9,34	5,09	-4,24
PLA2G6	0,30	0,46	0,16
PLA2G7	0,16	2,78	2,62
PLAA	0,09	-0,01	-0,10

PLAC1	-0,02	-0,18	-0,16
PLAC4	-0,12	0,24	0,36
PLAG1	0,75	0,23	-0,52
PLAGL2	-0,06	-1,03	-0,97
PLAU	0,12	6,11	5,99
PLAUR	-1,58	-0,01	1,58
PLB1	-0,64	-1,58	-0,94
PLCB1	0,92	-1,60	-2,52
PLCB3	0,50	-0,10	-0,60
PLCG2	0,43	0,71	0,28
PLCL1	0,03	1,92	1,89
PLCL2	-0,11	-2,00	-1,89
PLCXD3	0,13	0,16	0,03
PLD1	-0,92	0,62	1,54
PLD2	0,49	-0,64	-1,12
PLD3	-0,83	1,18	2,01
PLDN	1,81	1,87	0,07
PLEC1	0,68	-0,36	-1,04
PLEKHA1	0,73	0,79	0,06
PLEKHA9	-0,56	-1,22	-0,66
PLEKHB1	-0,21	3,28	3,50
PLEKHB2	-0,04	1,46	1,50
PLEKHG2	0,12	-0,22	-0,34
PLEKHG4	0,05	1,49	1,44
PLEKHG5	-0,33	0,61	0,94
PLEKHJ1	0,56	0,18	-0,38
PLEKHM1	-0,25	-0,49	-0,24
PLEKHM2	0,32	1,16	0,84
PLK3	-0,86	-1,11	-0,25
PLOD3	0,49	0,78	0,29
PLP2	-0,37	-2,68	-2,31
PLXDC2	-0,54	0,40	0,94
PLXNA1	0,05	3,32	3,27
PLXNC1	-1,28	-2,01	-0,74
PLXND1	-0,76	0,05	0,80
PMCHL1	-0,04	-0,62	-0,58
PMF1	0,06	-1,37	-1,43
PMFBP1	-0,25	3,35	3,60
PMM2	-0,40	0,12	0,51
PMP22	-1,78	2,57	4,35
PMP22CD	-0,06	-0,06	0,00
PMPCA	0,32	-0,13	-0,45
PMPCB	0,02	-0,28	-0,30
PMS1	0,56	-0,35	-0,91
PMS2	-1,70	-2,37	-0,67
PMS2L2	-1,66	-2,25	-0,59
PMS2L5	-1,12	-1,78	-0,66
PMVK	-0,10	0,20	0,30
PNKD	-1,54	-1,12	0,41
PNLDC1	0,10	0,34	0,24
PNLIPRP1	-1,36	-2,33	-0,97
PNLIPRP2	-0,08	-0,07	0,01
PNMA1	-0,19	2,02	2,22
PNMA5	-0,45	-6,12	-5,66
PNOC	0,57	-1,43	-2,00
PNPLA1	-1,31	-2,21	-0,89
PNPLA4	-1,53	-0,70	0,83
PNPLA5	-0,24	0,29	0,54
PNPO	-0,28	0,96	1,24

PNPT1	0,02	-0,99	-1,01
PODN	-0,95	-0,66	0,29
PODXL2	0,45	-1,52	-1,98
POFUT1	0,03	-0,58	-0,61
POFUT2	-0,25	-0,76	-0,50
POGK	0,23	0,43	0,21
POGZ	0,59	-0,98	-1,57
POLA2	-0,32	0,42	0,74
POLD3	-0,31	-1,48	-1,17
POLD4	-0,03	-0,34	-0,31
POLDIP2	-0,10	0,16	0,26
POLE	-0,14	-0,57	-0,44
POLE3	0,13	-0,08	-0,22
POLE4	0,31	0,22	-0,09
POLG2	0,63	-0,34	-0,97
POLH	-0,03	-0,12	-0,10
POLI	2,83	-5,47	-8,30
POLL	-0,26	-0,78	-0,52
POLR1A	-0,21	2,00	2,21
POLR1C	0,09	0,43	0,35
POLR2A	0,52	0,29	-0,22
POLR2B	0,26	-0,03	-0,29
POLR2C	-0,22	-0,56	-0,35
POLR2G	0,02	-0,05	-0,07
POLR2I	-0,30	0,03	0,32
POLR2J	0,01	-0,22	-0,23
POLR2K	0,04	-0,47	-0,51
POLR2L	0,67	0,58	-0,10
POLR3A	-0,05	0,02	0,07
POLR3B	-0,56	-0,12	0,45
POLR3C	0,25	-0,12	-0,37
POLR3D	0,18	0,13	-0,05
POLR3E	0,03	-0,32	-0,35
POLR3F	0,20	0,09	-0,11
POLR3K	0,07	1,00	0,93
POMGNT1	-0,67	0,89	1,56
POMT1	0,11	0,86	0,75
POMT2	0,46	0,15	-0,31
POMZP3	0,02	0,04	0,02
POP1	0,00	1,96	1,96
POPDC2	0,28	0,00	-0,28
PORCN	-0,47	-0,62	-0,15
POU2F1	0,23	-0,17	-0,40
POU5F1	-0,80	-0,29	0,51
PPA1	-0,31	-1,36	-1,06
PPA2	-0,49	1,16	1,65
PPAN	0,40	0,77	0,37
PPAP2B	0,75	12,12	11,37
PPAPDC1B	-0,53	0,70	1,24
PPAPDC2	0,14	1,62	1,48
PPAPDC3	-3,70	-4,60	-0,90
PPARBP	0,44	-0,05	-0,49
PPARD	-1,32	1,73	3,06
PPARG	0,19	5,61	5,42
PPARGC1A	3,01	-5,33	-8,34
PPAT	-0,17	1,27	1,44
PPBP	-2,41	-0,67	1,75
PPCS	0,24	0,46	0,21
PPEF1	-0,21	-2,15	-1,94

PPEF2	0,31	-0,13	-0,44
PPFIA4	0,39	-1,68	-2,07
PPFIBP2	1,70	1,88	0,18
PPHLN1	0,24	0,18	-0,06
PPID	-0,07	0,33	0,39
PPIF	-0,87	-1,18	-0,31
PPIH	0,33	0,09	-0,24
PPIL1	0,25	1,58	1,33
PPIL2	0,27	0,27	0,00
PPIL3	0,35	1,10	0,76
PPM1A	-0,05	-0,03	0,02
PPM1B	0,41	-1,52	-1,92
PPM1D	0,58	0,59	0,00
PPM1F	1,04	-1,63	-2,66
PPM1G	0,40	-0,06	-0,46
PPM1K	-0,32	0,33	0,65
PPM1M	0,56	0,68	0,12
PPM2C	0,45	-2,29	-2,74
PPME1	-0,27	1,42	1,69
PPOX	-0,14	-0,83	-0,69
PPP1CB	0,11	-1,88	-1,99
PPP1R10	0,96	1,35	0,39
PPP1R11	-0,23	-0,51	-0,28
PPP1R12A	0,62	-0,42	-1,04
PPP1R12C	0,27	-0,67	-0,94
PPP1R13L	0,52	-0,18	-0,70
PPP1R14B	0,35	1,17	0,82
PPP1R15B	0,73	-0,69	-1,42
PPP1R16A	1,02	2,01	0,99
PPP1R1B	0,37	0,48	0,11
PPP1R3D	0,20	-0,29	-0,48
PPP1R3F	-0,22	1,11	1,33
PPP1R7	1,16	1,64	0,47
PPP1R8	0,99	-0,38	-1,38
PPP2CB	-0,19	0,58	0,77
PPP2R1A	-0,16	0,34	0,49
PPP2R1B	-0,31	0,37	0,68
PPP2R2A	-0,04	0,46	0,51
PPP2R2B	2,10	4,46	2,36
PPP2R2D	0,18	0,38	0,20
PPP2R3A	-0,20	-0,57	-0,37
PPP2R3B	-0,12	-0,25	-0,13
PPP2R4	0,19	0,99	0,80
PPP2R5C	-0,21	-0,65	-0,44
PPP3CA	-0,38	-0,18	0,20
PPP3CB	-0,07	-0,45	-0,38
PPP3CC	-0,33	0,06	0,39
PPP3R1	-0,06	-0,77	-0,70
PPP4R1L	-0,37	-2,81	-2,44
PPP4R2	-0,03	-0,01	0,02
PPP5C	-0,15	-0,17	-0,02
PPP6C	0,19	-0,38	-0,57
PPRC1	0,25	0,22	-0,03
PPT2	-0,67	-0,71	-0,03
PPWD1	0,29	-0,97	-1,26
PQBP1	0,57	-0,64	-1,21
PQLC1	0,39	0,30	-0,09
PRAF2	0,02	-0,37	-0,39
PRAME	-0,69	-0,08	0,61

PRB4	-0,19	1,82	2,01
PRCC	0,24	-0,17	-0,40
PRCP	0,67	0,77	0,09
PRDM1	-0,51	0,77	1,29
PRDM10	0,02	-0,50	-0,52
PRDM15	0,04	0,38	0,35
PRDM4	0,04	0,60	0,56
PRDM7	0,87	-0,49	-1,36
PRDX1	-0,26	1,42	1,68
PRDX5	0,04	0,12	0,08
PRDX6	-0,64	0,30	0,94
PREI3	-0,18	-0,77	-0,58
PREP	-0,12	-0,11	0,01
PREPL	0,39	0,33	-0,06
PREX1	0,76	-2,18	-2,95
PRF1	0,38	0,13	-0,25
PRH1	-0,23	-0,90	-0,68
PRIM1	-1,08	0,31	1,39
PRIM2A	-0,39	-0,20	0,20
PRKAB2	0,01	-0,99	-0,99
PRKCA	-0,32	1,22	1,53
PRKCB1	-1,24	-2,79	-1,55
PRKCE	-0,68	-1,76	-1,08
PRKCH	-2,82	-1,71	1,10
PRKCI	0,70	-0,21	-0,91
PRKCQ	0,17	0,25	0,09
PRKCSH	-0,13	-0,89	-0,76
PRKD3	0,75	-0,02	-0,76
PRKDC	-0,08	-0,36	-0,28
PRKRIR	0,29	0,22	-0,07
PRMT2	0,31	-0,28	-0,60
PRMT3	-0,34	-0,26	0,08
PRMT5	0,05	-0,14	-0,19
PRMT7	-0,10	-0,45	-0,35
PRNPIP	-0,34	-0,42	-0,08
PROS1	-1,84	2,86	4,70
PROSC	0,03	-0,39	-0,42
PRPF18	-0,13	-0,37	-0,24
PRPF19	-0,15	-0,35	-0,19
PRPF31	0,02	-0,70	-0,72
PRPF38A	-0,11	-1,07	-0,96
PRPF38B	0,33	-0,72	-1,05
PRPF39	0,59	-1,59	-2,18
PRPF4	-0,06	0,03	0,09
PRPF4B	-0,22	-1,69	-1,47
PRPF6	-0,19	-0,62	-0,44
PRPF8	0,42	0,47	0,04
PRPS1	1,60	2,31	0,71
PRPS1L1	1,49	1,22	-0,27
PRPS2	0,01	0,31	0,31
PRPSAP1	-0,31	-0,68	-0,36
PRPSAP2	0,02	-0,70	-0,72
PRR11	-0,40	0,00	0,39
PRR14	0,63	-0,49	-1,12
PRR3	0,20	-0,47	-0,67
PRR4	0,55	0,49	-0,06
PRR5	-1,36	0,86	2,22
PRR8	0,58	-2,34	-2,92
PRRG1	-0,86	0,60	1,47

PRRG2	-1,93	-1,32	0,61
PRRG4	0,70	-1,25	-1,96
PRRT2	0,60	0,18	-0,42
PRRT3	2,07	2,11	0,03
PRSS2	-2,56	0,14	2,70
PRSS21	-0,27	2,37	2,64
PRSS3	-0,93	0,69	1,62
PRUNE	0,25	-0,71	-0,96
PRX	-0,50	-0,78	-0,29
PSCD1	0,20	-0,07	-0,27
PSCD2	0,78	-0,66	-1,44
PSCD3	-0,75	-0,85	-0,10
PSCDBP	0,60	-0,73	-1,33
PSD3	-1,43	6,61	8,03
PSEN1	-0,42	-0,20	0,22
PSEN2	0,35	2,81	2,47
PSENEN	-0,37	-0,56	-0,19
PSG9	0,05	-0,92	-0,97
PSMA1	-0,33	-0,18	0,15
PSMA2	0,26	-0,16	-0,43
PSMA3	-0,36	0,39	0,74
PSMA4	-0,13	-0,45	-0,32
PSMA5	-0,11	0,42	0,53
PSMA6	-0,32	0,03	0,35
PSMA7	-0,57	0,60	1,16
PSMA8	-0,65	-1,30	-0,64
PSMB1	-0,07	0,03	0,10
PSMB10	0,13	-0,64	-0,77
PSMB3	-0,16	0,02	0,18
PSMB6	-0,76	-0,13	0,63
PSMB7	-0,08	-0,31	-0,23
PSMB8	-0,66	-0,78	-0,11
PSMB9	-0,63	-1,79	-1,17
PSMC1	-0,27	0,36	0,63
PSMC3	-0,56	0,29	0,85
PSMC3IP	0,10	-0,02	-0,12
PSMC4	-0,28	0,64	0,92
PSMC5	0,14	0,02	-0,12
PSMC6	-0,24	0,55	0,79
PSMD1	-0,44	0,77	1,21
PSMD10	-0,51	0,60	1,11
PSMD13	-0,45	-0,57	-0,13
PSMD14	-0,18	1,69	1,87
PSMD2	-0,18	0,39	0,57
PSMD5	-0,31	0,28	0,59
PSMD7	-0,17	0,46	0,64
PSMD9	-0,91	-2,22	-1,31
PSME2	-0,35	-0,39	-0,05
PSME3	0,01	-0,49	-0,51
PSMF1	0,12	-0,27	-0,39
PSORS1C1	0,07	-0,58	-0,64
PSORS1C2	0,24	1,01	0,77
PSPC1	0,20	-0,80	-1,00
PSPH	-0,22	1,67	1,89
PSTPIP2	2,73	0,93	-1,79
PTAFR	-1,04	-1,00	0,04
PTCD1	0,25	0,86	0,62
PTCD2	-0,19	-0,38	-0,18
PTDSS1	-0,36	-0,34	0,02

PTDSS2	-0,07	-0,33	-0,26
PTEN	0,45	-1,83	-2,27
PTGER2	0,62	-0,22	-0,84
PTGER4	0,02	0,72	0,70
PTK2B	-1,59	-3,02	-1,42
PTP4A3	0,11	-1,99	-2,10
PTPLAD1	-0,46	2,53	2,99
PTPLAD2	0,08	-1,44	-1,52
PTPLB	0,54	0,45	-0,09
PTPN1	-0,51	0,25	0,75
PTPN12	-0,22	-0,34	-0,12
PTPN18	0,47	-0,22	-0,69
PTPN2	1,01	-0,29	-1,30
PTPN22	-0,99	-0,47	0,53
PTPN3	-0,48	0,18	0,66
PTPN5	-0,83	-1,00	-0,17
PTPN6	-0,41	-1,13	-0,72
PTPN7	0,61	2,83	2,22
PTPRA	-0,04	0,45	0,49
PTPRCAP	0,53	-0,29	-0,83
PTPRE	1,26	0,56	-0,70
PTPRO	1,00	5,37	4,37
PTPRR	-1,14	-0,54	0,59
PTRH1	0,31	1,00	0,68
PTS	-0,58	0,03	0,61
PTTG1	-2,43	0,82	3,25
PTX3	-2,09	-1,97	0,13
PUM2	0,62	-0,95	-1,57
PUS7L	0,27	0,84	0,57
PVR	0,55	0,36	-0,19
PVRL4	-0,59	1,95	2,54
PWP1	-0,11	0,27	0,38
PXMP2	-1,34	-0,39	0,95
PXN	0,89	-1,27	-2,16
PXT1	-0,08	-0,08	0,00
PYCARD	-0,42	-1,27	-0,84
PYCR2	-0,10	0,20	0,30
PYGM	-0,09	-0,18	-0,09
PYGO2	0,84	-0,09	-0,93
PYHIN1	-6,79	0,00	6,79
QARS	0,34	-0,06	-0,40
QDPR	-0,85	0,40	1,25
QPCT	0,26	1,43	1,17
QPCTL	-0,87	0,22	1,10
QRICH1	0,49	-0,30	-0,78
QRSL1	2,98	3,88	0,91
QTRT1	-0,34	-0,17	0,17
QTRTD1	0,10	-0,30	-0,40
R3HDM1	-0,16	-0,71	-0,55
R3HDM2	0,46	-1,03	-1,49
R3HDML	0,27	1,07	0,81
RAB10	0,31	0,45	0,15
RAB11FIP1	0,03	-0,91	-0,94
RAB11FIP2	-0,08	-0,37	-0,29
RAB11FIP3	0,47	0,24	-0,23
RAB12	0,52	-2,91	-3,43
RAB13	-1,12	2,30	3,43
RAB20	-0,70	0,39	1,09
RAB21	0,04	0,20	0,16

RAB22A	-0,10	0,37	0,47
RAB23	-1,37	1,02	2,40
RAB24	0,26	-2,62	-2,88
RAB27A	-1,83	-3,64	-1,81
RAB28	-0,56	-0,85	-0,29
RAB2B	0,07	0,52	0,46
RAB30	1,71	1,33	-0,38
RAB31	0,42	0,41	-0,01
RAB32	0,39	-0,02	-0,41
RAB33B	0,59	-1,92	-2,50
RAB35	0,64	0,44	-0,21
RAB36	-4,87	-0,90	3,97
RAB37	-0,18	-2,42	-2,24
RAB40C	0,26	-1,48	-1,74
RAB42	0,14	0,45	0,31
RAB4A	-0,19	-0,29	-0,11
RAB4B	-0,05	-0,17	-0,12
RAB5A	0,56	0,47	-0,09
RAB5B	0,22	-0,03	-0,25
RAB5C	0,14	-0,21	-0,36
RAB6A	0,19	0,07	-0,13
RAB7L1	0,01	0,12	0,11
RABGAP1	0,66	0,13	-0,53
RABGAP1L	0,11	-1,33	-1,44
RABGGTA	-0,48	0,11	0,60
RABGGTB	0,00	0,19	0,19
RABIF	-0,18	-0,94	-0,76
RABL2A	-0,11	-0,29	-0,18
RABL2B	0,29	-0,28	-0,57
RABL3	-0,35	1,80	2,14
RABL4	-0,63	0,06	0,68
RACGAP1	-0,60	1,48	2,08
RAD1	0,29	0,47	0,18
RAD17	-0,22	0,06	0,28
RAD18	-0,32	5,13	5,45
RAD23A	0,19	0,25	0,06
RAD23B	0,21	-1,08	-1,29
RAD50	0,14	-0,09	-0,23
RAD51	-7,89	0,39	8,28
RAD51AP1	-1,15	1,93	3,08
RAD51C	-0,69	0,91	1,60
RAD51L3	-0,17	0,35	0,52
RAD54L	-3,39	3,12	6,51
RAD54L2	0,40	0,03	-0,38
RAI1	0,56	-0,15	-0,70
RALA	-0,90	0,50	1,41
RALB	-0,29	-0,42	-0,13
RALGDS	-0,13	1,87	2,00
RALGPS1	0,07	-0,52	-0,59
RALY	0,63	-0,10	-0,73
RAMP1	8,22	9,11	0,89
RANBP1	-0,50	0,06	0,56
RANBP10	-0,27	-0,90	-0,63
RANBP3	0,60	0,46	-0,14
RANBP5	0,05	-0,58	-0,63
RANBP6	-0,18	-0,75	-0,57
RANBP9	0,36	0,26	-0,09
RAP1A	0,68	-0,26	-0,94
RAP1B	-0,14	-0,50	-0,35

RAP2C	0,20	-0,20	-0,41
RAPGEF1	0,21	1,08	0,87
RAPGEF3	-0,86	4,65	5,51
RAPGEF6	0,11	-0,48	-0,59
RAPH1	0,67	2,66	1,99
RAPSN	0,22	-0,08	-0,31
RARA	-0,20	-2,17	-1,97
RARRES1	-0,37	11,08	11,45
RARS	-0,21	0,71	0,93
RASAL2	0,09	4,31	4,23
RASD2	-0,98	-0,24	0,74
RASGEF1B	-2,67	-0,09	2,58
RASGRF1	-1,35	0,05	1,40
RASGRP1	1,36	7,21	5,85
RASGRP2	-0,29	-4,50	-4,21
RASGRP4	0,15	-3,05	-3,20
RASL10B	0,53	0,15	-0,38
RASL11B	-3,23	6,46	9,69
RASSF1	0,86	-0,59	-1,44
RASSF2	0,82	-1,57	-2,39
RASSF4	-1,17	0,00	1,17
RASSF5	-0,71	-1,69	-0,98
RASSF7	1,04	0,19	-0,84
RAVER1	0,30	-0,62	-0,93
RAXL1	0,04	-1,81	-1,85
RB1	0,16	-0,54	-0,70
RB1CC1	-0,01	-0,73	-0,72
RBAK	0,54	-0,06	-0,61
RBBP4	0,25	-0,32	-0,57
RBBP5	0,17	0,18	0,01
RBBP6	-0,40	-1,82	-1,42
RBBP8	-0,37	-0,86	-0,49
RBED1	0,50	-0,42	-0,92
RBJ	-0,48	-0,74	-0,26
RBKS	-0,08	-0,58	-0,50
RBL2	0,33	-0,91	-1,24
RBM10	0,38	-0,43	-0,80
RBM12	0,05	-0,34	-0,40
RBM13	-0,08	0,77	0,85
RBM14	0,34	-0,23	-0,58
RBM15B	0,27	-0,60	-0,87
RBM16	0,47	-0,64	-1,11
RBM17	-0,15	-1,77	-1,62
RBM18	-0,07	0,46	0,53
RBM22	0,17	-0,73	-0,90
RBM23	0,04	-0,75	-0,79
RBM25	0,30	-0,41	-0,71
RBM28	0,28	0,07	-0,21
RBM3	-0,21	-0,88	-0,67
RBM33	0,60	-1,86	-2,46
RBM34	0,10	0,00	-0,10
RBM4	0,72	-0,84	-1,56
RBM7	0,59	0,16	-0,43
RBM8A	-0,90	-0,68	0,22
RBMX	0,20	-0,60	-0,80
RBMX2	0,27	-0,38	-0,66
RCBTB1	0,73	0,87	0,13
RCBTB2	-2,27	-1,94	0,33
RCE1	0,37	-0,16	-0,52

RCHY1	0,36	-1,26	-1,62
RCL1	-0,77	1,28	2,05
RCN2	-0,31	1,39	1,70
RCN3	-0,29	-2,32	-2,02
RCOR3	0,52	-0,69	-1,21
RCP9	0,12	0,16	0,04
RDBP	0,03	-0,17	-0,20
RDH10	-0,64	0,91	1,55
RDH11	-0,20	1,29	1,49
RDH14	-0,19	0,24	0,43
RDH5	-0,02	-1,30	-1,27
RDH8	0,03	0,31	0,28
RDHE2	0,35	-0,13	-0,48
RDM1	-0,18	-0,02	0,15
RDX	-0,63	1,30	1,93
RECQL	0,15	0,18	0,03
RECQL4	-4,37	-2,62	1,75
RECQL5	0,22	-0,43	-0,65
REEP3	-0,78	1,50	2,28
REEP4	0,19	-0,91	-1,10
REEP5	0,97	0,26	-0,71
REG1A	0,55	0,28	-0,27
REP15	1,44	2,06	0,62
REPIN1	0,92	0,31	-0,62
REPS1	-0,13	-0,31	-0,19
REPS2	0,71	-1,51	-2,22
RER1	-0,04	0,92	0,96
RETNLB	0,00	-0,39	-0,39
REV3L	0,39	-1,05	-1,45
REXO2	-0,55	0,53	1,08
RFC1	-0,26	-0,43	-0,18
RFC3	-1,60	-0,77	0,82
RFC5	-0,77	-0,47	0,30
RFFL	0,11	-0,74	-0,85
RFK	-0,05	0,57	0,63
RFNG	0,34	0,50	0,16
RFP	0,19	-1,58	-1,77
RFT1	-0,19	0,73	0,91
RFWD2	0,03	-0,38	-0,40
RFWD3	-0,03	0,77	0,80
RFX2	0,03	-0,59	-0,63
RFX4	-0,42	-0,50	-0,08
RFXANK	0,18	-0,35	-0,53
RFXAP	1,40	0,72	-0,68
RFXDC2	-0,12	-0,17	-0,04
RG9MTD1	0,22	1,25	1,03
RGAG1	0,09	1,52	1,42
RGAG4	0,33	1,62	1,29
RGL1	1,60	4,49	2,89
RGL2	0,92	-0,63	-1,54
RGMA	0,02	-1,33	-1,35
RGR	0,76	-1,21	-1,97
RGS1	-0,72	9,13	9,85
RGS10	0,59	0,22	-0,37
RGS12	-0,17	2,39	2,56
RGS14	1,42	-1,17	-2,58
RGS20	-4,48	6,61	11,09
RHBDD2	-1,02	0,31	1,33
RHBDD3	0,14	0,85	0,70

RHBDF1	-0,78	7,11	7,88
RHOA	0,09	-0,38	-0,47
RHOBTB3	-1,27	8,00	9,26
RHOQ	-0,08	0,17	0,26
RHOT1	-0,25	-1,00	-0,75
RHOT2	0,24	-0,35	-0,60
RHOU	-0,93	-0,73	0,20
RHPN1	1,92	3,82	1,90
RIC8A	0,00	-0,06	-0,05
RIN2	0,18	0,46	0,29
RINT1	-0,34	-0,02	0,32
RIOK1	-0,44	-0,96	-0,52
RIOK2	0,02	0,63	0,62
RIPK1	0,11	-0,09	-0,20
RIPK5	-0,09	-0,28	-0,19
RIT1	-0,31	0,75	1,06
RLBP1	0,26	0,90	0,64
RLN1	-0,24	0,18	0,42
RMND5A	-0,36	-3,42	-3,06
RNASE1	3,64	5,28	1,64
RNASE10	-0,25	-0,09	0,16
RNASE2	-2,24	-8,06	-5,82
RNASE3	-1,24	-4,64	-3,41
RNASE4	-1,44	-3,41	-1,97
RNASEH1	-0,35	-0,49	-0,14
RNASEN	0,07	-0,26	-0,33
RNF10	0,08	-1,43	-1,51
RNF103	-0,48	0,24	0,72
RNF113A	0,03	-0,14	-0,17
RNF113B	-0,76	-0,78	-0,02
RNF12	-0,40	-1,41	-1,00
RNF121	-0,38	0,53	0,90
RNF123	-0,03	-0,18	-0,15
RNF125	-0,36	-1,24	-0,88
RNF13	0,09	0,46	0,37
RNF138	0,22	-1,94	-2,16
RNF144	-2,94	-3,28	-0,34
RNF146	0,27	0,03	-0,25
RNF166	0,42	-1,82	-2,24
RNF167	0,11	-0,29	-0,41
RNF168	0,34	-0,23	-0,58
RNF17	0,66	-0,14	-0,80
RNF170	0,36	1,07	0,70
RNF180	-0,02	-0,18	-0,17
RNF185	-0,17	0,90	1,07
RNF186	-0,51	-0,97	-0,47
RNF19	0,75	0,80	0,05
RNF20	0,20	-0,19	-0,40
RNF25	-0,31	-0,12	0,19
RNF26	-0,28	-0,34	-0,07
RNF31	0,78	-0,70	-1,48
RNF32	-0,19	-0,24	-0,04
RNF38	0,38	-1,08	-1,46
RNF40	0,32	-0,32	-0,64
RNF41	-0,32	-1,09	-0,77
RNF44	0,82	-1,64	-2,46
RNF6	-0,19	0,61	0,80
RNF7	-0,24	0,19	0,43
RNGTT	-0,26	0,25	0,51

RNH1	0,71	0,73	0,02
RNMT	0,44	0,87	0,43
RNMTL1	-0,01	0,03	0,04
RNPC2	0,53	-1,97	-2,50
RNPC3	0,04	-0,69	-0,73
RNPEP	-0,25	-0,05	0,20
RNUXA	-0,37	0,22	0,59
ROBO4	0,11	-0,92	-1,03
ROCK1	-0,25	-0,26	-0,01
ROCK2	0,12	-0,61	-0,73
ROD1	0,38	-0,61	-0,99
ROPN1L	-1,66	-5,38	-3,72
RORA	-0,57	-0,31	0,26
RORC	-1,14	-0,11	1,03
RP11-49G10.8	0,27	-1,03	-1,30
RP11-529I10.4	-0,35	-0,18	0,17
RP13-15M17.2	0,27	2,64	2,37
RP9	0,17	-0,07	-0,24
RPA1	-0,39	0,24	0,63
RPA3	-0,38	0,91	1,29
RPA4	0,24	-2,32	-2,56
RPGR	-0,61	-2,22	-1,61
RPH3A	-0,09	-0,90	-0,81
RPH3AL	-1,22	0,60	1,82
RPIA	0,83	-0,68	-1,52
RPL12	0,24	-0,12	-0,36
RPL13	0,32	-1,52	-1,84
RPL13A	0,59	-0,55	-1,14
RPL14	0,10	-0,84	-0,93
RPL15	-0,07	-0,99	-0,93
RPL17	0,13	-0,50	-0,63
RPL19	-0,03	-0,59	-0,55
RPL26	0,15	-0,79	-0,94
RPL26L1	-0,54	-0,30	0,24
RPL27	0,07	-0,33	-0,40
RPL27A	0,30	-0,92	-1,22
RPL29	-0,35	0,91	1,27
RPL30	-0,03	-0,68	-0,66
RPL32	0,11	-0,33	-0,44
RPL34	-0,03	-0,84	-0,81
RPL35A	0,06	-0,93	-0,99
RPL36A	0,37	-0,83	-1,20
RPL36AL	0,07	-0,12	-0,19
RPL37	-0,19	-1,49	-1,30
RPL37A	0,01	-0,67	-0,69
RPL38	0,08	-0,46	-0,54
RPL39	-0,01	-0,52	-0,50
RPL4	0,49	-0,68	-1,17
RPL41	-1,00	-0,91	0,09
RPL6	0,09	-0,31	-0,39
RPL7	0,17	-1,21	-1,39
RPL7A	0,32	-0,93	-1,26
RPL7L1	0,10	0,42	0,32
RPL8	0,18	-0,23	-0,41
RPL9	0,04	-0,22	-0,26
RPLP0	0,22	0,46	0,24
RPLP1	0,26	-0,74	-0,99
RPLP2	0,18	-0,46	-0,64
RPN2	-0,25	0,04	0,29

RPP14	0,00	0,21	0,21
RPP21	-0,03	-0,97	-0,94
RPP38	0,37	0,92	0,55
RPP40	-0,44	-1,76	-1,32
RPS11	0,18	-0,20	-0,38
RPS12	-0,13	-0,20	-0,06
RPS14	0,17	-0,58	-0,75
RPS16	0,09	-0,04	-0,13
RPS17	0,52	-0,23	-0,75
RPS18	0,37	-0,56	-0,93
RPS19	0,08	-0,04	-0,12
RPS21	-0,48	-0,49	-0,01
RPS23	0,22	-1,28	-1,51
RPS24	0,11	-0,03	-0,14
RPS25	0,05	-0,29	-0,34
RPS26	0,23	1,09	0,85
RPS27A	0,19	-0,47	-0,66
RPS27L	0,01	1,17	1,16
RPS28	0,18	0,07	-0,11
RPS29	0,07	0,13	0,06
RPS3	0,04	-0,66	-0,70
RPS4X	0,09	-0,54	-0,64
RPS5	-0,01	-0,17	-0,15
RPS6	0,05	-0,80	-0,84
RPS6KA1	-0,14	0,88	1,03
RPS6KB1	0,06	-1,44	-1,50
RPS6KL1	0,63	0,05	-0,58
RPS7	-0,73	0,18	0,91
RPSA	-0,15	-0,85	-0,69
RPUSD1	0,35	-0,60	-0,96
RPUSD3	-0,01	-0,01	0,01
RPUSD4	0,07	-0,50	-0,57
RQCD1	-0,15	1,33	1,49
RRAGA	-0,13	-0,03	0,10
RRAGB	-0,30	0,16	0,46
RRAGC	0,37	1,32	0,95
RRAS	-0,99	-0,48	0,51
RREB1	0,10	-0,03	-0,13
RRM1	-0,59	1,07	1,66
RRS1	2,32	2,06	-0,27
RS1	-0,34	0,37	0,72
RSAD2	-4,88	-6,88	-2,00
RSC1A1	-0,56	1,00	1,57
RSPO3	-1,97	2,34	4,31
RSPRY1	0,34	0,19	-0,15
RSRC1	-0,34	0,79	1,12
RTCD1	-0,14	1,29	1,43
RTEL1	0,49	0,58	0,09
RTF1	-0,35	-0,81	-0,46
RTN3	-0,29	-1,17	-0,87
RTN4	0,14	0,79	0,65
RTN4IP1	-0,52	0,69	1,22
RTN4R	0,43	4,80	4,36
RTN4RL2	-0,75	-0,53	0,21
RTTN	-1,35	-1,22	0,12
RUFY1	0,22	0,04	-0,18
RUFY3	1,33	-0,56	-1,88
RUNDC1	0,57	0,00	-0,57
RUNDC2A	0,16	-0,52	-0,68

RUNX1	0,39	0,11	-0,28
RUNX3	1,43	-0,63	-2,06
RUTBC1	0,37	0,07	-0,30
RUVBL2	-0,36	-0,60	-0,24
RWDD1	0,08	-1,97	-2,05
RWDD4A	-0,09	-0,11	-0,02
RXRB	-0,13	-0,61	-0,48
RYK	0,83	0,82	-0,01
RYR1	-0,32	0,05	0,37
RETSAT	-0,22	0,99	1,21
S100A10	-0,07	-0,62	-0,54
S100A11	-0,41	0,15	0,56
S100A12	-2,14	-15,63	-13,49
S100A13	-1,34	0,70	2,05
S100A16	-1,25	0,91	2,17
S100A3	-1,61	1,29	2,90
S100A4	-0,49	-0,75	-0,26
S100A5	1,11	1,29	0,19
S100P	-1,87	-2,48	-0,61
S100PBP	0,18	-0,31	-0,49
SAC	-0,03	-0,40	-0,37
SAC3D1	0,12	0,48	0,37
SACM1L	0,21	0,08	-0,13
SACS	1,44	1,10	-0,33
SAFB	0,42	-0,64	-1,05
SAFB2	0,74	-0,89	-1,64
SAG	-0,80	-2,54	-1,74
SAMD3	-0,06	-0,22	-0,17
SAMD4B	0,34	-0,17	-0,51
SAMD7	-0,30	-0,27	0,03
SAMD8	0,25	-0,33	-0,58
SAMHD1	-0,63	-1,12	-0,49
SAP130	-0,69	-0,09	0,59
SAP18	-0,19	-0,78	-0,59
SAP30BP	-0,01	-0,34	-0,34
SAP30L	0,34	-0,50	-0,85
SAR1B	-0,02	1,34	1,37
SARS	0,28	0,07	-0,21
SARS2	0,22	-0,22	-0,44
SAS10	0,17	0,25	0,08
SASS6	-0,35	-0,48	-0,13
SAT2	0,12	-1,01	-1,13
SATB1	0,14	-2,62	-2,76
SATL1	-0,36	0,21	0,58
SAV1	-0,85	1,61	2,46
SBDS	0,31	0,00	-0,31
SBF2	1,18	-0,61	-1,79
SCAMP1	-0,49	-0,22	0,27
SCAMP2	-0,36	0,20	0,56
SCAMP3	-0,49	0,85	1,34
SCAMP4	0,00	0,16	0,16
SCAND1	0,07	-0,63	-0,71
SCAND2	0,59	-0,34	-0,93
SCARB2	-0,69	0,33	1,02
SCCPDH	-1,44	0,24	1,68
SCD	-0,29	6,51	6,80
SCEL	0,34	4,12	3,78
SCFD2	-0,42	-0,48	-0,06
SCG5	-7,55	0,49	8,03

SCGB1C1	0,22	-0,57	-0,79
SCGB3A1	-1,78	-5,40	-3,62
SCGB3A2	0,37	-1,67	-2,04
SCGN	-1,23	-2,22	-1,00
SCIN	-1,57	3,58	5,15
SCLY	0,93	0,39	-0,54
SCMH1	-1,58	-0,97	0,60
SCML1	0,36	1,19	0,83
SCN11A	7,11	7,11	0,00
SCN1A	0,24	-0,54	-0,79
SCN7A	-0,45	-0,03	0,42
SCN9A	0,93	5,28	4,35
SCNM1	0,07	-0,71	-0,78
SCNN1B	2,45	0,06	-2,39
SCO1	-0,04	1,50	1,54
SCOC	-0,47	1,42	1,89
SCOTIN	-0,85	-2,16	-1,30
SCP2	-0,21	1,31	1,52
SCRG1	-0,07	-1,66	-1,59
SCRN3	-0,21	1,17	1,38
SCTR	4,50	-0,52	-5,02
SCYE1	0,82	-2,31	-3,13
SCYL2	0,22	0,44	0,22
SCYL3	0,26	-0,16	-0,42
SDAD1	0,10	0,25	0,15
SDC2	-0,53	3,76	4,29
SDC3	0,71	2,61	1,89
SDC4	-0,29	4,75	5,04
SDCBP	0,08	1,01	0,92
SDCBP2	1,05	1,14	0,09
SDCCAG10	-0,63	-0,82	-0,20
SDCCAG3	0,40	-0,76	-1,16
SDCCAG8	0,02	0,88	0,85
SDF2	0,29	0,30	0,01
SDF2L1	-0,66	0,00	0,66
SDHA	-0,35	0,45	0,80
SDHB	-0,38	0,30	0,69
SDHD	0,25	0,82	0,57
SDK2	0,91	0,03	-0,87
SDPR	1,96	2,32	0,36
SDSL	-1,32	2,11	3,43
SEC14L1	0,09	-0,14	-0,23
SEC23A	-0,13	0,15	0,29
SEC23IP	-0,24	-0,01	0,24
SEC24B	0,10	-0,76	-0,85
SEC24C	0,13	-0,01	-0,13
SEC24D	-0,33	0,04	0,37
SEC61A1	-0,08	0,10	0,18
SEC61B	0,22	0,35	0,13
SEC63	-0,31	-0,76	-0,45
SECISBP2	0,24	-1,00	-1,24
SEL1L	-0,31	0,25	0,56
SELI	-0,58	-0,32	0,27
SELM	-0,54	1,02	1,55
SEMA3C	1,68	2,18	0,50
SEMA3D	-0,25	-0,06	0,19
SEMA4A	0,38	-2,39	-2,77
SEMA4B	-0,68	-3,20	-2,52
SEMA4D	1,18	-1,82	-3,00

SEMA5B	0,32	0,51	0,19
SEMA6B	-0,46	0,46	0,92
SENP1	1,43	0,79	-0,64
SENP2	-0,11	-0,78	-0,67
SENP3	-0,06	-0,47	-0,41
SENP5	0,39	1,25	0,86
SENP7	0,61	-2,24	-2,85
SENP8	0,67	1,44	0,77
SEPHS2	-0,31	1,77	2,08
SEPN1	-1,02	0,44	1,46
SEPP1	-2,20	6,77	8,97
SERF2	0,04	0,96	0,92
SERINC1	0,15	0,25	0,10
SERINC4	0,21	-0,28	-0,49
SERINC5	0,76	0,05	-0,71
SERP1	0,42	-0,85	-1,27
SERPINA10	0,01	0,04	0,03
SERPINA12	0,19	0,41	0,22
SERPINA6	-0,13	-0,54	-0,41
SERPINB1	0,06	-0,99	-1,05
SERPINB2	0,51	-2,55	-3,06
SERPINB5	-0,86	-1,27	-0,41
SERPINB8	-0,34	0,04	0,38
SERPIND1	0,37	0,66	0,29
SERPINE1	0,31	7,93	7,62
SERPINF1	0,25	-1,09	-1,34
SERPINF2	2,51	1,76	-0,75
SERPING1	-2,28	-2,73	-0,45
SERPINI1	-1,93	3,85	5,78
SERTAD1	0,27	1,84	1,56
SERTAD3	1,04	-0,84	-1,87
SESN2	0,56	0,79	0,23
SESTD1	0,69	-2,03	-2,73
SET	0,24	-0,12	-0,36
SETBP1	6,59	-0,09	-6,68
SETD1A	0,61	-0,25	-0,86
SETD2	0,54	-1,27	-1,80
SETD3	-0,16	0,69	0,85
SETD5	-0,48	-0,60	-0,12
SETD6	0,19	-1,48	-1,67
SETDB2	0,32	1,73	1,41
SETMAR	-0,34	1,70	2,04
SF1	0,46	-1,93	-2,39
SF3A1	0,51	-0,67	-1,18
SF3A2	0,37	-1,01	-1,38
SF3A3	0,13	-0,72	-0,85
SF3B14	0,19	-0,10	-0,28
SF3B2	0,05	-0,18	-0,23
SF3B3	0,26	0,07	-0,19
SF3B4	0,37	-0,69	-1,06
SF4	0,28	-0,31	-0,59
SFI1	-0,59	-0,85	-0,26
SFMBT2	-0,21	-0,38	-0,17
SFRS1	0,13	-0,06	-0,19
SFRS11	0,30	-0,81	-1,11
SFRS12	0,17	-0,71	-0,88
SFRS14	0,24	-0,52	-0,76
SFRS15	0,71	0,32	-0,39
SFRS2	-0,18	-0,54	-0,36

SFRS3	0,04	-0,34	-0,38
SFRS6	-0,06	-1,38	-1,33
SFRS8	0,23	-0,96	-1,19
SFRS9	-0,02	-0,19	-0,17
SFT2D1	0,14	0,93	0,80
SFT2D3	0,46	0,09	-0,37
SFTPD	1,70	-7,67	-9,36
SFXN1	0,21	0,94	0,74
SFXN2	-2,80	1,98	4,78
SFXN4	-0,32	0,66	0,97
SFXN5	0,29	-0,81	-1,10
SGCA	0,36	-0,72	-1,08
SGCB	0,18	-0,03	-0,21
SGK	-0,50	0,87	1,37
SGOL2	-0,97	0,87	1,84
SGPL1	1,58	1,70	0,13
SGPP1	0,25	1,57	1,32
SGPP2	3,87	7,48	3,62
SGSH	0,59	-0,43	-1,01
SGTA	0,14	0,92	0,78
SGTB	-0,42	-0,93	-0,51
SH2D1A	0,14	-0,07	-0,21
SH2D3C	-0,42	-1,42	-1,00
SH2D4A	2,26	-0,11	-2,37
SH3BGR	-0,29	0,63	0,92
SH3BGRL	-0,13	0,05	0,18
SH3BGRL3	0,18	0,33	0,15
SH3BP5	-4,12	-1,63	2,49
SH3BP5L	-0,21	0,56	0,77
SH3GL1	0,29	-0,08	-0,38
SH3PX3	0,65	0,80	0,15
SH3PXD2B	-1,70	1,00	2,70
SH3RF2	-0,24	-0,61	-0,37
SH3TC1	0,14	-1,70	-1,84
SH3YL1	0,17	0,72	0,54
SHARPIN	-0,07	-0,09	-0,03
SHB	0,92	4,44	3,51
SHC1	0,14	0,07	-0,07
SHFM1	-0,38	0,50	0,88
SHMT1	-1,01	-1,34	-0,33
SHMT2	0,67	0,73	0,06
SHOX2	-0,72	-0,10	0,62
SHQ1	-0,35	-0,43	-0,08
SIAE	0,03	0,60	0,58
SIAH1	0,41	0,63	0,22
SIAH2	0,34	-1,40	-1,75
SIGLEC1	-1,31	-0,39	0,92
SIGLEC12	2,99	3,38	0,40
SIGLEC5	0,74	-0,02	-0,77
SIGLEC7	-0,26	0,27	0,53
SIGLEC9	0,06	0,71	0,64
SIL1	-0,87	0,79	1,66
SIN3A	0,25	-0,42	-0,67
SIN3B	0,84	-1,35	-2,19
SIP1	-0,17	-0,19	-0,03
SIPA1L1	0,04	-2,12	-2,16
SIPA1L2	-0,06	0,72	0,78
SIRPA	0,21	0,94	0,73
SIRPB1	-0,98	-2,81	-1,82

SIRPD	-1,04	-1,61	-0,58
SIRT1	1,18	-0,32	-1,50
SIRT2	0,30	-0,52	-0,82
SIRT5	-0,11	0,36	0,47
SIRT6	0,34	1,14	0,80
SIRT7	0,34	-0,53	-0,86
SIX5	1,12	1,65	0,53
SKIL	-0,64	-0,15	0,49
SKIP	0,49	-0,51	-1,00
SKIV2L	0,34	0,44	0,10
SKIV2L2	-0,06	0,35	0,42
SKP2	-0,03	-1,49	-1,47
SLA	2,21	1,94	-0,27
SLAMF1	2,67	5,19	2,52
SLAMF6	-1,82	0,80	2,63
SLAMF7	0,45	4,43	3,98
SLAMF8	-0,42	5,35	5,77
SLAMF9	-1,23	4,11	5,34
SLC10A2	-4,54	-8,04	-3,50
SLC10A3	0,34	0,32	-0,02
SLC11A1	-3,76	-4,71	-0,95
SLC11A2	1,04	2,72	1,68
SLC12A6	-0,66	-0,16	0,51
SLC12A7	0,31	-0,14	-0,45
SLC13A1	0,34	0,25	-0,09
SLC13A3	0,55	-0,30	-0,84
SLC13A4	0,06	-0,01	-0,07
SLC14A1	-0,62	-0,25	0,36
SLC15A4	-0,05	-0,39	-0,34
SLC16A5	-0,02	-1,70	-1,68
SLC16A7	-0,30	-1,60	-1,30
SLC16A9	6,03	8,78	2,75
SLC17A4	0,26	0,54	0,27
SLC18A1	0,58	-1,12	-1,71
SLC1A3	-1,86	3,88	5,74
SLC1A4	-2,02	0,43	2,45
SLC1A5	-0,26	0,36	0,62
SLC1A7	-3,53	-0,73	2,80
SLC20A1	-0,18	1,22	1,40
SLC20A2	-0,05	0,19	0,23
SLC22A1	0,21	2,26	2,04
SLC22A15	-1,38	-2,64	-1,26
SLC22A16	-1,44	-0,61	0,84
SLC22A18	-0,19	0,03	0,21
SLC22A18AS	0,34	1,31	0,96
SLC22A5	-0,38	0,33	0,71
SLC22A7	0,29	0,84	0,55
SLC23A1	-0,12	0,40	0,51
SLC23A2	-0,52	-0,20	0,32
SLC23A3	1,46	4,56	3,10
SLC24A1	-0,24	0,58	0,82
SLC24A2	-0,58	-0,79	-0,21
SLC24A6	-0,35	0,86	1,20
SLC25A1	0,31	1,29	0,98
SLC25A10	-0,57	0,32	0,88
SLC25A11	0,12	0,53	0,41
SLC25A13	-0,02	0,57	0,59
SLC25A14	-0,58	-1,06	-0,47
SLC25A15	0,45	0,51	0,06

SLC25A17	0,07	0,38	0,31
SLC25A22	0,14	-0,59	-0,73
SLC25A24	-0,16	0,38	0,54
SLC25A25	1,42	1,55	0,13
SLC25A26	-0,38	-0,08	0,30
SLC25A28	-0,26	-1,53	-1,27
SLC25A29	0,81	0,94	0,13
SLC25A31	-0,43	0,39	0,82
SLC25A32	0,01	-0,58	-0,59
SLC25A34	0,16	0,08	-0,07
SLC25A35	0,05	1,43	1,37
SLC25A37	-0,77	-3,51	-2,75
SLC25A5	-0,11	0,36	0,47
SLC26A1	0,02	-0,02	-0,04
SLC26A8	-0,22	-0,97	-0,75
SLC27A4	-3,12	-3,22	-0,09
SLC27A5	0,55	1,24	0,69
SLC28A3	-3,33	3,31	6,64
SLC29A1	-0,83	2,08	2,91
SLC29A2	-0,25	1,72	1,97
SLC29A3	-1,02	2,90	3,92
SLC2A3	0,60	-0,22	-0,82
SLC2A5	-2,87	1,24	4,11
SLC2A9	-1,21	-0,47	0,74
SLC30A5	-0,32	1,20	1,51
SLC30A6	0,39	1,45	1,05
SLC30A7	0,10	-0,26	-0,36
SLC31A1	-0,97	0,66	1,63
SLC31A2	-0,80	-0,90	-0,10
SLC33A1	0,03	0,87	0,84
SLC35A1	-0,30	-1,46	-1,16
SLC35A2	-0,11	0,96	1,06
SLC35A4	-0,18	-0,23	-0,05
SLC35A5	0,51	0,21	-0,30
SLC35B1	-0,60	-0,26	0,34
SLC35B2	1,16	0,16	-1,01
SLC35B3	0,01	0,07	0,06
SLC35B4	-0,65	-0,26	0,39
SLC35C2	-0,28	-0,92	-0,64
SLC35D2	0,39	-0,25	-0,64
SLC35E1	0,34	-0,05	-0,40
SLC36A1	-0,70	0,13	0,82
SLC36A4	-0,38	-0,71	-0,34
SLC37A1	0,24	0,31	0,08
SLC37A4	-0,23	2,35	2,58
SLC38A2	-0,45	-0,49	-0,04
SLC38A5	0,10	-0,11	-0,21
SLC38A6	0,91	5,17	4,26
SLC39A1	-0,15	0,88	1,03
SLC39A12	1,93	7,87	5,95
SLC39A3	-0,04	0,78	0,81
SLC39A4	-0,23	0,17	0,41
SLC39A5	0,13	0,50	0,37
SLC39A6	1,13	1,55	0,42
SLC39A7	-0,27	0,26	0,53
SLC39A9	0,37	0,99	0,61
SLC3A2	0,02	1,31	1,30
SLC40A1	0,38	-3,26	-3,63
SLC41A2	0,26	7,14	6,87

SLC43A1	-2,39	-2,43	-0,04
SLC43A3	-1,08	-0,36	0,71
SLC44A1	0,29	0,67	0,39
SLC45A2	-0,45	-2,97	-2,52
SLC4A1AP	-0,12	0,09	0,21
SLC4A8	-0,76	-0,33	0,43
SLC5A11	1,58	0,78	-0,80
SLC5A2	0,51	-0,57	-1,09
SLC5A3	1,17	3,35	2,17
SLC5A6	-0,23	0,70	0,93
SLC5A8	0,26	-1,79	-2,05
SLC6A13	-0,17	-0,77	-0,60
SLC6A16	-5,51	-3,45	2,06
SLC6A19	-0,36	0,81	1,17
SLC6A9	0,18	1,05	0,87
SLC7A11	-0,21	4,36	4,57
SLC7A13	-3,76	-2,18	1,58
SLC7A5	1,15	3,32	2,16
SLC7A6OS	0,12	0,15	0,03
SLC7A7	-0,94	-1,10	-0,17
SLC7A8	0,92	5,81	4,89
SLC8A1	-1,50	-0,45	1,05
SLC9A6	-0,26	0,38	0,64
SLC9A7	-0,31	2,98	3,29
SLCO1B1	-0,54	-0,46	0,08
SLCO2B1	-0,51	10,57	11,08
SLCO3A1	0,71	-1,20	-1,90
SLITRK2	0,66	0,99	0,33
SLTM	0,01	-1,06	-1,07
SLU7	-0,07	-0,84	-0,78
SLURP1	0,12	0,66	0,54
SMA4	-0,52	-0,11	0,42
SMA5	-0,13	-1,33	-1,21
SMAD1	0,70	0,71	0,01
SMAD2	-0,11	-0,63	-0,52
SMAD3	1,16	-4,04	-5,19
SMAD4	0,19	-0,19	-0,38
SMAD7	0,25	0,00	-0,24
SMAP1L	0,15	-2,12	-2,27
SMARCA4	0,19	-0,23	-0,42
SMARCA5	0,22	0,38	0,16
SMARCAD1	-0,34	-0,04	0,30
SMARCAL1	-0,19	-0,31	-0,12
SMARCC1	0,01	-0,75	-0,76
SMARCC2	0,52	-0,21	-0,73
SMARCD1	0,39	-0,45	-0,84
SMARCD2	0,48	-0,08	-0,56
SMCP	-0,15	-0,08	0,06
SMEK2	0,17	0,25	0,07
SMG5	0,30	0,00	-0,30
SMOX	0,86	1,84	0,98
SMPD1	-0,08	1,28	1,37
SMS	-0,20	0,20	0,40
SMTN	0,68	-0,48	-1,15
SMUG1	-0,09	0,30	0,40
SMYD2	-0,58	-0,49	0,09
SMYD3	-1,01	-0,29	0,73
SNAG1	-0,08	-0,43	-0,35
SNAP29	-0,14	-0,06	0,08

SNAPC1	1,81	-0,03	-1,84
SNAPC4	1,26	-0,32	-1,58
SNAPC5	-0,18	0,80	0,98
SNCA	-1,94	-0,71	1,23
SND1	0,00	0,23	0,23
SNIP	0,91	0,81	-0,11
SNIP1	0,09	-0,57	-0,66
SNN	-0,73	-1,19	-0,46
SNRK	0,00	-1,52	-1,52
SNRPA	-0,01	-0,70	-0,69
SNRPA1	-0,41	-0,57	-0,17
SNRPB	-0,14	-0,04	0,10
SNRPC	-0,19	0,32	0,51
SNRPD2	-0,41	-0,91	-0,50
SNRPD3	0,27	-0,25	-0,52
SNRPE	0,14	-0,68	-0,82
SNRPG	0,17	0,46	0,29
SNRPN	0,14	1,21	1,06
SNTB1	-3,23	-2,05	1,17
SNW1	0,02	-0,36	-0,39
SNX1	0,06	-1,01	-1,07
SNX12	0,29	1,08	0,79
SNX13	0,13	0,46	0,33
SNX14	0,15	0,47	0,32
SNX16	0,44	-0,50	-0,94
SNX17	-0,03	-0,51	-0,48
SNX19	-0,15	0,34	0,49
SNX2	-0,26	0,02	0,27
SNX24	-0,55	2,46	3,01
SNX25	0,83	1,82	1,00
SNX26	0,12	-0,61	-0,73
SNX5	0,14	0,79	0,65
SOAT1	0,40	2,35	1,95
SOCS2	0,57	4,59	4,02
SOCS4	0,03	0,18	0,15
SOCS6	0,70	1,06	0,37
SOCS7	0,66	0,28	-0,38
SOD2	-1,04	-1,42	-0,38
SOLH	0,80	-1,48	-2,28
SON	-0,09	-1,01	-0,92
SORBS1	-5,44	1,37	6,81
SORL1	-1,99	-5,28	-3,29
SORT1	-0,88	0,51	1,40
SOS1	-0,24	-0,26	-0,02
SOS2	0,34	-0,82	-1,17
SOST	-0,02	0,06	0,07
SOX15	0,88	-2,33	-3,21
SOX18	-0,06	0,15	0,21
SOX2	-0,45	-0,09	0,36
SOX30	0,00	0,42	0,42
SOX5	-0,31	-0,77	-0,46
SP1	0,18	-1,17	-1,35
SP100	0,05	-1,89	-1,94
SP110	-0,59	-2,36	-1,77
SP140	-1,71	-1,62	0,09
SPA17	-1,10	-0,12	0,98
SPACA4	0,60	0,57	-0,03
SPAG16	0,04	2,81	2,77
SPAG7	-0,11	-0,19	-0,08

SPAG9	0,01	0,83	0,82
SPARC	-7,42	5,91	13,33
SPATA1	1,65	0,32	-1,33
SPATA13	-1,85	-2,22	-0,37
SPATA2	-0,35	-1,21	-0,85
SPATA3	0,38	0,02	-0,36
SPATA5	-1,30	0,29	1,59
SPATA5L1	0,34	-0,15	-0,49
SPATA6	-4,70	-7,18	-2,48
SPATA9	-0,42	-0,39	0,03
SPCS1	0,00	0,18	0,18
SPCS2	-0,37	0,33	0,70
SPCS3	-0,32	0,15	0,47
SPDEF	0,09	0,17	0,08
SPECC1	-0,57	-0,34	0,24
SPG21	-0,42	0,22	0,64
SPG7	0,06	-0,85	-0,91
SPHAR	0,13	-0,61	-0,73
SPHK2	0,08	-0,12	-0,20
SPI1	0,45	0,01	-0,44
SPIC	-0,24	-0,42	-0,18
SPIN3	0,51	-0,62	-1,13
SPINK1	-2,32	4,46	6,78
SPINT2	2,88	2,74	-0,14
SPIRE1	-0,03	2,51	2,54
SPIRE2	-0,56	1,55	2,11
SPN	0,54	1,07	0,54
SPO11	-1,02	-0,22	0,80
SPOCD1	-1,03	6,30	7,33
SPOCK1	-0,19	-1,81	-1,62
SPOCK2	-0,56	-0,61	-0,05
SPP1	-0,45	9,98	10,43
SPPL2A	-0,30	0,92	1,22
SPPL2B	0,56	-1,69	-2,26
SPRED1	0,08	2,99	2,91
SPRR1A	-0,27	-0,22	0,05
SPRY2	0,16	6,36	6,20
SPRYD3	0,06	0,43	0,37
SPRYD4	-0,26	0,73	0,99
SPRYD5	0,06	0,38	0,32
SPSB1	0,03	4,87	4,84
SPSB2	0,04	-0,46	-0,50
SPSB3	0,67	-1,76	-2,42
SPTAN1	0,43	2,59	2,16
SPTB	0,10	0,62	0,52
SPTBN5	3,19	8,01	4,82
SPTLC1	-0,28	0,09	0,37
SPTLC2	-0,92	-2,79	-1,86
SPTY2D1	-0,37	0,16	0,53
SQLE	-0,47	3,51	3,98
SQRDL	-0,47	0,82	1,29
SQSTM1	-0,60	2,41	3,00
SR-A1	-0,28	0,01	0,29
SRBD1	-0,38	-0,68	-0,30
SRC	-0,65	0,37	1,02
SRCRB4D	-0,82	0,58	1,40
SRD5A1	0,14	-1,18	-1,31
SRD5A2L	-0,12	1,32	1,44
SRF	0,71	0,18	-0,53

SRI	0,04	0,08	0,04
SRP19	0,26	0,30	0,05
SRP9	-0,07	0,46	0,53
SRPK2	0,99	1,30	0,31
SRPR	-0,29	-0,27	0,02
SRR	0,31	1,53	1,22
SS18	0,03	-0,73	-0,76
SS18L1	0,50	0,14	-0,36
SS18L2	0,18	0,13	-0,06
SSBP1	-0,08	0,01	0,09
SSBP2	0,35	-2,22	-2,57
SSFA2	-0,16	-0,73	-0,57
SSH2	-0,13	-2,51	-2,39
SSNA1	0,11	-0,57	-0,67
SSR1	-0,25	-0,65	-0,40
SSR2	-0,52	-0,32	0,21
SSR4	-0,39	-0,37	0,01
SSSCA1	-0,12	-0,22	-0,10
SST	-0,26	-0,36	-0,10
SSTR2	-0,20	-0,19	0,01
SSU72	-0,19	0,13	0,33
SSX8	0,68	0,11	-0,56
ST13	0,14	1,32	1,18
ST14	0,49	2,31	1,82
ST18	0,04	8,00	7,96
ST3GAL1	-0,01	0,37	0,38
ST3GAL3	-0,19	0,00	0,19
ST3GAL4	0,11	0,90	0,80
ST3GAL6	-0,97	0,41	1,38
ST5	-0,40	1,74	2,14
ST6GAL1	1,50	0,92	-0,57
ST6GALNAC4	-0,88	0,95	1,83
ST7	0,65	3,28	2,63
ST7L	-0,07	0,99	1,05
STAB2	-0,03	0,47	0,50
STAC3	-0,83	-0,87	-0,04
STAG1	-0,07	0,04	0,11
STAG3	1,86	5,39	3,53
STAM	-0,31	0,68	0,99
STAMBP	0,04	0,45	0,41
STAMBPL1	2,60	3,87	1,27
STAP2	0,28	0,33	0,05
STARD13	-0,08	0,44	0,52
STARD3	0,97	0,66	-0,31
STARD4	-0,61	6,15	6,76
STARD5	-0,48	1,18	1,66
STARD8	-0,46	1,04	1,50
STAT1	-0,50	-1,00	-0,50
STAT2	-0,31	-1,16	-0,85
STAT3	-0,06	-0,43	-0,38
STAT4	-1,70	3,45	5,15
STAT5A	0,59	-0,02	-0,61
STAT5B	0,41	-1,40	-1,81
STAT6	0,53	-0,52	-1,05
STCH	0,29	-0,26	-0,55
STH	0,08	-0,81	-0,88
STIM1	-0,31	-0,96	-0,65
STIM2	0,65	0,35	-0,30
STIP1	0,10	1,93	1,83

STK11IP	0,09	-0,70	-0,80
STK16	0,30	0,04	-0,26
STK19	0,60	-0,08	-0,68
STK24	0,32	-0,09	-0,42
STK32B	0,26	0,26	0,00
STK35	-0,18	0,50	0,67
STK36	-0,75	-1,16	-0,41
STK38	-0,05	-0,67	-0,62
STK40	-0,45	-0,74	-0,30
STMN1	-1,49	1,57	3,06
STOML2	-0,16	0,10	0,26
STRAP	-0,06	-0,14	-0,08
STRN	-0,13	0,12	0,25
STRN3	-0,56	-0,45	0,11
STRN4	0,26	-1,21	-1,47
STS	-1,25	-1,87	-0,62
STS-1	-0,54	1,21	1,75
STT3A	0,34	1,14	0,80
STT3B	-0,33	0,13	0,46
STX10	-0,13	-1,22	-1,09
STX12	0,16	-0,27	-0,43
STX16	-0,19	-1,06	-0,87
STX6	-0,68	-1,01	-0,33
STX7	0,03	0,39	0,36
STX8	-0,24	-0,42	-0,18
STXBP2	0,13	-1,10	-1,23
STXBP3	-0,20	-0,48	-0,28
STYX	-0,42	-0,47	-0,04
STYXL1	-0,22	0,11	0,33
SUCLA2	0,01	1,00	1,00
SUCLG1	-0,23	0,10	0,33
SUCNR1	0,52	14,06	13,54
SUDS3	0,24	-0,05	-0,29
SUFU	0,74	0,98	0,24
SUGT1	0,05	-0,05	-0,10
SUHW4	-0,36	-1,80	-1,44
SULF2	4,04	0,05	-3,99
SULT1A3	-0,02	-2,18	-2,16
SULT1A4	0,02	-0,98	-1,01
SUMF2	0,10	-0,03	-0,13
SUMO2	0,28	-0,31	-0,59
SUOX	1,31	2,15	0,84
SUPT16H	0,18	-0,29	-0,47
SUPT3H	-0,08	-0,15	-0,08
SUPT4H1	-0,07	-0,58	-0,52
SUPT6H	0,16	-0,04	-0,20
SUPT7L	1,98	-0,60	-2,59
SUPV3L1	-0,22	-0,05	0,16
SURF1	0,01	0,01	0,00
SURF2	0,30	-0,16	-0,46
SURF4	-0,46	0,31	0,77
SURF5	0,52	0,20	-0,32
SURF6	0,69	-0,08	-0,77
SUSD2	-0,35	1,11	1,46
SUV39H1	-0,12	-0,18	-0,06
SUV39H2	0,55	0,41	-0,14
SVIL	-1,03	-1,06	-0,03
SYCP2	2,27	-2,99	-5,26
SYCP3	-1,35	-0,25	1,10

SYF2	0,18	-0,86	-1,04
SYK	0,26	-0,66	-0,91
SYMPK	0,66	0,19	-0,46
SYN1	-0,19	-0,04	0,15
SYNC1	0,17	4,78	4,61
SYNGR1	-2,72	-1,48	1,23
SYNJ1	0,81	0,54	-0,28
SYNPO2	0,15	0,39	0,23
SYP	-2,58	0,80	3,38
SYPL1	-0,71	-0,58	0,12
SYT11	-1,08	-1,89	-0,82
SYT17	4,56	5,05	0,49
SYT8	-0,14	0,16	0,30
SYTL3	-2,50	-2,56	-0,06
TA-NFKBH	0,18	-0,56	-0,73
TAAR2	-0,20	-0,16	0,04
TAAR6	-0,52	-0,57	-0,05
TAC4	-0,13	0,06	0,19
TACC3	-0,02	-2,02	-2,00
TADA1L	0,70	-0,37	-1,07
TADA2L	0,31	-0,24	-0,56
TADA3L	0,17	0,01	-0,16
TAF1	1,72	-1,72	-3,44
TAF10	-0,03	-0,46	-0,43
TAF12	0,03	-0,01	-0,04
TAF13	-0,28	0,77	1,04
TAF15	-0,01	-0,99	-0,98
TAF1C	0,43	-1,63	-2,06
TAF1L	0,59	-1,52	-2,11
TAF2	0,08	-0,21	-0,29
TAF5L	0,05	0,01	-0,03
TAF6	0,37	-0,03	-0,40
TAF6L	0,27	-0,48	-0,76
TAF9	0,01	0,49	0,49
TAGAP	-0,69	-2,13	-1,45
TAGLN2	-0,90	-1,59	-0,70
TAL2	0,10	-0,06	-0,16
TANK	0,03	0,24	0,21
TAOK2	0,35	0,28	-0,07
TAP1	-0,31	-0,39	-0,08
TAP2	-0,65	-1,30	-0,65
TARP	-2,26	-2,03	0,23
TARS	0,09	0,98	0,88
TAS1R2	-0,59	-0,56	0,03
TAS2R1	0,07	-0,81	-0,89
TAS2R10	0,68	1,00	0,32
TAS2R39	-0,40	0,45	0,85
TAS2R4	-0,14	-0,52	-0,39
TAS2R5	0,79	-0,14	-0,93
TAS2R60	0,42	0,19	-0,23
TASP1	-0,50	0,25	0,76
TATDN1	-0,49	-0,20	0,29
TATDN3	0,02	-0,09	-0,11
TAX1BP1	0,17	0,25	0,08
TAX1BP3	0,57	1,59	1,02
TAZ	0,24	-0,43	-0,67
TBC1D10A	0,69	-0,85	-1,54
TBC1D14	0,05	-0,75	-0,81
TBC1D15	-0,14	-0,07	0,07

TBC1D17	0,31	0,24	-0,07
TBC1D19	-0,20	0,89	1,09
TBC1D22A	0,73	0,82	0,10
TBC1D22B	-0,10	0,48	0,59
TBC1D3	0,54	-0,93	-1,47
TBC1D3C	0,49	-0,71	-1,20
TBC1D4	0,42	1,04	0,62
TBC1D5	-0,08	-0,08	-0,01
TBC1D7	-0,44	1,24	1,68
TBC1D8	1,06	-0,87	-1,93
TBCCD1	0,41	0,50	0,09
TBCE	-0,35	-0,09	0,26
TBK1	0,01	-0,28	-0,29
TBKBP1	0,37	0,10	-0,27
TBL2	-0,62	-0,03	0,59
TBL3	0,10	-0,60	-0,70
TBN	0,37	-0,06	-0,43
TBP	0,44	-0,20	-0,65
TBPL1	-0,01	-0,88	-0,87
TBRG1	-0,34	-0,61	-0,27
TBRG4	0,25	0,33	0,09
TBX10	-1,86	-1,18	0,68
TBX19	0,78	0,13	-0,65
TBX22	-0,54	0,19	0,73
TBX5	3,61	0,68	-2,93
TBXAS1	-0,36	-1,18	-0,82
TCEA2	0,31	-0,61	-0,92
TCEAL1	-0,19	1,03	1,22
TCEAL3	-0,30	1,26	1,56
TCEAL4	0,02	1,18	1,15
TCEAL8	-0,15	1,09	1,25
TCF12	0,26	0,49	0,22
TCF19	-2,97	-1,10	1,87
TCF20	-0,39	-0,78	-0,39
TCF23	0,19	0,17	-0,02
TCF3	1,21	-0,66	-1,87
TCF7	0,28	-0,10	-0,38
TCF7L2	-4,37	-5,26	-0,89
TCIRG1	-0,53	-0,06	0,47
TCL1B	-0,45	-0,52	-0,07
TCL6	-1,96	0,42	2,37
TCP1	0,13	-0,02	-0,16
TCP10L	-0,17	2,03	2,20
TCTA	0,37	0,26	-0,11
TDG	0,36	0,57	0,22
TDO2	-0,47	-1,08	-0,61
TDP1	0,09	-0,48	-0,57
TDRD1	0,11	-1,45	-1,56
TDRD6	-0,54	0,61	1,15
TDRD7	0,32	-0,08	-0,40
TDRD9	-2,05	-1,20	0,84
TEAD1	-0,13	0,49	0,62
TEAD2	-2,22	2,77	4,99
TEAD3	-1,28	0,35	1,62
TEC	-0,25	-0,34	-0,09
TEDDM1	0,27	0,23	-0,04
TEP1	0,25	-0,77	-1,02
TERF1	0,08	0,45	0,37
TERF2IP	-0,01	-0,14	-0,13

TES	0,63	-1,86	-2,49
TESC	-2,07	-5,66	-3,58
TESSP2	0,09	-0,34	-0,43
TESSP5	-0,42	-0,80	-0,38
TEX10	0,26	0,74	0,48
TEX13A	-0,63	-0,46	0,17
TEX2	-1,06	1,05	2,11
TEX264	-0,17	0,57	0,74
TFB1M	0,16	0,79	0,64
TFB2M	-0,35	0,23	0,58
TFDP1	-0,41	-0,54	-0,13
TFE3	0,00	-0,58	-0,58
TFF3	0,47	2,04	1,57
TFG	0,00	0,65	0,65
TFIP11	-0,06	-0,31	-0,25
TFPI	-1,10	7,67	8,77
TFPT	-0,11	1,65	1,76
TFR2	0,31	0,38	0,07
TGDS	0,42	0,85	0,43
TGFA	2,32	3,40	1,08
TGFBI	0,75	-0,14	-0,89
TGFBR1	-1,11	-0,05	1,07
TGFBR2	-0,04	0,05	0,09
TGM2	1,38	8,06	6,69
TGM3	-3,89	-2,96	0,93
TGM6	-0,37	-0,76	-0,39
TGM7	-0,10	0,14	0,24
TH	-0,59	-2,13	-1,54
THADA	-0,88	-0,66	0,22
THAP1	0,20	0,25	0,05
THAP10	-2,23	2,12	4,35
THAP11	0,31	-0,96	-1,28
THAP5	-0,64	-1,57	-0,93
THAP6	0,60	2,35	1,75
THAP7	0,42	0,31	-0,11
THAP8	-0,91	3,06	3,97
THBS3	1,60	1,25	-0,35
THEM2	-0,45	2,49	2,94
THEM4	-2,41	5,62	8,03
THEX1	2,74	3,54	0,80
THNSL1	0,20	2,19	1,99
THOC2	0,09	-0,49	-0,57
THOC4	0,13	-0,15	-0,28
THOC5	-0,87	-0,56	0,31
THOC6	0,67	-0,51	-1,19
THOC7	-0,07	-0,03	0,04
THOP1	0,21	0,84	0,62
THTPA	0,03	-1,11	-1,14
THUMPD1	0,11	-0,27	-0,38
THYN1	-0,11	-0,52	-0,41
TIAL1	-0,01	-0,58	-0,57
TIAM2	0,97	3,21	2,24
TICAM2	0,15	-1,17	-1,32
TIE1	-2,57	0,49	3,07
TIGA1	0,29	-1,71	-2,00
TIGD1	0,43	-0,67	-1,09
TIGD3	-0,47	-0,43	0,04
TIGD4	0,85	0,05	-0,80
TIGD5	0,57	-0,04	-0,62

TIGD6	-0,01	3,88	3,89
TIGD7	0,16	0,18	0,02
TIMM10	-0,58	1,52	2,10
TIMM17A	0,04	2,41	2,37
TIMM17B	0,09	-0,66	-0,75
TIMM22	0,19	-0,39	-0,58
TIMM23	-0,19	0,45	0,64
TIMM8A	-0,18	0,99	1,17
TIMM8B	0,08	0,65	0,57
TIMM9	-0,01	-0,63	-0,62
TIMP1	-1,10	-0,72	0,38
TIMP2	0,06	0,91	0,85
TIMP4	-1,94	0,68	2,61
TINAG	0,06	-0,22	-0,28
TINF2	-0,41	-0,45	-0,04
TINP1	-0,16	-0,85	-0,69
TJAP1	0,26	-0,49	-0,75
TJP1	0,56	1,04	0,48
TJP2	1,07	0,66	-0,41
TJP3	-0,02	4,00	4,02
TK1	-3,52	0,21	3,73
TK2	0,33	-0,10	-0,44
TKT	0,17	-0,76	-0,93
TLE6	-0,57	5,06	5,63
TLK1	0,44	0,80	0,35
TLN1	0,09	-0,36	-0,45
TLN2	0,72	6,62	5,90
TLR10	-0,24	-1,75	-1,50
TLR2	-1,15	-4,93	-3,78
TLR4	-0,42	-0,27	0,15
TLR6	-1,28	-1,17	0,10
TLR7	-3,48	-4,60	-1,12
TLR9	-0,18	-0,16	0,02
TM2D2	0,83	2,97	2,14
TM4SF1	-3,63	8,96	12,59
TM4SF19	-2,44	6,82	9,26
TM7SF2	0,43	2,26	1,83
TM7SF4	-1,24	15,22	16,46
TM9SF1	-0,12	0,64	0,76
TM9SF2	-0,07	0,11	0,19
TM9SF4	-0,08	-1,38	-1,30
TMBIM4	-0,11	-0,04	0,07
TMC4	-0,84	0,05	0,89
TMCC1	-0,21	0,04	0,25
TMCC2	4,44	5,46	1,02
TMCO1	-0,21	0,01	0,22
TMCO3	-0,14	1,55	1,69
TMCO5	-0,91	-0,54	0,37
TMED1	-0,21	-0,18	0,04
TMED10	-0,25	0,03	0,28
TMED4	-0,27	0,31	0,57
TMED5	0,20	0,50	0,30
TMED7	-0,30	-0,34	-0,04
TMED8	-0,42	-0,23	0,19
TMEFF2	-0,27	-0,38	-0,11
TMEM1	0,56	0,33	-0,23
TMEM101	-0,19	0,21	0,41
TMEM102	-2,90	-2,90	0,00
TMEM103	-0,31	-0,20	0,12

TMEM104	-1,09	1,14	2,23
TMEM11	-0,17	-0,67	-0,50
TMEM110	-0,87	0,36	1,23
TMEM111	0,49	1,45	0,96
TMEM115	-0,06	0,38	0,43
TMEM116	-0,22	0,10	0,32
TMEM118	-1,19	8,91	10,10
TMEM123	0,94	0,19	-0,75
TMEM126A	-0,18	0,96	1,14
TMEM126B	-0,41	0,51	0,92
TMEM127	0,42	0,59	0,18
TMEM128	-0,78	0,95	1,74
TMEM129	-0,01	-0,03	-0,02
TMEM134	0,04	0,40	0,35
TMEM137	0,77	-2,04	-2,81
TMEM138	-0,92	1,41	2,33
TMEM140	-0,63	3,52	4,15
TMEM141	-0,39	0,21	0,61
TMEM142A	0,37	0,21	-0,16
TMEM143	-0,14	0,67	0,80
TMEM144	0,71	1,51	0,80
TMEM147	-0,43	0,40	0,83
TMEM149	1,44	0,89	-0,55
TMEM14A	0,14	9,38	9,23
TMEM14B	-0,49	-0,16	0,32
TMEM16B	-0,90	-1,01	-0,11
TMEM16F	-0,04	-0,37	-0,33
TMEM18	0,34	-1,43	-1,77
TMEM19	0,18	1,05	0,87
TMEM24	0,09	0,29	0,20
TMEM26	1,91	2,66	0,75
TMEM29	-0,44	-1,22	-0,79
TMEM30A	-0,04	-0,57	-0,52
TMEM33	0,10	0,86	0,76
TMEM38A	-0,55	-1,98	-1,43
TMEM39A	0,13	0,39	0,26
TMEM4	-1,09	-0,60	0,49
TMEM41A	0,18	0,57	0,39
TMEM41B	0,15	1,36	1,21
TMEM43	0,04	-0,28	-0,32
TMEM44	-0,02	7,25	7,27
TMEM45A	-2,07	-0,52	1,55
TMEM48	-0,91	0,46	1,38
TMEM50B	0,12	-1,89	-2,02
TMEM51	0,58	3,22	2,64
TMEM53	-0,47	2,68	3,15
TMEM55A	0,78	-0,79	-1,57
TMEM55B	0,49	0,60	0,11
TMEM59	-0,07	0,33	0,40
TMEM60	0,19	1,57	1,38
TMEM62	-0,30	0,08	0,38
TMEM63B	0,26	0,63	0,37
TMEM65	-0,27	-0,66	-0,39
TMEM68	0,30	0,92	0,61
TMEM69	-0,29	-0,06	0,22
TMEM70	-0,12	-0,31	-0,19
TMEM71	0,99	-2,16	-3,15
TMEM77	-0,06	-0,12	-0,06
TMEM79	0,18	-0,20	-0,37

TMEM80	-0,07	-1,70	-1,63
TMEM81	0,34	-0,27	-0,60
TMEM85	-0,33	0,03	0,35
TMEM86A	0,54	1,77	1,24
TMEM86B	-0,10	-0,40	-0,30
TMEM87A	-0,05	-0,07	-0,02
TMEM87B	-0,50	0,16	0,66
TMEM88	-0,38	-1,62	-1,24
TMEM9	-0,31	-0,53	-0,23
TMEM93	-0,02	0,02	0,04
TMEM99	0,09	1,18	1,09
TMEM9B	0,12	0,16	0,04
TMF1	-0,04	-0,09	-0,06
TMLHE	0,21	0,04	-0,17
TMOD3	0,53	0,06	-0,47
TMOD4	0,66	-1,30	-1,96
TMPO	-0,87	-1,38	-0,52
TMPRSS4	-0,40	1,25	1,64
TMPRSS9	0,05	0,02	-0,04
TMSB10	0,03	0,18	0,15
TMTC1	0,88	0,32	-0,56
TMTC4	0,66	-0,14	-0,80
TNC	4,24	8,34	4,10
TNFAIP1	0,26	1,22	0,96
TNFAIP6	-2,55	-0,31	2,24
TNFAIP8L3	-0,28	10,53	10,81
TNFRSF10A	0,21	0,48	0,27
TNFRSF11B	1,21	1,42	0,21
TNFRSF12A	-0,37	2,46	2,82
TNFRSF13B	3,13	-1,03	-4,16
TNFRSF1A	0,06	-0,93	-0,99
TNFRSF1B	-0,43	-1,93	-1,49
TNFRSF25	1,81	0,24	-1,57
TNFRSF9	0,57	-1,43	-2,00
TNFSF12	-0,22	-0,23	-0,01
TNFSF12-TNFS	-0,38	-0,24	0,14
TNFSF13	-1,13	-1,00	0,13
TNFSF13B	0,66	-0,37	-1,03
TNFSF15	-0,05	0,26	0,30
TNFSF4	0,02	1,31	1,28
TNFSF5IP1	-0,03	-0,52	-0,49
TNFSF8	-0,87	-1,85	-0,98
TNIP1	1,24	0,03	-1,21
TNIP2	0,08	0,14	0,06
TNIP3	-6,88	-0,50	6,38
TNKS	0,96	0,61	-0,36
TNKS1BP1	-0,59	3,03	3,61
TNN	1,23	0,32	-0,91
TNNI3K	0,13	0,66	0,53
TNNT1	-4,41	-6,04	-1,63
TNP2	0,22	0,59	0,37
TNPO1	-0,04	1,08	1,11
TNPO2	0,48	0,58	0,09
TNPO3	0,09	-0,10	-0,19
TNR	-0,32	0,11	0,43
TNRC15	0,05	0,07	0,01
TNRC5	-0,44	-1,85	-1,41
TNRC6A	0,58	-0,19	-0,78
TNRC6B	0,16	-0,83	-0,99

TNS1	-0,80	1,84	2,64
TNS3	-1,00	0,98	1,98
TNS4	-0,56	-0,20	0,37
TOB1	-0,48	-0,46	0,02
TOE1	-0,15	-0,70	-0,55
TOM1L2	0,19	0,40	0,22
TOMM34	0,64	1,12	0,48
TOMM40	-0,82	1,19	2,01
TOMM7	-0,10	-0,36	-0,26
TOMM70A	0,06	0,87	0,81
TOP2A	-11,35	0,00	11,35
TOP3A	0,67	-1,07	-1,75
TOPORS	0,10	-0,20	-0,31
TOR1A	0,44	-0,06	-0,50
TOR1AIP1	-0,09	-0,48	-0,39
TOR1AIP2	-0,01	1,80	1,81
TOR1B	0,08	0,14	0,06
TOR2A	0,51	-0,84	-1,35
TOR3A	0,16	0,77	0,61
TP53	0,57	-0,35	-0,92
TP53AP1	-0,57	0,12	0,69
TP53BP1	0,30	1,39	1,08
TP53I11	-0,77	-0,18	0,60
TP53I3	-0,28	0,56	0,84
TP53INP1	0,25	-1,00	-1,25
TP53RK	0,15	0,73	0,58
TPCN1	0,19	0,12	-0,07
TPD52L2	0,23	-0,23	-0,45
TPD52L3	-0,06	-0,19	-0,13
TPM3	0,06	0,16	0,10
TPM4	0,49	1,54	1,05
TPMT	0,24	0,76	0,53
TPO	0,15	3,65	3,50
TPP1	-0,30	0,23	0,53
TPP2	0,77	-0,31	-1,07
TPRKB	-0,08	0,06	0,14
TPRX1	-0,57	-0,26	0,31
TPST2	-0,15	0,73	0,89
TPT1	-0,13	-0,22	-0,09
TRA16	-0,07	0,38	0,45
TRA2A	0,16	-1,14	-1,30
TRADD	0,18	-0,09	-0,27
TRAF3IP2	-2,77	-1,11	1,67
TRAF5	2,32	2,40	0,08
TRAK1	0,22	0,49	0,27
TRAK2	-0,21	0,42	0,63
TRAPPC1	-0,17	-0,45	-0,28
TRAPPC2	0,00	-0,49	-0,49
TRAPPC4	0,11	1,07	0,96
TRAPPC6B	-0,23	-0,09	0,14
TRDN	0,03	3,57	3,54
TREM2	0,55	11,91	11,36
TREML1	0,50	1,67	1,17
TREML4	-0,32	-0,57	-0,25
TREX1	-0,91	-0,22	0,69
TRIAD3	0,34	-0,69	-1,03
TRIAP1	0,20	0,64	0,44
TRIB1	-0,47	0,10	0,57
TRIB2	2,80	2,91	0,10

TRIB3	0,62	1,56	0,95
TRIM11	0,38	-0,36	-0,73
TRIM15	0,71	3,13	2,43
TRIM17	1,31	2,80	1,49
TRIM2	-6,98	-8,84	-1,86
TRIM23	-0,22	-0,20	0,02
TRIM25	-0,14	-0,36	-0,21
TRIM29	0,23	0,80	0,57
TRIM3	0,31	-0,50	-0,81
TRIM32	-0,04	2,15	2,19
TRIM34	-0,38	-1,16	-0,78
TRIM35	-0,30	2,54	2,84
TRIM38	0,04	-1,26	-1,30
TRIM39	0,19	0,45	0,26
TRIM4	0,28	-0,17	-0,45
TRIM41	0,13	-0,42	-0,54
TRIM43	0,11	-0,45	-0,55
TRIM45	0,44	-1,27	-1,71
TRIM5	0,28	-0,68	-0,95
TRIM54	-2,18	-0,63	1,55
TRIM6	-2,02	1,45	3,47
TRIM61	-0,63	-1,40	-0,76
TRIM65	0,03	1,18	1,16
TRIM67	-1,42	-0,37	1,05
TRIM73	-0,19	-0,28	-0,09
TRIOBP	-0,17	-0,93	-0,75
TRIP10	1,14	3,33	2,19
TRIP11	0,08	0,70	0,62
TRIP12	0,15	-0,09	-0,25
TRIP13	-3,37	4,92	8,29
TRIP4	0,00	0,10	0,10
TRIP6	-0,13	2,41	2,54
TRIT1	0,49	-1,17	-1,66
TRMT1	-1,01	-1,23	-0,21
TRMT12	-0,09	0,31	0,40
TRMU	0,34	0,87	0,53
TROVE2	0,75	0,32	-0,43
TRPC4AP	0,02	-0,31	-0,33
TRPC5	-4,44	-4,39	0,05
TRPM3	-0,77	-1,02	-0,25
TRPM7	-0,08	0,65	0,73
TRPS1	0,00	-1,66	-1,67
TRPV2	0,15	2,90	2,75
TRPV3	-0,51	-0,04	0,47
TRPV6	-1,17	6,74	7,91
TRUB1	-0,46	4,66	5,12
TRUB2	-0,02	0,35	0,38
TSC22D1	-3,99	0,14	4,13
TSC22D2	0,08	-0,65	-0,73
TSC22D3	-0,46	-4,25	-3,79
TSC22D4	0,56	-0,39	-0,95
TSFM	-0,60	0,05	0,65
TSG101	-0,09	0,09	0,18
TSGA14	-0,11	0,51	0,62
TSHZ1	-0,05	-1,16	-1,11
TSHZ3	0,51	-2,13	-2,64
TSKS	-1,90	-2,42	-0,52
TSN	0,21	-1,03	-1,24
TSNAX	-0,53	-0,32	0,21

TSP50	0,36	-0,21	-0,57
TSPAN1	-0,16	-0,18	-0,03
TSPAN10	-0,36	1,80	2,15
TSPAN14	-0,80	-0,09	0,71
TSPAN18	0,07	0,52	0,45
TSPAN3	-0,76	1,88	2,65
TSPAN31	-0,52	-0,11	0,41
TSPAN4	0,32	0,98	0,67
TSPAN7	9,08	9,08	0,00
TSPYL1	0,27	0,22	-0,05
TSPYL6	-0,03	-0,21	-0,18
TSR1	0,35	0,27	-0,09
TSSC1	-0,44	0,16	0,60
TSSC4	0,31	-0,70	-1,02
TSSK6	1,61	0,07	-1,54
TST	-0,47	-0,28	0,18
TSTA3	0,13	-0,01	-0,15
TTBK2	-0,45	-1,45	-1,01
TTC1	-0,33	0,28	0,62
TTC13	0,84	-0,35	-1,19
TTC14	0,23	-1,23	-1,46
TTC15	0,21	-0,27	-0,48
TTC16	-0,12	0,25	0,38
TTC17	0,40	-0,81	-1,20
TTC21A	0,69	-1,68	-2,37
TTC23	-0,37	4,71	5,08
TTC25	-1,33	-2,33	-1,00
TTC3	-0,11	0,45	0,56
TTC7A	0,58	0,36	-0,22
TTC8	-0,13	-0,22	-0,09
TTF1	-0,37	-0,76	-0,39
TTK	-3,80	6,38	10,18
TTLL13	-0,32	0,15	0,47
TTLL3	0,55	-0,72	-1,27
TTLL5	-0,18	-0,23	-0,05
TTN	0,03	-0,47	-0,51
TTRAP	-0,65	1,13	1,78
TTYH2	1,84	-0,10	-1,93
TUB	0,41	0,60	0,19
TUBB	-0,48	1,46	1,94
TUBB1	-0,14	-0,77	-0,63
TUBB2C	-0,09	1,43	1,52
TUBB3	-0,39	9,23	9,62
TUBB4Q	0,07	1,19	1,12
TUBB6	0,36	4,13	3,77
TUBB8	-0,21	1,53	1,74
TUBD1	0,84	0,38	-0,45
TUBE1	0,50	1,76	1,26
TUBG1	-0,46	2,21	2,67
TUBGCP2	0,13	-0,55	-0,69
TUBGCP6	0,32	-1,15	-1,47
TUFM	0,18	0,00	-0,18
TUFT1	-0,29	-0,07	0,23
TULP4	-0,33	0,64	0,97
TUSC2	-0,06	-0,27	-0,21
TUSC4	0,37	-0,23	-0,61
TWISTNB	-0,39	-0,04	0,35
TWSG1	-0,62	1,52	2,14
TXK	-0,20	0,31	0,52

TXLNA	0,58	0,78	0,21
TXLNB	-0,40	1,39	1,79
TXN2	0,13	0,52	0,40
TXNDC10	-0,22	-0,78	-0,56
TXNDC11	-0,53	-0,50	0,03
TXNDC12	-0,06	0,35	0,42
TXNDC13	-0,30	-0,15	0,15
TXNDC14	-0,12	0,80	0,93
TXNDC3	0,64	-0,82	-1,46
TXNDC4	0,04	-1,05	-1,09
TXNDC5	-0,71	0,23	0,94
TXNDC9	0,10	0,90	0,80
TXNIP	0,24	-2,18	-2,42
TXNL1	0,12	0,66	0,53
TXNL4A	0,25	-0,39	-0,65
TXNL4B	-0,75	-0,62	0,14
TXNL5	0,05	0,95	0,90
TXNL6	3,88	-1,64	-5,52
TXNRD1	-0,51	2,38	2,89
TXNRD2	-0,03	-0,64	-0,61
TYROBP	-0,38	-0,24	0,14
TYSND1	-0,04	0,27	0,31
U2AF1	0,07	-1,00	-1,07
U2AF1L4	1,44	0,49	-0,95
UAP1L1	0,76	1,46	0,70
UBA52	0,14	-0,08	-0,22
UBAP2	-0,62	-0,08	0,55
UBAP2L	1,42	1,15	-0,27
UBB	0,11	1,26	1,15
UBC	-0,03	0,00	0,03
UBD	5,50	4,03	-1,47
UBE1	0,19	-0,32	-0,52
UBE1C	-0,09	-0,68	-0,59
UBE1DC1	-0,15	-0,14	0,01
UBE1L2	-0,35	-0,21	0,14
UBE2A	0,17	-3,49	-3,66
UBE2B	0,14	-1,85	-1,99
UBE2C	-3,86	0,17	4,02
UBE2D2	-0,28	-0,86	-0,58
UBE2D3	-0,16	-1,02	-0,86
UBE2D4	-0,03	1,73	1,76
UBE2G2	0,44	-0,02	-0,46
UBE2H	-0,12	-0,29	-0,17
UBE2J2	0,31	-0,23	-0,54
UBE2L6	-0,24	-0,41	-0,18
UBE2M	0,00	0,31	0,32
UBE2O	0,18	-1,47	-1,65
UBE2Q1	0,11	0,09	-0,02
UBE2R2	0,06	-1,50	-1,56
UBE2T	-0,98	1,69	2,66
UBE2V1	-0,13	0,33	0,46
UBE2W	-0,02	0,56	0,58
UBE2Z	1,15	1,59	0,43
UBE3A	0,21	-0,25	-0,46
UBE3B	-0,01	-0,07	-0,06
UBE3C	0,06	0,09	0,03
UBE4A	-0,36	-0,62	-0,25
UBE4B	-0,22	-0,48	-0,26
UBIAD1	0,29	-0,03	-0,33

UBL3	0,96	0,77	-0,19
UBL4A	-0,03	0,88	0,91
UBL5	-0,15	0,41	0,56
UBL7	0,13	-0,17	-0,30
UBN1	0,57	0,39	-0,19
UBOX5	0,24	-0,85	-1,10
UBQLN1	-0,01	-0,09	-0,08
UBQLN2	0,17	-0,31	-0,48
UBQLN3	-0,20	0,81	1,01
UBQLN4	-0,16	0,36	0,51
UBR1	-0,49	-0,38	0,11
UBTD1	-0,64	0,99	1,62
UBTF	0,67	0,60	-0,07
UBXD2	0,01	0,20	0,20
UBXD5	-0,38	-1,49	-1,11
UCHL3	-1,31	-0,51	0,81
UCHL5	0,20	1,23	1,03
UCK2	-0,48	0,05	0,52
UCN	-0,14	-0,32	-0,18
UCN3	0,51	-0,15	-0,65
UCP3	0,83	0,00	-0,83
UCRC	0,02	1,58	1,57
UFC1	-0,16	0,05	0,21
UFD1L	-0,38	0,63	1,02
UFM1	-0,33	-0,17	0,17
UGCGL1	-0,15	-0,71	-0,56
UGDH	-0,32	1,24	1,56
UGT2B11	-0,75	-1,17	-0,42
UGT2B17	-3,23	-0,14	3,10
ULK3	-0,29	-0,34	-0,05
UMOD	-0,04	-0,69	-0,65
UNC119	0,78	-0,96	-1,74
UNC13D	0,15	-1,46	-1,61
UNC45A	-0,04	0,10	0,13
UNC50	-0,09	0,29	0,38
UNC5CL	0,37	-0,82	-1,19
UNC84A	0,28	-1,36	-1,64
UNC93A	-1,02	0,17	1,19
UNQ1940	0,28	-0,17	-0,45
UNQ473	0,13	0,04	-0,09
UNQ501	-1,01	-0,83	0,18
UNQ5830	-0,09	-0,33	-0,24
UNQ846	-0,16	-0,04	0,12
UPF3A	-0,10	-0,22	-0,12
UPK3B	-0,54	0,20	0,74
UQCRB	-0,52	0,62	1,14
UQCRC2	0,04	0,45	0,41
UQCRFS1	-0,01	0,14	0,15
UQCRH	0,22	0,29	0,07
UQCRQ	-0,08	0,75	0,84
UROD	-0,39	0,62	1,01
UROS	-0,05	1,07	1,12
USF1	0,23	-0,80	-1,03
USH2A	0,00	-0,09	-0,09
USH3A	0,86	1,28	0,42
USHBP1	-0,16	-0,24	-0,07
USMG5	0,06	0,16	0,10
USP1	0,19	-0,31	-0,50
USP10	-0,04	-0,67	-0,63

USP15	0,03	-1,75	-1,79
USP16	0,11	-0,12	-0,23
USP19	0,87	-0,78	-1,66
USP2	-1,02	-0,07	0,96
USP20	0,29	0,08	-0,21
USP21	0,37	-0,83	-1,21
USP25	-0,30	-1,61	-1,31
USP3	0,06	-2,00	-2,06
USP30	-0,70	0,70	1,40
USP32	-0,10	-1,75	-1,65
USP33	-0,21	-0,88	-0,67
USP34	0,11	-0,86	-0,98
USP35	0,49	-0,46	-0,95
USP36	0,88	-0,77	-1,65
USP37	0,55	1,00	0,45
USP38	0,05	0,85	0,81
USP39	-0,01	-0,53	-0,52
USP4	0,35	-0,33	-0,69
USP42	1,43	-1,41	-2,84
USP49	0,14	-1,32	-1,46
USP5	0,02	0,21	0,18
USP51	-0,46	-0,71	-0,26
USP52	0,09	-1,43	-1,51
USP53	0,59	0,58	-0,01
USP6	0,10	-1,13	-1,23
USP9X	-0,05	-0,16	-0,12
USPL1	0,00	-0,85	-0,84
UTP11L	-0,14	0,36	0,50
UTP14A	-0,34	-0,11	0,23
UTP14C	-0,17	1,12	1,29
UTP15	0,08	0,61	0,53
UTP20	3,02	-0,74	-3,76
UTS2	0,04	-0,09	-0,13
UTX	0,33	-1,59	-1,92
UVRAG	0,99	0,27	-0,73
UXS1	-0,43	0,13	0,55
UXT	0,19	-0,22	-0,42
VAC14	0,29	2,42	2,13
VAMP3	0,24	0,48	0,25
VAMP5	0,22	-1,73	-1,95
VAMP8	-0,07	0,45	0,52
VARS	0,35	0,76	0,40
VASH1	0,67	2,47	1,80
VASN	0,56	1,36	0,80
VAV1	-0,80	-1,06	-0,26
VAV2	1,50	0,35	-1,15
VBP1	-0,03	0,15	0,18
VCAM1	1,71	1,88	0,17
VCP	-0,22	0,56	0,78
VCPIP1	0,80	0,36	-0,44
VDAC1	0,11	1,04	0,93
VDAC2	-0,09	0,64	0,73
VDAC3	0,07	0,57	0,49
VDP	-0,13	0,59	0,71
VDR	-0,33	1,40	1,72
VEPH1	0,01	0,24	0,23
VEZT	-0,42	0,32	0,74
VHL	0,16	-0,91	-1,08
VIM	0,09	0,35	0,26

VKORC1	-0,22	1,04	1,26
VMO1	-1,20	-0,42	0,77
VPRBP	-0,24	0,11	0,34
VPREB3	-0,19	-0,14	0,05
VPS11	0,12	-0,11	-0,23
VPS13B	-0,17	-1,25	-1,08
VPS13D	0,18	-0,78	-0,96
VPS16	0,10	-0,29	-0,39
VPS24	-0,14	0,27	0,41
VPS25	-0,01	0,06	0,06
VPS26B	0,00	-0,70	-0,69
VPS29	-0,50	-0,49	0,00
VPS35	-0,08	0,48	0,56
VPS36	0,47	-1,02	-1,49
VPS37A	0,02	1,01	0,98
VPS37C	-0,10	0,98	1,09
VPS39	-0,27	-0,31	-0,04
VPS41	-0,21	0,93	1,14
VPS52	0,09	-0,10	-0,19
VPS53	-0,16	0,24	0,40
VRK1	-0,31	-0,48	-0,17
VRK3	0,21	-0,34	-0,55
VSIG4	-6,11	-2,05	4,06
VSIG9	-0,22	-0,27	-0,05
VTI1A	-0,08	-0,20	-0,12
VTI1B	-0,08	0,80	0,88
VWF	-1,70	0,66	2,36
WAPAL	-0,49	-0,48	0,00
WARS	-0,35	0,55	0,91
WAS	0,18	-1,59	-1,77
WASF2	0,13	-0,66	-0,79
WASF3	6,76	8,24	1,48
WASL	0,04	0,11	0,07
WBP2	0,07	0,78	0,71
WBSCR18	1,13	-0,57	-1,70
WBSCR19	0,13	-1,49	-1,62
WBSCR22	-0,02	-0,22	-0,20
WDFY1	-0,44	-0,53	-0,10
WDFY3	-0,16	-1,44	-1,28
WDHD1	-0,94	2,35	3,29
WDR1	0,15	0,48	0,33
WDR12	-0,07	1,41	1,49
WDR13	-0,04	-0,39	-0,35
WDR19	-0,52	-0,40	0,12
WDR20	0,17	0,57	0,40
WDR23	-0,62	-1,51	-0,89
WDR24	0,71	-0,89	-1,59
WDR25	-0,89	-1,09	-0,20
WDR26	-0,59	-1,44	-0,84
WDR35	-0,72	-0,83	-0,10
WDR37	0,00	0,70	0,70
WDR4	0,30	0,29	0,00
WDR40A	-0,87	-1,11	-0,23
WDR41	0,21	0,45	0,24
WDR44	0,00	-0,13	-0,13
WDR45	0,32	-1,07	-1,39
WDR46	0,23	-0,82	-1,05
WDR48	0,51	-0,31	-0,82
WDR51A	-1,20	3,72	4,92

WDR53	0,17	0,43	0,26
WDR55	-0,37	-0,37	0,00
WDR57	0,10	-0,20	-0,30
WDR5B	-0,07	0,09	0,15
WDR62	-0,50	0,29	0,79
WDR65	0,10	0,29	0,19
WDR66	1,35	2,51	1,16
WDR67	-0,27	0,94	1,21
WDR7	0,03	1,34	1,31
WDR71	0,12	0,17	0,06
WDR73	0,07	-0,16	-0,23
WDR74	-0,05	-0,67	-0,62
WDR75	0,38	-0,25	-0,63
WDR77	1,13	0,61	-0,52
WDR79	-0,18	-1,08	-0,89
WDR81	0,33	0,38	0,05
WDSOF1	-0,67	0,37	1,04
WDSUB1	-0,06	0,19	0,25
WEE1	-8,74	-0,71	8,03
WFIKKN2	0,93	3,10	2,17
WHSC1	-0,44	-0,37	0,07
WHSC1L1	0,08	-0,96	-1,04
WIBG	0,36	0,64	0,27
WIPI1	-0,58	0,42	1,00
WNK1	0,12	-0,65	-0,76
WNT5A	11,41	11,41	0,00
WNT5B	3,20	2,63	-0,57
WRN	-0,16	0,04	0,20
WSB1	0,29	-0,54	-0,83
WSB2	0,31	1,66	1,34
WTAP	0,13	-0,52	-0,65
WWC2	2,44	4,30	1,86
WWOX	-0,37	-0,20	0,17
WWP2	0,18	-1,70	-1,89
XAB1	-0,11	-0,37	-0,26
XAB2	0,30	-0,55	-0,85
XCR1	0,28	0,15	-0,14
XKR3	3,02	6,95	3,94
XKR4	3,78	3,78	0,00
XKR6	0,24	0,51	0,28
XPA	-0,03	-0,59	-0,57
XPC	0,11	0,55	0,45
XPNPEP1	0,27	0,51	0,24
XPNPEP2	1,14	2,40	1,26
XPO5	0,50	0,59	0,09
XPO6	1,00	0,55	-0,45
XPR1	-0,73	-0,08	0,64
XRCC3	-0,83	0,18	1,01
XRCC4	-1,03	-1,33	-0,30
XRCC5	-0,29	-0,90	-0,60
XRCC6	0,32	0,23	-0,09
XRN1	-0,30	-0,49	-0,19
XRN2	-0,18	-1,01	-0,82
XYLB	-0,15	1,05	1,20
XYLT1	1,81	0,65	-1,16
YARS	0,37	0,73	0,36
YARS2	-0,14	1,05	1,19
YIF1B	-0,04	0,68	0,72
YIPF1	-0,53	-0,18	0,35

YIPF2	-0,10	0,51	0,61
YIPF3	-0,03	-0,52	-0,49
YIPF4	0,42	0,96	0,54
YIPF5	-0,19	1,02	1,21
YIPF6	-0,10	0,81	0,91
YIPF7	0,32	-0,15	-0,47
YME1L1	0,07	0,32	0,26
YOD1	0,29	-1,34	-1,63
YPEL5	0,61	-0,60	-1,21
YRDC	0,41	-1,34	-1,75
YTHDC2	0,11	-1,15	-1,26
YTHDF1	0,35	-0,44	-0,79
YTHDF2	0,22	0,20	-0,02
YTHDF3	-0,17	-0,61	-0,44
YWHAH	0,67	1,18	0,52
YWHAZ	-0,27	-0,20	0,07
YY1	0,37	-0,40	-0,76
YY1AP1	0,34	-0,60	-0,94
ZADH2	0,37	-0,29	-0,66
ZAK	-0,08	-0,42	-0,34
ZAP70	0,18	-0,09	-0,27
ZBED3	0,56	0,51	-0,05
ZBP1	-0,62	-3,89	-3,27
ZBTB11	0,82	-1,06	-1,88
ZBTB17	0,32	-0,20	-0,52
ZBTB2	0,61	-1,16	-1,77
ZBTB22	0,05	-0,55	-0,60
ZBTB24	1,02	0,40	-0,62
ZBTB26	0,72	0,50	-0,22
ZBTB3	0,29	1,29	1,00
ZBTB32	0,95	0,95	-0,01
ZBTB39	0,27	-0,39	-0,66
ZBTB4	0,32	0,10	-0,22
ZBTB40	0,20	-0,55	-0,75
ZBTB9	-0,77	1,80	2,58
ZC3H10	0,48	1,26	0,78
ZC3H11A	0,26	-1,20	-1,45
ZC3H12A	-0,85	-0,78	0,06
ZC3H3	0,26	-0,69	-0,95
ZC3H7A	0,18	-1,19	-1,37
ZC3H8	0,67	0,81	0,15
ZC3HAV1	0,92	-0,32	-1,24
ZCCHC14	0,14	-0,05	-0,18
ZCCHC17	-0,46	-0,04	0,42
ZCCHC2	-0,30	-0,54	-0,24
ZCCHC3	0,27	-0,32	-0,59
ZCCHC9	-0,01	0,32	0,33
ZCRB1	0,26	0,99	0,73
ZDHHC12	-0,36	0,34	0,69
ZDHHC14	-0,67	0,26	0,92
ZDHHC16	-0,49	0,33	0,82
ZDHHC17	-0,03	-0,81	-0,79
ZDHHC19	-0,46	9,35	9,81
ZDHHC20	0,06	-0,64	-0,69
ZDHHC23	0,03	-0,71	-0,74
ZDHHC3	-0,70	-0,01	0,69
ZDHHC4	-0,31	-0,62	-0,31
ZDHHC5	-0,07	0,45	0,51
ZDHHC6	-0,18	-0,33	-0,16

ZDHHC9	-0,68	0,99	1,67
ZFAND1	-0,03	-1,28	-1,25
ZFAND2A	-0,03	-1,95	-1,92
ZFAND3	0,45	-1,03	-1,47
ZFP106	-0,34	-1,40	-1,06
ZFP161	0,12	-0,26	-0,39
ZFP36	-0,67	-3,36	-2,69
ZFP91	0,13	-0,43	-0,56
ZFPL1	-0,07	0,37	0,44
ZFR	-0,11	-0,44	-0,33
ZFYVE1	0,24	0,40	0,16
ZFYVE19	-0,07	-0,11	-0,04
ZFYVE21	0,54	1,40	0,87
ZHX3	0,01	0,67	0,66
ZKSCAN1	0,48	0,19	-0,28
ZMAT1	0,54	-1,02	-1,56
ZMAT2	-0,47	-0,60	-0,13
ZMAT4	-0,04	-0,48	-0,44
ZMAT5	-0,34	-0,36	-0,02
ZMPSTE24	-0,55	0,44	0,99
ZMYM3	0,31	0,02	-0,29
ZMYM4	-0,13	-1,10	-0,96
ZMYM6	-0,44	-0,39	0,05
ZMYND10	-0,17	-0,57	-0,39
ZMYND11	-0,29	0,00	0,29
ZMYND12	-0,93	0,30	1,23
ZMYND15	-1,52	-0,04	1,48
ZMYND17	0,29	-1,32	-1,61
ZMYND19	0,27	0,47	0,20
ZNF10	0,05	1,08	1,03
ZNF12	0,70	-0,21	-0,91
ZNF121	0,62	0,49	-0,13
ZNF132	0,77	5,02	4,25
ZNF133	-0,04	-0,48	-0,44
ZNF134	0,27	0,81	0,54
ZNF135	-0,14	0,03	0,17
ZNF136	0,56	0,09	-0,47
ZNF138	1,13	0,86	-0,27
ZNF140	0,61	1,42	0,80
ZNF142	0,86	-0,10	-0,96
ZNF146	-0,24	0,21	0,45
ZNF148	0,35	-0,57	-0,92
ZNF157	0,29	0,87	0,58
ZNF169	1,64	-0,58	-2,22
ZNF17	0,15	0,99	0,84
ZNF174	0,33	0,61	0,29
ZNF175	0,77	1,64	0,87
ZNF177	0,15	0,74	0,59
ZNF179	1,32	-0,84	-2,15
ZNF180	-0,18	0,15	0,33
ZNF181	0,52	1,82	1,29
ZNF184	0,02	0,39	0,36
ZNF185	0,03	-1,60	-1,63
ZNF2	0,30	0,46	0,16
ZNF20	0,57	0,56	-0,01
ZNF200	0,71	1,62	0,91
ZNF207	0,08	0,09	0,01
ZNF211	0,47	-0,86	-1,34
ZNF213	-0,04	0,27	0,31

ZNF214	-0,24	0,08	0,32
ZNF217	0,97	0,76	-0,21
ZNF219	-0,05	2,69	2,74
ZNF222	0,25	2,37	2,12
ZNF226	-0,28	-0,36	-0,08
ZNF227	0,32	-0,08	-0,40
ZNF228	-0,12	1,16	1,28
ZNF23	0,23	0,21	-0,01
ZNF230	0,46	0,24	-0,22
ZNF232	0,10	0,13	0,02
ZNF234	0,21	0,77	0,55
ZNF235	0,23	0,21	-0,01
ZNF239	-1,39	-2,38	-0,99
ZNF24	-0,43	-1,28	-0,85
ZNF248	0,64	0,40	-0,24
ZNF259	0,29	0,29	0,01
ZNF263	0,50	0,28	-0,22
ZNF268	0,26	1,14	0,87
ZNF271	-0,11	-0,38	-0,27
ZNF274	0,50	1,99	1,49
ZNF282	0,52	0,54	0,03
ZNF283	0,63	0,07	-0,56
ZNF289	0,31	-0,19	-0,50
ZNF294	0,17	-0,53	-0,69
ZNF3	0,20	-0,49	-0,68
ZNF30	0,47	2,90	2,43
ZNF304	0,23	1,08	0,85
ZNF317	0,97	1,11	0,14
ZNF318	0,13	0,14	0,01
ZNF322B	0,34	1,33	0,99
ZNF323	0,66	3,14	2,48
ZNF324	0,73	-0,13	-0,86
ZNF326	0,37	-0,30	-0,67
ZNF333	0,24	-1,82	-2,05
ZNF335	0,08	-1,49	-1,57
ZNF337	0,23	-0,53	-0,76
ZNF33A	1,30	-2,34	-3,64
ZNF342	1,15	-1,65	-2,81
ZNF343	0,24	-0,01	-0,25
ZNF345	-0,04	-0,46	-0,42
ZNF350	-0,04	1,14	1,18
ZNF358	0,15	-0,31	-0,46
ZNF364	0,29	0,26	-0,04
ZNF365	-2,62	4,35	6,97
ZNF366	1,38	4,53	3,15
ZNF367	-0,47	0,03	0,50
ZNF384	0,20	-0,92	-1,12
ZNF385	-0,17	-0,48	-0,31
ZNF395	-1,39	-3,34	-1,95
ZNF397	1,40	3,51	2,11
ZNF398	0,33	-0,49	-0,83
ZNF403	0,02	-0,15	-0,18
ZNF404	1,23	0,61	-0,62
ZNF407	0,19	-0,20	-0,40
ZNF408	0,71	-0,66	-1,37
ZNF41	-0,58	-0,93	-0,35
ZNF410	-0,68	-0,68	0,00
ZNF420	0,77	2,08	1,31
ZNF425	2,34	2,43	0,09

ZNF431	0,69	-0,67	-1,36
ZNF434	-0,12	0,30	0,41
ZNF436	0,09	0,29	0,20
ZNF444	-0,61	0,07	0,68
ZNF446	0,26	-0,65	-0,91
ZNF45	0,27	0,54	0,26
ZNF451	0,31	-0,76	-1,07
ZNF452	0,95	-0,02	-0,97
ZNF473	0,14	-0,44	-0,58
ZNF484	-0,57	-0,63	-0,05
ZNF496	0,18	0,06	-0,11
ZNF498	-0,02	-0,48	-0,46
ZNF509	-0,52	-0,15	0,36
ZNF511	0,00	-0,91	-0,91
ZNF512	0,30	-0,73	-1,03
ZNF513	0,45	-0,25	-0,70
ZNF524	0,38	-0,64	-1,02
ZNF526	0,49	-0,19	-0,68
ZNF529	0,40	1,05	0,64
ZNF530	0,65	1,18	0,53
ZNF536	-0,02	-0,21	-0,19
ZNF541	-0,98	2,65	3,63
ZNF544	-0,14	1,57	1,71
ZNF550	-0,32	-0,09	0,24
ZNF554	0,25	0,20	-0,05
ZNF558	0,11	-0,39	-0,50
ZNF561	0,50	1,06	0,56
ZNF564	0,73	0,66	-0,07
ZNF567	1,01	1,41	0,40
ZNF569	0,22	0,34	0,12
ZNF570	0,34	0,51	0,17
ZNF576	0,29	1,36	1,08
ZNF582	1,10	1,04	-0,06
ZNF583	-0,33	-0,41	-0,08
ZNF585A	-0,10	1,05	1,15
ZNF586	0,11	-0,84	-0,95
ZNF589	0,30	1,62	1,32
ZNF592	0,28	-0,84	-1,12
ZNF597	0,58	0,26	-0,32
ZNF606	1,11	1,60	0,49
ZNF609	0,40	0,30	-0,10
ZNF610	-0,52	1,96	2,48
ZNF611	-0,11	0,23	0,34
ZNF613	0,84	2,38	1,54
ZNF614	-0,06	1,41	1,46
ZNF621	-0,15	0,33	0,48
ZNF622	0,15	0,64	0,49
ZNF624	0,32	0,44	0,13
ZNF628	-0,09	-1,40	-1,31
ZNF630	0,88	-0,58	-1,46
ZNF644	0,32	-0,03	-0,35
ZNF646	0,24	0,00	-0,24
ZNF650	0,62	0,30	-0,31
ZNF653	0,47	-0,42	-0,90
ZNF654	0,58	0,16	-0,41
ZNF655	0,00	-0,44	-0,44
ZNF658	0,03	0,67	0,64
ZNF658B	0,07	1,10	1,02
ZNF663	-0,34	-0,33	0,02

ZNF664	-0,16	1,16	1,32
ZNF668	0,04	-0,20	-0,24
ZNF672	0,73	1,11	0,37
ZNF673	0,38	-0,53	-0,91
ZNF675	0,77	0,58	-0,19
ZNF679	0,88	0,20	-0,68
ZNF683	0,04	0,35	0,30
ZNF684	-0,12	0,19	0,30
ZNF69	0,39	0,75	0,36
ZNF691	0,50	1,41	0,91
ZNF692	0,13	-0,36	-0,49
ZNF696	0,88	1,23	0,35
ZNF700	0,44	-0,51	-0,95
ZNF706	0,09	0,10	0,01
ZNF713	-0,18	0,01	0,19
ZNF714	-0,22	0,89	1,11
ZNF720	0,23	0,48	0,25
ZNF721	0,61	0,48	-0,13
ZNF740	0,56	0,14	-0,43
ZNF747	-0,76	-0,49	0,28
ZNF75	0,78	-0,53	-1,31
ZNF75A	1,15	0,22	-0,93
ZNF76	-0,09	-0,10	-0,01
ZNF77	0,43	0,56	0,13
ZNF79	0,85	2,09	1,24
ZNF84	1,19	1,75	0,56
ZNFX1	0,31	1,13	0,82
ZNHIT1	0,04	0,28	0,24
ZNHIT2	0,40	-0,37	-0,77
ZNHIT3	-0,04	-0,26	-0,22
ZNHIT4	-0,21	-1,55	-1,34
ZNRD1	-0,34	-0,60	-0,27
ZNRF2	-0,33	-0,07	0,26
ZP4	0,13	0,53	0,40
ZRANB1	0,03	-0,23	-0,26
ZRANB3	-1,56	-0,32	1,24
ZSCAN2	0,22	-0,68	-0,89
ZSCAN5	-0,06	1,11	1,17
ZSWIM1	0,05	0,19	0,15
ZSWIM4	-0,49	0,41	0,90
ZW10	-0,21	0,99	1,20
ZWILCH	0,22	2,16	1,94
ZWINT	-1,54	1,23	2,77
ZXDB	0,52	-0,06	-0,57
ZXDC	-0,15	-1,62	-1,47
ZYX	-0,53	-0,63	-0,09
ZZEF1	0,22	-0,56	-0,78
ZZZ3	-0,50	0,18	0,69
BA16L21.2.1	0,22	1,15	0,93
DJ341D10.1	0,17	-0,51	-0,68
AIP	0,42	-0,19	-0,61
MGC40168	0,58	0,49	-0,09
IIP45	0,24	-0,77	-1,01
EIF3I	-0,24	-0,39	-0,15
ZRANB2	-0,08	-0,79	-0,71
LOC149620	0,17	1,23	1,06
SEC22B	-0,42	0,51	0,93
ATP1A1	-0,40	0,43	0,83
FCRLA	-2,86	1,68	4,54

QSOX1	1,01	2,26	1,25
SMC6	0,41	0,25	-0,16
HADH	-0,34	1,04	1,38
FOXN2	0,39	-0,47	-0,86
CA11	-1,62	3,19	4,81
REGL	0,87	-0,20	-1,07
CIAO1	-0,11	0,19	0,30
REV1	0,49	-0,55	-1,04
ZEB2	-0,18	-1,05	-0,87
SPC25	-8,73	0,00	8,73
GALNT7	0,10	0,05	-0,06
DPH3	-0,11	1,62	1,73
SEC22C	-0,32	1,37	1,69
CNBP	-0,23	-0,70	-0,47
B3GALNT1	3,12	8,52	5,40
APM-1	-0,32	0,52	0,85
SEC31A	-0,06	-0,24	-0,18
DSP	-5,44	-3,09	2,34
NAIP	0,50	-1,08	-1,58
POLQ	-2,42	1,49	3,91
CNR2	-0,05	0,68	0,73
LOC340156	0,16	-0,72	-0,88
MED20	-0,15	-1,15	-0,99
VEGFA	0,44	-0,55	-0,98
MCM9	0,51	-2,83	-3,33
MAGI1	-1,48	-2,15	-0,66
EIF3B	0,16	-0,26	-0,42
NPSR1	-0,02	-0,13	-0,10
EIF4H	-0,41	-0,77	-0,36
ZSCAN21	0,38	0,21	-0,17
MGA	0,22	0,00	-0,22
DEF6	0,21	-2,05	-2,26
FAM110B	3,88	2,24	-1,64
EIF3E	0,30	-0,56	-0,86
MED30	0,03	-1,29	-1,33
RIF1	-0,05	-0,34	-0,29
LOC441459	-0,59	-0,45	0,13
RMI1	0,36	1,11	0,75
RC3H2	0,21	-0,03	-0,24
BMI1	0,75	0,58	-0,17
ZCD1	0,41	1,78	1,37
PSAP	0,06	0,07	0,01
LCOR	0,49	-1,07	-1,56
SMC3	-0,18	-0,53	-0,35
CTR9	0,15	-0,06	-0,20
DPH4	-0,32	0,08	0,40
CLP1	0,28	-0,05	-0,34
RELT	-0,82	-2,68	-1,86
RSF1	0,62	0,72	0,10
ERC1	-0,24	-0,27	-0,03
CD27	-0,02	0,11	0,13
LOC144983	-0,33	2,51	2,84
TIFA	0,34	0,54	0,20
MGC13168	0,10	-0,04	-0,14
APPL2	0,60	1,12	0,52
ANKRD13A	0,55	-0,64	-1,20
TECT1	-0,31	0,71	1,02
C12ORF8	-0,23	-0,90	-0,67
DNCL1	0,16	0,85	0,68

LOC387921	0,34	1,73	1,39
KIAA0564	-1,21	0,31	1,52
LOC220416	-0,05	1,32	1,36
LOC440145	-0,21	0,27	0,48
DIS3	-0,52	-0,44	0,08
RBM26	0,29	-0,50	-0,79
C13ORF16	0,00	-0,63	-0,63
IL25	-0,39	0,60	0,99
REC8	0,11	-1,89	-2,00
EAPP	-0,05	-1,08	-1,04
TXNDC1	0,29	0,07	-0,22
SERPINA11	0,80	0,39	-0,41
KLC1	0,21	0,25	0,05
EIF3J	0,05	0,24	0,19
EID1	1,12	-0,66	-1,78
LIPH	-0,51	0,06	0,57
EDC3	-0,03	0,06	0,10
ZFAND6	-0,56	1,06	1,62
HN1L	0,29	-0,25	-0,53
KIAA0430	-0,08	-0,93	-0,85
CTF8	-0,03	0,10	0,13
NOB1	0,36	-1,18	-1,54
CHMP1A	0,11	0,05	-0,06
FRAG1	-0,34	0,49	0,83
GPR158L1	0,63	-1,07	-1,69
EPO	-0,26	-0,31	-0,04
NACA2	0,16	-0,77	-0,94
KIAA1303	0,04	0,37	0,33
ELP2	0,57	-0,12	-0,69
APM-1	-0,32	0,52	0,85
CDH1	0,56	1,23	0,67
EIF3G	0,63	-0,86	-1,49
MED26	0,53	-0,60	-1,13
NCAN	-0,09	-0,28	-0,19
TBCB	0,06	-0,30	-0,35
EIF3K	-0,12	-0,40	-0,28
MED29	0,06	-0,54	-0,59
EID2	0,30	-0,69	-0,99
PLAC8	-5,76	-10,39	-4,63
CPA6	1,88	0,14	-1,74
KLK7	-0,92	0,81	1,72
FIZ1	0,32	0,06	-0,26
ZSCAN22	1,01	0,66	-0,36
MZF1	0,30	-0,91	-1,21
FAM110A	-0,27	-1,94	-1,67
SIRPB2	-2,58	-4,55	-1,98
KIAA1434	-1,62	-1,54	0,08
GZF1	1,97	-1,21	-3,17
PIGU	-0,68	0,46	1,14
MYH14	0,43	0,22	-0,20
SNX21	-0,39	-1,16	-0,77
CTSA	-0,26	0,70	0,97
ZMYND8	-0,55	-0,14	0,41
C20ORF107	-0,29	-1,09	-0,80
SRM	-0,06	0,55	0,61
N6AMT1	-0,27	3,09	3,36
PWP2	0,26	-0,51	-0,77
LOC91353	-0,43	-0,54	-0,11
LOC402055	0,04	1,06	1,02

HSCB	0,01	-0,65	-0,66
PVALB	-0,67	1,69	2,36
FLJ46257	0,50	0,52	0,02
LOC401589	-1,33	0,64	1,97
HSD17B10	-0,17	-0,67	-0,50
FAM104B	-1,01	-0,06	0,95
LOC340527	-3,92	-5,30	-1,38
DKFZP564K142	0,49	-0,88	-1,36
TAF9L	-0,39	-0,43	-0,04
SRPX	-2,06	4,94	7,00
CENPI	-1,88	3,96	5,84
LOC255313	-0,70	-0,48	0,23
NOV	-4,44	-3,64	0,80
